# Hypoxia Molecular Characterization in Hepatocellular Carcinoma Identifies One Risk Signature and Two Nomograms for Clinical Management

**DOI:** 10.1155/2021/6664386

**Published:** 2021-01-20

**Authors:** Zaoqu Liu, Long Liu, Taoyuan Lu, Libo Wang, Zhaonan Li, Dechao Jiao, Xinwei Han

**Affiliations:** ^1^Department of Interventional Radiology, The First Affiliated Hospital of Zhengzhou University, Zhengzhou 450052, China; ^2^Interventional Institute of Zhengzhou University, Zhengzhou 450052, China; ^3^Interventional Treatment and Clinical Research Center of Henan Province, Zhengzhou 450052, China; ^4^Department of Hepatobiliary and Pancreatic Surgery, The First Affiliated Hospital of Zhengzhou University, Zhengzhou 450052, China; ^5^Department of Cerebrovascular Disease, Zhengzhou University People's Hospital, Zhengzhou 450003, China

## Abstract

Hypoxia is a universal feature in the tumor microenvironment (TME). Nonetheless, the heterogeneous hypoxia patterns of TME have still not been elucidated in hepatocellular carcinoma (HCC). Using consensus clustering algorithm and public datasets, we identified heterogeneous hypoxia subtypes. We also revealed the specific biological and clinical characteristics via bioinformatic methods. The principal component analysis algorithm was employed to develop a hypoxia-associated risk score (HARS). We identified the two hypoxia subtypes: low hypoxia pattern (C1) and high hypoxia pattern (C2). C1 was less sensitive to immunotherapy compared to C2, consistent with the lack of immune cells and immune checkpoints (ICPs) in C1, whereas C2 was the opposite. C2 displayed worse prognosis and higher sensitivity to obatoclax relative to C1, while C1 was more sensitive to sorafenib. The two subtypes also demonstrated subtype-specific genomic variations including mutation, copy number alteration, and methylation. Moreover, we developed and validated a risk signature: HARS, which had excellent performance for predicting prognosis and immunotherapy. We revealed two hypoxia subtypes with distinct biological and clinical characteristics in HCC, which enhanced the understanding of hypoxia pattern. The risk signature was a promising biomarker for predicting prognosis and immunotherapy.

## 1. Introduction

Hepatocellular carcinoma (HCC) is one of the most common liver malignancies, accounting for about 75%–80% of primary liver cancers, and is the fourth leading cause of cancer-related mortality globally [1]. HCC arises mainly from chronic hepatitis B and hepatitis C, alcohol addiction, metabolic liver disease, and exposure with aflatoxins and aristolochic acid [2]. Although the clinical diagnosis and treatment of HCC have been greatly improved, the therapy efficacy is still disappointing and the 5-year recurrence rate exceeds 70% [3]. Recently, immunotherapy has made tremendous progress as a novel treatment method in HCC, but so far, it benefited only a subset of patients [4, 5]. This might be due to lack the awareness with the heterogeneity of tumor microenvironment (TME) in HCC.

Hypoxia plays a vital role in TME and has intense correlations with tumor cells, immune cells, stromal cells, as well as plenty of cytokine and chemokine [6, 7]. The TME is the survival soil of tumor and has profound impacts on the tumorigenesis and progression of HCC [8]. Hypoxia is a common status of TME; it triggers the deposition and degradation of extracellular matrix and contributes to abnormal angiogenesis, desmoplasia, and inflammation, which further promotes the aggressiveness and metastasis of tumor [6, 9]. For example, hypoxia elevates the reactive oxygen species (ROS) level and upregulates the expression of VEGF, facilitating tumor angiogenesis [10]. The two transcription factors Snail and Twist are activated in TME with hypoxia, inducing epithelial-mesenchymal transition (EMT) [6]. Hypoxia is subjected to the metabolic reprogramming of tumor cells, while the changes in metabolism in turn have great impacts on the hypoxia status of tumor [11]. Moreover, the hypoxia status of HCC is associated with sorafenib resistance, adverse prognosis, and high risk of recurrence [12]. Therefore, the hypoxia status of TME is tightly associated with the biological characteristics and clinical outcomes of HCC.

In addition, hypoxic TME is essential to immune infiltration and immunotherapy of tumor [13]. Immune cells are important components of TME, which have intense cell-cell interactions with tumor cells [14]. Immunotherapy is gradually becoming a revolutionized and promising treatment for cancer, which can activate T cells to perform the cytotoxicity by blocking specific immune checkpoints, such as CTLA-4 and PD-L1 [15]. Previous study suggested that hypoxia inhibited the effector and activity of lymphocytes [16]. Moreover, hypoxic TME attracts massive tumor-associated macrophages (TAMs), myeloid-derived suppressor cells (MDSCs), and regulatory T cells, which directly restricts the immune function and increases the resistance of immunotherapy [17]. Increasing researches demonstrate that hypoxia induces plenty of adenosine distribution in TME, which further impair the effects of immunotherapy [13]. Therefore, the low rates of response to immunotherapy might be explained by the complex hypoxia status of TME to some extent. With the systemic exploration of heterogeneous hypoxia subtype of HCC, we may identify populations who present poor sensitivity to immunotherapy, making appropriate decisions for clinical therapy.

To this context, we performed consensus clustering of multiple cohorts based on the expression of hypoxia-associated genes (HAGs). Two subtypes were identified and then divided into C1 with the low hypoxia pattern and C2 with the high hypoxia pattern. Further studies revealed the two subtypes had significant heterogeneity in biological function, immune cell infiltration, molecular characteristics, and clinical outcomes. In addition, based on the heterogeneity of hypoxia in TME, we developed and validated a risk signature termed hypoxia-associated risk score (HARS) for predicting prognosis and immunotherapy. It turned out HARS has excellent performance for prognosis and immunotherapy. Combining the HARS and vital clinical features, we further constructed two nomograms for overall survival (OS) and relapse-free survival (RFS) to predict the probability of patient survival. Collectively, this study increased the understanding of hypoxia in TME and facilitated the precise therapy and clinical managements of HCC.

## 2. Materials and Methods

### 2.1. Data Collection and Processing

A total of 831 patients from three independent cohorts encompassing TCGA-LIHC, ICGC-LIRI-JP, and NCI (National Cancer Institute) datasets (GSE14520) were enrolled. The TCGA-LIHC RNA-seq data were downloaded from the Cancer Genome Atlas (TCGA, https://portal.gdc.cancer.gov/). The ICGC-LIRI-JP RNA-seq was obtained from the International Cancer Genome Consortium portal (ICGC, https://dcc.icgc.org/). For TCGA-LIHC and ICGC-LIRI-JP datasets, the RNA-seq data were converted to the log2 (TPM+1) value, enhancing the comparability among samples. The raw expression data of NCI cohort were downloaded from the Gene Expression Omnibus (GEO, http://www.ncbi.nlm.nih.gov/geo/), and we further applied the robust multiarray average algorithm (RMA) of affy package to accomplish data normalization. Corresponding clinical data were also downloaded. The somatic mutation, copy number alteration (CNA), and methylation 450 Bead Chip array of TCGA-LIHC were obtained from the TCGA portal. In addition, we also collected four eligible immunotherapy datasets, including GSE100797, GSE91061, VanAllen cohort, and IMvigor210 [18–21]. For the details of datasets retrieved, refer to Table S1. All expression data were processed by Z-score normalization for subsequent analysis. Batch correction was performed to remove batch effect by ComBat algorithm. According to previous researches [22, 23], we summarized 24 hypoxia-associated genes (HAGs) including ALDOA, ANGPTL4, BNC1, CA9, CDKN3, COL4A6, ENO1, FOSL1, GNAI1, LDHA, P4HA1, PGAM1, PGK1, SDC1, SLC16A1, SLC2A1, TPI1, VEGFA, ACOT7, ADM, MIF, MRPS17, NDRG1, and TUBB6 (Table S2).

### 2.2. Hypoxia Subtypes Identification in HCC

The metacohort including a total of 831 HCC patients was utilized to identify hypoxia subtype based on the expression profiles of 24 HAGs. Unsupervised clustering was executed by ConsensusClusterPlus package. In ConsensusClusterPlus function, the number of clustering iterations was set to 1000, with 80% cases extracted per iteration; the K-means algorithm and the Euclidean distance were adopted; all cases were categorized as *k* subgroups (*k* = 2∼9). The cumulative distribution function (CDF) and proportion of ambiguous clustering (PAC) were applied to determine the optimal cluster number [24]. Moreover, the Nbclust package was used to further verify the best cluster number.

### 2.3. Exploration of Specific Biological Function in Two Subtypes

To investigate the different biological functions between two hypoxia subtypes, we performed gene set variation analysis (GSVA), using Molecular Signatures Database (MSigDB) v7.1 Hallmark and KEGG gene sets. We identified the significantly different biological functions by limma package, setting the criterions as log2FC > 0.2 and adjusted *P* value <0.05. The adjusted *P* value was obtained from Benjamin–Hochberg multiple correction. In addition, we also retrieved some known gene sets to further reveal biological function of hypoxia subtypes [21] (Table S3).

### 2.4. Evaluation of Immune Infiltration and Immunotherapy Response

The ssGSEA algorithm was further utilized to assess the infiltration abundance of 23 immune cells including innate immune cells (activated dendritic cell, CD56^bright^ natural killer cell, CD56^dim^ natural killer cell, eosinophil, immature dendritic cell, macrophage, mast cell, MDSC, monocyte, natural killer cell, neutrophil, and plasmacytoid dendritic cell) and adaptive immune cells (activated B cell, activated CD4+ T cells, activated CD8+ T cell, gamma delta T cell, immature B cell, natural killer T cell, regulatory T cell, T follicular helper cell, type 1T helper cell, type 17T helper cell, and type 2T helper cell) [25]. The infiltration abundance of fibroblasts was assessed via Microenvironment Cell Population-counter (MCP-counter) algorithm [26]. For the details of cell markers, refer to Table S4. The tumor immune dysfunction and exclusion (TIDE, http://tide.dfci.harvard.edu) web tool was utilized to predict the response to immunotherapy of different hypoxia subtypes [27]. The unsupervised subclass mapping (submap) method was applied to evaluate the expression similarity of HCC patients with hypoxia subtypes and patients with different immunotherapy outcomes; if a pair's expression profiles shared more similarity, their clinical outcomes were more likely to be similar [28].

### 2.5. The Prediction of Drug Sensitivity in Different Hypoxia Subtypes

The pRRophetic algorithm was fitting in a linear ridge regression based on gene expression and drug sensitivity data [29]. We used pRRophetic package to exhibit the response to sorafenib and obatoclax. Drug sensitivity was quantified by half-maximal inhibitory concentration (IC50); the lower the IC50, the more the sensitivity.

### 2.6. Mutation-Driven Genes and Mutation Signatures of Hypoxia Subtypes

According to silent mutational genes and noncoding mutations, we assessed the background mutation rate (BMR) of each gene-patient-subtype combination by MutSigCV [30]. The screening threshold was set as *q* < 0.05, and then, we could obtain a series of significantly mutated genes (SMGs). The extractSignatures implemented in NMF *R* package [31] was utilized to extract mutation signatures from the mutation count matrix. The potential rank was set to 2∼6, and we found that the optimal rank of both subtypes was 3. Subsequently, we calculated the pairwise cosine similarity between 3 mutation signatures and COSMIC signatures, matching each other (https://cancer.sanger.ac.uk/cosmic/signatures).

### 2.7. Copy Number Alteration

GISTIC 2.0 was utilized to identify the remarkably amplified and deleted genome regions [32]. To further quantify the overall changes in the genome, fraction of genome alteration (FGA), fraction of genome gained (FGG), and fraction of genome lost (FGL) were calculated. FGA was defined as the percentage of fragment base number of genome variation and FGG/FGL only focused on the gain or loss of genome variation.

### 2.8. The Estimation of Global Methylation Levels in Hypoxia Subtypes

The global methylation level (GML) of each patient from the TCGA database was calculated as the averaged beta values of specific probes [33]. We further constructed a pipeline to identify methylation-driven genes (MDGs) of each hypoxia subtypes, as follows: (1) removed the CpG sites that the averaged beta values greater than 0.2 in normal samples; (2) set the cutoff as 0.3, further divided the beta value matrix into the binary matrix only contained methylation and unmethylation; (3) removed the CpG sites that the number of samples in the methylated group was less than 10% of all tumor samples; (4) the probes were labeled methylated silence when the gap of the corresponding gene expression in the unmethylated group was more than 1.64 standard deviation of the corresponding gene expression in the methylated group; (5) multiple probes were matched one gene if over 50% probes were identified epigenetic silence, and the gene was labeled MDG.

### 2.9. Construction of Hypoxia-Associated Risk Signature

The limma package was utilized to identify differentially expressed genes (DEGs) between two subtypes. The criterions were set as adjusted *P* value <0.05 and |logFC| > 1 to define DEGs. We then used the clusterProfiler package to display GO and KEGG enrichment analysis. The significantly biological function was extracted with an adjusted *P* value <0.05. The adjusted *P* value was obtained from Benjamin–Hochberg multiple correction. The STRING database (https://string-db.org/) obtained the protein-protein interaction (PPI) networks of DEGs. Based on maximal clique centrality (MCC) algorithm, we applied the molecular complex detection (MCODE) to extract key module via Cytoscape software. The top 10 genes were defined as key genes. The univariate Cox regression analysis revealed the prognostic significance of DEGs. We further assessed the accuracy of prognostic prediction based on the area under the ROC curve (AUC) value of each DEG. The principal component analysis (PCA) algorithm was employed to construct a hypoxia-associated risk score (HARS) according to the key genes. Based on the nearest centroid method and Pearson correlation, we developed a *R* package termed HCCS (https://github.com/Zaoqu-Liu/HCCS). The pipeline could divide each sample into the corresponding hypoxia subtype and calculate the HARS of each sample.

### 2.10. Immunotherapy Response Prediction

To evaluate the immunotherapy sensitivity based on HARS, four independent immunotherapy cohorts contained prognosis and immunotherapy information was retrieved: (1) GSE100797: adoptive T cell therapy (ACT) for melanoma patients [18]; (2) GSE91061: anti-CTLA4 and anti-PD-1 therapy for melanoma patients [19]; (3) VanAllen cohort: anti-CTLA4 therapy for metastatic melanoma patients [20]; (4) IMvigor210 cohort: anti-PD-L1 therapy for metastatic urothelial carcinoma patients [21]. Based on the Response Evaluation Criteria in Solid Tumors (RECIST) criterion, we categorized the patients with complete response (CR) as well as partial response (PR) as responders and the patients with stable disease (SD) and progressive disease (PD) as nonresponders. In addition, the patients with not evaluable (NE) were exclusive of our study. Ultimately, we recruited 21 patients (8 responders and 13 nonresponders) for GSE100797, 49 patients (10 responders and 39 nonresponders) for GSE91061, 39 patients (7 responders and 32 nonresponders) for VanAllen cohort, and 298 patients (68 responders and 230 nonresponders) for IMvigor210 cohort.

### 2.11. Statistical Analysis

Correlation analysis between the two variables was accomplished by Spearman or Pearson correlation analysis. We applied Wilcoxon rank-sum or *T* test to evaluate the difference when comparing between two continuous variables. As to more than two groups of contrast, the Kruskal–Wallis or ANOVA test was carried out. We adopted the chi-squared or Fisher's test to compare differences in categorical variables. The distributions of CNA on chromosome were shown by RCircos package. The raw Affymetrix microarray data were processed by Affy package. The Combat function of sva package was used to remove batch effect of different datasets. The GSVA and ssGSEA analysis was performed by gsva package. The infiltration abundance of fibroblasts was assessing via MCP-counter package. The efficacy of immunotherapy was evaluated by TIDE and submap algorithm. The pRRopheticPredict function in pRRophetic package was utilized to predict the sensitivity of two subtypes to sorafenib and obatoclax. GO and KEGG enrichment analysis was performed via clusterProfiler package. PPI networks were obtained from STRING databases and using the MCODE plug-in of Cytoscape software to extract key module from PPI. The nomogram was developed to assess individual outcome of HCC patients by using rms package. The median was used to divide samples into two groups. Survival curves were generated by the Kaplan–Meier method, and the statistically significant difference was identified by the log-rank test. The Cox regression analysis was estimated using survival package. The pROC package was used to assess the accuracy of HARS for predicting immunotherapy response. The timeROC package was performed to evaluate the accuracy of HARS for predicting prognosis. Calibration curves were calculated by calibrate function implemented in rms package. All statistical *P* values were two-sided, and *P* < 0.05 was deemed as statistically significance. *P* adjust value was obtained by Benjamini–Hochberg (BH) multiple test correction. All data processing was completed in *R* 3.6.3 software.

## 3. Results

### 3.1. Landscape of Genomic Variations in Hypoxia-Associated Genes in HCC

**Figure 1:**
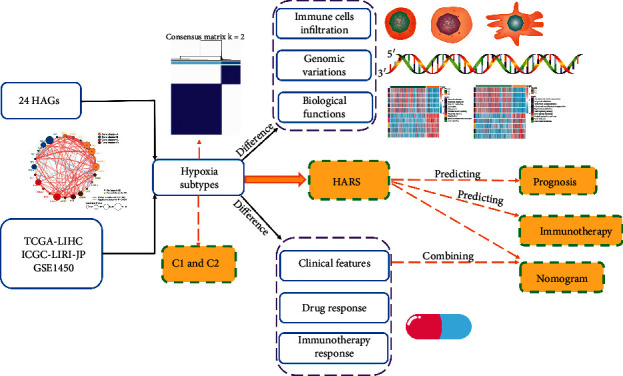
Overall workflow diagram of our research.

**Figure 2:**
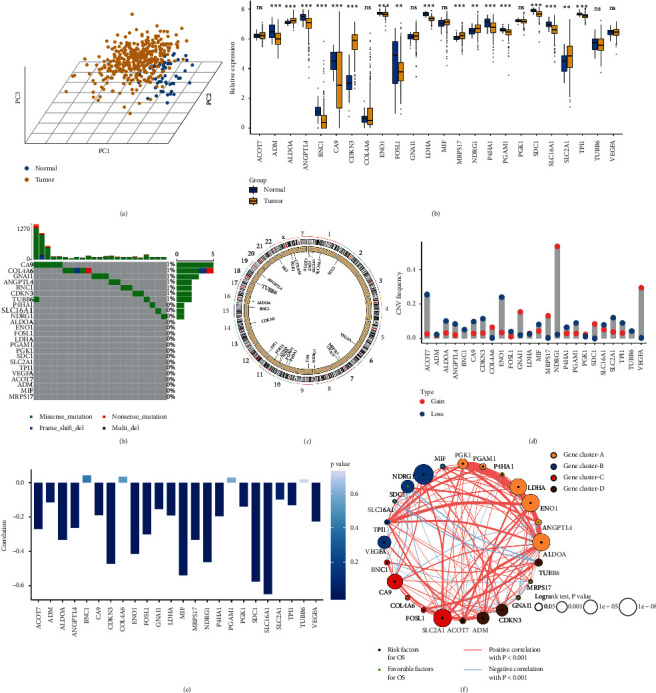
Landscape of genomic variations in hypoxia-associated molecules in TCGA-LIHC cohort. (a) Principal component analysis for the expression profiles of 24 hypoxia-associated genes (HAGs) to distinguish tumors from normal samples in TCGA-LIHC cohort. Tumor group is marked with yellow, and normal group is marked with blue. (b) The expression of 24 HAGs between normal and tumor groups. Normal, blue; tumor, yellow. ns, *P* > 0.05; ^*∗*^*P* < 0.05; ^*∗∗*^*P* < 0.01; ^*∗∗∗*^*P* < 0.001. (c) The mutation frequency of 24 HAGs. Each column represented individual patients. The upper bar plot showed TMB. The number on the right indicated the mutation frequency in each gene. The right bar plot showed the proportion of each variant type. (d) The location of copy number alteration (CNA) of HAGs on 23 chromosomes. (e) The CNA frequency of HAGs. The height of the column represented the alteration frequency. The loss frequency, blue dot; the gain frequency, red dot. (f) The correlation between methylation and expression of HAGs. (g) The interaction among 24 HAGs in HCC. The circle size represented the effect of each gene on the prognosis, and the range of values was calculated by the log-rank test was *P* < 1*e* − 8, *P* < 1*e* − 5, *P* < 0.001, and *P* < 0.05, respectively. Green dots in the circle, protective factors of prognosis; black dots in the circle, risk factors of prognosis. The lines linking regulators showed their interactions, and thickness showed the correlation strength between regulators. Negative correlation was marked with blue and positive correlation with red. The gene cluster A-D was marked with yellow, blue, red, and brown, respectively.

The workflow of our study is shown in Figure 1. A total of 24 hypoxia-associated genes (HAGs) were recruited in our research (Table S2) [22, 23]. Based on the expressions of 24 HAGs, we could distinguish HCC from normal tissues in the TCGA cohort (Figure 2(a)). Most of HAGs had significant differences between HCC and normal tissues (Figure 2(b)). To further explore the relationship between genomic alterations and expressions of 24 HAGs in HCC, the genomic alterations of these genes were summarized in the TCGA-LIHC project. We observed HAGs displayed scarce mutations, and over half of the molecules did not mutate (Figure 2(c)). The chromosomal locations where CNA experienced in HAGs are shown in Figure 2(d). It was nothing that CNA of these genes was broad, especially with copy number deletions (Figure 2(e)). Compared to normal samples, HAGs with deletions displayed lower expressions in HCC (e.g., ANGPTL4 and TPI1), while HAGs with amplification might promote mRNA expressions (e.g., MRPS17 and NDRG1). Moreover, DNA methylation negatively regulated many HAGs, e.g., SLC16A1, SDC1 and MIF, implying epigenetic silencing (Figure 2(f)). The above suggested the CNA and methylation might have a leading impact on HAG expressions compared to rare somatic mutations in HCC. We also found there were intense interactions and connections among HAGs, and most of HAGs were risk prognosis factors (Figure 2(g)).

### 3.2. Hypoxia Subtypes Were Identified in HCC

**Figure 3:**
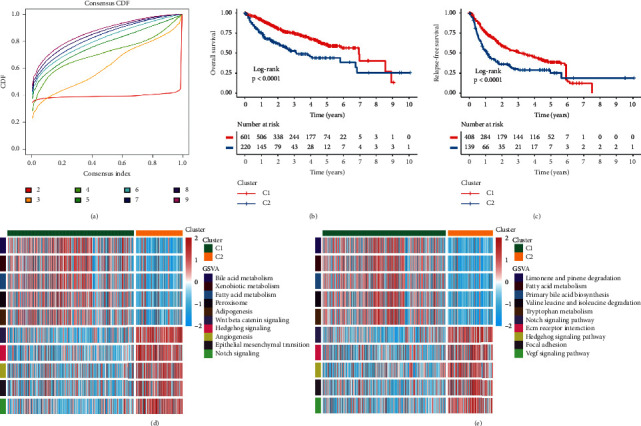
The prognostic significance and functional annotation of hypoxia subtypes in metacohort. (a) The CDF of consensus matrix for each *k* (indicated by colors). The lowest and flattest curve indicated the optimal *k* (*k* = 2). (b) Kaplan–Meier curves of OS for the two subtypes in metacohort. Log-rank test showed the *P* < 0.001. (c) Kaplan–Meier curves of RFS for the two subtypes in metacohort. Log-rank test showed the *P* < 0.001. (d) The activation of specific Hallmark pathways between the two subtypes. (e) The activation of specific KEGG pathways between the two subtypes.

According to the expression profiles of 24 HAGs, we performed consensus clustering analysis. The results indicated *k* = 2 was the optimal number (Figures S1(a) and S1(b)). Meanwhile, the cumulative distribution function (CDF) and NbClust also displayed 2 was the best classification (Figures 3(a) and S1(c)), verifying the robustness of our results. Thus, we divided 831 HCC patients into two clusters according to the above results: 608 cases in C1 and 223 cases in C2. Compared to C1, we found that most of HAGs were significantly upregulated in C2 (Figures S2(a) and S2(b)). Therefore, C1 was categorized as the low hypoxia pattern and C2 was the high hypoxia pattern. Consistent with this, C2 presented unfavorable overall survival (OS) and relapse-free survival (RFS) relative to C1 (Figures 3(b) and 3(c)). To further reveal the underlying biological characteristics, we performed GSVA enrichment analysis using Hallmark and KEGG gene sets. C1 was obviously enriched in metabolism-relevant pathways such as fatty acid metabolism, bile acid metabolism, and tryptophan metabolism, while C2 was mainly associated with angiogenesis relevant pathways such as angiogenesis, epithelial-mesenchymal transition (EMT), and VEGF signaling pathway (Figures 3(d) and 3(e)). Previous report demonstrated the high hypoxia pattern induced stromal activation and angiogenesis, which might further promote the progression and metastasis of tumor [34]. To better identify the biological significance of C2, we checked some known signatures [21]. The results suggested that C2 was associated with stromal relevant signatures, such as EMT1, EMT2, and EMT3 (Figure S2(c)). We further explored the infiltration abundance of fibroblasts by MCP-counter algorithm in two subtypes, which was also higher in C2 (Figure S2(d)). Overall, C1 was characterized as the low hypoxia pattern and metabolism-relevant function; C2 was characterized as the high hypoxia pattern and stromal activation.

### 3.3. Immune Infiltration and Immunotherapy Response of Molecular Subtypes

**Figure 4:**
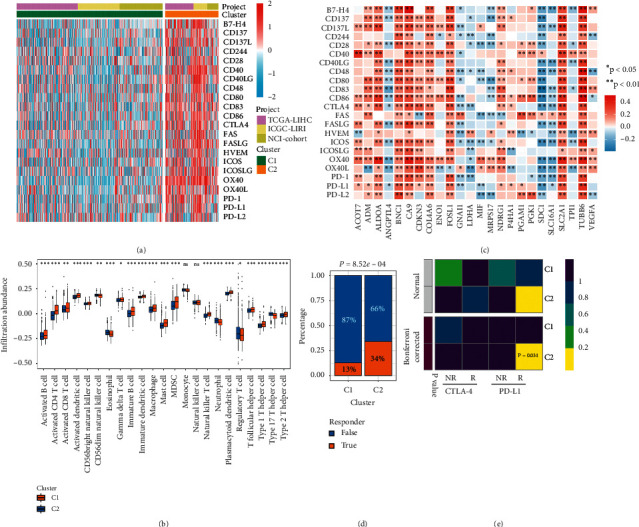
The difference of ICP expression, immune cells infiltration, and immunotherapy response between C1 and C2. (a) The expression heatmap of ICPs between C1 and C2 in metacohort. High expression, red; low expression, blue. (b) The abundance of 23 immune cell subsets infiltration was compared between the C1 and C2 in metacohort. ns, *P* > 0.05; ^*∗*^*P* < 0.05; ^*∗∗*^*P* < 0.01; ^*∗∗∗*^*P* < 0.001. (c) Correlations between immune checkpoints and HAGs in metacohort using Spearman analysis. Negative correlation was marked with blue, and positive correlation was marked with red. No asterisks represented no statistical significance; ^*∗*^*P* < 0.05; ^*∗∗*^*P* < 0.01. (d) Distribution of the immunotherapy response results predicted by TIDE algorithm between C1 and C2 in metacohort. Nonresponders, blue; responders, orange. (e) Submap analysis of the two subtypes and 47 previous melanoma patients with detailed immunotherapeutic information. NR represented nonresponders; R represented responders.

We further evaluated the immune checkpoints (ICPs) expression profile in the two hypoxia subtypes and found that C2 exhibited the higher expression level of ICPs, such as CTLA-4 and PD-L1 (Figures 4(a) and S3(a)). Consistent with this, C2 also displayed superior abundance of immune cell infiltration, such as CD4+ and CD8+ T cell (Figures 4(b) and S3(b)). These results implied that C2 might be more sensitive to immune checkpoint inhibitors (ICIs). In addition, HAGs exhibited significant correlations with ICPs and immune cells (e.g., B7-H4, CD4+ T cell, and CD8+ T cell) (Figures 4(c) and S3(c)). Subsequently, we applied the TIDE algorithm to assess the immunotherapy response. The results indicated C2 was almost three times the response of C1 (Figure 4(d)). We further utilized the submap algorithm to evaluate the similarity of the expression profile of two hypoxia subtypes and 47 pretreatment patients with the completely immunotherapy information [28, 35]. As expected, C2 was similar to patients with the treatment PD-L1 inhibitor (Bonferroni corrected *P* value = 0.031), which was in line with the high PD-L1 expression in C2 (Figure 4(e)).

### 3.4. The Clinical Characteristics of Two Hypoxia Subtypes

**Figure 5:**
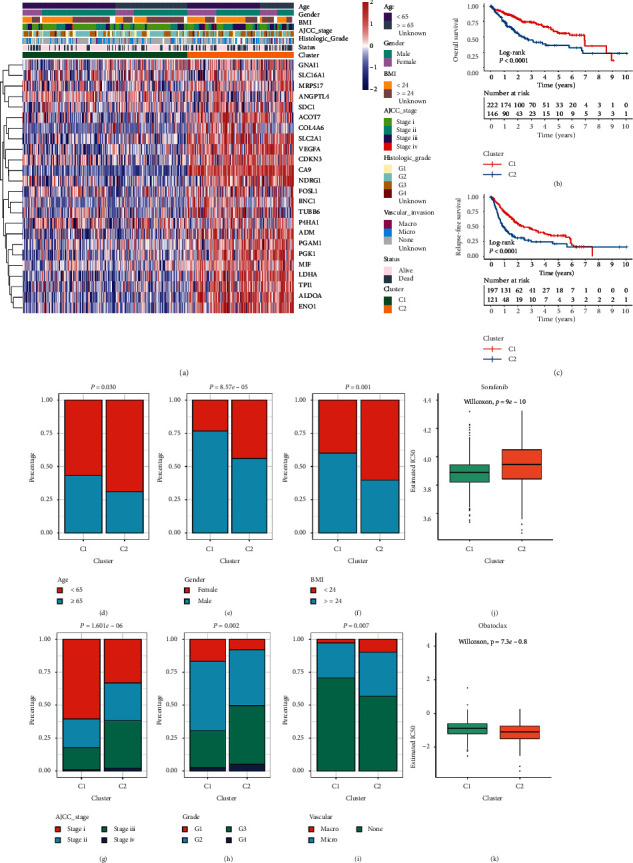
The clinical characteristics and prognosis of hypoxia subtypes in TCGA cohort. (a) The expression heatmap of 24 HAGs in TCGA-LIHC cohort. Survival status, age, gender, BMI, vascular invasion, histology grade, AJCC stage, and hypoxia subtypes were displayed as patient annotations. (b, c) Kaplan–Meier curves of OS (b) and RFS (c) between C1 and C2 in TCGA-LIHC cohort. (d–f) Composition percentage of clinical characteristics such as age (d), gender (e), and BMI (f) between C1 and C2. (g–i) Composition percentage of AJCC stage (g), grade (h), and vascular invasion (i) between C1 and C2. (j, k) Distribution of the estimated IC50 of sorafenib (j) and obatoclax (k) between C1 and C2 in TCGA-LIHC cohort.

Subsequently, we focused on the TCGA-LIHC cohort to further explore the clinical characteristics of two hypoxia subtypes due to it contained comprehensive omics data and clinical information. C2 had higher HAGs expression compared to C1 (e.g., CA9, VEGFA, and SLC2A1), exhibiting the high hypoxia pattern (Figure 5(a)). Apart from the molecular level, there were also differences in the distribution of clinicopathological characteristics and prognosis between the two subtypes. C1 with the low hypoxia pattern mainly presented in older, male, and higher BMI patients (Figures 5(d)–5(f)). C2 with the high hypoxia pattern mainly presented in patients with advanced AJCC stage, superior histological grade, and vascular invasion (Figures 5(g)–5(i)). These results indicated C2 was predominantly associated with malignant phenotype. The Kaplan–Meier analysis of OS and RFS suggested C1 had favorable prognosis (Figures 5(b) and 5(c)). In addition, the pRRophetic algorithm based on a linear ridge regression was further applied to estimate the sensitivity of each patient to sorafenib and obatoclax [29]. The half-maximal inhibitory concentration (IC50) was used to quantify the drug sensitivity, and a smaller IC50 indicated a higher drug sensitivity. The results revealed C1 was more sensitive to sorafenib compared to C2 (Figure 5(j)). Previous study demonstrated high hypoxia could limit the efficacy of sorafenib and cause tumor resistance by the massive expression of HAGs [36]. On the contrary, the high hypoxia pattern could enhance tumor cell responsiveness to obatoclax in HCC [37]. In line with this, C2 performed the stronger response to obatoclax (Figure 5(k)). Overall, the two hypoxia patterns demonstrated distinct molecular and clinical characteristics, and these results could guide personalized therapy and clinical management.

### 3.5. Genomic Alterations of Hypoxia Subtypes

**Figure 6:**
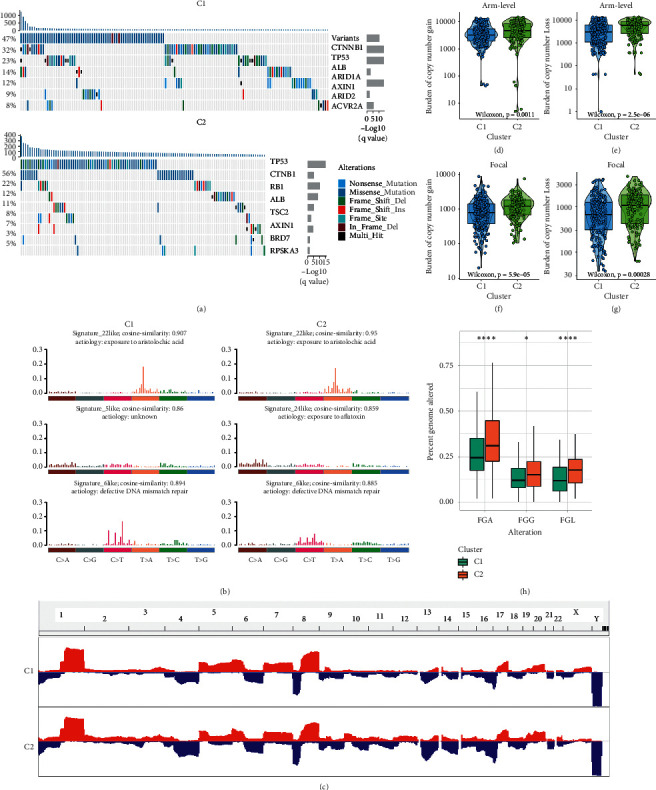
The genomic alterations of hypoxia subtypes in TCGA cohort. (a) Significantly mutated genes (SMGs) in two hypoxia subtypes. Each column represented individual patient. The upper bar plot showed TMB, and the percentage on the left showed the proportion of samples with mutations. The right bar plot indicated the MutSigCV q-value. The mutation alternations types were indicated by different colors. (b) Mutation signatures extracted from the two hypoxia subtypes. The three mutation signatures with the highest cosine similarity to COSMIC signatures exhibited in C1 and C2, respectively. The etiology of each signature and the cosine similarity between the original and the reconstructed mutation signatures were indicated. (c) Gain (red) or loss (blue) frequencies of copy number alterations (CNAs) in the chromosomes. (d–g) The burden of copy number gain (d) and loss (e) in arm level were compared between C1 and C2. The burden of copy number gain (f) and loss (g) in focal level were compared between C1 and C2. (h) The distribution of fraction genome altered (FGA), fraction genome gained (FGG), and fraction genome lost (FGL) in C1 and C2. ^*∗*^*P* < 0.05; ^*∗∗∗∗*^*P* < 0.0001.

C2 suggested the higher tumor mutation burden (TMB) trend compared with C1 although there was no pronounced difference in hypoxia subtypes (Figure S4(a)). Using MutSigCV algorithm, we identified 12 significantly mutated genes (SMGs) in two subtypes, and all genes had mutation rates greater than 5% (Figure 6(a) and Table S5). The mutation status of 12 SMGs significantly influenced their expression (Figure S4(b)). For example, the mutation group of TP53, AXIN1, BAP1, TSC2, and BRD7 had higher expression level compared to the wild group, whereas CTNNB1 and ALB were the opposite (Figure S4(b)). There were four common SMGs presented in both hypoxia subtypes, including CTNNB1, TP53, ALB, and AXIN1, implying these mutations were universal in HCC. Notably, C1 had dominant CTNNB1 mutation compared with C1 and specific SMGs associated with chromosome remodeling including ARID1A and ARID2. The key role in the cell cycle, TP53, and its mutations was more frequent in C2 relative to C1. Specific SMGs including RB1 and TSC2 in C2 also participated in the regulation of cell cycle. In addition, we further investigated the mutation signatures of HCC. The diverse mutation signatures reflected different carcinogenic processes. According to the NMF algorithm, three signatures were extracted in both subtypes and named based on COSMIC signatures by MutationalPatterns *R* package (Figures 6(b), S4(c), and S4(d)). In line with the higher TMB, C2 had superior proportion of signature 6, which represented defective DNA mismatch repair. Signature 24 associated with aflatoxin was also mainly displayed in C2, indicating a specific extrinsic carcinogenic process of C2. However, signature 5 was pertinent to age occupied the most proportion in C1, consistent with the dominant clinical features of C1 (Figures S4(e) and S4(f)).

We further applied GISTIC 2.0 software to decode the amplification and deletion of CNA on chromosomes (Figure 6(c) and Table S6). Compared to C1, we found C2 displayed higher burden of amplification and deletion at arm and focal level (Figures 6(d)–6(g)). In line with this, C2 also had superior FGA, FGG, and FGL (Figure 6(h)). This suggested C2 had superior genomic instability relative to C1. Consistent with previous study, the higher CNA load was associated with a higher immunogenicity and immune infiltration [38]. The recurrent CNAs in C2 included the amplification of 6q13 (CD109), 3q26.31(TNFSF10), 7q31.2(MET), and 8q24.21 (MYC) as well as the deletion of 10q23.31 (PTEN) and 8p23.2 (CSMD1). The specific CNA of C1 was mainly associated with cell proliferation, such as the amplification of 6p21.1 (VEGFA) and 7q21.3 (TAC1) as well as the deletion of 9p24.1 (JAK2). These results suggested the two subtypes had distinct CNA events, which not only might cause different immune infiltration but also promoted the target treatment of HCC.

### 3.6. Methylation Modification of Hypoxia Subtypes

**Figure 7:**
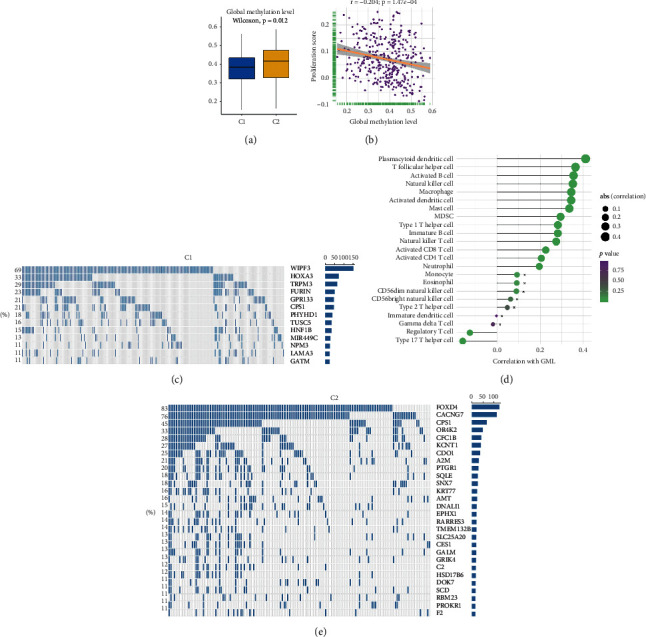
The DNA methylation modification of two hypoxia subtypes in TCGA-LIHC cohort. (a) The distribution of global methylation level (GML) in two hypoxia subtypes. (b) The correlation between GML and proliferation score. (c) Spearman correlation analysis between GML and 23 immune cells. The circle size represented the strength of correlation. (d, e) The methylation-driven genes (MDGs) in C1 (d) and C2 (e). Each column represented individual patients. The percentage on the left showed the proportion of samples in the whole that this gene was identified as an MDG. The right bar plot indicated the total number of samples identified as an MDG in each gene.

DNA methylation played an essential role in genes expression regulation. Global methylation levels (GMLs) referred to the large normally methylated genomic regions showing the systematic loss of DNA methylation [39]. In this research, C2 exhibited higher GMLs relative to C1 (Figure 7(a)). Previous study demonstrated that GMLs were negatively correlated with cell proliferation and positively correlated with CD8+ cell infiltration, and the methylation loss was associated with immune regulation, further affecting immunotherapy response [33]. Consistent with these, GMLs were significantly negatively correlated with proliferation score (Figure 7(b)). GMLs also exhibited positive correlation with immune cell infiltration, such as dendritic cell, B cell, and CD8+ T cell (Figure 7(c)). The difference of GMLs might impact the progression and immune status of tumor. Subsequently, we identified 13 and 28 methylation-driven genes (MDGs) in C1 and C2, respectively (Figures 7(d) and 7(e)). Liver-specific protein CPS1 was a common MDG in both subtypes. It was the first rate-limiting enzyme in urea cycle, involving tumor metabolism, while DNA methylation led to expression silencing of CPS1 in HCC [40]. Of note, WIPF3 was the most frequent MDG in C1. WIPF3 encoded actin cytoskeleton protein, impacting actin remodeling and cellular invasion [41], thus the silencing of WIPF3 promoted tumor growth. The suppressors FOXD4 and CDO1 were specific MDGs in C2. FOXD4 participated in immune system regulation, tumor progression, and metastasis by methylation modification [42]. Besides, it was reported that the promoter methylation of CDO1 might be a common event in human carcinogenesis [43]. The silencing of FOXD4 and CDO1 might accelerate carcinogenesis and development in HCC. Collectively, the two subtypes had specific MDGs, which might represent potential molecular target and promote the progression of the heterogeneity in HCC.

### 3.7. Development and Validation of a Hypoxia-Associated Risk Score

**Figure 8:**
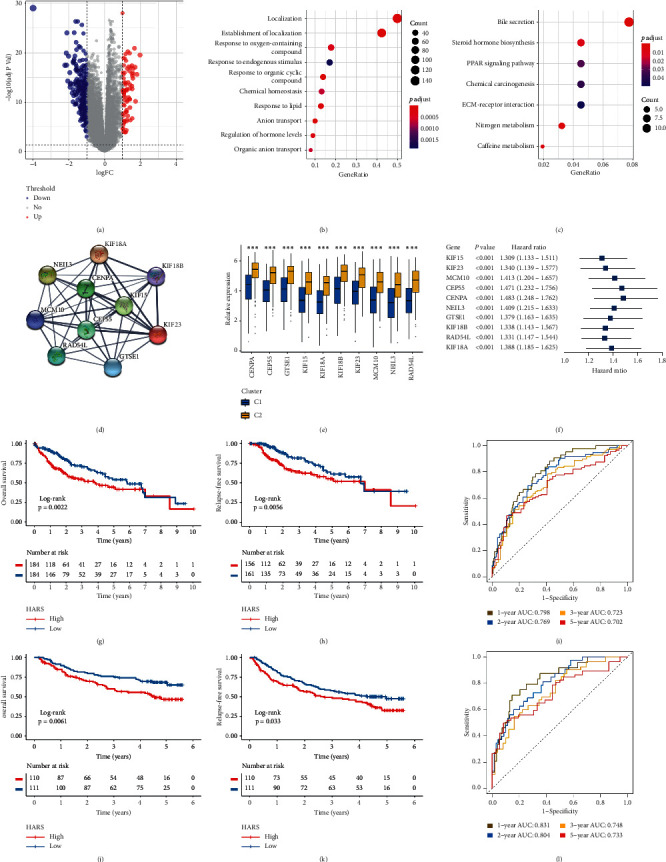
The development and validation of HARS in TCGA-LIHC and NCI cohorts. (a) Volcano plot displayed the differentially expressed genes (DEGs) between the two subtypes. Red dots represented upregulated genes, blue dots represented downregulated genes, and grey dots represented genes with no significance. (b, c) GO (b) and KEGG (c) enrichment analysis of 299 DEGs. The significantly biological functions were extracted with adjusted *P* value < 0.05. (d) Using MCODE plug-in of Cytoscape software, we extracted a key module including 10 genes. (e) The expression difference of 10 key genes between C1 and C2. ^*∗∗∗*^*P* < 0.001. (f) Univariate Cox regression analysis revealed the prognosis significance of 10 key genes, all of them were risk factors. (g, h) Kaplan–Meier curves for OS (g) and RFS (h) between low HARS and high HARS groups in TCGA-LIHC cohort. (i) Estimation of the prognosis prediction by receiver operating characteristic curve (ROC) in TCGA-LIHC cohort. (j, k) Kaplan–Meier curves for OS (j) and RFS (k) between low HARS and high HARS groups in NCI cohort. (l) Estimation of the prognosis prediction by ROC in NCI cohort.

A total of 299 DEGs were identified between C1 and C2, including 80 upregulated and 219 downregulated genes (Figure 8(a) and Table S7). GO and KEGG enrichment analysis suggested DEGs were mainly associated with molecular transport and metabolism, such as anion transport, bile secretion, and nitrogen metabolism (Figures 8(b) and 8(c)). According to these DEGs, we constructed protein-protein interaction (PPI) networks and further extracted a key module. The top 10 genes from MCC algorithm were filtered out as key genes (Figure 8(d)), which might play an essential role in the hypoxia pattern of HCC. Notably, it contained four kinesin family members. According to the previous literatures, KIF15 promoted the development of HCC by reactive oxygen species imbalance, while KIF18B advanced HCC progression through activating WNT pathway [44, 45]. We also found that all key genes had a higher expression in C2 (Figure 8(e)). The univariate Cox regression analysis further revealed the high expression of all key genes was significantly associated with unfavorable prognosis (Figure 8(e)). Subsequently, we evaluated the accuracy of 299 DEGs for predicting prognosis by ROC (Table S8). Interestingly, the top AUC value of nine genes were all from the key genes screened through MCC algorithm, and another key gene, RAD54L, ranked 11th (Table S8). These results not only indicated that these genes might be involved in shaping the hypoxia pattern of TME but also were not negligible for predicting the prognosis of HCC. Thus, we applied the principal component analysis (PCA) algorithm to develop a risk signature based on the 10 key genes. Principal component 1 was extracted to serve as the gene signature score. The gene signature score was named hypoxia-associated risk score (HARS). We calculated the HARS of each patient in TCGA, NCI, and ICGC cohorts and further classified HCC patients into high-risk group and low-risk group according to the median value of HARS. It turned out the low-risk group displayed favorable OS and RFS compared to high-risk group (Figures 8(g), 8(h), 8(j), 8(k), and S5(a)). In TCGA cohort, the AUCs of 1-, 2-, 3-, and 5-year prognostic prediction for HARS were 0.798, 0.769, 0.723, and 0.702, respectively (Figure 8(i)). In NCI cohort, 1-, 2-, 3-, and 5-year AUCs were 0.831, 0.804, 0.748, and 0.733, respectively (Figure 8(l)). In ICGC cohort, 1-, 2-, 3-, and 5-year AUCs were 0.771, 0.731, 0.725, and 0.714, respectively (Figure S5(b)). The above results demonstrated HARS performed well at prognostic prediction and displayed potential clinical value. In addition, based on the nearest centroid method and Pearson correlation, we developed a *R* package termed HCCS (https://github.com/Zaoqu-Liu/HCCS). The pipeline could divide each sample into the corresponding hypoxia subtype and calculate the HARS of each sample.

### 3.8. Two Nomograms for Assessing OS and RFS

**Figure 9:**
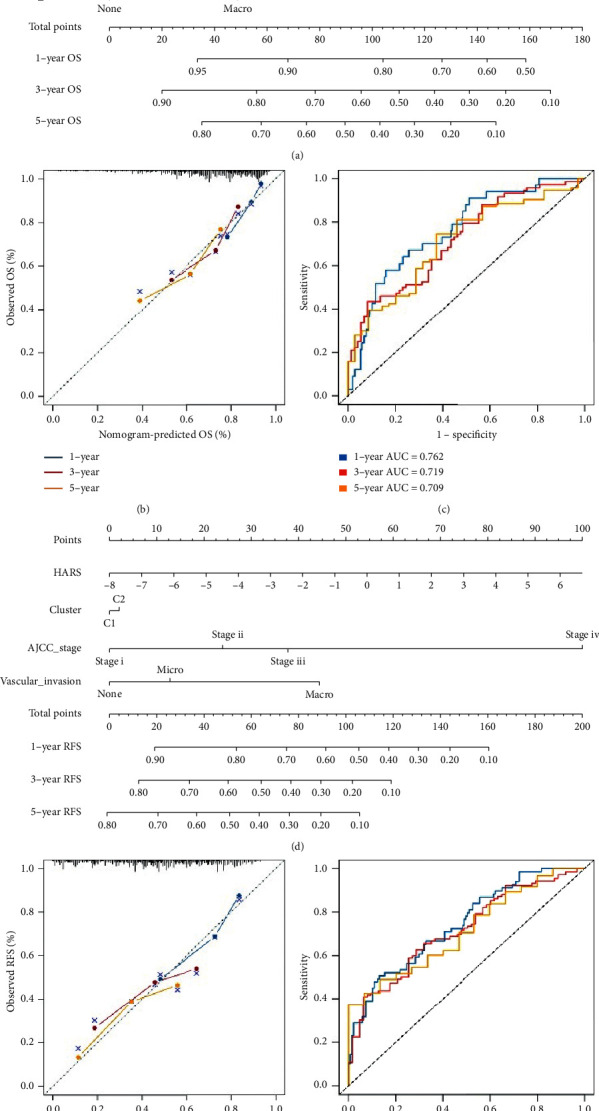
The construction of two nomograms predicting OS and RFS, respectively. (a–c) Nomogram for predicting the 1-, 3-, and 5-year OS of HCC patients (a). Calibration (b) and ROC curve (c) for evaluating the performance of nomogram predicting the 1-, 3-, and 5-year OS for HCC patients. (d–f) Nomogram for predicting the 1-, 3-, and 5-year RFS of HCC patients (d). Calibration (e) and ROC curve (f) for evaluating the performance of nomogram predicting the 1-, 3-, and 5-year RFS for HCC patients. Blue line, 1-year; red line, 3-year; yellow line, 5-year.

To facilitate clinical management of HARS, we also recruited the clinical features encompassing hypoxia subtypes, age, gender, histological grade, AJCC stage, and vascular invasion, and planning to develop a feasible nomogram. We found the HARS was an independent prognostic factor for both OS and RFS (Table S9). Moreover, univariate Cox analysis indicated both gender and histological grade had no prognostic significance for OS, and age, gender, as well as histological grade had no prognostic significance for RFS (Table S9). To facilitate clinical applicability, we included HARS and clinical features with univariate Cox *P* < 0.05 to develop two nomograms for predicting 1-, 3-, and 5-year OS and RFS, respectively (Figures 9(a) and 9(d)). Then, the calibration curve, Harrell's concordance index (C-index), and ROC were applied to assess the predictive ability of the two nomograms. The calibration curves for predicting 1-, 3-, and 5-year OS and RFS exhibited great agreement between expected value and actual observation (Figures 9(b) and 9(e)). The C-index was 0.714 (95% confidence interval [CI]: 0.65–0.78) and 0.713 (95% CI: 0.67–0.76) for the nomogram predicting OS and RFS. Besides, the 1-, 3-, and 5-year AUC values for the OS relevant- and RFS relevant-nomogram were 0.762, 0.719, and 0.709 and 0.743, 0.715, and 0.712, respectively (Figures 9(c) and 9(f)). The above indicated the two nomograms were reliable and promising clinical management tools, which could assist in making clinical decisions and recommendations.

### 3.9. Immunotherapy Response Prediction Based on HARS

**Figure 10:**
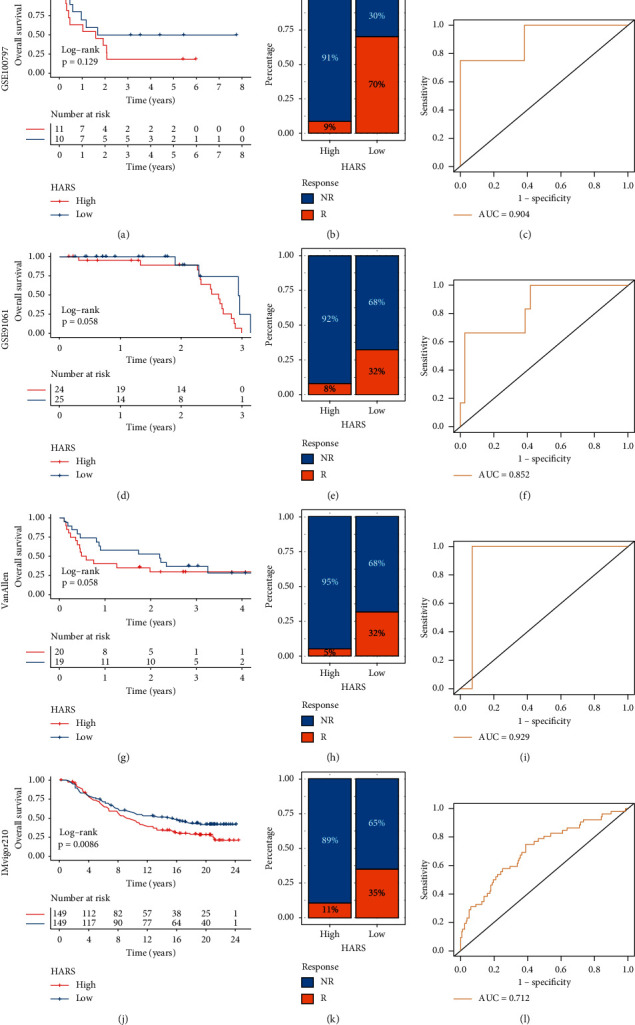
The performance of HARS for predicting the immunotherapy response. (a–c) The Kaplan–Meier analysis (a), immunotherapy response ratio (b), and ROC curve (c) of HARS in GSE100797 cohort. (d–f) The Kaplan–Meier analysis (d), immunotherapy response ratio (e), and ROC curve (f) of HARS in GSE91061 cohort. (g-i) The Kaplan–Meier analysis (g), immunotherapy response ratio (h), and ROC curve (i) of HARS in VanAllen cohort. (j–l) The Kaplan–Meier analysis (j), immunotherapy response ratio (k), and ROC curve (l) of HARS in IMvigor210 cohort.

As illustrated, HARS not only exhibited dominant correlation with ICPs such as CD80, CTLA4, PD-1, PD-L1, and FAS but also was significantly associated with Th2, Th17, CD4+ T cell, and natural killer T cell (Figures S6(a) and S6(b)). These results suggested the HARS might be a potential immunotherapy marker. Therefore, we further detected the clinical value of HARS in predicting the response to immunotherapy in four independent cohorts. Consistent with the previous results, high HARS decreased OS although three cohorts had no statistical significance (GSE100797: *P* = 0.129; GSE91061: *P* = 0.058; VanAllen cohort: *P* = 0.088), which might be due to the small sample size (Figures 10(a), 10(d), and 10(g)). For IMvigor210 cohort encompassed 298 eligible samples, the high HI groups exhibited significant unfavorable prognosis with *P* = 0.0086 (Figure 10(j)). In addition, it was exciting that the low HARS groups displayed superior response rate to immunotherapy, which was 3–6 times higher compared to the high HARS groups (GSE100797 : 64% vs 20%; GSE91061 : 32% vs 8%; VanAllen cohort: 30% vs 5%; IMvigor210 cohort: 30% vs 10%) (Figures 10(b), 10(e), 10(h), and 10(k)). The ROC was used to evaluate the accuracy of immunotherapy prediction based on HARS. We observed HARS affords a greater accuracy in the prediction of immunotherapeutic benefits, and the AUC values of GSE100797, GSE91061, VanAllen cohort, and IMvigor210 cohort were 0.904, 0.852, 0.929, and 0.712, respectively (Figures 10(c), 10(f), 10(i), and 10(l)). The above indicated HARS had the excellent performance for predicting immunotherapy response, and it was a reliable and promising immunotherapy marker.

## 4. Discussion

Hypoxia was a typical microenvironment characteristic in HCC and played an indispensable role in the progression, metastasis, treatment tolerance, and prognosis of tumor [6, 23]. In this research, we observed most of HAGs displayed aberrant expression and significant prognosis value in HCC. The CNA and methylation might have dominant regulation on HAGs relative to mutation. Therefore, according to the expression of HAGs, we decoded the two hypoxia subtypes in both metacohort and TCGA-LIHC cohort, C1 with the low hypoxia pattern and C2 with the high hypoxia pattern. The two hypoxia subtypes were characterized by distinct biological characteristics, immune cell infiltrations, ICPs expression, genomic drivers, and clinical outcomes. These results boosted the understanding of hypoxia pattern and facilitated the deciphering of molecular characteristics and precise treatment in HCC. In addition, we developed a risk signature and two nomograms to advance clinical management and immunotherapy.

Hypoxia was a significant barrier to antineoplastic therapy and a major contributor to immunotherapy resistance [13, 23]. Hypoxia had profound impacts on immune cells infiltration, angiogenesis, and even the decreasing drug penetration, ultimately giving rise in therapy resistance [6]. In our research, we observed C2 has a better response to immunotherapy via the prediction of TIDE and submap algorithm. C1 was characterized by the inferior infiltration of immune cells and fibroblasts cells, low expression of ICPs, and high metabolic activity. Previous study suggested that high metabolism activities disordered TME and eventually impaired the immune cell function [46]. Therefore, high metabolism activities as well as the absence of immune cells and ICPs in C1 might contribute to the unfavorable responses to immunotherapy. Conversely, due to the superior level of immune cells infiltration and ICP expression, C2 was significantly sensitive to immunotherapy such as anti-PD-L1. Notably, hypoxia could enhance the suppressive activities of immunosuppressive cells and decrease the cytotoxic effects of lymphocytes in TME [16]. Nonetheless, C2 still has more advantages for immunotherapy than C1 lacking immune cells. Overall, we recommended patients in the C2 subtype take consideration for immunotherapy. Immune infiltration was crucial to the tumor progression, indirectly affecting the prognosis of HCC patients by regulating the TME. For, example, MDSCs, macrophage, and mast cell were associated with short survival in HCC [1]. Interestedly, these cells were found to be highly expressed in C2, which might explain its adverse OS and RFS. In addition, genomic alternations such as mutation, CNA, and methylation modification had important connection with responsiveness to targeted drugs and clinical outcomes of HCC patients [2]. The two hypoxia subtypes showed distinct genomic alterations, which might be driving factors in the formation of different subtypes, prognostic predictors, and potential drug targets. C1 was mainly characterized by the driven mutations of CTNNB1, TP53, ARID1A, and ARID2. Previous studies demonstrated the mutation of CTNNB1 was a feature of “immune excluded subtype” in HCC, which displayed the tight correlation with T cell exclusion and ineffective immunotherapy by enhancing the activation of WNT signaling pathway [3]. Chromatin remodeling factors ARID1A and ARID2 were specific SMGs in C1. The mutation of ARID2 strengthened the cytotoxicity of T cell and improved clinical benefits of patients with massive immune cells infiltration [4]. In addition, C1 with the amplification of VEGFA was conducive to the formation of immunosuppressive microenvironment and induced T cell exhaustion, which eventually leads to immune escape [5]. These genomic-driven events might contribute to the immunodeficiency and immunotherapy resistance of C1. Furthermore, both C1 and C2 showed massive methylation modification which might contribute to tumorigenesis. Especially, in C2, the burden of methylation level was higher. With the development of epigenetics, methylation modification was responsible for caner initiation and methylation sites could be potential therapy targets [6]. For example, the methylation modification of CDO1 was common in multiple human cancer and the methylation silencing of CDO1 increased tumor cells growth [7]. Targeted the CDO1 might accomplish the precise therapy, making impressive effects. The high CNA burden was dominated genomic characteristic of C2, such as the amplification of 7q31.2 (MET) and 8q24.21 (MYC). Previous study demonstrated the high-level amplification of MET has driven the tumorigenesis and conferred an adverse prognosis [8]. MYC amplification improved the RUVBL2 expression, while the overexpression of RUVBL2 exhibited shorter RFS by regulating AKT and ERK/MAPK pathway in HCC [9]. RB1 identified as the first tumor suppressor gene in human history was specific SMG in C2. The mutation of RB1 was a characteristic of HCC patients who had high levels of drosophila prune protein (h-prune) [10]. High expression of h-prune promoted the tumor metastasis and performed the adverse OS and RFS [10]. Therefore, these molecular alterations further proved the C2 presented the poor OS and RFS. Overall, we had uncovered distinct genomic alterations, methylation modification, and immune in clinical performance between C1 and C2. The insensitive immunotherapy of C1 and inferior prognosis of C2 were deeply explained. Moreover, the comprehensive explorations of genomic alternations provided vital theoretical supports for precise treatment of HCC.

The hypoxia subtypes also demonstrated heterogeneous clinical outcomes. Compared to C1, the C2 displayed poor sensitivity to sorafenib. However, the antiangiogenic function of sorafenib led to intratumoral hypoxia, increasing the number of adapted hypoxia cells [53]. Therefore, the high hypoxia pattern could limit the efficacy of sorafenib, further giving rise to the poor sensitivity of C2 [36]. Moreover, C2 exhibited more sensitivity to obatoclax, which might be due to obatoclax promoted tumor cells apoptosis by antagonizing WNT/*β*-catenin signaling that significantly was enriched in C2 [54]. In addition, we developed a risk signature termed HARS. The HARS performed excellent at predicting the prognosis and was an independent risk prognosis signature for OS and RFS in HCC. Meanwhile, the HARS was also a robust indicator for immunotherapy, and the efficacy was verified in four independent cohorts. Besides, combining the HARS and vital clinical features, two nomograms with good performance were also constructed for assessing the possibility of OS and RFS to facilitate clinical management. The nomograms could be applied to provide more precise and individualized evaluation of OS and RFS for HCC patients. Eventually, based on the nearest centroid method and Pearson correlation, we developed a *R* package termed HCCS (https://github.com/Zaoqu-Liu/HCCS). The pipeline could divide each sample into the corresponding hypoxia subtype and calculate the HARS of each sample.

The study still had some limitations. Firstly, we indirectly evaluated the relative hypoxia status of each patients based on the expression of HAGs. Secondly, the prediction of HARS response to immunotherapy was verified based on four retrospective cohorts, but prospective cohort studies are still necessary.

## 5. Conclusions

We revealed two hypoxia subtypes with distinct biological function, immune cell infiltrations, ICPs expression, genomic driven events, and clinical outcomes in HCC. The HARS was a robust and promising indicator for prognosis and immunotherapy, and two nomograms were proposed to advance the prognosis assessment of HCC patients. These results enhanced the acknowledgment of hypoxia heterogeneity in HCC and facilitated individual therapy and clinical management.

## Data Availability

Publicly available datasets were analyzed in this study. These data can be found in The Cancer Genome Atlas (TCGA, https://portal.gdc.cancer.gov/), International Cancer Genome Consortium portal (ICGC, https://dcc.icgc.org/), and Gene Expression Omnibus (GEO, http://www.ncbi.nlm.nih.gov/geo/).

## Supplementary Materials

Supplementary Materials Figure S1: the identification of molecular subtypes in metacohort. (a) Using the unsupervised clustering algorithm to classify patients into different molecular subtypes in metacohort. The consensus score matrix of 831 HCC samples (*K* = 2∼9). A higher consensus score between two samples indicates they were more likely to be grouped into the same cluster in different iterations. The figure demonstrated *k* = 2 was the best choice. (b) The proportion of ambiguous clustering (PAC) score, a low value of PAC implied a flat middle segment in cumulative distribution functions (CDFs), allowing conjecture of the optimal *k* (*k* = 2) by the lowest PAC. (c) Recommended number of clusters using 26 criteria of Nbclust package in the metacohort. The highest of the column represented the optimal *k* (*k* = 2). Figure S2: the differences of HAG expression, known signatures, and fibroblast infiltration between C1 and C2 in metacohort. (a) The expression heatmap of 24 HAGs between C1 and C2. High expression, red; low expression, blue. (b) The expression box plot of 24 HAGs between C1 and C2. (c) Comparison of the signatures score between C1 and C2. (d) The abundance of fibroblasts was compared between C1 and C2. ns, *P* > 0.05; ^*∗*^*P* < 0.05; ^*∗∗*^*P* < 0.01; ^*∗∗∗*^*P* < 0.001. Figure S3: the difference of immune checkpoints (ICPs) and immune cells between C1 and C2. (a) The expression boxplot of ICPs between C1 and C2. ns, *P* > 0.05; ^*∗*^*P* < 0.05; ^*∗∗*^*P* < 0.01; ^*∗∗∗*^*P* < 0.001. (b) The heatmap of 23 immune cells between C1 and C2. High expression, red; low expression, blue. (c) Correlations between immune cells and HAGs using Spearman analysis. Negative correlation was marked with blue, and positive correlation was marked with red. No asterisks represented no statistical significance; ^*∗*^*P* < 0.05; ^*∗∗*^*P* < 0.01. Figure S4: the mutation signatures and significantly mutated genes (SMGs) in TCGA-LIHC cohort. (a) Comparison of tumor mutation burden (TMB) between C1 and C2. (b) The expression difference of 12 SMGs between mutation group and wild group. Mutation group, blue; wild group, yellow. (c, d) Using NMF package, the mutational signatures were extracted from the mutation count matrix. The optimal rank was both 3 (c, d). (e, f) The pie chart showed the proportion of mutation signatures in C1 (e) and C2 (f), respectively. Figure S5: the Kaplan–Meier and ROC analysis of HARS in ICGC cohort. (a) Kaplan–Meier curves for OS between low HARS and high HARS groups in ICGC cohort. (b) Estimation of the prognosis prediction by receiver operating characteristic curve (ROC) in ICGC cohort. Figure S6: correlations of HARS with immune checkpoints and immune cells. (a) Spearman correlation analysis between HARS and immune checkpoints. (b) Spearman correlation analysis between HARS and immune cells. The circle size represents the strength of correlation. Table S1: the details of seven datasets enrolled in this study. Table S2: a total of 24 hypoxia-associated genes (HAGs) were recruited in our research. Table S3: the known gene signatures enrolled in this study. Table S4: the gene sets for marking 23 immune cell types and fibroblasts. Table S5: the mutation-driven genes from MutSigCV algorithm in C1 and C2. ^*∗*^SMGs are marked in red; the common SMGs are marked with bold. Table S6: the significant focal copy number alterations (including amplification and deletion) of each subtype. Table S7: a total of 299 subtype-related differentially expressed genes (DEGs). ^*∗*^DEGs are marked in red. Table S8: the univariable Cox regression and ROC analysis of each DEGs. ^*∗*^The key genes are marked in red. Table S9: the Cox regression analysis of overall survival (OS) and relapse-free survival (RFS). ^*∗*^The factors with univariable Cox *P* value <0.05 are marked in green, and the factors with multivariable Cox *P* value <0.05 are marked in red.
