# Supplementary material for: Hypoxia Molecular Characterization in Hepatocellular Carcinoma Identifies One Risk Signature and Two Nomograms for Clinical Management
Source: J Oncol. 2021 Jan 20;2021:6664386. doi: 10.1155/2021/6664386 (PMC7846409; doi:10.1155/2021/6664386)
Supplement: Supplementary Materials — Figure S1: the identification of molecular subtypes in metacohort. (a) Using the unsupervised clustering algorithm to classify patients into different molecular subtypes in metacohort. The consensus score matrix of 831 HCC samples (K = 2∼9). A higher consensus score between two samples indicates they were more likely to be grouped into the same cluster in different iterations. The figure demonstrated k = 2 was the best choice. (b) The proportion of ambiguous clustering (PAC) score, a low value of PAC implied a flat middle segment in cumulative distribution functions (CDFs), allowing conjecture of the optimal k (k = 2) by the lowest PAC. (c) Recommended number of clusters using 26 criteria of Nbclust package in the metacohort. The highest of the column represented the optimal k (k = 2). Figure S2: the differences of HAG expression, known signatures, and fibroblast infiltration between C1 and C2 in metacohort. (a) The expression heatmap of 24 HAGs between C1 and C2. High expression, red; low expression, blue. (b) The expression box plot of 24 HAGs between C1 and C2. (c) Comparison of the signatures score between C1 and C2. (d) The abundance of fibroblasts was compared between C1 and C2. ns, P > 0.05; ∗P < 0.05; ∗∗P < 0.01; ∗∗∗P < 0.001. Figure S3: the difference of immune checkpoints (ICPs) and immune cells between C1 and C2. (a) The expression boxplot of ICPs between C1 and C2. ns, P > 0.05; ∗P < 0.05; ∗∗P < 0.01; ∗∗∗P < 0.001. (b) The heatmap of 23 immune cells between C1 and C2. High expression, red; low expression, blue. (c) Correlations between immune cells and HAGs using Spearman analysis. Negative correlation was marked with blue, and positive correlation was marked with red. No asterisks represented no statistical significance; ∗P < 0.05; ∗∗P < 0.01. Figure S4: the mutation signatures and significantly mutated genes (SMGs) in TCGA-LIHC cohort. (a) Comparison of tumor mutation burden (TMB) between C1 and C2. (b) The expression difference of 12 SMGs between muta [file 6664386.f1.zip › 6664386.f1/Table S1.docx]

| **Table S1: The details of seven datasets enrolled in this study.** | | | | | |
| --- | --- | --- | --- | --- | --- |
| **Accession number** | **Platform** | **Number**  **of** | **PMID** | **Survival**  **information** | **Immunotherapy**  **information** |
| TCGA-LIHC | Illumina RNAseq | 374 |  | Yes | No |
| ICGC-LIRI-JP | Illumina RNAseq | 210 |  | Yes | No |
| GSE14520 | Affymetrix Human Genome U133A Array  Affymetrix Human Genome U133A 2.0 Array | 247 | 19914252 | Yes | No |
| GSE100797 | Almac Diagnostics Custom Xcel array | 21 | 29170503 | Yes | Yes |
| GSE91061 | Affymetrix Human Genome U133 Plus 2.0 Array | 49 | 29033130 | Yes | Yes |
| VanAllen cohort | Affymetrix Human Genome U133 Plus 2.0 Array | 39 | 26359337 | Yes | Yes |
| IMvigor210 | Affymetrix Human Genome U133 Plus 2.0 Array | 298 | 29443960 | Yes | Yes |
