# Supplementary material for: Hypoxia Molecular Characterization in Hepatocellular Carcinoma Identifies One Risk Signature and Two Nomograms for Clinical Management
Source: J Oncol. 2021 Jan 20;2021:6664386. doi: 10.1155/2021/6664386 (PMC7846409; doi:10.1155/2021/6664386)
Supplement: Supplementary Materials — Figure S1: the identification of molecular subtypes in metacohort. (a) Using the unsupervised clustering algorithm to classify patients into different molecular subtypes in metacohort. The consensus score matrix of 831 HCC samples (K = 2∼9). A higher consensus score between two samples indicates they were more likely to be grouped into the same cluster in different iterations. The figure demonstrated k = 2 was the best choice. (b) The proportion of ambiguous clustering (PAC) score, a low value of PAC implied a flat middle segment in cumulative distribution functions (CDFs), allowing conjecture of the optimal k (k = 2) by the lowest PAC. (c) Recommended number of clusters using 26 criteria of Nbclust package in the metacohort. The highest of the column represented the optimal k (k = 2). Figure S2: the differences of HAG expression, known signatures, and fibroblast infiltration between C1 and C2 in metacohort. (a) The expression heatmap of 24 HAGs between C1 and C2. High expression, red; low expression, blue. (b) The expression box plot of 24 HAGs between C1 and C2. (c) Comparison of the signatures score between C1 and C2. (d) The abundance of fibroblasts was compared between C1 and C2. ns, P > 0.05; ∗P < 0.05; ∗∗P < 0.01; ∗∗∗P < 0.001. Figure S3: the difference of immune checkpoints (ICPs) and immune cells between C1 and C2. (a) The expression boxplot of ICPs between C1 and C2. ns, P > 0.05; ∗P < 0.05; ∗∗P < 0.01; ∗∗∗P < 0.001. (b) The heatmap of 23 immune cells between C1 and C2. High expression, red; low expression, blue. (c) Correlations between immune cells and HAGs using Spearman analysis. Negative correlation was marked with blue, and positive correlation was marked with red. No asterisks represented no statistical significance; ∗P < 0.05; ∗∗P < 0.01. Figure S4: the mutation signatures and significantly mutated genes (SMGs) in TCGA-LIHC cohort. (a) Comparison of tumor mutation burden (TMB) between C1 and C2. (b) The expression difference of 12 SMGs between muta [file 6664386.f1.zip › 6664386.f1/Table S5.docx]

| **Table S5: The mutation driven genes from MutSigCV algorithm in C1 and C2.**  **** SMGs are marked with bold.*** | | | | | | |
| --- | --- | --- | --- | --- | --- | --- |
| **C1** | |  | **C2** | | | |
| **Gene** | **P-value** | **Q-value** |  | **Gene** | **P-value** | **Q-value** |
| **ALB** | 0 | 0 |  | **TP53** | 0 | 0 |
| **AXIN1** | 0 | 0 |  | **RB1** | 6.66E-15 | 6.28E-11 |
| **TP53** | 0 | 0 |  | **BAP1** | 6.46E-13 | 4.06E-09 |
| **CTNNB1** | 8.22E-15 | 3.87E-11 |  | **CTNNB1** | 3.05E-09 | 1.44E-05 |
| **ACVR2A** | 8.00E-10 | 3.02E-06 |  | **ALB** | 3.93E-09 | 1.48E-05 |
| **ARID1A** | 2.96E-07 | 9.29E-04 |  | **AXIN1** | 1.78E-08 | 5.59E-05 |
| **ARID2** | 2.43E-06 | 6.54E-03 |  | **TSC2** | 1.10E-06 | 2.98E-03 |
| EEF1A1 | 1.12E-04 | 2.58E-01 |  | **BRD7** | 1.34E-05 | 3.16E-02 |
| KEAP1 | 1.23E-04 | 2.58E-01 |  | **RPS6KA3** | 2.81E-05 | 4.89E-02 |
| GABRG1 | 1.73E-04 | 3.26E-01 |  | GJB1 | 2.21E-04 | 4.17E-01 |
| BAP1 | 1.94E-04 | 3.33E-01 |  | SAA1 | 3.11E-04 | 4.99E-01 |
| CDKN2A | 2.17E-04 | 3.42E-01 |  | CDKN1A | 3.17E-04 | 4.99E-01 |
| NFE2L2 | 4.48E-04 | 6.50E-01 |  | KEAP1 | 4.23E-04 | 6.14E-01 |
| ZNF687 | 4.83E-04 | 6.51E-01 |  | SOCS6 | 1.02E-03 | 1 |
| HNF1A | 5.38E-04 | 6.76E-01 |  | UBE2D3 | 1.41E-03 | 1 |
| RB1 | 5.80E-04 | 6.84E-01 |  | STXBP3 | 1.59E-03 | 1 |
| GADL1 | 6.89E-04 | 7.60E-01 |  | FHOD3 | 1.70E-03 | 1 |
| LDOC1L | 7.26E-04 | 7.60E-01 |  | HIST1H4C | 1.94E-03 | 1 |
| SLC30A1 | 8.12E-04 | 8.07E-01 |  | COL2A1 | 1.98E-03 | 1 |
| LCE1E | 8.80E-04 | 8.30E-01 |  | CRIP3 | 2.71E-03 | 1 |
| CCDC137 | 9.31E-04 | 8.36E-01 |  | OR51G1 | 2.81E-03 | 1 |
| CDKN1B | 9.87E-04 | 8.46E-01 |  | KISS1 | 2.93E-03 | 1 |
| KRTAP22 | 1.20E-03 | 9.87E-01 |  | TIRAP | 2.97E-03 | 1 |
| CISH | 1.38E-03 | 1 |  | C4orf45 | 3.07E-03 | 1 |
| PRELID1 | 1.61E-03 | 1 |  | IDH1 | 3.20E-03 | 1 |
| RAB42 | 1.65E-03 | 1 |  | RPS9 | 3.22E-03 | 1 |
| TMEM33 | 1.86E-03 | 1 |  | ADRB3 | 3.29E-03 | 1 |
| NRAS | 1.90E-03 | 1 |  | HAGHL | 3.45E-03 | 1 |
| UPK3B | 1.99E-03 | 1 |  | PLA2G3 | 3.50E-03 | 1 |
| RASA1 | 2.00E-03 | 1 |  | FBXO42 | 3.50E-03 | 1 |
| SCAMP4 | 2.79E-03 | 1 |  | EIF4E1B | 3.53E-03 | 1 |
| C19orf48 | 2.87E-03 | 1 |  | DNAJB8 | 3.55E-03 | 1 |
| FILIP1 | 3.18E-03 | 1 |  | FAM168B | 4.63E-03 | 1 |
| IFITM3 | 3.27E-03 | 1 |  | UBD | 5.72E-03 | 1 |
| MRPL49 | 3.29E-03 | 1 |  | INSIG1 | 6.10E-03 | 1 |
| UBE2D3 | 3.55E-03 | 1 |  | CCDC53 | 6.14E-03 | 1 |
| PHGR1 | 3.77E-03 | 1 |  | HIST1H2B | 6.15E-03 | 1 |
| CIRBP | 3.82E-03 | 1 |  | RAB18 | 6.20E-03 | 1 |
| GOSR2 | 3.88E-03 | 1 |  | S100A7A | 6.28E-03 | 1 |
| KCNE3 | 3.95E-03 | 1 |  | TATDN3 | 6.75E-03 | 1 |
| MAP2K4 | 4.08E-03 | 1 |  | OPN4 | 6.99E-03 | 1 |
| UPF3B | 4.24E-03 | 1 |  | TINAG | 7.92E-03 | 1 |
| PLP1 | 4.39E-03 | 1 |  | NFATC2 | 8.15E-03 | 1 |
| CNBP | 4.40E-03 | 1 |  | PTEN | 8.15E-03 | 1 |
| MELK | 4.73E-03 | 1 |  | GABRA6 | 8.34E-03 | 1 |
| ZNF3 | 4.79E-03 | 1 |  | PRKDC | 8.41E-03 | 1 |
| DCDC1 | 4.86E-03 | 1 |  | EMP2 | 8.51E-03 | 1 |
| TNPO1 | 4.93E-03 | 1 |  | CREB3L3 | 8.54E-03 | 1 |

| STON1 | 5.22E-03 | 1 | PXMP4 | 8.78E-03 | 1 |
| --- | --- | --- | --- | --- | --- |
| PTP4A3 | 5.25E-03 | 1 | TBC1D26 | 8.86E-03 | 1 |
| CXorf56 | 5.32E-03 | 1 | ERCC3 | 8.95E-03 | 1 |
| CDKN1A | 5.38E-03 | 1 | RAB3C | 8.95E-03 | 1 |
| SIAH1 | 5.44E-03 | 1 | SH2B3 | 8.98E-03 | 1 |
| GMPPB | 5.57E-03 | 1 | TOB1 | 9.42E-03 | 1 |
| SLC26A7 | 5.87E-03 | 1 | H2AFY | 9.78E-03 | 1 |
| PSD2 | 5.96E-03 | 1 | PDLIM3 | 1.01E-02 | 1 |
| GRXCR1 | 6.15E-03 | 1 | FRZB | 1.05E-02 | 1 |
| TRPA1 | 6.17E-03 | 1 | MLST8 | 1.07E-02 | 1 |
| GPS2 | 6.28E-03 | 1 | OR10G8 | 1.08E-02 | 1 |
| HIST1H4K | 6.32E-03 | 1 | CD1B | 1.10E-02 | 1 |
| CHURC1 | 6.37E-03 | 1 | ADRA1D | 1.10E-02 | 1 |
| MCM9 | 6.48E-03 | 1 | CHGB | 1.13E-02 | 1 |
| STARD5 | 6.51E-03 | 1 | KLK6 | 1.15E-02 | 1 |
| FGA | 6.65E-03 | 1 | SLC22A8 | 1.16E-02 | 1 |
| EFNB2 | 6.77E-03 | 1 | PSIP1 | 1.18E-02 | 1 |
| PTRF | 6.86E-03 | 1 | KRTAP9-2 | 1.20E-02 | 1 |
| IL6ST | 7.21E-03 | 1 | ZMAT4 | 1.24E-02 | 1 |
| C4orf26 | 7.61E-03 | 1 | UCP1 | 1.29E-02 | 1 |
| BRD7 | 8.30E-03 | 1 | ARID1A | 1.30E-02 | 1 |
| TXN2 | 8.33E-03 | 1 | PRG3 | 1.32E-02 | 1 |
| AP2B1 | 8.41E-03 | 1 | PFKFB1 | 1.32E-02 | 1 |
| PHKG2 | 8.68E-03 | 1 | IL6ST | 1.38E-02 | 1 |
| BACE2 | 8.71E-03 | 1 | PRMT1 | 1.40E-02 | 1 |
| OCIAD2 | 8.74E-03 | 1 | RXRB | 1.43E-02 | 1 |
| ST8SIA4 | 8.79E-03 | 1 | TMEM215 | 1.43E-02 | 1 |
| ZNF716 | 8.91E-03 | 1 | OR8K5 | 1.43E-02 | 1 |
| CALML6 | 8.94E-03 | 1 | SLC25A45 | 1.48E-02 | 1 |
| IFI30 | 9.43E-03 | 1 | TMEM176B | 1.48E-02 | 1 |
| PIK3R1 | 9.73E-03 | 1 | HIBCH | 1.49E-02 | 1 |
| CLEC1B | 9.82E-03 | 1 | C6orf15 | 1.53E-02 | 1 |
| FRA10AC | 1.00E-02 | 1 | PNPLA1 | 1.55E-02 | 1 |
| PRRX1 | 1.00E-02 | 1 | ADPRHL1 | 1.57E-02 | 1 |
| VPS4B | 1.01E-02 | 1 | TRPV2 | 1.59E-02 | 1 |
| CNPY2 | 1.01E-02 | 1 | TAF1D | 1.60E-02 | 1 |
| HSD11B1 | 1.01E-02 | 1 | HNF4A | 1.60E-02 | 1 |
| TTC19 | 1.01E-02 | 1 | SLC27A3 | 1.62E-02 | 1 |
| FTCD | 1.03E-02 | 1 | ARFGAP1 | 1.70E-02 | 1 |
| KRTAP10 | 1.04E-02 | 1 | LACTB | 1.73E-02 | 1 |
| CDCP1 | 1.07E-02 | 1 | EIF5A | 1.74E-02 | 1 |
| WFDC5 | 1.07E-02 | 1 | C11orf57 | 1.76E-02 | 1 |
| GRIA1 | 1.09E-02 | 1 | TSTA3 | 1.77E-02 | 1 |
| MRPL22 | 1.09E-02 | 1 | CHST13 | 1.78E-02 | 1 |
| HSPA1L | 1.10E-02 | 1 | TAPBPL | 1.78E-02 | 1 |
| CREB3L3 | 1.12E-02 | 1 | IGDCC3 | 1.79E-02 | 1 |
| PRR23A | 1.12E-02 | 1 | RASSF10 | 1.79E-02 | 1 |
| OR6P1 | 1.13E-02 | 1 | CEACAM4 | 1.91E-02 | 1 |
| DEFB113 | 1.14E-02 | 1 | PPP2R4 | 1.95E-02 | 1 |
| SLC10A1 | 1.15E-02 | 1 | SLC25A12 | 2.01E-02 | 1 |
| COL6A6 | 1.16E-02 | 1 | GAL3ST3 | 2.03E-02 | 1 |
| GPSM3 | 1.19E-02 | 1 | DAZAP1 | 2.06E-02 | 1 |
| HMGB1 | 1.21E-02 | 1 | CACNA1E | 2.07E-02 | 1 |

| FZR1 | 1.24E-02 | 1 | CALB1 | 2.07E-02 | 1 |
| --- | --- | --- | --- | --- | --- |
| TF | 1.28E-02 | 1 | SPRR2G | 2.07E-02 | 1 |
| TMEM8B | 1.29E-02 | 1 | C1orf94 | 2.08E-02 | 1 |
| SFT2D2 | 1.31E-02 | 1 | MMACHC | 2.10E-02 | 1 |
| PODXL | 1.31E-02 | 1 | PLEKHG7 | 2.11E-02 | 1 |
| DYRK1A | 1.31E-02 | 1 | GJA8 | 2.13E-02 | 1 |
| ELF3 | 1.31E-02 | 1 | SPAG6 | 2.13E-02 | 1 |
| PLB1 | 1.35E-02 | 1 | BAG5 | 2.17E-02 | 1 |
| GUCY1A3 | 1.37E-02 | 1 | E2F6 | 2.17E-02 | 1 |
| OR2J3 | 1.38E-02 | 1 | EEF1G | 2.19E-02 | 1 |
| C9orf135 | 1.43E-02 | 1 | SLC33A1 | 2.20E-02 | 1 |
| APOM | 1.45E-02 | 1 | KRTAP22-1 | 2.21E-02 | 1 |
| IFIT3 | 1.47E-02 | 1 | LRRC69 | 2.22E-02 | 1 |
| PODNL1 | 1.47E-02 | 1 | ELL2 | 2.25E-02 | 1 |
| MED28 | 1.48E-02 | 1 | MAP2K2 | 2.27E-02 | 1 |
| CALR | 1.49E-02 | 1 | DEPDC1 | 2.33E-02 | 1 |
| CLEC3B | 1.49E-02 | 1 | KCNJ3 | 2.35E-02 | 1 |
| TNNI2 | 1.52E-02 | 1 | NR4A2 | 2.35E-02 | 1 |
| KIAA0513 | 1.53E-02 | 1 | UQCRH | 2.36E-02 | 1 |
| RAB18 | 1.55E-02 | 1 | CRYBA4 | 2.37E-02 | 1 |
| PTEN | 1.55E-02 | 1 | SEMA3E | 2.38E-02 | 1 |
| FLT3LG | 1.57E-02 | 1 | NUMB | 2.38E-02 | 1 |
| ADAM12 | 1.58E-02 | 1 | PAGE2 | 2.39E-02 | 1 |
| PDRG1 | 1.60E-02 | 1 | HS2ST1 | 2.42E-02 | 1 |
| RPS6KA3 | 1.62E-02 | 1 | ZNF880 | 2.43E-02 | 1 |
| CDH1 | 1.62E-02 | 1 | KRAS | 2.44E-02 | 1 |
| HMBS | 1.62E-02 | 1 | RASA1 | 2.46E-02 | 1 |
| OGN | 1.66E-02 | 1 | CHEK1 | 2.46E-02 | 1 |
| PGBD5 | 1.71E-02 | 1 | KRTAP1-3 | 2.47E-02 | 1 |
| TDG | 1.72E-02 | 1 | MAP4K5 | 2.47E-02 | 1 |
| EIF3J | 1.73E-02 | 1 | EMP1 | 2.47E-02 | 1 |
| SLC2A2 | 1.74E-02 | 1 | ACOX2 | 2.55E-02 | 1 |
| CLK2 | 1.75E-02 | 1 | SLC46A1 | 2.56E-02 | 1 |
| DHX57 | 1.78E-02 | 1 | TMEM218 | 2.56E-02 | 1 |
| SLC30A8 | 1.80E-02 | 1 | CEP120 | 2.58E-02 | 1 |
| COL11A2 | 1.81E-02 | 1 | FAM114A1 | 2.64E-02 | 1 |
| ARHGAP1 | 1.82E-02 | 1 | PTGR1 | 2.66E-02 | 1 |
| CHMP6 | 1.82E-02 | 1 | NLRP2 | 2.73E-02 | 1 |
| COQ6 | 1.85E-02 | 1 | HNRNPC | 2.76E-02 | 1 |
| CEACAM | 1.87E-02 | 1 | NOX3 | 2.77E-02 | 1 |
| BIRC7 | 1.87E-02 | 1 | LGALS9B | 2.78E-02 | 1 |
| CSNK1A1 | 1.88E-02 | 1 | ATP5L | 2.78E-02 | 1 |
| GLIS1 | 1.89E-02 | 1 | CYP4A22 | 2.79E-02 | 1 |
| REXO4 | 1.90E-02 | 1 | BBS7 | 2.81E-02 | 1 |
| PTTG2 | 1.90E-02 | 1 | UBE2D2 | 2.83E-02 | 1 |
| NOP10 | 1.91E-02 | 1 | ACAT2 | 2.83E-02 | 1 |
| ATP6V1D | 1.94E-02 | 1 | LMBR1 | 2.89E-02 | 1 |
| PHF10 | 1.99E-02 | 1 | NBEA | 2.89E-02 | 1 |
| CDH18 | 1.99E-02 | 1 | CNOT4 | 2.89E-02 | 1 |
| ST8SIA6 | 2.04E-02 | 1 | RUNX2 | 2.90E-02 | 1 |
| KRTAP10 | 2.05E-02 | 1 | ZBTB9 | 2.90E-02 | 1 |
| NLGN1 | 2.06E-02 | 1 | B2M | 2.91E-02 | 1 |
| ADH5 | 2.12E-02 | 1 | CHRNA9 | 2.94E-02 | 1 |

| GIMAP6 | 2.14E-02 | 1 | LGR6 | 2.94E-02 | 1 |
| --- | --- | --- | --- | --- | --- |
| C16orf86 | 2.15E-02 | 1 | TMPRSS3 | 2.96E-02 | 1 |
| MRPL35 | 2.17E-02 | 1 | ZNF141 | 2.97E-02 | 1 |
| CES2 | 2.18E-02 | 1 | ARID2 | 2.97E-02 | 1 |
| ZDHHC19 | 2.20E-02 | 1 | TSKU | 2.97E-02 | 1 |
| POC5 | 2.22E-02 | 1 | LRFN1 | 3.00E-02 | 1 |
| ALKBH4 | 2.23E-02 | 1 | KLHDC7B | 3.04E-02 | 1 |
| NDUFB6 | 2.25E-02 | 1 | NPVF | 3.05E-02 | 1 |
| C11orf1 | 2.25E-02 | 1 | THBS3 | 3.05E-02 | 1 |
| HAUS1 | 2.27E-02 | 1 | ARSD | 3.06E-02 | 1 |
| NMS | 2.28E-02 | 1 | TOMM22 | 3.06E-02 | 1 |
| BTRC | 2.30E-02 | 1 | RPS15 | 3.07E-02 | 1 |
| CAND1 | 2.32E-02 | 1 | FAM72A | 3.09E-02 | 1 |
| ANXA13 | 2.33E-02 | 1 | SULT2A1 | 3.13E-02 | 1 |
| WNT9B | 2.34E-02 | 1 | UBXN7 | 3.14E-02 | 1 |
| DOCK2 | 2.34E-02 | 1 | MASP2 | 3.15E-02 | 1 |
| PDZRN3 | 2.42E-02 | 1 | PPM1H | 3.17E-02 | 1 |
| PCDH11Y | 2.42E-02 | 1 | CHST3 | 3.19E-02 | 1 |
| ZBTB2 | 2.44E-02 | 1 | IMPA1 | 3.20E-02 | 1 |
| CTCF | 2.45E-02 | 1 | BRSK1 | 3.21E-02 | 1 |
| AFF4 | 2.45E-02 | 1 | FXYD6 | 3.21E-02 | 1 |
| MKNK2 | 2.47E-02 | 1 | DIXDC1 | 3.23E-02 | 1 |
| TCEA1 | 2.49E-02 | 1 | ABHD10 | 3.23E-02 | 1 |
| OR4M1 | 2.49E-02 | 1 | ZNF488 | 3.25E-02 | 1 |
| HLA-B | 2.50E-02 | 1 | PZP | 3.25E-02 | 1 |
| IRF2 | 2.52E-02 | 1 | ZNHIT6 | 3.25E-02 | 1 |
| VGLL1 | 2.53E-02 | 1 | RAB33A | 3.25E-02 | 1 |
| LCP2 | 2.55E-02 | 1 | COMMD6 | 3.26E-02 | 1 |
| RBM10 | 2.58E-02 | 1 | SNRPF | 3.29E-02 | 1 |
| OR5L1 | 2.59E-02 | 1 | NUP133 | 3.30E-02 | 1 |
| GIN1 | 2.60E-02 | 1 | LEAP2 | 3.30E-02 | 1 |
| CCDC23 | 2.60E-02 | 1 | MFGE8 | 3.30E-02 | 1 |
| NSUN4 | 2.60E-02 | 1 | SRRM3 | 3.32E-02 | 1 |
| OR2L8 | 2.61E-02 | 1 | OCLN | 3.33E-02 | 1 |
| DDRGK1 | 2.61E-02 | 1 | ID3 | 3.34E-02 | 1 |
| CR2 | 2.63E-02 | 1 | BEX4 | 3.36E-02 | 1 |
| CDH7 | 2.64E-02 | 1 | TRAPPC6B | 3.37E-02 | 1 |
| PDCL2 | 2.64E-02 | 1 | SLC12A5 | 3.39E-02 | 1 |
| RBBP4 | 2.64E-02 | 1 | CISD1 | 3.42E-02 | 1 |
| C10orf76 | 2.67E-02 | 1 | KCNJ4 | 3.44E-02 | 1 |
| JAM2 | 2.68E-02 | 1 | SPINT3 | 3.46E-02 | 1 |
| COL11A1 | 2.70E-02 | 1 | NXF3 | 3.48E-02 | 1 |
| CLDN5 | 2.70E-02 | 1 | PCK1 | 3.49E-02 | 1 |
| TLK2 | 2.73E-02 | 1 | CDKN2A | 3.50E-02 | 1 |
| LHFP | 2.75E-02 | 1 | FAM65B | 3.50E-02 | 1 |
| UGT2B7 | 2.76E-02 | 1 | IL17RD | 3.51E-02 | 1 |
| MCM3 | 2.77E-02 | 1 | RAD23B | 3.51E-02 | 1 |
| FAU | 2.79E-02 | 1 | EI24 | 3.53E-02 | 1 |
| APOB | 2.80E-02 | 1 | RXRA | 3.54E-02 | 1 |
| RPL22 | 2.83E-02 | 1 | PDZK1IP1 | 3.54E-02 | 1 |
| RRP1B | 2.83E-02 | 1 | CR1L | 3.55E-02 | 1 |
| NFKBIA | 2.83E-02 | 1 | RPL22 | 3.56E-02 | 1 |
| ZNF503 | 2.84E-02 | 1 | TLX3 | 3.60E-02 | 1 |

| TIMP4 | 2.84E-02 | 1 | CDA | 3.61E-02 | 1 |
| --- | --- | --- | --- | --- | --- |
| FGFBP1 | 2.85E-02 | 1 | HIVEP1 | 3.63E-02 | 1 |
| CNNM1 | 2.86E-02 | 1 | PDE6D | 3.66E-02 | 1 |
| SDS | 2.87E-02 | 1 | DEFB113 | 3.71E-02 | 1 |
| DCAF6 | 2.89E-02 | 1 | COL12A1 | 3.72E-02 | 1 |
| TOMM6 | 2.89E-02 | 1 | FAM81B | 3.73E-02 | 1 |
| C5orf38 | 2.94E-02 | 1 | NKAP | 3.78E-02 | 1 |
| CCS | 2.94E-02 | 1 | OR51V1 | 3.78E-02 | 1 |
| SLC34A1 | 2.95E-02 | 1 | SLC36A3 | 3.88E-02 | 1 |
| MBP | 2.96E-02 | 1 | EGFLAM | 3.88E-02 | 1 |
| SPCS2 | 2.96E-02 | 1 | KLHDC4 | 3.90E-02 | 1 |
| IFIT5 | 2.98E-02 | 1 | PDE4D | 3.90E-02 | 1 |
| CNGA3 | 3.00E-02 | 1 | SUV39H2 | 3.90E-02 | 1 |
| C3 | 3.02E-02 | 1 | ADCY5 | 3.91E-02 | 1 |
| SLC23A2 | 3.02E-02 | 1 | FAM98A | 3.91E-02 | 1 |
| PIK3C2B | 3.02E-02 | 1 | HSP90AA1 | 3.93E-02 | 1 |
| SLC17A3 | 3.03E-02 | 1 | NFASC | 3.94E-02 | 1 |
| ENAH | 3.04E-02 | 1 | CPA6 | 3.98E-02 | 1 |
| ADIPOQ | 3.06E-02 | 1 | SENP2 | 3.98E-02 | 1 |
| FURIN | 3.07E-02 | 1 | 2-Sep | 3.98E-02 | 1 |
| NIPA2 | 3.07E-02 | 1 | LRRK2 | 4.00E-02 | 1 |
| GPR4 | 3.10E-02 | 1 | OR2T27 | 4.01E-02 | 1 |
| NKX2-2 | 3.11E-02 | 1 | KRTAP25-1 | 4.04E-02 | 1 |
| SUGT1 | 3.12E-02 | 1 | C20orf24 | 4.05E-02 | 1 |
| FTMT | 3.13E-02 | 1 | C20orf27 | 4.07E-02 | 1 |
| CLGN | 3.14E-02 | 1 | FAM69B | 4.14E-02 | 1 |
| CAPZA1 | 3.15E-02 | 1 | SPACA4 | 4.15E-02 | 1 |
| HORMAD | 3.15E-02 | 1 | TRAF7 | 4.16E-02 | 1 |
| FAM71B | 3.16E-02 | 1 | RNF5 | 4.17E-02 | 1 |
| RNF145 | 3.16E-02 | 1 | MCCC1 | 4.19E-02 | 1 |
| PCK1 | 3.16E-02 | 1 | LGI3 | 4.20E-02 | 1 |
| VDAC1 | 3.17E-02 | 1 | CFP | 4.24E-02 | 1 |
| KY | 3.23E-02 | 1 | ITIH5 | 4.24E-02 | 1 |
| ALPP | 3.24E-02 | 1 | TERF1 | 4.24E-02 | 1 |
| MLF2 | 3.24E-02 | 1 | ZBTB48 | 4.25E-02 | 1 |
| HMGCR | 3.24E-02 | 1 | ADAD2 | 4.30E-02 | 1 |
| CYP2B6 | 3.26E-02 | 1 | WDR45 | 4.32E-02 | 1 |
| KRTAP5-6 | 3.28E-02 | 1 | RNASE4 | 4.33E-02 | 1 |
| MT1M | 3.28E-02 | 1 | GABARAP | 4.34E-02 | 1 |
| IL1R1 | 3.28E-02 | 1 | PIGQ | 4.35E-02 | 1 |
| OR5K1 | 3.31E-02 | 1 | SPEG | 4.40E-02 | 1 |
| CSAG1 | 3.31E-02 | 1 | OR5M10 | 4.45E-02 | 1 |
| LYZL6 | 3.32E-02 | 1 | ZFPL1 | 4.45E-02 | 1 |
| ZNF227 | 3.32E-02 | 1 | CTNNBL1 | 4.48E-02 | 1 |
| MBOAT1 | 3.33E-02 | 1 | ANKRD7 | 4.49E-02 | 1 |
| TMEM51 | 3.33E-02 | 1 | CYB5A | 4.50E-02 | 1 |
| C1R | 3.34E-02 | 1 | ACCS | 4.52E-02 | 1 |
| NCOA3 | 3.35E-02 | 1 | THADA | 4.53E-02 | 1 |
| ANKRD13 | 3.36E-02 | 1 | IRF2 | 4.54E-02 | 1 |
| LGALS12 | 3.38E-02 | 1 | OR10H1 | 4.55E-02 | 1 |
| PLA2G4A | 3.40E-02 | 1 | UBC | 4.61E-02 | 1 |
| SREBF1 | 3.42E-02 | 1 | NBPF3 | 4.61E-02 | 1 |
| ZNF800 | 3.43E-02 | 1 | MAP3K3 | 4.61E-02 | 1 |

| CYSLTR1 | 3.45E-02 | 1 | SIRPD | 4.62E-02 | 1 |
| --- | --- | --- | --- | --- | --- |
| SCGB2A2 | 3.46E-02 | 1 | TRIM49 | 4.64E-02 | 1 |
| GIPC1 | 3.46E-02 | 1 | FBXL7 | 4.67E-02 | 1 |
| CASP3 | 3.46E-02 | 1 | SLC6A5 | 4.67E-02 | 1 |
| OR2F2 | 3.48E-02 | 1 | BLID | 4.70E-02 | 1 |
| OTUB1 | 3.57E-02 | 1 | GNB5 | 4.73E-02 | 1 |
| GPR101 | 3.58E-02 | 1 | IGSF3 | 4.75E-02 | 1 |
| RCAN3 | 3.58E-02 | 1 | LCE1B | 4.80E-02 | 1 |
| TCTA | 3.62E-02 | 1 | IFNW1 | 4.81E-02 | 1 |
| FGF12 | 3.64E-02 | 1 | PCBD2 | 4.85E-02 | 1 |
| TMEM206 | 3.67E-02 | 1 | C9orf129 | 4.88E-02 | 1 |
| TMEM203 | 3.68E-02 | 1 | FAM150A | 4.88E-02 | 1 |
| AMZ2 | 3.68E-02 | 1 | COQ10B | 4.89E-02 | 1 |
| CHD5 | 3.69E-02 | 1 | ERRFI1 | 4.91E-02 | 1 |
| CHRM1 | 3.69E-02 | 1 | PRMT8 | 4.91E-02 | 1 |
| IGJ | 3.70E-02 | 1 | ST8SIA5 | 4.93E-02 | 1 |
| DAK | 3.75E-02 | 1 | SPEF1 | 4.95E-02 | 1 |
| HIST2H2A | 3.75E-02 | 1 | TMEM185A | 4.96E-02 | 1 |
| GALNT12 | 3.76E-02 | 1 | TMEM128 | 4.96E-02 | 1 |
| TEP1 | 3.79E-02 | 1 | NOL12 | 4.96E-02 | 1 |
| VWA5B2 | 3.80E-02 | 1 | MYOG | 4.97E-02 | 1 |
| PROM1 | 3.81E-02 | 1 | IGFL1 | 4.98E-02 | 1 |
| ERCC4 | 3.90E-02 | 1 | MPV17L | 4.99E-02 | 1 |
| KIF6 | 3.91E-02 | 1 | FAM73A | 5.02E-02 | 1 |
| PTPN3 | 3.92E-02 | 1 | ARHGAP26 | 5.02E-02 | 1 |
| SLAMF1 | 3.92E-02 | 1 | UBA52 | 5.04E-02 | 1 |
| C5orf60 | 3.93E-02 | 1 | PLD4 | 5.05E-02 | 1 |
| SIRPD | 4.00E-02 | 1 | MMP7 | 5.11E-02 | 1 |
| AKR1E2 | 4.01E-02 | 1 | VPS39 | 5.15E-02 | 1 |
| MORC3 | 4.02E-02 | 1 | SFXN4 | 5.19E-02 | 1 |
| DNAJC27 | 4.03E-02 | 1 | HGS | 5.26E-02 | 1 |
| MFSD3 | 4.04E-02 | 1 | KRTAP4-2 | 5.27E-02 | 1 |
| KCNIP1 | 4.04E-02 | 1 | USP37 | 5.28E-02 | 1 |
| KIF25 | 4.05E-02 | 1 | TNKS1BP1 | 5.28E-02 | 1 |
| HOXB4 | 4.05E-02 | 1 | MAPK9 | 5.29E-02 | 1 |
| LRRIQ1 | 4.09E-02 | 1 | ZNF226 | 5.33E-02 | 1 |
| KRT15 | 4.12E-02 | 1 | KRTAP3-3 | 5.34E-02 | 1 |
| RNF148 | 4.13E-02 | 1 | SPINK4 | 5.37E-02 | 1 |
| OR2B11 | 4.17E-02 | 1 | KRT10 | 5.37E-02 | 1 |
| KRT75 | 4.17E-02 | 1 | THBS2 | 5.37E-02 | 1 |
| KRT36 | 4.19E-02 | 1 | HIST1H3C | 5.38E-02 | 1 |
| TRIML1 | 4.19E-02 | 1 | LHB | 5.38E-02 | 1 |
| GABRA4 | 4.20E-02 | 1 | PDRG1 | 5.39E-02 | 1 |
| NPB | 4.20E-02 | 1 | CHD4 | 5.39E-02 | 1 |
| TFDP2 | 4.22E-02 | 1 | ARHGEF10 | 5.40E-02 | 1 |
| OR1S1 | 4.23E-02 | 1 | MFSD4 | 5.44E-02 | 1 |
| GZMA | 4.23E-02 | 1 | IGF2BP3 | 5.45E-02 | 1 |
| RPS6KL1 | 4.24E-02 | 1 | ASB10 | 5.50E-02 | 1 |
| KRTAP25 | 4.24E-02 | 1 | C6orf226 | 5.53E-02 | 1 |
| CYP2C8 | 4.26E-02 | 1 | ENKUR | 5.55E-02 | 1 |
| CHST10 | 4.27E-02 | 1 | RPL9 | 5.56E-02 | 1 |
| STK32B | 4.28E-02 | 1 | ADNP | 5.56E-02 | 1 |
| KIAA1324 | 4.30E-02 | 1 | 7-Mar | 5.60E-02 | 1 |

| SLC1A7 | 4.30E-02 | 1 | SNX10 | 5.64E-02 | 1 |
| --- | --- | --- | --- | --- | --- |
| ZNF365 | 4.33E-02 | 1 | CDH15 | 5.65E-02 | 1 |
| FAM153C | 4.35E-02 | 1 | KIT | 5.67E-02 | 1 |
| GYPE | 4.36E-02 | 1 | NGRN | 5.68E-02 | 1 |
| TM9SF2 | 4.36E-02 | 1 | TWSG1 | 5.70E-02 | 1 |
| HAMP | 4.36E-02 | 1 | SUPT16H | 5.70E-02 | 1 |
| HIST1H1C | 4.38E-02 | 1 | BHLHE40 | 5.71E-02 | 1 |
| PCDHB16 | 4.38E-02 | 1 | AGRP | 5.75E-02 | 1 |
| ITM2A | 4.38E-02 | 1 | TNN | 5.78E-02 | 1 |
| LILRA2 | 4.41E-02 | 1 | RPL36AL | 5.79E-02 | 1 |
| COMMD5 | 4.41E-02 | 1 | LARP6 | 5.80E-02 | 1 |
| ORMDL2 | 4.41E-02 | 1 | ENPP6 | 5.83E-02 | 1 |
| ALOX12B | 4.43E-02 | 1 | THEMIS | 5.84E-02 | 1 |
| POU3F2 | 4.48E-02 | 1 | NGFR | 5.84E-02 | 1 |
| NIPAL2 | 4.49E-02 | 1 | SLC24A4 | 5.85E-02 | 1 |
| PIGV | 4.50E-02 | 1 | HEATR3 | 5.86E-02 | 1 |
| MAP1S | 4.50E-02 | 1 | TRIM42 | 5.88E-02 | 1 |
| ZBTB8OS | 4.52E-02 | 1 | PRIMA1 | 5.90E-02 | 1 |
| NF2 | 4.57E-02 | 1 | SNTG2 | 5.92E-02 | 1 |
| ATF4 | 4.57E-02 | 1 | NAP1L5 | 5.94E-02 | 1 |
| HNRNPA1 | 4.58E-02 | 1 | BMPR2 | 5.95E-02 | 1 |
| PPDPF | 4.60E-02 | 1 | SLA2 | 5.97E-02 | 1 |
| KRTDAP | 4.61E-02 | 1 | TCF15 | 5.97E-02 | 1 |
| ARMCX3 | 4.61E-02 | 1 | CCDC65 | 5.98E-02 | 1 |
| RHEB | 4.63E-02 | 1 | EDN1 | 5.99E-02 | 1 |
| STEAP3 | 4.63E-02 | 1 | CRHR1 | 5.99E-02 | 1 |
| HHATL | 4.63E-02 | 1 | HNRNPD | 6.00E-02 | 1 |
| HIST2H2A | 4.63E-02 | 1 | NPEPL1 | 6.02E-02 | 1 |
| MAB21L1 | 4.64E-02 | 1 | DNAI2 | 6.02E-02 | 1 |
| ATP2A2 | 4.66E-02 | 1 | KYNU | 6.03E-02 | 1 |
| DYX1C1 | 4.66E-02 | 1 | WDR4 | 6.05E-02 | 1 |
| USP48 | 4.69E-02 | 1 | KDM3A | 6.06E-02 | 1 |
| PPARA | 4.69E-02 | 1 | PTK2B | 6.12E-02 | 1 |
| WDR38 | 4.69E-02 | 1 | B4GALNT3 | 6.13E-02 | 1 |
| SMG7 | 4.69E-02 | 1 | TAS1R1 | 6.14E-02 | 1 |
| RTKN2 | 4.70E-02 | 1 | ITGB3BP | 6.14E-02 | 1 |
| PABPC4L | 4.70E-02 | 1 | BMP15 | 6.18E-02 | 1 |
| HMGCS1 | 4.71E-02 | 1 | CD96 | 6.18E-02 | 1 |
| HIST1H4J | 4.73E-02 | 1 | RAB3D | 6.19E-02 | 1 |
| CXorf36 | 4.74E-02 | 1 | LMAN1L | 6.19E-02 | 1 |
| ILKAP | 4.74E-02 | 1 | RPS23 | 6.20E-02 | 1 |
| GPHN | 4.75E-02 | 1 | MAOB | 6.23E-02 | 1 |
| ITIH4 | 4.76E-02 | 1 | KRTAP10-4 | 6.24E-02 | 1 |
| CYP4B1 | 4.77E-02 | 1 | ITGAD | 6.28E-02 | 1 |
| GNG12 | 4.79E-02 | 1 | PDGFB | 6.28E-02 | 1 |
| HIST1H2A | 4.80E-02 | 1 | MAK | 6.28E-02 | 1 |
| EGFR | 4.81E-02 | 1 | C11orf70 | 6.29E-02 | 1 |
| SPRR4 | 4.82E-02 | 1 | CD44 | 6.30E-02 | 1 |
| GABRQ | 4.83E-02 | 1 | TRPT1 | 6.31E-02 | 1 |
| PCBP1 | 4.84E-02 | 1 | TMEM81 | 6.35E-02 | 1 |
| B4GALNT | 4.90E-02 | 1 | PLA2G2F | 6.35E-02 | 1 |
| ERCC2 | 4.91E-02 | 1 | PGLS | 6.38E-02 | 1 |
| MGLL | 4.93E-02 | 1 | ABCB5 | 6.39E-02 | 1 |

| HIST1H2B | 4.94E-02 | 1 | FATE1 | 6.39E-02 | 1 |
| --- | --- | --- | --- | --- | --- |
| CHST11 | 4.94E-02 | 1 | FOLR3 | 6.42E-02 | 1 |
| EPM2A | 4.96E-02 | 1 | C7orf61 | 6.42E-02 | 1 |
| B3GALNT | 4.96E-02 | 1 | SPATA16 | 6.42E-02 | 1 |
| FASN | 4.97E-02 | 1 | KIAA1033 | 6.43E-02 | 1 |
| TAB1 | 4.98E-02 | 1 | LCAT | 6.43E-02 | 1 |
| WNT9A | 5.00E-02 | 1 | TADA2B | 6.44E-02 | 1 |
| SLC1A6 | 5.01E-02 | 1 | R3HDML | 6.45E-02 | 1 |
| GPR78 | 5.02E-02 | 1 | NF2 | 6.47E-02 | 1 |
| KIT | 5.02E-02 | 1 | OLFM3 | 6.48E-02 | 1 |
| PALMD | 5.02E-02 | 1 | PLEKHG3 | 6.48E-02 | 1 |
| PTGR1 | 5.03E-02 | 1 | SELO | 6.50E-02 | 1 |
| HGFAC | 5.06E-02 | 1 | PMP22 | 6.50E-02 | 1 |
| CDKN1C | 5.06E-02 | 1 | SIK2 | 6.53E-02 | 1 |
| SUSD2 | 5.11E-02 | 1 | SWAP70 | 6.54E-02 | 1 |
| EPOR | 5.12E-02 | 1 | COL7A1 | 6.54E-02 | 1 |
| TACR3 | 5.13E-02 | 1 | CLCF1 | 6.56E-02 | 1 |
| VSIG4 | 5.13E-02 | 1 | MME | 6.56E-02 | 1 |
| SAMHD1 | 5.14E-02 | 1 | KIAA0586 | 6.57E-02 | 1 |
| GHDC | 5.15E-02 | 1 | SGOL2 | 6.58E-02 | 1 |
| TTR | 5.17E-02 | 1 | ULK1 | 6.58E-02 | 1 |
| LEO1 | 5.19E-02 | 1 | SAMD12 | 6.60E-02 | 1 |
| SEC63 | 5.20E-02 | 1 | NR3C2 | 6.61E-02 | 1 |
| TMEM56 | 5.20E-02 | 1 | SHFM1 | 6.61E-02 | 1 |
| RDH16 | 5.21E-02 | 1 | RPL13 | 6.62E-02 | 1 |
| PTPRQ | 5.21E-02 | 1 | SPRR2E | 6.62E-02 | 1 |
| CDA | 5.22E-02 | 1 | HTR3C | 6.62E-02 | 1 |
| BBOX1 | 5.23E-02 | 1 | SSU72 | 6.63E-02 | 1 |
| MEIS3 | 5.25E-02 | 1 | PSMB3 | 6.64E-02 | 1 |
| CSPG5 | 5.26E-02 | 1 | FASLG | 6.65E-02 | 1 |
| CWC27 | 5.27E-02 | 1 | ITGB2 | 6.65E-02 | 1 |
| STC2 | 5.27E-02 | 1 | KCNA5 | 6.67E-02 | 1 |
| RLN3 | 5.28E-02 | 1 | MSN | 6.69E-02 | 1 |
| HIST1H2A | 5.28E-02 | 1 | STX10 | 6.76E-02 | 1 |
| ABCG2 | 5.28E-02 | 1 | DTX3 | 6.77E-02 | 1 |
| CISD3 | 5.28E-02 | 1 | CCDC138 | 6.79E-02 | 1 |
| DKK2 | 5.28E-02 | 1 | NME6 | 6.79E-02 | 1 |
| IL17C | 5.29E-02 | 1 | TCF7L1 | 6.80E-02 | 1 |
| DGAT1 | 5.29E-02 | 1 | STMN1 | 6.80E-02 | 1 |
| PNMAL1 | 5.31E-02 | 1 | DMKN | 6.81E-02 | 1 |
| C19orf24 | 5.33E-02 | 1 | TRIM13 | 6.82E-02 | 1 |
| FCRLB | 5.35E-02 | 1 | MDFI | 6.85E-02 | 1 |
| RAB4A | 5.38E-02 | 1 | TSPAN16 | 6.87E-02 | 1 |
| RCOR3 | 5.39E-02 | 1 | SPRR2B | 6.87E-02 | 1 |
| ACPP | 5.39E-02 | 1 | C8orf22 | 6.88E-02 | 1 |
| CADM2 | 5.40E-02 | 1 | ATG16L1 | 6.93E-02 | 1 |
| AFMID | 5.41E-02 | 1 | ANGPTL4 | 6.97E-02 | 1 |
| FOXD4L5 | 5.43E-02 | 1 | SDF4 | 6.98E-02 | 1 |
| GPR146 | 5.44E-02 | 1 | HPR | 7.01E-02 | 1 |
| KIFC2 | 5.46E-02 | 1 | GPR63 | 7.02E-02 | 1 |
| NLRP12 | 5.46E-02 | 1 | ZCCHC2 | 7.02E-02 | 1 |
| WAPAL | 5.47E-02 | 1 | EFEMP1 | 7.04E-02 | 1 |
| FAAH | 5.49E-02 | 1 | LAMB3 | 7.07E-02 | 1 |

| TMEM128 | 5.50E-02 | 1 | KLF6 | 7.07E-02 | 1 |
| --- | --- | --- | --- | --- | --- |
| PIGS | 5.50E-02 | 1 | LRRC3 | 7.13E-02 | 1 |
| IRX4 | 5.50E-02 | 1 | GPR156 | 7.14E-02 | 1 |
| ADCY2 | 5.51E-02 | 1 | DUS4L | 7.17E-02 | 1 |
| MARCKS | 5.51E-02 | 1 | GP1BB | 7.22E-02 | 1 |
| SPRY2 | 5.52E-02 | 1 | RCOR3 | 7.28E-02 | 1 |
| DIRAS1 | 5.52E-02 | 1 | PSG7 | 7.29E-02 | 1 |
| CHRNA7 | 5.54E-02 | 1 | SEPP1 | 7.30E-02 | 1 |
| EPHA4 | 5.54E-02 | 1 | PDX1 | 7.30E-02 | 1 |
| UMODL1 | 5.56E-02 | 1 | NHLRC2 | 7.31E-02 | 1 |
| PCLO | 5.56E-02 | 1 | CLK1 | 7.33E-02 | 1 |
| IL12RB1 | 5.59E-02 | 1 | FHL5 | 7.38E-02 | 1 |
| DAZAP1 | 5.61E-02 | 1 | ETHE1 | 7.44E-02 | 1 |
| KCNN4 | 5.63E-02 | 1 | RASL11A | 7.45E-02 | 1 |
| SULT4A1 | 5.63E-02 | 1 | PLD5 | 7.45E-02 | 1 |
| GPX5 | 5.64E-02 | 1 | IMMP1L | 7.46E-02 | 1 |
| ELAC1 | 5.65E-02 | 1 | APOC1 | 7.48E-02 | 1 |
| MMP19 | 5.66E-02 | 1 | RHOXF1 | 7.49E-02 | 1 |
| CCL18 | 5.67E-02 | 1 | PLCB1 | 7.49E-02 | 1 |
| EIF5A | 5.69E-02 | 1 | ADAM12 | 7.50E-02 | 1 |
| NHLH2 | 5.70E-02 | 1 | GLE1 | 7.53E-02 | 1 |
| NPY2R | 5.72E-02 | 1 | FAM71C | 7.55E-02 | 1 |
| NUP133 | 5.74E-02 | 1 | SFTA2 | 7.55E-02 | 1 |
| UBL4B | 5.74E-02 | 1 | ZNF578 | 7.56E-02 | 1 |
| TMED1 | 5.76E-02 | 1 | KIAA1841 | 7.57E-02 | 1 |
| GOLGA6L | 5.76E-02 | 1 | C2orf80 | 7.58E-02 | 1 |
| NXF3 | 5.78E-02 | 1 | GNPNAT1 | 7.59E-02 | 1 |
| CCRN4L | 5.78E-02 | 1 | CCNJL | 7.66E-02 | 1 |
| NFYC | 5.80E-02 | 1 | GYPA | 7.66E-02 | 1 |
| MFSD6 | 5.81E-02 | 1 | TTC9 | 7.67E-02 | 1 |
| GFRA2 | 5.81E-02 | 1 | RPAP2 | 7.73E-02 | 1 |
| MTMR14 | 5.82E-02 | 1 | MED29 | 7.73E-02 | 1 |
| SEMA3C | 5.85E-02 | 1 | NOL4 | 7.74E-02 | 1 |
| CMTM4 | 5.86E-02 | 1 | BSN | 7.75E-02 | 1 |
| ALAD | 5.87E-02 | 1 | MEOX1 | 7.78E-02 | 1 |
| CPA6 | 5.88E-02 | 1 | COL16A1 | 7.79E-02 | 1 |
| TMEM44 | 5.88E-02 | 1 | MRC2 | 7.79E-02 | 1 |
| PCBP3 | 5.88E-02 | 1 | KRTAP4-3 | 7.81E-02 | 1 |
| GATSL2 | 5.89E-02 | 1 | UNC45B | 7.83E-02 | 1 |
| BET1 | 5.93E-02 | 1 | MSL1 | 7.84E-02 | 1 |
| TOPORS | 5.93E-02 | 1 | SSNA1 | 7.84E-02 | 1 |
| IGFL3 | 5.93E-02 | 1 | CEACAM3 | 7.85E-02 | 1 |
| BBS5 | 5.95E-02 | 1 | REEP5 | 7.86E-02 | 1 |
| WNT1 | 5.96E-02 | 1 | ICMT | 7.86E-02 | 1 |
| TRAIP | 5.98E-02 | 1 | RTP1 | 7.87E-02 | 1 |
| MAB21L2 | 5.99E-02 | 1 | ERBB3 | 7.88E-02 | 1 |
| ZNF785 | 5.99E-02 | 1 | ANO9 | 7.88E-02 | 1 |
| FAM81B | 6.02E-02 | 1 | CD81 | 7.97E-02 | 1 |
| CORO7 | 6.02E-02 | 1 | SAV1 | 7.99E-02 | 1 |
| LCN8 | 6.02E-02 | 1 | GGCX | 7.99E-02 | 1 |
| RHOB | 6.03E-02 | 1 | PECR | 8.00E-02 | 1 |
| JAM3 | 6.03E-02 | 1 | DSC2 | 8.00E-02 | 1 |
| OR8H2 | 6.04E-02 | 1 | FBLIM1 | 8.01E-02 | 1 |

| C9orf131 | 6.07E-02 | 1 | EBF3 | 8.01E-02 | 1 |
| --- | --- | --- | --- | --- | --- |
| DNM2 | 6.10E-02 | 1 | CPN1 | 8.02E-02 | 1 |
| RAI14 | 6.11E-02 | 1 | ANGPT4 | 8.03E-02 | 1 |
| TPO | 6.12E-02 | 1 | VIM | 8.12E-02 | 1 |
| GABRA6 | 6.13E-02 | 1 | DLX5 | 8.16E-02 | 1 |
| DEFA3 | 6.14E-02 | 1 | RAVER2 | 8.17E-02 | 1 |
| IL23A | 6.15E-02 | 1 | DSE | 8.17E-02 | 1 |
| HNRNPA2 | 6.16E-02 | 1 | TUBB8 | 8.17E-02 | 1 |
| ADAMTS | 6.16E-02 | 1 | FBXO43 | 8.19E-02 | 1 |
| ZBTB9 | 6.18E-02 | 1 | CRP | 8.19E-02 | 1 |
| PRICKLE4 | 6.19E-02 | 1 | EMCN | 8.21E-02 | 1 |
| GPR132 | 6.19E-02 | 1 | LRPPRC | 8.29E-02 | 1 |
| POLR3H | 6.22E-02 | 1 | NFE2L2 | 8.29E-02 | 1 |
| SRXN1 | 6.23E-02 | 1 | FAM124B | 8.29E-02 | 1 |
| NCF4 | 6.23E-02 | 1 | RBL2 | 8.29E-02 | 1 |
| SERPINB1 | 6.23E-02 | 1 | ZC3H18 | 8.30E-02 | 1 |
| ACHE | 6.24E-02 | 1 | PSTPIP1 | 8.31E-02 | 1 |
| CALCOCO | 6.24E-02 | 1 | NRBP2 | 8.33E-02 | 1 |
| UBE2N | 6.25E-02 | 1 | CDKL4 | 8.37E-02 | 1 |
| EME1 | 6.30E-02 | 1 | VAMP1 | 8.37E-02 | 1 |
| FMO3 | 6.31E-02 | 1 | SCLY | 8.37E-02 | 1 |
| CENPH | 6.33E-02 | 1 | KRT33B | 8.38E-02 | 1 |
| AXIN2 | 6.33E-02 | 1 | IDI1 | 8.38E-02 | 1 |
| ENGASE | 6.36E-02 | 1 | FRG2C | 8.39E-02 | 1 |
| PTPN9 | 6.37E-02 | 1 | SPATA3 | 8.40E-02 | 1 |
| SERP1 | 6.37E-02 | 1 | MTSS1 | 8.47E-02 | 1 |
| MGAT2 | 6.38E-02 | 1 | C8orf33 | 8.48E-02 | 1 |
| TNNC2 | 6.38E-02 | 1 | DSG2 | 8.48E-02 | 1 |
| ULK2 | 6.38E-02 | 1 | SEC14L4 | 8.49E-02 | 1 |
| ILDR1 | 6.39E-02 | 1 | EEF1A1 | 8.50E-02 | 1 |
| FABP1 | 6.41E-02 | 1 | NUDT14 | 8.50E-02 | 1 |
| AKR7A2 | 6.41E-02 | 1 | PSMB5 | 8.53E-02 | 1 |
| ATAD2 | 6.42E-02 | 1 | TAL2 | 8.53E-02 | 1 |
| POMT2 | 6.42E-02 | 1 | LYZL1 | 8.55E-02 | 1 |
| SEMA3E | 6.43E-02 | 1 | ANXA9 | 8.56E-02 | 1 |
| RSAD1 | 6.43E-02 | 1 | GTF2B | 8.57E-02 | 1 |
| PRCP | 6.45E-02 | 1 | SNRPD3 | 8.57E-02 | 1 |
| GRM2 | 6.45E-02 | 1 | MAGEB18 | 8.58E-02 | 1 |
| ARL9 | 6.46E-02 | 1 | SLC30A3 | 8.59E-02 | 1 |
| LILRB5 | 6.47E-02 | 1 | CNGA4 | 8.59E-02 | 1 |
| COX5A | 6.50E-02 | 1 | GPN2 | 8.60E-02 | 1 |
| OR6C65 | 6.51E-02 | 1 | CAPN11 | 8.61E-02 | 1 |
| SLC2A5 | 6.55E-02 | 1 | 1-Dec | 8.62E-02 | 1 |
| TXNRD1 | 6.57E-02 | 1 | OR10A3 | 8.63E-02 | 1 |
| NUDT10 | 6.62E-02 | 1 | CACNA2D1 | 8.66E-02 | 1 |
| YEATS4 | 6.63E-02 | 1 | VPREB3 | 8.69E-02 | 1 |
| HSD3B7 | 6.64E-02 | 1 | HOXA4 | 8.71E-02 | 1 |
| IL21R | 6.67E-02 | 1 | RHOU | 8.72E-02 | 1 |
| KDELR3 | 6.68E-02 | 1 | HIST1H4D | 8.74E-02 | 1 |
| TUBB2A | 6.69E-02 | 1 | CTSG | 8.75E-02 | 1 |
| ARRB1 | 6.69E-02 | 1 | XPO6 | 8.76E-02 | 1 |
| UMPS | 6.70E-02 | 1 | C10orf90 | 8.77E-02 | 1 |
| OR11H6 | 6.71E-02 | 1 | RGS19 | 8.80E-02 | 1 |

| SOCS3 | 6.73E-02 | 1 | GBAS | 8.81E-02 | 1 |
| --- | --- | --- | --- | --- | --- |
| PDHA2 | 6.74E-02 | 1 | IWS1 | 8.82E-02 | 1 |
| HTR7 | 6.75E-02 | 1 | NEU2 | 8.83E-02 | 1 |
| CRISP2 | 6.77E-02 | 1 | NKAIN4 | 8.84E-02 | 1 |
| PLAC1 | 6.80E-02 | 1 | OR8H2 | 8.86E-02 | 1 |
| NOS3 | 6.80E-02 | 1 | IL4 | 8.87E-02 | 1 |
| RBL1 | 6.80E-02 | 1 | PDCD7 | 8.87E-02 | 1 |
| CYTH1 | 6.81E-02 | 1 | TMEM59L | 8.87E-02 | 1 |
| ZFPM2 | 6.81E-02 | 1 | CDCA5 | 8.88E-02 | 1 |
| RPP14 | 6.81E-02 | 1 | NECAP1 | 8.91E-02 | 1 |
| RMND1 | 6.82E-02 | 1 | SAMD5 | 8.91E-02 | 1 |
| FMO2 | 6.82E-02 | 1 | GIMAP1 | 8.93E-02 | 1 |
| UGT2B4 | 6.82E-02 | 1 | KCNS2 | 8.93E-02 | 1 |
| FNDC5 | 6.83E-02 | 1 | NOVA2 | 8.95E-02 | 1 |
| DAPL1 | 6.83E-02 | 1 | CLSTN2 | 8.96E-02 | 1 |
| HOOK2 | 6.83E-02 | 1 | CHID1 | 8.98E-02 | 1 |
| TMEM200 | 6.84E-02 | 1 | SNRNP27 | 8.99E-02 | 1 |
| DCDC2 | 6.85E-02 | 1 | ITIH2 | 9.01E-02 | 1 |
| BEND3 | 6.86E-02 | 1 | RAB8B | 9.03E-02 | 1 |
| FCAR | 6.86E-02 | 1 | MAP4K2 | 9.04E-02 | 1 |
| SRC | 6.87E-02 | 1 | HSPH1 | 9.06E-02 | 1 |
| CNOT6 | 6.87E-02 | 1 | DPYSL5 | 9.07E-02 | 1 |
| SHISA5 | 6.87E-02 | 1 | SULT6B1 | 9.07E-02 | 1 |
| RXFP3 | 6.89E-02 | 1 | DEF6 | 9.08E-02 | 1 |
| MAOB | 6.90E-02 | 1 | PVRL2 | 9.09E-02 | 1 |
| TMEM14C | 6.93E-02 | 1 | FAM171B | 9.09E-02 | 1 |
| POU2AF1 | 6.96E-02 | 1 | CIDEA | 9.09E-02 | 1 |
| NDUFS6 | 6.98E-02 | 1 | TLR8 | 9.11E-02 | 1 |
| PIH1D2 | 7.00E-02 | 1 | CCT3 | 9.13E-02 | 1 |
| RNF123 | 7.02E-02 | 1 | OMP | 9.13E-02 | 1 |
| CABP5 | 7.02E-02 | 1 | RBM39 | 9.15E-02 | 1 |
| FBXW5 | 7.02E-02 | 1 | PHF10 | 9.18E-02 | 1 |
| TIAL1 | 7.03E-02 | 1 | EVC2 | 9.18E-02 | 1 |
| IL1B | 7.03E-02 | 1 | MYD88 | 9.18E-02 | 1 |
| CLEC12A | 7.05E-02 | 1 | NLGN1 | 9.22E-02 | 1 |
| G6PC | 7.09E-02 | 1 | PROK2 | 9.22E-02 | 1 |
| KLHDC10 | 7.09E-02 | 1 | AKTIP | 9.22E-02 | 1 |
| HNRNPUL | 7.09E-02 | 1 | CDK9 | 9.24E-02 | 1 |
| PADI1 | 7.10E-02 | 1 | AIM2 | 9.25E-02 | 1 |
| PRMT6 | 7.10E-02 | 1 | LCE3D | 9.27E-02 | 1 |
| TARBP2 | 7.10E-02 | 1 | GAST | 9.28E-02 | 1 |
| MCM7 | 7.12E-02 | 1 | PDZD9 | 9.28E-02 | 1 |
| DNAJC19 | 7.17E-02 | 1 | FBXO28 | 9.29E-02 | 1 |
| WISP3 | 7.17E-02 | 1 | NUDT5 | 9.30E-02 | 1 |
| OTX1 | 7.19E-02 | 1 | PLG | 9.31E-02 | 1 |
| PRIM1 | 7.19E-02 | 1 | RAB4B | 9.35E-02 | 1 |
| PIGC | 7.20E-02 | 1 | C11orf87 | 9.35E-02 | 1 |
| HRG | 7.22E-02 | 1 | MUC1 | 9.38E-02 | 1 |
| C11orf71 | 7.23E-02 | 1 | SSR3 | 9.39E-02 | 1 |
| ATP2B4 | 7.23E-02 | 1 | CNRIP1 | 9.39E-02 | 1 |
| FGGY | 7.24E-02 | 1 | ZCCHC4 | 9.42E-02 | 1 |
| SCP2 | 7.25E-02 | 1 | TMCC1 | 9.44E-02 | 1 |
| FAM160A | 7.27E-02 | 1 | SIM2 | 9.45E-02 | 1 |

| HEBP1 | 7.29E-02 | 1 | GPAM | 9.45E-02 | 1 |
| --- | --- | --- | --- | --- | --- |
| RGS7 | 7.29E-02 | 1 | CTSS | 9.45E-02 | 1 |
| TRIM26 | 7.29E-02 | 1 | MFSD5 | 9.48E-02 | 1 |
| SMARCE1 | 7.30E-02 | 1 | DEFB126 | 9.50E-02 | 1 |
| TYMP | 7.31E-02 | 1 | C22orf39 | 9.51E-02 | 1 |
| ANKRD22 | 7.33E-02 | 1 | GALM | 9.51E-02 | 1 |
| GUSB | 7.33E-02 | 1 | ADRM1 | 9.51E-02 | 1 |
| KRT85 | 7.34E-02 | 1 | SYT12 | 9.53E-02 | 1 |
| OPALIN | 7.38E-02 | 1 | SIGLEC11 | 9.53E-02 | 1 |
| WDFY4 | 7.41E-02 | 1 | KCTD20 | 9.55E-02 | 1 |
| ESM1 | 7.41E-02 | 1 | GPR82 | 9.58E-02 | 1 |
| PLA2G3 | 7.42E-02 | 1 | IRAK1 | 9.58E-02 | 1 |
| MRPL14 | 7.43E-02 | 1 | HPGD | 9.59E-02 | 1 |
| ZNRF1 | 7.43E-02 | 1 | RGSL1 | 9.59E-02 | 1 |
| ELSPBP1 | 7.44E-02 | 1 | LCE3E | 9.61E-02 | 1 |
| LHCGR | 7.44E-02 | 1 | OR10K1 | 9.62E-02 | 1 |
| CYP3A5 | 7.45E-02 | 1 | GNG4 | 9.63E-02 | 1 |
| SAMD12 | 7.45E-02 | 1 | KRTAP10-7 | 9.63E-02 | 1 |
| TAS2R38 | 7.45E-02 | 1 | USP44 | 9.66E-02 | 1 |
| CD1E | 7.45E-02 | 1 | GAD1 | 9.70E-02 | 1 |
| ITGB8 | 7.46E-02 | 1 | UBE2K | 9.70E-02 | 1 |
| LDB3 | 7.46E-02 | 1 | ASTL | 9.71E-02 | 1 |
| IL31 | 7.47E-02 | 1 | STXBP1 | 9.72E-02 | 1 |
| TMTC2 | 7.48E-02 | 1 | WDR35 | 9.76E-02 | 1 |
| CDH19 | 7.48E-02 | 1 | MAN2A1 | 9.80E-02 | 1 |
| KRTAP6-1 | 7.49E-02 | 1 | FOXS1 | 9.81E-02 | 1 |
| CNTROB | 7.49E-02 | 1 | HHLA2 | 9.82E-02 | 1 |
| AMICA1 | 7.50E-02 | 1 | IGF1R | 9.82E-02 | 1 |
| ZBED2 | 7.53E-02 | 1 | CYP2E1 | 9.83E-02 | 1 |
| SENP6 | 7.54E-02 | 1 | TMEM72 | 9.84E-02 | 1 |
| FAP | 7.55E-02 | 1 | ST3GAL2 | 9.85E-02 | 1 |
| SPA17 | 7.58E-02 | 1 | KIR2DL3 | 9.87E-02 | 1 |
| MYLK4 | 7.62E-02 | 1 | FBLN2 | 9.87E-02 | 1 |
| MPP1 | 7.62E-02 | 1 | EPHA4 | 9.89E-02 | 1 |
| LUC7L3 | 7.64E-02 | 1 | SRM | 9.92E-02 | 1 |
| PDE3A | 7.64E-02 | 1 | ZNF716 | 9.93E-02 | 1 |
| GCG | 7.65E-02 | 1 | HERC4 | 9.93E-02 | 1 |
| PAIP2 | 7.67E-02 | 1 | NDUFA5 | 9.94E-02 | 1 |
| KRT86 | 7.68E-02 | 1 | CA6 | 9.95E-02 | 1 |
| REEP6 | 7.69E-02 | 1 | SGTB | 9.97E-02 | 1 |
| APOL6 | 7.69E-02 | 1 | FAM3C | 1.00E-01 | 1 |
| ZNF282 | 7.75E-02 | 1 | ATP1A4 | 1.00E-01 | 1 |
| LRRC24 | 7.75E-02 | 1 | PCDH7 | 1.00E-01 | 1 |
| SLAMF9 | 7.78E-02 | 1 | DEFB125 | 1.00E-01 | 1 |
| SPI1 | 7.79E-02 | 1 | REEP2 | 1.00E-01 | 1 |
| FZD2 | 7.79E-02 | 1 | ALG5 | 1.00E-01 | 1 |
| LGALS7B | 7.80E-02 | 1 | KCNA10 | 1.00E-01 | 1 |
| SORCS3 | 7.80E-02 | 1 | DNAJA2 | 1.01E-01 | 1 |
| LYNX1 | 7.81E-02 | 1 | FAM47A | 1.01E-01 | 1 |
| HHAT | 7.81E-02 | 1 | CBFA2T2 | 1.01E-01 | 1 |
| CDH11 | 7.84E-02 | 1 | UBXN1 | 1.01E-01 | 1 |
| INSL5 | 7.87E-02 | 1 | OR10J5 | 1.01E-01 | 1 |
| TGFBI | 7.88E-02 | 1 | RPGRIP1 | 1.01E-01 | 1 |

| WFDC3 | 7.89E-02 | 1 | CPPED1 | 1.01E-01 | 1 |
| --- | --- | --- | --- | --- | --- |
| MICAL1 | 7.89E-02 | 1 | ITGA4 | 1.01E-01 | 1 |
| CLDN9 | 7.89E-02 | 1 | PCID2 | 1.01E-01 | 1 |
| GNL3 | 7.92E-02 | 1 | REPIN1 | 1.01E-01 | 1 |
| PLTP | 7.92E-02 | 1 | HIST1H4E | 1.02E-01 | 1 |
| KRTAP10 | 7.93E-02 | 1 | CWC27 | 1.02E-01 | 1 |
| C5orf22 | 7.93E-02 | 1 | OR5T3 | 1.02E-01 | 1 |
| FAM13C | 7.93E-02 | 1 | CEACAM1 | 1.02E-01 | 1 |
| CTRC | 7.93E-02 | 1 | GMPR2 | 1.02E-01 | 1 |
| IRX1 | 7.95E-02 | 1 | PAG1 | 1.02E-01 | 1 |
| ARID3A | 7.95E-02 | 1 | ST3GAL3 | 1.02E-01 | 1 |
| CACNG2 | 7.95E-02 | 1 | ELF3 | 1.02E-01 | 1 |
| ZNF556 | 7.97E-02 | 1 | RBM8A | 1.02E-01 | 1 |
| ACCSL | 7.98E-02 | 1 | ZFP37 | 1.03E-01 | 1 |
| SLC22A20 | 7.98E-02 | 1 | TNFAIP6 | 1.03E-01 | 1 |
| VPS25 | 8.01E-02 | 1 | COPS4 | 1.03E-01 | 1 |
| EPB41L3 | 8.01E-02 | 1 | CNGB3 | 1.03E-01 | 1 |
| SGCE | 8.04E-02 | 1 | RGL2 | 1.03E-01 | 1 |
| TCN1 | 8.05E-02 | 1 | TUBE1 | 1.03E-01 | 1 |
| TMEM176 | 8.06E-02 | 1 | C5orf45 | 1.03E-01 | 1 |
| GRAMD1 | 8.07E-02 | 1 | PLEKHB1 | 1.04E-01 | 1 |
| SLC35E1 | 8.08E-02 | 1 | CREG2 | 1.04E-01 | 1 |
| CRH | 8.09E-02 | 1 | AQP2 | 1.04E-01 | 1 |
| CA9 | 8.09E-02 | 1 | TYMP | 1.04E-01 | 1 |
| ZNF565 | 8.10E-02 | 1 | ATXN7L3 | 1.04E-01 | 1 |
| ZNF827 | 8.11E-02 | 1 | PLA2R1 | 1.04E-01 | 1 |
| MST1 | 8.12E-02 | 1 | OPRM1 | 1.05E-01 | 1 |
| AANAT | 8.14E-02 | 1 | ZNF75A | 1.05E-01 | 1 |
| DRAP1 | 8.14E-02 | 1 | KRTAP1-1 | 1.05E-01 | 1 |
| SYS1 | 8.14E-02 | 1 | ERCC1 | 1.05E-01 | 1 |
| DPP7 | 8.17E-02 | 1 | TPST1 | 1.05E-01 | 1 |
| FYCO1 | 8.17E-02 | 1 | SLC26A6 | 1.05E-01 | 1 |
| CLEC18C | 8.17E-02 | 1 | MPPED1 | 1.05E-01 | 1 |
| FAM46D | 8.18E-02 | 1 | FAM71D | 1.05E-01 | 1 |
| TBX6 | 8.19E-02 | 1 | SAGE1 | 1.05E-01 | 1 |
| TP53I11 | 8.19E-02 | 1 | SDC2 | 1.05E-01 | 1 |
| BHLHE41 | 8.21E-02 | 1 | ZNF135 | 1.05E-01 | 1 |
| RP9 | 8.24E-02 | 1 | 4-Mar | 1.06E-01 | 1 |
| NAT14 | 8.25E-02 | 1 | OR10R2 | 1.06E-01 | 1 |
| PTPRA | 8.28E-02 | 1 | ATL1 | 1.06E-01 | 1 |
| C16orf93 | 8.30E-02 | 1 | SULT1C4 | 1.06E-01 | 1 |
| GPSM1 | 8.31E-02 | 1 | ABR | 1.06E-01 | 1 |
| PLA2G6 | 8.31E-02 | 1 | ESX1 | 1.06E-01 | 1 |
| NXF1 | 8.32E-02 | 1 | MPHOSPH1 | 1.06E-01 | 1 |
| SLA2 | 8.34E-02 | 1 | KRTAP10-1 | 1.06E-01 | 1 |
| IL2RB | 8.37E-02 | 1 | IDH2 | 1.07E-01 | 1 |
| REPS1 | 8.38E-02 | 1 | ZNF227 | 1.07E-01 | 1 |
| CAPN6 | 8.40E-02 | 1 | PVRIG | 1.07E-01 | 1 |
| ZNRF3 | 8.41E-02 | 1 | FOXE3 | 1.07E-01 | 1 |
| DHX8 | 8.43E-02 | 1 | DGAT2L6 | 1.07E-01 | 1 |
| ADIG | 8.45E-02 | 1 | PCYT1A | 1.07E-01 | 1 |
| OR10A6 | 8.45E-02 | 1 | GYPB | 1.07E-01 | 1 |
| SLC10A7 | 8.48E-02 | 1 | SLC2A4RG | 1.07E-01 | 1 |

| LHFPL4 | 8.50E-02 | 1 | CRISP1 | 1.08E-01 | 1 |
| --- | --- | --- | --- | --- | --- |
| C7orf57 | 8.50E-02 | 1 | UBE2N | 1.08E-01 | 1 |
| RBL2 | 8.50E-02 | 1 | PPP1R13B | 1.08E-01 | 1 |
| C3orf58 | 8.52E-02 | 1 | FOXB1 | 1.08E-01 | 1 |
| C8orf31 | 8.52E-02 | 1 | ORM2 | 1.08E-01 | 1 |
| ARL5A | 8.56E-02 | 1 | KRT38 | 1.08E-01 | 1 |
| ANAPC11 | 8.58E-02 | 1 | APOB | 1.08E-01 | 1 |
| BTC | 8.60E-02 | 1 | HIST1H2BC | 1.08E-01 | 1 |
| CYP3A7 | 8.63E-02 | 1 | GJD2 | 1.09E-01 | 1 |
| HIST1H1E | 8.64E-02 | 1 | KRTAP10-2 | 1.09E-01 | 1 |
| ICT1 | 8.66E-02 | 1 | MC4R | 1.09E-01 | 1 |
| GLCE | 8.66E-02 | 1 | HDAC4 | 1.09E-01 | 1 |
| SLIT2 | 8.67E-02 | 1 | C8orf58 | 1.09E-01 | 1 |
| ACAT2 | 8.69E-02 | 1 | KPNA2 | 1.09E-01 | 1 |
| SCRN3 | 8.70E-02 | 1 | KIR2DL1 | 1.09E-01 | 1 |
| ZNF793 | 8.70E-02 | 1 | TOX4 | 1.09E-01 | 1 |
| C2orf70 | 8.70E-02 | 1 | CADPS | 1.09E-01 | 1 |
| LGSN | 8.71E-02 | 1 | SCOC | 1.09E-01 | 1 |
| KCNK9 | 8.75E-02 | 1 | NUDT6 | 1.10E-01 | 1 |
| FBXO8 | 8.76E-02 | 1 | ING5 | 1.10E-01 | 1 |
| FCGR1A | 8.76E-02 | 1 | DOCK10 | 1.10E-01 | 1 |
| FGF7 | 8.77E-02 | 1 | PRSS21 | 1.10E-01 | 1 |
| SLC16A4 | 8.78E-02 | 1 | TRMT61B | 1.10E-01 | 1 |
| IRS4 | 8.79E-02 | 1 | SHISA6 | 1.10E-01 | 1 |
| AVPR1A | 8.80E-02 | 1 | TSC1 | 1.10E-01 | 1 |
| LRRC57 | 8.82E-02 | 1 | ADAMTS3 | 1.10E-01 | 1 |
| PCP2 | 8.83E-02 | 1 | DCDC1 | 1.10E-01 | 1 |
| ZNF132 | 8.83E-02 | 1 | UCN | 1.10E-01 | 1 |
| MGAT5 | 8.83E-02 | 1 | RAB32 | 1.10E-01 | 1 |
| C1orf94 | 8.84E-02 | 1 | AHSA1 | 1.11E-01 | 1 |
| UBXN1 | 8.85E-02 | 1 | ACE | 1.11E-01 | 1 |
| WDR81 | 8.85E-02 | 1 | TYSND1 | 1.11E-01 | 1 |
| WEE2 | 8.85E-02 | 1 | OLA1 | 1.11E-01 | 1 |
| PDZD4 | 8.86E-02 | 1 | RNF175 | 1.11E-01 | 1 |
| ALOXE3 | 8.87E-02 | 1 | ORMDL1 | 1.11E-01 | 1 |
| WASF3 | 8.87E-02 | 1 | PDIK1L | 1.11E-01 | 1 |
| CD69 | 8.89E-02 | 1 | SHMT1 | 1.11E-01 | 1 |
| POTEF | 8.91E-02 | 1 | RESP18 | 1.11E-01 | 1 |
| CASS4 | 8.91E-02 | 1 | GTSF1L | 1.11E-01 | 1 |
| ELP4 | 8.93E-02 | 1 | NOSIP | 1.11E-01 | 1 |
| RSPO2 | 8.95E-02 | 1 | FASN | 1.12E-01 | 1 |
| UBXN2A | 9.00E-02 | 1 | NEUROD1 | 1.12E-01 | 1 |
| DHRS4L2 | 9.01E-02 | 1 | SIRPG | 1.12E-01 | 1 |
| ZNF239 | 9.01E-02 | 1 | TBX19 | 1.12E-01 | 1 |
| GAS2L1 | 9.02E-02 | 1 | AMZ1 | 1.12E-01 | 1 |
| MAP1LC3 | 9.02E-02 | 1 | GPR68 | 1.12E-01 | 1 |
| CCDC103 | 9.02E-02 | 1 | PPP1R11 | 1.12E-01 | 1 |
| LRCH3 | 9.05E-02 | 1 | ANXA7 | 1.12E-01 | 1 |
| PGS1 | 9.06E-02 | 1 | CORO1C | 1.12E-01 | 1 |
| ITIH3 | 9.09E-02 | 1 | TRO | 1.12E-01 | 1 |
| NPS | 9.16E-02 | 1 | CST6 | 1.12E-01 | 1 |
| PEX13 | 9.17E-02 | 1 | THSD1 | 1.13E-01 | 1 |
| REST | 9.18E-02 | 1 | GUCA2B | 1.13E-01 | 1 |

| SMEK1 | 9.19E-02 | 1 | OBFC1 | 1.13E-01 | 1 |
| --- | --- | --- | --- | --- | --- |
| BZW2 | 9.22E-02 | 1 | LPIN2 | 1.13E-01 | 1 |
| NME6 | 9.22E-02 | 1 | EPSTI1 | 1.13E-01 | 1 |
| S100A3 | 9.24E-02 | 1 | WASF2 | 1.13E-01 | 1 |
| CXorf38 | 9.25E-02 | 1 | CCDC71 | 1.13E-01 | 1 |
| IWS1 | 9.25E-02 | 1 | PLSCR1 | 1.13E-01 | 1 |
| SMARCA | 9.25E-02 | 1 | RAD51AP2 | 1.14E-01 | 1 |
| IFNA14 | 9.27E-02 | 1 | AGPAT3 | 1.14E-01 | 1 |
| C1orf158 | 9.27E-02 | 1 | PBRM1 | 1.14E-01 | 1 |
| OR4A47 | 9.28E-02 | 1 | ARTN | 1.14E-01 | 1 |
| HMGXB4 | 9.29E-02 | 1 | POPDC3 | 1.14E-01 | 1 |
| GRAP | 9.30E-02 | 1 | JKAMP | 1.14E-01 | 1 |
| CRYGD | 9.31E-02 | 1 | HSD17B3 | 1.14E-01 | 1 |
| MIB1 | 9.32E-02 | 1 | GNRH2 | 1.15E-01 | 1 |
| FBXO38 | 9.33E-02 | 1 | AWAT2 | 1.15E-01 | 1 |
| S100A5 | 9.36E-02 | 1 | MC2R | 1.15E-01 | 1 |
| CDC14B | 9.38E-02 | 1 | NR1I2 | 1.15E-01 | 1 |
| C5orf28 | 9.39E-02 | 1 | RASD2 | 1.15E-01 | 1 |
| OR6N2 | 9.40E-02 | 1 | PPBP | 1.15E-01 | 1 |
| NKIRAS2 | 9.40E-02 | 1 | GRK7 | 1.15E-01 | 1 |
| C6orf223 | 9.41E-02 | 1 | RPS4Y1 | 1.15E-01 | 1 |
| VWDE | 9.41E-02 | 1 | HIST1H3D | 1.15E-01 | 1 |
| NFIC | 9.42E-02 | 1 | CASP8 | 1.15E-01 | 1 |
| CHUK | 9.43E-02 | 1 | KLHL35 | 1.15E-01 | 1 |
| ATR | 9.45E-02 | 1 | HLA-DMA | 1.16E-01 | 1 |
| HEATR3 | 9.46E-02 | 1 | METTL11B | 1.16E-01 | 1 |
| OLFML2A | 9.49E-02 | 1 | DPEP2 | 1.16E-01 | 1 |
| C6orf165 | 9.49E-02 | 1 | VGLL4 | 1.16E-01 | 1 |
| THAP3 | 9.51E-02 | 1 | ZBTB1 | 1.16E-01 | 1 |
| HNRNPUL | 9.53E-02 | 1 | SLC39A8 | 1.16E-01 | 1 |
| GNB1 | 9.54E-02 | 1 | CALHM1 | 1.16E-01 | 1 |
| KCNN3 | 9.54E-02 | 1 | ZNF841 | 1.16E-01 | 1 |
| FAM131B | 9.59E-02 | 1 | GRM6 | 1.17E-01 | 1 |
| KRTAP21 | 9.59E-02 | 1 | OR14A16 | 1.17E-01 | 1 |
| ATP6V1F | 9.60E-02 | 1 | MNX1 | 1.17E-01 | 1 |
| OR2T6 | 9.63E-02 | 1 | TNP1 | 1.17E-01 | 1 |
| CEP68 | 9.63E-02 | 1 | TMEM38B | 1.17E-01 | 1 |
| PLEKHB2 | 9.64E-02 | 1 | STK32C | 1.17E-01 | 1 |
| CEACAM | 9.65E-02 | 1 | SPRR3 | 1.17E-01 | 1 |
| DYRK3 | 9.66E-02 | 1 | DRD2 | 1.18E-01 | 1 |
| MMP10 | 9.66E-02 | 1 | F13A1 | 1.18E-01 | 1 |
| ATP5B | 9.68E-02 | 1 | TMEM87B | 1.18E-01 | 1 |
| ADRBK2 | 9.69E-02 | 1 | TMEM110 | 1.18E-01 | 1 |
| NDUFB10 | 9.70E-02 | 1 | DMRTC2 | 1.18E-01 | 1 |
| ERRFI1 | 9.70E-02 | 1 | DACH2 | 1.18E-01 | 1 |
| KLRC1 | 9.71E-02 | 1 | PPARGC1B | 1.18E-01 | 1 |
| ZNF676 | 9.71E-02 | 1 | CAST | 1.18E-01 | 1 |
| ABCG1 | 9.72E-02 | 1 | GRSF1 | 1.18E-01 | 1 |
| CTSB | 9.72E-02 | 1 | FCN1 | 1.18E-01 | 1 |
| ARSI | 9.73E-02 | 1 | PCSK9 | 1.18E-01 | 1 |
| ANKRD34 | 9.73E-02 | 1 | GPR174 | 1.19E-01 | 1 |
| OR8K1 | 9.75E-02 | 1 | PRAMEF18 | 1.19E-01 | 1 |
| FUBP1 | 9.77E-02 | 1 | NADK | 1.19E-01 | 1 |

| RFPL4B | 9.78E-02 | 1 | H2AFY2 | 1.19E-01 | 1 |
| --- | --- | --- | --- | --- | --- |
| PDE6B | 9.82E-02 | 1 | THRSP | 1.19E-01 | 1 |
| KCNA1 | 9.82E-02 | 1 | DEFB116 | 1.19E-01 | 1 |
| AP3M2 | 9.82E-02 | 1 | OR2D3 | 1.19E-01 | 1 |
| KRTAP9-3 | 9.84E-02 | 1 | UCHL1 | 1.19E-01 | 1 |
| CDK5R2 | 9.84E-02 | 1 | KRTAP10-1 | 1.19E-01 | 1 |
| CRIP2 | 9.84E-02 | 1 | SHOX | 1.19E-01 | 1 |
| MGAT3 | 9.86E-02 | 1 | KPRP | 1.19E-01 | 1 |
| PRKAG2 | 9.88E-02 | 1 | CHUK | 1.19E-01 | 1 |
| PITX3 | 9.90E-02 | 1 | BTN2A2 | 1.19E-01 | 1 |
| NFXL1 | 9.90E-02 | 1 | MRPL47 | 1.19E-01 | 1 |
| TRPM8 | 9.93E-02 | 1 | KCTD15 | 1.19E-01 | 1 |
| KIF26A | 1.00E-01 | 1 | SERPINE2 | 1.20E-01 | 1 |
| NANP | 1.00E-01 | 1 | INTS3 | 1.20E-01 | 1 |
| ACAD11 | 1.00E-01 | 1 | GUCA2A | 1.20E-01 | 1 |
| LEF1 | 1.00E-01 | 1 | REEP6 | 1.20E-01 | 1 |
| PDZK1 | 1.01E-01 | 1 | PRLHR | 1.20E-01 | 1 |
| OR4N5 | 1.01E-01 | 1 | TREX2 | 1.21E-01 | 1 |
| ENTHD1 | 1.01E-01 | 1 | LY6G5B | 1.21E-01 | 1 |
| HIST1H3I | 1.02E-01 | 1 | MIDN | 1.21E-01 | 1 |
| C11orf54 | 1.02E-01 | 1 | QPCT | 1.21E-01 | 1 |
| SAR1B | 1.02E-01 | 1 | POLDIP3 | 1.21E-01 | 1 |
| TRAT1 | 1.02E-01 | 1 | TXNL1 | 1.22E-01 | 1 |
| PDE1C | 1.02E-01 | 1 | TNKS2 | 1.22E-01 | 1 |
| LYPLA2 | 1.02E-01 | 1 | TEX10 | 1.22E-01 | 1 |
| BRSK1 | 1.02E-01 | 1 | HOXB5 | 1.22E-01 | 1 |
| YIPF1 | 1.02E-01 | 1 | RNF133 | 1.22E-01 | 1 |
| KRT35 | 1.02E-01 | 1 | OR5B17 | 1.22E-01 | 1 |
| SRRT | 1.02E-01 | 1 | PHC3 | 1.22E-01 | 1 |
| FRMD1 | 1.02E-01 | 1 | TCEANC | 1.22E-01 | 1 |
| RIC3 | 1.02E-01 | 1 | KCNMB3 | 1.23E-01 | 1 |
| TSPAN4 | 1.02E-01 | 1 | ELK3 | 1.23E-01 | 1 |
| ERBB4 | 1.03E-01 | 1 | PNPT1 | 1.23E-01 | 1 |
| VPS37B | 1.03E-01 | 1 | SRRM4 | 1.23E-01 | 1 |
| BAX | 1.03E-01 | 1 | OR7A10 | 1.23E-01 | 1 |
| LRP8 | 1.03E-01 | 1 | ZNF565 | 1.23E-01 | 1 |
| OR8H1 | 1.03E-01 | 1 | HNRNPL | 1.23E-01 | 1 |
| FGB | 1.03E-01 | 1 | CCND2 | 1.24E-01 | 1 |
| COMP | 1.03E-01 | 1 | WNT8A | 1.24E-01 | 1 |
| ZDHHC1 | 1.03E-01 | 1 | CD6 | 1.24E-01 | 1 |
| MBD3L1 | 1.04E-01 | 1 | GMPS | 1.24E-01 | 1 |
| RCN2 | 1.04E-01 | 1 | SLC16A4 | 1.24E-01 | 1 |
| TMEM150 | 1.04E-01 | 1 | SH2D2A | 1.24E-01 | 1 |
| ZDHHC4 | 1.04E-01 | 1 | C1orf61 | 1.24E-01 | 1 |
| PTGER3 | 1.04E-01 | 1 | RUFY4 | 1.24E-01 | 1 |
| TOB2 | 1.04E-01 | 1 | CXCL14 | 1.24E-01 | 1 |
| ALPPL2 | 1.05E-01 | 1 | TDRD5 | 1.24E-01 | 1 |
| SP3 | 1.05E-01 | 1 | IL27RA | 1.24E-01 | 1 |
| CCT3 | 1.05E-01 | 1 | GRM4 | 1.24E-01 | 1 |
| ATCAY | 1.05E-01 | 1 | ETFDH | 1.24E-01 | 1 |
| PCDHB11 | 1.05E-01 | 1 | CRB3 | 1.24E-01 | 1 |
| NDUFAF4 | 1.05E-01 | 1 | LRRC28 | 1.24E-01 | 1 |
| FAM92A1 | 1.05E-01 | 1 | SLC16A6 | 1.24E-01 | 1 |

| PTPRK | 1.05E-01 | 1 | SLC9A4 | 1.24E-01 | 1 |
| --- | --- | --- | --- | --- | --- |
| TMEM177 | 1.05E-01 | 1 | CEACAM8 | 1.24E-01 | 1 |
| LYVE1 | 1.05E-01 | 1 | PADI3 | 1.25E-01 | 1 |
| GALNTL5 | 1.05E-01 | 1 | FLYWCH2 | 1.25E-01 | 1 |
| MKRN3 | 1.05E-01 | 1 | BACE1 | 1.25E-01 | 1 |
| GTF2E1 | 1.05E-01 | 1 | MXD1 | 1.25E-01 | 1 |
| FAM45A | 1.06E-01 | 1 | LAMP2 | 1.25E-01 | 1 |
| ARHGEF3 | 1.06E-01 | 1 | KIAA1147 | 1.25E-01 | 1 |
| C5orf51 | 1.06E-01 | 1 | KLHL21 | 1.25E-01 | 1 |
| EMB | 1.06E-01 | 1 | SNAP91 | 1.25E-01 | 1 |
| CST5 | 1.06E-01 | 1 | NUDT10 | 1.26E-01 | 1 |
| CCDC78 | 1.06E-01 | 1 | PACSIN3 | 1.26E-01 | 1 |
| TIMM13 | 1.06E-01 | 1 | HDAC9 | 1.26E-01 | 1 |
| OR51I1 | 1.06E-01 | 1 | KCNS3 | 1.26E-01 | 1 |
| C1orf68 | 1.06E-01 | 1 | OR8G1 | 1.26E-01 | 1 |
| TRIM61 | 1.06E-01 | 1 | DUOX2 | 1.26E-01 | 1 |
| PTPMT1 | 1.07E-01 | 1 | 11-Mar | 1.27E-01 | 1 |
| BIN1 | 1.07E-01 | 1 | RNASEH2C | 1.27E-01 | 1 |
| TSSC4 | 1.07E-01 | 1 | NCS1 | 1.27E-01 | 1 |
| PDDC1 | 1.07E-01 | 1 | AFF1 | 1.27E-01 | 1 |
| HIRIP3 | 1.07E-01 | 1 | ENO3 | 1.27E-01 | 1 |
| ADAM28 | 1.07E-01 | 1 | LCE4A | 1.27E-01 | 1 |
| HTR2C | 1.08E-01 | 1 | SLPI | 1.27E-01 | 1 |
| ADAT2 | 1.08E-01 | 1 | HIST1H4J | 1.27E-01 | 1 |
| GPR143 | 1.08E-01 | 1 | TTL | 1.28E-01 | 1 |
| SRP72 | 1.08E-01 | 1 | IQCA1 | 1.28E-01 | 1 |
| SLC26A11 | 1.08E-01 | 1 | CAMP | 1.28E-01 | 1 |
| DNAJB12 | 1.08E-01 | 1 | ARX | 1.28E-01 | 1 |
| CRIP3 | 1.08E-01 | 1 | DUSP6 | 1.28E-01 | 1 |
| GPR3 | 1.08E-01 | 1 | ATG4C | 1.28E-01 | 1 |
| WBSCR16 | 1.08E-01 | 1 | HOXA3 | 1.28E-01 | 1 |
| GLP1R | 1.08E-01 | 1 | LGALS7 | 1.28E-01 | 1 |
| ALG1L2 | 1.08E-01 | 1 | CD83 | 1.29E-01 | 1 |
| APP | 1.09E-01 | 1 | CAMK1D | 1.29E-01 | 1 |
| NME1 | 1.09E-01 | 1 | SERPINC1 | 1.29E-01 | 1 |
| IFI35 | 1.09E-01 | 1 | EPGN | 1.29E-01 | 1 |
| HOXA2 | 1.09E-01 | 1 | SLC6A11 | 1.29E-01 | 1 |
| CKS1B | 1.09E-01 | 1 | FLOT1 | 1.30E-01 | 1 |
| FAM83D | 1.09E-01 | 1 | POTEH | 1.30E-01 | 1 |
| FBXL4 | 1.09E-01 | 1 | DIRAS1 | 1.30E-01 | 1 |
| SCGB1D2 | 1.09E-01 | 1 | TRIM31 | 1.30E-01 | 1 |
| SNRNP40 | 1.09E-01 | 1 | L2HGDH | 1.30E-01 | 1 |
| PICK1 | 1.10E-01 | 1 | HAVCR1 | 1.30E-01 | 1 |
| OR13H1 | 1.10E-01 | 1 | TNFRSF10B | 1.30E-01 | 1 |
| LMBRD1 | 1.10E-01 | 1 | OR2A2 | 1.30E-01 | 1 |
| PSMA5 | 1.10E-01 | 1 | OAS1 | 1.30E-01 | 1 |
| CCK | 1.10E-01 | 1 | KRTAP13-3 | 1.30E-01 | 1 |
| CHCHD4 | 1.10E-01 | 1 | MTX3 | 1.30E-01 | 1 |
| STXBP1 | 1.10E-01 | 1 | IL2RA | 1.31E-01 | 1 |
| MYO3A | 1.11E-01 | 1 | IL12B | 1.31E-01 | 1 |
| AQP2 | 1.11E-01 | 1 | TSEN34 | 1.31E-01 | 1 |
| HLA-G | 1.11E-01 | 1 | CELA1 | 1.31E-01 | 1 |
| PLRG1 | 1.11E-01 | 1 | MPP5 | 1.31E-01 | 1 |

| MTMR6 | 1.11E-01 | 1 | OR5H14 | 1.31E-01 | 1 |
| --- | --- | --- | --- | --- | --- |
| C21orf58 | 1.11E-01 | 1 | SNX27 | 1.31E-01 | 1 |
| VCX | 1.11E-01 | 1 | KRT5 | 1.31E-01 | 1 |
| PCDHGA5 | 1.12E-01 | 1 | CRLF1 | 1.31E-01 | 1 |
| SF3B3 | 1.12E-01 | 1 | UBE2M | 1.31E-01 | 1 |
| ATP2C2 | 1.12E-01 | 1 | AKR7L | 1.31E-01 | 1 |
| PSMA1 | 1.12E-01 | 1 | BRCC3 | 1.32E-01 | 1 |
| CYP11B2 | 1.12E-01 | 1 | IAH1 | 1.32E-01 | 1 |
| EHBP1L1 | 1.12E-01 | 1 | YIPF3 | 1.32E-01 | 1 |
| TMX1 | 1.12E-01 | 1 | CELF3 | 1.33E-01 | 1 |
| SNAPC2 | 1.12E-01 | 1 | SETD8 | 1.33E-01 | 1 |
| PSG1 | 1.12E-01 | 1 | HPD | 1.33E-01 | 1 |
| DCAF4L1 | 1.12E-01 | 1 | ABCE1 | 1.33E-01 | 1 |
| LDHAL6A | 1.13E-01 | 1 | IP6K1 | 1.34E-01 | 1 |
| LRRC43 | 1.13E-01 | 1 | C1orf146 | 1.34E-01 | 1 |
| 4-Mar | 1.13E-01 | 1 | IRF9 | 1.34E-01 | 1 |
| AP2M1 | 1.13E-01 | 1 | SYNC | 1.34E-01 | 1 |
| ZG16B | 1.13E-01 | 1 | ANKRD13B | 1.34E-01 | 1 |
| ASAP3 | 1.13E-01 | 1 | COL18A1 | 1.34E-01 | 1 |
| PCDHGA4 | 1.13E-01 | 1 | GDF2 | 1.34E-01 | 1 |
| CYP8B1 | 1.13E-01 | 1 | DEFB114 | 1.34E-01 | 1 |
| PAX3 | 1.14E-01 | 1 | PGLYRP4 | 1.34E-01 | 1 |
| ATG5 | 1.14E-01 | 1 | OR10C1 | 1.34E-01 | 1 |
| PPAP2A | 1.14E-01 | 1 | HIST1H3F | 1.34E-01 | 1 |
| DDC | 1.14E-01 | 1 | GRN | 1.35E-01 | 1 |
| BPHL | 1.14E-01 | 1 | LAT | 1.35E-01 | 1 |
| PTGFR | 1.14E-01 | 1 | FXYD4 | 1.35E-01 | 1 |
| TRIP10 | 1.14E-01 | 1 | TDRD9 | 1.35E-01 | 1 |
| C6orf47 | 1.14E-01 | 1 | SDC4 | 1.35E-01 | 1 |
| ABHD2 | 1.14E-01 | 1 | ASS1 | 1.35E-01 | 1 |
| UBE2E1 | 1.14E-01 | 1 | ASAH1 | 1.35E-01 | 1 |
| CCDC53 | 1.14E-01 | 1 | SCP2 | 1.36E-01 | 1 |
| OR51A4 | 1.15E-01 | 1 | URM1 | 1.36E-01 | 1 |
| ATP8A2 | 1.15E-01 | 1 | CWF19L1 | 1.36E-01 | 1 |
| RFTN1 | 1.15E-01 | 1 | CD80 | 1.36E-01 | 1 |
| AOX1 | 1.15E-01 | 1 | USP40 | 1.36E-01 | 1 |
| FAM20C | 1.15E-01 | 1 | NCR2 | 1.36E-01 | 1 |
| CD300C | 1.15E-01 | 1 | ST6GALNA | 1.36E-01 | 1 |
| ATP2B2 | 1.15E-01 | 1 | MYO6 | 1.36E-01 | 1 |
| FAM131C | 1.15E-01 | 1 | PDK3 | 1.36E-01 | 1 |
| IGF2 | 1.15E-01 | 1 | CNTN2 | 1.37E-01 | 1 |
| SLC46A1 | 1.16E-01 | 1 | CD46 | 1.37E-01 | 1 |
| NAP1L5 | 1.16E-01 | 1 | BOD1 | 1.37E-01 | 1 |
| C12orf76 | 1.16E-01 | 1 | MLANA | 1.37E-01 | 1 |
| FCF1 | 1.16E-01 | 1 | PIN4 | 1.37E-01 | 1 |
| MAZ | 1.17E-01 | 1 | IRF2BP1 | 1.37E-01 | 1 |
| DPP9 | 1.17E-01 | 1 | LCOR | 1.37E-01 | 1 |
| GRM8 | 1.17E-01 | 1 | SNF8 | 1.37E-01 | 1 |
| UNC119B | 1.17E-01 | 1 | POLR1C | 1.37E-01 | 1 |
| KRT72 | 1.17E-01 | 1 | TRIP10 | 1.38E-01 | 1 |
| KRTAP5-4 | 1.17E-01 | 1 | TGFA | 1.38E-01 | 1 |
| PSMA8 | 1.17E-01 | 1 | OR9A2 | 1.38E-01 | 1 |
| EAF2 | 1.17E-01 | 1 | ZNF521 | 1.38E-01 | 1 |

| TMPRSS7 | 1.18E-01 | 1 | TRIM23 | 1.38E-01 | 1 |
| --- | --- | --- | --- | --- | --- |
| HTT | 1.18E-01 | 1 | OR8D1 | 1.38E-01 | 1 |
| SLC4A3 | 1.18E-01 | 1 | B3GNTL1 | 1.38E-01 | 1 |
| TMEM156 | 1.18E-01 | 1 | OR10K2 | 1.38E-01 | 1 |
| RNASE3 | 1.18E-01 | 1 | P2RY4 | 1.39E-01 | 1 |
| DTNA | 1.18E-01 | 1 | TAGAP | 1.39E-01 | 1 |
| IL17REL | 1.18E-01 | 1 | SAMD7 | 1.39E-01 | 1 |
| ZNF488 | 1.18E-01 | 1 | ASAP1 | 1.39E-01 | 1 |
| GOLGA7B | 1.18E-01 | 1 | TNFSF4 | 1.39E-01 | 1 |
| HIST1H4G | 1.18E-01 | 1 | WNT11 | 1.39E-01 | 1 |
| ARHGAP3 | 1.18E-01 | 1 | NOC3L | 1.39E-01 | 1 |
| UBE2Q2 | 1.18E-01 | 1 | RHCG | 1.39E-01 | 1 |
| DHX37 | 1.18E-01 | 1 | IRX4 | 1.39E-01 | 1 |
| TPM2 | 1.18E-01 | 1 | OR10T2 | 1.39E-01 | 1 |
| DUPD1 | 1.19E-01 | 1 | GCDH | 1.40E-01 | 1 |
| RGPD4 | 1.19E-01 | 1 | CSNK1E | 1.40E-01 | 1 |
| KLHL32 | 1.19E-01 | 1 | WBP2 | 1.40E-01 | 1 |
| CBX4 | 1.19E-01 | 1 | ENOSF1 | 1.40E-01 | 1 |
| SIGLEC10 | 1.19E-01 | 1 | TMEM44 | 1.40E-01 | 1 |
| C19orf38 | 1.19E-01 | 1 | OR4D5 | 1.40E-01 | 1 |
| ATP1A1 | 1.19E-01 | 1 | MYO1H | 1.40E-01 | 1 |
| TCF20 | 1.19E-01 | 1 | FAM92B | 1.40E-01 | 1 |
| ADH1B | 1.19E-01 | 1 | SLC22A9 | 1.40E-01 | 1 |
| RPLP0 | 1.19E-01 | 1 | DIO2 | 1.40E-01 | 1 |
| NFIX | 1.19E-01 | 1 | FAM32A | 1.40E-01 | 1 |
| GFM2 | 1.19E-01 | 1 | OR6C75 | 1.40E-01 | 1 |
| AQP6 | 1.19E-01 | 1 | NPY5R | 1.41E-01 | 1 |
| PSMC6 | 1.20E-01 | 1 | PDE7B | 1.41E-01 | 1 |
| TPRG1 | 1.20E-01 | 1 | HIST2H3D | 1.41E-01 | 1 |
| DNAJB4 | 1.20E-01 | 1 | TCF25 | 1.41E-01 | 1 |
| SFMBT2 | 1.20E-01 | 1 | CACNA1C | 1.41E-01 | 1 |
| FBXO27 | 1.20E-01 | 1 | KCNJ14 | 1.41E-01 | 1 |
| CACNA2D | 1.20E-01 | 1 | OPA1 | 1.41E-01 | 1 |
| SEMG2 | 1.21E-01 | 1 | SLC6A15 | 1.41E-01 | 1 |
| PSMC2 | 1.21E-01 | 1 | OCM | 1.42E-01 | 1 |
| ITPR3 | 1.21E-01 | 1 | CLEC2B | 1.42E-01 | 1 |
| ITGB3 | 1.21E-01 | 1 | RPL18A | 1.42E-01 | 1 |
| EIF2B2 | 1.21E-01 | 1 | TPH1 | 1.42E-01 | 1 |
| C17orf49 | 1.21E-01 | 1 | TTC38 | 1.42E-01 | 1 |
| BCAS1 | 1.21E-01 | 1 | RALGPS1 | 1.42E-01 | 1 |
| DKKL1 | 1.21E-01 | 1 | SLC25A39 | 1.42E-01 | 1 |
| PPOX | 1.21E-01 | 1 | HTR4 | 1.43E-01 | 1 |
| KRAS | 1.21E-01 | 1 | FN1 | 1.43E-01 | 1 |
| ASCL1 | 1.21E-01 | 1 | KLHL3 | 1.43E-01 | 1 |
| LSM10 | 1.21E-01 | 1 | NUTF2 | 1.43E-01 | 1 |
| SPIC | 1.22E-01 | 1 | CTU2 | 1.43E-01 | 1 |
| RPL35A | 1.22E-01 | 1 | GRXCR1 | 1.43E-01 | 1 |
| SARDH | 1.22E-01 | 1 | NIP7 | 1.43E-01 | 1 |
| GYLTL1B | 1.22E-01 | 1 | KCNA6 | 1.43E-01 | 1 |
| DNASE1L | 1.22E-01 | 1 | SYDE1 | 1.43E-01 | 1 |
| MRGPRF | 1.22E-01 | 1 | TACR2 | 1.43E-01 | 1 |
| OR51E2 | 1.22E-01 | 1 | SLC30A8 | 1.44E-01 | 1 |
| TTC38 | 1.22E-01 | 1 | TTC30A | 1.44E-01 | 1 |

| KRT34 | 1.23E-01 | 1 | AFM | 1.44E-01 | 1 |
| --- | --- | --- | --- | --- | --- |
| WBSCR22 | 1.23E-01 | 1 | EIF5AL1 | 1.44E-01 | 1 |
| CTNNA2 | 1.23E-01 | 1 | ARL8A | 1.44E-01 | 1 |
| ANKRD30 | 1.23E-01 | 1 | IGSF9B | 1.44E-01 | 1 |
| FOXK2 | 1.23E-01 | 1 | LY75 | 1.44E-01 | 1 |
| WDR60 | 1.23E-01 | 1 | ELMOD1 | 1.44E-01 | 1 |
| MECOM | 1.23E-01 | 1 | MAGI3 | 1.44E-01 | 1 |
| IFNB1 | 1.23E-01 | 1 | SPHK1 | 1.44E-01 | 1 |
| KRT2 | 1.23E-01 | 1 | PPM1D | 1.44E-01 | 1 |
| PDE7A | 1.23E-01 | 1 | PKNOX2 | 1.44E-01 | 1 |
| PSMB7 | 1.24E-01 | 1 | GPS2 | 1.45E-01 | 1 |
| MC1R | 1.24E-01 | 1 | PPP1R9B | 1.45E-01 | 1 |
| C1RL | 1.24E-01 | 1 | GABRG2 | 1.45E-01 | 1 |
| NEK2 | 1.24E-01 | 1 | MTMR14 | 1.45E-01 | 1 |
| ASMT | 1.24E-01 | 1 | NPBWR2 | 1.45E-01 | 1 |
| THOP1 | 1.24E-01 | 1 | TNFRSF4 | 1.45E-01 | 1 |
| GJA8 | 1.24E-01 | 1 | FAM134C | 1.45E-01 | 1 |
| RAB40B | 1.24E-01 | 1 | NUDT15 | 1.46E-01 | 1 |
| UBE2U | 1.24E-01 | 1 | KLF5 | 1.46E-01 | 1 |
| BACH1 | 1.24E-01 | 1 | TEAD1 | 1.46E-01 | 1 |
| SH3RF2 | 1.24E-01 | 1 | DLK2 | 1.46E-01 | 1 |
| PRRT1 | 1.24E-01 | 1 | OR10S1 | 1.46E-01 | 1 |
| SLC25A15 | 1.24E-01 | 1 | CTSH | 1.46E-01 | 1 |
| MT1E | 1.25E-01 | 1 | TMEM170A | 1.46E-01 | 1 |
| PCDHB14 | 1.25E-01 | 1 | PRF1 | 1.46E-01 | 1 |
| KRTAP5-3 | 1.25E-01 | 1 | SPATC1 | 1.46E-01 | 1 |
| ZNF383 | 1.25E-01 | 1 | CMAS | 1.46E-01 | 1 |
| U2AF2 | 1.25E-01 | 1 | MRAS | 1.47E-01 | 1 |
| ATOX1 | 1.25E-01 | 1 | PRSS8 | 1.47E-01 | 1 |
| RBPMS | 1.26E-01 | 1 | OR10G3 | 1.47E-01 | 1 |
| SF3B1 | 1.26E-01 | 1 | SPINK6 | 1.47E-01 | 1 |
| SYCP2 | 1.26E-01 | 1 | AKR1B15 | 1.47E-01 | 1 |
| C1QTNF4 | 1.26E-01 | 1 | DRP2 | 1.47E-01 | 1 |
| PANK4 | 1.26E-01 | 1 | WSB2 | 1.47E-01 | 1 |
| FXYD4 | 1.26E-01 | 1 | LYZL6 | 1.47E-01 | 1 |
| C1QTNF7 | 1.27E-01 | 1 | BATF | 1.47E-01 | 1 |
| ZNF706 | 1.27E-01 | 1 | PREB | 1.47E-01 | 1 |
| ZSCAN21 | 1.27E-01 | 1 | SLC38A6 | 1.47E-01 | 1 |
| CCDC152 | 1.27E-01 | 1 | RPL19 | 1.47E-01 | 1 |
| AQP3 | 1.27E-01 | 1 | ELF4 | 1.48E-01 | 1 |
| MMEL1 | 1.27E-01 | 1 | NDC80 | 1.48E-01 | 1 |
| LACTB2 | 1.28E-01 | 1 | MYO7B | 1.48E-01 | 1 |
| STAP2 | 1.28E-01 | 1 | BZRAP1 | 1.48E-01 | 1 |
| IFIT2 | 1.28E-01 | 1 | IL23A | 1.48E-01 | 1 |
| RFPL4A | 1.28E-01 | 1 | GORAB | 1.48E-01 | 1 |
| MRPL11 | 1.29E-01 | 1 | AP3M2 | 1.48E-01 | 1 |
| RPS27 | 1.29E-01 | 1 | BEND3 | 1.48E-01 | 1 |
| DPF1 | 1.29E-01 | 1 | HRH3 | 1.48E-01 | 1 |
| DCUN1D1 | 1.29E-01 | 1 | STK40 | 1.48E-01 | 1 |
| PTN | 1.29E-01 | 1 | CDK5RAP1 | 1.48E-01 | 1 |
| TMOD4 | 1.29E-01 | 1 | BCKDK | 1.48E-01 | 1 |
| KDELR2 | 1.29E-01 | 1 | TAS2R9 | 1.48E-01 | 1 |
| REG1A | 1.29E-01 | 1 | FAM127C | 1.48E-01 | 1 |

| DDB2 | 1.29E-01 | 1 | RPP40 | 1.48E-01 | 1 |
| --- | --- | --- | --- | --- | --- |
| BTLA | 1.29E-01 | 1 | AUP1 | 1.49E-01 | 1 |
| GH1 | 1.30E-01 | 1 | ARL9 | 1.49E-01 | 1 |
| PI4KB | 1.30E-01 | 1 | BUD13 | 1.49E-01 | 1 |
| STAR | 1.30E-01 | 1 | IGF2BP1 | 1.49E-01 | 1 |
| AADACL3 | 1.30E-01 | 1 | DYRK1A | 1.49E-01 | 1 |
| ANKH | 1.30E-01 | 1 | POLR3E | 1.49E-01 | 1 |
| PACSIN3 | 1.30E-01 | 1 | SHH | 1.49E-01 | 1 |
| OR5H14 | 1.31E-01 | 1 | PARP10 | 1.49E-01 | 1 |
| GPR160 | 1.31E-01 | 1 | HIST1H4B | 1.49E-01 | 1 |
| POLR3C | 1.31E-01 | 1 | MORN5 | 1.50E-01 | 1 |
| LDOC1 | 1.31E-01 | 1 | ARSK | 1.50E-01 | 1 |
| TBCC | 1.31E-01 | 1 | TFEB | 1.50E-01 | 1 |
| GPR25 | 1.31E-01 | 1 | RASA2 | 1.50E-01 | 1 |
| KCNJ8 | 1.31E-01 | 1 | RIPK1 | 1.50E-01 | 1 |
| TMEM61 | 1.31E-01 | 1 | IRX1 | 1.51E-01 | 1 |
| LDB1 | 1.32E-01 | 1 | KRT9 | 1.51E-01 | 1 |
| OR51V1 | 1.32E-01 | 1 | TMCO6 | 1.51E-01 | 1 |
| PKD2L2 | 1.32E-01 | 1 | C11orf49 | 1.51E-01 | 1 |
| RBKS | 1.32E-01 | 1 | MYOT | 1.51E-01 | 1 |
| HACL1 | 1.32E-01 | 1 | MEF2D | 1.51E-01 | 1 |
| TGFB1 | 1.32E-01 | 1 | HIST1H2AG | 1.51E-01 | 1 |
| RGS18 | 1.32E-01 | 1 | SLC2A6 | 1.51E-01 | 1 |
| CCKAR | 1.32E-01 | 1 | RCCD1 | 1.51E-01 | 1 |
| TADA2B | 1.32E-01 | 1 | PSMF1 | 1.51E-01 | 1 |
| PSMB11 | 1.32E-01 | 1 | OR2G2 | 1.51E-01 | 1 |
| NIPSNAP3 | 1.32E-01 | 1 | CT62 | 1.51E-01 | 1 |
| SLC39A8 | 1.32E-01 | 1 | NEFH | 1.52E-01 | 1 |
| TRAK2 | 1.33E-01 | 1 | IFNAR2 | 1.52E-01 | 1 |
| SEC22A | 1.33E-01 | 1 | MAP3K5 | 1.52E-01 | 1 |
| NOL7 | 1.33E-01 | 1 | TM6SF1 | 1.52E-01 | 1 |
| KCTD20 | 1.33E-01 | 1 | OR11H4 | 1.52E-01 | 1 |
| KIAA1191 | 1.33E-01 | 1 | LZTR1 | 1.52E-01 | 1 |
| DDI1 | 1.33E-01 | 1 | CD47 | 1.52E-01 | 1 |
| C16orf70 | 1.33E-01 | 1 | SYCP2 | 1.53E-01 | 1 |
| TMEFF2 | 1.33E-01 | 1 | MTMR4 | 1.53E-01 | 1 |
| TLX3 | 1.33E-01 | 1 | C18orf25 | 1.53E-01 | 1 |
| RAB23 | 1.34E-01 | 1 | GRIN3B | 1.53E-01 | 1 |
| SIGLEC15 | 1.34E-01 | 1 | RGS13 | 1.53E-01 | 1 |
| CA1 | 1.34E-01 | 1 | NME3 | 1.53E-01 | 1 |
| OR8B4 | 1.34E-01 | 1 | OR4Q3 | 1.53E-01 | 1 |
| OR52A5 | 1.34E-01 | 1 | LOXL1 | 1.53E-01 | 1 |
| APOA4 | 1.34E-01 | 1 | OR5D14 | 1.53E-01 | 1 |
| TMEM129 | 1.34E-01 | 1 | PRICKLE1 | 1.53E-01 | 1 |
| SNX11 | 1.34E-01 | 1 | MAT1A | 1.53E-01 | 1 |
| CD58 | 1.35E-01 | 1 | CCDC121 | 1.53E-01 | 1 |
| SUZ12 | 1.35E-01 | 1 | CDC25C | 1.53E-01 | 1 |
| PVRIG | 1.35E-01 | 1 | TPRX1 | 1.53E-01 | 1 |
| BRAP | 1.35E-01 | 1 | PARP11 | 1.54E-01 | 1 |
| VSTM2B | 1.35E-01 | 1 | TRAPPC3 | 1.54E-01 | 1 |
| TMEM39A | 1.35E-01 | 1 | PORCN | 1.54E-01 | 1 |
| GTF2B | 1.35E-01 | 1 | PPARD | 1.54E-01 | 1 |
| CATSPER | 1.35E-01 | 1 | CD5 | 1.54E-01 | 1 |

| DSG4 | 1.35E-01 | 1 | ZNF506 | 1.54E-01 | 1 |
| --- | --- | --- | --- | --- | --- |
| C2orf81 | 1.36E-01 | 1 | OR10G7 | 1.54E-01 | 1 |
| XKR4 | 1.36E-01 | 1 | XKR4 | 1.54E-01 | 1 |
| EBF1 | 1.36E-01 | 1 | FCRLB | 1.54E-01 | 1 |
| PPP2R5C | 1.36E-01 | 1 | ZC3H12D | 1.55E-01 | 1 |
| SMAD4 | 1.36E-01 | 1 | ZNF264 | 1.55E-01 | 1 |
| POTEH | 1.36E-01 | 1 | TTLL9 | 1.55E-01 | 1 |
| PRRT3 | 1.36E-01 | 1 | VN1R4 | 1.55E-01 | 1 |
| KRT76 | 1.37E-01 | 1 | FANK1 | 1.55E-01 | 1 |
| ACTC1 | 1.37E-01 | 1 | RNF6 | 1.55E-01 | 1 |
| CNOT2 | 1.37E-01 | 1 | TECR | 1.55E-01 | 1 |
| CCDC144 | 1.37E-01 | 1 | PLEKHF2 | 1.55E-01 | 1 |
| OR9Q2 | 1.37E-01 | 1 | LRFN2 | 1.56E-01 | 1 |
| OPN4 | 1.37E-01 | 1 | SHD | 1.56E-01 | 1 |
| TPM4 | 1.37E-01 | 1 | ENTHD1 | 1.56E-01 | 1 |
| FAM21C | 1.38E-01 | 1 | HCN4 | 1.56E-01 | 1 |
| DNAJB6 | 1.38E-01 | 1 | DYDC1 | 1.56E-01 | 1 |
| CNPY3 | 1.38E-01 | 1 | SH3GLB1 | 1.56E-01 | 1 |
| MRPS30 | 1.38E-01 | 1 | PDZD8 | 1.56E-01 | 1 |
| PRPH2 | 1.38E-01 | 1 | FBXL20 | 1.57E-01 | 1 |
| ICA1 | 1.38E-01 | 1 | PABPC4L | 1.57E-01 | 1 |
| EPT1 | 1.38E-01 | 1 | HCFC2 | 1.57E-01 | 1 |
| TTC1 | 1.38E-01 | 1 | TTC27 | 1.57E-01 | 1 |
| POR | 1.39E-01 | 1 | ZNF581 | 1.57E-01 | 1 |
| ZNF490 | 1.39E-01 | 1 | FLI1 | 1.57E-01 | 1 |
| TTYH1 | 1.39E-01 | 1 | SLC8A1 | 1.57E-01 | 1 |
| OR4X2 | 1.39E-01 | 1 | EPB41L4A | 1.58E-01 | 1 |
| FOXN2 | 1.39E-01 | 1 | DEFB129 | 1.58E-01 | 1 |
| LOR | 1.39E-01 | 1 | GSG1L | 1.58E-01 | 1 |
| OPRM1 | 1.39E-01 | 1 | NDUFA7 | 1.58E-01 | 1 |
| RFC2 | 1.39E-01 | 1 | RAB39B | 1.58E-01 | 1 |
| LCE1F | 1.40E-01 | 1 | MICALL1 | 1.58E-01 | 1 |
| OR5M9 | 1.40E-01 | 1 | ABHD4 | 1.59E-01 | 1 |
| SLC17A4 | 1.40E-01 | 1 | DCN | 1.59E-01 | 1 |
| PLA2G4F | 1.40E-01 | 1 | NECAB1 | 1.59E-01 | 1 |
| COL4A5 | 1.40E-01 | 1 | DDAH2 | 1.59E-01 | 1 |
| PPP2CB | 1.40E-01 | 1 | NOX1 | 1.59E-01 | 1 |
| FCRL1 | 1.40E-01 | 1 | ZDHHC22 | 1.59E-01 | 1 |
| ZNF772 | 1.40E-01 | 1 | HNRNPH1 | 1.59E-01 | 1 |
| HNRNPA3 | 1.40E-01 | 1 | KRT27 | 1.59E-01 | 1 |
| ZSCAN1 | 1.40E-01 | 1 | RAB6A | 1.60E-01 | 1 |
| PCIF1 | 1.40E-01 | 1 | C11orf24 | 1.60E-01 | 1 |
| OR6M1 | 1.41E-01 | 1 | ALDH4A1 | 1.60E-01 | 1 |
| SPERT | 1.41E-01 | 1 | BDH1 | 1.60E-01 | 1 |
| PCDH7 | 1.41E-01 | 1 | SLC4A1 | 1.60E-01 | 1 |
| C17orf50 | 1.41E-01 | 1 | CD55 | 1.60E-01 | 1 |
| CRLF1 | 1.41E-01 | 1 | ENDOG | 1.60E-01 | 1 |
| MTA2 | 1.41E-01 | 1 | PSKH1 | 1.60E-01 | 1 |
| SH2D5 | 1.41E-01 | 1 | CCDC43 | 1.60E-01 | 1 |
| TAT | 1.41E-01 | 1 | TMED10 | 1.60E-01 | 1 |
| PDE8A | 1.41E-01 | 1 | AUTS2 | 1.60E-01 | 1 |
| SHQ1 | 1.41E-01 | 1 | TNFRSF21 | 1.60E-01 | 1 |
| NRBP2 | 1.42E-01 | 1 | NOS3 | 1.60E-01 | 1 |

| CYSLTR2 | 1.42E-01 | 1 | VDAC1 | 1.60E-01 | 1 |
| --- | --- | --- | --- | --- | --- |
| OR4B1 | 1.42E-01 | 1 | PLEKHB2 | 1.60E-01 | 1 |
| FRS3 | 1.42E-01 | 1 | KRT82 | 1.61E-01 | 1 |
| KLHDC1 | 1.42E-01 | 1 | EVI5 | 1.61E-01 | 1 |
| GFRAL | 1.42E-01 | 1 | CERCAM | 1.61E-01 | 1 |
| WDR77 | 1.42E-01 | 1 | TAF15 | 1.61E-01 | 1 |
| ZNF581 | 1.42E-01 | 1 | NOTUM | 1.61E-01 | 1 |
| NPLOC4 | 1.43E-01 | 1 | HIST3H2A | 1.61E-01 | 1 |
| AGR2 | 1.43E-01 | 1 | OR8G5 | 1.61E-01 | 1 |
| IL18RAP | 1.43E-01 | 1 | ARMCX3 | 1.61E-01 | 1 |
| FOXE1 | 1.43E-01 | 1 | WSB1 | 1.62E-01 | 1 |
| TMEM110 | 1.43E-01 | 1 | GATAD2B | 1.62E-01 | 1 |
| POTEE | 1.43E-01 | 1 | OR5I1 | 1.62E-01 | 1 |
| CSHL1 | 1.43E-01 | 1 | BIN1 | 1.62E-01 | 1 |
| PDZD9 | 1.43E-01 | 1 | GPR173 | 1.62E-01 | 1 |
| OR4X1 | 1.43E-01 | 1 | IRAK4 | 1.62E-01 | 1 |
| C1QA | 1.43E-01 | 1 | CHDH | 1.62E-01 | 1 |
| BCL2 | 1.44E-01 | 1 | C22orf15 | 1.62E-01 | 1 |
| XBP1 | 1.44E-01 | 1 | COL8A2 | 1.62E-01 | 1 |
| DHX35 | 1.44E-01 | 1 | FAM134B | 1.62E-01 | 1 |
| BCL3 | 1.44E-01 | 1 | MATK | 1.62E-01 | 1 |
| RNF214 | 1.44E-01 | 1 | SNX15 | 1.62E-01 | 1 |
| HNRNPM | 1.44E-01 | 1 | PPP1R3B | 1.62E-01 | 1 |
| ELANE | 1.44E-01 | 1 | PNPLA3 | 1.62E-01 | 1 |
| CCDC3 | 1.44E-01 | 1 | TRIM24 | 1.62E-01 | 1 |
| MOCS2 | 1.44E-01 | 1 | STRADA | 1.62E-01 | 1 |
| IRAK1BP1 | 1.44E-01 | 1 | IGFL2 | 1.63E-01 | 1 |
| PLEKHG1 | 1.45E-01 | 1 | KRT28 | 1.63E-01 | 1 |
| PCSK9 | 1.45E-01 | 1 | CBWD5 | 1.63E-01 | 1 |
| VAV3 | 1.45E-01 | 1 | BAX | 1.63E-01 | 1 |
| CRHR1 | 1.45E-01 | 1 | MLXIPL | 1.63E-01 | 1 |
| EPHA2 | 1.45E-01 | 1 | CXCR3 | 1.63E-01 | 1 |
| CLEC17A | 1.45E-01 | 1 | ZBTB7C | 1.63E-01 | 1 |
| OPLAH | 1.45E-01 | 1 | NDUFA8 | 1.63E-01 | 1 |
| MAP3K15 | 1.45E-01 | 1 | ACBD5 | 1.63E-01 | 1 |
| HAPLN1 | 1.45E-01 | 1 | RFK | 1.64E-01 | 1 |
| MTMR11 | 1.45E-01 | 1 | FAM162A | 1.64E-01 | 1 |
| ANXA9 | 1.46E-01 | 1 | TMX2 | 1.64E-01 | 1 |
| CAPG | 1.46E-01 | 1 | TLR6 | 1.64E-01 | 1 |
| PANX3 | 1.46E-01 | 1 | SLC2A14 | 1.64E-01 | 1 |
| RBM5 | 1.46E-01 | 1 | AGFG1 | 1.64E-01 | 1 |
| RAVER2 | 1.46E-01 | 1 | ADORA2A | 1.64E-01 | 1 |
| ZDHHC9 | 1.46E-01 | 1 | CPNE3 | 1.64E-01 | 1 |
| KIAA0020 | 1.46E-01 | 1 | CCDC6 | 1.64E-01 | 1 |
| RAC2 | 1.47E-01 | 1 | CLEC18C | 1.65E-01 | 1 |
| PLIN2 | 1.47E-01 | 1 | SPEN | 1.65E-01 | 1 |
| BRCC3 | 1.47E-01 | 1 | CYBRD1 | 1.65E-01 | 1 |
| C7orf72 | 1.47E-01 | 1 | TMOD3 | 1.65E-01 | 1 |
| TLE1 | 1.47E-01 | 1 | LY86 | 1.65E-01 | 1 |
| DEGS1 | 1.47E-01 | 1 | PER2 | 1.65E-01 | 1 |
| TRIB1 | 1.47E-01 | 1 | ST6GALNA | 1.65E-01 | 1 |
| ECE1 | 1.47E-01 | 1 | EED | 1.65E-01 | 1 |
| C20orf96 | 1.47E-01 | 1 | NCR1 | 1.65E-01 | 1 |

| DEFB128 | 1.48E-01 | 1 | FAM179A | 1.66E-01 | 1 |
| --- | --- | --- | --- | --- | --- |
| DEPDC5 | 1.48E-01 | 1 | REC8 | 1.66E-01 | 1 |
| CPSF4 | 1.48E-01 | 1 | EVX2 | 1.66E-01 | 1 |
| TMEM71 | 1.48E-01 | 1 | PCSK7 | 1.66E-01 | 1 |
| PYCARD | 1.48E-01 | 1 | RAB19 | 1.66E-01 | 1 |
| TGFB1I1 | 1.48E-01 | 1 | ZNF681 | 1.66E-01 | 1 |
| RHO | 1.49E-01 | 1 | STOML3 | 1.67E-01 | 1 |
| MAN2B2 | 1.49E-01 | 1 | MRPL48 | 1.67E-01 | 1 |
| IQCC | 1.49E-01 | 1 | RAB9B | 1.67E-01 | 1 |
| HMG20A | 1.49E-01 | 1 | SMCP | 1.67E-01 | 1 |
| SYVN1 | 1.49E-01 | 1 | PNN | 1.67E-01 | 1 |
| ARPC3 | 1.49E-01 | 1 | AHSA2 | 1.67E-01 | 1 |
| NOX1 | 1.49E-01 | 1 | HIST1H2BL | 1.67E-01 | 1 |
| FOXD4L3 | 1.49E-01 | 1 | LRTOMT | 1.67E-01 | 1 |
| CMTM7 | 1.49E-01 | 1 | NCLN | 1.67E-01 | 1 |
| ATL3 | 1.49E-01 | 1 | PARVA | 1.67E-01 | 1 |
| BCO2 | 1.49E-01 | 1 | MAGEE2 | 1.67E-01 | 1 |
| EBNA1BP | 1.49E-01 | 1 | NEK11 | 1.68E-01 | 1 |
| LRRC10 | 1.50E-01 | 1 | TNFRSF18 | 1.68E-01 | 1 |
| KLF13 | 1.50E-01 | 1 | INSL4 | 1.68E-01 | 1 |
| ABHD11 | 1.50E-01 | 1 | HPX | 1.68E-01 | 1 |
| AFTPH | 1.50E-01 | 1 | SLC10A1 | 1.68E-01 | 1 |
| UBA6 | 1.50E-01 | 1 | GATA6 | 1.68E-01 | 1 |
| DCUN1D4 | 1.50E-01 | 1 | BCL11B | 1.68E-01 | 1 |
| PHPT1 | 1.50E-01 | 1 | PPP2R1B | 1.68E-01 | 1 |
| SHFM1 | 1.50E-01 | 1 | SLC7A13 | 1.68E-01 | 1 |
| LMBR1L | 1.50E-01 | 1 | PIP4K2A | 1.68E-01 | 1 |
| FARP1 | 1.51E-01 | 1 | ADAM17 | 1.68E-01 | 1 |
| SLAIN1 | 1.51E-01 | 1 | APOA1 | 1.69E-01 | 1 |
| ALK | 1.51E-01 | 1 | NEUROD4 | 1.69E-01 | 1 |
| BFSP2 | 1.51E-01 | 1 | C1QA | 1.69E-01 | 1 |
| C21orf91 | 1.51E-01 | 1 | CLDN4 | 1.69E-01 | 1 |
| FZD4 | 1.51E-01 | 1 | MRPS18C | 1.69E-01 | 1 |
| SNX12 | 1.51E-01 | 1 | KCNK9 | 1.69E-01 | 1 |
| CTF1 | 1.51E-01 | 1 | LYPLA1 | 1.69E-01 | 1 |
| RDH8 | 1.52E-01 | 1 | IQCG | 1.69E-01 | 1 |
| PPARGC1 | 1.52E-01 | 1 | SPINK9 | 1.69E-01 | 1 |
| ATAD1 | 1.52E-01 | 1 | KLHL7 | 1.70E-01 | 1 |
| PRELP | 1.52E-01 | 1 | FAM188A | 1.70E-01 | 1 |
| METTL7A | 1.52E-01 | 1 | RASGRP4 | 1.70E-01 | 1 |
| DMRTB1 | 1.52E-01 | 1 | VEGFC | 1.70E-01 | 1 |
| GAST | 1.52E-01 | 1 | SGSM3 | 1.70E-01 | 1 |
| FGD4 | 1.52E-01 | 1 | NUP62 | 1.70E-01 | 1 |
| MTSS1 | 1.52E-01 | 1 | SPATS2L | 1.70E-01 | 1 |
| CYB561D | 1.52E-01 | 1 | MPHOSPH9 | 1.71E-01 | 1 |
| PTDSS1 | 1.52E-01 | 1 | ZNF536 | 1.71E-01 | 1 |
| SFTA3 | 1.52E-01 | 1 | ZNF607 | 1.71E-01 | 1 |
| BICD1 | 1.53E-01 | 1 | CDKN2AIP | 1.71E-01 | 1 |
| MEFV | 1.53E-01 | 1 | RNF126 | 1.71E-01 | 1 |
| DEFA4 | 1.53E-01 | 1 | MSGN1 | 1.71E-01 | 1 |
| ARG2 | 1.53E-01 | 1 | HIPK2 | 1.71E-01 | 1 |
| HOXD4 | 1.53E-01 | 1 | JAM3 | 1.71E-01 | 1 |
| NDUFA2 | 1.53E-01 | 1 | ST7 | 1.71E-01 | 1 |

| PXT1 | 1.53E-01 | 1 | APOBEC3B | 1.71E-01 | 1 |
| --- | --- | --- | --- | --- | --- |
| ZNF527 | 1.53E-01 | 1 | FCER2 | 1.71E-01 | 1 |
| NR5A1 | 1.53E-01 | 1 | KRT81 | 1.72E-01 | 1 |
| C21orf59 | 1.53E-01 | 1 | MTMR7 | 1.72E-01 | 1 |
| PTPRCAP | 1.54E-01 | 1 | RNF123 | 1.72E-01 | 1 |
| FCN1 | 1.54E-01 | 1 | TRIM26 | 1.72E-01 | 1 |
| GMPPA | 1.54E-01 | 1 | CARD16 | 1.72E-01 | 1 |
| SFTPB | 1.54E-01 | 1 | PTPN12 | 1.72E-01 | 1 |
| FRG2C | 1.54E-01 | 1 | INHA | 1.72E-01 | 1 |
| PYGM | 1.54E-01 | 1 | STAT4 | 1.72E-01 | 1 |
| FOSB | 1.54E-01 | 1 | LCE1E | 1.72E-01 | 1 |
| CAMK2A | 1.54E-01 | 1 | HIST1H1E | 1.72E-01 | 1 |
| ZNF511 | 1.54E-01 | 1 | CRYBA1 | 1.73E-01 | 1 |
| IFT81 | 1.54E-01 | 1 | GABRB1 | 1.73E-01 | 1 |
| GAPVD1 | 1.54E-01 | 1 | SFT2D2 | 1.73E-01 | 1 |
| CRISP1 | 1.54E-01 | 1 | SCRIB | 1.73E-01 | 1 |
| NMUR2 | 1.55E-01 | 1 | TMEM223 | 1.73E-01 | 1 |
| CA11 | 1.55E-01 | 1 | PDE11A | 1.73E-01 | 1 |
| PRSS48 | 1.55E-01 | 1 | TSPAN9 | 1.73E-01 | 1 |
| TTLL1 | 1.55E-01 | 1 | SPI1 | 1.74E-01 | 1 |
| OR12D3 | 1.55E-01 | 1 | NCR3 | 1.74E-01 | 1 |
| CCDC13 | 1.55E-01 | 1 | ASL | 1.74E-01 | 1 |
| IL5 | 1.55E-01 | 1 | GOLT1B | 1.74E-01 | 1 |
| TCF25 | 1.55E-01 | 1 | IDE | 1.74E-01 | 1 |
| FZD9 | 1.55E-01 | 1 | IFNGR1 | 1.74E-01 | 1 |
| SHBG | 1.56E-01 | 1 | OXCT1 | 1.74E-01 | 1 |
| COL5A1 | 1.56E-01 | 1 | GGN | 1.74E-01 | 1 |
| ACSM2A | 1.56E-01 | 1 | ASPRV1 | 1.74E-01 | 1 |
| PAX8 | 1.56E-01 | 1 | DNAJB14 | 1.74E-01 | 1 |
| TRMU | 1.56E-01 | 1 | TRIM58 | 1.74E-01 | 1 |
| WWC2 | 1.56E-01 | 1 | GLRB | 1.74E-01 | 1 |
| SLC2A10 | 1.56E-01 | 1 | RPS2 | 1.74E-01 | 1 |
| CGB1 | 1.56E-01 | 1 | RETNLB | 1.74E-01 | 1 |
| RPRD1A | 1.56E-01 | 1 | LRIT1 | 1.75E-01 | 1 |
| COX6B1 | 1.56E-01 | 1 | MOCS1 | 1.75E-01 | 1 |
| ZFP42 | 1.56E-01 | 1 | PRKCE | 1.75E-01 | 1 |
| PRKRIR | 1.56E-01 | 1 | PRKACB | 1.75E-01 | 1 |
| PEAR1 | 1.57E-01 | 1 | FAM195A | 1.75E-01 | 1 |
| BEND4 | 1.57E-01 | 1 | AATK | 1.75E-01 | 1 |
| TRIM15 | 1.57E-01 | 1 | OR10X1 | 1.75E-01 | 1 |
| CD93 | 1.57E-01 | 1 | OLFML2B | 1.75E-01 | 1 |
| C9orf172 | 1.57E-01 | 1 | LILRA6 | 1.75E-01 | 1 |
| VAMP3 | 1.57E-01 | 1 | CNPY1 | 1.75E-01 | 1 |
| SPP2 | 1.57E-01 | 1 | RALBP1 | 1.76E-01 | 1 |
| CDK15 | 1.57E-01 | 1 | AADAC | 1.76E-01 | 1 |
| FCRL5 | 1.57E-01 | 1 | COPS7A | 1.76E-01 | 1 |
| FCHO2 | 1.57E-01 | 1 | FGFBP1 | 1.76E-01 | 1 |
| SIRPB1 | 1.57E-01 | 1 | GNAI1 | 1.76E-01 | 1 |
| FOXF1 | 1.58E-01 | 1 | ASPSCR1 | 1.76E-01 | 1 |
| MAGEB1 | 1.58E-01 | 1 | C8orf74 | 1.77E-01 | 1 |
| MRPL2 | 1.58E-01 | 1 | IGSF8 | 1.77E-01 | 1 |
| OR5M11 | 1.58E-01 | 1 | LRRC4B | 1.77E-01 | 1 |
| NEU2 | 1.58E-01 | 1 | RABGGTA | 1.77E-01 | 1 |

| RNF212 | 1.58E-01 | 1 | LCE1F | 1.77E-01 | 1 |
| --- | --- | --- | --- | --- | --- |
| ZNF324 | 1.58E-01 | 1 | NES | 1.77E-01 | 1 |
| WDR74 | 1.58E-01 | 1 | ZNF366 | 1.77E-01 | 1 |
| CLCN2 | 1.59E-01 | 1 | DRAP1 | 1.77E-01 | 1 |
| PATE3 | 1.59E-01 | 1 | SPATS2 | 1.77E-01 | 1 |
| MYBPC3 | 1.59E-01 | 1 | LHFPL5 | 1.78E-01 | 1 |
| IGFBP3 | 1.59E-01 | 1 | S100A7 | 1.78E-01 | 1 |
| TSC22D4 | 1.59E-01 | 1 | ARMC7 | 1.78E-01 | 1 |
| GJA5 | 1.59E-01 | 1 | OR6Y1 | 1.78E-01 | 1 |
| GNPTG | 1.59E-01 | 1 | GALK1 | 1.78E-01 | 1 |
| TBC1D10 | 1.59E-01 | 1 | PLD3 | 1.78E-01 | 1 |
| ASS1 | 1.59E-01 | 1 | CILP2 | 1.78E-01 | 1 |
| PYDC1 | 1.59E-01 | 1 | ZWINT | 1.79E-01 | 1 |
| TIMD4 | 1.59E-01 | 1 | HP | 1.79E-01 | 1 |
| HIST1H4I | 1.59E-01 | 1 | GPR89A | 1.79E-01 | 1 |
| PRSS1 | 1.59E-01 | 1 | ATP6V1F | 1.79E-01 | 1 |
| SLC24A4 | 1.59E-01 | 1 | BCDIN3D | 1.79E-01 | 1 |
| CHKB | 1.60E-01 | 1 | GLYAT | 1.79E-01 | 1 |
| UBR2 | 1.60E-01 | 1 | PHF14 | 1.79E-01 | 1 |
| NTN4 | 1.60E-01 | 1 | NR6A1 | 1.79E-01 | 1 |
| CSDE1 | 1.60E-01 | 1 | CTGF | 1.79E-01 | 1 |
| THAP10 | 1.60E-01 | 1 | MAP2K7 | 1.79E-01 | 1 |
| MAN2C1 | 1.60E-01 | 1 | ZBTB32 | 1.79E-01 | 1 |
| JPH1 | 1.60E-01 | 1 | TRIM39 | 1.79E-01 | 1 |
| CKM | 1.61E-01 | 1 | SLCO6A1 | 1.80E-01 | 1 |
| FAM78B | 1.61E-01 | 1 | SAMHD1 | 1.80E-01 | 1 |
| VARS | 1.61E-01 | 1 | CCR7 | 1.80E-01 | 1 |
| IRAK1 | 1.61E-01 | 1 | DMRT3 | 1.80E-01 | 1 |
| LPIN2 | 1.61E-01 | 1 | GLUD2 | 1.80E-01 | 1 |
| RPS6KA5 | 1.61E-01 | 1 | CCDC7 | 1.80E-01 | 1 |
| ISYNA1 | 1.61E-01 | 1 | HIST1H1C | 1.80E-01 | 1 |
| MAPK9 | 1.61E-01 | 1 | MBNL1 | 1.80E-01 | 1 |
| SUFU | 1.61E-01 | 1 | MBD1 | 1.80E-01 | 1 |
| TBX4 | 1.61E-01 | 1 | NUP93 | 1.80E-01 | 1 |
| PCDHB10 | 1.62E-01 | 1 | TXN2 | 1.80E-01 | 1 |
| APBB1 | 1.62E-01 | 1 | TRAM1L1 | 1.81E-01 | 1 |
| FAM47E | 1.62E-01 | 1 | CLC | 1.81E-01 | 1 |
| DLX5 | 1.62E-01 | 1 | C22orf29 | 1.81E-01 | 1 |
| MRPL51 | 1.62E-01 | 1 | IER2 | 1.81E-01 | 1 |
| MATN4 | 1.62E-01 | 1 | SGCA | 1.81E-01 | 1 |
| ZNF835 | 1.62E-01 | 1 | GGNBP2 | 1.82E-01 | 1 |
| KRTAP5-1 | 1.62E-01 | 1 | MED18 | 1.82E-01 | 1 |
| CGB8 | 1.63E-01 | 1 | MUSK | 1.82E-01 | 1 |
| CLDN12 | 1.63E-01 | 1 | KNTC1 | 1.82E-01 | 1 |
| GIGYF1 | 1.63E-01 | 1 | CCNA2 | 1.82E-01 | 1 |
| A1BG | 1.63E-01 | 1 | CETN2 | 1.82E-01 | 1 |
| ARHGAP3 | 1.63E-01 | 1 | WIF1 | 1.82E-01 | 1 |
| SLCO3A1 | 1.63E-01 | 1 | CTTNBP2N | 1.82E-01 | 1 |
| LTBR | 1.63E-01 | 1 | CXXC5 | 1.82E-01 | 1 |
| GZMK | 1.63E-01 | 1 | SMAD4 | 1.82E-01 | 1 |
| CPA3 | 1.63E-01 | 1 | FBXO36 | 1.82E-01 | 1 |
| CDC25B | 1.63E-01 | 1 | MCM7 | 1.83E-01 | 1 |
| EIF5 | 1.63E-01 | 1 | RHOH | 1.83E-01 | 1 |

| ING5 | 1.63E-01 | 1 | GRIA1 | 1.83E-01 | 1 |
| --- | --- | --- | --- | --- | --- |
| IL1F10 | 1.64E-01 | 1 | TOR3A | 1.83E-01 | 1 |
| C8orf22 | 1.64E-01 | 1 | CYP4B1 | 1.83E-01 | 1 |
| MPI | 1.64E-01 | 1 | LILRB5 | 1.83E-01 | 1 |
| LNPEP | 1.64E-01 | 1 | PHB2 | 1.84E-01 | 1 |
| UBR1 | 1.64E-01 | 1 | OR2L3 | 1.84E-01 | 1 |
| DIP2A | 1.64E-01 | 1 | COMP | 1.84E-01 | 1 |
| CD300LF | 1.64E-01 | 1 | RLN2 | 1.84E-01 | 1 |
| ANKRD44 | 1.64E-01 | 1 | AMPH | 1.84E-01 | 1 |
| GABRR1 | 1.65E-01 | 1 | MAVS | 1.84E-01 | 1 |
| AMTN | 1.65E-01 | 1 | DCC | 1.84E-01 | 1 |
| OR11H1 | 1.65E-01 | 1 | RPL11 | 1.84E-01 | 1 |
| CMPK2 | 1.65E-01 | 1 | RPL23A | 1.84E-01 | 1 |
| LHX2 | 1.65E-01 | 1 | PLCG1 | 1.84E-01 | 1 |
| IDH1 | 1.65E-01 | 1 | ARFRP1 | 1.84E-01 | 1 |
| ZNF735 | 1.65E-01 | 1 | COL5A1 | 1.84E-01 | 1 |
| GRB7 | 1.65E-01 | 1 | MS4A12 | 1.84E-01 | 1 |
| SLC35E3 | 1.65E-01 | 1 | DIP2C | 1.84E-01 | 1 |
| ADAT1 | 1.65E-01 | 1 | PSPN | 1.84E-01 | 1 |
| CLCNKA | 1.65E-01 | 1 | FHL3 | 1.85E-01 | 1 |
| RELA | 1.65E-01 | 1 | RAPGEF2 | 1.85E-01 | 1 |
| OR4D2 | 1.65E-01 | 1 | ADAM18 | 1.85E-01 | 1 |
| BTBD18 | 1.65E-01 | 1 | FAM118A | 1.85E-01 | 1 |
| CCDC102 | 1.65E-01 | 1 | PPEF2 | 1.85E-01 | 1 |
| SLC25A5 | 1.66E-01 | 1 | SLC2A11 | 1.85E-01 | 1 |
| CNTN3 | 1.66E-01 | 1 | SLC1A3 | 1.86E-01 | 1 |
| FFAR2 | 1.66E-01 | 1 | OSBPL1A | 1.86E-01 | 1 |
| PIK3R2 | 1.66E-01 | 1 | MAST4 | 1.86E-01 | 1 |
| GRIK3 | 1.66E-01 | 1 | ADH1B | 1.86E-01 | 1 |
| PTF1A | 1.66E-01 | 1 | MRPS26 | 1.86E-01 | 1 |
| TOMM5 | 1.66E-01 | 1 | PLEK2 | 1.86E-01 | 1 |
| ZNF679 | 1.66E-01 | 1 | TMPRSS11 | 1.86E-01 | 1 |
| OR2F1 | 1.66E-01 | 1 | IDUA | 1.86E-01 | 1 |
| FAH | 1.66E-01 | 1 | CEACAM1 | 1.87E-01 | 1 |
| STRAP | 1.66E-01 | 1 | STMN3 | 1.87E-01 | 1 |
| D4S234E | 1.66E-01 | 1 | CCDC129 | 1.87E-01 | 1 |
| HBD | 1.66E-01 | 1 | FAM160B2 | 1.87E-01 | 1 |
| IFNA7 | 1.66E-01 | 1 | MMP17 | 1.87E-01 | 1 |
| MLH3 | 1.66E-01 | 1 | UCKL1 | 1.87E-01 | 1 |
| PTPN14 | 1.66E-01 | 1 | SYT4 | 1.87E-01 | 1 |
| HIST2H2B | 1.67E-01 | 1 | TRNT1 | 1.88E-01 | 1 |
| TXLNB | 1.67E-01 | 1 | ZBTB20 | 1.88E-01 | 1 |
| PDSS2 | 1.67E-01 | 1 | MAF1 | 1.88E-01 | 1 |
| OR10G8 | 1.67E-01 | 1 | SAMSN1 | 1.88E-01 | 1 |
| NBEA | 1.67E-01 | 1 | PPP2CA | 1.88E-01 | 1 |
| SULT1C4 | 1.67E-01 | 1 | MICALL2 | 1.88E-01 | 1 |
| SUCNR1 | 1.67E-01 | 1 | FERMT1 | 1.88E-01 | 1 |
| CXCL5 | 1.67E-01 | 1 | ALPI | 1.89E-01 | 1 |
| IL13RA1 | 1.67E-01 | 1 | PODXL | 1.89E-01 | 1 |
| ZNF280C | 1.67E-01 | 1 | MFSD11 | 1.89E-01 | 1 |
| CCDC94 | 1.67E-01 | 1 | CCR6 | 1.89E-01 | 1 |
| TERF2IP | 1.67E-01 | 1 | TTC21A | 1.89E-01 | 1 |
| SPATA5L | 1.67E-01 | 1 | ABHD3 | 1.90E-01 | 1 |

| ECSCR | 1.67E-01 | 1 | HMGN5 | 1.90E-01 | 1 |
| --- | --- | --- | --- | --- | --- |
| NKX3-1 | 1.67E-01 | 1 | GREM1 | 1.90E-01 | 1 |
| NHP2 | 1.67E-01 | 1 | SCT | 1.90E-01 | 1 |
| KRTAP1-5 | 1.67E-01 | 1 | MEP1A | 1.90E-01 | 1 |
| C8orf4 | 1.67E-01 | 1 | RAB30 | 1.90E-01 | 1 |
| AQP11 | 1.68E-01 | 1 | ATP13A5 | 1.90E-01 | 1 |
| CCDC141 | 1.68E-01 | 1 | DDX47 | 1.90E-01 | 1 |
| GATA2 | 1.68E-01 | 1 | IMPAD1 | 1.90E-01 | 1 |
| CDC45 | 1.68E-01 | 1 | SYT10 | 1.90E-01 | 1 |
| NKX2-8 | 1.68E-01 | 1 | SLC24A2 | 1.90E-01 | 1 |
| OR10K2 | 1.68E-01 | 1 | PGGT1B | 1.90E-01 | 1 |
| RNASE7 | 1.68E-01 | 1 | FOXH1 | 1.90E-01 | 1 |
| ZAR1 | 1.68E-01 | 1 | PSMB6 | 1.90E-01 | 1 |
| STX8 | 1.68E-01 | 1 | VCX | 1.90E-01 | 1 |
| NPFF | 1.68E-01 | 1 | LMO4 | 1.91E-01 | 1 |
| APBB3 | 1.68E-01 | 1 | PES1 | 1.91E-01 | 1 |
| KRTAP11 | 1.68E-01 | 1 | DGCR14 | 1.91E-01 | 1 |
| HBE1 | 1.68E-01 | 1 | TTC23 | 1.91E-01 | 1 |
| ARAP3 | 1.68E-01 | 1 | CYP17A1 | 1.91E-01 | 1 |
| C11orf58 | 1.68E-01 | 1 | KIAA0319 | 1.92E-01 | 1 |
| IYD | 1.69E-01 | 1 | PYHIN1 | 1.92E-01 | 1 |
| SLC36A3 | 1.69E-01 | 1 | POTEF | 1.92E-01 | 1 |
| IRF9 | 1.69E-01 | 1 | CD164L2 | 1.92E-01 | 1 |
| APOC3 | 1.69E-01 | 1 | CDK8 | 1.92E-01 | 1 |
| RPL27 | 1.69E-01 | 1 | ZNF574 | 1.92E-01 | 1 |
| BSG | 1.69E-01 | 1 | SNX4 | 1.92E-01 | 1 |
| HBG2 | 1.69E-01 | 1 | CYB561 | 1.92E-01 | 1 |
| CCDC70 | 1.69E-01 | 1 | CRISPLD1 | 1.92E-01 | 1 |
| SMYD3 | 1.69E-01 | 1 | TBX20 | 1.92E-01 | 1 |
| ADAL | 1.69E-01 | 1 | SGCZ | 1.93E-01 | 1 |
| TULP2 | 1.69E-01 | 1 | SPPL2B | 1.93E-01 | 1 |
| METTL2B | 1.69E-01 | 1 | ZNF69 | 1.93E-01 | 1 |
| KIAA1429 | 1.69E-01 | 1 | OR13C3 | 1.93E-01 | 1 |
| ZBTB32 | 1.69E-01 | 1 | CISD3 | 1.93E-01 | 1 |
| OR9G1 | 1.69E-01 | 1 | PKD2L2 | 1.93E-01 | 1 |
| KIF2A | 1.70E-01 | 1 | AZU1 | 1.93E-01 | 1 |
| SLC44A3 | 1.70E-01 | 1 | TOP2A | 1.93E-01 | 1 |
| ADRBK1 | 1.70E-01 | 1 | KRTAP19-4 | 1.93E-01 | 1 |
| TGOLN2 | 1.70E-01 | 1 | TBX18 | 1.93E-01 | 1 |
| NANS | 1.70E-01 | 1 | AMOTL2 | 1.94E-01 | 1 |
| JAGN1 | 1.70E-01 | 1 | TBCC | 1.94E-01 | 1 |
| GRK1 | 1.70E-01 | 1 | P2RY10 | 1.94E-01 | 1 |
| EMCN | 1.70E-01 | 1 | RBM28 | 1.94E-01 | 1 |
| EPHB2 | 1.70E-01 | 1 | CHST15 | 1.94E-01 | 1 |
| GIMAP4 | 1.70E-01 | 1 | RANBP17 | 1.94E-01 | 1 |
| PRSS21 | 1.70E-01 | 1 | BTBD7 | 1.94E-01 | 1 |
| INS | 1.70E-01 | 1 | LRIG2 | 1.94E-01 | 1 |
| AKR1B1 | 1.70E-01 | 1 | TESK2 | 1.94E-01 | 1 |
| SLC26A10 | 1.70E-01 | 1 | CD302 | 1.94E-01 | 1 |
| SPINT1 | 1.70E-01 | 1 | THAP11 | 1.94E-01 | 1 |
| SALL4 | 1.70E-01 | 1 | SLC38A11 | 1.95E-01 | 1 |
| AMFR | 1.71E-01 | 1 | CADM3 | 1.95E-01 | 1 |
| C11orf57 | 1.71E-01 | 1 | RPRD1B | 1.95E-01 | 1 |

| MUC20 | 1.71E-01 | 1 | CRABP2 | 1.95E-01 | 1 |
| --- | --- | --- | --- | --- | --- |
| CHST2 | 1.71E-01 | 1 | MAN1A1 | 1.95E-01 | 1 |
| RHCG | 1.71E-01 | 1 | EDDM3A | 1.95E-01 | 1 |
| CLCN1 | 1.71E-01 | 1 | TMEM106A | 1.95E-01 | 1 |
| TPPP3 | 1.71E-01 | 1 | SLC39A12 | 1.95E-01 | 1 |
| CXorf40A | 1.71E-01 | 1 | SPCS3 | 1.95E-01 | 1 |
| KATNB1 | 1.71E-01 | 1 | NMU | 1.95E-01 | 1 |
| PDE9A | 1.71E-01 | 1 | GAS7 | 1.95E-01 | 1 |
| MAPKAP | 1.71E-01 | 1 | SLC6A6 | 1.95E-01 | 1 |
| ODF4 | 1.71E-01 | 1 | GPR148 | 1.96E-01 | 1 |
| LIPF | 1.71E-01 | 1 | ARNT | 1.96E-01 | 1 |
| JPH4 | 1.72E-01 | 1 | ANO4 | 1.96E-01 | 1 |
| TRIM29 | 1.72E-01 | 1 | TUBA3C | 1.96E-01 | 1 |
| CCNO | 1.72E-01 | 1 | KIAA0907 | 1.96E-01 | 1 |
| PSKH1 | 1.72E-01 | 1 | HDHD3 | 1.96E-01 | 1 |
| PDE11A | 1.72E-01 | 1 | COMMD8 | 1.96E-01 | 1 |
| MLXIPL | 1.72E-01 | 1 | PHF11 | 1.96E-01 | 1 |
| IL12B | 1.72E-01 | 1 | LCN15 | 1.96E-01 | 1 |
| NTS | 1.72E-01 | 1 | MYCT1 | 1.96E-01 | 1 |
| COL7A1 | 1.72E-01 | 1 | B4GALNT4 | 1.96E-01 | 1 |
| GZMH | 1.72E-01 | 1 | C20orf196 | 1.96E-01 | 1 |
| HSD17B7 | 1.72E-01 | 1 | P2RY1 | 1.96E-01 | 1 |
| FCGR3B | 1.72E-01 | 1 | DDX60L | 1.96E-01 | 1 |
| KCNH6 | 1.73E-01 | 1 | TMBIM1 | 1.96E-01 | 1 |
| ACOT13 | 1.73E-01 | 1 | PHACTR3 | 1.97E-01 | 1 |
| BLVRA | 1.73E-01 | 1 | TMEM40 | 1.97E-01 | 1 |
| MAPKAP | 1.73E-01 | 1 | ACVR1B | 1.97E-01 | 1 |
| TSPYL4 | 1.73E-01 | 1 | TMEM8C | 1.97E-01 | 1 |
| LGI2 | 1.73E-01 | 1 | PIGV | 1.97E-01 | 1 |
| LAPTM5 | 1.73E-01 | 1 | TCEB3 | 1.97E-01 | 1 |
| ZNF195 | 1.73E-01 | 1 | PAK7 | 1.98E-01 | 1 |
| MXD3 | 1.73E-01 | 1 | MAP3K4 | 1.98E-01 | 1 |
| HIPK3 | 1.73E-01 | 1 | ECT2 | 1.98E-01 | 1 |
| C11orf16 | 1.73E-01 | 1 | KCNE4 | 1.98E-01 | 1 |
| TEX11 | 1.74E-01 | 1 | MYO1F | 1.98E-01 | 1 |
| H2AFZ | 1.74E-01 | 1 | PRICKLE2 | 1.98E-01 | 1 |
| CDC42BP | 1.74E-01 | 1 | MARCO | 1.99E-01 | 1 |
| TMEM233 | 1.74E-01 | 1 | CENPO | 1.99E-01 | 1 |
| SLC26A4 | 1.74E-01 | 1 | FAM177B | 1.99E-01 | 1 |
| DIAPH1 | 1.74E-01 | 1 | PPP3CA | 1.99E-01 | 1 |
| PELO | 1.74E-01 | 1 | PDXDC1 | 1.99E-01 | 1 |
| GPR1 | 1.74E-01 | 1 | ZBTB10 | 1.99E-01 | 1 |
| CAPNS1 | 1.74E-01 | 1 | TSTD1 | 1.99E-01 | 1 |
| HDAC4 | 1.74E-01 | 1 | IL1RAP | 1.99E-01 | 1 |
| RUVBL2 | 1.74E-01 | 1 | GABRR3 | 1.99E-01 | 1 |
| MGST2 | 1.74E-01 | 1 | ATP12A | 1.99E-01 | 1 |
| TNFRSF25 | 1.74E-01 | 1 | FOXL1 | 2.00E-01 | 1 |
| C9orf9 | 1.75E-01 | 1 | OSBPL11 | 2.00E-01 | 1 |
| PSMD11 | 1.75E-01 | 1 | ARHGEF3 | 2.00E-01 | 1 |
| DACH1 | 1.75E-01 | 1 | ZNF630 | 2.00E-01 | 1 |
| MRPL46 | 1.75E-01 | 1 | BMP3 | 2.00E-01 | 1 |
| SEL1L3 | 1.75E-01 | 1 | FAT2 | 2.00E-01 | 1 |
| CSNK2A1 | 1.75E-01 | 1 | NOLC1 | 2.00E-01 | 1 |

| NME2 | 1.75E-01 | 1 | SLC35B2 | 2.00E-01 | 1 |
| --- | --- | --- | --- | --- | --- |
| CPA4 | 1.75E-01 | 1 | CHIC1 | 2.00E-01 | 1 |
| NOS1 | 1.75E-01 | 1 | ZNF85 | 2.01E-01 | 1 |
| C2orf68 | 1.75E-01 | 1 | RP1 | 2.01E-01 | 1 |
| AKR1C2 | 1.75E-01 | 1 | TIMD4 | 2.01E-01 | 1 |
| H2AFJ | 1.75E-01 | 1 | GPX1 | 2.01E-01 | 1 |
| SLFN11 | 1.76E-01 | 1 | GABRA4 | 2.01E-01 | 1 |
| LCK | 1.76E-01 | 1 | EBAG9 | 2.01E-01 | 1 |
| POLR3F | 1.76E-01 | 1 | C5orf38 | 2.01E-01 | 1 |
| BRMS1 | 1.76E-01 | 1 | ZBTB40 | 2.01E-01 | 1 |
| RGMA | 1.76E-01 | 1 | CENPA | 2.02E-01 | 1 |
| MYF5 | 1.76E-01 | 1 | SESTD1 | 2.02E-01 | 1 |
| FAM47B | 1.76E-01 | 1 | KCNC3 | 2.02E-01 | 1 |
| HDAC3 | 1.76E-01 | 1 | SEC23B | 2.02E-01 | 1 |
| TXLNG | 1.76E-01 | 1 | SUSD3 | 2.02E-01 | 1 |
| MAP4K2 | 1.76E-01 | 1 | OR51D1 | 2.02E-01 | 1 |
| NINJ1 | 1.76E-01 | 1 | ELOVL3 | 2.02E-01 | 1 |
| GPHB5 | 1.76E-01 | 1 | RSL24D1 | 2.02E-01 | 1 |
| CSN1S1 | 1.76E-01 | 1 | GABRA1 | 2.02E-01 | 1 |
| HIST1H2B | 1.76E-01 | 1 | ABRA | 2.02E-01 | 1 |
| HOXC10 | 1.76E-01 | 1 | CD79B | 2.02E-01 | 1 |
| CDC42 | 1.76E-01 | 1 | CLPB | 2.03E-01 | 1 |
| LYAR | 1.77E-01 | 1 | NKIRAS2 | 2.03E-01 | 1 |
| NCKAP1L | 1.77E-01 | 1 | CPSF2 | 2.03E-01 | 1 |
| FMN2 | 1.77E-01 | 1 | HMGB4 | 2.03E-01 | 1 |
| FATE1 | 1.77E-01 | 1 | AFG3L2 | 2.03E-01 | 1 |
| FUT1 | 1.77E-01 | 1 | SMC2 | 2.03E-01 | 1 |
| OR5J2 | 1.77E-01 | 1 | ADAM23 | 2.03E-01 | 1 |
| FKBP4 | 1.77E-01 | 1 | SUCLA2 | 2.04E-01 | 1 |
| CRP | 1.77E-01 | 1 | SPATA5 | 2.04E-01 | 1 |
| KCNMB2 | 1.77E-01 | 1 | ODC1 | 2.04E-01 | 1 |
| KLRC4 | 1.78E-01 | 1 | PDE1C | 2.04E-01 | 1 |
| PMEPA1 | 1.78E-01 | 1 | TF | 2.04E-01 | 1 |
| HIST1H3D | 1.78E-01 | 1 | C8A | 2.04E-01 | 1 |
| SHD | 1.78E-01 | 1 | GBP2 | 2.04E-01 | 1 |
| HS3ST1 | 1.78E-01 | 1 | NRAS | 2.04E-01 | 1 |
| HADHB | 1.78E-01 | 1 | EFHC2 | 2.04E-01 | 1 |
| ZNRD1 | 1.78E-01 | 1 | RD3 | 2.04E-01 | 1 |
| NOC4L | 1.78E-01 | 1 | ZNF76 | 2.04E-01 | 1 |
| PFN3 | 1.78E-01 | 1 | ZBED4 | 2.05E-01 | 1 |
| CTSK | 1.78E-01 | 1 | MFAP5 | 2.05E-01 | 1 |
| PNMA3 | 1.78E-01 | 1 | PAPD7 | 2.05E-01 | 1 |
| GNAZ | 1.79E-01 | 1 | FGFR4 | 2.05E-01 | 1 |
| UBOX5 | 1.79E-01 | 1 | USP2 | 2.05E-01 | 1 |
| MYO1G | 1.79E-01 | 1 | SLC9A3 | 2.05E-01 | 1 |
| PHLDA1 | 1.79E-01 | 1 | SLC39A10 | 2.05E-01 | 1 |
| OR5R1 | 1.79E-01 | 1 | HBE1 | 2.05E-01 | 1 |
| DPF3 | 1.79E-01 | 1 | ZNF454 | 2.06E-01 | 1 |
| BEST3 | 1.79E-01 | 1 | JAM2 | 2.06E-01 | 1 |
| VASH1 | 1.79E-01 | 1 | TMEM173 | 2.06E-01 | 1 |
| MKNK1 | 1.79E-01 | 1 | RPL7A | 2.06E-01 | 1 |
| ANKRD1 | 1.79E-01 | 1 | TIMP1 | 2.06E-01 | 1 |
| GDI2 | 1.79E-01 | 1 | LCE5A | 2.06E-01 | 1 |

| RWDD2B | 1.79E-01 | 1 | INS-IGF2 | 2.06E-01 | 1 |
| --- | --- | --- | --- | --- | --- |
| FGD5 | 1.79E-01 | 1 | RAPH1 | 2.06E-01 | 1 |
| EXO1 | 1.79E-01 | 1 | EIF3F | 2.06E-01 | 1 |
| EMP1 | 1.79E-01 | 1 | PTPRC | 2.06E-01 | 1 |
| ZDHHC20 | 1.80E-01 | 1 | CUBN | 2.06E-01 | 1 |
| CEP55 | 1.80E-01 | 1 | ST8SIA6 | 2.07E-01 | 1 |
| ATP6V1A | 1.80E-01 | 1 | CST8 | 2.07E-01 | 1 |
| KCTD16 | 1.80E-01 | 1 | TMIGD1 | 2.07E-01 | 1 |
| SAAL1 | 1.80E-01 | 1 | NARS | 2.07E-01 | 1 |
| SCAND1 | 1.80E-01 | 1 | ZNF669 | 2.07E-01 | 1 |
| SRCAP | 1.80E-01 | 1 | NPR2 | 2.07E-01 | 1 |
| HN1 | 1.80E-01 | 1 | KRTAP9-9 | 2.07E-01 | 1 |
| BATF2 | 1.80E-01 | 1 | FAM78A | 2.07E-01 | 1 |
| TMEM132 | 1.80E-01 | 1 | COPZ1 | 2.07E-01 | 1 |
| USP49 | 1.81E-01 | 1 | AK2 | 2.07E-01 | 1 |
| SMR3A | 1.81E-01 | 1 | MANEA | 2.07E-01 | 1 |
| CER1 | 1.81E-01 | 1 | HSD17B6 | 2.07E-01 | 1 |
| MTIF2 | 1.81E-01 | 1 | LEFTY1 | 2.07E-01 | 1 |
| SEC14L1 | 1.81E-01 | 1 | DDX50 | 2.07E-01 | 1 |
| KPTN | 1.81E-01 | 1 | PRPF19 | 2.07E-01 | 1 |
| CYLC1 | 1.81E-01 | 1 | OR2L13 | 2.08E-01 | 1 |
| KSR2 | 1.81E-01 | 1 | SYNCRIP | 2.08E-01 | 1 |
| OR2G2 | 1.81E-01 | 1 | DMRTA2 | 2.08E-01 | 1 |
| MTUS2 | 1.81E-01 | 1 | SGSM1 | 2.08E-01 | 1 |
| LZIC | 1.81E-01 | 1 | LCMT2 | 2.08E-01 | 1 |
| OR8D4 | 1.81E-01 | 1 | CYC1 | 2.08E-01 | 1 |
| HTR1E | 1.81E-01 | 1 | TMBIM6 | 2.08E-01 | 1 |
| RNF216 | 1.82E-01 | 1 | RFWD2 | 2.08E-01 | 1 |
| STK17A | 1.82E-01 | 1 | CD4 | 2.08E-01 | 1 |
| NDN | 1.82E-01 | 1 | TMPO | 2.08E-01 | 1 |
| TEX19 | 1.82E-01 | 1 | FCRL1 | 2.08E-01 | 1 |
| BTN1A1 | 1.82E-01 | 1 | OR8J1 | 2.08E-01 | 1 |
| HMSD | 1.82E-01 | 1 | LANCL1 | 2.09E-01 | 1 |
| ELF5 | 1.82E-01 | 1 | CAMKK2 | 2.09E-01 | 1 |
| FNDC1 | 1.82E-01 | 1 | AGPAT1 | 2.09E-01 | 1 |
| SNX8 | 1.82E-01 | 1 | CXorf58 | 2.09E-01 | 1 |
| A2ML1 | 1.82E-01 | 1 | ZNF567 | 2.09E-01 | 1 |
| CD207 | 1.82E-01 | 1 | ZNF576 | 2.09E-01 | 1 |
| LRTM1 | 1.82E-01 | 1 | SEMA4B | 2.09E-01 | 1 |
| WWC1 | 1.82E-01 | 1 | PRPSAP2 | 2.09E-01 | 1 |
| C19orf44 | 1.82E-01 | 1 | CGB2 | 2.09E-01 | 1 |
| ZFP36L2 | 1.83E-01 | 1 | ATP9A | 2.09E-01 | 1 |
| SERPINB6 | 1.83E-01 | 1 | FAM83E | 2.09E-01 | 1 |
| IGLL1 | 1.83E-01 | 1 | PSMA7 | 2.09E-01 | 1 |
| DNAJB14 | 1.83E-01 | 1 | SEZ6L2 | 2.09E-01 | 1 |
| CCR6 | 1.83E-01 | 1 | SEC11C | 2.09E-01 | 1 |
| SLC9A9 | 1.83E-01 | 1 | GMFG | 2.09E-01 | 1 |
| GPRASP2 | 1.83E-01 | 1 | NPTX2 | 2.09E-01 | 1 |
| ANAPC7 | 1.83E-01 | 1 | TFE3 | 2.09E-01 | 1 |
| EVC2 | 1.83E-01 | 1 | NCOA2 | 2.09E-01 | 1 |
| ADH4 | 1.83E-01 | 1 | MSH4 | 2.10E-01 | 1 |
| OR4Q3 | 1.84E-01 | 1 | KIAA1407 | 2.10E-01 | 1 |
| TMEM105 | 1.84E-01 | 1 | OR2M3 | 2.10E-01 | 1 |

| TAPT1 | 1.84E-01 | 1 | GEM | 2.10E-01 | 1 |
| --- | --- | --- | --- | --- | --- |
| TIMP1 | 1.84E-01 | 1 | BMS1 | 2.10E-01 | 1 |
| C4orf47 | 1.84E-01 | 1 | SFPQ | 2.10E-01 | 1 |
| CLSTN2 | 1.84E-01 | 1 | RRBP1 | 2.10E-01 | 1 |
| TRIM27 | 1.84E-01 | 1 | TMPRSS11 | 2.10E-01 | 1 |
| USP22 | 1.84E-01 | 1 | GLT8D2 | 2.10E-01 | 1 |
| FRMPD4 | 1.84E-01 | 1 | AP1G1 | 2.10E-01 | 1 |
| DYNC1LI | 1.84E-01 | 1 | NT5DC1 | 2.10E-01 | 1 |
| CCKBR | 1.84E-01 | 1 | KCTD21 | 2.10E-01 | 1 |
| LPPR4 | 1.84E-01 | 1 | MS4A15 | 2.10E-01 | 1 |
| ASF1A | 1.84E-01 | 1 | GEN1 | 2.10E-01 | 1 |
| NGFR | 1.84E-01 | 1 | TRIM4 | 2.10E-01 | 1 |
| CCBE1 | 1.85E-01 | 1 | CD19 | 2.10E-01 | 1 |
| TRIM71 | 1.85E-01 | 1 | BAHD1 | 2.10E-01 | 1 |
| SERTAD4 | 1.85E-01 | 1 | FAM83H | 2.11E-01 | 1 |
| MVK | 1.85E-01 | 1 | KEL | 2.11E-01 | 1 |
| KCNQ2 | 1.85E-01 | 1 | MTA1 | 2.11E-01 | 1 |
| GRIP1 | 1.85E-01 | 1 | NR0B2 | 2.11E-01 | 1 |
| CDC27 | 1.85E-01 | 1 | RASSF4 | 2.11E-01 | 1 |
| DAZL | 1.85E-01 | 1 | IKZF3 | 2.11E-01 | 1 |
| NXNL1 | 1.85E-01 | 1 | PMM1 | 2.11E-01 | 1 |
| KLK11 | 1.85E-01 | 1 | STAT2 | 2.11E-01 | 1 |
| ACTRT2 | 1.86E-01 | 1 | BZW1 | 2.11E-01 | 1 |
| DBF4B | 1.86E-01 | 1 | WFDC3 | 2.11E-01 | 1 |
| RBP5 | 1.86E-01 | 1 | NUSAP1 | 2.11E-01 | 1 |
| ARIH2 | 1.86E-01 | 1 | TMPRSS11 | 2.11E-01 | 1 |
| CDR2L | 1.86E-01 | 1 | SURF4 | 2.11E-01 | 1 |
| NUFIP1 | 1.86E-01 | 1 | ANKHD1-E | 2.12E-01 | 1 |
| CCNB1IP1 | 1.86E-01 | 1 | RAB31 | 2.12E-01 | 1 |
| RWDD2A | 1.86E-01 | 1 | AQP11 | 2.12E-01 | 1 |
| FGFRL1 | 1.86E-01 | 1 | DPYSL4 | 2.12E-01 | 1 |
| DHODH | 1.86E-01 | 1 | C6orf10 | 2.12E-01 | 1 |
| CASP4 | 1.86E-01 | 1 | GLB1L3 | 2.12E-01 | 1 |
| CNOT4 | 1.86E-01 | 1 | CSE1L | 2.12E-01 | 1 |
| FOXN4 | 1.87E-01 | 1 | COMMD2 | 2.12E-01 | 1 |
| PEX6 | 1.87E-01 | 1 | ARHGEF15 | 2.13E-01 | 1 |
| ODF2 | 1.87E-01 | 1 | TTLL3 | 2.13E-01 | 1 |
| OR2G6 | 1.87E-01 | 1 | CNGA3 | 2.13E-01 | 1 |
| FAM175B | 1.87E-01 | 1 | FANCB | 2.13E-01 | 1 |
| ULK3 | 1.87E-01 | 1 | RGS5 | 2.13E-01 | 1 |
| OR5B17 | 1.87E-01 | 1 | GPR83 | 2.13E-01 | 1 |
| AP2A2 | 1.87E-01 | 1 | ACADL | 2.13E-01 | 1 |
| TMEM165 | 1.87E-01 | 1 | SFRP2 | 2.13E-01 | 1 |
| SLC6A13 | 1.87E-01 | 1 | GFI1B | 2.13E-01 | 1 |
| RFC1 | 1.87E-01 | 1 | ZBED2 | 2.13E-01 | 1 |
| KLRC2 | 1.87E-01 | 1 | GRASP | 2.13E-01 | 1 |
| KRTAP12 | 1.88E-01 | 1 | BPTF | 2.14E-01 | 1 |
| SLC39A10 | 1.88E-01 | 1 | MRGPRE | 2.14E-01 | 1 |
| SDHA | 1.88E-01 | 1 | VRK2 | 2.14E-01 | 1 |
| C20orf202 | 1.88E-01 | 1 | TGFBR2 | 2.14E-01 | 1 |
| LILRB3 | 1.88E-01 | 1 | FOXRED2 | 2.14E-01 | 1 |
| MPEG1 | 1.88E-01 | 1 | TMEM127 | 2.14E-01 | 1 |
| CDK9 | 1.88E-01 | 1 | PGS1 | 2.14E-01 | 1 |

| ARL3 | 1.88E-01 | 1 | SMR3A | 2.14E-01 | 1 |
| --- | --- | --- | --- | --- | --- |
| OR52B4 | 1.88E-01 | 1 | MAL | 2.15E-01 | 1 |
| TUBG1 | 1.89E-01 | 1 | SEPHS2 | 2.15E-01 | 1 |
| KRT81 | 1.89E-01 | 1 | HNF1A | 2.15E-01 | 1 |
| SERPINB3 | 1.89E-01 | 1 | CRYGA | 2.15E-01 | 1 |
| TRA2A | 1.89E-01 | 1 | ZNF845 | 2.15E-01 | 1 |
| MVD | 1.89E-01 | 1 | IGFBP4 | 2.15E-01 | 1 |
| C9orf50 | 1.89E-01 | 1 | NEK10 | 2.15E-01 | 1 |
| FAM196B | 1.89E-01 | 1 | LARP1B | 2.16E-01 | 1 |
| SYT5 | 1.89E-01 | 1 | GPR142 | 2.16E-01 | 1 |
| WDR73 | 1.90E-01 | 1 | KCTD8 | 2.16E-01 | 1 |
| DDX46 | 1.90E-01 | 1 | LGSN | 2.16E-01 | 1 |
| HOXA6 | 1.90E-01 | 1 | GDPD4 | 2.16E-01 | 1 |
| HIGD1A | 1.90E-01 | 1 | SLC34A2 | 2.16E-01 | 1 |
| CDCA7 | 1.90E-01 | 1 | SLC22A18 | 2.16E-01 | 1 |
| ZNF589 | 1.90E-01 | 1 | SERPINB3 | 2.16E-01 | 1 |
| NUDC | 1.90E-01 | 1 | WT1 | 2.16E-01 | 1 |
| ZDHHC23 | 1.90E-01 | 1 | AVPR1B | 2.16E-01 | 1 |
| COL16A1 | 1.90E-01 | 1 | SLC7A4 | 2.16E-01 | 1 |
| LENG9 | 1.90E-01 | 1 | C1QTNF1 | 2.16E-01 | 1 |
| ZMAT5 | 1.90E-01 | 1 | NUDT13 | 2.16E-01 | 1 |
| HIST1H1B | 1.91E-01 | 1 | GAS2L1 | 2.17E-01 | 1 |
| OTUD3 | 1.91E-01 | 1 | HOXD4 | 2.17E-01 | 1 |
| C5 | 1.91E-01 | 1 | SERPINB4 | 2.17E-01 | 1 |
| ESR1 | 1.91E-01 | 1 | HRC | 2.17E-01 | 1 |
| SSBP2 | 1.91E-01 | 1 | CCNB1 | 2.17E-01 | 1 |
| CCT4 | 1.91E-01 | 1 | MDM1 | 2.17E-01 | 1 |
| INTS8 | 1.91E-01 | 1 | FLT4 | 2.17E-01 | 1 |
| TPT1 | 1.91E-01 | 1 | E2F8 | 2.18E-01 | 1 |
| HIPK2 | 1.91E-01 | 1 | SLC35C1 | 2.18E-01 | 1 |
| ZBTB12 | 1.91E-01 | 1 | IFRD1 | 2.18E-01 | 1 |
| ZP2 | 1.91E-01 | 1 | FIG4 | 2.18E-01 | 1 |
| CCDC84 | 1.91E-01 | 1 | GGT7 | 2.18E-01 | 1 |
| CFL1 | 1.91E-01 | 1 | YWHAE | 2.18E-01 | 1 |
| DNER | 1.92E-01 | 1 | KCTD16 | 2.18E-01 | 1 |
| PPP1CA | 1.92E-01 | 1 | SYNGR4 | 2.18E-01 | 1 |
| TREM1 | 1.92E-01 | 1 | SOST | 2.18E-01 | 1 |
| NLGN4Y | 1.92E-01 | 1 | ZNF93 | 2.18E-01 | 1 |
| MCTP1 | 1.92E-01 | 1 | LMNB1 | 2.18E-01 | 1 |
| IGDCC4 | 1.92E-01 | 1 | HS3ST6 | 2.18E-01 | 1 |
| PAQR8 | 1.92E-01 | 1 | CFI | 2.18E-01 | 1 |
| RBM18 | 1.92E-01 | 1 | ZNF107 | 2.18E-01 | 1 |
| PDK1 | 1.92E-01 | 1 | NAT8 | 2.18E-01 | 1 |
| PCYT2 | 1.92E-01 | 1 | SV2B | 2.18E-01 | 1 |
| LAG3 | 1.92E-01 | 1 | LAPTM5 | 2.18E-01 | 1 |
| EED | 1.92E-01 | 1 | YWHAG | 2.19E-01 | 1 |
| LONP2 | 1.92E-01 | 1 | BBS4 | 2.19E-01 | 1 |
| LSM1 | 1.92E-01 | 1 | PRSS45 | 2.19E-01 | 1 |
| USP5 | 1.92E-01 | 1 | ZNF215 | 2.19E-01 | 1 |
| FOXRED1 | 1.93E-01 | 1 | TUT1 | 2.19E-01 | 1 |
| NTRK2 | 1.93E-01 | 1 | STUB1 | 2.19E-01 | 1 |
| FETUB | 1.93E-01 | 1 | PUS1 | 2.19E-01 | 1 |
| LRCH2 | 1.93E-01 | 1 | DNASE1L1 | 2.19E-01 | 1 |

| OR5M3 | 1.93E-01 | 1 | TEX13A | 2.19E-01 | 1 |
| --- | --- | --- | --- | --- | --- |
| KIAA0907 | 1.93E-01 | 1 | GLO1 | 2.19E-01 | 1 |
| CLNK | 1.94E-01 | 1 | PTPN4 | 2.20E-01 | 1 |
| DUSP14 | 1.94E-01 | 1 | TMCO5A | 2.20E-01 | 1 |
| NGEF | 1.94E-01 | 1 | HSH2D | 2.20E-01 | 1 |
| FZD5 | 1.94E-01 | 1 | OPCML | 2.20E-01 | 1 |
| LAX1 | 1.94E-01 | 1 | LBX1 | 2.20E-01 | 1 |
| GBA3 | 1.94E-01 | 1 | PURA | 2.20E-01 | 1 |
| PI15 | 1.95E-01 | 1 | GGTLC2 | 2.21E-01 | 1 |
| CRISPLD1 | 1.95E-01 | 1 | TFAP2D | 2.21E-01 | 1 |
| KLHDC3 | 1.95E-01 | 1 | ZC3H12A | 2.21E-01 | 1 |
| PHTF1 | 1.95E-01 | 1 | L1CAM | 2.21E-01 | 1 |
| ISCU | 1.95E-01 | 1 | CAPN3 | 2.21E-01 | 1 |
| C9orf142 | 1.95E-01 | 1 | WDR61 | 2.21E-01 | 1 |
| ZNF347 | 1.95E-01 | 1 | C2orf57 | 2.21E-01 | 1 |
| MCOLN2 | 1.95E-01 | 1 | COL13A1 | 2.21E-01 | 1 |
| ARR3 | 1.95E-01 | 1 | PAEP | 2.21E-01 | 1 |
| KCNMB3 | 1.95E-01 | 1 | ARF1 | 2.22E-01 | 1 |
| FUT3 | 1.95E-01 | 1 | SNX8 | 2.22E-01 | 1 |
| RACGAP1 | 1.95E-01 | 1 | ZNF829 | 2.22E-01 | 1 |
| DNAJC1 | 1.96E-01 | 1 | CRY2 | 2.22E-01 | 1 |
| TMEM208 | 1.96E-01 | 1 | KIF3B | 2.22E-01 | 1 |
| ALDOB | 1.96E-01 | 1 | PDZD11 | 2.22E-01 | 1 |
| DOK6 | 1.96E-01 | 1 | CHIC2 | 2.22E-01 | 1 |
| IKZF1 | 1.96E-01 | 1 | C17orf64 | 2.22E-01 | 1 |
| HS3ST6 | 1.96E-01 | 1 | RARG | 2.22E-01 | 1 |
| DIO3 | 1.96E-01 | 1 | GRIP1 | 2.22E-01 | 1 |
| CIB4 | 1.96E-01 | 1 | FAHD2A | 2.22E-01 | 1 |
| C16orf90 | 1.96E-01 | 1 | PNPLA4 | 2.22E-01 | 1 |
| TMEM40 | 1.97E-01 | 1 | FHDC1 | 2.22E-01 | 1 |
| ATRNL1 | 1.97E-01 | 1 | RAB9A | 2.22E-01 | 1 |
| TBX18 | 1.97E-01 | 1 | ANG | 2.23E-01 | 1 |
| LAYN | 1.97E-01 | 1 | P2RY12 | 2.23E-01 | 1 |
| TAC3 | 1.97E-01 | 1 | MED13 | 2.23E-01 | 1 |
| CD244 | 1.97E-01 | 1 | ZNF862 | 2.23E-01 | 1 |
| STIP1 | 1.97E-01 | 1 | DEFB106B | 2.23E-01 | 1 |
| ACTG1 | 1.97E-01 | 1 | ANKRD13A | 2.23E-01 | 1 |
| CARD18 | 1.98E-01 | 1 | MFAP2 | 2.23E-01 | 1 |
| SOX9 | 1.98E-01 | 1 | NFE2L1 | 2.23E-01 | 1 |
| OR2L13 | 1.98E-01 | 1 | BAZ2A | 2.24E-01 | 1 |
| FAM98C | 1.98E-01 | 1 | ZNF700 | 2.24E-01 | 1 |
| EAF1 | 1.98E-01 | 1 | ZNF326 | 2.24E-01 | 1 |
| LHFPL5 | 1.98E-01 | 1 | NUF2 | 2.24E-01 | 1 |
| CCRL2 | 1.98E-01 | 1 | HIST1H2AK | 2.24E-01 | 1 |
| T | 1.98E-01 | 1 | SHKBP1 | 2.24E-01 | 1 |
| ZNF541 | 1.98E-01 | 1 | DNAJB6 | 2.24E-01 | 1 |
| SIX5 | 1.98E-01 | 1 | MLH3 | 2.24E-01 | 1 |
| KRT6B | 1.98E-01 | 1 | TMEM39A | 2.24E-01 | 1 |
| CORO1C | 1.98E-01 | 1 | C9orf57 | 2.24E-01 | 1 |
| MAP2K3 | 1.98E-01 | 1 | PIK3CA | 2.24E-01 | 1 |
| SLC29A4 | 1.99E-01 | 1 | GRIA2 | 2.25E-01 | 1 |
| SIAH3 | 1.99E-01 | 1 | CPZ | 2.25E-01 | 1 |
| IDH2 | 1.99E-01 | 1 | SMARCA4 | 2.25E-01 | 1 |

| NDRG2 | 1.99E-01 | 1 | MRPS15 | 2.25E-01 | 1 |
| --- | --- | --- | --- | --- | --- |
| MANF | 1.99E-01 | 1 | STAT5B | 2.25E-01 | 1 |
| TARM1 | 1.99E-01 | 1 | RANBP10 | 2.26E-01 | 1 |
| GBP2 | 1.99E-01 | 1 | APCS | 2.26E-01 | 1 |
| PIGZ | 1.99E-01 | 1 | GALNT5 | 2.26E-01 | 1 |
| DUOXA2 | 1.99E-01 | 1 | TNNI2 | 2.26E-01 | 1 |
| HIP1 | 2.00E-01 | 1 | EFR3B | 2.26E-01 | 1 |
| TRPC6 | 2.00E-01 | 1 | SCMH1 | 2.26E-01 | 1 |
| AOAH | 2.00E-01 | 1 | KRTAP10-9 | 2.26E-01 | 1 |
| BMP5 | 2.00E-01 | 1 | BACH1 | 2.26E-01 | 1 |
| PLEKHO1 | 2.00E-01 | 1 | EIF2AK2 | 2.26E-01 | 1 |
| EVC | 2.00E-01 | 1 | RBM18 | 2.26E-01 | 1 |
| SPN | 2.00E-01 | 1 | RPL8 | 2.26E-01 | 1 |
| FAM3A | 2.00E-01 | 1 | PHLDB3 | 2.26E-01 | 1 |
| MAP1B | 2.00E-01 | 1 | RAG1 | 2.27E-01 | 1 |
| OTOP1 | 2.00E-01 | 1 | CDT1 | 2.27E-01 | 1 |
| LRRC19 | 2.00E-01 | 1 | ARNTL2 | 2.27E-01 | 1 |
| PSMD12 | 2.01E-01 | 1 | TWISTNB | 2.27E-01 | 1 |
| TGIF2 | 2.01E-01 | 1 | CCND1 | 2.27E-01 | 1 |
| AP1G1 | 2.01E-01 | 1 | FBXO45 | 2.28E-01 | 1 |
| SMPD2 | 2.01E-01 | 1 | ITGB8 | 2.28E-01 | 1 |
| TMEM39B | 2.01E-01 | 1 | SH3D19 | 2.28E-01 | 1 |
| CCDC57 | 2.01E-01 | 1 | TBC1D10B | 2.28E-01 | 1 |
| CLEC9A | 2.01E-01 | 1 | FAIM3 | 2.28E-01 | 1 |
| ABAT | 2.01E-01 | 1 | HK3 | 2.29E-01 | 1 |
| CCDC80 | 2.01E-01 | 1 | KCNK6 | 2.29E-01 | 1 |
| SYTL4 | 2.01E-01 | 1 | CTDSP1 | 2.29E-01 | 1 |
| ZNF791 | 2.01E-01 | 1 | RAB7A | 2.29E-01 | 1 |
| ASCL4 | 2.02E-01 | 1 | WNT2 | 2.29E-01 | 1 |
| DPEP2 | 2.02E-01 | 1 | MS4A10 | 2.29E-01 | 1 |
| ATP6V0A | 2.02E-01 | 1 | C4orf26 | 2.29E-01 | 1 |
| GRIN1 | 2.02E-01 | 1 | TMEM62 | 2.29E-01 | 1 |
| SLC17A2 | 2.02E-01 | 1 | DENND2A | 2.29E-01 | 1 |
| GPR63 | 2.02E-01 | 1 | C1orf106 | 2.29E-01 | 1 |
| RASD1 | 2.02E-01 | 1 | SLC35F2 | 2.30E-01 | 1 |
| FBXL16 | 2.02E-01 | 1 | ARPC2 | 2.30E-01 | 1 |
| MFSD11 | 2.02E-01 | 1 | CRAT | 2.30E-01 | 1 |
| SPAST | 2.02E-01 | 1 | FCRL4 | 2.30E-01 | 1 |
| ADRA1D | 2.02E-01 | 1 | HIST1H3I | 2.30E-01 | 1 |
| NEU3 | 2.02E-01 | 1 | SESN2 | 2.30E-01 | 1 |
| DEFB129 | 2.02E-01 | 1 | SLFN11 | 2.30E-01 | 1 |
| CCDC17 | 2.02E-01 | 1 | GFRAL | 2.30E-01 | 1 |
| ARHGAP2 | 2.02E-01 | 1 | PGLYRP2 | 2.31E-01 | 1 |
| HIST1H3H | 2.02E-01 | 1 | EDN3 | 2.31E-01 | 1 |
| OR51S1 | 2.03E-01 | 1 | MID2 | 2.31E-01 | 1 |
| CCR3 | 2.03E-01 | 1 | PPIC | 2.31E-01 | 1 |
| COL18A1 | 2.03E-01 | 1 | CYP2R1 | 2.31E-01 | 1 |
| SUV39H1 | 2.03E-01 | 1 | KIF18B | 2.31E-01 | 1 |
| LRIG1 | 2.03E-01 | 1 | LMLN | 2.31E-01 | 1 |
| NCEH1 | 2.03E-01 | 1 | SRMS | 2.31E-01 | 1 |
| GSTA5 | 2.03E-01 | 1 | DMRT1 | 2.31E-01 | 1 |
| IRGC | 2.03E-01 | 1 | B9D2 | 2.31E-01 | 1 |
| FIGN | 2.03E-01 | 1 | UGT2B4 | 2.32E-01 | 1 |

| MORN1 | 2.04E-01 | 1 | FAM150B | 2.32E-01 | 1 |
| --- | --- | --- | --- | --- | --- |
| IKBIP | 2.04E-01 | 1 | AOC2 | 2.32E-01 | 1 |
| MAPK1 | 2.04E-01 | 1 | CAPN13 | 2.32E-01 | 1 |
| KRT1 | 2.04E-01 | 1 | HOXA5 | 2.32E-01 | 1 |
| CLDN16 | 2.04E-01 | 1 | PUS7 | 2.32E-01 | 1 |
| NOP56 | 2.04E-01 | 1 | EPHA8 | 2.32E-01 | 1 |
| OR5I1 | 2.04E-01 | 1 | ATF4 | 2.32E-01 | 1 |
| BYSL | 2.05E-01 | 1 | H2AFZ | 2.33E-01 | 1 |
| RIPK3 | 2.05E-01 | 1 | JUN | 2.33E-01 | 1 |
| GADD45G | 2.05E-01 | 1 | TNIP3 | 2.33E-01 | 1 |
| MOCS1 | 2.05E-01 | 1 | MS4A14 | 2.33E-01 | 1 |
| CCDC59 | 2.05E-01 | 1 | TBC1D19 | 2.33E-01 | 1 |
| SOCS2 | 2.05E-01 | 1 | VWDE | 2.33E-01 | 1 |
| DLX6 | 2.06E-01 | 1 | VASN | 2.34E-01 | 1 |
| PTGER2 | 2.06E-01 | 1 | VASP | 2.34E-01 | 1 |
| RAD1 | 2.06E-01 | 1 | ZXDB | 2.34E-01 | 1 |
| ADAMTS | 2.06E-01 | 1 | SUPT3H | 2.34E-01 | 1 |
| SUV39H2 | 2.06E-01 | 1 | PLXNA4 | 2.34E-01 | 1 |
| ZIC1 | 2.06E-01 | 1 | ANK1 | 2.34E-01 | 1 |
| KCNT1 | 2.06E-01 | 1 | ETV6 | 2.34E-01 | 1 |
| CS | 2.06E-01 | 1 | MYLK4 | 2.34E-01 | 1 |
| HDAC6 | 2.06E-01 | 1 | CFTR | 2.35E-01 | 1 |
| SVIP | 2.07E-01 | 1 | THRAP3 | 2.35E-01 | 1 |
| CST9L | 2.07E-01 | 1 | ALOX15B | 2.35E-01 | 1 |
| ASTE1 | 2.07E-01 | 1 | EXOC5 | 2.35E-01 | 1 |
| SPAG7 | 2.07E-01 | 1 | CDC42EP1 | 2.35E-01 | 1 |
| LYRM4 | 2.07E-01 | 1 | THY1 | 2.35E-01 | 1 |
| TRIT1 | 2.07E-01 | 1 | METTL2B | 2.35E-01 | 1 |
| FCRL2 | 2.07E-01 | 1 | C15orf53 | 2.35E-01 | 1 |
| ETV5 | 2.07E-01 | 1 | KIR3DL2 | 2.35E-01 | 1 |
| SYT7 | 2.07E-01 | 1 | KCNK16 | 2.35E-01 | 1 |
| HNF4G | 2.07E-01 | 1 | FAR1 | 2.35E-01 | 1 |
| DAPK2 | 2.07E-01 | 1 | C5orf52 | 2.35E-01 | 1 |
| FSHR | 2.07E-01 | 1 | DCAF12L2 | 2.36E-01 | 1 |
| BTBD8 | 2.08E-01 | 1 | SEC23IP | 2.36E-01 | 1 |
| DCAF17 | 2.08E-01 | 1 | ARMC6 | 2.36E-01 | 1 |
| SLC17A7 | 2.08E-01 | 1 | FRMD6 | 2.36E-01 | 1 |
| KCNA10 | 2.08E-01 | 1 | PPT2 | 2.36E-01 | 1 |
| ZSWIM1 | 2.08E-01 | 1 | AP3S1 | 2.36E-01 | 1 |
| IPO7 | 2.08E-01 | 1 | EXOSC3 | 2.36E-01 | 1 |
| RPA1 | 2.08E-01 | 1 | FAM111A | 2.36E-01 | 1 |
| OPRL1 | 2.08E-01 | 1 | CYLC1 | 2.36E-01 | 1 |
| CIB1 | 2.08E-01 | 1 | MAD1L1 | 2.36E-01 | 1 |
| SH3YL1 | 2.08E-01 | 1 | PSMA3 | 2.37E-01 | 1 |
| KRTAP17 | 2.08E-01 | 1 | PIGL | 2.37E-01 | 1 |
| SPATA18 | 2.08E-01 | 1 | PIGS | 2.37E-01 | 1 |
| PRR16 | 2.08E-01 | 1 | GEMIN5 | 2.37E-01 | 1 |
| COL19A1 | 2.08E-01 | 1 | SEMA5B | 2.37E-01 | 1 |
| CCDC79 | 2.09E-01 | 1 | ITGA9 | 2.37E-01 | 1 |
| ZNF804A | 2.09E-01 | 1 | RASGRP1 | 2.37E-01 | 1 |
| PSAP | 2.09E-01 | 1 | COL6A2 | 2.37E-01 | 1 |
| FEZF2 | 2.09E-01 | 1 | MORF4L2 | 2.37E-01 | 1 |
| SLC37A3 | 2.09E-01 | 1 | MAGEA11 | 2.37E-01 | 1 |

| HDDC3 | 2.09E-01 | 1 | TAF9B | 2.38E-01 | 1 |
| --- | --- | --- | --- | --- | --- |
| TMEM179 | 2.09E-01 | 1 | FLG | 2.38E-01 | 1 |
| CHD9 | 2.09E-01 | 1 | GBP3 | 2.38E-01 | 1 |
| PSMD8 | 2.09E-01 | 1 | HDC | 2.38E-01 | 1 |
| FABP7 | 2.10E-01 | 1 | PAPSS1 | 2.38E-01 | 1 |
| RBM20 | 2.10E-01 | 1 | SSX5 | 2.38E-01 | 1 |
| CXCL6 | 2.10E-01 | 1 | DNAJC18 | 2.38E-01 | 1 |
| SCCPDH | 2.10E-01 | 1 | SYAP1 | 2.39E-01 | 1 |
| OR51A7 | 2.10E-01 | 1 | RPL18 | 2.39E-01 | 1 |
| PTPRJ | 2.10E-01 | 1 | GNA12 | 2.39E-01 | 1 |
| TAAR5 | 2.10E-01 | 1 | MARK2 | 2.39E-01 | 1 |
| ARAF | 2.10E-01 | 1 | RNF11 | 2.39E-01 | 1 |
| KLK6 | 2.10E-01 | 1 | HELT | 2.39E-01 | 1 |
| DDX47 | 2.10E-01 | 1 | SNX5 | 2.39E-01 | 1 |
| STMN3 | 2.10E-01 | 1 | TATDN2 | 2.39E-01 | 1 |
| CATSPER | 2.10E-01 | 1 | RAG2 | 2.40E-01 | 1 |
| CWF19L1 | 2.10E-01 | 1 | ESR1 | 2.40E-01 | 1 |
| PON3 | 2.10E-01 | 1 | TPP1 | 2.40E-01 | 1 |
| CPSF1 | 2.11E-01 | 1 | DHRS3 | 2.40E-01 | 1 |
| HOXB2 | 2.11E-01 | 1 | LTK | 2.40E-01 | 1 |
| TRAF3 | 2.11E-01 | 1 | CYTIP | 2.40E-01 | 1 |
| ATP5G3 | 2.11E-01 | 1 | CACNB4 | 2.40E-01 | 1 |
| TTC5 | 2.11E-01 | 1 | 6-Mar | 2.40E-01 | 1 |
| HIST1H2A | 2.11E-01 | 1 | OR1J1 | 2.40E-01 | 1 |
| PMS1 | 2.11E-01 | 1 | ZNF638 | 2.40E-01 | 1 |
| ARL13B | 2.11E-01 | 1 | GSDMC | 2.40E-01 | 1 |
| TMPRSS1 | 2.11E-01 | 1 | ZNF496 | 2.40E-01 | 1 |
| EXD2 | 2.11E-01 | 1 | FLCN | 2.41E-01 | 1 |
| USP20 | 2.11E-01 | 1 | CCDC151 | 2.41E-01 | 1 |
| SIM2 | 2.12E-01 | 1 | DENND5B | 2.41E-01 | 1 |
| DIDO1 | 2.12E-01 | 1 | NETO1 | 2.41E-01 | 1 |
| ADRB2 | 2.12E-01 | 1 | CLK4 | 2.41E-01 | 1 |
| A1CF | 2.12E-01 | 1 | SLIT1 | 2.42E-01 | 1 |
| MYO7A | 2.12E-01 | 1 | OTOP1 | 2.42E-01 | 1 |
| TCAP | 2.12E-01 | 1 | FILIP1 | 2.42E-01 | 1 |
| ATXN7L2 | 2.12E-01 | 1 | GRPR | 2.42E-01 | 1 |
| TBC1D12 | 2.12E-01 | 1 | RGS10 | 2.42E-01 | 1 |
| ZSCAN16 | 2.13E-01 | 1 | GNB1 | 2.42E-01 | 1 |
| GPR87 | 2.13E-01 | 1 | RCN2 | 2.42E-01 | 1 |
| FOXN1 | 2.13E-01 | 1 | STARD8 | 2.42E-01 | 1 |
| ZNF7 | 2.13E-01 | 1 | GPR137B | 2.42E-01 | 1 |
| NPR3 | 2.13E-01 | 1 | SLC20A1 | 2.42E-01 | 1 |
| CIB3 | 2.13E-01 | 1 | PLXDC2 | 2.42E-01 | 1 |
| C1orf106 | 2.13E-01 | 1 | NMUR1 | 2.42E-01 | 1 |
| LBR | 2.13E-01 | 1 | ANKZF1 | 2.42E-01 | 1 |
| FAM63A | 2.14E-01 | 1 | ATP2A1 | 2.43E-01 | 1 |
| TUBB | 2.14E-01 | 1 | DNA2 | 2.43E-01 | 1 |
| RBM3 | 2.14E-01 | 1 | KLF9 | 2.43E-01 | 1 |
| TMPRSS3 | 2.14E-01 | 1 | PCDHB16 | 2.43E-01 | 1 |
| POU2F2 | 2.14E-01 | 1 | CTTNBP2 | 2.43E-01 | 1 |
| KRTAP4-1 | 2.14E-01 | 1 | SLC5A11 | 2.43E-01 | 1 |
| LITAF | 2.14E-01 | 1 | CDYL | 2.43E-01 | 1 |
| FIG4 | 2.14E-01 | 1 | FBXO44 | 2.43E-01 | 1 |

| SESN2 | 2.14E-01 | 1 | GUCY1B3 | 2.43E-01 | 1 |
| --- | --- | --- | --- | --- | --- |
| AVPI1 | 2.14E-01 | 1 | C6orf118 | 2.43E-01 | 1 |
| SAA1 | 2.14E-01 | 1 | IL34 | 2.43E-01 | 1 |
| NRARP | 2.15E-01 | 1 | ARHGEF17 | 2.44E-01 | 1 |
| CDC20B | 2.15E-01 | 1 | RGS16 | 2.44E-01 | 1 |
| KCNQ4 | 2.15E-01 | 1 | NBN | 2.44E-01 | 1 |
| FAM65C | 2.15E-01 | 1 | ACTR8 | 2.44E-01 | 1 |
| FYB | 2.15E-01 | 1 | CD1A | 2.44E-01 | 1 |
| GORASP2 | 2.15E-01 | 1 | ARL13B | 2.44E-01 | 1 |
| NASP | 2.15E-01 | 1 | ANKRD54 | 2.45E-01 | 1 |
| DCAF10 | 2.15E-01 | 1 | SBK2 | 2.45E-01 | 1 |
| KRTAP19 | 2.15E-01 | 1 | LOXL4 | 2.45E-01 | 1 |
| CHST15 | 2.15E-01 | 1 | NLGN3 | 2.45E-01 | 1 |
| ASH2L | 2.15E-01 | 1 | UCK2 | 2.45E-01 | 1 |
| GDAP2 | 2.15E-01 | 1 | MTSS1L | 2.45E-01 | 1 |
| CCNF | 2.15E-01 | 1 | ADHFE1 | 2.45E-01 | 1 |
| TMBIM6 | 2.15E-01 | 1 | ATP8A1 | 2.45E-01 | 1 |
| ADAD2 | 2.15E-01 | 1 | RAB43 | 2.46E-01 | 1 |
| LY96 | 2.15E-01 | 1 | ASAP2 | 2.46E-01 | 1 |
| NPAS2 | 2.15E-01 | 1 | NMUR2 | 2.46E-01 | 1 |
| KLF15 | 2.16E-01 | 1 | WDR31 | 2.46E-01 | 1 |
| ACO2 | 2.16E-01 | 1 | CARD11 | 2.46E-01 | 1 |
| GPR161 | 2.16E-01 | 1 | FTSJ2 | 2.47E-01 | 1 |
| OR8D2 | 2.16E-01 | 1 | GPRIN2 | 2.47E-01 | 1 |
| FLOT1 | 2.16E-01 | 1 | ZSCAN4 | 2.47E-01 | 1 |
| ELAVL1 | 2.16E-01 | 1 | C9orf64 | 2.47E-01 | 1 |
| GORAB | 2.16E-01 | 1 | SPACA3 | 2.47E-01 | 1 |
| SLC38A2 | 2.16E-01 | 1 | GPC1 | 2.47E-01 | 1 |
| IL20RA | 2.16E-01 | 1 | DPY19L1 | 2.47E-01 | 1 |
| NHLRC4 | 2.16E-01 | 1 | LAIR1 | 2.47E-01 | 1 |
| MMP3 | 2.16E-01 | 1 | HECTD3 | 2.47E-01 | 1 |
| ERLIN2 | 2.16E-01 | 1 | FANCF | 2.48E-01 | 1 |
| CELF1 | 2.16E-01 | 1 | ALLC | 2.48E-01 | 1 |
| OR51F1 | 2.16E-01 | 1 | H6PD | 2.48E-01 | 1 |
| NKD2 | 2.16E-01 | 1 | NALCN | 2.48E-01 | 1 |
| PPAP2C | 2.16E-01 | 1 | IBSP | 2.48E-01 | 1 |
| PHF23 | 2.16E-01 | 1 | TP73 | 2.48E-01 | 1 |
| NDUFB7 | 2.17E-01 | 1 | MS4A13 | 2.48E-01 | 1 |
| HMCN1 | 2.17E-01 | 1 | CTDSP2 | 2.48E-01 | 1 |
| OR2T27 | 2.17E-01 | 1 | HSDL2 | 2.48E-01 | 1 |
| PI16 | 2.17E-01 | 1 | MAPK1 | 2.48E-01 | 1 |
| TTC13 | 2.17E-01 | 1 | PYGM | 2.48E-01 | 1 |
| MRM1 | 2.17E-01 | 1 | TRMT6 | 2.48E-01 | 1 |
| PPIL1 | 2.17E-01 | 1 | TMEM101 | 2.49E-01 | 1 |
| RXRA | 2.17E-01 | 1 | STAG1 | 2.49E-01 | 1 |
| HK3 | 2.17E-01 | 1 | CATSPERB | 2.49E-01 | 1 |
| ANXA6 | 2.17E-01 | 1 | COPA | 2.49E-01 | 1 |
| SUN5 | 2.17E-01 | 1 | U2AF1L4 | 2.49E-01 | 1 |
| RNF31 | 2.18E-01 | 1 | DLX6 | 2.49E-01 | 1 |
| RSAD2 | 2.18E-01 | 1 | ZNF416 | 2.49E-01 | 1 |
| BDH1 | 2.18E-01 | 1 | CDH1 | 2.50E-01 | 1 |
| SYT14 | 2.18E-01 | 1 | SYNE2 | 2.50E-01 | 1 |
| RBM41 | 2.18E-01 | 1 | PLB1 | 2.50E-01 | 1 |

| NPY1R | 2.18E-01 | 1 | TBC1D14 | 2.50E-01 | 1 |
| --- | --- | --- | --- | --- | --- |
| MRGPRX | 2.18E-01 | 1 | CIZ1 | 2.50E-01 | 1 |
| TRIM64 | 2.18E-01 | 1 | PDE6A | 2.50E-01 | 1 |
| CXCL14 | 2.18E-01 | 1 | TMUB2 | 2.51E-01 | 1 |
| SLC38A7 | 2.18E-01 | 1 | RPS3A | 2.51E-01 | 1 |
| ABO | 2.18E-01 | 1 | OIT3 | 2.51E-01 | 1 |
| RAB11FIP | 2.19E-01 | 1 | ARHGEF7 | 2.51E-01 | 1 |
| BMPER | 2.19E-01 | 1 | ATP1A2 | 2.51E-01 | 1 |
| WBP4 | 2.19E-01 | 1 | C1GALT1C | 2.51E-01 | 1 |
| MEP1B | 2.19E-01 | 1 | RCC1 | 2.52E-01 | 1 |
| METAP2 | 2.19E-01 | 1 | ARMCX1 | 2.52E-01 | 1 |
| VKORC1L | 2.19E-01 | 1 | THAP10 | 2.52E-01 | 1 |
| NECAP1 | 2.19E-01 | 1 | TNKS | 2.52E-01 | 1 |
| OR11G2 | 2.19E-01 | 1 | PARP9 | 2.52E-01 | 1 |
| RB1CC1 | 2.20E-01 | 1 | IGFBP5 | 2.52E-01 | 1 |
| AFAP1L1 | 2.20E-01 | 1 | ZDHHC17 | 2.52E-01 | 1 |
| TRIM28 | 2.20E-01 | 1 | ALKBH7 | 2.52E-01 | 1 |
| OR5B12 | 2.20E-01 | 1 | MRPL19 | 2.52E-01 | 1 |
| SNURF | 2.20E-01 | 1 | SLC22A17 | 2.52E-01 | 1 |
| URM1 | 2.20E-01 | 1 | POP1 | 2.52E-01 | 1 |
| PSMA4 | 2.20E-01 | 1 | MAPK8IP1 | 2.53E-01 | 1 |
| DCLRE1C | 2.20E-01 | 1 | EXD3 | 2.53E-01 | 1 |
| NID1 | 2.20E-01 | 1 | PSMD14 | 2.53E-01 | 1 |
| SLC22A8 | 2.20E-01 | 1 | SGPL1 | 2.53E-01 | 1 |
| DDIT4 | 2.21E-01 | 1 | ZNF680 | 2.53E-01 | 1 |
| FOXRED2 | 2.21E-01 | 1 | CAND2 | 2.53E-01 | 1 |
| C7orf25 | 2.21E-01 | 1 | LRP3 | 2.53E-01 | 1 |
| RSL1D1 | 2.21E-01 | 1 | ATP1B2 | 2.53E-01 | 1 |
| OR1K1 | 2.21E-01 | 1 | AKT3 | 2.53E-01 | 1 |
| OPA1 | 2.21E-01 | 1 | C3orf30 | 2.53E-01 | 1 |
| KIAA1919 | 2.21E-01 | 1 | PKP3 | 2.53E-01 | 1 |
| RPRM | 2.21E-01 | 1 | ZNF143 | 2.53E-01 | 1 |
| TRAPPC6 | 2.21E-01 | 1 | B9D1 | 2.54E-01 | 1 |
| TOX4 | 2.21E-01 | 1 | SPRR4 | 2.54E-01 | 1 |
| DCN | 2.21E-01 | 1 | TACR3 | 2.54E-01 | 1 |
| FOXB2 | 2.21E-01 | 1 | WAS | 2.54E-01 | 1 |
| ELMOD2 | 2.21E-01 | 1 | PLCD1 | 2.54E-01 | 1 |
| SPHKAP | 2.21E-01 | 1 | PIK3CD | 2.54E-01 | 1 |
| EIF4A2 | 2.21E-01 | 1 | HSPA9 | 2.54E-01 | 1 |
| CDC5L | 2.21E-01 | 1 | OPALIN | 2.54E-01 | 1 |
| L3MBTL3 | 2.21E-01 | 1 | ZP1 | 2.54E-01 | 1 |
| PYGO1 | 2.21E-01 | 1 | ZNF197 | 2.54E-01 | 1 |
| GABRA1 | 2.21E-01 | 1 | DHRS4 | 2.55E-01 | 1 |
| CACNG8 | 2.22E-01 | 1 | AP2B1 | 2.55E-01 | 1 |
| ZNF17 | 2.22E-01 | 1 | PSMB11 | 2.55E-01 | 1 |
| BLID | 2.22E-01 | 1 | ABI2 | 2.55E-01 | 1 |
| FAM178B | 2.22E-01 | 1 | NR1I3 | 2.55E-01 | 1 |
| KRT79 | 2.22E-01 | 1 | RPUSD4 | 2.55E-01 | 1 |
| FAM151A | 2.22E-01 | 1 | MINPP1 | 2.55E-01 | 1 |
| OR5D13 | 2.22E-01 | 1 | SUOX | 2.56E-01 | 1 |
| ZNF230 | 2.23E-01 | 1 | POLD1 | 2.56E-01 | 1 |
| VEZF1 | 2.23E-01 | 1 | OR2M7 | 2.56E-01 | 1 |
| ZCCHC10 | 2.23E-01 | 1 | PFKFB3 | 2.56E-01 | 1 |

| HDGFL1 | 2.23E-01 | 1 | HOOK1 | 2.56E-01 | 1 |
| --- | --- | --- | --- | --- | --- |
| CAV1 | 2.23E-01 | 1 | DVL1 | 2.56E-01 | 1 |
| IGLON5 | 2.23E-01 | 1 | COMMD9 | 2.56E-01 | 1 |
| SYF2 | 2.23E-01 | 1 | DNMT3A | 2.56E-01 | 1 |
| IVL | 2.23E-01 | 1 | CPEB3 | 2.56E-01 | 1 |
| CLCN3 | 2.23E-01 | 1 | AHSG | 2.56E-01 | 1 |
| CXCL13 | 2.23E-01 | 1 | UBXN10 | 2.56E-01 | 1 |
| MIIP | 2.23E-01 | 1 | RIBC2 | 2.57E-01 | 1 |
| BVES | 2.23E-01 | 1 | TAS1R2 | 2.57E-01 | 1 |
| HKDC1 | 2.24E-01 | 1 | NDUFA10 | 2.57E-01 | 1 |
| AP1S2 | 2.24E-01 | 1 | CWC22 | 2.57E-01 | 1 |
| CPEB3 | 2.24E-01 | 1 | PTH2R | 2.57E-01 | 1 |
| TMEM161 | 2.24E-01 | 1 | DYNC1I1 | 2.57E-01 | 1 |
| DLL3 | 2.24E-01 | 1 | RSAD2 | 2.57E-01 | 1 |
| SCARB2 | 2.24E-01 | 1 | RTKN2 | 2.57E-01 | 1 |
| ACADL | 2.24E-01 | 1 | EFNA4 | 2.58E-01 | 1 |
| C5orf15 | 2.24E-01 | 1 | SYVN1 | 2.58E-01 | 1 |
| RCSD1 | 2.24E-01 | 1 | PICK1 | 2.58E-01 | 1 |
| CXCL1 | 2.25E-01 | 1 | CD109 | 2.58E-01 | 1 |
| TRPV1 | 2.25E-01 | 1 | INTS4 | 2.58E-01 | 1 |
| CCNE2 | 2.25E-01 | 1 | NAALADL | 2.58E-01 | 1 |
| RHBDL3 | 2.25E-01 | 1 | GRAMD1B | 2.58E-01 | 1 |
| SPTBN1 | 2.25E-01 | 1 | RGL1 | 2.58E-01 | 1 |
| NUDT11 | 2.25E-01 | 1 | SSTR4 | 2.58E-01 | 1 |
| ABCC8 | 2.25E-01 | 1 | WBSCR16 | 2.58E-01 | 1 |
| MANSC1 | 2.25E-01 | 1 | TRIM59 | 2.58E-01 | 1 |
| SAA2 | 2.25E-01 | 1 | POLG2 | 2.58E-01 | 1 |
| OR51F2 | 2.25E-01 | 1 | CDKN3 | 2.58E-01 | 1 |
| RAB14 | 2.25E-01 | 1 | FGFR2 | 2.58E-01 | 1 |
| C16orf58 | 2.25E-01 | 1 | VPS37D | 2.58E-01 | 1 |
| BSPH1 | 2.25E-01 | 1 | SMOC2 | 2.58E-01 | 1 |
| MOS | 2.26E-01 | 1 | ACR | 2.59E-01 | 1 |
| TBC1D14 | 2.26E-01 | 1 | RC3H2 | 2.59E-01 | 1 |
| DNAJC14 | 2.26E-01 | 1 | ITM2C | 2.59E-01 | 1 |
| PFDN5 | 2.26E-01 | 1 | OR8H1 | 2.59E-01 | 1 |
| IPO13 | 2.26E-01 | 1 | SH3BP5 | 2.59E-01 | 1 |
| P4HA2 | 2.26E-01 | 1 | LDB1 | 2.59E-01 | 1 |
| LGR5 | 2.26E-01 | 1 | HOXD9 | 2.59E-01 | 1 |
| ZACN | 2.26E-01 | 1 | STAT1 | 2.59E-01 | 1 |
| RARG | 2.27E-01 | 1 | ZNF821 | 2.60E-01 | 1 |
| TMEM170 | 2.27E-01 | 1 | LRRC45 | 2.60E-01 | 1 |
| MSTN | 2.27E-01 | 1 | SDCBP2 | 2.60E-01 | 1 |
| OR5H6 | 2.27E-01 | 1 | PKN1 | 2.60E-01 | 1 |
| CCDC8 | 2.27E-01 | 1 | SEMA6A | 2.60E-01 | 1 |
| SPINK6 | 2.27E-01 | 1 | IL10RB | 2.60E-01 | 1 |
| INTS3 | 2.27E-01 | 1 | PSG9 | 2.60E-01 | 1 |
| ZDHHC8 | 2.27E-01 | 1 | TTBK1 | 2.60E-01 | 1 |
| PRR18 | 2.27E-01 | 1 | CCAR1 | 2.60E-01 | 1 |
| KCTD18 | 2.27E-01 | 1 | RELL2 | 2.61E-01 | 1 |
| GPATCH2 | 2.27E-01 | 1 | OR1J2 | 2.61E-01 | 1 |
| TNF | 2.27E-01 | 1 | TCEAL4 | 2.61E-01 | 1 |
| AGPAT9 | 2.27E-01 | 1 | TGFBR3 | 2.61E-01 | 1 |
| TAF1B | 2.28E-01 | 1 | NLRP9 | 2.61E-01 | 1 |

| CCDC36 | 2.28E-01 | 1 | OR6F1 | 2.61E-01 | 1 |
| --- | --- | --- | --- | --- | --- |
| TGIF2LX | 2.28E-01 | 1 | SDCCAG3 | 2.61E-01 | 1 |
| OSBPL7 | 2.28E-01 | 1 | CLCA1 | 2.61E-01 | 1 |
| BEST2 | 2.28E-01 | 1 | ST5 | 2.61E-01 | 1 |
| POMC | 2.28E-01 | 1 | ANKRD26 | 2.61E-01 | 1 |
| GALK2 | 2.28E-01 | 1 | ZNF480 | 2.61E-01 | 1 |
| PRAMEF1 | 2.28E-01 | 1 | GGA2 | 2.62E-01 | 1 |
| SEC24B | 2.28E-01 | 1 | FXN | 2.62E-01 | 1 |
| TJP1 | 2.28E-01 | 1 | FETUB | 2.62E-01 | 1 |
| PHKA1 | 2.28E-01 | 1 | KANK4 | 2.63E-01 | 1 |
| OR6C4 | 2.28E-01 | 1 | FNDC8 | 2.63E-01 | 1 |
| SPEG | 2.29E-01 | 1 | ARFIP2 | 2.63E-01 | 1 |
| SPTY2D1 | 2.29E-01 | 1 | WDR74 | 2.63E-01 | 1 |
| ALS2CR1 | 2.29E-01 | 1 | HSP90AB1 | 2.63E-01 | 1 |
| YIF1B | 2.29E-01 | 1 | CLIP4 | 2.64E-01 | 1 |
| MXRA8 | 2.29E-01 | 1 | UTS2R | 2.64E-01 | 1 |
| COPS5 | 2.29E-01 | 1 | SLC25A43 | 2.64E-01 | 1 |
| FAM185A | 2.29E-01 | 1 | OR8A1 | 2.64E-01 | 1 |
| SPAG16 | 2.29E-01 | 1 | OR1J4 | 2.64E-01 | 1 |
| HOXA13 | 2.29E-01 | 1 | CCDC13 | 2.64E-01 | 1 |
| ANKRD34 | 2.29E-01 | 1 | STARD7 | 2.64E-01 | 1 |
| MYO1H | 2.30E-01 | 1 | RNF13 | 2.64E-01 | 1 |
| CAPN8 | 2.30E-01 | 1 | C10orf113 | 2.65E-01 | 1 |
| OR8B8 | 2.30E-01 | 1 | OR3A1 | 2.65E-01 | 1 |
| BTNL9 | 2.30E-01 | 1 | SUFU | 2.65E-01 | 1 |
| EPC2 | 2.30E-01 | 1 | BRD4 | 2.65E-01 | 1 |
| NAB1 | 2.30E-01 | 1 | SIX6 | 2.65E-01 | 1 |
| GM2A | 2.30E-01 | 1 | PHF21A | 2.65E-01 | 1 |
| MAGEE2 | 2.30E-01 | 1 | SCEL | 2.65E-01 | 1 |
| BBS12 | 2.30E-01 | 1 | TGM1 | 2.65E-01 | 1 |
| ATP12A | 2.30E-01 | 1 | FAM3D | 2.66E-01 | 1 |
| SERPINC1 | 2.30E-01 | 1 | TMED9 | 2.66E-01 | 1 |
| HERC2 | 2.30E-01 | 1 | COLEC11 | 2.66E-01 | 1 |
| HIATL1 | 2.30E-01 | 1 | PIWIL4 | 2.66E-01 | 1 |
| SLC39A9 | 2.30E-01 | 1 | NXNL1 | 2.66E-01 | 1 |
| HARS2 | 2.30E-01 | 1 | TMEM82 | 2.66E-01 | 1 |
| OR7G1 | 2.30E-01 | 1 | RSAD1 | 2.66E-01 | 1 |
| REM1 | 2.31E-01 | 1 | SYT7 | 2.66E-01 | 1 |
| FAM166B | 2.31E-01 | 1 | DKKL1 | 2.66E-01 | 1 |
| MPO | 2.31E-01 | 1 | LMOD1 | 2.66E-01 | 1 |
| TFAP2B | 2.31E-01 | 1 | RIMS3 | 2.67E-01 | 1 |
| MRPS5 | 2.31E-01 | 1 | SLC25A17 | 2.67E-01 | 1 |
| SLC22A12 | 2.31E-01 | 1 | RHBDL3 | 2.67E-01 | 1 |
| KIF19 | 2.31E-01 | 1 | ODF3L2 | 2.67E-01 | 1 |
| BCL7B | 2.32E-01 | 1 | ERCC5 | 2.67E-01 | 1 |
| ASPHD1 | 2.32E-01 | 1 | GLB1L | 2.67E-01 | 1 |
| VANGL1 | 2.32E-01 | 1 | ZHX1 | 2.67E-01 | 1 |
| KLHL26 | 2.32E-01 | 1 | CSTF2 | 2.67E-01 | 1 |
| CHRNB3 | 2.32E-01 | 1 | KIAA1217 | 2.67E-01 | 1 |
| HGD | 2.32E-01 | 1 | PGM3 | 2.68E-01 | 1 |
| HEMGN | 2.32E-01 | 1 | ACVR2B | 2.68E-01 | 1 |
| FNBP1 | 2.32E-01 | 1 | CXXC4 | 2.68E-01 | 1 |
| INPP5A | 2.32E-01 | 1 | HCFC1 | 2.68E-01 | 1 |

| ARID3C | 2.32E-01 | 1 | GAD2 | 2.68E-01 | 1 |
| --- | --- | --- | --- | --- | --- |
| SPTA1 | 2.33E-01 | 1 | CHL1 | 2.68E-01 | 1 |
| CST11 | 2.33E-01 | 1 | ZNF774 | 2.68E-01 | 1 |
| GALNT14 | 2.33E-01 | 1 | NDUFB5 | 2.69E-01 | 1 |
| ZSCAN12 | 2.33E-01 | 1 | CUX1 | 2.69E-01 | 1 |
| TFCP2 | 2.33E-01 | 1 | ABCA4 | 2.69E-01 | 1 |
| ACTR5 | 2.33E-01 | 1 | KRTAP5-2 | 2.69E-01 | 1 |
| PCDH18 | 2.33E-01 | 1 | TRPS1 | 2.69E-01 | 1 |
| PLD3 | 2.33E-01 | 1 | CCT5 | 2.69E-01 | 1 |
| SCD | 2.33E-01 | 1 | RHPN2 | 2.70E-01 | 1 |
| CHML | 2.33E-01 | 1 | CLEC12B | 2.70E-01 | 1 |
| WDR63 | 2.33E-01 | 1 | TOX | 2.70E-01 | 1 |
| KRTAP13 | 2.33E-01 | 1 | SH3TC2 | 2.70E-01 | 1 |
| PMVK | 2.33E-01 | 1 | HNF4G | 2.70E-01 | 1 |
| AGA | 2.33E-01 | 1 | TMED1 | 2.70E-01 | 1 |
| AGXT | 2.34E-01 | 1 | ZFP14 | 2.70E-01 | 1 |
| LDLRAD1 | 2.34E-01 | 1 | EPS8L1 | 2.70E-01 | 1 |
| GZMB | 2.34E-01 | 1 | PTCHD2 | 2.70E-01 | 1 |
| ZNF831 | 2.34E-01 | 1 | TMEM132D | 2.71E-01 | 1 |
| SLC2A6 | 2.34E-01 | 1 | TBC1D25 | 2.71E-01 | 1 |
| COX18 | 2.34E-01 | 1 | PDE1A | 2.71E-01 | 1 |
| MPRIP | 2.34E-01 | 1 | GBA2 | 2.71E-01 | 1 |
| SNCA | 2.34E-01 | 1 | TFB1M | 2.71E-01 | 1 |
| OCIAD1 | 2.34E-01 | 1 | ZCCHC14 | 2.71E-01 | 1 |
| GPR12 | 2.34E-01 | 1 | INSIG2 | 2.71E-01 | 1 |
| ITGB5 | 2.34E-01 | 1 | FAHD2B | 2.71E-01 | 1 |
| CMBL | 2.34E-01 | 1 | KIAA1024 | 2.71E-01 | 1 |
| KRTAP4-7 | 2.34E-01 | 1 | PDLIM4 | 2.72E-01 | 1 |
| DPP3 | 2.34E-01 | 1 | C14orf166 | 2.72E-01 | 1 |
| TPPP | 2.34E-01 | 1 | SNRPA1 | 2.72E-01 | 1 |
| CRMP1 | 2.35E-01 | 1 | CELA3B | 2.72E-01 | 1 |
| ANKFY1 | 2.35E-01 | 1 | HMX3 | 2.72E-01 | 1 |
| KRTAP4-1 | 2.35E-01 | 1 | F11R | 2.72E-01 | 1 |
| NUP37 | 2.35E-01 | 1 | TMEM55B | 2.72E-01 | 1 |
| EPHB3 | 2.35E-01 | 1 | ZBTB6 | 2.72E-01 | 1 |
| QRFP | 2.35E-01 | 1 | ARHGAP42 | 2.72E-01 | 1 |
| NR1H2 | 2.35E-01 | 1 | NIPAL4 | 2.72E-01 | 1 |
| DTX2 | 2.35E-01 | 1 | METTL6 | 2.72E-01 | 1 |
| PDHB | 2.35E-01 | 1 | TMEM184A | 2.73E-01 | 1 |
| PRG3 | 2.35E-01 | 1 | LHX5 | 2.73E-01 | 1 |
| ZNF639 | 2.35E-01 | 1 | AIM1 | 2.73E-01 | 1 |
| MRPS24 | 2.36E-01 | 1 | TUBG2 | 2.73E-01 | 1 |
| OVCH1 | 2.36E-01 | 1 | EFHD2 | 2.73E-01 | 1 |
| KRIT1 | 2.36E-01 | 1 | OR2S2 | 2.73E-01 | 1 |
| SERPINA7 | 2.36E-01 | 1 | TYW1B | 2.73E-01 | 1 |
| AKIRIN1 | 2.36E-01 | 1 | PITX1 | 2.73E-01 | 1 |
| GPX1 | 2.36E-01 | 1 | TMEM115 | 2.73E-01 | 1 |
| LCAT | 2.36E-01 | 1 | MTIF2 | 2.73E-01 | 1 |
| KBTBD2 | 2.36E-01 | 1 | OXR1 | 2.73E-01 | 1 |
| RGN | 2.36E-01 | 1 | MFSD2A | 2.74E-01 | 1 |
| THEG | 2.36E-01 | 1 | ADIPOR2 | 2.74E-01 | 1 |
| C14orf177 | 2.36E-01 | 1 | CPT1C | 2.74E-01 | 1 |
| DGKE | 2.36E-01 | 1 | PSD2 | 2.74E-01 | 1 |

| LFNG | 2.37E-01 | 1 | B4GALT6 | 2.74E-01 | 1 |
| --- | --- | --- | --- | --- | --- |
| FAM19A2 | 2.37E-01 | 1 | SYF2 | 2.74E-01 | 1 |
| KCNJ13 | 2.37E-01 | 1 | IDO1 | 2.74E-01 | 1 |
| PSEN2 | 2.37E-01 | 1 | KCNMA1 | 2.74E-01 | 1 |
| ASCC2 | 2.37E-01 | 1 | SDF2 | 2.74E-01 | 1 |
| MLLT1 | 2.38E-01 | 1 | ZNF354B | 2.74E-01 | 1 |
| FGD3 | 2.38E-01 | 1 | DUSP5 | 2.74E-01 | 1 |
| RARA | 2.38E-01 | 1 | SDHAF2 | 2.74E-01 | 1 |
| KCNAB2 | 2.38E-01 | 1 | TARS | 2.75E-01 | 1 |
| RPL4 | 2.38E-01 | 1 | CBX4 | 2.75E-01 | 1 |
| KCNK17 | 2.38E-01 | 1 | SIAH1 | 2.75E-01 | 1 |
| RXRB | 2.38E-01 | 1 | NPSR1 | 2.75E-01 | 1 |
| ZNF780A | 2.38E-01 | 1 | RAD54B | 2.75E-01 | 1 |
| HOXA4 | 2.38E-01 | 1 | ZNF16 | 2.75E-01 | 1 |
| CYBA | 2.38E-01 | 1 | ZC3H4 | 2.75E-01 | 1 |
| CHD1L | 2.38E-01 | 1 | STOML1 | 2.75E-01 | 1 |
| TIMELES | 2.38E-01 | 1 | OR5K2 | 2.75E-01 | 1 |
| EDA | 2.39E-01 | 1 | RHAG | 2.75E-01 | 1 |
| STX5 | 2.39E-01 | 1 | PTTG2 | 2.75E-01 | 1 |
| FAM92B | 2.39E-01 | 1 | ARRDC5 | 2.75E-01 | 1 |
| NEUROD1 | 2.39E-01 | 1 | SYT5 | 2.75E-01 | 1 |
| NMRAL1 | 2.39E-01 | 1 | FOXN1 | 2.75E-01 | 1 |
| MAT1A | 2.39E-01 | 1 | BSPRY | 2.76E-01 | 1 |
| MSGN1 | 2.39E-01 | 1 | THBD | 2.76E-01 | 1 |
| MMGT1 | 2.39E-01 | 1 | DHX9 | 2.76E-01 | 1 |
| C1orf87 | 2.39E-01 | 1 | IPP | 2.76E-01 | 1 |
| SEZ6L | 2.39E-01 | 1 | CRIM1 | 2.76E-01 | 1 |
| RPL5 | 2.39E-01 | 1 | OR1A1 | 2.76E-01 | 1 |
| REEP4 | 2.39E-01 | 1 | SOHLH2 | 2.76E-01 | 1 |
| SEMA4F | 2.39E-01 | 1 | AANAT | 2.76E-01 | 1 |
| TOMM22 | 2.40E-01 | 1 | TMEM98 | 2.76E-01 | 1 |
| TBC1D28 | 2.40E-01 | 1 | WDR90 | 2.76E-01 | 1 |
| FGF16 | 2.40E-01 | 1 | CSH1 | 2.76E-01 | 1 |
| LRRC4 | 2.40E-01 | 1 | AJAP1 | 2.76E-01 | 1 |
| F10 | 2.40E-01 | 1 | ITGA3 | 2.76E-01 | 1 |
| PLA2G4C | 2.40E-01 | 1 | STK19 | 2.76E-01 | 1 |
| AMIGO3 | 2.40E-01 | 1 | IL12A | 2.77E-01 | 1 |
| ORAI3 | 2.40E-01 | 1 | C1orf53 | 2.77E-01 | 1 |
| MYO18A | 2.40E-01 | 1 | CTSK | 2.77E-01 | 1 |
| ASRGL1 | 2.40E-01 | 1 | SFXN5 | 2.77E-01 | 1 |
| TCTN2 | 2.40E-01 | 1 | PARG | 2.77E-01 | 1 |
| DLX4 | 2.40E-01 | 1 | ZCCHC16 | 2.77E-01 | 1 |
| GH2 | 2.40E-01 | 1 | OSGEPL1 | 2.77E-01 | 1 |
| TM7SF2 | 2.41E-01 | 1 | USP48 | 2.77E-01 | 1 |
| SLC25A23 | 2.41E-01 | 1 | NFATC4 | 2.77E-01 | 1 |
| EHD4 | 2.41E-01 | 1 | EPB41L4B | 2.77E-01 | 1 |
| FAM124B | 2.41E-01 | 1 | STXBP6 | 2.78E-01 | 1 |
| SERPINA1 | 2.41E-01 | 1 | SLC25A32 | 2.78E-01 | 1 |
| CDHR2 | 2.41E-01 | 1 | EIF3H | 2.78E-01 | 1 |
| RTP2 | 2.41E-01 | 1 | PCGF2 | 2.78E-01 | 1 |
| CEPT1 | 2.41E-01 | 1 | NPHP4 | 2.78E-01 | 1 |
| MS4A15 | 2.41E-01 | 1 | STEAP2 | 2.78E-01 | 1 |
| CD200R1L | 2.41E-01 | 1 | SUPV3L1 | 2.78E-01 | 1 |

| COL9A2 | 2.41E-01 | 1 | EIF2B5 | 2.78E-01 | 1 |
| --- | --- | --- | --- | --- | --- |
| FANCE | 2.41E-01 | 1 | MKLN1 | 2.78E-01 | 1 |
| NUP214 | 2.41E-01 | 1 | ARMC2 | 2.78E-01 | 1 |
| PLEK2 | 2.41E-01 | 1 | SMC5 | 2.78E-01 | 1 |
| BRSK2 | 2.42E-01 | 1 | CCDC159 | 2.79E-01 | 1 |
| PGLS | 2.42E-01 | 1 | HS3ST1 | 2.79E-01 | 1 |
| SPOCK3 | 2.42E-01 | 1 | ISLR | 2.79E-01 | 1 |
| SCNM1 | 2.42E-01 | 1 | TMEM59 | 2.79E-01 | 1 |
| UCP2 | 2.42E-01 | 1 | ZC3H7B | 2.79E-01 | 1 |
| SERPINB4 | 2.42E-01 | 1 | MYBPHL | 2.79E-01 | 1 |
| WBSCR17 | 2.42E-01 | 1 | KCNK15 | 2.79E-01 | 1 |
| UBE2L3 | 2.42E-01 | 1 | POMC | 2.79E-01 | 1 |
| CLVS2 | 2.42E-01 | 1 | CHRM2 | 2.80E-01 | 1 |
| STK11 | 2.42E-01 | 1 | PRSS3 | 2.80E-01 | 1 |
| CHEK2 | 2.42E-01 | 1 | ZNF703 | 2.80E-01 | 1 |
| HCFC2 | 2.42E-01 | 1 | PTPRR | 2.80E-01 | 1 |
| LRRC25 | 2.42E-01 | 1 | ZBTB45 | 2.80E-01 | 1 |
| BST1 | 2.42E-01 | 1 | REXO1 | 2.80E-01 | 1 |
| STMN2 | 2.42E-01 | 1 | N4BP1 | 2.80E-01 | 1 |
| GSTZ1 | 2.42E-01 | 1 | LATS1 | 2.80E-01 | 1 |
| COL27A1 | 2.42E-01 | 1 | MYOD1 | 2.80E-01 | 1 |
| MS4A4A | 2.42E-01 | 1 | MAN1A2 | 2.81E-01 | 1 |
| CDKN2C | 2.42E-01 | 1 | PFKP | 2.81E-01 | 1 |
| CORO6 | 2.43E-01 | 1 | KRT34 | 2.81E-01 | 1 |
| SIX4 | 2.43E-01 | 1 | LLPH | 2.81E-01 | 1 |
| CERKL | 2.43E-01 | 1 | SORD | 2.81E-01 | 1 |
| UBFD1 | 2.43E-01 | 1 | TEAD3 | 2.81E-01 | 1 |
| OR4K2 | 2.43E-01 | 1 | RHBDL1 | 2.81E-01 | 1 |
| KLC2 | 2.43E-01 | 1 | DNM1L | 2.81E-01 | 1 |
| ZNF740 | 2.43E-01 | 1 | PDGFA | 2.81E-01 | 1 |
| RANBP10 | 2.43E-01 | 1 | MRPS35 | 2.81E-01 | 1 |
| FAM20A | 2.43E-01 | 1 | SLC25A10 | 2.81E-01 | 1 |
| TNFRSF10 | 2.43E-01 | 1 | SH3GL3 | 2.81E-01 | 1 |
| PPP2R5E | 2.43E-01 | 1 | KLK1 | 2.82E-01 | 1 |
| C1orf61 | 2.43E-01 | 1 | SLC25A22 | 2.82E-01 | 1 |
| SMAD5 | 2.43E-01 | 1 | FAM180A | 2.82E-01 | 1 |
| DBNL | 2.44E-01 | 1 | PER1 | 2.82E-01 | 1 |
| ZNF384 | 2.44E-01 | 1 | C15orf43 | 2.82E-01 | 1 |
| OR9I1 | 2.44E-01 | 1 | OR13G1 | 2.82E-01 | 1 |
| TMPRSS1 | 2.44E-01 | 1 | ATP6V0D2 | 2.82E-01 | 1 |
| LRRC14 | 2.44E-01 | 1 | CHMP1B | 2.82E-01 | 1 |
| KYNU | 2.44E-01 | 1 | NR3C1 | 2.82E-01 | 1 |
| PLAC8L1 | 2.44E-01 | 1 | SOX11 | 2.83E-01 | 1 |
| IL6 | 2.44E-01 | 1 | TMEM68 | 2.83E-01 | 1 |
| PRKAA1 | 2.44E-01 | 1 | GALNT6 | 2.83E-01 | 1 |
| CCDC37 | 2.44E-01 | 1 | KLHL33 | 2.83E-01 | 1 |
| PON2 | 2.44E-01 | 1 | EML4 | 2.83E-01 | 1 |
| TDGF1 | 2.44E-01 | 1 | CAPZA1 | 2.83E-01 | 1 |
| FAM171B | 2.44E-01 | 1 | KCNT1 | 2.84E-01 | 1 |
| ZNF280B | 2.45E-01 | 1 | TNRC18 | 2.84E-01 | 1 |
| LARP1 | 2.45E-01 | 1 | COX18 | 2.84E-01 | 1 |
| TMEM207 | 2.45E-01 | 1 | CTLA4 | 2.84E-01 | 1 |
| PCDHGB1 | 2.45E-01 | 1 | SLC25A41 | 2.84E-01 | 1 |

| XPNPEP3 | 2.45E-01 | 1 | RPA2 | 2.84E-01 | 1 |
| --- | --- | --- | --- | --- | --- |
| ADAMDE | 2.45E-01 | 1 | RHOV | 2.84E-01 | 1 |
| USP7 | 2.45E-01 | 1 | OGFOD2 | 2.84E-01 | 1 |
| LTK | 2.45E-01 | 1 | DDX58 | 2.85E-01 | 1 |
| SLC1A1 | 2.45E-01 | 1 | MBD3L2 | 2.85E-01 | 1 |
| EPHX1 | 2.45E-01 | 1 | HOXC12 | 2.85E-01 | 1 |
| PTK2 | 2.45E-01 | 1 | ARGFX | 2.85E-01 | 1 |
| CYP21A2 | 2.45E-01 | 1 | ABTB1 | 2.85E-01 | 1 |
| CCL16 | 2.45E-01 | 1 | IARS | 2.85E-01 | 1 |
| SLC11A1 | 2.45E-01 | 1 | IMPG1 | 2.85E-01 | 1 |
| NKX6-1 | 2.45E-01 | 1 | ELOVL7 | 2.86E-01 | 1 |
| CHRM3 | 2.46E-01 | 1 | CBFB | 2.86E-01 | 1 |
| SHMT2 | 2.46E-01 | 1 | GNB2 | 2.86E-01 | 1 |
| TYRO3 | 2.46E-01 | 1 | DTNB | 2.86E-01 | 1 |
| C3orf36 | 2.46E-01 | 1 | GPR119 | 2.86E-01 | 1 |
| RALA | 2.46E-01 | 1 | TBX4 | 2.86E-01 | 1 |
| TPPP2 | 2.46E-01 | 1 | PWWP2A | 2.86E-01 | 1 |
| KIF22 | 2.46E-01 | 1 | HOXA10 | 2.86E-01 | 1 |
| IRF7 | 2.46E-01 | 1 | TSSK3 | 2.86E-01 | 1 |
| EPB42 | 2.46E-01 | 1 | PRKCB | 2.86E-01 | 1 |
| ARL5B | 2.46E-01 | 1 | NEK6 | 2.86E-01 | 1 |
| CMTM6 | 2.46E-01 | 1 | OVCH1 | 2.86E-01 | 1 |
| CHMP5 | 2.46E-01 | 1 | DCAF4L2 | 2.86E-01 | 1 |
| PCDHGA8 | 2.46E-01 | 1 | ALKBH8 | 2.86E-01 | 1 |
| RHOC | 2.46E-01 | 1 | OR9K2 | 2.86E-01 | 1 |
| DUOXA1 | 2.46E-01 | 1 | AICDA | 2.87E-01 | 1 |
| SNAP29 | 2.46E-01 | 1 | PAPD5 | 2.87E-01 | 1 |
| PDCD2L | 2.46E-01 | 1 | ZNF426 | 2.87E-01 | 1 |
| SPATA20 | 2.46E-01 | 1 | SLC5A10 | 2.87E-01 | 1 |
| USP50 | 2.47E-01 | 1 | SP1 | 2.87E-01 | 1 |
| ZNF436 | 2.47E-01 | 1 | IFNA6 | 2.87E-01 | 1 |
| WISP2 | 2.47E-01 | 1 | PBX1 | 2.87E-01 | 1 |
| DKK3 | 2.47E-01 | 1 | DYSF | 2.87E-01 | 1 |
| PFN4 | 2.47E-01 | 1 | RWDD3 | 2.87E-01 | 1 |
| MYOC | 2.47E-01 | 1 | HEPACAM | 2.87E-01 | 1 |
| HAUS2 | 2.47E-01 | 1 | FAM168A | 2.87E-01 | 1 |
| ERMAP | 2.47E-01 | 1 | SLC36A2 | 2.87E-01 | 1 |
| C1orf100 | 2.47E-01 | 1 | KCNJ10 | 2.87E-01 | 1 |
| NMI | 2.47E-01 | 1 | FAM120A | 2.87E-01 | 1 |
| ACAD8 | 2.47E-01 | 1 | RAB27B | 2.87E-01 | 1 |
| COPZ1 | 2.47E-01 | 1 | PRKG2 | 2.87E-01 | 1 |
| ERP29 | 2.47E-01 | 1 | NMNAT3 | 2.87E-01 | 1 |
| SAMSN1 | 2.47E-01 | 1 | PIH1D2 | 2.88E-01 | 1 |
| AADAT | 2.47E-01 | 1 | PDXK | 2.88E-01 | 1 |
| MICALL2 | 2.47E-01 | 1 | SMPD2 | 2.88E-01 | 1 |
| MRPS35 | 2.47E-01 | 1 | NRSN1 | 2.88E-01 | 1 |
| MECP2 | 2.48E-01 | 1 | PPP5C | 2.88E-01 | 1 |
| CMTM2 | 2.48E-01 | 1 | NEU1 | 2.88E-01 | 1 |
| POTEG | 2.48E-01 | 1 | FOXR2 | 2.88E-01 | 1 |
| SLC25A12 | 2.48E-01 | 1 | TGFBRAP1 | 2.89E-01 | 1 |
| PLEKHN1 | 2.48E-01 | 1 | AHCTF1 | 2.89E-01 | 1 |
| FAM117B | 2.48E-01 | 1 | HLCS | 2.89E-01 | 1 |
| INTS12 | 2.48E-01 | 1 | C1RL | 2.89E-01 | 1 |

| GRB10 | 2.48E-01 | 1 | ZSWIM4 | 2.89E-01 | 1 |
| --- | --- | --- | --- | --- | --- |
| ALDH1A3 | 2.48E-01 | 1 | GLS | 2.89E-01 | 1 |
| NPAS3 | 2.48E-01 | 1 | ZNF831 | 2.90E-01 | 1 |
| SERTAD3 | 2.48E-01 | 1 | KLF11 | 2.90E-01 | 1 |
| SCARF1 | 2.48E-01 | 1 | EHMT2 | 2.90E-01 | 1 |
| OR56B1 | 2.49E-01 | 1 | FAM199X | 2.90E-01 | 1 |
| NT5M | 2.49E-01 | 1 | BRD2 | 2.90E-01 | 1 |
| PCDHGA1 | 2.49E-01 | 1 | KRTAP4-11 | 2.90E-01 | 1 |
| SIRPG | 2.49E-01 | 1 | VENTX | 2.90E-01 | 1 |
| THADA | 2.49E-01 | 1 | MLLT6 | 2.90E-01 | 1 |
| DLST | 2.49E-01 | 1 | RBMS2 | 2.90E-01 | 1 |
| TGM5 | 2.49E-01 | 1 | TUSC1 | 2.90E-01 | 1 |
| NINJ2 | 2.49E-01 | 1 | ATP5B | 2.90E-01 | 1 |
| RPL21 | 2.49E-01 | 1 | GNB4 | 2.91E-01 | 1 |
| MTM1 | 2.49E-01 | 1 | MATN3 | 2.91E-01 | 1 |
| DUSP13 | 2.49E-01 | 1 | IFNA5 | 2.91E-01 | 1 |
| FANCM | 2.49E-01 | 1 | NAGLU | 2.91E-01 | 1 |
| TXNDC17 | 2.50E-01 | 1 | IPO4 | 2.91E-01 | 1 |
| FDX1L | 2.50E-01 | 1 | DYM | 2.91E-01 | 1 |
| OSGIN1 | 2.50E-01 | 1 | CCDC134 | 2.91E-01 | 1 |
| OR1C1 | 2.50E-01 | 1 | SLK | 2.91E-01 | 1 |
| PRSS22 | 2.50E-01 | 1 | ZNF534 | 2.91E-01 | 1 |
| NRTN | 2.50E-01 | 1 | NR2F2 | 2.91E-01 | 1 |
| OR5V1 | 2.50E-01 | 1 | ZNF479 | 2.92E-01 | 1 |
| KCNJ6 | 2.50E-01 | 1 | CD99 | 2.92E-01 | 1 |
| SPINK14 | 2.50E-01 | 1 | SLC22A11 | 2.92E-01 | 1 |
| GPR37 | 2.50E-01 | 1 | CDAN1 | 2.92E-01 | 1 |
| GPRC5C | 2.50E-01 | 1 | CLIP2 | 2.92E-01 | 1 |
| LAT2 | 2.50E-01 | 1 | MC5R | 2.93E-01 | 1 |
| CFL2 | 2.51E-01 | 1 | TNFRSF10 | 2.93E-01 | 1 |
| ZNF576 | 2.51E-01 | 1 | RFX3 | 2.93E-01 | 1 |
| STOML1 | 2.51E-01 | 1 | FBP2 | 2.93E-01 | 1 |
| USP34 | 2.51E-01 | 1 | TAS2R39 | 2.93E-01 | 1 |
| TOX | 2.51E-01 | 1 | NCOR1 | 2.93E-01 | 1 |
| SLC5A1 | 2.51E-01 | 1 | AGPAT2 | 2.93E-01 | 1 |
| TMEM63B | 2.51E-01 | 1 | DUSP7 | 2.93E-01 | 1 |
| IER3 | 2.51E-01 | 1 | KIF4B | 2.93E-01 | 1 |
| PIWIL4 | 2.51E-01 | 1 | NPAT | 2.93E-01 | 1 |
| PRG2 | 2.51E-01 | 1 | TROVE2 | 2.93E-01 | 1 |
| CRYZ | 2.51E-01 | 1 | MKX | 2.93E-01 | 1 |
| NEK3 | 2.52E-01 | 1 | OR2V2 | 2.94E-01 | 1 |
| RTP1 | 2.52E-01 | 1 | NLRC3 | 2.94E-01 | 1 |
| CDH15 | 2.52E-01 | 1 | SLC34A3 | 2.94E-01 | 1 |
| POM121L | 2.52E-01 | 1 | DOK3 | 2.94E-01 | 1 |
| NR5A2 | 2.52E-01 | 1 | TMEM132A | 2.94E-01 | 1 |
| TMEM179 | 2.52E-01 | 1 | LAG3 | 2.94E-01 | 1 |
| DUS3L | 2.52E-01 | 1 | IL9R | 2.94E-01 | 1 |
| HNRNPC | 2.52E-01 | 1 | ZMYND12 | 2.94E-01 | 1 |
| ZNF790 | 2.52E-01 | 1 | SIRPB1 | 2.94E-01 | 1 |
| RNF111 | 2.53E-01 | 1 | FAM196B | 2.94E-01 | 1 |
| PLXDC2 | 2.53E-01 | 1 | KLHL30 | 2.94E-01 | 1 |
| NLE1 | 2.53E-01 | 1 | HLA-A | 2.94E-01 | 1 |
| BEST1 | 2.53E-01 | 1 | KANK1 | 2.95E-01 | 1 |

| PQBP1 | 2.53E-01 | 1 | OR5W2 | 2.95E-01 | 1 |
| --- | --- | --- | --- | --- | --- |
| SEC61A1 | 2.53E-01 | 1 | XG | 2.95E-01 | 1 |
| NXPH2 | 2.53E-01 | 1 | MOCS3 | 2.95E-01 | 1 |
| RTN1 | 2.53E-01 | 1 | GNMT | 2.95E-01 | 1 |
| SLITRK6 | 2.53E-01 | 1 | CSRP3 | 2.95E-01 | 1 |
| GXYLT1 | 2.53E-01 | 1 | SSTR5 | 2.95E-01 | 1 |
| ZC3H12A | 2.53E-01 | 1 | SERPINE1 | 2.95E-01 | 1 |
| ZSWIM2 | 2.53E-01 | 1 | FOLR2 | 2.95E-01 | 1 |
| CTHRC1 | 2.53E-01 | 1 | TBC1D20 | 2.96E-01 | 1 |
| GPRIN1 | 2.54E-01 | 1 | NEUROG1 | 2.96E-01 | 1 |
| SPAG8 | 2.54E-01 | 1 | BLM | 2.96E-01 | 1 |
| RAD21 | 2.54E-01 | 1 | SDCCAG8 | 2.96E-01 | 1 |
| ST7 | 2.54E-01 | 1 | DCAF5 | 2.96E-01 | 1 |
| PI4K2A | 2.54E-01 | 1 | RAB11FIP1 | 2.97E-01 | 1 |
| RNF11 | 2.54E-01 | 1 | GPR45 | 2.97E-01 | 1 |
| ASNS | 2.54E-01 | 1 | ZNF512B | 2.97E-01 | 1 |
| CILP | 2.54E-01 | 1 | PTPRN2 | 2.97E-01 | 1 |
| TMEM138 | 2.54E-01 | 1 | SLAMF7 | 2.97E-01 | 1 |
| TCF4 | 2.55E-01 | 1 | CD207 | 2.97E-01 | 1 |
| C10orf113 | 2.55E-01 | 1 | CEP78 | 2.97E-01 | 1 |
| MAVS | 2.55E-01 | 1 | CNR1 | 2.97E-01 | 1 |
| AP1G2 | 2.55E-01 | 1 | PARM1 | 2.97E-01 | 1 |
| MED24 | 2.55E-01 | 1 | CTNNA1 | 2.98E-01 | 1 |
| RHBDF1 | 2.55E-01 | 1 | UBE3B | 2.98E-01 | 1 |
| UBXN4 | 2.55E-01 | 1 | NR2E1 | 2.98E-01 | 1 |
| GPRC6A | 2.55E-01 | 1 | AAK1 | 2.98E-01 | 1 |
| EDN2 | 2.55E-01 | 1 | OR2D2 | 2.98E-01 | 1 |
| TTYH2 | 2.55E-01 | 1 | CFLAR | 2.98E-01 | 1 |
| TDP2 | 2.56E-01 | 1 | TUSC3 | 2.98E-01 | 1 |
| CTDSPL2 | 2.56E-01 | 1 | HOXA6 | 2.98E-01 | 1 |
| TMEM116 | 2.56E-01 | 1 | TRIM67 | 2.98E-01 | 1 |
| SPSB1 | 2.56E-01 | 1 | ADAM2 | 2.99E-01 | 1 |
| UBXN7 | 2.56E-01 | 1 | C10orf11 | 2.99E-01 | 1 |
| CNDP2 | 2.56E-01 | 1 | CRLS1 | 2.99E-01 | 1 |
| TCP11L2 | 2.56E-01 | 1 | YIF1A | 2.99E-01 | 1 |
| GEMIN6 | 2.56E-01 | 1 | GH2 | 2.99E-01 | 1 |
| CHPF | 2.57E-01 | 1 | SESN1 | 2.99E-01 | 1 |
| SLIT1 | 2.57E-01 | 1 | IGFBPL1 | 2.99E-01 | 1 |
| DNAJB13 | 2.57E-01 | 1 | CPS1 | 2.99E-01 | 1 |
| CYP4F3 | 2.57E-01 | 1 | FIBCD1 | 2.99E-01 | 1 |
| STAT3 | 2.57E-01 | 1 | SCCPDH | 2.99E-01 | 1 |
| FLT4 | 2.57E-01 | 1 | PIK3R3 | 3.00E-01 | 1 |
| ASXL1 | 2.57E-01 | 1 | PRKAR2B | 3.00E-01 | 1 |
| OR5AC2 | 2.57E-01 | 1 | KCNA1 | 3.00E-01 | 1 |
| ZNF185 | 2.57E-01 | 1 | PDCD6 | 3.00E-01 | 1 |
| BNIPL | 2.57E-01 | 1 | SEMA4A | 3.00E-01 | 1 |
| INADL | 2.57E-01 | 1 | EIF2AK4 | 3.00E-01 | 1 |
| DPP10 | 2.57E-01 | 1 | RASSF9 | 3.00E-01 | 1 |
| CDC34 | 2.58E-01 | 1 | WDR76 | 3.00E-01 | 1 |
| LCE5A | 2.58E-01 | 1 | FMR1 | 3.00E-01 | 1 |
| AASDH | 2.58E-01 | 1 | PUF60 | 3.01E-01 | 1 |
| COPS4 | 2.58E-01 | 1 | CCDC92 | 3.01E-01 | 1 |
| CWC25 | 2.58E-01 | 1 | ZNF579 | 3.01E-01 | 1 |

| CYP17A1 | 2.58E-01 | 1 | P2RX1 | 3.01E-01 | 1 |
| --- | --- | --- | --- | --- | --- |
| FUT8 | 2.58E-01 | 1 | ENOPH1 | 3.01E-01 | 1 |
| RAD9A | 2.58E-01 | 1 | ALPP | 3.01E-01 | 1 |
| DENND5B | 2.58E-01 | 1 | HOXC11 | 3.01E-01 | 1 |
| GALNT8 | 2.58E-01 | 1 | PRDM16 | 3.02E-01 | 1 |
| CARD11 | 2.58E-01 | 1 | EGFL7 | 3.02E-01 | 1 |
| SEPSECS | 2.58E-01 | 1 | ZC3H6 | 3.02E-01 | 1 |
| CEACAM | 2.59E-01 | 1 | PCDH15 | 3.02E-01 | 1 |
| ACTL9 | 2.59E-01 | 1 | KCNN4 | 3.02E-01 | 1 |
| CBX3 | 2.59E-01 | 1 | TULP4 | 3.02E-01 | 1 |
| TBL1XR1 | 2.59E-01 | 1 | KCNIP4 | 3.02E-01 | 1 |
| SMC3 | 2.59E-01 | 1 | SCN3B | 3.02E-01 | 1 |
| L1CAM | 2.59E-01 | 1 | CD37 | 3.02E-01 | 1 |
| GPNMB | 2.59E-01 | 1 | PRTG | 3.02E-01 | 1 |
| LRRN3 | 2.59E-01 | 1 | NHLRC3 | 3.02E-01 | 1 |
| NQO1 | 2.59E-01 | 1 | BCL3 | 3.03E-01 | 1 |
| MURC | 2.59E-01 | 1 | FREM2 | 3.03E-01 | 1 |
| ATP10B | 2.59E-01 | 1 | CX3CL1 | 3.03E-01 | 1 |
| HAND1 | 2.59E-01 | 1 | UGT2A3 | 3.03E-01 | 1 |
| RAB3GAP | 2.59E-01 | 1 | EDC4 | 3.03E-01 | 1 |
| SGPP2 | 2.59E-01 | 1 | C8orf86 | 3.03E-01 | 1 |
| SLC5A8 | 2.59E-01 | 1 | ITGB1BP2 | 3.03E-01 | 1 |
| SRPX | 2.59E-01 | 1 | CHRM4 | 3.04E-01 | 1 |
| PBRM1 | 2.60E-01 | 1 | PANX3 | 3.04E-01 | 1 |
| OR2T11 | 2.60E-01 | 1 | COQ6 | 3.04E-01 | 1 |
| SMOX | 2.60E-01 | 1 | SLC35A3 | 3.04E-01 | 1 |
| NDUFAF2 | 2.60E-01 | 1 | ZNF331 | 3.04E-01 | 1 |
| HAS1 | 2.60E-01 | 1 | HMG20A | 3.04E-01 | 1 |
| CRYGS | 2.60E-01 | 1 | KIAA1522 | 3.04E-01 | 1 |
| PRAME | 2.60E-01 | 1 | IL6R | 3.04E-01 | 1 |
| C14orf1 | 2.60E-01 | 1 | EXOC3L2 | 3.05E-01 | 1 |
| ETFDH | 2.60E-01 | 1 | KIF3C | 3.05E-01 | 1 |
| DMRTC2 | 2.60E-01 | 1 | MBD3 | 3.05E-01 | 1 |
| LEP | 2.60E-01 | 1 | ZNF28 | 3.05E-01 | 1 |
| OR1L3 | 2.60E-01 | 1 | KCND2 | 3.05E-01 | 1 |
| SMAGP | 2.61E-01 | 1 | IL1RAPL2 | 3.05E-01 | 1 |
| DSC1 | 2.61E-01 | 1 | CLEC4D | 3.05E-01 | 1 |
| DEFB110 | 2.61E-01 | 1 | DSCR3 | 3.05E-01 | 1 |
| C1orf168 | 2.61E-01 | 1 | PPP2R5B | 3.05E-01 | 1 |
| PRKX | 2.61E-01 | 1 | DDR1 | 3.05E-01 | 1 |
| LSAMP | 2.61E-01 | 1 | RNF115 | 3.05E-01 | 1 |
| GMNN | 2.61E-01 | 1 | HLA-E | 3.05E-01 | 1 |
| INHBA | 2.61E-01 | 1 | PDE4B | 3.06E-01 | 1 |
| OR12D2 | 2.61E-01 | 1 | OR52W1 | 3.06E-01 | 1 |
| AMPH | 2.61E-01 | 1 | GPR15 | 3.06E-01 | 1 |
| RNASE11 | 2.61E-01 | 1 | TTLL10 | 3.06E-01 | 1 |
| RILP | 2.61E-01 | 1 | CLRN3 | 3.06E-01 | 1 |
| CXCR3 | 2.62E-01 | 1 | TPM1 | 3.06E-01 | 1 |
| ZC3H14 | 2.62E-01 | 1 | SLC25A36 | 3.06E-01 | 1 |
| TUBA3E | 2.62E-01 | 1 | FRS3 | 3.06E-01 | 1 |
| PMCH | 2.62E-01 | 1 | WDR7 | 3.07E-01 | 1 |
| LCA5L | 2.62E-01 | 1 | WDR26 | 3.07E-01 | 1 |
| TRIM66 | 2.62E-01 | 1 | C11orf84 | 3.07E-01 | 1 |

| TDRD5 | 2.62E-01 | 1 | OR51A7 | 3.07E-01 | 1 |
| --- | --- | --- | --- | --- | --- |
| GLUL | 2.62E-01 | 1 | ATP6V1E1 | 3.07E-01 | 1 |
| ARF5 | 2.62E-01 | 1 | ZNF696 | 3.07E-01 | 1 |
| XPO6 | 2.62E-01 | 1 | CHPF | 3.07E-01 | 1 |
| LSS | 2.62E-01 | 1 | MYH11 | 3.08E-01 | 1 |
| OR2C3 | 2.62E-01 | 1 | NAGK | 3.08E-01 | 1 |
| AADACL2 | 2.62E-01 | 1 | DNAJC8 | 3.08E-01 | 1 |
| GART | 2.62E-01 | 1 | KIF6 | 3.08E-01 | 1 |
| GLRA1 | 2.62E-01 | 1 | SFXN2 | 3.08E-01 | 1 |
| SMAD3 | 2.62E-01 | 1 | UBE2S | 3.08E-01 | 1 |
| FARSA | 2.62E-01 | 1 | ZNF318 | 3.09E-01 | 1 |
| CCL14 | 2.62E-01 | 1 | HHIP | 3.09E-01 | 1 |
| COL1A1 | 2.62E-01 | 1 | PEPD | 3.09E-01 | 1 |
| LRRC8A | 2.62E-01 | 1 | EGF | 3.09E-01 | 1 |
| CAPZA2 | 2.62E-01 | 1 | SCTR | 3.09E-01 | 1 |
| NID2 | 2.63E-01 | 1 | CDHR1 | 3.10E-01 | 1 |
| ACOX2 | 2.63E-01 | 1 | PRSS42 | 3.10E-01 | 1 |
| KRT17 | 2.63E-01 | 1 | ZNF396 | 3.10E-01 | 1 |
| SPTBN4 | 2.63E-01 | 1 | KTN1 | 3.10E-01 | 1 |
| CPAMD8 | 2.63E-01 | 1 | ENTPD6 | 3.10E-01 | 1 |
| PACRGL | 2.63E-01 | 1 | ZNF430 | 3.10E-01 | 1 |
| DKC1 | 2.63E-01 | 1 | PATL2 | 3.10E-01 | 1 |
| HOXB13 | 2.63E-01 | 1 | GLI4 | 3.10E-01 | 1 |
| CLEC2B | 2.63E-01 | 1 | ZRANB3 | 3.10E-01 | 1 |
| FOXC2 | 2.63E-01 | 1 | HHLA1 | 3.11E-01 | 1 |
| IFITM5 | 2.63E-01 | 1 | MBOAT2 | 3.11E-01 | 1 |
| DMRTA1 | 2.63E-01 | 1 | HSPD1 | 3.11E-01 | 1 |
| RPL10A | 2.64E-01 | 1 | ARL5B | 3.11E-01 | 1 |
| RLN1 | 2.64E-01 | 1 | HOXC4 | 3.11E-01 | 1 |
| DFFB | 2.64E-01 | 1 | ZYG11B | 3.11E-01 | 1 |
| OR11L1 | 2.64E-01 | 1 | GTF3C2 | 3.11E-01 | 1 |
| RAB20 | 2.64E-01 | 1 | GABRG3 | 3.11E-01 | 1 |
| RNF220 | 2.64E-01 | 1 | C2CD2L | 3.12E-01 | 1 |
| CNOT1 | 2.64E-01 | 1 | MPP1 | 3.12E-01 | 1 |
| PCDH19 | 2.64E-01 | 1 | ESRP2 | 3.12E-01 | 1 |
| ZNF219 | 2.64E-01 | 1 | SLC38A7 | 3.12E-01 | 1 |
| CNRIP1 | 2.64E-01 | 1 | EXTL3 | 3.12E-01 | 1 |
| PLA2G2F | 2.64E-01 | 1 | LBR | 3.12E-01 | 1 |
| LMAN1L | 2.64E-01 | 1 | FOXD4L3 | 3.12E-01 | 1 |
| SEC23B | 2.65E-01 | 1 | AQP5 | 3.12E-01 | 1 |
| ANKRD53 | 2.65E-01 | 1 | ADRA2B | 3.12E-01 | 1 |
| IL12A | 2.65E-01 | 1 | PITPNM3 | 3.12E-01 | 1 |
| APOBEC3 | 2.65E-01 | 1 | ADARB2 | 3.12E-01 | 1 |
| FIGNL1 | 2.65E-01 | 1 | UBE2Q1 | 3.12E-01 | 1 |
| PPP2R4 | 2.65E-01 | 1 | BBS9 | 3.12E-01 | 1 |
| HNF1B | 2.65E-01 | 1 | ARVCF | 3.12E-01 | 1 |
| MTDH | 2.65E-01 | 1 | RBPJL | 3.13E-01 | 1 |
| AMPD1 | 2.65E-01 | 1 | CC2D1B | 3.13E-01 | 1 |
| AGFG2 | 2.66E-01 | 1 | CTNNAL1 | 3.13E-01 | 1 |
| TRIM65 | 2.66E-01 | 1 | TIMP3 | 3.13E-01 | 1 |
| MBD1 | 2.66E-01 | 1 | CAMK2G | 3.13E-01 | 1 |
| CYB561 | 2.66E-01 | 1 | CD22 | 3.13E-01 | 1 |
| ADAM30 | 2.66E-01 | 1 | MAML2 | 3.13E-01 | 1 |

| MPP6 | 2.66E-01 | 1 | PRDM5 | 3.13E-01 | 1 |
| --- | --- | --- | --- | --- | --- |
| IL1RAPL2 | 2.66E-01 | 1 | TFCP2 | 3.13E-01 | 1 |
| AAMP | 2.66E-01 | 1 | ARRDC3 | 3.14E-01 | 1 |
| USP6NL | 2.66E-01 | 1 | KCNG4 | 3.14E-01 | 1 |
| SPCS3 | 2.66E-01 | 1 | APEX1 | 3.14E-01 | 1 |
| COQ7 | 2.66E-01 | 1 | RCN3 | 3.14E-01 | 1 |
| PFKFB2 | 2.66E-01 | 1 | NRD1 | 3.14E-01 | 1 |
| FAM192A | 2.66E-01 | 1 | ZNF645 | 3.14E-01 | 1 |
| GRPEL2 | 2.66E-01 | 1 | PTRF | 3.14E-01 | 1 |
| DLK2 | 2.66E-01 | 1 | TFPT | 3.15E-01 | 1 |
| TUBB1 | 2.67E-01 | 1 | KRT83 | 3.15E-01 | 1 |
| TPTE | 2.67E-01 | 1 | TEDDM1 | 3.15E-01 | 1 |
| SEMA6D | 2.67E-01 | 1 | PSMB9 | 3.15E-01 | 1 |
| PIGM | 2.67E-01 | 1 | LMBR1L | 3.15E-01 | 1 |
| PLA2G15 | 2.67E-01 | 1 | TPK1 | 3.15E-01 | 1 |
| POSTN | 2.67E-01 | 1 | LRG1 | 3.15E-01 | 1 |
| SSX1 | 2.67E-01 | 1 | ESRRA | 3.16E-01 | 1 |
| RAX | 2.67E-01 | 1 | C12orf42 | 3.16E-01 | 1 |
| SRRM5 | 2.67E-01 | 1 | FBXO32 | 3.16E-01 | 1 |
| ZNF496 | 2.67E-01 | 1 | 8-Mar | 3.16E-01 | 1 |
| ITIH1 | 2.68E-01 | 1 | KBTBD4 | 3.16E-01 | 1 |
| PMP2 | 2.68E-01 | 1 | HNRNPUL1 | 3.16E-01 | 1 |
| RFWD3 | 2.68E-01 | 1 | TUBGCP6 | 3.16E-01 | 1 |
| RAP1A | 2.68E-01 | 1 | OR10Z1 | 3.16E-01 | 1 |
| ALLC | 2.68E-01 | 1 | PTPRQ | 3.16E-01 | 1 |
| INA | 2.68E-01 | 1 | GLI3 | 3.16E-01 | 1 |
| RAB1A | 2.68E-01 | 1 | FZD10 | 3.16E-01 | 1 |
| ZDHHC22 | 2.68E-01 | 1 | STK25 | 3.17E-01 | 1 |
| IPP | 2.68E-01 | 1 | DHX15 | 3.17E-01 | 1 |
| KCNA2 | 2.68E-01 | 1 | KLF8 | 3.17E-01 | 1 |
| ACSBG1 | 2.68E-01 | 1 | PGC | 3.17E-01 | 1 |
| LRRC36 | 2.68E-01 | 1 | ZNF619 | 3.17E-01 | 1 |
| TBC1D21 | 2.68E-01 | 1 | SLC30A6 | 3.17E-01 | 1 |
| RBBP5 | 2.68E-01 | 1 | CISH | 3.17E-01 | 1 |
| BBS2 | 2.68E-01 | 1 | CRLF3 | 3.18E-01 | 1 |
| MS4A10 | 2.68E-01 | 1 | NCK1 | 3.18E-01 | 1 |
| HCN1 | 2.68E-01 | 1 | PAN3 | 3.18E-01 | 1 |
| CACNA1B | 2.68E-01 | 1 | CYP27C1 | 3.18E-01 | 1 |
| MARK4 | 2.69E-01 | 1 | CDC7 | 3.18E-01 | 1 |
| PPP1R3F | 2.69E-01 | 1 | CRTC2 | 3.19E-01 | 1 |
| OR56A3 | 2.69E-01 | 1 | OXGR1 | 3.19E-01 | 1 |
| CUL1 | 2.69E-01 | 1 | GABRR2 | 3.19E-01 | 1 |
| SLC30A9 | 2.69E-01 | 1 | UBQLNL | 3.19E-01 | 1 |
| BLMH | 2.69E-01 | 1 | HGD | 3.19E-01 | 1 |
| PGLYRP4 | 2.69E-01 | 1 | UBLCP1 | 3.19E-01 | 1 |
| A4GALT | 2.69E-01 | 1 | ZSCAN2 | 3.20E-01 | 1 |
| ARHGEF1 | 2.69E-01 | 1 | MDFIC | 3.20E-01 | 1 |
| ASTL | 2.69E-01 | 1 | CBLN1 | 3.20E-01 | 1 |
| IGDCC3 | 2.69E-01 | 1 | KIRREL | 3.20E-01 | 1 |
| EFCAB7 | 2.69E-01 | 1 | KDM3B | 3.20E-01 | 1 |
| KPRP | 2.69E-01 | 1 | NT5C1B | 3.20E-01 | 1 |
| UBE2E2 | 2.69E-01 | 1 | FAM151B | 3.21E-01 | 1 |
| QPCTL | 2.69E-01 | 1 | CADM2 | 3.21E-01 | 1 |

| SLC27A6 | 2.69E-01 | 1 | ADSL | 3.21E-01 | 1 |
| --- | --- | --- | --- | --- | --- |
| OR4K1 | 2.70E-01 | 1 | ALDH3A1 | 3.21E-01 | 1 |
| GLIPR1 | 2.70E-01 | 1 | ZNF99 | 3.21E-01 | 1 |
| MYO6 | 2.70E-01 | 1 | EOMES | 3.21E-01 | 1 |
| MPHOSPH | 2.70E-01 | 1 | CPA1 | 3.21E-01 | 1 |
| PPP1R10 | 2.70E-01 | 1 | NTSR2 | 3.21E-01 | 1 |
| EPHB1 | 2.70E-01 | 1 | OR2J3 | 3.22E-01 | 1 |
| TMEM87A | 2.70E-01 | 1 | CHMP4C | 3.22E-01 | 1 |
| PTPRM | 2.70E-01 | 1 | EPHB1 | 3.22E-01 | 1 |
| MCF2L | 2.70E-01 | 1 | ADORA2B | 3.22E-01 | 1 |
| C8orf86 | 2.70E-01 | 1 | NPAS2 | 3.22E-01 | 1 |
| TRPV6 | 2.70E-01 | 1 | OR6X1 | 3.22E-01 | 1 |
| SNAPC3 | 2.70E-01 | 1 | RECQL4 | 3.22E-01 | 1 |
| BCAN | 2.70E-01 | 1 | BLNK | 3.22E-01 | 1 |
| CD5 | 2.71E-01 | 1 | VSIG2 | 3.22E-01 | 1 |
| TTC23L | 2.71E-01 | 1 | NPL | 3.22E-01 | 1 |
| ARL6IP5 | 2.71E-01 | 1 | LDOC1 | 3.23E-01 | 1 |
| COX4I2 | 2.71E-01 | 1 | UBA1 | 3.23E-01 | 1 |
| POLK | 2.71E-01 | 1 | GABBR2 | 3.23E-01 | 1 |
| P2RY1 | 2.71E-01 | 1 | GZMK | 3.23E-01 | 1 |
| RPS6KB1 | 2.71E-01 | 1 | THAP7 | 3.23E-01 | 1 |
| TMEM174 | 2.71E-01 | 1 | E2F1 | 3.23E-01 | 1 |
| ARHGAP2 | 2.71E-01 | 1 | PSME3 | 3.23E-01 | 1 |
| CLDN20 | 2.71E-01 | 1 | LRRC3B | 3.23E-01 | 1 |
| FAM58A | 2.71E-01 | 1 | INPP5A | 3.23E-01 | 1 |
| KRT6A | 2.72E-01 | 1 | PTGER3 | 3.23E-01 | 1 |
| UPF3A | 2.72E-01 | 1 | CARS2 | 3.23E-01 | 1 |
| STXBP2 | 2.72E-01 | 1 | APOOL | 3.23E-01 | 1 |
| SULF1 | 2.72E-01 | 1 | UBE2J2 | 3.24E-01 | 1 |
| MTF2 | 2.72E-01 | 1 | KLB | 3.24E-01 | 1 |
| MOSPD3 | 2.72E-01 | 1 | INPP4B | 3.24E-01 | 1 |
| ACTL6B | 2.72E-01 | 1 | ACAA1 | 3.24E-01 | 1 |
| PDE1B | 2.72E-01 | 1 | UBXN4 | 3.24E-01 | 1 |
| OR4N2 | 2.72E-01 | 1 | ZFP36 | 3.24E-01 | 1 |
| OR51G1 | 2.72E-01 | 1 | PPP2R2C | 3.24E-01 | 1 |
| ANO5 | 2.72E-01 | 1 | RAI2 | 3.24E-01 | 1 |
| WEE1 | 2.72E-01 | 1 | AKAP4 | 3.25E-01 | 1 |
| CIITA | 2.73E-01 | 1 | BCAS3 | 3.25E-01 | 1 |
| PRICKLE2 | 2.73E-01 | 1 | SLC16A1 | 3.25E-01 | 1 |
| B4GALT3 | 2.73E-01 | 1 | C2orf71 | 3.25E-01 | 1 |
| TRH | 2.73E-01 | 1 | SERPINH1 | 3.25E-01 | 1 |
| TUB | 2.73E-01 | 1 | ATP6AP1 | 3.26E-01 | 1 |
| SMPDL3A | 2.73E-01 | 1 | MAGIX | 3.26E-01 | 1 |
| TUBA3D | 2.73E-01 | 1 | GJC1 | 3.26E-01 | 1 |
| CASP10 | 2.73E-01 | 1 | BTBD11 | 3.26E-01 | 1 |
| CEP97 | 2.73E-01 | 1 | LTBP4 | 3.26E-01 | 1 |
| FAM168B | 2.73E-01 | 1 | RBM22 | 3.26E-01 | 1 |
| CD86 | 2.74E-01 | 1 | NOTO | 3.26E-01 | 1 |
| NTM | 2.74E-01 | 1 | ITGB6 | 3.26E-01 | 1 |
| ZAP70 | 2.74E-01 | 1 | MYPOP | 3.26E-01 | 1 |
| ECSIT | 2.74E-01 | 1 | MKRN1 | 3.26E-01 | 1 |
| RGMB | 2.74E-01 | 1 | ZNF385B | 3.26E-01 | 1 |
| TNIP1 | 2.74E-01 | 1 | EIF4H | 3.27E-01 | 1 |

| APBB1IP | 2.74E-01 | 1 | CRTC1 | 3.27E-01 | 1 |
| --- | --- | --- | --- | --- | --- |
| AFF1 | 2.74E-01 | 1 | TBPL2 | 3.27E-01 | 1 |
| ESRP2 | 2.74E-01 | 1 | DAO | 3.27E-01 | 1 |
| SPATA12 | 2.74E-01 | 1 | EIF4G1 | 3.27E-01 | 1 |
| ABCA7 | 2.74E-01 | 1 | PCDHB1 | 3.27E-01 | 1 |
| DAO | 2.75E-01 | 1 | PSG3 | 3.27E-01 | 1 |
| MAN2A2 | 2.75E-01 | 1 | ACAD11 | 3.27E-01 | 1 |
| PCDHB3 | 2.75E-01 | 1 | PLEKHM1 | 3.27E-01 | 1 |
| RND1 | 2.75E-01 | 1 | ADD1 | 3.27E-01 | 1 |
| FBXL18 | 2.75E-01 | 1 | MYO1E | 3.27E-01 | 1 |
| UNG | 2.75E-01 | 1 | ACOT12 | 3.27E-01 | 1 |
| CCDC155 | 2.75E-01 | 1 | TC2N | 3.28E-01 | 1 |
| DNAJC4 | 2.75E-01 | 1 | FAM53C | 3.28E-01 | 1 |
| PRDM11 | 2.75E-01 | 1 | PDGFC | 3.28E-01 | 1 |
| CTRL | 2.75E-01 | 1 | DDR2 | 3.28E-01 | 1 |
| PTPRN | 2.75E-01 | 1 | ABHD8 | 3.28E-01 | 1 |
| ARHGEF1 | 2.75E-01 | 1 | PSMC5 | 3.29E-01 | 1 |
| PPP1R12B | 2.76E-01 | 1 | FBXO18 | 3.29E-01 | 1 |
| DCX | 2.76E-01 | 1 | PRSS50 | 3.29E-01 | 1 |
| CACNA1S | 2.76E-01 | 1 | PRR5-ARH | 3.29E-01 | 1 |
| STX1B | 2.76E-01 | 1 | DPP9 | 3.29E-01 | 1 |
| TEKT3 | 2.76E-01 | 1 | PSG8 | 3.29E-01 | 1 |
| RUFY4 | 2.76E-01 | 1 | PADI2 | 3.29E-01 | 1 |
| C3orf30 | 2.76E-01 | 1 | MDH2 | 3.29E-01 | 1 |
| OR8K3 | 2.76E-01 | 1 | SIGLEC8 | 3.29E-01 | 1 |
| NXN | 2.76E-01 | 1 | TMEM39B | 3.29E-01 | 1 |
| NFATC2 | 2.76E-01 | 1 | SLC10A3 | 3.29E-01 | 1 |
| UST | 2.76E-01 | 1 | CES1 | 3.29E-01 | 1 |
| MPDU1 | 2.76E-01 | 1 | AMMECR1 | 3.29E-01 | 1 |
| ACSM5 | 2.76E-01 | 1 | RCSD1 | 3.30E-01 | 1 |
| KATNA1 | 2.76E-01 | 1 | R3HDM1 | 3.30E-01 | 1 |
| ATP8B3 | 2.77E-01 | 1 | CDK3 | 3.30E-01 | 1 |
| TSEN54 | 2.77E-01 | 1 | TMEM184B | 3.30E-01 | 1 |
| FOSL2 | 2.77E-01 | 1 | TAF10 | 3.30E-01 | 1 |
| NR4A1 | 2.77E-01 | 1 | MACROD1 | 3.30E-01 | 1 |
| EPC1 | 2.77E-01 | 1 | PIP4K2B | 3.30E-01 | 1 |
| LRRC56 | 2.77E-01 | 1 | PNPO | 3.30E-01 | 1 |
| C1orf174 | 2.77E-01 | 1 | SGK3 | 3.30E-01 | 1 |
| HIST1H3B | 2.77E-01 | 1 | HYAL3 | 3.30E-01 | 1 |
| EFCAB5 | 2.77E-01 | 1 | WDR63 | 3.31E-01 | 1 |
| FGG | 2.77E-01 | 1 | OR51T1 | 3.31E-01 | 1 |
| PIN1 | 2.77E-01 | 1 | DNAH7 | 3.31E-01 | 1 |
| ACVRL1 | 2.77E-01 | 1 | TBC1D2B | 3.31E-01 | 1 |
| OR1A2 | 2.77E-01 | 1 | TAOK1 | 3.31E-01 | 1 |
| SNTA1 | 2.77E-01 | 1 | SLC26A1 | 3.31E-01 | 1 |
| ORM1 | 2.77E-01 | 1 | TRIM54 | 3.31E-01 | 1 |
| ERF | 2.78E-01 | 1 | EPC1 | 3.31E-01 | 1 |
| CCR4 | 2.78E-01 | 1 | PEX6 | 3.31E-01 | 1 |
| NUDT18 | 2.78E-01 | 1 | NLRC5 | 3.32E-01 | 1 |
| IRF4 | 2.78E-01 | 1 | CCNT1 | 3.32E-01 | 1 |
| HHLA1 | 2.78E-01 | 1 | VAX1 | 3.32E-01 | 1 |
| INTS7 | 2.78E-01 | 1 | TRIM66 | 3.32E-01 | 1 |
| LIN28A | 2.79E-01 | 1 | KRTAP5-5 | 3.32E-01 | 1 |

| RLIM | 2.79E-01 | 1 | DNAJB5 | 3.32E-01 | 1 |
| --- | --- | --- | --- | --- | --- |
| DIS3L2 | 2.79E-01 | 1 | CABP4 | 3.32E-01 | 1 |
| HIBCH | 2.79E-01 | 1 | RFC5 | 3.32E-01 | 1 |
| CASC1 | 2.79E-01 | 1 | LGALS8 | 3.33E-01 | 1 |
| ZNF708 | 2.79E-01 | 1 | USH1C | 3.33E-01 | 1 |
| STXBP5L | 2.79E-01 | 1 | CSPG5 | 3.33E-01 | 1 |
| CDKN2AI | 2.79E-01 | 1 | LRRC15 | 3.33E-01 | 1 |
| SEL1L | 2.79E-01 | 1 | U2AF2 | 3.33E-01 | 1 |
| FZD7 | 2.79E-01 | 1 | C1GALT1 | 3.33E-01 | 1 |
| C14orf159 | 2.79E-01 | 1 | OR6B2 | 3.33E-01 | 1 |
| HMHA1 | 2.80E-01 | 1 | SF3A1 | 3.33E-01 | 1 |
| CD27 | 2.80E-01 | 1 | RAPGEF5 | 3.33E-01 | 1 |
| LIPC | 2.80E-01 | 1 | MCM10 | 3.33E-01 | 1 |
| RFXAP | 2.80E-01 | 1 | LAT2 | 3.33E-01 | 1 |
| RNASE10 | 2.80E-01 | 1 | IL1R1 | 3.34E-01 | 1 |
| MEN1 | 2.80E-01 | 1 | TLR9 | 3.34E-01 | 1 |
| NAT6 | 2.80E-01 | 1 | PLVAP | 3.34E-01 | 1 |
| TRIM60 | 2.80E-01 | 1 | ZC3H13 | 3.34E-01 | 1 |
| NCBP1 | 2.81E-01 | 1 | PGBD2 | 3.34E-01 | 1 |
| CENPV | 2.81E-01 | 1 | VIT | 3.34E-01 | 1 |
| DCXR | 2.81E-01 | 1 | METTL2A | 3.34E-01 | 1 |
| ZNF223 | 2.81E-01 | 1 | SRD5A2 | 3.34E-01 | 1 |
| ANAPC5 | 2.81E-01 | 1 | SLC16A14 | 3.34E-01 | 1 |
| P2RX1 | 2.81E-01 | 1 | NAT10 | 3.34E-01 | 1 |
| IMPA2 | 2.81E-01 | 1 | LHX6 | 3.34E-01 | 1 |
| IL31RA | 2.81E-01 | 1 | UBE2J1 | 3.34E-01 | 1 |
| FRRS1 | 2.81E-01 | 1 | CPNE5 | 3.35E-01 | 1 |
| DMKN | 2.81E-01 | 1 | PCNXL3 | 3.35E-01 | 1 |
| MRTO4 | 2.81E-01 | 1 | SLC5A1 | 3.35E-01 | 1 |
| CELA3B | 2.81E-01 | 1 | UBQLN2 | 3.35E-01 | 1 |
| ATG9B | 2.81E-01 | 1 | NCOA3 | 3.35E-01 | 1 |
| MYOM1 | 2.81E-01 | 1 | ZNF410 | 3.35E-01 | 1 |
| DDB1 | 2.81E-01 | 1 | PAX6 | 3.35E-01 | 1 |
| DNAJC16 | 2.81E-01 | 1 | SNRPN | 3.35E-01 | 1 |
| TP73 | 2.82E-01 | 1 | PGK2 | 3.36E-01 | 1 |
| SLC6A1 | 2.82E-01 | 1 | CLDN18 | 3.36E-01 | 1 |
| SF1 | 2.82E-01 | 1 | PRL | 3.36E-01 | 1 |
| OVOL1 | 2.82E-01 | 1 | CHAC1 | 3.36E-01 | 1 |
| CARKD | 2.82E-01 | 1 | SCAPER | 3.36E-01 | 1 |
| AGTRAP | 2.82E-01 | 1 | ADIPOR1 | 3.36E-01 | 1 |
| RBMXL3 | 2.82E-01 | 1 | EPM2AIP1 | 3.36E-01 | 1 |
| ZNF746 | 2.82E-01 | 1 | EDIL3 | 3.36E-01 | 1 |
| POLR1D | 2.82E-01 | 1 | WDR18 | 3.36E-01 | 1 |
| GABRA2 | 2.83E-01 | 1 | OR7A17 | 3.36E-01 | 1 |
| IDUA | 2.83E-01 | 1 | RBMXL2 | 3.37E-01 | 1 |
| MYT1L | 2.83E-01 | 1 | ENPP3 | 3.37E-01 | 1 |
| DDIT4L | 2.83E-01 | 1 | OR4A47 | 3.37E-01 | 1 |
| MCM10 | 2.83E-01 | 1 | C6orf141 | 3.37E-01 | 1 |
| CD300LB | 2.84E-01 | 1 | ZSWIM3 | 3.37E-01 | 1 |
| TTC7A | 2.84E-01 | 1 | SLC4A10 | 3.37E-01 | 1 |
| PDCD6 | 2.84E-01 | 1 | S1PR5 | 3.37E-01 | 1 |
| CCL8 | 2.84E-01 | 1 | OLIG2 | 3.37E-01 | 1 |
| RNMT | 2.84E-01 | 1 | ZMIZ1 | 3.37E-01 | 1 |

| SAP130 | 2.84E-01 | 1 | HYAL1 | 3.37E-01 | 1 |
| --- | --- | --- | --- | --- | --- |
| PNPLA5 | 2.84E-01 | 1 | LRRC8A | 3.37E-01 | 1 |
| PDF | 2.84E-01 | 1 | MAPK10 | 3.38E-01 | 1 |
| B3GAT2 | 2.85E-01 | 1 | CACNG7 | 3.38E-01 | 1 |
| PPIG | 2.85E-01 | 1 | MBD5 | 3.38E-01 | 1 |
| TCHHL1 | 2.85E-01 | 1 | CEP350 | 3.38E-01 | 1 |
| RPL10 | 2.85E-01 | 1 | SQLE | 3.38E-01 | 1 |
| TRPV5 | 2.85E-01 | 1 | PLBD2 | 3.38E-01 | 1 |
| DUSP15 | 2.85E-01 | 1 | CCR9 | 3.39E-01 | 1 |
| IL17RC | 2.85E-01 | 1 | TAS2R60 | 3.39E-01 | 1 |
| GABPB2 | 2.85E-01 | 1 | AKNA | 3.39E-01 | 1 |
| NIT2 | 2.85E-01 | 1 | TMPRSS6 | 3.39E-01 | 1 |
| USP17L2 | 2.85E-01 | 1 | CRMP1 | 3.39E-01 | 1 |
| MAD2L2 | 2.85E-01 | 1 | ATP9B | 3.39E-01 | 1 |
| MPV17L | 2.86E-01 | 1 | DUSP8 | 3.39E-01 | 1 |
| MRPL10 | 2.86E-01 | 1 | CHRNB2 | 3.39E-01 | 1 |
| NBPF15 | 2.86E-01 | 1 | NUP155 | 3.39E-01 | 1 |
| PRDX3 | 2.86E-01 | 1 | TMTC2 | 3.39E-01 | 1 |
| GLUD2 | 2.86E-01 | 1 | PKN3 | 3.39E-01 | 1 |
| ARID1B | 2.86E-01 | 1 | RPP30 | 3.40E-01 | 1 |
| SCFD2 | 2.86E-01 | 1 | GPRASP1 | 3.40E-01 | 1 |
| CELA3A | 2.86E-01 | 1 | GPR35 | 3.40E-01 | 1 |
| CYP2W1 | 2.86E-01 | 1 | DDX31 | 3.40E-01 | 1 |
| FBXL7 | 2.86E-01 | 1 | DAAM2 | 3.40E-01 | 1 |
| ZCRB1 | 2.86E-01 | 1 | BNIP2 | 3.40E-01 | 1 |
| ZFYVE27 | 2.87E-01 | 1 | TMEM196 | 3.40E-01 | 1 |
| NCOA7 | 2.87E-01 | 1 | PPFIBP2 | 3.40E-01 | 1 |
| OR6T1 | 2.87E-01 | 1 | PPM1E | 3.40E-01 | 1 |
| SUPT4H1 | 2.87E-01 | 1 | CDX4 | 3.41E-01 | 1 |
| ACBD6 | 2.87E-01 | 1 | MATN4 | 3.41E-01 | 1 |
| RAD54B | 2.87E-01 | 1 | KRT85 | 3.41E-01 | 1 |
| TOP3A | 2.87E-01 | 1 | KLHL29 | 3.41E-01 | 1 |
| ADAMTS | 2.87E-01 | 1 | OR6M1 | 3.41E-01 | 1 |
| GRM3 | 2.87E-01 | 1 | PIGM | 3.41E-01 | 1 |
| GRB14 | 2.87E-01 | 1 | BIRC8 | 3.41E-01 | 1 |
| PRUNE2 | 2.87E-01 | 1 | ATR | 3.41E-01 | 1 |
| RPL18A | 2.87E-01 | 1 | OR6N2 | 3.42E-01 | 1 |
| C17orf64 | 2.87E-01 | 1 | LRRC1 | 3.42E-01 | 1 |
| GSTM3 | 2.87E-01 | 1 | SETD2 | 3.42E-01 | 1 |
| GABBR1 | 2.87E-01 | 1 | TRMU | 3.42E-01 | 1 |
| KIAA1045 | 2.87E-01 | 1 | WNT6 | 3.42E-01 | 1 |
| KLF9 | 2.88E-01 | 1 | EHD1 | 3.42E-01 | 1 |
| DHCR24 | 2.88E-01 | 1 | C6orf58 | 3.42E-01 | 1 |
| MSX1 | 2.88E-01 | 1 | UBE2Q2 | 3.42E-01 | 1 |
| ZEB1 | 2.88E-01 | 1 | SERPINB6 | 3.42E-01 | 1 |
| C1orf43 | 2.88E-01 | 1 | OLFM1 | 3.42E-01 | 1 |
| ATP6V1E | 2.88E-01 | 1 | FLT1 | 3.42E-01 | 1 |
| PEPD | 2.88E-01 | 1 | CLCN5 | 3.42E-01 | 1 |
| PRTFDC1 | 2.88E-01 | 1 | OGDHL | 3.42E-01 | 1 |
| ATF6B | 2.88E-01 | 1 | CCDC60 | 3.43E-01 | 1 |
| BDKRB2 | 2.88E-01 | 1 | ZDHHC12 | 3.43E-01 | 1 |
| KIAA1598 | 2.88E-01 | 1 | RNF2 | 3.43E-01 | 1 |
| PRSS45 | 2.88E-01 | 1 | SHE | 3.43E-01 | 1 |

| AMHR2 | 2.88E-01 | 1 | CYB5R3 | 3.43E-01 | 1 |
| --- | --- | --- | --- | --- | --- |
| NCDN | 2.88E-01 | 1 | SPATA9 | 3.43E-01 | 1 |
| PRUNE | 2.89E-01 | 1 | ZNF248 | 3.43E-01 | 1 |
| NCALD | 2.89E-01 | 1 | TCTN2 | 3.43E-01 | 1 |
| ZNF394 | 2.89E-01 | 1 | TGOLN2 | 3.43E-01 | 1 |
| C14orf93 | 2.89E-01 | 1 | CALR | 3.43E-01 | 1 |
| ZDHHC14 | 2.89E-01 | 1 | TEKT2 | 3.43E-01 | 1 |
| CDYL | 2.89E-01 | 1 | CNOT2 | 3.44E-01 | 1 |
| SH3TC2 | 2.89E-01 | 1 | WDR55 | 3.44E-01 | 1 |
| RASSF9 | 2.89E-01 | 1 | C18orf54 | 3.44E-01 | 1 |
| HECW2 | 2.89E-01 | 1 | PHKA1 | 3.44E-01 | 1 |
| ENDOD1 | 2.89E-01 | 1 | TMEM30B | 3.44E-01 | 1 |
| PRL | 2.89E-01 | 1 | VWA5A | 3.44E-01 | 1 |
| ARSH | 2.89E-01 | 1 | DOCK8 | 3.44E-01 | 1 |
| IQCA1 | 2.89E-01 | 1 | OR10H4 | 3.44E-01 | 1 |
| CD70 | 2.89E-01 | 1 | IFNGR2 | 3.44E-01 | 1 |
| MRPS7 | 2.89E-01 | 1 | FBXW8 | 3.44E-01 | 1 |
| LRRC6 | 2.90E-01 | 1 | KIF19 | 3.44E-01 | 1 |
| TMEM222 | 2.90E-01 | 1 | MRGPRX1 | 3.45E-01 | 1 |
| STAC2 | 2.90E-01 | 1 | PCDHA12 | 3.45E-01 | 1 |
| ZNF134 | 2.90E-01 | 1 | PCDHA7 | 3.45E-01 | 1 |
| HNRNPH2 | 2.90E-01 | 1 | GLYATL1 | 3.45E-01 | 1 |
| SLC7A13 | 2.90E-01 | 1 | SP7 | 3.45E-01 | 1 |
| IL18 | 2.90E-01 | 1 | CREB3 | 3.45E-01 | 1 |
| FAM180A | 2.90E-01 | 1 | C1orf68 | 3.46E-01 | 1 |
| PTCD1 | 2.90E-01 | 1 | SLCO5A1 | 3.46E-01 | 1 |
| VBP1 | 2.90E-01 | 1 | ERGIC3 | 3.46E-01 | 1 |
| UCHL5 | 2.90E-01 | 1 | GSDMD | 3.46E-01 | 1 |
| FKBP11 | 2.91E-01 | 1 | MYO1B | 3.46E-01 | 1 |
| PDE6A | 2.91E-01 | 1 | OR10H2 | 3.46E-01 | 1 |
| GABRG2 | 2.91E-01 | 1 | SPEF2 | 3.46E-01 | 1 |
| SCO2 | 2.91E-01 | 1 | SQSTM1 | 3.47E-01 | 1 |
| SLC7A8 | 2.91E-01 | 1 | JAK3 | 3.47E-01 | 1 |
| HDAC10 | 2.91E-01 | 1 | USP29 | 3.47E-01 | 1 |
| WNT7A | 2.91E-01 | 1 | ACACA | 3.47E-01 | 1 |
| MBD3L5 | 2.91E-01 | 1 | TWIST1 | 3.47E-01 | 1 |
| IGSF3 | 2.91E-01 | 1 | EPM2A | 3.47E-01 | 1 |
| SEMA3D | 2.91E-01 | 1 | C5 | 3.47E-01 | 1 |
| JRK | 2.91E-01 | 1 | CEACAM7 | 3.47E-01 | 1 |
| BMPR1B | 2.92E-01 | 1 | CELF1 | 3.47E-01 | 1 |
| HAUS6 | 2.92E-01 | 1 | SYMPK | 3.47E-01 | 1 |
| EFEMP1 | 2.92E-01 | 1 | GUCY2D | 3.47E-01 | 1 |
| CAMK2N | 2.92E-01 | 1 | CDC14A | 3.47E-01 | 1 |
| ATF5 | 2.92E-01 | 1 | CNTD1 | 3.48E-01 | 1 |
| GALNT1 | 2.92E-01 | 1 | IFI44L | 3.48E-01 | 1 |
| OR5D18 | 2.92E-01 | 1 | KBTBD3 | 3.48E-01 | 1 |
| MAFA | 2.92E-01 | 1 | ECEL1 | 3.48E-01 | 1 |
| ESRRG | 2.92E-01 | 1 | ZC3H11A | 3.48E-01 | 1 |
| PLOD3 | 2.92E-01 | 1 | ZNF286A | 3.49E-01 | 1 |
| CAPSL | 2.92E-01 | 1 | SYT3 | 3.49E-01 | 1 |
| RBP4 | 2.93E-01 | 1 | XKR8 | 3.49E-01 | 1 |
| YME1L1 | 2.93E-01 | 1 | 10-Sep | 3.49E-01 | 1 |
| ANKRD52 | 2.93E-01 | 1 | PLEKHA6 | 3.49E-01 | 1 |

| ZNF844 | 2.93E-01 | 1 | TP63 | 3.49E-01 | 1 |
| --- | --- | --- | --- | --- | --- |
| STX10 | 2.93E-01 | 1 | INF2 | 3.49E-01 | 1 |
| ATF3 | 2.93E-01 | 1 | FEZF2 | 3.49E-01 | 1 |
| KCNMB1 | 2.93E-01 | 1 | PDCD1LG2 | 3.49E-01 | 1 |
| NKX3-2 | 2.93E-01 | 1 | MIB2 | 3.49E-01 | 1 |
| CHMP1B | 2.93E-01 | 1 | NXPH1 | 3.49E-01 | 1 |
| UBE2H | 2.94E-01 | 1 | CENPJ | 3.49E-01 | 1 |
| DTX3 | 2.94E-01 | 1 | ANKRD29 | 3.49E-01 | 1 |
| GPIHBP1 | 2.94E-01 | 1 | ACVR1C | 3.50E-01 | 1 |
| ATG3 | 2.94E-01 | 1 | SLC23A1 | 3.50E-01 | 1 |
| TFEB | 2.94E-01 | 1 | OR51B4 | 3.50E-01 | 1 |
| KLHL17 | 2.94E-01 | 1 | TRIP4 | 3.50E-01 | 1 |
| AQP5 | 2.94E-01 | 1 | LUZP4 | 3.50E-01 | 1 |
| MUSK | 2.95E-01 | 1 | NFXL1 | 3.50E-01 | 1 |
| LRAT | 2.95E-01 | 1 | KLK12 | 3.50E-01 | 1 |
| XG | 2.95E-01 | 1 | PKP2 | 3.50E-01 | 1 |
| GGT5 | 2.95E-01 | 1 | PLXNA1 | 3.50E-01 | 1 |
| PGBD2 | 2.95E-01 | 1 | NCF4 | 3.50E-01 | 1 |
| RCC1 | 2.95E-01 | 1 | EFCAB6 | 3.51E-01 | 1 |
| ATP8B2 | 2.95E-01 | 1 | C9orf172 | 3.51E-01 | 1 |
| NEK9 | 2.95E-01 | 1 | OR14J1 | 3.51E-01 | 1 |
| LIPH | 2.95E-01 | 1 | KIF7 | 3.51E-01 | 1 |
| TNS3 | 2.95E-01 | 1 | CES3 | 3.51E-01 | 1 |
| SNTB2 | 2.95E-01 | 1 | BTRC | 3.51E-01 | 1 |
| EFTUD2 | 2.95E-01 | 1 | NDUFS2 | 3.51E-01 | 1 |
| NR1H4 | 2.95E-01 | 1 | PHF7 | 3.51E-01 | 1 |
| PRKD2 | 2.95E-01 | 1 | AEBP1 | 3.52E-01 | 1 |
| PRKCG | 2.95E-01 | 1 | TNNT2 | 3.52E-01 | 1 |
| C6orf120 | 2.95E-01 | 1 | ZNF577 | 3.52E-01 | 1 |
| MRS2 | 2.95E-01 | 1 | PIKFYVE | 3.52E-01 | 1 |
| PRR14 | 2.95E-01 | 1 | NACAD | 3.52E-01 | 1 |
| NTSR2 | 2.95E-01 | 1 | SLC6A17 | 3.52E-01 | 1 |
| ZNF330 | 2.95E-01 | 1 | ZC3H12C | 3.52E-01 | 1 |
| SMUG1 | 2.95E-01 | 1 | EPT1 | 3.52E-01 | 1 |
| GRM7 | 2.95E-01 | 1 | SLC16A12 | 3.52E-01 | 1 |
| PIWIL3 | 2.96E-01 | 1 | B3GALT5 | 3.52E-01 | 1 |
| RNASET2 | 2.96E-01 | 1 | SLC30A2 | 3.53E-01 | 1 |
| KRTAP9-2 | 2.96E-01 | 1 | PASK | 3.53E-01 | 1 |
| GYS1 | 2.96E-01 | 1 | PTPN6 | 3.53E-01 | 1 |
| SELV | 2.96E-01 | 1 | LYSMD4 | 3.53E-01 | 1 |
| KLHL33 | 2.96E-01 | 1 | KIAA1429 | 3.53E-01 | 1 |
| KRTAP27 | 2.96E-01 | 1 | MACROD2 | 3.53E-01 | 1 |
| EIF2A | 2.96E-01 | 1 | SCUBE3 | 3.53E-01 | 1 |
| MRGPRE | 2.96E-01 | 1 | TULP3 | 3.53E-01 | 1 |
| TRIP13 | 2.96E-01 | 1 | DBP | 3.53E-01 | 1 |
| KLRC3 | 2.96E-01 | 1 | TUBA1A | 3.53E-01 | 1 |
| GJB2 | 2.96E-01 | 1 | OR5B12 | 3.53E-01 | 1 |
| LASP1 | 2.97E-01 | 1 | ERN2 | 3.53E-01 | 1 |
| PFKFB3 | 2.97E-01 | 1 | NLRP14 | 3.53E-01 | 1 |
| ROBO1 | 2.97E-01 | 1 | NFIC | 3.53E-01 | 1 |
| DRD3 | 2.97E-01 | 1 | LRRC14 | 3.53E-01 | 1 |
| HDAC2 | 2.97E-01 | 1 | SMEK2 | 3.54E-01 | 1 |
| LRRN2 | 2.97E-01 | 1 | LPCAT3 | 3.54E-01 | 1 |

| HAND2 | 2.97E-01 | 1 | TYR | 3.54E-01 | 1 |
| --- | --- | --- | --- | --- | --- |
| NPSR1 | 2.98E-01 | 1 | OR5D13 | 3.54E-01 | 1 |
| CMTM8 | 2.98E-01 | 1 | GPRIN1 | 3.54E-01 | 1 |
| PDGFRA | 2.98E-01 | 1 | CRHBP | 3.54E-01 | 1 |
| PTBP1 | 2.98E-01 | 1 | P4HTM | 3.54E-01 | 1 |
| SRRM2 | 2.98E-01 | 1 | TRIM16L | 3.55E-01 | 1 |
| MID1IP1 | 2.98E-01 | 1 | ZNF98 | 3.55E-01 | 1 |
| GPI | 2.98E-01 | 1 | LEFTY2 | 3.55E-01 | 1 |
| CYP11B1 | 2.98E-01 | 1 | OR4F15 | 3.55E-01 | 1 |
| BRIX1 | 2.98E-01 | 1 | EBF1 | 3.55E-01 | 1 |
| TTC21A | 2.98E-01 | 1 | AGPS | 3.55E-01 | 1 |
| BNIP3L | 2.98E-01 | 1 | VSIG4 | 3.55E-01 | 1 |
| SNX29 | 2.98E-01 | 1 | DES | 3.56E-01 | 1 |
| PPP1CC | 2.98E-01 | 1 | FGD6 | 3.56E-01 | 1 |
| GPR135 | 2.98E-01 | 1 | TSG101 | 3.56E-01 | 1 |
| APEX2 | 2.99E-01 | 1 | ZNF777 | 3.56E-01 | 1 |
| GHR | 2.99E-01 | 1 | RPL3 | 3.56E-01 | 1 |
| TTBK2 | 2.99E-01 | 1 | ID1 | 3.56E-01 | 1 |
| KIAA1257 | 2.99E-01 | 1 | MORF4L1 | 3.56E-01 | 1 |
| DHRS4 | 2.99E-01 | 1 | ELAVL1 | 3.56E-01 | 1 |
| PCSK1 | 2.99E-01 | 1 | MDC1 | 3.56E-01 | 1 |
| BCL11A | 2.99E-01 | 1 | DAPK2 | 3.56E-01 | 1 |
| SLC7A9 | 2.99E-01 | 1 | IGSF10 | 3.56E-01 | 1 |
| JMJD8 | 2.99E-01 | 1 | TMC2 | 3.56E-01 | 1 |
| RBM6 | 2.99E-01 | 1 | GALNT8 | 3.57E-01 | 1 |
| TMEM204 | 2.99E-01 | 1 | HAPLN1 | 3.57E-01 | 1 |
| SDC4 | 2.99E-01 | 1 | DUSP4 | 3.57E-01 | 1 |
| PLEKHA6 | 3.00E-01 | 1 | KIFC3 | 3.57E-01 | 1 |
| TTC9B | 3.00E-01 | 1 | KCNC2 | 3.57E-01 | 1 |
| DCLK1 | 3.00E-01 | 1 | CLP1 | 3.57E-01 | 1 |
| KPNA5 | 3.00E-01 | 1 | PHF20 | 3.57E-01 | 1 |
| DUS4L | 3.00E-01 | 1 | TSGA10IP | 3.57E-01 | 1 |
| PLEKHH3 | 3.00E-01 | 1 | AGBL2 | 3.57E-01 | 1 |
| TMEM8C | 3.00E-01 | 1 | YIPF2 | 3.57E-01 | 1 |
| KRTAP19 | 3.00E-01 | 1 | ANKRD44 | 3.57E-01 | 1 |
| NPM1 | 3.00E-01 | 1 | TIGD3 | 3.57E-01 | 1 |
| ATP6V0E | 3.00E-01 | 1 | CHD1L | 3.57E-01 | 1 |
| KATNAL2 | 3.00E-01 | 1 | EFEMP2 | 3.57E-01 | 1 |
| MAP3K11 | 3.01E-01 | 1 | TMPRSS7 | 3.57E-01 | 1 |
| LYL1 | 3.01E-01 | 1 | TLE1 | 3.57E-01 | 1 |
| NMBR | 3.01E-01 | 1 | NFE2 | 3.58E-01 | 1 |
| NCOA5 | 3.01E-01 | 1 | PXDNL | 3.58E-01 | 1 |
| DNAJA4 | 3.01E-01 | 1 | ADD2 | 3.58E-01 | 1 |
| MARVEL | 3.01E-01 | 1 | GRIPAP1 | 3.58E-01 | 1 |
| KCNQ5 | 3.01E-01 | 1 | TMEM163 | 3.58E-01 | 1 |
| IQCB1 | 3.01E-01 | 1 | OSR2 | 3.58E-01 | 1 |
| CALCA | 3.01E-01 | 1 | DPEP1 | 3.58E-01 | 1 |
| GABBR2 | 3.01E-01 | 1 | LOX | 3.58E-01 | 1 |
| OGG1 | 3.01E-01 | 1 | AP3D1 | 3.59E-01 | 1 |
| MRGPRX | 3.01E-01 | 1 | PAX4 | 3.59E-01 | 1 |
| TRAF3IP3 | 3.01E-01 | 1 | BMP2 | 3.59E-01 | 1 |
| PTPN11 | 3.01E-01 | 1 | LSM14A | 3.59E-01 | 1 |
| TAF1D | 3.02E-01 | 1 | DBN1 | 3.59E-01 | 1 |

| CCL25 | 3.02E-01 | 1 | GAB2 | 3.59E-01 | 1 |
| --- | --- | --- | --- | --- | --- |
| RQCD1 | 3.02E-01 | 1 | IL13RA1 | 3.59E-01 | 1 |
| CCDC114 | 3.02E-01 | 1 | CHCHD6 | 3.59E-01 | 1 |
| MTRF1L | 3.02E-01 | 1 | PRB3 | 3.59E-01 | 1 |
| VEGFC | 3.02E-01 | 1 | SPTAN1 | 3.59E-01 | 1 |
| KRT13 | 3.02E-01 | 1 | NCOA7 | 3.60E-01 | 1 |
| ACAD10 | 3.02E-01 | 1 | SLC22A16 | 3.60E-01 | 1 |
| GFOD2 | 3.02E-01 | 1 | TACC3 | 3.60E-01 | 1 |
| OR10J3 | 3.02E-01 | 1 | MYF5 | 3.60E-01 | 1 |
| ARHGEF7 | 3.02E-01 | 1 | AGAP2 | 3.60E-01 | 1 |
| NIF3L1 | 3.02E-01 | 1 | ARHGEF6 | 3.60E-01 | 1 |
| C9orf47 | 3.02E-01 | 1 | SYT15 | 3.60E-01 | 1 |
| ZIC2 | 3.03E-01 | 1 | NRIP2 | 3.60E-01 | 1 |
| MRC2 | 3.03E-01 | 1 | GPR84 | 3.60E-01 | 1 |
| HAVCR2 | 3.03E-01 | 1 | BLK | 3.60E-01 | 1 |
| TMEM115 | 3.03E-01 | 1 | KAT5 | 3.61E-01 | 1 |
| PLXNB2 | 3.03E-01 | 1 | NPTXR | 3.61E-01 | 1 |
| GOT1 | 3.03E-01 | 1 | STK32B | 3.61E-01 | 1 |
| AZIN1 | 3.03E-01 | 1 | ZEB2 | 3.61E-01 | 1 |
| POFUT1 | 3.03E-01 | 1 | SKAP1 | 3.61E-01 | 1 |
| MMP7 | 3.03E-01 | 1 | IBTK | 3.61E-01 | 1 |
| FAM184A | 3.03E-01 | 1 | PTPRZ1 | 3.62E-01 | 1 |
| KRT5 | 3.03E-01 | 1 | MAPK7 | 3.62E-01 | 1 |
| CCDC60 | 3.03E-01 | 1 | CLCN4 | 3.62E-01 | 1 |
| ABCA13 | 3.03E-01 | 1 | UEVLD | 3.62E-01 | 1 |
| DDX11 | 3.04E-01 | 1 | CCDC9 | 3.62E-01 | 1 |
| LDLRAD2 | 3.04E-01 | 1 | SPRY1 | 3.62E-01 | 1 |
| ENO2 | 3.04E-01 | 1 | CD38 | 3.63E-01 | 1 |
| ZNF276 | 3.04E-01 | 1 | ARFGEF2 | 3.63E-01 | 1 |
| PLXNA3 | 3.04E-01 | 1 | PCDHA13 | 3.63E-01 | 1 |
| CLN6 | 3.04E-01 | 1 | PITPNC1 | 3.63E-01 | 1 |
| MEOX1 | 3.04E-01 | 1 | ARHGAP15 | 3.63E-01 | 1 |
| MPHOSPH | 3.04E-01 | 1 | BTNL2 | 3.63E-01 | 1 |
| ALOX15B | 3.04E-01 | 1 | MTO1 | 3.63E-01 | 1 |
| SCARA5 | 3.05E-01 | 1 | FRMD8 | 3.63E-01 | 1 |
| SLC9A4 | 3.05E-01 | 1 | SLC25A38 | 3.63E-01 | 1 |
| TNNI3K | 3.05E-01 | 1 | CUZD1 | 3.64E-01 | 1 |
| KLF10 | 3.05E-01 | 1 | ATP13A4 | 3.64E-01 | 1 |
| N4BP1 | 3.05E-01 | 1 | AGAP1 | 3.65E-01 | 1 |
| PPP2R5B | 3.05E-01 | 1 | OR6C2 | 3.65E-01 | 1 |
| CACNB4 | 3.05E-01 | 1 | IMPA2 | 3.65E-01 | 1 |
| SECTM1 | 3.05E-01 | 1 | F10 | 3.65E-01 | 1 |
| C12orf74 | 3.05E-01 | 1 | HOXB1 | 3.65E-01 | 1 |
| ABCC6 | 3.06E-01 | 1 | AP3B2 | 3.65E-01 | 1 |
| IL17A | 3.06E-01 | 1 | ST14 | 3.65E-01 | 1 |
| BMF | 3.06E-01 | 1 | SLC22A25 | 3.65E-01 | 1 |
| DUSP22 | 3.06E-01 | 1 | DNAJC22 | 3.65E-01 | 1 |
| CCT8L2 | 3.06E-01 | 1 | ACTR3 | 3.65E-01 | 1 |
| MOB2 | 3.06E-01 | 1 | USHBP1 | 3.66E-01 | 1 |
| OR2AT4 | 3.06E-01 | 1 | EPB41L5 | 3.66E-01 | 1 |
| FABP9 | 3.06E-01 | 1 | EVPL | 3.66E-01 | 1 |
| CCL11 | 3.06E-01 | 1 | KIAA0319L | 3.66E-01 | 1 |
| PLCD3 | 3.06E-01 | 1 | GNL2 | 3.66E-01 | 1 |

| MAD2L1 | 3.06E-01 | 1 | ABCG5 | 3.66E-01 | 1 |
| --- | --- | --- | --- | --- | --- |
| TNFSF13B | 3.06E-01 | 1 | SH2D7 | 3.66E-01 | 1 |
| FAM167A | 3.07E-01 | 1 | SAMD14 | 3.66E-01 | 1 |
| TRAPPC4 | 3.07E-01 | 1 | SLC6A13 | 3.66E-01 | 1 |
| ALX3 | 3.07E-01 | 1 | LRRIQ3 | 3.67E-01 | 1 |
| GK2 | 3.07E-01 | 1 | TMEM143 | 3.67E-01 | 1 |
| FAHD2A | 3.07E-01 | 1 | UBAC2 | 3.67E-01 | 1 |
| TMEM187 | 3.07E-01 | 1 | CLEC17A | 3.67E-01 | 1 |
| APCS | 3.07E-01 | 1 | TLL1 | 3.68E-01 | 1 |
| REG4 | 3.07E-01 | 1 | C5orf60 | 3.68E-01 | 1 |
| TMED7 | 3.07E-01 | 1 | MVK | 3.68E-01 | 1 |
| PAQR5 | 3.07E-01 | 1 | ENTPD2 | 3.68E-01 | 1 |
| CD2BP2 | 3.07E-01 | 1 | SH3GL1 | 3.68E-01 | 1 |
| AICDA | 3.07E-01 | 1 | PLEKHA4 | 3.68E-01 | 1 |
| ANO10 | 3.07E-01 | 1 | SLC17A2 | 3.68E-01 | 1 |
| SLC44A5 | 3.08E-01 | 1 | MTAP | 3.68E-01 | 1 |
| PSG9 | 3.08E-01 | 1 | USP26 | 3.68E-01 | 1 |
| FNDC7 | 3.08E-01 | 1 | CALD1 | 3.68E-01 | 1 |
| CRYBA4 | 3.08E-01 | 1 | MS4A2 | 3.69E-01 | 1 |
| C12orf43 | 3.08E-01 | 1 | CNN1 | 3.69E-01 | 1 |
| GSDMB | 3.08E-01 | 1 | OR8D2 | 3.69E-01 | 1 |
| LHX9 | 3.08E-01 | 1 | GPAA1 | 3.69E-01 | 1 |
| DIABLO | 3.08E-01 | 1 | MICAL2 | 3.69E-01 | 1 |
| ADORA3 | 3.08E-01 | 1 | CUL9 | 3.69E-01 | 1 |
| SCAMP3 | 3.09E-01 | 1 | OLFML1 | 3.69E-01 | 1 |
| TOMM34 | 3.09E-01 | 1 | NIF3L1 | 3.70E-01 | 1 |
| APPL1 | 3.09E-01 | 1 | GOLM1 | 3.70E-01 | 1 |
| ARGFX | 3.09E-01 | 1 | OR10G9 | 3.70E-01 | 1 |
| LMAN1 | 3.09E-01 | 1 | TICAM1 | 3.70E-01 | 1 |
| ADCYAP1 | 3.09E-01 | 1 | DRG2 | 3.70E-01 | 1 |
| CUL4A | 3.09E-01 | 1 | DEDD2 | 3.70E-01 | 1 |
| SFXN2 | 3.09E-01 | 1 | MCM8 | 3.70E-01 | 1 |
| BIRC2 | 3.10E-01 | 1 | MCHR1 | 3.70E-01 | 1 |
| FAM179A | 3.10E-01 | 1 | GSTK1 | 3.70E-01 | 1 |
| PABPC5 | 3.10E-01 | 1 | HNRNPH2 | 3.70E-01 | 1 |
| SLCO5A1 | 3.10E-01 | 1 | DONSON | 3.70E-01 | 1 |
| AGR3 | 3.10E-01 | 1 | LILRA4 | 3.71E-01 | 1 |
| SOCS7 | 3.10E-01 | 1 | CACNG4 | 3.71E-01 | 1 |
| MON2 | 3.10E-01 | 1 | BAG4 | 3.71E-01 | 1 |
| GLI1 | 3.10E-01 | 1 | PPP1R3F | 3.71E-01 | 1 |
| RAD54L2 | 3.10E-01 | 1 | POM121C | 3.71E-01 | 1 |
| ACOX3 | 3.10E-01 | 1 | OR13C9 | 3.71E-01 | 1 |
| FAM150A | 3.10E-01 | 1 | ME2 | 3.71E-01 | 1 |
| CSRNP3 | 3.10E-01 | 1 | ACRBP | 3.71E-01 | 1 |
| RSF1 | 3.11E-01 | 1 | DYTN | 3.72E-01 | 1 |
| ATP13A4 | 3.11E-01 | 1 | OSCP1 | 3.72E-01 | 1 |
| ZNF397 | 3.11E-01 | 1 | CXCR4 | 3.72E-01 | 1 |
| ARHGAP2 | 3.11E-01 | 1 | CEP57 | 3.72E-01 | 1 |
| NOV | 3.11E-01 | 1 | PELP1 | 3.72E-01 | 1 |
| DUSP18 | 3.11E-01 | 1 | GPHN | 3.72E-01 | 1 |
| CBL | 3.11E-01 | 1 | IFI16 | 3.72E-01 | 1 |
| TRERF1 | 3.11E-01 | 1 | ECHS1 | 3.72E-01 | 1 |
| STRN | 3.11E-01 | 1 | RGS4 | 3.72E-01 | 1 |

| STMN1 | 3.11E-01 | 1 | MRPS2 | 3.72E-01 | 1 |
| --- | --- | --- | --- | --- | --- |
| SGK1 | 3.11E-01 | 1 | HMGCS1 | 3.72E-01 | 1 |
| FMO1 | 3.11E-01 | 1 | KRT32 | 3.72E-01 | 1 |
| EFCAB3 | 3.11E-01 | 1 | SLC12A8 | 3.72E-01 | 1 |
| C11orf73 | 3.11E-01 | 1 | HTR6 | 3.72E-01 | 1 |
| PVR | 3.12E-01 | 1 | GLRA4 | 3.72E-01 | 1 |
| PHACTR2 | 3.12E-01 | 1 | ZIM3 | 3.72E-01 | 1 |
| CD6 | 3.12E-01 | 1 | GFPT2 | 3.72E-01 | 1 |
| KIRREL2 | 3.12E-01 | 1 | CDK12 | 3.73E-01 | 1 |
| ATP8A1 | 3.12E-01 | 1 | SRGAP3 | 3.73E-01 | 1 |
| HEY1 | 3.12E-01 | 1 | PODNL1 | 3.73E-01 | 1 |
| SAFB2 | 3.12E-01 | 1 | OAT | 3.73E-01 | 1 |
| ARHGEF1 | 3.13E-01 | 1 | RADIL | 3.74E-01 | 1 |
| PIH1D1 | 3.13E-01 | 1 | C11orf95 | 3.74E-01 | 1 |
| FAM129A | 3.13E-01 | 1 | ANO7 | 3.74E-01 | 1 |
| NXNL2 | 3.13E-01 | 1 | CD300LB | 3.74E-01 | 1 |
| AK1 | 3.13E-01 | 1 | RBM14 | 3.74E-01 | 1 |
| IL24 | 3.13E-01 | 1 | ETV3 | 3.75E-01 | 1 |
| CDIPT | 3.13E-01 | 1 | RAD51 | 3.75E-01 | 1 |
| GYPA | 3.13E-01 | 1 | IPCEF1 | 3.75E-01 | 1 |
| SAMD5 | 3.13E-01 | 1 | SEMG1 | 3.75E-01 | 1 |
| GABRB2 | 3.13E-01 | 1 | OR1K1 | 3.75E-01 | 1 |
| E2F3 | 3.13E-01 | 1 | HSPA13 | 3.75E-01 | 1 |
| CCL13 | 3.13E-01 | 1 | LHFPL3 | 3.75E-01 | 1 |
| MMP13 | 3.13E-01 | 1 | CBL | 3.75E-01 | 1 |
| TP63 | 3.13E-01 | 1 | OR7D2 | 3.75E-01 | 1 |
| ZNRF4 | 3.13E-01 | 1 | SCRN3 | 3.75E-01 | 1 |
| ZNF296 | 3.13E-01 | 1 | TREX1 | 3.75E-01 | 1 |
| CC2D1A | 3.14E-01 | 1 | PDZD3 | 3.75E-01 | 1 |
| RORA | 3.14E-01 | 1 | DLK1 | 3.76E-01 | 1 |
| SNRK | 3.14E-01 | 1 | ZNF768 | 3.76E-01 | 1 |
| HSP90AA | 3.14E-01 | 1 | MUC7 | 3.76E-01 | 1 |
| FMN1 | 3.14E-01 | 1 | OR51L1 | 3.76E-01 | 1 |
| ADCK1 | 3.14E-01 | 1 | TNFRSF11B | 3.76E-01 | 1 |
| ZNF500 | 3.14E-01 | 1 | NR1H3 | 3.76E-01 | 1 |
| SDF2L1 | 3.14E-01 | 1 | FNBP1L | 3.76E-01 | 1 |
| PEX5L | 3.14E-01 | 1 | OMD | 3.76E-01 | 1 |
| BTBD3 | 3.14E-01 | 1 | WDR89 | 3.77E-01 | 1 |
| TUBB6 | 3.14E-01 | 1 | MED14 | 3.77E-01 | 1 |
| AIM1L | 3.14E-01 | 1 | LAMP1 | 3.77E-01 | 1 |
| EXD3 | 3.14E-01 | 1 | CCDC68 | 3.77E-01 | 1 |
| ATXN1 | 3.14E-01 | 1 | KIAA0368 | 3.77E-01 | 1 |
| ZNF395 | 3.14E-01 | 1 | OR4N5 | 3.77E-01 | 1 |
| TRPC1 | 3.14E-01 | 1 | ZNF835 | 3.77E-01 | 1 |
| LALBA | 3.14E-01 | 1 | CYP21A2 | 3.77E-01 | 1 |
| MFGE8 | 3.15E-01 | 1 | YBX2 | 3.77E-01 | 1 |
| PDS5B | 3.15E-01 | 1 | GALNT13 | 3.77E-01 | 1 |
| PAX1 | 3.15E-01 | 1 | STIP1 | 3.78E-01 | 1 |
| CELF5 | 3.15E-01 | 1 | FGFRL1 | 3.78E-01 | 1 |
| SLC30A4 | 3.15E-01 | 1 | CKAP2 | 3.78E-01 | 1 |
| PAICS | 3.15E-01 | 1 | OR8I2 | 3.78E-01 | 1 |
| SGTB | 3.15E-01 | 1 | ACSF3 | 3.79E-01 | 1 |
| ANKRD24 | 3.15E-01 | 1 | TCHH | 3.79E-01 | 1 |

| CC2D2B | 3.15E-01 | 1 | LRRC17 | 3.79E-01 | 1 |
| --- | --- | --- | --- | --- | --- |
| CEP70 | 3.15E-01 | 1 | KRTAP9-3 | 3.79E-01 | 1 |
| WDR12 | 3.15E-01 | 1 | GAS2 | 3.79E-01 | 1 |
| PLEKHM3 | 3.15E-01 | 1 | PNMA2 | 3.79E-01 | 1 |
| HES1 | 3.15E-01 | 1 | LHX3 | 3.79E-01 | 1 |
| CC2D2A | 3.15E-01 | 1 | EDA2R | 3.79E-01 | 1 |
| SLC5A12 | 3.15E-01 | 1 | LEP | 3.79E-01 | 1 |
| APPBP2 | 3.15E-01 | 1 | HHAT | 3.79E-01 | 1 |
| NOX5 | 3.15E-01 | 1 | ZNF804A | 3.79E-01 | 1 |
| HMOX1 | 3.16E-01 | 1 | NCSTN | 3.79E-01 | 1 |
| AP3S1 | 3.16E-01 | 1 | PSMD6 | 3.80E-01 | 1 |
| CDNF | 3.16E-01 | 1 | MYADML2 | 3.80E-01 | 1 |
| CPXM1 | 3.16E-01 | 1 | C8orf76 | 3.80E-01 | 1 |
| RAB26 | 3.16E-01 | 1 | MAGEH1 | 3.80E-01 | 1 |
| TTPAL | 3.16E-01 | 1 | SNW1 | 3.80E-01 | 1 |
| IGLL5 | 3.16E-01 | 1 | UACA | 3.80E-01 | 1 |
| GCN1L1 | 3.16E-01 | 1 | ZNF337 | 3.80E-01 | 1 |
| C8orf33 | 3.16E-01 | 1 | PSEN1 | 3.80E-01 | 1 |
| ING2 | 3.16E-01 | 1 | LAMB1 | 3.80E-01 | 1 |
| TAS2R39 | 3.16E-01 | 1 | CDHR4 | 3.80E-01 | 1 |
| RASA3 | 3.16E-01 | 1 | SMAD3 | 3.81E-01 | 1 |
| C17orf104 | 3.16E-01 | 1 | ABL1 | 3.81E-01 | 1 |
| ZXDC | 3.16E-01 | 1 | PTCH2 | 3.81E-01 | 1 |
| ADAM32 | 3.16E-01 | 1 | SPATA4 | 3.81E-01 | 1 |
| BASP1 | 3.16E-01 | 1 | WDR17 | 3.81E-01 | 1 |
| KRT32 | 3.16E-01 | 1 | FAM122A | 3.81E-01 | 1 |
| BCL6 | 3.16E-01 | 1 | CA8 | 3.82E-01 | 1 |
| CHSY3 | 3.16E-01 | 1 | IL23R | 3.82E-01 | 1 |
| HSPB2 | 3.17E-01 | 1 | GSR | 3.82E-01 | 1 |
| TCERG1 | 3.17E-01 | 1 | OR9A4 | 3.82E-01 | 1 |
| ZNF684 | 3.17E-01 | 1 | SYT6 | 3.82E-01 | 1 |
| PEX2 | 3.17E-01 | 1 | ZBTB37 | 3.82E-01 | 1 |
| ZBTB43 | 3.17E-01 | 1 | CALR3 | 3.82E-01 | 1 |
| IGFBP4 | 3.17E-01 | 1 | ZWILCH | 3.82E-01 | 1 |
| MYEOV | 3.17E-01 | 1 | MTRF1L | 3.82E-01 | 1 |
| PARM1 | 3.17E-01 | 1 | ASB16 | 3.83E-01 | 1 |
| TSKS | 3.17E-01 | 1 | RBP3 | 3.83E-01 | 1 |
| PTPN6 | 3.17E-01 | 1 | METTL1 | 3.83E-01 | 1 |
| PCSK7 | 3.18E-01 | 1 | ATP8A2 | 3.83E-01 | 1 |
| ANGPT1 | 3.18E-01 | 1 | KLHL23 | 3.83E-01 | 1 |
| APIP | 3.18E-01 | 1 | ITGAV | 3.83E-01 | 1 |
| WNT3A | 3.18E-01 | 1 | C9orf171 | 3.83E-01 | 1 |
| KPNA7 | 3.18E-01 | 1 | PRKCZ | 3.83E-01 | 1 |
| TTC26 | 3.18E-01 | 1 | MYB | 3.84E-01 | 1 |
| CRYGC | 3.18E-01 | 1 | FER1L5 | 3.84E-01 | 1 |
| MCL1 | 3.18E-01 | 1 | SLC36A1 | 3.84E-01 | 1 |
| GLDC | 3.18E-01 | 1 | ZIC4 | 3.84E-01 | 1 |
| MMACHC | 3.18E-01 | 1 | SLC29A4 | 3.84E-01 | 1 |
| FAM188A | 3.18E-01 | 1 | SUSD5 | 3.84E-01 | 1 |
| GHITM | 3.18E-01 | 1 | RASSF2 | 3.84E-01 | 1 |
| ESCO1 | 3.18E-01 | 1 | ZNF232 | 3.84E-01 | 1 |
| KRT7 | 3.19E-01 | 1 | ATP6V0A1 | 3.85E-01 | 1 |
| EGLN2 | 3.19E-01 | 1 | ANO5 | 3.85E-01 | 1 |

| PLCXD3 | 3.19E-01 | 1 | GPD1L | 3.85E-01 | 1 |
| --- | --- | --- | --- | --- | --- |
| TUBA8 | 3.19E-01 | 1 | HEMK1 | 3.85E-01 | 1 |
| HNRNPL | 3.19E-01 | 1 | ST7L | 3.85E-01 | 1 |
| GCC1 | 3.19E-01 | 1 | TTPA | 3.85E-01 | 1 |
| LRRC8E | 3.19E-01 | 1 | HSPA12B | 3.85E-01 | 1 |
| CNOT6L | 3.19E-01 | 1 | KRT25 | 3.85E-01 | 1 |
| PXK | 3.19E-01 | 1 | NEXN | 3.85E-01 | 1 |
| C6orf62 | 3.19E-01 | 1 | TMEM145 | 3.85E-01 | 1 |
| GK5 | 3.19E-01 | 1 | PRKACA | 3.85E-01 | 1 |
| TRA2B | 3.19E-01 | 1 | CRB1 | 3.86E-01 | 1 |
| NPFFR2 | 3.19E-01 | 1 | TPD52 | 3.86E-01 | 1 |
| CLTC | 3.19E-01 | 1 | SLC16A2 | 3.86E-01 | 1 |
| CCDC27 | 3.19E-01 | 1 | PTGDR | 3.86E-01 | 1 |
| RALY | 3.20E-01 | 1 | AZIN1 | 3.86E-01 | 1 |
| OPCML | 3.20E-01 | 1 | MRPL1 | 3.86E-01 | 1 |
| DOC2A | 3.20E-01 | 1 | BBS2 | 3.86E-01 | 1 |
| ZNF205 | 3.20E-01 | 1 | FNIP2 | 3.86E-01 | 1 |
| HRH3 | 3.20E-01 | 1 | SLC12A7 | 3.86E-01 | 1 |
| IL23R | 3.20E-01 | 1 | NAALAD2 | 3.86E-01 | 1 |
| SETD8 | 3.20E-01 | 1 | TAS2R30 | 3.86E-01 | 1 |
| WBSCR28 | 3.20E-01 | 1 | APBA1 | 3.86E-01 | 1 |
| MAPKAP1 | 3.20E-01 | 1 | DDX3Y | 3.86E-01 | 1 |
| NR0B2 | 3.20E-01 | 1 | OTX2 | 3.87E-01 | 1 |
| GPT2 | 3.20E-01 | 1 | TMEM53 | 3.87E-01 | 1 |
| TAB2 | 3.20E-01 | 1 | TOP1MT | 3.87E-01 | 1 |
| RAB19 | 3.20E-01 | 1 | LMF2 | 3.87E-01 | 1 |
| CDC25C | 3.20E-01 | 1 | DHX32 | 3.87E-01 | 1 |
| USP38 | 3.20E-01 | 1 | PRSS38 | 3.87E-01 | 1 |
| ITSN1 | 3.20E-01 | 1 | ARL13A | 3.87E-01 | 1 |
| GSPT1 | 3.20E-01 | 1 | OXSR1 | 3.87E-01 | 1 |
| TIMM44 | 3.20E-01 | 1 | VWA5B2 | 3.87E-01 | 1 |
| SYPL1 | 3.20E-01 | 1 | FSTL4 | 3.87E-01 | 1 |
| ITPKB | 3.21E-01 | 1 | SMC6 | 3.87E-01 | 1 |
| LARP1B | 3.21E-01 | 1 | RMI1 | 3.87E-01 | 1 |
| CD151 | 3.21E-01 | 1 | AR | 3.88E-01 | 1 |
| PAN2 | 3.21E-01 | 1 | ATG2A | 3.88E-01 | 1 |
| MYH7B | 3.21E-01 | 1 | PON3 | 3.88E-01 | 1 |
| CITED2 | 3.21E-01 | 1 | VCP | 3.88E-01 | 1 |
| EPAS1 | 3.21E-01 | 1 | RNF135 | 3.88E-01 | 1 |
| C3orf33 | 3.21E-01 | 1 | SLBP | 3.88E-01 | 1 |
| UBASH3B | 3.21E-01 | 1 | NR2F1 | 3.88E-01 | 1 |
| IRF1 | 3.21E-01 | 1 | HDAC2 | 3.88E-01 | 1 |
| PADI4 | 3.21E-01 | 1 | OR2A5 | 3.88E-01 | 1 |
| EGF | 3.21E-01 | 1 | KIF26A | 3.89E-01 | 1 |
| KCNJ14 | 3.21E-01 | 1 | ARHGAP27 | 3.89E-01 | 1 |
| RGS9BP | 3.22E-01 | 1 | EXOC6 | 3.89E-01 | 1 |
| CDH17 | 3.22E-01 | 1 | PLD2 | 3.89E-01 | 1 |
| HAX1 | 3.22E-01 | 1 | NSDHL | 3.89E-01 | 1 |
| QPCT | 3.22E-01 | 1 | MRAP2 | 3.89E-01 | 1 |
| PRKAB2 | 3.22E-01 | 1 | CCNA1 | 3.89E-01 | 1 |
| CACNA1E | 3.22E-01 | 1 | NYNRIN | 3.90E-01 | 1 |
| EEPD1 | 3.22E-01 | 1 | TMEM51 | 3.90E-01 | 1 |
| PIGW | 3.22E-01 | 1 | ROR2 | 3.90E-01 | 1 |

| KCNK2 | 3.22E-01 | 1 | IKZF1 | 3.90E-01 | 1 |
| --- | --- | --- | --- | --- | --- |
| GIMAP8 | 3.22E-01 | 1 | ATF6 | 3.90E-01 | 1 |
| ASXL2 | 3.23E-01 | 1 | IFRD2 | 3.90E-01 | 1 |
| HCCS | 3.23E-01 | 1 | TXNDC2 | 3.90E-01 | 1 |
| TNFSF15 | 3.23E-01 | 1 | UBP1 | 3.90E-01 | 1 |
| FLVCR1 | 3.23E-01 | 1 | GIT1 | 3.90E-01 | 1 |
| SMARCD | 3.23E-01 | 1 | TBL1XR1 | 3.90E-01 | 1 |
| NAPA | 3.23E-01 | 1 | TMEM30A | 3.91E-01 | 1 |
| CDKN3 | 3.23E-01 | 1 | FRA10AC1 | 3.91E-01 | 1 |
| CCDC86 | 3.23E-01 | 1 | CAPN6 | 3.91E-01 | 1 |
| RGS13 | 3.23E-01 | 1 | KRT40 | 3.91E-01 | 1 |
| FAM132A | 3.23E-01 | 1 | TTF1 | 3.91E-01 | 1 |
| SPAG5 | 3.24E-01 | 1 | RPH3A | 3.91E-01 | 1 |
| CDK2AP2 | 3.24E-01 | 1 | EPPK1 | 3.91E-01 | 1 |
| CLCA1 | 3.24E-01 | 1 | LUM | 3.91E-01 | 1 |
| GIMAP1 | 3.24E-01 | 1 | CNDP2 | 3.91E-01 | 1 |
| MSRB2 | 3.24E-01 | 1 | HECW1 | 3.91E-01 | 1 |
| ARMC10 | 3.24E-01 | 1 | CDC42BPA | 3.91E-01 | 1 |
| PTCHD1 | 3.24E-01 | 1 | EIF2B2 | 3.91E-01 | 1 |
| CDSN | 3.25E-01 | 1 | SMTNL1 | 3.91E-01 | 1 |
| FBXO47 | 3.25E-01 | 1 | OMG | 3.91E-01 | 1 |
| DDX59 | 3.25E-01 | 1 | IDH3G | 3.91E-01 | 1 |
| NFKBIL1 | 3.25E-01 | 1 | RFPL4A | 3.92E-01 | 1 |
| PTBP2 | 3.25E-01 | 1 | ZNF551 | 3.92E-01 | 1 |
| TAS2R10 | 3.25E-01 | 1 | TLE4 | 3.92E-01 | 1 |
| TBCCD1 | 3.25E-01 | 1 | SLCO3A1 | 3.92E-01 | 1 |
| RNF19A | 3.25E-01 | 1 | PDZD7 | 3.92E-01 | 1 |
| ANKRD11 | 3.25E-01 | 1 | CCNG1 | 3.92E-01 | 1 |
| SULT1A2 | 3.25E-01 | 1 | GLG1 | 3.92E-01 | 1 |
| MUC15 | 3.26E-01 | 1 | ANKRD12 | 3.92E-01 | 1 |
| SPAM1 | 3.26E-01 | 1 | ZNF470 | 3.92E-01 | 1 |
| FOXK1 | 3.26E-01 | 1 | MTHFD2 | 3.92E-01 | 1 |
| TCTEX1D | 3.26E-01 | 1 | EIF4G3 | 3.93E-01 | 1 |
| CHEK1 | 3.26E-01 | 1 | ST6GAL1 | 3.93E-01 | 1 |
| CYP2E1 | 3.26E-01 | 1 | LCLAT1 | 3.93E-01 | 1 |
| VIT | 3.26E-01 | 1 | AHR | 3.93E-01 | 1 |
| PTTG1 | 3.26E-01 | 1 | SERPINA9 | 3.93E-01 | 1 |
| WASL | 3.26E-01 | 1 | BRDT | 3.93E-01 | 1 |
| RASGRF1 | 3.26E-01 | 1 | GPRC5C | 3.93E-01 | 1 |
| SYNGR1 | 3.26E-01 | 1 | RBM11 | 3.93E-01 | 1 |
| NUP160 | 3.26E-01 | 1 | WDR87 | 3.93E-01 | 1 |
| KIR2DL3 | 3.26E-01 | 1 | KCNN2 | 3.94E-01 | 1 |
| ZNF804B | 3.26E-01 | 1 | OR51I2 | 3.94E-01 | 1 |
| FOLR1 | 3.26E-01 | 1 | SYT8 | 3.94E-01 | 1 |
| CD38 | 3.26E-01 | 1 | OR10Q1 | 3.94E-01 | 1 |
| SMNDC1 | 3.27E-01 | 1 | RORB | 3.94E-01 | 1 |
| PCDHB12 | 3.27E-01 | 1 | SLC16A9 | 3.94E-01 | 1 |
| TLE2 | 3.27E-01 | 1 | FCGRT | 3.94E-01 | 1 |
| FAM174A | 3.27E-01 | 1 | ZFAND1 | 3.95E-01 | 1 |
| SETD4 | 3.27E-01 | 1 | GRWD1 | 3.95E-01 | 1 |
| DYDC1 | 3.27E-01 | 1 | FOXI1 | 3.95E-01 | 1 |
| WDR45 | 3.27E-01 | 1 | TRPM3 | 3.95E-01 | 1 |
| PPP2CA | 3.27E-01 | 1 | DNAJB1 | 3.95E-01 | 1 |

| AK3 | 3.27E-01 | 1 | GNAT3 | 3.95E-01 | 1 |
| --- | --- | --- | --- | --- | --- |
| MSH6 | 3.27E-01 | 1 | RNF157 | 3.96E-01 | 1 |
| LPPR5 | 3.27E-01 | 1 | CLCN7 | 3.96E-01 | 1 |
| CYYR1 | 3.27E-01 | 1 | ATG4D | 3.96E-01 | 1 |
| PUS7L | 3.27E-01 | 1 | SNX33 | 3.96E-01 | 1 |
| AFM | 3.27E-01 | 1 | ZBED1 | 3.96E-01 | 1 |
| IMPACT | 3.28E-01 | 1 | RBM47 | 3.96E-01 | 1 |
| ESX1 | 3.28E-01 | 1 | NWD1 | 3.96E-01 | 1 |
| ZNF70 | 3.28E-01 | 1 | PGD | 3.96E-01 | 1 |
| FGF6 | 3.28E-01 | 1 | STK36 | 3.97E-01 | 1 |
| MYLK2 | 3.28E-01 | 1 | SNAP25 | 3.97E-01 | 1 |
| CEACAM | 3.28E-01 | 1 | NCOA5 | 3.97E-01 | 1 |
| TMCC1 | 3.28E-01 | 1 | IMPDH1 | 3.97E-01 | 1 |
| RASGRF2 | 3.28E-01 | 1 | PCSK4 | 3.97E-01 | 1 |
| ERG | 3.28E-01 | 1 | GPAT2 | 3.97E-01 | 1 |
| CDHR1 | 3.28E-01 | 1 | ACSL1 | 3.97E-01 | 1 |
| KCNJ3 | 3.29E-01 | 1 | MACC1 | 3.97E-01 | 1 |
| CIC | 3.29E-01 | 1 | SPTLC2 | 3.97E-01 | 1 |
| DBP | 3.29E-01 | 1 | HOXB2 | 3.97E-01 | 1 |
| GAB2 | 3.29E-01 | 1 | GNA15 | 3.97E-01 | 1 |
| RBM47 | 3.29E-01 | 1 | C1QTNF9B | 3.97E-01 | 1 |
| EXOC1 | 3.29E-01 | 1 | ZNF292 | 3.97E-01 | 1 |
| MIB2 | 3.29E-01 | 1 | WDR37 | 3.98E-01 | 1 |
| H6PD | 3.29E-01 | 1 | JMJD6 | 3.98E-01 | 1 |
| FTHL17 | 3.29E-01 | 1 | ZBTB43 | 3.98E-01 | 1 |
| INPP4B | 3.30E-01 | 1 | VAV1 | 3.98E-01 | 1 |
| CCR5 | 3.30E-01 | 1 | MAGEE1 | 3.98E-01 | 1 |
| ARPC1B | 3.30E-01 | 1 | GRM8 | 3.98E-01 | 1 |
| IRAK3 | 3.30E-01 | 1 | HPS6 | 3.98E-01 | 1 |
| SLC25A10 | 3.30E-01 | 1 | GRIN2D | 3.98E-01 | 1 |
| TM2D1 | 3.30E-01 | 1 | PADI4 | 3.98E-01 | 1 |
| RNASE6 | 3.30E-01 | 1 | FAM153B | 3.98E-01 | 1 |
| HSPA13 | 3.30E-01 | 1 | TCP11L1 | 3.98E-01 | 1 |
| HTRA2 | 3.30E-01 | 1 | PXK | 3.99E-01 | 1 |
| C3AR1 | 3.30E-01 | 1 | FNBP1 | 3.99E-01 | 1 |
| TMC7 | 3.30E-01 | 1 | ATOH1 | 3.99E-01 | 1 |
| ZNF22 | 3.30E-01 | 1 | ADAMTS14 | 3.99E-01 | 1 |
| MORF4L2 | 3.30E-01 | 1 | TNFSF10 | 3.99E-01 | 1 |
| SEC23A | 3.30E-01 | 1 | PNLDC1 | 3.99E-01 | 1 |
| SPATA16 | 3.30E-01 | 1 | NPAS4 | 3.99E-01 | 1 |
| CBFA2T2 | 3.30E-01 | 1 | STAG2 | 3.99E-01 | 1 |
| EEA1 | 3.30E-01 | 1 | LRRC8D | 3.99E-01 | 1 |
| ADAM23 | 3.30E-01 | 1 | RNF149 | 3.99E-01 | 1 |
| KLHL13 | 3.31E-01 | 1 | TMEFF2 | 3.99E-01 | 1 |
| CLK3 | 3.31E-01 | 1 | SUMF2 | 4.00E-01 | 1 |
| NOTO | 3.31E-01 | 1 | PLCL2 | 4.00E-01 | 1 |
| FNDC8 | 3.31E-01 | 1 | CPT1B | 4.00E-01 | 1 |
| CTDSP1 | 3.31E-01 | 1 | RBM34 | 4.00E-01 | 1 |
| VPS16 | 3.31E-01 | 1 | EFCAB7 | 4.00E-01 | 1 |
| HS1BP3 | 3.31E-01 | 1 | NTNG2 | 4.00E-01 | 1 |
| KLK7 | 3.31E-01 | 1 | PHF20L1 | 4.00E-01 | 1 |
| GPR137 | 3.31E-01 | 1 | KIAA2018 | 4.00E-01 | 1 |
| WDR33 | 3.31E-01 | 1 | NAP1L2 | 4.00E-01 | 1 |

| KLK10 | 3.31E-01 | 1 | WAPAL | 4.00E-01 | 1 |
| --- | --- | --- | --- | --- | --- |
| TMEM101 | 3.31E-01 | 1 | CDCA7 | 4.01E-01 | 1 |
| STUB1 | 3.31E-01 | 1 | QPCTL | 4.01E-01 | 1 |
| GATM | 3.31E-01 | 1 | PTPN22 | 4.01E-01 | 1 |
| GABRG3 | 3.32E-01 | 1 | ZDHHC20 | 4.01E-01 | 1 |
| CAMLG | 3.32E-01 | 1 | C1S | 4.01E-01 | 1 |
| FAM71A | 3.32E-01 | 1 | NTRK2 | 4.01E-01 | 1 |
| SIX2 | 3.32E-01 | 1 | DACT1 | 4.02E-01 | 1 |
| CEP57 | 3.32E-01 | 1 | ZNF451 | 4.02E-01 | 1 |
| TAF4B | 3.32E-01 | 1 | ISG20L2 | 4.02E-01 | 1 |
| SPHK1 | 3.32E-01 | 1 | SLCO1C1 | 4.02E-01 | 1 |
| C11orf70 | 3.33E-01 | 1 | ADH4 | 4.02E-01 | 1 |
| SAMD11 | 3.33E-01 | 1 | ALG3 | 4.02E-01 | 1 |
| DBX2 | 3.33E-01 | 1 | COL15A1 | 4.02E-01 | 1 |
| CD82 | 3.33E-01 | 1 | FCHO1 | 4.03E-01 | 1 |
| SEC31A | 3.33E-01 | 1 | CACNA1D | 4.03E-01 | 1 |
| PRDX4 | 3.33E-01 | 1 | ANKRD18A | 4.04E-01 | 1 |
| C4orf19 | 3.33E-01 | 1 | HEPACAM | 4.04E-01 | 1 |
| ZNF385B | 3.33E-01 | 1 | TIGD2 | 4.04E-01 | 1 |
| SLC38A9 | 3.34E-01 | 1 | FGA | 4.04E-01 | 1 |
| E2F1 | 3.34E-01 | 1 | ZDHHC21 | 4.04E-01 | 1 |
| RCHY1 | 3.34E-01 | 1 | ARNT2 | 4.04E-01 | 1 |
| TIGD5 | 3.34E-01 | 1 | MGAT1 | 4.04E-01 | 1 |
| AKIRIN2 | 3.34E-01 | 1 | SEL1L3 | 4.04E-01 | 1 |
| ARHGAP9 | 3.34E-01 | 1 | WTAP | 4.04E-01 | 1 |
| ZNF691 | 3.34E-01 | 1 | PCDHA8 | 4.04E-01 | 1 |
| BSCL2 | 3.34E-01 | 1 | RNF220 | 4.04E-01 | 1 |
| SFXN5 | 3.34E-01 | 1 | CCNDBP1 | 4.04E-01 | 1 |
| APOD | 3.35E-01 | 1 | PRKACG | 4.04E-01 | 1 |
| LILRA1 | 3.35E-01 | 1 | TUBAL3 | 4.04E-01 | 1 |
| PXDN | 3.35E-01 | 1 | PDE4C | 4.05E-01 | 1 |
| PENK | 3.35E-01 | 1 | PTGIR | 4.05E-01 | 1 |
| ISLR2 | 3.35E-01 | 1 | TNNT3 | 4.05E-01 | 1 |
| ERC1 | 3.35E-01 | 1 | KIAA1107 | 4.05E-01 | 1 |
| ZNF584 | 3.35E-01 | 1 | CACNB1 | 4.05E-01 | 1 |
| CLEC6A | 3.35E-01 | 1 | ARMCX6 | 4.05E-01 | 1 |
| ESRP1 | 3.35E-01 | 1 | GALNT14 | 4.05E-01 | 1 |
| ALDH16A | 3.35E-01 | 1 | ARID5B | 4.05E-01 | 1 |
| RAB15 | 3.36E-01 | 1 | CREB3L4 | 4.05E-01 | 1 |
| PDCD7 | 3.36E-01 | 1 | IL2RG | 4.06E-01 | 1 |
| NLGN4X | 3.36E-01 | 1 | CPXCR1 | 4.06E-01 | 1 |
| GPR19 | 3.36E-01 | 1 | OR6B1 | 4.06E-01 | 1 |
| PUS10 | 3.36E-01 | 1 | C2CD2 | 4.06E-01 | 1 |
| ZDHHC18 | 3.36E-01 | 1 | ADH1A | 4.06E-01 | 1 |
| SHOC2 | 3.36E-01 | 1 | HGF | 4.06E-01 | 1 |
| SLC25A34 | 3.36E-01 | 1 | ATF7IP | 4.06E-01 | 1 |
| PTGER1 | 3.36E-01 | 1 | TIGD5 | 4.06E-01 | 1 |
| FAM110A | 3.36E-01 | 1 | RUNX3 | 4.06E-01 | 1 |
| SMPD4 | 3.36E-01 | 1 | NTRK3 | 4.07E-01 | 1 |
| TMEM132 | 3.36E-01 | 1 | CD1D | 4.07E-01 | 1 |
| FAM101B | 3.36E-01 | 1 | PTPN9 | 4.07E-01 | 1 |
| OTOL1 | 3.37E-01 | 1 | HECTD2 | 4.07E-01 | 1 |
| TRAM1L1 | 3.37E-01 | 1 | LRCH3 | 4.07E-01 | 1 |

| CPVL | 3.37E-01 | 1 | CCNL1 | 4.07E-01 | 1 |
| --- | --- | --- | --- | --- | --- |
| SYNGR3 | 3.37E-01 | 1 | METRNL | 4.07E-01 | 1 |
| NLRC4 | 3.37E-01 | 1 | FLOT2 | 4.08E-01 | 1 |
| KRT20 | 3.37E-01 | 1 | OR5M9 | 4.08E-01 | 1 |
| GFPT1 | 3.37E-01 | 1 | SLC4A5 | 4.08E-01 | 1 |
| SSH3 | 3.37E-01 | 1 | MMP15 | 4.08E-01 | 1 |
| SMYD1 | 3.38E-01 | 1 | CLIP3 | 4.08E-01 | 1 |
| WBSCR27 | 3.38E-01 | 1 | TOMM70A | 4.08E-01 | 1 |
| SUDS3 | 3.38E-01 | 1 | TMEM74 | 4.08E-01 | 1 |
| FASTKD2 | 3.38E-01 | 1 | COL17A1 | 4.08E-01 | 1 |
| KRTAP9-9 | 3.38E-01 | 1 | TNFRSF1A | 4.08E-01 | 1 |
| EIF4E1B | 3.38E-01 | 1 | SCARA5 | 4.08E-01 | 1 |
| MRPL13 | 3.38E-01 | 1 | GABPB2 | 4.08E-01 | 1 |
| KCNA5 | 3.38E-01 | 1 | FKRP | 4.08E-01 | 1 |
| TM7SF3 | 3.38E-01 | 1 | FNDC3B | 4.09E-01 | 1 |
| SMARCD | 3.38E-01 | 1 | TTC3 | 4.09E-01 | 1 |
| OR10Q1 | 3.38E-01 | 1 | ZNF791 | 4.09E-01 | 1 |
| CRADD | 3.38E-01 | 1 | HOXC8 | 4.09E-01 | 1 |
| TTLL7 | 3.38E-01 | 1 | OR2W3 | 4.09E-01 | 1 |
| OR6X1 | 3.39E-01 | 1 | GARS | 4.09E-01 | 1 |
| DYNC1I2 | 3.39E-01 | 1 | GAN | 4.10E-01 | 1 |
| C1QL2 | 3.39E-01 | 1 | PTPN21 | 4.10E-01 | 1 |
| KIAA1522 | 3.39E-01 | 1 | PIGW | 4.10E-01 | 1 |
| WBP2 | 3.39E-01 | 1 | FILIP1L | 4.10E-01 | 1 |
| DHRS13 | 3.39E-01 | 1 | HADHB | 4.10E-01 | 1 |
| PIPOX | 3.39E-01 | 1 | ANO1 | 4.10E-01 | 1 |
| ARHGAP1 | 3.39E-01 | 1 | TRIM55 | 4.10E-01 | 1 |
| C16orf54 | 3.39E-01 | 1 | AKR1C1 | 4.10E-01 | 1 |
| SCAMP2 | 3.40E-01 | 1 | RASGRF1 | 4.10E-01 | 1 |
| PROC | 3.40E-01 | 1 | CMKLR1 | 4.10E-01 | 1 |
| FAM13B | 3.40E-01 | 1 | PTAR1 | 4.10E-01 | 1 |
| FBXO4 | 3.40E-01 | 1 | SYT17 | 4.10E-01 | 1 |
| CRX | 3.40E-01 | 1 | GPR61 | 4.11E-01 | 1 |
| PPP2R5D | 3.40E-01 | 1 | HEATR6 | 4.11E-01 | 1 |
| WNT8B | 3.40E-01 | 1 | LAMB2 | 4.11E-01 | 1 |
| PRC1 | 3.40E-01 | 1 | MEN1 | 4.11E-01 | 1 |
| OSR2 | 3.40E-01 | 1 | CKMT1B | 4.11E-01 | 1 |
| DHX9 | 3.40E-01 | 1 | CPSF4 | 4.11E-01 | 1 |
| C17orf59 | 3.40E-01 | 1 | T | 4.11E-01 | 1 |
| CRAMP1L | 3.40E-01 | 1 | ALDH18A1 | 4.11E-01 | 1 |
| SLC30A10 | 3.40E-01 | 1 | PRCC | 4.11E-01 | 1 |
| ALX4 | 3.40E-01 | 1 | TMCC3 | 4.12E-01 | 1 |
| ARPC1A | 3.41E-01 | 1 | PCM1 | 4.12E-01 | 1 |
| TP53RK | 3.41E-01 | 1 | MASTL | 4.12E-01 | 1 |
| RFESD | 3.41E-01 | 1 | CROCC | 4.12E-01 | 1 |
| KNG1 | 3.41E-01 | 1 | SPIRE1 | 4.12E-01 | 1 |
| RBBP6 | 3.41E-01 | 1 | NCAPG | 4.12E-01 | 1 |
| RHBDL2 | 3.41E-01 | 1 | GRAP2 | 4.12E-01 | 1 |
| CD9 | 3.41E-01 | 1 | SAMD4A | 4.12E-01 | 1 |
| SPIRE2 | 3.41E-01 | 1 | SLC1A7 | 4.12E-01 | 1 |
| FEM1A | 3.41E-01 | 1 | EZH1 | 4.12E-01 | 1 |
| KCNS1 | 3.41E-01 | 1 | LACE1 | 4.13E-01 | 1 |
| PSTPIP1 | 3.41E-01 | 1 | PAX2 | 4.13E-01 | 1 |

| OLA1 | 3.41E-01 | 1 | DFNB59 | 4.13E-01 | 1 |
| --- | --- | --- | --- | --- | --- |
| ZNF317 | 3.41E-01 | 1 | DNAJB7 | 4.13E-01 | 1 |
| C14orf105 | 3.41E-01 | 1 | RHOT1 | 4.13E-01 | 1 |
| EGFL8 | 3.41E-01 | 1 | ADAM8 | 4.13E-01 | 1 |
| TUSC3 | 3.41E-01 | 1 | SF3A2 | 4.13E-01 | 1 |
| SCFD1 | 3.42E-01 | 1 | NARS2 | 4.13E-01 | 1 |
| TNFSF18 | 3.42E-01 | 1 | DLG5 | 4.13E-01 | 1 |
| CAMK2B | 3.42E-01 | 1 | NDRG1 | 4.13E-01 | 1 |
| MSLN | 3.42E-01 | 1 | STYK1 | 4.13E-01 | 1 |
| STX18 | 3.42E-01 | 1 | ASB5 | 4.14E-01 | 1 |
| TUBGCP4 | 3.42E-01 | 1 | RTN1 | 4.14E-01 | 1 |
| EFNA3 | 3.42E-01 | 1 | EXO1 | 4.14E-01 | 1 |
| SCRT1 | 3.42E-01 | 1 | ADAMTS19 | 4.14E-01 | 1 |
| SARS2 | 3.42E-01 | 1 | FZD7 | 4.14E-01 | 1 |
| GDF9 | 3.42E-01 | 1 | MLH1 | 4.14E-01 | 1 |
| TMEM173 | 3.43E-01 | 1 | DLG2 | 4.14E-01 | 1 |
| IL34 | 3.43E-01 | 1 | RFC2 | 4.14E-01 | 1 |
| PGM2 | 3.43E-01 | 1 | KLHDC3 | 4.14E-01 | 1 |
| GIMAP5 | 3.43E-01 | 1 | LRRC4C | 4.15E-01 | 1 |
| C9orf152 | 3.43E-01 | 1 | LMF1 | 4.15E-01 | 1 |
| RUNX1T1 | 3.43E-01 | 1 | HIP1 | 4.15E-01 | 1 |
| MDGA1 | 3.43E-01 | 1 | CP | 4.15E-01 | 1 |
| ARHGAP2 | 3.43E-01 | 1 | CSNK1A1 | 4.15E-01 | 1 |
| ZIM3 | 3.43E-01 | 1 | ANAPC2 | 4.15E-01 | 1 |
| APLNR | 3.43E-01 | 1 | OAS2 | 4.16E-01 | 1 |
| NAT8 | 3.43E-01 | 1 | TMEFF1 | 4.16E-01 | 1 |
| ABHD12B | 3.43E-01 | 1 | PLS1 | 4.16E-01 | 1 |
| TEK | 3.44E-01 | 1 | NOV | 4.16E-01 | 1 |
| DAAM1 | 3.44E-01 | 1 | SLC45A3 | 4.16E-01 | 1 |
| CNN3 | 3.44E-01 | 1 | TNPO1 | 4.16E-01 | 1 |
| SFRP5 | 3.44E-01 | 1 | TMC6 | 4.16E-01 | 1 |
| PPP1R7 | 3.44E-01 | 1 | FAM65C | 4.16E-01 | 1 |
| TRIM52 | 3.44E-01 | 1 | LRP12 | 4.16E-01 | 1 |
| PRELID2 | 3.44E-01 | 1 | PTER | 4.16E-01 | 1 |
| CCR10 | 3.44E-01 | 1 | IDO2 | 4.16E-01 | 1 |
| TRIM41 | 3.44E-01 | 1 | WDR49 | 4.16E-01 | 1 |
| FN1 | 3.44E-01 | 1 | SCNN1A | 4.17E-01 | 1 |
| TAF9B | 3.44E-01 | 1 | PSAT1 | 4.17E-01 | 1 |
| PLEKHA1 | 3.44E-01 | 1 | ZNF687 | 4.17E-01 | 1 |
| KRT16 | 3.44E-01 | 1 | SEC14L3 | 4.17E-01 | 1 |
| CHRM2 | 3.44E-01 | 1 | ZNF532 | 4.17E-01 | 1 |
| RNASE2 | 3.44E-01 | 1 | CAMTA2 | 4.17E-01 | 1 |
| TADA1 | 3.45E-01 | 1 | CHGA | 4.18E-01 | 1 |
| CELF2 | 3.45E-01 | 1 | OR5AR1 | 4.18E-01 | 1 |
| KPNA1 | 3.45E-01 | 1 | PGM2L1 | 4.18E-01 | 1 |
| SLC25A44 | 3.45E-01 | 1 | PI4K2A | 4.18E-01 | 1 |
| SPOP | 3.45E-01 | 1 | ZNF585A | 4.18E-01 | 1 |
| PRKCZ | 3.45E-01 | 1 | SCYL2 | 4.19E-01 | 1 |
| C6orf25 | 3.45E-01 | 1 | BNIP3 | 4.19E-01 | 1 |
| ATG10 | 3.45E-01 | 1 | MEIS2 | 4.19E-01 | 1 |
| SDPR | 3.45E-01 | 1 | DDX10 | 4.19E-01 | 1 |
| B3GNT4 | 3.45E-01 | 1 | TCTN3 | 4.19E-01 | 1 |
| KIAA1958 | 3.45E-01 | 1 | TSTD2 | 4.19E-01 | 1 |

| TMEM102 | 3.45E-01 | 1 | NETO2 | 4.19E-01 | 1 |
| --- | --- | --- | --- | --- | --- |
| IFNA8 | 3.46E-01 | 1 | SCNN1D | 4.19E-01 | 1 |
| CAPN9 | 3.46E-01 | 1 | TAF5 | 4.19E-01 | 1 |
| SLC9A2 | 3.46E-01 | 1 | LRTM2 | 4.19E-01 | 1 |
| UBAP2 | 3.46E-01 | 1 | PDCD11 | 4.20E-01 | 1 |
| NR1H3 | 3.46E-01 | 1 | RCOR1 | 4.20E-01 | 1 |
| ABCG4 | 3.46E-01 | 1 | CCNO | 4.20E-01 | 1 |
| INHBE | 3.46E-01 | 1 | SFXN1 | 4.20E-01 | 1 |
| CEP350 | 3.47E-01 | 1 | TSEN2 | 4.20E-01 | 1 |
| VIPR2 | 3.47E-01 | 1 | ERAP2 | 4.20E-01 | 1 |
| DOCK4 | 3.47E-01 | 1 | GRHL2 | 4.21E-01 | 1 |
| XRCC6 | 3.47E-01 | 1 | VLDLR | 4.21E-01 | 1 |
| G2E3 | 3.47E-01 | 1 | CCKBR | 4.21E-01 | 1 |
| CROT | 3.47E-01 | 1 | MGAM | 4.21E-01 | 1 |
| PATL1 | 3.47E-01 | 1 | IPO7 | 4.21E-01 | 1 |
| DUSP2 | 3.47E-01 | 1 | SLC2A7 | 4.21E-01 | 1 |
| CLIP2 | 3.47E-01 | 1 | PHACTR4 | 4.21E-01 | 1 |
| PROM2 | 3.48E-01 | 1 | STK3 | 4.21E-01 | 1 |
| MBOAT7 | 3.48E-01 | 1 | QRFPR | 4.21E-01 | 1 |
| KDM4C | 3.48E-01 | 1 | OR2T12 | 4.21E-01 | 1 |
| TBCD | 3.48E-01 | 1 | ZFYVE26 | 4.22E-01 | 1 |
| PCDHB2 | 3.48E-01 | 1 | USP6 | 4.22E-01 | 1 |
| SCNN1D | 3.48E-01 | 1 | COL20A1 | 4.22E-01 | 1 |
| GREM2 | 3.48E-01 | 1 | SNTB1 | 4.22E-01 | 1 |
| NEDD4L | 3.48E-01 | 1 | MYO18A | 4.22E-01 | 1 |
| C4orf22 | 3.48E-01 | 1 | CEBPZ | 4.22E-01 | 1 |
| G6PC3 | 3.48E-01 | 1 | GLIS1 | 4.22E-01 | 1 |
| P4HA3 | 3.48E-01 | 1 | DYNC1LI1 | 4.22E-01 | 1 |
| FRMD5 | 3.48E-01 | 1 | BRF1 | 4.22E-01 | 1 |
| NR2F2 | 3.49E-01 | 1 | GAPDHS | 4.22E-01 | 1 |
| ROBO4 | 3.49E-01 | 1 | CECR2 | 4.22E-01 | 1 |
| BOLL | 3.49E-01 | 1 | CHRDL2 | 4.22E-01 | 1 |
| VPS36 | 3.49E-01 | 1 | CDH11 | 4.23E-01 | 1 |
| SEMA7A | 3.49E-01 | 1 | RAD9B | 4.23E-01 | 1 |
| ZBTB46 | 3.49E-01 | 1 | SCG2 | 4.23E-01 | 1 |
| LYPD6 | 3.49E-01 | 1 | OR2T4 | 4.23E-01 | 1 |
| ZNF727 | 3.49E-01 | 1 | GHDC | 4.23E-01 | 1 |
| BRIP1 | 3.49E-01 | 1 | ZP3 | 4.23E-01 | 1 |
| 6-Sep | 3.49E-01 | 1 | NEK2 | 4.23E-01 | 1 |
| CNN2 | 3.49E-01 | 1 | TRIM72 | 4.23E-01 | 1 |
| SLC19A2 | 3.49E-01 | 1 | SP140 | 4.23E-01 | 1 |
| PRPF8 | 3.49E-01 | 1 | SREBF2 | 4.23E-01 | 1 |
| EIF2AK4 | 3.49E-01 | 1 | CCDC47 | 4.23E-01 | 1 |
| MOG | 3.49E-01 | 1 | ZMYM4 | 4.23E-01 | 1 |
| GJA1 | 3.49E-01 | 1 | COG8 | 4.23E-01 | 1 |
| ACLY | 3.50E-01 | 1 | KPNA1 | 4.24E-01 | 1 |
| OR6B2 | 3.50E-01 | 1 | GRAMD2 | 4.24E-01 | 1 |
| OR5A1 | 3.50E-01 | 1 | GTPBP2 | 4.24E-01 | 1 |
| DNAJC17 | 3.50E-01 | 1 | ABI3BP | 4.24E-01 | 1 |
| OR10G2 | 3.50E-01 | 1 | PJA2 | 4.24E-01 | 1 |
| PRDX6 | 3.50E-01 | 1 | FAM160A1 | 4.24E-01 | 1 |
| SIDT2 | 3.51E-01 | 1 | TUBA3D | 4.24E-01 | 1 |
| CDC37 | 3.51E-01 | 1 | ZNF214 | 4.24E-01 | 1 |

| NXPH4 | 3.51E-01 | 1 | CXXC1 | 4.24E-01 | 1 |
| --- | --- | --- | --- | --- | --- |
| DLGAP1 | 3.51E-01 | 1 | GPR52 | 4.24E-01 | 1 |
| SLCO2A1 | 3.51E-01 | 1 | CHI3L2 | 4.24E-01 | 1 |
| APEX1 | 3.51E-01 | 1 | PRKCG | 4.24E-01 | 1 |
| TGDS | 3.51E-01 | 1 | XRN2 | 4.24E-01 | 1 |
| CGNL1 | 3.51E-01 | 1 | RPS6KB2 | 4.24E-01 | 1 |
| NUDT12 | 3.51E-01 | 1 | SKAP2 | 4.25E-01 | 1 |
| CXXC1 | 3.51E-01 | 1 | ATXN7L1 | 4.25E-01 | 1 |
| VCL | 3.52E-01 | 1 | CLASP2 | 4.25E-01 | 1 |
| NFATC4 | 3.52E-01 | 1 | MAMDC4 | 4.25E-01 | 1 |
| SMOC1 | 3.52E-01 | 1 | PSAP | 4.25E-01 | 1 |
| FAM163A | 3.52E-01 | 1 | NAP1L3 | 4.25E-01 | 1 |
| KCNIP2 | 3.52E-01 | 1 | AKR1D1 | 4.26E-01 | 1 |
| TNFRSF10 | 3.52E-01 | 1 | CCDC150 | 4.26E-01 | 1 |
| COL22A1 | 3.52E-01 | 1 | CCR1 | 4.26E-01 | 1 |
| GSTP1 | 3.52E-01 | 1 | DGAT1 | 4.26E-01 | 1 |
| CDKL1 | 3.52E-01 | 1 | DBR1 | 4.26E-01 | 1 |
| KRTAP4-5 | 3.52E-01 | 1 | ATP5A1 | 4.27E-01 | 1 |
| KIAA1841 | 3.52E-01 | 1 | PTPN14 | 4.27E-01 | 1 |
| NBAS | 3.53E-01 | 1 | ANKFN1 | 4.27E-01 | 1 |
| PTGS1 | 3.53E-01 | 1 | BCORL1 | 4.27E-01 | 1 |
| OR4D6 | 3.53E-01 | 1 | MEIS1 | 4.27E-01 | 1 |
| HUS1 | 3.53E-01 | 1 | AKR1C4 | 4.27E-01 | 1 |
| FADD | 3.53E-01 | 1 | ZFHX4 | 4.27E-01 | 1 |
| CHCHD2 | 3.53E-01 | 1 | TGFB1 | 4.28E-01 | 1 |
| HMGB2 | 3.53E-01 | 1 | TCP1 | 4.28E-01 | 1 |
| ACAP2 | 3.53E-01 | 1 | CCDC93 | 4.28E-01 | 1 |
| ADRA2A | 3.53E-01 | 1 | ROBO4 | 4.28E-01 | 1 |
| RAB31 | 3.53E-01 | 1 | BTNL3 | 4.28E-01 | 1 |
| TRIM46 | 3.53E-01 | 1 | ERC2 | 4.28E-01 | 1 |
| RLTPR | 3.53E-01 | 1 | ISM1 | 4.29E-01 | 1 |
| AQR | 3.53E-01 | 1 | SLC2A5 | 4.29E-01 | 1 |
| SLC25A42 | 3.53E-01 | 1 | SPATA17 | 4.29E-01 | 1 |
| DAPP1 | 3.53E-01 | 1 | PRPF38B | 4.29E-01 | 1 |
| ABLIM2 | 3.53E-01 | 1 | FOXC1 | 4.29E-01 | 1 |
| HSD17B6 | 3.53E-01 | 1 | SMPDL3B | 4.29E-01 | 1 |
| SUN1 | 3.53E-01 | 1 | RERE | 4.29E-01 | 1 |
| FAM71F2 | 3.54E-01 | 1 | AGPAT9 | 4.29E-01 | 1 |
| LIX1 | 3.54E-01 | 1 | USP17L2 | 4.29E-01 | 1 |
| COBLL1 | 3.54E-01 | 1 | ZNF207 | 4.29E-01 | 1 |
| S1PR3 | 3.54E-01 | 1 | YY1AP1 | 4.29E-01 | 1 |
| USP2 | 3.54E-01 | 1 | IL2RB | 4.30E-01 | 1 |
| MMP21 | 3.54E-01 | 1 | POMT2 | 4.30E-01 | 1 |
| MTMR9 | 3.54E-01 | 1 | TBX2 | 4.30E-01 | 1 |
| FGF3 | 3.54E-01 | 1 | KIAA1671 | 4.30E-01 | 1 |
| CLCN5 | 3.54E-01 | 1 | PDE5A | 4.31E-01 | 1 |
| SOS2 | 3.54E-01 | 1 | CKAP5 | 4.31E-01 | 1 |
| ACOX1 | 3.54E-01 | 1 | NTRK1 | 4.31E-01 | 1 |
| OR4D10 | 3.54E-01 | 1 | MSL2 | 4.31E-01 | 1 |
| CLSTN1 | 3.54E-01 | 1 | FGFR3 | 4.31E-01 | 1 |
| SMARCA | 3.55E-01 | 1 | SMC1A | 4.32E-01 | 1 |
| LAMB2 | 3.55E-01 | 1 | PCDHGB7 | 4.32E-01 | 1 |
| RUFY1 | 3.55E-01 | 1 | PRDM1 | 4.32E-01 | 1 |

| MARS | 3.55E-01 | 1 | C17orf96 | 4.32E-01 | 1 |
| --- | --- | --- | --- | --- | --- |
| HOXC11 | 3.55E-01 | 1 | PPFIA3 | 4.32E-01 | 1 |
| CXCR2 | 3.56E-01 | 1 | GDPD3 | 4.32E-01 | 1 |
| HMGB3 | 3.56E-01 | 1 | IZUMO1 | 4.32E-01 | 1 |
| SLC25A19 | 3.56E-01 | 1 | OR2T8 | 4.33E-01 | 1 |
| PITPNM2 | 3.56E-01 | 1 | OSTM1 | 4.33E-01 | 1 |
| OR2K2 | 3.56E-01 | 1 | PARD3 | 4.33E-01 | 1 |
| UBXN10 | 3.56E-01 | 1 | UPF3B | 4.33E-01 | 1 |
| ENOX2 | 3.56E-01 | 1 | OR51M1 | 4.33E-01 | 1 |
| RPL15 | 3.56E-01 | 1 | SYT11 | 4.33E-01 | 1 |
| MRPL28 | 3.56E-01 | 1 | ZFP28 | 4.33E-01 | 1 |
| LPO | 3.56E-01 | 1 | PLSCR4 | 4.33E-01 | 1 |
| DIRAS2 | 3.56E-01 | 1 | SLC25A21 | 4.33E-01 | 1 |
| STAG2 | 3.56E-01 | 1 | ZFP2 | 4.33E-01 | 1 |
| DCTD | 3.56E-01 | 1 | PCDHB3 | 4.33E-01 | 1 |
| CACNA2D | 3.56E-01 | 1 | UNC13C | 4.33E-01 | 1 |
| ZC3H8 | 3.56E-01 | 1 | CD1E | 4.33E-01 | 1 |
| ZCCHC7 | 3.56E-01 | 1 | CYP11B1 | 4.33E-01 | 1 |
| SLC7A6O | 3.56E-01 | 1 | STX3 | 4.34E-01 | 1 |
| NPM2 | 3.57E-01 | 1 | ZFP42 | 4.34E-01 | 1 |
| NMNAT1 | 3.57E-01 | 1 | ARID3A | 4.34E-01 | 1 |
| KCNAB1 | 3.57E-01 | 1 | OR11L1 | 4.34E-01 | 1 |
| REEP2 | 3.57E-01 | 1 | DCAF6 | 4.34E-01 | 1 |
| NAA15 | 3.57E-01 | 1 | CAMK1G | 4.35E-01 | 1 |
| ACACB | 3.57E-01 | 1 | POC1A | 4.35E-01 | 1 |
| FAM169B | 3.57E-01 | 1 | CAPN9 | 4.35E-01 | 1 |
| TMEM132 | 3.57E-01 | 1 | SNX31 | 4.35E-01 | 1 |
| NSMCE1 | 3.57E-01 | 1 | SLC37A4 | 4.35E-01 | 1 |
| RHBDD2 | 3.57E-01 | 1 | ITIH4 | 4.35E-01 | 1 |
| TMEM17 | 3.57E-01 | 1 | ANKRD53 | 4.35E-01 | 1 |
| SERPINE2 | 3.57E-01 | 1 | SEPN1 | 4.36E-01 | 1 |
| CYP27C1 | 3.57E-01 | 1 | TKT | 4.36E-01 | 1 |
| CDON | 3.57E-01 | 1 | SULT2B1 | 4.36E-01 | 1 |
| GGA2 | 3.57E-01 | 1 | RNPEP | 4.36E-01 | 1 |
| IGSF6 | 3.57E-01 | 1 | TMEM135 | 4.36E-01 | 1 |
| ELN | 3.57E-01 | 1 | ZDHHC14 | 4.36E-01 | 1 |
| TSKU | 3.57E-01 | 1 | PCDHB8 | 4.36E-01 | 1 |
| LRRC18 | 3.58E-01 | 1 | TMEM5 | 4.36E-01 | 1 |
| OR52E8 | 3.58E-01 | 1 | IGSF5 | 4.36E-01 | 1 |
| ASB17 | 3.58E-01 | 1 | TMEM175 | 4.37E-01 | 1 |
| BANK1 | 3.58E-01 | 1 | APC2 | 4.37E-01 | 1 |
| SNX27 | 3.58E-01 | 1 | IGFALS | 4.37E-01 | 1 |
| SPSB2 | 3.58E-01 | 1 | SLC22A15 | 4.37E-01 | 1 |
| TAS2R1 | 3.58E-01 | 1 | HGFAC | 4.37E-01 | 1 |
| ABCG5 | 3.58E-01 | 1 | PRMT2 | 4.37E-01 | 1 |
| HORMAD | 3.59E-01 | 1 | SULT1C3 | 4.37E-01 | 1 |
| HPS3 | 3.59E-01 | 1 | KCNK10 | 4.37E-01 | 1 |
| RPS7 | 3.59E-01 | 1 | SPPL2A | 4.38E-01 | 1 |
| NME5 | 3.59E-01 | 1 | SAMD8 | 4.38E-01 | 1 |
| GNB2L1 | 3.59E-01 | 1 | ARIH2 | 4.38E-01 | 1 |
| RNF128 | 3.59E-01 | 1 | FUT1 | 4.38E-01 | 1 |
| RPN2 | 3.59E-01 | 1 | ZNF714 | 4.38E-01 | 1 |
| FARS2 | 3.59E-01 | 1 | VCL | 4.38E-01 | 1 |

| NFU1 | 3.59E-01 | 1 | DDX5 | 4.38E-01 | 1 |
| --- | --- | --- | --- | --- | --- |
| TG | 3.59E-01 | 1 | CLDN8 | 4.38E-01 | 1 |
| TMED7-T | 3.59E-01 | 1 | EPHA3 | 4.39E-01 | 1 |
| FBLN1 | 3.59E-01 | 1 | TRIM28 | 4.39E-01 | 1 |
| CCNA1 | 3.59E-01 | 1 | LRFN3 | 4.39E-01 | 1 |
| PAQR3 | 3.59E-01 | 1 | TRIM43 | 4.39E-01 | 1 |
| METTL9 | 3.59E-01 | 1 | MLLT10 | 4.39E-01 | 1 |
| CYP2C18 | 3.59E-01 | 1 | FBXL4 | 4.39E-01 | 1 |
| PHTF2 | 3.60E-01 | 1 | PRR5 | 4.40E-01 | 1 |
| SLC16A5 | 3.60E-01 | 1 | RASGEF1A | 4.40E-01 | 1 |
| GPATCH8 | 3.60E-01 | 1 | SART1 | 4.40E-01 | 1 |
| CLEC4E | 3.60E-01 | 1 | TOM1L2 | 4.40E-01 | 1 |
| FAM57B | 3.60E-01 | 1 | HIST1H4G | 4.40E-01 | 1 |
| TEX2 | 3.60E-01 | 1 | SERTAD4 | 4.40E-01 | 1 |
| JAK1 | 3.60E-01 | 1 | TBX3 | 4.41E-01 | 1 |
| GGCX | 3.60E-01 | 1 | SLC6A4 | 4.41E-01 | 1 |
| OR4K17 | 3.60E-01 | 1 | TRHR | 4.41E-01 | 1 |
| SHANK1 | 3.60E-01 | 1 | RFTN2 | 4.42E-01 | 1 |
| ST5 | 3.60E-01 | 1 | PDK4 | 4.42E-01 | 1 |
| VCX3A | 3.60E-01 | 1 | SLC39A14 | 4.43E-01 | 1 |
| TTC14 | 3.60E-01 | 1 | KLF15 | 4.43E-01 | 1 |
| SLC4A8 | 3.61E-01 | 1 | INTU | 4.43E-01 | 1 |
| ATAD2B | 3.61E-01 | 1 | SOS1 | 4.43E-01 | 1 |
| CSNK1E | 3.61E-01 | 1 | APEH | 4.43E-01 | 1 |
| KIRREL | 3.61E-01 | 1 | GMIP | 4.43E-01 | 1 |
| FBXO18 | 3.61E-01 | 1 | LRMP | 4.43E-01 | 1 |
| CALU | 3.61E-01 | 1 | C6 | 4.44E-01 | 1 |
| B4GALT7 | 3.62E-01 | 1 | TMEM229A | 4.44E-01 | 1 |
| CARTPT | 3.62E-01 | 1 | SGOL1 | 4.44E-01 | 1 |
| ABCB5 | 3.62E-01 | 1 | MAEL | 4.44E-01 | 1 |
| HRH4 | 3.62E-01 | 1 | CSAD | 4.44E-01 | 1 |
| ZNF732 | 3.62E-01 | 1 | ZNF121 | 4.44E-01 | 1 |
| ARHGEF4 | 3.62E-01 | 1 | SH3YL1 | 4.44E-01 | 1 |
| KRT27 | 3.62E-01 | 1 | PHF21B | 4.44E-01 | 1 |
| OR5AU1 | 3.62E-01 | 1 | NXF1 | 4.44E-01 | 1 |
| C2CD2L | 3.62E-01 | 1 | BDNF | 4.44E-01 | 1 |
| ZMYND19 | 3.62E-01 | 1 | BDKRB2 | 4.44E-01 | 1 |
| GIMAP7 | 3.62E-01 | 1 | KIAA1958 | 4.45E-01 | 1 |
| EDEM2 | 3.62E-01 | 1 | TMEM132B | 4.45E-01 | 1 |
| MAPK10 | 3.63E-01 | 1 | SLC24A3 | 4.45E-01 | 1 |
| ISG20L2 | 3.63E-01 | 1 | DENND3 | 4.45E-01 | 1 |
| UPK1A | 3.63E-01 | 1 | OR52N1 | 4.45E-01 | 1 |
| SIRPB2 | 3.63E-01 | 1 | SSTR3 | 4.45E-01 | 1 |
| C16orf13 | 3.63E-01 | 1 | CABIN1 | 4.45E-01 | 1 |
| HAVCR1 | 3.63E-01 | 1 | HTR1D | 4.46E-01 | 1 |
| MAG | 3.63E-01 | 1 | GPR85 | 4.46E-01 | 1 |
| CTAG2 | 3.63E-01 | 1 | CLPTM1 | 4.46E-01 | 1 |
| OR5F1 | 3.63E-01 | 1 | DNASE2 | 4.46E-01 | 1 |
| GRAMD2 | 3.63E-01 | 1 | DUS1L | 4.46E-01 | 1 |
| RGS14 | 3.63E-01 | 1 | CEP76 | 4.46E-01 | 1 |
| TK1 | 3.63E-01 | 1 | GABPB1 | 4.46E-01 | 1 |
| GRTP1 | 3.63E-01 | 1 | KLHL36 | 4.46E-01 | 1 |
| AFF2 | 3.63E-01 | 1 | MYT1L | 4.46E-01 | 1 |

| RNF114 | 3.63E-01 | 1 | STAG3 | 4.46E-01 | 1 |
| --- | --- | --- | --- | --- | --- |
| KCNA6 | 3.63E-01 | 1 | SOX13 | 4.47E-01 | 1 |
| RNF151 | 3.64E-01 | 1 | RYR1 | 4.47E-01 | 1 |
| UBE2K | 3.64E-01 | 1 | SLC7A14 | 4.47E-01 | 1 |
| HMGCL | 3.64E-01 | 1 | MRPL3 | 4.47E-01 | 1 |
| P2RY6 | 3.64E-01 | 1 | ZNF74 | 4.47E-01 | 1 |
| RHCE | 3.64E-01 | 1 | ADCY2 | 4.47E-01 | 1 |
| FOXP4 | 3.64E-01 | 1 | C12orf4 | 4.47E-01 | 1 |
| TLR8 | 3.64E-01 | 1 | SP6 | 4.48E-01 | 1 |
| LIMK1 | 3.64E-01 | 1 | KNDC1 | 4.48E-01 | 1 |
| ZNF608 | 3.64E-01 | 1 | OR52E8 | 4.48E-01 | 1 |
| FRMD6 | 3.64E-01 | 1 | XPNPEP2 | 4.48E-01 | 1 |
| MATK | 3.64E-01 | 1 | ZNF2 | 4.48E-01 | 1 |
| ZNF429 | 3.64E-01 | 1 | IRF6 | 4.49E-01 | 1 |
| BCHE | 3.64E-01 | 1 | FAM83A | 4.49E-01 | 1 |
| ZNF627 | 3.64E-01 | 1 | LSG1 | 4.49E-01 | 1 |
| BRD9 | 3.64E-01 | 1 | P2RX6 | 4.49E-01 | 1 |
| NBR1 | 3.64E-01 | 1 | FAM13A | 4.49E-01 | 1 |
| TMEM45B | 3.64E-01 | 1 | TRAK1 | 4.49E-01 | 1 |
| MRPS9 | 3.65E-01 | 1 | RRAGA | 4.49E-01 | 1 |
| VSIG8 | 3.65E-01 | 1 | GBA | 4.49E-01 | 1 |
| KCNB2 | 3.65E-01 | 1 | PRAMEF17 | 4.49E-01 | 1 |
| PSG11 | 3.65E-01 | 1 | ACTR10 | 4.49E-01 | 1 |
| PGAM5 | 3.65E-01 | 1 | SLC29A1 | 4.49E-01 | 1 |
| HMGB4 | 3.65E-01 | 1 | SOX30 | 4.49E-01 | 1 |
| TBC1D9B | 3.65E-01 | 1 | ACAT1 | 4.49E-01 | 1 |
| IFFO1 | 3.65E-01 | 1 | EIF4G2 | 4.50E-01 | 1 |
| OR8I2 | 3.65E-01 | 1 | EFCAB3 | 4.50E-01 | 1 |
| TRPC7 | 3.65E-01 | 1 | RBM45 | 4.50E-01 | 1 |
| CDKAL1 | 3.65E-01 | 1 | DUOXA1 | 4.50E-01 | 1 |
| RAD51 | 3.65E-01 | 1 | OR2H1 | 4.50E-01 | 1 |
| FHOD3 | 3.65E-01 | 1 | GAB1 | 4.51E-01 | 1 |
| REV1 | 3.65E-01 | 1 | ZNF223 | 4.51E-01 | 1 |
| DMBX1 | 3.65E-01 | 1 | PNPLA5 | 4.51E-01 | 1 |
| TMEM214 | 3.66E-01 | 1 | LDB2 | 4.51E-01 | 1 |
| LDLRAD3 | 3.66E-01 | 1 | TMEM8B | 4.51E-01 | 1 |
| DRD2 | 3.66E-01 | 1 | PLCG2 | 4.52E-01 | 1 |
| TXNIP | 3.66E-01 | 1 | ST6GALNA | 4.52E-01 | 1 |
| LGR6 | 3.66E-01 | 1 | ZFYVE9 | 4.52E-01 | 1 |
| GPC4 | 3.66E-01 | 1 | TOE1 | 4.52E-01 | 1 |
| RAPGEF1 | 3.66E-01 | 1 | FAT3 | 4.53E-01 | 1 |
| SNW1 | 3.66E-01 | 1 | MNDA | 4.53E-01 | 1 |
| ZPLD1 | 3.66E-01 | 1 | FPR1 | 4.53E-01 | 1 |
| EOMES | 3.66E-01 | 1 | CDC14B | 4.53E-01 | 1 |
| C5orf45 | 3.66E-01 | 1 | ZNF324B | 4.53E-01 | 1 |
| LHPP | 3.67E-01 | 1 | FPR2 | 4.54E-01 | 1 |
| CD68 | 3.67E-01 | 1 | MYH15 | 4.54E-01 | 1 |
| TBX2 | 3.67E-01 | 1 | EIF3B | 4.54E-01 | 1 |
| FBXO44 | 3.67E-01 | 1 | APCDD1L | 4.54E-01 | 1 |
| VCAM1 | 3.67E-01 | 1 | OR2AE1 | 4.55E-01 | 1 |
| NUBPL | 3.67E-01 | 1 | ACTR6 | 4.55E-01 | 1 |
| ANKS1A | 3.67E-01 | 1 | PTK2 | 4.55E-01 | 1 |
| LIPN | 3.67E-01 | 1 | ZFP57 | 4.55E-01 | 1 |

| SLC9A7 | 3.67E-01 | 1 | ENGASE | 4.55E-01 | 1 |
| --- | --- | --- | --- | --- | --- |
| ZNF583 | 3.67E-01 | 1 | GDF5 | 4.55E-01 | 1 |
| EXOC3L2 | 3.68E-01 | 1 | GALNT12 | 4.55E-01 | 1 |
| KRTAP10 | 3.68E-01 | 1 | XKR6 | 4.55E-01 | 1 |
| KDM4D | 3.68E-01 | 1 | PGLYRP3 | 4.55E-01 | 1 |
| OR2T1 | 3.68E-01 | 1 | CEP68 | 4.55E-01 | 1 |
| MRPL30 | 3.68E-01 | 1 | MAB21L1 | 4.55E-01 | 1 |
| SYTL3 | 3.68E-01 | 1 | BMPR1A | 4.55E-01 | 1 |
| RC3H2 | 3.68E-01 | 1 | TH | 4.55E-01 | 1 |
| MS4A14 | 3.68E-01 | 1 | SEC31B | 4.55E-01 | 1 |
| BCL11B | 3.68E-01 | 1 | SACM1L | 4.56E-01 | 1 |
| FLOT2 | 3.68E-01 | 1 | MDM2 | 4.56E-01 | 1 |
| GPR162 | 3.68E-01 | 1 | SMC4 | 4.56E-01 | 1 |
| TXK | 3.68E-01 | 1 | ACAP2 | 4.56E-01 | 1 |
| ZBTB6 | 3.68E-01 | 1 | OR11A1 | 4.56E-01 | 1 |
| FAM20B | 3.68E-01 | 1 | RETSAT | 4.56E-01 | 1 |
| YAF2 | 3.68E-01 | 1 | GJD4 | 4.56E-01 | 1 |
| PRSS16 | 3.68E-01 | 1 | MS4A6E | 4.57E-01 | 1 |
| CSN3 | 3.69E-01 | 1 | PHGDH | 4.57E-01 | 1 |
| C11orf40 | 3.69E-01 | 1 | OR5H1 | 4.57E-01 | 1 |
| FBN1 | 3.69E-01 | 1 | FCHSD1 | 4.57E-01 | 1 |
| CDX2 | 3.69E-01 | 1 | ZNF711 | 4.57E-01 | 1 |
| LIM2 | 3.69E-01 | 1 | FAM135A | 4.57E-01 | 1 |
| PCDHA7 | 3.69E-01 | 1 | SYN3 | 4.57E-01 | 1 |
| PEBP1 | 3.69E-01 | 1 | SUV420H1 | 4.57E-01 | 1 |
| ROM1 | 3.69E-01 | 1 | PDCD1 | 4.57E-01 | 1 |
| ALPK2 | 3.69E-01 | 1 | PLCD3 | 4.57E-01 | 1 |
| HUNK | 3.69E-01 | 1 | CTSE | 4.58E-01 | 1 |
| ITM2B | 3.69E-01 | 1 | XPO5 | 4.58E-01 | 1 |
| SLC14A2 | 3.69E-01 | 1 | SPOP | 4.58E-01 | 1 |
| PRTG | 3.70E-01 | 1 | SLC45A4 | 4.58E-01 | 1 |
| KRTAP5-1 | 3.70E-01 | 1 | SH3BP2 | 4.58E-01 | 1 |
| NKAP | 3.70E-01 | 1 | TRAP1 | 4.59E-01 | 1 |
| NRXN1 | 3.70E-01 | 1 | ST8SIA4 | 4.59E-01 | 1 |
| LINGO1 | 3.70E-01 | 1 | PIWIL2 | 4.59E-01 | 1 |
| TWIST1 | 3.70E-01 | 1 | PLCH1 | 4.59E-01 | 1 |
| ASTN2 | 3.70E-01 | 1 | LCA5 | 4.59E-01 | 1 |
| FAM161B | 3.70E-01 | 1 | UPRT | 4.59E-01 | 1 |
| FILIP1L | 3.70E-01 | 1 | EIF3E | 4.59E-01 | 1 |
| GNB1L | 3.70E-01 | 1 | ELAVL3 | 4.59E-01 | 1 |
| PRKG1 | 3.70E-01 | 1 | FAM90A1 | 4.59E-01 | 1 |
| OR6B3 | 3.70E-01 | 1 | SBNO1 | 4.59E-01 | 1 |
| HDGFRP2 | 3.70E-01 | 1 | ATP11B | 4.60E-01 | 1 |
| POPDC2 | 3.70E-01 | 1 | SIRT2 | 4.60E-01 | 1 |
| RPP38 | 3.70E-01 | 1 | LINGO2 | 4.60E-01 | 1 |
| ECH1 | 3.71E-01 | 1 | CHN2 | 4.60E-01 | 1 |
| CUX2 | 3.71E-01 | 1 | CADM1 | 4.60E-01 | 1 |
| PEX14 | 3.71E-01 | 1 | DZIP3 | 4.60E-01 | 1 |
| CDC40 | 3.71E-01 | 1 | DMBX1 | 4.61E-01 | 1 |
| C1orf35 | 3.71E-01 | 1 | PELI3 | 4.61E-01 | 1 |
| CRLF2 | 3.71E-01 | 1 | GUF1 | 4.61E-01 | 1 |
| TAF1L | 3.71E-01 | 1 | CCHCR1 | 4.61E-01 | 1 |
| KRT26 | 3.71E-01 | 1 | ITGA11 | 4.61E-01 | 1 |

| TSC1 | 3.71E-01 | 1 | SPAG4 | 4.61E-01 | 1 |
| --- | --- | --- | --- | --- | --- |
| ACE2 | 3.71E-01 | 1 | ZDHHC2 | 4.61E-01 | 1 |
| GLRA3 | 3.71E-01 | 1 | HOMER1 | 4.61E-01 | 1 |
| CORO2B | 3.72E-01 | 1 | CBWD1 | 4.61E-01 | 1 |
| SHB | 3.72E-01 | 1 | ATP1B4 | 4.61E-01 | 1 |
| GPR35 | 3.72E-01 | 1 | ITGA10 | 4.62E-01 | 1 |
| ARTN | 3.72E-01 | 1 | POU6F2 | 4.62E-01 | 1 |
| SNX18 | 3.72E-01 | 1 | ITGA1 | 4.62E-01 | 1 |
| OR2B3 | 3.72E-01 | 1 | IVNS1ABP | 4.63E-01 | 1 |
| DAXX | 3.72E-01 | 1 | ARHGEF2 | 4.63E-01 | 1 |
| GALNT7 | 3.72E-01 | 1 | PLEKHA1 | 4.63E-01 | 1 |
| LRRTM1 | 3.72E-01 | 1 | ZNF781 | 4.63E-01 | 1 |
| SYT11 | 3.72E-01 | 1 | MAP3K14 | 4.63E-01 | 1 |
| EEF1E1 | 3.72E-01 | 1 | NOMO1 | 4.63E-01 | 1 |
| SLC11A2 | 3.72E-01 | 1 | OR7E24 | 4.63E-01 | 1 |
| MPPED1 | 3.72E-01 | 1 | SYNGAP1 | 4.63E-01 | 1 |
| IFNA13 | 3.72E-01 | 1 | SF3B1 | 4.64E-01 | 1 |
| MTHFD1 | 3.72E-01 | 1 | KCNK3 | 4.64E-01 | 1 |
| TRPC4AP | 3.72E-01 | 1 | CCDC87 | 4.64E-01 | 1 |
| GUCY1B3 | 3.73E-01 | 1 | GJA5 | 4.64E-01 | 1 |
| GHSR | 3.73E-01 | 1 | EPHX1 | 4.64E-01 | 1 |
| ERI3 | 3.73E-01 | 1 | GPM6B | 4.64E-01 | 1 |
| TNRC18 | 3.73E-01 | 1 | C22orf46 | 4.65E-01 | 1 |
| EN2 | 3.73E-01 | 1 | MAGED1 | 4.65E-01 | 1 |
| C1orf226 | 3.73E-01 | 1 | SF3B3 | 4.65E-01 | 1 |
| NAPB | 3.73E-01 | 1 | OTOF | 4.65E-01 | 1 |
| GRK4 | 3.73E-01 | 1 | B3GAT1 | 4.65E-01 | 1 |
| ENOX1 | 3.73E-01 | 1 | LPPR3 | 4.65E-01 | 1 |
| TCEA3 | 3.73E-01 | 1 | UBAP2 | 4.65E-01 | 1 |
| GMPS | 3.74E-01 | 1 | TRAF3IP1 | 4.65E-01 | 1 |
| TIAM2 | 3.74E-01 | 1 | RAF1 | 4.65E-01 | 1 |
| PF4V1 | 3.74E-01 | 1 | PHF8 | 4.66E-01 | 1 |
| NOP2 | 3.74E-01 | 1 | LIPK | 4.66E-01 | 1 |
| KAT2B | 3.74E-01 | 1 | EDC3 | 4.66E-01 | 1 |
| YWHAH | 3.74E-01 | 1 | BMP7 | 4.66E-01 | 1 |
| AFAP1 | 3.74E-01 | 1 | NMBR | 4.66E-01 | 1 |
| PCDHA5 | 3.74E-01 | 1 | UGT2B7 | 4.66E-01 | 1 |
| RCAN1 | 3.74E-01 | 1 | PTPRH | 4.66E-01 | 1 |
| EPPK1 | 3.74E-01 | 1 | KCNS1 | 4.67E-01 | 1 |
| CACHD1 | 3.75E-01 | 1 | OR2L2 | 4.67E-01 | 1 |
| PAPOLB | 3.75E-01 | 1 | RAB11FIP4 | 4.67E-01 | 1 |
| TSPAN18 | 3.75E-01 | 1 | BECN1 | 4.67E-01 | 1 |
| STAT4 | 3.75E-01 | 1 | XRRA1 | 4.67E-01 | 1 |
| IL7 | 3.75E-01 | 1 | ABCG4 | 4.68E-01 | 1 |
| ZC3H12C | 3.75E-01 | 1 | CCT7 | 4.68E-01 | 1 |
| SIGIRR | 3.75E-01 | 1 | BRD9 | 4.68E-01 | 1 |
| PRPF40A | 3.75E-01 | 1 | ATP10D | 4.68E-01 | 1 |
| CCDC67 | 3.75E-01 | 1 | EPN1 | 4.68E-01 | 1 |
| CIZ1 | 3.75E-01 | 1 | TMEM194A | 4.68E-01 | 1 |
| ISL2 | 3.75E-01 | 1 | FOXD2 | 4.68E-01 | 1 |
| KCNK13 | 3.75E-01 | 1 | TOM1 | 4.69E-01 | 1 |
| 12-Sep | 3.75E-01 | 1 | FAM117A | 4.69E-01 | 1 |
| SPACA3 | 3.76E-01 | 1 | CPA5 | 4.69E-01 | 1 |

| KBTBD12 | 3.76E-01 | 1 | RHOBTB3 | 4.69E-01 | 1 |
| --- | --- | --- | --- | --- | --- |
| GARNL3 | 3.76E-01 | 1 | PNKP | 4.70E-01 | 1 |
| PADI2 | 3.76E-01 | 1 | SMPD4 | 4.70E-01 | 1 |
| NEUROD4 | 3.76E-01 | 1 | SYNJ1 | 4.70E-01 | 1 |
| BEND5 | 3.76E-01 | 1 | APOBEC4 | 4.70E-01 | 1 |
| MAGEB2 | 3.76E-01 | 1 | ESYT2 | 4.70E-01 | 1 |
| APOBEC3 | 3.76E-01 | 1 | TOM1L1 | 4.70E-01 | 1 |
| TMEM55B | 3.76E-01 | 1 | GPR152 | 4.70E-01 | 1 |
| CCR7 | 3.76E-01 | 1 | ASNS | 4.70E-01 | 1 |
| CLVS1 | 3.76E-01 | 1 | ACPT | 4.70E-01 | 1 |
| TXLNA | 3.76E-01 | 1 | ARFGEF1 | 4.70E-01 | 1 |
| TRPV3 | 3.76E-01 | 1 | OTUD7B | 4.70E-01 | 1 |
| ST3GAL1 | 3.77E-01 | 1 | C3orf67 | 4.71E-01 | 1 |
| SCYL1 | 3.77E-01 | 1 | MAS1L | 4.71E-01 | 1 |
| PRPF4B | 3.77E-01 | 1 | GNAQ | 4.71E-01 | 1 |
| TRPS1 | 3.77E-01 | 1 | A1BG | 4.71E-01 | 1 |
| LGALS8 | 3.77E-01 | 1 | ABCA12 | 4.72E-01 | 1 |
| DGKQ | 3.77E-01 | 1 | ABCC1 | 4.72E-01 | 1 |
| TIMM50 | 3.77E-01 | 1 | OSBPL5 | 4.72E-01 | 1 |
| FOXP1 | 3.77E-01 | 1 | ADAMTSL | 4.72E-01 | 1 |
| BAZ2B | 3.77E-01 | 1 | CHRNA1 | 4.72E-01 | 1 |
| PCDHA12 | 3.77E-01 | 1 | ZNF222 | 4.72E-01 | 1 |
| CPB1 | 3.78E-01 | 1 | PSG2 | 4.72E-01 | 1 |
| SCN5A | 3.78E-01 | 1 | GPATCH4 | 4.72E-01 | 1 |
| CD80 | 3.78E-01 | 1 | FAM84A | 4.72E-01 | 1 |
| KRT38 | 3.78E-01 | 1 | ITPK1 | 4.73E-01 | 1 |
| ZNF518B | 3.78E-01 | 1 | CPA4 | 4.73E-01 | 1 |
| TIPRL | 3.78E-01 | 1 | PCDHGA1 | 4.73E-01 | 1 |
| JAKMIP1 | 3.78E-01 | 1 | SARDH | 4.73E-01 | 1 |
| LIPJ | 3.78E-01 | 1 | ZNF668 | 4.73E-01 | 1 |
| MAGEA6 | 3.78E-01 | 1 | ITGAL | 4.74E-01 | 1 |
| MYPOP | 3.78E-01 | 1 | SLC2A4 | 4.74E-01 | 1 |
| ZKSCAN1 | 3.78E-01 | 1 | MGAT4A | 4.74E-01 | 1 |
| IFNA6 | 3.78E-01 | 1 | KCNA7 | 4.74E-01 | 1 |
| C2orf43 | 3.78E-01 | 1 | CEP63 | 4.74E-01 | 1 |
| SPAG1 | 3.78E-01 | 1 | ZRSR2 | 4.74E-01 | 1 |
| ZCCHC3 | 3.78E-01 | 1 | PTX3 | 4.74E-01 | 1 |
| IFNA2 | 3.78E-01 | 1 | MRPS5 | 4.74E-01 | 1 |
| SIGLEC12 | 3.79E-01 | 1 | CPB1 | 4.75E-01 | 1 |
| PAM | 3.79E-01 | 1 | ARAF | 4.75E-01 | 1 |
| ZNF479 | 3.79E-01 | 1 | RNASEH2B | 4.75E-01 | 1 |
| PPP1R8 | 3.79E-01 | 1 | TUBA8 | 4.75E-01 | 1 |
| FCN3 | 3.79E-01 | 1 | OR12D3 | 4.75E-01 | 1 |
| PRX | 3.79E-01 | 1 | SPOCK1 | 4.76E-01 | 1 |
| CACNG1 | 3.79E-01 | 1 | USP20 | 4.76E-01 | 1 |
| CEBPE | 3.79E-01 | 1 | DENND4C | 4.76E-01 | 1 |
| RNF133 | 3.79E-01 | 1 | ZNF492 | 4.76E-01 | 1 |
| SLC22A3 | 3.79E-01 | 1 | PHC2 | 4.76E-01 | 1 |
| RHOG | 3.79E-01 | 1 | GPR65 | 4.76E-01 | 1 |
| SYCP2L | 3.79E-01 | 1 | B4GALT5 | 4.76E-01 | 1 |
| TANK | 3.79E-01 | 1 | ANKS3 | 4.76E-01 | 1 |
| PHACTR1 | 3.79E-01 | 1 | AMDHD1 | 4.76E-01 | 1 |
| MAP6D1 | 3.79E-01 | 1 | MICA | 4.76E-01 | 1 |

| SSRP1 | 3.79E-01 | 1 | BCLAF1 | 4.77E-01 | 1 |
| --- | --- | --- | --- | --- | --- |
| ARRDC5 | 3.79E-01 | 1 | DGKG | 4.77E-01 | 1 |
| CACNA2D | 3.80E-01 | 1 | INTS7 | 4.77E-01 | 1 |
| ATP1B3 | 3.80E-01 | 1 | PROKR2 | 4.77E-01 | 1 |
| SLC17A9 | 3.80E-01 | 1 | DDX4 | 4.77E-01 | 1 |
| MAP3K4 | 3.80E-01 | 1 | SLC44A1 | 4.77E-01 | 1 |
| SMURF1 | 3.80E-01 | 1 | RGS12 | 4.77E-01 | 1 |
| KCNA7 | 3.80E-01 | 1 | ATMIN | 4.77E-01 | 1 |
| DECR2 | 3.80E-01 | 1 | HIPK4 | 4.77E-01 | 1 |
| HECTD1 | 3.80E-01 | 1 | ARAP3 | 4.77E-01 | 1 |
| MITF | 3.80E-01 | 1 | ZIC2 | 4.77E-01 | 1 |
| AMMECR | 3.80E-01 | 1 | CHD1 | 4.77E-01 | 1 |
| SGMS1 | 3.81E-01 | 1 | NCDN | 4.77E-01 | 1 |
| CLYBL | 3.81E-01 | 1 | IL13RA2 | 4.78E-01 | 1 |
| TTC29 | 3.81E-01 | 1 | MRPS9 | 4.78E-01 | 1 |
| PRKCQ | 3.81E-01 | 1 | LMAN2L | 4.78E-01 | 1 |
| SMAD9 | 3.81E-01 | 1 | ZNF250 | 4.78E-01 | 1 |
| OR2M7 | 3.81E-01 | 1 | ESYT3 | 4.78E-01 | 1 |
| FAM153B | 3.81E-01 | 1 | TRAF3IP3 | 4.78E-01 | 1 |
| CLPP | 3.81E-01 | 1 | TAT | 4.78E-01 | 1 |
| COQ5 | 3.81E-01 | 1 | VN1R2 | 4.78E-01 | 1 |
| DLGAP4 | 3.82E-01 | 1 | ASB4 | 4.79E-01 | 1 |
| OR2W1 | 3.82E-01 | 1 | GRIN1 | 4.79E-01 | 1 |
| HTR5A | 3.82E-01 | 1 | ANKRD34B | 4.79E-01 | 1 |
| CTSW | 3.82E-01 | 1 | PRB2 | 4.79E-01 | 1 |
| OR1J2 | 3.82E-01 | 1 | MAP3K11 | 4.79E-01 | 1 |
| HOXB6 | 3.82E-01 | 1 | EHBP1 | 4.79E-01 | 1 |
| MAP9 | 3.82E-01 | 1 | ZNF568 | 4.79E-01 | 1 |
| FBXO17 | 3.82E-01 | 1 | NPAS1 | 4.79E-01 | 1 |
| PLG | 3.82E-01 | 1 | SMTN | 4.79E-01 | 1 |
| MOGAT1 | 3.82E-01 | 1 | OR51E1 | 4.79E-01 | 1 |
| TAGLN2 | 3.82E-01 | 1 | OR5V1 | 4.80E-01 | 1 |
| EHD1 | 3.83E-01 | 1 | SUPT6H | 4.80E-01 | 1 |
| ATP9A | 3.83E-01 | 1 | DCP2 | 4.80E-01 | 1 |
| CDH20 | 3.83E-01 | 1 | PTPRT | 4.80E-01 | 1 |
| MKI67 | 3.83E-01 | 1 | TLR3 | 4.80E-01 | 1 |
| ABHD1 | 3.83E-01 | 1 | RB1CC1 | 4.80E-01 | 1 |
| SCARF2 | 3.83E-01 | 1 | ALX1 | 4.80E-01 | 1 |
| ANKS6 | 3.83E-01 | 1 | ANXA10 | 4.80E-01 | 1 |
| ATF7IP | 3.83E-01 | 1 | GPR151 | 4.80E-01 | 1 |
| GPR174 | 3.83E-01 | 1 | COL9A1 | 4.80E-01 | 1 |
| TNFRSF21 | 3.83E-01 | 1 | GALNT7 | 4.81E-01 | 1 |
| ABCB10 | 3.83E-01 | 1 | MBTPS2 | 4.81E-01 | 1 |
| C6orf15 | 3.83E-01 | 1 | SEMA3A | 4.81E-01 | 1 |
| QRICH1 | 3.84E-01 | 1 | SIGLEC14 | 4.81E-01 | 1 |
| APOA1 | 3.84E-01 | 1 | ZNF678 | 4.81E-01 | 1 |
| SLC5A11 | 3.84E-01 | 1 | ZNF518B | 4.81E-01 | 1 |
| CDK3 | 3.84E-01 | 1 | GCN1L1 | 4.81E-01 | 1 |
| DBN1 | 3.84E-01 | 1 | AGBL3 | 4.81E-01 | 1 |
| GPR173 | 3.84E-01 | 1 | NKD1 | 4.81E-01 | 1 |
| TFB1M | 3.84E-01 | 1 | AGA | 4.81E-01 | 1 |
| AZGP1 | 3.84E-01 | 1 | CNOT6 | 4.81E-01 | 1 |
| HDGFRP3 | 3.84E-01 | 1 | PAPD4 | 4.81E-01 | 1 |

| KTN1 | 3.84E-01 | 1 | ZNF423 | 4.81E-01 | 1 |
| --- | --- | --- | --- | --- | --- |
| FAM120A | 3.84E-01 | 1 | TAS1R3 | 4.81E-01 | 1 |
| FFAR1 | 3.84E-01 | 1 | P4HA1 | 4.82E-01 | 1 |
| HAAO | 3.85E-01 | 1 | NEDD4L | 4.82E-01 | 1 |
| GPR139 | 3.85E-01 | 1 | MYOCD | 4.82E-01 | 1 |
| ALKBH5 | 3.85E-01 | 1 | GALNT10 | 4.82E-01 | 1 |
| DGKA | 3.85E-01 | 1 | DPYSL2 | 4.82E-01 | 1 |
| RGS16 | 3.85E-01 | 1 | PLEC | 4.82E-01 | 1 |
| SLC37A1 | 3.85E-01 | 1 | IRAK3 | 4.82E-01 | 1 |
| MAGEB4 | 3.85E-01 | 1 | PKMYT1 | 4.82E-01 | 1 |
| KLHL1 | 3.85E-01 | 1 | TCF7 | 4.83E-01 | 1 |
| COQ2 | 3.85E-01 | 1 | ACTL7A | 4.83E-01 | 1 |
| RBP3 | 3.85E-01 | 1 | VCPIP1 | 4.83E-01 | 1 |
| SGCA | 3.85E-01 | 1 | ZNF563 | 4.83E-01 | 1 |
| OR2M3 | 3.86E-01 | 1 | LRRC37B | 4.83E-01 | 1 |
| WIF1 | 3.86E-01 | 1 | CTPS2 | 4.83E-01 | 1 |
| SPEM1 | 3.86E-01 | 1 | KIAA1549 | 4.83E-01 | 1 |
| BTBD19 | 3.86E-01 | 1 | IL20RA | 4.83E-01 | 1 |
| LRRC69 | 3.86E-01 | 1 | CCDC64B | 4.83E-01 | 1 |
| GIMAP2 | 3.86E-01 | 1 | SPAG8 | 4.83E-01 | 1 |
| TMEM41A | 3.86E-01 | 1 | XPNPEP3 | 4.83E-01 | 1 |
| XKR5 | 3.86E-01 | 1 | ELAVL2 | 4.84E-01 | 1 |
| R3HDML | 3.86E-01 | 1 | PCNX | 4.84E-01 | 1 |
| SSR1 | 3.86E-01 | 1 | EXD2 | 4.84E-01 | 1 |
| CR1 | 3.86E-01 | 1 | 3-Sep | 4.84E-01 | 1 |
| CD46 | 3.86E-01 | 1 | PALM2-AK | 4.84E-01 | 1 |
| SCRIB | 3.86E-01 | 1 | GRID2IP | 4.84E-01 | 1 |
| ZNF302 | 3.87E-01 | 1 | OR1C1 | 4.84E-01 | 1 |
| NBN | 3.87E-01 | 1 | HOXB7 | 4.85E-01 | 1 |
| HGS | 3.87E-01 | 1 | PRSS16 | 4.85E-01 | 1 |
| CKMT1A | 3.87E-01 | 1 | KIRREL3 | 4.85E-01 | 1 |
| CKAP2L | 3.87E-01 | 1 | CCT6A | 4.85E-01 | 1 |
| SSTR5 | 3.87E-01 | 1 | TFAP2A | 4.85E-01 | 1 |
| COG5 | 3.87E-01 | 1 | KBTBD6 | 4.85E-01 | 1 |
| PAK6 | 3.87E-01 | 1 | GRAMD3 | 4.85E-01 | 1 |
| ATF7IP2 | 3.88E-01 | 1 | SORCS3 | 4.85E-01 | 1 |
| KNDC1 | 3.88E-01 | 1 | FOXO4 | 4.85E-01 | 1 |
| CNST | 3.88E-01 | 1 | TM9SF2 | 4.85E-01 | 1 |
| SMAD7 | 3.88E-01 | 1 | PHF6 | 4.85E-01 | 1 |
| GREB1 | 3.88E-01 | 1 | 9-Sep | 4.86E-01 | 1 |
| EPHB4 | 3.88E-01 | 1 | SPAG9 | 4.86E-01 | 1 |
| TMEM82 | 3.88E-01 | 1 | B3GALNT1 | 4.86E-01 | 1 |
| LRFN3 | 3.88E-01 | 1 | TSPYL6 | 4.86E-01 | 1 |
| EEF2 | 3.89E-01 | 1 | CUL4A | 4.86E-01 | 1 |
| FKBP15 | 3.89E-01 | 1 | DDI1 | 4.86E-01 | 1 |
| ANKRA2 | 3.89E-01 | 1 | DDX43 | 4.86E-01 | 1 |
| MATN2 | 3.89E-01 | 1 | SPINK5 | 4.86E-01 | 1 |
| CANX | 3.89E-01 | 1 | UTP15 | 4.87E-01 | 1 |
| TACO1 | 3.89E-01 | 1 | APPBP2 | 4.87E-01 | 1 |
| SUCLG1 | 3.89E-01 | 1 | FAM129C | 4.87E-01 | 1 |
| CYP2A7 | 3.89E-01 | 1 | KCNAB1 | 4.87E-01 | 1 |
| KLF16 | 3.89E-01 | 1 | DHX16 | 4.87E-01 | 1 |
| GLYR1 | 3.89E-01 | 1 | GTPBP4 | 4.88E-01 | 1 |

| RAET1G | 3.89E-01 | 1 | UAP1 | 4.88E-01 | 1 |
| --- | --- | --- | --- | --- | --- |
| PRKAR2B | 3.89E-01 | 1 | KCNG1 | 4.88E-01 | 1 |
| ZMYM5 | 3.89E-01 | 1 | PPP1R12C | 4.88E-01 | 1 |
| SSH2 | 3.89E-01 | 1 | ITGB5 | 4.88E-01 | 1 |
| CEACAM | 3.90E-01 | 1 | HAP1 | 4.88E-01 | 1 |
| RGL2 | 3.90E-01 | 1 | FMNL2 | 4.88E-01 | 1 |
| MLX | 3.90E-01 | 1 | GPR153 | 4.88E-01 | 1 |
| RFX7 | 3.90E-01 | 1 | STK35 | 4.89E-01 | 1 |
| PHF21B | 3.90E-01 | 1 | MSH5 | 4.89E-01 | 1 |
| TIGIT | 3.90E-01 | 1 | ZNF586 | 4.89E-01 | 1 |
| GDF15 | 3.90E-01 | 1 | MALT1 | 4.89E-01 | 1 |
| FOXL2 | 3.90E-01 | 1 | LAMP3 | 4.89E-01 | 1 |
| MTMR7 | 3.90E-01 | 1 | MAGEB4 | 4.89E-01 | 1 |
| ZNF366 | 3.90E-01 | 1 | FAM83C | 4.89E-01 | 1 |
| INSL6 | 3.90E-01 | 1 | HTATSF1 | 4.89E-01 | 1 |
| MRAP2 | 3.90E-01 | 1 | NBR1 | 4.89E-01 | 1 |
| ZNF414 | 3.90E-01 | 1 | FCGR1A | 4.89E-01 | 1 |
| SLC16A12 | 3.90E-01 | 1 | LARP4 | 4.90E-01 | 1 |
| UPK1B | 3.91E-01 | 1 | FAM155B | 4.90E-01 | 1 |
| SIPA1L2 | 3.91E-01 | 1 | GRIP2 | 4.90E-01 | 1 |
| ATOH1 | 3.91E-01 | 1 | LONRF3 | 4.90E-01 | 1 |
| MS4A5 | 3.91E-01 | 1 | TRPA1 | 4.90E-01 | 1 |
| UPP1 | 3.91E-01 | 1 | LAMA1 | 4.90E-01 | 1 |
| RPE65 | 3.91E-01 | 1 | PSG11 | 4.90E-01 | 1 |
| RGS6 | 3.91E-01 | 1 | ZDHHC4 | 4.90E-01 | 1 |
| CD40 | 3.91E-01 | 1 | TMEM19 | 4.90E-01 | 1 |
| CLTB | 3.91E-01 | 1 | SLC9A1 | 4.90E-01 | 1 |
| AKR1B10 | 3.91E-01 | 1 | ABCA3 | 4.91E-01 | 1 |
| LYPD5 | 3.91E-01 | 1 | ZNF559 | 4.91E-01 | 1 |
| KRTAP1-3 | 3.91E-01 | 1 | VWA1 | 4.91E-01 | 1 |
| NOX4 | 3.92E-01 | 1 | AP3B1 | 4.91E-01 | 1 |
| TXNDC15 | 3.92E-01 | 1 | TRMT2A | 4.91E-01 | 1 |
| DHX15 | 3.92E-01 | 1 | OTUD5 | 4.91E-01 | 1 |
| HOXB8 | 3.92E-01 | 1 | RASA3 | 4.91E-01 | 1 |
| ZCCHC12 | 3.92E-01 | 1 | SLC12A1 | 4.91E-01 | 1 |
| NEIL2 | 3.92E-01 | 1 | ELAC2 | 4.91E-01 | 1 |
| FAM71E2 | 3.92E-01 | 1 | CD177 | 4.91E-01 | 1 |
| ZNF594 | 3.93E-01 | 1 | CMPK2 | 4.91E-01 | 1 |
| ATRN | 3.93E-01 | 1 | CPSF3 | 4.91E-01 | 1 |
| ERLIN1 | 3.93E-01 | 1 | CCDC33 | 4.91E-01 | 1 |
| STRA8 | 3.93E-01 | 1 | HNRNPF | 4.92E-01 | 1 |
| CT47B1 | 3.93E-01 | 1 | KRT33A | 4.92E-01 | 1 |
| ZNF774 | 3.93E-01 | 1 | THOC5 | 4.92E-01 | 1 |
| VPRBP | 3.93E-01 | 1 | ALDH1B1 | 4.92E-01 | 1 |
| TAAR2 | 3.93E-01 | 1 | TET2 | 4.92E-01 | 1 |
| E2F4 | 3.93E-01 | 1 | BRAP | 4.92E-01 | 1 |
| INHA | 3.93E-01 | 1 | FOXD3 | 4.92E-01 | 1 |
| IFNA17 | 3.93E-01 | 1 | MUTYH | 4.92E-01 | 1 |
| CFH | 3.93E-01 | 1 | ARHGEF37 | 4.92E-01 | 1 |
| CHPF2 | 3.93E-01 | 1 | NOTCH3 | 4.93E-01 | 1 |
| GET4 | 3.93E-01 | 1 | GRK6 | 4.93E-01 | 1 |
| RARRES1 | 3.93E-01 | 1 | TMPRSS11 | 4.93E-01 | 1 |
| HMX2 | 3.94E-01 | 1 | KHSRP | 4.93E-01 | 1 |

| LMX1B | 3.94E-01 | 1 | DLD | 4.93E-01 | 1 |
| --- | --- | --- | --- | --- | --- |
| EFHB | 3.94E-01 | 1 | LAD1 | 4.93E-01 | 1 |
| INPP5E | 3.94E-01 | 1 | OSBP | 4.93E-01 | 1 |
| SNX21 | 3.94E-01 | 1 | SLC6A3 | 4.93E-01 | 1 |
| GGTLC1 | 3.94E-01 | 1 | COG3 | 4.94E-01 | 1 |
| SESTD1 | 3.94E-01 | 1 | ALDH1A2 | 4.94E-01 | 1 |
| GALK1 | 3.94E-01 | 1 | WHSC1 | 4.94E-01 | 1 |
| C9orf171 | 3.94E-01 | 1 | GRHL1 | 4.94E-01 | 1 |
| BTN2A2 | 3.95E-01 | 1 | ZSCAN23 | 4.94E-01 | 1 |
| GDF11 | 3.95E-01 | 1 | TNS3 | 4.94E-01 | 1 |
| MTX3 | 3.95E-01 | 1 | CNN3 | 4.94E-01 | 1 |
| ODF3 | 3.95E-01 | 1 | STEAP4 | 4.94E-01 | 1 |
| USP19 | 3.95E-01 | 1 | CHD6 | 4.95E-01 | 1 |
| INTS10 | 3.95E-01 | 1 | OR4A15 | 4.95E-01 | 1 |
| NPBWR1 | 3.95E-01 | 1 | TBX1 | 4.95E-01 | 1 |
| SUPV3L1 | 3.95E-01 | 1 | BIRC6 | 4.95E-01 | 1 |
| SOX21 | 3.96E-01 | 1 | OR9Q1 | 4.95E-01 | 1 |
| C11orf24 | 3.96E-01 | 1 | STK32A | 4.95E-01 | 1 |
| DMP1 | 3.96E-01 | 1 | NCKAP5L | 4.95E-01 | 1 |
| STXBP5 | 3.96E-01 | 1 | TYW1 | 4.95E-01 | 1 |
| DGKG | 3.96E-01 | 1 | PDIA2 | 4.95E-01 | 1 |
| NOL4 | 3.96E-01 | 1 | CCDC141 | 4.96E-01 | 1 |
| CTNND2 | 3.96E-01 | 1 | TCP11 | 4.96E-01 | 1 |
| MYOZ2 | 3.96E-01 | 1 | FOXJ3 | 4.96E-01 | 1 |
| CYP4A22 | 3.97E-01 | 1 | ZFYVE1 | 4.96E-01 | 1 |
| FOXJ1 | 3.97E-01 | 1 | SCYL3 | 4.96E-01 | 1 |
| GRIK4 | 3.97E-01 | 1 | FKBP9 | 4.96E-01 | 1 |
| CFI | 3.97E-01 | 1 | MMRN1 | 4.96E-01 | 1 |
| CPSF2 | 3.97E-01 | 1 | CCDC110 | 4.96E-01 | 1 |
| DARS | 3.97E-01 | 1 | CHST2 | 4.96E-01 | 1 |
| CREB3L1 | 3.97E-01 | 1 | DCT | 4.96E-01 | 1 |
| GLIPR1L2 | 3.97E-01 | 1 | PDE9A | 4.96E-01 | 1 |
| ZNF174 | 3.97E-01 | 1 | PCYOX1L | 4.97E-01 | 1 |
| PYHIN1 | 3.97E-01 | 1 | RLF | 4.97E-01 | 1 |
| SESN3 | 3.97E-01 | 1 | CYFIP2 | 4.97E-01 | 1 |
| MFAP3 | 3.97E-01 | 1 | GNAL | 4.97E-01 | 1 |
| WDR87 | 3.98E-01 | 1 | GMCL1 | 4.97E-01 | 1 |
| ODAM | 3.98E-01 | 1 | DOK1 | 4.98E-01 | 1 |
| KCNN2 | 3.98E-01 | 1 | EYA1 | 4.98E-01 | 1 |
| FAM120C | 3.98E-01 | 1 | SLC10A5 | 4.98E-01 | 1 |
| RBM7 | 3.98E-01 | 1 | GAGE2E | 4.98E-01 | 1 |
| FAM122B | 3.98E-01 | 1 | ABCA7 | 4.98E-01 | 1 |
| ACAD9 | 3.98E-01 | 1 | MMP1 | 4.99E-01 | 1 |
| NELL1 | 3.98E-01 | 1 | MRPS22 | 4.99E-01 | 1 |
| OSBPL11 | 3.98E-01 | 1 | KDM6A | 5.00E-01 | 1 |
| P2RY2 | 3.98E-01 | 1 | HLF | 5.00E-01 | 1 |
| SFI1 | 3.98E-01 | 1 | USP25 | 5.00E-01 | 1 |
| ZCWPW2 | 3.98E-01 | 1 | ZNF615 | 5.00E-01 | 1 |
| CPEB4 | 3.99E-01 | 1 | LHX1 | 5.00E-01 | 1 |
| FLNB | 3.99E-01 | 1 | PDGFRA | 5.00E-01 | 1 |
| CCDC96 | 3.99E-01 | 1 | FCAR | 5.00E-01 | 1 |
| C10orf107 | 3.99E-01 | 1 | CDC45 | 5.01E-01 | 1 |
| SF3B2 | 3.99E-01 | 1 | FAM189A1 | 5.01E-01 | 1 |

| FOXS1 | 3.99E-01 | 1 | NOS1AP | 5.01E-01 | 1 |
| --- | --- | --- | --- | --- | --- |
| LRP1B | 3.99E-01 | 1 | APBA2 | 5.01E-01 | 1 |
| ELOVL2 | 3.99E-01 | 1 | RRP9 | 5.01E-01 | 1 |
| KCNH1 | 3.99E-01 | 1 | CASP4 | 5.01E-01 | 1 |
| OTUD6A | 3.99E-01 | 1 | ZNF710 | 5.01E-01 | 1 |
| AKR7A3 | 4.00E-01 | 1 | ZNF549 | 5.01E-01 | 1 |
| PRSS55 | 4.00E-01 | 1 | REG1B | 5.01E-01 | 1 |
| MTA1 | 4.00E-01 | 1 | TRPC7 | 5.02E-01 | 1 |
| POLR2B | 4.00E-01 | 1 | ATP6V1B2 | 5.02E-01 | 1 |
| MFF | 4.00E-01 | 1 | BRIX1 | 5.02E-01 | 1 |
| ABCC12 | 4.00E-01 | 1 | ARHGAP24 | 5.02E-01 | 1 |
| NAA11 | 4.00E-01 | 1 | VEZT | 5.02E-01 | 1 |
| TTC30B | 4.00E-01 | 1 | VAT1L | 5.02E-01 | 1 |
| ANAPC2 | 4.00E-01 | 1 | NHSL2 | 5.02E-01 | 1 |
| HADHA | 4.00E-01 | 1 | SERPINA6 | 5.02E-01 | 1 |
| TMEM106 | 4.00E-01 | 1 | ZNF648 | 5.02E-01 | 1 |
| TGM3 | 4.00E-01 | 1 | APBA3 | 5.03E-01 | 1 |
| IDE | 4.00E-01 | 1 | SLC5A4 | 5.03E-01 | 1 |
| LRRC37B | 4.00E-01 | 1 | OR10A2 | 5.03E-01 | 1 |
| KLHL38 | 4.01E-01 | 1 | ADCY8 | 5.03E-01 | 1 |
| C7orf49 | 4.01E-01 | 1 | ZPLD1 | 5.03E-01 | 1 |
| GRAP2 | 4.01E-01 | 1 | MTF1 | 5.03E-01 | 1 |
| RDX | 4.01E-01 | 1 | KCMF1 | 5.03E-01 | 1 |
| SATB1 | 4.01E-01 | 1 | ZNF439 | 5.03E-01 | 1 |
| PAPL | 4.01E-01 | 1 | HTR7 | 5.04E-01 | 1 |
| COMMD3 | 4.01E-01 | 1 | TMEM57 | 5.04E-01 | 1 |
| LHFPL1 | 4.01E-01 | 1 | OR52R1 | 5.04E-01 | 1 |
| TSNAX | 4.01E-01 | 1 | ZNF41 | 5.05E-01 | 1 |
| TXNL1 | 4.01E-01 | 1 | LINGO1 | 5.05E-01 | 1 |
| SRRM4 | 4.01E-01 | 1 | PPAP2C | 5.05E-01 | 1 |
| PARVB | 4.01E-01 | 1 | CHPT1 | 5.05E-01 | 1 |
| RTEL1 | 4.01E-01 | 1 | RPTOR | 5.05E-01 | 1 |
| PLEKHA3 | 4.01E-01 | 1 | NKX2-1 | 5.05E-01 | 1 |
| PLIN4 | 4.01E-01 | 1 | ZSWIM1 | 5.05E-01 | 1 |
| PRB4 | 4.01E-01 | 1 | KPNA5 | 5.05E-01 | 1 |
| OR5M1 | 4.01E-01 | 1 | C16orf89 | 5.05E-01 | 1 |
| ALOX5 | 4.01E-01 | 1 | PUM2 | 5.05E-01 | 1 |
| NEUROG2 | 4.01E-01 | 1 | MEF2A | 5.05E-01 | 1 |
| PGAP1 | 4.02E-01 | 1 | C16orf70 | 5.05E-01 | 1 |
| PSMB10 | 4.02E-01 | 1 | LIFR | 5.06E-01 | 1 |
| TEKT2 | 4.02E-01 | 1 | KCNH6 | 5.06E-01 | 1 |
| FRMD7 | 4.02E-01 | 1 | CLCNKB | 5.06E-01 | 1 |
| SLC30A5 | 4.02E-01 | 1 | CHM | 5.06E-01 | 1 |
| ARFGEF2 | 4.02E-01 | 1 | COPS3 | 5.06E-01 | 1 |
| B3GAT3 | 4.03E-01 | 1 | EVI5L | 5.06E-01 | 1 |
| DDX18 | 4.03E-01 | 1 | RBM19 | 5.06E-01 | 1 |
| C10orf54 | 4.03E-01 | 1 | CTSD | 5.07E-01 | 1 |
| COL15A1 | 4.03E-01 | 1 | RNF128 | 5.07E-01 | 1 |
| FBXL17 | 4.03E-01 | 1 | MAGI1 | 5.07E-01 | 1 |
| GPRC5A | 4.03E-01 | 1 | PCDH17 | 5.07E-01 | 1 |
| DEK | 4.03E-01 | 1 | PRSS1 | 5.07E-01 | 1 |
| SNX6 | 4.03E-01 | 1 | SKI | 5.08E-01 | 1 |
| DNAJB2 | 4.03E-01 | 1 | IRX2 | 5.08E-01 | 1 |

| PRRG3 | 4.03E-01 | 1 | VPS41 | 5.08E-01 | 1 |
| --- | --- | --- | --- | --- | --- |
| ZNF382 | 4.04E-01 | 1 | PRMT7 | 5.08E-01 | 1 |
| KIAA1279 | 4.04E-01 | 1 | MYO3A | 5.08E-01 | 1 |
| C14orf28 | 4.04E-01 | 1 | COL1A1 | 5.08E-01 | 1 |
| C9orf72 | 4.04E-01 | 1 | OR1L8 | 5.08E-01 | 1 |
| COL23A1 | 4.04E-01 | 1 | PCDHA6 | 5.08E-01 | 1 |
| TNRC6A | 4.04E-01 | 1 | TGIF2LX | 5.08E-01 | 1 |
| NPC1L1 | 4.04E-01 | 1 | WASF1 | 5.09E-01 | 1 |
| SLC15A2 | 4.04E-01 | 1 | ERBB2IP | 5.09E-01 | 1 |
| TSPAN31 | 4.04E-01 | 1 | TTC5 | 5.09E-01 | 1 |
| PRAMEF6 | 4.04E-01 | 1 | GFM1 | 5.09E-01 | 1 |
| GRIN2C | 4.05E-01 | 1 | PRPF18 | 5.09E-01 | 1 |
| ARFGAP3 | 4.05E-01 | 1 | POLRMT | 5.09E-01 | 1 |
| FOXL1 | 4.05E-01 | 1 | ZNF385D | 5.09E-01 | 1 |
| ARC | 4.05E-01 | 1 | GSTA4 | 5.09E-01 | 1 |
| HEYL | 4.05E-01 | 1 | CR2 | 5.09E-01 | 1 |
| TCEANC | 4.05E-01 | 1 | ZDHHC5 | 5.10E-01 | 1 |
| TPP2 | 4.06E-01 | 1 | SH2B2 | 5.10E-01 | 1 |
| IPO4 | 4.06E-01 | 1 | CELF4 | 5.10E-01 | 1 |
| SLC12A2 | 4.06E-01 | 1 | SSX7 | 5.10E-01 | 1 |
| PCDHGB2 | 4.06E-01 | 1 | SLC17A1 | 5.10E-01 | 1 |
| PNLDC1 | 4.06E-01 | 1 | PLXNB2 | 5.10E-01 | 1 |
| EPSTI1 | 4.06E-01 | 1 | BTAF1 | 5.10E-01 | 1 |
| PSG3 | 4.06E-01 | 1 | WDR62 | 5.10E-01 | 1 |
| FUT7 | 4.06E-01 | 1 | RAVER1 | 5.11E-01 | 1 |
| UTP15 | 4.06E-01 | 1 | NR0B1 | 5.11E-01 | 1 |
| OR51E1 | 4.06E-01 | 1 | BARHL2 | 5.11E-01 | 1 |
| CCBL2 | 4.06E-01 | 1 | MFSD9 | 5.11E-01 | 1 |
| KRTAP24 | 4.06E-01 | 1 | SNRPB2 | 5.11E-01 | 1 |
| ZNF519 | 4.06E-01 | 1 | LRSAM1 | 5.11E-01 | 1 |
| NEDD1 | 4.06E-01 | 1 | CRHR2 | 5.11E-01 | 1 |
| MORC2 | 4.06E-01 | 1 | SENP6 | 5.11E-01 | 1 |
| MLC1 | 4.06E-01 | 1 | CDH20 | 5.11E-01 | 1 |
| RPL28 | 4.07E-01 | 1 | AGAP3 | 5.11E-01 | 1 |
| ZNF215 | 4.07E-01 | 1 | TEKT3 | 5.11E-01 | 1 |
| PRPF18 | 4.07E-01 | 1 | PLA1A | 5.11E-01 | 1 |
| UNC13D | 4.07E-01 | 1 | OR4K14 | 5.11E-01 | 1 |
| C19orf45 | 4.07E-01 | 1 | MYT1 | 5.11E-01 | 1 |
| FANK1 | 4.07E-01 | 1 | MMP20 | 5.11E-01 | 1 |
| SLC25A6 | 4.07E-01 | 1 | SMARCD1 | 5.12E-01 | 1 |
| RIMS2 | 4.07E-01 | 1 | PRDM13 | 5.12E-01 | 1 |
| YY1AP1 | 4.07E-01 | 1 | PRKCA | 5.12E-01 | 1 |
| BTBD6 | 4.08E-01 | 1 | PRKCH | 5.12E-01 | 1 |
| HSD17B8 | 4.08E-01 | 1 | FAM160A2 | 5.12E-01 | 1 |
| RPF2 | 4.08E-01 | 1 | DCAF11 | 5.12E-01 | 1 |
| STK39 | 4.08E-01 | 1 | C2CD3 | 5.12E-01 | 1 |
| PREX2 | 4.08E-01 | 1 | ZBTB17 | 5.13E-01 | 1 |
| ZNF507 | 4.08E-01 | 1 | PSMC6 | 5.13E-01 | 1 |
| MAGEA12 | 4.08E-01 | 1 | PSD | 5.13E-01 | 1 |
| PQLC3 | 4.08E-01 | 1 | SLC6A19 | 5.13E-01 | 1 |
| TEAD4 | 4.09E-01 | 1 | PRAMEF2 | 5.13E-01 | 1 |
| COL6A3 | 4.09E-01 | 1 | FMO2 | 5.13E-01 | 1 |
| KRTAP1-1 | 4.09E-01 | 1 | BRSK2 | 5.13E-01 | 1 |

| CEBPD | 4.09E-01 | 1 | DLGAP1 | 5.13E-01 | 1 |
| --- | --- | --- | --- | --- | --- |
| DNAJC13 | 4.09E-01 | 1 | DOCK2 | 5.13E-01 | 1 |
| MIPEP | 4.09E-01 | 1 | KCNA4 | 5.13E-01 | 1 |
| VTI1A | 4.09E-01 | 1 | PLXNA2 | 5.13E-01 | 1 |
| ALG5 | 4.09E-01 | 1 | ARHGEF33 | 5.13E-01 | 1 |
| ABT1 | 4.10E-01 | 1 | SNX13 | 5.14E-01 | 1 |
| SLC32A1 | 4.10E-01 | 1 | FMO1 | 5.14E-01 | 1 |
| SLC39A11 | 4.10E-01 | 1 | SHB | 5.14E-01 | 1 |
| XAB2 | 4.10E-01 | 1 | ZKSCAN3 | 5.14E-01 | 1 |
| C3orf67 | 4.10E-01 | 1 | FADD | 5.14E-01 | 1 |
| CXCL12 | 4.10E-01 | 1 | SPOCK3 | 5.14E-01 | 1 |
| PCYT1A | 4.10E-01 | 1 | CDH7 | 5.15E-01 | 1 |
| GBX1 | 4.10E-01 | 1 | SLC13A4 | 5.15E-01 | 1 |
| MTHFSD | 4.10E-01 | 1 | ATIC | 5.15E-01 | 1 |
| MTCH1 | 4.10E-01 | 1 | MAPT | 5.15E-01 | 1 |
| LRP12 | 4.10E-01 | 1 | ITFG2 | 5.15E-01 | 1 |
| PRKCSH | 4.10E-01 | 1 | GRM1 | 5.15E-01 | 1 |
| MEGF10 | 4.11E-01 | 1 | STAM | 5.15E-01 | 1 |
| SPTBN2 | 4.11E-01 | 1 | PTBP1 | 5.15E-01 | 1 |
| CBLL1 | 4.11E-01 | 1 | APLF | 5.15E-01 | 1 |
| STARD10 | 4.11E-01 | 1 | LPP | 5.15E-01 | 1 |
| SYCP3 | 4.11E-01 | 1 | RBM42 | 5.15E-01 | 1 |
| SHROOM | 4.11E-01 | 1 | XDH | 5.15E-01 | 1 |
| TJAP1 | 4.11E-01 | 1 | SCRN2 | 5.16E-01 | 1 |
| ZNF334 | 4.11E-01 | 1 | ESPNL | 5.16E-01 | 1 |
| EPHA8 | 4.11E-01 | 1 | MYLIP | 5.16E-01 | 1 |
| GJD4 | 4.12E-01 | 1 | ABCA9 | 5.16E-01 | 1 |
| TMEM67 | 4.12E-01 | 1 | PELI2 | 5.16E-01 | 1 |
| FAM98B | 4.12E-01 | 1 | KRT14 | 5.16E-01 | 1 |
| HDAC11 | 4.12E-01 | 1 | UGT1A8 | 5.16E-01 | 1 |
| TM6SF2 | 4.12E-01 | 1 | PAPOLA | 5.16E-01 | 1 |
| CAPN11 | 4.12E-01 | 1 | ITLN1 | 5.17E-01 | 1 |
| GATA6 | 4.12E-01 | 1 | PRAMEF4 | 5.17E-01 | 1 |
| SPINT2 | 4.12E-01 | 1 | CPNE6 | 5.17E-01 | 1 |
| BTN3A1 | 4.12E-01 | 1 | COL4A6 | 5.17E-01 | 1 |
| GPT | 4.12E-01 | 1 | ATP13A1 | 5.17E-01 | 1 |
| BRPF1 | 4.13E-01 | 1 | TRAK2 | 5.17E-01 | 1 |
| GINS4 | 4.13E-01 | 1 | PTGER4 | 5.17E-01 | 1 |
| TPSD1 | 4.13E-01 | 1 | WEE1 | 5.17E-01 | 1 |
| DCAF12 | 4.13E-01 | 1 | DHCR7 | 5.18E-01 | 1 |
| CHMP2B | 4.13E-01 | 1 | GRM3 | 5.18E-01 | 1 |
| ITPR1 | 4.13E-01 | 1 | ERCC4 | 5.18E-01 | 1 |
| PTGR2 | 4.13E-01 | 1 | MYBL2 | 5.18E-01 | 1 |
| EVPLL | 4.13E-01 | 1 | SLC6A7 | 5.19E-01 | 1 |
| NUDCD2 | 4.13E-01 | 1 | TFAP2C | 5.19E-01 | 1 |
| RHBDL1 | 4.13E-01 | 1 | EEA1 | 5.19E-01 | 1 |
| EFEMP2 | 4.13E-01 | 1 | HNRNPA2B | 5.19E-01 | 1 |
| ERC2 | 4.13E-01 | 1 | ECH1 | 5.19E-01 | 1 |
| GCAT | 4.13E-01 | 1 | DGKK | 5.19E-01 | 1 |
| RRM1 | 4.13E-01 | 1 | RPS6KA1 | 5.19E-01 | 1 |
| RAB40C | 4.14E-01 | 1 | SLC10A4 | 5.19E-01 | 1 |
| PASD1 | 4.14E-01 | 1 | N4BP3 | 5.19E-01 | 1 |
| RD3 | 4.14E-01 | 1 | RNF44 | 5.20E-01 | 1 |

| RHOJ | 4.14E-01 | 1 | ZNF277 | 5.20E-01 | 1 |
| --- | --- | --- | --- | --- | --- |
| MANEA | 4.14E-01 | 1 | PCDH11Y | 5.20E-01 | 1 |
| ZNF331 | 4.14E-01 | 1 | KCNH5 | 5.20E-01 | 1 |
| ZFP1 | 4.14E-01 | 1 | TRIM46 | 5.20E-01 | 1 |
| VAX1 | 4.15E-01 | 1 | KIF1B | 5.21E-01 | 1 |
| SIRT6 | 4.15E-01 | 1 | FGR | 5.21E-01 | 1 |
| XKR7 | 4.15E-01 | 1 | DSTYK | 5.21E-01 | 1 |
| SHPK | 4.15E-01 | 1 | UBA6 | 5.21E-01 | 1 |
| PNMT | 4.15E-01 | 1 | DNAH3 | 5.21E-01 | 1 |
| PDGFD | 4.15E-01 | 1 | CPE | 5.21E-01 | 1 |
| INSRR | 4.15E-01 | 1 | UGP2 | 5.21E-01 | 1 |
| NADSYN1 | 4.15E-01 | 1 | ZNF735 | 5.21E-01 | 1 |
| DDX50 | 4.15E-01 | 1 | TGS1 | 5.21E-01 | 1 |
| ABCC3 | 4.15E-01 | 1 | ZNF679 | 5.21E-01 | 1 |
| EDARAD | 4.15E-01 | 1 | NISCH | 5.21E-01 | 1 |
| SOCS5 | 4.15E-01 | 1 | C3orf58 | 5.21E-01 | 1 |
| CYP27A1 | 4.16E-01 | 1 | SLC4A2 | 5.21E-01 | 1 |
| HLA-E | 4.16E-01 | 1 | LPPR5 | 5.21E-01 | 1 |
| METTL5 | 4.16E-01 | 1 | GTF2F1 | 5.21E-01 | 1 |
| MLF1 | 4.16E-01 | 1 | PRDM15 | 5.22E-01 | 1 |
| SERPINB1 | 4.16E-01 | 1 | SUSD4 | 5.22E-01 | 1 |
| RGSL1 | 4.16E-01 | 1 | MAP2K6 | 5.22E-01 | 1 |
| MCC | 4.16E-01 | 1 | TLE2 | 5.22E-01 | 1 |
| TRAM2 | 4.16E-01 | 1 | OR1L4 | 5.22E-01 | 1 |
| SLC25A32 | 4.16E-01 | 1 | CSDE1 | 5.22E-01 | 1 |
| PCDHGA9 | 4.17E-01 | 1 | SEZ6L | 5.23E-01 | 1 |
| ACY3 | 4.17E-01 | 1 | MBOAT4 | 5.23E-01 | 1 |
| EGR4 | 4.17E-01 | 1 | NEUROD6 | 5.23E-01 | 1 |
| PDYN | 4.17E-01 | 1 | NPLOC4 | 5.23E-01 | 1 |
| TRPC3 | 4.17E-01 | 1 | SLC30A10 | 5.23E-01 | 1 |
| ACRC | 4.17E-01 | 1 | LPO | 5.23E-01 | 1 |
| PML | 4.17E-01 | 1 | NASP | 5.23E-01 | 1 |
| PKP4 | 4.17E-01 | 1 | ZFR | 5.23E-01 | 1 |
| NDST1 | 4.17E-01 | 1 | CLTCL1 | 5.23E-01 | 1 |
| PIP5K1B | 4.17E-01 | 1 | CDR2L | 5.24E-01 | 1 |
| WFDC1 | 4.17E-01 | 1 | AFAP1 | 5.24E-01 | 1 |
| SLC22A5 | 4.17E-01 | 1 | PCDHGA10 | 5.24E-01 | 1 |
| AWAT2 | 4.17E-01 | 1 | GDAP2 | 5.24E-01 | 1 |
| TMC6 | 4.17E-01 | 1 | ZNF485 | 5.24E-01 | 1 |
| OR1G1 | 4.17E-01 | 1 | SLC5A5 | 5.24E-01 | 1 |
| ABCA3 | 4.17E-01 | 1 | INPP5E | 5.24E-01 | 1 |
| CD300LG | 4.18E-01 | 1 | ADAMTS7 | 5.25E-01 | 1 |
| E4F1 | 4.18E-01 | 1 | CAP2 | 5.25E-01 | 1 |
| VPS41 | 4.18E-01 | 1 | PCDHGA12 | 5.25E-01 | 1 |
| FXR2 | 4.18E-01 | 1 | FAM20A | 5.25E-01 | 1 |
| SUPT5H | 4.18E-01 | 1 | LGI2 | 5.25E-01 | 1 |
| SGPP1 | 4.18E-01 | 1 | RYK | 5.25E-01 | 1 |
| DNASE1L | 4.18E-01 | 1 | NLRP13 | 5.25E-01 | 1 |
| CEP164 | 4.18E-01 | 1 | ATL2 | 5.26E-01 | 1 |
| SDF4 | 4.18E-01 | 1 | ADORA3 | 5.26E-01 | 1 |
| GTF3C4 | 4.19E-01 | 1 | SLC29A2 | 5.26E-01 | 1 |
| CDC37L1 | 4.19E-01 | 1 | ZNF665 | 5.26E-01 | 1 |
| MYO9B | 4.19E-01 | 1 | MYRIP | 5.26E-01 | 1 |

| POC1A | 4.19E-01 | 1 | MYOC | 5.26E-01 | 1 |
| --- | --- | --- | --- | --- | --- |
| AARS2 | 4.19E-01 | 1 | ING1 | 5.26E-01 | 1 |
| PLEKHA8 | 4.19E-01 | 1 | SLC5A7 | 5.26E-01 | 1 |
| NUS1 | 4.19E-01 | 1 | VIPR2 | 5.26E-01 | 1 |
| VWF | 4.19E-01 | 1 | FZD5 | 5.27E-01 | 1 |
| KCNMB4 | 4.19E-01 | 1 | GABBR1 | 5.27E-01 | 1 |
| RWDD3 | 4.19E-01 | 1 | IL12RB2 | 5.27E-01 | 1 |
| EXT2 | 4.19E-01 | 1 | RIMS1 | 5.27E-01 | 1 |
| SOX12 | 4.19E-01 | 1 | CIC | 5.28E-01 | 1 |
| KCNQ3 | 4.19E-01 | 1 | OR1L1 | 5.28E-01 | 1 |
| FGF23 | 4.19E-01 | 1 | KRT78 | 5.28E-01 | 1 |
| OSTM1 | 4.20E-01 | 1 | PALMD | 5.28E-01 | 1 |
| QTRT1 | 4.20E-01 | 1 | SLC7A3 | 5.28E-01 | 1 |
| PCDHGC3 | 4.20E-01 | 1 | CACNA1G | 5.28E-01 | 1 |
| SIGLEC8 | 4.20E-01 | 1 | SHOX2 | 5.28E-01 | 1 |
| TCF7L2 | 4.20E-01 | 1 | LCK | 5.28E-01 | 1 |
| ITGA7 | 4.20E-01 | 1 | PHACTR1 | 5.28E-01 | 1 |
| RSPH4A | 4.20E-01 | 1 | TLR4 | 5.29E-01 | 1 |
| PRR19 | 4.20E-01 | 1 | ACTL6A | 5.29E-01 | 1 |
| DCAF7 | 4.20E-01 | 1 | TACR1 | 5.29E-01 | 1 |
| OR4A15 | 4.20E-01 | 1 | SMAP2 | 5.29E-01 | 1 |
| LIPG | 4.20E-01 | 1 | ADAM11 | 5.29E-01 | 1 |
| SMG5 | 4.20E-01 | 1 | NTM | 5.29E-01 | 1 |
| AP3M1 | 4.20E-01 | 1 | TCTE1 | 5.29E-01 | 1 |
| CHD7 | 4.21E-01 | 1 | ILDR1 | 5.29E-01 | 1 |
| LRRC42 | 4.21E-01 | 1 | ILDR2 | 5.29E-01 | 1 |
| OR51G2 | 4.21E-01 | 1 | ATG16L2 | 5.29E-01 | 1 |
| KCNH4 | 4.21E-01 | 1 | IL5RA | 5.30E-01 | 1 |
| TNFRSF1 | 4.21E-01 | 1 | CEP170 | 5.30E-01 | 1 |
| ATP5C1 | 4.21E-01 | 1 | PKD1L3 | 5.30E-01 | 1 |
| PARP16 | 4.21E-01 | 1 | ZYX | 5.30E-01 | 1 |
| H2AFY2 | 4.21E-01 | 1 | CALCR | 5.30E-01 | 1 |
| HYOU1 | 4.21E-01 | 1 | FNDC7 | 5.30E-01 | 1 |
| OR2S2 | 4.21E-01 | 1 | TRIM77 | 5.30E-01 | 1 |
| BAHD1 | 4.21E-01 | 1 | GUCY1A3 | 5.30E-01 | 1 |
| PPP2R1A | 4.22E-01 | 1 | ZNF441 | 5.30E-01 | 1 |
| TMEM106 | 4.22E-01 | 1 | CHST10 | 5.31E-01 | 1 |
| HEMK1 | 4.22E-01 | 1 | SCN11A | 5.31E-01 | 1 |
| SLC22A15 | 4.23E-01 | 1 | PSMC4 | 5.31E-01 | 1 |
| LMTK2 | 4.23E-01 | 1 | RFTN1 | 5.31E-01 | 1 |
| SLC35B1 | 4.23E-01 | 1 | HMGCLL1 | 5.32E-01 | 1 |
| KIAA1586 | 4.23E-01 | 1 | CCDC144A | 5.32E-01 | 1 |
| PGA5 | 4.23E-01 | 1 | PLTP | 5.32E-01 | 1 |
| TFAP2D | 4.23E-01 | 1 | GK2 | 5.32E-01 | 1 |
| KCTD13 | 4.23E-01 | 1 | ZDHHC13 | 5.32E-01 | 1 |
| GTPBP1 | 4.23E-01 | 1 | PRMT6 | 5.32E-01 | 1 |
| IREB2 | 4.23E-01 | 1 | STXBP5L | 5.33E-01 | 1 |
| CDH13 | 4.24E-01 | 1 | PRAMEF19 | 5.33E-01 | 1 |
| AACS | 4.24E-01 | 1 | ZAP70 | 5.33E-01 | 1 |
| DRD1 | 4.24E-01 | 1 | PAQR9 | 5.33E-01 | 1 |
| SLC36A2 | 4.24E-01 | 1 | GALNT3 | 5.34E-01 | 1 |
| XPO1 | 4.24E-01 | 1 | SLC39A6 | 5.34E-01 | 1 |
| SEC13 | 4.24E-01 | 1 | ARHGAP23 | 5.34E-01 | 1 |

| TNN | 4.25E-01 | 1 | WDR91 | 5.34E-01 | 1 |
| --- | --- | --- | --- | --- | --- |
| OR51B6 | 4.25E-01 | 1 | ITGA2B | 5.34E-01 | 1 |
| EIF3B | 4.25E-01 | 1 | WWC1 | 5.34E-01 | 1 |
| SIK2 | 4.25E-01 | 1 | BMX | 5.34E-01 | 1 |
| PDXP | 4.25E-01 | 1 | EGFL6 | 5.34E-01 | 1 |
| TCF7 | 4.25E-01 | 1 | CHRNA4 | 5.35E-01 | 1 |
| ZNF764 | 4.25E-01 | 1 | KBTBD8 | 5.35E-01 | 1 |
| MEX3B | 4.25E-01 | 1 | MMP3 | 5.35E-01 | 1 |
| SLA | 4.25E-01 | 1 | RASGRF2 | 5.35E-01 | 1 |
| PIK3CA | 4.25E-01 | 1 | TOR1AIP2 | 5.35E-01 | 1 |
| DNAJC11 | 4.26E-01 | 1 | FYN | 5.36E-01 | 1 |
| ZC4H2 | 4.26E-01 | 1 | TEX15 | 5.36E-01 | 1 |
| APOBEC3 | 4.26E-01 | 1 | TTC31 | 5.36E-01 | 1 |
| ABHD12 | 4.26E-01 | 1 | AMY2A | 5.36E-01 | 1 |
| EDNRA | 4.26E-01 | 1 | SLC4A3 | 5.36E-01 | 1 |
| FBN2 | 4.26E-01 | 1 | SPTY2D1 | 5.36E-01 | 1 |
| IFNA5 | 4.26E-01 | 1 | XRCC5 | 5.36E-01 | 1 |
| GFAP | 4.26E-01 | 1 | ABCC10 | 5.36E-01 | 1 |
| BCAP31 | 4.26E-01 | 1 | OR6A2 | 5.36E-01 | 1 |
| TMBIM4 | 4.26E-01 | 1 | GTF2IRD2 | 5.36E-01 | 1 |
| ABCF3 | 4.26E-01 | 1 | ZNF256 | 5.36E-01 | 1 |
| SDK1 | 4.26E-01 | 1 | RXRG | 5.36E-01 | 1 |
| ZW10 | 4.26E-01 | 1 | POTEC | 5.36E-01 | 1 |
| ISLR | 4.26E-01 | 1 | ANGEL2 | 5.37E-01 | 1 |
| MYEF2 | 4.26E-01 | 1 | KIAA1586 | 5.37E-01 | 1 |
| OR51D1 | 4.27E-01 | 1 | CHPF2 | 5.37E-01 | 1 |
| OMA1 | 4.27E-01 | 1 | KRT6C | 5.37E-01 | 1 |
| MLLT3 | 4.27E-01 | 1 | INTS9 | 5.37E-01 | 1 |
| CCDC105 | 4.27E-01 | 1 | PCDHGA5 | 5.37E-01 | 1 |
| WDR49 | 4.27E-01 | 1 | HK2 | 5.37E-01 | 1 |
| LRBA | 4.27E-01 | 1 | KLF12 | 5.37E-01 | 1 |
| ZNF843 | 4.27E-01 | 1 | EDEM1 | 5.37E-01 | 1 |
| ERAP1 | 4.27E-01 | 1 | HEXA | 5.37E-01 | 1 |
| ATXN7L3 | 4.27E-01 | 1 | FBXO30 | 5.37E-01 | 1 |
| SCUBE3 | 4.27E-01 | 1 | DTX1 | 5.37E-01 | 1 |
| VPS53 | 4.27E-01 | 1 | DSEL | 5.37E-01 | 1 |
| ART4 | 4.27E-01 | 1 | C7orf72 | 5.38E-01 | 1 |
| STAP1 | 4.27E-01 | 1 | CEP70 | 5.38E-01 | 1 |
| SAMD3 | 4.28E-01 | 1 | TAF4B | 5.38E-01 | 1 |
| LPCAT4 | 4.28E-01 | 1 | CCDC14 | 5.38E-01 | 1 |
| KLHDC9 | 4.28E-01 | 1 | CYP7A1 | 5.39E-01 | 1 |
| C1orf127 | 4.28E-01 | 1 | PDLIM7 | 5.39E-01 | 1 |
| MTSS1L | 4.28E-01 | 1 | FBF1 | 5.39E-01 | 1 |
| MMD2 | 4.28E-01 | 1 | CRYZL1 | 5.39E-01 | 1 |
| VRK2 | 4.29E-01 | 1 | USH1G | 5.39E-01 | 1 |
| P2RY14 | 4.29E-01 | 1 | UMODL1 | 5.39E-01 | 1 |
| ANO4 | 4.29E-01 | 1 | LSR | 5.39E-01 | 1 |
| MEGF9 | 4.29E-01 | 1 | ADCY4 | 5.39E-01 | 1 |
| FUCA2 | 4.29E-01 | 1 | TTC39C | 5.39E-01 | 1 |
| PIK3CG | 4.29E-01 | 1 | HTR1E | 5.40E-01 | 1 |
| PAX7 | 4.29E-01 | 1 | ERP44 | 5.40E-01 | 1 |
| NFRKB | 4.30E-01 | 1 | ZIK1 | 5.40E-01 | 1 |
| PDK2 | 4.30E-01 | 1 | SLC13A2 | 5.40E-01 | 1 |

| DALRD3 | 4.30E-01 | 1 | IL12RB1 | 5.40E-01 | 1 |
| --- | --- | --- | --- | --- | --- |
| EHF | 4.30E-01 | 1 | DYNC1LI2 | 5.41E-01 | 1 |
| GNB2 | 4.30E-01 | 1 | GPC2 | 5.41E-01 | 1 |
| B3GNT6 | 4.30E-01 | 1 | PABPC1L | 5.41E-01 | 1 |
| SUPT16H | 4.30E-01 | 1 | SYNRG | 5.41E-01 | 1 |
| PTCHD2 | 4.30E-01 | 1 | SSH3 | 5.41E-01 | 1 |
| CNGB1 | 4.30E-01 | 1 | CDCP1 | 5.41E-01 | 1 |
| DHRS1 | 4.30E-01 | 1 | SIGLEC6 | 5.41E-01 | 1 |
| SIPA1 | 4.30E-01 | 1 | ARHGAP31 | 5.41E-01 | 1 |
| SRR | 4.30E-01 | 1 | DAGLA | 5.41E-01 | 1 |
| C17orf75 | 4.30E-01 | 1 | AMIGO2 | 5.41E-01 | 1 |
| DCUN1D5 | 4.30E-01 | 1 | USP19 | 5.41E-01 | 1 |
| DOPEY2 | 4.30E-01 | 1 | POGK | 5.41E-01 | 1 |
| FRG2B | 4.30E-01 | 1 | PVR | 5.42E-01 | 1 |
| NDUFS1 | 4.31E-01 | 1 | TBC1D22A | 5.42E-01 | 1 |
| MGAT5B | 4.31E-01 | 1 | ANKDD1A | 5.42E-01 | 1 |
| CACNB1 | 4.31E-01 | 1 | SPHK2 | 5.42E-01 | 1 |
| GNAI2 | 4.31E-01 | 1 | IQCE | 5.42E-01 | 1 |
| ME2 | 4.31E-01 | 1 | CARD6 | 5.42E-01 | 1 |
| SF3A1 | 4.31E-01 | 1 | NOX5 | 5.42E-01 | 1 |
| SLC47A1 | 4.31E-01 | 1 | CDC6 | 5.42E-01 | 1 |
| DLK1 | 4.31E-01 | 1 | ADCK4 | 5.43E-01 | 1 |
| VPS8 | 4.31E-01 | 1 | EPB41 | 5.43E-01 | 1 |
| SLC12A1 | 4.31E-01 | 1 | MBD4 | 5.43E-01 | 1 |
| ZNF454 | 4.31E-01 | 1 | RBMS3 | 5.43E-01 | 1 |
| ECEL1 | 4.31E-01 | 1 | VAC14 | 5.43E-01 | 1 |
| ZNF510 | 4.31E-01 | 1 | TLR2 | 5.43E-01 | 1 |
| KRT3 | 4.32E-01 | 1 | TXK | 5.43E-01 | 1 |
| PTGIS | 4.32E-01 | 1 | WFIKKN2 | 5.43E-01 | 1 |
| MAPT | 4.32E-01 | 1 | MPO | 5.43E-01 | 1 |
| ZFP2 | 4.32E-01 | 1 | CPXM2 | 5.43E-01 | 1 |
| GPRIN3 | 4.32E-01 | 1 | SHISA9 | 5.43E-01 | 1 |
| SEC31B | 4.32E-01 | 1 | CNTNAP2 | 5.44E-01 | 1 |
| CCND2 | 4.32E-01 | 1 | GIN1 | 5.44E-01 | 1 |
| FXR1 | 4.32E-01 | 1 | ZNF493 | 5.44E-01 | 1 |
| DYRK1B | 4.32E-01 | 1 | WEE2 | 5.44E-01 | 1 |
| ZNF595 | 4.32E-01 | 1 | ZNF202 | 5.44E-01 | 1 |
| NLRP9 | 4.32E-01 | 1 | CYP11B2 | 5.44E-01 | 1 |
| LRWD1 | 4.33E-01 | 1 | SARM1 | 5.44E-01 | 1 |
| TLX2 | 4.33E-01 | 1 | RAB3IP | 5.44E-01 | 1 |
| GPR52 | 4.33E-01 | 1 | FAM9A | 5.45E-01 | 1 |
| JUNB | 4.33E-01 | 1 | CCDC39 | 5.45E-01 | 1 |
| SRRM1 | 4.33E-01 | 1 | DCDC2 | 5.45E-01 | 1 |
| GPR84 | 4.33E-01 | 1 | ZNF419 | 5.45E-01 | 1 |
| MRPS31 | 4.33E-01 | 1 | SLC17A9 | 5.45E-01 | 1 |
| OR5AN1 | 4.33E-01 | 1 | EFR3A | 5.45E-01 | 1 |
| ADAMTS | 4.33E-01 | 1 | CELSR2 | 5.45E-01 | 1 |
| IL17RD | 4.33E-01 | 1 | LSS | 5.45E-01 | 1 |
| CATSPER | 4.33E-01 | 1 | ASPH | 5.45E-01 | 1 |
| SLBP | 4.34E-01 | 1 | ELL | 5.46E-01 | 1 |
| ZNF660 | 4.34E-01 | 1 | FGFR1 | 5.46E-01 | 1 |
| IRF3 | 4.34E-01 | 1 | PTCHD3 | 5.46E-01 | 1 |
| ENOSF1 | 4.34E-01 | 1 | CLSTN3 | 5.46E-01 | 1 |

| CDRT15 | 4.34E-01 | 1 | CDON | 5.46E-01 | 1 |
| --- | --- | --- | --- | --- | --- |
| CCDC138 | 4.34E-01 | 1 | IGSF9 | 5.46E-01 | 1 |
| HPN | 4.34E-01 | 1 | KIAA0355 | 5.46E-01 | 1 |
| CALHM2 | 4.35E-01 | 1 | PRPF31 | 5.47E-01 | 1 |
| ACOT12 | 4.35E-01 | 1 | OR2T11 | 5.47E-01 | 1 |
| RTP4 | 4.35E-01 | 1 | FAM71A | 5.47E-01 | 1 |
| AHDC1 | 4.35E-01 | 1 | C1orf127 | 5.48E-01 | 1 |
| REM2 | 4.35E-01 | 1 | SMURF2 | 5.48E-01 | 1 |
| LRRC49 | 4.35E-01 | 1 | HCRTR2 | 5.48E-01 | 1 |
| ALPK1 | 4.35E-01 | 1 | AOX1 | 5.48E-01 | 1 |
| ST6GALN | 4.35E-01 | 1 | SBSN | 5.48E-01 | 1 |
| TPRX1 | 4.35E-01 | 1 | PTX4 | 5.48E-01 | 1 |
| HLA-DQA | 4.35E-01 | 1 | PHLDB1 | 5.48E-01 | 1 |
| HOXA7 | 4.35E-01 | 1 | C14orf39 | 5.48E-01 | 1 |
| NYNRIN | 4.35E-01 | 1 | EPHB2 | 5.49E-01 | 1 |
| TUBD1 | 4.36E-01 | 1 | FNTA | 5.49E-01 | 1 |
| ELP3 | 4.36E-01 | 1 | GRIK3 | 5.49E-01 | 1 |
| 7-Sep | 4.36E-01 | 1 | TCFL5 | 5.49E-01 | 1 |
| DNAH11 | 4.36E-01 | 1 | ZNF263 | 5.49E-01 | 1 |
| CLEC7A | 4.36E-01 | 1 | EHHADH | 5.49E-01 | 1 |
| STYXL1 | 4.36E-01 | 1 | FAM117B | 5.50E-01 | 1 |
| TBL2 | 4.36E-01 | 1 | MORC1 | 5.50E-01 | 1 |
| SNX2 | 4.36E-01 | 1 | DNAH10 | 5.50E-01 | 1 |
| C1orf177 | 4.36E-01 | 1 | AGRN | 5.50E-01 | 1 |
| CALB1 | 4.36E-01 | 1 | RNF17 | 5.51E-01 | 1 |
| FAM69B | 4.37E-01 | 1 | VPS52 | 5.51E-01 | 1 |
| RGL1 | 4.37E-01 | 1 | SLITRK4 | 5.51E-01 | 1 |
| LRRC61 | 4.37E-01 | 1 | STMN4 | 5.51E-01 | 1 |
| SGCD | 4.37E-01 | 1 | TRPM2 | 5.51E-01 | 1 |
| AJAP1 | 4.37E-01 | 1 | SV2A | 5.51E-01 | 1 |
| YY2 | 4.37E-01 | 1 | TRPM8 | 5.51E-01 | 1 |
| CBLB | 4.37E-01 | 1 | ZDBF2 | 5.52E-01 | 1 |
| CACNG6 | 4.37E-01 | 1 | ANKK1 | 5.52E-01 | 1 |
| SLC39A12 | 4.37E-01 | 1 | URGCP | 5.52E-01 | 1 |
| FOXH1 | 4.37E-01 | 1 | PCDHA3 | 5.52E-01 | 1 |
| HEXIM1 | 4.38E-01 | 1 | TCEB3B | 5.52E-01 | 1 |
| NFIA | 4.38E-01 | 1 | DAAM1 | 5.52E-01 | 1 |
| TSC2 | 4.38E-01 | 1 | MAGEC3 | 5.52E-01 | 1 |
| OR52R1 | 4.38E-01 | 1 | RAI14 | 5.52E-01 | 1 |
| SLC43A2 | 4.38E-01 | 1 | FRRS1 | 5.52E-01 | 1 |
| CGB7 | 4.38E-01 | 1 | NSUN6 | 5.52E-01 | 1 |
| APOBEC4 | 4.38E-01 | 1 | C3orf20 | 5.52E-01 | 1 |
| GABRP | 4.38E-01 | 1 | LRRC32 | 5.52E-01 | 1 |
| ZNF318 | 4.39E-01 | 1 | SMPD3 | 5.53E-01 | 1 |
| MYD88 | 4.39E-01 | 1 | UNC93A | 5.53E-01 | 1 |
| NOX3 | 4.39E-01 | 1 | ANKIB1 | 5.53E-01 | 1 |
| PALM | 4.39E-01 | 1 | ITGAE | 5.54E-01 | 1 |
| ZDHHC13 | 4.39E-01 | 1 | SLC13A3 | 5.54E-01 | 1 |
| FRZB | 4.39E-01 | 1 | PCDHA2 | 5.54E-01 | 1 |
| RGS3 | 4.39E-01 | 1 | VAMP7 | 5.54E-01 | 1 |
| LRIT1 | 4.39E-01 | 1 | PGM5 | 5.54E-01 | 1 |
| AATF | 4.39E-01 | 1 | COL6A6 | 5.54E-01 | 1 |
| PPHLN1 | 4.39E-01 | 1 | CUX2 | 5.54E-01 | 1 |

| CDC7 | 4.39E-01 | 1 | ZNF440 | 5.54E-01 | 1 |
| --- | --- | --- | --- | --- | --- |
| MAGEC2 | 4.39E-01 | 1 | WDR20 | 5.55E-01 | 1 |
| TEAD3 | 4.39E-01 | 1 | SLFN13 | 5.55E-01 | 1 |
| ERGIC1 | 4.39E-01 | 1 | PRICKLE3 | 5.55E-01 | 1 |
| CRY2 | 4.39E-01 | 1 | CYP39A1 | 5.55E-01 | 1 |
| PTGIR | 4.39E-01 | 1 | ANXA2 | 5.55E-01 | 1 |
| RRP8 | 4.40E-01 | 1 | KIRREL2 | 5.56E-01 | 1 |
| COLQ | 4.40E-01 | 1 | IL17RE | 5.56E-01 | 1 |
| STAC | 4.40E-01 | 1 | SHISA7 | 5.57E-01 | 1 |
| OR2AK2 | 4.40E-01 | 1 | SIX5 | 5.57E-01 | 1 |
| LTB4R | 4.40E-01 | 1 | ONECUT3 | 5.57E-01 | 1 |
| SYT16 | 4.40E-01 | 1 | KRT31 | 5.57E-01 | 1 |
| DUS1L | 4.40E-01 | 1 | DHX8 | 5.57E-01 | 1 |
| ATP2B3 | 4.40E-01 | 1 | DPCR1 | 5.57E-01 | 1 |
| SLC22A18 | 4.40E-01 | 1 | PDE3A | 5.58E-01 | 1 |
| PKN3 | 4.40E-01 | 1 | HSPA6 | 5.58E-01 | 1 |
| PHF20L1 | 4.41E-01 | 1 | ATM | 5.58E-01 | 1 |
| PPAPDC2 | 4.41E-01 | 1 | SLC44A2 | 5.58E-01 | 1 |
| STK4 | 4.41E-01 | 1 | DDX19B | 5.58E-01 | 1 |
| NDUFA9 | 4.41E-01 | 1 | HNRNPUL2 | 5.59E-01 | 1 |
| NUDT19 | 4.41E-01 | 1 | HSPA5 | 5.59E-01 | 1 |
| NAGA | 4.41E-01 | 1 | SLTM | 5.59E-01 | 1 |
| IDH3G | 4.41E-01 | 1 | TMEM117 | 5.59E-01 | 1 |
| CRELD2 | 4.41E-01 | 1 | DDX46 | 5.59E-01 | 1 |
| ADCY4 | 4.41E-01 | 1 | ASXL1 | 5.59E-01 | 1 |
| OR5H2 | 4.41E-01 | 1 | SCAI | 5.59E-01 | 1 |
| FAM83H | 4.41E-01 | 1 | KDR | 5.59E-01 | 1 |
| ZNF284 | 4.41E-01 | 1 | SLC25A13 | 5.59E-01 | 1 |
| KRT40 | 4.41E-01 | 1 | ARMC9 | 5.60E-01 | 1 |
| LRFN2 | 4.42E-01 | 1 | AQP1 | 5.60E-01 | 1 |
| RNF115 | 4.42E-01 | 1 | ADAMTSL | 5.61E-01 | 1 |
| XPNPEP1 | 4.42E-01 | 1 | OTOL1 | 5.61E-01 | 1 |
| C1orf112 | 4.42E-01 | 1 | NCAPD2 | 5.61E-01 | 1 |
| NCOR2 | 4.42E-01 | 1 | TTYH1 | 5.62E-01 | 1 |
| LZTS1 | 4.42E-01 | 1 | SLC47A1 | 5.62E-01 | 1 |
| MTF1 | 4.42E-01 | 1 | ZDHHC15 | 5.62E-01 | 1 |
| TATDN2 | 4.42E-01 | 1 | EDNRB | 5.62E-01 | 1 |
| KDM4A | 4.42E-01 | 1 | PDLIM5 | 5.62E-01 | 1 |
| SIRT2 | 4.42E-01 | 1 | HSP90B1 | 5.63E-01 | 1 |
| IDI1 | 4.42E-01 | 1 | PAK3 | 5.63E-01 | 1 |
| HAS3 | 4.42E-01 | 1 | AP2A2 | 5.63E-01 | 1 |
| TST | 4.43E-01 | 1 | OR2T1 | 5.63E-01 | 1 |
| PRG4 | 4.43E-01 | 1 | LRFN5 | 5.63E-01 | 1 |
| AKAP8 | 4.43E-01 | 1 | NOS1 | 5.63E-01 | 1 |
| GDE1 | 4.43E-01 | 1 | TPP2 | 5.63E-01 | 1 |
| RCOR1 | 4.43E-01 | 1 | ARID1B | 5.63E-01 | 1 |
| TAS2R16 | 4.43E-01 | 1 | OPRK1 | 5.64E-01 | 1 |
| YARS2 | 4.43E-01 | 1 | POM121L2 | 5.64E-01 | 1 |
| HOXC4 | 4.43E-01 | 1 | ADCY1 | 5.64E-01 | 1 |
| RPL10L | 4.43E-01 | 1 | FAM126A | 5.64E-01 | 1 |
| DCAF12L | 4.44E-01 | 1 | ZNF92 | 5.64E-01 | 1 |
| SLC9A3 | 4.44E-01 | 1 | PRCP | 5.65E-01 | 1 |
| PRKDC | 4.44E-01 | 1 | ACPP | 5.65E-01 | 1 |

| AVPR2 | 4.44E-01 | 1 | DCST1 | 5.65E-01 | 1 |
| --- | --- | --- | --- | --- | --- |
| NCK1 | 4.44E-01 | 1 | TRAF6 | 5.65E-01 | 1 |
| DCTN2 | 4.44E-01 | 1 | HDAC5 | 5.65E-01 | 1 |
| TAS2R50 | 4.44E-01 | 1 | PARP1 | 5.66E-01 | 1 |
| IL6R | 4.44E-01 | 1 | WSCD2 | 5.66E-01 | 1 |
| ZC3H12D | 4.44E-01 | 1 | SLCO2A1 | 5.66E-01 | 1 |
| C2orf80 | 4.44E-01 | 1 | SMURF1 | 5.66E-01 | 1 |
| ADAR | 4.44E-01 | 1 | MED25 | 5.66E-01 | 1 |
| SLAMF8 | 4.44E-01 | 1 | TAPT1 | 5.66E-01 | 1 |
| RNF144B | 4.45E-01 | 1 | ST6GALNA | 5.66E-01 | 1 |
| VWC2L | 4.45E-01 | 1 | EWSR1 | 5.66E-01 | 1 |
| ARMCX2 | 4.45E-01 | 1 | PHLPP2 | 5.67E-01 | 1 |
| KBTBD6 | 4.45E-01 | 1 | CDH22 | 5.67E-01 | 1 |
| MOGAT3 | 4.45E-01 | 1 | PML | 5.67E-01 | 1 |
| KRTAP10 | 4.45E-01 | 1 | GIGYF1 | 5.67E-01 | 1 |
| KIF17 | 4.45E-01 | 1 | HFM1 | 5.67E-01 | 1 |
| GCM1 | 4.45E-01 | 1 | SPTBN1 | 5.67E-01 | 1 |
| AMMECR | 4.45E-01 | 1 | ZFP90 | 5.68E-01 | 1 |
| P2RY12 | 4.45E-01 | 1 | DLG3 | 5.68E-01 | 1 |
| HSDL2 | 4.45E-01 | 1 | WNK3 | 5.68E-01 | 1 |
| SDCCAG8 | 4.45E-01 | 1 | SPAST | 5.68E-01 | 1 |
| VSIG2 | 4.46E-01 | 1 | MCTP2 | 5.68E-01 | 1 |
| PIGN | 4.46E-01 | 1 | CCDC88A | 5.68E-01 | 1 |
| CHRM4 | 4.46E-01 | 1 | AIRE | 5.68E-01 | 1 |
| CCDC64B | 4.46E-01 | 1 | C1orf27 | 5.69E-01 | 1 |
| DYNC1LI | 4.46E-01 | 1 | BTN2A1 | 5.69E-01 | 1 |
| TPM1 | 4.46E-01 | 1 | GREB1 | 5.69E-01 | 1 |
| SH3RF3 | 4.46E-01 | 1 | LTF | 5.69E-01 | 1 |
| IQGAP2 | 4.46E-01 | 1 | TMEM132E | 5.69E-01 | 1 |
| ZNF668 | 4.46E-01 | 1 | PPRC1 | 5.69E-01 | 1 |
| SRPK1 | 4.46E-01 | 1 | TCF20 | 5.69E-01 | 1 |
| PTPRB | 4.46E-01 | 1 | MMP12 | 5.69E-01 | 1 |
| ZNF20 | 4.46E-01 | 1 | MYBPC2 | 5.69E-01 | 1 |
| MCFD2 | 4.46E-01 | 1 | SPTBN4 | 5.70E-01 | 1 |
| BMX | 4.47E-01 | 1 | NFIB | 5.70E-01 | 1 |
| OR51B2 | 4.47E-01 | 1 | MIER1 | 5.71E-01 | 1 |
| AIFM1 | 4.47E-01 | 1 | CNKSR1 | 5.71E-01 | 1 |
| ZDBF2 | 4.47E-01 | 1 | FUCA2 | 5.71E-01 | 1 |
| SLC22A2 | 4.47E-01 | 1 | IL22RA1 | 5.71E-01 | 1 |
| RHBDD1 | 4.47E-01 | 1 | PCK2 | 5.71E-01 | 1 |
| KIAA1211 | 4.47E-01 | 1 | MED24 | 5.72E-01 | 1 |
| ZNF264 | 4.47E-01 | 1 | VPS45 | 5.72E-01 | 1 |
| SLC25A22 | 4.47E-01 | 1 | NKPD1 | 5.72E-01 | 1 |
| ENOPH1 | 4.47E-01 | 1 | GCC1 | 5.72E-01 | 1 |
| TMEM140 | 4.47E-01 | 1 | LIPI | 5.72E-01 | 1 |
| TRABD | 4.47E-01 | 1 | EZR | 5.72E-01 | 1 |
| SCOC | 4.48E-01 | 1 | LCORL | 5.72E-01 | 1 |
| CHRNA3 | 4.48E-01 | 1 | SRCAP | 5.72E-01 | 1 |
| MAPK14 | 4.48E-01 | 1 | TNIP1 | 5.72E-01 | 1 |
| ANKRD55 | 4.48E-01 | 1 | CDKL5 | 5.73E-01 | 1 |
| GNA15 | 4.48E-01 | 1 | INHBA | 5.73E-01 | 1 |
| OR9A4 | 4.48E-01 | 1 | DSCAM | 5.73E-01 | 1 |
| ZMAT4 | 4.48E-01 | 1 | ZNF274 | 5.73E-01 | 1 |

| NANOG | 4.48E-01 | 1 | RAD50 | 5.73E-01 | 1 |
| --- | --- | --- | --- | --- | --- |
| APOE | 4.48E-01 | 1 | MAG | 5.73E-01 | 1 |
| MUC1 | 4.48E-01 | 1 | MRPL21 | 5.74E-01 | 1 |
| NIT1 | 4.48E-01 | 1 | ANKRD6 | 5.74E-01 | 1 |
| WNT4 | 4.49E-01 | 1 | RFWD3 | 5.74E-01 | 1 |
| PTCD2 | 4.49E-01 | 1 | KRT4 | 5.74E-01 | 1 |
| MAK | 4.49E-01 | 1 | MAP3K9 | 5.74E-01 | 1 |
| OR6C3 | 4.49E-01 | 1 | ATP2B2 | 5.75E-01 | 1 |
| TTC24 | 4.49E-01 | 1 | GCLC | 5.75E-01 | 1 |
| DSE | 4.49E-01 | 1 | GPSM1 | 5.75E-01 | 1 |
| IL4R | 4.49E-01 | 1 | FSD2 | 5.75E-01 | 1 |
| NAALAD | 4.49E-01 | 1 | SORT1 | 5.75E-01 | 1 |
| BHMT2 | 4.49E-01 | 1 | ZNF611 | 5.75E-01 | 1 |
| CTDSPL | 4.49E-01 | 1 | ZNF727 | 5.75E-01 | 1 |
| AGPAT5 | 4.49E-01 | 1 | CYP2C9 | 5.75E-01 | 1 |
| FMR1 | 4.49E-01 | 1 | TGM7 | 5.76E-01 | 1 |
| YTHDF1 | 4.49E-01 | 1 | MRPL32 | 5.76E-01 | 1 |
| LAMP3 | 4.50E-01 | 1 | PPIAL4A | 5.76E-01 | 1 |
| DENND2C | 4.50E-01 | 1 | POSTN | 5.76E-01 | 1 |
| ABCA4 | 4.50E-01 | 1 | COL27A1 | 5.76E-01 | 1 |
| PRR23B | 4.50E-01 | 1 | KRT1 | 5.76E-01 | 1 |
| LARS2 | 4.50E-01 | 1 | PHLDB2 | 5.76E-01 | 1 |
| C17orf51 | 4.50E-01 | 1 | DPP8 | 5.77E-01 | 1 |
| B4GALT2 | 4.50E-01 | 1 | LRRC37A3 | 5.77E-01 | 1 |
| KBTBD7 | 4.50E-01 | 1 | TARS2 | 5.77E-01 | 1 |
| OR2J2 | 4.50E-01 | 1 | UNC13D | 5.77E-01 | 1 |
| MTAP | 4.50E-01 | 1 | ZFP91 | 5.77E-01 | 1 |
| N4BP3 | 4.51E-01 | 1 | SCN1A | 5.77E-01 | 1 |
| RP1 | 4.51E-01 | 1 | PLEKHO1 | 5.78E-01 | 1 |
| ATP13A1 | 4.51E-01 | 1 | SLC13A1 | 5.78E-01 | 1 |
| POLG2 | 4.51E-01 | 1 | ZNF766 | 5.78E-01 | 1 |
| TEKT4 | 4.51E-01 | 1 | SLC12A4 | 5.78E-01 | 1 |
| ICAM1 | 4.51E-01 | 1 | CDH9 | 5.78E-01 | 1 |
| TREML1 | 4.51E-01 | 1 | MAOA | 5.78E-01 | 1 |
| LCN2 | 4.51E-01 | 1 | FASTKD1 | 5.78E-01 | 1 |
| PDLIM2 | 4.51E-01 | 1 | PLOD3 | 5.79E-01 | 1 |
| MASP2 | 4.51E-01 | 1 | RBM12B | 5.79E-01 | 1 |
| ZNF248 | 4.51E-01 | 1 | ATP6V0A4 | 5.79E-01 | 1 |
| MAGED1 | 4.52E-01 | 1 | TRMT5 | 5.79E-01 | 1 |
| FASTK | 4.52E-01 | 1 | MLKL | 5.79E-01 | 1 |
| PRSS38 | 4.52E-01 | 1 | DDX41 | 5.79E-01 | 1 |
| HNRNPA | 4.52E-01 | 1 | ANO10 | 5.79E-01 | 1 |
| PPIP5K1 | 4.52E-01 | 1 | KIAA1683 | 5.80E-01 | 1 |
| CNNM2 | 4.52E-01 | 1 | PRKD2 | 5.80E-01 | 1 |
| NDST2 | 4.52E-01 | 1 | KRT72 | 5.80E-01 | 1 |
| ADAM18 | 4.52E-01 | 1 | PAK4 | 5.80E-01 | 1 |
| TPD52L1 | 4.53E-01 | 1 | FNIP1 | 5.80E-01 | 1 |
| EPHA5 | 4.53E-01 | 1 | JMJD1C | 5.80E-01 | 1 |
| KIF5C | 4.53E-01 | 1 | JAKMIP3 | 5.81E-01 | 1 |
| C2orf74 | 4.53E-01 | 1 | ANLN | 5.81E-01 | 1 |
| CUZD1 | 4.53E-01 | 1 | WDFY2 | 5.81E-01 | 1 |
| SYNPR | 4.53E-01 | 1 | SEMA7A | 5.81E-01 | 1 |
| NPFFR1 | 4.53E-01 | 1 | MRVI1 | 5.82E-01 | 1 |

| FGF10 | 4.53E-01 | 1 | TAF1C | 5.82E-01 | 1 |
| --- | --- | --- | --- | --- | --- |
| JAKMIP3 | 4.53E-01 | 1 | LAMC2 | 5.82E-01 | 1 |
| PIGU | 4.53E-01 | 1 | CASC1 | 5.82E-01 | 1 |
| CD1B | 4.54E-01 | 1 | DAK | 5.82E-01 | 1 |
| SEPHS2 | 4.54E-01 | 1 | PAPSS2 | 5.82E-01 | 1 |
| PBX2 | 4.54E-01 | 1 | ESPN | 5.83E-01 | 1 |
| SENP5 | 4.54E-01 | 1 | ARHGAP18 | 5.83E-01 | 1 |
| OR3A1 | 4.55E-01 | 1 | ABCA1 | 5.83E-01 | 1 |
| HLA-A | 4.55E-01 | 1 | SHC1 | 5.83E-01 | 1 |
| WDR88 | 4.55E-01 | 1 | ZNF91 | 5.83E-01 | 1 |
| CTH | 4.55E-01 | 1 | IL1RL2 | 5.83E-01 | 1 |
| ANGPTL7 | 4.55E-01 | 1 | ZNF695 | 5.83E-01 | 1 |
| SLC4A1 | 4.55E-01 | 1 | STRN4 | 5.83E-01 | 1 |
| DVL2 | 4.55E-01 | 1 | CRY1 | 5.83E-01 | 1 |
| CD1A | 4.55E-01 | 1 | FIGNL1 | 5.83E-01 | 1 |
| NLK | 4.55E-01 | 1 | IPO5 | 5.84E-01 | 1 |
| TOR1B | 4.55E-01 | 1 | CYP2A6 | 5.84E-01 | 1 |
| STK38 | 4.55E-01 | 1 | NT5E | 5.84E-01 | 1 |
| ACOT1 | 4.56E-01 | 1 | PABPC3 | 5.84E-01 | 1 |
| KIAA0355 | 4.56E-01 | 1 | MIPOL1 | 5.84E-01 | 1 |
| NMUR1 | 4.56E-01 | 1 | ERC1 | 5.84E-01 | 1 |
| FERMT2 | 4.56E-01 | 1 | STOX1 | 5.84E-01 | 1 |
| OR1A1 | 4.56E-01 | 1 | RASAL1 | 5.85E-01 | 1 |
| SH3GL3 | 4.56E-01 | 1 | SV2C | 5.85E-01 | 1 |
| NRD1 | 4.56E-01 | 1 | STIM1 | 5.85E-01 | 1 |
| NFKBIZ | 4.56E-01 | 1 | DSP | 5.85E-01 | 1 |
| TMEM192 | 4.56E-01 | 1 | PGBD1 | 5.85E-01 | 1 |
| CRYBB1 | 4.56E-01 | 1 | ATXN7L2 | 5.85E-01 | 1 |
| AKR1D1 | 4.56E-01 | 1 | DCX | 5.85E-01 | 1 |
| COBL | 4.56E-01 | 1 | MPP2 | 5.85E-01 | 1 |
| OR10A7 | 4.56E-01 | 1 | ZNF43 | 5.85E-01 | 1 |
| SLC35D1 | 4.56E-01 | 1 | KCNN1 | 5.85E-01 | 1 |
| CHRNA6 | 4.56E-01 | 1 | C11orf80 | 5.86E-01 | 1 |
| MYOCD | 4.57E-01 | 1 | PCDHB15 | 5.86E-01 | 1 |
| TPX2 | 4.57E-01 | 1 | KRT37 | 5.86E-01 | 1 |
| PDZRN4 | 4.57E-01 | 1 | ZFP36L2 | 5.86E-01 | 1 |
| HSPA5 | 4.57E-01 | 1 | FMNL3 | 5.86E-01 | 1 |
| CPNE2 | 4.57E-01 | 1 | CNGA2 | 5.86E-01 | 1 |
| SPARC | 4.57E-01 | 1 | BCAT1 | 5.86E-01 | 1 |
| TRIM2 | 4.57E-01 | 1 | ADAM21 | 5.87E-01 | 1 |
| PCDHB13 | 4.58E-01 | 1 | CECR1 | 5.87E-01 | 1 |
| TAOK3 | 4.58E-01 | 1 | LPIN1 | 5.87E-01 | 1 |
| LPIN1 | 4.58E-01 | 1 | KLHL14 | 5.87E-01 | 1 |
| HERC6 | 4.58E-01 | 1 | LHX9 | 5.87E-01 | 1 |
| OR4F4 | 4.58E-01 | 1 | TMC4 | 5.88E-01 | 1 |
| MLST8 | 4.58E-01 | 1 | TTC12 | 5.88E-01 | 1 |
| TMEM45A | 4.58E-01 | 1 | ZRANB1 | 5.88E-01 | 1 |
| CDHR4 | 4.58E-01 | 1 | ZNF671 | 5.88E-01 | 1 |
| PHEX | 4.58E-01 | 1 | DSPP | 5.88E-01 | 1 |
| LILRA5 | 4.58E-01 | 1 | GLUL | 5.88E-01 | 1 |
| USP54 | 4.58E-01 | 1 | CSF3R | 5.88E-01 | 1 |
| GSK3B | 4.59E-01 | 1 | PRR16 | 5.88E-01 | 1 |
| SYT8 | 4.59E-01 | 1 | ZNF442 | 5.88E-01 | 1 |

| SUSD5 | 4.59E-01 | 1 | RXFP1 | 5.88E-01 | 1 |
| --- | --- | --- | --- | --- | --- |
| TAS2R13 | 4.59E-01 | 1 | MCOLN2 | 5.89E-01 | 1 |
| SDHB | 4.59E-01 | 1 | ATHL1 | 5.89E-01 | 1 |
| OR5AP2 | 4.59E-01 | 1 | AIM1L | 5.89E-01 | 1 |
| SLC2A4R | 4.59E-01 | 1 | IL4I1 | 5.89E-01 | 1 |
| LRRC47 | 4.59E-01 | 1 | RECQL | 5.89E-01 | 1 |
| ALKBH3 | 4.59E-01 | 1 | ZNF148 | 5.89E-01 | 1 |
| RAB40A | 4.59E-01 | 1 | BTN3A1 | 5.89E-01 | 1 |
| ZNF518A | 4.59E-01 | 1 | FAM184A | 5.90E-01 | 1 |
| TERF2 | 4.59E-01 | 1 | POC1B | 5.90E-01 | 1 |
| DENND1C | 4.59E-01 | 1 | KRT74 | 5.90E-01 | 1 |
| MPST | 4.60E-01 | 1 | C9 | 5.90E-01 | 1 |
| MDFIC | 4.60E-01 | 1 | LRRC48 | 5.90E-01 | 1 |
| TTLL9 | 4.60E-01 | 1 | SLC26A2 | 5.90E-01 | 1 |
| MYCBPA | 4.60E-01 | 1 | MYO5A | 5.91E-01 | 1 |
| GBE1 | 4.60E-01 | 1 | ANKRD34C | 5.91E-01 | 1 |
| FGF21 | 4.60E-01 | 1 | AKNAD1 | 5.91E-01 | 1 |
| FGR | 4.60E-01 | 1 | C1orf111 | 5.91E-01 | 1 |
| SPESP1 | 4.60E-01 | 1 | CLPTM1L | 5.91E-01 | 1 |
| ATP6V0A | 4.60E-01 | 1 | PCDHAC2 | 5.91E-01 | 1 |
| SERPING1 | 4.60E-01 | 1 | LAMC1 | 5.91E-01 | 1 |
| SPG20 | 4.60E-01 | 1 | ELMO3 | 5.91E-01 | 1 |
| ANXA3 | 4.60E-01 | 1 | BMP4 | 5.92E-01 | 1 |
| RAB11FIP | 4.60E-01 | 1 | ADCY9 | 5.92E-01 | 1 |
| SPSB4 | 4.60E-01 | 1 | UNK | 5.92E-01 | 1 |
| OSCP1 | 4.60E-01 | 1 | ETS1 | 5.92E-01 | 1 |
| KRT83 | 4.60E-01 | 1 | SLC44A5 | 5.92E-01 | 1 |
| PACSIN1 | 4.60E-01 | 1 | DAB1 | 5.92E-01 | 1 |
| PRR5 | 4.61E-01 | 1 | C12orf56 | 5.92E-01 | 1 |
| OR2T35 | 4.61E-01 | 1 | ADRB2 | 5.93E-01 | 1 |
| SLC25A3 | 4.61E-01 | 1 | TMC5 | 5.93E-01 | 1 |
| MLYCD | 4.61E-01 | 1 | IL18RAP | 5.93E-01 | 1 |
| MDH1 | 4.61E-01 | 1 | PPFIA1 | 5.93E-01 | 1 |
| SGK2 | 4.61E-01 | 1 | WDR78 | 5.93E-01 | 1 |
| ITIH5 | 4.61E-01 | 1 | CAPN10 | 5.93E-01 | 1 |
| ZMYND12 | 4.61E-01 | 1 | C5orf22 | 5.94E-01 | 1 |
| OR10X1 | 4.61E-01 | 1 | TUBGCP5 | 5.94E-01 | 1 |
| SKIV2L | 4.61E-01 | 1 | EIF3D | 5.95E-01 | 1 |
| P2RX6 | 4.61E-01 | 1 | ETF1 | 5.95E-01 | 1 |
| BTBD2 | 4.61E-01 | 1 | EMILIN2 | 5.95E-01 | 1 |
| ACSL6 | 4.61E-01 | 1 | ASTN1 | 5.95E-01 | 1 |
| NFYA | 4.62E-01 | 1 | GLB1 | 5.96E-01 | 1 |
| PTPN13 | 4.62E-01 | 1 | HTR2C | 5.96E-01 | 1 |
| SYT13 | 4.62E-01 | 1 | SCN4A | 5.96E-01 | 1 |
| ITPRIPL1 | 4.62E-01 | 1 | SLC7A11 | 5.97E-01 | 1 |
| MTOR | 4.62E-01 | 1 | RFX1 | 5.97E-01 | 1 |
| TUFM | 4.62E-01 | 1 | MAP3K7 | 5.97E-01 | 1 |
| C17orf96 | 4.62E-01 | 1 | PRSS37 | 5.97E-01 | 1 |
| MAP4K4 | 4.62E-01 | 1 | ADAD1 | 5.97E-01 | 1 |
| PBX4 | 4.62E-01 | 1 | MCF2L | 5.97E-01 | 1 |
| CHST13 | 4.62E-01 | 1 | FLAD1 | 5.97E-01 | 1 |
| SORCS2 | 4.62E-01 | 1 | KRT71 | 5.98E-01 | 1 |
| ASB12 | 4.62E-01 | 1 | RNF216 | 5.98E-01 | 1 |

| TOR3A | 4.62E-01 | 1 | TRPV3 | 5.98E-01 | 1 |
| --- | --- | --- | --- | --- | --- |
| MAP3K7 | 4.63E-01 | 1 | NPTN | 5.98E-01 | 1 |
| WNT5B | 4.63E-01 | 1 | ALAS1 | 5.98E-01 | 1 |
| ERGIC3 | 4.63E-01 | 1 | TRPV4 | 5.99E-01 | 1 |
| FBXL6 | 4.63E-01 | 1 | DYRK4 | 5.99E-01 | 1 |
| EGFL7 | 4.63E-01 | 1 | ZNF280C | 5.99E-01 | 1 |
| BIRC6 | 4.63E-01 | 1 | CEP164 | 5.99E-01 | 1 |
| CLEC1A | 4.63E-01 | 1 | PHKA2 | 5.99E-01 | 1 |
| ARG1 | 4.63E-01 | 1 | ARHGAP10 | 5.99E-01 | 1 |
| SH3BP4 | 4.63E-01 | 1 | SLC2A12 | 6.00E-01 | 1 |
| SERPINA1 | 4.63E-01 | 1 | EIF5B | 6.00E-01 | 1 |
| MAP3K2 | 4.63E-01 | 1 | PPP2R5D | 6.00E-01 | 1 |
| OR5B3 | 4.63E-01 | 1 | ZFP36L1 | 6.00E-01 | 1 |
| TLE6 | 4.63E-01 | 1 | ARHGEF11 | 6.00E-01 | 1 |
| PLOD2 | 4.63E-01 | 1 | GTF2I | 6.00E-01 | 1 |
| SETD1A | 4.64E-01 | 1 | ZNF713 | 6.00E-01 | 1 |
| CEACAM | 4.64E-01 | 1 | GABRA5 | 6.01E-01 | 1 |
| CD1C | 4.64E-01 | 1 | CPNE9 | 6.01E-01 | 1 |
| C8orf76 | 4.64E-01 | 1 | AKAP12 | 6.01E-01 | 1 |
| UBASH3A | 4.64E-01 | 1 | ADAMTS10 | 6.01E-01 | 1 |
| NUP188 | 4.64E-01 | 1 | PGR | 6.01E-01 | 1 |
| RNF135 | 4.64E-01 | 1 | SLC24A5 | 6.01E-01 | 1 |
| TROAP | 4.64E-01 | 1 | SLC45A1 | 6.01E-01 | 1 |
| RGS1 | 4.64E-01 | 1 | DFNA5 | 6.01E-01 | 1 |
| MED14 | 4.64E-01 | 1 | CHRNB3 | 6.02E-01 | 1 |
| GSTO1 | 4.64E-01 | 1 | CYLD | 6.02E-01 | 1 |
| ABCE1 | 4.64E-01 | 1 | HSD3B1 | 6.02E-01 | 1 |
| BACE1 | 4.64E-01 | 1 | ASCC2 | 6.02E-01 | 1 |
| KIAA0895 | 4.65E-01 | 1 | FHAD1 | 6.02E-01 | 1 |
| MDM1 | 4.65E-01 | 1 | MOCOS | 6.03E-01 | 1 |
| VN1R2 | 4.65E-01 | 1 | RBM33 | 6.03E-01 | 1 |
| NTN1 | 4.65E-01 | 1 | SSX2IP | 6.03E-01 | 1 |
| OR1J1 | 4.65E-01 | 1 | FBXW7 | 6.03E-01 | 1 |
| PAX4 | 4.65E-01 | 1 | EPHA7 | 6.03E-01 | 1 |
| ARMC9 | 4.65E-01 | 1 | LAMA4 | 6.03E-01 | 1 |
| PHF13 | 4.65E-01 | 1 | LIMA1 | 6.04E-01 | 1 |
| AGBL2 | 4.65E-01 | 1 | WARS2 | 6.04E-01 | 1 |
| RDH10 | 4.65E-01 | 1 | HSPA12A | 6.04E-01 | 1 |
| SLC9A6 | 4.65E-01 | 1 | RSC1A1 | 6.04E-01 | 1 |
| HOXD12 | 4.65E-01 | 1 | GRIK4 | 6.04E-01 | 1 |
| SAGE1 | 4.66E-01 | 1 | THAP4 | 6.04E-01 | 1 |
| OR1N1 | 4.66E-01 | 1 | UNC80 | 6.04E-01 | 1 |
| SCML1 | 4.66E-01 | 1 | CASK | 6.04E-01 | 1 |
| RCCD1 | 4.66E-01 | 1 | DNAJC6 | 6.04E-01 | 1 |
| DNAH17 | 4.66E-01 | 1 | DCBLD2 | 6.04E-01 | 1 |
| ZNF257 | 4.66E-01 | 1 | PYROXD1 | 6.05E-01 | 1 |
| RFTN2 | 4.66E-01 | 1 | AGAP4 | 6.05E-01 | 1 |
| MYF6 | 4.66E-01 | 1 | SCNN1G | 6.06E-01 | 1 |
| TRNT1 | 4.67E-01 | 1 | ST8SIA2 | 6.06E-01 | 1 |
| PNLIPRP1 | 4.67E-01 | 1 | MAP7D3 | 6.06E-01 | 1 |
| FUT5 | 4.67E-01 | 1 | TASP1 | 6.06E-01 | 1 |
| MED17 | 4.67E-01 | 1 | LYN | 6.06E-01 | 1 |
| MSLNL | 4.67E-01 | 1 | SLCO1B1 | 6.07E-01 | 1 |

| ZNF786 | 4.67E-01 | 1 | CRBN | 6.07E-01 | 1 |
| --- | --- | --- | --- | --- | --- |
| SH3BP5L | 4.67E-01 | 1 | PATZ1 | 6.07E-01 | 1 |
| STARD7 | 4.67E-01 | 1 | TRPC1 | 6.07E-01 | 1 |
| FEN1 | 4.67E-01 | 1 | NRXN3 | 6.07E-01 | 1 |
| USP8 | 4.67E-01 | 1 | NLRX1 | 6.07E-01 | 1 |
| CCDC71 | 4.67E-01 | 1 | TMEM151B | 6.07E-01 | 1 |
| CD109 | 4.67E-01 | 1 | ZNF853 | 6.07E-01 | 1 |
| DLEC1 | 4.67E-01 | 1 | TAB3 | 6.08E-01 | 1 |
| TRIM54 | 4.67E-01 | 1 | PRKRIR | 6.08E-01 | 1 |
| NEUROD6 | 4.68E-01 | 1 | BRWD1 | 6.08E-01 | 1 |
| TTBK1 | 4.68E-01 | 1 | PLEKHM2 | 6.08E-01 | 1 |
| GJB4 | 4.68E-01 | 1 | N4BP2L2 | 6.09E-01 | 1 |
| WNT7B | 4.68E-01 | 1 | CAPS2 | 6.09E-01 | 1 |
| PDIA6 | 4.68E-01 | 1 | ATP1A1 | 6.09E-01 | 1 |
| CASQ1 | 4.69E-01 | 1 | FZD1 | 6.09E-01 | 1 |
| PTH2R | 4.69E-01 | 1 | EML5 | 6.09E-01 | 1 |
| ACR | 4.69E-01 | 1 | GABRA3 | 6.10E-01 | 1 |
| DGCR8 | 4.69E-01 | 1 | IFT52 | 6.10E-01 | 1 |
| CD209 | 4.69E-01 | 1 | DDX20 | 6.10E-01 | 1 |
| ACAA2 | 4.69E-01 | 1 | RFX6 | 6.10E-01 | 1 |
| RAB3IP | 4.69E-01 | 1 | UGT2B17 | 6.10E-01 | 1 |
| TBL3 | 4.70E-01 | 1 | SCNN1B | 6.10E-01 | 1 |
| USPL1 | 4.70E-01 | 1 | F2 | 6.10E-01 | 1 |
| CACNA1G | 4.70E-01 | 1 | PMPCB | 6.11E-01 | 1 |
| OXSM | 4.70E-01 | 1 | DNMT1 | 6.11E-01 | 1 |
| SAG | 4.70E-01 | 1 | GEMIN8 | 6.11E-01 | 1 |
| TMEM38A | 4.70E-01 | 1 | KALRN | 6.11E-01 | 1 |
| APBA2 | 4.70E-01 | 1 | CHERP | 6.11E-01 | 1 |
| ANKRD12 | 4.70E-01 | 1 | MYH4 | 6.11E-01 | 1 |
| CEP120 | 4.70E-01 | 1 | ADCY6 | 6.11E-01 | 1 |
| VIM | 4.70E-01 | 1 | SLC43A1 | 6.11E-01 | 1 |
| TMEM202 | 4.70E-01 | 1 | EXT1 | 6.11E-01 | 1 |
| MTHFD2L | 4.71E-01 | 1 | NEK9 | 6.11E-01 | 1 |
| STAB1 | 4.71E-01 | 1 | ADAMTS18 | 6.11E-01 | 1 |
| MYT1 | 4.71E-01 | 1 | PKHD1L1 | 6.12E-01 | 1 |
| SLC35E4 | 4.71E-01 | 1 | ADCYAP1R | 6.12E-01 | 1 |
| HAUS8 | 4.71E-01 | 1 | KIAA2026 | 6.12E-01 | 1 |
| TAF7L | 4.71E-01 | 1 | HR | 6.12E-01 | 1 |
| SMARCA | 4.71E-01 | 1 | UGT1A3 | 6.12E-01 | 1 |
| PLCXD1 | 4.71E-01 | 1 | HIRIP3 | 6.13E-01 | 1 |
| SERGEF | 4.71E-01 | 1 | HTR5A | 6.13E-01 | 1 |
| LRP5 | 4.71E-01 | 1 | TPO | 6.13E-01 | 1 |
| FOXG1 | 4.71E-01 | 1 | SH3RF3 | 6.13E-01 | 1 |
| TREML2 | 4.71E-01 | 1 | AMPD1 | 6.14E-01 | 1 |
| NCOA6 | 4.72E-01 | 1 | CARD10 | 6.14E-01 | 1 |
| ST8SIA2 | 4.72E-01 | 1 | BICD2 | 6.14E-01 | 1 |
| NHS | 4.72E-01 | 1 | COBLL1 | 6.14E-01 | 1 |
| SCAMP1 | 4.72E-01 | 1 | TEX11 | 6.14E-01 | 1 |
| SBF1 | 4.72E-01 | 1 | UBR2 | 6.14E-01 | 1 |
| BCAT2 | 4.72E-01 | 1 | CPNE1 | 6.15E-01 | 1 |
| MTNR1B | 4.72E-01 | 1 | USP8 | 6.15E-01 | 1 |
| PIM1 | 4.72E-01 | 1 | GLRA2 | 6.15E-01 | 1 |
| DENND3 | 4.72E-01 | 1 | EPS8L3 | 6.15E-01 | 1 |

| UROC1 | 4.72E-01 | 1 | OTOP2 | 6.15E-01 | 1 |
| --- | --- | --- | --- | --- | --- |
| ZNF560 | 4.72E-01 | 1 | FSIP1 | 6.16E-01 | 1 |
| DEPDC1B | 4.72E-01 | 1 | ZNF486 | 6.16E-01 | 1 |
| KIAA1217 | 4.72E-01 | 1 | XRN1 | 6.16E-01 | 1 |
| C14orf39 | 4.73E-01 | 1 | CSN1S1 | 6.16E-01 | 1 |
| DMPK | 4.73E-01 | 1 | DGKE | 6.16E-01 | 1 |
| ZNF528 | 4.73E-01 | 1 | EXOC3 | 6.16E-01 | 1 |
| TMEM163 | 4.73E-01 | 1 | CPSF1 | 6.16E-01 | 1 |
| ANKHD1- | 4.73E-01 | 1 | FOXP1 | 6.16E-01 | 1 |
| LCA5 | 4.73E-01 | 1 | ZFP82 | 6.16E-01 | 1 |
| RASAL2 | 4.73E-01 | 1 | GIMAP8 | 6.17E-01 | 1 |
| OSBPL8 | 4.73E-01 | 1 | SLIT2 | 6.17E-01 | 1 |
| OR4C6 | 4.73E-01 | 1 | NID2 | 6.17E-01 | 1 |
| HDAC5 | 4.73E-01 | 1 | JAKMIP1 | 6.17E-01 | 1 |
| PTPN2 | 4.73E-01 | 1 | BAZ1B | 6.17E-01 | 1 |
| GLRA4 | 4.73E-01 | 1 | PCOLCE2 | 6.17E-01 | 1 |
| API5 | 4.73E-01 | 1 | FBXL19 | 6.17E-01 | 1 |
| ZCCHC16 | 4.73E-01 | 1 | PPP1R10 | 6.18E-01 | 1 |
| FOS | 4.73E-01 | 1 | PRR12 | 6.18E-01 | 1 |
| FOXJ2 | 4.73E-01 | 1 | TSNARE1 | 6.18E-01 | 1 |
| FOXD1 | 4.73E-01 | 1 | IKBKB | 6.18E-01 | 1 |
| SLC25A24 | 4.73E-01 | 1 | VWCE | 6.18E-01 | 1 |
| MYO16 | 4.74E-01 | 1 | ACO1 | 6.18E-01 | 1 |
| FGF20 | 4.74E-01 | 1 | RUFY3 | 6.19E-01 | 1 |
| DACT2 | 4.74E-01 | 1 | MUC17 | 6.19E-01 | 1 |
| PLEKHG4 | 4.74E-01 | 1 | ZNF526 | 6.19E-01 | 1 |
| ZNF362 | 4.74E-01 | 1 | PCDHB6 | 6.19E-01 | 1 |
| GPR149 | 4.74E-01 | 1 | KCNQ3 | 6.19E-01 | 1 |
| TAS2R20 | 4.74E-01 | 1 | NUDCD1 | 6.20E-01 | 1 |
| CHL1 | 4.74E-01 | 1 | TKTL2 | 6.20E-01 | 1 |
| OR7G3 | 4.74E-01 | 1 | MOSPD2 | 6.20E-01 | 1 |
| GPR141 | 4.74E-01 | 1 | CDHR3 | 6.20E-01 | 1 |
| PRCC | 4.74E-01 | 1 | MUC5B | 6.20E-01 | 1 |
| ODC1 | 4.74E-01 | 1 | UNC13A | 6.20E-01 | 1 |
| USP45 | 4.74E-01 | 1 | IL31RA | 6.20E-01 | 1 |
| FAM81A | 4.74E-01 | 1 | OR2T3 | 6.20E-01 | 1 |
| PCDH11X | 4.74E-01 | 1 | KATNAL2 | 6.20E-01 | 1 |
| SNIP1 | 4.74E-01 | 1 | ZNF592 | 6.21E-01 | 1 |
| SDC3 | 4.74E-01 | 1 | WDR33 | 6.21E-01 | 1 |
| OR9A2 | 4.74E-01 | 1 | TMEM64 | 6.21E-01 | 1 |
| TAS2R31 | 4.75E-01 | 1 | ADCY3 | 6.21E-01 | 1 |
| PREB | 4.75E-01 | 1 | ABCC4 | 6.21E-01 | 1 |
| ABRA | 4.75E-01 | 1 | TCF7L2 | 6.21E-01 | 1 |
| YTHDC1 | 4.75E-01 | 1 | DDX11 | 6.21E-01 | 1 |
| TSNARE1 | 4.75E-01 | 1 | NLRP3 | 6.21E-01 | 1 |
| OR8D1 | 4.75E-01 | 1 | TP53BP1 | 6.21E-01 | 1 |
| VSIG10L | 4.75E-01 | 1 | STAB1 | 6.22E-01 | 1 |
| BOP1 | 4.75E-01 | 1 | TAF3 | 6.22E-01 | 1 |
| ZNF675 | 4.75E-01 | 1 | ADAM7 | 6.22E-01 | 1 |
| CCT7 | 4.75E-01 | 1 | CEACAM5 | 6.22E-01 | 1 |
| FAM160B | 4.75E-01 | 1 | DHX37 | 6.22E-01 | 1 |
| FUZ | 4.76E-01 | 1 | RNF40 | 6.22E-01 | 1 |
| CKMT2 | 4.76E-01 | 1 | RHPN1 | 6.22E-01 | 1 |

| SFMBT1 | 4.76E-01 | 1 | PCDHB2 | 6.22E-01 | 1 |
| --- | --- | --- | --- | --- | --- |
| MED13 | 4.76E-01 | 1 | AKAP10 | 6.23E-01 | 1 |
| ACTN3 | 4.76E-01 | 1 | COL1A2 | 6.23E-01 | 1 |
| GLUD1 | 4.76E-01 | 1 | LILRB4 | 6.23E-01 | 1 |
| TMEM79 | 4.76E-01 | 1 | ARMC3 | 6.23E-01 | 1 |
| CCR1 | 4.76E-01 | 1 | SALL1 | 6.23E-01 | 1 |
| RAP1GAP | 4.76E-01 | 1 | KIAA1462 | 6.24E-01 | 1 |
| OR4P4 | 4.76E-01 | 1 | ZNF71 | 6.24E-01 | 1 |
| FAM181A | 4.77E-01 | 1 | LILRA1 | 6.24E-01 | 1 |
| C2CD4B | 4.77E-01 | 1 | CRNN | 6.24E-01 | 1 |
| ZNF285 | 4.77E-01 | 1 | ZNF17 | 6.24E-01 | 1 |
| RDH12 | 4.77E-01 | 1 | AKAP13 | 6.24E-01 | 1 |
| OR4K15 | 4.77E-01 | 1 | NUP210L | 6.25E-01 | 1 |
| ZNF101 | 4.77E-01 | 1 | ZSCAN29 | 6.25E-01 | 1 |
| TYW1B | 4.77E-01 | 1 | ULK2 | 6.25E-01 | 1 |
| OR4C15 | 4.77E-01 | 1 | NSD1 | 6.25E-01 | 1 |
| MGA | 4.77E-01 | 1 | POU3F2 | 6.25E-01 | 1 |
| ARHGAP1 | 4.77E-01 | 1 | EYA4 | 6.26E-01 | 1 |
| SAV1 | 4.78E-01 | 1 | FANCA | 6.26E-01 | 1 |
| CSNK1G3 | 4.78E-01 | 1 | UBA2 | 6.26E-01 | 1 |
| L3MBTL2 | 4.78E-01 | 1 | IQSEC3 | 6.26E-01 | 1 |
| ZFP57 | 4.78E-01 | 1 | ZNF408 | 6.27E-01 | 1 |
| SPZ1 | 4.78E-01 | 1 | APBB1 | 6.27E-01 | 1 |
| PDIA3 | 4.78E-01 | 1 | COG7 | 6.27E-01 | 1 |
| CHST12 | 4.78E-01 | 1 | MYOF | 6.27E-01 | 1 |
| TTF1 | 4.78E-01 | 1 | ALK | 6.27E-01 | 1 |
| PIP5K1C | 4.79E-01 | 1 | ABCC3 | 6.28E-01 | 1 |
| DCAF11 | 4.79E-01 | 1 | ABCC9 | 6.28E-01 | 1 |
| PUM1 | 4.79E-01 | 1 | ATP2A3 | 6.28E-01 | 1 |
| VAX2 | 4.79E-01 | 1 | COG5 | 6.29E-01 | 1 |
| CALHM3 | 4.79E-01 | 1 | ATP8B2 | 6.29E-01 | 1 |
| TAS2R7 | 4.79E-01 | 1 | DPYD | 6.29E-01 | 1 |
| VAMP1 | 4.79E-01 | 1 | VWA3B | 6.29E-01 | 1 |
| AGAP3 | 4.79E-01 | 1 | RHOBTB1 | 6.29E-01 | 1 |
| PDCL3 | 4.79E-01 | 1 | BEND5 | 6.29E-01 | 1 |
| TEX101 | 4.79E-01 | 1 | QRICH1 | 6.29E-01 | 1 |
| PER2 | 4.79E-01 | 1 | APBB2 | 6.29E-01 | 1 |
| TUBB2B | 4.79E-01 | 1 | CUL2 | 6.30E-01 | 1 |
| EHHADH | 4.79E-01 | 1 | ZNF217 | 6.30E-01 | 1 |
| LRP4 | 4.79E-01 | 1 | EPHA1 | 6.30E-01 | 1 |
| ZNF578 | 4.79E-01 | 1 | PTPN11 | 6.30E-01 | 1 |
| ASAH2 | 4.79E-01 | 1 | ZCCHC6 | 6.30E-01 | 1 |
| LMCD1 | 4.79E-01 | 1 | PNLIPRP1 | 6.30E-01 | 1 |
| TRAK1 | 4.79E-01 | 1 | ZNF709 | 6.31E-01 | 1 |
| TEX14 | 4.80E-01 | 1 | MAGEC2 | 6.31E-01 | 1 |
| TAOK1 | 4.80E-01 | 1 | C14orf169 | 6.31E-01 | 1 |
| ALDH6A1 | 4.80E-01 | 1 | SLC6A20 | 6.31E-01 | 1 |
| TMEM108 | 4.80E-01 | 1 | HUNK | 6.31E-01 | 1 |
| LPCAT3 | 4.80E-01 | 1 | ZNF30 | 6.31E-01 | 1 |
| SOX30 | 4.80E-01 | 1 | ZNF407 | 6.31E-01 | 1 |
| APC | 4.80E-01 | 1 | CLCN3 | 6.31E-01 | 1 |
| OR52D1 | 4.80E-01 | 1 | UNC5D | 6.32E-01 | 1 |
| FCGR2A | 4.80E-01 | 1 | CCDC146 | 6.32E-01 | 1 |

| CALD1 | 4.80E-01 | 1 | KIR3DL3 | 6.32E-01 | 1 |
| --- | --- | --- | --- | --- | --- |
| LAMB4 | 4.80E-01 | 1 | ELMO1 | 6.32E-01 | 1 |
| DPP8 | 4.80E-01 | 1 | KCNA3 | 6.32E-01 | 1 |
| CDH5 | 4.81E-01 | 1 | ARAP1 | 6.33E-01 | 1 |
| REL | 4.81E-01 | 1 | SIM1 | 6.33E-01 | 1 |
| ELMO2 | 4.81E-01 | 1 | ZNF266 | 6.33E-01 | 1 |
| CNP | 4.81E-01 | 1 | NOP56 | 6.33E-01 | 1 |
| TAPBP | 4.81E-01 | 1 | PIK3C2B | 6.33E-01 | 1 |
| ADAM22 | 4.81E-01 | 1 | KIF20B | 6.35E-01 | 1 |
| MTMR12 | 4.81E-01 | 1 | FUT9 | 6.35E-01 | 1 |
| CCDC92 | 4.81E-01 | 1 | MSLN | 6.36E-01 | 1 |
| C2orf73 | 4.81E-01 | 1 | TMEM63B | 6.36E-01 | 1 |
| SEC61A2 | 4.82E-01 | 1 | KIF18A | 6.36E-01 | 1 |
| ADCY3 | 4.82E-01 | 1 | GTF2IRD1 | 6.36E-01 | 1 |
| P2RY10 | 4.82E-01 | 1 | ZNF613 | 6.36E-01 | 1 |
| NTHL1 | 4.82E-01 | 1 | USP35 | 6.36E-01 | 1 |
| GPR137B | 4.82E-01 | 1 | TMC8 | 6.36E-01 | 1 |
| CAMKK1 | 4.82E-01 | 1 | GPR50 | 6.36E-01 | 1 |
| C6orf222 | 4.82E-01 | 1 | RTF1 | 6.37E-01 | 1 |
| OR10G7 | 4.82E-01 | 1 | HAS1 | 6.37E-01 | 1 |
| PM20D1 | 4.82E-01 | 1 | SMARCC2 | 6.37E-01 | 1 |
| OGDH | 4.82E-01 | 1 | DENND4B | 6.37E-01 | 1 |
| CHST1 | 4.83E-01 | 1 | PIWIL1 | 6.38E-01 | 1 |
| BAG4 | 4.83E-01 | 1 | IKZF2 | 6.38E-01 | 1 |
| FAM155A | 4.83E-01 | 1 | TEX2 | 6.38E-01 | 1 |
| PLXNB3 | 4.83E-01 | 1 | ABCB7 | 6.38E-01 | 1 |
| P2RX7 | 4.83E-01 | 1 | ZMIZ2 | 6.38E-01 | 1 |
| CRNKL1 | 4.83E-01 | 1 | CYLC2 | 6.38E-01 | 1 |
| CPNE6 | 4.83E-01 | 1 | EBF4 | 6.38E-01 | 1 |
| EEF1A2 | 4.83E-01 | 1 | TGM6 | 6.39E-01 | 1 |
| RNF44 | 4.83E-01 | 1 | KRT6B | 6.39E-01 | 1 |
| SYN1 | 4.83E-01 | 1 | CSRNP3 | 6.39E-01 | 1 |
| NSUN2 | 4.83E-01 | 1 | TEX14 | 6.39E-01 | 1 |
| C15orf26 | 4.83E-01 | 1 | L3MBTL3 | 6.39E-01 | 1 |
| SIRT1 | 4.83E-01 | 1 | OR5L1 | 6.39E-01 | 1 |
| OR2A14 | 4.84E-01 | 1 | FAM193A | 6.39E-01 | 1 |
| FAM65A | 4.84E-01 | 1 | ADAMTS16 | 6.39E-01 | 1 |
| OR1M1 | 4.84E-01 | 1 | PABPC5 | 6.40E-01 | 1 |
| DLG1 | 4.84E-01 | 1 | DNAH6 | 6.40E-01 | 1 |
| CAB39 | 4.84E-01 | 1 | UBR5 | 6.40E-01 | 1 |
| IL12RB2 | 4.84E-01 | 1 | SLC17A8 | 6.40E-01 | 1 |
| PSMF1 | 4.84E-01 | 1 | SENP7 | 6.40E-01 | 1 |
| CLP1 | 4.84E-01 | 1 | PDE6B | 6.41E-01 | 1 |
| SBNO2 | 4.84E-01 | 1 | USP4 | 6.41E-01 | 1 |
| REV3L | 4.84E-01 | 1 | TNS4 | 6.41E-01 | 1 |
| KIAA1407 | 4.84E-01 | 1 | SENP5 | 6.41E-01 | 1 |
| C19orf26 | 4.84E-01 | 1 | TMPRSS15 | 6.41E-01 | 1 |
| SLC12A8 | 4.84E-01 | 1 | NAF1 | 6.41E-01 | 1 |
| QTRTD1 | 4.85E-01 | 1 | OSBPL6 | 6.42E-01 | 1 |
| TRAF1 | 4.85E-01 | 1 | ZNF391 | 6.42E-01 | 1 |
| LGR4 | 4.85E-01 | 1 | BOC | 6.42E-01 | 1 |
| IMPG1 | 4.85E-01 | 1 | MTMR1 | 6.42E-01 | 1 |
| CALHM1 | 4.85E-01 | 1 | PLK4 | 6.42E-01 | 1 |

| DRP2 | 4.85E-01 | 1 | RGS22 | 6.42E-01 | 1 |
| --- | --- | --- | --- | --- | --- |
| TSPAN19 | 4.85E-01 | 1 | CC2D1A | 6.42E-01 | 1 |
| KCNJ15 | 4.85E-01 | 1 | RGPD4 | 6.42E-01 | 1 |
| HIPK4 | 4.85E-01 | 1 | COL10A1 | 6.43E-01 | 1 |
| SLC34A2 | 4.85E-01 | 1 | DDX42 | 6.43E-01 | 1 |
| SLC1A2 | 4.85E-01 | 1 | OR5D18 | 6.43E-01 | 1 |
| NKIRAS1 | 4.85E-01 | 1 | FPGT | 6.43E-01 | 1 |
| ZBP1 | 4.85E-01 | 1 | SYNPO2 | 6.43E-01 | 1 |
| CYP2D6 | 4.85E-01 | 1 | CNTNAP3 | 6.43E-01 | 1 |
| DHX29 | 4.85E-01 | 1 | SHROOM4 | 6.43E-01 | 1 |
| PPP1R16A | 4.85E-01 | 1 | MAP4K1 | 6.44E-01 | 1 |
| SMARCA | 4.86E-01 | 1 | TECPR1 | 6.44E-01 | 1 |
| ARHGAP2 | 4.86E-01 | 1 | LIMK2 | 6.44E-01 | 1 |
| MRPL37 | 4.86E-01 | 1 | TBX22 | 6.44E-01 | 1 |
| KCTD8 | 4.86E-01 | 1 | PCF11 | 6.44E-01 | 1 |
| ZNF14 | 4.86E-01 | 1 | IQGAP3 | 6.45E-01 | 1 |
| EVX1 | 4.86E-01 | 1 | ZNF677 | 6.45E-01 | 1 |
| SRMS | 4.86E-01 | 1 | PMS1 | 6.45E-01 | 1 |
| PARS2 | 4.86E-01 | 1 | AFTPH | 6.45E-01 | 1 |
| RPS6KA2 | 4.87E-01 | 1 | ZZZ3 | 6.45E-01 | 1 |
| SYNGAP1 | 4.87E-01 | 1 | DMXL2 | 6.45E-01 | 1 |
| FAS | 4.87E-01 | 1 | BRPF1 | 6.45E-01 | 1 |
| UBTFL1 | 4.87E-01 | 1 | LEO1 | 6.45E-01 | 1 |
| OR2A12 | 4.87E-01 | 1 | HCN1 | 6.46E-01 | 1 |
| HCN4 | 4.87E-01 | 1 | WFS1 | 6.46E-01 | 1 |
| EPDR1 | 4.87E-01 | 1 | EXOC7 | 6.46E-01 | 1 |
| ADAM11 | 4.87E-01 | 1 | GFM2 | 6.46E-01 | 1 |
| KDM2A | 4.87E-01 | 1 | KLHL13 | 6.46E-01 | 1 |
| AKT2 | 4.87E-01 | 1 | PAMR1 | 6.47E-01 | 1 |
| AMY2B | 4.87E-01 | 1 | GOLIM4 | 6.47E-01 | 1 |
| BMP7 | 4.88E-01 | 1 | NLRP6 | 6.47E-01 | 1 |
| ADAM17 | 4.88E-01 | 1 | ANKRD52 | 6.47E-01 | 1 |
| ABCA9 | 4.88E-01 | 1 | PNPLA8 | 6.48E-01 | 1 |
| USP4 | 4.88E-01 | 1 | LRCH2 | 6.48E-01 | 1 |
| CDCP2 | 4.88E-01 | 1 | PGM2 | 6.48E-01 | 1 |
| CHAT | 4.88E-01 | 1 | SLC26A7 | 6.48E-01 | 1 |
| LYPD4 | 4.88E-01 | 1 | PARD3B | 6.48E-01 | 1 |
| ST7L | 4.88E-01 | 1 | SPAG16 | 6.48E-01 | 1 |
| ASH1L | 4.88E-01 | 1 | RYR3 | 6.48E-01 | 1 |
| HFE2 | 4.88E-01 | 1 | LDLR | 6.49E-01 | 1 |
| TMEM225 | 4.88E-01 | 1 | PPOX | 6.49E-01 | 1 |
| PARP11 | 4.88E-01 | 1 | SHCBP1 | 6.49E-01 | 1 |
| SF3A3 | 4.89E-01 | 1 | ZNF394 | 6.49E-01 | 1 |
| NPHP4 | 4.89E-01 | 1 | TSNAXIP1 | 6.49E-01 | 1 |
| RNF8 | 4.89E-01 | 1 | PODXL2 | 6.50E-01 | 1 |
| PSMC1 | 4.89E-01 | 1 | ZNF175 | 6.50E-01 | 1 |
| ATRX | 4.89E-01 | 1 | ESPL1 | 6.50E-01 | 1 |
| GLP2R | 4.89E-01 | 1 | FAM135B | 6.50E-01 | 1 |
| PDLIM4 | 4.89E-01 | 1 | ZNF334 | 6.50E-01 | 1 |
| RAB3IL1 | 4.89E-01 | 1 | CSTF2T | 6.50E-01 | 1 |
| OR1D2 | 4.89E-01 | 1 | CLIP1 | 6.50E-01 | 1 |
| MGAM | 4.89E-01 | 1 | SYT16 | 6.51E-01 | 1 |
| PRPS1L1 | 4.89E-01 | 1 | SASH1 | 6.51E-01 | 1 |

| FOXN3 | 4.89E-01 | 1 | DTX4 | 6.51E-01 | 1 |
| --- | --- | --- | --- | --- | --- |
| GPRIN2 | 4.89E-01 | 1 | ERN1 | 6.51E-01 | 1 |
| KAL1 | 4.89E-01 | 1 | NOBOX | 6.52E-01 | 1 |
| IL10RA | 4.89E-01 | 1 | RIN3 | 6.52E-01 | 1 |
| LIAS | 4.89E-01 | 1 | TDRD1 | 6.52E-01 | 1 |
| ASAP1 | 4.89E-01 | 1 | AKAP3 | 6.53E-01 | 1 |
| RPS6KA4 | 4.89E-01 | 1 | ZNF595 | 6.53E-01 | 1 |
| SETMAR | 4.89E-01 | 1 | MTNR1B | 6.53E-01 | 1 |
| PLEKHG2 | 4.89E-01 | 1 | COL4A1 | 6.53E-01 | 1 |
| SKIV2L2 | 4.89E-01 | 1 | ASB2 | 6.53E-01 | 1 |
| ZKSCAN3 | 4.90E-01 | 1 | ABCD1 | 6.53E-01 | 1 |
| OSGEPL1 | 4.90E-01 | 1 | ADAMTS6 | 6.54E-01 | 1 |
| C2orf71 | 4.90E-01 | 1 | TBC1D9 | 6.54E-01 | 1 |
| KLHL15 | 4.90E-01 | 1 | STK10 | 6.54E-01 | 1 |
| MIDN | 4.90E-01 | 1 | SLC44A3 | 6.54E-01 | 1 |
| OR14I1 | 4.90E-01 | 1 | C17orf70 | 6.54E-01 | 1 |
| KIFAP3 | 4.90E-01 | 1 | SP4 | 6.54E-01 | 1 |
| HECW1 | 4.90E-01 | 1 | MYH9 | 6.54E-01 | 1 |
| OR56B4 | 4.90E-01 | 1 | SLITRK2 | 6.54E-01 | 1 |
| SUMF1 | 4.90E-01 | 1 | PHTF2 | 6.54E-01 | 1 |
| CPN1 | 4.90E-01 | 1 | TRIM22 | 6.54E-01 | 1 |
| KCNA4 | 4.90E-01 | 1 | MATN2 | 6.54E-01 | 1 |
| SAMD1 | 4.90E-01 | 1 | ZMYM1 | 6.54E-01 | 1 |
| ABCC1 | 4.91E-01 | 1 | STAC | 6.55E-01 | 1 |
| SLC29A3 | 4.91E-01 | 1 | MTMR3 | 6.55E-01 | 1 |
| FOXE3 | 4.91E-01 | 1 | DCAF8L2 | 6.55E-01 | 1 |
| A4GNT | 4.91E-01 | 1 | PRKCQ | 6.55E-01 | 1 |
| ATXN7 | 4.91E-01 | 1 | TCF21 | 6.56E-01 | 1 |
| SETD2 | 4.91E-01 | 1 | XAB2 | 6.56E-01 | 1 |
| LGI1 | 4.91E-01 | 1 | NCKAP1L | 6.56E-01 | 1 |
| TNFRSF10 | 4.91E-01 | 1 | SLC2A2 | 6.56E-01 | 1 |
| MAP4 | 4.91E-01 | 1 | LARGE | 6.56E-01 | 1 |
| ZNF157 | 4.91E-01 | 1 | NAA16 | 6.57E-01 | 1 |
| OR9G4 | 4.92E-01 | 1 | PALM3 | 6.57E-01 | 1 |
| OR8G5 | 4.92E-01 | 1 | RPRD2 | 6.57E-01 | 1 |
| RYK | 4.92E-01 | 1 | POTEG | 6.57E-01 | 1 |
| PHC1 | 4.92E-01 | 1 | CLPX | 6.58E-01 | 1 |
| NKX2-5 | 4.92E-01 | 1 | TLK2 | 6.58E-01 | 1 |
| VCX3B | 4.92E-01 | 1 | MYEF2 | 6.58E-01 | 1 |
| GUCY2D | 4.93E-01 | 1 | PCDHGB2 | 6.58E-01 | 1 |
| PANK2 | 4.93E-01 | 1 | FAF1 | 6.58E-01 | 1 |
| TAF5 | 4.93E-01 | 1 | NRCAM | 6.59E-01 | 1 |
| GDAP1 | 4.93E-01 | 1 | KRT39 | 6.59E-01 | 1 |
| ESAM | 4.93E-01 | 1 | AARS | 6.59E-01 | 1 |
| PTPN5 | 4.93E-01 | 1 | ZNF468 | 6.59E-01 | 1 |
| PGM3 | 4.94E-01 | 1 | ARHGAP6 | 6.59E-01 | 1 |
| WDR78 | 4.94E-01 | 1 | AFF2 | 6.59E-01 | 1 |
| EXOC3 | 4.94E-01 | 1 | DPYSL3 | 6.59E-01 | 1 |
| ATP4A | 4.94E-01 | 1 | CLDN17 | 6.59E-01 | 1 |
| SLC26A9 | 4.94E-01 | 1 | DSCAML1 | 6.59E-01 | 1 |
| AGT | 4.94E-01 | 1 | MYPN | 6.59E-01 | 1 |
| ASGR1 | 4.94E-01 | 1 | MCPH1 | 6.60E-01 | 1 |
| DNAH12 | 4.94E-01 | 1 | WDR60 | 6.60E-01 | 1 |

| CCDC81 | 4.94E-01 | 1 | LRIG3 | 6.60E-01 | 1 |
| --- | --- | --- | --- | --- | --- |
| MAGIX | 4.94E-01 | 1 | IARS2 | 6.60E-01 | 1 |
| FA2H | 4.94E-01 | 1 | TBC1D12 | 6.60E-01 | 1 |
| SLC5A9 | 4.94E-01 | 1 | MDGA1 | 6.60E-01 | 1 |
| SLC22A25 | 4.95E-01 | 1 | ZFP30 | 6.60E-01 | 1 |
| GLB1L | 4.95E-01 | 1 | INPP5F | 6.61E-01 | 1 |
| CLRN1 | 4.95E-01 | 1 | MYH2 | 6.61E-01 | 1 |
| ATP2C1 | 4.95E-01 | 1 | ZSCAN10 | 6.61E-01 | 1 |
| MUT | 4.95E-01 | 1 | FSCB | 6.61E-01 | 1 |
| CHAF1B | 4.95E-01 | 1 | GTPBP1 | 6.61E-01 | 1 |
| SLC13A4 | 4.95E-01 | 1 | OS9 | 6.62E-01 | 1 |
| ZNF329 | 4.95E-01 | 1 | COL9A3 | 6.62E-01 | 1 |
| CLUL1 | 4.95E-01 | 1 | STON2 | 6.62E-01 | 1 |
| FAM124A | 4.95E-01 | 1 | AKAP1 | 6.63E-01 | 1 |
| EWSR1 | 4.96E-01 | 1 | SAMD9 | 6.63E-01 | 1 |
| RUNDC1 | 4.96E-01 | 1 | ZNF813 | 6.63E-01 | 1 |
| LIMS2 | 4.96E-01 | 1 | PARP2 | 6.63E-01 | 1 |
| NIPA1 | 4.96E-01 | 1 | CXorf23 | 6.63E-01 | 1 |
| CYTIP | 4.96E-01 | 1 | GRAMD4 | 6.63E-01 | 1 |
| LAD1 | 4.96E-01 | 1 | ACTRT1 | 6.64E-01 | 1 |
| COL6A1 | 4.97E-01 | 1 | ZNF667 | 6.64E-01 | 1 |
| LRRC2 | 4.97E-01 | 1 | IGDCC4 | 6.64E-01 | 1 |
| LIMK2 | 4.97E-01 | 1 | CDH18 | 6.64E-01 | 1 |
| FIBCD1 | 4.97E-01 | 1 | ALDH1L2 | 6.64E-01 | 1 |
| HDLBP | 4.97E-01 | 1 | CPEB4 | 6.65E-01 | 1 |
| ASPN | 4.97E-01 | 1 | TTC8 | 6.65E-01 | 1 |
| TMEM183 | 4.97E-01 | 1 | VWA5B1 | 6.65E-01 | 1 |
| DMTF1 | 4.97E-01 | 1 | USP54 | 6.65E-01 | 1 |
| PER1 | 4.97E-01 | 1 | ZFX | 6.66E-01 | 1 |
| EML3 | 4.97E-01 | 1 | FBXO15 | 6.66E-01 | 1 |
| CNGB3 | 4.97E-01 | 1 | AVL9 | 6.66E-01 | 1 |
| OR5K2 | 4.98E-01 | 1 | KRT6A | 6.66E-01 | 1 |
| HP1BP3 | 4.98E-01 | 1 | CHD5 | 6.66E-01 | 1 |
| CCDC6 | 4.98E-01 | 1 | SBF2 | 6.66E-01 | 1 |
| GLO1 | 4.98E-01 | 1 | LMOD3 | 6.67E-01 | 1 |
| TCP11 | 4.98E-01 | 1 | ELMO2 | 6.67E-01 | 1 |
| C11orf42 | 4.98E-01 | 1 | ZNF839 | 6.67E-01 | 1 |
| ARSG | 4.98E-01 | 1 | LRRK1 | 6.67E-01 | 1 |
| ZBTB7B | 4.98E-01 | 1 | NEK4 | 6.67E-01 | 1 |
| WHSC1L1 | 4.98E-01 | 1 | AFAP1L1 | 6.67E-01 | 1 |
| SAMD9 | 4.98E-01 | 1 | CTCF | 6.67E-01 | 1 |
| NKAPL | 4.98E-01 | 1 | AMBRA1 | 6.67E-01 | 1 |
| CCDC113 | 4.98E-01 | 1 | LONRF1 | 6.68E-01 | 1 |
| KCND1 | 4.98E-01 | 1 | MCM6 | 6.68E-01 | 1 |
| MCF2 | 4.98E-01 | 1 | KIF11 | 6.68E-01 | 1 |
| ZHX1 | 4.98E-01 | 1 | ZNF510 | 6.68E-01 | 1 |
| GALR2 | 4.99E-01 | 1 | MGEA5 | 6.68E-01 | 1 |
| TNFRSF8 | 4.99E-01 | 1 | PCDHA9 | 6.68E-01 | 1 |
| BIRC8 | 4.99E-01 | 1 | CYP2A13 | 6.68E-01 | 1 |
| SLC4A9 | 4.99E-01 | 1 | NCOA1 | 6.68E-01 | 1 |
| MAGEE1 | 4.99E-01 | 1 | ROS1 | 6.68E-01 | 1 |
| KPNA2 | 4.99E-01 | 1 | HIF3A | 6.69E-01 | 1 |
| LRRC17 | 4.99E-01 | 1 | FANCD2 | 6.69E-01 | 1 |

| RASSF4 | 4.99E-01 | 1 | C8B | 6.69E-01 | 1 |
| --- | --- | --- | --- | --- | --- |
| SMYD4 | 4.99E-01 | 1 | FRK | 6.69E-01 | 1 |
| DNAH5 | 4.99E-01 | 1 | CDH3 | 6.69E-01 | 1 |
| HSF1 | 4.99E-01 | 1 | ZYG11A | 6.69E-01 | 1 |
| GJC2 | 4.99E-01 | 1 | FRMD1 | 6.70E-01 | 1 |
| ISX | 5.00E-01 | 1 | PCDHGA4 | 6.70E-01 | 1 |
| HOXC6 | 5.00E-01 | 1 | RTL1 | 6.70E-01 | 1 |
| GLIS3 | 5.00E-01 | 1 | GPR155 | 6.70E-01 | 1 |
| DDX17 | 5.00E-01 | 1 | LRRC6 | 6.70E-01 | 1 |
| LRRC39 | 5.00E-01 | 1 | OR4C46 | 6.71E-01 | 1 |
| DIO2 | 5.00E-01 | 1 | FURIN | 6.71E-01 | 1 |
| NR0B1 | 5.00E-01 | 1 | COL5A2 | 6.71E-01 | 1 |
| SMARCA | 5.00E-01 | 1 | SRGAP1 | 6.71E-01 | 1 |
| ANKRD20 | 5.01E-01 | 1 | MYO9B | 6.71E-01 | 1 |
| SORD | 5.01E-01 | 1 | C1orf87 | 6.71E-01 | 1 |
| FAM76B | 5.01E-01 | 1 | HTRA3 | 6.71E-01 | 1 |
| PTPRE | 5.01E-01 | 1 | KCNK2 | 6.72E-01 | 1 |
| OR2A4 | 5.01E-01 | 1 | PROM1 | 6.72E-01 | 1 |
| PRDM7 | 5.01E-01 | 1 | MYBPC3 | 6.72E-01 | 1 |
| ADORA2A | 5.01E-01 | 1 | PCDH9 | 6.73E-01 | 1 |
| LYN | 5.01E-01 | 1 | KIAA1468 | 6.73E-01 | 1 |
| ZNF862 | 5.01E-01 | 1 | REV1 | 6.73E-01 | 1 |
| ZFPM1 | 5.01E-01 | 1 | PRDM4 | 6.73E-01 | 1 |
| ZNF358 | 5.01E-01 | 1 | EPB41L1 | 6.74E-01 | 1 |
| INSR | 5.01E-01 | 1 | CARD8 | 6.74E-01 | 1 |
| WISP1 | 5.02E-01 | 1 | ZNF653 | 6.74E-01 | 1 |
| FAM53A | 5.02E-01 | 1 | ATXN1L | 6.74E-01 | 1 |
| DBH | 5.02E-01 | 1 | FOXP2 | 6.74E-01 | 1 |
| CPNE3 | 5.02E-01 | 1 | PRPF40A | 6.74E-01 | 1 |
| EIF2B3 | 5.02E-01 | 1 | BMP2K | 6.75E-01 | 1 |
| ABCA6 | 5.02E-01 | 1 | PLCB3 | 6.75E-01 | 1 |
| ZNF417 | 5.03E-01 | 1 | ZNF790 | 6.75E-01 | 1 |
| CFHR4 | 5.03E-01 | 1 | PAH | 6.75E-01 | 1 |
| LRRC48 | 5.03E-01 | 1 | PROX1 | 6.75E-01 | 1 |
| NOL6 | 5.03E-01 | 1 | CFHR5 | 6.75E-01 | 1 |
| KCNK3 | 5.03E-01 | 1 | ITGB4 | 6.75E-01 | 1 |
| SPATA5 | 5.03E-01 | 1 | GALNTL6 | 6.75E-01 | 1 |
| ZNF391 | 5.03E-01 | 1 | NIN | 6.75E-01 | 1 |
| SUPT3H | 5.03E-01 | 1 | TYK2 | 6.75E-01 | 1 |
| MYOD1 | 5.03E-01 | 1 | SETX | 6.76E-01 | 1 |
| ISPD | 5.03E-01 | 1 | PPP2R1A | 6.76E-01 | 1 |
| ACTL7A | 5.03E-01 | 1 | CEP97 | 6.76E-01 | 1 |
| ZNF625 | 5.03E-01 | 1 | MPRIP | 6.77E-01 | 1 |
| LATS1 | 5.03E-01 | 1 | CASR | 6.77E-01 | 1 |
| FBXO9 | 5.03E-01 | 1 | FER1L6 | 6.77E-01 | 1 |
| UNC5CL | 5.04E-01 | 1 | PROM2 | 6.77E-01 | 1 |
| OLFM1 | 5.04E-01 | 1 | GPRC6A | 6.77E-01 | 1 |
| CFC1 | 5.04E-01 | 1 | ACSM2B | 6.77E-01 | 1 |
| ARFGAP1 | 5.04E-01 | 1 | GOLGA1 | 6.77E-01 | 1 |
| ZMIZ2 | 5.04E-01 | 1 | GAB4 | 6.78E-01 | 1 |
| ACTN2 | 5.04E-01 | 1 | KCNB2 | 6.78E-01 | 1 |
| MAP2K2 | 5.04E-01 | 1 | OLFML2A | 6.78E-01 | 1 |
| MYH15 | 5.04E-01 | 1 | ZNF267 | 6.78E-01 | 1 |

| COL5A3 | 5.04E-01 | 1 | ATRNL1 | 6.78E-01 | 1 |
| --- | --- | --- | --- | --- | --- |
| 2-Sep | 5.04E-01 | 1 | GTF2IRD2B | 6.78E-01 | 1 |
| PRKCH | 5.05E-01 | 1 | ESRRG | 6.79E-01 | 1 |
| SYT6 | 5.05E-01 | 1 | NADSYN1 | 6.79E-01 | 1 |
| SUSD1 | 5.05E-01 | 1 | MGAT5 | 6.79E-01 | 1 |
| CABP1 | 5.05E-01 | 1 | UGT2A1 | 6.79E-01 | 1 |
| ZBTB49 | 5.05E-01 | 1 | RALGAPB | 6.79E-01 | 1 |
| LPAR1 | 5.06E-01 | 1 | ERCC6L | 6.80E-01 | 1 |
| POP4 | 5.06E-01 | 1 | ANKS6 | 6.80E-01 | 1 |
| SERPINA1 | 5.06E-01 | 1 | UGGT1 | 6.80E-01 | 1 |
| ZC3H6 | 5.06E-01 | 1 | ZNF780B | 6.80E-01 | 1 |
| CCDC146 | 5.06E-01 | 1 | CASC5 | 6.80E-01 | 1 |
| CD300E | 5.06E-01 | 1 | JPH1 | 6.80E-01 | 1 |
| GRIP2 | 5.06E-01 | 1 | ABCD2 | 6.80E-01 | 1 |
| OR5M10 | 5.06E-01 | 1 | SLC12A3 | 6.81E-01 | 1 |
| VWC2 | 5.06E-01 | 1 | NOVA1 | 6.81E-01 | 1 |
| FOXO4 | 5.06E-01 | 1 | SPATA20 | 6.81E-01 | 1 |
| OR10A3 | 5.06E-01 | 1 | MMP16 | 6.81E-01 | 1 |
| SNX16 | 5.06E-01 | 1 | RAB3GAP2 | 6.82E-01 | 1 |
| PPP1R3A | 5.07E-01 | 1 | AGL | 6.82E-01 | 1 |
| OR4M2 | 5.07E-01 | 1 | FOXK1 | 6.82E-01 | 1 |
| CD2AP | 5.07E-01 | 1 | RRM1 | 6.82E-01 | 1 |
| EMILIN3 | 5.07E-01 | 1 | STK39 | 6.82E-01 | 1 |
| CTNNA1 | 5.08E-01 | 1 | CUL4B | 6.83E-01 | 1 |
| RBM17 | 5.08E-01 | 1 | POTEM | 6.83E-01 | 1 |
| FARSB | 5.08E-01 | 1 | KL | 6.83E-01 | 1 |
| CAND2 | 5.08E-01 | 1 | CARD14 | 6.83E-01 | 1 |
| FUT10 | 5.08E-01 | 1 | NOL6 | 6.83E-01 | 1 |
| CDAN1 | 5.08E-01 | 1 | OR4C11 | 6.84E-01 | 1 |
| OR1Q1 | 5.09E-01 | 1 | GRIA4 | 6.84E-01 | 1 |
| NIPAL4 | 5.09E-01 | 1 | SLC41A3 | 6.84E-01 | 1 |
| EHMT2 | 5.09E-01 | 1 | LRRC16A | 6.84E-01 | 1 |
| CTR9 | 5.09E-01 | 1 | OBSL1 | 6.84E-01 | 1 |
| DAZAP2 | 5.09E-01 | 1 | C7 | 6.85E-01 | 1 |
| SLC19A1 | 5.09E-01 | 1 | TRIM32 | 6.85E-01 | 1 |
| SPATA21 | 5.09E-01 | 1 | IMMT | 6.85E-01 | 1 |
| SLC41A1 | 5.09E-01 | 1 | ASAH2 | 6.85E-01 | 1 |
| ASB6 | 5.09E-01 | 1 | GEMIN4 | 6.85E-01 | 1 |
| HS3ST4 | 5.09E-01 | 1 | OR4C6 | 6.85E-01 | 1 |
| METTL1 | 5.10E-01 | 1 | PTPRB | 6.85E-01 | 1 |
| ITGAM | 5.10E-01 | 1 | ZNF560 | 6.86E-01 | 1 |
| SATL1 | 5.10E-01 | 1 | LRRC37A2 | 6.86E-01 | 1 |
| GALNT11 | 5.10E-01 | 1 | PCDHA5 | 6.86E-01 | 1 |
| CCNL2 | 5.10E-01 | 1 | EML2 | 6.86E-01 | 1 |
| CSAD | 5.10E-01 | 1 | ECE2 | 6.87E-01 | 1 |
| E2F7 | 5.10E-01 | 1 | EXOC4 | 6.87E-01 | 1 |
| VANGL2 | 5.10E-01 | 1 | DAGLB | 6.87E-01 | 1 |
| FHOD1 | 5.10E-01 | 1 | PCDH10 | 6.87E-01 | 1 |
| CYP46A1 | 5.10E-01 | 1 | RIBC1 | 6.87E-01 | 1 |
| EIF4A3 | 5.10E-01 | 1 | TUBGCP2 | 6.88E-01 | 1 |
| OR2T12 | 5.10E-01 | 1 | MYLK3 | 6.88E-01 | 1 |
| SNX20 | 5.10E-01 | 1 | CDH4 | 6.88E-01 | 1 |
| TMPRSS1 | 5.10E-01 | 1 | APAF1 | 6.89E-01 | 1 |

| AKAP5 | 5.11E-01 | 1 | ANKRD32 | 6.89E-01 | 1 |
| --- | --- | --- | --- | --- | --- |
| CEP290 | 5.11E-01 | 1 | UBAP2L | 6.89E-01 | 1 |
| MEPE | 5.11E-01 | 1 | DNER | 6.89E-01 | 1 |
| MBOAT4 | 5.11E-01 | 1 | TMEM200C | 6.89E-01 | 1 |
| PRB2 | 5.11E-01 | 1 | KIF27 | 6.89E-01 | 1 |
| HNRNPF | 5.11E-01 | 1 | FXR1 | 6.89E-01 | 1 |
| CXCR6 | 5.11E-01 | 1 | FES | 6.89E-01 | 1 |
| TYW1 | 5.11E-01 | 1 | FBXL5 | 6.90E-01 | 1 |
| SELPLG | 5.11E-01 | 1 | ADNP2 | 6.90E-01 | 1 |
| FGF14 | 5.11E-01 | 1 | GPC5 | 6.90E-01 | 1 |
| STAB2 | 5.11E-01 | 1 | SCIN | 6.91E-01 | 1 |
| C16orf46 | 5.11E-01 | 1 | KIF21B | 6.91E-01 | 1 |
| MUC7 | 5.11E-01 | 1 | OR2T2 | 6.91E-01 | 1 |
| PBK | 5.12E-01 | 1 | SIGLEC12 | 6.91E-01 | 1 |
| ZNF536 | 5.12E-01 | 1 | PRSS36 | 6.91E-01 | 1 |
| GPR61 | 5.12E-01 | 1 | PDE10A | 6.91E-01 | 1 |
| VTN | 5.12E-01 | 1 | SLC35B4 | 6.92E-01 | 1 |
| ITGB2 | 5.12E-01 | 1 | FRMD4B | 6.92E-01 | 1 |
| IFNAR2 | 5.12E-01 | 1 | INSRR | 6.92E-01 | 1 |
| ELFN2 | 5.13E-01 | 1 | MYH8 | 6.92E-01 | 1 |
| ANKMY2 | 5.13E-01 | 1 | KCNQ5 | 6.92E-01 | 1 |
| OR2A25 | 5.13E-01 | 1 | ITGA6 | 6.92E-01 | 1 |
| RNF146 | 5.13E-01 | 1 | DPPA4 | 6.92E-01 | 1 |
| SCPEP1 | 5.13E-01 | 1 | MYO15A | 6.93E-01 | 1 |
| PRLHR | 5.13E-01 | 1 | KIF16B | 6.93E-01 | 1 |
| BCAT1 | 5.13E-01 | 1 | CR1 | 6.93E-01 | 1 |
| GTPBP10 | 5.13E-01 | 1 | HECTD1 | 6.93E-01 | 1 |
| RASSF8 | 5.13E-01 | 1 | SCN3A | 6.93E-01 | 1 |
| POU4F1 | 5.13E-01 | 1 | DPY19L3 | 6.94E-01 | 1 |
| TMC3 | 5.14E-01 | 1 | ANXA6 | 6.94E-01 | 1 |
| CCDC125 | 5.14E-01 | 1 | FAM21C | 6.94E-01 | 1 |
| MGAT1 | 5.14E-01 | 1 | PARP12 | 6.94E-01 | 1 |
| OSBPL3 | 5.14E-01 | 1 | PCNXL2 | 6.94E-01 | 1 |
| PNO1 | 5.14E-01 | 1 | SRBD1 | 6.94E-01 | 1 |
| HRH1 | 5.14E-01 | 1 | PC | 6.94E-01 | 1 |
| LAMC2 | 5.14E-01 | 1 | SATB1 | 6.95E-01 | 1 |
| TSPYL1 | 5.15E-01 | 1 | RAP1GAP2 | 6.95E-01 | 1 |
| GSDMD | 5.15E-01 | 1 | SFI1 | 6.96E-01 | 1 |
| GGT1 | 5.15E-01 | 1 | NRP2 | 6.96E-01 | 1 |
| CATSPER | 5.15E-01 | 1 | VAV3 | 6.96E-01 | 1 |
| KDM4B | 5.15E-01 | 1 | WDR64 | 6.96E-01 | 1 |
| ZNF71 | 5.15E-01 | 1 | CLCN1 | 6.96E-01 | 1 |
| RXFP1 | 5.15E-01 | 1 | ATAD3B | 6.97E-01 | 1 |
| GAGE2E | 5.15E-01 | 1 | KRT76 | 6.98E-01 | 1 |
| RNF121 | 5.15E-01 | 1 | UBQLN3 | 6.98E-01 | 1 |
| GPR85 | 5.15E-01 | 1 | CCDC136 | 6.98E-01 | 1 |
| FBLN7 | 5.15E-01 | 1 | ZBTB38 | 6.98E-01 | 1 |
| ACAP1 | 5.15E-01 | 1 | SULF1 | 6.99E-01 | 1 |
| CYP4Z1 | 5.15E-01 | 1 | TGFB2 | 6.99E-01 | 1 |
| OR4S2 | 5.15E-01 | 1 | PLCL1 | 6.99E-01 | 1 |
| DENND5A | 5.15E-01 | 1 | PRPF4B | 6.99E-01 | 1 |
| PFAS | 5.15E-01 | 1 | MPPED2 | 6.99E-01 | 1 |
| ATP6V1B | 5.15E-01 | 1 | FAM161A | 6.99E-01 | 1 |

| OR51I2 | 5.16E-01 | 1 | JPH3 | 6.99E-01 | 1 |
| --- | --- | --- | --- | --- | --- |
| LCMT1 | 5.16E-01 | 1 | CD36 | 7.00E-01 | 1 |
| PLA2G4E | 5.16E-01 | 1 | FAM13B | 7.00E-01 | 1 |
| PAPD4 | 5.16E-01 | 1 | LRP8 | 7.00E-01 | 1 |
| SMAD6 | 5.16E-01 | 1 | SAA2 | 7.01E-01 | 1 |
| TKTL1 | 5.16E-01 | 1 | PDHA2 | 7.01E-01 | 1 |
| CDK18 | 5.16E-01 | 1 | UBE4A | 7.01E-01 | 1 |
| ZNF471 | 5.16E-01 | 1 | PIK3R4 | 7.01E-01 | 1 |
| FAM171A | 5.16E-01 | 1 | GABRQ | 7.01E-01 | 1 |
| SMAP2 | 5.16E-01 | 1 | OBSCN | 7.01E-01 | 1 |
| CASZ1 | 5.16E-01 | 1 | ZNF20 | 7.01E-01 | 1 |
| DNAJC6 | 5.16E-01 | 1 | NBPF15 | 7.02E-01 | 1 |
| UEVLD | 5.16E-01 | 1 | ITIH3 | 7.02E-01 | 1 |
| DNM1 | 5.16E-01 | 1 | SLC12A2 | 7.02E-01 | 1 |
| C16orf71 | 5.16E-01 | 1 | SRRT | 7.02E-01 | 1 |
| PSD4 | 5.16E-01 | 1 | NRP1 | 7.02E-01 | 1 |
| MYO9A | 5.17E-01 | 1 | TRPM1 | 7.02E-01 | 1 |
| KRT84 | 5.17E-01 | 1 | ZNF224 | 7.02E-01 | 1 |
| IPCEF1 | 5.17E-01 | 1 | SLITRK6 | 7.02E-01 | 1 |
| PAK4 | 5.17E-01 | 1 | GAS2L3 | 7.02E-01 | 1 |
| LSG1 | 5.17E-01 | 1 | HACE1 | 7.02E-01 | 1 |
| CCDC91 | 5.17E-01 | 1 | LMBRD2 | 7.03E-01 | 1 |
| ASB4 | 5.17E-01 | 1 | ITGA2 | 7.03E-01 | 1 |
| SUPT6H | 5.17E-01 | 1 | ARHGAP22 | 7.03E-01 | 1 |
| RIMKLA | 5.17E-01 | 1 | ACVR2A | 7.03E-01 | 1 |
| TNIP3 | 5.17E-01 | 1 | ZNF518A | 7.04E-01 | 1 |
| HTRA1 | 5.18E-01 | 1 | NUP88 | 7.04E-01 | 1 |
| CBWD5 | 5.18E-01 | 1 | PHF12 | 7.04E-01 | 1 |
| ZBTB11 | 5.18E-01 | 1 | PCDHGB5 | 7.04E-01 | 1 |
| SNTG2 | 5.18E-01 | 1 | ALPK1 | 7.05E-01 | 1 |
| OR1L8 | 5.18E-01 | 1 | PHF2 | 7.05E-01 | 1 |
| ANKRD34 | 5.18E-01 | 1 | GRB10 | 7.05E-01 | 1 |
| TARS2 | 5.18E-01 | 1 | TTC14 | 7.05E-01 | 1 |
| CCNC | 5.18E-01 | 1 | SEL1L2 | 7.05E-01 | 1 |
| POLD1 | 5.19E-01 | 1 | CHD2 | 7.05E-01 | 1 |
| RFX6 | 5.19E-01 | 1 | CLCN2 | 7.05E-01 | 1 |
| ATAD5 | 5.19E-01 | 1 | ST18 | 7.06E-01 | 1 |
| IGFBP5 | 5.19E-01 | 1 | PRDM10 | 7.06E-01 | 1 |
| ADAM19 | 5.19E-01 | 1 | FMNL1 | 7.06E-01 | 1 |
| WDR41 | 5.19E-01 | 1 | PACS2 | 7.06E-01 | 1 |
| GPC2 | 5.19E-01 | 1 | AASDH | 7.07E-01 | 1 |
| BRCA1 | 5.19E-01 | 1 | KHDRBS2 | 7.07E-01 | 1 |
| NT5DC1 | 5.19E-01 | 1 | UGT2B10 | 7.07E-01 | 1 |
| RPTN | 5.19E-01 | 1 | ZNF281 | 7.07E-01 | 1 |
| FAF2 | 5.19E-01 | 1 | MCF2L2 | 7.07E-01 | 1 |
| RABL2A | 5.19E-01 | 1 | UNC5C | 7.07E-01 | 1 |
| FAM193A | 5.19E-01 | 1 | BCL11A | 7.08E-01 | 1 |
| RHOBTB1 | 5.19E-01 | 1 | DOCK11 | 7.08E-01 | 1 |
| ITGA3 | 5.19E-01 | 1 | HEG1 | 7.08E-01 | 1 |
| PSMC5 | 5.19E-01 | 1 | POLQ | 7.08E-01 | 1 |
| TBC1D10 | 5.20E-01 | 1 | DCHS2 | 7.09E-01 | 1 |
| SPON1 | 5.20E-01 | 1 | WDR47 | 7.09E-01 | 1 |
| KHDC1L | 5.20E-01 | 1 | PPFIBP1 | 7.09E-01 | 1 |

| CDKL4 | 5.20E-01 | 1 | ZNF571 | 7.09E-01 | 1 |
| --- | --- | --- | --- | --- | --- |
| URGCP | 5.20E-01 | 1 | KIF26B | 7.09E-01 | 1 |
| SLCO1B3 | 5.20E-01 | 1 | EHBP1L1 | 7.10E-01 | 1 |
| PWWP2B | 5.20E-01 | 1 | OR7D4 | 7.10E-01 | 1 |
| SDK2 | 5.20E-01 | 1 | IRS4 | 7.10E-01 | 1 |
| DDX6 | 5.20E-01 | 1 | ZNF814 | 7.10E-01 | 1 |
| KCNG3 | 5.20E-01 | 1 | HSPA4 | 7.11E-01 | 1 |
| KIAA0391 | 5.21E-01 | 1 | TMTC3 | 7.11E-01 | 1 |
| TMEM232 | 5.21E-01 | 1 | THSD7A | 7.11E-01 | 1 |
| MMP16 | 5.21E-01 | 1 | SRRM5 | 7.11E-01 | 1 |
| TTC33 | 5.21E-01 | 1 | SLC4A7 | 7.11E-01 | 1 |
| CYP4A11 | 5.21E-01 | 1 | ELFN2 | 7.11E-01 | 1 |
| KBTBD8 | 5.21E-01 | 1 | HIPK1 | 7.11E-01 | 1 |
| PNMAL2 | 5.21E-01 | 1 | YEATS2 | 7.11E-01 | 1 |
| NUDCD3 | 5.21E-01 | 1 | FAM13C | 7.12E-01 | 1 |
| MYH9 | 5.21E-01 | 1 | PTPN5 | 7.12E-01 | 1 |
| PALM2-A | 5.21E-01 | 1 | BHLHB9 | 7.12E-01 | 1 |
| TRIM67 | 5.21E-01 | 1 | DIDO1 | 7.13E-01 | 1 |
| ZNF696 | 5.21E-01 | 1 | OFD1 | 7.14E-01 | 1 |
| ZDHHC16 | 5.22E-01 | 1 | ANKS1B | 7.14E-01 | 1 |
| SLC45A4 | 5.22E-01 | 1 | MEGF10 | 7.14E-01 | 1 |
| ATOH8 | 5.22E-01 | 1 | AMPD2 | 7.14E-01 | 1 |
| FAM189A | 5.22E-01 | 1 | LTA4H | 7.14E-01 | 1 |
| VPS54 | 5.22E-01 | 1 | OR2AG2 | 7.14E-01 | 1 |
| HSPD1 | 5.22E-01 | 1 | BCAN | 7.15E-01 | 1 |
| ZCCHC18 | 5.22E-01 | 1 | DISP2 | 7.15E-01 | 1 |
| GAS2L2 | 5.22E-01 | 1 | ZNF415 | 7.15E-01 | 1 |
| ZNF768 | 5.22E-01 | 1 | COL28A1 | 7.15E-01 | 1 |
| POLD2 | 5.22E-01 | 1 | FOXM1 | 7.15E-01 | 1 |
| CADM3 | 5.22E-01 | 1 | ADAM9 | 7.15E-01 | 1 |
| QRFPR | 5.22E-01 | 1 | ARHGEF18 | 7.16E-01 | 1 |
| RASAL1 | 5.22E-01 | 1 | HIST1H3B | 7.16E-01 | 1 |
| TBX10 | 5.22E-01 | 1 | GSG2 | 7.16E-01 | 1 |
| OSGIN2 | 5.22E-01 | 1 | FLT3 | 7.16E-01 | 1 |
| DDX49 | 5.22E-01 | 1 | SLC8A3 | 7.16E-01 | 1 |
| TIGD7 | 5.23E-01 | 1 | PCSK2 | 7.17E-01 | 1 |
| ACBD3 | 5.23E-01 | 1 | GLB1L2 | 7.17E-01 | 1 |
| PRDM13 | 5.23E-01 | 1 | SSC5D | 7.17E-01 | 1 |
| SPOCD1 | 5.23E-01 | 1 | QSOX1 | 7.18E-01 | 1 |
| CCDC130 | 5.23E-01 | 1 | PCDHAC1 | 7.18E-01 | 1 |
| SCNN1G | 5.24E-01 | 1 | LPA | 7.18E-01 | 1 |
| ARFIP1 | 5.24E-01 | 1 | PCDHGA3 | 7.19E-01 | 1 |
| FOXD4 | 5.24E-01 | 1 | RNGTT | 7.19E-01 | 1 |
| PUS1 | 5.24E-01 | 1 | ARHGEF5 | 7.19E-01 | 1 |
| GSG1L | 5.24E-01 | 1 | FMN1 | 7.19E-01 | 1 |
| HTRA4 | 5.24E-01 | 1 | LUZP2 | 7.19E-01 | 1 |
| USP1 | 5.24E-01 | 1 | COL6A5 | 7.19E-01 | 1 |
| PSMB5 | 5.25E-01 | 1 | PLAA | 7.20E-01 | 1 |
| GGA1 | 5.25E-01 | 1 | SORL1 | 7.20E-01 | 1 |
| GABPB1 | 5.25E-01 | 1 | KCNH2 | 7.20E-01 | 1 |
| TMEM132 | 5.25E-01 | 1 | THSD7B | 7.20E-01 | 1 |
| ECE2 | 5.25E-01 | 1 | JMY | 7.21E-01 | 1 |
| FRG2 | 5.25E-01 | 1 | SPOCD1 | 7.21E-01 | 1 |

| TMEM132 | 5.25E-01 | 1 | CHADL | 7.21E-01 | 1 |
| --- | --- | --- | --- | --- | --- |
| BBS7 | 5.25E-01 | 1 | TMC7 | 7.21E-01 | 1 |
| TRIP11 | 5.25E-01 | 1 | RABGAP1L | 7.21E-01 | 1 |
| PHF1 | 5.25E-01 | 1 | PTPRO | 7.21E-01 | 1 |
| DLGAP3 | 5.25E-01 | 1 | ATXN7 | 7.21E-01 | 1 |
| ZNF543 | 5.25E-01 | 1 | SLC4A11 | 7.22E-01 | 1 |
| HS3ST5 | 5.26E-01 | 1 | NRXN2 | 7.22E-01 | 1 |
| GMIP | 5.26E-01 | 1 | C5orf42 | 7.22E-01 | 1 |
| PARP6 | 5.26E-01 | 1 | TBCD | 7.23E-01 | 1 |
| POU5F2 | 5.26E-01 | 1 | CCDC88C | 7.23E-01 | 1 |
| NODAL | 5.26E-01 | 1 | RLTPR | 7.23E-01 | 1 |
| A2M | 5.26E-01 | 1 | HDLBP | 7.23E-01 | 1 |
| MRPS27 | 5.26E-01 | 1 | NCAM2 | 7.23E-01 | 1 |
| TANC2 | 5.26E-01 | 1 | CSMD2 | 7.24E-01 | 1 |
| EVI2B | 5.26E-01 | 1 | SLCO2B1 | 7.24E-01 | 1 |
| KIAA0430 | 5.27E-01 | 1 | DSC3 | 7.24E-01 | 1 |
| MYC | 5.27E-01 | 1 | SLC39A4 | 7.24E-01 | 1 |
| XPO5 | 5.27E-01 | 1 | AMOT | 7.24E-01 | 1 |
| MBTD1 | 5.27E-01 | 1 | PLEKHG4B | 7.25E-01 | 1 |
| RGS2 | 5.27E-01 | 1 | FBN2 | 7.25E-01 | 1 |
| USP27X | 5.27E-01 | 1 | WDR27 | 7.25E-01 | 1 |
| ZNF398 | 5.27E-01 | 1 | GRIK1 | 7.25E-01 | 1 |
| ZFAT | 5.28E-01 | 1 | ACAP1 | 7.25E-01 | 1 |
| PHF8 | 5.28E-01 | 1 | RAI1 | 7.25E-01 | 1 |
| RABGEF1 | 5.28E-01 | 1 | XPOT | 7.25E-01 | 1 |
| BRD4 | 5.28E-01 | 1 | PTPN23 | 7.25E-01 | 1 |
| PURG | 5.28E-01 | 1 | RAPGEF1 | 7.25E-01 | 1 |
| PPIP5K2 | 5.28E-01 | 1 | NCAPD3 | 7.26E-01 | 1 |
| B3GNT5 | 5.28E-01 | 1 | LHCGR | 7.26E-01 | 1 |
| CA4 | 5.28E-01 | 1 | CD101 | 7.26E-01 | 1 |
| POTEC | 5.28E-01 | 1 | SEMA6C | 7.26E-01 | 1 |
| KRT39 | 5.28E-01 | 1 | CTNND2 | 7.26E-01 | 1 |
| THUMPD | 5.28E-01 | 1 | ACSBG2 | 7.26E-01 | 1 |
| CPNE8 | 5.28E-01 | 1 | LILRB1 | 7.26E-01 | 1 |
| SCN2B | 5.28E-01 | 1 | CTDP1 | 7.26E-01 | 1 |
| ZNF606 | 5.28E-01 | 1 | TRAPPC10 | 7.28E-01 | 1 |
| RRP15 | 5.29E-01 | 1 | CNTN5 | 7.28E-01 | 1 |
| UNC5D | 5.29E-01 | 1 | DENND1B | 7.28E-01 | 1 |
| OR56A5 | 5.29E-01 | 1 | CASS4 | 7.29E-01 | 1 |
| SPPL3 | 5.29E-01 | 1 | FIGN | 7.29E-01 | 1 |
| SP9 | 5.29E-01 | 1 | SSH1 | 7.29E-01 | 1 |
| OR52E4 | 5.29E-01 | 1 | MXRA5 | 7.29E-01 | 1 |
| TNMD | 5.29E-01 | 1 | ADAMTSL | 7.29E-01 | 1 |
| LUZP4 | 5.29E-01 | 1 | SND1 | 7.29E-01 | 1 |
| PAK3 | 5.29E-01 | 1 | NNT | 7.29E-01 | 1 |
| PTCD3 | 5.29E-01 | 1 | PLA2G4A | 7.29E-01 | 1 |
| DPP4 | 5.30E-01 | 1 | DISC1 | 7.29E-01 | 1 |
| ST3GAL6 | 5.30E-01 | 1 | DGKD | 7.30E-01 | 1 |
| EPN2 | 5.30E-01 | 1 | SEMA6D | 7.30E-01 | 1 |
| NAAA | 5.30E-01 | 1 | CD163 | 7.30E-01 | 1 |
| ELF4 | 5.30E-01 | 1 | MCTP1 | 7.30E-01 | 1 |
| ANKRD10 | 5.30E-01 | 1 | CLTC | 7.30E-01 | 1 |
| FAM184B | 5.31E-01 | 1 | HHIPL2 | 7.30E-01 | 1 |

| PITX2 | 5.31E-01 | 1 | IQGAP1 | 7.31E-01 | 1 |
| --- | --- | --- | --- | --- | --- |
| L1TD1 | 5.31E-01 | 1 | TMEM214 | 7.31E-01 | 1 |
| MAGEA11 | 5.31E-01 | 1 | POTEE | 7.31E-01 | 1 |
| GPR83 | 5.31E-01 | 1 | THBS1 | 7.31E-01 | 1 |
| PHYHIP | 5.31E-01 | 1 | CHAT | 7.31E-01 | 1 |
| TIGD2 | 5.31E-01 | 1 | JAK1 | 7.31E-01 | 1 |
| SLC12A7 | 5.31E-01 | 1 | MAGEL2 | 7.32E-01 | 1 |
| PTPN23 | 5.32E-01 | 1 | LONP2 | 7.32E-01 | 1 |
| OR6K2 | 5.32E-01 | 1 | STOX2 | 7.32E-01 | 1 |
| PLD1 | 5.32E-01 | 1 | BRCA1 | 7.32E-01 | 1 |
| NPTX1 | 5.32E-01 | 1 | CENPE | 7.33E-01 | 1 |
| OR4F17 | 5.32E-01 | 1 | USP42 | 7.33E-01 | 1 |
| MAGI2 | 5.32E-01 | 1 | APP | 7.33E-01 | 1 |
| HDAC8 | 5.32E-01 | 1 | PCDHGB4 | 7.33E-01 | 1 |
| TDRD10 | 5.32E-01 | 1 | PCDH12 | 7.33E-01 | 1 |
| ZNF468 | 5.32E-01 | 1 | ZNF624 | 7.33E-01 | 1 |
| SLC25A31 | 5.32E-01 | 1 | LMTK3 | 7.34E-01 | 1 |
| POGK | 5.33E-01 | 1 | GNE | 7.34E-01 | 1 |
| CD200R1 | 5.33E-01 | 1 | TTLL7 | 7.34E-01 | 1 |
| HTR2B | 5.33E-01 | 1 | ABCB1 | 7.34E-01 | 1 |
| CNOT10 | 5.33E-01 | 1 | SEMA6B | 7.34E-01 | 1 |
| HEPH | 5.33E-01 | 1 | PCDHGB1 | 7.34E-01 | 1 |
| STXBP3 | 5.33E-01 | 1 | STAT6 | 7.35E-01 | 1 |
| EPM2AIP1 | 5.33E-01 | 1 | THAP9 | 7.35E-01 | 1 |
| CHMP7 | 5.33E-01 | 1 | CST11 | 7.35E-01 | 1 |
| ELK4 | 5.33E-01 | 1 | LRIG1 | 7.35E-01 | 1 |
| GMEB1 | 5.33E-01 | 1 | ZNF229 | 7.35E-01 | 1 |
| GGPS1 | 5.34E-01 | 1 | DPP6 | 7.35E-01 | 1 |
| TLL1 | 5.34E-01 | 1 | SOX5 | 7.35E-01 | 1 |
| FGD6 | 5.34E-01 | 1 | ATP13A2 | 7.35E-01 | 1 |
| ZBTB39 | 5.34E-01 | 1 | TARBP1 | 7.35E-01 | 1 |
| PLIN1 | 5.34E-01 | 1 | NCAPG2 | 7.36E-01 | 1 |
| PLEKHA7 | 5.34E-01 | 1 | MIB1 | 7.36E-01 | 1 |
| LRRC55 | 5.34E-01 | 1 | AHDC1 | 7.36E-01 | 1 |
| BPNT1 | 5.34E-01 | 1 | ERBB4 | 7.37E-01 | 1 |
| OR2T10 | 5.34E-01 | 1 | CLEC16A | 7.37E-01 | 1 |
| COL4A4 | 5.34E-01 | 1 | REST | 7.37E-01 | 1 |
| EPB41L5 | 5.34E-01 | 1 | RNASE1 | 7.37E-01 | 1 |
| KRT19 | 5.34E-01 | 1 | MON2 | 7.37E-01 | 1 |
| SCUBE2 | 5.34E-01 | 1 | KDM4C | 7.37E-01 | 1 |
| TYR | 5.34E-01 | 1 | TECTA | 7.37E-01 | 1 |
| OR5T1 | 5.34E-01 | 1 | IREB2 | 7.38E-01 | 1 |
| OR6B1 | 5.34E-01 | 1 | ANGPT1 | 7.38E-01 | 1 |
| ITFG2 | 5.35E-01 | 1 | NFAT5 | 7.38E-01 | 1 |
| NSF | 5.35E-01 | 1 | CREBBP | 7.38E-01 | 1 |
| ADRA1B | 5.35E-01 | 1 | MED12L | 7.38E-01 | 1 |
| PCMTD1 | 5.35E-01 | 1 | PCSK5 | 7.39E-01 | 1 |
| PCDHGA1 | 5.35E-01 | 1 | ELFN1 | 7.39E-01 | 1 |
| OR6K3 | 5.35E-01 | 1 | TGM4 | 7.39E-01 | 1 |
| DERA | 5.35E-01 | 1 | PPFIA2 | 7.40E-01 | 1 |
| TCN2 | 5.35E-01 | 1 | UNC45A | 7.40E-01 | 1 |
| POU5F1B | 5.35E-01 | 1 | FBXL18 | 7.40E-01 | 1 |
| PCDHA11 | 5.35E-01 | 1 | OXSM | 7.40E-01 | 1 |

| ASPSCR1 | 5.36E-01 | 1 | PLEKHH1 | 7.40E-01 | 1 |
| --- | --- | --- | --- | --- | --- |
| KLF8 | 5.36E-01 | 1 | CSF2RB | 7.40E-01 | 1 |
| TSEN34 | 5.36E-01 | 1 | AASS | 7.40E-01 | 1 |
| EGR3 | 5.36E-01 | 1 | PLCH2 | 7.41E-01 | 1 |
| EID3 | 5.36E-01 | 1 | ENPEP | 7.41E-01 | 1 |
| ITGA2B | 5.36E-01 | 1 | ATP4A | 7.41E-01 | 1 |
| CTTN | 5.36E-01 | 1 | APOH | 7.41E-01 | 1 |
| UBE2Q1 | 5.36E-01 | 1 | GPATCH2 | 7.41E-01 | 1 |
| RIN3 | 5.36E-01 | 1 | SLIT3 | 7.42E-01 | 1 |
| MACC1 | 5.36E-01 | 1 | FAM129A | 7.42E-01 | 1 |
| CCDC14 | 5.36E-01 | 1 | RNF180 | 7.42E-01 | 1 |
| KIF1C | 5.36E-01 | 1 | HYOU1 | 7.42E-01 | 1 |
| ERBB2IP | 5.36E-01 | 1 | FMN2 | 7.42E-01 | 1 |
| NCAPH | 5.36E-01 | 1 | ZNF540 | 7.42E-01 | 1 |
| TRIM37 | 5.36E-01 | 1 | PEG3 | 7.42E-01 | 1 |
| LUM | 5.36E-01 | 1 | TBC1D16 | 7.43E-01 | 1 |
| SLC39A4 | 5.37E-01 | 1 | KIFAP3 | 7.43E-01 | 1 |
| KLHL9 | 5.37E-01 | 1 | ZNF528 | 7.43E-01 | 1 |
| HIF1A | 5.37E-01 | 1 | STIM2 | 7.43E-01 | 1 |
| CRISPLD2 | 5.37E-01 | 1 | PARP8 | 7.43E-01 | 1 |
| UBA5 | 5.37E-01 | 1 | UBN1 | 7.43E-01 | 1 |
| PTPN22 | 5.37E-01 | 1 | RYR2 | 7.43E-01 | 1 |
| PLEKHM2 | 5.37E-01 | 1 | DUOX1 | 7.44E-01 | 1 |
| SLK | 5.37E-01 | 1 | SEC24A | 7.44E-01 | 1 |
| WTAP | 5.37E-01 | 1 | EIF2AK3 | 7.44E-01 | 1 |
| OR2T34 | 5.37E-01 | 1 | PIK3R1 | 7.44E-01 | 1 |
| SRL | 5.38E-01 | 1 | TMTC1 | 7.45E-01 | 1 |
| COX11 | 5.38E-01 | 1 | COL11A2 | 7.45E-01 | 1 |
| IQUB | 5.38E-01 | 1 | ATP2B1 | 7.45E-01 | 1 |
| KCNU1 | 5.38E-01 | 1 | SLMAP | 7.45E-01 | 1 |
| DYSF | 5.38E-01 | 1 | RBM46 | 7.45E-01 | 1 |
| TBX19 | 5.38E-01 | 1 | DNHD1 | 7.45E-01 | 1 |
| TMX3 | 5.38E-01 | 1 | PTPRM | 7.46E-01 | 1 |
| RHPN2 | 5.38E-01 | 1 | TMF1 | 7.46E-01 | 1 |
| ME1 | 5.38E-01 | 1 | ZP4 | 7.46E-01 | 1 |
| CYP11A1 | 5.39E-01 | 1 | TDRD6 | 7.46E-01 | 1 |
| KIF21A | 5.39E-01 | 1 | BCOR | 7.46E-01 | 1 |
| CXorf57 | 5.39E-01 | 1 | ATP10B | 7.46E-01 | 1 |
| PLEK | 5.39E-01 | 1 | CBLB | 7.46E-01 | 1 |
| TRPM2 | 5.39E-01 | 1 | ZNF823 | 7.47E-01 | 1 |
| AP1M1 | 5.39E-01 | 1 | PSD4 | 7.47E-01 | 1 |
| SNX17 | 5.39E-01 | 1 | SLITRK1 | 7.47E-01 | 1 |
| PLIN3 | 5.39E-01 | 1 | SLC17A6 | 7.48E-01 | 1 |
| CHRNA2 | 5.39E-01 | 1 | KAL1 | 7.48E-01 | 1 |
| FBXW2 | 5.39E-01 | 1 | SSH2 | 7.49E-01 | 1 |
| ZNF830 | 5.39E-01 | 1 | BAZ2B | 7.49E-01 | 1 |
| GPR148 | 5.39E-01 | 1 | DZIP1 | 7.49E-01 | 1 |
| ZNF610 | 5.39E-01 | 1 | RBM15 | 7.49E-01 | 1 |
| FLT3 | 5.39E-01 | 1 | TNRC6B | 7.49E-01 | 1 |
| ING3 | 5.39E-01 | 1 | WWP2 | 7.50E-01 | 1 |
| SRPK3 | 5.40E-01 | 1 | CDH17 | 7.50E-01 | 1 |
| EDEM3 | 5.40E-01 | 1 | PTPN13 | 7.50E-01 | 1 |
| PCDHA13 | 5.40E-01 | 1 | ADAM22 | 7.50E-01 | 1 |

| ZFR2 | 5.40E-01 | 1 | DCLK1 | 7.50E-01 | 1 |
| --- | --- | --- | --- | --- | --- |
| TRHR | 5.40E-01 | 1 | GLIS3 | 7.51E-01 | 1 |
| WNT2 | 5.40E-01 | 1 | PHKB | 7.51E-01 | 1 |
| ZSCAN23 | 5.40E-01 | 1 | TMC1 | 7.51E-01 | 1 |
| DPY19L3 | 5.40E-01 | 1 | CDH2 | 7.51E-01 | 1 |
| GSN | 5.40E-01 | 1 | A2M | 7.51E-01 | 1 |
| LRP1 | 5.40E-01 | 1 | C14orf37 | 7.51E-01 | 1 |
| CCDC89 | 5.40E-01 | 1 | TANC2 | 7.51E-01 | 1 |
| PRSS23 | 5.41E-01 | 1 | CCDC30 | 7.52E-01 | 1 |
| PLA2G7 | 5.41E-01 | 1 | SPAM1 | 7.52E-01 | 1 |
| CHSY1 | 5.41E-01 | 1 | KDM2B | 7.52E-01 | 1 |
| SPNS1 | 5.41E-01 | 1 | KIAA1524 | 7.53E-01 | 1 |
| DDX42 | 5.41E-01 | 1 | OR8K3 | 7.54E-01 | 1 |
| RFPL3 | 5.41E-01 | 1 | ODF2 | 7.54E-01 | 1 |
| C1QTNF9 | 5.41E-01 | 1 | SULF2 | 7.54E-01 | 1 |
| CILP2 | 5.41E-01 | 1 | MAP4 | 7.55E-01 | 1 |
| GPR22 | 5.42E-01 | 1 | MUT | 7.55E-01 | 1 |
| HCRTR1 | 5.42E-01 | 1 | MTR | 7.55E-01 | 1 |
| VWA2 | 5.42E-01 | 1 | HIPK3 | 7.56E-01 | 1 |
| LRRC16A | 5.42E-01 | 1 | EPHB3 | 7.56E-01 | 1 |
| CTDP1 | 5.42E-01 | 1 | HTR1A | 7.56E-01 | 1 |
| IKZF3 | 5.42E-01 | 1 | IPO9 | 7.56E-01 | 1 |
| PPARG | 5.42E-01 | 1 | ANO3 | 7.56E-01 | 1 |
| PDK4 | 5.42E-01 | 1 | C21orf59 | 7.57E-01 | 1 |
| INTS5 | 5.43E-01 | 1 | PPIP5K2 | 7.57E-01 | 1 |
| AKAP3 | 5.43E-01 | 1 | NLGN4Y | 7.58E-01 | 1 |
| MNT | 5.43E-01 | 1 | TTC28 | 7.58E-01 | 1 |
| RUNX2 | 5.43E-01 | 1 | PDCD6IP | 7.58E-01 | 1 |
| AGPAT6 | 5.43E-01 | 1 | GRIN3A | 7.58E-01 | 1 |
| GJA4 | 5.43E-01 | 1 | COL4A5 | 7.58E-01 | 1 |
| CCDC50 | 5.43E-01 | 1 | RANBP6 | 7.59E-01 | 1 |
| KIAA0408 | 5.43E-01 | 1 | HEATR1 | 7.59E-01 | 1 |
| HTR3B | 5.43E-01 | 1 | PLD1 | 7.59E-01 | 1 |
| CREB3L2 | 5.44E-01 | 1 | UBA7 | 7.59E-01 | 1 |
| PKLR | 5.44E-01 | 1 | FRMPD4 | 7.60E-01 | 1 |
| RBM42 | 5.44E-01 | 1 | OSBPL7 | 7.60E-01 | 1 |
| ATG4A | 5.44E-01 | 1 | CLCA4 | 7.60E-01 | 1 |
| QRSL1 | 5.44E-01 | 1 | SNCAIP | 7.60E-01 | 1 |
| KEL | 5.44E-01 | 1 | AMY1C | 7.61E-01 | 1 |
| FAM117A | 5.44E-01 | 1 | EPHB6 | 7.61E-01 | 1 |
| HABP4 | 5.44E-01 | 1 | SMARCA1 | 7.61E-01 | 1 |
| MATR3 | 5.44E-01 | 1 | ZNF878 | 7.61E-01 | 1 |
| SCD5 | 5.44E-01 | 1 | ZNF431 | 7.61E-01 | 1 |
| OR13G1 | 5.45E-01 | 1 | CCDC88B | 7.62E-01 | 1 |
| GABRR2 | 5.45E-01 | 1 | GATA3 | 7.62E-01 | 1 |
| DBT | 5.45E-01 | 1 | SHANK2 | 7.62E-01 | 1 |
| KLF11 | 5.45E-01 | 1 | PRRT4 | 7.63E-01 | 1 |
| KPNA4 | 5.45E-01 | 1 | URB2 | 7.63E-01 | 1 |
| LETMD1 | 5.45E-01 | 1 | VSIG10L | 7.63E-01 | 1 |
| NCOR1 | 5.45E-01 | 1 | ANKRD20A | 7.63E-01 | 1 |
| ZSWIM6 | 5.45E-01 | 1 | ADAM10 | 7.64E-01 | 1 |
| SLC13A3 | 5.45E-01 | 1 | CDK13 | 7.64E-01 | 1 |
| KRT80 | 5.45E-01 | 1 | MED23 | 7.64E-01 | 1 |

| MRPS22 | 5.45E-01 | 1 | ITSN2 | 7.64E-01 | 1 |
| --- | --- | --- | --- | --- | --- |
| SLC30A6 | 5.45E-01 | 1 | SHC4 | 7.64E-01 | 1 |
| FUT9 | 5.46E-01 | 1 | JAK2 | 7.64E-01 | 1 |
| C10orf88 | 5.46E-01 | 1 | DNAH1 | 7.64E-01 | 1 |
| TMPRSS1 | 5.46E-01 | 1 | ASB13 | 7.65E-01 | 1 |
| EDN1 | 5.46E-01 | 1 | CHRD | 7.65E-01 | 1 |
| ST14 | 5.46E-01 | 1 | KIAA1210 | 7.66E-01 | 1 |
| SLFN14 | 5.46E-01 | 1 | SALL4 | 7.66E-01 | 1 |
| ADAM2 | 5.46E-01 | 1 | IDS | 7.66E-01 | 1 |
| DISC1 | 5.46E-01 | 1 | TOPBP1 | 7.67E-01 | 1 |
| OR13C8 | 5.46E-01 | 1 | USP47 | 7.67E-01 | 1 |
| LEKR1 | 5.46E-01 | 1 | KLHL4 | 7.67E-01 | 1 |
| MCCC2 | 5.46E-01 | 1 | ENTPD1 | 7.67E-01 | 1 |
| FST | 5.46E-01 | 1 | OCA2 | 7.67E-01 | 1 |
| HCLS1 | 5.46E-01 | 1 | NCOA6 | 7.68E-01 | 1 |
| PRMT8 | 5.46E-01 | 1 | PIGN | 7.68E-01 | 1 |
| BTAF1 | 5.46E-01 | 1 | DHX57 | 7.68E-01 | 1 |
| NME7 | 5.46E-01 | 1 | MYOM1 | 7.68E-01 | 1 |
| PLAG1 | 5.46E-01 | 1 | LRRN4 | 7.70E-01 | 1 |
| CACNA1H | 5.47E-01 | 1 | TLR7 | 7.71E-01 | 1 |
| MEP1A | 5.47E-01 | 1 | IFT140 | 7.71E-01 | 1 |
| TRIM4 | 5.47E-01 | 1 | CHTF18 | 7.71E-01 | 1 |
| TMEFF1 | 5.47E-01 | 1 | ABCB6 | 7.72E-01 | 1 |
| OR3A3 | 5.47E-01 | 1 | SH3PXD2B | 7.72E-01 | 1 |
| MMP14 | 5.47E-01 | 1 | SYT9 | 7.72E-01 | 1 |
| IGF2BP2 | 5.47E-01 | 1 | ADAMTS9 | 7.72E-01 | 1 |
| EMILIN1 | 5.47E-01 | 1 | AXIN2 | 7.73E-01 | 1 |
| BTNL3 | 5.47E-01 | 1 | RNF20 | 7.73E-01 | 1 |
| STK35 | 5.47E-01 | 1 | FOXG1 | 7.73E-01 | 1 |
| F5 | 5.47E-01 | 1 | PKHD1 | 7.74E-01 | 1 |
| EXOC6B | 5.48E-01 | 1 | FOXR1 | 7.74E-01 | 1 |
| MMP17 | 5.48E-01 | 1 | XIRP2 | 7.74E-01 | 1 |
| TULP3 | 5.48E-01 | 1 | PHC1 | 7.75E-01 | 1 |
| USP37 | 5.48E-01 | 1 | MAMLD1 | 7.75E-01 | 1 |
| TRIM42 | 5.48E-01 | 1 | PCSK6 | 7.75E-01 | 1 |
| SCRN1 | 5.48E-01 | 1 | MYCBP2 | 7.75E-01 | 1 |
| TAF1 | 5.48E-01 | 1 | ZFP64 | 7.75E-01 | 1 |
| CPSF7 | 5.48E-01 | 1 | XPO1 | 7.76E-01 | 1 |
| CDS1 | 5.48E-01 | 1 | NLRP12 | 7.76E-01 | 1 |
| DHTKD1 | 5.48E-01 | 1 | DNAH8 | 7.76E-01 | 1 |
| CD33 | 5.48E-01 | 1 | SPTA1 | 7.76E-01 | 1 |
| TACR1 | 5.48E-01 | 1 | TRHDE | 7.76E-01 | 1 |
| EPS8 | 5.49E-01 | 1 | NLRP4 | 7.77E-01 | 1 |
| RNPEP | 5.49E-01 | 1 | KIF2B | 7.77E-01 | 1 |
| SLC38A3 | 5.49E-01 | 1 | USP53 | 7.77E-01 | 1 |
| PRDM4 | 5.49E-01 | 1 | TAOK2 | 7.78E-01 | 1 |
| CHST7 | 5.49E-01 | 1 | ULK4 | 7.78E-01 | 1 |
| TREML4 | 5.49E-01 | 1 | AGBL5 | 7.78E-01 | 1 |
| LGI4 | 5.49E-01 | 1 | PDE8A | 7.79E-01 | 1 |
| OR52N5 | 5.49E-01 | 1 | PMS2 | 7.79E-01 | 1 |
| ARHGEF3 | 5.49E-01 | 1 | ACAD8 | 7.79E-01 | 1 |
| ACIN1 | 5.49E-01 | 1 | ABCB11 | 7.79E-01 | 1 |
| SPHK2 | 5.49E-01 | 1 | ALMS1 | 7.79E-01 | 1 |

| EXOSC7 | 5.49E-01 | 1 | KCNQ2 | 7.80E-01 | 1 |
| --- | --- | --- | --- | --- | --- |
| ZNF229 | 5.49E-01 | 1 | TRIP12 | 7.80E-01 | 1 |
| PSG6 | 5.50E-01 | 1 | LEMD3 | 7.81E-01 | 1 |
| DPPA2 | 5.50E-01 | 1 | KIAA0922 | 7.81E-01 | 1 |
| DDX21 | 5.50E-01 | 1 | FRMD4A | 7.81E-01 | 1 |
| CPB2 | 5.50E-01 | 1 | PKDREJ | 7.82E-01 | 1 |
| EXOSC10 | 5.50E-01 | 1 | ZNF827 | 7.82E-01 | 1 |
| SLC16A13 | 5.50E-01 | 1 | TLR5 | 7.82E-01 | 1 |
| CCPG1 | 5.50E-01 | 1 | DLEC1 | 7.82E-01 | 1 |
| SERPIND1 | 5.50E-01 | 1 | HMMR | 7.82E-01 | 1 |
| UCP1 | 5.51E-01 | 1 | CAD | 7.83E-01 | 1 |
| PSEN1 | 5.51E-01 | 1 | TP53BP2 | 7.83E-01 | 1 |
| ERO1LB | 5.51E-01 | 1 | IL16 | 7.83E-01 | 1 |
| CD276 | 5.51E-01 | 1 | CWF19L2 | 7.83E-01 | 1 |
| OR2L3 | 5.51E-01 | 1 | EXOC2 | 7.83E-01 | 1 |
| LDHB | 5.51E-01 | 1 | KIAA0232 | 7.83E-01 | 1 |
| GLYATL1 | 5.51E-01 | 1 | KIAA1324L | 7.83E-01 | 1 |
| ARHGAP5 | 5.51E-01 | 1 | NEO1 | 7.84E-01 | 1 |
| RASD2 | 5.52E-01 | 1 | PROS1 | 7.84E-01 | 1 |
| NRBP1 | 5.52E-01 | 1 | DCLRE1A | 7.84E-01 | 1 |
| TBL1Y | 5.52E-01 | 1 | OSBPL3 | 7.84E-01 | 1 |
| PCDHB6 | 5.52E-01 | 1 | SCUBE1 | 7.84E-01 | 1 |
| ADCK4 | 5.52E-01 | 1 | PLXNB3 | 7.84E-01 | 1 |
| TINF2 | 5.52E-01 | 1 | CELSR1 | 7.84E-01 | 1 |
| OR5AR1 | 5.52E-01 | 1 | NCOR2 | 7.85E-01 | 1 |
| ZNF714 | 5.52E-01 | 1 | FAM155A | 7.85E-01 | 1 |
| ZCCHC5 | 5.52E-01 | 1 | PCLO | 7.86E-01 | 1 |
| PLCL1 | 5.52E-01 | 1 | RFX7 | 7.86E-01 | 1 |
| TEAD2 | 5.53E-01 | 1 | PCDH18 | 7.86E-01 | 1 |
| SH2D3A | 5.53E-01 | 1 | SPAG17 | 7.86E-01 | 1 |
| SLC7A4 | 5.53E-01 | 1 | PALB2 | 7.86E-01 | 1 |
| OR10R2 | 5.53E-01 | 1 | POLN | 7.87E-01 | 1 |
| EFR3A | 5.53E-01 | 1 | PPP1R9A | 7.87E-01 | 1 |
| POLE2 | 5.53E-01 | 1 | ATP2B3 | 7.87E-01 | 1 |
| LIG1 | 5.53E-01 | 1 | KDM5B | 7.87E-01 | 1 |
| ZNF286A | 5.53E-01 | 1 | MAP1B | 7.87E-01 | 1 |
| NACC2 | 5.53E-01 | 1 | CLCA2 | 7.87E-01 | 1 |
| OR5H15 | 5.53E-01 | 1 | CSPG4 | 7.87E-01 | 1 |
| FRMPD1 | 5.54E-01 | 1 | CAMTA1 | 7.87E-01 | 1 |
| ACVR1 | 5.54E-01 | 1 | MST1R | 7.88E-01 | 1 |
| LGALS3B | 5.54E-01 | 1 | PLA2G4E | 7.89E-01 | 1 |
| CD3EAP | 5.54E-01 | 1 | ZNF398 | 7.89E-01 | 1 |
| OR5T3 | 5.54E-01 | 1 | PSMD1 | 7.90E-01 | 1 |
| ARMC3 | 5.54E-01 | 1 | ARHGAP17 | 7.90E-01 | 1 |
| SLC2A13 | 5.54E-01 | 1 | CCNB3 | 7.90E-01 | 1 |
| COG1 | 5.54E-01 | 1 | NOTCH2 | 7.90E-01 | 1 |
| TM9SF3 | 5.54E-01 | 1 | CYP3A43 | 7.90E-01 | 1 |
| ALPI | 5.54E-01 | 1 | CDH26 | 7.90E-01 | 1 |
| GPLD1 | 5.54E-01 | 1 | FLYWCH1 | 7.91E-01 | 1 |
| IGF2R | 5.54E-01 | 1 | CNOT1 | 7.91E-01 | 1 |
| TMPO | 5.55E-01 | 1 | ZNF708 | 7.91E-01 | 1 |
| LCP1 | 5.55E-01 | 1 | PPAN-P2RY | 7.91E-01 | 1 |
| TMEM63C | 5.55E-01 | 1 | GRIA3 | 7.91E-01 | 1 |

| QRICH2 | 5.55E-01 | 1 | EP400 | 7.92E-01 | 1 |
| --- | --- | --- | --- | --- | --- |
| FCAMR | 5.55E-01 | 1 | ZHX2 | 7.93E-01 | 1 |
| GLRA2 | 5.55E-01 | 1 | ALDH16A1 | 7.93E-01 | 1 |
| PPP1R15B | 5.55E-01 | 1 | USP38 | 7.93E-01 | 1 |
| NCAM1 | 5.55E-01 | 1 | UBR4 | 7.94E-01 | 1 |
| C2 | 5.55E-01 | 1 | CACNA2D4 | 7.94E-01 | 1 |
| KCNJ10 | 5.55E-01 | 1 | SMCHD1 | 7.94E-01 | 1 |
| NUDT17 | 5.55E-01 | 1 | SDK2 | 7.94E-01 | 1 |
| POM121 | 5.55E-01 | 1 | RAPGEF6 | 7.94E-01 | 1 |
| AKT3 | 5.56E-01 | 1 | NUP153 | 7.94E-01 | 1 |
| CARS | 5.56E-01 | 1 | ZNF585B | 7.94E-01 | 1 |
| PCDHGC4 | 5.56E-01 | 1 | HEATR5B | 7.94E-01 | 1 |
| POGZ | 5.56E-01 | 1 | TET3 | 7.96E-01 | 1 |
| RCBTB2 | 5.56E-01 | 1 | WDHD1 | 7.96E-01 | 1 |
| SMARCB1 | 5.56E-01 | 1 | FANCM | 7.96E-01 | 1 |
| RANBP2 | 5.57E-01 | 1 | WDFY4 | 7.96E-01 | 1 |
| MFSD5 | 5.57E-01 | 1 | IQSEC2 | 7.96E-01 | 1 |
| SLC2A4 | 5.57E-01 | 1 | PCDHA1 | 7.97E-01 | 1 |
| ARHGAP1 | 5.57E-01 | 1 | ANKRD17 | 7.97E-01 | 1 |
| OCRL | 5.57E-01 | 1 | MCF2 | 7.97E-01 | 1 |
| LARP4B | 5.57E-01 | 1 | ADAMTSL | 7.97E-01 | 1 |
| TUBAL3 | 5.57E-01 | 1 | ZNF676 | 7.97E-01 | 1 |
| ZNF263 | 5.57E-01 | 1 | VPS8 | 7.97E-01 | 1 |
| CNPY4 | 5.57E-01 | 1 | ZBTB11 | 7.98E-01 | 1 |
| OR10A5 | 5.57E-01 | 1 | CTNNA3 | 7.98E-01 | 1 |
| SLC2A3 | 5.58E-01 | 1 | CACHD1 | 7.98E-01 | 1 |
| SEMA3B | 5.58E-01 | 1 | PIK3C2A | 7.98E-01 | 1 |
| ST6GALN | 5.58E-01 | 1 | PRPF8 | 7.99E-01 | 1 |
| DES | 5.58E-01 | 1 | TOPORS | 7.99E-01 | 1 |
| HGF | 5.58E-01 | 1 | FNBP4 | 7.99E-01 | 1 |
| PARG | 5.58E-01 | 1 | ODF2L | 8.00E-01 | 1 |
| DUSP1 | 5.58E-01 | 1 | PDE4A | 8.00E-01 | 1 |
| SGCB | 5.58E-01 | 1 | PNLIP | 8.00E-01 | 1 |
| PHACTR3 | 5.58E-01 | 1 | CACNA1H | 8.00E-01 | 1 |
| SKIL | 5.58E-01 | 1 | RGAG1 | 8.01E-01 | 1 |
| OR2T33 | 5.59E-01 | 1 | TRAPPC9 | 8.01E-01 | 1 |
| CYP7B1 | 5.59E-01 | 1 | KCP | 8.01E-01 | 1 |
| MAP7 | 5.59E-01 | 1 | NAALADL | 8.01E-01 | 1 |
| LRIT3 | 5.59E-01 | 1 | DCTN1 | 8.02E-01 | 1 |
| RPH3AL | 5.59E-01 | 1 | HELZ | 8.02E-01 | 1 |
| SLC27A3 | 5.59E-01 | 1 | GOLGA3 | 8.02E-01 | 1 |
| PAK2 | 5.59E-01 | 1 | SORCS1 | 8.02E-01 | 1 |
| OR6K6 | 5.59E-01 | 1 | NOX4 | 8.02E-01 | 1 |
| RBM27 | 5.60E-01 | 1 | CLMN | 8.02E-01 | 1 |
| ACER3 | 5.60E-01 | 1 | NBPF9 | 8.03E-01 | 1 |
| ENPP6 | 5.60E-01 | 1 | PCDHGA9 | 8.03E-01 | 1 |
| GAB3 | 5.60E-01 | 1 | NPR1 | 8.04E-01 | 1 |
| HTR3E | 5.60E-01 | 1 | SREBF1 | 8.04E-01 | 1 |
| ZNF311 | 5.60E-01 | 1 | UNC13B | 8.04E-01 | 1 |
| CUBN | 5.61E-01 | 1 | AGTPBP1 | 8.05E-01 | 1 |
| ADAM7 | 5.61E-01 | 1 | SEMA3C | 8.05E-01 | 1 |
| TRIP12 | 5.61E-01 | 1 | POLR2A | 8.05E-01 | 1 |
| MPP3 | 5.61E-01 | 1 | DNM3 | 8.06E-01 | 1 |

| ABLIM1 | 5.61E-01 | 1 | KIF15 | 8.07E-01 | 1 |
| --- | --- | --- | --- | --- | --- |
| OSBPL9 | 5.61E-01 | 1 | MERTK | 8.07E-01 | 1 |
| BAIAP2L1 | 5.61E-01 | 1 | CCDC132 | 8.07E-01 | 1 |
| HSD3B1 | 5.61E-01 | 1 | GPR149 | 8.07E-01 | 1 |
| MEX3D | 5.61E-01 | 1 | WNK2 | 8.08E-01 | 1 |
| PCDHGA1 | 5.61E-01 | 1 | GPR101 | 8.08E-01 | 1 |
| SLC1A3 | 5.61E-01 | 1 | KCNH1 | 8.09E-01 | 1 |
| WDR46 | 5.62E-01 | 1 | HPSE | 8.09E-01 | 1 |
| FAM134A | 5.62E-01 | 1 | PCDHGA8 | 8.09E-01 | 1 |
| C3orf20 | 5.62E-01 | 1 | PITPNM1 | 8.10E-01 | 1 |
| RNF130 | 5.62E-01 | 1 | ENAM | 8.10E-01 | 1 |
| SOHLH1 | 5.62E-01 | 1 | EGFR | 8.10E-01 | 1 |
| STK31 | 5.62E-01 | 1 | HELB | 8.11E-01 | 1 |
| SMPD1 | 5.62E-01 | 1 | NDST3 | 8.11E-01 | 1 |
| OLFM3 | 5.63E-01 | 1 | LENG8 | 8.11E-01 | 1 |
| OR4D5 | 5.63E-01 | 1 | SETBP1 | 8.11E-01 | 1 |
| RBMXL2 | 5.63E-01 | 1 | WBSCR17 | 8.11E-01 | 1 |
| LDB2 | 5.63E-01 | 1 | RBMXL3 | 8.11E-01 | 1 |
| OR13C5 | 5.63E-01 | 1 | FAM133A | 8.12E-01 | 1 |
| ZBTB22 | 5.63E-01 | 1 | ZNF469 | 8.12E-01 | 1 |
| MAP2K1 | 5.63E-01 | 1 | LRRIQ1 | 8.12E-01 | 1 |
| SPATA7 | 5.64E-01 | 1 | ZNF335 | 8.12E-01 | 1 |
| CARD8 | 5.64E-01 | 1 | C9orf84 | 8.12E-01 | 1 |
| CCNB3 | 5.64E-01 | 1 | DHX36 | 8.12E-01 | 1 |
| GATAD2A | 5.64E-01 | 1 | ZNF675 | 8.12E-01 | 1 |
| TOE1 | 5.64E-01 | 1 | ZNF836 | 8.13E-01 | 1 |
| TRIML2 | 5.64E-01 | 1 | GRM7 | 8.13E-01 | 1 |
| RTN4R | 5.64E-01 | 1 | FCRL5 | 8.14E-01 | 1 |
| OR2H2 | 5.64E-01 | 1 | SCARF2 | 8.14E-01 | 1 |
| NAGLU | 5.64E-01 | 1 | KCNH7 | 8.14E-01 | 1 |
| UGT1A10 | 5.65E-01 | 1 | ANKRD27 | 8.15E-01 | 1 |
| KCNJ1 | 5.65E-01 | 1 | PMP2 | 8.17E-01 | 1 |
| PRAMEF1 | 5.65E-01 | 1 | CHSY3 | 8.17E-01 | 1 |
| ZNF695 | 5.65E-01 | 1 | DCHS1 | 8.18E-01 | 1 |
| BIN2 | 5.65E-01 | 1 | BMPER | 8.18E-01 | 1 |
| ZNF423 | 5.65E-01 | 1 | ROBO2 | 8.18E-01 | 1 |
| TBC1D9 | 5.66E-01 | 1 | PPP1R12A | 8.19E-01 | 1 |
| OR6A2 | 5.66E-01 | 1 | BRCA2 | 8.19E-01 | 1 |
| P2RY13 | 5.66E-01 | 1 | LRP4 | 8.19E-01 | 1 |
| NAPSA | 5.66E-01 | 1 | ATP7A | 8.19E-01 | 1 |
| PPT1 | 5.66E-01 | 1 | LRRC16B | 8.20E-01 | 1 |
| TAF6L | 5.66E-01 | 1 | PLEKHG1 | 8.20E-01 | 1 |
| F2RL3 | 5.66E-01 | 1 | CHD3 | 8.21E-01 | 1 |
| OAT | 5.66E-01 | 1 | LTBP2 | 8.21E-01 | 1 |
| STRADA | 5.66E-01 | 1 | NUP210 | 8.21E-01 | 1 |
| BTBD16 | 5.66E-01 | 1 | GPATCH8 | 8.21E-01 | 1 |
| CD19 | 5.66E-01 | 1 | TANC1 | 8.21E-01 | 1 |
| PNKP | 5.66E-01 | 1 | MCM2 | 8.22E-01 | 1 |
| NKX2-4 | 5.67E-01 | 1 | NCAM1 | 8.23E-01 | 1 |
| KCNH5 | 5.67E-01 | 1 | CDK5RAP2 | 8.23E-01 | 1 |
| KLHL22 | 5.67E-01 | 1 | HECW2 | 8.23E-01 | 1 |
| C10orf90 | 5.67E-01 | 1 | DGKI | 8.23E-01 | 1 |
| KHDRBS3 | 5.67E-01 | 1 | SECISBP2L | 8.23E-01 | 1 |

| OTOP2 | 5.67E-01 | 1 | DMBT1 | 8.24E-01 | 1 |
| --- | --- | --- | --- | --- | --- |
| GCLC | 5.67E-01 | 1 | MORC4 | 8.24E-01 | 1 |
| RDH13 | 5.67E-01 | 1 | ZNF420 | 8.24E-01 | 1 |
| FLI1 | 5.67E-01 | 1 | PYGB | 8.24E-01 | 1 |
| ZNF451 | 5.67E-01 | 1 | TLN1 | 8.25E-01 | 1 |
| ARHGEF3 | 5.67E-01 | 1 | RNASEL | 8.25E-01 | 1 |
| TINAG | 5.68E-01 | 1 | ZNF628 | 8.26E-01 | 1 |
| GPAA1 | 5.68E-01 | 1 | PDZRN3 | 8.26E-01 | 1 |
| TSPYL2 | 5.68E-01 | 1 | ZNF646 | 8.27E-01 | 1 |
| SELENBP | 5.68E-01 | 1 | SCN2A | 8.28E-01 | 1 |
| TRDMT1 | 5.68E-01 | 1 | TBC1D4 | 8.28E-01 | 1 |
| RNF40 | 5.68E-01 | 1 | SORBS1 | 8.28E-01 | 1 |
| OXGR1 | 5.69E-01 | 1 | DUSP27 | 8.28E-01 | 1 |
| GRIA4 | 5.69E-01 | 1 | SLC9A2 | 8.29E-01 | 1 |
| DOT1L | 5.69E-01 | 1 | HRNR | 8.29E-01 | 1 |
| TTC25 | 5.69E-01 | 1 | NIPBL | 8.29E-01 | 1 |
| HEG1 | 5.69E-01 | 1 | AHNAK | 8.30E-01 | 1 |
| DPP6 | 5.69E-01 | 1 | ATP2A2 | 8.30E-01 | 1 |
| ZBTB8B | 5.69E-01 | 1 | PCDHB4 | 8.30E-01 | 1 |
| PGBD4 | 5.69E-01 | 1 | COL19A1 | 8.30E-01 | 1 |
| FANCC | 5.69E-01 | 1 | CEP290 | 8.31E-01 | 1 |
| BTD | 5.69E-01 | 1 | ATRN | 8.31E-01 | 1 |
| TARSL2 | 5.70E-01 | 1 | LAMB4 | 8.32E-01 | 1 |
| SIAE | 5.70E-01 | 1 | ZNF808 | 8.32E-01 | 1 |
| FMOD | 5.70E-01 | 1 | COL9A2 | 8.32E-01 | 1 |
| SNX9 | 5.70E-01 | 1 | COL5A3 | 8.33E-01 | 1 |
| PKD2L1 | 5.70E-01 | 1 | IQUB | 8.33E-01 | 1 |
| BCLAF1 | 5.70E-01 | 1 | DAB2IP | 8.33E-01 | 1 |
| PTCHD3 | 5.70E-01 | 1 | SMARCA2 | 8.34E-01 | 1 |
| CCDC54 | 5.70E-01 | 1 | PLXND1 | 8.34E-01 | 1 |
| SCAP | 5.71E-01 | 1 | MYO10 | 8.35E-01 | 1 |
| FKBP6 | 5.71E-01 | 1 | TJP1 | 8.35E-01 | 1 |
| ZNF645 | 5.71E-01 | 1 | MUC6 | 8.35E-01 | 1 |
| CRTC3 | 5.71E-01 | 1 | WRN | 8.35E-01 | 1 |
| RTN4IP1 | 5.71E-01 | 1 | ZNF429 | 8.36E-01 | 1 |
| ARHGEF6 | 5.71E-01 | 1 | FLNA | 8.36E-01 | 1 |
| ALDH1A1 | 5.71E-01 | 1 | HERC2 | 8.36E-01 | 1 |
| PLEKHH2 | 5.71E-01 | 1 | HEPH | 8.37E-01 | 1 |
| DHRS7C | 5.71E-01 | 1 | SLITRK5 | 8.37E-01 | 1 |
| SLC6A4 | 5.71E-01 | 1 | EPRS | 8.37E-01 | 1 |
| MGRN1 | 5.71E-01 | 1 | DICER1 | 8.38E-01 | 1 |
| WDR36 | 5.71E-01 | 1 | C12orf40 | 8.38E-01 | 1 |
| CENPE | 5.71E-01 | 1 | NCKAP1 | 8.38E-01 | 1 |
| PRKACA | 5.72E-01 | 1 | ZNF253 | 8.39E-01 | 1 |
| PCGF6 | 5.72E-01 | 1 | DNAJC13 | 8.39E-01 | 1 |
| EHD3 | 5.72E-01 | 1 | SGK223 | 8.39E-01 | 1 |
| RANBP3 | 5.72E-01 | 1 | MPDZ | 8.39E-01 | 1 |
| ING1 | 5.72E-01 | 1 | PLCB4 | 8.39E-01 | 1 |
| CABLES2 | 5.73E-01 | 1 | CDH10 | 8.39E-01 | 1 |
| SEZ6L2 | 5.73E-01 | 1 | YTHDC2 | 8.40E-01 | 1 |
| KCND2 | 5.73E-01 | 1 | ATAD2 | 8.40E-01 | 1 |
| PDE12 | 5.73E-01 | 1 | HTT | 8.40E-01 | 1 |
| CNTN1 | 5.73E-01 | 1 | SEC16B | 8.40E-01 | 1 |

| PGK2 | 5.73E-01 | 1 | CGNL1 | 8.40E-01 | 1 |
| --- | --- | --- | --- | --- | --- |
| LCMT2 | 5.73E-01 | 1 | NOS2 | 8.40E-01 | 1 |
| PABPC1L | 5.73E-01 | 1 | POF1B | 8.40E-01 | 1 |
| LGALS9 | 5.74E-01 | 1 | SETD1A | 8.41E-01 | 1 |
| FBXW11 | 5.74E-01 | 1 | REV3L | 8.41E-01 | 1 |
| BLNK | 5.74E-01 | 1 | DTNA | 8.41E-01 | 1 |
| KRTAP26 | 5.74E-01 | 1 | PRSS12 | 8.41E-01 | 1 |
| DDO | 5.74E-01 | 1 | TIAM2 | 8.41E-01 | 1 |
| PLK1 | 5.74E-01 | 1 | OCRL | 8.41E-01 | 1 |
| FAM169A | 5.75E-01 | 1 | ZFAT | 8.41E-01 | 1 |
| DOK5 | 5.75E-01 | 1 | RIPK3 | 8.42E-01 | 1 |
| IFNAR1 | 5.75E-01 | 1 | ZNFX1 | 8.42E-01 | 1 |
| PIK3C2G | 5.75E-01 | 1 | SYT1 | 8.42E-01 | 1 |
| NR1D2 | 5.75E-01 | 1 | ATAD2B | 8.42E-01 | 1 |
| RPS6KA1 | 5.75E-01 | 1 | ANKRD36B | 8.42E-01 | 1 |
| PEG10 | 5.75E-01 | 1 | KIAA0556 | 8.43E-01 | 1 |
| PFKFB1 | 5.75E-01 | 1 | PCDH19 | 8.43E-01 | 1 |
| F2 | 5.75E-01 | 1 | WDR11 | 8.44E-01 | 1 |
| CLPTM1L | 5.75E-01 | 1 | BBX | 8.45E-01 | 1 |
| BTBD11 | 5.75E-01 | 1 | TRPM6 | 8.45E-01 | 1 |
| FNTB | 5.75E-01 | 1 | NLRP7 | 8.45E-01 | 1 |
| RIMBP2 | 5.75E-01 | 1 | FSTL5 | 8.45E-01 | 1 |
| LOX | 5.75E-01 | 1 | CDH19 | 8.46E-01 | 1 |
| TTC27 | 5.75E-01 | 1 | XCL2 | 8.46E-01 | 1 |
| EML2 | 5.75E-01 | 1 | PCDHB9 | 8.46E-01 | 1 |
| MAPKAP | 5.76E-01 | 1 | SCN5A | 8.46E-01 | 1 |
| FBXO15 | 5.76E-01 | 1 | SYCP1 | 8.46E-01 | 1 |
| HACE1 | 5.76E-01 | 1 | PIGO | 8.47E-01 | 1 |
| FAM73A | 5.76E-01 | 1 | UHRF2 | 8.47E-01 | 1 |
| GOLGA4 | 5.76E-01 | 1 | PCDHB10 | 8.47E-01 | 1 |
| PCDHGA7 | 5.76E-01 | 1 | RIMBP2 | 8.47E-01 | 1 |
| SACM1L | 5.76E-01 | 1 | RSPH10B | 8.47E-01 | 1 |
| SLC16A14 | 5.77E-01 | 1 | ANKRD11 | 8.48E-01 | 1 |
| SETD6 | 5.77E-01 | 1 | GRID1 | 8.48E-01 | 1 |
| GLIPR1L1 | 5.77E-01 | 1 | MN1 | 8.48E-01 | 1 |
| DNM3 | 5.77E-01 | 1 | USP24 | 8.48E-01 | 1 |
| KRBA1 | 5.77E-01 | 1 | RBM12 | 8.49E-01 | 1 |
| PRR12 | 5.77E-01 | 1 | NF1 | 8.49E-01 | 1 |
| GJD2 | 5.77E-01 | 1 | LCT | 8.49E-01 | 1 |
| RABEP2 | 5.77E-01 | 1 | TRIOBP | 8.49E-01 | 1 |
| DNA2 | 5.77E-01 | 1 | MAP3K1 | 8.49E-01 | 1 |
| GPR142 | 5.77E-01 | 1 | CHRM3 | 8.50E-01 | 1 |
| TRIM10 | 5.77E-01 | 1 | CHD8 | 8.50E-01 | 1 |
| VSX2 | 5.78E-01 | 1 | SVEP1 | 8.51E-01 | 1 |
| MAP3K1 | 5.78E-01 | 1 | TRIO | 8.51E-01 | 1 |
| FEM1C | 5.78E-01 | 1 | MYO7A | 8.51E-01 | 1 |
| ZNF837 | 5.78E-01 | 1 | MACF1 | 8.51E-01 | 1 |
| OR51T1 | 5.78E-01 | 1 | TMEM132C | 8.52E-01 | 1 |
| WDR17 | 5.78E-01 | 1 | SETD1B | 8.52E-01 | 1 |
| DUSP27 | 5.78E-01 | 1 | TMC3 | 8.52E-01 | 1 |
| C8B | 5.78E-01 | 1 | GRIK2 | 8.52E-01 | 1 |
| XRCC5 | 5.78E-01 | 1 | CWH43 | 8.53E-01 | 1 |
| POF1B | 5.78E-01 | 1 | KIF4A | 8.53E-01 | 1 |

| TTC22 | 5.78E-01 | 1 | CC2D2A | 8.53E-01 | 1 |
| --- | --- | --- | --- | --- | --- |
| OGFR | 5.78E-01 | 1 | ABCB4 | 8.54E-01 | 1 |
| ABCA12 | 5.78E-01 | 1 | PIK3CB | 8.54E-01 | 1 |
| DENND4C | 5.79E-01 | 1 | SLC4A4 | 8.54E-01 | 1 |
| TRIM21 | 5.79E-01 | 1 | ADAMTS1 | 8.54E-01 | 1 |
| NDST4 | 5.79E-01 | 1 | NCAN | 8.54E-01 | 1 |
| KITLG | 5.79E-01 | 1 | PCDH1 | 8.55E-01 | 1 |
| HHIP | 5.79E-01 | 1 | SBF1 | 8.55E-01 | 1 |
| ELMOD1 | 5.79E-01 | 1 | HDX | 8.55E-01 | 1 |
| TCEB3 | 5.79E-01 | 1 | MET | 8.56E-01 | 1 |
| STX6 | 5.79E-01 | 1 | MYO5C | 8.56E-01 | 1 |
| LILRA6 | 5.79E-01 | 1 | SPHKAP | 8.56E-01 | 1 |
| ZNF563 | 5.79E-01 | 1 | FNDC3A | 8.57E-01 | 1 |
| RPF1 | 5.79E-01 | 1 | ADAMTS12 | 8.57E-01 | 1 |
| SYT3 | 5.79E-01 | 1 | TTLL4 | 8.57E-01 | 1 |
| PCCA | 5.80E-01 | 1 | SLC8A2 | 8.57E-01 | 1 |
| NLRP7 | 5.80E-01 | 1 | ZSWIM6 | 8.57E-01 | 1 |
| LPIN3 | 5.80E-01 | 1 | FAM71E2 | 8.58E-01 | 1 |
| CNTN6 | 5.80E-01 | 1 | AFF3 | 8.58E-01 | 1 |
| ACSF2 | 5.80E-01 | 1 | PTCH1 | 8.58E-01 | 1 |
| SLC6A17 | 5.80E-01 | 1 | COL3A1 | 8.59E-01 | 1 |
| PRRC1 | 5.80E-01 | 1 | PTPRN | 8.59E-01 | 1 |
| SERPINB2 | 5.80E-01 | 1 | PCDH11X | 8.59E-01 | 1 |
| GJB6 | 5.80E-01 | 1 | GNAS | 8.59E-01 | 1 |
| CTTNBP2 | 5.80E-01 | 1 | ABCC8 | 8.59E-01 | 1 |
| PRSS35 | 5.80E-01 | 1 | FAM65A | 8.59E-01 | 1 |
| TSFM | 5.80E-01 | 1 | CSMD3 | 8.59E-01 | 1 |
| NIN | 5.80E-01 | 1 | MAP4K4 | 8.59E-01 | 1 |
| CPT1A | 5.80E-01 | 1 | OR6P1 | 8.59E-01 | 1 |
| TRIM68 | 5.80E-01 | 1 | AKR1C3 | 8.60E-01 | 1 |
| NCAPH2 | 5.81E-01 | 1 | PER3 | 8.60E-01 | 1 |
| C22orf29 | 5.81E-01 | 1 | ITPR2 | 8.60E-01 | 1 |
| OR2T3 | 5.81E-01 | 1 | TRDN | 8.61E-01 | 1 |
| CSK | 5.81E-01 | 1 | FAM111B | 8.61E-01 | 1 |
| DDX56 | 5.81E-01 | 1 | KDELR1 | 8.61E-01 | 1 |
| OR7A5 | 5.81E-01 | 1 | STK11IP | 8.61E-01 | 1 |
| ZNF304 | 5.81E-01 | 1 | LRP2 | 8.61E-01 | 1 |
| ACP2 | 5.81E-01 | 1 | LYST | 8.62E-01 | 1 |
| IK | 5.81E-01 | 1 | TNS1 | 8.62E-01 | 1 |
| GAB4 | 5.81E-01 | 1 | ATP2C2 | 8.62E-01 | 1 |
| GRM1 | 5.81E-01 | 1 | GREB1L | 8.62E-01 | 1 |
| CSNK1G1 | 5.81E-01 | 1 | CCDC73 | 8.63E-01 | 1 |
| MATN3 | 5.81E-01 | 1 | TACC2 | 8.64E-01 | 1 |
| MBIP | 5.82E-01 | 1 | PARP4 | 8.64E-01 | 1 |
| OR4K13 | 5.82E-01 | 1 | PGAP1 | 8.64E-01 | 1 |
| PTPRC | 5.82E-01 | 1 | PPL | 8.65E-01 | 1 |
| CHGA | 5.82E-01 | 1 | TRPM7 | 8.65E-01 | 1 |
| OTOA | 5.82E-01 | 1 | FAM179B | 8.65E-01 | 1 |
| CABIN1 | 5.82E-01 | 1 | SIN3B | 8.66E-01 | 1 |
| RECQL4 | 5.82E-01 | 1 | ATG2B | 8.66E-01 | 1 |
| GFI1 | 5.82E-01 | 1 | SYDE2 | 8.66E-01 | 1 |
| LRRC40 | 5.82E-01 | 1 | ADAM29 | 8.67E-01 | 1 |
| LY9 | 5.82E-01 | 1 | MGA | 8.67E-01 | 1 |

| GPR34 | 5.83E-01 | 1 | COL4A2 | 8.67E-01 | 1 |
| --- | --- | --- | --- | --- | --- |
| NOD2 | 5.83E-01 | 1 | PLXNA3 | 8.68E-01 | 1 |
| NCK2 | 5.83E-01 | 1 | GIGYF2 | 8.69E-01 | 1 |
| NMT1 | 5.83E-01 | 1 | ZNF804B | 8.69E-01 | 1 |
| CNTN2 | 5.83E-01 | 1 | LRRCC1 | 8.70E-01 | 1 |
| ATRIP | 5.83E-01 | 1 | KCNH3 | 8.71E-01 | 1 |
| BCL9 | 5.83E-01 | 1 | LRRN1 | 8.71E-01 | 1 |
| PKN2 | 5.83E-01 | 1 | IFNA4 | 8.72E-01 | 1 |
| KCNJ12 | 5.83E-01 | 1 | VWF | 8.72E-01 | 1 |
| LINGO3 | 5.83E-01 | 1 | NPHP3 | 8.72E-01 | 1 |
| NR2F1 | 5.83E-01 | 1 | A2ML1 | 8.73E-01 | 1 |
| SIGLEC1 | 5.83E-01 | 1 | SCML2 | 8.73E-01 | 1 |
| ETS2 | 5.83E-01 | 1 | MYH13 | 8.73E-01 | 1 |
| ANO6 | 5.84E-01 | 1 | TRPC3 | 8.74E-01 | 1 |
| ADNP2 | 5.84E-01 | 1 | INTS1 | 8.74E-01 | 1 |
| CAGE1 | 5.84E-01 | 1 | ZNF582 | 8.74E-01 | 1 |
| AMZ1 | 5.84E-01 | 1 | KIAA0430 | 8.74E-01 | 1 |
| PCDHB7 | 5.84E-01 | 1 | MECOM | 8.74E-01 | 1 |
| ATAD3B | 5.84E-01 | 1 | SCN10A | 8.75E-01 | 1 |
| CELSR3 | 5.84E-01 | 1 | KIAA0100 | 8.76E-01 | 1 |
| GSTCD | 5.84E-01 | 1 | NBEAL2 | 8.77E-01 | 1 |
| GYS2 | 5.84E-01 | 1 | PMFBP1 | 8.78E-01 | 1 |
| GLYATL2 | 5.84E-01 | 1 | SUSD2 | 8.78E-01 | 1 |
| DTNB | 5.84E-01 | 1 | RPS6KA6 | 8.79E-01 | 1 |
| FAM46A | 5.85E-01 | 1 | HAS2 | 8.79E-01 | 1 |
| KRT33A | 5.85E-01 | 1 | KIF14 | 8.79E-01 | 1 |
| RANBP3L | 5.85E-01 | 1 | NUP205 | 8.80E-01 | 1 |
| PPP2R2C | 5.85E-01 | 1 | ARHGAP30 | 8.81E-01 | 1 |
| FHAD1 | 5.85E-01 | 1 | DSG1 | 8.81E-01 | 1 |
| CCDC68 | 5.85E-01 | 1 | POM121L12 | 8.82E-01 | 1 |
| CTNNBL1 | 5.85E-01 | 1 | SEMA3D | 8.82E-01 | 1 |
| CABLES1 | 5.85E-01 | 1 | ALPK2 | 8.82E-01 | 1 |
| APAF1 | 5.85E-01 | 1 | VWA3A | 8.82E-01 | 1 |
| ADH1C | 5.85E-01 | 1 | TMEM131 | 8.82E-01 | 1 |
| CDH8 | 5.85E-01 | 1 | AKAP6 | 8.82E-01 | 1 |
| IL1RAPL1 | 5.86E-01 | 1 | NAV2 | 8.83E-01 | 1 |
| VPS4A | 5.86E-01 | 1 | GPC6 | 8.83E-01 | 1 |
| COL9A3 | 5.86E-01 | 1 | KIAA0753 | 8.84E-01 | 1 |
| DOK3 | 5.86E-01 | 1 | PALLD | 8.84E-01 | 1 |
| SYTL1 | 5.86E-01 | 1 | GRIN2A | 8.84E-01 | 1 |
| TPRN | 5.86E-01 | 1 | LRRTM1 | 8.85E-01 | 1 |
| DMRT2 | 5.86E-01 | 1 | PMVK | 8.85E-01 | 1 |
| CRHR2 | 5.86E-01 | 1 | FLNB | 8.85E-01 | 1 |
| CAPZA3 | 5.86E-01 | 1 | SEL1L | 8.86E-01 | 1 |
| GLI4 | 5.86E-01 | 1 | VPS13B | 8.86E-01 | 1 |
| PHYHIPL | 5.86E-01 | 1 | ZBBX | 8.86E-01 | 1 |
| TRDN | 5.86E-01 | 1 | EPB41L3 | 8.86E-01 | 1 |
| HARS | 5.86E-01 | 1 | HMGXB3 | 8.87E-01 | 1 |
| ZBTB7C | 5.86E-01 | 1 | SIPA1L3 | 8.88E-01 | 1 |
| SLC27A1 | 5.86E-01 | 1 | MDGA2 | 8.88E-01 | 1 |
| WNK4 | 5.87E-01 | 1 | FOLH1 | 8.88E-01 | 1 |
| MEGF11 | 5.87E-01 | 1 | ITGAM | 8.88E-01 | 1 |
| OR10H1 | 5.87E-01 | 1 | TTN | 8.88E-01 | 1 |

| UNC5A | 5.87E-01 | 1 | ZNF608 | 8.89E-01 | 1 |
| --- | --- | --- | --- | --- | --- |
| FNTA | 5.87E-01 | 1 | CPAMD8 | 8.89E-01 | 1 |
| DZIP3 | 5.87E-01 | 1 | ASCC3 | 8.89E-01 | 1 |
| DPYS | 5.87E-01 | 1 | KIF1C | 8.90E-01 | 1 |
| MAN2A1 | 5.87E-01 | 1 | ADCY10 | 8.91E-01 | 1 |
| KIAA2018 | 5.87E-01 | 1 | LYPD3 | 8.91E-01 | 1 |
| FPR2 | 5.87E-01 | 1 | MYO1C | 8.93E-01 | 1 |
| CARD6 | 5.87E-01 | 1 | CACNA1A | 8.94E-01 | 1 |
| CHRND | 5.87E-01 | 1 | TRPC6 | 8.94E-01 | 1 |
| COIL | 5.87E-01 | 1 | EPHA6 | 8.94E-01 | 1 |
| TBC1D22 | 5.88E-01 | 1 | SYNM | 8.94E-01 | 1 |
| C17orf74 | 5.88E-01 | 1 | SIN3A | 8.95E-01 | 1 |
| DSCAML1 | 5.88E-01 | 1 | PCDHGA6 | 8.95E-01 | 1 |
| USP40 | 5.88E-01 | 1 | STIL | 8.95E-01 | 1 |
| HERPUD2 | 5.88E-01 | 1 | PREX1 | 8.95E-01 | 1 |
| ANKRD13 | 5.88E-01 | 1 | NINL | 8.95E-01 | 1 |
| NRM | 5.88E-01 | 1 | BCL9 | 8.95E-01 | 1 |
| TRAF5 | 5.88E-01 | 1 | ATAD5 | 8.96E-01 | 1 |
| UBA1 | 5.88E-01 | 1 | DYNC2H1 | 8.96E-01 | 1 |
| PPP3CA | 5.89E-01 | 1 | LPIN3 | 8.96E-01 | 1 |
| MUC6 | 5.89E-01 | 1 | DNAH2 | 8.97E-01 | 1 |
| POTEM | 5.89E-01 | 1 | RUNX1T1 | 8.97E-01 | 1 |
| SP1 | 5.89E-01 | 1 | STON1-GT | 8.97E-01 | 1 |
| XYLT1 | 5.89E-01 | 1 | CDH13 | 8.98E-01 | 1 |
| RBPJ | 5.89E-01 | 1 | ADAMTS8 | 8.98E-01 | 1 |
| CAMK1G | 5.89E-01 | 1 | KIF1A | 8.98E-01 | 1 |
| CD22 | 5.89E-01 | 1 | PTPRS | 8.98E-01 | 1 |
| UXS1 | 5.89E-01 | 1 | CAND1 | 8.98E-01 | 1 |
| ICAM5 | 5.89E-01 | 1 | CYP4A11 | 8.98E-01 | 1 |
| SP140L | 5.89E-01 | 1 | COL22A1 | 8.99E-01 | 1 |
| NCOA4 | 5.89E-01 | 1 | C10orf71 | 8.99E-01 | 1 |
| CTNND1 | 5.89E-01 | 1 | PLXNC1 | 9.00E-01 | 1 |
| TNFRSF11 | 5.90E-01 | 1 | SIPA1L2 | 9.00E-01 | 1 |
| LCTL | 5.90E-01 | 1 | EPHA10 | 9.01E-01 | 1 |
| SLC27A4 | 5.90E-01 | 1 | MEGF8 | 9.01E-01 | 1 |
| PRAMEF1 | 5.90E-01 | 1 | CTNNA2 | 9.01E-01 | 1 |
| KHNYN | 5.90E-01 | 1 | MRC1 | 9.02E-01 | 1 |
| TRPM4 | 5.91E-01 | 1 | TAF2 | 9.02E-01 | 1 |
| PKP1 | 5.91E-01 | 1 | ZFPM2 | 9.07E-01 | 1 |
| PLCD4 | 5.91E-01 | 1 | SYNE1 | 9.07E-01 | 1 |
| NSUN7 | 5.91E-01 | 1 | PKD1L1 | 9.08E-01 | 1 |
| RGS20 | 5.91E-01 | 1 | TG | 9.08E-01 | 1 |
| PCDHGB3 | 5.91E-01 | 1 | GLI2 | 9.09E-01 | 1 |
| PRAMEF2 | 5.91E-01 | 1 | PRKD1 | 9.09E-01 | 1 |
| KLHL28 | 5.91E-01 | 1 | TNNI3K | 9.09E-01 | 1 |
| PABPC3 | 5.92E-01 | 1 | CNTN1 | 9.09E-01 | 1 |
| PPEF2 | 5.92E-01 | 1 | MYH7B | 9.09E-01 | 1 |
| OR8J1 | 5.92E-01 | 1 | RANBP2 | 9.09E-01 | 1 |
| DHX16 | 5.92E-01 | 1 | MADD | 9.10E-01 | 1 |
| OVGP1 | 5.92E-01 | 1 | MYH1 | 9.11E-01 | 1 |
| DICER1 | 5.92E-01 | 1 | PARP14 | 9.12E-01 | 1 |
| SLC6A11 | 5.92E-01 | 1 | ZNF189 | 9.12E-01 | 1 |
| COL9A1 | 5.92E-01 | 1 | ARHGAP39 | 9.13E-01 | 1 |

| AREG | 5.93E-01 | 1 | PSME4 | 9.13E-01 | 1 |
| --- | --- | --- | --- | --- | --- |
| TTC39C | 5.93E-01 | 1 | ATP1A3 | 9.13E-01 | 1 |
| OR14A16 | 5.93E-01 | 1 | CDC42BPB | 9.14E-01 | 1 |
| RPRD2 | 5.93E-01 | 1 | NOTCH4 | 9.14E-01 | 1 |
| DMRT3 | 5.93E-01 | 1 | ZNF208 | 9.15E-01 | 1 |
| CCDC7 | 5.93E-01 | 1 | FYCO1 | 9.15E-01 | 1 |
| ELL2 | 5.93E-01 | 1 | INPPL1 | 9.15E-01 | 1 |
| PRAMEF4 | 5.93E-01 | 1 | ZMYM2 | 9.16E-01 | 1 |
| SH3BP2 | 5.93E-01 | 1 | ZC3H7A | 9.16E-01 | 1 |
| MIER1 | 5.93E-01 | 1 | AIFM1 | 9.16E-01 | 1 |
| HLX | 5.94E-01 | 1 | MAN2A2 | 9.16E-01 | 1 |
| ZNF480 | 5.94E-01 | 1 | DACH1 | 9.17E-01 | 1 |
| SERPINI2 | 5.94E-01 | 1 | AQR | 9.17E-01 | 1 |
| CBFA2T3 | 5.94E-01 | 1 | ANKRD30B | 9.17E-01 | 1 |
| MBOAT2 | 5.94E-01 | 1 | TMEM2 | 9.18E-01 | 1 |
| RGS22 | 5.94E-01 | 1 | TEK | 9.19E-01 | 1 |
| ITGBL1 | 5.94E-01 | 1 | KIF13A | 9.19E-01 | 1 |
| EDAR | 5.94E-01 | 1 | ITPR3 | 9.19E-01 | 1 |
| CLEC18B | 5.94E-01 | 1 | SPTBN5 | 9.19E-01 | 1 |
| C16orf78 | 5.94E-01 | 1 | CELSR3 | 9.20E-01 | 1 |
| CCNB1 | 5.94E-01 | 1 | JARID2 | 9.20E-01 | 1 |
| XIRP2 | 5.94E-01 | 1 | CACNA1S | 9.20E-01 | 1 |
| UNKL | 5.94E-01 | 1 | SCN8A | 9.21E-01 | 1 |
| ITGA11 | 5.94E-01 | 1 | EXPH5 | 9.21E-01 | 1 |
| RPS6KA6 | 5.94E-01 | 1 | CDH6 | 9.21E-01 | 1 |
| GRIN2A | 5.94E-01 | 1 | RIMS2 | 9.21E-01 | 1 |
| ATF6 | 5.94E-01 | 1 | BNC2 | 9.22E-01 | 1 |
| C10orf12 | 5.95E-01 | 1 | CCDC80 | 9.23E-01 | 1 |
| SSTR4 | 5.95E-01 | 1 | ERCC6 | 9.23E-01 | 1 |
| UGT1A4 | 5.95E-01 | 1 | TRPM4 | 9.23E-01 | 1 |
| TLE4 | 5.95E-01 | 1 | VPS13A | 9.24E-01 | 1 |
| MVP | 5.95E-01 | 1 | LRRTM4 | 9.24E-01 | 1 |
| PPFIA3 | 5.95E-01 | 1 | RPS6KC1 | 9.24E-01 | 1 |
| OR2T2 | 5.95E-01 | 1 | SIGLEC1 | 9.24E-01 | 1 |
| IL10 | 5.95E-01 | 1 | ATP8B3 | 9.24E-01 | 1 |
| CRY1 | 5.96E-01 | 1 | MUC16 | 9.24E-01 | 1 |
| WDR25 | 5.96E-01 | 1 | GRID2 | 9.25E-01 | 1 |
| SLC3A2 | 5.96E-01 | 1 | KIAA1614 | 9.25E-01 | 1 |
| SLC8A1 | 5.96E-01 | 1 | UGGT2 | 9.25E-01 | 1 |
| GOLGA1 | 5.96E-01 | 1 | ADAMTS2 | 9.25E-01 | 1 |
| PLEKHA5 | 5.96E-01 | 1 | PHIP | 9.25E-01 | 1 |
| CNTLN | 5.96E-01 | 1 | DOPEY1 | 9.26E-01 | 1 |
| UGT1A3 | 5.96E-01 | 1 | ALDH1L1 | 9.26E-01 | 1 |
| SNUPN | 5.96E-01 | 1 | COL24A1 | 9.27E-01 | 1 |
| WIPF1 | 5.96E-01 | 1 | TET1 | 9.27E-01 | 1 |
| IKZF4 | 5.96E-01 | 1 | FNDC1 | 9.27E-01 | 1 |
| LRRC45 | 5.96E-01 | 1 | MYH14 | 9.27E-01 | 1 |
| CIR1 | 5.96E-01 | 1 | LRP6 | 9.28E-01 | 1 |
| C9 | 5.97E-01 | 1 | UHRF1BP1 | 9.28E-01 | 1 |
| SPATA17 | 5.97E-01 | 1 | COL4A4 | 9.29E-01 | 1 |
| OR2AG1 | 5.97E-01 | 1 | CNTNAP4 | 9.29E-01 | 1 |
| ADCY9 | 5.97E-01 | 1 | WISP2 | 9.30E-01 | 1 |
| MARK3 | 5.97E-01 | 1 | SNRNP200 | 9.30E-01 | 1 |

| COL6A5 | 5.97E-01 | 1 | CLASP1 | 9.32E-01 | 1 |
| --- | --- | --- | --- | --- | --- |
| PYGO2 | 5.97E-01 | 1 | MIA3 | 9.32E-01 | 1 |
| ZKSCAN4 | 5.97E-01 | 1 | UBE3A | 9.32E-01 | 1 |
| SLC2A11 | 5.97E-01 | 1 | ZAN | 9.33E-01 | 1 |
| CSF1 | 5.97E-01 | 1 | NBAS | 9.33E-01 | 1 |
| RP1L1 | 5.97E-01 | 1 | USP16 | 9.33E-01 | 1 |
| HKR1 | 5.97E-01 | 1 | PLCE1 | 9.35E-01 | 1 |
| SSX2IP | 5.98E-01 | 1 | SDK1 | 9.35E-01 | 1 |
| GTF3C3 | 5.98E-01 | 1 | RGPD8 | 9.36E-01 | 1 |
| OR2L2 | 5.98E-01 | 1 | ZNF658 | 9.37E-01 | 1 |
| OR5W2 | 5.98E-01 | 1 | ST6GAL2 | 9.37E-01 | 1 |
| IMPA1 | 5.98E-01 | 1 | DOPEY2 | 9.37E-01 | 1 |
| KIF5A | 5.98E-01 | 1 | PRDM2 | 9.38E-01 | 1 |
| OR6C76 | 5.98E-01 | 1 | ZFC3H1 | 9.38E-01 | 1 |
| STRBP | 5.98E-01 | 1 | CHD9 | 9.38E-01 | 1 |
| HR | 5.98E-01 | 1 | MYH3 | 9.39E-01 | 1 |
| ARHGAP2 | 5.98E-01 | 1 | CDH8 | 9.39E-01 | 1 |
| MALT1 | 5.98E-01 | 1 | TNR | 9.40E-01 | 1 |
| AQP4 | 5.99E-01 | 1 | QRICH2 | 9.40E-01 | 1 |
| TMF1 | 5.99E-01 | 1 | INO80 | 9.40E-01 | 1 |
| TRMT12 | 5.99E-01 | 1 | CADPS2 | 9.40E-01 | 1 |
| PHRF1 | 5.99E-01 | 1 | MAP1A | 9.40E-01 | 1 |
| ZFP82 | 5.99E-01 | 1 | ZNF462 | 9.41E-01 | 1 |
| ZSCAN5A | 5.99E-01 | 1 | SETD5 | 9.41E-01 | 1 |
| TOP3B | 5.99E-01 | 1 | ZZEF1 | 9.41E-01 | 1 |
| RPS6KB2 | 5.99E-01 | 1 | NELL1 | 9.41E-01 | 1 |
| GPR158 | 5.99E-01 | 1 | ROBO3 | 9.41E-01 | 1 |
| NTRK1 | 6.00E-01 | 1 | MED12 | 9.42E-01 | 1 |
| CACNG3 | 6.00E-01 | 1 | OR5T2 | 9.42E-01 | 1 |
| NLRC3 | 6.00E-01 | 1 | KCNH4 | 9.42E-01 | 1 |
| PCDHGB7 | 6.00E-01 | 1 | DLC1 | 9.43E-01 | 1 |
| MPPED2 | 6.00E-01 | 1 | ACSS3 | 9.44E-01 | 1 |
| WWC3 | 6.00E-01 | 1 | PCNT | 9.45E-01 | 1 |
| TGFBR1 | 6.00E-01 | 1 | SVIL | 9.45E-01 | 1 |
| PRAMEF2 | 6.01E-01 | 1 | KIAA0226 | 9.45E-01 | 1 |
| PSMD2 | 6.01E-01 | 1 | C4B | 9.45E-01 | 1 |
| WDR5 | 6.01E-01 | 1 | POLE | 9.45E-01 | 1 |
| GOLGA6B | 6.01E-01 | 1 | EML6 | 9.46E-01 | 1 |
| OR6C70 | 6.01E-01 | 1 | PRDM9 | 9.46E-01 | 1 |
| BICD2 | 6.01E-01 | 1 | NEURL4 | 9.46E-01 | 1 |
| PCDHGA1 | 6.02E-01 | 1 | ANK3 | 9.46E-01 | 1 |
| ETV3 | 6.02E-01 | 1 | ATG9A | 9.46E-01 | 1 |
| LILRA4 | 6.02E-01 | 1 | MAP2 | 9.47E-01 | 1 |
| FZD3 | 6.02E-01 | 1 | CACNA1I | 9.47E-01 | 1 |
| DNAH1 | 6.02E-01 | 1 | BAIAP2 | 9.47E-01 | 1 |
| VPS33A | 6.02E-01 | 1 | COBL | 9.47E-01 | 1 |
| OR2G3 | 6.02E-01 | 1 | VCAN | 9.48E-01 | 1 |
| FAM21A | 6.02E-01 | 1 | APC | 9.48E-01 | 1 |
| IARS2 | 6.02E-01 | 1 | RP1L1 | 9.48E-01 | 1 |
| ATG4C | 6.02E-01 | 1 | WNK4 | 9.49E-01 | 1 |
| LEMD2 | 6.02E-01 | 1 | TRRAP | 9.49E-01 | 1 |
| OR6C2 | 6.02E-01 | 1 | TRPC4 | 9.49E-01 | 1 |
| UBQLN1 | 6.02E-01 | 1 | GCLM | 9.49E-01 | 1 |

| TTLL6 | 6.03E-01 | 1 | BDP1 | 9.49E-01 | 1 |
| --- | --- | --- | --- | --- | --- |
| KCNC4 | 6.03E-01 | 1 | ROBO1 | 9.50E-01 | 1 |
| SYMPK | 6.03E-01 | 1 | HUWE1 | 9.50E-01 | 1 |
| CCAR1 | 6.03E-01 | 1 | USP34 | 9.51E-01 | 1 |
| AGFG1 | 6.03E-01 | 1 | DNAH11 | 9.51E-01 | 1 |
| SLC39A7 | 6.03E-01 | 1 | NOTCH1 | 9.51E-01 | 1 |
| EEF1D | 6.04E-01 | 1 | OR8J3 | 9.51E-01 | 1 |
| RAB37 | 6.04E-01 | 1 | SLITRK3 | 9.51E-01 | 1 |
| PCDHGB4 | 6.04E-01 | 1 | NAV3 | 9.52E-01 | 1 |
| CHRNA4 | 6.04E-01 | 1 | EPHA5 | 9.52E-01 | 1 |
| ZNF669 | 6.04E-01 | 1 | DOCK6 | 9.53E-01 | 1 |
| GATAD2B | 6.04E-01 | 1 | RIF1 | 9.53E-01 | 1 |
| ZNF787 | 6.04E-01 | 1 | LRBA | 9.54E-01 | 1 |
| SPAG6 | 6.04E-01 | 1 | GON4L | 9.54E-01 | 1 |
| ADSS | 6.04E-01 | 1 | PKP1 | 9.55E-01 | 1 |
| SLC44A2 | 6.04E-01 | 1 | NEBL | 9.55E-01 | 1 |
| ZNF638 | 6.04E-01 | 1 | RICTOR | 9.55E-01 | 1 |
| ARHGAP1 | 6.05E-01 | 1 | CITED2 | 9.56E-01 | 1 |
| KDR | 6.05E-01 | 1 | COL11A1 | 9.56E-01 | 1 |
| ZNF486 | 6.05E-01 | 1 | OR2T10 | 9.56E-01 | 1 |
| CRTC2 | 6.05E-01 | 1 | ANAPC1 | 9.57E-01 | 1 |
| PDCD4 | 6.05E-01 | 1 | C3 | 9.57E-01 | 1 |
| ZNF569 | 6.05E-01 | 1 | LRP1B | 9.57E-01 | 1 |
| AQP1 | 6.05E-01 | 1 | KCNT2 | 9.57E-01 | 1 |
| CASP8 | 6.05E-01 | 1 | RPAP1 | 9.57E-01 | 1 |
| TRIM6 | 6.05E-01 | 1 | PKD1 | 9.57E-01 | 1 |
| KANK3 | 6.05E-01 | 1 | ACACB | 9.58E-01 | 1 |
| GPCPD1 | 6.06E-01 | 1 | PDE4DIP | 9.59E-01 | 1 |
| DEAF1 | 6.06E-01 | 1 | PI4KA | 9.60E-01 | 1 |
| NPRL3 | 6.06E-01 | 1 | IGF2R | 9.60E-01 | 1 |
| ALDH1L2 | 6.06E-01 | 1 | XIRP1 | 9.60E-01 | 1 |
| CENPB | 6.06E-01 | 1 | SPTBN2 | 9.60E-01 | 1 |
| UGT1A1 | 6.06E-01 | 1 | USH2A | 9.60E-01 | 1 |
| OR52E2 | 6.06E-01 | 1 | HCCS | 9.61E-01 | 1 |
| DNASE2B | 6.06E-01 | 1 | CNTNAP5 | 9.61E-01 | 1 |
| SOS1 | 6.06E-01 | 1 | EIF4ENIF1 | 9.61E-01 | 1 |
| DMXL2 | 6.07E-01 | 1 | VPS13C | 9.62E-01 | 1 |
| ANGPTL1 | 6.07E-01 | 1 | GUCY1A2 | 9.62E-01 | 1 |
| ADD3 | 6.07E-01 | 1 | USP9Y | 9.63E-01 | 1 |
| AMPD3 | 6.07E-01 | 1 | CASP8AP2 | 9.63E-01 | 1 |
| FBXO42 | 6.07E-01 | 1 | PDZD2 | 9.63E-01 | 1 |
| ZNF773 | 6.07E-01 | 1 | CHD7 | 9.63E-01 | 1 |
| NCL | 6.08E-01 | 1 | MYH6 | 9.64E-01 | 1 |
| C4BPA | 6.08E-01 | 1 | NUMA1 | 9.64E-01 | 1 |
| SLC2A12 | 6.08E-01 | 1 | FLG2 | 9.64E-01 | 1 |
| TMEM201 | 6.08E-01 | 1 | LMO7 | 9.64E-01 | 1 |
| TGFBRAP | 6.08E-01 | 1 | PLEKHH2 | 9.64E-01 | 1 |
| HIF3A | 6.08E-01 | 1 | ANKRD30A | 9.65E-01 | 1 |
| TCHH | 6.08E-01 | 1 | C2orf16 | 9.65E-01 | 1 |
| GLE1 | 6.08E-01 | 1 | CMYA5 | 9.65E-01 | 1 |
| HLA-DMB | 6.08E-01 | 1 | ANKRD36 | 9.65E-01 | 1 |
| TMEM161 | 6.08E-01 | 1 | HSPG2 | 9.66E-01 | 1 |
| FHDC1 | 6.08E-01 | 1 | HEATR5A | 9.66E-01 | 1 |

| MSH5 | 6.09E-01 | 1 | SAR1B | 9.66E-01 | 1 |
| --- | --- | --- | --- | --- | --- |
| CHD1 | 6.09E-01 | 1 | THOC2 | 9.67E-01 | 1 |
| LRRC4C | 6.09E-01 | 1 | FBN3 | 9.67E-01 | 1 |
| ZNF737 | 6.09E-01 | 1 | KCNJ2 | 9.67E-01 | 1 |
| ELK3 | 6.09E-01 | 1 | ABCC11 | 9.68E-01 | 1 |
| SERPINA5 | 6.09E-01 | 1 | N4BP2 | 9.68E-01 | 1 |
| ZNF841 | 6.09E-01 | 1 | EP300 | 9.69E-01 | 1 |
| FGD2 | 6.09E-01 | 1 | FRMPD2 | 9.69E-01 | 1 |
| MKRN1 | 6.09E-01 | 1 | RPGR | 9.70E-01 | 1 |
| SLC24A1 | 6.10E-01 | 1 | GRM5 | 9.70E-01 | 1 |
| FDPS | 6.10E-01 | 1 | EYS | 9.71E-01 | 1 |
| POLR1A | 6.10E-01 | 1 | FSIP2 | 9.71E-01 | 1 |
| TCTE1 | 6.10E-01 | 1 | INADL | 9.71E-01 | 1 |
| COL28A1 | 6.10E-01 | 1 | MGAT4C | 9.71E-01 | 1 |
| OAS1 | 6.10E-01 | 1 | CEP192 | 9.71E-01 | 1 |
| GRIA2 | 6.10E-01 | 1 | SHANK1 | 9.71E-01 | 1 |
| IRAK4 | 6.10E-01 | 1 | DOCK1 | 9.72E-01 | 1 |
| GPR137C | 6.11E-01 | 1 | ZNF33A | 9.72E-01 | 1 |
| FAM69A | 6.11E-01 | 1 | TAAR1 | 9.72E-01 | 1 |
| STK3 | 6.11E-01 | 1 | NUP214 | 9.72E-01 | 1 |
| PBX1 | 6.11E-01 | 1 | ALS2 | 9.72E-01 | 1 |
| ZNF557 | 6.11E-01 | 1 | MAGEB6 | 9.72E-01 | 1 |
| TCTN1 | 6.11E-01 | 1 | MS4A3 | 9.72E-01 | 1 |
| ZNF683 | 6.11E-01 | 1 | MYH7 | 9.72E-01 | 1 |
| KRT74 | 6.11E-01 | 1 | CENPF | 9.72E-01 | 1 |
| ACVR2B | 6.12E-01 | 1 | TAF1 | 9.73E-01 | 1 |
| CHST3 | 6.12E-01 | 1 | MAPKBP1 | 9.73E-01 | 1 |
| MC5R | 6.12E-01 | 1 | FAT1 | 9.73E-01 | 1 |
| GPM6A | 6.12E-01 | 1 | COL6A3 | 9.73E-01 | 1 |
| CCR2 | 6.12E-01 | 1 | LRP1 | 9.73E-01 | 1 |
| MSL3 | 6.12E-01 | 1 | ZNF236 | 9.74E-01 | 1 |
| KCNK18 | 6.13E-01 | 1 | DMXL1 | 9.74E-01 | 1 |
| BLM | 6.13E-01 | 1 | ITPR1 | 9.74E-01 | 1 |
| GRIPAP1 | 6.13E-01 | 1 | DDX60 | 9.74E-01 | 1 |
| ADAM20 | 6.13E-01 | 1 | MYO3B | 9.75E-01 | 1 |
| KIF9 | 6.13E-01 | 1 | PDS5B | 9.75E-01 | 1 |
| RAPGEF2 | 6.13E-01 | 1 | FRMPD1 | 9.76E-01 | 1 |
| MC3R | 6.13E-01 | 1 | DMD | 9.76E-01 | 1 |
| RGS4 | 6.13E-01 | 1 | UBE3C | 9.76E-01 | 1 |
| SLC16A9 | 6.13E-01 | 1 | MTNR1A | 9.76E-01 | 1 |
| PCDHA10 | 6.13E-01 | 1 | DOCK4 | 9.77E-01 | 1 |
| VPS37A | 6.13E-01 | 1 | NPAS3 | 9.77E-01 | 1 |
| PTPN21 | 6.14E-01 | 1 | CNTN6 | 9.77E-01 | 1 |
| EHD2 | 6.14E-01 | 1 | NPEPPS | 9.77E-01 | 1 |
| KIAA1549 | 6.14E-01 | 1 | TBC1D8 | 9.77E-01 | 1 |
| SIRT3 | 6.14E-01 | 1 | BAZ1A | 9.77E-01 | 1 |
| MAGEL2 | 6.14E-01 | 1 | URB1 | 9.78E-01 | 1 |
| ANKLE1 | 6.14E-01 | 1 | CNTN3 | 9.78E-01 | 1 |
| SLC43A1 | 6.14E-01 | 1 | AKAP9 | 9.78E-01 | 1 |
| CHIT1 | 6.14E-01 | 1 | TDRD3 | 9.78E-01 | 1 |
| RBMXL1 | 6.15E-01 | 1 | VPS13D | 9.79E-01 | 1 |
| SFPQ | 6.15E-01 | 1 | TSHZ2 | 9.79E-01 | 1 |
| SHPRH | 6.15E-01 | 1 | PAPPA | 9.79E-01 | 1 |

| ST3GAL5 | 6.16E-01 | 1 | ARHGEF12 | 9.80E-01 | 1 |
| --- | --- | --- | --- | --- | --- |
| GRM4 | 6.16E-01 | 1 | RIMBP3 | 9.80E-01 | 1 |
| FBXO24 | 6.16E-01 | 1 | PLXNB1 | 9.80E-01 | 1 |
| SPATA13 | 6.16E-01 | 1 | CFH | 9.81E-01 | 1 |
| SERINC1 | 6.16E-01 | 1 | IGFN1 | 9.81E-01 | 1 |
| ZNF880 | 6.16E-01 | 1 | CDH23 | 9.81E-01 | 1 |
| KLHL8 | 6.17E-01 | 1 | MGLL | 9.81E-01 | 1 |
| SLC2A9 | 6.17E-01 | 1 | ASXL3 | 9.81E-01 | 1 |
| SERPINA1 | 6.17E-01 | 1 | TIAM1 | 9.81E-01 | 1 |
| NR4A3 | 6.17E-01 | 1 | PXDN | 9.81E-01 | 1 |
| TMPRSS2 | 6.17E-01 | 1 | TBR1 | 9.82E-01 | 1 |
| PPP1R13B | 6.17E-01 | 1 | ITSN1 | 9.82E-01 | 1 |
| XRN1 | 6.17E-01 | 1 | AHNAK2 | 9.82E-01 | 1 |
| NR1D1 | 6.17E-01 | 1 | UTRN | 9.82E-01 | 1 |
| USP32 | 6.17E-01 | 1 | ZNF644 | 9.82E-01 | 1 |
| MLLT4 | 6.17E-01 | 1 | CACNA2D3 | 9.83E-01 | 1 |
| WDR27 | 6.17E-01 | 1 | STK31 | 9.83E-01 | 1 |
| BMP6 | 6.18E-01 | 1 | MTUS2 | 9.83E-01 | 1 |
| CRTAM | 6.18E-01 | 1 | MTOR | 9.84E-01 | 1 |
| SYT10 | 6.18E-01 | 1 | PRUNE2 | 9.84E-01 | 1 |
| UACA | 6.18E-01 | 1 | ABCA13 | 9.84E-01 | 1 |
| PRLR | 6.18E-01 | 1 | GPR158 | 9.84E-01 | 1 |
| TGFB2 | 6.18E-01 | 1 | DNM2 | 9.84E-01 | 1 |
| WFIKKN2 | 6.18E-01 | 1 | PDZRN4 | 9.84E-01 | 1 |
| IKBKB | 6.18E-01 | 1 | ANK2 | 9.85E-01 | 1 |
| TMTC1 | 6.18E-01 | 1 | RTTN | 9.85E-01 | 1 |
| PAH | 6.19E-01 | 1 | MAGI2 | 9.85E-01 | 1 |
| YLPM1 | 6.19E-01 | 1 | NLRP5 | 9.86E-01 | 1 |
| SLC12A6 | 6.19E-01 | 1 | PPAT | 9.86E-01 | 1 |
| FAM160A | 6.19E-01 | 1 | NRG3 | 9.86E-01 | 1 |
| STEAP4 | 6.19E-01 | 1 | SAMD9L | 9.86E-01 | 1 |
| KPNB1 | 6.19E-01 | 1 | DNAH12 | 9.87E-01 | 1 |
| SLC25A40 | 6.19E-01 | 1 | BCL9L | 9.87E-01 | 1 |
| SLC22A24 | 6.19E-01 | 1 | MCM3AP | 9.87E-01 | 1 |
| OR51A2 | 6.19E-01 | 1 | CCDC63 | 9.87E-01 | 1 |
| PPP2R3B | 6.19E-01 | 1 | SI | 9.87E-01 | 1 |
| PDZD2 | 6.19E-01 | 1 | PAPPA2 | 9.87E-01 | 1 |
| PLCL2 | 6.19E-01 | 1 | NKTR | 9.87E-01 | 1 |
| SPATA6 | 6.19E-01 | 1 | PREX2 | 9.88E-01 | 1 |
| ZNF222 | 6.19E-01 | 1 | IQGAP2 | 9.88E-01 | 1 |
| NFS1 | 6.19E-01 | 1 | SACS | 9.88E-01 | 1 |
| CYP2C9 | 6.20E-01 | 1 | NUAK2 | 9.88E-01 | 1 |
| PPFIBP1 | 6.20E-01 | 1 | SCN9A | 9.88E-01 | 1 |
| ZNF440 | 6.20E-01 | 1 | ZNF273 | 9.89E-01 | 1 |
| SIPA1L1 | 6.20E-01 | 1 | MAST1 | 9.89E-01 | 1 |
| TNRC6B | 6.20E-01 | 1 | BAHCC1 | 9.89E-01 | 1 |
| INPP5D | 6.20E-01 | 1 | MYO18B | 9.89E-01 | 1 |
| LANCL3 | 6.21E-01 | 1 | FRYL | 9.89E-01 | 1 |
| DHX33 | 6.21E-01 | 1 | WNK1 | 9.89E-01 | 1 |
| ZC3H15 | 6.21E-01 | 1 | FRAS1 | 9.89E-01 | 1 |
| C12orf42 | 6.21E-01 | 1 | GCC2 | 9.90E-01 | 1 |
| CLIP4 | 6.21E-01 | 1 | SSPO | 9.90E-01 | 1 |
| ANKRD13 | 6.21E-01 | 1 | FCGBP | 9.90E-01 | 1 |

| FAM98A | 6.22E-01 | 1 | CNTLN | 9.90E-01 | 1 |
| --- | --- | --- | --- | --- | --- |
| ENTPD7 | 6.22E-01 | 1 | ROCK1 | 9.90E-01 | 1 |
| SCAF1 | 6.22E-01 | 1 | HMCN1 | 9.90E-01 | 1 |
| STRA6 | 6.22E-01 | 1 | CEP135 | 9.91E-01 | 1 |
| ELF1 | 6.22E-01 | 1 | PHF3 | 9.91E-01 | 1 |
| NEK10 | 6.22E-01 | 1 | MICAL3 | 9.92E-01 | 1 |
| SCN9A | 6.22E-01 | 1 | TAF1L | 9.92E-01 | 1 |
| DZIP1 | 6.23E-01 | 1 | SH3TC1 | 9.92E-01 | 1 |
| SMG1 | 6.23E-01 | 1 | KDM6B | 9.92E-01 | 1 |
| HM13 | 6.23E-01 | 1 | MKI67 | 9.92E-01 | 1 |
| SPATA19 | 6.23E-01 | 1 | PNPLA7 | 9.92E-01 | 1 |
| CACNA2D | 6.23E-01 | 1 | PTPRD | 9.92E-01 | 1 |
| FCHSD1 | 6.23E-01 | 1 | GRIN2B | 9.92E-01 | 1 |
| TLR3 | 6.23E-01 | 1 | TRANK1 | 9.93E-01 | 1 |
| DUSP6 | 6.24E-01 | 1 | HEPHL1 | 9.93E-01 | 1 |
| SELO | 6.24E-01 | 1 | LTBP1 | 9.93E-01 | 1 |
| TCF12 | 6.24E-01 | 1 | ACTN2 | 9.94E-01 | 1 |
| ACTR1B | 6.24E-01 | 1 | PLEKHA5 | 9.94E-01 | 1 |
| CCDC15 | 6.24E-01 | 1 | UTP20 | 9.94E-01 | 1 |
| CCDC74A | 6.24E-01 | 1 | DYNC1H1 | 9.94E-01 | 1 |
| SPATA22 | 6.24E-01 | 1 | PKP4 | 9.94E-01 | 1 |
| RFPL2 | 6.24E-01 | 1 | KDM5D | 9.94E-01 | 1 |
| SH2B1 | 6.24E-01 | 1 | TRPM5 | 9.94E-01 | 1 |
| PTK2B | 6.24E-01 | 1 | CDHR2 | 9.94E-01 | 1 |
| TIPARP | 6.24E-01 | 1 | LAMC3 | 9.94E-01 | 1 |
| CWF19L2 | 6.25E-01 | 1 | ZHX3 | 9.95E-01 | 1 |
| ATAD3A | 6.25E-01 | 1 | MEI1 | 9.95E-01 | 1 |
| MMP11 | 6.25E-01 | 1 | ARHGAP32 | 9.95E-01 | 1 |
| PGR | 6.25E-01 | 1 | PPP1R3A | 9.95E-01 | 1 |
| POM121L | 6.25E-01 | 1 | MED13L | 9.95E-01 | 1 |
| FUS | 6.25E-01 | 1 | FREM1 | 9.96E-01 | 1 |
| NECAB3 | 6.25E-01 | 1 | TNC | 9.96E-01 | 1 |
| NFKB2 | 6.25E-01 | 1 | DNAH17 | 9.96E-01 | 1 |
| SLFN13 | 6.25E-01 | 1 | DST | 9.96E-01 | 1 |
| OR56A4 | 6.26E-01 | 1 | DNAH9 | 9.96E-01 | 1 |
| PRDM16 | 6.26E-01 | 1 | USP9X | 9.96E-01 | 1 |
| OR51L1 | 6.26E-01 | 1 | GOLGB1 | 9.96E-01 | 1 |
| NPTX2 | 6.26E-01 | 1 | TEP1 | 9.96E-01 | 1 |
| NETO1 | 6.26E-01 | 1 | MAN2C1 | 9.96E-01 | 1 |
| OR13D1 | 6.26E-01 | 1 | WDR36 | 9.97E-01 | 1 |
| XRCC4 | 6.26E-01 | 1 | DLG1 | 9.97E-01 | 1 |
| CHD4 | 6.26E-01 | 1 | ASPM | 9.97E-01 | 1 |
| OR52A1 | 6.26E-01 | 1 | DNAH5 | 9.97E-01 | 1 |
| ZEB2 | 6.26E-01 | 1 | WDFY3 | 9.97E-01 | 1 |
| UTP6 | 6.26E-01 | 1 | FLNC | 9.97E-01 | 1 |
| ANLN | 6.26E-01 | 1 | JMJD7-PLA | 9.97E-01 | 1 |
| ZNF33A | 6.26E-01 | 1 | NBPF10 | 9.97E-01 | 1 |
| MYBPH | 6.26E-01 | 1 | AKAP11 | 9.97E-01 | 1 |
| LMNA | 6.27E-01 | 1 | CRB2 | 9.97E-01 | 1 |
| CLEC14A | 6.27E-01 | 1 | LAMA3 | 9.97E-01 | 1 |
| MYH11 | 6.27E-01 | 1 | RALGAPA1 | 9.97E-01 | 1 |
| CPOX | 6.27E-01 | 1 | LGI4 | 9.97E-01 | 1 |
| TMCO3 | 6.27E-01 | 1 | LAMA5 | 9.98E-01 | 1 |

| EPS15 | 6.27E-01 | 1 | FOLR1 | 9.98E-01 | 1 |
| --- | --- | --- | --- | --- | --- |
| PMFBP1 | 6.27E-01 | 1 | CNIH2 | 9.98E-01 | 1 |
| ZIC3 | 6.28E-01 | 1 | PHRF1 | 9.98E-01 | 1 |
| ADAMTS | 6.28E-01 | 1 | CEP250 | 9.98E-01 | 1 |
| CHRNB2 | 6.28E-01 | 1 | ASH1L | 9.98E-01 | 1 |
| NT5DC3 | 6.28E-01 | 1 | ATP11C | 9.98E-01 | 1 |
| ZBTB24 | 6.28E-01 | 1 | GPR179 | 9.98E-01 | 1 |
| ZNF777 | 6.28E-01 | 1 | SRRM2 | 9.98E-01 | 1 |
| RHBDF2 | 6.28E-01 | 1 | CHRNA2 | 9.98E-01 | 1 |
| ARID5B | 6.28E-01 | 1 | CIT | 9.98E-01 | 1 |
| ASB7 | 6.28E-01 | 1 | CACNA1F | 9.98E-01 | 1 |
| CD5L | 6.28E-01 | 1 | DOCK5 | 9.99E-01 | 1 |
| AGTR1 | 6.28E-01 | 1 | NCKAP5 | 9.99E-01 | 1 |
| ZNF408 | 6.29E-01 | 1 | PKD1L2 | 9.99E-01 | 1 |
| SLC9A1 | 6.29E-01 | 1 | ACIN1 | 9.99E-01 | 1 |
| SLC22A11 | 6.29E-01 | 1 | BASP1 | 9.99E-01 | 1 |
| HNF4A | 6.29E-01 | 1 | AFF4 | 9.99E-01 | 1 |
| HNRNPK | 6.29E-01 | 1 | RELN | 9.99E-01 | 1 |
| WDR24 | 6.29E-01 | 1 | COL14A1 | 9.99E-01 | 1 |
| ESPN | 6.29E-01 | 1 | ABCC2 | 9.99E-01 | 1 |
| ANK1 | 6.30E-01 | 1 | MYLK | 9.99E-01 | 1 |
| SLC25A46 | 6.30E-01 | 1 | MYO16 | 9.99E-01 | 1 |
| ATF2 | 6.30E-01 | 1 | KIDINS220 | 9.99E-01 | 1 |
| EXOC4 | 6.30E-01 | 1 | LRRC7 | 9.99E-01 | 1 |
| GSDMC | 6.30E-01 | 1 | NRXN1 | 9.99E-01 | 1 |
| PHACTR4 | 6.30E-01 | 1 | HERC1 | 9.99E-01 | 1 |
| SORL1 | 6.30E-01 | 1 | FBN1 | 9.99E-01 | 1 |
| PHF2 | 6.30E-01 | 1 | SFMBT2 | 9.99E-01 | 1 |
| EN1 | 6.30E-01 | 1 | KIAA1109 | 9.99E-01 | 1 |
| WBP11 | 6.30E-01 | 1 | CSMD1 | 9.99E-01 | 1 |
| PAPPA | 6.30E-01 | 1 | TNXB | 9.99E-01 | 1 |
| ITK | 6.31E-01 | 1 | RNF213 | 9.99E-01 | 1 |
| PXDNL | 6.31E-01 | 1 | KCNU1 | 9.99E-01 | 1 |
| FBXO40 | 6.31E-01 | 1 | DPP10 | 1.00E+00 | 1 |
| USP3 | 6.31E-01 | 1 | HYDIN | 1.00E+00 | 1 |
| PRR5-AR | 6.31E-01 | 1 | PCDHA10 | 1.00E+00 | 1 |
| OR4C12 | 6.31E-01 | 1 | TOP2B | 1.00E+00 | 1 |
| EXOC8 | 6.31E-01 | 1 | RFC1 | 1.00E+00 | 1 |
| TTC16 | 6.31E-01 | 1 | RCC2 | 1.00E+00 | 1 |
| GTPBP2 | 6.31E-01 | 1 | TECRL | 1.00E+00 | 1 |
| C14orf80 | 6.32E-01 | 1 | CACNA1B | 1.00E+00 | 1 |
| CTU2 | 6.32E-01 | 1 | NELL2 | 1.00E+00 | 1 |
| PCDHB9 | 6.32E-01 | 1 | STAB2 | 1.00E+00 | 1 |
| DCHS2 | 6.32E-01 | 1 | MDN1 | 1.00E+00 | 1 |
| ZNF544 | 6.32E-01 | 1 | ZFHX3 | 1.00E+00 | 1 |
| PRDM6 | 6.32E-01 | 1 | DNAH14 | 1.00E+00 | 1 |
| SLC12A4 | 6.32E-01 | 1 | FAT4 | 1.00E+00 | 1 |
| EIF2S3 | 6.32E-01 | 1 | HDAC7 | 1.00E+00 | 1 |
| VIPR1 | 6.32E-01 | 1 | FGF20 | 1.00E+00 | 1 |
| DDX1 | 6.32E-01 | 1 | TMEM92 | 1.00E+00 | 1 |
| SOX3 | 6.32E-01 | 1 | TSLP | 1.00E+00 | 1 |
| MAGEC3 | 6.32E-01 | 1 | ADAMTS20 | 1.00E+00 | 1 |
| GDPD1 | 6.32E-01 | 1 | HIVEP2 | 1.00E+00 | 1 |

| CD177 | 6.33E-01 | 1 | NEB | 1.00E+00 | 1 |
| --- | --- | --- | --- | --- | --- |
| GRIA3 | 6.33E-01 | 1 | PCBP3 | 1.00E+00 | 1 |
| PDHX | 6.33E-01 | 1 | VPS33A | 1.00E+00 | 1 |
| IGSF9B | 6.33E-01 | 1 | ZPBP | 1.00E+00 | 1 |
| IBTK | 6.33E-01 | 1 | OR8B8 | 1.00E+00 | 1 |
| ERCC3 | 6.33E-01 | 1 | MRPS33 | 1.00E+00 | 1 |
| UNC5B | 6.33E-01 | 1 | MRPL45 | 1.00E+00 | 1 |
| NPC1 | 6.33E-01 | 1 | KIF21A | 1.00E+00 | 1 |
| DYRK2 | 6.34E-01 | 1 | NLGN4X | 1.00E+00 | 1 |
| SLC45A1 | 6.34E-01 | 1 | CSNK1A1L | 1.00E+00 | 1 |
| GRIK1 | 6.34E-01 | 1 | IVL | 1.00E+00 | 1 |
| ZGPAT | 6.34E-01 | 1 | MUC4 | 1.00E+00 | 1 |
| PLXNA2 | 6.34E-01 | 1 | A1CF | 1 | 1 |
| SREBF2 | 6.34E-01 | 1 | A2BP1 | 1 | 1 |
| FHL1 | 6.34E-01 | 1 | A2LD1 | 1 | 1 |
| ZNF577 | 6.34E-01 | 1 | A4GALT | 1 | 1 |
| NEFL | 6.35E-01 | 1 | A4GNT | 1 | 1 |
| TFCP2L1 | 6.35E-01 | 1 | AAAS | 1 | 1 |
| ARHGAP1 | 6.35E-01 | 1 | AACS | 1 | 1 |
| STXBP4 | 6.35E-01 | 1 | AADACL2 | 1 | 1 |
| PPFIA4 | 6.35E-01 | 1 | AADACL3 | 1 | 1 |
| EZH1 | 6.35E-01 | 1 | AADACL4 | 1 | 1 |
| RPAP2 | 6.35E-01 | 1 | AADAT | 1 | 1 |
| DNMT3A | 6.35E-01 | 1 | AAGAB | 1 | 1 |
| UBP1 | 6.35E-01 | 1 | AAMP | 1 | 1 |
| CPT2 | 6.35E-01 | 1 | AARS2 | 1 | 1 |
| CES1 | 6.35E-01 | 1 | AARSD1 | 1 | 1 |
| ZNF813 | 6.35E-01 | 1 | AASDHPPT | 1 | 1 |
| FER1L5 | 6.35E-01 | 1 | AATF | 1 | 1 |
| PAMR1 | 6.35E-01 | 1 | ABAT | 1 | 1 |
| KIN | 6.36E-01 | 1 | ABCA10 | 1 | 1 |
| PTPDC1 | 6.36E-01 | 1 | ABCA2 | 1 | 1 |
| DET1 | 6.36E-01 | 1 | ABCA5 | 1 | 1 |
| ZBTB1 | 6.36E-01 | 1 | ABCA6 | 1 | 1 |
| KIAA1715 | 6.36E-01 | 1 | ABCA8 | 1 | 1 |
| TBCE | 6.36E-01 | 1 | ABCB10 | 1 | 1 |
| HMMR | 6.36E-01 | 1 | ABCB8 | 1 | 1 |
| LOXL1 | 6.36E-01 | 1 | ABCB9 | 1 | 1 |
| FECH | 6.36E-01 | 1 | ABCC12 | 1 | 1 |
| SND1 | 6.37E-01 | 1 | ABCC5 | 1 | 1 |
| DPY19L2 | 6.37E-01 | 1 | ABCC6 | 1 | 1 |
| CACNB2 | 6.37E-01 | 1 | ABCD3 | 1 | 1 |
| STK11IP | 6.37E-01 | 1 | ABCD4 | 1 | 1 |
| STX16 | 6.37E-01 | 1 | ABCF1 | 1 | 1 |
| FIP1L1 | 6.37E-01 | 1 | ABCF2 | 1 | 1 |
| VSTM1 | 6.37E-01 | 1 | ABCF3 | 1 | 1 |
| SYCP1 | 6.38E-01 | 1 | ABCG1 | 1 | 1 |
| ADAM21 | 6.38E-01 | 1 | ABCG2 | 1 | 1 |
| ASAP2 | 6.38E-01 | 1 | ABCG8 | 1 | 1 |
| STAT5A | 6.38E-01 | 1 | ABHD1 | 1 | 1 |
| IQSEC2 | 6.38E-01 | 1 | ABHD11 | 1 | 1 |
| CCDC33 | 6.38E-01 | 1 | ABHD12 | 1 | 1 |
| MAGEB18 | 6.38E-01 | 1 | ABHD12B | 1 | 1 |

| AIPL1 | 6.39E-01 | 1 | ABHD13 | 1 | 1 |
| --- | --- | --- | --- | --- | --- |
| NUP155 | 6.39E-01 | 1 | ABHD14A | 1 | 1 |
| ZNF474 | 6.40E-01 | 1 | ABHD14B | 1 | 1 |
| ILDR2 | 6.40E-01 | 1 | ABHD15 | 1 | 1 |
| WSCD1 | 6.40E-01 | 1 | ABHD2 | 1 | 1 |
| PACS2 | 6.40E-01 | 1 | ABHD5 | 1 | 1 |
| CTNNA3 | 6.40E-01 | 1 | ABHD6 | 1 | 1 |
| AKAP1 | 6.40E-01 | 1 | ABI1 | 1 | 1 |
| CHST8 | 6.40E-01 | 1 | ABI3 | 1 | 1 |
| PYGL | 6.40E-01 | 1 | ABL2 | 1 | 1 |
| KDM5C | 6.41E-01 | 1 | ABLIM1 | 1 | 1 |
| PASK | 6.41E-01 | 1 | ABLIM2 | 1 | 1 |
| C1orf116 | 6.41E-01 | 1 | ABLIM3 | 1 | 1 |
| DCAF4L2 | 6.41E-01 | 1 | ABO | 1 | 1 |
| SAMD4A | 6.41E-01 | 1 | ABP1 | 1 | 1 |
| ALG1 | 6.41E-01 | 1 | ABT1 | 1 | 1 |
| GBP5 | 6.41E-01 | 1 | ABTB2 | 1 | 1 |
| NR2C2 | 6.42E-01 | 1 | ACAA2 | 1 | 1 |
| ACTBL2 | 6.42E-01 | 1 | ACAD10 | 1 | 1 |
| NEURL1B | 6.42E-01 | 1 | ACAD9 | 1 | 1 |
| PHGDH | 6.42E-01 | 1 | ACADM | 1 | 1 |
| LRIG3 | 6.42E-01 | 1 | ACADS | 1 | 1 |
| KLHL34 | 6.42E-01 | 1 | ACADSB | 1 | 1 |
| DDX43 | 6.42E-01 | 1 | ACADVL | 1 | 1 |
| WDR1 | 6.42E-01 | 1 | ACAN | 1 | 1 |
| SHH | 6.42E-01 | 1 | ACAP3 | 1 | 1 |
| B4GALNT | 6.43E-01 | 1 | ACBD3 | 1 | 1 |
| UNC13A | 6.43E-01 | 1 | ACBD4 | 1 | 1 |
| ABI2 | 6.43E-01 | 1 | ACBD6 | 1 | 1 |
| CNGA1 | 6.43E-01 | 1 | ACBD7 | 1 | 1 |
| HEATR5B | 6.43E-01 | 1 | ACCN1 | 1 | 1 |
| CCDC116 | 6.43E-01 | 1 | ACCN2 | 1 | 1 |
| PLAT | 6.43E-01 | 1 | ACCN3 | 1 | 1 |
| ZSCAN22 | 6.43E-01 | 1 | ACCN4 | 1 | 1 |
| SIM1 | 6.44E-01 | 1 | ACCN5 | 1 | 1 |
| RASGRP3 | 6.44E-01 | 1 | ACCSL | 1 | 1 |
| LAP3 | 6.44E-01 | 1 | ACD | 1 | 1 |
| SLC10A5 | 6.44E-01 | 1 | ACE2 | 1 | 1 |
| ITGAD | 6.44E-01 | 1 | ACER1 | 1 | 1 |
| OR2M5 | 6.44E-01 | 1 | ACER2 | 1 | 1 |
| GALNT3 | 6.44E-01 | 1 | ACER3 | 1 | 1 |
| AVPR1B | 6.45E-01 | 1 | ACHE | 1 | 1 |
| TMEM200 | 6.45E-01 | 1 | ACLY | 1 | 1 |
| ARHGEF1 | 6.45E-01 | 1 | ACMSD | 1 | 1 |
| HNRNPH1 | 6.45E-01 | 1 | ACN9 | 1 | 1 |
| CDH9 | 6.45E-01 | 1 | ACO2 | 1 | 1 |
| RUNDC3B | 6.45E-01 | 1 | ACOT1 | 1 | 1 |
| MARCO | 6.46E-01 | 1 | ACOT11 | 1 | 1 |
| SLFN12L | 6.46E-01 | 1 | ACOT13 | 1 | 1 |
| TNFRSF11 | 6.46E-01 | 1 | ACOT2 | 1 | 1 |
| CAPS2 | 6.46E-01 | 1 | ACOT4 | 1 | 1 |
| SLC8A2 | 6.46E-01 | 1 | ACOT6 | 1 | 1 |
| PELP1 | 6.46E-01 | 1 | ACOT7 | 1 | 1 |

| CDC42BP | 6.46E-01 | 1 | ACOT8 | 1 | 1 |
| --- | --- | --- | --- | --- | --- |
| KIAA0195 | 6.46E-01 | 1 | ACOT9 | 1 | 1 |
| THBS3 | 6.46E-01 | 1 | ACOX1 | 1 | 1 |
| ITCH | 6.46E-01 | 1 | ACOX3 | 1 | 1 |
| ARMC8 | 6.46E-01 | 1 | ACOXL | 1 | 1 |
| SP140 | 6.46E-01 | 1 | ACP1 | 1 | 1 |
| SMCR8 | 6.47E-01 | 1 | ACP2 | 1 | 1 |
| ELF2 | 6.47E-01 | 1 | ACP5 | 1 | 1 |
| ZNF619 | 6.47E-01 | 1 | ACP6 | 1 | 1 |
| HTR1F | 6.47E-01 | 1 | ACPL2 | 1 | 1 |
| KCNT2 | 6.47E-01 | 1 | ACRC | 1 | 1 |
| ERN2 | 6.47E-01 | 1 | ACRV1 | 1 | 1 |
| MEIS1 | 6.47E-01 | 1 | ACSBG1 | 1 | 1 |
| SP8 | 6.47E-01 | 1 | ACSF2 | 1 | 1 |
| MCOLN3 | 6.47E-01 | 1 | ACSL3 | 1 | 1 |
| B3GALT5 | 6.47E-01 | 1 | ACSL4 | 1 | 1 |
| RBM28 | 6.47E-01 | 1 | ACSL5 | 1 | 1 |
| FPGT | 6.47E-01 | 1 | ACSL6 | 1 | 1 |
| ZNF512 | 6.47E-01 | 1 | ACSM1 | 1 | 1 |
| ESR2 | 6.48E-01 | 1 | ACSM2A | 1 | 1 |
| UGT2A1 | 6.48E-01 | 1 | ACSM3 | 1 | 1 |
| ZNF506 | 6.48E-01 | 1 | ACSM4 | 1 | 1 |
| SERBP1 | 6.48E-01 | 1 | ACSM5 | 1 | 1 |
| ANKZF1 | 6.48E-01 | 1 | ACSS1 | 1 | 1 |
| SPOCK1 | 6.48E-01 | 1 | ACSS2 | 1 | 1 |
| C12orf4 | 6.49E-01 | 1 | ACTA1 | 1 | 1 |
| LPPR3 | 6.49E-01 | 1 | ACTA2 | 1 | 1 |
| GDNF | 6.49E-01 | 1 | ACTB | 1 | 1 |
| ZNF611 | 6.49E-01 | 1 | ACTBL2 | 1 | 1 |
| FAM65B | 6.49E-01 | 1 | ACTC1 | 1 | 1 |
| PSD3 | 6.49E-01 | 1 | ACTG1 | 1 | 1 |
| KLC4 | 6.49E-01 | 1 | ACTG2 | 1 | 1 |
| TBC1D17 | 6.50E-01 | 1 | ACTL6B | 1 | 1 |
| RUSC1 | 6.50E-01 | 1 | ACTL7B | 1 | 1 |
| ZNF680 | 6.50E-01 | 1 | ACTL8 | 1 | 1 |
| HS3ST3B1 | 6.50E-01 | 1 | ACTL9 | 1 | 1 |
| PPP2R3C | 6.50E-01 | 1 | ACTN1 | 1 | 1 |
| ACAT1 | 6.50E-01 | 1 | ACTN3 | 1 | 1 |
| IMMT | 6.50E-01 | 1 | ACTN4 | 1 | 1 |
| TRIM3 | 6.50E-01 | 1 | ACTR1A | 1 | 1 |
| WASH1 | 6.50E-01 | 1 | ACTR1B | 1 | 1 |
| CWH43 | 6.50E-01 | 1 | ACTR2 | 1 | 1 |
| WDR47 | 6.50E-01 | 1 | ACTR3B | 1 | 1 |
| HMGCLL | 6.51E-01 | 1 | ACTR3C | 1 | 1 |
| OR5D16 | 6.51E-01 | 1 | ACTR5 | 1 | 1 |
| MAP7D2 | 6.51E-01 | 1 | ACTRT2 | 1 | 1 |
| MED12 | 6.51E-01 | 1 | ACVR1 | 1 | 1 |
| KIAA0141 | 6.51E-01 | 1 | ACVRL1 | 1 | 1 |
| DUSP16 | 6.51E-01 | 1 | ACY1 | 1 | 1 |
| PIKFYVE | 6.51E-01 | 1 | ACY3 | 1 | 1 |
| TRIM5 | 6.52E-01 | 1 | ACYP1 | 1 | 1 |
| RIPK2 | 6.52E-01 | 1 | ACYP2 | 1 | 1 |
| PIGR | 6.52E-01 | 1 | ADA | 1 | 1 |

| USP47 | 6.52E-01 | 1 | ADAL | 1 | 1 |
| --- | --- | --- | --- | --- | --- |
| WFS1 | 6.52E-01 | 1 | ADAM15 | 1 | 1 |
| XKR6 | 6.52E-01 | 1 | ADAM19 | 1 | 1 |
| C18orf8 | 6.52E-01 | 1 | ADAM20 | 1 | 1 |
| METTL13 | 6.52E-01 | 1 | ADAM28 | 1 | 1 |
| SCMH1 | 6.52E-01 | 1 | ADAM30 | 1 | 1 |
| SENP7 | 6.52E-01 | 1 | ADAM32 | 1 | 1 |
| NOS1AP | 6.52E-01 | 1 | ADAM33 | 1 | 1 |
| PAK7 | 6.52E-01 | 1 | ADAMDEC | 1 | 1 |
| CCT2 | 6.53E-01 | 1 | ADAMTS13 | 1 | 1 |
| MCM5 | 6.53E-01 | 1 | ADAMTS15 | 1 | 1 |
| HSPA12B | 6.53E-01 | 1 | ADAMTS17 | 1 | 1 |
| BARD1 | 6.53E-01 | 1 | ADAMTS4 | 1 | 1 |
| SASH1 | 6.53E-01 | 1 | ADAMTS5 | 1 | 1 |
| OR2M2 | 6.53E-01 | 1 | ADAMTSL | 1 | 1 |
| C2orf42 | 6.53E-01 | 1 | ADAP1 | 1 | 1 |
| SMCHD1 | 6.53E-01 | 1 | ADAP2 | 1 | 1 |
| PIAS3 | 6.53E-01 | 1 | ADAR | 1 | 1 |
| OPRK1 | 6.53E-01 | 1 | ADARB1 | 1 | 1 |
| NFKB1 | 6.53E-01 | 1 | ADAT1 | 1 | 1 |
| ERCC8 | 6.54E-01 | 1 | ADAT2 | 1 | 1 |
| CAMTA1 | 6.54E-01 | 1 | ADAT3 | 1 | 1 |
| BRD1 | 6.54E-01 | 1 | ADC | 1 | 1 |
| ZBTB5 | 6.54E-01 | 1 | ADCK1 | 1 | 1 |
| SMEK2 | 6.54E-01 | 1 | ADCK2 | 1 | 1 |
| WNK2 | 6.54E-01 | 1 | ADCK5 | 1 | 1 |
| DIP2B | 6.54E-01 | 1 | ADCY7 | 1 | 1 |
| BPTF | 6.55E-01 | 1 | ADCYAP1 | 1 | 1 |
| ANGPTL3 | 6.55E-01 | 1 | ADD3 | 1 | 1 |
| FAM200B | 6.55E-01 | 1 | ADH1C | 1 | 1 |
| CCDC66 | 6.55E-01 | 1 | ADH5 | 1 | 1 |
| KIFC3 | 6.55E-01 | 1 | ADH6 | 1 | 1 |
| RASSF10 | 6.55E-01 | 1 | ADH7 | 1 | 1 |
| PLCH2 | 6.55E-01 | 1 | ADI1 | 1 | 1 |
| COL3A1 | 6.55E-01 | 1 | ADIG | 1 | 1 |
| AMDHD1 | 6.55E-01 | 1 | ADIPOQ | 1 | 1 |
| SLC38A1 | 6.55E-01 | 1 | ADK | 1 | 1 |
| TMEM151 | 6.55E-01 | 1 | ADM | 1 | 1 |
| CGN | 6.55E-01 | 1 | ADM2 | 1 | 1 |
| RFX5 | 6.55E-01 | 1 | ADO | 1 | 1 |
| PPP1R16B | 6.55E-01 | 1 | ADORA1 | 1 | 1 |
| SHC2 | 6.55E-01 | 1 | ADPGK | 1 | 1 |
| STAT1 | 6.56E-01 | 1 | ADPRH | 1 | 1 |
| DNAH6 | 6.56E-01 | 1 | ADPRHL2 | 1 | 1 |
| IL1RL2 | 6.56E-01 | 1 | ADRA1A | 1 | 1 |
| ZNF630 | 6.56E-01 | 1 | ADRA1B | 1 | 1 |
| ZFYVE28 | 6.57E-01 | 1 | ADRA2A | 1 | 1 |
| TRIL | 6.57E-01 | 1 | ADRA2C | 1 | 1 |
| EPS8L1 | 6.57E-01 | 1 | ADRB1 | 1 | 1 |
| TTC3 | 6.57E-01 | 1 | ADRBK1 | 1 | 1 |
| KLHL2 | 6.57E-01 | 1 | ADRBK2 | 1 | 1 |
| EIF3E | 6.57E-01 | 1 | ADSS | 1 | 1 |
| COG7 | 6.57E-01 | 1 | ADSSL1 | 1 | 1 |

| KRT37 | 6.57E-01 | 1 | AEBP2 | 1 | 1 |
| --- | --- | --- | --- | --- | --- |
| ZCCHC14 | 6.58E-01 | 1 | AEN | 1 | 1 |
| ZNF710 | 6.58E-01 | 1 | AES | 1 | 1 |
| KRT71 | 6.58E-01 | 1 | AFAP1L2 | 1 | 1 |
| ZFP64 | 6.58E-01 | 1 | AFMID | 1 | 1 |
| LYST | 6.58E-01 | 1 | AFP | 1 | 1 |
| PIK3CB | 6.58E-01 | 1 | AG2 | 1 | 1 |
| SEMA3A | 6.58E-01 | 1 | AGAP11 | 1 | 1 |
| AKAP13 | 6.58E-01 | 1 | AGAP5 | 1 | 1 |
| FAM69C | 6.58E-01 | 1 | AGAP6 | 1 | 1 |
| HOXB7 | 6.58E-01 | 1 | AGAP7 | 1 | 1 |
| CHRNA5 | 6.58E-01 | 1 | AGAP8 | 1 | 1 |
| NUP98 | 6.59E-01 | 1 | AGBL1 | 1 | 1 |
| SLMAP | 6.59E-01 | 1 | AGBL4 | 1 | 1 |
| LTBP1 | 6.59E-01 | 1 | AGER | 1 | 1 |
| VPS33B | 6.59E-01 | 1 | AGFG2 | 1 | 1 |
| MUC5B | 6.59E-01 | 1 | AGGF1 | 1 | 1 |
| LRRC14B | 6.59E-01 | 1 | AGK | 1 | 1 |
| SLC40A1 | 6.59E-01 | 1 | AGMAT | 1 | 1 |
| B3GALTL | 6.59E-01 | 1 | AGPAT4 | 1 | 1 |
| COL10A1 | 6.60E-01 | 1 | AGPAT5 | 1 | 1 |
| UBR3 | 6.60E-01 | 1 | AGPAT6 | 1 | 1 |
| CORIN | 6.60E-01 | 1 | AGPHD1 | 1 | 1 |
| TBX3 | 6.60E-01 | 1 | AGR2 | 1 | 1 |
| IRX5 | 6.61E-01 | 1 | AGR3 | 1 | 1 |
| IRX6 | 6.61E-01 | 1 | AGT | 1 | 1 |
| PRPF39 | 6.61E-01 | 1 | AGTR1 | 1 | 1 |
| LETM2 | 6.61E-01 | 1 | AGTR2 | 1 | 1 |
| ZNF750 | 6.61E-01 | 1 | AGTRAP | 1 | 1 |
| ISL1 | 6.61E-01 | 1 | AGXT | 1 | 1 |
| SPTLC2 | 6.61E-01 | 1 | AGXT2 | 1 | 1 |
| ITGB7 | 6.61E-01 | 1 | AGXT2L1 | 1 | 1 |
| SRPK2 | 6.61E-01 | 1 | AGXT2L2 | 1 | 1 |
| NAA16 | 6.61E-01 | 1 | AHCY | 1 | 1 |
| ATMIN | 6.61E-01 | 1 | AHCYL1 | 1 | 1 |
| FBXO7 | 6.61E-01 | 1 | AHCYL2 | 1 | 1 |
| SYNPO2L | 6.61E-01 | 1 | AHI1 | 1 | 1 |
| MEIS2 | 6.61E-01 | 1 | AHRR | 1 | 1 |
| ALS2CL | 6.62E-01 | 1 | AHSP | 1 | 1 |
| AGGF1 | 6.62E-01 | 1 | AIDA | 1 | 1 |
| UNC93B1 | 6.62E-01 | 1 | AIF1 | 1 | 1 |
| C1orf101 | 6.62E-01 | 1 | AIF1L | 1 | 1 |
| TMCO4 | 6.62E-01 | 1 | AIFM2 | 1 | 1 |
| CHRM5 | 6.62E-01 | 1 | AIFM3 | 1 | 1 |
| ACSF3 | 6.62E-01 | 1 | AIG1 | 1 | 1 |
| HNRNPR | 6.62E-01 | 1 | AIMP1 | 1 | 1 |
| TNR | 6.63E-01 | 1 | AIMP2 | 1 | 1 |
| LRRTM3 | 6.63E-01 | 1 | AIP | 1 | 1 |
| CCNE1 | 6.63E-01 | 1 | AIPL1 | 1 | 1 |
| RPGRIP1 | 6.63E-01 | 1 | AK1 | 1 | 1 |
| MAST2 | 6.63E-01 | 1 | AK3 | 1 | 1 |
| ACSM4 | 6.63E-01 | 1 | AK3L1 | 1 | 1 |
| ZNF37A | 6.63E-01 | 1 | AK5 | 1 | 1 |

| DNTTIP2 | 6.63E-01 | 1 | AK7 | 1 | 1 |
| --- | --- | --- | --- | --- | --- |
| ATP6V1H | 6.63E-01 | 1 | AKAP14 | 1 | 1 |
| KIAA2022 | 6.63E-01 | 1 | AKAP2 | 1 | 1 |
| DDN | 6.63E-01 | 1 | AKAP5 | 1 | 1 |
| PKNOX2 | 6.63E-01 | 1 | AKAP7 | 1 | 1 |
| TPBG | 6.64E-01 | 1 | AKAP8 | 1 | 1 |
| UTP14A | 6.64E-01 | 1 | AKAP8L | 1 | 1 |
| ZNF776 | 6.64E-01 | 1 | AKD1 | 1 | 1 |
| BTBD9 | 6.64E-01 | 1 | AKIRIN1 | 1 | 1 |
| MGAT4A | 6.64E-01 | 1 | AKIRIN2 | 1 | 1 |
| HIRA | 6.64E-01 | 1 | AKR1A1 | 1 | 1 |
| MYBL1 | 6.64E-01 | 1 | AKR1B1 | 1 | 1 |
| LARP6 | 6.64E-01 | 1 | AKR1B10 | 1 | 1 |
| COL20A1 | 6.65E-01 | 1 | AKR1C2 | 1 | 1 |
| SEMA6C | 6.65E-01 | 1 | AKR1E2 | 1 | 1 |
| PCSK4 | 6.65E-01 | 1 | AKR7A2 | 1 | 1 |
| ACOT2 | 6.66E-01 | 1 | AKR7A3 | 1 | 1 |
| NSD1 | 6.66E-01 | 1 | AKT1 | 1 | 1 |
| AURKA | 6.66E-01 | 1 | AKT1S1 | 1 | 1 |
| GULP1 | 6.66E-01 | 1 | AKT2 | 1 | 1 |
| RNF112 | 6.66E-01 | 1 | ALAD | 1 | 1 |
| LMBRD2 | 6.66E-01 | 1 | ALAS2 | 1 | 1 |
| PALM3 | 6.66E-01 | 1 | ALCAM | 1 | 1 |
| NLRP6 | 6.66E-01 | 1 | ALDH1A1 | 1 | 1 |
| DACT1 | 6.66E-01 | 1 | ALDH1A3 | 1 | 1 |
| ZNF253 | 6.66E-01 | 1 | ALDH2 | 1 | 1 |
| C5orf34 | 6.66E-01 | 1 | ALDH3A2 | 1 | 1 |
| ANO9 | 6.67E-01 | 1 | ALDH3B1 | 1 | 1 |
| TGM7 | 6.67E-01 | 1 | ALDH3B2 | 1 | 1 |
| BNC1 | 6.67E-01 | 1 | ALDH5A1 | 1 | 1 |
| ITGA1 | 6.67E-01 | 1 | ALDH6A1 | 1 | 1 |
| PCDHA2 | 6.68E-01 | 1 | ALDH7A1 | 1 | 1 |
| THOC5 | 6.68E-01 | 1 | ALDH8A1 | 1 | 1 |
| SMTN | 6.68E-01 | 1 | ALDH9A1 | 1 | 1 |
| NEU1 | 6.68E-01 | 1 | ALDOA | 1 | 1 |
| CDKL3 | 6.68E-01 | 1 | ALDOB | 1 | 1 |
| HSPA6 | 6.68E-01 | 1 | ALDOC | 1 | 1 |
| ACOT4 | 6.68E-01 | 1 | ALG1 | 1 | 1 |
| ZNF442 | 6.68E-01 | 1 | ALG10 | 1 | 1 |
| TAS1R3 | 6.68E-01 | 1 | ALG10B | 1 | 1 |
| NAA25 | 6.68E-01 | 1 | ALG11 | 1 | 1 |
| ARMC4 | 6.69E-01 | 1 | ALG12 | 1 | 1 |
| SALL2 | 6.69E-01 | 1 | ALG13 | 1 | 1 |
| TRPV4 | 6.69E-01 | 1 | ALG14 | 1 | 1 |
| TASP1 | 6.69E-01 | 1 | ALG1L | 1 | 1 |
| CSGALNA | 6.70E-01 | 1 | ALG1L2 | 1 | 1 |
| ZNHIT6 | 6.70E-01 | 1 | ALG2 | 1 | 1 |
| RAG2 | 6.70E-01 | 1 | ALG6 | 1 | 1 |
| PRF1 | 6.70E-01 | 1 | ALG8 | 1 | 1 |
| PRKAG3 | 6.70E-01 | 1 | ALG9 | 1 | 1 |
| CCDC30 | 6.70E-01 | 1 | ALKBH1 | 1 | 1 |
| PCDH17 | 6.70E-01 | 1 | ALKBH2 | 1 | 1 |
| FAM83C | 6.70E-01 | 1 | ALKBH3 | 1 | 1 |

| AIRE | 6.70E-01 | 1 | ALKBH4 | 1 | 1 |
| --- | --- | --- | --- | --- | --- |
| LGMN | 6.70E-01 | 1 | ALKBH5 | 1 | 1 |
| ZNF335 | 6.71E-01 | 1 | ALKBH6 | 1 | 1 |
| THSD1 | 6.71E-01 | 1 | ALOX12 | 1 | 1 |
| TSSC1 | 6.71E-01 | 1 | ALOX12B | 1 | 1 |
| ZNF709 | 6.71E-01 | 1 | ALOX15 | 1 | 1 |
| TMEM63A | 6.72E-01 | 1 | ALOX5 | 1 | 1 |
| ARHGAP3 | 6.72E-01 | 1 | ALOX5AP | 1 | 1 |
| MYH6 | 6.72E-01 | 1 | ALOXE3 | 1 | 1 |
| ENTPD1 | 6.72E-01 | 1 | ALPK3 | 1 | 1 |
| COPA | 6.72E-01 | 1 | ALPL | 1 | 1 |
| ANO1 | 6.72E-01 | 1 | ALPPL2 | 1 | 1 |
| SLC27A5 | 6.72E-01 | 1 | ALS2CL | 1 | 1 |
| SYN2 | 6.72E-01 | 1 | ALS2CR11 | 1 | 1 |
| HSPA9 | 6.72E-01 | 1 | ALS2CR12 | 1 | 1 |
| DIAPH3 | 6.72E-01 | 1 | ALS2CR4 | 1 | 1 |
| ABCB8 | 6.73E-01 | 1 | ALS2CR8 | 1 | 1 |
| SH2B3 | 6.73E-01 | 1 | ALX3 | 1 | 1 |
| RALGAPA | 6.73E-01 | 1 | ALX4 | 1 | 1 |
| CPXCR1 | 6.73E-01 | 1 | AMAC1 | 1 | 1 |
| SLC7A3 | 6.73E-01 | 1 | AMAC1L2 | 1 | 1 |
| ZNF76 | 6.73E-01 | 1 | AMAC1L3 | 1 | 1 |
| KHSRP | 6.73E-01 | 1 | AMACR | 1 | 1 |
| TDRD9 | 6.73E-01 | 1 | AMBN | 1 | 1 |
| PHLDB3 | 6.74E-01 | 1 | AMBP | 1 | 1 |
| CEACAM | 6.74E-01 | 1 | AMD1 | 1 | 1 |
| KDM1B | 6.74E-01 | 1 | AMDHD2 | 1 | 1 |
| SLC19A3 | 6.74E-01 | 1 | AMELX | 1 | 1 |
| ZNF143 | 6.74E-01 | 1 | AMELY | 1 | 1 |
| DVL1 | 6.74E-01 | 1 | AMFR | 1 | 1 |
| ABCB11 | 6.74E-01 | 1 | AMH | 1 | 1 |
| DEPDC7 | 6.74E-01 | 1 | AMHR2 | 1 | 1 |
| IZUMO1 | 6.74E-01 | 1 | AMICA1 | 1 | 1 |
| GPR65 | 6.74E-01 | 1 | AMIGO1 | 1 | 1 |
| ABCD2 | 6.74E-01 | 1 | AMIGO3 | 1 | 1 |
| CAMTA2 | 6.75E-01 | 1 | AMMECR1 | 1 | 1 |
| CTCFL | 6.75E-01 | 1 | AMN | 1 | 1 |
| RNF43 | 6.75E-01 | 1 | AMN1 | 1 | 1 |
| KRBA2 | 6.75E-01 | 1 | AMOTL1 | 1 | 1 |
| QSOX2 | 6.76E-01 | 1 | AMPD3 | 1 | 1 |
| SLCO1C1 | 6.76E-01 | 1 | AMT | 1 | 1 |
| ANKRD27 | 6.76E-01 | 1 | AMTN | 1 | 1 |
| AGAP1 | 6.76E-01 | 1 | AMY1A | 1 | 1 |
| RAP1GAP | 6.76E-01 | 1 | AMY1B | 1 | 1 |
| MTMR2 | 6.76E-01 | 1 | AMY2B | 1 | 1 |
| KRT31 | 6.76E-01 | 1 | AMZ2 | 1 | 1 |
| TLL2 | 6.76E-01 | 1 | ANAPC10 | 1 | 1 |
| SIK3 | 6.76E-01 | 1 | ANAPC11 | 1 | 1 |
| KRT14 | 6.76E-01 | 1 | ANAPC13 | 1 | 1 |
| RNF180 | 6.76E-01 | 1 | ANAPC16 | 1 | 1 |
| LRIG2 | 6.76E-01 | 1 | ANAPC4 | 1 | 1 |
| RIOK3 | 6.76E-01 | 1 | ANAPC5 | 1 | 1 |
| ABHD13 | 6.76E-01 | 1 | ANAPC7 | 1 | 1 |

| HCRTR2 | 6.77E-01 | 1 | ANGEL1 | 1 | 1 |
| --- | --- | --- | --- | --- | --- |
| TBR1 | 6.77E-01 | 1 | ANGPT2 | 1 | 1 |
| AP3B1 | 6.77E-01 | 1 | ANGPTL1 | 1 | 1 |
| MYOM2 | 6.77E-01 | 1 | ANGPTL2 | 1 | 1 |
| KCND3 | 6.77E-01 | 1 | ANGPTL3 | 1 | 1 |
| GTF2H4 | 6.77E-01 | 1 | ANGPTL5 | 1 | 1 |
| NBPF6 | 6.77E-01 | 1 | ANGPTL6 | 1 | 1 |
| CLCN6 | 6.77E-01 | 1 | ANGPTL7 | 1 | 1 |
| UGGT2 | 6.77E-01 | 1 | ANKAR | 1 | 1 |
| PCDHA1 | 6.77E-01 | 1 | ANKFY1 | 1 | 1 |
| POU3F4 | 6.77E-01 | 1 | ANKH | 1 | 1 |
| ZNF415 | 6.77E-01 | 1 | ANKHD1 | 1 | 1 |
| ZC3H3 | 6.78E-01 | 1 | ANKLE1 | 1 | 1 |
| KRT18 | 6.78E-01 | 1 | ANKLE2 | 1 | 1 |
| LTBP3 | 6.78E-01 | 1 | ANKMY1 | 1 | 1 |
| IMPG2 | 6.78E-01 | 1 | ANKMY2 | 1 | 1 |
| ZNF799 | 6.78E-01 | 1 | ANKRA2 | 1 | 1 |
| DDX4 | 6.79E-01 | 1 | ANKRD1 | 1 | 1 |
| ADARB2 | 6.79E-01 | 1 | ANKRD10 | 1 | 1 |
| KIF14 | 6.79E-01 | 1 | ANKRD13C | 1 | 1 |
| BUB1 | 6.79E-01 | 1 | ANKRD13D | 1 | 1 |
| RNF219 | 6.79E-01 | 1 | ANKRD16 | 1 | 1 |
| NUPL1 | 6.79E-01 | 1 | ANKRD2 | 1 | 1 |
| IFI44L | 6.79E-01 | 1 | ANKRD20A | 1 | 1 |
| MAP3K12 | 6.80E-01 | 1 | ANKRD20A | 1 | 1 |
| DAGLB | 6.80E-01 | 1 | ANKRD20A | 1 | 1 |
| NLRP3 | 6.80E-01 | 1 | ANKRD22 | 1 | 1 |
| SLC35F2 | 6.80E-01 | 1 | ANKRD23 | 1 | 1 |
| NRCAM | 6.80E-01 | 1 | ANKRD24 | 1 | 1 |
| CLCA2 | 6.80E-01 | 1 | ANKRD28 | 1 | 1 |
| FAM24A | 6.80E-01 | 1 | ANKRD31 | 1 | 1 |
| ZNF461 | 6.81E-01 | 1 | ANKRD33 | 1 | 1 |
| MAP3K10 | 6.81E-01 | 1 | ANKRD33B | 1 | 1 |
| CADPS2 | 6.81E-01 | 1 | ANKRD34A | 1 | 1 |
| MEOX2 | 6.81E-01 | 1 | ANKRD35 | 1 | 1 |
| SP110 | 6.82E-01 | 1 | ANKRD37 | 1 | 1 |
| GP2 | 6.82E-01 | 1 | ANKRD39 | 1 | 1 |
| CDC42BP | 6.82E-01 | 1 | ANKRD40 | 1 | 1 |
| RPAP3 | 6.82E-01 | 1 | ANKRD42 | 1 | 1 |
| BICC1 | 6.82E-01 | 1 | ANKRD43 | 1 | 1 |
| PDE4D | 6.82E-01 | 1 | ANKRD45 | 1 | 1 |
| DAB2 | 6.83E-01 | 1 | ANKRD46 | 1 | 1 |
| FSCN1 | 6.83E-01 | 1 | ANKRD49 | 1 | 1 |
| PSKH2 | 6.83E-01 | 1 | ANKRD5 | 1 | 1 |
| XPO7 | 6.83E-01 | 1 | ANKRD50 | 1 | 1 |
| LOXL2 | 6.83E-01 | 1 | ANKRD55 | 1 | 1 |
| AMACR | 6.83E-01 | 1 | ANKRD56 | 1 | 1 |
| GALC | 6.83E-01 | 1 | ANKRD57 | 1 | 1 |
| EPS8L3 | 6.83E-01 | 1 | ANKRD58 | 1 | 1 |
| HJURP | 6.83E-01 | 1 | ANKRD9 | 1 | 1 |
| LRRK1 | 6.83E-01 | 1 | ANKS1A | 1 | 1 |
| FREM2 | 6.83E-01 | 1 | ANKS4B | 1 | 1 |
| PLXNC1 | 6.84E-01 | 1 | ANO2 | 1 | 1 |

| TCF3 | 6.84E-01 | 1 | ANO6 | 1 | 1 |
| --- | --- | --- | --- | --- | --- |
| DDX3Y | 6.84E-01 | 1 | ANO8 | 1 | 1 |
| MIA3 | 6.84E-01 | 1 | ANP32A | 1 | 1 |
| THEMIS | 6.84E-01 | 1 | ANP32B | 1 | 1 |
| HSF2 | 6.84E-01 | 1 | ANP32C | 1 | 1 |
| ASMTL | 6.84E-01 | 1 | ANP32D | 1 | 1 |
| TTC39A | 6.84E-01 | 1 | ANP32E | 1 | 1 |
| DIS3L | 6.84E-01 | 1 | ANPEP | 1 | 1 |
| ITGA8 | 6.84E-01 | 1 | ANTXR1 | 1 | 1 |
| CTSC | 6.84E-01 | 1 | ANTXR2 | 1 | 1 |
| DDX53 | 6.84E-01 | 1 | ANUBL1 | 1 | 1 |
| GMCL1 | 6.85E-01 | 1 | ANXA1 | 1 | 1 |
| KCNS3 | 6.85E-01 | 1 | ANXA11 | 1 | 1 |
| CYP26B1 | 6.85E-01 | 1 | ANXA13 | 1 | 1 |
| SRRM3 | 6.85E-01 | 1 | ANXA3 | 1 | 1 |
| LMNB2 | 6.85E-01 | 1 | ANXA4 | 1 | 1 |
| DLD | 6.85E-01 | 1 | ANXA5 | 1 | 1 |
| RMI1 | 6.85E-01 | 1 | ANXA8 | 1 | 1 |
| CSTF2T | 6.85E-01 | 1 | ANXA8L1 | 1 | 1 |
| GRK7 | 6.85E-01 | 1 | ANXA8L2 | 1 | 1 |
| DENND4A | 6.86E-01 | 1 | AOAH | 1 | 1 |
| LONRF3 | 6.86E-01 | 1 | AOC3 | 1 | 1 |
| CCDC39 | 6.86E-01 | 1 | AP1AR | 1 | 1 |
| EDC3 | 6.86E-01 | 1 | AP1B1 | 1 | 1 |
| ESCO2 | 6.86E-01 | 1 | AP1G2 | 1 | 1 |
| ATG4B | 6.86E-01 | 1 | AP1M1 | 1 | 1 |
| KANK1 | 6.86E-01 | 1 | AP1M2 | 1 | 1 |
| OR14C36 | 6.86E-01 | 1 | AP1S1 | 1 | 1 |
| NNT | 6.87E-01 | 1 | AP1S2 | 1 | 1 |
| FRK | 6.87E-01 | 1 | AP1S3 | 1 | 1 |
| TRIM43 | 6.87E-01 | 1 | AP2A1 | 1 | 1 |
| VCP | 6.87E-01 | 1 | AP2M1 | 1 | 1 |
| NRG3 | 6.87E-01 | 1 | AP2S1 | 1 | 1 |
| PARP12 | 6.87E-01 | 1 | AP3M1 | 1 | 1 |
| NEK8 | 6.87E-01 | 1 | AP3S2 | 1 | 1 |
| KLHL10 | 6.87E-01 | 1 | AP4B1 | 1 | 1 |
| ZNF251 | 6.87E-01 | 1 | AP4E1 | 1 | 1 |
| MAN1A2 | 6.87E-01 | 1 | AP4M1 | 1 | 1 |
| GNAT3 | 6.87E-01 | 1 | AP4S1 | 1 | 1 |
| ZBED1 | 6.88E-01 | 1 | APBB1IP | 1 | 1 |
| SLC35F5 | 6.88E-01 | 1 | APBB3 | 1 | 1 |
| JAG2 | 6.88E-01 | 1 | APCDD1 | 1 | 1 |
| PTER | 6.88E-01 | 1 | APEX2 | 1 | 1 |
| ZBTB17 | 6.88E-01 | 1 | APH1A | 1 | 1 |
| KIAA0368 | 6.88E-01 | 1 | APH1B | 1 | 1 |
| LRRC27 | 6.88E-01 | 1 | API5 | 1 | 1 |
| ZFP37 | 6.88E-01 | 1 | APIP | 1 | 1 |
| UGT2B28 | 6.88E-01 | 1 | APITD1 | 1 | 1 |
| FERMT1 | 6.88E-01 | 1 | APLN | 1 | 1 |
| ZNF628 | 6.89E-01 | 1 | APLNR | 1 | 1 |
| DCAF13 | 6.89E-01 | 1 | APLP1 | 1 | 1 |
| AP3D1 | 6.89E-01 | 1 | APLP2 | 1 | 1 |
| FAM198B | 6.89E-01 | 1 | APOA1BP | 1 | 1 |

| BBS9 | 6.89E-01 | 1 | APOA2 | 1 | 1 |
| --- | --- | --- | --- | --- | --- |
| SLC7A7 | 6.89E-01 | 1 | APOA4 | 1 | 1 |
| PDHA1 | 6.89E-01 | 1 | APOA5 | 1 | 1 |
| MCM3AP | 6.89E-01 | 1 | APOB48R | 1 | 1 |
| ZNF100 | 6.89E-01 | 1 | APOBEC1 | 1 | 1 |
| KLHL35 | 6.89E-01 | 1 | APOBEC2 | 1 | 1 |
| CENPI | 6.89E-01 | 1 | APOBEC3A | 1 | 1 |
| SOAT2 | 6.90E-01 | 1 | APOBEC3C | 1 | 1 |
| FGFR4 | 6.90E-01 | 1 | APOBEC3D | 1 | 1 |
| GRHL2 | 6.90E-01 | 1 | APOBEC3F | 1 | 1 |
| ZNF202 | 6.90E-01 | 1 | APOBEC3G | 1 | 1 |
| ZNF443 | 6.90E-01 | 1 | APOBEC3H | 1 | 1 |
| CLCNKB | 6.90E-01 | 1 | APOC2 | 1 | 1 |
| TRPV2 | 6.90E-01 | 1 | APOC3 | 1 | 1 |
| MBD5 | 6.90E-01 | 1 | APOC4 | 1 | 1 |
| NVL | 6.90E-01 | 1 | APOD | 1 | 1 |
| ACSL3 | 6.90E-01 | 1 | APOE | 1 | 1 |
| CLOCK | 6.90E-01 | 1 | APOF | 1 | 1 |
| CD248 | 6.90E-01 | 1 | APOL1 | 1 | 1 |
| NBPF1 | 6.91E-01 | 1 | APOL2 | 1 | 1 |
| ZNF662 | 6.91E-01 | 1 | APOL3 | 1 | 1 |
| DTX4 | 6.91E-01 | 1 | APOL4 | 1 | 1 |
| SLC5A5 | 6.91E-01 | 1 | APOL5 | 1 | 1 |
| ZNF221 | 6.91E-01 | 1 | APOL6 | 1 | 1 |
| KCP | 6.91E-01 | 1 | APOLD1 | 1 | 1 |
| ROCK1 | 6.91E-01 | 1 | APOM | 1 | 1 |
| FNIP1 | 6.92E-01 | 1 | APOO | 1 | 1 |
| KIF15 | 6.92E-01 | 1 | APPL1 | 1 | 1 |
| DHX32 | 6.92E-01 | 1 | APPL2 | 1 | 1 |
| BPI | 6.92E-01 | 1 | APRT | 1 | 1 |
| SLC17A8 | 6.92E-01 | 1 | APTX | 1 | 1 |
| PCSK2 | 6.92E-01 | 1 | AQP10 | 1 | 1 |
| SCARB1 | 6.92E-01 | 1 | AQP12A | 1 | 1 |
| BCORL1 | 6.92E-01 | 1 | AQP12B | 1 | 1 |
| CPS1 | 6.92E-01 | 1 | AQP3 | 1 | 1 |
| CTNNAL1 | 6.92E-01 | 1 | AQP4 | 1 | 1 |
| SAFB | 6.93E-01 | 1 | AQP6 | 1 | 1 |
| TMPRSS9 | 6.93E-01 | 1 | AQP7 | 1 | 1 |
| GPR50 | 6.93E-01 | 1 | AQP8 | 1 | 1 |
| CCT5 | 6.93E-01 | 1 | AQP9 | 1 | 1 |
| TTLL12 | 6.93E-01 | 1 | AQPEP | 1 | 1 |
| MAP4K5 | 6.93E-01 | 1 | ARAP2 | 1 | 1 |
| NEBL | 6.93E-01 | 1 | ARC | 1 | 1 |
| WDR43 | 6.93E-01 | 1 | ARCN1 | 1 | 1 |
| GPR152 | 6.93E-01 | 1 | AREG | 1 | 1 |
| ZNF214 | 6.93E-01 | 1 | ARF3 | 1 | 1 |
| THRAP3 | 6.93E-01 | 1 | ARF4 | 1 | 1 |
| NAV1 | 6.93E-01 | 1 | ARF5 | 1 | 1 |
| MAPK4 | 6.93E-01 | 1 | ARF6 | 1 | 1 |
| EZH2 | 6.93E-01 | 1 | ARFGAP2 | 1 | 1 |
| ACAP3 | 6.93E-01 | 1 | ARFGAP3 | 1 | 1 |
| MAPK7 | 6.93E-01 | 1 | ARFIP1 | 1 | 1 |
| STT3B | 6.94E-01 | 1 | ARG1 | 1 | 1 |

| B3GALT1 | 6.94E-01 | 1 | ARG2 | 1 | 1 |
| --- | --- | --- | --- | --- | --- |
| ZNF57 | 6.94E-01 | 1 | ARGLU1 | 1 | 1 |
| ADI1 | 6.94E-01 | 1 | ARHGAP1 | 1 | 1 |
| PLD5 | 6.94E-01 | 1 | ARHGAP11 | 1 | 1 |
| CPT1B | 6.94E-01 | 1 | ARHGAP11 | 1 | 1 |
| ITGA9 | 6.94E-01 | 1 | ARHGAP12 | 1 | 1 |
| CHTF18 | 6.94E-01 | 1 | ARHGAP19 | 1 | 1 |
| MUM1 | 6.94E-01 | 1 | ARHGAP20 | 1 | 1 |
| MARVEL | 6.94E-01 | 1 | ARHGAP21 | 1 | 1 |
| MARS2 | 6.94E-01 | 1 | ARHGAP25 | 1 | 1 |
| MYH4 | 6.95E-01 | 1 | ARHGAP28 | 1 | 1 |
| STIL | 6.95E-01 | 1 | ARHGAP29 | 1 | 1 |
| LZTR1 | 6.95E-01 | 1 | ARHGAP33 | 1 | 1 |
| PPP2R2A | 6.95E-01 | 1 | ARHGAP36 | 1 | 1 |
| HDAC9 | 6.95E-01 | 1 | ARHGAP4 | 1 | 1 |
| NEK5 | 6.95E-01 | 1 | ARHGAP40 | 1 | 1 |
| CCDC87 | 6.95E-01 | 1 | ARHGAP5 | 1 | 1 |
| IL4I1 | 6.95E-01 | 1 | ARHGAP8 | 1 | 1 |
| SCEL | 6.96E-01 | 1 | ARHGAP9 | 1 | 1 |
| CTTNBP2 | 6.96E-01 | 1 | ARHGDIA | 1 | 1 |
| PIP4K2C | 6.96E-01 | 1 | ARHGDIB | 1 | 1 |
| WDR91 | 6.96E-01 | 1 | ARHGDIG | 1 | 1 |
| DYNC1I1 | 6.96E-01 | 1 | ARHGEF1 | 1 | 1 |
| RBM25 | 6.96E-01 | 1 | ARHGEF10 | 1 | 1 |
| DYM | 6.96E-01 | 1 | ARHGEF16 | 1 | 1 |
| FPR3 | 6.96E-01 | 1 | ARHGEF19 | 1 | 1 |
| CYP2F1 | 6.96E-01 | 1 | ARHGEF35 | 1 | 1 |
| ZNF654 | 6.96E-01 | 1 | ARHGEF38 | 1 | 1 |
| RTF1 | 6.97E-01 | 1 | ARHGEF4 | 1 | 1 |
| GABRA5 | 6.97E-01 | 1 | ARHGEF9 | 1 | 1 |
| DPYD | 6.97E-01 | 1 | ARID3B | 1 | 1 |
| TTLL8 | 6.97E-01 | 1 | ARID3C | 1 | 1 |
| TULP4 | 6.97E-01 | 1 | ARID4A | 1 | 1 |
| ZP4 | 6.97E-01 | 1 | ARID4B | 1 | 1 |
| KRT23 | 6.97E-01 | 1 | ARID5A | 1 | 1 |
| PJA2 | 6.97E-01 | 1 | ARIH1 | 1 | 1 |
| FER | 6.98E-01 | 1 | ARL1 | 1 | 1 |
| MAGEF1 | 6.98E-01 | 1 | ARL10 | 1 | 1 |
| CSMD3 | 6.98E-01 | 1 | ARL11 | 1 | 1 |
| UBE3C | 6.98E-01 | 1 | ARL14 | 1 | 1 |
| TRAPPC1 | 6.98E-01 | 1 | ARL15 | 1 | 1 |
| RANGAP1 | 6.98E-01 | 1 | ARL16 | 1 | 1 |
| SYDE1 | 6.98E-01 | 1 | ARL17A | 1 | 1 |
| CHRD | 6.98E-01 | 1 | ARL17B | 1 | 1 |
| GALNT9 | 6.98E-01 | 1 | ARL2 | 1 | 1 |
| PLXNB1 | 6.98E-01 | 1 | ARL2BP | 1 | 1 |
| SAMD7 | 6.98E-01 | 1 | ARL3 | 1 | 1 |
| ZXDB | 6.98E-01 | 1 | ARL4A | 1 | 1 |
| STOX1 | 6.99E-01 | 1 | ARL4C | 1 | 1 |
| LTF | 6.99E-01 | 1 | ARL4D | 1 | 1 |
| DAG1 | 6.99E-01 | 1 | ARL5A | 1 | 1 |
| GRIK2 | 6.99E-01 | 1 | ARL5C | 1 | 1 |
| PIF1 | 6.99E-01 | 1 | ARL6 | 1 | 1 |

| SMC5 | 6.99E-01 | 1 | ARL6IP1 | 1 | 1 |
| --- | --- | --- | --- | --- | --- |
| TTLL3 | 7.00E-01 | 1 | ARL6IP4 | 1 | 1 |
| ZFYVE16 | 7.00E-01 | 1 | ARL6IP5 | 1 | 1 |
| ZFP36 | 7.00E-01 | 1 | ARL6IP6 | 1 | 1 |
| HERC1 | 7.00E-01 | 1 | ARL8B | 1 | 1 |
| RASGRP4 | 7.00E-01 | 1 | ARMC1 | 1 | 1 |
| ZNF596 | 7.00E-01 | 1 | ARMC10 | 1 | 1 |
| CLPTM1 | 7.00E-01 | 1 | ARMC4 | 1 | 1 |
| TAP1 | 7.00E-01 | 1 | ARMC5 | 1 | 1 |
| THRB | 7.00E-01 | 1 | ARMC8 | 1 | 1 |
| ANTXR2 | 7.00E-01 | 1 | ARMCX2 | 1 | 1 |
| DMBT1 | 7.00E-01 | 1 | ARMCX5 | 1 | 1 |
| PDE4C | 7.00E-01 | 1 | ARMS2 | 1 | 1 |
| DCAF5 | 7.00E-01 | 1 | ARNTL | 1 | 1 |
| TECRL | 7.01E-01 | 1 | ARPC1A | 1 | 1 |
| ABCB6 | 7.01E-01 | 1 | ARPC1B | 1 | 1 |
| NLRP5 | 7.01E-01 | 1 | ARPC3 | 1 | 1 |
| CLK4 | 7.01E-01 | 1 | ARPC4 | 1 | 1 |
| SHANK2 | 7.01E-01 | 1 | ARPC5 | 1 | 1 |
| CCT6B | 7.01E-01 | 1 | ARPC5L | 1 | 1 |
| HK1 | 7.01E-01 | 1 | ARPM1 | 1 | 1 |
| CRNN | 7.01E-01 | 1 | ARPP19 | 1 | 1 |
| TRHDE | 7.01E-01 | 1 | ARPP21 | 1 | 1 |
| PTH1R | 7.01E-01 | 1 | ARR3 | 1 | 1 |
| PREPL | 7.02E-01 | 1 | ARRB1 | 1 | 1 |
| MCTP2 | 7.02E-01 | 1 | ARRB2 | 1 | 1 |
| MST1R | 7.02E-01 | 1 | ARRDC1 | 1 | 1 |
| DCAF12L | 7.02E-01 | 1 | ARRDC2 | 1 | 1 |
| FAM189B | 7.02E-01 | 1 | ARRDC4 | 1 | 1 |
| SYNRG | 7.02E-01 | 1 | ARSA | 1 | 1 |
| EPB41L2 | 7.02E-01 | 1 | ARSB | 1 | 1 |
| CNTFR | 7.02E-01 | 1 | ARSE | 1 | 1 |
| SEMA6B | 7.03E-01 | 1 | ARSF | 1 | 1 |
| WDR93 | 7.03E-01 | 1 | ARSG | 1 | 1 |
| SLC36A4 | 7.03E-01 | 1 | ARSH | 1 | 1 |
| PIBF1 | 7.03E-01 | 1 | ARSI | 1 | 1 |
| CTAGE1 | 7.03E-01 | 1 | ARSJ | 1 | 1 |
| LONRF1 | 7.03E-01 | 1 | ART1 | 1 | 1 |
| ZNF763 | 7.03E-01 | 1 | ART3 | 1 | 1 |
| TRAP1 | 7.03E-01 | 1 | ART4 | 1 | 1 |
| PLXNA4 | 7.03E-01 | 1 | ART5 | 1 | 1 |
| AFP | 7.04E-01 | 1 | ARV1 | 1 | 1 |
| SLCO1B1 | 7.04E-01 | 1 | AS3MT | 1 | 1 |
| HLCS | 7.04E-01 | 1 | ASAH2B | 1 | 1 |
| ARID5A | 7.04E-01 | 1 | ASAM | 1 | 1 |
| FSCB | 7.04E-01 | 1 | ASAP3 | 1 | 1 |
| SGCZ | 7.05E-01 | 1 | ASB1 | 1 | 1 |
| HRNR | 7.05E-01 | 1 | ASB11 | 1 | 1 |
| ANKIB1 | 7.05E-01 | 1 | ASB12 | 1 | 1 |
| GAB1 | 7.05E-01 | 1 | ASB14 | 1 | 1 |
| MED15 | 7.05E-01 | 1 | ASB15 | 1 | 1 |
| LENG8 | 7.05E-01 | 1 | ASB17 | 1 | 1 |
| MANBA | 7.05E-01 | 1 | ASB18 | 1 | 1 |

| GAA | 7.05E-01 | 1 | ASB3 | 1 | 1 |
| --- | --- | --- | --- | --- | --- |
| CNTNAP5 | 7.06E-01 | 1 | ASB6 | 1 | 1 |
| CPSF3 | 7.06E-01 | 1 | ASB7 | 1 | 1 |
| FTSJ3 | 7.06E-01 | 1 | ASB8 | 1 | 1 |
| KDELC2 | 7.06E-01 | 1 | ASB9 | 1 | 1 |
| WT1 | 7.06E-01 | 1 | ASCC1 | 1 | 1 |
| PRDM9 | 7.06E-01 | 1 | ASCL1 | 1 | 1 |
| VARS2 | 7.06E-01 | 1 | ASCL2 | 1 | 1 |
| CHST9 | 7.06E-01 | 1 | ASCL3 | 1 | 1 |
| GLI3 | 7.06E-01 | 1 | ASCL4 | 1 | 1 |
| ABCC4 | 7.06E-01 | 1 | ASF1A | 1 | 1 |
| ZNF665 | 7.06E-01 | 1 | ASF1B | 1 | 1 |
| TMEM211 | 7.06E-01 | 1 | ASGR1 | 1 | 1 |
| ERO1L | 7.07E-01 | 1 | ASGR2 | 1 | 1 |
| SLC44A4 | 7.07E-01 | 1 | ASH2L | 1 | 1 |
| ZRANB1 | 7.07E-01 | 1 | ASIP | 1 | 1 |
| CAPN14 | 7.07E-01 | 1 | ASMT | 1 | 1 |
| RYR2 | 7.07E-01 | 1 | ASMTL | 1 | 1 |
| ATP6V1B | 7.07E-01 | 1 | ASNA1 | 1 | 1 |
| EXOC7 | 7.07E-01 | 1 | ASNSD1 | 1 | 1 |
| TMEM5 | 7.07E-01 | 1 | ASPA | 1 | 1 |
| VPS39 | 7.08E-01 | 1 | ASPDH | 1 | 1 |
| KCTD19 | 7.08E-01 | 1 | ASPG | 1 | 1 |
| SV2B | 7.08E-01 | 1 | ASPHD1 | 1 | 1 |
| ZNF561 | 7.08E-01 | 1 | ASPHD2 | 1 | 1 |
| SMYD2 | 7.08E-01 | 1 | ASPN | 1 | 1 |
| ATP1A2 | 7.08E-01 | 1 | ASRGL1 | 1 | 1 |
| USP9X | 7.09E-01 | 1 | ASTE1 | 1 | 1 |
| ZNF652 | 7.09E-01 | 1 | ASTN2 | 1 | 1 |
| CA10 | 7.09E-01 | 1 | ASXL2 | 1 | 1 |
| KIAA0556 | 7.09E-01 | 1 | ASZ1 | 1 | 1 |
| CECR5 | 7.09E-01 | 1 | ATAD1 | 1 | 1 |
| PECR | 7.09E-01 | 1 | ATAD3A | 1 | 1 |
| PCDHGA6 | 7.09E-01 | 1 | ATAD3C | 1 | 1 |
| NPY5R | 7.09E-01 | 1 | ATCAY | 1 | 1 |
| PRDX1 | 7.09E-01 | 1 | ATE1 | 1 | 1 |
| ACAN | 7.10E-01 | 1 | ATF1 | 1 | 1 |
| SLC22A10 | 7.10E-01 | 1 | ATF2 | 1 | 1 |
| TAF3 | 7.10E-01 | 1 | ATF3 | 1 | 1 |
| ARNT2 | 7.10E-01 | 1 | ATF5 | 1 | 1 |
| PLEKHG5 | 7.10E-01 | 1 | ATF6B | 1 | 1 |
| ADCY10 | 7.10E-01 | 1 | ATF7 | 1 | 1 |
| CARNS1 | 7.10E-01 | 1 | ATF7IP2 | 1 | 1 |
| SBSN | 7.11E-01 | 1 | ATG10 | 1 | 1 |
| GPAT2 | 7.11E-01 | 1 | ATG12 | 1 | 1 |
| SLU7 | 7.11E-01 | 1 | ATG3 | 1 | 1 |
| XPOT | 7.11E-01 | 1 | ATG4A | 1 | 1 |
| NEDD9 | 7.11E-01 | 1 | ATG4B | 1 | 1 |
| KDELC1 | 7.11E-01 | 1 | ATG5 | 1 | 1 |
| CCIN | 7.11E-01 | 1 | ATG7 | 1 | 1 |
| SALL3 | 7.11E-01 | 1 | ATG9B | 1 | 1 |
| TNPO2 | 7.11E-01 | 1 | ATL3 | 1 | 1 |
| ZNF567 | 7.11E-01 | 1 | ATN1 | 1 | 1 |

| RBM15B | 7.12E-01 | 1 | ATOH7 | 1 | 1 |
| --- | --- | --- | --- | --- | --- |
| KCNB1 | 7.12E-01 | 1 | ATOH8 | 1 | 1 |
| CASQ2 | 7.12E-01 | 1 | ATOX1 | 1 | 1 |
| GRIK5 | 7.12E-01 | 1 | ATP10A | 1 | 1 |
| SCN3B | 7.12E-01 | 1 | ATP11A | 1 | 1 |
| KLHL5 | 7.12E-01 | 1 | ATP13A3 | 1 | 1 |
| FLRT3 | 7.12E-01 | 1 | ATP1B1 | 1 | 1 |
| NWD1 | 7.12E-01 | 1 | ATP1B3 | 1 | 1 |
| KLHL23 | 7.12E-01 | 1 | ATP2B4 | 1 | 1 |
| SLC28A3 | 7.13E-01 | 1 | ATP2C1 | 1 | 1 |
| DOCK10 | 7.13E-01 | 1 | ATP4B | 1 | 1 |
| ZNF19 | 7.13E-01 | 1 | ATP5C1 | 1 | 1 |
| SLC22A17 | 7.13E-01 | 1 | ATP5D | 1 | 1 |
| MED16 | 7.13E-01 | 1 | ATP5E | 1 | 1 |
| LIG3 | 7.13E-01 | 1 | ATP5F1 | 1 | 1 |
| MTMR8 | 7.13E-01 | 1 | ATP5G1 | 1 | 1 |
| SPIRE1 | 7.13E-01 | 1 | ATP5G2 | 1 | 1 |
| FOXD4L4 | 7.14E-01 | 1 | ATP5G3 | 1 | 1 |
| PTPRS | 7.14E-01 | 1 | ATP5H | 1 | 1 |
| LHX8 | 7.14E-01 | 1 | ATP5I | 1 | 1 |
| ADAMTS | 7.14E-01 | 1 | ATP5J | 1 | 1 |
| ZSCAN18 | 7.15E-01 | 1 | ATP5J2 | 1 | 1 |
| TFDP1 | 7.15E-01 | 1 | ATP5L2 | 1 | 1 |
| OSBPL10 | 7.15E-01 | 1 | ATP5O | 1 | 1 |
| IFI16 | 7.15E-01 | 1 | ATP5S | 1 | 1 |
| RAP1GDS | 7.15E-01 | 1 | ATP5SL | 1 | 1 |
| OR5L2 | 7.15E-01 | 1 | ATP6AP1L | 1 | 1 |
| YTHDF3 | 7.15E-01 | 1 | ATP6AP2 | 1 | 1 |
| CD36 | 7.15E-01 | 1 | ATP6V0A2 | 1 | 1 |
| RANBP6 | 7.15E-01 | 1 | ATP6V0B | 1 | 1 |
| TBCK | 7.15E-01 | 1 | ATP6V0C | 1 | 1 |
| DEF6 | 7.15E-01 | 1 | ATP6V0D1 | 1 | 1 |
| SIGLEC9 | 7.15E-01 | 1 | ATP6V0E1 | 1 | 1 |
| GOLGA6C | 7.16E-01 | 1 | ATP6V0E2 | 1 | 1 |
| ZSCAN2 | 7.16E-01 | 1 | ATP6V1A | 1 | 1 |
| UNC45B | 7.16E-01 | 1 | ATP6V1B1 | 1 | 1 |
| MKKS | 7.16E-01 | 1 | ATP6V1C1 | 1 | 1 |
| CASD1 | 7.17E-01 | 1 | ATP6V1C2 | 1 | 1 |
| CCDC151 | 7.17E-01 | 1 | ATP6V1D | 1 | 1 |
| KIRREL3 | 7.17E-01 | 1 | ATP6V1E2 | 1 | 1 |
| DCST1 | 7.17E-01 | 1 | ATP6V1G1 | 1 | 1 |
| PDS5A | 7.17E-01 | 1 | ATP6V1G2 | 1 | 1 |
| MOCOS | 7.17E-01 | 1 | ATP6V1G3 | 1 | 1 |
| HEPACA | 7.17E-01 | 1 | ATP6V1H | 1 | 1 |
| UNC13B | 7.17E-01 | 1 | ATP7B | 1 | 1 |
| SLC5A4 | 7.17E-01 | 1 | ATP8B1 | 1 | 1 |
| KLHL11 | 7.17E-01 | 1 | ATP8B4 | 1 | 1 |
| UBE2O | 7.17E-01 | 1 | ATPAF1 | 1 | 1 |
| KCNH2 | 7.18E-01 | 1 | ATPAF2 | 1 | 1 |
| MNS1 | 7.18E-01 | 1 | ATPBD4 | 1 | 1 |
| TRAF7 | 7.18E-01 | 1 | ATPIF1 | 1 | 1 |
| ARMCX1 | 7.18E-01 | 1 | ATRIP | 1 | 1 |
| TTLL2 | 7.18E-01 | 1 | ATRX | 1 | 1 |

| GRIN2D | 7.18E-01 | 1 | ATXN1 | 1 | 1 |
| --- | --- | --- | --- | --- | --- |
| LSR | 7.18E-01 | 1 | ATXN10 | 1 | 1 |
| CELSR2 | 7.18E-01 | 1 | ATXN2 | 1 | 1 |
| ESYT3 | 7.18E-01 | 1 | ATXN2L | 1 | 1 |
| EXTL1 | 7.18E-01 | 1 | ATXN3 | 1 | 1 |
| TMEM150 | 7.19E-01 | 1 | ATXN3L | 1 | 1 |
| ZNF644 | 7.19E-01 | 1 | ATXN7L3B | 1 | 1 |
| ARHGEF1 | 7.19E-01 | 1 | AUH | 1 | 1 |
| POFUT2 | 7.19E-01 | 1 | AURKA | 1 | 1 |
| CRTAC1 | 7.19E-01 | 1 | AURKAIP1 | 1 | 1 |
| GIT1 | 7.19E-01 | 1 | AURKB | 1 | 1 |
| TAGAP | 7.19E-01 | 1 | AURKC | 1 | 1 |
| DCBLD1 | 7.19E-01 | 1 | AVEN | 1 | 1 |
| ZBTB10 | 7.19E-01 | 1 | AVIL | 1 | 1 |
| ATP2B1 | 7.19E-01 | 1 | AVP | 1 | 1 |
| ZNF181 | 7.20E-01 | 1 | AVPI1 | 1 | 1 |
| ZNF770 | 7.20E-01 | 1 | AVPR1A | 1 | 1 |
| ZMYND11 | 7.20E-01 | 1 | AVPR2 | 1 | 1 |
| PPM1D | 7.21E-01 | 1 | AWAT1 | 1 | 1 |
| C7orf43 | 7.21E-01 | 1 | AXL | 1 | 1 |
| C16orf89 | 7.21E-01 | 1 | AZGP1 | 1 | 1 |
| KRT73 | 7.21E-01 | 1 | AZI1 | 1 | 1 |
| SGOL2 | 7.21E-01 | 1 | AZI2 | 1 | 1 |
| ADAMTS | 7.21E-01 | 1 | B3GALNT2 | 1 | 1 |
| TDP1 | 7.21E-01 | 1 | B3GALT1 | 1 | 1 |
| LILRB4 | 7.22E-01 | 1 | B3GALT2 | 1 | 1 |
| ITGA5 | 7.22E-01 | 1 | B3GALT4 | 1 | 1 |
| VIL1 | 7.22E-01 | 1 | B3GALT6 | 1 | 1 |
| NR4A2 | 7.22E-01 | 1 | B3GALTL | 1 | 1 |
| ROBO3 | 7.22E-01 | 1 | B3GAT2 | 1 | 1 |
| PRDM8 | 7.22E-01 | 1 | B3GAT3 | 1 | 1 |
| DNAH14 | 7.22E-01 | 1 | B3GNT1 | 1 | 1 |
| LPCAT2 | 7.22E-01 | 1 | B3GNT2 | 1 | 1 |
| NCSTN | 7.22E-01 | 1 | B3GNT3 | 1 | 1 |
| C11orf80 | 7.23E-01 | 1 | B3GNT4 | 1 | 1 |
| MKLN1 | 7.23E-01 | 1 | B3GNT5 | 1 | 1 |
| SBF2 | 7.23E-01 | 1 | B3GNT6 | 1 | 1 |
| EFTUD1 | 7.23E-01 | 1 | B3GNT7 | 1 | 1 |
| CCDC47 | 7.23E-01 | 1 | B3GNT8 | 1 | 1 |
| AKAP11 | 7.23E-01 | 1 | B3GNT9 | 1 | 1 |
| FAR2 | 7.23E-01 | 1 | B4GALNT1 | 1 | 1 |
| MCAM | 7.23E-01 | 1 | B4GALNT2 | 1 | 1 |
| FAM179B | 7.24E-01 | 1 | B4GALT1 | 1 | 1 |
| PTPRN2 | 7.24E-01 | 1 | B4GALT2 | 1 | 1 |
| TUBA3C | 7.24E-01 | 1 | B4GALT3 | 1 | 1 |
| ARHGEF1 | 7.24E-01 | 1 | B4GALT4 | 1 | 1 |
| NUF2 | 7.24E-01 | 1 | B4GALT7 | 1 | 1 |
| GALNT6 | 7.25E-01 | 1 | BAALC | 1 | 1 |
| ZNF697 | 7.25E-01 | 1 | BAAT | 1 | 1 |
| ATXN7L1 | 7.25E-01 | 1 | BACE2 | 1 | 1 |
| DLG5 | 7.25E-01 | 1 | BACH2 | 1 | 1 |
| C6orf118 | 7.26E-01 | 1 | BAD | 1 | 1 |
| RECQL | 7.26E-01 | 1 | BAG1 | 1 | 1 |

| GDF10 | 7.26E-01 | 1 | BAG2 | 1 | 1 |
| --- | --- | --- | --- | --- | --- |
| GAS2L3 | 7.26E-01 | 1 | BAG3 | 1 | 1 |
| CDC73 | 7.26E-01 | 1 | BAGE | 1 | 1 |
| COL4A1 | 7.26E-01 | 1 | BAGE2 | 1 | 1 |
| CCR8 | 7.27E-01 | 1 | BAGE3 | 1 | 1 |
| EPHB6 | 7.27E-01 | 1 | BAGE4 | 1 | 1 |
| LLGL1 | 7.27E-01 | 1 | BAGE5 | 1 | 1 |
| GAD2 | 7.27E-01 | 1 | BAI1 | 1 | 1 |
| TRIOBP | 7.27E-01 | 1 | BAI2 | 1 | 1 |
| CD44 | 7.27E-01 | 1 | BAI3 | 1 | 1 |
| EPHA3 | 7.28E-01 | 1 | BAIAP2L1 | 1 | 1 |
| PRMT3 | 7.28E-01 | 1 | BAIAP2L2 | 1 | 1 |
| SH3KBP1 | 7.28E-01 | 1 | BAIAP3 | 1 | 1 |
| TROVE2 | 7.28E-01 | 1 | BAK1 | 1 | 1 |
| KIAA0232 | 7.28E-01 | 1 | BAMBI | 1 | 1 |
| GZF1 | 7.28E-01 | 1 | BANF1 | 1 | 1 |
| PSD | 7.28E-01 | 1 | BANF2 | 1 | 1 |
| PSG2 | 7.28E-01 | 1 | BANK1 | 1 | 1 |
| ZNF587 | 7.29E-01 | 1 | BANP | 1 | 1 |
| KLHL6 | 7.29E-01 | 1 | BARD1 | 1 | 1 |
| GPR37L1 | 7.29E-01 | 1 | BARHL1 | 1 | 1 |
| GAD1 | 7.29E-01 | 1 | BARX1 | 1 | 1 |
| DNMT3B | 7.29E-01 | 1 | BARX2 | 1 | 1 |
| KIAA1033 | 7.29E-01 | 1 | BAT1 | 1 | 1 |
| CCDC82 | 7.29E-01 | 1 | BAT2 | 1 | 1 |
| MAP3K13 | 7.30E-01 | 1 | BAT2L1 | 1 | 1 |
| CEP192 | 7.30E-01 | 1 | BAT2L2 | 1 | 1 |
| DPY19L1 | 7.30E-01 | 1 | BAT3 | 1 | 1 |
| BAAT | 7.30E-01 | 1 | BAT4 | 1 | 1 |
| PER3 | 7.30E-01 | 1 | BAT5 | 1 | 1 |
| PRSS50 | 7.30E-01 | 1 | BATF2 | 1 | 1 |
| PTAR1 | 7.30E-01 | 1 | BATF3 | 1 | 1 |
| SLCO4C1 | 7.30E-01 | 1 | BBC3 | 1 | 1 |
| ENPP2 | 7.30E-01 | 1 | BBOX1 | 1 | 1 |
| ZNF23 | 7.30E-01 | 1 | BBS1 | 1 | 1 |
| INPP5F | 7.31E-01 | 1 | BBS10 | 1 | 1 |
| FASTKD1 | 7.31E-01 | 1 | BBS12 | 1 | 1 |
| ANKRD50 | 7.31E-01 | 1 | BBS5 | 1 | 1 |
| TLN1 | 7.31E-01 | 1 | BCAM | 1 | 1 |
| PLEKHG3 | 7.31E-01 | 1 | BCAP29 | 1 | 1 |
| MYSM1 | 7.31E-01 | 1 | BCAP31 | 1 | 1 |
| NEFH | 7.31E-01 | 1 | BCAR1 | 1 | 1 |
| FDXACB1 | 7.31E-01 | 1 | BCAR3 | 1 | 1 |
| SLC18A2 | 7.32E-01 | 1 | BCAS1 | 1 | 1 |
| PGBD1 | 7.32E-01 | 1 | BCAS2 | 1 | 1 |
| PKD2 | 7.32E-01 | 1 | BCAS4 | 1 | 1 |
| KIF7 | 7.32E-01 | 1 | BCAT2 | 1 | 1 |
| ASTN1 | 7.32E-01 | 1 | BCCIP | 1 | 1 |
| ITGA6 | 7.32E-01 | 1 | BCHE | 1 | 1 |
| TECPR2 | 7.32E-01 | 1 | BCKDHA | 1 | 1 |
| KIAA1161 | 7.32E-01 | 1 | BCKDHB | 1 | 1 |
| ZNF516 | 7.32E-01 | 1 | BCL10 | 1 | 1 |
| UBQLN3 | 7.32E-01 | 1 | BCL2 | 1 | 1 |

| TBC1D22 | 7.32E-01 | 1 | BCL2A1 | 1 | 1 |
| --- | --- | --- | --- | --- | --- |
| ATP11B | 7.32E-01 | 1 | BCL2L1 | 1 | 1 |
| SLC5A7 | 7.33E-01 | 1 | BCL2L10 | 1 | 1 |
| APBA1 | 7.33E-01 | 1 | BCL2L11 | 1 | 1 |
| RUFY3 | 7.33E-01 | 1 | BCL2L12 | 1 | 1 |
| GRID1 | 7.33E-01 | 1 | BCL2L13 | 1 | 1 |
| WAC | 7.33E-01 | 1 | BCL2L14 | 1 | 1 |
| PCDHA6 | 7.33E-01 | 1 | BCL2L15 | 1 | 1 |
| ZUFSP | 7.33E-01 | 1 | BCL2L2 | 1 | 1 |
| SMARCC | 7.33E-01 | 1 | BCL6 | 1 | 1 |
| ADAMTS | 7.33E-01 | 1 | BCL6B | 1 | 1 |
| COL5A2 | 7.33E-01 | 1 | BCL7A | 1 | 1 |
| NRXN3 | 7.34E-01 | 1 | BCL7B | 1 | 1 |
| ADORA1 | 7.34E-01 | 1 | BCL7C | 1 | 1 |
| CENPF | 7.34E-01 | 1 | BCMO1 | 1 | 1 |
| C9orf84 | 7.34E-01 | 1 | BCO2 | 1 | 1 |
| OR2T8 | 7.34E-01 | 1 | BCORL2 | 1 | 1 |
| ITPRIP | 7.34E-01 | 1 | BCR | 1 | 1 |
| CYFIP2 | 7.34E-01 | 1 | BCS1L | 1 | 1 |
| STS | 7.35E-01 | 1 | BDH2 | 1 | 1 |
| MUC16 | 7.35E-01 | 1 | BDKRB1 | 1 | 1 |
| PNPLA7 | 7.35E-01 | 1 | BEAN | 1 | 1 |
| LRRC8D | 7.35E-01 | 1 | BEGAIN | 1 | 1 |
| AQP10 | 7.35E-01 | 1 | BEND2 | 1 | 1 |
| ABCF1 | 7.36E-01 | 1 | BEND4 | 1 | 1 |
| BCOR | 7.36E-01 | 1 | BEND6 | 1 | 1 |
| CPNE7 | 7.36E-01 | 1 | BEND7 | 1 | 1 |
| VSIG10 | 7.36E-01 | 1 | BEST1 | 1 | 1 |
| TNK2 | 7.36E-01 | 1 | BEST2 | 1 | 1 |
| ADAMTS | 7.36E-01 | 1 | BEST3 | 1 | 1 |
| PRRT4 | 7.36E-01 | 1 | BEST4 | 1 | 1 |
| ANKRD20 | 7.37E-01 | 1 | BET1 | 1 | 1 |
| IL17RB | 7.37E-01 | 1 | BET1L | 1 | 1 |
| MTMR4 | 7.37E-01 | 1 | BET3L | 1 | 1 |
| KRT6C | 7.37E-01 | 1 | BEX1 | 1 | 1 |
| SYT1 | 7.37E-01 | 1 | BEX2 | 1 | 1 |
| AHNAK2 | 7.37E-01 | 1 | BEX5 | 1 | 1 |
| RGS12 | 7.37E-01 | 1 | BFAR | 1 | 1 |
| FAM83G | 7.37E-01 | 1 | BFSP1 | 1 | 1 |
| TRPM5 | 7.37E-01 | 1 | BFSP2 | 1 | 1 |
| SHC1 | 7.38E-01 | 1 | BGLAP | 1 | 1 |
| OAS2 | 7.38E-01 | 1 | BGN | 1 | 1 |
| ISM2 | 7.38E-01 | 1 | BHLHA15 | 1 | 1 |
| UBC | 7.38E-01 | 1 | BHLHA9 | 1 | 1 |
| SMC1B | 7.38E-01 | 1 | BHLHE22 | 1 | 1 |
| THBD | 7.38E-01 | 1 | BHLHE23 | 1 | 1 |
| TRAF6 | 7.39E-01 | 1 | BHLHE41 | 1 | 1 |
| ZYG11A | 7.39E-01 | 1 | BHMT | 1 | 1 |
| PCDHA8 | 7.39E-01 | 1 | BHMT2 | 1 | 1 |
| DDX60L | 7.39E-01 | 1 | BICC1 | 1 | 1 |
| CHD3 | 7.39E-01 | 1 | BICD1 | 1 | 1 |
| GGN | 7.40E-01 | 1 | BID | 1 | 1 |
| CIT | 7.40E-01 | 1 | BIK | 1 | 1 |

| NT5C1B | 7.40E-01 | 1 | BIN2 | 1 | 1 |
| --- | --- | --- | --- | --- | --- |
| CUX1 | 7.40E-01 | 1 | BIN3 | 1 | 1 |
| SH3PXD2 | 7.41E-01 | 1 | BIRC2 | 1 | 1 |
| HYAL4 | 7.41E-01 | 1 | BIRC3 | 1 | 1 |
| HSPA8 | 7.41E-01 | 1 | BIRC5 | 1 | 1 |
| BCR | 7.41E-01 | 1 | BIRC7 | 1 | 1 |
| RGL3 | 7.41E-01 | 1 | BIVM | 1 | 1 |
| SP4 | 7.42E-01 | 1 | BLCAP | 1 | 1 |
| PRAMEF1 | 7.42E-01 | 1 | BLMH | 1 | 1 |
| HTR1B | 7.42E-01 | 1 | BLOC1S1 | 1 | 1 |
| NT5C2 | 7.42E-01 | 1 | BLOC1S2 | 1 | 1 |
| DCST2 | 7.42E-01 | 1 | BLOC1S3 | 1 | 1 |
| NPR2 | 7.42E-01 | 1 | BLVRA | 1 | 1 |
| ZNF618 | 7.42E-01 | 1 | BLVRB | 1 | 1 |
| RUFY2 | 7.42E-01 | 1 | BLZF1 | 1 | 1 |
| ZC3H7B | 7.43E-01 | 1 | BMF | 1 | 1 |
| JARID2 | 7.43E-01 | 1 | BMI1 | 1 | 1 |
| DCAF8L1 | 7.43E-01 | 1 | BMP1 | 1 | 1 |
| CPNE4 | 7.43E-01 | 1 | BMP10 | 1 | 1 |
| EPS8L2 | 7.44E-01 | 1 | BMP5 | 1 | 1 |
| CCDC88B | 7.44E-01 | 1 | BMP6 | 1 | 1 |
| GCNT7 | 7.44E-01 | 1 | BMP8A | 1 | 1 |
| PRKG2 | 7.44E-01 | 1 | BMP8B | 1 | 1 |
| ACACA | 7.44E-01 | 1 | BMPR1B | 1 | 1 |
| MFSD6L | 7.44E-01 | 1 | BNC1 | 1 | 1 |
| MCM4 | 7.45E-01 | 1 | BNIP1 | 1 | 1 |
| PRKCB | 7.45E-01 | 1 | BNIP3L | 1 | 1 |
| ZNF502 | 7.45E-01 | 1 | BNIPL | 1 | 1 |
| L3MBTL4 | 7.45E-01 | 1 | BOD1L | 1 | 1 |
| GNAI1 | 7.46E-01 | 1 | BOK | 1 | 1 |
| IL9R | 7.46E-01 | 1 | BOLA1 | 1 | 1 |
| SEC14L5 | 7.46E-01 | 1 | BOLA2 | 1 | 1 |
| ADCY6 | 7.46E-01 | 1 | BOLA2B | 1 | 1 |
| CSTF1 | 7.46E-01 | 1 | BOLA3 | 1 | 1 |
| EXOC5 | 7.46E-01 | 1 | BOLL | 1 | 1 |
| C2orf16 | 7.46E-01 | 1 | BOP1 | 1 | 1 |
| EYS | 7.46E-01 | 1 | BPGM | 1 | 1 |
| TRPC4 | 7.47E-01 | 1 | BPHL | 1 | 1 |
| MDH1B | 7.47E-01 | 1 | BPI | 1 | 1 |
| SH3PXD2 | 7.47E-01 | 1 | BPIL1 | 1 | 1 |
| MX2 | 7.47E-01 | 1 | BPIL2 | 1 | 1 |
| SDAD1 | 7.47E-01 | 1 | BPIL3 | 1 | 1 |
| ZNF83 | 7.47E-01 | 1 | BPNT1 | 1 | 1 |
| ANKRD28 | 7.47E-01 | 1 | BPY2 | 1 | 1 |
| UMOD | 7.47E-01 | 1 | BPY2B | 1 | 1 |
| ATP8B1 | 7.47E-01 | 1 | BPY2C | 1 | 1 |
| EIF3C | 7.48E-01 | 1 | BRAF | 1 | 1 |
| MAN1A1 | 7.48E-01 | 1 | BRD1 | 1 | 1 |
| GTF3C5 | 7.48E-01 | 1 | BRD3 | 1 | 1 |
| USH2A | 7.49E-01 | 1 | BRD8 | 1 | 1 |
| ZNF792 | 7.49E-01 | 1 | BRE | 1 | 1 |
| FTO | 7.49E-01 | 1 | BRF2 | 1 | 1 |
| WIZ | 7.49E-01 | 1 | BRI3 | 1 | 1 |

| ZNF878 | 7.49E-01 | 1 | BRI3BP | 1 | 1 |
| --- | --- | --- | --- | --- | --- |
| CNKSR2 | 7.49E-01 | 1 | BRIP1 | 1 | 1 |
| MYO1F | 7.49E-01 | 1 | BRMS1 | 1 | 1 |
| SERAC1 | 7.49E-01 | 1 | BRMS1L | 1 | 1 |
| PLCD1 | 7.49E-01 | 1 | BRP44 | 1 | 1 |
| KIAA1755 | 7.49E-01 | 1 | BRP44L | 1 | 1 |
| FANCD2 | 7.50E-01 | 1 | BRPF3 | 1 | 1 |
| IL18R1 | 7.50E-01 | 1 | BRS3 | 1 | 1 |
| CDH4 | 7.50E-01 | 1 | BRWD3 | 1 | 1 |
| RASGEF1 | 7.50E-01 | 1 | BSCL2 | 1 | 1 |
| ARPP21 | 7.50E-01 | 1 | BSDC1 | 1 | 1 |
| ZNF133 | 7.50E-01 | 1 | BSG | 1 | 1 |
| OSBPL6 | 7.51E-01 | 1 | BSND | 1 | 1 |
| UNK | 7.51E-01 | 1 | BSPH1 | 1 | 1 |
| FCRL3 | 7.51E-01 | 1 | BST1 | 1 | 1 |
| C10orf71 | 7.51E-01 | 1 | BST2 | 1 | 1 |
| MYO10 | 7.51E-01 | 1 | BSX | 1 | 1 |
| NOBOX | 7.51E-01 | 1 | BTBD1 | 1 | 1 |
| SORBS2 | 7.52E-01 | 1 | BTBD10 | 1 | 1 |
| DIAPH2 | 7.52E-01 | 1 | BTBD12 | 1 | 1 |
| ALDH1L1 | 7.52E-01 | 1 | BTBD16 | 1 | 1 |
| TRIM25 | 7.52E-01 | 1 | BTBD17 | 1 | 1 |
| SMC1A | 7.52E-01 | 1 | BTBD18 | 1 | 1 |
| MYNN | 7.52E-01 | 1 | BTBD19 | 1 | 1 |
| RECK | 7.53E-01 | 1 | BTBD2 | 1 | 1 |
| ALDH1B1 | 7.53E-01 | 1 | BTBD3 | 1 | 1 |
| ZNF484 | 7.53E-01 | 1 | BTBD6 | 1 | 1 |
| SEMA3G | 7.53E-01 | 1 | BTBD8 | 1 | 1 |
| ACADM | 7.53E-01 | 1 | BTBD9 | 1 | 1 |
| DOCK9 | 7.53E-01 | 1 | BTC | 1 | 1 |
| ZMYM3 | 7.54E-01 | 1 | BTD | 1 | 1 |
| NUP88 | 7.54E-01 | 1 | BTF3 | 1 | 1 |
| NLRX1 | 7.54E-01 | 1 | BTF3L4 | 1 | 1 |
| TOX2 | 7.54E-01 | 1 | BTG1 | 1 | 1 |
| LAMB3 | 7.54E-01 | 1 | BTG2 | 1 | 1 |
| NUP210L | 7.54E-01 | 1 | BTG3 | 1 | 1 |
| HGSNAT | 7.54E-01 | 1 | BTG4 | 1 | 1 |
| SLC4A2 | 7.54E-01 | 1 | BTK | 1 | 1 |
| IL1RAP | 7.55E-01 | 1 | BTLA | 1 | 1 |
| TAB3 | 7.55E-01 | 1 | BTN1A1 | 1 | 1 |
| RPTOR | 7.55E-01 | 1 | BTN3A2 | 1 | 1 |
| PANK1 | 7.55E-01 | 1 | BTN3A3 | 1 | 1 |
| FH | 7.56E-01 | 1 | BTNL8 | 1 | 1 |
| TPCN1 | 7.56E-01 | 1 | BTNL9 | 1 | 1 |
| RNF20 | 7.56E-01 | 1 | BUB1 | 1 | 1 |
| ITGB6 | 7.56E-01 | 1 | BUB1B | 1 | 1 |
| EMILIN2 | 7.56E-01 | 1 | BUB3 | 1 | 1 |
| ABR | 7.56E-01 | 1 | BUD31 | 1 | 1 |
| ZNF197 | 7.56E-01 | 1 | BVES | 1 | 1 |
| SCLT1 | 7.56E-01 | 1 | BYSL | 1 | 1 |
| SPINK5 | 7.56E-01 | 1 | BZW2 | 1 | 1 |
| SH3BP1 | 7.56E-01 | 1 | C10orf10 | 1 | 1 |
| DHRS3 | 7.57E-01 | 1 | C10orf105 | 1 | 1 |

| PTCH2 | 7.57E-01 | 1 | C10orf107 | 1 | 1 |
| --- | --- | --- | --- | --- | --- |
| TRO | 7.57E-01 | 1 | C10orf111 | 1 | 1 |
| AMBN | 7.57E-01 | 1 | C10orf114 | 1 | 1 |
| C8orf34 | 7.57E-01 | 1 | C10orf116 | 1 | 1 |
| OAS3 | 7.57E-01 | 1 | C10orf118 | 1 | 1 |
| KIF11 | 7.57E-01 | 1 | C10orf119 | 1 | 1 |
| HMGCS2 | 7.57E-01 | 1 | C10orf12 | 1 | 1 |
| RSBN1 | 7.57E-01 | 1 | C10orf120 | 1 | 1 |
| ENAM | 7.58E-01 | 1 | C10orf122 | 1 | 1 |
| TLR9 | 7.58E-01 | 1 | C10orf125 | 1 | 1 |
| PABPC1 | 7.58E-01 | 1 | C10orf128 | 1 | 1 |
| RFWD2 | 7.59E-01 | 1 | C10orf129 | 1 | 1 |
| ZNF460 | 7.59E-01 | 1 | C10orf131 | 1 | 1 |
| SLFN12 | 7.59E-01 | 1 | C10orf137 | 1 | 1 |
| AOC3 | 7.59E-01 | 1 | C10orf140 | 1 | 1 |
| KIF1B | 7.59E-01 | 1 | C10orf18 | 1 | 1 |
| ARAP1 | 7.59E-01 | 1 | C10orf2 | 1 | 1 |
| PLEKHG4 | 7.59E-01 | 1 | C10orf25 | 1 | 1 |
| ANTXR1 | 7.59E-01 | 1 | C10orf26 | 1 | 1 |
| WDR72 | 7.59E-01 | 1 | C10orf27 | 1 | 1 |
| ZFR | 7.59E-01 | 1 | C10orf28 | 1 | 1 |
| INO80 | 7.60E-01 | 1 | C10orf32 | 1 | 1 |
| ABCD4 | 7.60E-01 | 1 | C10orf35 | 1 | 1 |
| HIP1R | 7.60E-01 | 1 | C10orf46 | 1 | 1 |
| ZNF236 | 7.60E-01 | 1 | C10orf47 | 1 | 1 |
| CSMD2 | 7.60E-01 | 1 | C10orf53 | 1 | 1 |
| COL1A2 | 7.60E-01 | 1 | C10orf54 | 1 | 1 |
| AGAP4 | 7.60E-01 | 1 | C10orf55 | 1 | 1 |
| DAOA | 7.60E-01 | 1 | C10orf57 | 1 | 1 |
| ARRDC4 | 7.60E-01 | 1 | C10orf58 | 1 | 1 |
| CYBB | 7.61E-01 | 1 | C10orf62 | 1 | 1 |
| BOC | 7.61E-01 | 1 | C10orf67 | 1 | 1 |
| SALL1 | 7.61E-01 | 1 | C10orf68 | 1 | 1 |
| NOTCH1 | 7.61E-01 | 1 | C10orf72 | 1 | 1 |
| WDR48 | 7.61E-01 | 1 | C10orf76 | 1 | 1 |
| SON | 7.62E-01 | 1 | C10orf78 | 1 | 1 |
| EFR3B | 7.62E-01 | 1 | C10orf79 | 1 | 1 |
| PHIP | 7.62E-01 | 1 | C10orf81 | 1 | 1 |
| PRAMEF1 | 7.62E-01 | 1 | C10orf82 | 1 | 1 |
| PCNXL3 | 7.62E-01 | 1 | C10orf84 | 1 | 1 |
| PPM1H | 7.62E-01 | 1 | C10orf88 | 1 | 1 |
| TJP3 | 7.62E-01 | 1 | C10orf91 | 1 | 1 |
| EVPL | 7.62E-01 | 1 | C10orf93 | 1 | 1 |
| MAPK8IP | 7.62E-01 | 1 | C10orf95 | 1 | 1 |
| KIF27 | 7.63E-01 | 1 | C10orf96 | 1 | 1 |
| DSC2 | 7.63E-01 | 1 | C10orf99 | 1 | 1 |
| TMEM144 | 7.63E-01 | 1 | C11orf1 | 1 | 1 |
| TPTE2 | 7.63E-01 | 1 | C11orf10 | 1 | 1 |
| KRTAP13 | 7.63E-01 | 1 | C11orf16 | 1 | 1 |
| HOOK1 | 7.63E-01 | 1 | C11orf17 | 1 | 1 |
| TGS1 | 7.63E-01 | 1 | C11orf2 | 1 | 1 |
| TTLL4 | 7.63E-01 | 1 | C11orf20 | 1 | 1 |
| ZNF320 | 7.63E-01 | 1 | C11orf21 | 1 | 1 |

| CSRP2BP | 7.64E-01 | 1 | C11orf30 | 1 | 1 |
| --- | --- | --- | --- | --- | --- |
| SEMA4D | 7.64E-01 | 1 | C11orf31 | 1 | 1 |
| RECQL5 | 7.64E-01 | 1 | C11orf34 | 1 | 1 |
| MRVI1 | 7.64E-01 | 1 | C11orf35 | 1 | 1 |
| LIN54 | 7.64E-01 | 1 | C11orf40 | 1 | 1 |
| HDAC1 | 7.64E-01 | 1 | C11orf41 | 1 | 1 |
| NLGN2 | 7.64E-01 | 1 | C11orf42 | 1 | 1 |
| MTBP | 7.64E-01 | 1 | C11orf45 | 1 | 1 |
| DOCK3 | 7.65E-01 | 1 | C11orf46 | 1 | 1 |
| SNX7 | 7.65E-01 | 1 | C11orf48 | 1 | 1 |
| IQGAP1 | 7.65E-01 | 1 | C11orf51 | 1 | 1 |
| MYO5A | 7.66E-01 | 1 | C11orf52 | 1 | 1 |
| NAT10 | 7.66E-01 | 1 | C11orf53 | 1 | 1 |
| PARN | 7.66E-01 | 1 | C11orf54 | 1 | 1 |
| SART3 | 7.66E-01 | 1 | C11orf58 | 1 | 1 |
| C16orf62 | 7.66E-01 | 1 | C11orf59 | 1 | 1 |
| SPAG9 | 7.66E-01 | 1 | C11orf61 | 1 | 1 |
| KIF1A | 7.66E-01 | 1 | C11orf63 | 1 | 1 |
| LIPI | 7.66E-01 | 1 | C11orf65 | 1 | 1 |
| ANAPC4 | 7.66E-01 | 1 | C11orf66 | 1 | 1 |
| SLC8A3 | 7.66E-01 | 1 | C11orf67 | 1 | 1 |
| DGKD | 7.67E-01 | 1 | C11orf68 | 1 | 1 |
| ABCC10 | 7.67E-01 | 1 | C11orf71 | 1 | 1 |
| UBE3B | 7.67E-01 | 1 | C11orf73 | 1 | 1 |
| SCIN | 7.67E-01 | 1 | C11orf74 | 1 | 1 |
| THSD7B | 7.67E-01 | 1 | C11orf75 | 1 | 1 |
| PNPLA6 | 7.67E-01 | 1 | C11orf82 | 1 | 1 |
| ABTB2 | 7.67E-01 | 1 | C11orf83 | 1 | 1 |
| KIF24 | 7.67E-01 | 1 | C11orf85 | 1 | 1 |
| TBX5 | 7.67E-01 | 1 | C11orf86 | 1 | 1 |
| SCN2A | 7.67E-01 | 1 | C11orf88 | 1 | 1 |
| SLC6A12 | 7.68E-01 | 1 | C11orf9 | 1 | 1 |
| NTNG1 | 7.68E-01 | 1 | C11orf90 | 1 | 1 |
| USP29 | 7.68E-01 | 1 | C11orf91 | 1 | 1 |
| PCDHB15 | 7.68E-01 | 1 | C11orf92 | 1 | 1 |
| IL27RA | 7.69E-01 | 1 | C11orf93 | 1 | 1 |
| NSMAF | 7.69E-01 | 1 | C11orf94 | 1 | 1 |
| ZNF211 | 7.69E-01 | 1 | C12orf10 | 1 | 1 |
| LRRK2 | 7.69E-01 | 1 | C12orf11 | 1 | 1 |
| MBTPS1 | 7.69E-01 | 1 | C12orf12 | 1 | 1 |
| ASZ1 | 7.70E-01 | 1 | C12orf23 | 1 | 1 |
| WDR70 | 7.70E-01 | 1 | C12orf24 | 1 | 1 |
| CYP2R1 | 7.70E-01 | 1 | C12orf26 | 1 | 1 |
| SHC4 | 7.70E-01 | 1 | C12orf29 | 1 | 1 |
| SIGLEC5 | 7.70E-01 | 1 | C12orf34 | 1 | 1 |
| ZDHHC17 | 7.70E-01 | 1 | C12orf35 | 1 | 1 |
| KDM1A | 7.71E-01 | 1 | C12orf36 | 1 | 1 |
| DQX1 | 7.71E-01 | 1 | C12orf39 | 1 | 1 |
| PMS2 | 7.71E-01 | 1 | C12orf41 | 1 | 1 |
| ASB15 | 7.71E-01 | 1 | C12orf43 | 1 | 1 |
| ZNF549 | 7.71E-01 | 1 | C12orf44 | 1 | 1 |
| SLC18A1 | 7.72E-01 | 1 | C12orf45 | 1 | 1 |
| CASKIN2 | 7.72E-01 | 1 | C12orf48 | 1 | 1 |

| ACSM3 | 7.72E-01 | 1 | C12orf49 | 1 | 1 |
| --- | --- | --- | --- | --- | --- |
| ZNF700 | 7.72E-01 | 1 | C12orf5 | 1 | 1 |
| KLHDC7A | 7.73E-01 | 1 | C12orf50 | 1 | 1 |
| DOCK1 | 7.73E-01 | 1 | C12orf51 | 1 | 1 |
| TMC2 | 7.73E-01 | 1 | C12orf52 | 1 | 1 |
| SLITRK2 | 7.73E-01 | 1 | C12orf53 | 1 | 1 |
| MMP2 | 7.74E-01 | 1 | C12orf54 | 1 | 1 |
| IGF1R | 7.74E-01 | 1 | C12orf57 | 1 | 1 |
| FAM9A | 7.74E-01 | 1 | C12orf59 | 1 | 1 |
| ZNF292 | 7.74E-01 | 1 | C12orf60 | 1 | 1 |
| PKHD1 | 7.74E-01 | 1 | C12orf61 | 1 | 1 |
| ROBO2 | 7.74E-01 | 1 | C12orf62 | 1 | 1 |
| NEFM | 7.74E-01 | 1 | C12orf63 | 1 | 1 |
| DNAH7 | 7.74E-01 | 1 | C12orf64 | 1 | 1 |
| LRRTM2 | 7.74E-01 | 1 | C12orf65 | 1 | 1 |
| TRPM6 | 7.74E-01 | 1 | C12orf66 | 1 | 1 |
| RANBP9 | 7.74E-01 | 1 | C12orf68 | 1 | 1 |
| SOHLH2 | 7.74E-01 | 1 | C12orf69 | 1 | 1 |
| FLG | 7.75E-01 | 1 | C12orf70 | 1 | 1 |
| PPAN-P2R | 7.75E-01 | 1 | C12orf71 | 1 | 1 |
| XPO4 | 7.75E-01 | 1 | C12orf72 | 1 | 1 |
| PTPN12 | 7.75E-01 | 1 | C12orf73 | 1 | 1 |
| PPARGC1 | 7.75E-01 | 1 | C12orf74 | 1 | 1 |
| MYBL2 | 7.76E-01 | 1 | C12orf75 | 1 | 1 |
| CLEC16A | 7.76E-01 | 1 | C12orf76 | 1 | 1 |
| ADAMTS | 7.76E-01 | 1 | C12orf77 | 1 | 1 |
| KRT9 | 7.76E-01 | 1 | C13orf1 | 1 | 1 |
| ADAMTS | 7.76E-01 | 1 | C13orf15 | 1 | 1 |
| PCDH8 | 7.76E-01 | 1 | C13orf16 | 1 | 1 |
| MYO1A | 7.76E-01 | 1 | C13orf18 | 1 | 1 |
| GPAM | 7.76E-01 | 1 | C13orf23 | 1 | 1 |
| TUBB8 | 7.77E-01 | 1 | C13orf26 | 1 | 1 |
| ZNF432 | 7.77E-01 | 1 | C13orf27 | 1 | 1 |
| DAGLA | 7.77E-01 | 1 | C13orf28 | 1 | 1 |
| UBAC1 | 7.77E-01 | 1 | C13orf30 | 1 | 1 |
| EML1 | 7.78E-01 | 1 | C13orf31 | 1 | 1 |
| SLC4A10 | 7.78E-01 | 1 | C13orf33 | 1 | 1 |
| LMBR1 | 7.78E-01 | 1 | C13orf34 | 1 | 1 |
| SLC34A3 | 7.78E-01 | 1 | C13orf35 | 1 | 1 |
| TSGA10 | 7.78E-01 | 1 | C13orf36 | 1 | 1 |
| PCNT | 7.78E-01 | 1 | C13orf37 | 1 | 1 |
| DYNC1H1 | 7.78E-01 | 1 | C13orf38 | 1 | 1 |
| AMBRA1 | 7.78E-01 | 1 | C13orf39 | 1 | 1 |
| MED23 | 7.79E-01 | 1 | C14orf1 | 1 | 1 |
| MAMDC2 | 7.79E-01 | 1 | C14orf101 | 1 | 1 |
| ZNF586 | 7.79E-01 | 1 | C14orf102 | 1 | 1 |
| AP4E1 | 7.79E-01 | 1 | C14orf104 | 1 | 1 |
| UBTF | 7.79E-01 | 1 | C14orf105 | 1 | 1 |
| GANAB | 7.79E-01 | 1 | C14orf106 | 1 | 1 |
| ZFYVE26 | 7.79E-01 | 1 | C14orf109 | 1 | 1 |
| TGM4 | 7.79E-01 | 1 | C14orf115 | 1 | 1 |
| ARVCF | 7.79E-01 | 1 | C14orf118 | 1 | 1 |
| ZNF483 | 7.80E-01 | 1 | C14orf119 | 1 | 1 |

| COL4A6 | 7.80E-01 | 1 | C14orf126 | 1 | 1 |
| --- | --- | --- | --- | --- | --- |
| PIK3AP1 | 7.80E-01 | 1 | C14orf129 | 1 | 1 |
| CHRNA1 | 7.80E-01 | 1 | C14orf135 | 1 | 1 |
| ITGAL | 7.80E-01 | 1 | C14orf138 | 1 | 1 |
| SNX14 | 7.80E-01 | 1 | C14orf142 | 1 | 1 |
| UBA7 | 7.80E-01 | 1 | C14orf143 | 1 | 1 |
| BCAS3 | 7.80E-01 | 1 | C14orf145 | 1 | 1 |
| DIP2C | 7.81E-01 | 1 | C14orf147 | 1 | 1 |
| LAS1L | 7.81E-01 | 1 | C14orf148 | 1 | 1 |
| AKAP8L | 7.81E-01 | 1 | C14orf149 | 1 | 1 |
| CAPN7 | 7.81E-01 | 1 | C14orf153 | 1 | 1 |
| USP36 | 7.82E-01 | 1 | C14orf156 | 1 | 1 |
| SUN2 | 7.82E-01 | 1 | C14orf159 | 1 | 1 |
| HHIPL2 | 7.82E-01 | 1 | C14orf166B | 1 | 1 |
| DNAJC21 | 7.82E-01 | 1 | C14orf174 | 1 | 1 |
| CACNA1A | 7.82E-01 | 1 | C14orf176 | 1 | 1 |
| PCDH20 | 7.82E-01 | 1 | C14orf177 | 1 | 1 |
| EIF4G3 | 7.82E-01 | 1 | C14orf178 | 1 | 1 |
| IPO11 | 7.82E-01 | 1 | C14orf179 | 1 | 1 |
| ECM2 | 7.82E-01 | 1 | C14orf180 | 1 | 1 |
| PKDREJ | 7.82E-01 | 1 | C14orf181 | 1 | 1 |
| MCM6 | 7.82E-01 | 1 | C14orf182 | 1 | 1 |
| PEG3 | 7.83E-01 | 1 | C14orf183 | 1 | 1 |
| POLR3E | 7.83E-01 | 1 | C14orf184 | 1 | 1 |
| KCNV2 | 7.83E-01 | 1 | C14orf2 | 1 | 1 |
| OR4A16 | 7.83E-01 | 1 | C14orf21 | 1 | 1 |
| CNGA4 | 7.84E-01 | 1 | C14orf28 | 1 | 1 |
| CADM1 | 7.84E-01 | 1 | C14orf38 | 1 | 1 |
| CRB1 | 7.84E-01 | 1 | C14orf4 | 1 | 1 |
| ROR2 | 7.84E-01 | 1 | C14orf43 | 1 | 1 |
| BAIAP3 | 7.84E-01 | 1 | C14orf45 | 1 | 1 |
| EIF4ENIF1 | 7.84E-01 | 1 | C14orf49 | 1 | 1 |
| MASP1 | 7.84E-01 | 1 | C14orf50 | 1 | 1 |
| NEIL3 | 7.85E-01 | 1 | C14orf68 | 1 | 1 |
| NGLY1 | 7.85E-01 | 1 | C14orf73 | 1 | 1 |
| PWWP2A | 7.85E-01 | 1 | C14orf79 | 1 | 1 |
| DLG3 | 7.85E-01 | 1 | C14orf80 | 1 | 1 |
| STAT6 | 7.85E-01 | 1 | C14orf93 | 1 | 1 |
| CEP135 | 7.85E-01 | 1 | C15orf17 | 1 | 1 |
| ATIC | 7.85E-01 | 1 | C15orf2 | 1 | 1 |
| GTF3C1 | 7.85E-01 | 1 | C15orf23 | 1 | 1 |
| HELZ | 7.85E-01 | 1 | C15orf24 | 1 | 1 |
| TNKS | 7.85E-01 | 1 | C15orf26 | 1 | 1 |
| FBXW7 | 7.85E-01 | 1 | C15orf27 | 1 | 1 |
| GALNTL6 | 7.85E-01 | 1 | C15orf29 | 1 | 1 |
| FRMPD2 | 7.85E-01 | 1 | C15orf32 | 1 | 1 |
| KIF13A | 7.86E-01 | 1 | C15orf33 | 1 | 1 |
| MAGEC1 | 7.86E-01 | 1 | C15orf38 | 1 | 1 |
| ZNF98 | 7.86E-01 | 1 | C15orf39 | 1 | 1 |
| NUP93 | 7.86E-01 | 1 | C15orf40 | 1 | 1 |
| ZBTB38 | 7.86E-01 | 1 | C15orf41 | 1 | 1 |
| MFN2 | 7.86E-01 | 1 | C15orf42 | 1 | 1 |
| MICAL2 | 7.86E-01 | 1 | C15orf44 | 1 | 1 |

| TLE3 | 7.86E-01 | 1 | C15orf48 | 1 | 1 |
| --- | --- | --- | --- | --- | --- |
| AGBL3 | 7.86E-01 | 1 | C15orf52 | 1 | 1 |
| PHC2 | 7.86E-01 | 1 | C15orf54 | 1 | 1 |
| CHIA | 7.86E-01 | 1 | C15orf55 | 1 | 1 |
| PGM5 | 7.86E-01 | 1 | C15orf56 | 1 | 1 |
| CCT6A | 7.87E-01 | 1 | C15orf57 | 1 | 1 |
| PDE10A | 7.87E-01 | 1 | C15orf58 | 1 | 1 |
| OTOF | 7.87E-01 | 1 | C15orf59 | 1 | 1 |
| UBE4A | 7.87E-01 | 1 | C15orf60 | 1 | 1 |
| SNAP91 | 7.87E-01 | 1 | C15orf61 | 1 | 1 |
| AMOTL2 | 7.87E-01 | 1 | C15orf62 | 1 | 1 |
| KIAA1324 | 7.87E-01 | 1 | C15orf63 | 1 | 1 |
| PTK7 | 7.88E-01 | 1 | C16orf11 | 1 | 1 |
| PRAMEF1 | 7.88E-01 | 1 | C16orf13 | 1 | 1 |
| SNX19 | 7.88E-01 | 1 | C16orf3 | 1 | 1 |
| RAVER1 | 7.88E-01 | 1 | C16orf42 | 1 | 1 |
| NAALAD | 7.88E-01 | 1 | C16orf45 | 1 | 1 |
| ZFP91 | 7.88E-01 | 1 | C16orf46 | 1 | 1 |
| ZNF208 | 7.88E-01 | 1 | C16orf48 | 1 | 1 |
| TBC1D5 | 7.88E-01 | 1 | C16orf5 | 1 | 1 |
| ZNF234 | 7.89E-01 | 1 | C16orf52 | 1 | 1 |
| SMO | 7.89E-01 | 1 | C16orf53 | 1 | 1 |
| ROR1 | 7.89E-01 | 1 | C16orf54 | 1 | 1 |
| TMC1 | 7.89E-01 | 1 | C16orf55 | 1 | 1 |
| LRRC7 | 7.89E-01 | 1 | C16orf57 | 1 | 1 |
| KLHL29 | 7.89E-01 | 1 | C16orf58 | 1 | 1 |
| GJA10 | 7.89E-01 | 1 | C16orf59 | 1 | 1 |
| CLMN | 7.89E-01 | 1 | C16orf61 | 1 | 1 |
| FLNC | 7.89E-01 | 1 | C16orf62 | 1 | 1 |
| RTKN | 7.89E-01 | 1 | C16orf63 | 1 | 1 |
| IFT140 | 7.89E-01 | 1 | C16orf68 | 1 | 1 |
| DDX26B | 7.89E-01 | 1 | C16orf7 | 1 | 1 |
| CHM | 7.89E-01 | 1 | C16orf71 | 1 | 1 |
| TECPR1 | 7.89E-01 | 1 | C16orf72 | 1 | 1 |
| LRCH1 | 7.89E-01 | 1 | C16orf73 | 1 | 1 |
| RGAG1 | 7.90E-01 | 1 | C16orf74 | 1 | 1 |
| PRAMEF7 | 7.90E-01 | 1 | C16orf75 | 1 | 1 |
| NF1 | 7.90E-01 | 1 | C16orf78 | 1 | 1 |
| SYNJ1 | 7.90E-01 | 1 | C16orf79 | 1 | 1 |
| AP3B2 | 7.90E-01 | 1 | C16orf80 | 1 | 1 |
| PDE5A | 7.91E-01 | 1 | C16orf82 | 1 | 1 |
| ZNF845 | 7.91E-01 | 1 | C16orf86 | 1 | 1 |
| NAIP | 7.91E-01 | 1 | C16orf87 | 1 | 1 |
| E2F8 | 7.91E-01 | 1 | C16orf88 | 1 | 1 |
| PCM1 | 7.91E-01 | 1 | C16orf90 | 1 | 1 |
| CPEB2 | 7.91E-01 | 1 | C16orf91 | 1 | 1 |
| ZFHX4 | 7.91E-01 | 1 | C16orf92 | 1 | 1 |
| RYR1 | 7.92E-01 | 1 | C16orf93 | 1 | 1 |
| ZNF354B | 7.92E-01 | 1 | C17orf100 | 1 | 1 |
| SHROOM | 7.92E-01 | 1 | C17orf101 | 1 | 1 |
| ZNF12 | 7.92E-01 | 1 | C17orf102 | 1 | 1 |
| ZNF469 | 7.92E-01 | 1 | C17orf103 | 1 | 1 |
| MDN1 | 7.92E-01 | 1 | C17orf104 | 1 | 1 |

| FBXW10 | 7.92E-01 | 1 | C17orf105 | 1 | 1 |
| --- | --- | --- | --- | --- | --- |
| ZNF658 | 7.93E-01 | 1 | C17orf106 | 1 | 1 |
| CCDC102 | 7.93E-01 | 1 | C17orf107 | 1 | 1 |
| CLSTN3 | 7.93E-01 | 1 | C17orf108 | 1 | 1 |
| OSBPL5 | 7.93E-01 | 1 | C17orf28 | 1 | 1 |
| OBSL1 | 7.93E-01 | 1 | C17orf37 | 1 | 1 |
| PTPRG | 7.93E-01 | 1 | C17orf39 | 1 | 1 |
| TNFRSF4 | 7.93E-01 | 1 | C17orf42 | 1 | 1 |
| TLR7 | 7.93E-01 | 1 | C17orf46 | 1 | 1 |
| DCC | 7.94E-01 | 1 | C17orf47 | 1 | 1 |
| PLXND1 | 7.94E-01 | 1 | C17orf48 | 1 | 1 |
| PRDM10 | 7.94E-01 | 1 | C17orf49 | 1 | 1 |
| ADNP | 7.94E-01 | 1 | C17orf50 | 1 | 1 |
| MTMR1 | 7.94E-01 | 1 | C17orf51 | 1 | 1 |
| MAST4 | 7.94E-01 | 1 | C17orf53 | 1 | 1 |
| EIF4G2 | 7.94E-01 | 1 | C17orf55 | 1 | 1 |
| NOD1 | 7.94E-01 | 1 | C17orf56 | 1 | 1 |
| HPSE2 | 7.95E-01 | 1 | C17orf57 | 1 | 1 |
| LOXL3 | 7.95E-01 | 1 | C17orf58 | 1 | 1 |
| AMY2A | 7.95E-01 | 1 | C17orf59 | 1 | 1 |
| SLC4A11 | 7.95E-01 | 1 | C17orf60 | 1 | 1 |
| FRMD4A | 7.96E-01 | 1 | C17orf61 | 1 | 1 |
| DMXL1 | 7.96E-01 | 1 | C17orf62 | 1 | 1 |
| SYNM | 7.96E-01 | 1 | C17orf63 | 1 | 1 |
| GGNBP2 | 7.96E-01 | 1 | C17orf65 | 1 | 1 |
| ZMYM2 | 7.96E-01 | 1 | C17orf66 | 1 | 1 |
| GPC3 | 7.96E-01 | 1 | C17orf67 | 1 | 1 |
| PARD3B | 7.96E-01 | 1 | C17orf68 | 1 | 1 |
| SLC38A10 | 7.96E-01 | 1 | C17orf71 | 1 | 1 |
| SLC17A6 | 7.96E-01 | 1 | C17orf72 | 1 | 1 |
| TP53BP2 | 7.96E-01 | 1 | C17orf74 | 1 | 1 |
| NOVA1 | 7.97E-01 | 1 | C17orf75 | 1 | 1 |
| C9orf3 | 7.97E-01 | 1 | C17orf76 | 1 | 1 |
| NFASC | 7.97E-01 | 1 | C17orf77 | 1 | 1 |
| EIF3D | 7.98E-01 | 1 | C17orf78 | 1 | 1 |
| DPYSL3 | 7.98E-01 | 1 | C17orf79 | 1 | 1 |
| ITGAE | 7.98E-01 | 1 | C17orf80 | 1 | 1 |
| TDRD1 | 7.98E-01 | 1 | C17orf81 | 1 | 1 |
| SLC26A5 | 7.98E-01 | 1 | C17orf82 | 1 | 1 |
| FZD1 | 7.98E-01 | 1 | C17orf85 | 1 | 1 |
| KCNJ16 | 7.98E-01 | 1 | C17orf87 | 1 | 1 |
| RAB3GAP | 7.99E-01 | 1 | C17orf89 | 1 | 1 |
| C3orf17 | 7.99E-01 | 1 | C17orf90 | 1 | 1 |
| NLRP4 | 7.99E-01 | 1 | C17orf95 | 1 | 1 |
| PLOD1 | 7.99E-01 | 1 | C17orf97 | 1 | 1 |
| EPHA7 | 7.99E-01 | 1 | C17orf98 | 1 | 1 |
| CHAF1A | 7.99E-01 | 1 | C17orf99 | 1 | 1 |
| ZFC3H1 | 8.00E-01 | 1 | C18orf1 | 1 | 1 |
| KRT77 | 8.00E-01 | 1 | C18orf10 | 1 | 1 |
| ZNF532 | 8.00E-01 | 1 | C18orf19 | 1 | 1 |
| TIE1 | 8.00E-01 | 1 | C18orf21 | 1 | 1 |
| NPAS4 | 8.00E-01 | 1 | C18orf22 | 1 | 1 |
| YTHDC2 | 8.00E-01 | 1 | C18orf26 | 1 | 1 |

| GP6 | 8.01E-01 | 1 | C18orf32 | 1 | 1 |
| --- | --- | --- | --- | --- | --- |
| NLRP2 | 8.01E-01 | 1 | C18orf34 | 1 | 1 |
| ABCG8 | 8.01E-01 | 1 | C18orf45 | 1 | 1 |
| UGT2B15 | 8.01E-01 | 1 | C18orf55 | 1 | 1 |
| TERT | 8.01E-01 | 1 | C18orf56 | 1 | 1 |
| ELOVL4 | 8.01E-01 | 1 | C18orf62 | 1 | 1 |
| ARHGAP3 | 8.01E-01 | 1 | C18orf8 | 1 | 1 |
| OR5K4 | 8.01E-01 | 1 | C19orf10 | 1 | 1 |
| COL4A3 | 8.02E-01 | 1 | C19orf12 | 1 | 1 |
| PI4KA | 8.02E-01 | 1 | C19orf18 | 1 | 1 |
| IKBKE | 8.02E-01 | 1 | C19orf2 | 1 | 1 |
| SI | 8.02E-01 | 1 | C19orf20 | 1 | 1 |
| RASGRP1 | 8.02E-01 | 1 | C19orf21 | 1 | 1 |
| FBXO10 | 8.03E-01 | 1 | C19orf22 | 1 | 1 |
| PIK3R4 | 8.03E-01 | 1 | C19orf24 | 1 | 1 |
| ANKS1B | 8.03E-01 | 1 | C19orf25 | 1 | 1 |
| LUZP2 | 8.03E-01 | 1 | C19orf26 | 1 | 1 |
| CUL2 | 8.03E-01 | 1 | C19orf28 | 1 | 1 |
| UBAP2L | 8.03E-01 | 1 | C19orf29 | 1 | 1 |
| MACF1 | 8.03E-01 | 1 | C19orf33 | 1 | 1 |
| SCAPER | 8.03E-01 | 1 | C19orf35 | 1 | 1 |
| NINL | 8.03E-01 | 1 | C19orf36 | 1 | 1 |
| ARAP2 | 8.04E-01 | 1 | C19orf38 | 1 | 1 |
| MON1B | 8.04E-01 | 1 | C19orf39 | 1 | 1 |
| TBC1D8 | 8.04E-01 | 1 | C19orf40 | 1 | 1 |
| CDKL5 | 8.04E-01 | 1 | C19orf41 | 1 | 1 |
| AIM1 | 8.05E-01 | 1 | C19orf42 | 1 | 1 |
| TAS1R2 | 8.05E-01 | 1 | C19orf43 | 1 | 1 |
| ZC3H13 | 8.05E-01 | 1 | C19orf44 | 1 | 1 |
| HSH2D | 8.05E-01 | 1 | C19orf45 | 1 | 1 |
| NBPF9 | 8.05E-01 | 1 | C19orf46 | 1 | 1 |
| IGSF5 | 8.05E-01 | 1 | C19orf47 | 1 | 1 |
| NFATC3 | 8.05E-01 | 1 | C19orf48 | 1 | 1 |
| CSF2RB | 8.05E-01 | 1 | C19orf50 | 1 | 1 |
| MYOF | 8.05E-01 | 1 | C19orf51 | 1 | 1 |
| ZNF350 | 8.06E-01 | 1 | C19orf52 | 1 | 1 |
| EP400 | 8.06E-01 | 1 | C19orf53 | 1 | 1 |
| ADCY8 | 8.06E-01 | 1 | C19orf54 | 1 | 1 |
| COPB1 | 8.06E-01 | 1 | C19orf55 | 1 | 1 |
| USP43 | 8.06E-01 | 1 | C19orf56 | 1 | 1 |
| OR5AS1 | 8.06E-01 | 1 | C19orf57 | 1 | 1 |
| WWOX | 8.06E-01 | 1 | C19orf59 | 1 | 1 |
| FZD10 | 8.06E-01 | 1 | C19orf6 | 1 | 1 |
| SLC6A2 | 8.07E-01 | 1 | C19orf60 | 1 | 1 |
| NOSTRIN | 8.07E-01 | 1 | C19orf61 | 1 | 1 |
| NLRP1 | 8.07E-01 | 1 | C19orf62 | 1 | 1 |
| ZNF667 | 8.07E-01 | 1 | C19orf63 | 1 | 1 |
| MYH7 | 8.07E-01 | 1 | C19orf66 | 1 | 1 |
| ANKRD32 | 8.07E-01 | 1 | C19orf69 | 1 | 1 |
| CPEB1 | 8.08E-01 | 1 | C19orf70 | 1 | 1 |
| PCDH9 | 8.08E-01 | 1 | C19orf71 | 1 | 1 |
| SEMA6A | 8.08E-01 | 1 | C19orf73 | 1 | 1 |
| RBBP8 | 8.08E-01 | 1 | C19orf75 | 1 | 1 |

| CYFIP1 | 8.09E-01 | 1 | C19orf76 | 1 | 1 |
| --- | --- | --- | --- | --- | --- |
| LRRIQ4 | 8.09E-01 | 1 | C19orf77 | 1 | 1 |
| LCT | 8.09E-01 | 1 | C1D | 1 | 1 |
| LRFN5 | 8.09E-01 | 1 | C1QB | 1 | 1 |
| DLG4 | 8.09E-01 | 1 | C1QBP | 1 | 1 |
| RSPH6A | 8.09E-01 | 1 | C1QC | 1 | 1 |
| PCDHGA2 | 8.09E-01 | 1 | C1QL1 | 1 | 1 |
| SELE | 8.09E-01 | 1 | C1QL2 | 1 | 1 |
| PCDHB5 | 8.10E-01 | 1 | C1QL3 | 1 | 1 |
| ATP2A1 | 8.10E-01 | 1 | C1QL4 | 1 | 1 |
| NHSL1 | 8.11E-01 | 1 | C1QTNF2 | 1 | 1 |
| ZNF445 | 8.11E-01 | 1 | C1QTNF3 | 1 | 1 |
| AKAP6 | 8.11E-01 | 1 | C1QTNF4 | 1 | 1 |
| ELAC2 | 8.11E-01 | 1 | C1QTNF5 | 1 | 1 |
| SV2A | 8.11E-01 | 1 | C1QTNF6 | 1 | 1 |
| ETAA1 | 8.11E-01 | 1 | C1QTNF7 | 1 | 1 |
| COL6A2 | 8.12E-01 | 1 | C1QTNF8 | 1 | 1 |
| ITIH2 | 8.12E-01 | 1 | C1QTNF9 | 1 | 1 |
| DSCAM | 8.12E-01 | 1 | C1R | 1 | 1 |
| ITFG1 | 8.12E-01 | 1 | C1orf100 | 1 | 1 |
| ZNF256 | 8.12E-01 | 1 | C1orf101 | 1 | 1 |
| ARMCX6 | 8.12E-01 | 1 | C1orf103 | 1 | 1 |
| MAP3K9 | 8.12E-01 | 1 | C1orf104 | 1 | 1 |
| ZC3H18 | 8.12E-01 | 1 | C1orf105 | 1 | 1 |
| AGBL4 | 8.13E-01 | 1 | C1orf107 | 1 | 1 |
| PROS1 | 8.13E-01 | 1 | C1orf109 | 1 | 1 |
| SEMA5B | 8.13E-01 | 1 | C1orf110 | 1 | 1 |
| ANKLE2 | 8.13E-01 | 1 | C1orf112 | 1 | 1 |
| CD163L1 | 8.13E-01 | 1 | C1orf113 | 1 | 1 |
| RABEP1 | 8.14E-01 | 1 | C1orf114 | 1 | 1 |
| ZMYM1 | 8.14E-01 | 1 | C1orf115 | 1 | 1 |
| UGT2B10 | 8.14E-01 | 1 | C1orf116 | 1 | 1 |
| MLXIP | 8.14E-01 | 1 | C1orf122 | 1 | 1 |
| SLCO6A1 | 8.14E-01 | 1 | C1orf123 | 1 | 1 |
| PCDHGA3 | 8.15E-01 | 1 | C1orf124 | 1 | 1 |
| ZMIZ1 | 8.15E-01 | 1 | C1orf125 | 1 | 1 |
| PCDH15 | 8.15E-01 | 1 | C1orf128 | 1 | 1 |
| EIF2AK3 | 8.15E-01 | 1 | C1orf129 | 1 | 1 |
| OGDHL | 8.15E-01 | 1 | C1orf130 | 1 | 1 |
| NEURL4 | 8.16E-01 | 1 | C1orf131 | 1 | 1 |
| RALGAPB | 8.16E-01 | 1 | C1orf135 | 1 | 1 |
| ZNF512B | 8.16E-01 | 1 | C1orf14 | 1 | 1 |
| KNTC1 | 8.16E-01 | 1 | C1orf141 | 1 | 1 |
| CNTN4 | 8.16E-01 | 1 | C1orf144 | 1 | 1 |
| ZNF534 | 8.17E-01 | 1 | C1orf150 | 1 | 1 |
| PEX1 | 8.17E-01 | 1 | C1orf151 | 1 | 1 |
| UTY | 8.17E-01 | 1 | C1orf156 | 1 | 1 |
| MMP8 | 8.17E-01 | 1 | C1orf158 | 1 | 1 |
| KLB | 8.17E-01 | 1 | C1orf159 | 1 | 1 |
| DDX20 | 8.17E-01 | 1 | C1orf161 | 1 | 1 |
| ULK1 | 8.17E-01 | 1 | C1orf162 | 1 | 1 |
| NLRP10 | 8.17E-01 | 1 | C1orf163 | 1 | 1 |
| TSHZ2 | 8.17E-01 | 1 | C1orf168 | 1 | 1 |

| KANK4 | 8.18E-01 | 1 | C1orf172 | 1 | 1 |
| --- | --- | --- | --- | --- | --- |
| COPB2 | 8.18E-01 | 1 | C1orf173 | 1 | 1 |
| EXOC6 | 8.18E-01 | 1 | C1orf174 | 1 | 1 |
| ZNF75D | 8.18E-01 | 1 | C1orf175 | 1 | 1 |
| SPG11 | 8.19E-01 | 1 | C1orf177 | 1 | 1 |
| OSBP | 8.19E-01 | 1 | C1orf182 | 1 | 1 |
| MYO5C | 8.20E-01 | 1 | C1orf183 | 1 | 1 |
| ZNF420 | 8.20E-01 | 1 | C1orf185 | 1 | 1 |
| ZNF582 | 8.20E-01 | 1 | C1orf186 | 1 | 1 |
| DCLRE1A | 8.20E-01 | 1 | C1orf187 | 1 | 1 |
| ABCC5 | 8.21E-01 | 1 | C1orf189 | 1 | 1 |
| ZBTB4 | 8.21E-01 | 1 | C1orf190 | 1 | 1 |
| SETDB1 | 8.21E-01 | 1 | C1orf192 | 1 | 1 |
| STARD13 | 8.21E-01 | 1 | C1orf194 | 1 | 1 |
| CAPRIN2 | 8.21E-01 | 1 | C1orf198 | 1 | 1 |
| TBL1X | 8.21E-01 | 1 | C1orf201 | 1 | 1 |
| VWA3A | 8.21E-01 | 1 | C1orf204 | 1 | 1 |
| PODXL2 | 8.22E-01 | 1 | C1orf21 | 1 | 1 |
| FAM102B | 8.22E-01 | 1 | C1orf210 | 1 | 1 |
| FBRSL1 | 8.22E-01 | 1 | C1orf212 | 1 | 1 |
| HK2 | 8.22E-01 | 1 | C1orf213 | 1 | 1 |
| OR10Z1 | 8.22E-01 | 1 | C1orf216 | 1 | 1 |
| MYH2 | 8.22E-01 | 1 | C1orf223 | 1 | 1 |
| MERTK | 8.23E-01 | 1 | C1orf226 | 1 | 1 |
| NR1I2 | 8.23E-01 | 1 | C1orf227 | 1 | 1 |
| RFX4 | 8.23E-01 | 1 | C1orf228 | 1 | 1 |
| FAM196A | 8.24E-01 | 1 | C1orf229 | 1 | 1 |
| CD101 | 8.24E-01 | 1 | C1orf230 | 1 | 1 |
| WDR66 | 8.24E-01 | 1 | C1orf25 | 1 | 1 |
| MTHFR | 8.24E-01 | 1 | C1orf26 | 1 | 1 |
| PHKB | 8.24E-01 | 1 | C1orf31 | 1 | 1 |
| GBP6 | 8.25E-01 | 1 | C1orf35 | 1 | 1 |
| DACH2 | 8.25E-01 | 1 | C1orf38 | 1 | 1 |
| ITGA4 | 8.25E-01 | 1 | C1orf43 | 1 | 1 |
| MED13L | 8.25E-01 | 1 | C1orf49 | 1 | 1 |
| FNDC3B | 8.25E-01 | 1 | C1orf50 | 1 | 1 |
| ZNF568 | 8.25E-01 | 1 | C1orf51 | 1 | 1 |
| WDR90 | 8.25E-01 | 1 | C1orf52 | 1 | 1 |
| PARP10 | 8.25E-01 | 1 | C1orf54 | 1 | 1 |
| WDR35 | 8.26E-01 | 1 | C1orf55 | 1 | 1 |
| COL14A1 | 8.26E-01 | 1 | C1orf56 | 1 | 1 |
| FZD8 | 8.26E-01 | 1 | C1orf57 | 1 | 1 |
| PCDHGB6 | 8.26E-01 | 1 | C1orf58 | 1 | 1 |
| F13B | 8.26E-01 | 1 | C1orf59 | 1 | 1 |
| PICALM | 8.26E-01 | 1 | C1orf63 | 1 | 1 |
| TAOK2 | 8.27E-01 | 1 | C1orf64 | 1 | 1 |
| TBK1 | 8.27E-01 | 1 | C1orf65 | 1 | 1 |
| MYCBP2 | 8.28E-01 | 1 | C1orf66 | 1 | 1 |
| PCDHB4 | 8.28E-01 | 1 | C1orf69 | 1 | 1 |
| SLC12A3 | 8.28E-01 | 1 | C1orf70 | 1 | 1 |
| OR5H1 | 8.28E-01 | 1 | C1orf74 | 1 | 1 |
| NLRP13 | 8.28E-01 | 1 | C1orf77 | 1 | 1 |
| PRICKLE1 | 8.28E-01 | 1 | C1orf83 | 1 | 1 |

| NUP210 | 8.28E-01 | 1 | C1orf84 | 1 | 1 |
| --- | --- | --- | --- | --- | --- |
| FMO4 | 8.28E-01 | 1 | C1orf85 | 1 | 1 |
| ZNF615 | 8.28E-01 | 1 | C1orf86 | 1 | 1 |
| TBC1D1 | 8.29E-01 | 1 | C1orf88 | 1 | 1 |
| TRPM7 | 8.29E-01 | 1 | C1orf89 | 1 | 1 |
| KCNK10 | 8.29E-01 | 1 | C1orf9 | 1 | 1 |
| TRIM6-TR | 8.29E-01 | 1 | C1orf91 | 1 | 1 |
| KL | 8.29E-01 | 1 | C1orf92 | 1 | 1 |
| PDILT | 8.29E-01 | 1 | C1orf93 | 1 | 1 |
| EIF3A | 8.29E-01 | 1 | C1orf95 | 1 | 1 |
| TMEM131 | 8.29E-01 | 1 | C1orf96 | 1 | 1 |
| TARS | 8.29E-01 | 1 | C2 | 1 | 1 |
| ECT2 | 8.30E-01 | 1 | C20orf103 | 1 | 1 |
| PDGFRB | 8.30E-01 | 1 | C20orf106 | 1 | 1 |
| ADAMTS | 8.30E-01 | 1 | C20orf107 | 1 | 1 |
| ZNF226 | 8.30E-01 | 1 | C20orf108 | 1 | 1 |
| USP6 | 8.31E-01 | 1 | C20orf11 | 1 | 1 |
| FOLH1 | 8.31E-01 | 1 | C20orf111 | 1 | 1 |
| AMOTL1 | 8.31E-01 | 1 | C20orf112 | 1 | 1 |
| SCN1A | 8.31E-01 | 1 | C20orf114 | 1 | 1 |
| TMEM168 | 8.31E-01 | 1 | C20orf117 | 1 | 1 |
| AASS | 8.31E-01 | 1 | C20orf118 | 1 | 1 |
| AEBP1 | 8.32E-01 | 1 | C20orf12 | 1 | 1 |
| ZNF225 | 8.32E-01 | 1 | C20orf123 | 1 | 1 |
| ZNF571 | 8.32E-01 | 1 | C20orf132 | 1 | 1 |
| DHRS9 | 8.32E-01 | 1 | C20orf134 | 1 | 1 |
| C5orf42 | 8.33E-01 | 1 | C20orf135 | 1 | 1 |
| SYNE2 | 8.33E-01 | 1 | C20orf141 | 1 | 1 |
| IRGQ | 8.33E-01 | 1 | C20orf144 | 1 | 1 |
| ZFP62 | 8.33E-01 | 1 | C20orf151 | 1 | 1 |
| STRC | 8.33E-01 | 1 | C20orf152 | 1 | 1 |
| EIF4G1 | 8.33E-01 | 1 | C20orf160 | 1 | 1 |
| PPP1R12A | 8.33E-01 | 1 | C20orf165 | 1 | 1 |
| NAP1L2 | 8.33E-01 | 1 | C20orf166 | 1 | 1 |
| ST6GAL2 | 8.33E-01 | 1 | C20orf173 | 1 | 1 |
| LARGE | 8.34E-01 | 1 | C20orf177 | 1 | 1 |
| NOMO1 | 8.34E-01 | 1 | C20orf185 | 1 | 1 |
| PCSK5 | 8.34E-01 | 1 | C20orf186 | 1 | 1 |
| UPF2 | 8.34E-01 | 1 | C20orf194 | 1 | 1 |
| COL4A2 | 8.35E-01 | 1 | C20orf195 | 1 | 1 |
| IFT172 | 8.35E-01 | 1 | C20orf197 | 1 | 1 |
| KIAA0226 | 8.35E-01 | 1 | C20orf20 | 1 | 1 |
| LRPPRC | 8.35E-01 | 1 | C20orf201 | 1 | 1 |
| ATP1A4 | 8.35E-01 | 1 | C20orf202 | 1 | 1 |
| AFF3 | 8.35E-01 | 1 | C20orf203 | 1 | 1 |
| PCDHB8 | 8.35E-01 | 1 | C20orf26 | 1 | 1 |
| CCDC144 | 8.35E-01 | 1 | C20orf29 | 1 | 1 |
| LATS2 | 8.36E-01 | 1 | C20orf3 | 1 | 1 |
| SLITRK5 | 8.36E-01 | 1 | C20orf30 | 1 | 1 |
| TARBP1 | 8.36E-01 | 1 | C20orf4 | 1 | 1 |
| HERC3 | 8.37E-01 | 1 | C20orf43 | 1 | 1 |
| NACAD | 8.37E-01 | 1 | C20orf46 | 1 | 1 |
| CDH26 | 8.37E-01 | 1 | C20orf54 | 1 | 1 |

| CKAP5 | 8.37E-01 | 1 | C20orf7 | 1 | 1 |
| --- | --- | --- | --- | --- | --- |
| ZNF142 | 8.37E-01 | 1 | C20orf70 | 1 | 1 |
| DUOX2 | 8.37E-01 | 1 | C20orf71 | 1 | 1 |
| ANKRD35 | 8.37E-01 | 1 | C20orf72 | 1 | 1 |
| EPX | 8.37E-01 | 1 | C20orf79 | 1 | 1 |
| IFT122 | 8.37E-01 | 1 | C20orf85 | 1 | 1 |
| CYLD | 8.37E-01 | 1 | C20orf94 | 1 | 1 |
| ZNF217 | 8.38E-01 | 1 | C20orf96 | 1 | 1 |
| IKBKAP | 8.38E-01 | 1 | C21orf2 | 1 | 1 |
| PKD1L3 | 8.38E-01 | 1 | C21orf29 | 1 | 1 |
| CARD14 | 8.38E-01 | 1 | C21orf33 | 1 | 1 |
| PHF14 | 8.38E-01 | 1 | C21orf45 | 1 | 1 |
| NPNT | 8.38E-01 | 1 | C21orf56 | 1 | 1 |
| RRBP1 | 8.38E-01 | 1 | C21orf57 | 1 | 1 |
| RIMS1 | 8.39E-01 | 1 | C21orf58 | 1 | 1 |
| IRS2 | 8.39E-01 | 1 | C21orf63 | 1 | 1 |
| ZCCHC2 | 8.40E-01 | 1 | C21orf7 | 1 | 1 |
| SBNO1 | 8.40E-01 | 1 | C21orf70 | 1 | 1 |
| ARMC2 | 8.40E-01 | 1 | C21orf91 | 1 | 1 |
| RPH3A | 8.40E-01 | 1 | C22orf13 | 1 | 1 |
| PTPRU | 8.40E-01 | 1 | C22orf23 | 1 | 1 |
| DDX10 | 8.40E-01 | 1 | C22orf24 | 1 | 1 |
| IFT57 | 8.40E-01 | 1 | C22orf25 | 1 | 1 |
| LEMD3 | 8.41E-01 | 1 | C22orf26 | 1 | 1 |
| ZNF287 | 8.41E-01 | 1 | C22orf28 | 1 | 1 |
| RIMBP3 | 8.41E-01 | 1 | C22orf30 | 1 | 1 |
| ACSL5 | 8.41E-01 | 1 | C22orf31 | 1 | 1 |
| MGEA5 | 8.42E-01 | 1 | C22orf32 | 1 | 1 |
| AK7 | 8.42E-01 | 1 | C22orf33 | 1 | 1 |
| CNTNAP2 | 8.42E-01 | 1 | C22orf36 | 1 | 1 |
| ST6GALN | 8.42E-01 | 1 | C22orf40 | 1 | 1 |
| FRAS1 | 8.42E-01 | 1 | C22orf41 | 1 | 1 |
| RSC1A1 | 8.42E-01 | 1 | C22orf42 | 1 | 1 |
| FMNL1 | 8.42E-01 | 1 | C22orf43 | 1 | 1 |
| SMG6 | 8.42E-01 | 1 | C22orf9 | 1 | 1 |
| TTK | 8.43E-01 | 1 | C2CD4A | 1 | 1 |
| USH1C | 8.43E-01 | 1 | C2CD4B | 1 | 1 |
| MNDA | 8.43E-01 | 1 | C2CD4C | 1 | 1 |
| CNBD1 | 8.43E-01 | 1 | C2CD4D | 1 | 1 |
| ABCA2 | 8.43E-01 | 1 | C2orf15 | 1 | 1 |
| NRSN2 | 8.44E-01 | 1 | C2orf18 | 1 | 1 |
| CDH2 | 8.44E-01 | 1 | C2orf24 | 1 | 1 |
| C15orf39 | 8.44E-01 | 1 | C2orf27A | 1 | 1 |
| NCAM2 | 8.44E-01 | 1 | C2orf27B | 1 | 1 |
| MORC1 | 8.44E-01 | 1 | C2orf28 | 1 | 1 |
| WDR19 | 8.45E-01 | 1 | C2orf29 | 1 | 1 |
| SRBD1 | 8.45E-01 | 1 | C2orf3 | 1 | 1 |
| ZNF823 | 8.45E-01 | 1 | C2orf34 | 1 | 1 |
| MYPN | 8.45E-01 | 1 | C2orf39 | 1 | 1 |
| TNRC6C | 8.45E-01 | 1 | C2orf40 | 1 | 1 |
| ZNF273 | 8.46E-01 | 1 | C2orf42 | 1 | 1 |
| MAML3 | 8.46E-01 | 1 | C2orf43 | 1 | 1 |
| PIK3C2A | 8.46E-01 | 1 | C2orf44 | 1 | 1 |

| DSP | 8.46E-01 | 1 | C2orf47 | 1 | 1 |
| --- | --- | --- | --- | --- | --- |
| THAP9 | 8.46E-01 | 1 | C2orf48 | 1 | 1 |
| HIPK1 | 8.46E-01 | 1 | C2orf49 | 1 | 1 |
| PCDHB1 | 8.46E-01 | 1 | C2orf50 | 1 | 1 |
| SEMA4G | 8.46E-01 | 1 | C2orf51 | 1 | 1 |
| HPS4 | 8.46E-01 | 1 | C2orf53 | 1 | 1 |
| OLFML2B | 8.47E-01 | 1 | C2orf54 | 1 | 1 |
| ZNF598 | 8.47E-01 | 1 | C2orf55 | 1 | 1 |
| SOX5 | 8.47E-01 | 1 | C2orf56 | 1 | 1 |
| NLRP8 | 8.47E-01 | 1 | C2orf60 | 1 | 1 |
| CEP63 | 8.47E-01 | 1 | C2orf61 | 1 | 1 |
| PITPNM3 | 8.47E-01 | 1 | C2orf62 | 1 | 1 |
| GRM5 | 8.47E-01 | 1 | C2orf63 | 1 | 1 |
| CCNT2 | 8.48E-01 | 1 | C2orf64 | 1 | 1 |
| ZNF649 | 8.48E-01 | 1 | C2orf65 | 1 | 1 |
| EIF3H | 8.48E-01 | 1 | C2orf66 | 1 | 1 |
| SLC5A2 | 8.48E-01 | 1 | C2orf67 | 1 | 1 |
| RAD50 | 8.48E-01 | 1 | C2orf68 | 1 | 1 |
| RNF10 | 8.48E-01 | 1 | C2orf69 | 1 | 1 |
| SORCS1 | 8.49E-01 | 1 | C2orf7 | 1 | 1 |
| UHRF1BP | 8.49E-01 | 1 | C2orf70 | 1 | 1 |
| AHI1 | 8.49E-01 | 1 | C2orf72 | 1 | 1 |
| SPTLC3 | 8.49E-01 | 1 | C2orf73 | 1 | 1 |
| DHX30 | 8.49E-01 | 1 | C2orf74 | 1 | 1 |
| NCOA2 | 8.49E-01 | 1 | C2orf76 | 1 | 1 |
| WDR64 | 8.49E-01 | 1 | C2orf77 | 1 | 1 |
| ACE | 8.49E-01 | 1 | C2orf78 | 1 | 1 |
| MMP27 | 8.49E-01 | 1 | C2orf79 | 1 | 1 |
| PRSS12 | 8.49E-01 | 1 | C2orf81 | 1 | 1 |
| LAMA3 | 8.49E-01 | 1 | C2orf82 | 1 | 1 |
| CTAGE4 | 8.50E-01 | 1 | C2orf83 | 1 | 1 |
| PLEKHM1 | 8.50E-01 | 1 | C2orf84 | 1 | 1 |
| KDM3B | 8.50E-01 | 1 | C2orf85 | 1 | 1 |
| MFN1 | 8.51E-01 | 1 | C2orf86 | 1 | 1 |
| DZIP1L | 8.51E-01 | 1 | C2orf88 | 1 | 1 |
| PSMD1 | 8.51E-01 | 1 | C2orf89 | 1 | 1 |
| CEL | 8.51E-01 | 1 | C3AR1 | 1 | 1 |
| BRWD3 | 8.51E-01 | 1 | C3orf1 | 1 | 1 |
| LILRB1 | 8.51E-01 | 1 | C3orf10 | 1 | 1 |
| CWC22 | 8.51E-01 | 1 | C3orf14 | 1 | 1 |
| ACO1 | 8.52E-01 | 1 | C3orf15 | 1 | 1 |
| SPTAN1 | 8.52E-01 | 1 | C3orf16 | 1 | 1 |
| TTC37 | 8.52E-01 | 1 | C3orf17 | 1 | 1 |
| YES1 | 8.52E-01 | 1 | C3orf18 | 1 | 1 |
| ZZZ3 | 8.52E-01 | 1 | C3orf19 | 1 | 1 |
| BMP2K | 8.53E-01 | 1 | C3orf21 | 1 | 1 |
| LDLR | 8.53E-01 | 1 | C3orf22 | 1 | 1 |
| DLL1 | 8.53E-01 | 1 | C3orf23 | 1 | 1 |
| ADD2 | 8.53E-01 | 1 | C3orf24 | 1 | 1 |
| SEMA5A | 8.53E-01 | 1 | C3orf26 | 1 | 1 |
| TOP1 | 8.53E-01 | 1 | C3orf27 | 1 | 1 |
| FGFR2 | 8.53E-01 | 1 | C3orf31 | 1 | 1 |
| TOP2B | 8.53E-01 | 1 | C3orf32 | 1 | 1 |

| MYH10 | 8.53E-01 | 1 | C3orf33 | 1 | 1 |
| --- | --- | --- | --- | --- | --- |
| ZNF626 | 8.53E-01 | 1 | C3orf34 | 1 | 1 |
| SEL1L2 | 8.53E-01 | 1 | C3orf35 | 1 | 1 |
| PARK2 | 8.53E-01 | 1 | C3orf36 | 1 | 1 |
| SLC24A3 | 8.53E-01 | 1 | C3orf37 | 1 | 1 |
| TMEM2 | 8.53E-01 | 1 | C3orf38 | 1 | 1 |
| NOTCH3 | 8.54E-01 | 1 | C3orf39 | 1 | 1 |
| WDR11 | 8.54E-01 | 1 | C3orf43 | 1 | 1 |
| THRSP | 8.54E-01 | 1 | C3orf45 | 1 | 1 |
| TET1 | 8.54E-01 | 1 | C3orf52 | 1 | 1 |
| KDM6A | 8.55E-01 | 1 | C3orf54 | 1 | 1 |
| ABCC2 | 8.55E-01 | 1 | C3orf55 | 1 | 1 |
| ULK4 | 8.55E-01 | 1 | C3orf57 | 1 | 1 |
| ARID4A | 8.55E-01 | 1 | C3orf59 | 1 | 1 |
| USP26 | 8.56E-01 | 1 | C3orf62 | 1 | 1 |
[truncated: 385,194 more chars]
