# Supplementary material for: Hypoxia Molecular Characterization in Hepatocellular Carcinoma Identifies One Risk Signature and Two Nomograms for Clinical Management
Source: J Oncol. 2021 Jan 20;2021:6664386. doi: 10.1155/2021/6664386 (PMC7846409; doi:10.1155/2021/6664386)
Supplement: Supplementary Materials — Figure S1: the identification of molecular subtypes in metacohort. (a) Using the unsupervised clustering algorithm to classify patients into different molecular subtypes in metacohort. The consensus score matrix of 831 HCC samples (K = 2∼9). A higher consensus score between two samples indicates they were more likely to be grouped into the same cluster in different iterations. The figure demonstrated k = 2 was the best choice. (b) The proportion of ambiguous clustering (PAC) score, a low value of PAC implied a flat middle segment in cumulative distribution functions (CDFs), allowing conjecture of the optimal k (k = 2) by the lowest PAC. (c) Recommended number of clusters using 26 criteria of Nbclust package in the metacohort. The highest of the column represented the optimal k (k = 2). Figure S2: the differences of HAG expression, known signatures, and fibroblast infiltration between C1 and C2 in metacohort. (a) The expression heatmap of 24 HAGs between C1 and C2. High expression, red; low expression, blue. (b) The expression box plot of 24 HAGs between C1 and C2. (c) Comparison of the signatures score between C1 and C2. (d) The abundance of fibroblasts was compared between C1 and C2. ns, P > 0.05; ∗P < 0.05; ∗∗P < 0.01; ∗∗∗P < 0.001. Figure S3: the difference of immune checkpoints (ICPs) and immune cells between C1 and C2. (a) The expression boxplot of ICPs between C1 and C2. ns, P > 0.05; ∗P < 0.05; ∗∗P < 0.01; ∗∗∗P < 0.001. (b) The heatmap of 23 immune cells between C1 and C2. High expression, red; low expression, blue. (c) Correlations between immune cells and HAGs using Spearman analysis. Negative correlation was marked with blue, and positive correlation was marked with red. No asterisks represented no statistical significance; ∗P < 0.05; ∗∗P < 0.01. Figure S4: the mutation signatures and significantly mutated genes (SMGs) in TCGA-LIHC cohort. (a) Comparison of tumor mutation burden (TMB) between C1 and C2. (b) The expression difference of 12 SMGs between muta [file 6664386.f1.zip › 6664386.f1/Table S7.docx]

| **Table S7: A total of 299 subtype-related differentially expressed genes (DEGs).**  **** DEGs are marked with bold.*** | | | | | | |
| --- | --- | --- | --- | --- | --- | --- |
| **GeneID** | **logFC** | **AveExpr** | **t** | **P.Value** | **adj.P.Val** | **B** |
| **CA9** | -3.9904 | 3.2874 | -33.2792 | 0.0000 | 0.0000 | 246.2320 |
| **SLC27A5** | 1.0079 | 6.8293 | 13.0895 | 0.0000 | 0.0000 | 62.9900 |
| PEBP1 | 0.2569 | 7.9481 | 12.6800 | 0.0000 | 0.0000 | 59.3733 |
| POR | 0.3201 | 7.3921 | 12.5739 | 0.0000 | 0.0000 | 58.4437 |
| **COL9A2** | -1.4945 | 3.6631 | -12.5418 | 0.0000 | 0.0000 | 58.1626 |
| CYB5A | 0.4391 | 7.0914 | 12.5255 | 0.0000 | 0.0000 | 58.0210 |
| **C3orf52** | -1.6091 | 1.5237 | -12.5159 | 0.0000 | 0.0000 | 57.9369 |
| CAT | 0.3244 | 7.3942 | 12.4002 | 0.0000 | 0.0000 | 56.9282 |
| ALAS1 | 0.3536 | 7.3592 | 12.3077 | 0.0000 | 0.0000 | 56.1246 |
| **PPFIA4** | -1.2676 | 1.1952 | -12.2933 | 0.0000 | 0.0000 | 55.9999 |
| CHPT1 | 0.3255 | 6.8079 | 12.0403 | 0.0000 | 0.0000 | 53.8164 |
| HSD17B4 | 0.3470 | 7.0494 | 12.0285 | 0.0000 | 0.0000 | 53.7145 |
| CRYL1 | 0.3690 | 7.2222 | 11.9976 | 0.0000 | 0.0000 | 53.4500 |
| ALAD | 0.3575 | 7.0342 | 11.9316 | 0.0000 | 0.0000 | 52.8844 |
| ALDH2 | 0.3655 | 7.2817 | 11.9098 | 0.0000 | 0.0000 | 52.6977 |
| EPHX1 | 0.3451 | 7.9721 | 11.8951 | 0.0000 | 0.0000 | 52.5723 |
| HAGH | 0.4102 | 6.9005 | 11.8470 | 0.0000 | 0.0000 | 52.1610 |
| **NXPH4** | -1.6913 | 3.9769 | -11.8285 | 0.0000 | 0.0000 | 52.0034 |
| **FOXJ1** | -2.0998 | 2.4152 | -11.8126 | 0.0000 | 0.0000 | 51.8682 |
| **ENO2** | -1.1880 | 4.1022 | -11.7740 | 0.0000 | 0.0000 | 51.5389 |
| METTL7A | 0.3213 | 7.4760 | 11.7088 | 0.0000 | 0.0000 | 50.9854 |
| CES2 | 0.5261 | 7.2976 | 11.7047 | 0.0000 | 0.0000 | 50.9505 |
| CYP27A1 | 0.4498 | 7.5233 | 11.7046 | 0.0000 | 0.0000 | 50.9500 |
| ADI1 | 0.3457 | 7.2798 | 11.5672 | 0.0000 | 0.0000 | 49.7867 |
| GRHPR | 0.3490 | 7.0819 | 11.5487 | 0.0000 | 0.0000 | 49.6310 |
| ALDH5A1 | 0.4389 | 6.7893 | 11.5473 | 0.0000 | 0.0000 | 49.6189 |
| PCK2 | 0.4629 | 7.3007 | 11.4488 | 0.0000 | 0.0000 | 48.7904 |
| GCDH | 0.4328 | 6.6099 | 11.4480 | 0.0000 | 0.0000 | 48.7837 |
| SAR1B | 0.3687 | 6.5791 | 11.4466 | 0.0000 | 0.0000 | 48.7716 |
| COL18A1 | 0.2643 | 7.4174 | 11.4310 | 0.0000 | 0.0000 | 48.6408 |
| INSIG1 | 0.4738 | 7.3779 | 11.3887 | 0.0000 | 0.0000 | 48.2862 |
| UBB | 0.1814 | 7.8287 | 11.3717 | 0.0000 | 0.0000 | 48.1438 |
| PCCB | 0.3485 | 6.6586 | 11.3541 | 0.0000 | 0.0000 | 47.9969 |
| SPATS2 | -0.4555 | 5.4700 | -11.3164 | 0.0000 | 0.0000 | 47.6818 |
| HSD17B8 | 0.3768 | 6.9843 | 11.2599 | 0.0000 | 0.0000 | 47.2100 |
| NUDT7 | 0.5417 | 6.1107 | 11.2222 | 0.0000 | 0.0000 | 46.8964 |
| **EGLN3** | -1.5815 | 3.8903 | -11.2212 | 0.0000 | 0.0000 | 46.8884 |
| DCXR | 0.4114 | 7.6252 | 11.2063 | 0.0000 | 0.0000 | 46.7640 |
| ECI2 | 0.2904 | 7.1147 | 11.0466 | 0.0000 | 0.0000 | 45.4418 |
| SERPING1 | 0.2588 | 7.8975 | 11.0386 | 0.0000 | 0.0000 | 45.3752 |
| PGRMC1 | 0.2557 | 7.6055 | 11.0127 | 0.0000 | 0.0000 | 45.1623 |
| **TYRO3** | -1.4248 | 3.7813 | -10.9866 | 0.0000 | 0.0000 | 44.9474 |
| IGFBP4 | 0.2293 | 7.7957 | 10.9666 | 0.0000 | 0.0000 | 44.7823 |
| PEX19 | 0.2294 | 6.9702 | 10.9420 | 0.0000 | 0.0000 | 44.5806 |
| EMCN | 0.8042 | 5.0703 | 10.9392 | 0.0000 | 0.0000 | 44.5575 |
| CLEC3B | 0.8064 | 5.9808 | 10.9382 | 0.0000 | 0.0000 | 44.5492 |
| **GIPR** | -1.3556 | 2.0628 | -10.9044 | 0.0000 | 0.0000 | 44.2717 |
| SOD1 | 0.2427 | 7.7259 | 10.8660 | 0.0000 | 0.0000 | 43.9571 |
| **SLC10A1** | 1.6611 | 6.4175 | 10.8544 | 0.0000 | 0.0000 | 43.8623 |

| SORD | 0.5321 | 6.8480 | 10.8015 | 0.0000 | 0.0000 | 43.4299 |
| --- | --- | --- | --- | --- | --- | --- |
| DHRS4 | 0.3566 | 6.5289 | 10.7900 | 0.0000 | 0.0000 | 43.3364 |
| NDFIP1 | 0.1910 | 7.0151 | 10.7862 | 0.0000 | 0.0000 | 43.3058 |
| **WNT7B** | -1.5590 | 1.1849 | -10.7687 | 0.0000 | 0.0000 | 43.1632 |
| **MPP2** | -1.1831 | 1.6874 | -10.7089 | 0.0000 | 0.0000 | 42.6760 |
| QDPR | 0.3358 | 7.0251 | 10.7008 | 0.0000 | 0.0000 | 42.6105 |
| SEQ | 0.2511 | 6.8266 | 10.6533 | 0.0000 | 0.0000 | 42.2251 |
| **PFKFB1** | 1.2951 | 5.7447 | 10.6489 | 0.0000 | 0.0000 | 42.1898 |
| GSTK1 | 0.2268 | 7.2964 | 10.5816 | 0.0000 | 0.0000 | 41.6456 |
| FH | 0.2339 | 7.4427 | 10.5739 | 0.0000 | 0.0000 | 41.5839 |
| SLCO2B1 | 0.5371 | 6.6512 | 10.5694 | 0.0000 | 0.0000 | 41.5471 |
| FDX1 | 0.2941 | 6.6546 | 10.5651 | 0.0000 | 0.0000 | 41.5128 |
| **EPO** | -2.1332 | 3.1441 | -10.5527 | 0.0000 | 0.0000 | 41.4124 |
| **FABP4** | 1.3888 | 5.3531 | 10.5411 | 0.0000 | 0.0000 | 41.3191 |
| ECHS1 | 0.2326 | 7.7493 | 10.5397 | 0.0000 | 0.0000 | 41.3075 |
| **CEP55** | -1.0655 | 4.3724 | -10.5138 | 0.0000 | 0.0000 | 41.0996 |
| DECR1 | 0.2723 | 7.2375 | 10.5087 | 0.0000 | 0.0000 | 41.0583 |
| RNF24 | -0.6388 | 4.5907 | -10.4954 | 0.0000 | 0.0000 | 40.9516 |
| ABAT | 0.6443 | 6.7568 | 10.4862 | 0.0000 | 0.0000 | 40.8776 |
| **ADRB2** | 1.1758 | 4.7988 | 10.4837 | 0.0000 | 0.0000 | 40.8576 |
| DIAPH1 | 0.2202 | 6.9713 | 10.4678 | 0.0000 | 0.0000 | 40.7300 |
| HAAO | 0.4521 | 7.0655 | 10.4622 | 0.0000 | 0.0000 | 40.6847 |
| RHOB | 0.2629 | 7.5989 | 10.4497 | 0.0000 | 0.0000 | 40.5844 |
| DCAF11 | 0.2864 | 6.8434 | 10.4292 | 0.0000 | 0.0000 | 40.4202 |
| EPHX2 | 0.5079 | 6.7804 | 10.4266 | 0.0000 | 0.0000 | 40.3994 |
| **GLYAT** | 1.9721 | 5.7150 | 10.4199 | 0.0000 | 0.0000 | 40.3461 |
| FBXO7 | 0.2374 | 6.6344 | 10.3765 | 0.0000 | 0.0000 | 39.9988 |
| CENPO | -0.6957 | 4.6684 | -10.3748 | 0.0000 | 0.0000 | 39.9855 |
| **RASAL1** | -1.6515 | 2.0815 | -10.3607 | 0.0000 | 0.0000 | 39.8731 |
| **SYT13** | -2.0058 | 2.0974 | -10.3504 | 0.0000 | 0.0000 | 39.7909 |
| MFSD10 | -0.3893 | 6.2053 | -10.3351 | 0.0000 | 0.0000 | 39.6690 |
| CHAF1B | -0.9777 | 4.4868 | -10.3310 | 0.0000 | 0.0000 | 39.6363 |
| TMBIM6 | 0.1663 | 7.6779 | 10.3270 | 0.0000 | 0.0000 | 39.6044 |
| BDH1 | 0.7924 | 6.5600 | 10.2839 | 0.0000 | 0.0000 | 39.2615 |
| C1S | 0.2892 | 7.6872 | 10.2736 | 0.0000 | 0.0000 | 39.1797 |
| RGN | 0.6123 | 7.0509 | 10.2561 | 0.0000 | 0.0000 | 39.0408 |
| SLC1A5 | -0.7426 | 5.8294 | -10.2347 | 0.0000 | 0.0000 | 38.8708 |
| **AQP9** | 1.0601 | 7.0017 | 10.2312 | 0.0000 | 0.0000 | 38.8430 |
| **ACSM5** | 1.1391 | 6.2809 | 10.1976 | 0.0000 | 0.0000 | 38.5772 |
| DHRS1 | 0.4339 | 6.4857 | 10.1928 | 0.0000 | 0.0000 | 38.5389 |
| HSDL2 | 0.2634 | 7.0668 | 10.1718 | 0.0000 | 0.0000 | 38.3735 |
| MTSS1 | 0.3049 | 6.7422 | 10.1629 | 0.0000 | 0.0000 | 38.3033 |
| PHF21A | -0.4012 | 5.0647 | -10.1585 | 0.0000 | 0.0000 | 38.2686 |
| ALDH9A1 | 0.2280 | 7.1175 | 10.1495 | 0.0000 | 0.0000 | 38.1973 |
| **EFNA5** | -1.6937 | 2.1646 | -10.1463 | 0.0000 | 0.0000 | 38.1718 |
| KLF9 | 0.4790 | 6.6594 | 10.1345 | 0.0000 | 0.0000 | 38.0790 |
| PQLC1 | 0.2820 | 6.9472 | 10.1293 | 0.0000 | 0.0000 | 38.0378 |
| APOC1 | 0.3046 | 8.2120 | 10.1192 | 0.0000 | 0.0000 | 37.9583 |
| **LRP8** | -1.0505 | 2.0785 | -10.1132 | 0.0000 | 0.0000 | 37.9110 |
| ALDH7A1 | 0.3696 | 6.8248 | 10.1055 | 0.0000 | 0.0000 | 37.8505 |
| **FCHO1** | -1.2325 | 2.9938 | -10.1034 | 0.0000 | 0.0000 | 37.8343 |
| RCL1 | 0.4859 | 6.1389 | 10.0633 | 0.0000 | 0.0000 | 37.5186 |
| LIMK1 | -0.5538 | 5.3471 | -10.0497 | 0.0000 | 0.0000 | 37.4125 |

| **ACSM2A** | 1.1962 | 6.4325 | 10.0478 | 0.0000 | 0.0000 | 37.3976 |
| --- | --- | --- | --- | --- | --- | --- |
| **PLEKHB1** | -1.7100 | 2.9328 | -10.0476 | 0.0000 | 0.0000 | 37.3961 |
| MSRA | 0.4428 | 6.1091 | 10.0468 | 0.0000 | 0.0000 | 37.3894 |
| **EPHB3** | -1.3800 | 2.9750 | -10.0440 | 0.0000 | 0.0000 | 37.3675 |
| PCTP | 0.3432 | 6.4438 | 10.0384 | 0.0000 | 0.0000 | 37.3233 |
| LMNB2 | -0.3742 | 5.9322 | -10.0256 | 0.0000 | 0.0000 | 37.2230 |
| IVD | 0.2836 | 6.8747 | 10.0124 | 0.0000 | 0.0000 | 37.1200 |
| PDK4 | 0.6464 | 6.6545 | 10.0017 | 0.0000 | 0.0000 | 37.0359 |
| GOT2 | 0.2559 | 7.2766 | 9.9779 | 0.0000 | 0.0000 | 36.8499 |
| **PROM1** | -1.7602 | 1.6791 | -9.9719 | 0.0000 | 0.0000 | 36.8032 |
| DHTKD1 | 0.3757 | 6.8800 | 9.9627 | 0.0000 | 0.0000 | 36.7314 |
| PFKFB4 | -0.8851 | 3.8476 | -9.9545 | 0.0000 | 0.0000 | 36.6675 |
| ACOX1 | 0.2989 | 6.9091 | 9.9394 | 0.0000 | 0.0000 | 36.5500 |
| SDC2 | 0.2344 | 7.3993 | 9.9284 | 0.0000 | 0.0000 | 36.4639 |
| EHHADH | 0.5774 | 6.9533 | 9.9167 | 0.0000 | 0.0000 | 36.3731 |
| ALDH6A1 | 0.5034 | 6.8283 | 9.9082 | 0.0000 | 0.0000 | 36.3071 |
| **FRAS1** | -1.7355 | 2.1206 | -9.9068 | 0.0000 | 0.0000 | 36.2955 |
| HADH | 0.2774 | 7.0484 | 9.8885 | 0.0000 | 0.0000 | 36.1537 |
| KIF2C | -0.9288 | 5.1396 | -9.8827 | 0.0000 | 0.0000 | 36.1085 |
| MYO1B | 0.3146 | 6.8398 | 9.8750 | 0.0000 | 0.0000 | 36.0484 |
| RBL1 | -0.6686 | 4.4647 | -9.8560 | 0.0000 | 0.0000 | 35.9012 |
| DHRS12 | 0.4329 | 5.8354 | 9.8417 | 0.0000 | 0.0000 | 35.7906 |
| ORC6 | -0.9257 | 4.0023 | -9.8356 | 0.0000 | 0.0000 | 35.7432 |
| **FA2H** | -1.6099 | 1.7419 | -9.8281 | 0.0000 | 0.0000 | 35.6847 |
| ALDH1A1 | 0.3496 | 7.7408 | 9.7983 | 0.0000 | 0.0000 | 35.4541 |
| PRR11 | -0.8944 | 4.6815 | -9.7904 | 0.0000 | 0.0000 | 35.3936 |
| LPCAT1 | -0.5325 | 5.9546 | -9.7875 | 0.0000 | 0.0000 | 35.3713 |
| CD14 | 0.3341 | 7.5169 | 9.7812 | 0.0000 | 0.0000 | 35.3224 |
| **KIF23** | -1.0471 | 4.2263 | -9.7751 | 0.0000 | 0.0000 | 35.2751 |
| CCL14 | 0.9021 | 4.1656 | 9.7726 | 0.0000 | 0.0000 | 35.2556 |
| HOMER3 | -0.6168 | 5.5125 | -9.7656 | 0.0000 | 0.0000 | 35.2021 |
| ANXA7 | 0.1432 | 7.1845 | 9.7574 | 0.0000 | 0.0000 | 35.1385 |
| **TFF1** | -2.1193 | 2.0317 | -9.7559 | 0.0000 | 0.0000 | 35.1272 |
| MCCC2 | 0.2375 | 6.8253 | 9.7535 | 0.0000 | 0.0000 | 35.1087 |
| SH3D21 | -0.7930 | 3.4502 | -9.7481 | 0.0000 | 0.0000 | 35.0668 |
| WDR62 | -0.8984 | 3.9941 | -9.7451 | 0.0000 | 0.0000 | 35.0439 |
| **AGR2** | -2.2104 | 2.4796 | -9.7442 | 0.0000 | 0.0000 | 35.0366 |
| **NR1I3** | 1.0823 | 6.2752 | 9.7424 | 0.0000 | 0.0000 | 35.0234 |
| **INPP5J** | -1.2014 | 2.4981 | -9.7273 | 0.0000 | 0.0000 | 34.9071 |
| TMEM132A | -0.9392 | 4.4902 | -9.7228 | 0.0000 | 0.0000 | 34.8718 |
| **SPP2** | 1.5205 | 6.5043 | 9.7198 | 0.0000 | 0.0000 | 34.8489 |
| ETNK2 | 0.7450 | 6.7145 | 9.7088 | 0.0000 | 0.0000 | 34.7644 |
| ANXA6 | 0.3006 | 7.0659 | 9.7033 | 0.0000 | 0.0000 | 34.7220 |
| **RAD54L** | -1.1096 | 3.8454 | -9.6985 | 0.0000 | 0.0000 | 34.6854 |
| FAM57A | -0.6939 | 4.9361 | -9.6810 | 0.0000 | 0.0000 | 34.5508 |
| **GAL3ST1** | -1.5583 | 4.7962 | -9.6772 | 0.0000 | 0.0000 | 34.5220 |
| SRD5A1 | 0.5334 | 5.9593 | 9.6760 | 0.0000 | 0.0000 | 34.5127 |
| ANG | 0.3966 | 7.5990 | 9.6744 | 0.0000 | 0.0000 | 34.5002 |
| LAMP1 | 0.1815 | 7.3359 | 9.6584 | 0.0000 | 0.0000 | 34.3775 |
| WDHD1 | -0.7829 | 4.0358 | -9.6577 | 0.0000 | 0.0000 | 34.3722 |
| **CCL16** | 1.3431 | 6.6251 | 9.6525 | 0.0000 | 0.0000 | 34.3322 |
| CRAT | 0.2234 | 7.1008 | 9.6524 | 0.0000 | 0.0000 | 34.3316 |
| CDO1 | 0.8930 | 7.0881 | 9.6512 | 0.0000 | 0.0000 | 34.3223 |

| DNASE1L3 | 0.9906 | 5.4572 | 9.6498 | 0.0000 | 0.0000 | 34.3119 |
| --- | --- | --- | --- | --- | --- | --- |
| SELENBP1 | 0.4341 | 7.2077 | 9.6491 | 0.0000 | 0.0000 | 34.3060 |
| DRP2 | -0.6078 | 0.5114 | -9.6342 | 0.0000 | 0.0000 | 34.1924 |
| G6PD | -0.5330 | 6.1926 | -9.6279 | 0.0000 | 0.0000 | 34.1437 |
| **SLC1A2** | 1.7004 | 4.8440 | 9.6148 | 0.0000 | 0.0000 | 34.0436 |
| FMO4 | 0.6272 | 6.2146 | 9.6123 | 0.0000 | 0.0000 | 34.0244 |
| RAMP3 | 0.6774 | 6.1690 | 9.6114 | 0.0000 | 0.0000 | 34.0175 |
| NCDN | -0.3325 | 5.6446 | -9.6076 | 0.0000 | 0.0000 | 33.9885 |
| **CFHR4** | 1.4444 | 5.7771 | 9.6074 | 0.0000 | 0.0000 | 33.9874 |
| ABI2 | -0.4810 | 4.4480 | -9.5979 | 0.0000 | 0.0000 | 33.9149 |
| TACC3 | -0.5313 | 5.7504 | -9.5909 | 0.0000 | 0.0000 | 33.8609 |
| **SPHK1** | -1.2975 | 4.7756 | -9.5901 | 0.0000 | 0.0000 | 33.8550 |
| RBP4 | 0.3576 | 8.1957 | 9.5857 | 0.0000 | 0.0000 | 33.8219 |
| C6orf106 | 0.1630 | 7.2130 | 9.5843 | 0.0000 | 0.0000 | 33.8107 |
| CRY2 | 0.3132 | 6.5266 | 9.5837 | 0.0000 | 0.0000 | 33.8066 |
| IL6R | 0.4144 | 6.6904 | 9.5736 | 0.0000 | 0.0000 | 33.7292 |
| SLC25A13 | 0.2567 | 6.9855 | 9.5543 | 0.0000 | 0.0000 | 33.5819 |
| ACAA1 | 0.3229 | 6.8833 | 9.5436 | 0.0000 | 0.0000 | 33.5006 |
| ORC1 | -0.8647 | 4.5353 | -9.5362 | 0.0000 | 0.0000 | 33.4443 |
| **MMP10** | -1.5570 | 1.9879 | -9.5320 | 0.0000 | 0.0000 | 33.4124 |
| TMEM51 | -0.7710 | 5.2763 | -9.5233 | 0.0000 | 0.0000 | 33.3460 |
| **AR** | 1.2647 | 5.6088 | 9.5185 | 0.0000 | 0.0000 | 33.3095 |
| **EPN3** | -1.1614 | 1.3762 | -9.5151 | 0.0000 | 0.0000 | 33.2844 |
| **GYS2** | 1.5913 | 5.6641 | 9.5108 | 0.0000 | 0.0000 | 33.2512 |
| PCBD1 | 0.2144 | 7.4322 | 9.4929 | 0.0000 | 0.0000 | 33.1157 |
| N4BP2L1 | 0.4515 | 5.7897 | 9.4903 | 0.0000 | 0.0000 | 33.0959 |
| NDUFAF1 | 0.2376 | 6.6058 | 9.4877 | 0.0000 | 0.0000 | 33.0764 |
| EIF4EBP2 | 0.2051 | 6.9915 | 9.4793 | 0.0000 | 0.0000 | 33.0126 |
| **GTSE1** | -1.0140 | 4.3959 | -9.4713 | 0.0000 | 0.0000 | 32.9518 |
| PDE2A | 0.8260 | 4.5218 | 9.4516 | 0.0000 | 0.0000 | 32.8030 |
| ADH1B | 0.8640 | 7.2793 | 9.4501 | 0.0000 | 0.0000 | 32.7910 |
| CCNG1 | 0.1924 | 7.0512 | 9.4431 | 0.0000 | 0.0000 | 32.7382 |
| ECM2 | 0.7472 | 5.7125 | 9.4423 | 0.0000 | 0.0000 | 32.7323 |
| TM7SF2 | 0.4543 | 7.0823 | 9.4388 | 0.0000 | 0.0000 | 32.7061 |
| **PKP3** | -1.5963 | 2.0288 | -9.4342 | 0.0000 | 0.0000 | 32.6711 |
| **RIBC2** | -1.3263 | 3.4702 | -9.4311 | 0.0000 | 0.0000 | 32.6480 |
| **SEMA3E** | -1.5476 | 1.1688 | -9.4302 | 0.0000 | 0.0000 | 32.6408 |
| GSTO1 | 0.2363 | 7.5680 | 9.4280 | 0.0000 | 0.0000 | 32.6242 |
| ACO1 | 0.2671 | 6.7904 | 9.4193 | 0.0000 | 0.0000 | 32.5587 |
| FAM107A | 0.7786 | 4.6041 | 9.4190 | 0.0000 | 0.0000 | 32.5562 |
| ACOX2 | 0.6381 | 6.7605 | 9.4126 | 0.0000 | 0.0000 | 32.5082 |
| LPCAT4 | -0.6440 | 4.7993 | -9.4080 | 0.0000 | 0.0000 | 32.4735 |
| ATOX1 | 0.3019 | 6.9068 | 9.4030 | 0.0000 | 0.0000 | 32.4354 |
| MRPS31 | 0.2557 | 6.3201 | 9.4016 | 0.0000 | 0.0000 | 32.4254 |
| C4orf46 | -0.5243 | 4.5765 | -9.3903 | 0.0000 | 0.0000 | 32.3400 |
| NFE2L3 | -0.7489 | 4.8809 | -9.3893 | 0.0000 | 0.0000 | 32.3321 |
| SEPHS2 | 0.1912 | 7.6340 | 9.3863 | 0.0000 | 0.0000 | 32.3095 |
| HIGD1A | 0.2389 | 7.1211 | 9.3859 | 0.0000 | 0.0000 | 32.3065 |
| APOE | 0.2270 | 8.2310 | 9.3812 | 0.0000 | 0.0000 | 32.2716 |
| **DMBT1** | -1.3045 | 1.0255 | -9.3769 | 0.0000 | 0.0000 | 32.2391 |
| CLPX | 0.2158 | 6.5467 | 9.3765 | 0.0000 | 0.0000 | 32.2356 |
| ACOT13 | 0.2612 | 6.6128 | 9.3755 | 0.0000 | 0.0000 | 32.2286 |
| SCP2 | 0.3237 | 7.0753 | 9.3737 | 0.0000 | 0.0000 | 32.2148 |

| **PTHLH** | -1.4929 | 2.5903 | -9.3733 | 0.0000 | 0.0000 | 32.2119 |
| --- | --- | --- | --- | --- | --- | --- |
| ACAA2 | 0.2905 | 7.1613 | 9.3690 | 0.0000 | 0.0000 | 32.1795 |
| PLK1 | -0.8966 | 5.0502 | -9.3592 | 0.0000 | 0.0000 | 32.1054 |
| TST | 0.2566 | 7.5849 | 9.3524 | 0.0000 | 0.0000 | 32.0546 |
| **CA5A** | 1.3851 | 4.8243 | 9.3503 | 0.0000 | 0.0000 | 32.0389 |
| OCEL1 | 0.2702 | 6.8389 | 9.3445 | 0.0000 | 0.0000 | 31.9953 |
| SLC25A42 | 0.4210 | 6.6283 | 9.3436 | 0.0000 | 0.0000 | 31.9882 |
| PLVAP | 0.2607 | 7.1199 | 9.3406 | 0.0000 | 0.0000 | 31.9663 |
| MTHFD1 | 0.3936 | 6.7771 | 9.3389 | 0.0000 | 0.0000 | 31.9529 |
| TTC38 | 0.2718 | 7.1371 | 9.3383 | 0.0000 | 0.0000 | 31.9484 |
| **CLEC5A** | -1.1680 | 1.7766 | -9.3332 | 0.0000 | 0.0000 | 31.9101 |
| SLC31A1 | 0.2481 | 6.8123 | 9.3274 | 0.0000 | 0.0000 | 31.8666 |
| SERINC3 | 0.1324 | 7.0193 | 9.3211 | 0.0000 | 0.0000 | 31.8199 |
| **IL20RA** | -1.5429 | 1.2543 | -9.3156 | 0.0000 | 0.0000 | 31.7785 |
| FMO3 | 0.7684 | 7.1058 | 9.3039 | 0.0000 | 0.0000 | 31.6909 |
| PRDX3 | 0.1849 | 7.2901 | 9.2984 | 0.0000 | 0.0000 | 31.6498 |
| POLDIP2 | 0.1422 | 7.4642 | 9.2963 | 0.0000 | 0.0000 | 31.6333 |
| CDCA4 | -0.4391 | 5.4532 | -9.2900 | 0.0000 | 0.0000 | 31.5869 |
| KCNJ8 | 0.5976 | 6.4629 | 9.2779 | 0.0000 | 0.0000 | 31.4962 |
| SARDH | 0.5890 | 6.6533 | 9.2753 | 0.0000 | 0.0000 | 31.4763 |
| SIAH2 | 0.2521 | 6.9131 | 9.2579 | 0.0000 | 0.0000 | 31.3467 |
| GHITM | 0.1582 | 7.3984 | 9.2568 | 0.0000 | 0.0000 | 31.3383 |
| ACADSB | 0.4100 | 6.9242 | 9.2392 | 0.0000 | 0.0000 | 31.2068 |
| **TFF2** | -1.9355 | 1.8853 | -9.2349 | 0.0000 | 0.0000 | 31.1752 |
| EI24 | 0.1762 | 7.1570 | 9.2316 | 0.0000 | 0.0000 | 31.1504 |
| **PCK1** | 1.1357 | 6.8286 | 9.2310 | 0.0000 | 0.0000 | 31.1456 |
| **ABCA6** | 1.0208 | 5.6093 | 9.2293 | 0.0000 | 0.0000 | 31.1327 |
| SMARCD1 | -0.1842 | 6.2680 | -9.2291 | 0.0000 | 0.0000 | 31.1315 |
| B3GALT5 | -0.9722 | 0.6696 | -9.2112 | 0.0000 | 0.0000 | 30.9984 |
| RNASE4 | 0.6072 | 6.0513 | 9.2067 | 0.0000 | 0.0000 | 30.9648 |
| **ADORA2B** | -1.1478 | 2.9917 | -9.1985 | 0.0000 | 0.0000 | 30.9040 |
| FBXO31 | 0.4207 | 6.3216 | 9.1934 | 0.0000 | 0.0000 | 30.8655 |
| **MCM10** | -1.0306 | 3.8244 | -9.1929 | 0.0000 | 0.0000 | 30.8619 |
| ECHDC2 | 0.3646 | 6.5886 | 9.1867 | 0.0000 | 0.0000 | 30.8158 |
| **LECT2** | 1.5829 | 6.2029 | 9.1858 | 0.0000 | 0.0000 | 30.8095 |
| E2F2 | -0.9870 | 3.5441 | -9.1856 | 0.0000 | 0.0000 | 30.8076 |
| SUOX | 0.2771 | 6.4162 | 9.1786 | 0.0000 | 0.0000 | 30.7558 |
| **CYP3A43** | 1.5177 | 3.7926 | 9.1749 | 0.0000 | 0.0000 | 30.7279 |
| TACO1 | 0.2217 | 6.8886 | 9.1746 | 0.0000 | 0.0000 | 30.7260 |
| LPAR2 | -0.9248 | 4.4416 | -9.1731 | 0.0000 | 0.0000 | 30.7149 |
| PC | 0.4013 | 7.0088 | 9.1629 | 0.0000 | 0.0000 | 30.6391 |
| ST3GAL1 | 0.4494 | 6.5733 | 9.1497 | 0.0000 | 0.0000 | 30.5410 |
| RXRA | 0.2575 | 6.7784 | 9.1495 | 0.0000 | 0.0000 | 30.5399 |
| **KIF18A** | -1.0193 | 3.6304 | -9.1448 | 0.0000 | 0.0000 | 30.5049 |
| **CDH17** | -1.3984 | 1.2627 | -9.1330 | 0.0000 | 0.0000 | 30.4179 |
| MLYCD | 0.5044 | 5.0447 | 9.1245 | 0.0000 | 0.0000 | 30.3544 |
| **MASP2** | 1.0656 | 6.7268 | 9.1172 | 0.0000 | 0.0000 | 30.3006 |
| GINS3 | -0.6078 | 4.1894 | -9.1138 | 0.0000 | 0.0000 | 30.2754 |
| **CYP7A1** | 1.8000 | 5.6980 | 9.1115 | 0.0000 | 0.0000 | 30.2584 |
| ACAT1 | 0.2941 | 7.1488 | 9.1054 | 0.0000 | 0.0000 | 30.2133 |
| CENPE | -0.9324 | 3.7005 | -9.1032 | 0.0000 | 0.0000 | 30.1973 |
| FZD1 | -0.8194 | 4.3600 | -9.0999 | 0.0000 | 0.0000 | 30.1723 |
| ECT2 | -0.7182 | 5.1660 | -9.0998 | 0.0000 | 0.0000 | 30.1718 |

| DHRS3 | 0.2151 | 7.3767 | 9.0962 | 0.0000 | 0.0000 | 30.1453 |
| --- | --- | --- | --- | --- | --- | --- |
| **HK2** | -1.3230 | 3.9340 | -9.0888 | 0.0000 | 0.0000 | 30.0909 |
| YEATS2 | -0.4081 | 5.0837 | -9.0865 | 0.0000 | 0.0000 | 30.0735 |
| PALMD | 0.6390 | 5.7862 | 9.0815 | 0.0000 | 0.0000 | 30.0371 |
| ZFAND5 | 0.2991 | 6.9035 | 9.0799 | 0.0000 | 0.0000 | 30.0247 |
| ETFA | 0.2015 | 7.0811 | 9.0623 | 0.0000 | 0.0000 | 29.8956 |
| SOX4 | -0.8131 | 5.6761 | -9.0613 | 0.0000 | 0.0000 | 29.8880 |
| ACSF2 | 0.3566 | 6.5055 | 9.0610 | 0.0000 | 0.0000 | 29.8853 |
| OSGIN1 | 0.4959 | 7.0943 | 9.0523 | 0.0000 | 0.0000 | 29.8218 |
| PLAUR | -0.7711 | 4.6154 | -9.0391 | 0.0000 | 0.0000 | 29.7246 |
| FANCI | -0.6589 | 4.9339 | -9.0381 | 0.0000 | 0.0000 | 29.7170 |
| PHYH | 0.2918 | 7.2926 | 9.0363 | 0.0000 | 0.0000 | 29.7041 |
| **IL4I1** | -1.0374 | 4.3388 | -9.0302 | 0.0000 | 0.0000 | 29.6592 |
| ESD | 0.2033 | 6.9544 | 9.0275 | 0.0000 | 0.0000 | 29.6392 |
| BPHL | 0.3559 | 6.3968 | 9.0179 | 0.0000 | 0.0000 | 29.5683 |
| RUNDC3A | -0.9711 | 1.5279 | -9.0130 | 0.0000 | 0.0000 | 29.5324 |
| ARMC9 | -0.7664 | 3.2283 | -9.0128 | 0.0000 | 0.0000 | 29.5314 |
| **CYP2A6** | 1.5487 | 6.5741 | 9.0022 | 0.0000 | 0.0000 | 29.4532 |
| SHCBP1 | -0.9956 | 4.0454 | -8.9936 | 0.0000 | 0.0000 | 29.3901 |
| TRIM45 | -0.7428 | 4.2952 | -8.9879 | 0.0000 | 0.0000 | 29.3484 |
| **MUC1** | -1.2919 | 3.1873 | -8.9840 | 0.0000 | 0.0000 | 29.3199 |
| SDHD | 0.1904 | 7.0747 | 8.9837 | 0.0000 | 0.0000 | 29.3179 |
| ACRV1 | -0.7643 | 1.3140 | -8.9820 | 0.0000 | 0.0000 | 29.3052 |
| GLUD1 | 0.2362 | 7.4707 | 8.9810 | 0.0000 | 0.0000 | 29.2981 |
| BLVRB | 0.2291 | 7.4074 | 8.9766 | 0.0000 | 0.0000 | 29.2657 |
| SLC6A12 | 0.9611 | 5.9390 | 8.9666 | 0.0000 | 0.0000 | 29.1921 |
| SCN4A | 0.8929 | 2.7135 | 8.9556 | 0.0000 | 0.0000 | 29.1120 |
| PXMP2 | 0.3314 | 7.0910 | 8.9493 | 0.0000 | 0.0000 | 29.0655 |
| XRCC2 | -0.9364 | 3.5927 | -8.9488 | 0.0000 | 0.0000 | 29.0621 |
| TRIP13 | -0.8405 | 4.6673 | -8.9453 | 0.0000 | 0.0000 | 29.0363 |
| ROBO4 | 0.4905 | 5.4280 | 8.9416 | 0.0000 | 0.0000 | 29.0098 |
| HPD | 0.9528 | 7.3615 | 8.9327 | 0.0000 | 0.0000 | 28.9445 |
| BTD | 0.2950 | 6.6617 | 8.9263 | 0.0000 | 0.0000 | 28.8981 |
| MMD | -0.4645 | 5.9345 | -8.9170 | 0.0000 | 0.0000 | 28.8303 |
| **ULBP1** | -1.0671 | 1.4820 | -8.9148 | 0.0000 | 0.0000 | 28.8135 |
| SEC14L2 | 0.6928 | 6.5510 | 8.9119 | 0.0000 | 0.0000 | 28.7929 |
| ASMTL | 0.2717 | 6.5475 | 8.9091 | 0.0000 | 0.0000 | 28.7724 |
| SUCLG2 | 0.2329 | 7.1074 | 8.9076 | 0.0000 | 0.0000 | 28.7612 |
| KIF11 | -0.6713 | 4.9301 | -8.9073 | 0.0000 | 0.0000 | 28.7593 |
| SLC25A30 | 0.4796 | 6.0839 | 8.9017 | 0.0000 | 0.0000 | 28.7182 |
| **CENPA** | -1.0025 | 4.6153 | -8.8942 | 0.0000 | 0.0000 | 28.6635 |
| **KIF18B** | -1.0122 | 4.4454 | -8.8930 | 0.0000 | 0.0000 | 28.6553 |
| ERCC6L | -0.9642 | 3.1743 | -8.8930 | 0.0000 | 0.0000 | 28.6551 |
| IGFBP2 | 0.6068 | 7.0957 | 8.8890 | 0.0000 | 0.0000 | 28.6260 |
| **EPHB6** | -1.1626 | 3.8442 | -8.8856 | 0.0000 | 0.0000 | 28.6013 |
| PAFAH1B3 | -0.5190 | 6.3247 | -8.8803 | 0.0000 | 0.0000 | 28.5627 |
| ADK | 0.2803 | 6.6383 | 8.8784 | 0.0000 | 0.0000 | 28.5490 |
| UAP1L1 | -0.9139 | 4.3831 | -8.8737 | 0.0000 | 0.0000 | 28.5149 |
| ELL2 | 0.3272 | 6.6636 | 8.8678 | 0.0000 | 0.0000 | 28.4720 |
| LRRC3 | 0.8808 | 5.6098 | 8.8576 | 0.0000 | 0.0000 | 28.3973 |
| MTCH2 | 0.1452 | 7.2232 | 8.8527 | 0.0000 | 0.0000 | 28.3622 |
| NIT1 | 0.2096 | 6.6829 | 8.8496 | 0.0000 | 0.0000 | 28.3397 |
| SRSF5 | 0.1421 | 6.9047 | 8.8491 | 0.0000 | 0.0000 | 28.3355 |

| **ABCB11** | 1.5711 | 5.1531 | 8.8393 | 0.0000 | 0.0000 | 28.2650 |
| --- | --- | --- | --- | --- | --- | --- |
| MYBL2 | -0.8967 | 5.7266 | -8.8390 | 0.0000 | 0.0000 | 28.2627 |
| **TAT** | 1.0737 | 6.8697 | 8.8365 | 0.0000 | 0.0000 | 28.2447 |
| ATP13A2 | -0.3415 | 5.7201 | -8.8315 | 0.0000 | 0.0000 | 28.2081 |
| MKI67 | -0.8343 | 5.1039 | -8.8290 | 0.0000 | 0.0000 | 28.1899 |
| STX1A | -0.5473 | 4.0006 | -8.8259 | 0.0000 | 0.0000 | 28.1678 |
| MGST2 | 0.2399 | 7.0329 | 8.8246 | 0.0000 | 0.0000 | 28.1584 |
| IMPDH1 | -0.6080 | 5.4802 | -8.8241 | 0.0000 | 0.0000 | 28.1544 |
| BUB1B | -0.9597 | 4.5360 | -8.8218 | 0.0000 | 0.0000 | 28.1378 |
| OS9 | 0.1368 | 7.3220 | 8.8205 | 0.0000 | 0.0000 | 28.1285 |
| SLC30A1 | 0.3257 | 6.7158 | 8.8130 | 0.0000 | 0.0000 | 28.0744 |
| CASP2 | -0.3681 | 5.3290 | -8.8101 | 0.0000 | 0.0000 | 28.0531 |
| PUS3 | 0.2174 | 6.4412 | 8.7958 | 0.0000 | 0.0000 | 27.9500 |
| REEP5 | 0.1673 | 7.0876 | 8.7898 | 0.0000 | 0.0000 | 27.9068 |
| KLHDC10 | 0.2730 | 6.3850 | 8.7894 | 0.0000 | 0.0000 | 27.9035 |
| DLGAP5 | -0.9075 | 4.7164 | -8.7888 | 0.0000 | 0.0000 | 27.8990 |
| MUT | 0.2667 | 6.9325 | 8.7856 | 0.0000 | 0.0000 | 27.8764 |
| **GCNT3** | -1.5466 | 3.2938 | -8.7831 | 0.0000 | 0.0000 | 27.8579 |
| **DNM1** | -1.1088 | 3.5077 | -8.7672 | 0.0000 | 0.0000 | 27.7430 |
| GPD2 | -0.4456 | 5.0686 | -8.7569 | 0.0000 | 0.0000 | 27.6688 |
| SLC2A1 | -0.8914 | 4.7452 | -8.7546 | 0.0000 | 0.0000 | 27.6524 |
| RAD23B | 0.1345 | 7.1526 | 8.7540 | 0.0000 | 0.0000 | 27.6484 |
| **CYP26B1** | -1.3988 | 2.6110 | -8.7528 | 0.0000 | 0.0000 | 27.6392 |
| CDCA8 | -0.6632 | 5.4695 | -8.7489 | 0.0000 | 0.0000 | 27.6114 |
| **SLC6A8** | -1.0593 | 5.1588 | -8.7363 | 0.0000 | 0.0000 | 27.5206 |
| ZDHHC13 | -0.9099 | 3.6907 | -8.7306 | 0.0000 | 0.0000 | 27.4799 |
| ORAI2 | -0.6671 | 4.1282 | -8.7301 | 0.0000 | 0.0000 | 27.4762 |
| PNMA1 | -0.4331 | 5.8820 | -8.7163 | 0.0000 | 0.0000 | 27.3770 |
| ETFDH | 0.3453 | 6.4606 | 8.7092 | 0.0000 | 0.0000 | 27.3259 |
| ITPR2 | 0.4599 | 6.2369 | 8.7053 | 0.0000 | 0.0000 | 27.2976 |
| UBE2B | 0.1627 | 6.7707 | 8.7031 | 0.0000 | 0.0000 | 27.2820 |
| STEAP1B | -0.9737 | 1.5298 | -8.7005 | 0.0000 | 0.0000 | 27.2635 |
| HMGCL | 0.2866 | 6.8936 | 8.7002 | 0.0000 | 0.0000 | 27.2613 |
| MTF2 | -0.3494 | 5.1875 | -8.6998 | 0.0000 | 0.0000 | 27.2586 |
| DCAF8 | 0.1842 | 6.5892 | 8.6914 | 0.0000 | 0.0000 | 27.1983 |
| ARHGEF2 | -0.4933 | 5.5249 | -8.6854 | 0.0000 | 0.0000 | 27.1551 |
| BUB1 | -0.8887 | 4.5701 | -8.6819 | 0.0000 | 0.0000 | 27.1298 |
| CHEK1 | -0.6047 | 4.6977 | -8.6818 | 0.0000 | 0.0000 | 27.1294 |
| MPP3 | -0.9937 | 3.4868 | -8.6792 | 0.0000 | 0.0000 | 27.1105 |
| KCNMB3 | -0.8164 | 2.2635 | -8.6715 | 0.0000 | 0.0000 | 27.0552 |
| **TRPV6** | -1.1837 | 0.9923 | -8.6704 | 0.0000 | 0.0000 | 27.0477 |
| IL11 | -0.8951 | 1.2215 | -8.6695 | 0.0000 | 0.0000 | 27.0411 |
| TCTN2 | -0.7358 | 4.1664 | -8.6665 | 0.0000 | 0.0000 | 27.0193 |
| SFXN1 | 0.2522 | 6.5371 | 8.6624 | 0.0000 | 0.0000 | 26.9902 |
| FAH | 0.3046 | 6.8783 | 8.6613 | 0.0000 | 0.0000 | 26.9821 |
| MOCS2 | 0.2409 | 6.4336 | 8.6609 | 0.0000 | 0.0000 | 26.9795 |
| AMFR | 0.2030 | 6.9271 | 8.6571 | 0.0000 | 0.0000 | 26.9523 |
| RNFT2 | -0.9911 | 2.9148 | -8.6542 | 0.0000 | 0.0000 | 26.9318 |
| **USH1C** | -2.0527 | 3.0707 | -8.6516 | 0.0000 | 0.0000 | 26.9131 |
| GAMT | 0.2911 | 7.5502 | 8.6510 | 0.0000 | 0.0000 | 26.9089 |
| GADD45A | 0.3199 | 6.9118 | 8.6483 | 0.0000 | 0.0000 | 26.8893 |
| ABCC1 | -0.8025 | 4.8850 | -8.6473 | 0.0000 | 0.0000 | 26.8821 |
| ACSL1 | 0.4027 | 7.2148 | 8.6451 | 0.0000 | 0.0000 | 26.8663 |

| **OBSCN** | -1.2551 | 2.2879 | -8.6392 | 0.0000 | 0.0000 | 26.8244 |
| --- | --- | --- | --- | --- | --- | --- |
| HDHD3 | 0.2090 | 7.0451 | 8.6351 | 0.0000 | 0.0000 | 26.7948 |
| HNMT | 0.2512 | 6.8384 | 8.6345 | 0.0000 | 0.0000 | 26.7909 |
| ISOC1 | 0.2277 | 6.8990 | 8.6321 | 0.0000 | 0.0000 | 26.7734 |
| PIPOX | 0.5617 | 7.1848 | 8.6295 | 0.0000 | 0.0000 | 26.7552 |
| KLF15 | 0.5493 | 6.8903 | 8.6275 | 0.0000 | 0.0000 | 26.7408 |
| **VTCN1** | -1.6149 | 1.9962 | -8.6274 | 0.0000 | 0.0000 | 26.7398 |
| FAM83E | -0.9629 | 0.9263 | -8.6260 | 0.0000 | 0.0000 | 26.7298 |
| SLC47A1 | 0.6417 | 6.5239 | 8.6221 | 0.0000 | 0.0000 | 26.7023 |
| **BCL11A** | -1.0888 | 1.7901 | -8.6195 | 0.0000 | 0.0000 | 26.6839 |
| SLC25A20 | 0.2767 | 6.9848 | 8.6193 | 0.0000 | 0.0000 | 26.6822 |
| **ARNT2** | -1.4542 | 2.9100 | -8.6173 | 0.0000 | 0.0000 | 26.6677 |
| CACNB1 | -0.7392 | 2.5834 | -8.6145 | 0.0000 | 0.0000 | 26.6478 |
| MOCS1 | 0.3336 | 6.3782 | 8.6134 | 0.0000 | 0.0000 | 26.6402 |
| SIRT5 | 0.2876 | 6.0525 | 8.6059 | 0.0000 | 0.0000 | 26.5867 |
| PGRMC2 | 0.1702 | 6.8866 | 8.6058 | 0.0000 | 0.0000 | 26.5863 |
| HSPA9 | 0.1422 | 7.4086 | 8.5900 | 0.0000 | 0.0000 | 26.4732 |
| PSMC3IP | -0.6721 | 4.1561 | -8.5866 | 0.0000 | 0.0000 | 26.4495 |
| **GPRIN2** | -1.3360 | 1.1742 | -8.5862 | 0.0000 | 0.0000 | 26.4464 |
| GINS1 | -0.7687 | 5.0429 | -8.5855 | 0.0000 | 0.0000 | 26.4414 |
| CDC7 | -0.6684 | 4.6153 | -8.5617 | 0.0000 | 0.0000 | 26.2725 |
| STIL | -0.7465 | 3.9166 | -8.5598 | 0.0000 | 0.0000 | 26.2588 |
| **STEAP4** | 1.1381 | 4.0793 | 8.5589 | 0.0000 | 0.0000 | 26.2521 |
| LARP4 | 0.2028 | 6.4008 | 8.5571 | 0.0000 | 0.0000 | 26.2394 |
| RIT1 | -0.2536 | 5.7984 | -8.5568 | 0.0000 | 0.0000 | 26.2377 |
| NUP62 | -0.1862 | 6.2877 | -8.5568 | 0.0000 | 0.0000 | 26.2373 |
| POLQ | -0.9395 | 3.0051 | -8.5541 | 0.0000 | 0.0000 | 26.2184 |
| **KLHDC8A** | -1.1675 | 1.5634 | -8.5538 | 0.0000 | 0.0000 | 26.2161 |
| UQCRC2 | 0.1783 | 6.9616 | 8.5513 | 0.0000 | 0.0000 | 26.1986 |
| **C1orf116** | -1.4695 | 2.8465 | -8.5507 | 0.0000 | 0.0000 | 26.1940 |
| **ABCA8** | 1.3087 | 4.5336 | 8.5458 | 0.0000 | 0.0000 | 26.1594 |
| DCAF6 | 0.1746 | 6.7781 | 8.5414 | 0.0000 | 0.0000 | 26.1280 |
| HLF | 0.7742 | 6.1988 | 8.5341 | 0.0000 | 0.0000 | 26.0765 |
| LIMS2 | 0.5290 | 5.7635 | 8.5325 | 0.0000 | 0.0000 | 26.0649 |
| TMC6 | -0.7156 | 5.0306 | -8.5320 | 0.0000 | 0.0000 | 26.0615 |
| AACS | -0.5672 | 4.5090 | -8.5283 | 0.0000 | 0.0000 | 26.0354 |
| BICD1 | -0.8152 | 3.0051 | -8.5178 | 0.0000 | 0.0000 | 25.9610 |
| CCNE1 | -0.8662 | 4.6932 | -8.5153 | 0.0000 | 0.0000 | 25.9437 |
| SMG9 | -0.2999 | 5.5754 | -8.5145 | 0.0000 | 0.0000 | 25.9377 |
| **ARL14** | -1.6062 | 2.6423 | -8.5126 | 0.0000 | 0.0000 | 25.9243 |
| CENPI | -0.8800 | 3.5625 | -8.5126 | 0.0000 | 0.0000 | 25.9243 |
| ACBD4 | 0.3627 | 6.5519 | 8.5094 | 0.0000 | 0.0000 | 25.9013 |
| MAD2L1 | -0.6614 | 4.7758 | -8.5010 | 0.0000 | 0.0000 | 25.8425 |
| TM6SF2 | 0.8509 | 5.7998 | 8.5008 | 0.0000 | 0.0000 | 25.8409 |
| **NTS** | -1.8830 | 2.8582 | -8.4977 | 0.0000 | 0.0000 | 25.8187 |
| ALDH3A2 | 0.2550 | 7.1369 | 8.4972 | 0.0000 | 0.0000 | 25.8156 |
| **DAO** | 1.0963 | 6.0944 | 8.4943 | 0.0000 | 0.0000 | 25.7951 |
| **OTC** | 1.1890 | 6.7338 | 8.4920 | 0.0000 | 0.0000 | 25.7784 |
| ACSL5 | 0.4897 | 6.7859 | 8.4907 | 0.0000 | 0.0000 | 25.7693 |
| HPR | 0.8905 | 7.2594 | 8.4839 | 0.0000 | 0.0000 | 25.7214 |
| ADH5 | 0.1886 | 7.1225 | 8.4837 | 0.0000 | 0.0000 | 25.7202 |
| CDC6 | -0.7283 | 5.1908 | -8.4813 | 0.0000 | 0.0000 | 25.7034 |
| GLRX5 | 0.1980 | 6.9290 | 8.4809 | 0.0000 | 0.0000 | 25.7001 |

| CCNF | -0.6098 | 4.7831 | -8.4775 | 0.0000 | 0.0000 | 25.6761 |
| --- | --- | --- | --- | --- | --- | --- |
| PLK4 | -0.7283 | 3.9014 | -8.4761 | 0.0000 | 0.0000 | 25.6667 |
| **CLSPN** | -1.0152 | 3.0078 | -8.4718 | 0.0000 | 0.0000 | 25.6358 |
| GRINA | 0.1678 | 7.5998 | 8.4649 | 0.0000 | 0.0000 | 25.5878 |
| SMOX | -0.6704 | 5.5176 | -8.4632 | 0.0000 | 0.0000 | 25.5753 |
| PSORS1C1 | -0.9802 | 1.7190 | -8.4631 | 0.0000 | 0.0000 | 25.5748 |
| TSKU | 0.5393 | 7.0022 | 8.4575 | 0.0000 | 0.0000 | 25.5356 |
| CCS | 0.2244 | 6.8908 | 8.4548 | 0.0000 | 0.0000 | 25.5166 |
| TTR | 0.5453 | 7.7902 | 8.4493 | 0.0000 | 0.0000 | 25.4774 |
| NCAPD2 | -0.3645 | 5.8685 | -8.4492 | 0.0000 | 0.0000 | 25.4768 |
| CDC25A | -0.8389 | 4.2771 | -8.4488 | 0.0000 | 0.0000 | 25.4739 |
| ALDH1L1 | 0.9677 | 6.5764 | 8.4453 | 0.0000 | 0.0000 | 25.4494 |
| NCAPH | -0.7572 | 4.8704 | -8.4428 | 0.0000 | 0.0000 | 25.4322 |
| CHST12 | -0.3585 | 4.2844 | -8.4416 | 0.0000 | 0.0000 | 25.4238 |
| GPSM2 | -0.6359 | 4.0099 | -8.4324 | 0.0000 | 0.0000 | 25.3584 |
| SURF1 | 0.1913 | 7.0586 | 8.4320 | 0.0000 | 0.0000 | 25.3561 |
| AIFM1 | 0.1955 | 7.0755 | 8.4304 | 0.0000 | 0.0000 | 25.3448 |
| **SLC34A2** | -1.7275 | 2.1157 | -8.4228 | 0.0000 | 0.0000 | 25.2915 |
| PTMS | 0.1822 | 7.7756 | 8.4213 | 0.0000 | 0.0000 | 25.2808 |
| UGP2 | 0.2437 | 7.0796 | 8.4209 | 0.0000 | 0.0000 | 25.2783 |
| HAUS6 | -0.4068 | 4.9329 | -8.4207 | 0.0000 | 0.0000 | 25.2767 |
| **FCGBP** | -1.0540 | 3.5401 | -8.4196 | 0.0000 | 0.0000 | 25.2687 |
| RAP1GAP | -0.6330 | 6.0180 | -8.4151 | 0.0000 | 0.0000 | 25.2376 |
| **KRT19** | -1.9155 | 3.8829 | -8.4140 | 0.0000 | 0.0000 | 25.2295 |
| **TNNT1** | -1.4461 | 2.0615 | -8.4080 | 0.0000 | 0.0000 | 25.1872 |
| ASF1B | -0.6505 | 5.5625 | -8.4053 | 0.0000 | 0.0000 | 25.1684 |
| UQCRQ | 0.2043 | 7.6180 | 8.4050 | 0.0000 | 0.0000 | 25.1661 |
| GIT1 | -0.2113 | 6.1042 | -8.4023 | 0.0000 | 0.0000 | 25.1478 |
| PTPRN | -0.4880 | 0.4391 | -8.3986 | 0.0000 | 0.0000 | 25.1216 |
| **GPLD1** | 1.1700 | 5.3779 | 8.3926 | 0.0000 | 0.0000 | 25.0797 |
| **ZNF239** | -1.3030 | 3.3262 | -8.3897 | 0.0000 | 0.0000 | 25.0592 |
| TROAP | -0.8869 | 4.9772 | -8.3895 | 0.0000 | 0.0000 | 25.0576 |
| DGCR6L | 0.2098 | 7.2113 | 8.3888 | 0.0000 | 0.0000 | 25.0527 |
| **MAPK13** | -1.1473 | 4.5205 | -8.3818 | 0.0000 | 0.0000 | 25.0040 |
| SLC25A15 | 0.5162 | 6.5141 | 8.3816 | 0.0000 | 0.0000 | 25.0023 |
| **REN** | 1.4176 | 3.6644 | 8.3816 | 0.0000 | 0.0000 | 25.0021 |
| **KIF15** | -1.0010 | 3.7813 | -8.3718 | 0.0000 | 0.0000 | 24.9336 |
| ZNHIT1 | 0.2308 | 6.9693 | 8.3654 | 0.0000 | 0.0000 | 24.8889 |
| **FOLR1** | -1.5707 | 2.5941 | -8.3607 | 0.0000 | 0.0000 | 24.8564 |
| **TPBG** | -1.1859 | 2.6310 | -8.3600 | 0.0000 | 0.0000 | 24.8513 |
| GNMT | 0.8544 | 6.7199 | 8.3559 | 0.0000 | 0.0000 | 24.8229 |
| SERPINC1 | 0.6618 | 7.9065 | 8.3546 | 0.0000 | 0.0000 | 24.8139 |
| **DLX4** | -1.0339 | 1.2940 | -8.3515 | 0.0000 | 0.0000 | 24.7920 |
| PSMC2 | 0.1369 | 7.0112 | 8.3394 | 0.0000 | 0.0000 | 24.7078 |
| ATF7IP2 | 0.4833 | 5.6421 | 8.3379 | 0.0000 | 0.0000 | 24.6968 |
| TOB1 | 0.2621 | 7.1451 | 8.3223 | 0.0000 | 0.0000 | 24.5885 |
| PMPCB | 0.1698 | 6.6690 | 8.3172 | 0.0000 | 0.0000 | 24.5529 |
| CDK16 | -0.2094 | 6.2988 | -8.3170 | 0.0000 | 0.0000 | 24.5514 |
| RDH16 | 0.9994 | 6.5488 | 8.3140 | 0.0000 | 0.0000 | 24.5305 |
| SIRT3 | 0.2094 | 6.0763 | 8.3121 | 0.0000 | 0.0000 | 24.5174 |
| ASS1 | 0.2879 | 7.6090 | 8.3094 | 0.0000 | 0.0000 | 24.4987 |
| **NEIL3** | -1.0531 | 3.5912 | -8.3015 | 0.0000 | 0.0000 | 24.4433 |
| CDC20 | -0.6784 | 6.0005 | -8.2993 | 0.0000 | 0.0000 | 24.4284 |

| EFHC2 | -0.9207 | 0.8777 | -8.2991 | 0.0000 | 0.0000 | 24.4269 |
| --- | --- | --- | --- | --- | --- | --- |
| AP4M1 | -0.2927 | 5.3319 | -8.2977 | 0.0000 | 0.0000 | 24.4171 |
| LAPTM4A | 0.1088 | 7.5309 | 8.2949 | 0.0000 | 0.0000 | 24.3974 |
| SAP30L | 0.2108 | 6.0655 | 8.2947 | 0.0000 | 0.0000 | 24.3963 |
| PLXNA1 | -0.5548 | 5.0990 | -8.2947 | 0.0000 | 0.0000 | 24.3962 |
| **PKIA** | -1.1171 | 1.8297 | -8.2934 | 0.0000 | 0.0000 | 24.3869 |
| GABARAPL1 | 0.3698 | 6.7333 | 8.2890 | 0.0000 | 0.0000 | 24.3564 |
| **SPINT1** | -1.3895 | 4.7348 | -8.2877 | 0.0000 | 0.0000 | 24.3478 |
| **SLC6A14** | -1.0970 | 0.7392 | -8.2845 | 0.0000 | 0.0000 | 24.3252 |
| F12 | 0.6658 | 7.3912 | 8.2800 | 0.0000 | 0.0000 | 24.2939 |
| **CYP3A4** | 1.5194 | 6.2511 | 8.2784 | 0.0000 | 0.0000 | 24.2832 |
| COBLL1 | 0.4973 | 5.7625 | 8.2681 | 0.0000 | 0.0000 | 24.2119 |
| S1PR1 | 0.4204 | 6.1383 | 8.2671 | 0.0000 | 0.0000 | 24.2043 |
| C12orf49 | -0.4926 | 5.3719 | -8.2657 | 0.0000 | 0.0000 | 24.1950 |
| PNPO | 0.3033 | 6.7624 | 8.2525 | 0.0000 | 0.0000 | 24.1037 |
| C15orf39 | -0.3696 | 5.7351 | -8.2479 | 0.0000 | 0.0000 | 24.0713 |
| TUFM | 0.1398 | 7.4880 | 8.2403 | 0.0000 | 0.0000 | 24.0189 |
| MIA3 | 0.2486 | 6.5972 | 8.2393 | 0.0000 | 0.0000 | 24.0120 |
| SPC25 | -0.7434 | 4.8971 | -8.2392 | 0.0000 | 0.0000 | 24.0115 |
| CENPF | -0.8275 | 4.9264 | -8.2347 | 0.0000 | 0.0000 | 23.9804 |
| RANBP10 | 0.2982 | 5.9645 | 8.2287 | 0.0000 | 0.0000 | 23.9386 |
| CDCA3 | -0.7710 | 4.6116 | -8.2282 | 0.0000 | 0.0000 | 23.9351 |
| CASQ2 | 0.9989 | 3.2200 | 8.2228 | 0.0000 | 0.0000 | 23.8981 |
| BLM | -0.8857 | 3.5996 | -8.2087 | 0.0000 | 0.0000 | 23.8004 |
| **ADAM12** | -1.1461 | 2.2983 | -8.2079 | 0.0000 | 0.0000 | 23.7954 |
| PCYOX1L | -0.6636 | 3.6786 | -8.2056 | 0.0000 | 0.0000 | 23.7793 |
| CTSO | 0.3170 | 6.7241 | 8.2038 | 0.0000 | 0.0000 | 23.7667 |
| **CTNND2** | -1.9216 | 2.5491 | -8.2027 | 0.0000 | 0.0000 | 23.7595 |
| RETSAT | 0.2446 | 7.0633 | 8.1969 | 0.0000 | 0.0000 | 23.7190 |
| C1R | 0.2440 | 7.5622 | 8.1966 | 0.0000 | 0.0000 | 23.7172 |
| BNIP3 | 0.2371 | 6.9242 | 8.1940 | 0.0000 | 0.0000 | 23.6993 |
| KIF4A | -0.8152 | 5.0717 | -8.1915 | 0.0000 | 0.0000 | 23.6820 |
| **FABP6** | -1.3710 | 1.4508 | -8.1890 | 0.0000 | 0.0000 | 23.6650 |
| SHMT1 | 0.4167 | 6.8930 | 8.1890 | 0.0000 | 0.0000 | 23.6648 |
| PGLYRP4 | -0.6310 | 0.4630 | -8.1879 | 0.0000 | 0.0000 | 23.6574 |
| RAD51 | -0.7337 | 4.4553 | -8.1855 | 0.0000 | 0.0000 | 23.6408 |
| RNF14 | 0.1636 | 6.4547 | 8.1823 | 0.0000 | 0.0000 | 23.6184 |
| **GULP1** | -1.3532 | 1.9158 | -8.1813 | 0.0000 | 0.0000 | 23.6121 |
| **SIX2** | -1.3452 | 2.0591 | -8.1812 | 0.0000 | 0.0000 | 23.6113 |
| MCM6 | -0.3307 | 6.2091 | -8.1809 | 0.0000 | 0.0000 | 23.6088 |
| NRSN2 | -0.8014 | 5.3656 | -8.1751 | 0.0000 | 0.0000 | 23.5690 |
| DDN | -0.7261 | 0.8266 | -8.1695 | 0.0000 | 0.0000 | 23.5303 |
| DDX11 | -0.5588 | 4.8308 | -8.1659 | 0.0000 | 0.0000 | 23.5058 |
| DLL3 | -0.7777 | 0.6136 | -8.1646 | 0.0000 | 0.0000 | 23.4966 |
| CNBP | 0.1047 | 7.5102 | 8.1641 | 0.0000 | 0.0000 | 23.4936 |
| NAA40 | -0.3420 | 5.3736 | -8.1635 | 0.0000 | 0.0000 | 23.4894 |
| TNFRSF11A | -0.9147 | 3.0458 | -8.1620 | 0.0000 | 0.0000 | 23.4788 |
| GMEB1 | -0.2307 | 5.1406 | -8.1611 | 0.0000 | 0.0000 | 23.4727 |
| SDHA | 0.1936 | 6.9222 | 8.1564 | 0.0000 | 0.0000 | 23.4403 |
| **PAQR5** | -1.4998 | 4.0382 | -8.1519 | 0.0000 | 0.0000 | 23.4097 |
| TPX2 | -0.5032 | 6.1071 | -8.1514 | 0.0000 | 0.0000 | 23.4059 |
| RAD51D | -0.3868 | 4.3508 | -8.1457 | 0.0000 | 0.0000 | 23.3669 |
| SLC27A2 | 0.6204 | 6.9684 | 8.1428 | 0.0000 | 0.0000 | 23.3470 |

| OTUD3 | -0.5670 | 3.8183 | -8.1390 | 0.0000 | 0.0000 | 23.3211 |
| --- | --- | --- | --- | --- | --- | --- |
| PAQR4 | -0.6628 | 5.1599 | -8.1385 | 0.0000 | 0.0000 | 23.3178 |
| **PRSS22** | -1.4300 | 1.6522 | -8.1379 | 0.0000 | 0.0000 | 23.3136 |
| HIBCH | 0.2737 | 6.1921 | 8.1346 | 0.0000 | 0.0000 | 23.2909 |
| SPARCL1 | 0.5156 | 6.6747 | 8.1328 | 0.0000 | 0.0000 | 23.2786 |
| MTMR2 | -0.4481 | 5.1150 | -8.1258 | 0.0000 | 0.0000 | 23.2303 |
| RAB7A | 0.0863 | 7.3480 | 8.1230 | 0.0000 | 0.0000 | 23.2112 |
| **ITGB6** | -1.1139 | 1.7917 | -8.1215 | 0.0000 | 0.0000 | 23.2012 |
| HINT1 | 0.1456 | 7.6095 | 8.1185 | 0.0000 | 0.0000 | 23.1802 |
| **ITPR3** | -1.1327 | 3.7724 | -8.1173 | 0.0000 | 0.0000 | 23.1722 |
| KIF3C | -0.7771 | 3.6489 | -8.1112 | 0.0000 | 0.0000 | 23.1309 |
| E2F6 | -0.2758 | 5.2524 | -8.1106 | 0.0000 | 0.0000 | 23.1261 |
| GADD45B | 0.3128 | 7.1108 | 8.1035 | 0.0000 | 0.0000 | 23.0781 |
| B2M | 0.1484 | 7.8938 | 8.1028 | 0.0000 | 0.0000 | 23.0732 |
| SIGMAR1 | 0.1706 | 7.3948 | 8.1017 | 0.0000 | 0.0000 | 23.0653 |
| **SRD5A2** | 1.4025 | 4.6382 | 8.0989 | 0.0000 | 0.0000 | 23.0465 |
| KLF12 | 0.5050 | 5.2276 | 8.0954 | 0.0000 | 0.0000 | 23.0226 |
| ST3GAL6 | 0.6356 | 5.5994 | 8.0945 | 0.0000 | 0.0000 | 23.0163 |
| **DLG3** | -1.0209 | 3.8297 | -8.0916 | 0.0000 | 0.0000 | 22.9968 |
| ABCC6 | 0.4315 | 6.6684 | 8.0873 | 0.0000 | 0.0000 | 22.9674 |
| **LARP6** | -1.1620 | 3.5842 | -8.0868 | 0.0000 | 0.0000 | 22.9637 |
| PYGL | 0.2853 | 6.9224 | 8.0836 | 0.0000 | 0.0000 | 22.9418 |
| **SMPDL3B** | -1.3050 | 3.0945 | -8.0812 | 0.0000 | 0.0000 | 22.9254 |
| CDC14B | 0.5768 | 5.4731 | 8.0804 | 0.0000 | 0.0000 | 22.9200 |
| LHPP | 0.4088 | 6.4129 | 8.0798 | 0.0000 | 0.0000 | 22.9161 |
| MPDZ | 0.6273 | 5.6375 | 8.0784 | 0.0000 | 0.0000 | 22.9060 |
| **DSG1** | 1.5340 | 3.8231 | 8.0762 | 0.0000 | 0.0000 | 22.8911 |
| TBC1D30 | -0.9860 | 3.4042 | -8.0718 | 0.0000 | 0.0000 | 22.8612 |
| **EPS8L1** | -1.2336 | 2.7320 | -8.0701 | 0.0000 | 0.0000 | 22.8497 |
| SKA1 | -0.8982 | 4.7230 | -8.0699 | 0.0000 | 0.0000 | 22.8481 |
| SULT2A1 | 0.9031 | 7.3278 | 8.0691 | 0.0000 | 0.0000 | 22.8431 |
| **CBLN1** | 1.2551 | 3.8461 | 8.0664 | 0.0000 | 0.0000 | 22.8248 |
| STX3 | -0.4075 | 5.6578 | -8.0658 | 0.0000 | 0.0000 | 22.8202 |
| NPTX1 | -0.8667 | 0.7308 | -8.0623 | 0.0000 | 0.0000 | 22.7969 |
| PID1 | 0.3985 | 6.4589 | 8.0595 | 0.0000 | 0.0000 | 22.7775 |
| PLAC1 | -0.7554 | 0.6171 | -8.0573 | 0.0000 | 0.0000 | 22.7626 |
| **DIRAS2** | -1.3661 | 1.7505 | -8.0565 | 0.0000 | 0.0000 | 22.7570 |
| **MST1R** | -1.2154 | 2.7121 | -8.0563 | 0.0000 | 0.0000 | 22.7555 |
| SYBU | 0.6575 | 5.9818 | 8.0527 | 0.0000 | 0.0000 | 22.7310 |
| IL13RA1 | 0.1813 | 7.0418 | 8.0502 | 0.0000 | 0.0000 | 22.7142 |
| **TTC39A** | -1.1593 | 3.9996 | -8.0500 | 0.0000 | 0.0000 | 22.7130 |
| CDC45 | -0.7085 | 5.0587 | -8.0491 | 0.0000 | 0.0000 | 22.7066 |
| MICU1 | 0.1762 | 6.7722 | 8.0485 | 0.0000 | 0.0000 | 22.7023 |
| NCOA4 | 0.1606 | 7.2712 | 8.0484 | 0.0000 | 0.0000 | 22.7016 |
| HADHA | 0.1292 | 7.3759 | 8.0474 | 0.0000 | 0.0000 | 22.6950 |
| **A4GNT** | -1.2230 | 1.0404 | -8.0451 | 0.0000 | 0.0000 | 22.6794 |
| SLC16A2 | 0.5463 | 6.4826 | 8.0446 | 0.0000 | 0.0000 | 22.6761 |
| GFOD2 | 0.2920 | 5.7087 | 8.0365 | 0.0000 | 0.0000 | 22.6206 |
| GLI1 | -0.9063 | 2.8227 | -8.0352 | 0.0000 | 0.0000 | 22.6121 |
| NDUFS2 | 0.1490 | 7.2502 | 8.0345 | 0.0000 | 0.0000 | 22.6076 |
| KIF5A | -0.7422 | 0.8573 | -8.0341 | 0.0000 | 0.0000 | 22.6044 |
| BFSP1 | -0.6855 | 3.4248 | -8.0295 | 0.0000 | 0.0000 | 22.5731 |
| **MUC5B** | -1.7460 | 1.6417 | -8.0273 | 0.0000 | 0.0000 | 22.5583 |

| CIT | -0.7209 | 3.8449 | -8.0226 | 0.0000 | 0.0000 | 22.5262 |
| --- | --- | --- | --- | --- | --- | --- |
| DGKZ | -0.2020 | 5.7217 | -8.0219 | 0.0000 | 0.0000 | 22.5218 |
| **TCN1** | -1.2483 | 1.1229 | -8.0219 | 0.0000 | 0.0000 | 22.5215 |
| FCGRT | 0.1871 | 7.2727 | 8.0213 | 0.0000 | 0.0000 | 22.5175 |
| GADD45G | 0.4172 | 6.9812 | 8.0162 | 0.0000 | 0.0000 | 22.4830 |
| SS18L1 | 0.3122 | 6.1116 | 8.0162 | 0.0000 | 0.0000 | 22.4827 |
| RNF34 | -0.2066 | 5.8168 | -8.0147 | 0.0000 | 0.0000 | 22.4732 |
| FBXO41 | -0.9543 | 2.5991 | -8.0139 | 0.0000 | 0.0000 | 22.4677 |
| PPP2R3A | -0.7563 | 3.3346 | -8.0062 | 0.0000 | 0.0000 | 22.4151 |
| ZCCHC6 | 0.3417 | 5.9977 | 8.0045 | 0.0000 | 0.0000 | 22.4033 |
| PLCB3 | -0.2195 | 5.8280 | -8.0034 | 0.0000 | 0.0000 | 22.3963 |
| INSR | 0.2105 | 6.6646 | 8.0019 | 0.0000 | 0.0000 | 22.3862 |
| **SLC7A1** | -1.0536 | 4.2167 | -8.0004 | 0.0000 | 0.0000 | 22.3759 |
| **GJB3** | -1.2626 | 2.0197 | -7.9991 | 0.0000 | 0.0000 | 22.3673 |
| MST1 | 0.4706 | 7.0295 | 7.9988 | 0.0000 | 0.0000 | 22.3652 |
| NKAIN1 | -0.8035 | 0.8223 | -7.9979 | 0.0000 | 0.0000 | 22.3586 |
| **NECAB2** | 1.2055 | 5.2651 | 7.9967 | 0.0000 | 0.0000 | 22.3507 |
| MCM2 | -0.4724 | 6.0114 | -7.9957 | 0.0000 | 0.0000 | 22.3437 |
| **SYNGR3** | -1.0519 | 1.9668 | -7.9954 | 0.0000 | 0.0000 | 22.3423 |
| KHK | 0.4056 | 7.1573 | 7.9898 | 0.0000 | 0.0000 | 22.3040 |
| **UPB1** | 1.0261 | 6.4983 | 7.9897 | 0.0000 | 0.0000 | 22.3030 |
| BHMT | 0.9714 | 6.7683 | 7.9817 | 0.0000 | 0.0000 | 22.2490 |
| DCLRE1C | -0.5328 | 4.0756 | -7.9799 | 0.0000 | 0.0000 | 22.2371 |
| C16orf45 | 0.6109 | 5.6615 | 7.9737 | 0.0000 | 0.0000 | 22.1953 |
| CHRNA5 | -0.8765 | 1.8048 | -7.9723 | 0.0000 | 0.0000 | 22.1856 |
| AMPD3 | -0.7429 | 3.5939 | -7.9711 | 0.0000 | 0.0000 | 22.1775 |
| NCAPG | -0.7897 | 4.9182 | -7.9703 | 0.0000 | 0.0000 | 22.1721 |
| FIS1 | 0.1787 | 7.3368 | 7.9698 | 0.0000 | 0.0000 | 22.1688 |
| PTGS1 | -0.8819 | 3.9178 | -7.9667 | 0.0000 | 0.0000 | 22.1476 |
| HADHB | 0.1561 | 7.1733 | 7.9665 | 0.0000 | 0.0000 | 22.1464 |
| **MCOLN3** | -1.3781 | 1.6558 | -7.9621 | 0.0000 | 0.0000 | 22.1165 |
| CENPM | -0.7237 | 5.4486 | -7.9595 | 0.0000 | 0.0000 | 22.0990 |
| HSD17B10 | 0.1747 | 7.4680 | 7.9588 | 0.0000 | 0.0000 | 22.0941 |
| HHLA2 | -0.9449 | 0.8073 | -7.9586 | 0.0000 | 0.0000 | 22.0929 |
| KNTC1 | -0.6436 | 4.5108 | -7.9581 | 0.0000 | 0.0000 | 22.0897 |
| **FETUB** | 1.1024 | 6.4530 | 7.9562 | 0.0000 | 0.0000 | 22.0766 |
| BARD1 | -0.6795 | 3.9755 | -7.9553 | 0.0000 | 0.0000 | 22.0709 |
| PPID | 0.1741 | 6.6523 | 7.9539 | 0.0000 | 0.0000 | 22.0611 |
| HCN4 | -0.7611 | 0.5558 | -7.9510 | 0.0000 | 0.0000 | 22.0417 |
| TEX15 | -0.7214 | 0.3638 | -7.9458 | 0.0000 | 0.0000 | 22.0067 |
| CHN2 | 0.5494 | 5.5340 | 7.9447 | 0.0000 | 0.0000 | 21.9994 |
| ARHGAP11A | -0.7016 | 4.6222 | -7.9443 | 0.0000 | 0.0000 | 21.9963 |
| ACYP1 | -0.3587 | 5.0049 | -7.9438 | 0.0000 | 0.0000 | 21.9928 |
| TMEM165 | -0.2868 | 5.7999 | -7.9416 | 0.0000 | 0.0000 | 21.9783 |
| **MYRIP** | 1.2186 | 5.1824 | 7.9376 | 0.0000 | 0.0000 | 21.9512 |
| **CYP4F2** | 1.0320 | 6.4871 | 7.9376 | 0.0000 | 0.0000 | 21.9511 |
| KDR | 0.5537 | 5.7084 | 7.9344 | 0.0000 | 0.0000 | 21.9300 |
| GLT8D1 | 0.1882 | 6.6447 | 7.9269 | 0.0000 | 0.0000 | 21.8794 |
| CCDC28A | 0.1959 | 6.7035 | 7.9193 | 0.0000 | 0.0000 | 21.8280 |
| **SLC6A13** | 1.0897 | 4.4808 | 7.9188 | 0.0000 | 0.0000 | 21.8248 |
| KIAA0141 | 0.1685 | 6.4883 | 7.9162 | 0.0000 | 0.0000 | 21.8075 |
| **FXYD3** | -1.4835 | 3.1367 | -7.9135 | 0.0000 | 0.0000 | 21.7891 |
| MYB | -0.8558 | 1.8836 | -7.9075 | 0.0000 | 0.0000 | 21.7486 |

| SPCS1 | 0.1682 | 6.8909 | 7.9043 | 0.0000 | 0.0000 | 21.7269 |
| --- | --- | --- | --- | --- | --- | --- |
| MMRN2 | 0.3956 | 5.8090 | 7.9024 | 0.0000 | 0.0000 | 21.7147 |
| SCCPDH | 0.2449 | 7.3502 | 7.9011 | 0.0000 | 0.0000 | 21.7058 |
| PEMT | 0.2985 | 6.9401 | 7.8891 | 0.0000 | 0.0000 | 21.6254 |
| PKMYT1 | -0.7279 | 4.5407 | -7.8865 | 0.0000 | 0.0000 | 21.6079 |
| UQCRC1 | 0.1560 | 7.4055 | 7.8837 | 0.0000 | 0.0000 | 21.5889 |
| KIF20A | -0.7788 | 5.1379 | -7.8783 | 0.0000 | 0.0000 | 21.5528 |
| ST6GALNAC4 | -0.5309 | 5.4408 | -7.8750 | 0.0000 | 0.0000 | 21.5304 |
| TTPA | 0.7896 | 6.4805 | 7.8738 | 0.0000 | 0.0000 | 21.5228 |
| **MUC6** | -1.4940 | 1.7193 | -7.8735 | 0.0000 | 0.0000 | 21.5208 |
| MTMR10 | 0.2556 | 5.8466 | 7.8717 | 0.0000 | 0.0000 | 21.5085 |
| **HTR3A** | -1.0627 | 0.9792 | -7.8685 | 0.0000 | 0.0000 | 21.4870 |
| PPP2R5A | 0.1685 | 6.9817 | 7.8680 | 0.0000 | 0.0000 | 21.4836 |
| **TTYH1** | -1.1490 | 1.9390 | -7.8643 | 0.0000 | 0.0000 | 21.4589 |
| KCTD17 | -0.7594 | 5.4186 | -7.8583 | 0.0000 | 0.0000 | 21.4184 |
| **SSTR5** | -1.6556 | 1.5639 | -7.8506 | 0.0000 | 0.0000 | 21.3671 |
| RAB38 | -0.9801 | 3.2234 | -7.8486 | 0.0000 | 0.0000 | 21.3540 |
| C11orf49 | -0.5411 | 4.8250 | -7.8483 | 0.0000 | 0.0000 | 21.3521 |
| GATM | 0.3520 | 7.4072 | 7.8458 | 0.0000 | 0.0000 | 21.3351 |
| C1orf159 | -0.3252 | 4.8246 | -7.8457 | 0.0000 | 0.0000 | 21.3347 |
| BAK1 | -0.3216 | 6.1803 | -7.8435 | 0.0000 | 0.0000 | 21.3200 |
| EAPP | 0.1439 | 6.6772 | 7.8391 | 0.0000 | 0.0000 | 21.2901 |
| APOC3 | 0.4510 | 8.1567 | 7.8378 | 0.0000 | 0.0000 | 21.2817 |
| **CFHR2** | 1.0276 | 5.9631 | 7.8295 | 0.0000 | 0.0000 | 21.2262 |
| SORBS2 | 0.5358 | 5.9023 | 7.8253 | 0.0000 | 0.0000 | 21.1979 |
| **TNFAIP6** | -1.1952 | 2.3202 | -7.8240 | 0.0000 | 0.0000 | 21.1896 |
| **LPA** | 1.1044 | 5.1343 | 7.8233 | 0.0000 | 0.0000 | 21.1845 |
| DBF4 | -0.4081 | 4.8354 | -7.8205 | 0.0000 | 0.0000 | 21.1663 |
| SOX11 | -0.6601 | 0.6147 | -7.8174 | 0.0000 | 0.0000 | 21.1457 |
| ZBED5 | -0.2854 | 5.1541 | -7.8160 | 0.0000 | 0.0000 | 21.1360 |
| DEXI | 0.2969 | 5.9573 | 7.8128 | 0.0000 | 0.0000 | 21.1145 |
| VAMP7 | 0.1954 | 6.7744 | 7.8126 | 0.0000 | 0.0000 | 21.1137 |
| HPCA | -0.6582 | 0.8170 | -7.8091 | 0.0000 | 0.0000 | 21.0902 |
| GPX3 | 0.2923 | 7.5809 | 7.8084 | 0.0000 | 0.0000 | 21.0854 |
| ECI1 | 0.2013 | 7.0877 | 7.8051 | 0.0000 | 0.0000 | 21.0636 |
| PSME1 | 0.1342 | 7.4845 | 7.8035 | 0.0000 | 0.0000 | 21.0530 |
| **APLP1** | -1.1920 | 3.2762 | -7.8001 | 0.0000 | 0.0000 | 21.0299 |
| TRPM2 | -0.7454 | 3.8198 | -7.7967 | 0.0000 | 0.0000 | 21.0078 |
| POLA1 | -0.4044 | 4.9554 | -7.7965 | 0.0000 | 0.0000 | 21.0063 |
| DEPDC1 | -0.9286 | 4.0001 | -7.7962 | 0.0000 | 0.0000 | 21.0045 |
| SLC38A1 | -0.9467 | 5.2341 | -7.7913 | 0.0000 | 0.0000 | 20.9719 |
| **LIF** | -1.2150 | 3.9555 | -7.7913 | 0.0000 | 0.0000 | 20.9716 |
| RPGRIP1L | -0.4708 | 2.7374 | -7.7873 | 0.0000 | 0.0000 | 20.9449 |
| RFWD3 | -0.2984 | 5.4120 | -7.7861 | 0.0000 | 0.0000 | 20.9368 |
| GFOD1 | 0.4678 | 4.6256 | 7.7839 | 0.0000 | 0.0000 | 20.9226 |
| SLC25A28 | 0.1765 | 6.7932 | 7.7672 | 0.0000 | 0.0000 | 20.8113 |
| **CXCL3** | -1.0847 | 2.5180 | -7.7625 | 0.0000 | 0.0000 | 20.7800 |
| CROCC | -0.3627 | 4.9310 | -7.7584 | 0.0000 | 0.0000 | 20.7533 |
| **FXYD2** | -1.8047 | 3.0879 | -7.7584 | 0.0000 | 0.0000 | 20.7529 |
| AGRN | -0.2575 | 6.6013 | -7.7577 | 0.0000 | 0.0000 | 20.7488 |
| CACNA1G | -0.4695 | 0.4025 | -7.7561 | 0.0000 | 0.0000 | 20.7376 |
| MRPS18B | 0.1566 | 7.2265 | 7.7558 | 0.0000 | 0.0000 | 20.7356 |
| KDELR2 | 0.1149 | 7.4086 | 7.7530 | 0.0000 | 0.0000 | 20.7175 |

| ABCB4 | 0.8458 | 6.2881 | 7.7528 | 0.0000 | 0.0000 | 20.7158 |
| --- | --- | --- | --- | --- | --- | --- |
| PRMT2 | -0.2609 | 5.7076 | -7.7526 | 0.0000 | 0.0000 | 20.7149 |
| IKBKE | -0.7828 | 4.8662 | -7.7516 | 0.0000 | 0.0000 | 20.7079 |
| **APOF** | 1.0956 | 6.3608 | 7.7507 | 0.0000 | 0.0000 | 20.7019 |
| SLC38A4 | 0.6825 | 7.0185 | 7.7496 | 0.0000 | 0.0000 | 20.6946 |
| POLD3 | -0.3158 | 5.2660 | -7.7495 | 0.0000 | 0.0000 | 20.6945 |
| HSD17B6 | 0.6649 | 7.3278 | 7.7449 | 0.0000 | 0.0000 | 20.6639 |
| HJURP | -0.7830 | 4.9650 | -7.7442 | 0.0000 | 0.0000 | 20.6589 |
| MLLT3 | -0.9195 | 3.2047 | -7.7428 | 0.0000 | 0.0000 | 20.6496 |
| SPRYD7 | 0.2702 | 5.9885 | 7.7416 | 0.0000 | 0.0000 | 20.6419 |
| 43893.0000 | -0.7750 | 3.6015 | -7.7412 | 0.0000 | 0.0000 | 20.6392 |
| RBKS | 0.3066 | 6.1044 | 7.7399 | 0.0000 | 0.0000 | 20.6305 |
| ASRGL1 | -0.7767 | 4.5679 | -7.7381 | 0.0000 | 0.0000 | 20.6189 |
| CLIP2 | -0.7010 | 5.1229 | -7.7370 | 0.0000 | 0.0000 | 20.6117 |
| CPSF6 | -0.1609 | 6.1259 | -7.7303 | 0.0000 | 0.0000 | 20.5672 |
| LRP1 | 0.2168 | 6.9214 | 7.7240 | 0.0000 | 0.0000 | 20.5254 |
| DNAJB9 | 0.2202 | 6.9468 | 7.7237 | 0.0000 | 0.0000 | 20.5231 |
| SH3BP1 | -0.7119 | 4.5345 | -7.7231 | 0.0000 | 0.0000 | 20.5197 |
| **FAM155B** | -1.3618 | 2.6983 | -7.7193 | 0.0000 | 0.0000 | 20.4943 |
| PCLO | -0.9005 | 0.8398 | -7.7136 | 0.0000 | 0.0000 | 20.4569 |
| DNAH7 | -0.4365 | 0.5542 | -7.7122 | 0.0000 | 0.0000 | 20.4476 |
| NDUFB6 | 0.1534 | 7.0683 | 7.7095 | 0.0000 | 0.0000 | 20.4296 |
| **CPS1** | 1.0903 | 6.8897 | 7.7070 | 0.0000 | 0.0000 | 20.4128 |
| EEF2 | 0.1099 | 7.8272 | 7.7051 | 0.0000 | 0.0000 | 20.4007 |
| **ITGB8** | -1.1647 | 1.4395 | -7.7037 | 0.0000 | 0.0000 | 20.3912 |
| MRPS22 | 0.1453 | 6.2241 | 7.7035 | 0.0000 | 0.0000 | 20.3903 |
| RGS2 | -0.5953 | 5.8935 | -7.7032 | 0.0000 | 0.0000 | 20.3878 |
| LRRC8B | -0.7117 | 4.3851 | -7.7007 | 0.0000 | 0.0000 | 20.3716 |
| CDK1 | -0.6321 | 5.5180 | -7.6978 | 0.0000 | 0.0000 | 20.3522 |
| PCYT2 | 0.2384 | 6.9417 | 7.6932 | 0.0000 | 0.0000 | 20.3223 |
| DECR2 | 0.2538 | 6.8742 | 7.6909 | 0.0000 | 0.0000 | 20.3067 |
| IGSF3 | -0.9884 | 4.6685 | -7.6882 | 0.0000 | 0.0000 | 20.2892 |
| **CDCP1** | -1.1251 | 2.9016 | -7.6856 | 0.0000 | 0.0000 | 20.2721 |
| TSPYL1 | 0.1852 | 6.7204 | 7.6840 | 0.0000 | 0.0000 | 20.2616 |
| CYP2J2 | 0.5405 | 6.7227 | 7.6824 | 0.0000 | 0.0000 | 20.2507 |
| TRAIP | -0.6486 | 4.5753 | -7.6751 | 0.0000 | 0.0000 | 20.2029 |
| CHAD | 0.6820 | 6.1588 | 7.6720 | 0.0000 | 0.0000 | 20.1823 |
| TUBB3 | -0.8593 | 1.2152 | -7.6672 | 0.0000 | 0.0000 | 20.1506 |
| OSBP2 | -0.8249 | 3.8174 | -7.6642 | 0.0000 | 0.0000 | 20.1310 |
| SLC35F2 | -0.9006 | 3.1805 | -7.6587 | 0.0000 | 0.0000 | 20.0948 |
| **SLC4A3** | -1.2507 | 2.2420 | -7.6580 | 0.0000 | 0.0000 | 20.0902 |
| SLC6A1 | 0.7290 | 6.5798 | 7.6576 | 0.0000 | 0.0000 | 20.0880 |
| SORBS3 | 0.2099 | 6.5989 | 7.6561 | 0.0000 | 0.0000 | 20.0781 |
| CLU | 0.2087 | 7.8815 | 7.6535 | 0.0000 | 0.0000 | 20.0612 |
| SERINC1 | 0.1550 | 7.1927 | 7.6501 | 0.0000 | 0.0000 | 20.0386 |
| POLD1 | -0.2828 | 5.9124 | -7.6500 | 0.0000 | 0.0000 | 20.0380 |
| TRAF5 | -0.5989 | 4.2059 | -7.6482 | 0.0000 | 0.0000 | 20.0259 |
| ABCG2 | 0.8004 | 5.5338 | 7.6433 | 0.0000 | 0.0000 | 19.9943 |
| GCLC | 0.2804 | 6.4988 | 7.6420 | 0.0000 | 0.0000 | 19.9852 |
| DDR1 | -0.8609 | 5.2302 | -7.6418 | 0.0000 | 0.0000 | 19.9842 |
| **FHOD3** | -1.2313 | 2.0821 | -7.6391 | 0.0000 | 0.0000 | 19.9661 |
| OSBPL10 | -0.8312 | 2.9148 | -7.6386 | 0.0000 | 0.0000 | 19.9631 |
| **CXCL5** | -1.6101 | 2.2789 | -7.6366 | 0.0000 | 0.0000 | 19.9500 |

| **CHST4** | -1.5762 | 1.9079 | -7.6350 | 0.0000 | 0.0000 | 19.9397 |
| --- | --- | --- | --- | --- | --- | --- |
| TSR2 | 0.1246 | 6.9788 | 7.6350 | 0.0000 | 0.0000 | 19.9395 |
| STX6 | -0.2445 | 5.6423 | -7.6324 | 0.0000 | 0.0000 | 19.9225 |
| PRC1 | -0.5752 | 5.4776 | -7.6315 | 0.0000 | 0.0000 | 19.9166 |
| ATAD5 | -0.6273 | 3.6121 | -7.6294 | 0.0000 | 0.0000 | 19.9026 |
| GPM6B | -0.6569 | 1.9501 | -7.6193 | 0.0000 | 0.0000 | 19.8366 |
| GPT | 0.5185 | 6.8740 | 7.6193 | 0.0000 | 0.0000 | 19.8365 |
| LAP3 | 0.2288 | 6.9708 | 7.6186 | 0.0000 | 0.0000 | 19.8322 |
| PRDX6 | 0.1545 | 7.6763 | 7.6184 | 0.0000 | 0.0000 | 19.8307 |
| CCNB2 | -0.6885 | 5.4502 | -7.6178 | 0.0000 | 0.0000 | 19.8272 |
| METTL9 | -0.2565 | 5.7327 | -7.6088 | 0.0000 | 0.0000 | 19.7678 |
| FBXL5 | 0.1929 | 6.7016 | 7.6066 | 0.0000 | 0.0000 | 19.7537 |
| PAX9 | -0.5495 | 0.4380 | -7.6051 | 0.0000 | 0.0000 | 19.7438 |
| NDUFA4 | 0.1546 | 7.2948 | 7.6025 | 0.0000 | 0.0000 | 19.7266 |
| NDRG2 | 0.2555 | 6.8279 | 7.6011 | 0.0000 | 0.0000 | 19.7179 |
| SMARCD3 | -0.6916 | 4.6090 | -7.6009 | 0.0000 | 0.0000 | 19.7161 |
| MCM3 | -0.2053 | 6.7566 | -7.5994 | 0.0000 | 0.0000 | 19.7067 |
| RACGAP1 | -0.4373 | 5.5980 | -7.5989 | 0.0000 | 0.0000 | 19.7033 |
| CD3EAP | -0.3906 | 4.6778 | -7.5965 | 0.0000 | 0.0000 | 19.6877 |
| UAP1 | 0.2016 | 6.9576 | 7.5956 | 0.0000 | 0.0000 | 19.6819 |
| ITGB4 | -0.6322 | 5.0619 | -7.5924 | 0.0000 | 0.0000 | 19.6609 |
| PDZD7 | -0.6356 | 1.5311 | -7.5921 | 0.0000 | 0.0000 | 19.6587 |
| CPEB3 | 0.6009 | 4.6893 | 7.5868 | 0.0000 | 0.0000 | 19.6245 |
| ALDH4A1 | 0.2548 | 7.2866 | 7.5854 | 0.0000 | 0.0000 | 19.6151 |
| NDC80 | -0.6417 | 5.0953 | -7.5839 | 0.0000 | 0.0000 | 19.6054 |
| CCNB1 | -0.4722 | 6.1408 | -7.5826 | 0.0000 | 0.0000 | 19.5966 |
| CBX2 | -0.7808 | 4.1071 | -7.5779 | 0.0000 | 0.0000 | 19.5663 |
| NIT2 | 0.1980 | 6.3731 | 7.5766 | 0.0000 | 0.0000 | 19.5575 |
| SLC11A1 | -0.7281 | 3.1238 | -7.5765 | 0.0000 | 0.0000 | 19.5572 |
| ATP5J | 0.1608 | 7.2770 | 7.5754 | 0.0000 | 0.0000 | 19.5497 |
| MYL12B | 0.1191 | 7.6242 | 7.5699 | 0.0000 | 0.0000 | 19.5139 |
| **PITX1** | -1.7165 | 3.1428 | -7.5617 | 0.0000 | 0.0000 | 19.4608 |
| PROS1 | 0.2754 | 7.0546 | 7.5612 | 0.0000 | 0.0000 | 19.4577 |
| CBR1 | 0.2453 | 7.3671 | 7.5602 | 0.0000 | 0.0000 | 19.4512 |
| FUCA1 | 0.1753 | 7.0774 | 7.5587 | 0.0000 | 0.0000 | 19.4414 |
| FAM118A | -0.4212 | 5.2193 | -7.5585 | 0.0000 | 0.0000 | 19.4398 |
| STAT5B | 0.1661 | 6.3685 | 7.5548 | 0.0000 | 0.0000 | 19.4160 |
| CEBPB | 0.2001 | 7.2361 | 7.5463 | 0.0000 | 0.0000 | 19.3607 |
| SLC38A3 | 0.5233 | 7.3824 | 7.5440 | 0.0000 | 0.0000 | 19.3455 |
| IL6ST | 0.1884 | 6.8440 | 7.5399 | 0.0000 | 0.0000 | 19.3190 |
| TDG | -0.2712 | 5.4101 | -7.5363 | 0.0000 | 0.0000 | 19.2958 |
| F10 | 0.3638 | 7.3256 | 7.5354 | 0.0000 | 0.0000 | 19.2901 |
| EZH2 | -0.4593 | 5.4261 | -7.5354 | 0.0000 | 0.0000 | 19.2900 |
| SEL1L3 | -0.8040 | 5.3122 | -7.5353 | 0.0000 | 0.0000 | 19.2892 |
| OSBPL7 | -0.6928 | 3.8012 | -7.5352 | 0.0000 | 0.0000 | 19.2882 |
| ATP6V0E2 | 0.3408 | 6.8573 | 7.5336 | 0.0000 | 0.0000 | 19.2780 |
| IL17B | -0.7411 | 1.1655 | -7.5314 | 0.0000 | 0.0000 | 19.2636 |
| PDLIM7 | -0.3321 | 5.9166 | -7.5312 | 0.0000 | 0.0000 | 19.2624 |
| MAT2B | 0.1997 | 6.4936 | 7.5304 | 0.0000 | 0.0000 | 19.2573 |
| OXTR | -0.8864 | 2.5601 | -7.5290 | 0.0000 | 0.0000 | 19.2481 |
| **ZNF711** | -1.1354 | 2.6185 | -7.5266 | 0.0000 | 0.0000 | 19.2326 |
| FFAR2 | -0.9327 | 2.1710 | -7.5265 | 0.0000 | 0.0000 | 19.2321 |
| **HOXB9** | -1.0042 | 0.9421 | -7.5221 | 0.0000 | 0.0000 | 19.2038 |

| EBAG9 | 0.2145 | 6.3575 | 7.5170 | 0.0000 | 0.0000 | 19.1702 |
| --- | --- | --- | --- | --- | --- | --- |
| KREMEN2 | -0.9201 | 1.9159 | -7.5130 | 0.0000 | 0.0000 | 19.1448 |
| LPIN2 | 0.2719 | 6.8426 | 7.5130 | 0.0000 | 0.0000 | 19.1447 |
| GALT | 0.2194 | 6.7223 | 7.5122 | 0.0000 | 0.0000 | 19.1396 |
| ABHD6 | 0.3800 | 6.3100 | 7.5117 | 0.0000 | 0.0000 | 19.1363 |
| MXI1 | 0.2373 | 6.3358 | 7.5064 | 0.0000 | 0.0000 | 19.1018 |
| CALCR | -0.7533 | 0.7632 | -7.5055 | 0.0000 | 0.0000 | 19.0962 |
| CALB2 | -0.9542 | 1.1936 | -7.5046 | 0.0000 | 0.0000 | 19.0899 |
| NKX3-2 | -0.9391 | 1.0197 | -7.5033 | 0.0000 | 0.0000 | 19.0815 |
| EXO1 | -0.7987 | 4.4325 | -7.5014 | 0.0000 | 0.0000 | 19.0697 |
| **SLC22A1** | 1.0727 | 6.4081 | 7.4987 | 0.0000 | 0.0000 | 19.0521 |
| DHODH | 0.4124 | 6.0960 | 7.4969 | 0.0000 | 0.0000 | 19.0405 |
| **OLFM4** | -1.1790 | 1.0308 | -7.4924 | 0.0000 | 0.0000 | 19.0114 |
| **GRAMD1B** | -1.1780 | 3.5030 | -7.4917 | 0.0000 | 0.0000 | 19.0069 |
| DUSP10 | 0.3020 | 6.4802 | 7.4831 | 0.0000 | 0.0000 | 18.9511 |
| TMEM176B | 0.2580 | 7.8551 | 7.4830 | 0.0000 | 0.0000 | 18.9504 |
| **CFHR5** | 1.1391 | 6.4596 | 7.4825 | 0.0000 | 0.0000 | 18.9473 |
| DPP4 | 0.4927 | 6.5528 | 7.4789 | 0.0000 | 0.0000 | 18.9238 |
| CYP2D6 | 0.7062 | 7.0539 | 7.4786 | 0.0000 | 0.0000 | 18.9220 |
| IFIT1 | 0.5296 | 6.0713 | 7.4780 | 0.0000 | 0.0000 | 18.9184 |
| SFI1 | -0.4755 | 4.7312 | -7.4757 | 0.0000 | 0.0000 | 18.9035 |
| CDT1 | -0.6395 | 5.4800 | -7.4756 | 0.0000 | 0.0000 | 18.9029 |
| MELK | -0.7105 | 5.0551 | -7.4750 | 0.0000 | 0.0000 | 18.8987 |
| PCYOX1 | 0.1986 | 6.8723 | 7.4745 | 0.0000 | 0.0000 | 18.8956 |
| EIF5 | 0.1512 | 6.8431 | 7.4745 | 0.0000 | 0.0000 | 18.8953 |
| DBNDD2 | -0.7149 | 3.1652 | -7.4713 | 0.0000 | 0.0000 | 18.8748 |
| CTH | 0.6437 | 6.4781 | 7.4651 | 0.0000 | 0.0000 | 18.8352 |
| CALD1 | 0.1655 | 6.9147 | 7.4614 | 0.0000 | 0.0000 | 18.8114 |
| SDCBP | 0.1891 | 6.9869 | 7.4612 | 0.0000 | 0.0000 | 18.8095 |
| SERPINF1 | 0.2669 | 7.6487 | 7.4605 | 0.0000 | 0.0000 | 18.8050 |
| CLDN15 | 0.5144 | 6.4185 | 7.4575 | 0.0000 | 0.0000 | 18.7862 |
| SLC9A1 | -0.3823 | 4.9947 | -7.4544 | 0.0000 | 0.0000 | 18.7661 |
| RUNDC3B | 0.8975 | 4.5916 | 7.4519 | 0.0000 | 0.0000 | 18.7497 |
| DLC1 | 0.4031 | 5.5085 | 7.4484 | 0.0000 | 0.0000 | 18.7275 |
| STARD5 | 0.7053 | 4.0855 | 7.4484 | 0.0000 | 0.0000 | 18.7270 |
| DNAJC10 | -0.2919 | 5.3805 | -7.4462 | 0.0000 | 0.0000 | 18.7133 |
| SDC1 | 0.1877 | 7.6579 | 7.4444 | 0.0000 | 0.0000 | 18.7018 |
| UBE2S | -0.3693 | 5.9731 | -7.4444 | 0.0000 | 0.0000 | 18.7016 |
| SLC9A3R2 | 0.2373 | 7.2054 | 7.4433 | 0.0000 | 0.0000 | 18.6944 |
| CYP2C9 | 0.7637 | 7.0566 | 7.4407 | 0.0000 | 0.0000 | 18.6777 |
| VAMP5 | 0.1979 | 7.2943 | 7.4400 | 0.0000 | 0.0000 | 18.6734 |
| ALOXE3 | -0.5227 | 0.3807 | -7.4397 | 0.0000 | 0.0000 | 18.6713 |
| CYP2B6 | 0.8898 | 6.3238 | 7.4391 | 0.0000 | 0.0000 | 18.6677 |
| IRS1 | 0.3502 | 6.1767 | 7.4391 | 0.0000 | 0.0000 | 18.6675 |
| **SPIB** | -1.1401 | 2.0085 | -7.4387 | 0.0000 | 0.0000 | 18.6646 |
| SERPINF2 | 0.3864 | 7.7553 | 7.4297 | 0.0000 | 0.0000 | 18.6072 |
| **ZG16** | 1.3340 | 4.7874 | 7.4278 | 0.0000 | 0.0000 | 18.5948 |
| LCAT | 0.5468 | 6.4236 | 7.4245 | 0.0000 | 0.0000 | 18.5735 |
| SDHC | 0.1789 | 6.4772 | 7.4211 | 0.0000 | 0.0000 | 18.5520 |
| PAPSS2 | 0.3336 | 6.6243 | 7.4206 | 0.0000 | 0.0000 | 18.5486 |
| MDH2 | 0.1530 | 7.3766 | 7.4196 | 0.0000 | 0.0000 | 18.5425 |
| FBP1 | 0.4251 | 7.3287 | 7.4192 | 0.0000 | 0.0000 | 18.5394 |
| RORA | 0.5182 | 5.0842 | 7.4152 | 0.0000 | 0.0000 | 18.5141 |

| APOLD1 | 0.4712 | 5.5959 | 7.4151 | 0.0000 | 0.0000 | 18.5133 |
| --- | --- | --- | --- | --- | --- | --- |
| **TGFA** | -1.0802 | 3.6901 | -7.4017 | 0.0000 | 0.0000 | 18.4272 |
| **NPPB** | -1.0863 | 1.1062 | -7.3999 | 0.0000 | 0.0000 | 18.4157 |
| ETFB | 0.2334 | 7.1790 | 7.3971 | 0.0000 | 0.0000 | 18.3976 |
| ELF4 | -0.6647 | 4.5077 | -7.3927 | 0.0000 | 0.0000 | 18.3700 |
| SLC10A3 | -0.2520 | 6.0789 | -7.3908 | 0.0000 | 0.0000 | 18.3575 |
| VASP | -0.1551 | 6.7310 | -7.3894 | 0.0000 | 0.0000 | 18.3488 |
| PSRC1 | -0.5635 | 4.9978 | -7.3881 | 0.0000 | 0.0000 | 18.3403 |
| TCF3 | -0.2231 | 6.1030 | -7.3867 | 0.0000 | 0.0000 | 18.3316 |
| CDIPT | 0.1516 | 7.0472 | 7.3850 | 0.0000 | 0.0000 | 18.3203 |
| **GAST** | -1.3775 | 1.1159 | -7.3844 | 0.0000 | 0.0000 | 18.3166 |
| CLCN2 | -0.3538 | 5.1124 | -7.3809 | 0.0000 | 0.0000 | 18.2940 |
| HELLS | -0.7364 | 4.0189 | -7.3804 | 0.0000 | 0.0000 | 18.2908 |
| TIE1 | 0.4286 | 5.6506 | 7.3801 | 0.0000 | 0.0000 | 18.2890 |
| C1orf115 | 0.2231 | 7.1226 | 7.3795 | 0.0000 | 0.0000 | 18.2853 |
| PMVK | 0.1646 | 7.3428 | 7.3792 | 0.0000 | 0.0000 | 18.2833 |
| TLL2 | -0.6139 | 1.0477 | -7.3782 | 0.0000 | 0.0000 | 18.2769 |
| TMEM140 | 0.2125 | 6.8750 | 7.3767 | 0.0000 | 0.0000 | 18.2671 |
| GPX4 | 0.1400 | 7.6357 | 7.3756 | 0.0000 | 0.0000 | 18.2603 |
| B9D2 | -0.3456 | 5.3707 | -7.3752 | 0.0000 | 0.0000 | 18.2578 |
| C5orf30 | -0.9652 | 3.9664 | -7.3737 | 0.0000 | 0.0000 | 18.2482 |
| SIK2 | 0.2629 | 6.4265 | 7.3709 | 0.0000 | 0.0000 | 18.2307 |
| **KCNJ16** | -1.2108 | 1.1995 | -7.3707 | 0.0000 | 0.0000 | 18.2289 |
| BCKDHB | 0.3734 | 6.1272 | 7.3680 | 0.0000 | 0.0000 | 18.2115 |
| CRADD | 0.2738 | 5.9047 | 7.3674 | 0.0000 | 0.0000 | 18.2082 |
| LARP4B | -0.1808 | 5.7563 | -7.3674 | 0.0000 | 0.0000 | 18.2081 |
| MRPL46 | 0.2882 | 5.7178 | 7.3668 | 0.0000 | 0.0000 | 18.2040 |
| RAMP1 | 0.5065 | 7.1241 | 7.3656 | 0.0000 | 0.0000 | 18.1965 |
| SCMH1 | -0.2588 | 5.7808 | -7.3633 | 0.0000 | 0.0000 | 18.1815 |
| G6PC | 0.7093 | 7.2305 | 7.3599 | 0.0000 | 0.0000 | 18.1604 |
| **LEFTY1** | -1.1916 | 2.7545 | -7.3596 | 0.0000 | 0.0000 | 18.1580 |
| SAT1 | 0.1719 | 7.4703 | 7.3588 | 0.0000 | 0.0000 | 18.1528 |
| TRIM46 | -0.7162 | 1.9329 | -7.3578 | 0.0000 | 0.0000 | 18.1464 |
| ZBTB16 | 0.9960 | 4.3348 | 7.3548 | 0.0000 | 0.0000 | 18.1276 |
| TEKT2 | -0.7252 | 1.1218 | -7.3527 | 0.0000 | 0.0000 | 18.1144 |
| HDAC6 | 0.2506 | 6.2822 | 7.3503 | 0.0000 | 0.0000 | 18.0991 |
| BARX2 | -0.7683 | 0.7576 | -7.3490 | 0.0000 | 0.0000 | 18.0906 |
| MPZL1 | -0.2031 | 6.4199 | -7.3483 | 0.0000 | 0.0000 | 18.0861 |
| NAGLU | 0.1790 | 6.9151 | 7.3479 | 0.0000 | 0.0000 | 18.0837 |
| UBC | 0.1073 | 7.5122 | 7.3460 | 0.0000 | 0.0000 | 18.0715 |
| APEH | 0.1418 | 6.9487 | 7.3425 | 0.0000 | 0.0000 | 18.0491 |
| PECR | 0.2859 | 6.9482 | 7.3403 | 0.0000 | 0.0000 | 18.0353 |
| RARRES2 | 0.2772 | 7.7802 | 7.3400 | 0.0000 | 0.0000 | 18.0334 |
| ITGA3 | -0.7153 | 4.5692 | -7.3385 | 0.0000 | 0.0000 | 18.0234 |
| ST13 | 0.1114 | 7.2579 | 7.3366 | 0.0000 | 0.0000 | 18.0114 |
| ZNF532 | -0.6301 | 4.2362 | -7.3358 | 0.0000 | 0.0000 | 18.0065 |
| PLSCR2 | -0.5434 | 0.6463 | -7.3296 | 0.0000 | 0.0000 | 17.9671 |
| CHST10 | -0.8773 | 3.7344 | -7.3293 | 0.0000 | 0.0000 | 17.9652 |
| BIRC5 | -0.6234 | 5.8084 | -7.3270 | 0.0000 | 0.0000 | 17.9509 |
| TMEM158 | -0.9293 | 3.2669 | -7.3260 | 0.0000 | 0.0000 | 17.9442 |
| CYTH1 | 0.1592 | 6.2966 | 7.3239 | 0.0000 | 0.0000 | 17.9307 |
| ST6GALNAC5 | -0.8966 | 1.1960 | -7.3224 | 0.0000 | 0.0000 | 17.9216 |
| **NEB** | -1.1611 | 3.0979 | -7.3222 | 0.0000 | 0.0000 | 17.9202 |

| **OVOL2** | -1.1701 | 1.4008 | -7.3219 | 0.0000 | 0.0000 | 17.9180 |
| --- | --- | --- | --- | --- | --- | --- |
| DPF1 | -0.5413 | 0.9525 | -7.3216 | 0.0000 | 0.0000 | 17.9161 |
| RFC4 | -0.3078 | 5.9868 | -7.3206 | 0.0000 | 0.0000 | 17.9097 |
| SDHB | 0.1720 | 7.1428 | 7.3183 | 0.0000 | 0.0000 | 17.8955 |
| FEN1 | -0.2240 | 6.5124 | -7.3170 | 0.0000 | 0.0000 | 17.8868 |
| H1F0 | 0.1394 | 7.5552 | 7.3145 | 0.0000 | 0.0000 | 17.8712 |
| KRT15 | -0.8693 | 0.7671 | -7.3131 | 0.0000 | 0.0000 | 17.8624 |
| CYP4F3 | 0.6454 | 6.8152 | 7.3128 | 0.0000 | 0.0000 | 17.8605 |
| SSTR3 | -0.8436 | 0.8648 | -7.3107 | 0.0000 | 0.0000 | 17.8469 |
| PMAIP1 | -0.9727 | 2.9322 | -7.3099 | 0.0000 | 0.0000 | 17.8417 |
| **PAPPA2** | 1.3826 | 2.8451 | 7.3097 | 0.0000 | 0.0000 | 17.8409 |
| TTK | -0.8410 | 4.3036 | -7.3061 | 0.0000 | 0.0000 | 17.8176 |
| TIPIN | -0.3192 | 4.9887 | -7.3036 | 0.0000 | 0.0000 | 17.8016 |
| AUH | 0.2278 | 6.2674 | 7.3006 | 0.0000 | 0.0000 | 17.7831 |
| LEPR | 0.8204 | 6.0483 | 7.2925 | 0.0000 | 0.0000 | 17.7313 |
| **LAMC2** | -1.2594 | 2.6414 | -7.2887 | 0.0000 | 0.0000 | 17.7074 |
| MYO3A | -0.7446 | 0.3687 | -7.2876 | 0.0000 | 0.0000 | 17.7004 |
| SLC7A7 | -0.5776 | 4.9419 | -7.2826 | 0.0000 | 0.0000 | 17.6685 |
| ABCG5 | 0.8332 | 6.2324 | 7.2817 | 0.0000 | 0.0000 | 17.6631 |
| SORL1 | 0.5360 | 5.7317 | 7.2771 | 0.0000 | 0.0000 | 17.6338 |
| **MOGAT2** | 1.4048 | 4.9813 | 7.2731 | 0.0000 | 0.0000 | 17.6085 |
| IQGAP2 | 0.2829 | 6.7178 | 7.2701 | 0.0000 | 0.0000 | 17.5900 |
| FZD2 | -0.8725 | 3.3587 | -7.2701 | 0.0000 | 0.0000 | 17.5899 |
| **MMP1** | -1.1966 | 3.2932 | -7.2656 | 0.0000 | 0.0000 | 17.5614 |
| DYRK2 | -0.4332 | 5.1120 | -7.2642 | 0.0000 | 0.0000 | 17.5525 |
| PPP4R4 | 0.6943 | 4.3197 | 7.2600 | 0.0000 | 0.0000 | 17.5257 |
| DUSP1 | 0.2474 | 7.3434 | 7.2581 | 0.0000 | 0.0000 | 17.5141 |
| NBEAL2 | -0.3709 | 5.2199 | -7.2536 | 0.0000 | 0.0000 | 17.4858 |
| DERA | 0.1894 | 6.6962 | 7.2535 | 0.0000 | 0.0000 | 17.4847 |
| FANCE | -0.5102 | 4.8923 | -7.2534 | 0.0000 | 0.0000 | 17.4841 |
| KIFC1 | -0.6244 | 5.6244 | -7.2532 | 0.0000 | 0.0000 | 17.4832 |
| RFX2 | -0.5542 | 3.4589 | -7.2530 | 0.0000 | 0.0000 | 17.4815 |
| CALCOCO2 | 0.1360 | 6.5827 | 7.2524 | 0.0000 | 0.0000 | 17.4782 |
| PON3 | 0.4748 | 6.8500 | 7.2516 | 0.0000 | 0.0000 | 17.4728 |
| SLC35D1 | 0.2742 | 6.4758 | 7.2484 | 0.0000 | 0.0000 | 17.4525 |
| TMEM206 | -0.3425 | 5.0491 | -7.2478 | 0.0000 | 0.0000 | 17.4488 |
| **POF1B** | -1.5000 | 2.6580 | -7.2477 | 0.0000 | 0.0000 | 17.4485 |
| CAPN11 | 0.5521 | 2.6210 | 7.2468 | 0.0000 | 0.0000 | 17.4423 |
| DDI2 | 0.2654 | 5.9628 | 7.2450 | 0.0000 | 0.0000 | 17.4315 |
| C1RL | 0.2202 | 6.8812 | 7.2449 | 0.0000 | 0.0000 | 17.4304 |
| PPARA | 0.3349 | 6.1252 | 7.2397 | 0.0000 | 0.0000 | 17.3981 |
| HLA-E | 0.1362 | 7.6437 | 7.2393 | 0.0000 | 0.0000 | 17.3955 |
| DRAM1 | -0.3166 | 5.7017 | -7.2361 | 0.0000 | 0.0000 | 17.3754 |
| TOP2A | -0.6126 | 5.8468 | -7.2351 | 0.0000 | 0.0000 | 17.3687 |
| **ESR1** | 1.1276 | 3.0196 | 7.2346 | 0.0000 | 0.0000 | 17.3658 |
| ITM2B | 0.1568 | 7.1221 | 7.2322 | 0.0000 | 0.0000 | 17.3507 |
| FAM90A1 | -0.8585 | 1.5687 | -7.2310 | 0.0000 | 0.0000 | 17.3432 |
| DPYS | 0.8230 | 6.9004 | 7.2300 | 0.0000 | 0.0000 | 17.3368 |
| RAD54B | -0.5144 | 3.2866 | -7.2282 | 0.0000 | 0.0000 | 17.3252 |
| CSAD | 0.4104 | 6.0585 | 7.2274 | 0.0000 | 0.0000 | 17.3206 |
| FZD7 | -0.9143 | 3.9136 | -7.2263 | 0.0000 | 0.0000 | 17.3138 |
| BSDC1 | 0.1523 | 6.6288 | 7.2185 | 0.0000 | 0.0000 | 17.2641 |
| TOLLIP | 0.1435 | 6.9183 | 7.2149 | 0.0000 | 0.0000 | 17.2416 |

| PPP4R2 | 0.1782 | 6.4907 | 7.2136 | 0.0000 | 0.0000 | 17.2336 |
| --- | --- | --- | --- | --- | --- | --- |
| CADM1 | 0.4571 | 6.2025 | 7.2111 | 0.0000 | 0.0000 | 17.2177 |
| ZNF273 | -0.5711 | 2.7549 | -7.2074 | 0.0000 | 0.0000 | 17.1949 |
| IFI30 | -0.5791 | 3.5278 | -7.2073 | 0.0000 | 0.0000 | 17.1941 |
| MARCKSL1 | -0.2403 | 7.0069 | -7.2051 | 0.0000 | 0.0000 | 17.1803 |
| GPD1L | -0.5820 | 4.9310 | -7.2039 | 0.0000 | 0.0000 | 17.1725 |
| **HAO2** | 1.2763 | 5.7191 | 7.1992 | 0.0000 | 0.0000 | 17.1430 |
| PAK4 | -0.2079 | 6.1347 | -7.1988 | 0.0000 | 0.0000 | 17.1404 |
| ITCH | 0.1816 | 6.3559 | 7.1983 | 0.0000 | 0.0000 | 17.1375 |
| **SPDEF** | -1.2819 | 1.9817 | -7.1933 | 0.0000 | 0.0000 | 17.1064 |
| SLC6A6 | -0.9463 | 4.1407 | -7.1928 | 0.0000 | 0.0000 | 17.1031 |
| ANKRD46 | 0.3020 | 5.8710 | 7.1825 | 0.0000 | 0.0000 | 17.0381 |
| PFKP | -0.6917 | 5.0891 | -7.1814 | 0.0000 | 0.0000 | 17.0317 |
| SIX3 | -0.7289 | 0.4589 | -7.1782 | 0.0000 | 0.0000 | 17.0113 |
| S100PBP | -0.3114 | 4.8246 | -7.1778 | 0.0000 | 0.0000 | 17.0089 |
| PLBD1 | -0.7980 | 4.8994 | -7.1734 | 0.0000 | 0.0000 | 16.9817 |
| TREM1 | -0.9924 | 2.0365 | -7.1720 | 0.0000 | 0.0000 | 16.9727 |
| RNF39 | -0.8349 | 2.8181 | -7.1713 | 0.0000 | 0.0000 | 16.9683 |
| **S100A2** | -1.0774 | 2.8452 | -7.1705 | 0.0000 | 0.0000 | 16.9635 |
| APOM | 0.4081 | 7.4475 | 7.1695 | 0.0000 | 0.0000 | 16.9567 |
| CNOT6 | -0.2853 | 5.5024 | -7.1658 | 0.0000 | 0.0000 | 16.9339 |
| KANK3 | 0.4970 | 4.8939 | 7.1637 | 0.0000 | 0.0000 | 16.9210 |
| CDH5 | 0.3753 | 5.9894 | 7.1603 | 0.0000 | 0.0000 | 16.8998 |
| STK17A | -0.2872 | 5.7484 | -7.1593 | 0.0000 | 0.0000 | 16.8933 |
| UGT8 | -0.9948 | 1.1303 | -7.1587 | 0.0000 | 0.0000 | 16.8898 |
| NAP1L1 | -0.2000 | 6.4495 | -7.1584 | 0.0000 | 0.0000 | 16.8879 |
| CDADC1 | 0.3082 | 5.3252 | 7.1571 | 0.0000 | 0.0000 | 16.8796 |
| SLC25A38 | 0.1701 | 6.7706 | 7.1561 | 0.0000 | 0.0000 | 16.8733 |
| MGMT | 0.2782 | 6.8483 | 7.1498 | 0.0000 | 0.0000 | 16.8342 |
| FMO5 | 0.4746 | 6.9855 | 7.1473 | 0.0000 | 0.0000 | 16.8182 |
| CPT2 | 0.2430 | 6.7020 | 7.1443 | 0.0000 | 0.0000 | 16.7998 |
| GMPR2 | 0.1251 | 6.6342 | 7.1438 | 0.0000 | 0.0000 | 16.7965 |
| **SALL2** | -1.1475 | 3.5333 | -7.1425 | 0.0000 | 0.0000 | 16.7886 |
| C16orf70 | 0.3029 | 6.0229 | 7.1419 | 0.0000 | 0.0000 | 16.7845 |
| DNMT3A | -0.3872 | 5.2597 | -7.1417 | 0.0000 | 0.0000 | 16.7832 |
| SEC62 | 0.1325 | 6.7313 | 7.1415 | 0.0000 | 0.0000 | 16.7823 |
| PLG | 0.6693 | 7.3813 | 7.1385 | 0.0000 | 0.0000 | 16.7637 |
| ASL | 0.2659 | 7.1389 | 7.1360 | 0.0000 | 0.0000 | 16.7476 |
| CCR10 | -0.7141 | 2.3641 | -7.1330 | 0.0000 | 0.0000 | 16.7291 |
| DKKL1 | -0.9570 | 2.1563 | -7.1308 | 0.0000 | 0.0000 | 16.7152 |
| ACADVL | 0.1592 | 7.4123 | 7.1302 | 0.0000 | 0.0000 | 16.7116 |
| DTL | -0.6739 | 5.0771 | -7.1272 | 0.0000 | 0.0000 | 16.6933 |
| SAP18 | 0.1278 | 6.9986 | 7.1248 | 0.0000 | 0.0000 | 16.6780 |
| ZNF195 | -0.2475 | 5.2847 | -7.1239 | 0.0000 | 0.0000 | 16.6722 |
| PAOX | 0.3264 | 6.1877 | 7.1217 | 0.0000 | 0.0000 | 16.6585 |
| NLGN3 | -0.5739 | 1.7605 | -7.1211 | 0.0000 | 0.0000 | 16.6548 |
| MRPL2 | 0.1631 | 6.8857 | 7.1199 | 0.0000 | 0.0000 | 16.6473 |
| **CYP2A7** | 1.7178 | 4.4217 | 7.1184 | 0.0000 | 0.0000 | 16.6384 |
| PSAP | 0.0952 | 7.8313 | 7.1184 | 0.0000 | 0.0000 | 16.6383 |
| LRP12 | -0.7846 | 3.3127 | -7.1179 | 0.0000 | 0.0000 | 16.6349 |
| GALNT7 | -0.8156 | 3.2452 | -7.1161 | 0.0000 | 0.0000 | 16.6238 |
| LACTB2 | 0.2369 | 6.7329 | 7.1158 | 0.0000 | 0.0000 | 16.6220 |
| C17orf53 | -0.6107 | 4.4546 | -7.1137 | 0.0000 | 0.0000 | 16.6089 |

| **HAVCR1** | -1.2496 | 1.8663 | -7.1135 | 0.0000 | 0.0000 | 16.6077 |
| --- | --- | --- | --- | --- | --- | --- |
| ALDH3B1 | -0.4753 | 5.1899 | -7.1106 | 0.0000 | 0.0000 | 16.5899 |
| RGS17 | -0.5702 | 1.2719 | -7.1077 | 0.0000 | 0.0000 | 16.5716 |
| RAB6B | -0.5337 | 4.0085 | -7.1050 | 0.0000 | 0.0000 | 16.5552 |
| **KCNF1** | -1.3358 | 2.4742 | -7.1038 | 0.0000 | 0.0000 | 16.5476 |
| POLA2 | -0.3088 | 5.4619 | -7.1001 | 0.0000 | 0.0000 | 16.5244 |
| ACOT11 | -0.8344 | 1.7243 | -7.1001 | 0.0000 | 0.0000 | 16.5243 |
| IRF6 | 0.6460 | 6.5448 | 7.0998 | 0.0000 | 0.0000 | 16.5224 |
| TPMT | 0.1887 | 6.7860 | 7.0989 | 0.0000 | 0.0000 | 16.5171 |
| CD302 | 0.4249 | 6.0154 | 7.0983 | 0.0000 | 0.0000 | 16.5130 |
| ZWINT | -0.4031 | 6.0836 | -7.0970 | 0.0000 | 0.0000 | 16.5053 |
| TANC2 | -0.8196 | 2.5795 | -7.0966 | 0.0000 | 0.0000 | 16.5028 |
| SLC2A2 | 0.6753 | 7.2013 | 7.0959 | 0.0000 | 0.0000 | 16.4986 |
| GLUL | 0.3007 | 7.4629 | 7.0953 | 0.0000 | 0.0000 | 16.4946 |
| MAT1A | 0.4986 | 7.5189 | 7.0916 | 0.0000 | 0.0000 | 16.4716 |
| FAHD2A | 0.2376 | 6.0651 | 7.0914 | 0.0000 | 0.0000 | 16.4706 |
| ACADM | 0.2872 | 6.7412 | 7.0911 | 0.0000 | 0.0000 | 16.4685 |
| ENPP1 | 0.3606 | 6.2275 | 7.0908 | 0.0000 | 0.0000 | 16.4669 |
| HAO1 | 0.6803 | 7.1760 | 7.0907 | 0.0000 | 0.0000 | 16.4659 |
| **NR1I2** | 1.2430 | 5.3400 | 7.0903 | 0.0000 | 0.0000 | 16.4634 |
| TTLL1 | -0.3235 | 5.2834 | -7.0880 | 0.0000 | 0.0000 | 16.4493 |
| PTP4A1 | 0.1549 | 7.3973 | 7.0827 | 0.0000 | 0.0000 | 16.4166 |
| CD101 | -0.6308 | 2.9214 | -7.0822 | 0.0000 | 0.0000 | 16.4133 |
| **PRAME** | -1.7560 | 2.1776 | -7.0821 | 0.0000 | 0.0000 | 16.4131 |
| ABCA12 | -0.5727 | 0.5089 | -7.0797 | 0.0000 | 0.0000 | 16.3980 |
| QPRT | 0.2743 | 7.1880 | 7.0796 | 0.0000 | 0.0000 | 16.3972 |
| TBX19 | -0.4589 | 3.8647 | -7.0773 | 0.0000 | 0.0000 | 16.3832 |
| PFKFB3 | -0.6030 | 5.5855 | -7.0750 | 0.0000 | 0.0000 | 16.3691 |
| C2 | 0.3095 | 7.3842 | 7.0678 | 0.0000 | 0.0000 | 16.3244 |
| LRRC20 | 0.2660 | 6.5618 | 7.0673 | 0.0000 | 0.0000 | 16.3210 |
| CYP4A11 | 0.7634 | 6.9622 | 7.0661 | 0.0000 | 0.0000 | 16.3140 |
| TSPAN9 | 0.2065 | 6.6217 | 7.0656 | 0.0000 | 0.0000 | 16.3108 |
| DCAF16 | -0.3324 | 5.4842 | -7.0649 | 0.0000 | 0.0000 | 16.3060 |
| FBXO5 | -0.5159 | 4.4736 | -7.0614 | 0.0000 | 0.0000 | 16.2846 |
| TNFSF15 | -0.9429 | 2.5917 | -7.0603 | 0.0000 | 0.0000 | 16.2781 |
| CFH | 0.2985 | 7.4774 | 7.0572 | 0.0000 | 0.0000 | 16.2589 |
| AFM | 0.8383 | 6.8581 | 7.0571 | 0.0000 | 0.0000 | 16.2577 |
| BCAT1 | -0.6509 | 3.5773 | -7.0558 | 0.0000 | 0.0000 | 16.2501 |
| SLCO2A1 | 0.9778 | 4.8498 | 7.0556 | 0.0000 | 0.0000 | 16.2487 |
| FSCN2 | -0.7159 | 1.3732 | -7.0540 | 0.0000 | 0.0000 | 16.2389 |
| ESPL1 | -0.7715 | 4.4705 | -7.0533 | 0.0000 | 0.0000 | 16.2343 |
| XRCC3 | -0.4197 | 4.6091 | -7.0517 | 0.0000 | 0.0000 | 16.2248 |
| PCYT1B | -0.6922 | 0.7773 | -7.0491 | 0.0000 | 0.0000 | 16.2086 |
| IER3 | -0.4141 | 6.6134 | -7.0485 | 0.0000 | 0.0000 | 16.2051 |
| **SCTR** | -1.1251 | 3.1333 | -7.0476 | 0.0000 | 0.0000 | 16.1992 |
| AURKB | -0.6230 | 5.4197 | -7.0470 | 0.0000 | 0.0000 | 16.1955 |
| HSD11B1 | 0.8286 | 6.9074 | 7.0406 | 0.0000 | 0.0000 | 16.1560 |
| ZMYND8 | -0.2460 | 5.6556 | -7.0405 | 0.0000 | 0.0000 | 16.1558 |
| IRS2 | 0.3804 | 6.3747 | 7.0387 | 0.0000 | 0.0000 | 16.1442 |
| DUSP4 | -0.8178 | 3.5979 | -7.0366 | 0.0000 | 0.0000 | 16.1317 |
| ADH6 | 0.5622 | 6.9507 | 7.0352 | 0.0000 | 0.0000 | 16.1228 |
| NPAS1 | -0.8251 | 2.6782 | -7.0331 | 0.0000 | 0.0000 | 16.1097 |
| AFF4 | 0.2123 | 6.2825 | 7.0328 | 0.0000 | 0.0000 | 16.1083 |

| SFXN3 | -0.4254 | 5.4663 | -7.0303 | 0.0000 | 0.0000 | 16.0926 |
| --- | --- | --- | --- | --- | --- | --- |
| GGCX | 0.1802 | 6.8227 | 7.0283 | 0.0000 | 0.0000 | 16.0800 |
| ENTPD5 | 0.3563 | 6.6505 | 7.0271 | 0.0000 | 0.0000 | 16.0726 |
| TMED7 | 0.1572 | 6.9421 | 7.0252 | 0.0000 | 0.0000 | 16.0614 |
| ZCCHC24 | 0.2555 | 6.2477 | 7.0227 | 0.0000 | 0.0000 | 16.0455 |
| GPRC5D | -0.7661 | 2.6889 | -7.0223 | 0.0000 | 0.0000 | 16.0436 |
| ADAM28 | -0.8864 | 2.3933 | -7.0205 | 0.0000 | 0.0000 | 16.0323 |
| GNG11 | 0.2896 | 6.1572 | 7.0191 | 0.0000 | 0.0000 | 16.0238 |
| DHCR24 | 0.1502 | 7.7547 | 7.0186 | 0.0000 | 0.0000 | 16.0206 |
| C6 | 0.7023 | 6.8302 | 7.0180 | 0.0000 | 0.0000 | 16.0168 |
| GRM4 | -0.3763 | 0.2761 | -7.0166 | 0.0000 | 0.0000 | 16.0085 |
| ADO | -0.1718 | 5.7737 | -7.0125 | 0.0000 | 0.0000 | 15.9829 |
| PEX11B | 0.1300 | 6.8036 | 7.0103 | 0.0000 | 0.0000 | 15.9698 |
| **MNX1** | -1.1316 | 1.0011 | -7.0088 | 0.0000 | 0.0000 | 15.9604 |
| TESC | -0.8630 | 5.6457 | -7.0085 | 0.0000 | 0.0000 | 15.9583 |
| PPT1 | -0.1758 | 6.7282 | -7.0073 | 0.0000 | 0.0000 | 15.9513 |
| B3GALNT1 | -0.6969 | 3.8766 | -7.0054 | 0.0000 | 0.0000 | 15.9395 |
| CLSTN1 | -0.3195 | 6.3253 | -7.0052 | 0.0000 | 0.0000 | 15.9384 |
| SLC39A14 | 0.2195 | 7.0833 | 7.0030 | 0.0000 | 0.0000 | 15.9245 |
| LRRC1 | -0.9462 | 4.8148 | -7.0027 | 0.0000 | 0.0000 | 15.9228 |
| TMCO6 | 0.2878 | 6.1874 | 7.0006 | 0.0000 | 0.0000 | 15.9097 |
| **TMEM156** | -1.0390 | 4.0104 | -7.0003 | 0.0000 | 0.0000 | 15.9077 |
| PDE4A | -0.6743 | 4.4572 | -6.9991 | 0.0000 | 0.0000 | 15.9004 |
| DERL1 | 0.1628 | 6.7152 | 6.9990 | 0.0000 | 0.0000 | 15.9000 |
| RAB11B | 0.1170 | 7.1531 | 6.9936 | 0.0000 | 0.0000 | 15.8667 |
| KIF24 | -0.6213 | 3.3804 | -6.9909 | 0.0000 | 0.0000 | 15.8503 |
| CDK5R2 | -0.5359 | 0.3283 | -6.9883 | 0.0000 | 0.0000 | 15.8346 |
| KIF14 | -0.7752 | 3.7943 | -6.9882 | 0.0000 | 0.0000 | 15.8339 |
| NR3C1 | 0.2132 | 6.2025 | 6.9880 | 0.0000 | 0.0000 | 15.8326 |
| PGM1 | 0.2046 | 7.0831 | 6.9875 | 0.0000 | 0.0000 | 15.8294 |
| NFE2L1 | 0.1334 | 7.2263 | 6.9859 | 0.0000 | 0.0000 | 15.8194 |
| HSF2BP | -0.7011 | 2.6902 | -6.9814 | 0.0000 | 0.0000 | 15.7920 |
| NUDT13 | 0.4554 | 5.1282 | 6.9808 | 0.0000 | 0.0000 | 15.7883 |
| CARD9 | -0.5471 | 3.2740 | -6.9793 | 0.0000 | 0.0000 | 15.7793 |
| MCM4 | -0.3078 | 6.1388 | -6.9779 | 0.0000 | 0.0000 | 15.7706 |
| MRPS28 | 0.1960 | 6.5854 | 6.9777 | 0.0000 | 0.0000 | 15.7691 |
| CIB2 | -0.8432 | 4.2836 | -6.9767 | 0.0000 | 0.0000 | 15.7632 |
| DDT | 0.2211 | 7.2625 | 6.9680 | 0.0000 | 0.0000 | 15.7098 |
| PLCG2 | 0.4678 | 5.2422 | 6.9664 | 0.0000 | 0.0000 | 15.7000 |
| TRO | 0.8033 | 4.4835 | 6.9658 | 0.0000 | 0.0000 | 15.6964 |
| CNTNAP1 | -0.7267 | 3.7726 | -6.9657 | 0.0000 | 0.0000 | 15.6959 |
| **SLC5A1** | -1.1622 | 1.3871 | -6.9653 | 0.0000 | 0.0000 | 15.6933 |
| ZNF107 | -0.6406 | 3.6911 | -6.9650 | 0.0000 | 0.0000 | 15.6913 |
| RORC | 0.5902 | 6.6021 | 6.9618 | 0.0000 | 0.0000 | 15.6721 |
| MAPK7 | -0.2848 | 5.1412 | -6.9604 | 0.0000 | 0.0000 | 15.6632 |
| NFIC | 0.2218 | 6.7299 | 6.9593 | 0.0000 | 0.0000 | 15.6566 |
| F7 | 0.5479 | 6.9762 | 6.9588 | 0.0000 | 0.0000 | 15.6539 |
| LAMP2 | 0.1542 | 7.2295 | 6.9575 | 0.0000 | 0.0000 | 15.6454 |
| NIPSNAP1 | 0.1614 | 7.3297 | 6.9568 | 0.0000 | 0.0000 | 15.6414 |
| PJA2 | 0.1738 | 6.7159 | 6.9542 | 0.0000 | 0.0000 | 15.6253 |
| **NR0B1** | -1.2051 | 1.0583 | -6.9531 | 0.0000 | 0.0000 | 15.6190 |
| NUP93 | -0.2538 | 5.1588 | -6.9513 | 0.0000 | 0.0000 | 15.6080 |
| **KCNH2** | -1.1590 | 1.6706 | -6.9472 | 0.0000 | 0.0000 | 15.5829 |

| SIL1 | 0.1833 | 7.0622 | 6.9426 | 0.0000 | 0.0000 | 15.5544 |
| --- | --- | --- | --- | --- | --- | --- |
| **HOXB13** | -1.1415 | 1.0404 | -6.9415 | 0.0000 | 0.0000 | 15.5479 |
| **MMP7** | -1.5801 | 3.8317 | -6.9400 | 0.0000 | 0.0000 | 15.5389 |
| HDAC7 | -0.3443 | 5.5475 | -6.9398 | 0.0000 | 0.0000 | 15.5374 |
| APOA1 | 0.4226 | 8.1518 | 6.9381 | 0.0000 | 0.0000 | 15.5270 |
| C11orf71 | 0.2738 | 6.0248 | 6.9371 | 0.0000 | 0.0000 | 15.5212 |
| GNB3 | -0.5862 | 1.2722 | -6.9327 | 0.0000 | 0.0000 | 15.4945 |
| ETV5 | -0.4529 | 5.3614 | -6.9312 | 0.0000 | 0.0000 | 15.4848 |
| TRDMT1 | -0.5298 | 2.3446 | -6.9260 | 0.0000 | 0.0000 | 15.4536 |
| ACAN | -0.9705 | 1.8457 | -6.9258 | 0.0000 | 0.0000 | 15.4520 |
| LAMA1 | -0.8463 | 0.9012 | -6.9192 | 0.0000 | 0.0000 | 15.4121 |
| GPD1 | 0.6979 | 6.2962 | 6.9173 | 0.0000 | 0.0000 | 15.4002 |
| ADIPOR1 | 0.1111 | 7.1845 | 6.9165 | 0.0000 | 0.0000 | 15.3953 |
| OSBPL3 | -0.5535 | 4.5232 | -6.9161 | 0.0000 | 0.0000 | 15.3933 |
| SPEF1 | -0.7768 | 1.7545 | -6.9156 | 0.0000 | 0.0000 | 15.3902 |
| YIF1A | 0.1399 | 7.1947 | 6.9145 | 0.0000 | 0.0000 | 15.3836 |
| CRBN | 0.1619 | 5.8198 | 6.9120 | 0.0000 | 0.0000 | 15.3680 |
| GSTZ1 | 0.4720 | 5.7169 | 6.9104 | 0.0000 | 0.0000 | 15.3581 |
| MYCT1 | 0.4418 | 5.1699 | 6.9101 | 0.0000 | 0.0000 | 15.3566 |
| **PCSK1N** | -1.5882 | 2.7042 | -6.9095 | 0.0000 | 0.0000 | 15.3527 |
| SLIT1 | -0.5015 | 1.0229 | -6.9092 | 0.0000 | 0.0000 | 15.3511 |
| SH3BP4 | 0.2844 | 6.3980 | 6.9073 | 0.0000 | 0.0000 | 15.3398 |
| CCNJL | -0.9131 | 2.3913 | -6.9039 | 0.0000 | 0.0000 | 15.3191 |
| DLST | 0.1556 | 6.8493 | 6.9026 | 0.0000 | 0.0000 | 15.3112 |
| NDUFS4 | 0.1568 | 7.1247 | 6.9010 | 0.0000 | 0.0000 | 15.3015 |
| SCRN1 | -0.7003 | 4.5004 | -6.8992 | 0.0000 | 0.0000 | 15.2906 |
| MED22 | -0.2563 | 5.5218 | -6.8980 | 0.0000 | 0.0000 | 15.2833 |
| TUBA1C | -0.2699 | 6.2882 | -6.8962 | 0.0000 | 0.0000 | 15.2720 |
| MTFR1 | 0.2207 | 6.3999 | 6.8947 | 0.0000 | 0.0000 | 15.2632 |
| MLXIPL | 0.3890 | 7.1910 | 6.8922 | 0.0000 | 0.0000 | 15.2478 |
| IQSEC1 | 0.1701 | 6.6585 | 6.8920 | 0.0000 | 0.0000 | 15.2467 |
| OSBP | 0.1339 | 6.7494 | 6.8878 | 0.0000 | 0.0000 | 15.2212 |
| KIAA1024 | -0.6239 | 1.0812 | -6.8871 | 0.0000 | 0.0000 | 15.2171 |
| **LYPD1** | -1.1679 | 4.2362 | -6.8853 | 0.0000 | 0.0000 | 15.2060 |
| MRPL16 | 0.1263 | 6.8538 | 6.8853 | 0.0000 | 0.0000 | 15.2059 |
| MARK2 | -0.1813 | 5.9585 | -6.8825 | 0.0000 | 0.0000 | 15.1888 |
| SH2B2 | -0.4379 | 4.6268 | -6.8801 | 0.0000 | 0.0000 | 15.1743 |
| RHOF | -0.7493 | 2.5145 | -6.8797 | 0.0000 | 0.0000 | 15.1719 |
| SNTB1 | 0.2688 | 6.8365 | 6.8773 | 0.0000 | 0.0000 | 15.1574 |
| FOXL1 | -0.8714 | 2.2876 | -6.8700 | 0.0000 | 0.0000 | 15.1133 |
| EPAS1 | 0.1628 | 6.9351 | 6.8685 | 0.0000 | 0.0000 | 15.1043 |
| TAPT1 | 0.2465 | 5.8711 | 6.8650 | 0.0000 | 0.0000 | 15.0830 |
| ALDOB | 0.4761 | 7.8640 | 6.8638 | 0.0000 | 0.0000 | 15.0758 |
| EMILIN2 | -0.6526 | 4.3551 | -6.8633 | 0.0000 | 0.0000 | 15.0728 |
| **SFRP5** | -1.6465 | 2.4052 | -6.8610 | 0.0000 | 0.0000 | 15.0588 |
| PRIM2 | -0.2964 | 5.1968 | -6.8588 | 0.0000 | 0.0000 | 15.0452 |
| SLC9A5 | -0.5312 | 1.6095 | -6.8578 | 0.0000 | 0.0000 | 15.0397 |
| CNOT2 | -0.1391 | 5.5932 | -6.8573 | 0.0000 | 0.0000 | 15.0366 |
| MRPL19 | 0.1366 | 6.4266 | 6.8551 | 0.0000 | 0.0000 | 15.0229 |
| **F9** | 1.0708 | 6.6496 | 6.8515 | 0.0000 | 0.0000 | 15.0011 |
| TTF2 | -0.4892 | 4.0916 | -6.8483 | 0.0000 | 0.0000 | 14.9822 |
| IFITM3 | 0.1356 | 7.9757 | 6.8479 | 0.0000 | 0.0000 | 14.9795 |
| PLAGL2 | -0.3545 | 5.1574 | -6.8476 | 0.0000 | 0.0000 | 14.9775 |

| LCN1 | -0.5763 | 0.4918 | -6.8470 | 0.0000 | 0.0000 | 14.9741 |
| --- | --- | --- | --- | --- | --- | --- |
| LYPLA1 | 0.1838 | 6.6325 | 6.8469 | 0.0000 | 0.0000 | 14.9738 |
| ASCL2 | -0.9013 | 2.7755 | -6.8449 | 0.0000 | 0.0000 | 14.9616 |
| RAB40A | -0.5006 | 1.2161 | -6.8423 | 0.0000 | 0.0000 | 14.9459 |
| PLXNA3 | -0.6709 | 4.5965 | -6.8400 | 0.0000 | 0.0000 | 14.9319 |
| GMIP | -0.3455 | 5.4158 | -6.8395 | 0.0000 | 0.0000 | 14.9287 |
| PIK3R1 | 0.3307 | 6.2426 | 6.8394 | 0.0000 | 0.0000 | 14.9280 |
| RCAN1 | 0.3316 | 6.0971 | 6.8384 | 0.0000 | 0.0000 | 14.9222 |
| OBP2A | -0.5220 | 0.4211 | -6.8382 | 0.0000 | 0.0000 | 14.9213 |
| **IGF2BP3** | -1.2805 | 2.2901 | -6.8366 | 0.0000 | 0.0000 | 14.9113 |
| SNX3 | 0.1038 | 7.2993 | 6.8342 | 0.0000 | 0.0000 | 14.8969 |
| PHEX | -0.9194 | 1.6246 | -6.8336 | 0.0000 | 0.0000 | 14.8932 |
| BRCA1 | -0.5164 | 4.3961 | -6.8317 | 0.0000 | 0.0000 | 14.8819 |
| F13B | 0.7376 | 6.8729 | 6.8284 | 0.0000 | 0.0000 | 14.8617 |
| ARHGAP22 | -0.6429 | 2.9085 | -6.8272 | 0.0000 | 0.0000 | 14.8548 |
| KLKB1 | 0.4412 | 6.7914 | 6.8259 | 0.0000 | 0.0000 | 14.8466 |
| **MFAP2** | -1.1498 | 3.3772 | -6.8248 | 0.0000 | 0.0000 | 14.8405 |
| SLC2A6 | -0.7040 | 5.1769 | -6.8212 | 0.0000 | 0.0000 | 14.8185 |
| FOXM1 | -0.6438 | 5.3701 | -6.8210 | 0.0000 | 0.0000 | 14.8173 |
| **SNAP25** | -1.2581 | 3.5961 | -6.8187 | 0.0000 | 0.0000 | 14.8034 |
| CPN2 | 0.7611 | 7.0755 | 6.8181 | 0.0000 | 0.0000 | 14.8001 |
| FOXO1 | 0.3353 | 5.8362 | 6.8128 | 0.0000 | 0.0000 | 14.7679 |
| WWP1 | 0.1846 | 6.7906 | 6.8112 | 0.0000 | 0.0000 | 14.7585 |
| CLDN18 | -0.9094 | 1.0867 | -6.8099 | 0.0000 | 0.0000 | 14.7507 |
| C16orf59 | -0.5897 | 4.8756 | -6.8097 | 0.0000 | 0.0000 | 14.7492 |
| HPX | 0.4711 | 7.7430 | 6.8073 | 0.0000 | 0.0000 | 14.7346 |
| TTC31 | 0.1887 | 6.2257 | 6.8058 | 0.0000 | 0.0000 | 14.7257 |
| LDB2 | 0.4881 | 5.1339 | 6.8050 | 0.0000 | 0.0000 | 14.7208 |
| SLC25A44 | 0.1890 | 6.4372 | 6.8038 | 0.0000 | 0.0000 | 14.7141 |
| PDSS2 | 0.1991 | 6.1705 | 6.8035 | 0.0000 | 0.0000 | 14.7118 |
| F8 | 0.5553 | 4.7170 | 6.8032 | 0.0000 | 0.0000 | 14.7102 |
| RAB3IL1 | -0.5616 | 5.3156 | -6.8001 | 0.0000 | 0.0000 | 14.6916 |
| VANGL1 | -0.3308 | 4.7890 | -6.7990 | 0.0000 | 0.0000 | 14.6847 |
| DNAJA1 | 0.1239 | 7.3327 | 6.7966 | 0.0000 | 0.0000 | 14.6708 |
| NPR1 | 0.4556 | 5.1706 | 6.7963 | 0.0000 | 0.0000 | 14.6688 |
| VPS37C | -0.1703 | 5.8536 | -6.7943 | 0.0000 | 0.0000 | 14.6566 |
| ABCC10 | -0.3151 | 5.1776 | -6.7932 | 0.0000 | 0.0000 | 14.6504 |
| NDUFA2 | 0.1595 | 7.2922 | 6.7903 | 0.0000 | 0.0000 | 14.6329 |
| LMAN2 | 0.1207 | 7.5671 | 6.7889 | 0.0000 | 0.0000 | 14.6244 |
| INSL3 | -0.8418 | 1.6879 | -6.7813 | 0.0000 | 0.0000 | 14.5790 |
| **AKR1D1** | 1.1131 | 5.8742 | 6.7801 | 0.0000 | 0.0000 | 14.5714 |
| **CTSE** | -1.2781 | 1.8785 | -6.7762 | 0.0000 | 0.0000 | 14.5483 |
| HP | 0.5183 | 7.8440 | 6.7736 | 0.0000 | 0.0000 | 14.5328 |
| **ADORA1** | -1.1440 | 2.9676 | -6.7721 | 0.0000 | 0.0000 | 14.5236 |
| GPR63 | -0.5065 | 0.7727 | -6.7717 | 0.0000 | 0.0000 | 14.5213 |
| EIF1 | 0.1071 | 7.5643 | 6.7701 | 0.0000 | 0.0000 | 14.5118 |
| SPG21 | 0.1043 | 6.9205 | 6.7692 | 0.0000 | 0.0000 | 14.5066 |
| ARHGEF15 | 0.4022 | 5.0509 | 6.7680 | 0.0000 | 0.0000 | 14.4992 |
| ATP5B | 0.1030 | 7.7290 | 6.7676 | 0.0000 | 0.0000 | 14.4969 |
| ALG5 | 0.1560 | 6.7367 | 6.7668 | 0.0000 | 0.0000 | 14.4918 |
| KLC2 | -0.3097 | 5.5078 | -6.7614 | 0.0000 | 0.0000 | 14.4595 |
| CHST11 | -0.6852 | 4.6744 | -6.7607 | 0.0000 | 0.0000 | 14.4555 |
| CDK19 | -0.3353 | 5.2072 | -6.7592 | 0.0000 | 0.0000 | 14.4466 |

| TSPAN6 | 0.2041 | 6.8654 | 6.7578 | 0.0000 | 0.0000 | 14.4382 |
| --- | --- | --- | --- | --- | --- | --- |
| C3 | 0.2116 | 8.0409 | 6.7574 | 0.0000 | 0.0000 | 14.4358 |
| CLDN14 | 0.6133 | 6.0278 | 6.7570 | 0.0000 | 0.0000 | 14.4335 |
| CYBA | -0.4246 | 6.3794 | -6.7550 | 0.0000 | 0.0000 | 14.4211 |
| NUP188 | -0.2202 | 5.7819 | -6.7544 | 0.0000 | 0.0000 | 14.4179 |
| CEP135 | -0.5066 | 3.3107 | -6.7459 | 0.0000 | 0.0000 | 14.3671 |
| GRIK5 | -0.6083 | 1.5310 | -6.7452 | 0.0000 | 0.0000 | 14.3631 |
| AKR1A1 | 0.1475 | 7.2774 | 6.7437 | 0.0000 | 0.0000 | 14.3537 |
| ZWILCH | -0.3125 | 5.1053 | -6.7427 | 0.0000 | 0.0000 | 14.3479 |
| KIAA1324 | -0.8738 | 2.2891 | -6.7420 | 0.0000 | 0.0000 | 14.3440 |
| DEPTOR | 0.2475 | 6.4336 | 6.7385 | 0.0000 | 0.0000 | 14.3226 |
| NKX2-8 | -0.6108 | 0.3747 | -6.7367 | 0.0000 | 0.0000 | 14.3120 |
| SFPQ | -0.0804 | 6.8072 | -6.7360 | 0.0000 | 0.0000 | 14.3081 |
| NDUFC2 | 0.1757 | 6.9245 | 6.7359 | 0.0000 | 0.0000 | 14.3072 |
| NFKBIA | 0.1436 | 7.1918 | 6.7314 | 0.0000 | 0.0000 | 14.2808 |
| NUCB1 | 0.1208 | 7.5014 | 6.7308 | 0.0000 | 0.0000 | 14.2771 |
| RNF5 | 0.1260 | 7.3450 | 6.7299 | 0.0000 | 0.0000 | 14.2713 |
| WDR59 | 0.2037 | 5.9699 | 6.7293 | 0.0000 | 0.0000 | 14.2678 |
| GRIN2D | -0.7382 | 2.5917 | -6.7276 | 0.0000 | 0.0000 | 14.2576 |
| CACNB3 | -0.5596 | 3.4585 | -6.7268 | 0.0000 | 0.0000 | 14.2532 |
| F3 | -0.8337 | 4.0101 | -6.7238 | 0.0000 | 0.0000 | 14.2354 |
| JAM2 | 0.6298 | 4.2870 | 6.7218 | 0.0000 | 0.0000 | 14.2230 |
| HSD17B11 | 0.1755 | 7.2900 | 6.7206 | 0.0000 | 0.0000 | 14.2162 |
| MRPL49 | 0.1199 | 6.7860 | 6.7190 | 0.0000 | 0.0000 | 14.2066 |
| GNAZ | -0.9780 | 4.9212 | -6.7188 | 0.0000 | 0.0000 | 14.2056 |
| NDST1 | 0.1892 | 6.6005 | 6.7162 | 0.0000 | 0.0000 | 14.1903 |
| SACS | -0.6183 | 3.2823 | -6.7158 | 0.0000 | 0.0000 | 14.1879 |
| MBOAT7 | -0.1486 | 6.4773 | -6.7138 | 0.0000 | 0.0000 | 14.1759 |
| GRIK1 | -0.5012 | 1.2027 | -6.7138 | 0.0000 | 0.0000 | 14.1759 |
| FBN2 | -0.5161 | 0.8033 | -6.7135 | 0.0000 | 0.0000 | 14.1739 |
| C8A | 0.5819 | 7.1507 | 6.7126 | 0.0000 | 0.0000 | 14.1685 |
| H2AFX | -0.2245 | 6.6637 | -6.7119 | 0.0000 | 0.0000 | 14.1645 |
| TFR2 | 0.4647 | 7.4021 | 6.7119 | 0.0000 | 0.0000 | 14.1643 |
| EFR3A | 0.2216 | 6.5574 | 6.7118 | 0.0000 | 0.0000 | 14.1636 |
| ALDH8A1 | 0.6810 | 6.6350 | 6.7096 | 0.0000 | 0.0000 | 14.1509 |
| RIC8B | -0.2906 | 5.1195 | -6.7078 | 0.0000 | 0.0000 | 14.1398 |
| SLC37A1 | -0.4778 | 4.9459 | -6.7058 | 0.0000 | 0.0000 | 14.1279 |
| MED15 | -0.1630 | 6.0235 | -6.7056 | 0.0000 | 0.0000 | 14.1268 |
| ARL6IP1 | 0.1306 | 7.2123 | 6.7040 | 0.0000 | 0.0000 | 14.1172 |
| ACAD10 | 0.2069 | 6.0749 | 6.7035 | 0.0000 | 0.0000 | 14.1147 |
| **TMC5** | -1.3870 | 2.9734 | -6.7017 | 0.0000 | 0.0000 | 14.1036 |
| FUT3 | -0.9462 | 2.1575 | -6.7015 | 0.0000 | 0.0000 | 14.1029 |
| CASP5 | -0.6765 | 1.4192 | -6.6965 | 0.0000 | 0.0000 | 14.0728 |
| FUT8 | -0.6471 | 3.8175 | -6.6964 | 0.0000 | 0.0000 | 14.0725 |
| ATP8A2 | -0.6240 | 0.8738 | -6.6963 | 0.0000 | 0.0000 | 14.0715 |
| CCDC30 | -0.4194 | 1.5653 | -6.6952 | 0.0000 | 0.0000 | 14.0651 |
| NEK2 | -0.7082 | 5.1437 | -6.6945 | 0.0000 | 0.0000 | 14.0608 |
| TM9SF2 | 0.1169 | 7.2289 | 6.6944 | 0.0000 | 0.0000 | 14.0603 |
| PRKG2 | -0.5179 | 0.4825 | -6.6941 | 0.0000 | 0.0000 | 14.0584 |
| **INHA** | -1.1816 | 2.2219 | -6.6918 | 0.0000 | 0.0000 | 14.0453 |
| LMNB1 | -0.3867 | 6.0210 | -6.6914 | 0.0000 | 0.0000 | 14.0427 |
| ARL15 | 0.2223 | 5.9232 | 6.6912 | 0.0000 | 0.0000 | 14.0414 |
| UBE2C | -0.5168 | 6.1892 | -6.6861 | 0.0000 | 0.0000 | 14.0114 |

| AASS | 0.8823 | 5.1158 | 6.6818 | 0.0000 | 0.0000 | 13.9856 |
| --- | --- | --- | --- | --- | --- | --- |
| CTSF | 0.2591 | 7.0690 | 6.6818 | 0.0000 | 0.0000 | 13.9856 |
| STAT2 | 0.1485 | 6.6908 | 6.6788 | 0.0000 | 0.0000 | 13.9681 |
| FAM120A | 0.1004 | 6.8357 | 6.6759 | 0.0000 | 0.0000 | 13.9511 |
| CES3 | 0.7837 | 5.7774 | 6.6759 | 0.0000 | 0.0000 | 13.9509 |
| GYS1 | -0.1959 | 5.8872 | -6.6730 | 0.0000 | 0.0000 | 13.9337 |
| PKLR | 0.7355 | 6.8739 | 6.6730 | 0.0000 | 0.0000 | 13.9336 |
| REPS2 | 0.5158 | 4.7730 | 6.6711 | 0.0000 | 0.0000 | 13.9226 |
| FBXL2 | -0.6026 | 2.3109 | -6.6698 | 0.0000 | 0.0000 | 13.9145 |
| GJA3 | -0.6247 | 0.7018 | -6.6694 | 0.0000 | 0.0000 | 13.9121 |
| FCGR1B | -0.6805 | 1.7189 | -6.6693 | 0.0000 | 0.0000 | 13.9120 |
| MTHFS | 0.3032 | 6.4922 | 6.6666 | 0.0000 | 0.0000 | 13.8959 |
| WSB1 | -0.2528 | 5.9162 | -6.6651 | 0.0000 | 0.0000 | 13.8870 |
| GCSH | 0.2803 | 5.9970 | 6.6598 | 0.0000 | 0.0000 | 13.8558 |
| CASZ1 | -0.5742 | 3.1456 | -6.6585 | 0.0000 | 0.0000 | 13.8480 |
| CYP19A1 | -0.9906 | 1.0428 | -6.6564 | 0.0000 | 0.0000 | 13.8355 |
| QSER1 | -0.2899 | 5.1483 | -6.6553 | 0.0000 | 0.0000 | 13.8291 |
| CLMN | 0.3528 | 5.6350 | 6.6530 | 0.0000 | 0.0000 | 13.8156 |
| BHMT2 | 0.5087 | 7.2086 | 6.6524 | 0.0000 | 0.0000 | 13.8116 |
| FUT4 | -0.8038 | 4.0326 | -6.6503 | 0.0000 | 0.0000 | 13.7994 |
| NPHP1 | -0.5456 | 1.9499 | -6.6473 | 0.0000 | 0.0000 | 13.7819 |
| ZNF587 | -0.3728 | 4.6622 | -6.6471 | 0.0000 | 0.0000 | 13.7803 |
| STYK1 | -0.6311 | 0.8711 | -6.6466 | 0.0000 | 0.0000 | 13.7775 |
| FTL | 0.1318 | 8.4560 | 6.6444 | 0.0000 | 0.0000 | 13.7648 |
| ITGA1 | 0.2185 | 6.1653 | 6.6444 | 0.0000 | 0.0000 | 13.7644 |
| E2F3 | -0.3694 | 5.4435 | -6.6427 | 0.0000 | 0.0000 | 13.7548 |
| EEF1A1 | 0.0977 | 7.8370 | 6.6418 | 0.0000 | 0.0000 | 13.7494 |
| ZNF391 | -0.9590 | 2.1703 | -6.6417 | 0.0000 | 0.0000 | 13.7485 |
| TNFAIP1 | 0.1134 | 6.8470 | 6.6402 | 0.0000 | 0.0000 | 13.7399 |
| **ETV4** | -1.1043 | 5.0680 | -6.6400 | 0.0000 | 0.0000 | 13.7384 |
| DBF4B | -0.4502 | 4.1417 | -6.6398 | 0.0000 | 0.0000 | 13.7378 |
| MAK | -0.4455 | 1.5171 | -6.6376 | 0.0000 | 0.0000 | 13.7245 |
| RMND5A | 0.1845 | 6.5618 | 6.6369 | 0.0000 | 0.0000 | 13.7203 |
| POP1 | -0.2842 | 4.7423 | -6.6322 | 0.0000 | 0.0000 | 13.6925 |
| DNMT1 | -0.3222 | 5.7797 | -6.6269 | 0.0000 | 0.0000 | 13.6617 |
| KLF6 | 0.2312 | 6.7408 | 6.6225 | 0.0000 | 0.0000 | 13.6354 |
| CPNE3 | 0.1702 | 6.5725 | 6.6218 | 0.0000 | 0.0000 | 13.6315 |
| F5 | 0.3780 | 7.2163 | 6.6215 | 0.0000 | 0.0000 | 13.6299 |
| TMED3 | -0.9363 | 4.5034 | -6.6210 | 0.0000 | 0.0000 | 13.6265 |
| MYLIP | -0.3769 | 5.3463 | -6.6204 | 0.0000 | 0.0000 | 13.6234 |
| CPPED1 | 0.2261 | 6.4890 | 6.6168 | 0.0000 | 0.0000 | 13.6022 |
| **SCGB2A1** | -1.2925 | 2.2571 | -6.6162 | 0.0000 | 0.0000 | 13.5983 |
| SEMA3G | 0.5298 | 5.1170 | 6.6157 | 0.0000 | 0.0000 | 13.5953 |
| DBN1 | -0.4899 | 5.6781 | -6.6143 | 0.0000 | 0.0000 | 13.5874 |
| DLGAP4 | -0.1624 | 6.2507 | -6.6134 | 0.0000 | 0.0000 | 13.5822 |
| **BICC1** | -1.1689 | 4.5216 | -6.6132 | 0.0000 | 0.0000 | 13.5807 |
| ITIH3 | 0.4522 | 7.5845 | 6.6129 | 0.0000 | 0.0000 | 13.5789 |
| ZNF124 | -0.5723 | 3.7015 | -6.6114 | 0.0000 | 0.0000 | 13.5705 |
| **CYP2A13** | 1.4129 | 2.9394 | 6.6093 | 0.0000 | 0.0000 | 13.5579 |
| FGD6 | -0.5057 | 4.6426 | -6.6091 | 0.0000 | 0.0000 | 13.5567 |
| RRNAD1 | 0.1512 | 6.5581 | 6.6089 | 0.0000 | 0.0000 | 13.5559 |
| 44078.0000 | 0.5640 | 5.4898 | 6.6083 | 0.0000 | 0.0000 | 13.5523 |
| CHD5 | -0.3655 | 0.3569 | -6.6059 | 0.0000 | 0.0000 | 13.5379 |

| ADAM8 | -0.4842 | 4.6680 | -6.6050 | 0.0000 | 0.0000 | 13.5327 |
| --- | --- | --- | --- | --- | --- | --- |
| RAPGEF4 | 0.4578 | 5.3490 | 6.6047 | 0.0000 | 0.0000 | 13.5310 |
| NEK11 | -0.6335 | 2.9728 | -6.6035 | 0.0000 | 0.0000 | 13.5241 |
| KCNQ3 | -0.5435 | 1.0918 | -6.6032 | 0.0000 | 0.0000 | 13.5221 |
| **MYEF2** | -1.0309 | 1.9548 | -6.5999 | 0.0000 | 0.0000 | 13.5026 |
| **B3GNT3** | -1.3150 | 4.8894 | -6.5991 | 0.0000 | 0.0000 | 13.4980 |
| **GUCA2A** | -1.3553 | 2.2512 | -6.5976 | 0.0000 | 0.0000 | 13.4894 |
| RIN1 | -0.5658 | 3.8843 | -6.5974 | 0.0000 | 0.0000 | 13.4880 |
| AHSG | 0.5758 | 7.8324 | 6.5972 | 0.0000 | 0.0000 | 13.4867 |
| PHLDA2 | -0.6606 | 5.7561 | -6.5917 | 0.0000 | 0.0000 | 13.4545 |
| PTPRO | -0.6655 | 2.0415 | -6.5915 | 0.0000 | 0.0000 | 13.4532 |
| SUPT3H | -0.4485 | 4.3891 | -6.5903 | 0.0000 | 0.0000 | 13.4466 |
| PACS1 | -0.2328 | 5.9504 | -6.5882 | 0.0000 | 0.0000 | 13.4339 |
| ZNF93 | -0.7929 | 2.9207 | -6.5873 | 0.0000 | 0.0000 | 13.4287 |
| TSC22D3 | 0.3736 | 6.5833 | 6.5864 | 0.0000 | 0.0000 | 13.4237 |
| CREG1 | 0.1799 | 7.3759 | 6.5862 | 0.0000 | 0.0000 | 13.4227 |
| OASL | 0.5576 | 5.8202 | 6.5861 | 0.0000 | 0.0000 | 13.4217 |
| FANCG | -0.3040 | 5.5512 | -6.5854 | 0.0000 | 0.0000 | 13.4176 |
| PPP2R1B | 0.2538 | 6.6226 | 6.5831 | 0.0000 | 0.0000 | 13.4041 |
| KIF2A | -0.3519 | 5.0745 | -6.5828 | 0.0000 | 0.0000 | 13.4023 |
| RNF167 | 0.1120 | 7.0710 | 6.5825 | 0.0000 | 0.0000 | 13.4008 |
| GALNT6 | -0.6104 | 3.2290 | -6.5807 | 0.0000 | 0.0000 | 13.3904 |
| HDAC2 | -0.2364 | 5.5151 | -6.5773 | 0.0000 | 0.0000 | 13.3703 |
| ENTPD3 | -0.8441 | 1.1073 | -6.5758 | 0.0000 | 0.0000 | 13.3618 |
| CTSC | -0.3910 | 6.0322 | -6.5758 | 0.0000 | 0.0000 | 13.3617 |
| DCTN4 | 0.1279 | 6.3699 | 6.5748 | 0.0000 | 0.0000 | 13.3558 |
| FAM60A | -0.5019 | 5.0384 | -6.5726 | 0.0000 | 0.0000 | 13.3426 |
| POMT2 | -0.2693 | 4.8238 | -6.5718 | 0.0000 | 0.0000 | 13.3382 |
| TMED2 | 0.0882 | 7.3595 | 6.5700 | 0.0000 | 0.0000 | 13.3277 |
| NUDT1 | -0.3229 | 5.9009 | -6.5688 | 0.0000 | 0.0000 | 13.3205 |
| STMN1 | -0.2554 | 6.4707 | -6.5653 | 0.0000 | 0.0000 | 13.3003 |
| **CEACAM6** | -1.1252 | 1.1821 | -6.5629 | 0.0000 | 0.0000 | 13.2859 |
| PGAP3 | 0.1723 | 6.6776 | 6.5623 | 0.0000 | 0.0000 | 13.2825 |
| PBLD | 0.4263 | 6.5313 | 6.5620 | 0.0000 | 0.0000 | 13.2809 |
| AVPI1 | 0.2354 | 6.6672 | 6.5620 | 0.0000 | 0.0000 | 13.2807 |
| PRSS21 | -0.9574 | 1.2409 | -6.5614 | 0.0000 | 0.0000 | 13.2775 |
| **AREG** | -1.0238 | 3.2222 | -6.5610 | 0.0000 | 0.0000 | 13.2747 |
| CD34 | 0.3106 | 5.9407 | 6.5562 | 0.0000 | 0.0000 | 13.2472 |
| METTL4 | -0.2731 | 4.6269 | -6.5543 | 0.0000 | 0.0000 | 13.2360 |
| G6PC3 | -0.1888 | 6.0346 | -6.5524 | 0.0000 | 0.0000 | 13.2250 |
| SLC25A1 | 0.1368 | 7.4289 | 6.5524 | 0.0000 | 0.0000 | 13.2249 |
| EFHC1 | -0.4016 | 4.1981 | -6.5521 | 0.0000 | 0.0000 | 13.2231 |
| MAPRE1 | -0.1335 | 6.7408 | -6.5470 | 0.0000 | 0.0000 | 13.1934 |
| CES1 | 0.4236 | 7.7758 | 6.5418 | 0.0000 | 0.0000 | 13.1632 |
| CST8 | -0.5727 | 0.2507 | -6.5417 | 0.0000 | 0.0000 | 13.1623 |
| SV2A | -0.7618 | 2.8957 | -6.5388 | 0.0000 | 0.0000 | 13.1457 |
| KAT2B | 0.3356 | 6.0203 | 6.5380 | 0.0000 | 0.0000 | 13.1410 |
| ACAT2 | 0.2485 | 6.6365 | 6.5370 | 0.0000 | 0.0000 | 13.1351 |
| ECSIT | 0.1474 | 6.8799 | 6.5325 | 0.0000 | 0.0000 | 13.1088 |
| CPA2 | -0.8983 | 0.7514 | -6.5317 | 0.0000 | 0.0000 | 13.1043 |
| APOA2 | 0.3602 | 8.3462 | 6.5298 | 0.0000 | 0.0000 | 13.0930 |
| PITX2 | -0.8280 | 0.6422 | -6.5269 | 0.0000 | 0.0000 | 13.0761 |
| MPV17 | -0.1911 | 5.9747 | -6.5207 | 0.0000 | 0.0000 | 13.0401 |

| HNRNPK | 0.0627 | 7.3430 | 6.5200 | 0.0000 | 0.0000 | 13.0362 |
| --- | --- | --- | --- | --- | --- | --- |
| HYAL1 | 0.3083 | 7.0376 | 6.5174 | 0.0000 | 0.0000 | 13.0208 |
| CDKL1 | -0.6112 | 1.7728 | -6.5139 | 0.0000 | 0.0000 | 13.0005 |
| LHFPL2 | -0.5798 | 4.8026 | -6.5124 | 0.0000 | 0.0000 | 12.9919 |
| TFAP2A | -0.9456 | 2.0254 | -6.5103 | 0.0000 | 0.0000 | 12.9800 |
| SLC23A2 | 0.3403 | 6.3288 | 6.5076 | 0.0000 | 0.0000 | 12.9639 |
| NDUFA1 | 0.1316 | 7.5965 | 6.5072 | 0.0000 | 0.0000 | 12.9616 |
| CYP24A1 | -0.5761 | 0.3958 | -6.5048 | 0.0000 | 0.0000 | 12.9481 |
| VWF | 0.4360 | 6.1023 | 6.4998 | 0.0000 | 0.0000 | 12.9189 |
| ACYP2 | 0.2773 | 5.4765 | 6.4992 | 0.0000 | 0.0000 | 12.9153 |
| SRP14 | 0.0899 | 7.4229 | 6.4992 | 0.0000 | 0.0000 | 12.9153 |
| MGAT4B | 0.1510 | 7.1714 | 6.4946 | 0.0000 | 0.0000 | 12.8886 |
| RWDD2B | 0.2059 | 6.0248 | 6.4936 | 0.0000 | 0.0000 | 12.8827 |
| AMD1 | -0.1875 | 6.0588 | -6.4875 | 0.0000 | 0.0000 | 12.8475 |
| CD24 | -0.9491 | 6.3638 | -6.4854 | 0.0000 | 0.0000 | 12.8352 |
| **CAPN6** | -1.2680 | 1.9018 | -6.4848 | 0.0000 | 0.0000 | 12.8320 |
| FAM182B | -0.6459 | 1.4074 | -6.4845 | 0.0000 | 0.0000 | 12.8303 |
| **CXCL1** | -1.3157 | 4.3207 | -6.4835 | 0.0000 | 0.0000 | 12.8246 |
| OAZ2 | 0.1212 | 6.4489 | 6.4834 | 0.0000 | 0.0000 | 12.8236 |
| SLC25A5 | 0.1071 | 7.6425 | 6.4827 | 0.0000 | 0.0000 | 12.8197 |
| PLEKHB2 | -0.2191 | 6.0413 | -6.4812 | 0.0000 | 0.0000 | 12.8113 |
| CFB | 0.3106 | 7.2676 | 6.4805 | 0.0000 | 0.0000 | 12.8069 |
| ARNTL2 | -0.7663 | 4.5700 | -6.4792 | 0.0000 | 0.0000 | 12.7997 |
| **CEACAM7** | -1.0286 | 0.7301 | -6.4738 | 0.0000 | 0.0000 | 12.7685 |
| MASP1 | 0.5296 | 6.1342 | 6.4718 | 0.0000 | 0.0000 | 12.7570 |
| ACADS | 0.2054 | 6.9892 | 6.4715 | 0.0000 | 0.0000 | 12.7552 |
| RSAD1 | 0.1447 | 6.6181 | 6.4696 | 0.0000 | 0.0000 | 12.7440 |
| F11 | 0.5829 | 6.5180 | 6.4667 | 0.0000 | 0.0000 | 12.7272 |
| RNF125 | 0.6462 | 4.6779 | 6.4651 | 0.0000 | 0.0000 | 12.7180 |
| PRKAG2 | 0.3649 | 5.6285 | 6.4640 | 0.0000 | 0.0000 | 12.7120 |
| NCBP2 | -0.1086 | 6.3969 | -6.4633 | 0.0000 | 0.0000 | 12.7080 |
| ANXA8 | -0.7815 | 0.9986 | -6.4621 | 0.0000 | 0.0000 | 12.7008 |
| PROC | 0.4075 | 7.3965 | 6.4618 | 0.0000 | 0.0000 | 12.6993 |
| RPL36AL | 0.1239 | 7.6308 | 6.4609 | 0.0000 | 0.0000 | 12.6940 |
| CDH6 | -0.7768 | 3.5249 | -6.4599 | 0.0000 | 0.0000 | 12.6881 |
| RBM17 | -0.1106 | 6.3262 | -6.4566 | 0.0000 | 0.0000 | 12.6688 |
| TRIM26 | 0.1238 | 6.8553 | 6.4562 | 0.0000 | 0.0000 | 12.6666 |
| SEMA4A | -0.6769 | 3.5978 | -6.4556 | 0.0000 | 0.0000 | 12.6635 |
| ASNS | -0.8144 | 4.6949 | -6.4530 | 0.0000 | 0.0000 | 12.6480 |
| MRPL24 | 0.1532 | 7.3485 | 6.4523 | 0.0000 | 0.0000 | 12.6441 |
| TNFRSF1A | 0.1168 | 7.0735 | 6.4522 | 0.0000 | 0.0000 | 12.6437 |
| RAB2A | 0.1314 | 6.9163 | 6.4511 | 0.0000 | 0.0000 | 12.6372 |
| KLK6 | -0.7100 | 0.6011 | -6.4485 | 0.0000 | 0.0000 | 12.6223 |
| ANKLE2 | -0.1751 | 5.4639 | -6.4482 | 0.0000 | 0.0000 | 12.6204 |
| NBR1 | 0.1434 | 6.7562 | 6.4476 | 0.0000 | 0.0000 | 12.6172 |
| CCDC28B | -0.5553 | 4.8968 | -6.4463 | 0.0000 | 0.0000 | 12.6097 |
| RNF130 | 0.1669 | 6.4090 | 6.4430 | 0.0000 | 0.0000 | 12.5904 |
| ARG1 | 0.6664 | 7.2450 | 6.4421 | 0.0000 | 0.0000 | 12.5857 |
| TCF15 | 0.6326 | 3.4981 | 6.4416 | 0.0000 | 0.0000 | 12.5825 |
| ABCC9 | 0.7876 | 4.7831 | 6.4412 | 0.0000 | 0.0000 | 12.5801 |
| GRIN1 | -0.5187 | 0.5774 | -6.4409 | 0.0000 | 0.0000 | 12.5784 |
| GJA4 | 0.3375 | 6.2028 | 6.4393 | 0.0000 | 0.0000 | 12.5696 |
| IGFBP3 | -0.2693 | 6.9054 | -6.4385 | 0.0000 | 0.0000 | 12.5646 |

| SCUBE3 | -0.5175 | 1.5099 | -6.4383 | 0.0000 | 0.0000 | 12.5638 |
| --- | --- | --- | --- | --- | --- | --- |
| COX6C | 0.1758 | 7.3368 | 6.4361 | 0.0000 | 0.0000 | 12.5509 |
| IQCA1 | -0.6497 | 1.5162 | -6.4358 | 0.0000 | 0.0000 | 12.5490 |
| TTC26 | -0.5242 | 3.7775 | -6.4356 | 0.0000 | 0.0000 | 12.5480 |
| ABTB2 | 0.3664 | 5.7874 | 6.4355 | 0.0000 | 0.0000 | 12.5478 |
| PTBP2 | -0.3610 | 4.2219 | -6.4347 | 0.0000 | 0.0000 | 12.5429 |
| DCHS2 | -0.4074 | 0.3698 | -6.4338 | 0.0000 | 0.0000 | 12.5375 |
| HMGCS1 | 0.2514 | 7.0041 | 6.4333 | 0.0000 | 0.0000 | 12.5346 |
| AVPR2 | 0.7085 | 1.9320 | 6.4332 | 0.0000 | 0.0000 | 12.5343 |
| FCGR1A | -0.8063 | 3.3315 | -6.4313 | 0.0000 | 0.0000 | 12.5232 |
| SCAP | 0.1251 | 6.8368 | 6.4310 | 0.0000 | 0.0000 | 12.5219 |
| PHF8 | 0.2879 | 5.8661 | 6.4272 | 0.0000 | 0.0000 | 12.4997 |
| OPLAH | 0.2445 | 6.6864 | 6.4242 | 0.0000 | 0.0000 | 12.4823 |
| NPAS2 | -0.4936 | 4.9078 | -6.4237 | 0.0000 | 0.0000 | 12.4800 |
| HEBP1 | 0.1628 | 6.7528 | 6.4232 | 0.0000 | 0.0000 | 12.4769 |
| MAP7D1 | -0.1682 | 6.2357 | -6.4221 | 0.0000 | 0.0000 | 12.4704 |
| LYRM1 | 0.1833 | 6.4839 | 6.4196 | 0.0000 | 0.0000 | 12.4564 |
| NRF1 | -0.1503 | 5.5612 | -6.4189 | 0.0000 | 0.0000 | 12.4524 |
| APH1A | 0.0886 | 7.3148 | 6.4148 | 0.0000 | 0.0000 | 12.4284 |
| GSTA1 | 0.6668 | 7.5539 | 6.4131 | 0.0000 | 0.0000 | 12.4186 |
| DNASE1 | -0.3972 | 3.7648 | -6.4121 | 0.0000 | 0.0000 | 12.4133 |
| BLVRA | -0.3623 | 6.3676 | -6.4079 | 0.0000 | 0.0000 | 12.3893 |
| ID2 | 0.1700 | 7.2558 | 6.4074 | 0.0000 | 0.0000 | 12.3861 |
| CSRNP2 | -0.2469 | 5.5034 | -6.4050 | 0.0000 | 0.0000 | 12.3726 |
| EMP2 | 0.2458 | 6.4087 | 6.4020 | 0.0000 | 0.0000 | 12.3551 |
| SERHL2 | -0.6685 | 3.3588 | -6.4003 | 0.0000 | 0.0000 | 12.3453 |
| **KCNB1** | 1.2289 | 3.0768 | 6.3990 | 0.0000 | 0.0000 | 12.3380 |
| MYO16 | 0.7508 | 2.7544 | 6.3982 | 0.0000 | 0.0000 | 12.3333 |
| AGXT | 0.6046 | 7.4606 | 6.3977 | 0.0000 | 0.0000 | 12.3303 |
| RAB4A | 0.1519 | 6.7721 | 6.3961 | 0.0000 | 0.0000 | 12.3216 |
| GLB1L2 | -0.9008 | 1.6326 | -6.3959 | 0.0000 | 0.0000 | 12.3203 |
| TSPAN3 | -0.2179 | 6.3333 | -6.3954 | 0.0000 | 0.0000 | 12.3172 |
| MRPL39 | 0.1409 | 6.6617 | 6.3945 | 0.0000 | 0.0000 | 12.3120 |
| ITGA2B | -0.5684 | 1.1445 | -6.3939 | 0.0000 | 0.0000 | 12.3090 |
| RING1 | 0.1188 | 7.0267 | 6.3934 | 0.0000 | 0.0000 | 12.3058 |
| RAD51AP1 | -0.5917 | 4.7824 | -6.3929 | 0.0000 | 0.0000 | 12.3033 |
| IMPA1 | 0.2046 | 6.3220 | 6.3919 | 0.0000 | 0.0000 | 12.2972 |
| **SLCO4C1** | -1.3336 | 2.6497 | -6.3917 | 0.0000 | 0.0000 | 12.2963 |
| EDEM1 | 0.1926 | 6.4747 | 6.3914 | 0.0000 | 0.0000 | 12.2945 |
| SLC37A4 | 0.2166 | 6.8577 | 6.3906 | 0.0000 | 0.0000 | 12.2898 |
| BRCA2 | -0.5497 | 3.0255 | -6.3895 | 0.0000 | 0.0000 | 12.2835 |
| KEAP1 | 0.1231 | 6.8723 | 6.3872 | 0.0000 | 0.0000 | 12.2705 |
| TBC1D10B | -0.1550 | 5.8960 | -6.3839 | 0.0000 | 0.0000 | 12.2514 |
| CTDSP1 | 0.1028 | 7.0400 | 6.3829 | 0.0000 | 0.0000 | 12.2460 |
| PRPSAP1 | 0.1772 | 6.5101 | 6.3805 | 0.0000 | 0.0000 | 12.2320 |
| NUP107 | -0.2241 | 5.6024 | -6.3801 | 0.0000 | 0.0000 | 12.2299 |
| C2orf72 | 0.2173 | 7.0921 | 6.3766 | 0.0000 | 0.0000 | 12.2097 |
| LMF1 | 0.4030 | 5.4214 | 6.3755 | 0.0000 | 0.0000 | 12.2037 |
| DR1 | -0.1737 | 5.7461 | -6.3730 | 0.0000 | 0.0000 | 12.1891 |
| MRPL15 | 0.1391 | 7.1767 | 6.3727 | 0.0000 | 0.0000 | 12.1874 |
| S100A11 | -0.2034 | 7.2629 | -6.3676 | 0.0000 | 0.0000 | 12.1586 |
| FARS2 | 0.1723 | 6.3272 | 6.3668 | 0.0000 | 0.0000 | 12.1540 |
| ATP5H | 0.1270 | 7.2462 | 6.3649 | 0.0000 | 0.0000 | 12.1434 |

| SAMHD1 | 0.2042 | 6.5443 | 6.3639 | 0.0000 | 0.0000 | 12.1376 |
| --- | --- | --- | --- | --- | --- | --- |
| ADRA2B | 0.5981 | 3.7505 | 6.3633 | 0.0000 | 0.0000 | 12.1343 |
| BEST1 | -0.6148 | 2.7317 | -6.3628 | 0.0000 | 0.0000 | 12.1310 |
| CSNK1E | -0.1567 | 6.2752 | -6.3600 | 0.0000 | 0.0000 | 12.1154 |
| GGH | 0.3313 | 7.1136 | 6.3587 | 0.0000 | 0.0000 | 12.1079 |
| WDR76 | -0.5382 | 4.8375 | -6.3579 | 0.0000 | 0.0000 | 12.1032 |
| CD58 | -0.2282 | 5.8696 | -6.3577 | 0.0000 | 0.0000 | 12.1020 |
| KCTD7 | -0.3885 | 4.3379 | -6.3548 | 0.0000 | 0.0000 | 12.0857 |
| RBBP8 | -0.3155 | 5.5137 | -6.3544 | 0.0000 | 0.0000 | 12.0835 |
| PRKAR1A | 0.1367 | 6.9542 | 6.3504 | 0.0000 | 0.0000 | 12.0606 |
| CLCNKB | -0.5361 | 0.6202 | -6.3489 | 0.0000 | 0.0000 | 12.0522 |
| RAB36 | -0.8227 | 2.2714 | -6.3482 | 0.0000 | 0.0000 | 12.0481 |
| ITGA2 | -0.7905 | 4.1893 | -6.3479 | 0.0000 | 0.0000 | 12.0465 |
| GABARAPL2 | 0.1169 | 6.7709 | 6.3478 | 0.0000 | 0.0000 | 12.0456 |
| PIP5K1C | -0.1917 | 5.7919 | -6.3467 | 0.0000 | 0.0000 | 12.0397 |
| PPP6R2 | 0.1506 | 6.9338 | 6.3465 | 0.0000 | 0.0000 | 12.0383 |
| MEPCE | 0.1239 | 6.7875 | 6.3451 | 0.0000 | 0.0000 | 12.0305 |
| ZNF142 | -0.2380 | 5.1720 | -6.3448 | 0.0000 | 0.0000 | 12.0289 |
| **HUNK** | -1.0896 | 2.7674 | -6.3440 | 0.0000 | 0.0000 | 12.0241 |
| ITIH1 | 0.4286 | 7.6427 | 6.3396 | 0.0000 | 0.0000 | 11.9994 |
| LGALS14 | -0.8171 | 0.4978 | -6.3379 | 0.0000 | 0.0000 | 11.9897 |
| ALB | 0.2437 | 8.4357 | 6.3340 | 0.0000 | 0.0000 | 11.9673 |
| COX17 | 0.1506 | 6.8226 | 6.3315 | 0.0000 | 0.0000 | 11.9530 |
| CMPK1 | 0.1029 | 7.1648 | 6.3304 | 0.0000 | 0.0000 | 11.9468 |
| IGFBP7 | 0.1593 | 7.4254 | 6.3294 | 0.0000 | 0.0000 | 11.9414 |
| POFUT1 | 0.1435 | 6.6708 | 6.3292 | 0.0000 | 0.0000 | 11.9402 |
| TUBAL3 | -0.6490 | 0.5319 | -6.3280 | 0.0000 | 0.0000 | 11.9333 |
| CATSPERB | -0.8033 | 1.4339 | -6.3277 | 0.0000 | 0.0000 | 11.9318 |
| ISCU | 0.1176 | 6.7253 | 6.3262 | 0.0000 | 0.0000 | 11.9232 |
| KLHL29 | -0.8443 | 3.9804 | -6.3258 | 0.0000 | 0.0000 | 11.9209 |
| MRPL34 | 0.1421 | 7.0746 | 6.3216 | 0.0000 | 0.0000 | 11.8967 |
| XYLB | 0.3823 | 6.1132 | 6.3204 | 0.0000 | 0.0000 | 11.8903 |
| POLRMT | 0.1418 | 6.6761 | 6.3197 | 0.0000 | 0.0000 | 11.8862 |
| AGPAT3 | 0.1668 | 6.5584 | 6.3187 | 0.0000 | 0.0000 | 11.8807 |
| FHL3 | -0.2712 | 6.0854 | -6.3187 | 0.0000 | 0.0000 | 11.8805 |
| **TMPRSS3** | -1.2528 | 3.4663 | -6.3180 | 0.0000 | 0.0000 | 11.8764 |
| PEPD | 0.1667 | 7.0723 | 6.3164 | 0.0000 | 0.0000 | 11.8674 |
| PRRX2 | -0.9761 | 1.7288 | -6.3151 | 0.0000 | 0.0000 | 11.8601 |
| KCNG1 | -0.8764 | 1.2862 | -6.3118 | 0.0000 | 0.0000 | 11.8414 |
| ST6GAL1 | 0.2478 | 7.2678 | 6.3112 | 0.0000 | 0.0000 | 11.8382 |
| AMACR | 0.5075 | 5.9055 | 6.3108 | 0.0000 | 0.0000 | 11.8356 |
| MSH2 | -0.2557 | 5.7850 | -6.3095 | 0.0000 | 0.0000 | 11.8283 |
| NDUFV1 | 0.1294 | 7.1895 | 6.3090 | 0.0000 | 0.0000 | 11.8257 |
| CREBL2 | 0.1691 | 6.6000 | 6.3049 | 0.0000 | 0.0000 | 11.8025 |
| **SEMA3C** | -1.0108 | 2.3608 | -6.3035 | 0.0000 | 0.0000 | 11.7945 |
| GANAB | 0.0793 | 7.3769 | 6.3034 | 0.0000 | 0.0000 | 11.7942 |
| SNX4 | 0.1314 | 6.7020 | 6.3030 | 0.0000 | 0.0000 | 11.7915 |
| TMEM208 | 0.1578 | 7.0531 | 6.3028 | 0.0000 | 0.0000 | 11.7907 |
| MYO19 | -0.2805 | 5.3777 | -6.3019 | 0.0000 | 0.0000 | 11.7856 |
| CHML | -0.5435 | 5.0085 | -6.3006 | 0.0000 | 0.0000 | 11.7779 |
| CLCF1 | -0.5700 | 5.0227 | -6.2998 | 0.0000 | 0.0000 | 11.7736 |
| BIRC7 | -0.9320 | 1.8195 | -6.2991 | 0.0000 | 0.0000 | 11.7693 |
| SNRNP40 | -0.1578 | 6.0115 | -6.2971 | 0.0000 | 0.0000 | 11.7580 |

| SLCO1B1 | 0.6680 | 6.8894 | 6.2941 | 0.0000 | 0.0000 | 11.7411 |
| --- | --- | --- | --- | --- | --- | --- |
| GALK1 | 0.2216 | 7.0661 | 6.2926 | 0.0000 | 0.0000 | 11.7331 |
| LIPA | 0.1534 | 6.8336 | 6.2883 | 0.0000 | 0.0000 | 11.7084 |
| TCTA | 0.1513 | 6.7887 | 6.2865 | 0.0000 | 0.0000 | 11.6987 |
| SUMO1 | 0.0903 | 7.1938 | 6.2848 | 0.0000 | 0.0000 | 11.6888 |
| CRABP2 | -0.8024 | 3.8486 | -6.2844 | 0.0000 | 0.0000 | 11.6865 |
| PBK | -0.5994 | 5.1948 | -6.2843 | 0.0000 | 0.0000 | 11.6862 |
| SCN5A | -0.3893 | 0.4261 | -6.2832 | 0.0000 | 0.0000 | 11.6799 |
| SEC24A | 0.2133 | 6.2269 | 6.2805 | 0.0000 | 0.0000 | 11.6643 |
| C4BPB | 0.4343 | 7.2530 | 6.2800 | 0.0000 | 0.0000 | 11.6619 |
| NUSAP1 | -0.3516 | 6.1430 | -6.2794 | 0.0000 | 0.0000 | 11.6581 |
| PHLPP1 | 0.2930 | 5.7291 | 6.2786 | 0.0000 | 0.0000 | 11.6539 |
| EIF4H | 0.0842 | 7.1763 | 6.2783 | 0.0000 | 0.0000 | 11.6520 |
| **PRSS16** | -1.1296 | 1.3577 | -6.2781 | 0.0000 | 0.0000 | 11.6513 |
| ECH1 | 0.1565 | 7.4977 | 6.2775 | 0.0000 | 0.0000 | 11.6477 |
| TONSL | -0.3640 | 5.3092 | -6.2764 | 0.0000 | 0.0000 | 11.6413 |
| KNG1 | 0.4762 | 7.7719 | 6.2752 | 0.0000 | 0.0000 | 11.6348 |
| TRPC1 | -0.7036 | 3.1573 | -6.2751 | 0.0000 | 0.0000 | 11.6344 |
| AKR1C4 | 0.5707 | 7.1758 | 6.2749 | 0.0000 | 0.0000 | 11.6330 |
| TNIK | -0.8526 | 3.8642 | -6.2704 | 0.0000 | 0.0000 | 11.6075 |
| INCENP | -0.4142 | 5.2054 | -6.2701 | 0.0000 | 0.0000 | 11.6057 |
| **HIST3H2A** | -1.1060 | 3.8859 | -6.2694 | 0.0000 | 0.0000 | 11.6018 |
| EEF1E1 | -0.2632 | 5.7439 | -6.2692 | 0.0000 | 0.0000 | 11.6010 |
| SEMA6A | -0.7714 | 4.2125 | -6.2690 | 0.0000 | 0.0000 | 11.5999 |
| COX7C | 0.1198 | 7.5346 | 6.2676 | 0.0000 | 0.0000 | 11.5918 |
| HLA-C | 0.1421 | 7.7908 | 6.2652 | 0.0000 | 0.0000 | 11.5783 |
| TBC1D22B | -0.2339 | 5.5363 | -6.2610 | 0.0000 | 0.0000 | 11.5545 |
| NDUFA10 | 0.1277 | 6.5175 | 6.2607 | 0.0000 | 0.0000 | 11.5530 |
| CCNA2 | -0.5158 | 5.5651 | -6.2592 | 0.0000 | 0.0000 | 11.5443 |
| LRRC49 | -0.5113 | 1.5309 | -6.2579 | 0.0000 | 0.0000 | 11.5371 |
| ATP6V0E1 | 0.1163 | 7.4279 | 6.2570 | 0.0000 | 0.0000 | 11.5321 |
| PRKCI | -0.2290 | 5.5597 | -6.2566 | 0.0000 | 0.0000 | 11.5300 |
| PSMF1 | 0.1094 | 7.0012 | 6.2549 | 0.0000 | 0.0000 | 11.5201 |
| EGFL6 | -0.8179 | 1.7859 | -6.2543 | 0.0000 | 0.0000 | 11.5171 |
| HMGA2 | -0.9414 | 0.8177 | -6.2534 | 0.0000 | 0.0000 | 11.5121 |
| CNN3 | 0.1478 | 7.1470 | 6.2532 | 0.0000 | 0.0000 | 11.5106 |
| MRPL40 | 0.1534 | 6.9362 | 6.2524 | 0.0000 | 0.0000 | 11.5064 |
| **GFRA1** | 1.2177 | 4.6657 | 6.2520 | 0.0000 | 0.0000 | 11.5038 |
| OPRD1 | -0.4065 | 0.8561 | -6.2439 | 0.0000 | 0.0000 | 11.4587 |
| **ACADL** | 1.2128 | 4.3974 | 6.2421 | 0.0000 | 0.0000 | 11.4484 |
| ZNF432 | -0.4126 | 4.4757 | -6.2407 | 0.0000 | 0.0000 | 11.4407 |
| ARSJ | -0.8819 | 3.2545 | -6.2406 | 0.0000 | 0.0000 | 11.4398 |
| ALOX5 | -0.7380 | 4.4658 | -6.2405 | 0.0000 | 0.0000 | 11.4393 |
| ZNF26 | -0.3383 | 3.6481 | -6.2403 | 0.0000 | 0.0000 | 11.4384 |
| PGM5 | 0.8733 | 3.4920 | 6.2401 | 0.0000 | 0.0000 | 11.4373 |
| ERG | 0.4420 | 4.5090 | 6.2391 | 0.0000 | 0.0000 | 11.4314 |
| MRPL44 | 0.1117 | 6.7878 | 6.2374 | 0.0000 | 0.0000 | 11.4224 |
| MICAL1 | -0.4497 | 5.1362 | -6.2372 | 0.0000 | 0.0000 | 11.4212 |
| HGD | 0.4214 | 7.3506 | 6.2361 | 0.0000 | 0.0000 | 11.4150 |
| ENPEP | 0.4866 | 5.8078 | 6.2344 | 0.0000 | 0.0000 | 11.4051 |
| TPH1 | -0.5702 | 1.1840 | -6.2311 | 0.0000 | 0.0000 | 11.3866 |
| ITPK1 | 0.1806 | 6.6884 | 6.2310 | 0.0000 | 0.0000 | 11.3862 |
| ARHGEF38 | -0.8097 | 1.5451 | -6.2273 | 0.0000 | 0.0000 | 11.3657 |

| ACOT1 | 0.4818 | 5.9125 | 6.2264 | 0.0000 | 0.0000 | 11.3603 |
| --- | --- | --- | --- | --- | --- | --- |
| ANKRD7 | -0.6173 | 0.6152 | -6.2244 | 0.0000 | 0.0000 | 11.3492 |
| KIF20B | -0.4718 | 4.3198 | -6.2219 | 0.0000 | 0.0000 | 11.3352 |
| OIP5 | -0.5656 | 4.8993 | -6.2217 | 0.0000 | 0.0000 | 11.3344 |
| GNE | 0.3066 | 6.5078 | 6.2203 | 0.0000 | 0.0000 | 11.3261 |
| FRMD4A | -0.4703 | 4.4919 | -6.2199 | 0.0000 | 0.0000 | 11.3241 |
| KLHL2 | 0.2893 | 5.9195 | 6.2188 | 0.0000 | 0.0000 | 11.3180 |
| SNRPA1 | -0.1725 | 6.1494 | -6.2183 | 0.0000 | 0.0000 | 11.3152 |
| ITGB3BP | -0.3067 | 5.0050 | -6.2172 | 0.0000 | 0.0000 | 11.3091 |
| TMEM14A | 0.1574 | 7.1721 | 6.2161 | 0.0000 | 0.0000 | 11.3029 |
| LAMP3 | -0.7898 | 3.8333 | -6.2140 | 0.0000 | 0.0000 | 11.2912 |
| DOK1 | -0.4356 | 4.7855 | -6.2118 | 0.0000 | 0.0000 | 11.2788 |
| ADAMTS3 | -0.7698 | 1.5538 | -6.2109 | 0.0000 | 0.0000 | 11.2739 |
| PRKRA | -0.1295 | 5.9515 | -6.2082 | 0.0000 | 0.0000 | 11.2588 |
| ENG | 0.2262 | 6.7615 | 6.2078 | 0.0000 | 0.0000 | 11.2564 |
| GPR161 | -0.5866 | 2.7545 | -6.2065 | 0.0000 | 0.0000 | 11.2491 |
| ZNF286A | -0.4399 | 2.8953 | -6.2054 | 0.0000 | 0.0000 | 11.2429 |
| BHLHE41 | -0.8750 | 3.5791 | -6.2048 | 0.0000 | 0.0000 | 11.2397 |
| ERP44 | 0.1168 | 6.7986 | 6.2046 | 0.0000 | 0.0000 | 11.2386 |
| PSMB7 | 0.1188 | 7.3526 | 6.2041 | 0.0000 | 0.0000 | 11.2355 |
| HERPUD1 | 0.1521 | 6.8845 | 6.1998 | 0.0000 | 0.0000 | 11.2117 |
| UBXN2B | 0.2318 | 6.1464 | 6.1995 | 0.0000 | 0.0000 | 11.2102 |
| THNSL1 | 0.2616 | 5.8593 | 6.1983 | 0.0000 | 0.0000 | 11.2035 |
| CORO1C | -0.1362 | 6.5090 | -6.1974 | 0.0000 | 0.0000 | 11.1983 |
| TMEM110 | 0.3461 | 5.0350 | 6.1971 | 0.0000 | 0.0000 | 11.1964 |
| DGKG | -0.7350 | 2.5102 | -6.1957 | 0.0000 | 0.0000 | 11.1886 |
| TMEM57 | 0.1430 | 6.6504 | 6.1935 | 0.0000 | 0.0000 | 11.1766 |
| BAAT | 0.4534 | 7.4610 | 6.1895 | 0.0000 | 0.0000 | 11.1544 |
| **NSUN7** | -1.0171 | 1.5778 | -6.1889 | 0.0000 | 0.0000 | 11.1507 |
| THUMPD2 | -0.2566 | 5.0561 | -6.1879 | 0.0000 | 0.0000 | 11.1456 |
| MBOAT2 | -0.7647 | 2.5407 | -6.1852 | 0.0000 | 0.0000 | 11.1305 |
| PHLDB1 | -0.6064 | 3.8819 | -6.1845 | 0.0000 | 0.0000 | 11.1266 |
| SLCO5A1 | -0.6197 | 1.0996 | -6.1845 | 0.0000 | 0.0000 | 11.1263 |
| CUEDC1 | -0.2948 | 5.6967 | -6.1828 | 0.0000 | 0.0000 | 11.1167 |
| HDC | 0.7897 | 2.4211 | 6.1815 | 0.0000 | 0.0000 | 11.1099 |
| IL17RC | 0.1877 | 6.5069 | 6.1807 | 0.0000 | 0.0000 | 11.1050 |
| PLIN3 | -0.1509 | 6.5904 | -6.1795 | 0.0000 | 0.0000 | 11.0983 |
| ANGPTL4 | 0.4150 | 6.9597 | 6.1792 | 0.0000 | 0.0000 | 11.0968 |
| MMP11 | -0.7302 | 5.0605 | -6.1787 | 0.0000 | 0.0000 | 11.0941 |
| SOCS2 | 0.6047 | 5.0214 | 6.1781 | 0.0000 | 0.0000 | 11.0908 |
| **IBSP** | -1.1151 | 1.6747 | -6.1737 | 0.0000 | 0.0000 | 11.0665 |
| DCLRE1B | -0.3025 | 4.9173 | -6.1732 | 0.0000 | 0.0000 | 11.0635 |
| FAM8A1 | 0.1976 | 6.8476 | 6.1724 | 0.0000 | 0.0000 | 11.0591 |
| COPS7B | -0.1531 | 5.9015 | -6.1707 | 0.0000 | 0.0000 | 11.0497 |
| CUZD1 | -0.6089 | 1.8550 | -6.1705 | 0.0000 | 0.0000 | 11.0484 |
| SH2D3A | -0.6515 | 3.7440 | -6.1699 | 0.0000 | 0.0000 | 11.0454 |
| NLGN1 | -0.5248 | 0.4481 | -6.1699 | 0.0000 | 0.0000 | 11.0451 |
| **PAEP** | -1.4230 | 1.9600 | -6.1695 | 0.0000 | 0.0000 | 11.0430 |
| SMPDL3A | 0.2276 | 6.6494 | 6.1689 | 0.0000 | 0.0000 | 11.0393 |
| FAM163A | -0.6449 | 1.2110 | -6.1673 | 0.0000 | 0.0000 | 11.0308 |
| SUN2 | 0.1674 | 6.8708 | 6.1640 | 0.0000 | 0.0000 | 11.0125 |
| ABCB9 | -0.5331 | 2.8361 | -6.1639 | 0.0000 | 0.0000 | 11.0116 |
| **GPR27** | -1.0302 | 1.5620 | -6.1629 | 0.0000 | 0.0000 | 11.0061 |

| GAD1 | -0.8847 | 1.2982 | -6.1605 | 0.0000 | 0.0000 | 10.9928 |
| --- | --- | --- | --- | --- | --- | --- |
| ROR1 | -0.9178 | 2.3282 | -6.1588 | 0.0000 | 0.0000 | 10.9834 |
| CST6 | -0.7669 | 1.2330 | -6.1584 | 0.0000 | 0.0000 | 10.9812 |
| KMO | 0.7901 | 5.1311 | 6.1576 | 0.0000 | 0.0000 | 10.9766 |
| SLC26A1 | 0.3734 | 5.9719 | 6.1559 | 0.0000 | 0.0000 | 10.9674 |
| DDRGK1 | 0.1265 | 7.2265 | 6.1542 | 0.0000 | 0.0000 | 10.9580 |
| EBP | 0.1832 | 7.3710 | 6.1529 | 0.0000 | 0.0000 | 10.9508 |
| ZSCAN5A | -0.3308 | 3.5744 | -6.1507 | 0.0000 | 0.0000 | 10.9386 |
| MAGT1 | 0.1214 | 6.9436 | 6.1498 | 0.0000 | 0.0000 | 10.9334 |
| **ADRA1A** | 1.1192 | 3.3514 | 6.1486 | 0.0000 | 0.0000 | 10.9267 |
| SCIN | -0.6660 | 1.2969 | -6.1471 | 0.0000 | 0.0000 | 10.9183 |
| RNF144A | -0.5448 | 4.3275 | -6.1469 | 0.0000 | 0.0000 | 10.9175 |
| UQCR11 | 0.1553 | 7.1205 | 6.1462 | 0.0000 | 0.0000 | 10.9136 |
| TRIM36 | -0.6535 | 1.9261 | -6.1456 | 0.0000 | 0.0000 | 10.9101 |
| DBT | 0.2679 | 5.6015 | 6.1454 | 0.0000 | 0.0000 | 10.9089 |
| TRPV5 | -0.3629 | 0.2828 | -6.1442 | 0.0000 | 0.0000 | 10.9022 |
| NOVA1 | -0.8890 | 2.0462 | -6.1434 | 0.0000 | 0.0000 | 10.8980 |
| IQCC | -0.3616 | 4.4549 | -6.1381 | 0.0000 | 0.0000 | 10.8685 |
| FAU | 0.1116 | 7.6344 | 6.1351 | 0.0000 | 0.0000 | 10.8522 |
| P4HB | 0.1028 | 7.8330 | 6.1329 | 0.0000 | 0.0000 | 10.8398 |
| ATP7B | 0.4186 | 5.5982 | 6.1264 | 0.0000 | 0.0000 | 10.8039 |
| MANBA | -0.2193 | 5.4566 | -6.1243 | 0.0000 | 0.0000 | 10.7923 |
| NDUFA4L2 | -0.3340 | 6.1768 | -6.1240 | 0.0000 | 0.0000 | 10.7908 |
| ADH1A | 0.5925 | 7.2825 | 6.1240 | 0.0000 | 0.0000 | 10.7906 |
| C11orf1 | 0.3408 | 5.4319 | 6.1217 | 0.0000 | 0.0000 | 10.7779 |
| NEDD4 | 0.4013 | 5.8067 | 6.1215 | 0.0000 | 0.0000 | 10.7770 |
| JAKMIP2 | 0.7350 | 2.4345 | 6.1214 | 0.0000 | 0.0000 | 10.7764 |
| LAMTOR2 | 0.1475 | 7.2673 | 6.1197 | 0.0000 | 0.0000 | 10.7670 |
| HSPA12A | -0.6738 | 3.4056 | -6.1196 | 0.0000 | 0.0000 | 10.7665 |
| TEK | 0.7335 | 4.3356 | 6.1190 | 0.0000 | 0.0000 | 10.7631 |
| SEMA4D | -0.6015 | 3.6760 | -6.1190 | 0.0000 | 0.0000 | 10.7628 |
| DRD1 | 0.9423 | 1.7968 | 6.1169 | 0.0000 | 0.0000 | 10.7516 |
| NCAPG2 | -0.3807 | 5.0859 | -6.1156 | 0.0000 | 0.0000 | 10.7442 |
| TNNI2 | -0.7231 | 3.9685 | -6.1147 | 0.0000 | 0.0000 | 10.7395 |
| TXN2 | 0.1188 | 7.1971 | 6.1116 | 0.0000 | 0.0000 | 10.7224 |
| DAGLA | -0.8592 | 3.8944 | -6.1109 | 0.0000 | 0.0000 | 10.7181 |
| SKP1 | 0.1143 | 6.6802 | 6.1105 | 0.0000 | 0.0000 | 10.7164 |
| SLC22A7 | 0.8304 | 6.7645 | 6.1096 | 0.0000 | 0.0000 | 10.7111 |
| CEP164 | -0.2423 | 4.8609 | -6.1087 | 0.0000 | 0.0000 | 10.7060 |
| USP12 | 0.2188 | 5.9925 | 6.1072 | 0.0000 | 0.0000 | 10.6982 |
| GDF11 | -0.5571 | 3.6444 | -6.1050 | 0.0000 | 0.0000 | 10.6856 |
| ADH1C | 0.7246 | 7.1189 | 6.1043 | 0.0000 | 0.0000 | 10.6818 |
| ASGR1 | 0.3566 | 7.4363 | 6.1029 | 0.0000 | 0.0000 | 10.6740 |
| FUBP1 | -0.1703 | 6.1895 | -6.1026 | 0.0000 | 0.0000 | 10.6728 |
| USP7 | 0.1019 | 6.4885 | 6.1025 | 0.0000 | 0.0000 | 10.6722 |
| AANAT | -0.5389 | 1.1529 | -6.1024 | 0.0000 | 0.0000 | 10.6716 |
| ARFGAP2 | 0.0998 | 6.7507 | 6.1009 | 0.0000 | 0.0000 | 10.6635 |
| NFKBIE | -0.2602 | 6.2661 | -6.1004 | 0.0000 | 0.0000 | 10.6606 |
| GPHN | 0.3356 | 5.8071 | 6.0997 | 0.0000 | 0.0000 | 10.6568 |
| ANXA10 | 0.8687 | 5.5767 | 6.0977 | 0.0000 | 0.0000 | 10.6455 |
| HS3ST3B1 | 0.5856 | 5.7471 | 6.0971 | 0.0000 | 0.0000 | 10.6425 |
| SRC | -0.5025 | 5.7135 | -6.0964 | 0.0000 | 0.0000 | 10.6385 |
| AMT | 0.3104 | 6.3986 | 6.0957 | 0.0000 | 0.0000 | 10.6346 |

| HAUS3 | -0.2356 | 4.8783 | -6.0950 | 0.0000 | 0.0000 | 10.6309 |
| --- | --- | --- | --- | --- | --- | --- |
| PKIG | 0.1386 | 6.5734 | 6.0939 | 0.0000 | 0.0000 | 10.6246 |
| APH1B | -0.4080 | 4.7059 | -6.0936 | 0.0000 | 0.0000 | 10.6229 |
| TMEM70 | 0.1862 | 6.6178 | 6.0926 | 0.0000 | 0.0000 | 10.6173 |
| CSF3R | -0.6624 | 3.6556 | -6.0918 | 0.0000 | 0.0000 | 10.6133 |
| EED | -0.1959 | 5.1774 | -6.0904 | 0.0000 | 0.0000 | 10.6057 |
| ARHGAP33 | -0.4033 | 4.6542 | -6.0893 | 0.0000 | 0.0000 | 10.5993 |
| RPL26L1 | 0.1374 | 6.7489 | 6.0839 | 0.0000 | 0.0000 | 10.5699 |
| ASNSD1 | 0.0921 | 6.6948 | 6.0809 | 0.0000 | 0.0000 | 10.5533 |
| COPS7A | 0.1030 | 6.8539 | 6.0796 | 0.0000 | 0.0000 | 10.5463 |
| GRIN2A | -0.7720 | 0.9248 | -6.0780 | 0.0000 | 0.0000 | 10.5371 |
| PEX11A | 0.2487 | 6.1664 | 6.0772 | 0.0000 | 0.0000 | 10.5329 |
| SERPIND1 | 0.4747 | 7.6304 | 6.0750 | 0.0000 | 0.0000 | 10.5209 |
| SLC4A5 | -0.3988 | 1.0892 | -6.0748 | 0.0000 | 0.0000 | 10.5199 |
| RAI1 | -0.4257 | 4.9527 | -6.0739 | 0.0000 | 0.0000 | 10.5148 |
| TIMM17A | 0.1481 | 6.8673 | 6.0734 | 0.0000 | 0.0000 | 10.5119 |
| ZNF446 | -0.2195 | 4.9304 | -6.0721 | 0.0000 | 0.0000 | 10.5047 |
| B3GNTL1 | -0.3873 | 4.3910 | -6.0718 | 0.0000 | 0.0000 | 10.5032 |
| GINS4 | -0.6646 | 3.6086 | -6.0715 | 0.0000 | 0.0000 | 10.5015 |
| PDP1 | -0.6485 | 4.3730 | -6.0710 | 0.0000 | 0.0000 | 10.4987 |
| PDGFRL | -0.6141 | 3.8709 | -6.0705 | 0.0000 | 0.0000 | 10.4958 |
| SERPINH1 | -0.1728 | 6.7567 | -6.0696 | 0.0000 | 0.0000 | 10.4912 |
| ZNF248 | -0.2819 | 4.5170 | -6.0685 | 0.0000 | 0.0000 | 10.4854 |
| DNAH17 | -0.4728 | 1.7975 | -6.0643 | 0.0000 | 0.0000 | 10.4621 |
| LAMA5 | -0.3637 | 5.7755 | -6.0639 | 0.0000 | 0.0000 | 10.4601 |
| TMPRSS6 | 0.5333 | 6.9525 | 6.0628 | 0.0000 | 0.0000 | 10.4540 |
| CFI | 0.2188 | 7.3440 | 6.0612 | 0.0000 | 0.0000 | 10.4452 |
| STRN4 | -0.1228 | 6.2789 | -6.0586 | 0.0000 | 0.0000 | 10.4311 |
| TBCA | 0.1269 | 7.0348 | 6.0553 | 0.0000 | 0.0000 | 10.4130 |
| SLC28A1 | 0.9370 | 5.6742 | 6.0539 | 0.0000 | 0.0000 | 10.4051 |
| RRM2 | -0.4606 | 5.8799 | -6.0524 | 0.0000 | 0.0000 | 10.3970 |
| LIG1 | -0.2254 | 5.9486 | -6.0518 | 0.0000 | 0.0000 | 10.3935 |
| SUCLG1 | 0.1292 | 6.9781 | 6.0515 | 0.0000 | 0.0000 | 10.3921 |
| NOL12 | -0.2720 | 4.7337 | -6.0507 | 0.0000 | 0.0000 | 10.3876 |
| ANAPC1 | -0.2775 | 4.2568 | -6.0497 | 0.0000 | 0.0000 | 10.3822 |
| RLN2 | -0.4729 | 0.5981 | -6.0456 | 0.0000 | 0.0000 | 10.3596 |
| AP3M2 | -0.3248 | 4.8037 | -6.0421 | 0.0000 | 0.0000 | 10.3405 |
| PEX5 | 0.1522 | 6.4922 | 6.0420 | 0.0000 | 0.0000 | 10.3400 |
| UBAP2 | -0.2367 | 5.3259 | -6.0416 | 0.0000 | 0.0000 | 10.3379 |
| GPX1 | 0.1260 | 7.6618 | 6.0400 | 0.0000 | 0.0000 | 10.3288 |
| FERMT2 | 0.2224 | 6.1421 | 6.0395 | 0.0000 | 0.0000 | 10.3261 |
| NMU | -0.7127 | 0.9832 | -6.0366 | 0.0000 | 0.0000 | 10.3107 |
| **NPTX2** | -1.3112 | 3.1094 | -6.0365 | 0.0000 | 0.0000 | 10.3100 |
| **TMEM100** | 1.0057 | 3.4765 | 6.0352 | 0.0000 | 0.0000 | 10.3028 |
| RFNG | 0.1619 | 6.8027 | 6.0337 | 0.0000 | 0.0000 | 10.2947 |
| TRMU | -0.2219 | 5.5851 | -6.0335 | 0.0000 | 0.0000 | 10.2935 |
| SLN | -0.7993 | 0.7728 | -6.0333 | 0.0000 | 0.0000 | 10.2924 |
| PMEL | 0.4674 | 4.8133 | 6.0331 | 0.0000 | 0.0000 | 10.2912 |
| BCL10 | -0.1681 | 5.8194 | -6.0320 | 0.0000 | 0.0000 | 10.2853 |
| ZNF185 | -0.3896 | 4.2362 | -6.0303 | 0.0000 | 0.0000 | 10.2761 |
| FZD4 | 0.2912 | 5.9051 | 6.0297 | 0.0000 | 0.0000 | 10.2729 |
| STAU1 | 0.0962 | 7.1633 | 6.0257 | 0.0000 | 0.0000 | 10.2509 |
| DUOX1 | -0.7022 | 3.0517 | -6.0256 | 0.0000 | 0.0000 | 10.2506 |

| JTB | 0.1021 | 7.5142 | 6.0246 | 0.0000 | 0.0000 | 10.2452 |
| --- | --- | --- | --- | --- | --- | --- |
| **DUOX2** | -1.2582 | 2.3210 | -6.0234 | 0.0000 | 0.0000 | 10.2384 |
| H6PD | 0.2175 | 6.8268 | 6.0231 | 0.0000 | 0.0000 | 10.2367 |
| DMWD | -0.1602 | 5.6039 | -6.0227 | 0.0000 | 0.0000 | 10.2345 |
| LIPC | 0.4947 | 6.5923 | 6.0225 | 0.0000 | 0.0000 | 10.2333 |
| RAVER2 | -0.7067 | 4.2144 | -6.0217 | 0.0000 | 0.0000 | 10.2294 |
| AMIGO2 | -0.7080 | 4.3544 | -6.0215 | 0.0000 | 0.0000 | 10.2283 |
| RFX4 | -0.3281 | 0.2438 | -6.0212 | 0.0000 | 0.0000 | 10.2266 |
| HRG | 0.7397 | 7.3649 | 6.0187 | 0.0000 | 0.0000 | 10.2126 |
| COX6A1 | 0.1154 | 7.4887 | 6.0180 | 0.0000 | 0.0000 | 10.2089 |
| SYPL1 | 0.1025 | 7.0121 | 6.0177 | 0.0000 | 0.0000 | 10.2073 |
| OXA1L | 0.0999 | 7.0073 | 6.0161 | 0.0000 | 0.0000 | 10.1987 |
| HOXA6 | -0.7435 | 0.8817 | -6.0155 | 0.0000 | 0.0000 | 10.1954 |
| COL11A1 | -0.9965 | 1.5839 | -6.0155 | 0.0000 | 0.0000 | 10.1952 |
| RAB8A | 0.1308 | 6.3329 | 6.0153 | 0.0000 | 0.0000 | 10.1945 |
| SLC39A9 | 0.1324 | 6.5922 | 6.0102 | 0.0000 | 0.0000 | 10.1664 |
| IFT57 | -0.5273 | 5.0632 | -6.0087 | 0.0000 | 0.0000 | 10.1585 |
| NT5DC2 | -0.4851 | 5.6967 | -6.0084 | 0.0000 | 0.0000 | 10.1566 |
| C11orf80 | -0.4530 | 4.8291 | -6.0063 | 0.0000 | 0.0000 | 10.1452 |
| AKR7A3 | 0.6755 | 6.5891 | 6.0058 | 0.0000 | 0.0000 | 10.1426 |
| PACRG | 0.8712 | 3.5663 | 6.0047 | 0.0000 | 0.0000 | 10.1365 |
| UCHL1 | -0.9871 | 3.7113 | -6.0033 | 0.0000 | 0.0000 | 10.1292 |
| CPT1A | 0.1998 | 6.8553 | 5.9980 | 0.0000 | 0.0000 | 10.1000 |
| BCAS3 | 0.2616 | 5.1990 | 5.9960 | 0.0000 | 0.0000 | 10.0895 |
| CTDSPL | -0.2060 | 6.1615 | -5.9954 | 0.0000 | 0.0000 | 10.0862 |
| STK39 | -0.6712 | 5.0756 | -5.9947 | 0.0000 | 0.0000 | 10.0825 |
| CLTCL1 | 0.4488 | 4.7567 | 5.9945 | 0.0000 | 0.0000 | 10.0814 |
| CDH10 | -0.7107 | 0.4701 | -5.9923 | 0.0000 | 0.0000 | 10.0694 |
| CBX5 | -0.2459 | 5.7291 | -5.9903 | 0.0000 | 0.0000 | 10.0582 |
| MMP13 | -0.5820 | 0.4254 | -5.9899 | 0.0000 | 0.0000 | 10.0565 |
| KRTAP1-1 | -0.5783 | 0.4818 | -5.9880 | 0.0000 | 0.0000 | 10.0459 |
| RPRM | -0.6237 | 0.5567 | -5.9810 | 0.0000 | 0.0000 | 10.0082 |
| NDUFAB1 | 0.1362 | 7.0767 | 5.9798 | 0.0000 | 0.0000 | 10.0013 |
| CDKN1B | 0.1225 | 6.7074 | 5.9792 | 0.0000 | 0.0000 | 9.9983 |
| FAM162A | 0.1739 | 6.8454 | 5.9788 | 0.0000 | 0.0000 | 9.9962 |
| ABCA5 | 0.3064 | 5.6046 | 5.9734 | 0.0000 | 0.0000 | 9.9670 |
| SLC2A9 | 0.8240 | 4.8747 | 5.9723 | 0.0000 | 0.0000 | 9.9610 |
| AZGP1 | 0.4184 | 7.5607 | 5.9695 | 0.0000 | 0.0000 | 9.9458 |
| BCL9 | -0.3347 | 5.5828 | -5.9681 | 0.0000 | 0.0000 | 9.9383 |
| MAOB | 0.3105 | 7.2964 | 5.9678 | 0.0000 | 0.0000 | 9.9363 |
| SPTB | -0.6028 | 1.6263 | -5.9673 | 0.0000 | 0.0000 | 9.9336 |
| POMP | 0.1195 | 7.2642 | 5.9664 | 0.0000 | 0.0000 | 9.9289 |
| KLHDC2 | 0.2297 | 6.1758 | 5.9654 | 0.0000 | 0.0000 | 9.9235 |
| CNTRL | -0.3729 | 4.5483 | -5.9652 | 0.0000 | 0.0000 | 9.9226 |
| COX5B | 0.1263 | 7.4615 | 5.9645 | 0.0000 | 0.0000 | 9.9186 |
| **RAB25** | -1.1559 | 2.0665 | -5.9644 | 0.0000 | 0.0000 | 9.9179 |
| PIGV | 0.2119 | 6.1239 | 5.9642 | 0.0000 | 0.0000 | 9.9169 |
| CAPN10 | -0.2182 | 5.1388 | -5.9637 | 0.0000 | 0.0000 | 9.9143 |
| DNA2 | -0.4583 | 4.2619 | -5.9635 | 0.0000 | 0.0000 | 9.9133 |
| NDE1 | -0.3694 | 4.6371 | -5.9597 | 0.0000 | 0.0000 | 9.8925 |
| RAD23A | 0.0846 | 7.0767 | 5.9583 | 0.0000 | 0.0000 | 9.8849 |
| **NEBL** | -1.0505 | 2.5510 | -5.9580 | 0.0000 | 0.0000 | 9.8833 |
| CCDC88C | -0.5517 | 3.9232 | -5.9565 | 0.0000 | 0.0000 | 9.8754 |

| RBM38 | -0.2157 | 6.2167 | -5.9559 | 0.0000 | 0.0000 | 9.8724 |
| --- | --- | --- | --- | --- | --- | --- |
| COX8A | 0.1016 | 7.7327 | 5.9549 | 0.0000 | 0.0000 | 9.8669 |
| PIK3R2 | -0.5116 | 3.0957 | -5.9543 | 0.0000 | 0.0000 | 9.8636 |
| FDXR | 0.2593 | 6.2856 | 5.9538 | 0.0000 | 0.0000 | 9.8611 |
| HMOX2 | 0.1671 | 6.6964 | 5.9537 | 0.0000 | 0.0000 | 9.8602 |
| E2F8 | -0.7697 | 3.9381 | -5.9523 | 0.0000 | 0.0000 | 9.8530 |
| LMO2 | 0.3074 | 5.5901 | 5.9501 | 0.0000 | 0.0000 | 9.8409 |
| NDUFB8 | 0.1623 | 6.8375 | 5.9479 | 0.0000 | 0.0000 | 9.8292 |
| COL16A1 | -0.8687 | 3.9379 | -5.9475 | 0.0000 | 0.0000 | 9.8267 |
| TMEM59 | 0.1030 | 7.0147 | 5.9433 | 0.0000 | 0.0000 | 9.8044 |
| NFS1 | 0.1342 | 6.1782 | 5.9428 | 0.0000 | 0.0000 | 9.8012 |
| ZNF589 | -0.3210 | 4.4425 | -5.9411 | 0.0000 | 0.0000 | 9.7922 |
| MYL12A | 0.1055 | 7.3068 | 5.9410 | 0.0000 | 0.0000 | 9.7917 |
| CNPY4 | -0.2260 | 5.5823 | -5.9357 | 0.0000 | 0.0000 | 9.7632 |
| SPR | 0.1275 | 7.2196 | 5.9350 | 0.0000 | 0.0000 | 9.7594 |
| COPZ2 | 0.3443 | 6.3534 | 5.9337 | 0.0000 | 0.0000 | 9.7525 |
| CLPP | 0.1345 | 6.8747 | 5.9327 | 0.0000 | 0.0000 | 9.7471 |
| PTTG1 | -0.4629 | 6.0477 | -5.9315 | 0.0000 | 0.0000 | 9.7405 |
| PDLIM1 | 0.1379 | 7.2878 | 5.9281 | 0.0000 | 0.0000 | 9.7223 |
| SLCO4A1 | -0.8818 | 2.7209 | -5.9275 | 0.0000 | 0.0000 | 9.7191 |
| LMX1B | -0.7708 | 0.7942 | -5.9270 | 0.0000 | 0.0000 | 9.7165 |
| MLF2 | 0.0990 | 7.2712 | 5.9262 | 0.0000 | 0.0000 | 9.7118 |
| L1CAM | -0.6337 | 1.0744 | -5.9256 | 0.0000 | 0.0000 | 9.7089 |
| SOX12 | -0.3382 | 5.9650 | -5.9254 | 0.0000 | 0.0000 | 9.7077 |
| LMOD1 | 0.5850 | 4.9769 | 5.9252 | 0.0000 | 0.0000 | 9.7068 |
| QPCT | -0.6634 | 4.1588 | -5.9249 | 0.0000 | 0.0000 | 9.7052 |
| **IGF2BP2** | -1.0472 | 4.7779 | -5.9238 | 0.0000 | 0.0000 | 9.6992 |
| CDC37L1 | 0.2132 | 6.1921 | 5.9232 | 0.0000 | 0.0000 | 9.6962 |
| PRKX | -0.6210 | 4.3395 | -5.9231 | 0.0000 | 0.0000 | 9.6956 |
| **CLDN10** | -1.1888 | 1.8738 | -5.9223 | 0.0000 | 0.0000 | 9.6910 |
| CBFB | -0.1798 | 5.9898 | -5.9218 | 0.0000 | 0.0000 | 9.6883 |
| PLCH1 | -0.7101 | 1.6765 | -5.9217 | 0.0000 | 0.0000 | 9.6881 |
| CALR | 0.0929 | 7.8950 | 5.9212 | 0.0000 | 0.0000 | 9.6850 |
| MACROD1 | 0.3043 | 6.4302 | 5.9191 | 0.0000 | 0.0000 | 9.6740 |
| LRCH3 | -0.1837 | 5.1355 | -5.9174 | 0.0000 | 0.0000 | 9.6650 |
| LAT2 | -0.4282 | 4.8233 | -5.9171 | 0.0000 | 0.0000 | 9.6633 |
| GINS2 | -0.4214 | 5.3316 | -5.9163 | 0.0000 | 0.0000 | 9.6591 |
| STOM | 0.1593 | 7.3552 | 5.9154 | 0.0000 | 0.0000 | 9.6539 |
| HPSE | -0.6118 | 3.4199 | -5.9145 | 0.0000 | 0.0000 | 9.6490 |
| RNASE2 | -0.9495 | 2.7486 | -5.9139 | 0.0000 | 0.0000 | 9.6460 |
| HAPLN1 | -0.6210 | 0.6873 | -5.9134 | 0.0000 | 0.0000 | 9.6432 |
| MMP16 | -0.5937 | 1.1252 | -5.9121 | 0.0000 | 0.0000 | 9.6364 |
| PON1 | 0.6358 | 7.0823 | 5.9116 | 0.0000 | 0.0000 | 9.6334 |
| TNFRSF21 | -0.4143 | 6.0762 | -5.9108 | 0.0000 | 0.0000 | 9.6296 |
| TSPAN15 | -0.3721 | 5.8327 | -5.9107 | 0.0000 | 0.0000 | 9.6287 |
| CLEC1A | 0.4693 | 3.5334 | 5.9105 | 0.0000 | 0.0000 | 9.6275 |
| ZMYM4 | -0.1817 | 5.7472 | -5.9063 | 0.0000 | 0.0000 | 9.6049 |
| PNPLA4 | 0.2130 | 6.0981 | 5.9049 | 0.0000 | 0.0000 | 9.5979 |
| ALDOA | -0.1445 | 7.2422 | -5.9043 | 0.0000 | 0.0000 | 9.5946 |
| APBA1 | 0.7162 | 4.4168 | 5.9020 | 0.0000 | 0.0000 | 9.5824 |
| IFNAR1 | 0.1181 | 6.6364 | 5.9013 | 0.0000 | 0.0000 | 9.5782 |
| CBX1 | -0.1388 | 6.4702 | -5.9010 | 0.0000 | 0.0000 | 9.5768 |
| SPA17 | -0.4677 | 3.9435 | -5.9010 | 0.0000 | 0.0000 | 9.5767 |

| NCK2 | -0.4100 | 5.9527 | -5.8987 | 0.0000 | 0.0000 | 9.5644 |
| --- | --- | --- | --- | --- | --- | --- |
| CTSZ | 0.1161 | 7.5501 | 5.8962 | 0.0000 | 0.0000 | 9.5511 |
| RCC1 | -0.1812 | 6.2249 | -5.8949 | 0.0000 | 0.0000 | 9.5443 |
| GCNT1 | -0.6284 | 3.0577 | -5.8947 | 0.0000 | 0.0000 | 9.5428 |
| ADCY9 | 0.2330 | 5.9056 | 5.8945 | 0.0000 | 0.0000 | 9.5421 |
| ATP2C2 | -0.6992 | 0.8824 | -5.8930 | 0.0000 | 0.0000 | 9.5341 |
| TGFBR3 | 0.4743 | 5.2584 | 5.8919 | 0.0000 | 0.0000 | 9.5280 |
| SSTR2 | -0.8629 | 3.2534 | -5.8899 | 0.0000 | 0.0000 | 9.5175 |
| TK2 | 0.1921 | 6.0109 | 5.8892 | 0.0000 | 0.0000 | 9.5138 |
| **CA4** | 1.0590 | 2.5243 | 5.8892 | 0.0000 | 0.0000 | 9.5137 |
| ZNF202 | -0.2247 | 4.8965 | -5.8889 | 0.0000 | 0.0000 | 9.5117 |
| HOXA11 | -0.8561 | 1.0464 | -5.8879 | 0.0000 | 0.0000 | 9.5069 |
| PROZ | 0.7127 | 6.1063 | 5.8875 | 0.0000 | 0.0000 | 9.5044 |
| IDI1 | 0.1739 | 7.0860 | 5.8870 | 0.0000 | 0.0000 | 9.5019 |
| **MMP12** | -1.1850 | 2.6165 | -5.8854 | 0.0000 | 0.0000 | 9.4930 |
| RAP1GAP2 | -0.8450 | 3.4076 | -5.8847 | 0.0000 | 0.0000 | 9.4894 |
| FN1 | 0.1210 | 7.6598 | 5.8841 | 0.0000 | 0.0000 | 9.4864 |
| ELAVL4 | -0.3078 | 0.5400 | -5.8837 | 0.0000 | 0.0000 | 9.4843 |
| **AQP8** | 1.2883 | 3.5365 | 5.8817 | 0.0000 | 0.0000 | 9.4736 |
| MTTP | 0.6970 | 6.6609 | 5.8811 | 0.0000 | 0.0000 | 9.4701 |
| FAM153A | -0.5484 | 0.7442 | -5.8798 | 0.0000 | 0.0000 | 9.4635 |
| ATP6V1G1 | 0.1034 | 7.2845 | 5.8786 | 0.0000 | 0.0000 | 9.4568 |
| MAST2 | -0.2006 | 5.8229 | -5.8780 | 0.0000 | 0.0000 | 9.4534 |
| PDLIM2 | 0.3235 | 4.8793 | 5.8757 | 0.0000 | 0.0000 | 9.4414 |
| TEF | 0.2482 | 6.2927 | 5.8753 | 0.0000 | 0.0000 | 9.4392 |
| MFSD6 | -0.4494 | 5.0075 | -5.8750 | 0.0000 | 0.0000 | 9.4376 |
| TEX264 | 0.1352 | 6.6698 | 5.8749 | 0.0000 | 0.0000 | 9.4370 |
| B3GNT4 | -0.4997 | 1.7140 | -5.8723 | 0.0000 | 0.0000 | 9.4230 |
| C4BPA | 0.4124 | 7.6486 | 5.8715 | 0.0000 | 0.0000 | 9.4188 |
| CALM1 | 0.0997 | 6.9209 | 5.8688 | 0.0000 | 0.0000 | 9.4045 |
| PHTF2 | -0.2857 | 4.8799 | -5.8680 | 0.0000 | 0.0000 | 9.4001 |
| C21orf62 | -0.3646 | 0.5568 | -5.8677 | 0.0000 | 0.0000 | 9.3987 |
| SLC44A4 | -0.7691 | 2.9430 | -5.8665 | 0.0000 | 0.0000 | 9.3922 |
| ATP1B3 | -0.2619 | 6.4113 | -5.8661 | 0.0000 | 0.0000 | 9.3904 |
| ISOC2 | 0.1685 | 6.9837 | 5.8656 | 0.0000 | 0.0000 | 9.3873 |
| NNT | 0.2485 | 6.6510 | 5.8647 | 0.0000 | 0.0000 | 9.3826 |
| ALG8 | 0.1237 | 6.6734 | 5.8640 | 0.0000 | 0.0000 | 9.3789 |
| ADIPOR2 | 0.1453 | 6.8436 | 5.8619 | 0.0000 | 0.0000 | 9.3678 |
| PEX16 | 0.1397 | 6.6637 | 5.8613 | 0.0000 | 0.0000 | 9.3643 |
| SLC28A3 | -0.7877 | 1.0048 | -5.8590 | 0.0000 | 0.0000 | 9.3522 |
| MT2A | 0.2775 | 7.5011 | 5.8560 | 0.0000 | 0.0000 | 9.3363 |
| GPR35 | -0.9708 | 3.3689 | -5.8560 | 0.0000 | 0.0000 | 9.3363 |
| QSOX1 | -0.5575 | 5.8256 | -5.8557 | 0.0000 | 0.0000 | 9.3346 |
| KLK11 | -0.7759 | 0.7203 | -5.8557 | 0.0000 | 0.0000 | 9.3344 |
| OGFRL1 | -0.3851 | 4.6442 | -5.8538 | 0.0000 | 0.0000 | 9.3245 |
| RABEPK | 0.2514 | 6.5233 | 5.8533 | 0.0000 | 0.0000 | 9.3218 |
| PASK | -0.4198 | 3.8760 | -5.8504 | 0.0000 | 0.0000 | 9.3066 |
| ABHD2 | 0.2199 | 6.7597 | 5.8501 | 0.0000 | 0.0000 | 9.3049 |
| GOLGA5 | 0.1196 | 6.7141 | 5.8491 | 0.0000 | 0.0000 | 9.2998 |
| POLR2E | 0.1083 | 7.0715 | 5.8484 | 0.0000 | 0.0000 | 9.2956 |
| BACE2 | -0.7605 | 4.6892 | -5.8475 | 0.0000 | 0.0000 | 9.2908 |
| BTBD3 | -0.2756 | 5.5211 | -5.8473 | 0.0000 | 0.0000 | 9.2899 |
| FOXD1 | -0.6050 | 0.6556 | -5.8461 | 0.0000 | 0.0000 | 9.2836 |

| SLC46A3 | 0.5914 | 6.0327 | 5.8449 | 0.0000 | 0.0000 | 9.2772 |
| --- | --- | --- | --- | --- | --- | --- |
| CEBPD | 0.2130 | 7.1087 | 5.8438 | 0.0000 | 0.0000 | 9.2716 |
| LMO7 | 0.2961 | 5.7796 | 5.8426 | 0.0000 | 0.0000 | 9.2648 |
| AP3S1 | 0.1286 | 6.6738 | 5.8413 | 0.0000 | 0.0000 | 9.2582 |
| **CFTR** | -1.1367 | 1.7074 | -5.8405 | 0.0000 | 0.0000 | 9.2538 |
| REEP2 | -0.7875 | 2.6631 | -5.8372 | 0.0000 | 0.0000 | 9.2363 |
| CLCNKA | -0.8744 | 2.1822 | -5.8364 | 0.0000 | 0.0000 | 9.2318 |
| APBB3 | 0.3003 | 5.7914 | 5.8356 | 0.0000 | 0.0000 | 9.2279 |
| AQP7 | 0.5101 | 5.0841 | 5.8350 | 0.0000 | 0.0000 | 9.2245 |
| UBE2I | -0.1268 | 6.1443 | -5.8340 | 0.0000 | 0.0000 | 9.2190 |
| CDKN3 | -0.4893 | 5.6943 | -5.8339 | 0.0000 | 0.0000 | 9.2190 |
| TMCC2 | -0.5885 | 2.3612 | -5.8338 | 0.0000 | 0.0000 | 9.2181 |
| PLEK2 | -0.8979 | 5.1522 | -5.8337 | 0.0000 | 0.0000 | 9.2180 |
| CEP97 | -0.3917 | 3.8609 | -5.8337 | 0.0000 | 0.0000 | 9.2178 |
| GDPD3 | -0.4988 | 4.0665 | -5.8332 | 0.0000 | 0.0000 | 9.2149 |
| SPAG8 | -0.4214 | 2.6466 | -5.8329 | 0.0000 | 0.0000 | 9.2134 |
| REEP4 | -0.1898 | 6.1518 | -5.8322 | 0.0000 | 0.0000 | 9.2099 |
| GAP43 | -0.7798 | 1.4802 | -5.8319 | 0.0000 | 0.0000 | 9.2083 |
| DMRT1 | -0.2537 | 0.1443 | -5.8316 | 0.0000 | 0.0000 | 9.2067 |
| ATPIF1 | 0.1283 | 7.1965 | 5.8312 | 0.0000 | 0.0000 | 9.2046 |
| TFG | 0.0818 | 6.9624 | 5.8305 | 0.0000 | 0.0000 | 9.2008 |
| COL8A2 | -0.8632 | 2.9929 | -5.8300 | 0.0000 | 0.0000 | 9.1979 |
| RNLS | 0.4233 | 5.2818 | 5.8299 | 0.0000 | 0.0000 | 9.1976 |
| EHBP1L1 | -0.2055 | 5.9655 | -5.8297 | 0.0000 | 0.0000 | 9.1964 |
| ZNF8 | -0.3552 | 3.7433 | -5.8275 | 0.0000 | 0.0000 | 9.1847 |
| ERN2 | -0.5348 | 0.4616 | -5.8266 | 0.0000 | 0.0000 | 9.1800 |
| BMPR1B | -0.6603 | 1.0067 | -5.8254 | 0.0000 | 0.0000 | 9.1738 |
| STIM1 | 0.1339 | 6.6127 | 5.8247 | 0.0000 | 0.0000 | 9.1699 |
| EDNRB | 0.4017 | 5.6933 | 5.8236 | 0.0000 | 0.0000 | 9.1642 |
| ATF6 | 0.1584 | 6.4939 | 5.8232 | 0.0000 | 0.0000 | 9.1621 |
| CCDC25 | 0.1942 | 6.2209 | 5.8227 | 0.0000 | 0.0000 | 9.1595 |
| RNF13 | 0.1247 | 6.6020 | 5.8226 | 0.0000 | 0.0000 | 9.1589 |
| FMR1 | 0.2116 | 6.1475 | 5.8155 | 0.0000 | 0.0000 | 9.1211 |
| PRDM11 | -0.4273 | 3.1984 | -5.8137 | 0.0000 | 0.0000 | 9.1114 |
| GLS | -0.4491 | 5.5799 | -5.8131 | 0.0000 | 0.0000 | 9.1085 |
| IDH1 | 0.1261 | 7.2828 | 5.8122 | 0.0000 | 0.0000 | 9.1037 |
| ITPKA | -0.6832 | 5.3638 | -5.8118 | 0.0000 | 0.0000 | 9.1014 |
| CEP250 | -0.2394 | 5.0921 | -5.8113 | 0.0000 | 0.0000 | 9.0989 |
| GTPBP4 | -0.1393 | 6.1169 | -5.8106 | 0.0000 | 0.0000 | 9.0954 |
| HNRNPH2 | 0.0927 | 7.0409 | 5.8096 | 0.0000 | 0.0000 | 9.0899 |
| CALCRL | 0.4896 | 5.3161 | 5.8075 | 0.0000 | 0.0000 | 9.0787 |
| APLNR | 0.5898 | 5.6984 | 5.8064 | 0.0000 | 0.0000 | 9.0732 |
| NASP | -0.1606 | 6.2985 | -5.8057 | 0.0000 | 0.0000 | 9.0695 |
| MOSPD3 | 0.1570 | 6.6814 | 5.8052 | 0.0000 | 0.0000 | 9.0669 |
| ZKSCAN1 | 0.1703 | 6.5013 | 5.8051 | 0.0000 | 0.0000 | 9.0662 |
| UGT1A1 | 0.9231 | 5.8745 | 5.8050 | 0.0000 | 0.0000 | 9.0657 |
| ADAMTS5 | -0.5738 | 3.5751 | -5.8047 | 0.0000 | 0.0000 | 9.0642 |
| **ANXA13** | -1.0608 | 5.0895 | -5.8044 | 0.0000 | 0.0000 | 9.0626 |
| SMYD5 | -0.1540 | 6.2570 | -5.8011 | 0.0000 | 0.0000 | 9.0450 |
| TULP1 | -0.2827 | 0.2882 | -5.7998 | 0.0000 | 0.0000 | 9.0380 |
| TYMS | -0.3240 | 6.1490 | -5.7995 | 0.0000 | 0.0000 | 9.0365 |
| GRIP1 | -0.5855 | 0.8792 | -5.7969 | 0.0000 | 0.0000 | 9.0230 |
| DCTN5 | -0.1712 | 5.4895 | -5.7932 | 0.0000 | 0.0000 | 9.0035 |

| DDX5 | 0.0783 | 7.1746 | 5.7930 | 0.0000 | 0.0000 | 9.0022 |
| --- | --- | --- | --- | --- | --- | --- |
| NTN1 | 0.7025 | 5.0241 | 5.7908 | 0.0000 | 0.0000 | 8.9907 |
| TTLL4 | -0.3832 | 5.4075 | -5.7907 | 0.0000 | 0.0000 | 8.9902 |
| IL1B | -0.7294 | 2.9858 | -5.7906 | 0.0000 | 0.0000 | 8.9897 |
| NCAPD3 | -0.3103 | 4.9879 | -5.7883 | 0.0000 | 0.0000 | 8.9775 |
| MATN1 | -0.3180 | 0.7032 | -5.7876 | 0.0000 | 0.0000 | 8.9738 |
| ASPM | -0.6147 | 4.8329 | -5.7856 | 0.0000 | 0.0000 | 8.9631 |
| NSMCE4A | 0.1354 | 6.2764 | 5.7851 | 0.0000 | 0.0000 | 8.9607 |
| LRRC2 | 0.6805 | 4.8759 | 5.7843 | 0.0000 | 0.0000 | 8.9566 |
| SLC25A21 | -0.5281 | 1.1300 | -5.7837 | 0.0000 | 0.0000 | 8.9530 |
| RHOA | 0.0645 | 7.5061 | 5.7836 | 0.0000 | 0.0000 | 8.9525 |
| C2CD3 | -0.2746 | 4.5356 | -5.7820 | 0.0000 | 0.0000 | 8.9443 |
| AADAC | 0.4966 | 7.3526 | 5.7815 | 0.0000 | 0.0000 | 8.9415 |
| USP1 | -0.1882 | 6.0369 | -5.7801 | 0.0000 | 0.0000 | 8.9345 |
| **ESRP1** | -1.2456 | 1.7934 | -5.7780 | 0.0000 | 0.0000 | 8.9231 |
| BMP8B | -0.9711 | 3.1877 | -5.7778 | 0.0000 | 0.0000 | 8.9220 |
| SHOX2 | -0.6419 | 1.2599 | -5.7773 | 0.0000 | 0.0000 | 8.9194 |
| TRPC7 | -0.3057 | 0.2458 | -5.7762 | 0.0000 | 0.0000 | 8.9135 |
| ENTPD2 | -0.6685 | 4.6198 | -5.7759 | 0.0000 | 0.0000 | 8.9119 |
| ADAM22 | -0.7073 | 1.8735 | -5.7758 | 0.0000 | 0.0000 | 8.9116 |
| CD1A | -0.7204 | 1.3859 | -5.7750 | 0.0000 | 0.0000 | 8.9071 |
| TNFAIP8 | -0.4857 | 4.2604 | -5.7732 | 0.0000 | 0.0000 | 8.8980 |
| TRIAP1 | 0.1055 | 6.9161 | 5.7731 | 0.0000 | 0.0000 | 8.8973 |
| NAGA | 0.1620 | 6.6934 | 5.7725 | 0.0000 | 0.0000 | 8.8944 |
| MYL6 | 0.0934 | 7.5017 | 5.7721 | 0.0000 | 0.0000 | 8.8921 |
| HMGA1 | -0.1972 | 6.9628 | -5.7720 | 0.0000 | 0.0000 | 8.8918 |
| DMPK | -0.2844 | 5.3241 | -5.7714 | 0.0000 | 0.0000 | 8.8883 |
| MMADHC | 0.1029 | 7.0284 | 5.7705 | 0.0000 | 0.0000 | 8.8836 |
| RFX3 | -0.3971 | 3.2868 | -5.7701 | 0.0000 | 0.0000 | 8.8815 |
| TINF2 | 0.0991 | 6.5721 | 5.7693 | 0.0000 | 0.0000 | 8.8775 |
| PRDX2 | 0.1138 | 7.4265 | 5.7640 | 0.0000 | 0.0000 | 8.8494 |
| SMPD1 | 0.1434 | 6.9515 | 5.7638 | 0.0000 | 0.0000 | 8.8487 |
| THRB | 0.2902 | 5.7126 | 5.7604 | 0.0000 | 0.0000 | 8.8306 |
| EDA | -0.8391 | 3.5080 | -5.7596 | 0.0000 | 0.0000 | 8.8264 |
| PITPNM3 | -0.6297 | 1.4078 | -5.7591 | 0.0000 | 0.0000 | 8.8239 |
| HMGCS2 | 0.3999 | 7.6921 | 5.7582 | 0.0000 | 0.0000 | 8.8190 |
| VTI1B | 0.1196 | 6.4388 | 5.7576 | 0.0000 | 0.0000 | 8.8161 |
| FGF9 | -0.4824 | 0.4814 | -5.7574 | 0.0000 | 0.0000 | 8.8149 |
| KCNQ1 | -0.4907 | 4.7135 | -5.7571 | 0.0000 | 0.0000 | 8.8133 |
| TCOF1 | -0.1997 | 5.7733 | -5.7563 | 0.0000 | 0.0000 | 8.8092 |
| CDX2 | -0.6778 | 0.6984 | -5.7552 | 0.0000 | 0.0000 | 8.8030 |
| ZDHHC7 | -0.1651 | 6.1093 | -5.7534 | 0.0000 | 0.0000 | 8.7940 |
| MICALL1 | -0.1751 | 5.7652 | -5.7520 | 0.0000 | 0.0000 | 8.7867 |
| IFNGR1 | 0.1738 | 6.9248 | 5.7519 | 0.0000 | 0.0000 | 8.7859 |
| TBX3 | 0.6455 | 5.8775 | 5.7511 | 0.0000 | 0.0000 | 8.7815 |
| HOXD11 | -0.5304 | 0.3606 | -5.7511 | 0.0000 | 0.0000 | 8.7815 |
| TRIM17 | -0.8704 | 1.7070 | -5.7510 | 0.0000 | 0.0000 | 8.7811 |
| DLK2 | -0.5261 | 3.9476 | -5.7505 | 0.0000 | 0.0000 | 8.7785 |
| SNPH | -0.6339 | 2.6352 | -5.7505 | 0.0000 | 0.0000 | 8.7784 |
| GNPDA1 | -0.1833 | 6.2134 | -5.7501 | 0.0000 | 0.0000 | 8.7767 |
| PITX3 | -0.3253 | 0.3591 | -5.7486 | 0.0000 | 0.0000 | 8.7685 |
| FAM105A | -0.6058 | 3.8473 | -5.7460 | 0.0000 | 0.0000 | 8.7551 |
| PPM1A | 0.1670 | 6.0209 | 5.7434 | 0.0000 | 0.0000 | 8.7412 |

| NUDT11 | -0.6882 | 1.0766 | -5.7418 | 0.0000 | 0.0000 | 8.7330 |
| --- | --- | --- | --- | --- | --- | --- |
| ESYT1 | 0.1070 | 6.9323 | 5.7417 | 0.0000 | 0.0000 | 8.7327 |
| F11R | 0.1809 | 6.9894 | 5.7414 | 0.0000 | 0.0000 | 8.7307 |
| ESPN | 0.4198 | 6.6852 | 5.7379 | 0.0000 | 0.0000 | 8.7124 |
| SLC34A1 | 0.7098 | 2.0136 | 5.7363 | 0.0000 | 0.0000 | 8.7042 |
| CREB5 | -0.5976 | 2.8571 | -5.7359 | 0.0000 | 0.0000 | 8.7022 |
| EIF6 | 0.1078 | 7.4518 | 5.7355 | 0.0000 | 0.0000 | 8.7002 |
| HSPA14 | -0.1619 | 5.6811 | -5.7353 | 0.0000 | 0.0000 | 8.6988 |
| ADAMTS6 | -0.6090 | 1.7708 | -5.7340 | 0.0000 | 0.0000 | 8.6920 |
| CBX7 | 0.2566 | 5.7238 | 5.7335 | 0.0000 | 0.0000 | 8.6896 |
| PSMA7 | 0.0952 | 7.4299 | 5.7332 | 0.0000 | 0.0000 | 8.6881 |
| ACACB | 0.3384 | 5.9768 | 5.7316 | 0.0000 | 0.0000 | 8.6795 |
| PRR7 | -0.5333 | 4.7779 | -5.7304 | 0.0000 | 0.0000 | 8.6734 |
| PTPN13 | -0.7626 | 2.1200 | -5.7292 | 0.0000 | 0.0000 | 8.6668 |
| FAIM | -0.2968 | 5.2116 | -5.7291 | 0.0000 | 0.0000 | 8.6663 |
| SLC19A2 | 0.2459 | 6.2729 | 5.7285 | 0.0000 | 0.0000 | 8.6635 |
| ARF1 | 0.0793 | 7.5682 | 5.7282 | 0.0000 | 0.0000 | 8.6620 |
| SERPINA10 | 0.4595 | 6.9856 | 5.7280 | 0.0000 | 0.0000 | 8.6606 |
| AKR7A2 | 0.1357 | 6.9797 | 5.7272 | 0.0000 | 0.0000 | 8.6568 |
| CAPG | -0.4367 | 6.2196 | -5.7257 | 0.0000 | 0.0000 | 8.6488 |
| LRRFIP2 | 0.1309 | 6.0345 | 5.7252 | 0.0000 | 0.0000 | 8.6462 |
| MYBPC2 | -0.5619 | 0.7810 | -5.7243 | 0.0000 | 0.0000 | 8.6414 |
| SEC61B | 0.1098 | 7.4638 | 5.7241 | 0.0000 | 0.0000 | 8.6405 |
| RNF219 | -0.2736 | 4.8953 | -5.7233 | 0.0000 | 0.0000 | 8.6362 |
| MGAT1 | 0.0959 | 7.0913 | 5.7223 | 0.0000 | 0.0000 | 8.6309 |
| CUX2 | 0.9062 | 5.2841 | 5.7208 | 0.0000 | 0.0000 | 8.6228 |
| CKMT1A | -0.8525 | 1.2156 | -5.7207 | 0.0000 | 0.0000 | 8.6227 |
| UBE2D3 | 0.0958 | 6.7597 | 5.7207 | 0.0000 | 0.0000 | 8.6225 |
| CBR4 | 0.2606 | 6.0399 | 5.7188 | 0.0000 | 0.0000 | 8.6125 |
| SYK | -0.5113 | 4.6873 | -5.7184 | 0.0000 | 0.0000 | 8.6105 |
| NDUFB7 | 0.1207 | 7.6344 | 5.7180 | 0.0000 | 0.0000 | 8.6085 |
| PHB2 | 0.0981 | 7.3607 | 5.7177 | 0.0000 | 0.0000 | 8.6068 |
| CAMLG | 0.1227 | 6.6620 | 5.7177 | 0.0000 | 0.0000 | 8.6067 |
| CDC25B | -0.2077 | 6.4943 | -5.7176 | 0.0000 | 0.0000 | 8.6065 |
| ADM2 | -0.6291 | 5.4251 | -5.7156 | 0.0000 | 0.0000 | 8.5960 |
| RASSF8 | -0.5128 | 4.4003 | -5.7140 | 0.0000 | 0.0000 | 8.5876 |
| ATG4A | 0.1673 | 6.1671 | 5.7135 | 0.0000 | 0.0000 | 8.5851 |
| ATP6V0A4 | -0.3524 | 0.3339 | -5.7128 | 0.0000 | 0.0000 | 8.5811 |
| SP4 | -0.3585 | 4.2404 | -5.7115 | 0.0000 | 0.0000 | 8.5743 |
| UNC13A | -0.7140 | 1.1521 | -5.7107 | 0.0000 | 0.0000 | 8.5706 |
| KCNS1 | -0.6385 | 0.7942 | -5.7106 | 0.0000 | 0.0000 | 8.5698 |
| TNFSF9 | -0.8779 | 3.0496 | -5.7087 | 0.0000 | 0.0000 | 8.5602 |
| GRPEL1 | 0.1438 | 6.5728 | 5.7084 | 0.0000 | 0.0000 | 8.5582 |
| ZNF14 | -0.6790 | 3.8720 | -5.7077 | 0.0000 | 0.0000 | 8.5549 |
| PDHB | 0.1180 | 6.6965 | 5.7071 | 0.0000 | 0.0000 | 8.5514 |
| ZNF84 | -0.2386 | 5.0252 | -5.7051 | 0.0000 | 0.0000 | 8.5410 |
| SYCE1L | -0.6223 | 3.2506 | -5.7043 | 0.0000 | 0.0000 | 8.5369 |
| CUTA | 0.1186 | 7.4420 | 5.7036 | 0.0000 | 0.0000 | 8.5334 |
| AGAP1 | -0.3086 | 4.7994 | -5.7032 | 0.0000 | 0.0000 | 8.5314 |
| DOK3 | -0.4203 | 4.2283 | -5.7029 | 0.0000 | 0.0000 | 8.5296 |
| C10orf10 | 0.2281 | 7.1455 | 5.7024 | 0.0000 | 0.0000 | 8.5273 |
| MT1X | 0.4993 | 6.7795 | 5.7024 | 0.0000 | 0.0000 | 8.5272 |
| IGF1R | -0.7186 | 3.0906 | -5.7007 | 0.0000 | 0.0000 | 8.5181 |

| PLXNB3 | -0.8904 | 2.4053 | -5.6953 | 0.0000 | 0.0000 | 8.4901 |
| --- | --- | --- | --- | --- | --- | --- |
| **PRSS12** | -1.0089 | 2.0913 | -5.6943 | 0.0000 | 0.0000 | 8.4850 |
| PLEKHA2 | -0.3779 | 5.0488 | -5.6927 | 0.0000 | 0.0000 | 8.4764 |
| CYP3A5 | 0.4398 | 6.8327 | 5.6925 | 0.0000 | 0.0000 | 8.4755 |
| KIAA1614 | -0.5522 | 2.5982 | -5.6923 | 0.0000 | 0.0000 | 8.4747 |
| CREB3L1 | -0.8697 | 3.8785 | -5.6911 | 0.0000 | 0.0000 | 8.4684 |
| MCM5 | -0.2185 | 6.2690 | -5.6907 | 0.0000 | 0.0000 | 8.4664 |
| MBL2 | 0.7959 | 6.2473 | 5.6902 | 0.0000 | 0.0000 | 8.4637 |
| ANO1 | 0.6668 | 5.8250 | 5.6902 | 0.0000 | 0.0000 | 8.4635 |
| PRDX1 | 0.1039 | 7.6746 | 5.6881 | 0.0000 | 0.0000 | 8.4525 |
| DSC3 | -0.5822 | 0.6279 | -5.6873 | 0.0000 | 0.0000 | 8.4487 |
| PHIP | -0.2790 | 5.0997 | -5.6871 | 0.0000 | 0.0000 | 8.4475 |
| MERTK | 0.4225 | 5.6044 | 5.6861 | 0.0000 | 0.0000 | 8.4421 |
| CAD | -0.2212 | 5.8389 | -5.6856 | 0.0000 | 0.0000 | 8.4398 |
| NMT2 | 0.2309 | 6.1081 | 5.6856 | 0.0000 | 0.0000 | 8.4396 |
| CTR9 | 0.1505 | 6.4850 | 5.6808 | 0.0000 | 0.0000 | 8.4148 |
| TMEM5 | -0.1681 | 5.1471 | -5.6805 | 0.0000 | 0.0000 | 8.4131 |
| TGM1 | -0.4708 | 2.1487 | -5.6799 | 0.0000 | 0.0000 | 8.4101 |
| GAL3ST4 | -0.6702 | 3.8263 | -5.6795 | 0.0000 | 0.0000 | 8.4080 |
| NUDT6 | 0.5324 | 3.9568 | 5.6792 | 0.0000 | 0.0000 | 8.4064 |
| SLC17A2 | 0.7396 | 6.1751 | 5.6783 | 0.0000 | 0.0000 | 8.4020 |
| SMAGP | -0.3366 | 5.4467 | -5.6779 | 0.0000 | 0.0000 | 8.3999 |
| TMEM204 | 0.2790 | 6.0812 | 5.6725 | 0.0000 | 0.0000 | 8.3716 |
| RAP2C | 0.1663 | 6.5103 | 5.6723 | 0.0000 | 0.0000 | 8.3707 |
| SDS | 0.7901 | 6.5953 | 5.6703 | 0.0000 | 0.0000 | 8.3604 |
| MIS18A | -0.2204 | 5.9068 | -5.6689 | 0.0000 | 0.0000 | 8.3533 |
| DNPEP | 0.1040 | 6.7028 | 5.6673 | 0.0000 | 0.0000 | 8.3445 |
| MSH3 | 0.1901 | 5.7291 | 5.6663 | 0.0000 | 0.0000 | 8.3396 |
| KLF5 | -0.8436 | 4.5286 | -5.6659 | 0.0000 | 0.0000 | 8.3376 |
| SMARCD2 | 0.1115 | 6.8756 | 5.6645 | 0.0000 | 0.0000 | 8.3301 |
| COQ9 | 0.1686 | 6.6258 | 5.6638 | 0.0000 | 0.0000 | 8.3267 |
| LOX | -0.6099 | 4.5297 | -5.6637 | 0.0000 | 0.0000 | 8.3261 |
| HSP90AB1 | 0.0819 | 7.7905 | 5.6625 | 0.0000 | 0.0000 | 8.3200 |
| E2F5 | -0.4310 | 4.4028 | -5.6620 | 0.0000 | 0.0000 | 8.3171 |
| KDM1A | -0.1193 | 6.2662 | -5.6611 | 0.0000 | 0.0000 | 8.3125 |
| SCARB2 | 0.0982 | 7.1258 | 5.6563 | 0.0000 | 0.0000 | 8.2880 |
| ASB13 | 0.1996 | 6.6748 | 5.6562 | 0.0000 | 0.0000 | 8.2873 |
| LGALS9 | -0.3401 | 5.8310 | -5.6562 | 0.0000 | 0.0000 | 8.2872 |
| ZNF430 | -0.6199 | 2.9955 | -5.6556 | 0.0000 | 0.0000 | 8.2841 |
| MET | 0.2002 | 6.7084 | 5.6530 | 0.0000 | 0.0000 | 8.2707 |
| GAS7 | -0.5986 | 3.7265 | -5.6505 | 0.0000 | 0.0000 | 8.2580 |
| APOC2 | 0.4904 | 7.0009 | 5.6497 | 0.0000 | 0.0000 | 8.2538 |
| N4BP3 | -0.5864 | 3.1543 | -5.6470 | 0.0000 | 0.0000 | 8.2399 |
| KLK1 | -0.5590 | 0.7569 | -5.6461 | 0.0000 | 0.0000 | 8.2353 |
| GOT1 | 0.1918 | 7.4067 | 5.6461 | 0.0000 | 0.0000 | 8.2351 |
| NUBPL | 0.3397 | 4.9570 | 5.6458 | 0.0000 | 0.0000 | 8.2335 |
| ALDH1B1 | 0.1990 | 7.0382 | 5.6457 | 0.0000 | 0.0000 | 8.2331 |
| OSM | -0.7089 | 2.8635 | -5.6457 | 0.0000 | 0.0000 | 8.2327 |
| VEZF1 | -0.1496 | 6.0253 | -5.6443 | 0.0000 | 0.0000 | 8.2259 |
| PSD3 | 0.4953 | 4.9661 | 5.6432 | 0.0000 | 0.0000 | 8.2202 |
| CPB2 | 0.3605 | 7.6272 | 5.6419 | 0.0000 | 0.0000 | 8.2133 |
| DHPS | 0.1116 | 6.7461 | 5.6386 | 0.0000 | 0.0000 | 8.1962 |
| CRISP2 | -0.7722 | 0.6325 | -5.6335 | 0.0000 | 0.0000 | 8.1700 |

| COX15 | 0.1117 | 6.4322 | 5.6322 | 0.0000 | 0.0000 | 8.1633 |
| --- | --- | --- | --- | --- | --- | --- |
| FBXO46 | -0.1790 | 6.0060 | -5.6310 | 0.0000 | 0.0000 | 8.1572 |
| **PAGE4** | 1.5322 | 2.5790 | 5.6295 | 0.0000 | 0.0000 | 8.1494 |
| SDC4 | 0.1383 | 7.5304 | 5.6293 | 0.0000 | 0.0000 | 8.1484 |
| **SUSD4** | -1.2285 | 4.3315 | -5.6278 | 0.0000 | 0.0000 | 8.1405 |
| ADAMTS9 | -0.4502 | 4.5308 | -5.6277 | 0.0000 | 0.0000 | 8.1399 |
| BTF3 | 0.0929 | 7.4724 | 5.6266 | 0.0000 | 0.0000 | 8.1343 |
| FEM1C | 0.1888 | 5.9612 | 5.6266 | 0.0000 | 0.0000 | 8.1342 |
| TRPA1 | -0.5088 | 0.6490 | -5.6258 | 0.0000 | 0.0000 | 8.1303 |
| TXNDC15 | 0.1117 | 6.3488 | 5.6250 | 0.0000 | 0.0000 | 8.1264 |
| **NRCAM** | -1.1366 | 3.0987 | -5.6234 | 0.0000 | 0.0000 | 8.1180 |
| PPM1H | -0.7373 | 4.4462 | -5.6233 | 0.0000 | 0.0000 | 8.1174 |
| SEC14L3 | 0.9616 | 2.1572 | 5.6218 | 0.0000 | 0.0000 | 8.1099 |
| MX2 | -0.6383 | 3.7166 | -5.6213 | 0.0000 | 0.0000 | 8.1072 |
| IL12A | -0.6295 | 1.8863 | -5.6211 | 0.0000 | 0.0000 | 8.1059 |
| AP1S1 | 0.1115 | 7.0583 | 5.6209 | 0.0000 | 0.0000 | 8.1051 |
| ACPP | -0.7322 | 1.1645 | -5.6171 | 0.0000 | 0.0000 | 8.0853 |
| ZNF280B | -0.6031 | 1.0218 | -5.6170 | 0.0000 | 0.0000 | 8.0851 |
| FKBP10 | -0.4485 | 5.6719 | -5.6159 | 0.0000 | 0.0000 | 8.0792 |
| TMED10 | 0.0954 | 7.1927 | 5.6153 | 0.0000 | 0.0000 | 8.0760 |
| CHRND | -0.7097 | 0.7801 | -5.6151 | 0.0000 | 0.0000 | 8.0750 |
| CADPS | -0.6836 | 0.6931 | -5.6138 | 0.0000 | 0.0000 | 8.0687 |
| DHX34 | -0.1815 | 5.9213 | -5.6111 | 0.0000 | 0.0000 | 8.0546 |
| HSP90AA1 | 0.0896 | 7.5478 | 5.6101 | 0.0000 | 0.0000 | 8.0495 |
| ACSS3 | 0.6708 | 5.6500 | 5.6100 | 0.0000 | 0.0000 | 8.0490 |
| GNG7 | 0.4946 | 5.1301 | 5.6078 | 0.0000 | 0.0000 | 8.0376 |
| SLC6A15 | -0.6190 | 0.3649 | -5.6077 | 0.0000 | 0.0000 | 8.0372 |
| INSRR | -0.5338 | 0.7375 | -5.6064 | 0.0000 | 0.0000 | 8.0308 |
| UNC119 | -0.2923 | 5.7658 | -5.6063 | 0.0000 | 0.0000 | 8.0300 |
| FXYD6 | 0.4244 | 5.1491 | 5.6025 | 0.0000 | 0.0000 | 8.0103 |
| ADAP1 | -0.7464 | 3.4568 | -5.6015 | 0.0000 | 0.0000 | 8.0054 |
| CCL7 | -0.5712 | 0.5795 | -5.6007 | 0.0000 | 0.0000 | 8.0012 |
| WRAP53 | -0.2427 | 5.0441 | -5.6005 | 0.0000 | 0.0000 | 8.0002 |
| CNOT3 | -0.1274 | 6.0220 | -5.5999 | 0.0000 | 0.0000 | 7.9970 |
| VPS37B | -0.2680 | 5.2877 | -5.5986 | 0.0000 | 0.0000 | 7.9907 |
| PTPRE | -0.5304 | 3.8786 | -5.5979 | 0.0000 | 0.0000 | 7.9869 |
| FN3K | 0.1894 | 6.8034 | 5.5966 | 0.0000 | 0.0000 | 7.9800 |
| PLCB1 | -0.6342 | 4.2898 | -5.5960 | 0.0000 | 0.0000 | 7.9772 |
| DIO1 | 0.6299 | 6.7166 | 5.5954 | 0.0000 | 0.0000 | 7.9742 |
| KCND2 | -0.6219 | 0.9377 | -5.5943 | 0.0000 | 0.0000 | 7.9683 |
| RGS19 | -0.2676 | 5.8133 | -5.5926 | 0.0000 | 0.0000 | 7.9597 |
| LRRN2 | -0.7906 | 3.3638 | -5.5909 | 0.0000 | 0.0000 | 7.9512 |
| INTS8 | -0.2046 | 5.6473 | -5.5898 | 0.0000 | 0.0000 | 7.9454 |
| FOXN1 | -0.3935 | 0.3517 | -5.5850 | 0.0000 | 0.0000 | 7.9207 |
| APOL1 | 0.1865 | 7.1948 | 5.5831 | 0.0000 | 0.0000 | 7.9113 |
| RAD1 | -0.1630 | 5.5020 | -5.5823 | 0.0000 | 0.0000 | 7.9070 |
| CACNA1D | -0.6807 | 3.4727 | -5.5822 | 0.0000 | 0.0000 | 7.9065 |
| BAIAP2L2 | -0.6104 | 5.4805 | -5.5821 | 0.0000 | 0.0000 | 7.9062 |
| B3GALT2 | -0.7766 | 1.9316 | -5.5820 | 0.0000 | 0.0000 | 7.9054 |
| MICALL2 | -0.3255 | 5.2980 | -5.5818 | 0.0000 | 0.0000 | 7.9047 |
| TCF25 | 0.1321 | 6.5392 | 5.5817 | 0.0000 | 0.0000 | 7.9041 |
| AMBP | 0.2651 | 8.1659 | 5.5802 | 0.0000 | 0.0000 | 7.8964 |
| NDUFS1 | 0.1282 | 6.4230 | 5.5794 | 0.0000 | 0.0000 | 7.8922 |

| NSUN6 | 0.2139 | 6.1456 | 5.5788 | 0.0000 | 0.0000 | 7.8890 |
| --- | --- | --- | --- | --- | --- | --- |
| NCOA2 | 0.2163 | 6.2070 | 5.5787 | 0.0000 | 0.0000 | 7.8883 |
| SPP1 | -0.7149 | 6.8045 | -5.5783 | 0.0000 | 0.0000 | 7.8866 |
| PYCR1 | -0.9228 | 5.2083 | -5.5775 | 0.0000 | 0.0000 | 7.8823 |
| EHF | -0.9209 | 4.0704 | -5.5774 | 0.0000 | 0.0000 | 7.8821 |
| CDKL2 | -0.3065 | 0.3359 | -5.5772 | 0.0000 | 0.0000 | 7.8807 |
| CDS2 | 0.1552 | 6.0044 | 5.5757 | 0.0000 | 0.0000 | 7.8732 |
| SLC7A6 | -0.5084 | 3.7327 | -5.5749 | 0.0000 | 0.0000 | 7.8692 |
| INPP4A | -0.2774 | 4.5256 | -5.5716 | 0.0000 | 0.0000 | 7.8522 |
| MPDU1 | 0.1395 | 6.8896 | 5.5716 | 0.0000 | 0.0000 | 7.8521 |
| KSR1 | -0.4796 | 3.9514 | -5.5711 | 0.0000 | 0.0000 | 7.8499 |
| MGEA5 | 0.1036 | 6.5627 | 5.5700 | 0.0000 | 0.0000 | 7.8443 |
| PSMD4 | 0.1121 | 7.3900 | 5.5669 | 0.0000 | 0.0000 | 7.8284 |
| ZNF277 | 0.1610 | 6.1251 | 5.5669 | 0.0000 | 0.0000 | 7.8282 |
| FAM198B | 0.4017 | 5.0572 | 5.5661 | 0.0000 | 0.0000 | 7.8240 |
| SLC12A2 | -0.5579 | 4.8777 | -5.5653 | 0.0000 | 0.0000 | 7.8201 |
| TPP1 | 0.1005 | 7.2044 | 5.5646 | 0.0000 | 0.0000 | 7.8165 |
| SLC22A2 | 0.5087 | 0.7751 | 5.5592 | 0.0000 | 0.0000 | 7.7890 |
| TMEM176A | 0.2502 | 7.5917 | 5.5591 | 0.0000 | 0.0000 | 7.7882 |
| ZNF230 | -0.3426 | 3.6585 | -5.5570 | 0.0000 | 0.0000 | 7.7780 |
| SCN3B | -0.3889 | 0.6143 | -5.5561 | 0.0000 | 0.0000 | 7.7731 |
| CYP2C8 | 0.6490 | 6.9147 | 5.5558 | 0.0000 | 0.0000 | 7.7717 |
| SRP9 | 0.0860 | 7.3960 | 5.5537 | 0.0000 | 0.0000 | 7.7611 |
| CUL1 | 0.1009 | 6.8269 | 5.5528 | 0.0000 | 0.0000 | 7.7564 |
| PSMB4 | 0.0930 | 7.5533 | 5.5507 | 0.0000 | 0.0000 | 7.7458 |
| PRKAB2 | 0.2046 | 6.5930 | 5.5503 | 0.0000 | 0.0000 | 7.7435 |
| GDPD2 | -0.3145 | 0.6724 | -5.5493 | 0.0000 | 0.0000 | 7.7384 |
| GLTP | -0.1158 | 6.4018 | -5.5486 | 0.0000 | 0.0000 | 7.7350 |
| **EPCAM** | -1.3107 | 4.2203 | -5.5472 | 0.0000 | 0.0000 | 7.7279 |
| VCP | 0.0740 | 7.2495 | 5.5464 | 0.0000 | 0.0000 | 7.7236 |
| STK16 | 0.1288 | 6.3114 | 5.5463 | 0.0000 | 0.0000 | 7.7234 |
| ABCC8 | -0.6512 | 1.0567 | -5.5454 | 0.0000 | 0.0000 | 7.7185 |
| BMP8A | -0.5998 | 2.0449 | -5.5452 | 0.0000 | 0.0000 | 7.7179 |
| HOXC13 | -0.6314 | 0.5941 | -5.5428 | 0.0000 | 0.0000 | 7.7057 |
| TRAPPC6A | 0.1556 | 7.0204 | 5.5421 | 0.0000 | 0.0000 | 7.7021 |
| PINK1 | 0.2984 | 6.1482 | 5.5399 | 0.0000 | 0.0000 | 7.6905 |
| RHOQ | -0.2365 | 5.8309 | -5.5389 | 0.0000 | 0.0000 | 7.6858 |
| CLIC3 | -0.7152 | 3.6810 | -5.5336 | 0.0000 | 0.0000 | 7.6588 |
| TRPM8 | 0.8990 | 4.7518 | 5.5297 | 0.0000 | 0.0000 | 7.6386 |
| DAZAP2 | 0.0747 | 7.0371 | 5.5281 | 0.0000 | 0.0000 | 7.6306 |
| NEO1 | -0.2463 | 5.7027 | -5.5271 | 0.0000 | 0.0000 | 7.6259 |
| SLC9A2 | -0.4856 | 0.6869 | -5.5262 | 0.0000 | 0.0000 | 7.6211 |
| S100A3 | -0.7438 | 3.1725 | -5.5258 | 0.0000 | 0.0000 | 7.6190 |
| **ASPHD1** | -1.0649 | 4.0686 | -5.5235 | 0.0000 | 0.0000 | 7.6074 |
| PSMC3 | 0.0938 | 7.2927 | 5.5223 | 0.0000 | 0.0000 | 7.6015 |
| TRAM1 | 0.1018 | 7.3070 | 5.5223 | 0.0000 | 0.0000 | 7.6012 |
| FGGY | 0.4663 | 6.2671 | 5.5221 | 0.0000 | 0.0000 | 7.6004 |
| **CNKSR1** | -1.0012 | 2.4454 | -5.5220 | 0.0000 | 0.0000 | 7.6000 |
| EREG | -0.8529 | 1.0581 | -5.5216 | 0.0000 | 0.0000 | 7.5978 |
| ATP7A | -0.3525 | 4.0786 | -5.5212 | 0.0000 | 0.0000 | 7.5956 |
| IL1A | -0.3898 | 0.6578 | -5.5210 | 0.0000 | 0.0000 | 7.5949 |
| FKBP9 | -0.1406 | 6.3435 | -5.5199 | 0.0000 | 0.0000 | 7.5891 |
| CIRBP | 0.1236 | 6.8965 | 5.5184 | 0.0000 | 0.0000 | 7.5816 |

| ERMAP | 0.2379 | 5.5970 | 5.5176 | 0.0000 | 0.0000 | 7.5775 |
| --- | --- | --- | --- | --- | --- | --- |
| WASL | 0.1105 | 6.7304 | 5.5130 | 0.0000 | 0.0000 | 7.5544 |
| HLX | 0.2999 | 5.5331 | 5.5117 | 0.0000 | 0.0000 | 7.5478 |
| BDKRB1 | -0.7455 | 2.4352 | -5.5117 | 0.0000 | 0.0000 | 7.5477 |
| CYP4F12 | 0.5559 | 5.8623 | 5.5084 | 0.0000 | 0.0000 | 7.5310 |
| SLC25A24 | -0.6738 | 3.9786 | -5.5058 | 0.0000 | 0.0000 | 7.5177 |
| SERP1 | 0.0891 | 7.0208 | 5.5049 | 0.0000 | 0.0000 | 7.5134 |
| DNMT3L | 0.9393 | 2.2185 | 5.5043 | 0.0000 | 0.0000 | 7.5102 |
| **WFDC2** | -1.2175 | 3.6152 | -5.5042 | 0.0000 | 0.0000 | 7.5095 |
| HDDC2 | -0.2269 | 5.5120 | -5.5035 | 0.0000 | 0.0000 | 7.5062 |
| IQGAP1 | -0.2949 | 5.5865 | -5.5032 | 0.0000 | 0.0000 | 7.5046 |
| DZIP1 | -0.6784 | 2.9578 | -5.5027 | 0.0000 | 0.0000 | 7.5022 |
| BAP1 | 0.1315 | 6.7100 | 5.5023 | 0.0000 | 0.0000 | 7.5002 |
| PTPRB | 0.4854 | 4.6149 | 5.5021 | 0.0000 | 0.0000 | 7.4990 |
| C8B | 0.4712 | 7.2847 | 5.5018 | 0.0000 | 0.0000 | 7.4976 |
| CYTH2 | -0.1466 | 5.9808 | -5.5010 | 0.0000 | 0.0000 | 7.4936 |
| DLGAP1 | -0.4652 | 0.7313 | -5.4984 | 0.0000 | 0.0000 | 7.4803 |
| CXorf36 | 0.2939 | 5.0130 | 5.4983 | 0.0000 | 0.0000 | 7.4797 |
| PCCA | 0.2235 | 6.3085 | 5.4947 | 0.0000 | 0.0000 | 7.4617 |
| NRGN | -0.3707 | 5.2923 | -5.4945 | 0.0000 | 0.0000 | 7.4607 |
| CEP170 | -0.3579 | 4.5457 | -5.4944 | 0.0000 | 0.0000 | 7.4599 |
| ZNF580 | -0.2255 | 5.7975 | -5.4939 | 0.0000 | 0.0000 | 7.4576 |
| **PTGES** | -1.0485 | 3.8164 | -5.4928 | 0.0000 | 0.0000 | 7.4521 |
| **GCGR** | 1.2553 | 4.9088 | 5.4925 | 0.0000 | 0.0000 | 7.4504 |
| **EPS8L3** | -1.0931 | 4.8532 | -5.4924 | 0.0000 | 0.0000 | 7.4502 |
| ACSL6 | 0.8703 | 3.5310 | 5.4898 | 0.0000 | 0.0000 | 7.4368 |
| RAB5B | 0.0709 | 6.8326 | 5.4890 | 0.0000 | 0.0000 | 7.4328 |
| ANAPC13 | 0.0832 | 6.8003 | 5.4867 | 0.0000 | 0.0000 | 7.4214 |
| CELA3A | -0.7531 | 0.8300 | -5.4867 | 0.0000 | 0.0000 | 7.4211 |
| SPINK4 | -0.8984 | 1.1135 | -5.4855 | 0.0000 | 0.0000 | 7.4151 |
| RPS25 | 0.0905 | 7.6860 | 5.4806 | 0.0000 | 0.0000 | 7.3907 |
| UNC93B1 | -0.1611 | 6.2808 | -5.4804 | 0.0000 | 0.0000 | 7.3894 |
| **GABRB3** | -1.0355 | 1.5010 | -5.4799 | 0.0000 | 0.0000 | 7.3871 |
| PEX14 | 0.1676 | 6.3662 | 5.4777 | 0.0000 | 0.0000 | 7.3761 |
| PPP1R9A | -0.8238 | 2.9305 | -5.4775 | 0.0000 | 0.0000 | 7.3751 |
| OFD1 | -0.1707 | 5.6906 | -5.4773 | 0.0000 | 0.0000 | 7.3738 |
| STRA6 | -0.7706 | 1.3430 | -5.4758 | 0.0000 | 0.0000 | 7.3662 |
| MDH1 | 0.1350 | 7.0519 | 5.4751 | 0.0000 | 0.0000 | 7.3631 |
| SLC15A2 | -0.4366 | 2.0710 | -5.4734 | 0.0000 | 0.0000 | 7.3544 |
| C14orf132 | -0.8325 | 3.3901 | -5.4719 | 0.0000 | 0.0000 | 7.3467 |
| TXNRD2 | 0.2099 | 6.3339 | 5.4714 | 0.0000 | 0.0000 | 7.3445 |
| NDUFB1 | 0.1510 | 6.9371 | 5.4699 | 0.0000 | 0.0000 | 7.3365 |
| SECISBP2 | 0.1305 | 5.8906 | 5.4698 | 0.0000 | 0.0000 | 7.3364 |
| NDP | -0.4770 | 0.5108 | -5.4690 | 0.0000 | 0.0000 | 7.3323 |
| ZNF783 | -0.4050 | 4.1653 | -5.4689 | 0.0000 | 0.0000 | 7.3315 |
| LPAR3 | -0.8296 | 0.9467 | -5.4687 | 0.0000 | 0.0000 | 7.3306 |
| SRR | 0.2776 | 4.7565 | 5.4672 | 0.0000 | 0.0000 | 7.3234 |
| TIMM10 | 0.1256 | 7.1421 | 5.4641 | 0.0000 | 0.0000 | 7.3076 |
| CRMP1 | -0.7442 | 3.9732 | -5.4600 | 0.0000 | 0.0000 | 7.2870 |
| TMEM187 | 0.2148 | 6.1492 | 5.4590 | 0.0000 | 0.0000 | 7.2817 |
| NOTCH4 | 0.2784 | 5.2960 | 5.4585 | 0.0000 | 0.0000 | 7.2792 |
| MITF | -0.4796 | 3.6674 | -5.4583 | 0.0000 | 0.0000 | 7.2786 |
| CLDN6 | -0.6703 | 1.2888 | -5.4574 | 0.0000 | 0.0000 | 7.2739 |

| SERPINA6 | 0.2974 | 7.6040 | 5.4559 | 0.0000 | 0.0000 | 7.2665 |
| --- | --- | --- | --- | --- | --- | --- |
| KCNK2 | -0.7600 | 0.8286 | -5.4554 | 0.0000 | 0.0000 | 7.2637 |
| PMEPA1 | -0.5969 | 4.9838 | -5.4552 | 0.0000 | 0.0000 | 7.2631 |
| USH2A | 0.8198 | 2.8580 | 5.4550 | 0.0000 | 0.0000 | 7.2619 |
| CA14 | 0.7065 | 4.5218 | 5.4529 | 0.0000 | 0.0000 | 7.2511 |
| IK | 0.0796 | 6.8845 | 5.4523 | 0.0000 | 0.0000 | 7.2482 |
| UBE2H | 0.1238 | 6.6957 | 5.4510 | 0.0000 | 0.0000 | 7.2416 |
| CYC1 | 0.1181 | 7.4439 | 5.4498 | 0.0000 | 0.0000 | 7.2359 |
| ACTB | 0.0691 | 7.9973 | 5.4474 | 0.0000 | 0.0000 | 7.2239 |
| **PODXL2** | -1.0498 | 4.1031 | -5.4467 | 0.0000 | 0.0000 | 7.2204 |
| POU2F1 | -0.2237 | 4.7239 | -5.4464 | 0.0000 | 0.0000 | 7.2189 |
| PRPH | -0.3996 | 0.4322 | -5.4462 | 0.0000 | 0.0000 | 7.2175 |
| AHNAK2 | -0.8539 | 1.9951 | -5.4459 | 0.0000 | 0.0000 | 7.2162 |
| CNDP2 | 0.1361 | 6.7374 | 5.4455 | 0.0000 | 0.0000 | 7.2145 |
| CYP4F11 | 0.5073 | 6.6798 | 5.4447 | 0.0000 | 0.0000 | 7.2103 |
| GCAT | 0.2888 | 6.7232 | 5.4447 | 0.0000 | 0.0000 | 7.2102 |
| IFIT5 | 0.2014 | 5.9339 | 5.4435 | 0.0000 | 0.0000 | 7.2041 |
| **CYP1A2** | 1.4279 | 4.0927 | 5.4415 | 0.0000 | 0.0000 | 7.1944 |
| RAB11FIP5 | -0.3177 | 5.5302 | -5.4396 | 0.0000 | 0.0000 | 7.1845 |
| UPP1 | -0.2596 | 5.7118 | -5.4393 | 0.0000 | 0.0000 | 7.1833 |
| GLMN | -0.2152 | 5.0004 | -5.4390 | 0.0000 | 0.0000 | 7.1816 |
| NDUFS7 | 0.1692 | 6.4465 | 5.4382 | 0.0000 | 0.0000 | 7.1776 |
| EP400 | -0.2130 | 5.1148 | -5.4358 | 0.0000 | 0.0000 | 7.1657 |
| SETD3 | 0.1007 | 6.4371 | 5.4352 | 0.0000 | 0.0000 | 7.1625 |
| RPP25 | -0.4701 | 5.0944 | -5.4325 | 0.0000 | 0.0000 | 7.1491 |
| SNAPC1 | -0.2261 | 5.0208 | -5.4272 | 0.0000 | 0.0000 | 7.1227 |
| MEIS2 | -0.4030 | 4.7819 | -5.4270 | 0.0000 | 0.0000 | 7.1215 |
| SUV39H2 | -0.2176 | 5.2285 | -5.4266 | 0.0000 | 0.0000 | 7.1197 |
| DIAPH3 | -0.6207 | 3.4130 | -5.4258 | 0.0000 | 0.0000 | 7.1159 |
| CYP27B1 | -0.6375 | 2.9647 | -5.4256 | 0.0000 | 0.0000 | 7.1145 |
| SERINC5 | 0.2112 | 6.4770 | 5.4248 | 0.0000 | 0.0000 | 7.1108 |
| ZFC3H1 | -0.2505 | 5.1068 | -5.4248 | 0.0000 | 0.0000 | 7.1106 |
| SLC25A46 | 0.1732 | 5.9579 | 5.4245 | 0.0000 | 0.0000 | 7.1089 |
| GLRX | 0.2928 | 6.2058 | 5.4237 | 0.0000 | 0.0000 | 7.1053 |
| SLC25A10 | 0.1824 | 6.9200 | 5.4231 | 0.0000 | 0.0000 | 7.1022 |
| BCL2L2-PABPN1 | -0.4894 | 2.2258 | -5.4220 | 0.0000 | 0.0000 | 7.0967 |
| STX5 | 0.0971 | 6.8755 | 5.4195 | 0.0000 | 0.0000 | 7.0841 |
| HIPK2 | 0.2769 | 5.8835 | 5.4193 | 0.0000 | 0.0000 | 7.0832 |
| DFNA5 | -0.6669 | 4.1764 | -5.4192 | 0.0000 | 0.0000 | 7.0827 |
| ABCC5 | -0.2509 | 4.9815 | -5.4186 | 0.0000 | 0.0000 | 7.0798 |
| CAST | 0.1239 | 6.5079 | 5.4180 | 0.0000 | 0.0000 | 7.0767 |
| TFB2M | 0.1500 | 6.7159 | 5.4170 | 0.0000 | 0.0000 | 7.0716 |
| BAIAP2 | 0.2983 | 6.1694 | 5.4165 | 0.0000 | 0.0000 | 7.0694 |
| WBP2 | 0.1034 | 7.0434 | 5.4155 | 0.0000 | 0.0000 | 7.0640 |
| **PPP1R14D** | -1.0459 | 2.6700 | -5.4142 | 0.0000 | 0.0000 | 7.0575 |
| NLRP2 | -0.7937 | 1.6324 | -5.4131 | 0.0000 | 0.0000 | 7.0524 |
| PRDM4 | -0.1581 | 5.6744 | -5.4122 | 0.0000 | 0.0000 | 7.0478 |
| TNNT2 | -0.6797 | 1.3070 | -5.4100 | 0.0000 | 0.0000 | 7.0369 |
| **CYP11A1** | 1.0841 | 4.3663 | 5.4097 | 0.0000 | 0.0000 | 7.0353 |
| P2RY6 | -0.6518 | 3.5038 | -5.4052 | 0.0000 | 0.0000 | 7.0128 |
| MAGEA10 | -0.6839 | 0.5157 | -5.4051 | 0.0000 | 0.0000 | 7.0124 |
| CACNA2D2 | -0.5644 | 2.3319 | -5.4042 | 0.0000 | 0.0000 | 7.0079 |
| FMNL1 | -0.4413 | 4.7910 | -5.4032 | 0.0000 | 0.0000 | 7.0032 |

| SLC25A4 | 0.1785 | 6.4582 | 5.4026 | 0.0000 | 0.0000 | 6.9999 |
| --- | --- | --- | --- | --- | --- | --- |
| PNPLA2 | 0.1265 | 6.9303 | 5.4013 | 0.0000 | 0.0000 | 6.9936 |
| MMP9 | -0.7112 | 5.3753 | -5.4011 | 0.0000 | 0.0000 | 6.9927 |
| ATRN | 0.1657 | 6.8426 | 5.4007 | 0.0000 | 0.0000 | 6.9906 |
| HIST1H1D | -0.6037 | 0.9526 | -5.4006 | 0.0000 | 0.0000 | 6.9899 |
| NMD3 | 0.1097 | 6.5146 | 5.3998 | 0.0000 | 0.0000 | 6.9862 |
| HDAC9 | -0.5144 | 1.8583 | -5.3997 | 0.0000 | 0.0000 | 6.9855 |
| PKNOX1 | -0.1445 | 5.2029 | -5.3994 | 0.0000 | 0.0000 | 6.9842 |
| TLE3 | -0.3911 | 5.2464 | -5.3994 | 0.0000 | 0.0000 | 6.9840 |
| CHCHD2 | 0.0836 | 7.5864 | 5.3987 | 0.0000 | 0.0000 | 6.9808 |
| PDK3 | -0.4198 | 4.7757 | -5.3980 | 0.0000 | 0.0000 | 6.9771 |
| PRR15L | -0.9922 | 4.5530 | -5.3974 | 0.0000 | 0.0000 | 6.9742 |
| **GLRB** | -1.1216 | 2.7890 | -5.3962 | 0.0000 | 0.0000 | 6.9683 |
| BLMH | -0.4312 | 5.7373 | -5.3959 | 0.0000 | 0.0000 | 6.9664 |
| PBXIP1 | 0.1064 | 7.0432 | 5.3949 | 0.0000 | 0.0000 | 6.9619 |
| ADAP2 | -0.3275 | 5.1347 | -5.3945 | 0.0000 | 0.0000 | 6.9597 |
| TNFSF4 | -0.5033 | 4.1587 | -5.3945 | 0.0000 | 0.0000 | 6.9597 |
| XDH | 0.6633 | 5.9145 | 5.3935 | 0.0000 | 0.0000 | 6.9546 |
| SYCP2 | -0.5308 | 1.6791 | -5.3933 | 0.0000 | 0.0000 | 6.9536 |
| PDCL3 | -0.1222 | 6.2535 | -5.3926 | 0.0000 | 0.0000 | 6.9501 |
| DUS1L | 0.1239 | 6.9117 | 5.3918 | 0.0000 | 0.0000 | 6.9465 |
| MGST3 | 0.1514 | 6.4839 | 5.3906 | 0.0000 | 0.0000 | 6.9402 |
| ZMYM1 | -0.2817 | 4.7736 | -5.3895 | 0.0000 | 0.0000 | 6.9348 |
| RNPS1 | -0.1056 | 6.2323 | -5.3886 | 0.0000 | 0.0000 | 6.9305 |
| GPRC5A | -0.7382 | 1.6703 | -5.3881 | 0.0000 | 0.0000 | 6.9280 |
| GRM3 | -0.4877 | 0.4724 | -5.3868 | 0.0000 | 0.0000 | 6.9213 |
| ZBED4 | -0.2343 | 5.3495 | -5.3853 | 0.0000 | 0.0000 | 6.9142 |
| APCS | 0.3778 | 7.7837 | 5.3840 | 0.0000 | 0.0000 | 6.9075 |
| **PDX1** | -1.3459 | 2.6775 | -5.3839 | 0.0000 | 0.0000 | 6.9068 |
| STAP2 | 0.2120 | 6.8831 | 5.3830 | 0.0000 | 0.0000 | 6.9026 |
| GTF3A | 0.1265 | 7.0575 | 5.3825 | 0.0000 | 0.0000 | 6.9000 |
| HAUS4 | 0.2506 | 6.1999 | 5.3817 | 0.0000 | 0.0000 | 6.8959 |
| ATP5A1 | 0.1007 | 7.1220 | 5.3814 | 0.0000 | 0.0000 | 6.8947 |
| RNPEP | 0.1119 | 6.8930 | 5.3805 | 0.0000 | 0.0000 | 6.8903 |
| **NAT2** | 1.1138 | 4.7143 | 5.3802 | 0.0000 | 0.0000 | 6.8887 |
| ASB6 | -0.1359 | 5.7936 | -5.3797 | 0.0000 | 0.0000 | 6.8860 |
| ATP1A1 | -0.1059 | 7.2666 | -5.3786 | 0.0000 | 0.0000 | 6.8809 |
| VIL1 | -0.8955 | 5.4379 | -5.3786 | 0.0000 | 0.0000 | 6.8808 |
| BCAS4 | -0.5657 | 3.7618 | -5.3783 | 0.0000 | 0.0000 | 6.8794 |
| GPC4 | -0.9114 | 3.8287 | -5.3751 | 0.0000 | 0.0000 | 6.8632 |
| OLR1 | -0.9476 | 2.6301 | -5.3740 | 0.0000 | 0.0000 | 6.8580 |
| HES2 | -0.7094 | 1.5961 | -5.3726 | 0.0000 | 0.0000 | 6.8509 |
| MARCKS | -0.1993 | 6.7327 | -5.3694 | 0.0000 | 0.0000 | 6.8352 |
| FOXD4 | -0.5620 | 1.7782 | -5.3682 | 0.0000 | 0.0000 | 6.8291 |
| SLC7A11 | -0.9279 | 3.1904 | -5.3681 | 0.0000 | 0.0000 | 6.8287 |
| MRPS35 | 0.0836 | 7.0836 | 5.3678 | 0.0000 | 0.0000 | 6.8272 |
| SHPK | 0.2897 | 5.0786 | 5.3676 | 0.0000 | 0.0000 | 6.8261 |
| NRBF2 | 0.1175 | 6.5567 | 5.3670 | 0.0000 | 0.0000 | 6.8232 |
| ANGPTL3 | 0.4970 | 7.2145 | 5.3665 | 0.0000 | 0.0000 | 6.8209 |
| PECAM1 | 0.1787 | 6.4923 | 5.3661 | 0.0000 | 0.0000 | 6.8187 |
| CD207 | -0.8067 | 2.1773 | -5.3653 | 0.0000 | 0.0000 | 6.8150 |
| SNRK | 0.1735 | 5.9317 | 5.3649 | 0.0000 | 0.0000 | 6.8131 |
| SLC36A1 | -0.3096 | 4.8318 | -5.3648 | 0.0000 | 0.0000 | 6.8126 |

| RAB27B | -0.8598 | 2.8145 | -5.3626 | 0.0000 | 0.0000 | 6.8017 |
| --- | --- | --- | --- | --- | --- | --- |
| SLC43A1 | 0.1615 | 7.1558 | 5.3602 | 0.0000 | 0.0000 | 6.7899 |
| AHCY | 0.1104 | 7.3159 | 5.3588 | 0.0000 | 0.0000 | 6.7829 |
| ADORA3 | -0.5586 | 3.6794 | -5.3549 | 0.0000 | 0.0000 | 6.7637 |
| INHBC | 0.5919 | 6.4272 | 5.3524 | 0.0000 | 0.0000 | 6.7510 |
| HEXB | 0.1155 | 7.0201 | 5.3504 | 0.0000 | 0.0000 | 6.7415 |
| PTDSS2 | -0.1907 | 5.8686 | -5.3499 | 0.0000 | 0.0000 | 6.7389 |
| WIPI2 | 0.1039 | 6.4099 | 5.3488 | 0.0000 | 0.0000 | 6.7332 |
| ZNF133 | -0.2091 | 5.1352 | -5.3487 | 0.0000 | 0.0000 | 6.7328 |
| PPP1R13L | -0.2825 | 5.5112 | -5.3472 | 0.0000 | 0.0000 | 6.7256 |
| LPO | -0.3225 | 0.3138 | -5.3470 | 0.0000 | 0.0000 | 6.7247 |
| NMT1 | 0.0871 | 6.7517 | 5.3456 | 0.0000 | 0.0000 | 6.7177 |
| MAP3K12 | -0.3644 | 3.5686 | -5.3456 | 0.0000 | 0.0000 | 6.7173 |
| ARSA | 0.1665 | 6.8721 | 5.3444 | 0.0000 | 0.0000 | 6.7115 |
| H2AFY | -0.1277 | 6.1505 | -5.3405 | 0.0000 | 0.0000 | 6.6925 |
| IL32 | 0.2134 | 7.3832 | 5.3395 | 0.0000 | 0.0000 | 6.6875 |
| **CYP1A1** | 1.2318 | 4.2835 | 5.3382 | 0.0000 | 0.0000 | 6.6812 |
| SDC3 | -0.2610 | 6.1029 | -5.3380 | 0.0000 | 0.0000 | 6.6800 |
| MYH15 | -0.2824 | 0.4145 | -5.3362 | 0.0000 | 0.0000 | 6.6711 |
| KIF1C | 0.1314 | 6.8003 | 5.3359 | 0.0000 | 0.0000 | 6.6698 |
| WDR73 | -0.3030 | 3.1035 | -5.3357 | 0.0000 | 0.0000 | 6.6688 |
| CMAS | 0.1073 | 6.8353 | 5.3337 | 0.0000 | 0.0000 | 6.6588 |
| NDRG3 | -0.1457 | 6.2875 | -5.3322 | 0.0000 | 0.0000 | 6.6514 |
| GNA14 | 0.5844 | 3.9115 | 5.3320 | 0.0000 | 0.0000 | 6.6506 |
| RCN2 | -0.2267 | 5.4828 | -5.3313 | 0.0000 | 0.0000 | 6.6470 |
| UBE2D4 | 0.1538 | 5.9644 | 5.3310 | 0.0000 | 0.0000 | 6.6455 |
| FTCD | 0.5627 | 7.0941 | 5.3278 | 0.0000 | 0.0000 | 6.6301 |
| IFNGR2 | -0.1349 | 6.6982 | -5.3274 | 0.0000 | 0.0000 | 6.6280 |
| SEC23A | 0.1842 | 6.2597 | 5.3274 | 0.0000 | 0.0000 | 6.6278 |
| ZNF207 | -0.1033 | 6.0503 | -5.3271 | 0.0000 | 0.0000 | 6.6262 |
| KIF26B | -0.8080 | 2.3363 | -5.3269 | 0.0000 | 0.0000 | 6.6257 |
| ETS2 | 0.1547 | 7.0374 | 5.3268 | 0.0000 | 0.0000 | 6.6252 |
| GTF3C2 | -0.1151 | 6.0423 | -5.3255 | 0.0000 | 0.0000 | 6.6188 |
| DLX6 | -0.8325 | 0.8715 | -5.3233 | 0.0000 | 0.0000 | 6.6079 |
| VRK1 | -0.2155 | 5.4864 | -5.3225 | 0.0000 | 0.0000 | 6.6037 |
| ARTN | -0.5706 | 2.2891 | -5.3223 | 0.0000 | 0.0000 | 6.6031 |
| PSMB6 | 0.1116 | 7.2771 | 5.3199 | 0.0000 | 0.0000 | 6.5909 |
| DSCC1 | -0.3426 | 4.9340 | -5.3184 | 0.0000 | 0.0000 | 6.5838 |
| POU5F1 | -0.6114 | 3.8583 | -5.3174 | 0.0000 | 0.0000 | 6.5790 |
| PTAFR | -0.4981 | 4.4986 | -5.3148 | 0.0000 | 0.0000 | 6.5661 |
| TRMT1L | 0.1608 | 6.0363 | 5.3110 | 0.0000 | 0.0000 | 6.5472 |
| RUSC1 | -0.2207 | 5.8437 | -5.3098 | 0.0000 | 0.0000 | 6.5414 |
| ZNF85 | -0.6108 | 2.9306 | -5.3091 | 0.0000 | 0.0000 | 6.5382 |
| FLT4 | 0.3528 | 4.9706 | 5.3080 | 0.0000 | 0.0000 | 6.5327 |
| ETV6 | -0.1859 | 5.5853 | -5.3059 | 0.0000 | 0.0000 | 6.5225 |
| DYNLT3 | 0.1739 | 6.4493 | 5.3024 | 0.0000 | 0.0000 | 6.5055 |
| SPAG6 | -0.4593 | 0.5935 | -5.3015 | 0.0000 | 0.0000 | 6.5010 |
| TMCO1 | 0.0971 | 7.0145 | 5.2982 | 0.0000 | 0.0000 | 6.4845 |
| AJAP1 | -0.4701 | 0.6056 | -5.2978 | 0.0000 | 0.0000 | 6.4830 |
| PCGF3 | -0.1552 | 5.8489 | -5.2976 | 0.0000 | 0.0000 | 6.4818 |
| TF | 0.3459 | 7.8533 | 5.2971 | 0.0000 | 0.0000 | 6.4794 |
| SSPN | -0.7144 | 2.7051 | -5.2935 | 0.0000 | 0.0000 | 6.4616 |
| ZBTB40 | -0.2209 | 5.1537 | -5.2931 | 0.0000 | 0.0000 | 6.4598 |

| TULP3 | -0.1871 | 5.8423 | -5.2929 | 0.0000 | 0.0000 | 6.4587 |
| --- | --- | --- | --- | --- | --- | --- |
| AVPR1B | -0.2185 | 0.1757 | -5.2926 | 0.0000 | 0.0000 | 6.4571 |
| G3BP1 | 0.1099 | 6.4757 | 5.2920 | 0.0000 | 0.0000 | 6.4543 |
| EVC | -0.9601 | 3.8049 | -5.2919 | 0.0000 | 0.0000 | 6.4541 |
| AP3B2 | -0.4888 | 0.6114 | -5.2918 | 0.0000 | 0.0000 | 6.4533 |
| SAA1 | 0.6478 | 7.2638 | 5.2915 | 0.0000 | 0.0000 | 6.4520 |
| UBL5 | 0.1083 | 7.2802 | 5.2907 | 0.0000 | 0.0000 | 6.4478 |
| TUT1 | 0.1668 | 6.1036 | 5.2903 | 0.0000 | 0.0000 | 6.4458 |
| PIP4K2A | -0.2018 | 5.8366 | -5.2902 | 0.0000 | 0.0000 | 6.4455 |
| ZNF117 | -0.4525 | 3.8039 | -5.2877 | 0.0000 | 0.0000 | 6.4333 |
| IKZF4 | -0.3154 | 3.7752 | -5.2876 | 0.0000 | 0.0000 | 6.4329 |
| HDLBP | 0.0785 | 7.1742 | 5.2870 | 0.0000 | 0.0000 | 6.4301 |
| DNAJA2 | 0.1298 | 6.5719 | 5.2865 | 0.0000 | 0.0000 | 6.4276 |
| CDKN2D | -0.2211 | 5.5290 | -5.2855 | 0.0000 | 0.0000 | 6.4224 |
| GTDC1 | -0.2665 | 4.1828 | -5.2854 | 0.0000 | 0.0000 | 6.4222 |
| FAM35A | 0.1496 | 6.2675 | 5.2849 | 0.0000 | 0.0000 | 6.4195 |
| LRRK1 | -0.5128 | 2.5703 | -5.2803 | 0.0000 | 0.0000 | 6.3970 |
| NARS2 | 0.1630 | 6.1999 | 5.2799 | 0.0000 | 0.0000 | 6.3954 |
| RAD9A | -0.1823 | 5.6966 | -5.2797 | 0.0000 | 0.0000 | 6.3945 |
| CTBP2 | -0.5261 | 3.9136 | -5.2795 | 0.0000 | 0.0000 | 6.3933 |
| RNF186 | -0.8205 | 1.9679 | -5.2793 | 0.0000 | 0.0000 | 6.3925 |
| TGFB2 | -0.7181 | 3.5209 | -5.2780 | 0.0000 | 0.0000 | 6.3860 |
| CDHR5 | 0.5979 | 6.9627 | 5.2778 | 0.0000 | 0.0000 | 6.3850 |
| TOR1AIP2 | 0.1673 | 6.3714 | 5.2777 | 0.0000 | 0.0000 | 6.3845 |
| RCE1 | -0.1580 | 5.8669 | -5.2731 | 0.0000 | 0.0000 | 6.3623 |
| RPN1 | 0.0724 | 7.4451 | 5.2729 | 0.0000 | 0.0000 | 6.3612 |
| MGAT5 | -0.2777 | 5.4381 | -5.2696 | 0.0000 | 0.0000 | 6.3452 |
| CETP | 0.4420 | 5.1486 | 5.2675 | 0.0000 | 0.0000 | 6.3349 |
| RXRB | 0.1101 | 6.5673 | 5.2671 | 0.0000 | 0.0000 | 6.3330 |
| MPP6 | -0.2733 | 5.2601 | -5.2659 | 0.0000 | 0.0000 | 6.3269 |
| PEX6 | 0.1659 | 6.7499 | 5.2657 | 0.0000 | 0.0000 | 6.3261 |
| CELF3 | -0.3219 | 0.4351 | -5.2645 | 0.0000 | 0.0000 | 6.3201 |
| RRN3 | 0.1411 | 6.3232 | 5.2644 | 0.0000 | 0.0000 | 6.3196 |
| SLC7A10 | -0.9717 | 1.4950 | -5.2636 | 0.0000 | 0.0000 | 6.3158 |
| RAMP2 | 0.2369 | 6.4152 | 5.2633 | 0.0000 | 0.0000 | 6.3145 |
| PRCP | 0.2201 | 6.1469 | 5.2630 | 0.0000 | 0.0000 | 6.3127 |
| XPC | 0.1689 | 6.2468 | 5.2629 | 0.0000 | 0.0000 | 6.3122 |
| PEX2 | 0.1290 | 6.3094 | 5.2628 | 0.0000 | 0.0000 | 6.3121 |
| BLCAP | 0.1540 | 6.4416 | 5.2612 | 0.0000 | 0.0000 | 6.3042 |
| CHAF1A | -0.2148 | 5.8322 | -5.2573 | 0.0000 | 0.0000 | 6.2852 |
| GPR19 | -0.5822 | 2.4873 | -5.2572 | 0.0000 | 0.0000 | 6.2845 |
| AP1G2 | -0.4448 | 4.9555 | -5.2564 | 0.0000 | 0.0000 | 6.2810 |
| RAB14 | 0.0908 | 6.8686 | 5.2558 | 0.0000 | 0.0000 | 6.2780 |
| GAA | 0.1307 | 7.0546 | 5.2555 | 0.0000 | 0.0000 | 6.2763 |
| NFATC4 | -0.5208 | 4.0043 | -5.2549 | 0.0000 | 0.0000 | 6.2736 |
| PPP3R1 | 0.0817 | 6.6283 | 5.2531 | 0.0000 | 0.0000 | 6.2646 |
| EIF5A2 | -0.7725 | 4.1267 | -5.2521 | 0.0000 | 0.0000 | 6.2599 |
| ATP1A3 | -0.5841 | 1.3838 | -5.2521 | 0.0000 | 0.0000 | 6.2597 |
| PHC2 | -0.0957 | 6.8528 | -5.2519 | 0.0000 | 0.0000 | 6.2590 |
| HAX1 | 0.0968 | 7.2070 | 5.2508 | 0.0000 | 0.0000 | 6.2536 |
| DOLPP1 | 0.1277 | 6.5566 | 5.2499 | 0.0000 | 0.0000 | 6.2495 |
| S100A5 | -0.4973 | 0.7369 | -5.2494 | 0.0000 | 0.0000 | 6.2467 |
| ENDOG | 0.2076 | 6.2470 | 5.2493 | 0.0000 | 0.0000 | 6.2464 |

| TMEM43 | -0.1131 | 6.4540 | -5.2476 | 0.0000 | 0.0000 | 6.2378 |
| --- | --- | --- | --- | --- | --- | --- |
| RAB3D | -0.6591 | 4.3115 | -5.2471 | 0.0000 | 0.0000 | 6.2357 |
| PRKCD | -0.2155 | 6.0785 | -5.2469 | 0.0000 | 0.0000 | 6.2345 |
| PPP1R10 | 0.1080 | 6.7587 | 5.2464 | 0.0000 | 0.0000 | 6.2324 |
| BCORL1 | -0.3384 | 5.0106 | -5.2457 | 0.0000 | 0.0000 | 6.2286 |
| YTHDF3 | 0.1257 | 6.5140 | 5.2451 | 0.0000 | 0.0000 | 6.2261 |
| NRN1 | 0.4673 | 4.7217 | 5.2425 | 0.0000 | 0.0000 | 6.2132 |
| PLP2 | -0.2531 | 6.7896 | -5.2418 | 0.0000 | 0.0000 | 6.2101 |
| FOXF2 | -0.7144 | 2.2337 | -5.2416 | 0.0000 | 0.0000 | 6.2092 |
| TAL1 | 0.4616 | 3.0139 | 5.2411 | 0.0000 | 0.0000 | 6.2065 |
| GTPBP2 | -0.1523 | 6.2787 | -5.2402 | 0.0000 | 0.0000 | 6.2020 |
| CARD14 | -0.5214 | 1.5716 | -5.2401 | 0.0000 | 0.0000 | 6.2016 |
| NUP210 | -0.1992 | 6.2095 | -5.2376 | 0.0000 | 0.0000 | 6.1897 |
| CANX | 0.0775 | 7.5250 | 5.2353 | 0.0000 | 0.0000 | 6.1783 |
| DAD1 | 0.0813 | 7.5775 | 5.2348 | 0.0000 | 0.0000 | 6.1761 |
| **COL4A5** | -1.0076 | 3.1552 | -5.2347 | 0.0000 | 0.0000 | 6.1754 |
| TTC1 | 0.1006 | 6.9034 | 5.2345 | 0.0000 | 0.0000 | 6.1747 |
| NELL1 | -0.4318 | 0.2491 | -5.2345 | 0.0000 | 0.0000 | 6.1747 |
| LAMB1 | -0.2891 | 6.2332 | -5.2331 | 0.0000 | 0.0000 | 6.1676 |
| RCAN3 | -0.4890 | 4.4129 | -5.2315 | 0.0000 | 0.0000 | 6.1601 |
| TXN | 0.1158 | 7.6913 | 5.2310 | 0.0000 | 0.0000 | 6.1576 |
| ZNF287 | -0.6163 | 2.5238 | -5.2308 | 0.0000 | 0.0000 | 6.1567 |
| NPHS2 | -0.3540 | 0.2115 | -5.2308 | 0.0000 | 0.0000 | 6.1564 |
| HR | -0.6666 | 1.8051 | -5.2296 | 0.0000 | 0.0000 | 6.1509 |
| PMCH | -0.4805 | 0.7360 | -5.2294 | 0.0000 | 0.0000 | 6.1499 |
| IGFBP1 | 0.4042 | 7.5435 | 5.2287 | 0.0000 | 0.0000 | 6.1464 |
| CDK2 | -0.1982 | 5.9733 | -5.2280 | 0.0000 | 0.0000 | 6.1430 |
| DNMT3B | -0.4798 | 3.9241 | -5.2276 | 0.0000 | 0.0000 | 6.1413 |
| CPEB1 | -0.5302 | 0.6869 | -5.2275 | 0.0000 | 0.0000 | 6.1406 |
| TMEM59L | -0.5906 | 1.4795 | -5.2265 | 0.0000 | 0.0000 | 6.1360 |
| BRPF1 | -0.1570 | 5.4838 | -5.2265 | 0.0000 | 0.0000 | 6.1357 |
| FAM96B | 0.1105 | 7.2775 | 5.2246 | 0.0000 | 0.0000 | 6.1267 |
| TMEM47 | 0.4810 | 5.5394 | 5.2241 | 0.0000 | 0.0000 | 6.1243 |
| C2orf42 | 0.1487 | 5.5424 | 5.2236 | 0.0000 | 0.0000 | 6.1220 |
| CD177 | -0.7246 | 1.5745 | -5.2232 | 0.0000 | 0.0000 | 6.1200 |
| CHRNB2 | -0.4211 | 0.8454 | -5.2223 | 0.0000 | 0.0000 | 6.1156 |
| RASSF9 | -0.7852 | 2.5803 | -5.2218 | 0.0000 | 0.0000 | 6.1131 |
| FOXJ3 | -0.1547 | 5.7803 | -5.2187 | 0.0000 | 0.0000 | 6.0981 |
| SULT1A1 | 0.3755 | 6.2110 | 5.2177 | 0.0000 | 0.0000 | 6.0931 |
| CHRNA6 | -0.3777 | 0.5060 | -5.2174 | 0.0000 | 0.0000 | 6.0919 |
| PPARGC1A | 0.7164 | 5.3813 | 5.2155 | 0.0000 | 0.0000 | 6.0825 |
| SLC25A3 | 0.0866 | 7.1560 | 5.2137 | 0.0000 | 0.0000 | 6.0737 |
| TES | -0.3322 | 5.8904 | -5.2110 | 0.0000 | 0.0000 | 6.0608 |
| DSG2 | -0.6390 | 5.6249 | -5.2063 | 0.0000 | 0.0000 | 6.0381 |
| OLFML3 | -0.5196 | 5.2972 | -5.2057 | 0.0000 | 0.0000 | 6.0352 |
| POM121L2 | -0.2074 | 0.1278 | -5.2050 | 0.0000 | 0.0000 | 6.0321 |
| ZNF586 | -0.2926 | 4.4815 | -5.2034 | 0.0000 | 0.0000 | 6.0241 |
| HSPB7 | -0.7419 | 2.5421 | -5.2014 | 0.0000 | 0.0000 | 6.0145 |
| TLX1 | 0.8247 | 3.8956 | 5.2012 | 0.0000 | 0.0000 | 6.0136 |
| ADCY10 | 0.6300 | 3.9000 | 5.2002 | 0.0000 | 0.0000 | 6.0087 |
| MMP14 | -0.2049 | 6.6263 | -5.1992 | 0.0000 | 0.0000 | 6.0040 |
| RGS20 | -0.5441 | 1.1353 | -5.1985 | 0.0000 | 0.0000 | 6.0007 |
| BCKDK | 0.1255 | 6.7645 | 5.1982 | 0.0000 | 0.0000 | 5.9993 |

| HOXC6 | -0.7975 | 1.1769 | -5.1971 | 0.0000 | 0.0000 | 5.9941 |
| --- | --- | --- | --- | --- | --- | --- |
| ZNF816 | -0.6763 | 3.7528 | -5.1957 | 0.0000 | 0.0000 | 5.9874 |
| NUP37 | -0.1624 | 5.7243 | -5.1953 | 0.0000 | 0.0000 | 5.9853 |
| RPL30 | 0.1120 | 7.5845 | 5.1948 | 0.0000 | 0.0000 | 5.9827 |
| **SAA2-SAA4** | 1.1095 | 5.4265 | 5.1939 | 0.0000 | 0.0000 | 5.9784 |
| MIP | 0.5463 | 1.7789 | 5.1937 | 0.0000 | 0.0000 | 5.9776 |
| EDF1 | 0.1024 | 7.7073 | 5.1924 | 0.0000 | 0.0000 | 5.9711 |
| HEATR6 | -0.2163 | 5.2086 | -5.1916 | 0.0000 | 0.0000 | 5.9676 |
| DDX28 | 0.1572 | 6.1653 | 5.1910 | 0.0000 | 0.0000 | 5.9644 |
| E2F1 | -0.3849 | 6.0378 | -5.1909 | 0.0000 | 0.0000 | 5.9640 |
| HDAC11 | -0.2362 | 5.8810 | -5.1901 | 0.0000 | 0.0000 | 5.9602 |
| HNF1B | -0.6246 | 5.3228 | -5.1891 | 0.0000 | 0.0000 | 5.9554 |
| IL1RN | 0.3868 | 6.4261 | 5.1884 | 0.0000 | 0.0000 | 5.9520 |
| DROSHA | -0.1281 | 5.9003 | -5.1857 | 0.0000 | 0.0000 | 5.9393 |
| ZNF611 | -0.6221 | 3.3240 | -5.1855 | 0.0000 | 0.0000 | 5.9382 |
| POFUT2 | -0.1769 | 5.7043 | -5.1842 | 0.0000 | 0.0000 | 5.9319 |
| ABHD10 | 0.1076 | 6.5031 | 5.1819 | 0.0000 | 0.0000 | 5.9211 |
| NEFH | -0.5875 | 2.0824 | -5.1818 | 0.0000 | 0.0000 | 5.9203 |
| KCNMB2 | -0.3811 | 0.5552 | -5.1812 | 0.0000 | 0.0000 | 5.9173 |
| PRKAR2B | -0.6680 | 2.7748 | -5.1801 | 0.0000 | 0.0000 | 5.9122 |
| IFT52 | -0.1586 | 5.9450 | -5.1772 | 0.0000 | 0.0000 | 5.8983 |
| PUS7L | -0.2431 | 4.4549 | -5.1763 | 0.0000 | 0.0000 | 5.8939 |
| SMO | 0.2823 | 6.5898 | 5.1759 | 0.0000 | 0.0000 | 5.8923 |
| **CD5L** | 1.0829 | 4.3589 | 5.1741 | 0.0000 | 0.0000 | 5.8834 |
| SLC38A2 | 0.1629 | 6.9518 | 5.1710 | 0.0000 | 0.0000 | 5.8683 |
| UBE2V2 | 0.1382 | 6.3122 | 5.1709 | 0.0000 | 0.0000 | 5.8679 |
| RAPGEF2 | 0.2891 | 5.4941 | 5.1692 | 0.0000 | 0.0000 | 5.8601 |
| DDO | 0.2789 | 5.9587 | 5.1688 | 0.0000 | 0.0000 | 5.8582 |
| ZNF215 | -0.7246 | 1.8337 | -5.1672 | 0.0000 | 0.0000 | 5.8504 |
| SLC20A2 | 0.2263 | 6.2682 | 5.1659 | 0.0000 | 0.0000 | 5.8443 |
| ALDH3B2 | -0.6502 | 0.6940 | -5.1654 | 0.0000 | 0.0000 | 5.8420 |
| ARAF | 0.1038 | 6.8714 | 5.1629 | 0.0000 | 0.0000 | 5.8298 |
| UNC5B | -0.4434 | 5.0244 | -5.1596 | 0.0000 | 0.0000 | 5.8139 |
| KPNA2 | -0.1507 | 6.7392 | -5.1570 | 0.0000 | 0.0000 | 5.8015 |
| ALOX12B | -0.4037 | 0.4679 | -5.1561 | 0.0000 | 0.0000 | 5.7974 |
| TMEM159 | -0.5005 | 4.2797 | -5.1552 | 0.0000 | 0.0000 | 5.7929 |
| RASA3 | -0.4346 | 4.9679 | -5.1549 | 0.0000 | 0.0000 | 5.7915 |
| EPB41L4B | 0.4286 | 5.9504 | 5.1500 | 0.0000 | 0.0000 | 5.7679 |
| CDC37 | 0.0733 | 7.1624 | 5.1486 | 0.0000 | 0.0000 | 5.7613 |
| RPS27 | 0.0910 | 7.8882 | 5.1485 | 0.0000 | 0.0000 | 5.7610 |
| RAD54L2 | 0.2177 | 5.2713 | 5.1483 | 0.0000 | 0.0000 | 5.7598 |
| **CLDN4** | -1.0495 | 4.6090 | -5.1478 | 0.0000 | 0.0000 | 5.7577 |
| GSTCD | -0.3573 | 4.2207 | -5.1475 | 0.0000 | 0.0000 | 5.7563 |
| EIF4G1 | 0.0744 | 7.2049 | 5.1472 | 0.0000 | 0.0000 | 5.7549 |
| FOXN3 | 0.1962 | 5.9953 | 5.1466 | 0.0000 | 0.0000 | 5.7519 |
| HSD17B7 | 0.2528 | 5.9574 | 5.1459 | 0.0000 | 0.0000 | 5.7484 |
| F2 | 0.3661 | 7.7711 | 5.1458 | 0.0000 | 0.0000 | 5.7479 |
| SLC6A3 | -0.5759 | 0.6399 | -5.1441 | 0.0000 | 0.0000 | 5.7400 |
| GOLM1 | -0.3269 | 6.5955 | -5.1433 | 0.0000 | 0.0000 | 5.7360 |
| MZF1 | -0.2197 | 5.1738 | -5.1423 | 0.0000 | 0.0000 | 5.7315 |
| HIST1H2AJ | -0.6780 | 1.2114 | -5.1400 | 0.0000 | 0.0000 | 5.7204 |
| VEZT | -0.1708 | 5.7825 | -5.1394 | 0.0000 | 0.0000 | 5.7176 |
| RGSL1 | 0.9056 | 1.3152 | 5.1389 | 0.0000 | 0.0000 | 5.7152 |

| HNRNPA2B1 | 0.0483 | 7.3195 | 5.1345 | 0.0000 | 0.0000 | 5.6939 |
| --- | --- | --- | --- | --- | --- | --- |
| RHBDF2 | -0.2723 | 5.6146 | -5.1340 | 0.0000 | 0.0000 | 5.6919 |
| COLEC12 | -0.7415 | 2.8561 | -5.1340 | 0.0000 | 0.0000 | 5.6917 |
| IFT88 | 0.2102 | 5.3511 | 5.1332 | 0.0000 | 0.0000 | 5.6879 |
| MCFD2 | 0.1108 | 6.9241 | 5.1322 | 0.0000 | 0.0000 | 5.6834 |
| RTN4 | 0.1145 | 7.0818 | 5.1321 | 0.0000 | 0.0000 | 5.6825 |
| CRTC3 | -0.1956 | 5.3341 | -5.1320 | 0.0000 | 0.0000 | 5.6821 |
| STX7 | -0.2172 | 5.1620 | -5.1311 | 0.0000 | 0.0000 | 5.6780 |
| DENND3 | -0.3993 | 3.8537 | -5.1311 | 0.0000 | 0.0000 | 5.6779 |
| AKAP1 | 0.1151 | 6.7613 | 5.1308 | 0.0000 | 0.0000 | 5.6767 |
| CDC25C | -0.5979 | 4.6486 | -5.1288 | 0.0000 | 0.0000 | 5.6671 |
| PRKCSH | 0.0750 | 7.3598 | 5.1277 | 0.0000 | 0.0000 | 5.6620 |
| FMO2 | 0.7104 | 2.9327 | 5.1264 | 0.0000 | 0.0000 | 5.6554 |
| KIAA0930 | -0.1911 | 6.2058 | -5.1263 | 0.0000 | 0.0000 | 5.6553 |
| CDK20 | -0.3821 | 4.1708 | -5.1256 | 0.0000 | 0.0000 | 5.6516 |
| GFRA2 | 0.5483 | 3.2042 | 5.1248 | 0.0000 | 0.0000 | 5.6482 |
| METTL8 | -0.2107 | 4.4324 | -5.1241 | 0.0000 | 0.0000 | 5.6447 |
| MAN2A1 | 0.1510 | 6.3126 | 5.1240 | 0.0000 | 0.0000 | 5.6440 |
| **GP2** | -1.0759 | 1.5265 | -5.1232 | 0.0000 | 0.0000 | 5.6402 |
| TRAF2 | -0.1559 | 6.2846 | -5.1226 | 0.0000 | 0.0000 | 5.6375 |
| ABR | -0.4339 | 4.5886 | -5.1220 | 0.0000 | 0.0000 | 5.6347 |
| ZNF280A | -0.4318 | 0.3616 | -5.1216 | 0.0000 | 0.0000 | 5.6329 |
| IFRD2 | 0.1139 | 6.7308 | 5.1215 | 0.0000 | 0.0000 | 5.6322 |
| PRDM5 | -0.5262 | 1.3553 | -5.1193 | 0.0000 | 0.0000 | 5.6220 |
| APOL6 | 0.2225 | 6.3925 | 5.1188 | 0.0000 | 0.0000 | 5.6193 |
| EIF2AK1 | 0.0729 | 7.0541 | 5.1188 | 0.0000 | 0.0000 | 5.6192 |
| TRIP10 | -0.2044 | 5.9637 | -5.1173 | 0.0000 | 0.0000 | 5.6125 |
| CRYM | 0.5240 | 5.4036 | 5.1166 | 0.0000 | 0.0000 | 5.6090 |
| CAV3 | -0.1933 | 0.1634 | -5.1162 | 0.0000 | 0.0000 | 5.6072 |
| TGFBI | 0.1785 | 6.9097 | 5.1157 | 0.0000 | 0.0000 | 5.6048 |
| TPM4 | -0.1768 | 6.6654 | -5.1148 | 0.0000 | 0.0000 | 5.6005 |
| IQSEC2 | -0.4615 | 3.4911 | -5.1147 | 0.0000 | 0.0000 | 5.5999 |
| ADA | -0.2781 | 5.4359 | -5.1143 | 0.0000 | 0.0000 | 5.5980 |
| CYP17A1 | 0.9485 | 5.1024 | 5.1132 | 0.0000 | 0.0000 | 5.5929 |
| SEL1L | 0.1483 | 6.6661 | 5.1131 | 0.0000 | 0.0000 | 5.5926 |
| NEFL | -0.5817 | 0.5963 | -5.1121 | 0.0000 | 0.0000 | 5.5878 |
| MRPL41 | 0.1408 | 7.2305 | 5.1117 | 0.0000 | 0.0000 | 5.5856 |
| TTLL5 | -0.2389 | 4.4376 | -5.1111 | 0.0000 | 0.0000 | 5.5831 |
| HS3ST1 | -0.5232 | 2.6523 | -5.1101 | 0.0000 | 0.0000 | 5.5782 |
| ORC2 | -0.1818 | 5.4150 | -5.1075 | 0.0000 | 0.0000 | 5.5659 |
| FYN | 0.2659 | 5.4782 | 5.1069 | 0.0000 | 0.0000 | 5.5632 |
| MAOA | 0.2510 | 6.8812 | 5.1057 | 0.0000 | 0.0000 | 5.5572 |
| TCEA1 | 0.1176 | 6.6125 | 5.1055 | 0.0000 | 0.0000 | 5.5563 |
| ADRA1B | 0.6862 | 4.7190 | 5.1050 | 0.0000 | 0.0000 | 5.5541 |
| CEACAM1 | 0.2991 | 6.5138 | 5.1050 | 0.0000 | 0.0000 | 5.5538 |
| DYSF | 0.2629 | 5.9844 | 5.1041 | 0.0000 | 0.0000 | 5.5495 |
| DHCR7 | 0.2057 | 7.1350 | 5.1015 | 0.0000 | 0.0000 | 5.5372 |
| RBM12 | -0.1187 | 6.0770 | -5.1011 | 0.0000 | 0.0000 | 5.5357 |
| TPD52L2 | -0.0897 | 6.7287 | -5.0989 | 0.0000 | 0.0000 | 5.5253 |
| RBPJ | -0.1197 | 5.8577 | -5.0979 | 0.0000 | 0.0000 | 5.5202 |
| RBBP4 | -0.1256 | 6.3505 | -5.0975 | 0.0000 | 0.0000 | 5.5183 |
| TGFB1 | -0.2928 | 6.2645 | -5.0949 | 0.0000 | 0.0000 | 5.5063 |
| SEMA3A | -0.6471 | 1.6762 | -5.0949 | 0.0000 | 0.0000 | 5.5061 |

| GALNT3 | -0.6913 | 2.4817 | -5.0938 | 0.0000 | 0.0000 | 5.5007 |
| --- | --- | --- | --- | --- | --- | --- |
| GDF10 | -0.9314 | 1.5755 | -5.0928 | 0.0000 | 0.0000 | 5.4960 |
| LRRC36 | -0.5216 | 0.9841 | -5.0922 | 0.0000 | 0.0000 | 5.4934 |
| BRSK2 | -0.7622 | 1.4766 | -5.0918 | 0.0000 | 0.0000 | 5.4914 |
| OR2H2 | -0.3455 | 0.2821 | -5.0904 | 0.0000 | 0.0000 | 5.4851 |
| CNNM4 | -0.2292 | 5.2840 | -5.0903 | 0.0000 | 0.0000 | 5.4845 |
| CEP152 | -0.3996 | 3.3195 | -5.0894 | 0.0000 | 0.0000 | 5.4803 |
| ACR | 0.5738 | 2.2647 | 5.0893 | 0.0000 | 0.0000 | 5.4796 |
| GPR17 | 0.6917 | 2.2513 | 5.0889 | 0.0000 | 0.0000 | 5.4778 |
| TMEM184B | -0.1400 | 6.2806 | -5.0887 | 0.0000 | 0.0000 | 5.4769 |
| BAZ2A | -0.1623 | 5.9897 | -5.0875 | 0.0000 | 0.0000 | 5.4711 |
| NPEPL1 | -0.2603 | 4.8876 | -5.0870 | 0.0000 | 0.0000 | 5.4688 |
| CKM | -0.5257 | 1.2410 | -5.0865 | 0.0000 | 0.0000 | 5.4665 |
| KIAA0319 | -0.4525 | 1.1312 | -5.0859 | 0.0000 | 0.0000 | 5.4634 |
| NCAM1 | -0.7009 | 1.6762 | -5.0856 | 0.0000 | 0.0000 | 5.4622 |
| NDUFA5 | 0.1543 | 6.1159 | 5.0849 | 0.0000 | 0.0000 | 5.4590 |
| LHX1 | -0.3389 | 0.1994 | -5.0845 | 0.0000 | 0.0000 | 5.4570 |
| SPINT2 | -0.6545 | 4.9482 | -5.0825 | 0.0000 | 0.0000 | 5.4477 |
| KLHDC3 | 0.0953 | 7.2769 | 5.0821 | 0.0000 | 0.0000 | 5.4454 |
| SNRPD1 | -0.1539 | 6.2023 | -5.0816 | 0.0000 | 0.0000 | 5.4432 |
| MTIF2 | 0.0874 | 6.5915 | 5.0811 | 0.0000 | 0.0000 | 5.4409 |
| HKDC1 | -0.7726 | 5.7710 | -5.0800 | 0.0000 | 0.0000 | 5.4357 |
| DUSP3 | 0.0934 | 6.9865 | 5.0800 | 0.0000 | 0.0000 | 5.4357 |
| DTX3 | -0.4566 | 4.7775 | -5.0797 | 0.0000 | 0.0000 | 5.4343 |
| FXN | 0.2038 | 5.8537 | 5.0776 | 0.0000 | 0.0000 | 5.4242 |
| ILF3 | -0.0812 | 6.6138 | -5.0745 | 0.0000 | 0.0000 | 5.4100 |
| SERPINA1 | 0.1456 | 8.2621 | 5.0744 | 0.0000 | 0.0000 | 5.4094 |
| LHX3 | 0.9254 | 1.4856 | 5.0703 | 0.0000 | 0.0000 | 5.3902 |
| MORF4L2 | 0.0717 | 7.3067 | 5.0684 | 0.0000 | 0.0000 | 5.3811 |
| KRT3 | -0.1882 | 0.1007 | -5.0680 | 0.0000 | 0.0000 | 5.3792 |
| PSMB5 | 0.0783 | 7.2939 | 5.0665 | 0.0000 | 0.0000 | 5.3719 |
| PTGR2 | 0.2056 | 5.3848 | 5.0653 | 0.0000 | 0.0000 | 5.3665 |
| ATP5I | 0.1197 | 7.4942 | 5.0637 | 0.0000 | 0.0000 | 5.3591 |
| MAGI1 | 0.1995 | 5.5220 | 5.0630 | 0.0000 | 0.0000 | 5.3556 |
| MPST | 0.1468 | 7.1513 | 5.0619 | 0.0000 | 0.0000 | 5.3502 |
| GRHL2 | -0.7622 | 1.1390 | -5.0616 | 0.0000 | 0.0000 | 5.3491 |
| PARK7 | 0.1164 | 7.3547 | 5.0615 | 0.0000 | 0.0000 | 5.3483 |
| EPM2A | 0.2837 | 4.4753 | 5.0614 | 0.0000 | 0.0000 | 5.3483 |
| HLA-B | 0.1336 | 7.8142 | 5.0611 | 0.0000 | 0.0000 | 5.3467 |
| TUBG2 | -0.1925 | 5.7339 | -5.0602 | 0.0000 | 0.0000 | 5.3425 |
| GRIA4 | -0.2110 | 0.1731 | -5.0556 | 0.0000 | 0.0000 | 5.3209 |
| NUP205 | -0.1894 | 5.7463 | -5.0532 | 0.0000 | 0.0000 | 5.3096 |
| ANKRA2 | 0.1874 | 5.9456 | 5.0518 | 0.0000 | 0.0000 | 5.3031 |
| SALL1 | 0.3618 | 6.2081 | 5.0506 | 0.0000 | 0.0000 | 5.2973 |
| **CA12** | -1.0171 | 4.0016 | -5.0505 | 0.0000 | 0.0000 | 5.2970 |
| HRH1 | -0.4341 | 3.1105 | -5.0492 | 0.0000 | 0.0000 | 5.2908 |
| WDR91 | -0.1961 | 5.6604 | -5.0486 | 0.0000 | 0.0000 | 5.2877 |
| PRUNE2 | -0.5654 | 1.7250 | -5.0483 | 0.0000 | 0.0000 | 5.2863 |
| AGMAT | 0.2963 | 6.8356 | 5.0479 | 0.0000 | 0.0000 | 5.2845 |
| GALNT12 | -0.7696 | 2.4106 | -5.0469 | 0.0000 | 0.0000 | 5.2801 |
| C1D | 0.1373 | 5.8329 | 5.0458 | 0.0000 | 0.0000 | 5.2746 |
| GABRQ | -0.5221 | 1.1938 | -5.0456 | 0.0000 | 0.0000 | 5.2740 |
| CD81 | 0.1500 | 7.2568 | 5.0453 | 0.0000 | 0.0000 | 5.2725 |

| RFC5 | -0.1681 | 5.8809 | -5.0447 | 0.0000 | 0.0000 | 5.2695 |
| --- | --- | --- | --- | --- | --- | --- |
| PHC1 | -0.4662 | 3.1987 | -5.0436 | 0.0000 | 0.0000 | 5.2644 |
| SULT1A2 | 0.5406 | 5.8130 | 5.0425 | 0.0000 | 0.0000 | 5.2593 |
| TMPRSS4 | -0.4923 | 0.7580 | -5.0416 | 0.0000 | 0.0000 | 5.2552 |
| TLR5 | -0.5278 | 4.1128 | -5.0397 | 0.0000 | 0.0000 | 5.2460 |
| DUSP13 | -0.7003 | 1.0855 | -5.0389 | 0.0000 | 0.0000 | 5.2424 |
| NR1H3 | 0.1281 | 6.6542 | 5.0380 | 0.0000 | 0.0000 | 5.2384 |
| KCNV1 | -0.2205 | 0.1291 | -5.0380 | 0.0000 | 0.0000 | 5.2383 |
| SLC4A8 | -0.2899 | 0.7884 | -5.0376 | 0.0000 | 0.0000 | 5.2365 |
| TNFRSF9 | -0.7416 | 2.3978 | -5.0371 | 0.0000 | 0.0000 | 5.2341 |
| FUT7 | -0.5676 | 1.7165 | -5.0359 | 0.0000 | 0.0000 | 5.2286 |
| MLF1 | -0.6765 | 2.7419 | -5.0359 | 0.0000 | 0.0000 | 5.2283 |
| GTPBP10 | 0.1738 | 5.2306 | 5.0347 | 0.0000 | 0.0000 | 5.2225 |
| DNAJA3 | 0.1220 | 6.4853 | 5.0345 | 0.0000 | 0.0000 | 5.2217 |
| NEUROG3 | -0.3248 | 0.3160 | -5.0343 | 0.0000 | 0.0000 | 5.2208 |
| TXNRD3 | 0.1756 | 5.5089 | 5.0339 | 0.0000 | 0.0000 | 5.2191 |
| DMD | 0.4482 | 4.9612 | 5.0334 | 0.0000 | 0.0000 | 5.2167 |
| ZNF292 | -0.3443 | 4.6334 | -5.0328 | 0.0000 | 0.0000 | 5.2138 |
| BLOC1S1 | 0.1330 | 7.0265 | 5.0319 | 0.0000 | 0.0000 | 5.2098 |
| C6orf62 | 0.0834 | 7.1021 | 5.0296 | 0.0000 | 0.0000 | 5.1989 |
| RUSC2 | -0.1973 | 5.8011 | -5.0294 | 0.0000 | 0.0000 | 5.1979 |
| ZBED1 | 0.1152 | 6.6756 | 5.0289 | 0.0000 | 0.0000 | 5.1954 |
| KDM5C | -0.1217 | 6.2891 | -5.0284 | 0.0000 | 0.0000 | 5.1932 |
| YIPF3 | 0.0852 | 7.2254 | 5.0272 | 0.0000 | 0.0000 | 5.1879 |
| TMEM53 | 0.1759 | 6.3096 | 5.0265 | 0.0000 | 0.0000 | 5.1845 |
| EHBP1 | 0.1890 | 6.1444 | 5.0262 | 0.0000 | 0.0000 | 5.1831 |
| SLC6A9 | -0.6124 | 4.0603 | -5.0256 | 0.0000 | 0.0000 | 5.1799 |
| DDC | 0.6488 | 6.2051 | 5.0242 | 0.0000 | 0.0000 | 5.1738 |
| MRPS33 | 0.1399 | 6.3008 | 5.0208 | 0.0000 | 0.0000 | 5.1579 |
| CCL15 | 0.5007 | 6.3579 | 5.0203 | 0.0000 | 0.0000 | 5.1553 |
| HPN | 0.2710 | 7.4601 | 5.0190 | 0.0000 | 0.0000 | 5.1491 |
| GSDMD | 0.1274 | 7.0075 | 5.0180 | 0.0000 | 0.0000 | 5.1447 |
| SMAD9 | -0.4843 | 2.9808 | -5.0180 | 0.0000 | 0.0000 | 5.1446 |
| RMND1 | 0.1333 | 6.4295 | 5.0174 | 0.0000 | 0.0000 | 5.1417 |
| AGT | 0.1299 | 8.0426 | 5.0164 | 0.0000 | 0.0000 | 5.1371 |
| TGFBR2 | 0.1638 | 6.6985 | 5.0160 | 0.0000 | 0.0000 | 5.1355 |
| GPR1 | -0.5694 | 0.9879 | -5.0154 | 0.0000 | 0.0000 | 5.1324 |
| FAM32A | 0.0752 | 7.1112 | 5.0152 | 0.0000 | 0.0000 | 5.1317 |
| NFIX | 0.2067 | 6.3746 | 5.0150 | 0.0000 | 0.0000 | 5.1308 |
| SND1 | 0.0788 | 7.1209 | 5.0142 | 0.0000 | 0.0000 | 5.1268 |
| C8G | 0.2611 | 7.6328 | 5.0131 | 0.0000 | 0.0000 | 5.1218 |
| MRPS27 | 0.1127 | 6.4109 | 5.0121 | 0.0000 | 0.0000 | 5.1170 |
| CBS | 0.6397 | 4.4240 | 5.0114 | 0.0000 | 0.0000 | 5.1137 |
| ZFHX2 | -0.4249 | 1.9644 | -5.0112 | 0.0000 | 0.0000 | 5.1128 |
| RYR1 | -0.4894 | 1.4754 | -5.0104 | 0.0000 | 0.0000 | 5.1092 |
| COX5A | 0.0980 | 7.3669 | 5.0103 | 0.0000 | 0.0000 | 5.1088 |
| WNT10B | -0.5883 | 1.7030 | -5.0101 | 0.0000 | 0.0000 | 5.1080 |
| OXSM | 0.1249 | 6.1472 | 5.0098 | 0.0000 | 0.0000 | 5.1062 |
| AGPAT4 | -0.5723 | 3.4829 | -5.0090 | 0.0000 | 0.0000 | 5.1029 |
| PTGES3 | 0.0614 | 7.3543 | 5.0078 | 0.0000 | 0.0000 | 5.0973 |
| TNMD | 0.4872 | 0.8311 | 5.0063 | 0.0000 | 0.0000 | 5.0903 |
| DGKH | -0.3665 | 3.2688 | -5.0049 | 0.0000 | 0.0000 | 5.0834 |
| NUCKS1 | 0.0817 | 7.2091 | 5.0032 | 0.0000 | 0.0000 | 5.0759 |

| ACOXL | -0.3338 | 0.7346 | -5.0030 | 0.0000 | 0.0000 | 5.0748 |
| --- | --- | --- | --- | --- | --- | --- |
| TEAD4 | -0.4324 | 5.1220 | -5.0003 | 0.0000 | 0.0000 | 5.0623 |
| PEX12 | 0.2145 | 5.6683 | 4.9998 | 0.0000 | 0.0000 | 5.0599 |
| ETV1 | -0.6245 | 3.9511 | -4.9984 | 0.0000 | 0.0000 | 5.0531 |
| SLC26A2 | -0.3306 | 4.6486 | -4.9979 | 0.0000 | 0.0000 | 5.0510 |
| AKR1B1 | -0.3435 | 5.8419 | -4.9966 | 0.0000 | 0.0000 | 5.0451 |
| COX4I1 | 0.1196 | 7.3113 | 4.9966 | 0.0000 | 0.0000 | 5.0451 |
| PRMT1 | -0.1138 | 6.6039 | -4.9951 | 0.0000 | 0.0000 | 5.0379 |
| NCS1 | -0.5691 | 4.7045 | -4.9937 | 0.0000 | 0.0000 | 5.0315 |
| MCM7 | -0.1381 | 6.7561 | -4.9933 | 0.0000 | 0.0000 | 5.0298 |
| RPP14 | 0.1353 | 5.4627 | 4.9931 | 0.0000 | 0.0000 | 5.0287 |
| CHRNA1 | -0.5694 | 1.0007 | -4.9931 | 0.0000 | 0.0000 | 5.0284 |
| CNGB3 | -0.4117 | 0.5904 | -4.9906 | 0.0000 | 0.0000 | 5.0171 |
| MDC1 | -0.1876 | 5.8201 | -4.9898 | 0.0000 | 0.0000 | 5.0135 |
| UCP3 | -0.3519 | 2.4890 | -4.9896 | 0.0000 | 0.0000 | 5.0123 |
| MIA-RAB4B | -0.1864 | 0.1446 | -4.9874 | 0.0000 | 0.0000 | 5.0019 |
| ALPL | 0.5392 | 6.1429 | 4.9868 | 0.0000 | 0.0000 | 4.9993 |
| UGT2B4 | 0.3779 | 7.5994 | 4.9866 | 0.0000 | 0.0000 | 4.9985 |
| CCDC93 | -0.1890 | 5.5096 | -4.9842 | 0.0000 | 0.0000 | 4.9871 |
| PDE3B | 0.3868 | 5.3570 | 4.9841 | 0.0000 | 0.0000 | 4.9869 |
| AATK | -0.6166 | 3.0806 | -4.9834 | 0.0000 | 0.0000 | 4.9836 |
| YPEL5 | 0.0802 | 6.8561 | 4.9831 | 0.0000 | 0.0000 | 4.9820 |
| IL2RA | -0.6758 | 2.9571 | -4.9830 | 0.0000 | 0.0000 | 4.9816 |
| TIAM2 | -0.4398 | 3.4029 | -4.9825 | 0.0000 | 0.0000 | 4.9792 |
| ATP5F1 | 0.0853 | 7.0803 | 4.9813 | 0.0000 | 0.0000 | 4.9737 |
| CEACAM3 | -0.4165 | 0.8950 | -4.9805 | 0.0000 | 0.0000 | 4.9699 |
| GTF2IRD1 | -0.2888 | 5.2837 | -4.9801 | 0.0000 | 0.0000 | 4.9685 |
| KRCC1 | 0.1352 | 6.6136 | 4.9801 | 0.0000 | 0.0000 | 4.9683 |
| OPRL1 | -0.4947 | 3.0120 | -4.9799 | 0.0000 | 0.0000 | 4.9673 |
| PREB | 0.1123 | 7.0260 | 4.9782 | 0.0000 | 0.0000 | 4.9597 |
| LY6H | -0.7362 | 2.5756 | -4.9782 | 0.0000 | 0.0000 | 4.9594 |
| JUND | 0.1198 | 7.4005 | 4.9768 | 0.0000 | 0.0000 | 4.9529 |
| TLK2 | -0.1280 | 5.4365 | -4.9757 | 0.0000 | 0.0000 | 4.9476 |
| IQCE | -0.2407 | 5.0964 | -4.9754 | 0.0000 | 0.0000 | 4.9467 |
| PDIA4 | 0.0872 | 7.5298 | 4.9743 | 0.0000 | 0.0000 | 4.9415 |
| TCP10L | 0.8129 | 4.1312 | 4.9731 | 0.0000 | 0.0000 | 4.9359 |
| EYA1 | -0.5498 | 0.6811 | -4.9729 | 0.0000 | 0.0000 | 4.9349 |
| NRIP3 | -0.4512 | 1.6057 | -4.9729 | 0.0000 | 0.0000 | 4.9348 |
| IL21R | -0.6583 | 2.2697 | -4.9710 | 0.0000 | 0.0000 | 4.9260 |
| B4GALT1 | 0.1049 | 7.0295 | 4.9699 | 0.0000 | 0.0000 | 4.9210 |
| IFIT3 | 0.2312 | 6.3808 | 4.9693 | 0.0000 | 0.0000 | 4.9184 |
| TCEAL4 | 0.1039 | 6.9312 | 4.9693 | 0.0000 | 0.0000 | 4.9184 |
| PSMB3 | 0.1015 | 7.5139 | 4.9693 | 0.0000 | 0.0000 | 4.9182 |
| TNIP3 | -0.4859 | 0.9705 | -4.9686 | 0.0000 | 0.0000 | 4.9149 |
| ITGA5 | -0.1504 | 6.6020 | -4.9679 | 0.0000 | 0.0000 | 4.9119 |
| SIPA1L3 | -0.2784 | 5.4408 | -4.9668 | 0.0000 | 0.0000 | 4.9065 |
| CHMP2A | 0.1091 | 7.3039 | 4.9667 | 0.0000 | 0.0000 | 4.9063 |
| ZNF473 | -0.1968 | 4.8147 | -4.9665 | 0.0000 | 0.0000 | 4.9054 |
| CT62 | -0.2812 | 0.3401 | -4.9650 | 0.0000 | 0.0000 | 4.8984 |
| ACTL6A | -0.1552 | 6.1911 | -4.9649 | 0.0000 | 0.0000 | 4.8980 |
| FGA | 0.2827 | 8.1120 | 4.9625 | 0.0000 | 0.0000 | 4.8867 |
| IARS2 | 0.1036 | 7.0439 | 4.9620 | 0.0000 | 0.0000 | 4.8844 |
| S100A6 | -0.2226 | 7.1144 | -4.9618 | 0.0000 | 0.0000 | 4.8833 |

| CA2 | 0.3044 | 6.7092 | 4.9601 | 0.0000 | 0.0000 | 4.8759 |
| --- | --- | --- | --- | --- | --- | --- |
| NUP155 | -0.1722 | 5.5391 | -4.9593 | 0.0000 | 0.0000 | 4.8722 |
| FKBP2 | 0.1213 | 7.3100 | 4.9583 | 0.0000 | 0.0000 | 4.8671 |
| PSMB1 | 0.1046 | 7.3102 | 4.9580 | 0.0000 | 0.0000 | 4.8658 |
| LIN7A | 0.2950 | 5.8734 | 4.9574 | 0.0000 | 0.0000 | 4.8631 |
| CSTF2 | -0.1671 | 5.8985 | -4.9549 | 0.0000 | 0.0000 | 4.8515 |
| RRAGA | 0.0840 | 7.1113 | 4.9548 | 0.0000 | 0.0000 | 4.8513 |
| PSD4 | 0.2056 | 5.9762 | 4.9537 | 0.0000 | 0.0000 | 4.8460 |
| TFRC | -0.1928 | 6.4194 | -4.9530 | 0.0000 | 0.0000 | 4.8427 |
| PDCD1 | -0.7911 | 3.5252 | -4.9529 | 0.0000 | 0.0000 | 4.8425 |
| TMEM45A | -0.7018 | 5.2639 | -4.9528 | 0.0000 | 0.0000 | 4.8421 |
| DBI | 0.1010 | 7.3579 | 4.9517 | 0.0000 | 0.0000 | 4.8370 |
| CYB5R2 | -0.5006 | 3.4696 | -4.9517 | 0.0000 | 0.0000 | 4.8367 |
| TMEM143 | 0.1987 | 5.7158 | 4.9505 | 0.0000 | 0.0000 | 4.8312 |
| POLR2K | 0.1071 | 7.0049 | 4.9500 | 0.0000 | 0.0000 | 4.8291 |
| SCG2 | -0.6814 | 1.5757 | -4.9485 | 0.0000 | 0.0000 | 4.8223 |
| SLC17A4 | 0.5135 | 6.1960 | 4.9477 | 0.0000 | 0.0000 | 4.8183 |
| GLYR1 | 0.1088 | 6.6317 | 4.9471 | 0.0000 | 0.0000 | 4.8156 |
| HSPD1 | 0.0820 | 7.5117 | 4.9467 | 0.0000 | 0.0000 | 4.8136 |
| CISH | 0.2733 | 6.0512 | 4.9431 | 0.0000 | 0.0000 | 4.7970 |
| SH3TC1 | -0.2347 | 5.4551 | -4.9425 | 0.0000 | 0.0000 | 4.7943 |
| CTSB | 0.1075 | 7.5102 | 4.9413 | 0.0000 | 0.0000 | 4.7890 |
| MFSD1 | 0.1125 | 6.4906 | 4.9404 | 0.0000 | 0.0000 | 4.7849 |
| LHX5 | -0.2036 | 0.1682 | -4.9399 | 0.0000 | 0.0000 | 4.7824 |
| ANGPT2 | -0.4255 | 4.4887 | -4.9394 | 0.0000 | 0.0000 | 4.7802 |
| AKR1C1 | 0.3631 | 7.0315 | 4.9387 | 0.0000 | 0.0000 | 4.7768 |
| **DHRS2** | 1.0523 | 4.6172 | 4.9385 | 0.0000 | 0.0000 | 4.7759 |
| CPA4 | -0.5848 | 1.0978 | -4.9373 | 0.0000 | 0.0000 | 4.7706 |
| 43891.0000 | -0.3964 | 3.3393 | -4.9360 | 0.0000 | 0.0000 | 4.7646 |
| R3HDM1 | -0.1578 | 5.4679 | -4.9357 | 0.0000 | 0.0000 | 4.7631 |
| VKORC1 | 0.1447 | 6.9513 | 4.9343 | 0.0000 | 0.0000 | 4.7566 |
| TOPBP1 | -0.1976 | 5.7210 | -4.9335 | 0.0000 | 0.0000 | 4.7530 |
| CSDE1 | 0.0711 | 7.2531 | 4.9316 | 0.0000 | 0.0000 | 4.7443 |
| CLGN | -0.8867 | 4.8836 | -4.9315 | 0.0000 | 0.0000 | 4.7439 |
| CER1 | -0.3600 | 0.1733 | -4.9313 | 0.0000 | 0.0000 | 4.7429 |
| SLC30A10 | 0.4385 | 6.0623 | 4.9292 | 0.0000 | 0.0000 | 4.7333 |
| GNA15 | -0.4368 | 4.3983 | -4.9291 | 0.0000 | 0.0000 | 4.7328 |
| GABRA5 | -0.4290 | 0.2772 | -4.9288 | 0.0000 | 0.0000 | 4.7312 |
| PYCARD | -0.3744 | 6.0572 | -4.9273 | 0.0000 | 0.0000 | 4.7247 |
| ELOVL2 | 0.4103 | 6.6080 | 4.9260 | 0.0000 | 0.0000 | 4.7187 |
| ASGR2 | 0.2625 | 7.5795 | 4.9256 | 0.0000 | 0.0000 | 4.7167 |
| PNRC1 | 0.1134 | 6.9865 | 4.9252 | 0.0000 | 0.0000 | 4.7149 |
| KIAA0907 | -0.1720 | 5.9748 | -4.9248 | 0.0000 | 0.0000 | 4.7128 |
| GLP1R | -0.8042 | 1.0267 | -4.9247 | 0.0000 | 0.0000 | 4.7125 |
| LIN7C | 0.1123 | 6.2608 | 4.9247 | 0.0000 | 0.0000 | 4.7124 |
| SEC24B | 0.1902 | 6.1837 | 4.9231 | 0.0000 | 0.0000 | 4.7051 |
| IGSF1 | -0.8525 | 3.3895 | -4.9228 | 0.0000 | 0.0000 | 4.7037 |
| MTHFD2 | -0.4521 | 4.4492 | -4.9223 | 0.0000 | 0.0000 | 4.7017 |
| UBAP1 | 0.0789 | 6.8175 | 4.9222 | 0.0000 | 0.0000 | 4.7009 |
| TAPBP | 0.0939 | 7.2861 | 4.9218 | 0.0000 | 0.0000 | 4.6994 |
| ARNT | 0.1249 | 6.4979 | 4.9210 | 0.0000 | 0.0000 | 4.6957 |
| PML | -0.1585 | 5.6723 | -4.9201 | 0.0000 | 0.0000 | 4.6914 |
| RTN1 | -0.5536 | 3.2555 | -4.9192 | 0.0000 | 0.0000 | 4.6872 |

| TP53BP1 | -0.4251 | 4.3088 | -4.9191 | 0.0000 | 0.0000 | 4.6867 |
| --- | --- | --- | --- | --- | --- | --- |
| HSPA1A | 0.1557 | 7.1732 | 4.9186 | 0.0000 | 0.0000 | 4.6847 |
| ILVBL | 0.1366 | 6.7109 | 4.9173 | 0.0000 | 0.0000 | 4.6785 |
| AOX1 | 0.4666 | 7.1484 | 4.9157 | 0.0000 | 0.0000 | 4.6715 |
| TCEAL1 | 0.1834 | 6.2670 | 4.9146 | 0.0000 | 0.0000 | 4.6662 |
| SLC35D2 | 0.1188 | 6.8827 | 4.9137 | 0.0000 | 0.0000 | 4.6622 |
| AUP1 | 0.0806 | 7.2871 | 4.9118 | 0.0000 | 0.0000 | 4.6533 |
| VOPP1 | -0.1719 | 6.0228 | -4.9114 | 0.0000 | 0.0000 | 4.6516 |
| NMB | -0.3301 | 5.6798 | -4.9111 | 0.0000 | 0.0000 | 4.6504 |
| SLC2A4RG | 0.1145 | 7.2420 | 4.9107 | 0.0000 | 0.0000 | 4.6481 |
| SMC4 | -0.4818 | 5.0485 | -4.9104 | 0.0000 | 0.0000 | 4.6467 |
| HSP90B1 | 0.0776 | 7.7142 | 4.9100 | 0.0000 | 0.0000 | 4.6451 |
| UTP20 | -0.2195 | 5.0906 | -4.9087 | 0.0000 | 0.0000 | 4.6393 |
| AIMP2 | 0.1313 | 6.6117 | 4.9084 | 0.0000 | 0.0000 | 4.6380 |
| RAPGEF3 | 0.3405 | 3.8511 | 4.9080 | 0.0000 | 0.0000 | 4.6359 |
| GPR85 | -0.4195 | 1.5957 | -4.9075 | 0.0000 | 0.0000 | 4.6338 |
| TFPI | 0.3082 | 6.7030 | 4.9075 | 0.0000 | 0.0000 | 4.6336 |
| PPP5C | -0.1049 | 6.2250 | -4.9065 | 0.0000 | 0.0000 | 4.6290 |
| CENPJ | -0.3137 | 4.4755 | -4.9062 | 0.0000 | 0.0000 | 4.6276 |
| NSA2 | 0.1082 | 6.6235 | 4.9041 | 0.0000 | 0.0000 | 4.6179 |
| LRP5 | 0.1359 | 6.9384 | 4.9037 | 0.0000 | 0.0000 | 4.6163 |
| IMPAD1 | 0.1431 | 6.6675 | 4.9031 | 0.0000 | 0.0000 | 4.6135 |
| MDFI | -0.7050 | 3.8990 | -4.9028 | 0.0000 | 0.0000 | 4.6120 |
| CYP2U1 | 0.3887 | 4.6342 | 4.9010 | 0.0000 | 0.0000 | 4.6039 |
| CDR1 | -0.2335 | 0.1547 | -4.8989 | 0.0000 | 0.0000 | 4.5944 |
| SSBP2 | -0.5413 | 3.3053 | -4.8980 | 0.0000 | 0.0000 | 4.5901 |
| PCDH12 | 0.2581 | 5.3604 | 4.8973 | 0.0000 | 0.0000 | 4.5868 |
| CRHBP | 0.7879 | 3.3890 | 4.8955 | 0.0000 | 0.0000 | 4.5789 |
| SNX2 | 0.0942 | 6.5776 | 4.8955 | 0.0000 | 0.0000 | 4.5788 |
| ACTA1 | -0.5226 | 2.3645 | -4.8955 | 0.0000 | 0.0000 | 4.5786 |
| DDX24 | 0.0967 | 6.5198 | 4.8955 | 0.0000 | 0.0000 | 4.5786 |
| ADAM9 | -0.3151 | 5.7852 | -4.8948 | 0.0000 | 0.0000 | 4.5756 |
| TK1 | -0.2172 | 6.6524 | -4.8946 | 0.0000 | 0.0000 | 4.5745 |
| S100A9 | -0.3507 | 6.4177 | -4.8923 | 0.0000 | 0.0000 | 4.5643 |
| TCEA2 | 0.2333 | 6.3458 | 4.8920 | 0.0000 | 0.0000 | 4.5629 |
| TRAPPC9 | 0.1583 | 6.1091 | 4.8918 | 0.0000 | 0.0000 | 4.5620 |
| PELI1 | -0.2716 | 5.7368 | -4.8909 | 0.0000 | 0.0000 | 4.5578 |
| **DKK1** | -1.1467 | 3.3435 | -4.8908 | 0.0000 | 0.0000 | 4.5574 |
| TCP11 | 0.4098 | 1.0120 | 4.8898 | 0.0000 | 0.0000 | 4.5526 |
| WDR45 | 0.1127 | 6.5071 | 4.8889 | 0.0000 | 0.0000 | 4.5487 |
| CDKN1C | -0.5372 | 4.8126 | -4.8885 | 0.0000 | 0.0000 | 4.5466 |
| SMARCA4 | -0.1253 | 6.2643 | -4.8884 | 0.0000 | 0.0000 | 4.5465 |
| **CXCL6** | -1.2179 | 3.2358 | -4.8883 | 0.0000 | 0.0000 | 4.5460 |
| GUK1 | 0.1148 | 7.1501 | 4.8835 | 0.0000 | 0.0000 | 4.5241 |
| CEBPA | 0.1880 | 7.2257 | 4.8826 | 0.0000 | 0.0000 | 4.5200 |
| NUP43 | -0.1334 | 6.0033 | -4.8826 | 0.0000 | 0.0000 | 4.5197 |
| SH2D4A | 0.2316 | 6.1441 | 4.8824 | 0.0000 | 0.0000 | 4.5192 |
| ARID3A | -0.5382 | 4.7706 | -4.8811 | 0.0000 | 0.0000 | 4.5131 |
| CRLF1 | -0.5127 | 3.3673 | -4.8804 | 0.0000 | 0.0000 | 4.5099 |
| CBL | -0.3409 | 4.4723 | -4.8786 | 0.0000 | 0.0000 | 4.5015 |
| MGAT4A | -0.2349 | 5.4839 | -4.8782 | 0.0000 | 0.0000 | 4.4997 |
| NFX1 | 0.1108 | 6.1583 | 4.8776 | 0.0000 | 0.0000 | 4.4972 |
| CELA3B | -0.4554 | 0.5092 | -4.8771 | 0.0000 | 0.0000 | 4.4949 |

| SRPX2 | -0.5381 | 4.5436 | -4.8761 | 0.0000 | 0.0000 | 4.4902 |
| --- | --- | --- | --- | --- | --- | --- |
| NPEPPS | -0.1712 | 5.7738 | -4.8758 | 0.0000 | 0.0000 | 4.4889 |
| LLPH | -0.1161 | 5.3931 | -4.8751 | 0.0000 | 0.0000 | 4.4859 |
| DTYMK | -0.1462 | 6.4821 | -4.8745 | 0.0000 | 0.0000 | 4.4831 |
| HOXB6 | -0.5191 | 3.1194 | -4.8737 | 0.0000 | 0.0000 | 4.4793 |
| MYLK | 0.3493 | 5.5692 | 4.8734 | 0.0000 | 0.0000 | 4.4780 |
| CDS1 | -0.9119 | 3.5235 | -4.8719 | 0.0000 | 0.0000 | 4.4714 |
| ZNF35 | -0.3393 | 4.5701 | -4.8719 | 0.0000 | 0.0000 | 4.4710 |
| DPM1 | 0.0787 | 6.8906 | 4.8712 | 0.0000 | 0.0000 | 4.4681 |
| ITIH5 | -0.8362 | 2.8851 | -4.8707 | 0.0000 | 0.0000 | 4.4655 |
| PLAC4 | -0.3456 | 0.2741 | -4.8697 | 0.0000 | 0.0000 | 4.4609 |
| S100A14 | -0.7122 | 5.8264 | -4.8678 | 0.0000 | 0.0000 | 4.4527 |
| FST | 0.4129 | 6.8223 | 4.8674 | 0.0000 | 0.0000 | 4.4508 |
| ACTR1B | 0.0768 | 6.8369 | 4.8661 | 0.0000 | 0.0000 | 4.4449 |
| CHMP5 | 0.0824 | 6.8122 | 4.8655 | 0.0000 | 0.0000 | 4.4422 |
| NME6 | -0.1404 | 5.5349 | -4.8653 | 0.0000 | 0.0000 | 4.4410 |
| PSORS1C2 | -0.3947 | 0.4887 | -4.8635 | 0.0000 | 0.0000 | 4.4327 |
| C9orf78 | 0.0935 | 6.5217 | 4.8629 | 0.0000 | 0.0000 | 4.4303 |
| HYAL4 | -0.3973 | 0.4604 | -4.8624 | 0.0000 | 0.0000 | 4.4279 |
| TECR | 0.1093 | 6.8791 | 4.8616 | 0.0000 | 0.0000 | 4.4244 |
| GAS2 | 0.3980 | 5.7158 | 4.8609 | 0.0000 | 0.0000 | 4.4211 |
| KIAA0753 | -0.2628 | 4.4811 | -4.8607 | 0.0000 | 0.0000 | 4.4203 |
| OR2H1 | -0.2961 | 0.2317 | -4.8605 | 0.0000 | 0.0000 | 4.4195 |
| GSN | 0.1836 | 6.6450 | 4.8591 | 0.0000 | 0.0000 | 4.4129 |
| ZNF668 | -0.3703 | 4.0186 | -4.8584 | 0.0000 | 0.0000 | 4.4096 |
| TSPAN31 | 0.1861 | 6.1214 | 4.8581 | 0.0000 | 0.0000 | 4.4083 |
| KLK10 | -0.5729 | 0.8467 | -4.8574 | 0.0000 | 0.0000 | 4.4054 |
| DNAJB12 | 0.0920 | 6.4178 | 4.8574 | 0.0000 | 0.0000 | 4.4051 |
| RENBP | -0.4120 | 5.5983 | -4.8570 | 0.0000 | 0.0000 | 4.4034 |
| TXNIP | 0.1761 | 7.1858 | 4.8562 | 0.0000 | 0.0000 | 4.3996 |
| PSMC6 | 0.0888 | 6.2879 | 4.8548 | 0.0000 | 0.0000 | 4.3934 |
| MAP1S | -0.1312 | 6.0802 | -4.8544 | 0.0000 | 0.0000 | 4.3918 |
| NAPA | 0.1214 | 6.6752 | 4.8539 | 0.0000 | 0.0000 | 4.3895 |
| POU3F2 | -0.4952 | 0.7250 | -4.8533 | 0.0000 | 0.0000 | 4.3867 |
| SLC25A16 | 0.1712 | 5.7415 | 4.8529 | 0.0000 | 0.0000 | 4.3847 |
| FAM114A2 | 0.1413 | 5.5742 | 4.8519 | 0.0000 | 0.0000 | 4.3802 |
| TWIST1 | -0.6209 | 2.0572 | -4.8517 | 0.0000 | 0.0000 | 4.3793 |
| C16orf95 | 0.3475 | 3.6731 | 4.8516 | 0.0000 | 0.0000 | 4.3790 |
| HOMER2 | 0.5014 | 5.4040 | 4.8516 | 0.0000 | 0.0000 | 4.3788 |
| CRISP3 | -0.8433 | 1.1318 | -4.8515 | 0.0000 | 0.0000 | 4.3784 |
| VAV3 | -0.5299 | 3.6901 | -4.8514 | 0.0000 | 0.0000 | 4.3780 |
| AAK1 | -0.2345 | 4.6267 | -4.8511 | 0.0000 | 0.0000 | 4.3765 |
| MMACHC | 0.1746 | 5.9573 | 4.8506 | 0.0000 | 0.0000 | 4.3745 |
| RNF139 | 0.1048 | 6.5849 | 4.8492 | 0.0000 | 0.0000 | 4.3683 |
| RECQL4 | -0.3204 | 5.7998 | -4.8490 | 0.0000 | 0.0000 | 4.3671 |
| CLDN5 | 0.3661 | 5.8591 | 4.8489 | 0.0000 | 0.0000 | 4.3665 |
| UPK1B | -0.5385 | 0.6856 | -4.8484 | 0.0000 | 0.0000 | 4.3644 |
| KAZALD1 | -0.5393 | 3.9455 | -4.8479 | 0.0000 | 0.0000 | 4.3622 |
| BMP7 | -0.7582 | 0.9449 | -4.8475 | 0.0000 | 0.0000 | 4.3605 |
| ANKRD36B | -0.3771 | 1.3458 | -4.8473 | 0.0000 | 0.0000 | 4.3595 |
| VPS28 | 0.1291 | 7.1380 | 4.8465 | 0.0000 | 0.0000 | 4.3557 |
| GLP2R | -0.5740 | 1.0388 | -4.8463 | 0.0000 | 0.0000 | 4.3549 |
| APOOL | 0.1905 | 5.9893 | 4.8444 | 0.0000 | 0.0000 | 4.3463 |

| CCNH | 0.1339 | 5.8994 | 4.8434 | 0.0000 | 0.0000 | 4.3419 |
| --- | --- | --- | --- | --- | --- | --- |
| TMX2 | 0.0753 | 7.0697 | 4.8425 | 0.0000 | 0.0000 | 4.3379 |
| DNM1L | -0.1209 | 6.0237 | -4.8414 | 0.0000 | 0.0000 | 4.3327 |
| PTP4A3 | -0.3378 | 6.1000 | -4.8394 | 0.0000 | 0.0000 | 4.3238 |
| MSRB2 | 0.1313 | 6.9058 | 4.8374 | 0.0000 | 0.0000 | 4.3146 |
| STAT6 | 0.1481 | 6.7325 | 4.8369 | 0.0000 | 0.0000 | 4.3124 |
| SOAT1 | -0.1541 | 5.9914 | -4.8365 | 0.0000 | 0.0000 | 4.3108 |
| TMEM185B | -0.1733 | 6.0736 | -4.8362 | 0.0000 | 0.0000 | 4.3091 |
| FPR2 | -0.5084 | 1.1539 | -4.8360 | 0.0000 | 0.0000 | 4.3084 |
| NBEA | -0.6044 | 2.0499 | -4.8343 | 0.0000 | 0.0000 | 4.3008 |
| MYBL1 | -0.3948 | 3.8512 | -4.8339 | 0.0000 | 0.0000 | 4.2988 |
| NRXN1 | -0.1856 | 0.1453 | -4.8338 | 0.0000 | 0.0000 | 4.2984 |
| RGS1 | -0.6000 | 5.2770 | -4.8338 | 0.0000 | 0.0000 | 4.2982 |
| MSC | -0.6867 | 4.9020 | -4.8333 | 0.0000 | 0.0000 | 4.2962 |
| C12orf4 | -0.1538 | 5.2059 | -4.8333 | 0.0000 | 0.0000 | 4.2959 |
| HAP1 | -0.5003 | 1.0907 | -4.8320 | 0.0000 | 0.0000 | 4.2901 |
| ISYNA1 | -0.3561 | 5.7914 | -4.8310 | 0.0000 | 0.0000 | 4.2859 |
| PRDM16 | -0.7463 | 2.0476 | -4.8308 | 0.0000 | 0.0000 | 4.2848 |
| FAM114A1 | 0.1113 | 6.6199 | 4.8302 | 0.0000 | 0.0000 | 4.2820 |
| VAMP2 | 0.1336 | 6.4676 | 4.8299 | 0.0000 | 0.0000 | 4.2807 |
| SLC39A6 | -0.1694 | 6.2420 | -4.8294 | 0.0000 | 0.0000 | 4.2783 |
| POU2F2 | -0.4665 | 2.8673 | -4.8292 | 0.0000 | 0.0000 | 4.2777 |
| ARMC1 | 0.1146 | 6.5054 | 4.8257 | 0.0000 | 0.0000 | 4.2618 |
| CSF1 | -0.2483 | 5.9807 | -4.8257 | 0.0000 | 0.0000 | 4.2617 |
| KLC1 | -0.1898 | 4.4496 | -4.8223 | 0.0000 | 0.0000 | 4.2464 |
| TBC1D9B | 0.1132 | 6.5238 | 4.8214 | 0.0000 | 0.0000 | 4.2422 |
| A1CF | 0.3795 | 6.6908 | 4.8203 | 0.0000 | 0.0000 | 4.2375 |
| SH2D2A | -0.5626 | 4.2871 | -4.8195 | 0.0000 | 0.0000 | 4.2337 |
| PRSS1 | -0.6034 | 0.5455 | -4.8182 | 0.0000 | 0.0000 | 4.2280 |
| RPAP3 | -0.1032 | 5.7518 | -4.8173 | 0.0000 | 0.0000 | 4.2237 |
| KCNQ4 | -0.5551 | 2.2508 | -4.8166 | 0.0000 | 0.0000 | 4.2206 |
| SNAP91 | -0.4004 | 0.3032 | -4.8149 | 0.0000 | 0.0000 | 4.2132 |
| EHMT2 | -0.1435 | 6.2804 | -4.8142 | 0.0000 | 0.0000 | 4.2101 |
| GCHFR | 0.2499 | 6.3939 | 4.8137 | 0.0000 | 0.0000 | 4.2076 |
| AHNAK | 0.1368 | 6.7823 | 4.8131 | 0.0000 | 0.0000 | 4.2051 |
| FNBP1L | -0.1971 | 5.9726 | -4.8128 | 0.0000 | 0.0000 | 4.2037 |
| SMC2 | -0.2710 | 5.2401 | -4.8126 | 0.0000 | 0.0000 | 4.2028 |
| RPL10 | 0.0858 | 7.6433 | 4.8125 | 0.0000 | 0.0000 | 4.2025 |
| CD80 | -0.5376 | 1.9216 | -4.8116 | 0.0000 | 0.0000 | 4.1984 |
| JAK3 | -0.5104 | 4.3313 | -4.8110 | 0.0000 | 0.0000 | 4.1957 |
| APRT | 0.1157 | 7.2231 | 4.8102 | 0.0000 | 0.0000 | 4.1921 |
| IKZF5 | 0.1806 | 5.6644 | 4.8094 | 0.0000 | 0.0000 | 4.1886 |
| RPIA | -0.1124 | 6.1213 | -4.8072 | 0.0000 | 0.0000 | 4.1782 |
| RABL2A | -0.2512 | 3.9196 | -4.8063 | 0.0000 | 0.0000 | 4.1744 |
| ARHGAP4 | -0.3069 | 5.8354 | -4.8047 | 0.0000 | 0.0000 | 4.1672 |
| MTDH | 0.1121 | 6.8991 | 4.8042 | 0.0000 | 0.0000 | 4.1649 |
| GABRA3 | -0.8909 | 1.1915 | -4.8039 | 0.0000 | 0.0000 | 4.1636 |
| CCDC40 | -0.3709 | 3.2287 | -4.8036 | 0.0000 | 0.0000 | 4.1623 |
| UBL3 | 0.1529 | 6.4362 | 4.8032 | 0.0000 | 0.0000 | 4.1603 |
| SCARB1 | 0.1406 | 7.1564 | 4.8025 | 0.0000 | 0.0000 | 4.1574 |
| CABIN1 | -0.1422 | 5.8619 | -4.8004 | 0.0000 | 0.0000 | 4.1481 |
| HIST1H1B | -0.6194 | 1.7058 | -4.8004 | 0.0000 | 0.0000 | 4.1481 |
| 43898.0000 | 0.1586 | 6.1024 | 4.7997 | 0.0000 | 0.0000 | 4.1448 |

| SERPINE2 | -0.6854 | 4.7381 | -4.7992 | 0.0000 | 0.0000 | 4.1425 |
| --- | --- | --- | --- | --- | --- | --- |
| CYP2W1 | -0.6231 | 1.7665 | -4.7985 | 0.0000 | 0.0000 | 4.1393 |
| TTF1 | -0.1527 | 5.3534 | -4.7982 | 0.0000 | 0.0000 | 4.1380 |
| UNC119B | -0.2596 | 5.5166 | -4.7972 | 0.0000 | 0.0000 | 4.1333 |
| LUZP1 | -0.2316 | 4.8596 | -4.7948 | 0.0000 | 0.0000 | 4.1226 |
| MLX | 0.1017 | 6.6266 | 4.7940 | 0.0000 | 0.0000 | 4.1193 |
| CD83 | -0.2955 | 5.3295 | -4.7932 | 0.0000 | 0.0000 | 4.1154 |
| CD70 | -0.6340 | 2.1740 | -4.7922 | 0.0000 | 0.0000 | 4.1113 |
| SLC16A1 | 0.2387 | 6.5905 | 4.7901 | 0.0000 | 0.0000 | 4.1019 |
| MAPKAPK5 | -0.1244 | 5.1988 | -4.7895 | 0.0000 | 0.0000 | 4.0991 |
| CDK4 | -0.1392 | 6.5912 | -4.7882 | 0.0000 | 0.0000 | 4.0932 |
| ST18 | -0.3340 | 0.3697 | -4.7869 | 0.0000 | 0.0000 | 4.0872 |
| BMX | 0.5403 | 2.5067 | 4.7867 | 0.0000 | 0.0000 | 4.0864 |
| NDUFA6 | 0.0961 | 7.2600 | 4.7862 | 0.0000 | 0.0000 | 4.0843 |
| PDCL | -0.1361 | 5.6867 | -4.7816 | 0.0000 | 0.0000 | 4.0637 |
| ARHGAP28 | -0.5568 | 1.8122 | -4.7802 | 0.0000 | 0.0000 | 4.0572 |
| GCH1 | 0.2687 | 6.4070 | 4.7783 | 0.0000 | 0.0000 | 4.0489 |
| P2RX5-TAX1BP3 | -0.2569 | 1.5040 | -4.7771 | 0.0000 | 0.0000 | 4.0434 |
| UGT2B15 | 0.5730 | 7.0450 | 4.7760 | 0.0000 | 0.0000 | 4.0385 |
| DPYSL4 | -0.7538 | 2.1120 | -4.7754 | 0.0000 | 0.0000 | 4.0358 |
| MYBPC3 | -0.3690 | 0.8513 | -4.7753 | 0.0000 | 0.0000 | 4.0356 |
| CARD10 | -0.1813 | 6.1973 | -4.7752 | 0.0000 | 0.0000 | 4.0348 |
| PMS1 | -0.1652 | 5.0780 | -4.7749 | 0.0000 | 0.0000 | 4.0335 |
| MEAF6 | 0.1173 | 6.6034 | 4.7728 | 0.0000 | 0.0000 | 4.0242 |
| MAP9 | -0.5817 | 1.6563 | -4.7716 | 0.0000 | 0.0000 | 4.0191 |
| ECHDC3 | 0.4559 | 6.8139 | 4.7698 | 0.0000 | 0.0000 | 4.0111 |
| NPFFR1 | -0.2168 | 0.3005 | -4.7686 | 0.0000 | 0.0000 | 4.0054 |
| NPHP4 | -0.6164 | 3.0357 | -4.7681 | 0.0000 | 0.0000 | 4.0033 |
| CHD3 | -0.3676 | 5.4002 | -4.7672 | 0.0000 | 0.0000 | 3.9990 |
| SAGE1 | -0.5618 | 0.4139 | -4.7661 | 0.0000 | 0.0000 | 3.9945 |
| GLO1 | 0.0938 | 7.2093 | 4.7658 | 0.0000 | 0.0000 | 3.9929 |
| H2AFZ | -0.0961 | 7.1254 | -4.7640 | 0.0000 | 0.0000 | 3.9849 |
| TNS4 | -0.4551 | 1.0006 | -4.7619 | 0.0000 | 0.0000 | 3.9757 |
| KEL | -0.7336 | 2.0099 | -4.7601 | 0.0000 | 0.0000 | 3.9676 |
| APOB | 0.2990 | 7.6667 | 4.7598 | 0.0000 | 0.0000 | 3.9663 |
| CCDC69 | 0.2707 | 6.0374 | 4.7598 | 0.0000 | 0.0000 | 3.9661 |
| SCG3 | -0.5511 | 0.8845 | -4.7598 | 0.0000 | 0.0000 | 3.9660 |
| CTNNB1 | 0.0891 | 7.0404 | 4.7595 | 0.0000 | 0.0000 | 3.9650 |
| SLC8A2 | -0.3500 | 0.6150 | -4.7580 | 0.0000 | 0.0000 | 3.9582 |
| SLC38A10 | 0.0945 | 7.0465 | 4.7580 | 0.0000 | 0.0000 | 3.9582 |
| OTOF | -0.4088 | 0.9286 | -4.7559 | 0.0000 | 0.0000 | 3.9487 |
| COQ10B | 0.1015 | 6.5264 | 4.7551 | 0.0000 | 0.0000 | 3.9454 |
| EFNA3 | -0.4563 | 4.7505 | -4.7547 | 0.0000 | 0.0000 | 3.9437 |
| NR2F6 | 0.1150 | 7.0852 | 4.7544 | 0.0000 | 0.0000 | 3.9423 |
| PYGB | -0.1415 | 6.7000 | -4.7542 | 0.0000 | 0.0000 | 3.9412 |
| MMP19 | -0.4716 | 4.4259 | -4.7536 | 0.0000 | 0.0000 | 3.9387 |
| SRP54 | 0.1035 | 6.6150 | 4.7518 | 0.0000 | 0.0000 | 3.9306 |
| TPRA1 | 0.0864 | 6.5672 | 4.7513 | 0.0000 | 0.0000 | 3.9284 |
| PNKP | -0.1203 | 6.2253 | -4.7500 | 0.0000 | 0.0000 | 3.9228 |
| G0S2 | 0.3109 | 7.0556 | 4.7490 | 0.0000 | 0.0000 | 3.9183 |
| RER1 | 0.1003 | 6.8163 | 4.7480 | 0.0000 | 0.0000 | 3.9137 |
| OXR1 | 0.2241 | 5.8273 | 4.7471 | 0.0000 | 0.0000 | 3.9095 |
| HCFC1 | -0.1013 | 6.2472 | -4.7457 | 0.0000 | 0.0000 | 3.9036 |

| DOT1L | -0.2204 | 5.4758 | -4.7448 | 0.0000 | 0.0000 | 3.8996 |
| --- | --- | --- | --- | --- | --- | --- |
| SRPK3 | -0.4775 | 1.8924 | -4.7430 | 0.0000 | 0.0000 | 3.8913 |
| SULF1 | -0.7302 | 4.2428 | -4.7419 | 0.0000 | 0.0000 | 3.8866 |
| FOXD3 | -0.3161 | 0.2593 | -4.7416 | 0.0000 | 0.0000 | 3.8854 |
| TOMM20 | 0.0832 | 7.2726 | 4.7414 | 0.0000 | 0.0000 | 3.8843 |
| H2AFY2 | -0.5236 | 5.7773 | -4.7410 | 0.0000 | 0.0000 | 3.8827 |
| PIGP | 0.1857 | 5.3552 | 4.7406 | 0.0000 | 0.0000 | 3.8809 |
| SPICE1 | -0.2834 | 4.3144 | -4.7404 | 0.0000 | 0.0000 | 3.8798 |
| NAMPT | 0.2433 | 6.5576 | 4.7403 | 0.0000 | 0.0000 | 3.8797 |
| RPA4 | -0.3760 | 1.0548 | -4.7401 | 0.0000 | 0.0000 | 3.8785 |
| PITPNB | 0.1013 | 6.5598 | 4.7384 | 0.0000 | 0.0000 | 3.8713 |
| FGL1 | 0.3076 | 7.5941 | 4.7382 | 0.0000 | 0.0000 | 3.8702 |
| PPIB | 0.0797 | 7.6184 | 4.7382 | 0.0000 | 0.0000 | 3.8701 |
| TNFRSF10C | -0.5170 | 3.9559 | -4.7380 | 0.0000 | 0.0000 | 3.8692 |
| GGTLC1 | -0.5386 | 1.1278 | -4.7362 | 0.0000 | 0.0000 | 3.8611 |
| EVPL | -0.9193 | 2.0913 | -4.7350 | 0.0000 | 0.0000 | 3.8558 |
| GLS2 | 0.9974 | 3.4774 | 4.7349 | 0.0000 | 0.0000 | 3.8553 |
| TMC7 | -0.4972 | 3.3932 | -4.7346 | 0.0000 | 0.0000 | 3.8542 |
| KRT32 | -0.1235 | 0.0784 | -4.7339 | 0.0000 | 0.0000 | 3.8509 |
| BCAP31 | 0.0946 | 7.5005 | 4.7333 | 0.0000 | 0.0000 | 3.8483 |
| ABCG1 | -0.3818 | 5.2572 | -4.7317 | 0.0000 | 0.0000 | 3.8415 |
| PGS1 | -0.1378 | 5.6097 | -4.7304 | 0.0000 | 0.0000 | 3.8355 |
| GMPS | -0.1286 | 6.0409 | -4.7303 | 0.0000 | 0.0000 | 3.8349 |
| POGK | -0.1564 | 6.0752 | -4.7296 | 0.0000 | 0.0000 | 3.8318 |
| CUEDC2 | 0.0958 | 6.9943 | 4.7284 | 0.0000 | 0.0000 | 3.8269 |
| HOPX | -0.5279 | 3.0681 | -4.7278 | 0.0000 | 0.0000 | 3.8241 |
| C5 | 0.2359 | 7.2558 | 4.7265 | 0.0000 | 0.0000 | 3.8180 |
| BCL7B | 0.0911 | 6.5811 | 4.7258 | 0.0000 | 0.0000 | 3.8151 |
| CAMKV | -0.4493 | 0.4648 | -4.7255 | 0.0000 | 0.0000 | 3.8139 |
| LIMD2 | -0.3999 | 5.3452 | -4.7240 | 0.0000 | 0.0000 | 3.8070 |
| HIGD2A | 0.0964 | 7.4579 | 4.7239 | 0.0000 | 0.0000 | 3.8067 |
| TNFSF10 | 0.1905 | 6.8726 | 4.7233 | 0.0000 | 0.0000 | 3.8041 |
| MPPED1 | 0.8842 | 4.0709 | 4.7222 | 0.0000 | 0.0000 | 3.7990 |
| RB1CC1 | 0.1831 | 6.2609 | 4.7205 | 0.0000 | 0.0000 | 3.7918 |
| KCNJ13 | -0.2925 | 0.2884 | -4.7191 | 0.0000 | 0.0000 | 3.7856 |
| OGDHL | 0.6166 | 6.1654 | 4.7177 | 0.0000 | 0.0000 | 3.7792 |
| USP46 | -0.2504 | 4.4428 | -4.7175 | 0.0000 | 0.0000 | 3.7785 |
| PNMA2 | -0.6361 | 2.0501 | -4.7167 | 0.0000 | 0.0000 | 3.7749 |
| RPL19 | 0.0782 | 7.8125 | 4.7163 | 0.0000 | 0.0000 | 3.7732 |
| CP | 0.3476 | 7.2241 | 4.7161 | 0.0000 | 0.0000 | 3.7723 |
| B4GALT5 | -0.1434 | 6.3967 | -4.7156 | 0.0000 | 0.0000 | 3.7699 |
| CHRNA10 | -0.3264 | 2.4720 | -4.7150 | 0.0000 | 0.0000 | 3.7675 |
| SLC4A7 | -0.4392 | 4.0132 | -4.7150 | 0.0000 | 0.0000 | 3.7675 |
| PDIA3 | 0.0718 | 7.5485 | 4.7149 | 0.0000 | 0.0000 | 3.7671 |
| CAMKMT | -0.2161 | 4.6132 | -4.7138 | 0.0000 | 0.0000 | 3.7620 |
| PFN2 | -0.4934 | 5.1933 | -4.7134 | 0.0000 | 0.0000 | 3.7605 |
| NDUFA8 | 0.0968 | 7.2237 | 4.7124 | 0.0000 | 0.0000 | 3.7561 |
| BRIX1 | -0.1349 | 5.9093 | -4.7123 | 0.0000 | 0.0000 | 3.7555 |
| BCAS1 | -0.8243 | 2.3984 | -4.7114 | 0.0000 | 0.0000 | 3.7515 |
| HLA-A | 0.0998 | 7.8211 | 4.7104 | 0.0000 | 0.0000 | 3.7469 |
| OLA1 | -0.1103 | 6.4049 | -4.7095 | 0.0000 | 0.0000 | 3.7430 |
| IQCB1 | -0.1788 | 5.6116 | -4.7086 | 0.0000 | 0.0000 | 3.7390 |
| TSGA10 | -0.2945 | 2.9740 | -4.7082 | 0.0000 | 0.0000 | 3.7374 |

| KCNJ10 | -0.6562 | 1.9349 | -4.7059 | 0.0000 | 0.0000 | 3.7271 |
| --- | --- | --- | --- | --- | --- | --- |
| NCF1 | -0.5260 | 3.1739 | -4.7056 | 0.0000 | 0.0000 | 3.7258 |
| PLXDC1 | -0.4402 | 4.0369 | -4.7025 | 0.0000 | 0.0000 | 3.7121 |
| ADORA2A | 0.4473 | 3.6408 | 4.7012 | 0.0000 | 0.0000 | 3.7064 |
| FLRT1 | -0.4650 | 1.8152 | -4.7007 | 0.0000 | 0.0000 | 3.7041 |
| P2RX5 | -0.6837 | 2.2114 | -4.6999 | 0.0000 | 0.0000 | 3.7008 |
| RAP2B | -0.1699 | 5.9097 | -4.6996 | 0.0000 | 0.0000 | 3.6995 |
| ABCF3 | 0.0705 | 6.5299 | 4.6988 | 0.0000 | 0.0000 | 3.6961 |
| PPEF1 | -0.4623 | 1.1725 | -4.6985 | 0.0000 | 0.0000 | 3.6946 |
| DAP3 | 0.0872 | 6.9620 | 4.6985 | 0.0000 | 0.0000 | 3.6944 |
| PCGF2 | -0.2059 | 5.9034 | -4.6985 | 0.0000 | 0.0000 | 3.6944 |
| TACSTD2 | -0.8093 | 3.6551 | -4.6951 | 0.0000 | 0.0000 | 3.6797 |
| ZBED2 | -0.7064 | 1.6530 | -4.6949 | 0.0000 | 0.0000 | 3.6785 |
| TGFBRAP1 | -0.1572 | 5.4282 | -4.6941 | 0.0000 | 0.0000 | 3.6750 |
| FANCL | -0.1277 | 5.9002 | -4.6931 | 0.0000 | 0.0000 | 3.6709 |
| DHX29 | 0.1086 | 6.2501 | 4.6919 | 0.0000 | 0.0000 | 3.6657 |
| SEZ6L | -0.3499 | 0.5101 | -4.6919 | 0.0000 | 0.0000 | 3.6653 |
| MEF2B | -0.4086 | 2.4960 | -4.6892 | 0.0000 | 0.0000 | 3.6537 |
| MRS2 | 0.1272 | 6.3118 | 4.6891 | 0.0000 | 0.0000 | 3.6533 |
| IFI27 | 0.3812 | 6.7159 | 4.6884 | 0.0000 | 0.0000 | 3.6499 |
| OSMR | -0.4748 | 5.4024 | -4.6873 | 0.0000 | 0.0000 | 3.6452 |
| ULBP2 | -0.6360 | 3.2006 | -4.6871 | 0.0000 | 0.0000 | 3.6445 |
| YIPF1 | 0.0881 | 6.8111 | 4.6866 | 0.0000 | 0.0000 | 3.6420 |
| FAS | 0.3715 | 5.5176 | 4.6852 | 0.0000 | 0.0000 | 3.6361 |
| RIPK1 | 0.0862 | 6.4572 | 4.6841 | 0.0000 | 0.0000 | 3.6313 |
| LIPG | 0.5240 | 5.2270 | 4.6840 | 0.0000 | 0.0000 | 3.6308 |
| TAOK3 | 0.1699 | 5.6584 | 4.6809 | 0.0000 | 0.0000 | 3.6172 |
| CBFA2T2 | -0.2106 | 5.2882 | -4.6797 | 0.0000 | 0.0000 | 3.6119 |
| COX7B | 0.1118 | 7.1598 | 4.6793 | 0.0000 | 0.0000 | 3.6101 |
| PPME1 | -0.1081 | 5.7964 | -4.6792 | 0.0000 | 0.0000 | 3.6097 |
| **PI3** | -1.0614 | 3.8642 | -4.6769 | 0.0000 | 0.0000 | 3.5996 |
| HOXB8 | -0.5183 | 0.8724 | -4.6765 | 0.0000 | 0.0000 | 3.5980 |
| IL27RA | -0.3031 | 5.2609 | -4.6759 | 0.0000 | 0.0000 | 3.5951 |
| SLC25A11 | 0.1101 | 6.9146 | 4.6753 | 0.0000 | 0.0000 | 3.5923 |
| EPYC | -0.2459 | 0.2553 | -4.6752 | 0.0000 | 0.0000 | 3.5920 |
| PF4V1 | -0.9849 | 2.0632 | -4.6749 | 0.0000 | 0.0000 | 3.5907 |
| VEGFB | -0.2906 | 6.7345 | -4.6732 | 0.0000 | 0.0000 | 3.5834 |
| GCOM1 | -0.1923 | 0.3453 | -4.6703 | 0.0000 | 0.0000 | 3.5705 |
| HOXD1 | -0.6507 | 1.1580 | -4.6694 | 0.0000 | 0.0000 | 3.5667 |
| AHDC1 | -0.2185 | 5.3053 | -4.6692 | 0.0000 | 0.0000 | 3.5658 |
| ISL1 | -0.2965 | 0.2090 | -4.6681 | 0.0000 | 0.0000 | 3.5609 |
| VCAN | -0.7768 | 4.2271 | -4.6672 | 0.0000 | 0.0000 | 3.5571 |
| DMP1 | -0.2460 | 0.3028 | -4.6663 | 0.0000 | 0.0000 | 3.5532 |
| AGTR1 | 0.3867 | 6.2301 | 4.6634 | 0.0000 | 0.0000 | 3.5403 |
| MKL1 | -0.1163 | 5.8897 | -4.6623 | 0.0000 | 0.0000 | 3.5354 |
| CDR2L | -0.3976 | 4.6919 | -4.6609 | 0.0000 | 0.0000 | 3.5296 |
| SOX17 | 0.3572 | 4.5146 | 4.6606 | 0.0000 | 0.0000 | 3.5283 |
| PRPF38B | -0.1279 | 6.0027 | -4.6578 | 0.0000 | 0.0000 | 3.5158 |
| FCGR2B | -0.7057 | 3.1798 | -4.6577 | 0.0000 | 0.0000 | 3.5156 |
| ZNF675 | -0.4467 | 3.4392 | -4.6567 | 0.0000 | 0.0000 | 3.5109 |
| FCGR2A | -0.3623 | 5.1447 | -4.6566 | 0.0000 | 0.0000 | 3.5104 |
| THBS4 | 0.7049 | 4.8663 | 4.6563 | 0.0000 | 0.0000 | 3.5095 |
| HAUS5 | -0.1588 | 5.6002 | -4.6563 | 0.0000 | 0.0000 | 3.5092 |

| SOCS7 | -0.2969 | 4.6582 | -4.6553 | 0.0000 | 0.0000 | 3.5051 |
| --- | --- | --- | --- | --- | --- | --- |
| ITGAM | -0.4745 | 4.6679 | -4.6521 | 0.0000 | 0.0000 | 3.4911 |
| TRAFD1 | -0.1179 | 6.1335 | -4.6521 | 0.0000 | 0.0000 | 3.4909 |
| APOH | 0.2835 | 8.1371 | 4.6508 | 0.0000 | 0.0000 | 3.4851 |
| AGL | 0.2376 | 5.7458 | 4.6502 | 0.0000 | 0.0000 | 3.4829 |
| ASB8 | 0.0963 | 6.2711 | 4.6501 | 0.0000 | 0.0000 | 3.4821 |
| UXT | 0.0920 | 7.1228 | 4.6498 | 0.0000 | 0.0000 | 3.4810 |
| AKR1C2 | 0.4585 | 6.8042 | 4.6492 | 0.0000 | 0.0000 | 3.4782 |
| TMCO3 | -0.2642 | 5.7169 | -4.6487 | 0.0000 | 0.0000 | 3.4763 |
| MAP1A | -0.5456 | 2.8243 | -4.6478 | 0.0000 | 0.0000 | 3.4721 |
| CHMP6 | 0.1065 | 6.6605 | 4.6473 | 0.0000 | 0.0000 | 3.4701 |
| GNRH1 | -0.3766 | 3.1392 | -4.6471 | 0.0000 | 0.0000 | 3.4690 |
| ZNF234 | -0.2583 | 4.5010 | -4.6470 | 0.0000 | 0.0000 | 3.4689 |
| GAN | -0.2484 | 3.2365 | -4.6448 | 0.0000 | 0.0000 | 3.4591 |
| HSPA5 | 0.0779 | 7.6897 | 4.6444 | 0.0000 | 0.0000 | 3.4575 |
| VEGFA | -0.1481 | 6.4295 | -4.6433 | 0.0000 | 0.0000 | 3.4525 |
| IFT140 | -0.2760 | 4.5247 | -4.6433 | 0.0000 | 0.0000 | 3.4525 |
| RGPD3 | 0.3072 | 0.9719 | 4.6431 | 0.0000 | 0.0000 | 3.4518 |
| NFIL3 | 0.1534 | 6.7777 | 4.6413 | 0.0000 | 0.0000 | 3.4440 |
| SPC24 | -0.3839 | 5.6073 | -4.6412 | 0.0000 | 0.0000 | 3.4434 |
| RRAGC | -0.1376 | 5.7296 | -4.6411 | 0.0000 | 0.0000 | 3.4431 |
| CTNNA3 | 0.5985 | 1.5146 | 4.6411 | 0.0000 | 0.0000 | 3.4430 |
| ALX1 | -0.6143 | 0.5208 | -4.6395 | 0.0000 | 0.0000 | 3.4360 |
| LMO3 | -0.3715 | 1.1175 | -4.6386 | 0.0000 | 0.0000 | 3.4323 |
| TERF2IP | 0.0950 | 6.5904 | 4.6382 | 0.0000 | 0.0000 | 3.4303 |
| TRPV4 | -0.7115 | 4.2378 | -4.6370 | 0.0000 | 0.0000 | 3.4252 |
| PPP2R5C | 0.1152 | 6.0616 | 4.6347 | 0.0000 | 0.0000 | 3.4149 |
| RHOBTB3 | 0.2619 | 6.0560 | 4.6336 | 0.0000 | 0.0000 | 3.4103 |
| CAND2 | -0.6105 | 2.5413 | -4.6314 | 0.0000 | 0.0000 | 3.4006 |
| CAPN9 | -0.6539 | 2.0580 | -4.6307 | 0.0000 | 0.0000 | 3.3979 |
| MED28 | -0.1454 | 5.5438 | -4.6304 | 0.0000 | 0.0000 | 3.3966 |
| RBM34 | -0.2387 | 3.8479 | -4.6303 | 0.0000 | 0.0000 | 3.3960 |
| HOXC10 | -0.8471 | 0.9983 | -4.6295 | 0.0000 | 0.0000 | 3.3926 |
| EN2 | -0.6596 | 0.9494 | -4.6269 | 0.0000 | 0.0000 | 3.3813 |
| VSTM4 | 0.5062 | 4.8798 | 4.6251 | 0.0000 | 0.0000 | 3.3735 |
| APOL3 | 0.2830 | 6.1849 | 4.6244 | 0.0000 | 0.0000 | 3.3701 |
| UBE2Z | -0.0791 | 6.6099 | -4.6241 | 0.0000 | 0.0000 | 3.3690 |
| IL11RA | 0.2097 | 5.8889 | 4.6219 | 0.0000 | 0.0000 | 3.3593 |
| STC2 | -0.4554 | 4.6242 | -4.6183 | 0.0000 | 0.0000 | 3.3437 |
| ZNF141 | -0.5190 | 3.6421 | -4.6180 | 0.0000 | 0.0000 | 3.3423 |
| GRIK4 | -0.6496 | 1.4708 | -4.6167 | 0.0000 | 0.0000 | 3.3366 |
| PEX3 | 0.1673 | 6.0841 | 4.6164 | 0.0000 | 0.0000 | 3.3356 |
| EZR | -0.1567 | 6.8401 | -4.6153 | 0.0000 | 0.0000 | 3.3310 |
| MPI | 0.1548 | 5.9410 | 4.6143 | 0.0000 | 0.0000 | 3.3265 |
| LARP7 | 0.1086 | 6.2110 | 4.6140 | 0.0000 | 0.0000 | 3.3252 |
| CBX6 | -0.4306 | 5.2602 | -4.6138 | 0.0000 | 0.0000 | 3.3241 |
| BRD9 | -0.1474 | 5.4897 | -4.6126 | 0.0000 | 0.0000 | 3.3191 |
| S100B | -0.6433 | 3.2054 | -4.6124 | 0.0000 | 0.0000 | 3.3181 |
| LRRC8D | 0.1267 | 6.3423 | 4.6118 | 0.0000 | 0.0000 | 3.3157 |
| ATP10B | -0.5865 | 0.8257 | -4.6105 | 0.0000 | 0.0000 | 3.3100 |
| WNT2B | -0.3820 | 1.9318 | -4.6089 | 0.0000 | 0.0000 | 3.3031 |
| NCF2 | -0.3835 | 5.4209 | -4.6080 | 0.0000 | 0.0000 | 3.2989 |
| ERAP1 | 0.1822 | 6.2758 | 4.6071 | 0.0000 | 0.0000 | 3.2954 |

| CLNS1A | 0.0989 | 6.6925 | 4.6071 | 0.0000 | 0.0000 | 3.2951 |
| --- | --- | --- | --- | --- | --- | --- |
| CCNE2 | -0.4852 | 3.5673 | -4.6070 | 0.0000 | 0.0000 | 3.2950 |
| KCNJ15 | -0.5504 | 2.1014 | -4.6064 | 0.0000 | 0.0000 | 3.2924 |
| EMD | 0.0877 | 6.9536 | 4.6063 | 0.0000 | 0.0000 | 3.2916 |
| VIPR1 | 0.6403 | 2.9340 | 4.6054 | 0.0000 | 0.0000 | 3.2877 |
| DFFB | -0.2513 | 4.2581 | -4.6031 | 0.0000 | 0.0000 | 3.2778 |
| CDH18 | -0.4584 | 0.3933 | -4.6023 | 0.0000 | 0.0000 | 3.2746 |
| JRKL | -0.2655 | 5.0975 | -4.6022 | 0.0000 | 0.0000 | 3.2741 |
| L1TD1 | -0.3330 | 0.4884 | -4.6015 | 0.0000 | 0.0000 | 3.2708 |
| ADAMTSL4 | 0.3457 | 5.8336 | 4.6003 | 0.0000 | 0.0000 | 3.2658 |
| FAM184A | 0.5536 | 4.1468 | 4.5997 | 0.0000 | 0.0000 | 3.2631 |
| RIMS3 | -0.4722 | 2.8966 | -4.5993 | 0.0000 | 0.0000 | 3.2615 |
| PPIH | -0.1224 | 6.3844 | -4.5986 | 0.0000 | 0.0000 | 3.2586 |
| NSDHL | 0.1282 | 6.8672 | 4.5982 | 0.0000 | 0.0000 | 3.2567 |
| FBLN5 | 0.4956 | 5.5040 | 4.5979 | 0.0000 | 0.0000 | 3.2556 |
| TREM2 | -0.4839 | 5.5065 | -4.5957 | 0.0000 | 0.0000 | 3.2461 |
| KLHL18 | -0.1532 | 4.8099 | -4.5954 | 0.0000 | 0.0000 | 3.2445 |
| GPR37L1 | -0.4598 | 1.7399 | -4.5950 | 0.0000 | 0.0000 | 3.2430 |
| ISCA1 | 0.1026 | 6.4801 | 4.5933 | 0.0000 | 0.0000 | 3.2355 |
| KRT4 | -0.4672 | 0.4104 | -4.5928 | 0.0000 | 0.0000 | 3.2334 |
| APLP2 | 0.0773 | 7.3519 | 4.5923 | 0.0000 | 0.0000 | 3.2313 |
| MTM1 | 0.2463 | 5.7118 | 4.5912 | 0.0000 | 0.0000 | 3.2266 |
| FSCN3 | -0.2081 | 0.5340 | -4.5912 | 0.0000 | 0.0000 | 3.2265 |
| ACVR1 | -0.1612 | 6.1027 | -4.5912 | 0.0000 | 0.0000 | 3.2263 |
| SRSF10 | -0.0851 | 6.0311 | -4.5902 | 0.0000 | 0.0000 | 3.2221 |
| CSNK1G1 | -0.1911 | 4.7436 | -4.5902 | 0.0000 | 0.0000 | 3.2220 |
| RPL10A | 0.0813 | 7.7152 | 4.5900 | 0.0000 | 0.0000 | 3.2212 |
| ATP2B1 | -0.2034 | 5.6777 | -4.5895 | 0.0000 | 0.0000 | 3.2191 |
| GJA5 | 0.4359 | 5.1304 | 4.5893 | 0.0000 | 0.0000 | 3.2185 |
| SULT1C2 | -0.9438 | 4.0782 | -4.5892 | 0.0000 | 0.0000 | 3.2177 |
| TLR7 | -0.5382 | 2.5746 | -4.5879 | 0.0000 | 0.0000 | 3.2124 |
| PAK6 | -0.1408 | 0.1725 | -4.5879 | 0.0000 | 0.0000 | 3.2124 |
| ZNF365 | -0.2963 | 0.4927 | -4.5872 | 0.0000 | 0.0000 | 3.2092 |
| TAF1B | -0.2030 | 5.0397 | -4.5870 | 0.0000 | 0.0000 | 3.2084 |
| KDSR | 0.1119 | 6.2060 | 4.5867 | 0.0000 | 0.0000 | 3.2070 |
| VGLL1 | -0.4977 | 0.4912 | -4.5854 | 0.0000 | 0.0000 | 3.2014 |
| RERE | -0.1695 | 5.9059 | -4.5842 | 0.0000 | 0.0000 | 3.1964 |
| TNFRSF8 | -0.4614 | 1.9285 | -4.5841 | 0.0000 | 0.0000 | 3.1960 |
| MOCOS | 0.2347 | 6.0195 | 4.5839 | 0.0000 | 0.0000 | 3.1952 |
| NDUFB11 | 0.0995 | 7.3701 | 4.5829 | 0.0000 | 0.0000 | 3.1908 |
| COL10A1 | -0.8607 | 1.9273 | -4.5822 | 0.0000 | 0.0000 | 3.1875 |
| IL9R | -0.3329 | 0.7826 | -4.5815 | 0.0000 | 0.0000 | 3.1847 |
| MRPS14 | 0.0962 | 6.5377 | 4.5804 | 0.0000 | 0.0000 | 3.1798 |
| PRPH2 | -0.4929 | 1.6458 | -4.5791 | 0.0000 | 0.0000 | 3.1742 |
| **ZFY** | 1.0472 | 2.9738 | 4.5789 | 0.0000 | 0.0000 | 3.1736 |
| CD36 | 0.4361 | 5.7890 | 4.5786 | 0.0000 | 0.0000 | 3.1722 |
| COL11A2 | -0.5319 | 2.1564 | -4.5781 | 0.0000 | 0.0000 | 3.1702 |
| GREB1 | 0.6910 | 4.0070 | 4.5764 | 0.0000 | 0.0000 | 3.1629 |
| POLD4 | 0.1349 | 6.5912 | 4.5762 | 0.0000 | 0.0000 | 3.1619 |
| CNGA1 | 0.6571 | 4.9905 | 4.5755 | 0.0000 | 0.0000 | 3.1587 |
| PTEN | 0.1602 | 6.1703 | 4.5753 | 0.0000 | 0.0000 | 3.1581 |
| MAP3K1 | -0.1972 | 5.4210 | -4.5717 | 0.0000 | 0.0000 | 3.1426 |
| PPIF | 0.1075 | 7.1409 | 4.5713 | 0.0000 | 0.0000 | 3.1409 |

| ZNF160 | -0.4289 | 4.2982 | -4.5708 | 0.0000 | 0.0000 | 3.1388 |
| --- | --- | --- | --- | --- | --- | --- |
| FAM53B | -0.2148 | 5.4327 | -4.5706 | 0.0000 | 0.0000 | 3.1378 |
| RABEP2 | -0.1721 | 5.7446 | -4.5697 | 0.0000 | 0.0000 | 3.1339 |
| WIF1 | -0.7036 | 0.7878 | -4.5697 | 0.0000 | 0.0000 | 3.1337 |
| OGDH | 0.1054 | 6.9111 | 4.5690 | 0.0000 | 0.0000 | 3.1311 |
| NAT1 | 0.3015 | 4.9001 | 4.5680 | 0.0000 | 0.0000 | 3.1266 |
| GDAP1 | -0.4694 | 4.5923 | -4.5674 | 0.0000 | 0.0000 | 3.1239 |
| CDSN | -0.1264 | 0.0818 | -4.5673 | 0.0000 | 0.0000 | 3.1234 |
| NBL1 | -0.4642 | 4.6622 | -4.5652 | 0.0000 | 0.0000 | 3.1146 |
| GABRR1 | -0.2931 | 0.3365 | -4.5652 | 0.0000 | 0.0000 | 3.1145 |
| FAM135A | -0.3405 | 4.0486 | -4.5647 | 0.0000 | 0.0000 | 3.1122 |
| SLCO1A2 | 0.5922 | 3.2041 | 4.5639 | 0.0000 | 0.0000 | 3.1090 |
| NPR2 | 0.4007 | 5.7787 | 4.5636 | 0.0000 | 0.0000 | 3.1077 |
| ZMAT4 | -0.3536 | 0.2994 | -4.5620 | 0.0000 | 0.0000 | 3.1008 |
| CTNNBL1 | 0.1240 | 6.6383 | 4.5599 | 0.0000 | 0.0000 | 3.0916 |
| RPL3 | 0.0786 | 7.7841 | 4.5591 | 0.0000 | 0.0000 | 3.0885 |
| ETHE1 | 0.1393 | 6.6757 | 4.5586 | 0.0000 | 0.0000 | 3.0861 |
| DDAH1 | 0.1943 | 6.6393 | 4.5584 | 0.0000 | 0.0000 | 3.0853 |
| EYA3 | -0.1482 | 5.3654 | -4.5583 | 0.0000 | 0.0000 | 3.0848 |
| ECEL1 | -0.8961 | 1.8467 | -4.5575 | 0.0000 | 0.0000 | 3.0813 |
| CRHR1 | -0.3471 | 0.1912 | -4.5572 | 0.0000 | 0.0000 | 3.0802 |
| TBC1D17 | 0.0965 | 6.5542 | 4.5567 | 0.0000 | 0.0000 | 3.0779 |
| SFSWAP | -0.1206 | 5.6152 | -4.5566 | 0.0000 | 0.0000 | 3.0777 |
| KCND1 | -0.4178 | 2.6100 | -4.5558 | 0.0000 | 0.0000 | 3.0743 |
| TET3 | -0.2941 | 4.7001 | -4.5549 | 0.0000 | 0.0000 | 3.0704 |
| PDK2 | 0.2007 | 6.1468 | 4.5539 | 0.0000 | 0.0000 | 3.0660 |
| NDUFAF3 | 0.0972 | 6.9456 | 4.5520 | 0.0000 | 0.0000 | 3.0579 |
| ARL4C | -0.3261 | 5.7821 | -4.5515 | 0.0000 | 0.0000 | 3.0556 |
| SLC7A8 | -0.4304 | 4.2686 | -4.5512 | 0.0000 | 0.0000 | 3.0545 |
| LRFN4 | -0.4799 | 3.9272 | -4.5501 | 0.0000 | 0.0000 | 3.0498 |
| IFIT2 | 0.2868 | 5.9175 | 4.5501 | 0.0000 | 0.0000 | 3.0496 |
| GBX2 | -0.3456 | 0.5622 | -4.5482 | 0.0000 | 0.0000 | 3.0418 |
| SDK2 | -0.7106 | 1.6646 | -4.5481 | 0.0000 | 0.0000 | 3.0414 |
| SOX9 | -0.6359 | 5.7386 | -4.5473 | 0.0000 | 0.0000 | 3.0378 |
| TRMT112 | 0.0785 | 7.3241 | 4.5463 | 0.0000 | 0.0000 | 3.0336 |
| ZNF529 | -0.2469 | 4.5629 | -4.5420 | 0.0000 | 0.0000 | 3.0153 |
| TAPBPL | 0.1884 | 6.4295 | 4.5406 | 0.0000 | 0.0000 | 3.0091 |
| BSPRY | -0.7568 | 3.9691 | -4.5404 | 0.0000 | 0.0000 | 3.0083 |
| FLNC | -0.8388 | 3.5496 | -4.5402 | 0.0000 | 0.0000 | 3.0075 |
| TBX10 | 0.5964 | 3.6133 | 4.5399 | 0.0000 | 0.0000 | 3.0063 |
| KRT6B | -0.7160 | 1.0902 | -4.5379 | 0.0000 | 0.0000 | 2.9977 |
| TRAF3 | -0.2110 | 5.1997 | -4.5377 | 0.0000 | 0.0000 | 2.9967 |
| DVL3 | -0.0897 | 6.5017 | -4.5374 | 0.0000 | 0.0000 | 2.9954 |
| ZNF83 | -0.5871 | 4.5133 | -4.5372 | 0.0000 | 0.0000 | 2.9948 |
| SLC24A3 | -0.6986 | 2.3085 | -4.5364 | 0.0000 | 0.0000 | 2.9911 |
| AK5 | -0.3490 | 0.6722 | -4.5348 | 0.0000 | 0.0000 | 2.9845 |
| INPP5F | -0.2511 | 4.6037 | -4.5341 | 0.0000 | 0.0000 | 2.9813 |
| ANAPC2 | 0.0990 | 6.3719 | 4.5333 | 0.0000 | 0.0000 | 2.9781 |
| MCOLN1 | 0.1123 | 6.3448 | 4.5333 | 0.0000 | 0.0000 | 2.9780 |
| HOXB7 | -0.5440 | 3.8681 | -4.5318 | 0.0000 | 0.0000 | 2.9714 |
| FBXW12 | -0.3625 | 0.9709 | -4.5312 | 0.0000 | 0.0000 | 2.9689 |
| EMP1 | 0.3494 | 5.4381 | 4.5310 | 0.0000 | 0.0000 | 2.9683 |
| TPSG1 | -0.8543 | 2.1723 | -4.5307 | 0.0000 | 0.0000 | 2.9668 |

| PRKCG | -0.2859 | 0.3593 | -4.5305 | 0.0000 | 0.0000 | 2.9661 |
| --- | --- | --- | --- | --- | --- | --- |
| RPS14 | 0.0946 | 7.5137 | 4.5298 | 0.0000 | 0.0000 | 2.9630 |
| FGG | 0.2801 | 7.9998 | 4.5294 | 0.0000 | 0.0000 | 2.9611 |
| IBTK | 0.1356 | 6.3361 | 4.5288 | 0.0000 | 0.0000 | 2.9586 |
| IL33 | 0.4685 | 5.0506 | 4.5285 | 0.0000 | 0.0000 | 2.9576 |
| FANCF | -0.1305 | 5.7095 | -4.5280 | 0.0000 | 0.0000 | 2.9554 |
| STK17B | -0.3861 | 4.7466 | -4.5270 | 0.0000 | 0.0000 | 2.9512 |
| YIPF6 | 0.1064 | 6.1710 | 4.5266 | 0.0000 | 0.0000 | 2.9492 |
| AVEN | 0.1420 | 6.2746 | 4.5258 | 0.0000 | 0.0000 | 2.9458 |
| EPS15L1 | -0.1464 | 5.5051 | -4.5249 | 0.0000 | 0.0000 | 2.9421 |
| AGFG2 | 0.1331 | 6.7783 | 4.5247 | 0.0000 | 0.0000 | 2.9411 |
| FAAH | 0.2221 | 6.4618 | 4.5222 | 0.0000 | 0.0000 | 2.9306 |
| TBC1D2 | 0.2120 | 5.8006 | 4.5217 | 0.0000 | 0.0000 | 2.9284 |
| RAB13 | 0.0866 | 7.0648 | 4.5209 | 0.0000 | 0.0000 | 2.9250 |
| DNAJA4 | -0.5184 | 3.7258 | -4.5202 | 0.0000 | 0.0000 | 2.9220 |
| L3MBTL1 | -0.3814 | 2.9639 | -4.5170 | 0.0000 | 0.0000 | 2.9083 |
| NDUFB5 | 0.0879 | 6.6027 | 4.5163 | 0.0000 | 0.0000 | 2.9055 |
| ANKRD36 | -0.3770 | 1.7363 | -4.5158 | 0.0000 | 0.0000 | 2.9033 |
| STX18 | -0.1143 | 5.9773 | -4.5153 | 0.0000 | 0.0000 | 2.9012 |
| ABCC2 | 0.3963 | 6.6864 | 4.5147 | 0.0000 | 0.0000 | 2.8989 |
| WASF1 | -0.2755 | 5.4629 | -4.5147 | 0.0000 | 0.0000 | 2.8986 |
| MADD | -0.1085 | 5.8951 | -4.5133 | 0.0000 | 0.0000 | 2.8927 |
| ACSM3 | 0.5041 | 5.2030 | 4.5131 | 0.0000 | 0.0000 | 2.8920 |
| MTMR4 | 0.1657 | 6.3188 | 4.5116 | 0.0000 | 0.0000 | 2.8857 |
| GPR182 | 0.6599 | 2.0331 | 4.5112 | 0.0000 | 0.0000 | 2.8838 |
| CRK | 0.0972 | 6.6548 | 4.5100 | 0.0000 | 0.0000 | 2.8787 |
| COX7A2 | 0.1054 | 7.2451 | 4.5096 | 0.0000 | 0.0000 | 2.8770 |
| SPATA6 | -0.4897 | 3.3364 | -4.5087 | 0.0000 | 0.0000 | 2.8732 |
| BBS7 | -0.3490 | 4.3439 | -4.5087 | 0.0000 | 0.0000 | 2.8730 |
| SLC13A1 | -0.2727 | 0.1894 | -4.5086 | 0.0000 | 0.0000 | 2.8730 |
| PLA2G4A | -0.5739 | 2.7366 | -4.5084 | 0.0000 | 0.0000 | 2.8718 |
| DLX2 | -0.5135 | 0.6582 | -4.5080 | 0.0000 | 0.0000 | 2.8702 |
| CHI3L2 | -0.6259 | 2.1394 | -4.5074 | 0.0000 | 0.0000 | 2.8678 |
| AIM2 | -0.6575 | 2.4045 | -4.5065 | 0.0000 | 0.0000 | 2.8639 |
| RGS4 | -0.6601 | 3.1656 | -4.5060 | 0.0000 | 0.0000 | 2.8617 |
| GOLGA6A | 0.4183 | 0.6605 | 4.5037 | 0.0000 | 0.0000 | 2.8519 |
| **UTY** | 1.0116 | 2.9167 | 4.5013 | 0.0000 | 0.0000 | 2.8417 |
| EMP3 | -0.2463 | 6.0641 | -4.5004 | 0.0000 | 0.0000 | 2.8379 |
| ALOX5AP | -0.5046 | 4.7892 | -4.5003 | 0.0000 | 0.0000 | 2.8377 |
| TNIP1 | 0.0997 | 7.0301 | 4.5003 | 0.0000 | 0.0000 | 2.8375 |
| PLTP | -0.3254 | 6.2075 | -4.5002 | 0.0000 | 0.0000 | 2.8373 |
| GRIA2 | -0.3859 | 0.3528 | -4.4999 | 0.0000 | 0.0000 | 2.8360 |
| GRB7 | -0.3596 | 5.6203 | -4.4999 | 0.0000 | 0.0000 | 2.8359 |
| HOXA1 | -0.4206 | 1.4935 | -4.4997 | 0.0000 | 0.0000 | 2.8352 |
| HNF4A | 0.3099 | 7.0385 | 4.4995 | 0.0000 | 0.0000 | 2.8343 |
| VLDLR | -0.7803 | 3.2490 | -4.4994 | 0.0000 | 0.0000 | 2.8338 |
| SMAD2 | -0.1563 | 4.8823 | -4.4985 | 0.0000 | 0.0000 | 2.8299 |
| LSS | 0.1742 | 6.6291 | 4.4981 | 0.0000 | 0.0000 | 2.8283 |
| KCNN4 | -0.5435 | 3.2677 | -4.4967 | 0.0000 | 0.0000 | 2.8222 |
| MYD88 | 0.1134 | 6.5362 | 4.4958 | 0.0000 | 0.0000 | 2.8183 |
| USP47 | 0.1028 | 6.1363 | 4.4952 | 0.0000 | 0.0000 | 2.8160 |
| COMMD8 | -0.1648 | 6.0022 | -4.4945 | 0.0000 | 0.0000 | 2.8128 |
| MBD5 | -0.2263 | 4.0124 | -4.4937 | 0.0000 | 0.0000 | 2.8096 |

| PGF | -0.4013 | 4.6735 | -4.4936 | 0.0000 | 0.0000 | 2.8093 |
| --- | --- | --- | --- | --- | --- | --- |
| ZBBX | -0.2313 | 0.2131 | -4.4935 | 0.0000 | 0.0000 | 2.8089 |
| ITIH4 | 0.3884 | 6.0929 | 4.4929 | 0.0000 | 0.0000 | 2.8063 |
| C14orf79 | -0.2400 | 4.5670 | -4.4925 | 0.0000 | 0.0000 | 2.8044 |
| MTNR1B | -0.6956 | 0.6841 | -4.4907 | 0.0000 | 0.0000 | 2.7968 |
| NVL | -0.1461 | 5.5968 | -4.4884 | 0.0000 | 0.0000 | 2.7872 |
| KDM5B | -0.3038 | 5.4094 | -4.4871 | 0.0000 | 0.0000 | 2.7815 |
| ITGAV | -0.2194 | 6.1852 | -4.4866 | 0.0000 | 0.0000 | 2.7796 |
| POLR3D | -0.2006 | 5.0629 | -4.4849 | 0.0000 | 0.0000 | 2.7723 |
| DHX58 | 0.2344 | 5.9053 | 4.4849 | 0.0000 | 0.0000 | 2.7723 |
| OPN1SW | -0.3337 | 4.7647 | -4.4847 | 0.0000 | 0.0000 | 2.7716 |
| CLPTM1 | 0.0778 | 7.1005 | 4.4847 | 0.0000 | 0.0000 | 2.7714 |
| RAB1B | 0.0584 | 7.2698 | 4.4835 | 0.0000 | 0.0000 | 2.7663 |
| APOBEC3C | -0.3490 | 5.3291 | -4.4830 | 0.0000 | 0.0000 | 2.7643 |
| MKKS | -0.1196 | 6.3901 | -4.4829 | 0.0000 | 0.0000 | 2.7639 |
| CDK5R1 | -0.4100 | 3.6869 | -4.4815 | 0.0000 | 0.0000 | 2.7581 |
| MAP3K13 | 0.1792 | 5.7035 | 4.4814 | 0.0000 | 0.0000 | 2.7574 |
| SOX2 | -0.7644 | 1.2724 | -4.4778 | 0.0000 | 0.0000 | 2.7424 |
| KLHL35 | -0.4474 | 1.1106 | -4.4778 | 0.0000 | 0.0000 | 2.7422 |
| MATN3 | -0.7838 | 2.4960 | -4.4773 | 0.0000 | 0.0000 | 2.7403 |
| DNAJC9 | -0.2359 | 5.4486 | -4.4765 | 0.0000 | 0.0000 | 2.7370 |
| SLC17A1 | 0.4930 | 5.9169 | 4.4764 | 0.0000 | 0.0000 | 2.7364 |
| GPR132 | -0.5092 | 3.1038 | -4.4762 | 0.0000 | 0.0000 | 2.7355 |
| BBS1 | -0.2784 | 2.4350 | -4.4760 | 0.0000 | 0.0000 | 2.7349 |
| SERPINI2 | -0.2938 | 0.2996 | -4.4746 | 0.0000 | 0.0000 | 2.7287 |
| WWOX | 0.1987 | 5.4252 | 4.4732 | 0.0000 | 0.0000 | 2.7231 |
| C11orf16 | -0.2307 | 0.7585 | -4.4726 | 0.0000 | 0.0000 | 2.7202 |
| ORM1 | 0.2541 | 8.0961 | 4.4716 | 0.0000 | 0.0000 | 2.7162 |
| PTGER4 | -0.4752 | 4.0709 | -4.4714 | 0.0000 | 0.0000 | 2.7153 |
| DUSP9 | -0.6388 | 5.2418 | -4.4709 | 0.0000 | 0.0000 | 2.7133 |
| UFM1 | 0.1047 | 6.5569 | 4.4706 | 0.0000 | 0.0000 | 2.7122 |
| KIF5C | -0.5462 | 1.9650 | -4.4699 | 0.0000 | 0.0000 | 2.7089 |
| ORAI3 | 0.1527 | 6.4534 | 4.4699 | 0.0000 | 0.0000 | 2.7088 |
| TAF4B | -0.5412 | 1.9648 | -4.4685 | 0.0000 | 0.0000 | 2.7030 |
| RHOH | -0.5273 | 2.9814 | -4.4685 | 0.0000 | 0.0000 | 2.7030 |
| ERLIN1 | 0.1443 | 6.5031 | 4.4671 | 0.0000 | 0.0000 | 2.6970 |
| GNGT1 | -0.7224 | 1.0630 | -4.4669 | 0.0000 | 0.0000 | 2.6963 |
| UBE2G2 | 0.0850 | 6.3654 | 4.4656 | 0.0000 | 0.0000 | 2.6910 |
| FGB | 0.2668 | 8.0681 | 4.4640 | 0.0000 | 0.0000 | 2.6844 |
| TMEM30A | 0.0857 | 6.8354 | 4.4640 | 0.0000 | 0.0000 | 2.6842 |
| PSMA3 | 0.0831 | 6.9358 | 4.4629 | 0.0000 | 0.0000 | 2.6796 |
| CSF2RA | -0.5115 | 4.0753 | -4.4610 | 0.0000 | 0.0000 | 2.6714 |
| CNGA3 | -0.1574 | 0.0859 | -4.4596 | 0.0000 | 0.0000 | 2.6658 |
| PLEKHO1 | -0.2594 | 5.6538 | -4.4585 | 0.0000 | 0.0000 | 2.6611 |
| ARHGAP35 | 0.1101 | 6.4523 | 4.4566 | 0.0000 | 0.0000 | 2.6529 |
| HDAC1 | -0.0802 | 6.8145 | -4.4559 | 0.0000 | 0.0000 | 2.6502 |
| MKRN1 | 0.0706 | 6.5727 | 4.4545 | 0.0000 | 0.0000 | 2.6442 |
| SULT2B1 | -0.7364 | 2.0071 | -4.4540 | 0.0000 | 0.0000 | 2.6423 |
| SERPINB3 | -0.5024 | 0.4485 | -4.4515 | 0.0000 | 0.0000 | 2.6315 |
| DEAF1 | -0.1275 | 5.9927 | -4.4505 | 0.0000 | 0.0000 | 2.6275 |
| ACAD8 | 0.1519 | 5.7749 | 4.4505 | 0.0000 | 0.0000 | 2.6274 |
| FGFBP1 | -0.4898 | 0.5721 | -4.4497 | 0.0000 | 0.0000 | 2.6242 |
| LRRC19 | -0.6024 | 1.4767 | -4.4495 | 0.0000 | 0.0000 | 2.6234 |

| DVL2 | -0.1639 | 5.7952 | -4.4494 | 0.0000 | 0.0000 | 2.6229 |
| --- | --- | --- | --- | --- | --- | --- |
| ARF3 | -0.0627 | 6.7650 | -4.4492 | 0.0000 | 0.0000 | 2.6218 |
| SART3 | -0.1041 | 5.9817 | -4.4485 | 0.0000 | 0.0000 | 2.6188 |
| PROP1 | -0.1114 | 0.0759 | -4.4482 | 0.0000 | 0.0000 | 2.6178 |
| ZBTB32 | -0.4519 | 2.0252 | -4.4479 | 0.0000 | 0.0000 | 2.6165 |
| TFF3 | -0.5723 | 5.2116 | -4.4469 | 0.0000 | 0.0000 | 2.6123 |
| JUP | 0.0907 | 7.0686 | 4.4466 | 0.0000 | 0.0000 | 2.6112 |
| AAMP | 0.0628 | 7.0494 | 4.4456 | 0.0000 | 0.0000 | 2.6070 |
| KCNN1 | -0.5315 | 1.5181 | -4.4451 | 0.0000 | 0.0000 | 2.6049 |
| RFC2 | -0.1022 | 6.5712 | -4.4443 | 0.0000 | 0.0000 | 2.6015 |
| USP3 | -0.1063 | 5.6838 | -4.4443 | 0.0000 | 0.0000 | 2.6014 |
| TKTL1 | -0.6612 | 1.1844 | -4.4417 | 0.0000 | 0.0000 | 2.5906 |
| PDE4D | -0.4540 | 2.9702 | -4.4400 | 0.0000 | 0.0000 | 2.5835 |
| LMBRD1 | 0.1103 | 6.8735 | 4.4399 | 0.0000 | 0.0000 | 2.5828 |
| TIMM8A | 0.1789 | 6.0133 | 4.4399 | 0.0000 | 0.0000 | 2.5828 |
| ZNF235 | -0.2557 | 3.4586 | -4.4397 | 0.0000 | 0.0000 | 2.5823 |
| ATF3 | 0.2225 | 6.2362 | 4.4391 | 0.0000 | 0.0000 | 2.5798 |
| ZNF354A | -0.3910 | 4.9203 | -4.4390 | 0.0000 | 0.0000 | 2.5792 |
| RNF11 | 0.0998 | 6.7541 | 4.4387 | 0.0000 | 0.0000 | 2.5779 |
| HIST1H1C | 0.1699 | 7.5343 | 4.4386 | 0.0000 | 0.0000 | 2.5773 |
| LFNG | -0.3590 | 4.9005 | -4.4384 | 0.0000 | 0.0000 | 2.5768 |
| SNTA1 | 0.1509 | 6.5514 | 4.4383 | 0.0000 | 0.0000 | 2.5764 |
| STOML2 | 0.0826 | 7.2227 | 4.4374 | 0.0000 | 0.0000 | 2.5724 |
| ELOVL5 | 0.1242 | 6.9464 | 4.4371 | 0.0000 | 0.0000 | 2.5711 |
| B4GALT4 | -0.1399 | 5.5398 | -4.4355 | 0.0000 | 0.0000 | 2.5645 |
| NR1H4 | 0.2647 | 6.7500 | 4.4342 | 0.0000 | 0.0000 | 2.5593 |
| ATN1 | -0.0980 | 6.5966 | -4.4322 | 0.0000 | 0.0000 | 2.5505 |
| SCAMP4 | -0.1035 | 6.2440 | -4.4318 | 0.0000 | 0.0000 | 2.5492 |
| FLVCR2 | 0.2864 | 5.5721 | 4.4318 | 0.0000 | 0.0000 | 2.5489 |
| SCD5 | -0.5800 | 3.5286 | -4.4313 | 0.0000 | 0.0000 | 2.5471 |
| FAM49B | -0.1887 | 5.7900 | -4.4300 | 0.0000 | 0.0000 | 2.5414 |
| C1QL1 | -0.8246 | 4.6066 | -4.4291 | 0.0000 | 0.0000 | 2.5378 |
| OSTF1 | 0.0778 | 6.8197 | 4.4272 | 0.0000 | 0.0000 | 2.5299 |
| ADH7 | 0.5264 | 1.1808 | 4.4268 | 0.0000 | 0.0000 | 2.5283 |
| CRYAA | 0.4976 | 0.9033 | 4.4246 | 0.0000 | 0.0000 | 2.5188 |
| SLC2A8 | 0.1329 | 6.3358 | 4.4230 | 0.0000 | 0.0000 | 2.5121 |
| TGOLN2 | 0.0948 | 7.0918 | 4.4227 | 0.0000 | 0.0000 | 2.5108 |
| ALPP | -0.2295 | 0.2375 | -4.4224 | 0.0000 | 0.0000 | 2.5099 |
| UTF1 | -0.2693 | 0.3681 | -4.4217 | 0.0000 | 0.0000 | 2.5067 |
| ICAM5 | -0.6411 | 1.4704 | -4.4211 | 0.0000 | 0.0000 | 2.5042 |
| AQP1 | 0.2686 | 6.5415 | 4.4188 | 0.0000 | 0.0000 | 2.4949 |
| FKBP8 | 0.0739 | 7.4118 | 4.4180 | 0.0000 | 0.0000 | 2.4912 |
| RASIP1 | 0.2241 | 5.4993 | 4.4164 | 0.0000 | 0.0000 | 2.4848 |
| NFYB | 0.1455 | 6.1893 | 4.4142 | 0.0000 | 0.0000 | 2.4755 |
| SLITRK5 | -0.2179 | 0.3252 | -4.4131 | 0.0000 | 0.0000 | 2.4708 |
| TMBIM4 | 0.0995 | 6.5869 | 4.4129 | 0.0000 | 0.0000 | 2.4699 |
| OXCT1 | -0.5689 | 3.8831 | -4.4113 | 0.0000 | 0.0000 | 2.4634 |
| ZNF227 | -0.2014 | 4.8693 | -4.4108 | 0.0000 | 0.0000 | 2.4614 |
| PSMD3 | 0.0669 | 6.9660 | 4.4107 | 0.0000 | 0.0000 | 2.4610 |
| SLC39A8 | 0.2598 | 5.8680 | 4.4107 | 0.0000 | 0.0000 | 2.4609 |
| ADRM1 | 0.0725 | 7.2650 | 4.4083 | 0.0000 | 0.0000 | 2.4508 |
| NTRK1 | -0.3350 | 0.8856 | -4.4079 | 0.0000 | 0.0000 | 2.4492 |
| HMMR | -0.4160 | 5.1008 | -4.4077 | 0.0000 | 0.0000 | 2.4487 |

| TMEM123 | 0.0980 | 7.2947 | 4.4068 | 0.0000 | 0.0000 | 2.4449 |
| --- | --- | --- | --- | --- | --- | --- |
| CENPQ | -0.2426 | 5.4299 | -4.4066 | 0.0000 | 0.0000 | 2.4440 |
| VDAC1 | 0.0691 | 7.3664 | 4.4062 | 0.0000 | 0.0000 | 2.4424 |
| TEX10 | -0.1579 | 5.2860 | -4.4056 | 0.0000 | 0.0000 | 2.4398 |
| CPN1 | 0.4763 | 6.6738 | 4.4044 | 0.0000 | 0.0000 | 2.4346 |
| FAM193A | -0.1217 | 5.7603 | -4.4041 | 0.0000 | 0.0000 | 2.4333 |
| GALNT4 | -0.4146 | 1.1776 | -4.4035 | 0.0000 | 0.0000 | 2.4311 |
| MYH9 | 0.0668 | 7.3555 | 4.4035 | 0.0000 | 0.0000 | 2.4309 |
| ZNF45 | -0.2343 | 4.9888 | -4.4026 | 0.0000 | 0.0000 | 2.4272 |
| PPP2CA | 0.0627 | 6.7930 | 4.4013 | 0.0000 | 0.0000 | 2.4218 |
| FBXO38 | 0.1471 | 5.7344 | 4.4013 | 0.0000 | 0.0000 | 2.4218 |
| RYK | -0.1242 | 5.6845 | -4.4012 | 0.0000 | 0.0000 | 2.4214 |
| MRFAP1L1 | 0.0673 | 6.9199 | 4.3997 | 0.0000 | 0.0000 | 2.4153 |
| USP48 | -0.1132 | 5.6847 | -4.3995 | 0.0000 | 0.0000 | 2.4144 |
| PPP2R2B | -0.5182 | 1.4172 | -4.3990 | 0.0000 | 0.0000 | 2.4124 |
| ARL2 | -0.1715 | 6.3448 | -4.3989 | 0.0000 | 0.0000 | 2.4118 |
| PFKM | -0.3365 | 5.0242 | -4.3984 | 0.0000 | 0.0000 | 2.4099 |
| RBM28 | -0.1509 | 4.8698 | -4.3981 | 0.0000 | 0.0000 | 2.4085 |
| VCX2 | -0.3962 | 0.3361 | -4.3980 | 0.0000 | 0.0000 | 2.4080 |
| CALCOCO1 | 0.1440 | 6.0531 | 4.3952 | 0.0000 | 0.0000 | 2.3965 |
| ZNF22 | 0.1152 | 6.4111 | 4.3940 | 0.0000 | 0.0000 | 2.3916 |
| HINFP | -0.1422 | 4.8335 | -4.3939 | 0.0000 | 0.0000 | 2.3913 |
| HCRTR1 | -0.2038 | 0.4538 | -4.3935 | 0.0000 | 0.0000 | 2.3893 |
| CNGB1 | -0.4073 | 0.5824 | -4.3932 | 0.0000 | 0.0000 | 2.3881 |
| DSN1 | -0.1449 | 6.1701 | -4.3923 | 0.0000 | 0.0000 | 2.3846 |
| ZNF74 | -0.2083 | 4.8814 | -4.3918 | 0.0000 | 0.0000 | 2.3823 |
| IFNB1 | -0.1972 | 0.1521 | -4.3880 | 0.0000 | 0.0000 | 2.3667 |
| KCNK15 | -0.7675 | 1.5672 | -4.3872 | 0.0000 | 0.0000 | 2.3633 |
| UCP1 | -0.2560 | 0.2635 | -4.3868 | 0.0000 | 0.0000 | 2.3617 |
| ADNP2 | -0.1679 | 5.4660 | -4.3865 | 0.0000 | 0.0000 | 2.3603 |
| TOE1 | -0.1310 | 5.6950 | -4.3845 | 0.0000 | 0.0000 | 2.3523 |
| PEF1 | 0.0793 | 6.9968 | 4.3844 | 0.0000 | 0.0000 | 2.3518 |
| KCNMB4 | -0.4795 | 3.1770 | -4.3837 | 0.0000 | 0.0000 | 2.3489 |
| MRPL35 | 0.0805 | 6.4383 | 4.3830 | 0.0000 | 0.0000 | 2.3460 |
| HIST1H4A | -0.5478 | 1.0928 | -4.3827 | 0.0000 | 0.0000 | 2.3448 |
| AURKA | -0.2522 | 6.0387 | -4.3817 | 0.0000 | 0.0000 | 2.3406 |
| BTC | -0.6446 | 3.2216 | -4.3784 | 0.0000 | 0.0001 | 2.3267 |
| RHCE | 0.5987 | 3.1731 | 4.3754 | 0.0000 | 0.0001 | 2.3146 |
| RGS5 | 0.2576 | 6.5139 | 4.3745 | 0.0000 | 0.0001 | 2.3106 |
| MXD3 | -0.2956 | 5.1805 | -4.3741 | 0.0000 | 0.0001 | 2.3089 |
| SYT1 | -0.8656 | 2.8264 | -4.3739 | 0.0000 | 0.0001 | 2.3084 |
| FNBP4 | -0.1444 | 5.8052 | -4.3721 | 0.0000 | 0.0001 | 2.3010 |
| TDRD3 | 0.1550 | 5.2301 | 4.3715 | 0.0000 | 0.0001 | 2.2985 |
| MSH5 | -0.4817 | 3.2410 | -4.3712 | 0.0000 | 0.0001 | 2.2971 |
| HOXC4 | -0.5615 | 1.7073 | -4.3691 | 0.0000 | 0.0001 | 2.2884 |
| SPAG7 | 0.1237 | 6.6048 | 4.3683 | 0.0000 | 0.0001 | 2.2852 |
| DENND2D | -0.3597 | 5.1899 | -4.3674 | 0.0000 | 0.0001 | 2.2814 |
| EFR3B | -0.3246 | 1.5896 | -4.3650 | 0.0000 | 0.0001 | 2.2717 |
| INSL4 | -0.2835 | 0.1823 | -4.3645 | 0.0000 | 0.0001 | 2.2695 |
| RND1 | 0.2983 | 6.5188 | 4.3644 | 0.0000 | 0.0001 | 2.2692 |
| SLC7A9 | 0.4651 | 5.9897 | 4.3642 | 0.0000 | 0.0001 | 2.2682 |
| CASC1 | -0.3595 | 1.0766 | -4.3632 | 0.0000 | 0.0001 | 2.2641 |
| MRPS10 | 0.0707 | 6.8517 | 4.3630 | 0.0000 | 0.0001 | 2.2633 |

| OLFML2B | -0.3500 | 5.2666 | -4.3627 | 0.0000 | 0.0001 | 2.2621 |
| --- | --- | --- | --- | --- | --- | --- |
| ZNF211 | -0.2677 | 4.8484 | -4.3627 | 0.0000 | 0.0001 | 2.2619 |
| FAT2 | -0.2608 | 0.5541 | -4.3626 | 0.0000 | 0.0001 | 2.2617 |
| **EIF1AY** | 1.2177 | 3.8664 | 4.3623 | 0.0000 | 0.0001 | 2.2605 |
| FOXK2 | -0.1332 | 5.7878 | -4.3616 | 0.0000 | 0.0001 | 2.2576 |
| TNNI3 | -0.7095 | 1.1752 | -4.3613 | 0.0000 | 0.0001 | 2.2562 |
| PRPS1L1 | -0.3451 | 0.2858 | -4.3612 | 0.0000 | 0.0001 | 2.2557 |
| UQCR10 | 0.0917 | 7.4329 | 4.3610 | 0.0000 | 0.0001 | 2.2549 |
| RPS12 | 0.0817 | 7.9549 | 4.3608 | 0.0000 | 0.0001 | 2.2541 |
| ZNF480 | -0.1853 | 5.4551 | -4.3604 | 0.0000 | 0.0001 | 2.2527 |
| ZNF267 | -0.2153 | 4.4801 | -4.3604 | 0.0000 | 0.0001 | 2.2525 |
| EPHA5 | -0.2949 | 0.1995 | -4.3574 | 0.0000 | 0.0001 | 2.2400 |
| BDNF | -0.3662 | 0.8517 | -4.3564 | 0.0000 | 0.0001 | 2.2359 |
| FUT2 | -0.6549 | 3.3188 | -4.3558 | 0.0000 | 0.0001 | 2.2335 |
| ZNF335 | -0.1136 | 5.6029 | -4.3551 | 0.0000 | 0.0001 | 2.2306 |
| PPP1R12A | -0.1951 | 5.1994 | -4.3549 | 0.0000 | 0.0001 | 2.2301 |
| DEF6 | -0.3313 | 5.2492 | -4.3548 | 0.0000 | 0.0001 | 2.2295 |
| GCC2 | -0.2348 | 4.9877 | -4.3544 | 0.0000 | 0.0001 | 2.2278 |
| MEFV | -0.3637 | 1.7167 | -4.3540 | 0.0000 | 0.0001 | 2.2261 |
| UPF3B | -0.1400 | 5.8824 | -4.3531 | 0.0000 | 0.0001 | 2.2223 |
| HIF1A | -0.1768 | 6.4695 | -4.3530 | 0.0000 | 0.0001 | 2.2221 |
| ARMCX6 | -0.5011 | 4.4256 | -4.3523 | 0.0000 | 0.0001 | 2.2191 |
| ARHGAP8 | -0.3904 | 0.6725 | -4.3514 | 0.0000 | 0.0001 | 2.2157 |
| CEP290 | -0.2805 | 4.2830 | -4.3509 | 0.0000 | 0.0001 | 2.2134 |
| MFN2 | 0.0936 | 6.8802 | 4.3509 | 0.0000 | 0.0001 | 2.2134 |
| ADAR | 0.0710 | 7.0517 | 4.3506 | 0.0000 | 0.0001 | 2.2123 |
| KRT16 | -0.7637 | 1.1245 | -4.3496 | 0.0000 | 0.0001 | 2.2080 |
| PODNL1 | -0.4575 | 1.9975 | -4.3460 | 0.0000 | 0.0001 | 2.1933 |
| CYP7B1 | 0.4700 | 5.3980 | 4.3433 | 0.0000 | 0.0001 | 2.1821 |
| IRF4 | -0.6323 | 2.1633 | -4.3432 | 0.0000 | 0.0001 | 2.1817 |
| **UPK3A** | -1.0825 | 3.5743 | -4.3428 | 0.0000 | 0.0001 | 2.1803 |
| BCAS2 | 0.0735 | 6.8532 | 4.3415 | 0.0000 | 0.0001 | 2.1750 |
| KLK14 | -0.4707 | 1.4795 | -4.3396 | 0.0000 | 0.0001 | 2.1672 |
| ZNF701 | -0.5129 | 3.0797 | -4.3376 | 0.0000 | 0.0001 | 2.1589 |
| SLC39A4 | -0.7189 | 4.6712 | -4.3358 | 0.0000 | 0.0001 | 2.1513 |
| GIMAP6 | 0.2817 | 5.3735 | 4.3356 | 0.0000 | 0.0001 | 2.1506 |
| EHD4 | 0.1275 | 6.2038 | 4.3353 | 0.0000 | 0.0001 | 2.1495 |
| SERPINE1 | -0.2742 | 6.7986 | -4.3351 | 0.0000 | 0.0001 | 2.1487 |
| ZNF550 | -0.3925 | 4.3993 | -4.3349 | 0.0000 | 0.0001 | 2.1478 |
| ELP3 | 0.1336 | 6.0345 | 4.3327 | 0.0000 | 0.0001 | 2.1386 |
| CTSD | 0.0832 | 7.8597 | 4.3323 | 0.0000 | 0.0001 | 2.1370 |
| SULT1B1 | 0.6623 | 4.5636 | 4.3312 | 0.0000 | 0.0001 | 2.1324 |
| ZNF606 | -0.3522 | 4.3773 | -4.3305 | 0.0000 | 0.0001 | 2.1298 |
| SIDT2 | 0.1249 | 6.0412 | 4.3284 | 0.0000 | 0.0001 | 2.1210 |
| FGF23 | -0.3470 | 0.2949 | -4.3276 | 0.0000 | 0.0001 | 2.1178 |
| CHST1 | -0.4878 | 4.3187 | -4.3273 | 0.0000 | 0.0001 | 2.1164 |
| DTX2 | -0.1447 | 5.5448 | -4.3262 | 0.0000 | 0.0001 | 2.1122 |
| USE1 | 0.1312 | 6.5365 | 4.3253 | 0.0000 | 0.0001 | 2.1086 |
| CSPP1 | -0.2410 | 5.1336 | -4.3253 | 0.0000 | 0.0001 | 2.1084 |
| PARD6B | -0.2411 | 5.1158 | -4.3244 | 0.0000 | 0.0001 | 2.1049 |
| TSFM | 0.0932 | 6.5482 | 4.3225 | 0.0000 | 0.0001 | 2.0968 |
| MAP3K9 | -0.2923 | 3.8528 | -4.3208 | 0.0000 | 0.0001 | 2.0901 |
| MCRS1 | -0.0715 | 6.6306 | -4.3205 | 0.0000 | 0.0001 | 2.0890 |

| ATP5D | 0.1217 | 7.1688 | 4.3198 | 0.0000 | 0.0001 | 2.0860 |
| --- | --- | --- | --- | --- | --- | --- |
| RPS18 | 0.0780 | 7.8808 | 4.3197 | 0.0000 | 0.0001 | 2.0857 |
| CDH11 | -0.6200 | 3.2428 | -4.3195 | 0.0000 | 0.0001 | 2.0845 |
| TM4SF5 | 0.3476 | 7.4521 | 4.3187 | 0.0000 | 0.0001 | 2.0816 |
| FDPS | 0.1251 | 7.2096 | 4.3182 | 0.0000 | 0.0001 | 2.0792 |
| HAUS7 | -0.3330 | 3.8750 | -4.3175 | 0.0000 | 0.0001 | 2.0767 |
| ATAT1 | -0.2501 | 5.1296 | -4.3175 | 0.0000 | 0.0001 | 2.0764 |
| LILRB5 | 0.4186 | 4.1068 | 4.3160 | 0.0000 | 0.0001 | 2.0705 |
| HIST1H1E | -0.5265 | 3.1914 | -4.3155 | 0.0000 | 0.0001 | 2.0684 |
| MYOM1 | 0.4751 | 5.7812 | 4.3145 | 0.0000 | 0.0001 | 2.0644 |
| RNF128 | 0.1464 | 6.9194 | 4.3141 | 0.0000 | 0.0001 | 2.0627 |
| ADD3 | -0.2921 | 5.6782 | -4.3138 | 0.0000 | 0.0001 | 2.0616 |
| FABP5 | -0.2969 | 5.4043 | -4.3137 | 0.0000 | 0.0001 | 2.0610 |
| IDH3B | 0.0768 | 6.9958 | 4.3137 | 0.0000 | 0.0001 | 2.0609 |
| PLAGL1 | -0.5325 | 2.9646 | -4.3129 | 0.0000 | 0.0001 | 2.0576 |
| HDGF | 0.0629 | 7.4616 | 4.3105 | 0.0000 | 0.0001 | 2.0478 |
| GPKOW | 0.0818 | 6.6884 | 4.3104 | 0.0000 | 0.0001 | 2.0473 |
| CACNA1F | -0.3296 | 0.8099 | -4.3093 | 0.0000 | 0.0001 | 2.0430 |
| CTAGE5 | 0.1884 | 4.7625 | 4.3091 | 0.0000 | 0.0001 | 2.0423 |
| ITFG1 | 0.1615 | 6.2117 | 4.3090 | 0.0000 | 0.0001 | 2.0420 |
| VNN2 | -0.6120 | 5.1777 | -4.3086 | 0.0000 | 0.0001 | 2.0404 |
| HNRNPF | 0.0561 | 7.2507 | 4.3081 | 0.0000 | 0.0001 | 2.0380 |
| SIM2 | -0.4180 | 2.4255 | -4.3072 | 0.0000 | 0.0001 | 2.0343 |
| PTK2B | 0.2288 | 5.2805 | 4.3071 | 0.0000 | 0.0001 | 2.0343 |
| NAGK | -0.1099 | 5.7983 | -4.3070 | 0.0000 | 0.0001 | 2.0337 |
| TAX1BP1 | 0.0826 | 6.6234 | 4.3068 | 0.0000 | 0.0001 | 2.0328 |
| PUF60 | 0.1014 | 7.0901 | 4.3059 | 0.0000 | 0.0001 | 2.0293 |
| TACR2 | -0.4973 | 1.8621 | -4.3059 | 0.0000 | 0.0001 | 2.0292 |
| MYL6B | -0.1786 | 6.2133 | -4.3041 | 0.0000 | 0.0001 | 2.0217 |
| GCC1 | 0.1258 | 5.9661 | 4.3022 | 0.0000 | 0.0001 | 2.0142 |
| SMPD2 | -0.1628 | 5.8226 | -4.2989 | 0.0000 | 0.0001 | 2.0007 |
| IZUMO4 | 0.4125 | 4.2033 | 4.2988 | 0.0000 | 0.0001 | 2.0005 |
| TPM2 | -0.2060 | 6.3417 | -4.2986 | 0.0000 | 0.0001 | 1.9993 |
| MTNR1A | -0.2742 | 0.3173 | -4.2984 | 0.0000 | 0.0001 | 1.9986 |
| SLC16A6 | -0.4927 | 3.0330 | -4.2966 | 0.0000 | 0.0001 | 1.9913 |
| FPR1 | -0.5721 | 3.7700 | -4.2952 | 0.0000 | 0.0001 | 1.9855 |
| ING4 | 0.1104 | 6.3525 | 4.2947 | 0.0000 | 0.0001 | 1.9837 |
| CREB1 | -0.1479 | 5.2469 | -4.2947 | 0.0000 | 0.0001 | 1.9835 |
| LMO1 | -0.4348 | 0.4959 | -4.2932 | 0.0000 | 0.0001 | 1.9776 |
| GUSB | 0.0932 | 7.1092 | 4.2919 | 0.0000 | 0.0001 | 1.9721 |
| SGSH | -0.1250 | 5.8304 | -4.2908 | 0.0000 | 0.0001 | 1.9677 |
| BNC1 | -0.3253 | 0.5731 | -4.2892 | 0.0000 | 0.0001 | 1.9612 |
| BMS1 | -0.0833 | 5.9374 | -4.2876 | 0.0000 | 0.0001 | 1.9548 |
| RBM12B | -0.2457 | 4.6465 | -4.2868 | 0.0000 | 0.0001 | 1.9515 |
| RELA | 0.0582 | 6.6511 | 4.2867 | 0.0000 | 0.0001 | 1.9509 |
| PLAU | -0.2652 | 5.4195 | -4.2863 | 0.0000 | 0.0001 | 1.9493 |
| SOX21 | -0.4136 | 0.5020 | -4.2826 | 0.0000 | 0.0001 | 1.9346 |
| PDE9A | -0.5766 | 4.5287 | -4.2818 | 0.0000 | 0.0001 | 1.9313 |
| IL18 | -0.4305 | 4.7808 | -4.2812 | 0.0000 | 0.0001 | 1.9286 |
| EFNA1 | 0.1215 | 7.4653 | 4.2801 | 0.0000 | 0.0001 | 1.9243 |
| GTF3C4 | -0.1596 | 5.2816 | -4.2795 | 0.0000 | 0.0001 | 1.9217 |
| MRPL28 | 0.1054 | 6.9692 | 4.2772 | 0.0000 | 0.0001 | 1.9126 |
| CRLF3 | -0.1962 | 5.0889 | -4.2771 | 0.0000 | 0.0001 | 1.9120 |

| ZNF223 | -0.3448 | 2.6819 | -4.2770 | 0.0000 | 0.0001 | 1.9116 |
| --- | --- | --- | --- | --- | --- | --- |
| GRID2 | -0.1658 | 0.1402 | -4.2763 | 0.0000 | 0.0001 | 1.9089 |
| MLEC | 0.0893 | 7.0656 | 4.2752 | 0.0000 | 0.0001 | 1.9045 |
| BATF3 | -0.4092 | 3.4418 | -4.2745 | 0.0000 | 0.0001 | 1.9018 |
| ARHGAP1 | -0.0767 | 6.5723 | -4.2732 | 0.0000 | 0.0001 | 1.8963 |
| UQCRB | 0.1249 | 6.9257 | 4.2718 | 0.0000 | 0.0001 | 1.8908 |
| GPS1 | 0.0894 | 6.8699 | 4.2703 | 0.0000 | 0.0001 | 1.8845 |
| DMC1 | -0.5280 | 1.8309 | -4.2701 | 0.0000 | 0.0001 | 1.8837 |
| SNX17 | 0.0596 | 7.0090 | 4.2699 | 0.0000 | 0.0001 | 1.8829 |
| P2RX4 | -0.2139 | 5.7086 | -4.2696 | 0.0000 | 0.0001 | 1.8820 |
| GNRHR | -0.2529 | 0.6014 | -4.2696 | 0.0000 | 0.0001 | 1.8818 |
| CYB561 | -0.3714 | 5.9077 | -4.2691 | 0.0000 | 0.0001 | 1.8797 |
| CCIN | -0.3324 | 0.7357 | -4.2687 | 0.0000 | 0.0001 | 1.8782 |
| ANKRD27 | -0.2060 | 5.3817 | -4.2671 | 0.0000 | 0.0001 | 1.8715 |
| BCL2A1 | -0.5097 | 4.4020 | -4.2670 | 0.0000 | 0.0001 | 1.8712 |
| MBNL3 | 0.3198 | 6.3359 | 4.2659 | 0.0000 | 0.0001 | 1.8668 |
| RNF41 | -0.0923 | 5.7916 | -4.2658 | 0.0000 | 0.0001 | 1.8663 |
| C14orf105 | -0.4882 | 5.3731 | -4.2653 | 0.0000 | 0.0001 | 1.8646 |
| RGS10 | -0.2831 | 5.8599 | -4.2646 | 0.0000 | 0.0001 | 1.8615 |
| NUDT3 | -0.1393 | 5.2770 | -4.2641 | 0.0000 | 0.0001 | 1.8596 |
| GHR | 0.4250 | 5.8823 | 4.2640 | 0.0000 | 0.0001 | 1.8592 |
| PHF7 | 0.2313 | 4.9258 | 4.2612 | 0.0000 | 0.0001 | 1.8479 |
| ZNF81 | -0.2857 | 2.8893 | -4.2607 | 0.0000 | 0.0001 | 1.8458 |
| TUB | -0.7154 | 2.8058 | -4.2583 | 0.0000 | 0.0001 | 1.8362 |
| SLC2A4 | 0.4991 | 4.5379 | 4.2580 | 0.0000 | 0.0001 | 1.8348 |
| A2M | 0.1635 | 7.6886 | 4.2576 | 0.0000 | 0.0001 | 1.8333 |
| FABP1 | 0.4750 | 7.4755 | 4.2555 | 0.0000 | 0.0001 | 1.8248 |
| LAIR1 | -0.3850 | 4.6178 | -4.2553 | 0.0000 | 0.0001 | 1.8240 |
| TOR1A | 0.0916 | 6.4563 | 4.2526 | 0.0000 | 0.0001 | 1.8134 |
| CDK5RAP3 | 0.0946 | 6.7972 | 4.2526 | 0.0000 | 0.0001 | 1.8132 |
| KIAA1456 | -0.6218 | 1.9849 | -4.2526 | 0.0000 | 0.0001 | 1.8131 |
| SPECC1L | 0.1227 | 5.9955 | 4.2519 | 0.0000 | 0.0001 | 1.8102 |
| HIRIP3 | 0.1558 | 6.0792 | 4.2510 | 0.0000 | 0.0001 | 1.8069 |
| CCDC81 | -0.2714 | 1.6147 | -4.2510 | 0.0000 | 0.0001 | 1.8067 |
| HIVEP3 | -0.3835 | 3.1417 | -4.2485 | 0.0000 | 0.0001 | 1.7968 |
| CDYL | -0.1479 | 5.5155 | -4.2483 | 0.0000 | 0.0001 | 1.7958 |
| FAM131A | -0.2147 | 4.9261 | -4.2473 | 0.0000 | 0.0001 | 1.7920 |
| EFHD1 | 0.4623 | 5.5093 | 4.2473 | 0.0000 | 0.0001 | 1.7919 |
| MIS18BP1 | -0.2871 | 4.6823 | -4.2473 | 0.0000 | 0.0001 | 1.7917 |
| CCDC47 | 0.0823 | 6.9874 | 4.2470 | 0.0000 | 0.0001 | 1.7908 |
| FAT4 | 0.5442 | 3.1032 | 4.2460 | 0.0000 | 0.0001 | 1.7865 |
| FJX1 | -0.3026 | 4.7941 | -4.2445 | 0.0000 | 0.0001 | 1.7807 |
| IL15 | -0.4153 | 3.0801 | -4.2439 | 0.0000 | 0.0001 | 1.7781 |
| UMPS | 0.0990 | 6.0425 | 4.2437 | 0.0000 | 0.0001 | 1.7772 |
| UBA5 | 0.0877 | 6.0581 | 4.2420 | 0.0000 | 0.0001 | 1.7706 |
| CYTH4 | -0.3214 | 4.7986 | -4.2417 | 0.0000 | 0.0001 | 1.7695 |
| CHST2 | -0.3129 | 4.3230 | -4.2412 | 0.0000 | 0.0001 | 1.7673 |
| RAB33A | -0.4621 | 2.9331 | -4.2411 | 0.0000 | 0.0001 | 1.7669 |
| MAP3K6 | -0.2444 | 5.0680 | -4.2399 | 0.0000 | 0.0001 | 1.7621 |
| PACS2 | -0.1268 | 5.5926 | -4.2374 | 0.0000 | 0.0001 | 1.7520 |
| EIF1B | 0.0714 | 6.7759 | 4.2359 | 0.0000 | 0.0001 | 1.7459 |
| RAB8B | -0.1864 | 5.6586 | -4.2357 | 0.0000 | 0.0001 | 1.7451 |
| IARS | -0.1204 | 6.3376 | -4.2354 | 0.0000 | 0.0001 | 1.7439 |

| SHBG | 0.5300 | 5.8707 | 4.2353 | 0.0000 | 0.0001 | 1.7437 |
| --- | --- | --- | --- | --- | --- | --- |
| THPO | 0.3706 | 6.2346 | 4.2352 | 0.0000 | 0.0001 | 1.7433 |
| POPDC3 | -0.8418 | 1.3651 | -4.2350 | 0.0000 | 0.0001 | 1.7426 |
| GPATCH2 | -0.1919 | 4.7434 | -4.2347 | 0.0000 | 0.0001 | 1.7412 |
| NKX2-5 | -0.4619 | 0.4690 | -4.2339 | 0.0000 | 0.0001 | 1.7380 |
| HSPE1 | 0.0890 | 7.3765 | 4.2328 | 0.0000 | 0.0001 | 1.7336 |
| **PEG10** | -1.0519 | 4.5810 | -4.2327 | 0.0000 | 0.0001 | 1.7330 |
| ZNF222 | -0.2982 | 4.4428 | -4.2310 | 0.0000 | 0.0001 | 1.7265 |
| ATP6V1B1 | -0.5789 | 2.0928 | -4.2293 | 0.0000 | 0.0001 | 1.7194 |
| ALPPL2 | -0.2947 | 0.2752 | -4.2276 | 0.0000 | 0.0001 | 1.7126 |
| RALA | -0.0697 | 6.4534 | -4.2275 | 0.0000 | 0.0001 | 1.7124 |
| NDRG4 | -0.4804 | 2.3540 | -4.2264 | 0.0000 | 0.0001 | 1.7080 |
| DSTYK | -0.2202 | 4.9532 | -4.2260 | 0.0000 | 0.0001 | 1.7061 |
| CCNG2 | -0.2509 | 5.0081 | -4.2252 | 0.0000 | 0.0001 | 1.7033 |
| ZHX3 | 0.1828 | 5.4906 | 4.2245 | 0.0000 | 0.0001 | 1.7005 |
| MCF2L2 | -0.4532 | 0.9896 | -4.2237 | 0.0000 | 0.0001 | 1.6969 |
| ZNF43 | -0.5268 | 3.3316 | -4.2203 | 0.0000 | 0.0001 | 1.6834 |
| ATG12 | 0.0958 | 5.8945 | 4.2201 | 0.0000 | 0.0001 | 1.6828 |
| KLRC3 | -0.2417 | 0.3181 | -4.2197 | 0.0000 | 0.0001 | 1.6809 |
| POLE2 | -0.3197 | 4.8884 | -4.2193 | 0.0000 | 0.0001 | 1.6794 |
| **MEP1A** | -1.0022 | 2.7368 | -4.2180 | 0.0000 | 0.0001 | 1.6743 |
| HTR1F | -0.2553 | 0.4120 | -4.2174 | 0.0000 | 0.0001 | 1.6721 |
| AURKAIP1 | 0.1007 | 7.2772 | 4.2170 | 0.0000 | 0.0001 | 1.6702 |
| GJB1 | 0.2548 | 7.5271 | 4.2165 | 0.0000 | 0.0001 | 1.6682 |
| SP100 | 0.1477 | 5.9265 | 4.2162 | 0.0000 | 0.0001 | 1.6673 |
| GDAP1L1 | -0.3503 | 0.6233 | -4.2162 | 0.0000 | 0.0001 | 1.6671 |
| MPG | 0.1052 | 6.6465 | 4.2155 | 0.0000 | 0.0001 | 1.6644 |
| CELF1 | 0.0611 | 6.4518 | 4.2146 | 0.0000 | 0.0001 | 1.6608 |
| CDH3 | -0.4860 | 1.2616 | -4.2127 | 0.0000 | 0.0001 | 1.6530 |
| ASB1 | -0.1764 | 5.3271 | -4.2119 | 0.0000 | 0.0001 | 1.6500 |
| PRPF39 | -0.1467 | 5.5285 | -4.2117 | 0.0000 | 0.0001 | 1.6491 |
| SRRT | -0.0569 | 6.7519 | -4.2116 | 0.0000 | 0.0001 | 1.6486 |
| TRNAU1AP | -0.1468 | 5.5549 | -4.2111 | 0.0000 | 0.0001 | 1.6467 |
| SAE1 | -0.0751 | 6.8374 | -4.2104 | 0.0000 | 0.0001 | 1.6437 |
| ZNF426 | -0.4111 | 4.2249 | -4.2103 | 0.0000 | 0.0001 | 1.6435 |
| ATP8B2 | -0.3721 | 4.9832 | -4.2102 | 0.0000 | 0.0001 | 1.6430 |
| NAA15 | -0.1217 | 5.5652 | -4.2099 | 0.0000 | 0.0001 | 1.6417 |
| CHFR | -0.2519 | 4.5722 | -4.2098 | 0.0000 | 0.0001 | 1.6416 |
| KLRF1 | 0.4434 | 2.7628 | 4.2097 | 0.0000 | 0.0001 | 1.6412 |
| ATR | -0.1798 | 4.9053 | -4.2096 | 0.0000 | 0.0001 | 1.6409 |
| SYNJ2 | -0.2635 | 5.2590 | -4.2094 | 0.0000 | 0.0001 | 1.6399 |
| HIST1H2BK | 0.1454 | 7.3086 | 4.2090 | 0.0000 | 0.0001 | 1.6384 |
| DDX39A | -0.1236 | 6.6360 | -4.2084 | 0.0000 | 0.0001 | 1.6359 |
| LDLR | 0.1660 | 6.4827 | 4.2079 | 0.0000 | 0.0001 | 1.6340 |
| CRELD1 | 0.0975 | 6.5505 | 4.2078 | 0.0000 | 0.0001 | 1.6335 |
| FGF5 | -0.2509 | 0.1481 | -4.2059 | 0.0000 | 0.0001 | 1.6258 |
| GMCL1 | -0.1460 | 5.8780 | -4.2055 | 0.0000 | 0.0001 | 1.6244 |
| NDOR1 | -0.1727 | 5.6355 | -4.2047 | 0.0000 | 0.0001 | 1.6213 |
| PAGE1 | -0.9637 | 1.5820 | -4.2042 | 0.0000 | 0.0001 | 1.6193 |
| TREH | 0.7310 | 3.7202 | 4.2042 | 0.0000 | 0.0001 | 1.6190 |
| TFCP2L1 | -0.5510 | 1.6563 | -4.2034 | 0.0000 | 0.0001 | 1.6160 |
| EXOG | -0.2156 | 4.3881 | -4.2017 | 0.0000 | 0.0001 | 1.6094 |
| PVR | 0.1032 | 6.7934 | 4.2016 | 0.0000 | 0.0001 | 1.6089 |

| AZIN1 | 0.1188 | 6.7868 | 4.2016 | 0.0000 | 0.0001 | 1.6087 |
| --- | --- | --- | --- | --- | --- | --- |
| GLRA2 | -0.0956 | 0.0634 | -4.2009 | 0.0000 | 0.0001 | 1.6061 |
| FGFR1 | -0.4479 | 4.1934 | -4.1998 | 0.0000 | 0.0001 | 1.6018 |
| ENAH | -0.2316 | 5.9158 | -4.1979 | 0.0000 | 0.0001 | 1.5942 |
| CIB1 | 0.0803 | 7.3860 | 4.1978 | 0.0000 | 0.0001 | 1.5938 |
| ADCY7 | -0.4970 | 2.7571 | -4.1975 | 0.0000 | 0.0001 | 1.5925 |
| CNTN5 | -0.3798 | 0.5315 | -4.1973 | 0.0000 | 0.0001 | 1.5918 |
| DPM3 | 0.1196 | 7.2656 | 4.1960 | 0.0000 | 0.0001 | 1.5866 |
| SCML1 | 0.2040 | 5.9545 | 4.1959 | 0.0000 | 0.0001 | 1.5859 |
| PRPS1 | 0.0950 | 6.7805 | 4.1959 | 0.0000 | 0.0001 | 1.5859 |
| BEAN1 | -0.4464 | 1.6411 | -4.1954 | 0.0000 | 0.0001 | 1.5841 |
| ARAP2 | -0.3618 | 4.0598 | -4.1954 | 0.0000 | 0.0001 | 1.5840 |
| GTF2B | 0.0869 | 6.4777 | 4.1953 | 0.0000 | 0.0001 | 1.5836 |
| MRPL4 | 0.1039 | 6.6356 | 4.1931 | 0.0000 | 0.0001 | 1.5749 |
| RNF10 | 0.0565 | 6.9068 | 4.1926 | 0.0000 | 0.0001 | 1.5732 |
| OPA1 | 0.1041 | 6.1793 | 4.1914 | 0.0000 | 0.0001 | 1.5682 |
| KLK7 | -0.3284 | 0.3288 | -4.1913 | 0.0000 | 0.0001 | 1.5678 |
| SLC10A2 | -0.1879 | 0.1110 | -4.1912 | 0.0000 | 0.0001 | 1.5675 |
| VILL | -0.3538 | 3.0869 | -4.1906 | 0.0000 | 0.0001 | 1.5650 |
| SIRPB1 | -0.4673 | 1.9710 | -4.1904 | 0.0000 | 0.0001 | 1.5642 |
| PSTPIP2 | 0.3147 | 5.5351 | 4.1889 | 0.0000 | 0.0001 | 1.5582 |
| SLC13A4 | -0.3517 | 1.8113 | -4.1881 | 0.0000 | 0.0001 | 1.5550 |
| RAB31 | -0.3044 | 5.2352 | -4.1881 | 0.0000 | 0.0001 | 1.5549 |
| EGF | -0.7518 | 1.3338 | -4.1863 | 0.0000 | 0.0001 | 1.5480 |
| ANK3 | -0.5698 | 2.5861 | -4.1861 | 0.0000 | 0.0001 | 1.5470 |
| CRTC1 | -0.1688 | 5.2057 | -4.1860 | 0.0000 | 0.0001 | 1.5468 |
| RBFOX1 | -0.1700 | 0.1306 | -4.1844 | 0.0000 | 0.0001 | 1.5405 |
| SLC25A36 | -0.4266 | 3.6263 | -4.1842 | 0.0000 | 0.0001 | 1.5397 |
| PIK3CD | -0.3686 | 4.4485 | -4.1836 | 0.0000 | 0.0001 | 1.5373 |
| PXMP4 | 0.1597 | 6.0147 | 4.1834 | 0.0000 | 0.0001 | 1.5363 |
| ASAP1 | -0.2380 | 5.5552 | -4.1828 | 0.0000 | 0.0001 | 1.5342 |
| CCT6B | 0.4313 | 3.4290 | 4.1818 | 0.0000 | 0.0001 | 1.5300 |
| PJA1 | -0.1937 | 5.8304 | -4.1816 | 0.0000 | 0.0001 | 1.5291 |
| MRPS18C | 0.1273 | 5.4875 | 4.1799 | 0.0000 | 0.0001 | 1.5226 |
| GRM7 | -0.3769 | 0.5654 | -4.1798 | 0.0000 | 0.0001 | 1.5222 |
| BZW2 | -0.1312 | 6.3183 | -4.1798 | 0.0000 | 0.0001 | 1.5222 |
| ELOVL4 | -0.5166 | 1.7741 | -4.1791 | 0.0000 | 0.0001 | 1.5193 |
| ZNF665 | -0.4490 | 1.6564 | -4.1784 | 0.0000 | 0.0001 | 1.5165 |
| ERC2 | -0.3856 | 0.6601 | -4.1771 | 0.0000 | 0.0001 | 1.5115 |
| C19orf66 | 0.1311 | 6.6775 | 4.1768 | 0.0000 | 0.0001 | 1.5104 |
| VSNL1 | 0.6787 | 5.3548 | 4.1736 | 0.0000 | 0.0001 | 1.4976 |
| HOXA10 | -0.8940 | 2.6526 | -4.1721 | 0.0000 | 0.0001 | 1.4916 |
| SEC63 | 0.0942 | 6.5466 | 4.1719 | 0.0000 | 0.0001 | 1.4910 |
| NHP2 | 0.0887 | 6.9904 | 4.1714 | 0.0000 | 0.0001 | 1.4888 |
| FZD3 | -0.4583 | 2.5187 | -4.1712 | 0.0000 | 0.0001 | 1.4879 |
| CCDC6 | -0.1067 | 5.9438 | -4.1708 | 0.0000 | 0.0001 | 1.4865 |
| FECH | 0.1522 | 6.1469 | 4.1702 | 0.0000 | 0.0001 | 1.4839 |
| CLPB | 0.1440 | 5.9930 | 4.1689 | 0.0000 | 0.0001 | 1.4790 |
| ERMP1 | -0.1792 | 5.9174 | -4.1679 | 0.0000 | 0.0001 | 1.4750 |
| ANKRD53 | -0.5105 | 2.4000 | -4.1676 | 0.0000 | 0.0001 | 1.4737 |
| NDRG1 | -0.1823 | 6.6941 | -4.1650 | 0.0000 | 0.0001 | 1.4635 |
| RPS27L | 0.1352 | 6.3815 | 4.1633 | 0.0000 | 0.0001 | 1.4566 |
| ADAM18 | -0.1949 | 0.1160 | -4.1629 | 0.0000 | 0.0001 | 1.4552 |

| AFAP1 | -0.3979 | 4.2100 | -4.1622 | 0.0000 | 0.0001 | 1.4524 |
| --- | --- | --- | --- | --- | --- | --- |
| GALR2 | -0.4287 | 1.8440 | -4.1617 | 0.0000 | 0.0001 | 1.4506 |
| DBR1 | -0.1456 | 5.6156 | -4.1612 | 0.0000 | 0.0001 | 1.4485 |
| GNAI2 | 0.0610 | 6.9851 | 4.1611 | 0.0000 | 0.0001 | 1.4481 |
| APOBEC1 | -0.2895 | 0.1877 | -4.1599 | 0.0000 | 0.0001 | 1.4433 |
| MAN1C1 | 0.4561 | 4.8122 | 4.1582 | 0.0000 | 0.0001 | 1.4367 |
| LGI2 | -0.5262 | 2.5852 | -4.1581 | 0.0000 | 0.0001 | 1.4361 |
| TRIM32 | -0.1651 | 5.3569 | -4.1580 | 0.0000 | 0.0001 | 1.4359 |
| CCL23 | 0.4708 | 3.2696 | 4.1577 | 0.0000 | 0.0001 | 1.4348 |
| HCFC2 | 0.1860 | 5.2434 | 4.1571 | 0.0000 | 0.0001 | 1.4324 |
| RNF122 | -0.2183 | 5.2980 | -4.1570 | 0.0000 | 0.0001 | 1.4320 |
| **KDM5D** | 1.0593 | 3.5451 | 4.1561 | 0.0000 | 0.0001 | 1.4285 |
| IL3RA | 0.1785 | 5.8268 | 4.1561 | 0.0000 | 0.0001 | 1.4282 |
| RDH11 | 0.1185 | 6.8244 | 4.1551 | 0.0000 | 0.0001 | 1.4243 |
| ERP29 | 0.0716 | 7.3222 | 4.1543 | 0.0000 | 0.0001 | 1.4214 |
| COX6B1 | 0.0883 | 7.6946 | 4.1533 | 0.0000 | 0.0001 | 1.4174 |
| PCDHA5 | -0.2611 | 0.3420 | -4.1533 | 0.0000 | 0.0001 | 1.4174 |
| DGAT1 | 0.1247 | 6.7990 | 4.1530 | 0.0000 | 0.0001 | 1.4161 |
| PCSK6 | 0.3447 | 6.0926 | 4.1523 | 0.0000 | 0.0001 | 1.4133 |
| MAPK12 | -0.4747 | 4.2055 | -4.1505 | 0.0000 | 0.0001 | 1.4063 |
| PAN2 | 0.1571 | 6.0141 | 4.1502 | 0.0000 | 0.0001 | 1.4051 |
| FNDC8 | -0.2535 | 0.4766 | -4.1498 | 0.0000 | 0.0001 | 1.4036 |
| RNASEH2A | -0.1664 | 6.3885 | -4.1497 | 0.0000 | 0.0001 | 1.4031 |
| ZBTB17 | -0.0963 | 5.9727 | -4.1486 | 0.0000 | 0.0001 | 1.3990 |
| TMEM151B | -0.1517 | 0.3450 | -4.1486 | 0.0000 | 0.0001 | 1.3986 |
| ACIN1 | -0.0654 | 6.4226 | -4.1482 | 0.0000 | 0.0001 | 1.3974 |
| ALX4 | -0.2524 | 0.2888 | -4.1470 | 0.0000 | 0.0001 | 1.3924 |
| CD300A | -0.3267 | 5.0088 | -4.1467 | 0.0000 | 0.0001 | 1.3913 |
| ITIH2 | 0.2406 | 7.8412 | 4.1440 | 0.0000 | 0.0001 | 1.3805 |
| PTPN2 | -0.1461 | 5.5715 | -4.1436 | 0.0000 | 0.0001 | 1.3790 |
| C1orf105 | 0.5095 | 2.9870 | 4.1427 | 0.0000 | 0.0001 | 1.3755 |
| AGBL5 | -0.1467 | 5.8871 | -4.1425 | 0.0000 | 0.0001 | 1.3748 |
| MRC2 | -0.4759 | 4.7895 | -4.1398 | 0.0000 | 0.0001 | 1.3640 |
| AMPD2 | 0.1275 | 6.2922 | 4.1397 | 0.0000 | 0.0001 | 1.3638 |
| VAX2 | -0.6369 | 2.5655 | -4.1377 | 0.0000 | 0.0001 | 1.3558 |
| TM9SF3 | 0.0610 | 6.8816 | 4.1370 | 0.0000 | 0.0001 | 1.3531 |
| SPATS2L | -0.1001 | 6.5830 | -4.1366 | 0.0000 | 0.0001 | 1.3516 |
| NDUFB4 | 0.0686 | 7.1905 | 4.1365 | 0.0000 | 0.0001 | 1.3513 |
| ITM2C | -0.2323 | 6.6729 | -4.1360 | 0.0000 | 0.0001 | 1.3493 |
| SMARCA5 | -0.1155 | 6.0998 | -4.1359 | 0.0000 | 0.0001 | 1.3487 |
| LLGL1 | -0.2338 | 5.0729 | -4.1345 | 0.0000 | 0.0001 | 1.3433 |
| KRT24 | -0.2356 | 0.1903 | -4.1339 | 0.0000 | 0.0001 | 1.3411 |
| PCDHGA11 | -0.4088 | 1.3666 | -4.1338 | 0.0000 | 0.0001 | 1.3404 |
| CLIC1 | -0.0895 | 7.3844 | -4.1329 | 0.0000 | 0.0001 | 1.3370 |
| ARHGAP26 | -0.2487 | 4.5612 | -4.1326 | 0.0000 | 0.0001 | 1.3357 |
| FKBP14 | -0.2334 | 4.9768 | -4.1322 | 0.0000 | 0.0001 | 1.3341 |
| ZNF226 | -0.1955 | 4.8732 | -4.1317 | 0.0000 | 0.0001 | 1.3322 |
| MIER2 | -0.2125 | 5.6114 | -4.1316 | 0.0000 | 0.0001 | 1.3317 |
| PIGT | 0.0617 | 7.2645 | 4.1309 | 0.0000 | 0.0001 | 1.3291 |
| CHORDC1 | -0.2378 | 4.8731 | -4.1308 | 0.0000 | 0.0001 | 1.3289 |
| FNDC3A | 0.1747 | 6.2956 | 4.1305 | 0.0000 | 0.0001 | 1.3276 |
| EID1 | 0.0840 | 7.1069 | 4.1302 | 0.0000 | 0.0001 | 1.3263 |
| COL15A1 | 0.3746 | 5.4487 | 4.1296 | 0.0000 | 0.0001 | 1.3242 |

| BRDT | -0.4598 | 0.4092 | -4.1282 | 0.0000 | 0.0001 | 1.3186 |
| --- | --- | --- | --- | --- | --- | --- |
| SMTN | -0.1806 | 5.5619 | -4.1277 | 0.0000 | 0.0001 | 1.3168 |
| RGR | -0.0997 | 0.0643 | -4.1277 | 0.0000 | 0.0001 | 1.3165 |
| TFAP4 | -0.2002 | 4.7868 | -4.1251 | 0.0000 | 0.0001 | 1.3064 |
| PPP1R3D | -0.3715 | 3.8536 | -4.1247 | 0.0000 | 0.0001 | 1.3047 |
| CSNK2A1 | -0.0840 | 6.4309 | -4.1232 | 0.0000 | 0.0001 | 1.2989 |
| ODF2 | -0.1302 | 5.7924 | -4.1232 | 0.0000 | 0.0001 | 1.2988 |
| UBE2E3 | -0.0803 | 6.5465 | -4.1223 | 0.0000 | 0.0001 | 1.2956 |
| RPL15 | 0.0686 | 7.3612 | 4.1218 | 0.0000 | 0.0001 | 1.2937 |
| KRT83 | -0.3653 | 0.4528 | -4.1212 | 0.0000 | 0.0001 | 1.2910 |
| **RPS4Y1** | 1.1887 | 5.2623 | 4.1207 | 0.0000 | 0.0001 | 1.2890 |
| C7 | 0.7439 | 5.3460 | 4.1195 | 0.0000 | 0.0001 | 1.2845 |
| MAGEA11 | -0.6335 | 0.7049 | -4.1187 | 0.0000 | 0.0001 | 1.2814 |
| **DDX3Y** | 1.1415 | 4.0415 | 4.1183 | 0.0000 | 0.0001 | 1.2798 |
| SH2D3C | 0.2025 | 5.4699 | 4.1175 | 0.0000 | 0.0001 | 1.2766 |
| HDAC3 | 0.0790 | 6.4265 | 4.1173 | 0.0000 | 0.0001 | 1.2758 |
| ZRSR2 | -0.1179 | 5.7546 | -4.1155 | 0.0000 | 0.0001 | 1.2686 |
| KCNC4 | -0.3341 | 2.4204 | -4.1148 | 0.0000 | 0.0001 | 1.2662 |
| ZFP36L1 | 0.0843 | 7.1232 | 4.1139 | 0.0000 | 0.0001 | 1.2624 |
| TJAP1 | -0.1069 | 5.9454 | -4.1137 | 0.0000 | 0.0001 | 1.2619 |
| EIF3G | 0.0821 | 7.0916 | 4.1134 | 0.0000 | 0.0001 | 1.2607 |
| LIMK2 | -0.2152 | 5.9194 | -4.1129 | 0.0000 | 0.0001 | 1.2587 |
| CNTN1 | -0.7057 | 1.0428 | -4.1124 | 0.0000 | 0.0001 | 1.2568 |
| SERPINB4 | -0.3968 | 0.3209 | -4.1116 | 0.0000 | 0.0001 | 1.2537 |
| PLS3 | 0.1000 | 6.9406 | 4.1108 | 0.0000 | 0.0001 | 1.2506 |
| ENY2 | 0.1221 | 6.4900 | 4.1103 | 0.0000 | 0.0001 | 1.2486 |
| RBPJL | -0.3095 | 0.3987 | -4.1093 | 0.0000 | 0.0001 | 1.2446 |
| CCZ1 | -0.2605 | 4.1401 | -4.1090 | 0.0000 | 0.0001 | 1.2433 |
| NOD1 | -0.2131 | 4.1743 | -4.1089 | 0.0000 | 0.0001 | 1.2431 |
| PYGO1 | -0.4064 | 1.1203 | -4.1079 | 0.0000 | 0.0001 | 1.2389 |
| CTLA4 | -0.6369 | 3.2001 | -4.1071 | 0.0000 | 0.0001 | 1.2360 |
| DYNC2LI1 | -0.1392 | 5.3413 | -4.1068 | 0.0000 | 0.0001 | 1.2350 |
| TRANK1 | 0.2822 | 5.1932 | 4.1056 | 0.0000 | 0.0001 | 1.2303 |
| THOC1 | -0.1433 | 5.3283 | -4.1044 | 0.0000 | 0.0001 | 1.2253 |
| CSRNP3 | -0.4427 | 1.3072 | -4.1042 | 0.0000 | 0.0001 | 1.2246 |
| DUSP2 | -0.4530 | 4.7347 | -4.1041 | 0.0000 | 0.0001 | 1.2244 |
| RPS6KA6 | -0.5241 | 0.8412 | -4.1033 | 0.0001 | 0.0001 | 1.2213 |
| ADAM17 | -0.3323 | 4.8796 | -4.1033 | 0.0001 | 0.0001 | 1.2211 |
| CKAP4 | -0.1041 | 6.9991 | -4.1029 | 0.0001 | 0.0001 | 1.2198 |
| POLR2F | -0.1704 | 0.3669 | -4.1022 | 0.0001 | 0.0001 | 1.2171 |
| ARSD | 0.1931 | 6.2915 | 4.1015 | 0.0001 | 0.0001 | 1.2142 |
| TMED5 | 0.1200 | 6.5903 | 4.1013 | 0.0001 | 0.0001 | 1.2135 |
| HPSE2 | -0.2930 | 0.5269 | -4.1008 | 0.0001 | 0.0001 | 1.2114 |
| PVALB | -0.5420 | 1.4457 | -4.1001 | 0.0001 | 0.0001 | 1.2087 |
| RNF17 | -0.5852 | 0.6747 | -4.1001 | 0.0001 | 0.0001 | 1.2087 |
| HIC2 | -0.3363 | 4.3841 | -4.0981 | 0.0001 | 0.0001 | 1.2010 |
| MSMB | -0.7679 | 2.7937 | -4.0981 | 0.0001 | 0.0001 | 1.2009 |
| SOCS5 | -0.1402 | 5.5621 | -4.0974 | 0.0001 | 0.0002 | 1.1982 |
| AMH | -0.6204 | 2.1168 | -4.0961 | 0.0001 | 0.0002 | 1.1931 |
| KRT81 | -0.6231 | 1.9173 | -4.0953 | 0.0001 | 0.0002 | 1.1900 |
| ZMYM6 | -0.1345 | 4.4768 | -4.0950 | 0.0001 | 0.0002 | 1.1888 |
| C5orf42 | -0.3345 | 3.6197 | -4.0935 | 0.0001 | 0.0002 | 1.1829 |
| PAPSS1 | -0.1465 | 6.0274 | -4.0908 | 0.0001 | 0.0002 | 1.1724 |

| CHGA | -0.5975 | 1.2714 | -4.0902 | 0.0001 | 0.0002 | 1.1701 |
| --- | --- | --- | --- | --- | --- | --- |
| RBM4B | -0.1220 | 5.5153 | -4.0890 | 0.0001 | 0.0002 | 1.1653 |
| KCNJ14 | -0.3590 | 2.8489 | -4.0876 | 0.0001 | 0.0002 | 1.1599 |
| MATN4 | -0.3949 | 1.0088 | -4.0865 | 0.0001 | 0.0002 | 1.1559 |
| STK32B | -0.4027 | 1.8187 | -4.0854 | 0.0001 | 0.0002 | 1.1516 |
| SMPD3 | -0.4191 | 2.7076 | -4.0851 | 0.0001 | 0.0002 | 1.1502 |
| SERPINB2 | -0.3758 | 0.4475 | -4.0828 | 0.0001 | 0.0002 | 1.1413 |
| TFAP2C | -0.6136 | 1.0762 | -4.0825 | 0.0001 | 0.0002 | 1.1401 |
| MTO1 | 0.1472 | 5.2908 | 4.0807 | 0.0001 | 0.0002 | 1.1334 |
| GLI2 | -0.5266 | 1.5859 | -4.0805 | 0.0001 | 0.0002 | 1.1325 |
| COBL | 0.3129 | 5.5204 | 4.0804 | 0.0001 | 0.0002 | 1.1320 |
| SPAG5 | -0.2451 | 5.9097 | -4.0797 | 0.0001 | 0.0002 | 1.1294 |
| ZSWIM1 | -0.1233 | 5.6708 | -4.0790 | 0.0001 | 0.0002 | 1.1265 |
| TTPAL | 0.2035 | 5.8087 | 4.0778 | 0.0001 | 0.0002 | 1.1220 |
| CAND1 | -0.1059 | 5.9749 | -4.0764 | 0.0001 | 0.0002 | 1.1167 |
| LIAS | 0.1249 | 5.9819 | 4.0764 | 0.0001 | 0.0002 | 1.1164 |
| MMP17 | -0.7038 | 2.4750 | -4.0754 | 0.0001 | 0.0002 | 1.1128 |
| AOC3 | 0.2142 | 5.7157 | 4.0750 | 0.0001 | 0.0002 | 1.1112 |
| VHL | -0.1737 | 6.0056 | -4.0749 | 0.0001 | 0.0002 | 1.1106 |
| FOXG1 | -0.2907 | 0.1605 | -4.0739 | 0.0001 | 0.0002 | 1.1068 |
| SLC2A5 | -0.7898 | 3.1621 | -4.0728 | 0.0001 | 0.0002 | 1.1026 |
| POLR2C | 0.0982 | 6.6657 | 4.0726 | 0.0001 | 0.0002 | 1.1019 |
| CDH20 | -0.1871 | 0.4463 | -4.0714 | 0.0001 | 0.0002 | 1.0970 |
| RELB | -0.1664 | 6.2811 | -4.0712 | 0.0001 | 0.0002 | 1.0963 |
| SNTG1 | 0.6742 | 1.3868 | 4.0710 | 0.0001 | 0.0002 | 1.0957 |
| GC | 0.2158 | 7.9675 | 4.0708 | 0.0001 | 0.0002 | 1.0949 |
| SEMA4F | -0.5232 | 4.1354 | -4.0704 | 0.0001 | 0.0002 | 1.0935 |
| CTBS | 0.1742 | 5.7266 | 4.0701 | 0.0001 | 0.0002 | 1.0922 |
| FBLN2 | 0.5934 | 5.0861 | 4.0692 | 0.0001 | 0.0002 | 1.0887 |
| TMSB4Y | 0.7949 | 2.5882 | 4.0678 | 0.0001 | 0.0002 | 1.0834 |
| PAX8 | -0.4369 | 3.5514 | -4.0673 | 0.0001 | 0.0002 | 1.0814 |
| SEC31B | 0.3431 | 3.8805 | 4.0660 | 0.0001 | 0.0002 | 1.0763 |
| SERPINA4 | 0.4535 | 7.1393 | 4.0646 | 0.0001 | 0.0002 | 1.0710 |
| PDLIM4 | -0.5418 | 2.8527 | -4.0645 | 0.0001 | 0.0002 | 1.0704 |
| AVPR1A | 0.8457 | 3.8554 | 4.0629 | 0.0001 | 0.0002 | 1.0643 |
| NAIP | -0.2680 | 1.0767 | -4.0609 | 0.0001 | 0.0002 | 1.0564 |
| ANXA5 | -0.1256 | 7.1815 | -4.0590 | 0.0001 | 0.0002 | 1.0491 |
| RANBP9 | 0.0945 | 6.5290 | 4.0588 | 0.0001 | 0.0002 | 1.0483 |
| ASPSCR1 | 0.1960 | 6.1604 | 4.0582 | 0.0001 | 0.0002 | 1.0462 |
| SNCB | -0.3105 | 0.4795 | -4.0572 | 0.0001 | 0.0002 | 1.0422 |
| GNPAT | 0.0862 | 6.8953 | 4.0570 | 0.0001 | 0.0002 | 1.0415 |
| MAPKAP1 | 0.0720 | 6.4590 | 4.0559 | 0.0001 | 0.0002 | 1.0372 |
| AP3S2 | 0.1397 | 5.4901 | 4.0557 | 0.0001 | 0.0002 | 1.0366 |
| SMURF2 | -0.2152 | 5.0605 | -4.0541 | 0.0001 | 0.0002 | 1.0302 |
| GPX7 | -0.4271 | 5.3886 | -4.0540 | 0.0001 | 0.0002 | 1.0301 |
| MCHR1 | 0.6172 | 4.1324 | 4.0530 | 0.0001 | 0.0002 | 1.0261 |
| SLC16A5 | -0.3634 | 3.8199 | -4.0529 | 0.0001 | 0.0002 | 1.0256 |
| FAM3A | 0.1056 | 6.6086 | 4.0507 | 0.0001 | 0.0002 | 1.0174 |
| KCNV2 | -0.2124 | 0.4154 | -4.0505 | 0.0001 | 0.0002 | 1.0163 |
| EGR4 | -0.3240 | 0.6075 | -4.0497 | 0.0001 | 0.0002 | 1.0133 |
| KLK13 | -0.4880 | 0.6431 | -4.0469 | 0.0001 | 0.0002 | 1.0027 |
| NEK3 | 0.2940 | 5.1660 | 4.0448 | 0.0001 | 0.0002 | 0.9944 |
| DLD | 0.1034 | 6.6837 | 4.0443 | 0.0001 | 0.0002 | 0.9924 |

| MGA | -0.2045 | 4.7205 | -4.0421 | 0.0001 | 0.0002 | 0.9842 |
| --- | --- | --- | --- | --- | --- | --- |
| MGAM | -0.5938 | 1.3223 | -4.0418 | 0.0001 | 0.0002 | 0.9829 |
| FOXI1 | -0.2048 | 0.1592 | -4.0411 | 0.0001 | 0.0002 | 0.9804 |
| PDIA2 | -0.7843 | 2.4085 | -4.0399 | 0.0001 | 0.0002 | 0.9755 |
| HSPA6 | -0.4162 | 4.7594 | -4.0375 | 0.0001 | 0.0002 | 0.9665 |
| PIK3C2B | -0.2289 | 5.3461 | -4.0374 | 0.0001 | 0.0002 | 0.9659 |
| BCOR | -0.2089 | 5.5376 | -4.0362 | 0.0001 | 0.0002 | 0.9612 |
| PABPC1 | 0.0871 | 7.5941 | 4.0360 | 0.0001 | 0.0002 | 0.9605 |
| IL2RG | -0.3553 | 5.7754 | -4.0354 | 0.0001 | 0.0002 | 0.9583 |
| MAGEA8 | -0.6896 | 0.9885 | -4.0325 | 0.0001 | 0.0002 | 0.9471 |
| KCNAB3 | -0.3226 | 1.7686 | -4.0319 | 0.0001 | 0.0002 | 0.9447 |
| SCEL | -0.2533 | 0.2712 | -4.0316 | 0.0001 | 0.0002 | 0.9436 |
| GDI2 | 0.0591 | 7.2370 | 4.0287 | 0.0001 | 0.0002 | 0.9326 |
| NUPR1 | 0.1648 | 7.0747 | 4.0272 | 0.0001 | 0.0002 | 0.9267 |
| MECR | 0.1176 | 6.0380 | 4.0263 | 0.0001 | 0.0002 | 0.9236 |
| CKS2 | -0.1236 | 6.9074 | -4.0242 | 0.0001 | 0.0002 | 0.9155 |
| LDB1 | -0.1354 | 6.2284 | -4.0241 | 0.0001 | 0.0002 | 0.9148 |
| COPA | 0.0713 | 7.1079 | 4.0236 | 0.0001 | 0.0002 | 0.9131 |
| RABL3 | 0.0955 | 5.9075 | 4.0236 | 0.0001 | 0.0002 | 0.9130 |
| NDUFV2 | 0.1377 | 6.3327 | 4.0231 | 0.0001 | 0.0002 | 0.9113 |
| RMND5B | 0.0977 | 5.9796 | 4.0190 | 0.0001 | 0.0002 | 0.8956 |
| TMEM127 | 0.0583 | 6.7223 | 4.0170 | 0.0001 | 0.0002 | 0.8880 |
| CCKBR | -0.3240 | 0.3055 | -4.0149 | 0.0001 | 0.0002 | 0.8798 |
| SNX19 | 0.1707 | 6.1025 | 4.0142 | 0.0001 | 0.0002 | 0.8772 |
| GRM8 | -0.6287 | 1.3775 | -4.0142 | 0.0001 | 0.0002 | 0.8769 |
| MECP2 | -0.1404 | 5.0837 | -4.0139 | 0.0001 | 0.0002 | 0.8761 |
| CDKN2A | -0.4848 | 5.2701 | -4.0124 | 0.0001 | 0.0002 | 0.8702 |
| GDPD5 | -0.3100 | 4.4501 | -4.0122 | 0.0001 | 0.0002 | 0.8694 |
| CCL20 | -0.6158 | 6.0900 | -4.0121 | 0.0001 | 0.0002 | 0.8691 |
| ELOVL6 | 0.2896 | 6.0179 | 4.0110 | 0.0001 | 0.0002 | 0.8647 |
| LILRB4 | -0.4162 | 4.1998 | -4.0101 | 0.0001 | 0.0002 | 0.8612 |
| PSKH1 | 0.1199 | 6.2508 | 4.0088 | 0.0001 | 0.0002 | 0.8564 |
| SLC22A3 | 0.3787 | 6.1380 | 4.0085 | 0.0001 | 0.0002 | 0.8553 |
| TALDO1 | 0.0926 | 7.3123 | 4.0070 | 0.0001 | 0.0002 | 0.8495 |
| LONP1 | 0.0802 | 6.9729 | 4.0061 | 0.0001 | 0.0002 | 0.8461 |
| ATP5C1 | 0.0669 | 7.4256 | 4.0060 | 0.0001 | 0.0002 | 0.8459 |
| PLA2R1 | -0.4834 | 2.0517 | -4.0059 | 0.0001 | 0.0002 | 0.8453 |
| FANCA | -0.2879 | 4.4108 | -4.0056 | 0.0001 | 0.0002 | 0.8441 |
| STRADA | -0.2300 | 4.2732 | -4.0055 | 0.0001 | 0.0002 | 0.8437 |
| PLAC8 | -0.5381 | 3.0125 | -4.0047 | 0.0001 | 0.0002 | 0.8407 |
| CEP72 | -0.2681 | 4.9519 | -4.0040 | 0.0001 | 0.0002 | 0.8383 |
| HS2ST1 | -0.1107 | 5.9347 | -4.0037 | 0.0001 | 0.0002 | 0.8370 |
| NOP10 | 0.0732 | 7.4714 | 4.0037 | 0.0001 | 0.0002 | 0.8369 |
| RPS20 | 0.0879 | 7.7035 | 4.0033 | 0.0001 | 0.0002 | 0.8355 |
| BAMBI | -0.4177 | 6.3164 | -4.0017 | 0.0001 | 0.0002 | 0.8292 |
| CHAC1 | -0.4652 | 5.0473 | -4.0016 | 0.0001 | 0.0002 | 0.8288 |
| FPGS | 0.1033 | 6.6991 | 4.0014 | 0.0001 | 0.0002 | 0.8282 |
| S100P | -0.8438 | 5.1192 | -4.0006 | 0.0001 | 0.0002 | 0.8251 |
| NOD2 | -0.4316 | 3.1122 | -4.0002 | 0.0001 | 0.0002 | 0.8236 |
| EIF4A2 | 0.0767 | 7.0567 | 3.9994 | 0.0001 | 0.0002 | 0.8205 |
| AARS | 0.0773 | 7.0964 | 3.9991 | 0.0001 | 0.0002 | 0.8195 |
| BCL2L13 | 0.0905 | 6.2410 | 3.9976 | 0.0001 | 0.0002 | 0.8137 |
| MAPKBP1 | -0.1946 | 4.4643 | -3.9957 | 0.0001 | 0.0002 | 0.8064 |

| ADPGK | -0.0916 | 5.9880 | -3.9933 | 0.0001 | 0.0002 | 0.7973 |
| --- | --- | --- | --- | --- | --- | --- |
| ATF7 | -0.1302 | 5.5078 | -3.9932 | 0.0001 | 0.0002 | 0.7969 |
| UBA2 | -0.0772 | 6.5656 | -3.9927 | 0.0001 | 0.0002 | 0.7953 |
| RHBDF1 | -0.2359 | 5.3848 | -3.9917 | 0.0001 | 0.0002 | 0.7911 |
| TDO2 | 0.5013 | 6.4350 | 3.9913 | 0.0001 | 0.0002 | 0.7897 |
| GRTP1 | 0.1843 | 6.4671 | 3.9911 | 0.0001 | 0.0002 | 0.7891 |
| TRPC3 | -0.4796 | 0.8006 | -3.9911 | 0.0001 | 0.0002 | 0.7889 |
| CCK | -0.3838 | 0.4426 | -3.9911 | 0.0001 | 0.0002 | 0.7889 |
| MED4 | 0.0830 | 6.4993 | 3.9909 | 0.0001 | 0.0002 | 0.7881 |
| SGPL1 | 0.1134 | 6.3969 | 3.9908 | 0.0001 | 0.0002 | 0.7880 |
| CRYBA2 | -0.4810 | 0.7706 | -3.9899 | 0.0001 | 0.0002 | 0.7845 |
| ZNHIT6 | -0.1605 | 5.0423 | -3.9894 | 0.0001 | 0.0002 | 0.7824 |
| ZBTB7C | 0.5556 | 2.5313 | 3.9893 | 0.0001 | 0.0002 | 0.7822 |
| MAN2C1 | 0.1708 | 6.0638 | 3.9886 | 0.0001 | 0.0002 | 0.7794 |
| PDSS1 | -0.1683 | 5.6945 | -3.9885 | 0.0001 | 0.0002 | 0.7791 |
| PLGLB1 | 0.5445 | 3.8448 | 3.9875 | 0.0001 | 0.0002 | 0.7752 |
| PDE3A | -0.4644 | 3.7789 | -3.9848 | 0.0001 | 0.0002 | 0.7649 |
| NUBP2 | 0.0893 | 6.5709 | 3.9845 | 0.0001 | 0.0002 | 0.7640 |
| ATP13A3 | 0.1270 | 6.5086 | 3.9836 | 0.0001 | 0.0002 | 0.7605 |
| C11orf63 | -0.4156 | 2.0003 | -3.9835 | 0.0001 | 0.0002 | 0.7601 |
| CNPPD1 | 0.0652 | 6.8933 | 3.9832 | 0.0001 | 0.0002 | 0.7591 |
| ARID5B | -0.2532 | 5.1164 | -3.9830 | 0.0001 | 0.0002 | 0.7581 |
| ETAA1 | -0.1232 | 5.5795 | -3.9829 | 0.0001 | 0.0002 | 0.7580 |
| SYDE1 | -0.2917 | 5.0776 | -3.9820 | 0.0001 | 0.0002 | 0.7546 |
| CXorf57 | -0.4408 | 3.6717 | -3.9819 | 0.0001 | 0.0002 | 0.7542 |
| PAICS | 0.0843 | 6.7438 | 3.9811 | 0.0001 | 0.0002 | 0.7512 |
| CABYR | -0.7231 | 3.6780 | -3.9801 | 0.0001 | 0.0002 | 0.7472 |
| STARD7 | 0.0542 | 7.0068 | 3.9800 | 0.0001 | 0.0002 | 0.7469 |
| CALU | -0.0869 | 6.7996 | -3.9799 | 0.0001 | 0.0002 | 0.7463 |
| WDR4 | -0.1592 | 5.6767 | -3.9792 | 0.0001 | 0.0002 | 0.7438 |
| ZNF813 | -0.5955 | 3.5361 | -3.9791 | 0.0001 | 0.0002 | 0.7435 |
| ACTN3 | -0.2995 | 0.6438 | -3.9786 | 0.0001 | 0.0002 | 0.7416 |
| CD2AP | -0.1151 | 6.2035 | -3.9785 | 0.0001 | 0.0002 | 0.7413 |
| RPS11 | 0.0702 | 7.9125 | 3.9765 | 0.0001 | 0.0002 | 0.7336 |
| SELE | 0.6104 | 3.0667 | 3.9755 | 0.0001 | 0.0002 | 0.7299 |
| CD9 | 0.1823 | 6.5458 | 3.9753 | 0.0001 | 0.0002 | 0.7292 |
| DEPDC5 | 0.1891 | 5.3192 | 3.9753 | 0.0001 | 0.0002 | 0.7292 |
| GABRG3 | -0.3569 | 0.5180 | -3.9731 | 0.0001 | 0.0002 | 0.7208 |
| CANT1 | -0.0827 | 6.5010 | -3.9726 | 0.0001 | 0.0002 | 0.7189 |
| FAM189B | -0.1355 | 6.2155 | -3.9726 | 0.0001 | 0.0002 | 0.7187 |
| ZFP36 | 0.1149 | 7.1835 | 3.9722 | 0.0001 | 0.0002 | 0.7175 |
| GRM1 | -0.2161 | 0.3151 | -3.9715 | 0.0001 | 0.0002 | 0.7144 |
| GALNT14 | -0.4503 | 2.2963 | -3.9691 | 0.0001 | 0.0002 | 0.7055 |
| ABCB8 | 0.1312 | 6.0655 | 3.9682 | 0.0001 | 0.0002 | 0.7021 |
| PIAS3 | -0.2440 | 5.4191 | -3.9679 | 0.0001 | 0.0002 | 0.7011 |
| NDUFS6 | 0.0943 | 7.2832 | 3.9670 | 0.0001 | 0.0002 | 0.6976 |
| GYG1 | -0.1284 | 6.0577 | -3.9664 | 0.0001 | 0.0002 | 0.6955 |
| MAB21L1 | -0.3220 | 0.5885 | -3.9661 | 0.0001 | 0.0002 | 0.6940 |
| DBH | 0.5354 | 4.4520 | 3.9659 | 0.0001 | 0.0002 | 0.6934 |
| TMOD1 | 0.5121 | 4.9570 | 3.9655 | 0.0001 | 0.0002 | 0.6918 |
| ST5 | -0.2788 | 5.3166 | -3.9654 | 0.0001 | 0.0002 | 0.6915 |
| NFYA | -0.1288 | 6.1134 | -3.9652 | 0.0001 | 0.0002 | 0.6908 |
| PPAT | -0.1910 | 5.1496 | -3.9647 | 0.0001 | 0.0002 | 0.6890 |

| BCL7A | -0.1704 | 5.5150 | -3.9643 | 0.0001 | 0.0002 | 0.6875 |
| --- | --- | --- | --- | --- | --- | --- |
| FOXE1 | -0.4191 | 0.4833 | -3.9637 | 0.0001 | 0.0002 | 0.6852 |
| ASB12 | -0.4150 | 1.3292 | -3.9633 | 0.0001 | 0.0002 | 0.6835 |
| NHLH1 | -0.2980 | 0.9933 | -3.9619 | 0.0001 | 0.0003 | 0.6783 |
| KDM3A | -0.1700 | 5.3436 | -3.9616 | 0.0001 | 0.0003 | 0.6773 |
| RGS7 | -0.3498 | 0.4369 | -3.9612 | 0.0001 | 0.0003 | 0.6757 |
| COL4A6 | -0.5022 | 1.0106 | -3.9611 | 0.0001 | 0.0003 | 0.6751 |
| DDX52 | -0.1108 | 5.5136 | -3.9610 | 0.0001 | 0.0003 | 0.6750 |
| SIX5 | -0.2740 | 5.4243 | -3.9603 | 0.0001 | 0.0003 | 0.6722 |
| CHRFAM7A | -0.2005 | 0.3019 | -3.9596 | 0.0001 | 0.0003 | 0.6696 |
| MTMR7 | -0.4966 | 3.3325 | -3.9575 | 0.0001 | 0.0003 | 0.6615 |
| GRPR | 0.7347 | 2.1548 | 3.9574 | 0.0001 | 0.0003 | 0.6612 |
| PDHX | 0.0886 | 6.3208 | 3.9538 | 0.0001 | 0.0003 | 0.6478 |
| GSS | 0.0738 | 7.0175 | 3.9533 | 0.0001 | 0.0003 | 0.6459 |
| QTRT1 | 0.1099 | 6.5467 | 3.9507 | 0.0001 | 0.0003 | 0.6362 |
| ZNF157 | -0.2118 | 0.3214 | -3.9506 | 0.0001 | 0.0003 | 0.6356 |
| WDR74 | 0.0984 | 6.1986 | 3.9504 | 0.0001 | 0.0003 | 0.6349 |
| FZD9 | -0.6786 | 1.8599 | -3.9504 | 0.0001 | 0.0003 | 0.6349 |
| WBP4 | 0.1058 | 5.8835 | 3.9503 | 0.0001 | 0.0003 | 0.6344 |
| DNAJC6 | -0.4855 | 3.8223 | -3.9496 | 0.0001 | 0.0003 | 0.6321 |
| FLNA | -0.1762 | 6.5931 | -3.9488 | 0.0001 | 0.0003 | 0.6291 |
| PRDM13 | -0.2109 | 0.1324 | -3.9486 | 0.0001 | 0.0003 | 0.6283 |
| AMPD1 | -0.3592 | 0.5917 | -3.9438 | 0.0001 | 0.0003 | 0.6102 |
| RPAP2 | -0.1606 | 4.1815 | -3.9437 | 0.0001 | 0.0003 | 0.6098 |
| HMGB2 | -0.1259 | 6.6551 | -3.9430 | 0.0001 | 0.0003 | 0.6070 |
| SEZ6L2 | -0.7961 | 4.9383 | -3.9418 | 0.0001 | 0.0003 | 0.6028 |
| PANK3 | 0.1418 | 6.2476 | 3.9404 | 0.0001 | 0.0003 | 0.5974 |
| RPS28 | 0.0920 | 7.4880 | 3.9404 | 0.0001 | 0.0003 | 0.5973 |
| TUBD1 | -0.1569 | 5.1625 | -3.9396 | 0.0001 | 0.0003 | 0.5942 |
| TRIM23 | 0.1590 | 5.4248 | 3.9385 | 0.0001 | 0.0003 | 0.5901 |
| ZNF442 | 0.2173 | 3.3459 | 3.9365 | 0.0001 | 0.0003 | 0.5825 |
| MPP1 | 0.1929 | 6.1103 | 3.9351 | 0.0001 | 0.0003 | 0.5772 |
| BEGAIN | -0.5640 | 1.5848 | -3.9350 | 0.0001 | 0.0003 | 0.5770 |
| DPP6 | -0.1623 | 0.2880 | -3.9347 | 0.0001 | 0.0003 | 0.5760 |
| CLDN11 | -0.5685 | 3.0220 | -3.9341 | 0.0001 | 0.0003 | 0.5735 |
| RRAD | -0.5436 | 4.3477 | -3.9340 | 0.0001 | 0.0003 | 0.5734 |
| ITGB1BP1 | -0.1236 | 5.8748 | -3.9338 | 0.0001 | 0.0003 | 0.5725 |
| HHLA3 | 0.1942 | 6.1986 | 3.9332 | 0.0001 | 0.0003 | 0.5704 |
| KIF3A | -0.2238 | 4.3106 | -3.9330 | 0.0001 | 0.0003 | 0.5695 |
| PURG | -0.2142 | 0.3590 | -3.9317 | 0.0001 | 0.0003 | 0.5648 |
| BACE1 | 0.1336 | 6.0856 | 3.9314 | 0.0001 | 0.0003 | 0.5635 |
| OGFR | 0.0868 | 6.7349 | 3.9299 | 0.0001 | 0.0003 | 0.5578 |
| NACAD | -0.3860 | 1.6383 | -3.9293 | 0.0001 | 0.0003 | 0.5557 |
| SLC25A23 | 0.1257 | 6.5204 | 3.9289 | 0.0001 | 0.0003 | 0.5543 |
| RPL39L | -0.5552 | 5.3612 | -3.9285 | 0.0001 | 0.0003 | 0.5527 |
| MCTP2 | -0.6097 | 2.2056 | -3.9284 | 0.0001 | 0.0003 | 0.5521 |
| ZNF221 | -0.3725 | 2.5764 | -3.9272 | 0.0001 | 0.0003 | 0.5476 |
| YKT6 | -0.0751 | 6.6085 | -3.9267 | 0.0001 | 0.0003 | 0.5460 |
| GNG13 | -0.2764 | 0.4253 | -3.9264 | 0.0001 | 0.0003 | 0.5447 |
| RAB17 | 0.3019 | 6.5770 | 3.9255 | 0.0001 | 0.0003 | 0.5413 |
| IVNS1ABP | -0.1137 | 6.3725 | -3.9245 | 0.0001 | 0.0003 | 0.5377 |
| LEPROT | 0.0839 | 6.7318 | 3.9197 | 0.0001 | 0.0003 | 0.5196 |
| VCL | -0.1522 | 5.8632 | -3.9196 | 0.0001 | 0.0003 | 0.5194 |

| SIGLEC9 | -0.3732 | 3.8775 | -3.9182 | 0.0001 | 0.0003 | 0.5140 |
| --- | --- | --- | --- | --- | --- | --- |
| TMSB15A | -0.4914 | 2.3270 | -3.9150 | 0.0001 | 0.0003 | 0.5022 |
| RUNX2 | -0.4400 | 2.1801 | -3.9123 | 0.0001 | 0.0003 | 0.4919 |
| TMED9 | 0.0638 | 7.4668 | 3.9119 | 0.0001 | 0.0003 | 0.4907 |
| SYT5 | -0.4208 | 1.0036 | -3.9118 | 0.0001 | 0.0003 | 0.4903 |
| FGF8 | -0.3336 | 0.3942 | -3.9117 | 0.0001 | 0.0003 | 0.4897 |
| ZNF135 | -0.5496 | 2.4019 | -3.9116 | 0.0001 | 0.0003 | 0.4895 |
| DDX41 | 0.0724 | 6.7247 | 3.9110 | 0.0001 | 0.0003 | 0.4871 |
| RPL29 | 0.0769 | 7.6753 | 3.9105 | 0.0001 | 0.0003 | 0.4851 |
| SOX18 | 0.2062 | 5.9442 | 3.9098 | 0.0001 | 0.0003 | 0.4829 |
| PMM1 | 0.1298 | 6.4705 | 3.9093 | 0.0001 | 0.0003 | 0.4808 |
| ATP8B3 | -0.4156 | 3.2530 | -3.9088 | 0.0001 | 0.0003 | 0.4789 |
| GABRA2 | -0.5853 | 0.6638 | -3.9083 | 0.0001 | 0.0003 | 0.4771 |
| JMJD6 | -0.1323 | 5.8646 | -3.9079 | 0.0001 | 0.0003 | 0.4757 |
| PEX13 | 0.0889 | 6.1254 | 3.9079 | 0.0001 | 0.0003 | 0.4757 |
| PVRIG | -0.2909 | 0.9076 | -3.9078 | 0.0001 | 0.0003 | 0.4753 |
| BRS3 | -0.2307 | 0.2559 | -3.9077 | 0.0001 | 0.0003 | 0.4749 |
| LGALS8 | 0.1374 | 6.2489 | 3.9074 | 0.0001 | 0.0003 | 0.4738 |
| EPB41L2 | -0.2402 | 5.4494 | -3.9073 | 0.0001 | 0.0003 | 0.4734 |
| PLK3 | -0.2181 | 5.2061 | -3.9058 | 0.0001 | 0.0003 | 0.4678 |
| FGFR2 | -0.8461 | 5.0912 | -3.9053 | 0.0001 | 0.0003 | 0.4660 |
| EPPK1 | -0.7152 | 3.0696 | -3.9037 | 0.0001 | 0.0003 | 0.4599 |
| PDE6B | -0.4479 | 2.4282 | -3.9036 | 0.0001 | 0.0003 | 0.4594 |
| MSX2 | -0.4777 | 0.9035 | -3.9032 | 0.0001 | 0.0003 | 0.4581 |
| MAU2 | -0.1234 | 5.6400 | -3.9029 | 0.0001 | 0.0003 | 0.4571 |
| C5orf15 | 0.0669 | 6.9372 | 3.9026 | 0.0001 | 0.0003 | 0.4559 |
| HOOK2 | -0.1894 | 5.6137 | -3.9006 | 0.0001 | 0.0003 | 0.4484 |
| CCNJ | -0.4787 | 4.0355 | -3.9003 | 0.0001 | 0.0003 | 0.4473 |
| SLC22A8 | -0.4167 | 0.4802 | -3.8996 | 0.0001 | 0.0003 | 0.4446 |
| SPTBN1 | 0.0877 | 6.8939 | 3.8991 | 0.0001 | 0.0003 | 0.4428 |
| PCNT | -0.1605 | 5.2925 | -3.8989 | 0.0001 | 0.0003 | 0.4420 |
| NTAN1 | 0.1388 | 6.1390 | 3.8985 | 0.0001 | 0.0003 | 0.4407 |
| IL37 | -0.3616 | 0.7160 | -3.8979 | 0.0001 | 0.0003 | 0.4384 |
| INTS5 | 0.0768 | 6.3599 | 3.8974 | 0.0001 | 0.0003 | 0.4366 |
| RALY | -0.0776 | 6.6819 | -3.8965 | 0.0001 | 0.0003 | 0.4333 |
| IRAK4 | -0.1101 | 5.3467 | -3.8959 | 0.0001 | 0.0003 | 0.4310 |
| SYN1 | -0.5417 | 2.9615 | -3.8958 | 0.0001 | 0.0003 | 0.4304 |
| RPL34 | 0.0922 | 7.4086 | 3.8920 | 0.0001 | 0.0003 | 0.4165 |
| RNF146 | 0.1082 | 6.0603 | 3.8917 | 0.0001 | 0.0003 | 0.4154 |
| TLR2 | -0.4613 | 4.4894 | -3.8913 | 0.0001 | 0.0003 | 0.4139 |
| SLC6A20 | -0.4750 | 0.7533 | -3.8893 | 0.0001 | 0.0003 | 0.4064 |
| PLEKHH3 | -0.1640 | 5.8852 | -3.8882 | 0.0001 | 0.0003 | 0.4021 |
| NECAB3 | 0.1308 | 6.4407 | 3.8876 | 0.0001 | 0.0003 | 0.4001 |
| GNB1L | -0.2316 | 4.3833 | -3.8875 | 0.0001 | 0.0003 | 0.3996 |
| SERPINI1 | -0.3218 | 5.1875 | -3.8869 | 0.0001 | 0.0003 | 0.3976 |
| CD3D | -0.4082 | 5.4519 | -3.8868 | 0.0001 | 0.0003 | 0.3971 |
| TIMM8B | 0.0926 | 7.0042 | 3.8867 | 0.0001 | 0.0003 | 0.3966 |
| ZFAND6 | 0.0654 | 6.5849 | 3.8856 | 0.0001 | 0.0003 | 0.3926 |
| CR2 | -0.5275 | 0.9874 | -3.8852 | 0.0001 | 0.0003 | 0.3910 |
| VDR | -0.5052 | 3.8855 | -3.8842 | 0.0001 | 0.0003 | 0.3875 |
| ATP12A | -0.4010 | 0.3657 | -3.8836 | 0.0001 | 0.0003 | 0.3853 |
| HCAR3 | -0.5388 | 1.4447 | -3.8834 | 0.0001 | 0.0003 | 0.3845 |
| CRYZ | 0.1348 | 7.0231 | 3.8815 | 0.0001 | 0.0003 | 0.3775 |

| SLC22A5 | -0.1901 | 4.6503 | -3.8815 | 0.0001 | 0.0003 | 0.3774 |
| --- | --- | --- | --- | --- | --- | --- |
| DAP | 0.0734 | 7.2414 | 3.8815 | 0.0001 | 0.0003 | 0.3773 |
| C1orf56 | 0.1493 | 6.1010 | 3.8813 | 0.0001 | 0.0003 | 0.3767 |
| VPS4B | 0.0971 | 6.2567 | 3.8802 | 0.0001 | 0.0003 | 0.3727 |
| SOX5 | 0.3920 | 4.3425 | 3.8790 | 0.0001 | 0.0003 | 0.3680 |
| GJB4 | -0.3616 | 0.5224 | -3.8784 | 0.0001 | 0.0003 | 0.3661 |
| HMGCR | 0.1571 | 6.4712 | 3.8780 | 0.0001 | 0.0003 | 0.3646 |
| FGFR3 | -0.2266 | 6.4910 | -3.8773 | 0.0001 | 0.0003 | 0.3619 |
| MAGEC3 | -0.3906 | 0.4110 | -3.8771 | 0.0001 | 0.0003 | 0.3610 |
| PSMC5 | 0.0782 | 7.0397 | 3.8766 | 0.0001 | 0.0003 | 0.3592 |
| KRT2 | -0.1912 | 0.2169 | -3.8764 | 0.0001 | 0.0003 | 0.3585 |
| BMPR1A | -0.1676 | 4.8331 | -3.8762 | 0.0001 | 0.0003 | 0.3577 |
| TBC1D2B | 0.1980 | 5.7757 | 3.8759 | 0.0001 | 0.0003 | 0.3566 |
| PTPN18 | 0.0732 | 6.3769 | 3.8758 | 0.0001 | 0.0003 | 0.3564 |
| PHTF1 | -0.2099 | 4.7539 | -3.8749 | 0.0001 | 0.0003 | 0.3532 |
| ABCD3 | 0.1037 | 6.5981 | 3.8748 | 0.0001 | 0.0003 | 0.3526 |
| FHOD1 | -0.2370 | 5.0286 | -3.8740 | 0.0001 | 0.0003 | 0.3496 |
| MRPS16 | 0.0676 | 7.0031 | 3.8726 | 0.0001 | 0.0003 | 0.3447 |
| TRPC5 | 0.5041 | 1.2919 | 3.8718 | 0.0001 | 0.0003 | 0.3417 |
| CCL25 | 0.8505 | 4.0256 | 3.8715 | 0.0001 | 0.0003 | 0.3403 |
| C21orf59 | -0.1085 | 5.8685 | -3.8688 | 0.0001 | 0.0004 | 0.3303 |
| PRAMEF10 | 0.9228 | 2.4370 | 3.8683 | 0.0001 | 0.0004 | 0.3284 |
| CCDC88A | -0.2518 | 4.9367 | -3.8670 | 0.0001 | 0.0004 | 0.3237 |
| GFPT2 | -0.6525 | 2.8053 | -3.8659 | 0.0001 | 0.0004 | 0.3196 |
| GFAP | -0.5049 | 1.5242 | -3.8658 | 0.0001 | 0.0004 | 0.3192 |
| GRIK2 | -0.4809 | 0.8751 | -3.8630 | 0.0001 | 0.0004 | 0.3092 |
| RALGDS | -0.1430 | 5.6804 | -3.8625 | 0.0001 | 0.0004 | 0.3071 |
| TRIB1 | 0.1297 | 6.8018 | 3.8624 | 0.0001 | 0.0004 | 0.3070 |
| LGALS3BP | -0.1968 | 7.2769 | -3.8616 | 0.0001 | 0.0004 | 0.3038 |
| SLC5A6 | 0.1534 | 6.6163 | 3.8608 | 0.0001 | 0.0004 | 0.3011 |
| CDKN2B | -0.2969 | 5.1095 | -3.8606 | 0.0001 | 0.0004 | 0.3000 |
| ZNF814 | -0.2572 | 4.0787 | -3.8603 | 0.0001 | 0.0004 | 0.2992 |
| RARS2 | 0.0923 | 6.4413 | 3.8588 | 0.0001 | 0.0004 | 0.2937 |
| LAMB2 | 0.1247 | 6.9042 | 3.8584 | 0.0001 | 0.0004 | 0.2922 |
| NDUFC1 | 0.0908 | 6.8403 | 3.8569 | 0.0001 | 0.0004 | 0.2866 |
| USP21 | -0.1250 | 5.9183 | -3.8569 | 0.0001 | 0.0004 | 0.2865 |
| TDP2 | 0.0816 | 6.7397 | 3.8564 | 0.0001 | 0.0004 | 0.2848 |
| TMEM120B | -0.1556 | 4.8533 | -3.8564 | 0.0001 | 0.0004 | 0.2846 |
| TNFAIP2 | -0.1852 | 6.3630 | -3.8554 | 0.0001 | 0.0004 | 0.2811 |
| WISP1 | -0.5175 | 3.1003 | -3.8551 | 0.0001 | 0.0004 | 0.2800 |
| CYP2E1 | 0.6955 | 6.6507 | 3.8538 | 0.0001 | 0.0004 | 0.2749 |
| PTPN14 | -0.3915 | 3.7779 | -3.8537 | 0.0001 | 0.0004 | 0.2747 |
| PLIN1 | 0.5015 | 4.7026 | 3.8529 | 0.0001 | 0.0004 | 0.2716 |
| RAE1 | -0.0972 | 5.9155 | -3.8524 | 0.0001 | 0.0004 | 0.2701 |
| TAGLN3 | -0.2803 | 0.4451 | -3.8523 | 0.0001 | 0.0004 | 0.2695 |
| CA11 | -0.3182 | 4.7210 | -3.8521 | 0.0001 | 0.0004 | 0.2689 |
| C9orf3 | 0.1275 | 5.7560 | 3.8519 | 0.0001 | 0.0004 | 0.2682 |
| RALGPS2 | 0.1704 | 6.1138 | 3.8519 | 0.0001 | 0.0004 | 0.2681 |
| SLC27A6 | -0.1750 | 0.1611 | -3.8515 | 0.0001 | 0.0004 | 0.2668 |
| ZNF337 | -0.3946 | 3.2128 | -3.8500 | 0.0001 | 0.0004 | 0.2611 |
| AFF2 | -0.4417 | 0.7886 | -3.8499 | 0.0001 | 0.0004 | 0.2606 |
| DHX16 | 0.0725 | 6.5355 | 3.8492 | 0.0001 | 0.0004 | 0.2583 |
| HTR2C | -0.3599 | 0.3673 | -3.8490 | 0.0001 | 0.0004 | 0.2576 |

| IL10 | -0.4279 | 1.8925 | -3.8486 | 0.0001 | 0.0004 | 0.2560 |
| --- | --- | --- | --- | --- | --- | --- |
| GPBP1L1 | 0.0750 | 6.5666 | 3.8486 | 0.0001 | 0.0004 | 0.2558 |
| NKX6-1 | -0.2001 | 0.1065 | -3.8484 | 0.0001 | 0.0004 | 0.2552 |
| THYN1 | 0.0933 | 6.5624 | 3.8479 | 0.0001 | 0.0004 | 0.2536 |
| CCDC94 | 0.0894 | 6.6158 | 3.8471 | 0.0001 | 0.0004 | 0.2506 |
| LY6G6C | -0.3285 | 0.5714 | -3.8468 | 0.0001 | 0.0004 | 0.2494 |
| UBA6 | -0.1470 | 5.3243 | -3.8466 | 0.0001 | 0.0004 | 0.2485 |
| GDAP2 | -0.1603 | 4.6692 | -3.8462 | 0.0001 | 0.0004 | 0.2472 |
| PIGG | -0.1268 | 5.4889 | -3.8451 | 0.0001 | 0.0004 | 0.2431 |
| GAS6 | 0.2449 | 6.2669 | 3.8447 | 0.0001 | 0.0004 | 0.2415 |
| FZD10 | -0.4939 | 1.3488 | -3.8440 | 0.0001 | 0.0004 | 0.2389 |
| MSH6 | -0.1150 | 5.8958 | -3.8436 | 0.0001 | 0.0004 | 0.2375 |
| ADAMTS20 | -0.2750 | 0.3312 | -3.8417 | 0.0001 | 0.0004 | 0.2307 |
| BTN1A1 | -0.2424 | 0.3797 | -3.8411 | 0.0001 | 0.0004 | 0.2285 |
| GPAA1 | 0.0922 | 7.3034 | 3.8408 | 0.0001 | 0.0004 | 0.2273 |
| UBXN8 | 0.1722 | 5.9233 | 3.8398 | 0.0001 | 0.0004 | 0.2235 |
| NOVA2 | 0.2611 | 3.4247 | 3.8397 | 0.0001 | 0.0004 | 0.2234 |
| RPL21 | 0.0909 | 7.2871 | 3.8390 | 0.0001 | 0.0004 | 0.2207 |
| MRPL42 | -0.0943 | 5.4397 | -3.8380 | 0.0001 | 0.0004 | 0.2169 |
| TLX2 | -0.4061 | 0.6769 | -3.8378 | 0.0001 | 0.0004 | 0.2164 |
| PLEKHF1 | 0.2742 | 5.9775 | 3.8370 | 0.0001 | 0.0004 | 0.2134 |
| NAALAD2 | -0.4737 | 2.1444 | -3.8367 | 0.0001 | 0.0004 | 0.2123 |
| RAD51C | -0.1525 | 5.2219 | -3.8364 | 0.0001 | 0.0004 | 0.2110 |
| FTSJ1 | -0.0817 | 6.2636 | -3.8361 | 0.0001 | 0.0004 | 0.2102 |
| CKAP2 | -0.2219 | 5.4146 | -3.8354 | 0.0001 | 0.0004 | 0.2074 |
| GNAT1 | 0.4800 | 2.6644 | 3.8334 | 0.0001 | 0.0004 | 0.2002 |
| KHDC1L | -0.4248 | 0.4242 | -3.8329 | 0.0001 | 0.0004 | 0.1983 |
| DAB2 | -0.2591 | 5.8648 | -3.8320 | 0.0001 | 0.0004 | 0.1952 |
| PSMD8 | 0.0611 | 7.1424 | 3.8320 | 0.0001 | 0.0004 | 0.1952 |
| TNFRSF4 | -0.2725 | 5.2003 | -3.8308 | 0.0001 | 0.0004 | 0.1906 |
| GFM1 | 0.1026 | 6.1523 | 3.8307 | 0.0001 | 0.0004 | 0.1901 |
| FCGR3B | -0.5881 | 2.3519 | -3.8300 | 0.0002 | 0.0004 | 0.1877 |
| DRAP1 | 0.0741 | 7.1689 | 3.8286 | 0.0002 | 0.0004 | 0.1826 |
| MNT | -0.1533 | 4.9255 | -3.8272 | 0.0002 | 0.0004 | 0.1774 |
| 44076.0000 | -0.0734 | 6.7823 | -3.8267 | 0.0002 | 0.0004 | 0.1758 |
| KPNA3 | 0.1084 | 6.2581 | 3.8259 | 0.0002 | 0.0004 | 0.1726 |
| ZNF780B | -0.2521 | 4.1711 | -3.8249 | 0.0002 | 0.0004 | 0.1691 |
| APOL5 | 0.7453 | 2.5534 | 3.8245 | 0.0002 | 0.0004 | 0.1675 |
| RNF103 | 0.1061 | 6.3390 | 3.8243 | 0.0002 | 0.0004 | 0.1667 |
| CEACAM5 | -0.5053 | 0.6374 | -3.8242 | 0.0002 | 0.0004 | 0.1666 |
| SAMM50 | 0.1130 | 6.4067 | 3.8242 | 0.0002 | 0.0004 | 0.1664 |
| XAGE1B | -0.2842 | 0.3471 | -3.8239 | 0.0002 | 0.0004 | 0.1654 |
| TFAP2B | -0.1890 | 0.1205 | -3.8232 | 0.0002 | 0.0004 | 0.1628 |
| RAB5C | 0.0572 | 6.9971 | 3.8230 | 0.0002 | 0.0004 | 0.1621 |
| KRT31 | -0.1620 | 0.1263 | -3.8227 | 0.0002 | 0.0004 | 0.1611 |
| MEA1 | 0.0683 | 7.2899 | 3.8204 | 0.0002 | 0.0004 | 0.1526 |
| MAP2K6 | -0.4364 | 4.2081 | -3.8203 | 0.0002 | 0.0004 | 0.1524 |
| LRP6 | 0.1598 | 5.9169 | 3.8202 | 0.0002 | 0.0004 | 0.1519 |
| BAG6 | 0.0547 | 7.2130 | 3.8198 | 0.0002 | 0.0004 | 0.1504 |
| KDELC1 | -0.2049 | 5.7215 | -3.8192 | 0.0002 | 0.0004 | 0.1483 |
| RIOK3 | 0.0811 | 6.5536 | 3.8180 | 0.0002 | 0.0004 | 0.1439 |
| SNX1 | 0.0868 | 6.3686 | 3.8180 | 0.0002 | 0.0004 | 0.1439 |
| CPNE7 | -0.6327 | 3.4386 | -3.8177 | 0.0002 | 0.0004 | 0.1426 |

| WIPF1 | -0.2694 | 5.1214 | -3.8171 | 0.0002 | 0.0004 | 0.1405 |
| --- | --- | --- | --- | --- | --- | --- |
| OCA2 | -0.7417 | 2.1070 | -3.8164 | 0.0002 | 0.0004 | 0.1380 |
| OPHN1 | -0.3951 | 2.7311 | -3.8164 | 0.0002 | 0.0004 | 0.1380 |
| ESRRA | 0.0849 | 6.9141 | 3.8151 | 0.0002 | 0.0004 | 0.1333 |
| FOXD2 | -0.4491 | 3.6599 | -3.8146 | 0.0002 | 0.0004 | 0.1316 |
| HCRTR2 | -0.1536 | 0.1611 | -3.8137 | 0.0002 | 0.0004 | 0.1280 |
| SERPINA7 | 0.5811 | 6.7829 | 3.8136 | 0.0002 | 0.0004 | 0.1279 |
| NAA11 | -0.7479 | 1.0794 | -3.8123 | 0.0002 | 0.0004 | 0.1229 |
| PPP4R1 | -0.1015 | 6.0159 | -3.8119 | 0.0002 | 0.0004 | 0.1215 |
| GTF2IRD2B | 0.2270 | 4.1406 | 3.8118 | 0.0002 | 0.0004 | 0.1211 |
| STK4 | -0.1168 | 5.5943 | -3.8113 | 0.0002 | 0.0004 | 0.1195 |
| SLC22A18 | 0.2609 | 6.6633 | 3.8112 | 0.0002 | 0.0004 | 0.1191 |
| NDUFB2 | 0.0988 | 6.8188 | 3.8104 | 0.0002 | 0.0004 | 0.1163 |
| CPZ | -0.4029 | 0.7096 | -3.8101 | 0.0002 | 0.0004 | 0.1150 |
| PMPCA | 0.1048 | 6.5772 | 3.8098 | 0.0002 | 0.0004 | 0.1141 |
| ZNF225 | -0.2137 | 3.6703 | -3.8092 | 0.0002 | 0.0004 | 0.1117 |
| NIPAL3 | -0.1798 | 4.7188 | -3.8090 | 0.0002 | 0.0004 | 0.1111 |
| XRCC1 | -0.0795 | 6.3273 | -3.8088 | 0.0002 | 0.0004 | 0.1104 |
| ITGAE | -0.2557 | 4.6623 | -3.8088 | 0.0002 | 0.0004 | 0.1102 |
| NOTCH1 | -0.2125 | 5.4916 | -3.8087 | 0.0002 | 0.0004 | 0.1100 |
| FRAT1 | 0.1737 | 6.0260 | 3.8084 | 0.0002 | 0.0004 | 0.1089 |
| IL22 | -0.1032 | 0.0869 | -3.8069 | 0.0002 | 0.0004 | 0.1033 |
| ACTL8 | -0.7815 | 1.5039 | -3.8067 | 0.0002 | 0.0004 | 0.1027 |
| FTH1 | 0.0646 | 7.7317 | 3.8055 | 0.0002 | 0.0004 | 0.0981 |
| B3GALT4 | -0.3753 | 4.5785 | -3.8046 | 0.0002 | 0.0004 | 0.0952 |
| OAZ3 | -0.2612 | 3.5632 | -3.8040 | 0.0002 | 0.0004 | 0.0929 |
| ZNF180 | -0.1665 | 4.8217 | -3.8035 | 0.0002 | 0.0004 | 0.0911 |
| NUDCD3 | 0.0759 | 6.2937 | 3.8026 | 0.0002 | 0.0004 | 0.0877 |
| ATOH1 | -0.2734 | 0.1982 | -3.8025 | 0.0002 | 0.0004 | 0.0873 |
| AKAP6 | 0.3402 | 2.6517 | 3.8024 | 0.0002 | 0.0004 | 0.0870 |
| DENND5B | 0.1868 | 5.3500 | 3.8018 | 0.0002 | 0.0004 | 0.0848 |
| PSG3 | -0.1392 | 0.0864 | -3.8002 | 0.0002 | 0.0004 | 0.0789 |
| SNAP29 | 0.0761 | 6.5164 | 3.7997 | 0.0002 | 0.0004 | 0.0772 |
| KPNB1 | -0.0571 | 6.7964 | -3.7996 | 0.0002 | 0.0004 | 0.0769 |
| SST | -0.4902 | 0.4688 | -3.7995 | 0.0002 | 0.0004 | 0.0763 |
| HMGN1 | -0.0947 | 6.6581 | -3.7982 | 0.0002 | 0.0005 | 0.0718 |
| COQ6 | 0.1287 | 5.3165 | 3.7982 | 0.0002 | 0.0005 | 0.0716 |
| PIAS2 | -0.1413 | 4.5209 | -3.7974 | 0.0002 | 0.0005 | 0.0688 |
| AGFG1 | -0.0977 | 6.0558 | -3.7949 | 0.0002 | 0.0005 | 0.0599 |
| SRCAP | -0.1042 | 5.9663 | -3.7940 | 0.0002 | 0.0005 | 0.0566 |
| SPI1 | -0.2181 | 5.9439 | -3.7939 | 0.0002 | 0.0005 | 0.0563 |
| PPP1R11 | 0.0642 | 6.9692 | 3.7937 | 0.0002 | 0.0005 | 0.0554 |
| MID1IP1 | -0.1265 | 6.7784 | -3.7933 | 0.0002 | 0.0005 | 0.0539 |
| TRRAP | -0.1489 | 5.6755 | -3.7923 | 0.0002 | 0.0005 | 0.0504 |
| KIF3B | 0.0780 | 6.4984 | 3.7922 | 0.0002 | 0.0005 | 0.0501 |
| PADI2 | -0.5584 | 2.5059 | -3.7915 | 0.0002 | 0.0005 | 0.0474 |
| APEX1 | 0.0527 | 7.2715 | 3.7914 | 0.0002 | 0.0005 | 0.0472 |
| C1GALT1C1 | 0.0880 | 6.6667 | 3.7910 | 0.0002 | 0.0005 | 0.0456 |
| COX16 | 0.0874 | 6.4539 | 3.7907 | 0.0002 | 0.0005 | 0.0447 |
| GLRX2 | 0.1249 | 6.4004 | 3.7905 | 0.0002 | 0.0005 | 0.0439 |
| CDC34 | 0.0824 | 7.1184 | 3.7883 | 0.0002 | 0.0005 | 0.0360 |
| CROT | 0.1872 | 6.1410 | 3.7883 | 0.0002 | 0.0005 | 0.0360 |
| GAD2 | -0.2014 | 0.0945 | -3.7874 | 0.0002 | 0.0005 | 0.0325 |

| DBNDD1 | 0.2980 | 5.9389 | 3.7874 | 0.0002 | 0.0005 | 0.0324 |
| --- | --- | --- | --- | --- | --- | --- |
| TBXAS1 | -0.2931 | 4.5340 | -3.7873 | 0.0002 | 0.0005 | 0.0323 |
| PTPRS | -0.6451 | 3.7720 | -3.7857 | 0.0002 | 0.0005 | 0.0263 |
| C1orf216 | -0.1779 | 5.5752 | -3.7856 | 0.0002 | 0.0005 | 0.0262 |
| RALGAPB | -0.1144 | 5.5860 | -3.7848 | 0.0002 | 0.0005 | 0.0231 |
| ASAP3 | 0.2434 | 5.7428 | 3.7835 | 0.0002 | 0.0005 | 0.0183 |
| LGALS7 | -0.3490 | 0.3960 | -3.7832 | 0.0002 | 0.0005 | 0.0175 |
| CD68 | -0.3581 | 4.0384 | -3.7827 | 0.0002 | 0.0005 | 0.0155 |
| GOLGA3 | -0.0819 | 6.1194 | -3.7823 | 0.0002 | 0.0005 | 0.0141 |
| CEACAM21 | -0.4171 | 3.3731 | -3.7821 | 0.0002 | 0.0005 | 0.0133 |
| IMMT | 0.0596 | 6.8496 | 3.7809 | 0.0002 | 0.0005 | 0.0091 |
| API5 | 0.0587 | 6.6260 | 3.7809 | 0.0002 | 0.0005 | 0.0089 |
| HOXC11 | -0.3109 | 0.2946 | -3.7795 | 0.0002 | 0.0005 | 0.0039 |
| HLA-DQB2 | -0.5316 | 4.9115 | -3.7785 | 0.0002 | 0.0005 | 0.0002 |
| ATP5G3 | 0.0854 | 7.0488 | 3.7766 | 0.0002 | 0.0005 | -0.0064 |
| POLB | 0.1820 | 5.8867 | 3.7761 | 0.0002 | 0.0005 | -0.0084 |
| ZMYND10 | -0.3650 | 2.6755 | -3.7758 | 0.0002 | 0.0005 | -0.0095 |
| MAF | 0.1411 | 6.1429 | 3.7757 | 0.0002 | 0.0005 | -0.0098 |
| TMEM30B | 0.3864 | 5.5202 | 3.7755 | 0.0002 | 0.0005 | -0.0107 |
| PIGA | -0.1680 | 5.3149 | -3.7750 | 0.0002 | 0.0005 | -0.0123 |
| TLE1 | 0.1067 | 6.7612 | 3.7749 | 0.0002 | 0.0005 | -0.0125 |
| TSG101 | 0.0554 | 6.7011 | 3.7721 | 0.0002 | 0.0005 | -0.0228 |
| ACOX3 | 0.1271 | 6.1473 | 3.7705 | 0.0002 | 0.0005 | -0.0286 |
| PAK3 | -0.5561 | 1.2100 | -3.7693 | 0.0002 | 0.0005 | -0.0327 |
| HEY1 | -0.2408 | 5.0838 | -3.7684 | 0.0002 | 0.0005 | -0.0361 |
| FDFT1 | 0.1141 | 6.7989 | 3.7679 | 0.0002 | 0.0005 | -0.0379 |
| SLC35E2 | -0.3053 | 2.7351 | -3.7676 | 0.0002 | 0.0005 | -0.0389 |
| UBA52 | 0.0744 | 7.4441 | 3.7666 | 0.0002 | 0.0005 | -0.0428 |
| SPACA1 | 0.3488 | 0.5457 | 3.7663 | 0.0002 | 0.0005 | -0.0437 |
| SPRY4 | -0.2087 | 5.6563 | -3.7640 | 0.0002 | 0.0005 | -0.0519 |
| TNC | -0.4749 | 4.6434 | -3.7624 | 0.0002 | 0.0005 | -0.0577 |
| ANXA4 | -0.1316 | 6.6727 | -3.7619 | 0.0002 | 0.0005 | -0.0594 |
| MBNL2 | 0.1656 | 6.3968 | 3.7619 | 0.0002 | 0.0005 | -0.0596 |
| TUBA3C | -0.8447 | 1.3949 | -3.7616 | 0.0002 | 0.0005 | -0.0606 |
| THEG | -0.0806 | 0.0688 | -3.7604 | 0.0002 | 0.0005 | -0.0649 |
| WDR41 | -0.1235 | 5.5465 | -3.7604 | 0.0002 | 0.0005 | -0.0650 |
| DNAL4 | -0.1067 | 5.9315 | -3.7600 | 0.0002 | 0.0005 | -0.0665 |
| ARHGEF16 | -0.5653 | 4.9048 | -3.7598 | 0.0002 | 0.0005 | -0.0672 |
| OPRM1 | -0.0247 | 0.0180 | -3.7596 | 0.0002 | 0.0005 | -0.0680 |
| COMP | -0.8103 | 2.7058 | -3.7595 | 0.0002 | 0.0005 | -0.0682 |
| PTK7 | -0.5412 | 4.1343 | -3.7588 | 0.0002 | 0.0005 | -0.0709 |
| COPS6 | 0.0717 | 7.0551 | 3.7587 | 0.0002 | 0.0005 | -0.0709 |
| SPINK1 | -0.7293 | 6.3927 | -3.7584 | 0.0002 | 0.0005 | -0.0723 |
| PRRC2A | -0.0779 | 6.7426 | -3.7580 | 0.0002 | 0.0005 | -0.0736 |
| ZC3H13 | 0.1836 | 5.7698 | 3.7569 | 0.0002 | 0.0005 | -0.0777 |
| CST4 | -0.5615 | 0.6799 | -3.7563 | 0.0002 | 0.0005 | -0.0797 |
| GPR25 | -0.3346 | 0.7275 | -3.7561 | 0.0002 | 0.0005 | -0.0804 |
| KCNK5 | -0.4915 | 5.0111 | -3.7554 | 0.0002 | 0.0005 | -0.0828 |
| SUPT4H1 | 0.0609 | 7.0148 | 3.7547 | 0.0002 | 0.0005 | -0.0854 |
| ZNF330 | 0.1055 | 6.3575 | 3.7541 | 0.0002 | 0.0005 | -0.0875 |
| SLC25A32 | 0.1150 | 6.1342 | 3.7541 | 0.0002 | 0.0005 | -0.0875 |
| GALR3 | 0.5939 | 2.3364 | 3.7541 | 0.0002 | 0.0005 | -0.0878 |
| GNAI3 | -0.1005 | 5.4437 | -3.7531 | 0.0002 | 0.0005 | -0.0912 |

| NTHL1 | 0.1659 | 6.5540 | 3.7525 | 0.0002 | 0.0005 | -0.0934 |
| --- | --- | --- | --- | --- | --- | --- |
| RAF1 | 0.0706 | 6.6705 | 3.7516 | 0.0002 | 0.0005 | -0.0967 |
| LIMS1 | -0.1383 | 5.8816 | -3.7512 | 0.0002 | 0.0005 | -0.0980 |
| TBCE | 0.0917 | 6.6350 | 3.7508 | 0.0002 | 0.0005 | -0.0993 |
| ADAM2 | -0.1412 | 0.0870 | -3.7507 | 0.0002 | 0.0005 | -0.0999 |
| CLIP3 | -0.3194 | 4.5143 | -3.7504 | 0.0002 | 0.0005 | -0.1011 |
| B4GALT6 | -0.3359 | 3.9170 | -3.7501 | 0.0002 | 0.0005 | -0.1019 |
| RFXAP | -0.2378 | 4.2439 | -3.7500 | 0.0002 | 0.0005 | -0.1023 |
| UROD | 0.0848 | 7.0232 | 3.7498 | 0.0002 | 0.0005 | -0.1032 |
| RRM1 | -0.0959 | 6.4410 | -3.7497 | 0.0002 | 0.0005 | -0.1035 |
| ACP2 | 0.0784 | 6.9379 | 3.7495 | 0.0002 | 0.0005 | -0.1043 |
| MAGEA4 | -0.5263 | 0.5040 | -3.7494 | 0.0002 | 0.0005 | -0.1046 |
| PRMT3 | -0.1719 | 5.2176 | -3.7487 | 0.0002 | 0.0005 | -0.1068 |
| NEK9 | 0.0935 | 6.1327 | 3.7468 | 0.0002 | 0.0005 | -0.1139 |
| HOXB1 | -0.0859 | 0.0602 | -3.7444 | 0.0002 | 0.0005 | -0.1223 |
| JARID2 | -0.1552 | 5.3736 | -3.7432 | 0.0002 | 0.0005 | -0.1268 |
| TMPRSS11E | -0.2539 | 0.3348 | -3.7427 | 0.0002 | 0.0005 | -0.1286 |
| APOBR | -0.2369 | 4.9711 | -3.7399 | 0.0002 | 0.0006 | -0.1385 |
| INSIG2 | 0.1475 | 6.3833 | 3.7393 | 0.0002 | 0.0006 | -0.1406 |
| ASNA1 | 0.0685 | 7.0367 | 3.7391 | 0.0002 | 0.0006 | -0.1416 |
| RPL13A | 0.0679 | 7.8826 | 3.7370 | 0.0002 | 0.0006 | -0.1488 |
| ABCA3 | -0.3914 | 4.2879 | -3.7370 | 0.0002 | 0.0006 | -0.1489 |
| MOG | -0.1004 | 0.1090 | -3.7352 | 0.0002 | 0.0006 | -0.1552 |
| CST2 | -0.7557 | 2.4980 | -3.7342 | 0.0002 | 0.0006 | -0.1589 |
| DEFB126 | -0.3296 | 0.2935 | -3.7341 | 0.0002 | 0.0006 | -0.1594 |
| RAC3 | 0.2330 | 6.3165 | 3.7337 | 0.0002 | 0.0006 | -0.1609 |
| ZNF143 | -0.1151 | 5.5081 | -3.7335 | 0.0002 | 0.0006 | -0.1616 |
| ZNF780A | -0.1527 | 4.7330 | -3.7331 | 0.0002 | 0.0006 | -0.1629 |
| LAPTM4B | -0.1944 | 6.9023 | -3.7319 | 0.0002 | 0.0006 | -0.1671 |
| IFNG | -0.5651 | 1.8328 | -3.7317 | 0.0002 | 0.0006 | -0.1677 |
| TUSC3 | -0.5174 | 4.3584 | -3.7317 | 0.0002 | 0.0006 | -0.1679 |
| NOX1 | -0.3048 | 3.3638 | -3.7313 | 0.0002 | 0.0006 | -0.1695 |
| PPIE | -0.1156 | 5.8058 | -3.7310 | 0.0002 | 0.0006 | -0.1703 |
| HOXB5 | -0.3566 | 3.3292 | -3.7300 | 0.0002 | 0.0006 | -0.1741 |
| CEP85 | -0.2368 | 5.1075 | -3.7297 | 0.0002 | 0.0006 | -0.1749 |
| SEMA4C | -0.1460 | 5.9254 | -3.7289 | 0.0002 | 0.0006 | -0.1781 |
| HMGB1 | 0.0623 | 6.7411 | 3.7285 | 0.0002 | 0.0006 | -0.1792 |
| MAP3K11 | 0.0760 | 6.6564 | 3.7284 | 0.0002 | 0.0006 | -0.1796 |
| KPNA1 | 0.0852 | 6.0046 | 3.7279 | 0.0002 | 0.0006 | -0.1816 |
| CXXC4 | -0.5016 | 2.9797 | -3.7269 | 0.0002 | 0.0006 | -0.1852 |
| SLC17A3 | 0.4671 | 5.2036 | 3.7268 | 0.0002 | 0.0006 | -0.1854 |
| IGLL1 | -0.3885 | 0.7002 | -3.7259 | 0.0002 | 0.0006 | -0.1885 |
| C14orf2 | 0.1023 | 6.7963 | 3.7257 | 0.0002 | 0.0006 | -0.1894 |
| ZNF669 | -0.3156 | 4.7568 | -3.7255 | 0.0002 | 0.0006 | -0.1899 |
| DGKA | -0.3291 | 3.8248 | -3.7255 | 0.0002 | 0.0006 | -0.1900 |
| NAP1L3 | -0.5401 | 2.4724 | -3.7241 | 0.0002 | 0.0006 | -0.1951 |
| CCR3 | -0.5386 | 1.0911 | -3.7238 | 0.0002 | 0.0006 | -0.1961 |
| SMAP1 | -0.1601 | 5.2438 | -3.7236 | 0.0002 | 0.0006 | -0.1969 |
| RXRG | 0.7144 | 2.4316 | 3.7234 | 0.0002 | 0.0006 | -0.1976 |
| PAM | -0.2806 | 5.2535 | -3.7232 | 0.0002 | 0.0006 | -0.1983 |
| ZNF710 | -0.2670 | 5.2610 | -3.7227 | 0.0002 | 0.0006 | -0.1999 |
| GMNN | -0.1459 | 6.6176 | -3.7227 | 0.0002 | 0.0006 | -0.2000 |
| MRPL22 | 0.1056 | 6.0938 | 3.7226 | 0.0002 | 0.0006 | -0.2006 |

| NAV2 | -0.2970 | 5.3562 | -3.7224 | 0.0002 | 0.0006 | -0.2010 |
| --- | --- | --- | --- | --- | --- | --- |
| PEX7 | 0.1317 | 5.8449 | 3.7224 | 0.0002 | 0.0006 | -0.2011 |
| RREB1 | 0.1316 | 5.8103 | 3.7220 | 0.0002 | 0.0006 | -0.2025 |
| RPLP1 | 0.0695 | 7.7673 | 3.7211 | 0.0002 | 0.0006 | -0.2056 |
| FAM69A | 0.1727 | 6.0683 | 3.7193 | 0.0002 | 0.0006 | -0.2122 |
| GPN2 | -0.0838 | 5.9821 | -3.7184 | 0.0002 | 0.0006 | -0.2155 |
| FRMD8 | -0.0822 | 6.2634 | -3.7181 | 0.0002 | 0.0006 | -0.2165 |
| CKAP5 | -0.0849 | 6.3173 | -3.7177 | 0.0002 | 0.0006 | -0.2177 |
| MYH14 | 0.1752 | 6.6401 | 3.7176 | 0.0002 | 0.0006 | -0.2181 |
| MPP5 | 0.1438 | 5.5464 | 3.7176 | 0.0002 | 0.0006 | -0.2184 |
| CRYBB2 | -0.4709 | 2.0210 | -3.7171 | 0.0002 | 0.0006 | -0.2198 |
| PDZD3 | -0.4005 | 0.9346 | -3.7171 | 0.0002 | 0.0006 | -0.2199 |
| FUS | -0.0747 | 6.6885 | -3.7163 | 0.0002 | 0.0006 | -0.2227 |
| HRK | -0.2758 | 0.3240 | -3.7161 | 0.0002 | 0.0006 | -0.2237 |
| AVP | -0.5051 | 0.8162 | -3.7151 | 0.0002 | 0.0006 | -0.2273 |
| DHX15 | -0.0860 | 6.2508 | -3.7136 | 0.0002 | 0.0006 | -0.2326 |
| RAB23 | -0.3954 | 3.7191 | -3.7131 | 0.0002 | 0.0006 | -0.2344 |
| ABL1 | -0.1168 | 6.1872 | -3.7129 | 0.0002 | 0.0006 | -0.2350 |
| MZB1 | -0.6942 | 3.6593 | -3.7126 | 0.0002 | 0.0006 | -0.2361 |
| FNDC3B | -0.1352 | 6.0919 | -3.7111 | 0.0002 | 0.0006 | -0.2413 |
| SPRR3 | -0.4687 | 0.5777 | -3.7107 | 0.0002 | 0.0006 | -0.2428 |
| EPB41L5 | 0.1543 | 5.8300 | 3.7106 | 0.0002 | 0.0006 | -0.2433 |
| RET | -0.5628 | 1.7677 | -3.7102 | 0.0002 | 0.0006 | -0.2447 |
| GUCY2C | -0.7442 | 2.7150 | -3.7101 | 0.0002 | 0.0006 | -0.2449 |
| RARG | -0.2525 | 4.6758 | -3.7096 | 0.0002 | 0.0006 | -0.2468 |
| ARF5 | 0.0612 | 7.2477 | 3.7088 | 0.0002 | 0.0006 | -0.2497 |
| FGF4 | -0.3248 | 0.2359 | -3.7086 | 0.0002 | 0.0006 | -0.2503 |
| PPP1CC | -0.0645 | 6.5963 | -3.7059 | 0.0002 | 0.0006 | -0.2597 |
| CCNA1 | -0.2473 | 0.3912 | -3.7045 | 0.0002 | 0.0006 | -0.2646 |
| PRODH2 | 0.4744 | 6.6139 | 3.7025 | 0.0002 | 0.0006 | -0.2718 |
| NSMAF | -0.2103 | 5.1893 | -3.7005 | 0.0002 | 0.0006 | -0.2788 |
| PPIL6 | -0.3020 | 2.1075 | -3.7001 | 0.0002 | 0.0006 | -0.2803 |
| INTS9 | -0.1637 | 5.0848 | -3.6989 | 0.0002 | 0.0006 | -0.2847 |
| PHF20 | -0.0974 | 5.6195 | -3.6982 | 0.0002 | 0.0006 | -0.2871 |
| NOL9 | -0.1204 | 5.3003 | -3.6975 | 0.0003 | 0.0006 | -0.2896 |
| CLEC2D | -0.3527 | 3.7158 | -3.6969 | 0.0003 | 0.0006 | -0.2917 |
| TMEM39B | -0.0888 | 5.8718 | -3.6961 | 0.0003 | 0.0006 | -0.2946 |
| PSMG2 | 0.0627 | 6.5640 | 3.6958 | 0.0003 | 0.0006 | -0.2956 |
| SIK3 | 0.1324 | 5.6887 | 3.6942 | 0.0003 | 0.0006 | -0.3012 |
| HIST1H2AC | 0.1548 | 7.0795 | 3.6938 | 0.0003 | 0.0006 | -0.3027 |
| WNT4 | -0.7574 | 3.5139 | -3.6937 | 0.0003 | 0.0006 | -0.3030 |
| NDUFS3 | 0.0796 | 6.8573 | 3.6936 | 0.0003 | 0.0006 | -0.3033 |
| ABCB7 | 0.0958 | 6.1961 | 3.6935 | 0.0003 | 0.0006 | -0.3037 |
| DYNC2H1 | -0.3725 | 2.0660 | -3.6927 | 0.0003 | 0.0007 | -0.3066 |
| PIGZ | -0.2653 | 4.9254 | -3.6923 | 0.0003 | 0.0007 | -0.3080 |
| LHX2 | -0.4912 | 2.7699 | -3.6918 | 0.0003 | 0.0007 | -0.3098 |
| AK2 | 0.0750 | 6.7999 | 3.6918 | 0.0003 | 0.0007 | -0.3099 |
| FAM149A | 0.3208 | 5.4416 | 3.6899 | 0.0003 | 0.0007 | -0.3165 |
| GRM5 | -0.1949 | 0.1788 | -3.6891 | 0.0003 | 0.0007 | -0.3195 |
| RHEB | 0.0696 | 6.7346 | 3.6890 | 0.0003 | 0.0007 | -0.3197 |
| XPNPEP1 | -0.0872 | 6.0145 | -3.6886 | 0.0003 | 0.0007 | -0.3212 |
| PRTN3 | -0.4922 | 0.9879 | -3.6883 | 0.0003 | 0.0007 | -0.3221 |
| BSN | 0.4077 | 2.7495 | 3.6878 | 0.0003 | 0.0007 | -0.3239 |

| SMARCAL1 | -0.0921 | 5.7654 | -3.6875 | 0.0003 | 0.0007 | -0.3251 |
| --- | --- | --- | --- | --- | --- | --- |
| SGMS1 | 0.1350 | 5.8802 | 3.6875 | 0.0003 | 0.0007 | -0.3252 |
| TOX | -0.6064 | 3.0850 | -3.6868 | 0.0003 | 0.0007 | -0.3274 |
| COQ4 | 0.1006 | 6.4650 | 3.6868 | 0.0003 | 0.0007 | -0.3275 |
| LAD1 | -0.4999 | 6.2853 | -3.6866 | 0.0003 | 0.0007 | -0.3281 |
| S100A8 | -0.4284 | 4.9074 | -3.6856 | 0.0003 | 0.0007 | -0.3316 |
| CTRL | -0.1570 | 0.3729 | -3.6843 | 0.0003 | 0.0007 | -0.3365 |
| SELP | 0.5379 | 3.2332 | 3.6839 | 0.0003 | 0.0007 | -0.3378 |
| USP2 | 0.3648 | 5.0034 | 3.6794 | 0.0003 | 0.0007 | -0.3537 |
| CLPS | -0.4035 | 0.3498 | -3.6793 | 0.0003 | 0.0007 | -0.3539 |
| GPR22 | -0.1113 | 0.2098 | -3.6793 | 0.0003 | 0.0007 | -0.3539 |
| CBX3 | -0.0544 | 6.8688 | -3.6793 | 0.0003 | 0.0007 | -0.3541 |
| SERTAD3 | -0.1087 | 6.2484 | -3.6788 | 0.0003 | 0.0007 | -0.3557 |
| TACR3 | -0.1539 | 0.1014 | -3.6768 | 0.0003 | 0.0007 | -0.3628 |
| RAC2 | -0.2588 | 5.9068 | -3.6767 | 0.0003 | 0.0007 | -0.3633 |
| PDE4DIP | 0.2236 | 5.4007 | 3.6745 | 0.0003 | 0.0007 | -0.3709 |
| RPRD1A | -0.1030 | 5.9176 | -3.6740 | 0.0003 | 0.0007 | -0.3727 |
| MAMLD1 | -0.4446 | 4.3355 | -3.6728 | 0.0003 | 0.0007 | -0.3769 |
| ATXN7L1 | 0.2341 | 4.4050 | 3.6724 | 0.0003 | 0.0007 | -0.3783 |
| TMEM144 | -0.4608 | 3.2378 | -3.6716 | 0.0003 | 0.0007 | -0.3813 |
| ZNF551 | -0.3409 | 3.7909 | -3.6711 | 0.0003 | 0.0007 | -0.3828 |
| CDHR2 | -0.7968 | 4.4163 | -3.6707 | 0.0003 | 0.0007 | -0.3843 |
| KBTBD2 | -0.0690 | 6.1977 | -3.6699 | 0.0003 | 0.0007 | -0.3869 |
| CACNA1E | -0.3790 | 0.6672 | -3.6699 | 0.0003 | 0.0007 | -0.3872 |
| CDKN2C | -0.2119 | 5.9098 | -3.6690 | 0.0003 | 0.0007 | -0.3902 |
| IL1R1 | 0.1514 | 6.4678 | 3.6687 | 0.0003 | 0.0007 | -0.3915 |
| FOLH1 | 0.3697 | 5.2413 | 3.6680 | 0.0003 | 0.0007 | -0.3938 |
| CLK3 | -0.0928 | 5.0778 | -3.6665 | 0.0003 | 0.0007 | -0.3991 |
| EFNA4 | -0.1494 | 6.2762 | -3.6656 | 0.0003 | 0.0007 | -0.4021 |
| GRP | -0.5328 | 0.8875 | -3.6651 | 0.0003 | 0.0007 | -0.4040 |
| FERMT1 | -0.7049 | 3.4521 | -3.6645 | 0.0003 | 0.0007 | -0.4062 |
| LGALS3 | -0.2201 | 6.6114 | -3.6644 | 0.0003 | 0.0007 | -0.4066 |
| NFE2L2 | 0.1065 | 6.6039 | 3.6640 | 0.0003 | 0.0007 | -0.4079 |
| SLC5A5 | -0.3653 | 0.7465 | -3.6639 | 0.0003 | 0.0007 | -0.4081 |
| PRNP | -0.2248 | 6.5070 | -3.6639 | 0.0003 | 0.0007 | -0.4082 |
| TNFRSF11B | -0.6114 | 4.5389 | -3.6628 | 0.0003 | 0.0007 | -0.4122 |
| MEGF9 | 0.1466 | 6.2635 | 3.6626 | 0.0003 | 0.0007 | -0.4129 |
| SIDT1 | -0.3538 | 2.2619 | -3.6624 | 0.0003 | 0.0007 | -0.4135 |
| S1PR2 | -0.3006 | 5.6344 | -3.6622 | 0.0003 | 0.0007 | -0.4143 |
| DNAAF2 | 0.1218 | 5.9277 | 3.6621 | 0.0003 | 0.0007 | -0.4146 |
| TUBA8 | -0.3210 | 1.4739 | -3.6618 | 0.0003 | 0.0007 | -0.4156 |
| GPS2 | -0.2137 | 4.3224 | -3.6611 | 0.0003 | 0.0007 | -0.4179 |
| DKK4 | 0.9334 | 3.0430 | 3.6609 | 0.0003 | 0.0007 | -0.4189 |
| MAN2B2 | 0.1178 | 6.2496 | 3.6605 | 0.0003 | 0.0007 | -0.4203 |
| LIN28A | -0.2841 | 0.2687 | -3.6599 | 0.0003 | 0.0007 | -0.4222 |
| GIN1 | 0.1194 | 4.6832 | 3.6590 | 0.0003 | 0.0007 | -0.4254 |
| ZNF821 | -0.2535 | 3.9058 | -3.6589 | 0.0003 | 0.0007 | -0.4259 |
| KIAA0319L | -0.0794 | 6.3864 | -3.6586 | 0.0003 | 0.0007 | -0.4270 |
| MAP7 | 0.3030 | 5.7794 | 3.6583 | 0.0003 | 0.0007 | -0.4279 |
| GFRA3 | -0.5574 | 1.1055 | -3.6581 | 0.0003 | 0.0007 | -0.4287 |
| RPL11 | 0.0639 | 7.7166 | 3.6580 | 0.0003 | 0.0007 | -0.4288 |
| LPAR1 | -0.5965 | 2.6830 | -3.6576 | 0.0003 | 0.0007 | -0.4305 |
| IL36RN | -0.1376 | 0.1207 | -3.6567 | 0.0003 | 0.0007 | -0.4336 |

| CHRNA9 | -0.2110 | 0.1915 | -3.6565 | 0.0003 | 0.0007 | -0.4341 |
| --- | --- | --- | --- | --- | --- | --- |
| HBB | 0.3048 | 6.1287 | 3.6557 | 0.0003 | 0.0007 | -0.4369 |
| FIP1L1 | -0.1102 | 5.6453 | -3.6546 | 0.0003 | 0.0007 | -0.4410 |
| MXD1 | -0.2001 | 5.0606 | -3.6543 | 0.0003 | 0.0007 | -0.4417 |
| TAS2R1 | -0.1938 | 0.1519 | -3.6528 | 0.0003 | 0.0007 | -0.4472 |
| EIF5A | 0.0697 | 7.4497 | 3.6525 | 0.0003 | 0.0007 | -0.4483 |
| ZNF345 | -0.2206 | 3.6550 | -3.6522 | 0.0003 | 0.0007 | -0.4494 |
| RBBP9 | 0.1393 | 6.2672 | 3.6521 | 0.0003 | 0.0007 | -0.4497 |
| ME2 | -0.1761 | 5.3726 | -3.6512 | 0.0003 | 0.0008 | -0.4526 |
| STMN3 | -0.3906 | 4.8117 | -3.6498 | 0.0003 | 0.0008 | -0.4577 |
| ECM1 | -0.3225 | 5.0676 | -3.6496 | 0.0003 | 0.0008 | -0.4582 |
| CCDC33 | -0.1333 | 0.1217 | -3.6495 | 0.0003 | 0.0008 | -0.4586 |
| BCL11B | -0.4389 | 2.5761 | -3.6490 | 0.0003 | 0.0008 | -0.4604 |
| SETD4 | -0.1397 | 4.8179 | -3.6490 | 0.0003 | 0.0008 | -0.4606 |
| KRT75 | -0.2448 | 0.2492 | -3.6488 | 0.0003 | 0.0008 | -0.4612 |
| SEMA6C | 0.2472 | 5.5310 | 3.6486 | 0.0003 | 0.0008 | -0.4618 |
| HBP1 | 0.1769 | 5.9881 | 3.6485 | 0.0003 | 0.0008 | -0.4623 |
| VRK2 | -0.1952 | 5.2651 | -3.6477 | 0.0003 | 0.0008 | -0.4650 |
| PARP16 | 0.1086 | 5.8712 | 3.6466 | 0.0003 | 0.0008 | -0.4688 |
| KIFAP3 | 0.1307 | 6.1006 | 3.6449 | 0.0003 | 0.0008 | -0.4749 |
| ANKRD11 | -0.1281 | 5.5288 | -3.6445 | 0.0003 | 0.0008 | -0.4761 |
| AUTS2 | 0.4173 | 5.0027 | 3.6444 | 0.0003 | 0.0008 | -0.4766 |
| RAB35 | -0.0656 | 6.3400 | -3.6441 | 0.0003 | 0.0008 | -0.4776 |
| SIGLEC5 | -0.3262 | 1.4656 | -3.6441 | 0.0003 | 0.0008 | -0.4777 |
| ARL4D | 0.1840 | 6.6830 | 3.6437 | 0.0003 | 0.0008 | -0.4792 |
| GIT2 | -0.1877 | 4.5811 | -3.6431 | 0.0003 | 0.0008 | -0.4811 |
| PSG4 | -0.2112 | 0.2442 | -3.6431 | 0.0003 | 0.0008 | -0.4812 |
| MRC1 | 0.3494 | 5.3708 | 3.6414 | 0.0003 | 0.0008 | -0.4872 |
| CUTC | 0.1022 | 6.3985 | 3.6412 | 0.0003 | 0.0008 | -0.4878 |
| PDHA1 | 0.0714 | 6.8242 | 3.6401 | 0.0003 | 0.0008 | -0.4915 |
| VN1R1 | -0.4328 | 2.7913 | -3.6399 | 0.0003 | 0.0008 | -0.4924 |
| KCTD3 | 0.0872 | 6.8631 | 3.6392 | 0.0003 | 0.0008 | -0.4948 |
| C10orf95 | -0.3214 | 2.0567 | -3.6376 | 0.0003 | 0.0008 | -0.5002 |
| HRH2 | -0.4521 | 1.4492 | -3.6368 | 0.0003 | 0.0008 | -0.5030 |
| LTB | -0.3765 | 5.6798 | -3.6367 | 0.0003 | 0.0008 | -0.5035 |
| KLK12 | -0.1739 | 0.1566 | -3.6355 | 0.0003 | 0.0008 | -0.5077 |
| RPN2 | 0.0487 | 7.5488 | 3.6352 | 0.0003 | 0.0008 | -0.5088 |
| U2SURP | -0.1072 | 5.9710 | -3.6350 | 0.0003 | 0.0008 | -0.5095 |
| STXBP5L | -0.1487 | 0.1502 | -3.6336 | 0.0003 | 0.0008 | -0.5143 |
| DYRK3 | -0.3133 | 4.2817 | -3.6323 | 0.0003 | 0.0008 | -0.5189 |
| PSPH | -0.1614 | 6.2524 | -3.6322 | 0.0003 | 0.0008 | -0.5191 |
| CD300C | -0.4088 | 3.5364 | -3.6317 | 0.0003 | 0.0008 | -0.5208 |
| COL5A3 | 0.3270 | 5.9830 | 3.6316 | 0.0003 | 0.0008 | -0.5212 |
| NAT6 | 0.1462 | 5.8834 | 3.6314 | 0.0003 | 0.0008 | -0.5221 |
| TGFB3 | -0.3032 | 5.0474 | -3.6313 | 0.0003 | 0.0008 | -0.5221 |
| MT1E | 0.4009 | 6.6458 | 3.6308 | 0.0003 | 0.0008 | -0.5240 |
| SLC16A7 | -0.5291 | 4.1530 | -3.6294 | 0.0003 | 0.0008 | -0.5287 |
| SUGP2 | -0.1451 | 5.7312 | -3.6292 | 0.0003 | 0.0008 | -0.5296 |
| YOD1 | -0.1784 | 5.1325 | -3.6291 | 0.0003 | 0.0008 | -0.5299 |
| SFN | -0.5557 | 5.9440 | -3.6288 | 0.0003 | 0.0008 | -0.5311 |
| ZNF492 | -0.4332 | 0.9275 | -3.6287 | 0.0003 | 0.0008 | -0.5315 |
| CAP2 | 0.2203 | 6.1898 | 3.6284 | 0.0003 | 0.0008 | -0.5326 |
| POLR2L | 0.0794 | 7.4160 | 3.6272 | 0.0003 | 0.0008 | -0.5364 |

| OLAH | -0.3224 | 0.7136 | -3.6269 | 0.0003 | 0.0008 | -0.5376 |
| --- | --- | --- | --- | --- | --- | --- |
| CAPN3 | 0.3959 | 2.6395 | 3.6261 | 0.0003 | 0.0008 | -0.5405 |
| THOC5 | -0.1155 | 5.6554 | -3.6251 | 0.0003 | 0.0008 | -0.5440 |
| SLFN12 | -0.3901 | 3.3287 | -3.6242 | 0.0003 | 0.0008 | -0.5469 |
| USP49 | -0.2131 | 3.6824 | -3.6241 | 0.0003 | 0.0008 | -0.5474 |
| GBA | 0.0971 | 6.8517 | 3.6228 | 0.0003 | 0.0008 | -0.5520 |
| GTF2H5 | 0.1283 | 5.9427 | 3.6227 | 0.0003 | 0.0008 | -0.5523 |
| RBM15 | -0.1054 | 5.3492 | -3.6223 | 0.0003 | 0.0008 | -0.5537 |
| ART4 | 0.4804 | 5.2995 | 3.6220 | 0.0003 | 0.0008 | -0.5547 |
| FOXE3 | -0.2531 | 0.5993 | -3.6214 | 0.0003 | 0.0008 | -0.5567 |
| P2RX3 | 0.4858 | 2.1156 | 3.6213 | 0.0003 | 0.0008 | -0.5572 |
| SPIN2A | -0.1751 | 0.4481 | -3.6210 | 0.0003 | 0.0008 | -0.5581 |
| MPHOSPH10 | 0.0776 | 6.2469 | 3.6205 | 0.0003 | 0.0008 | -0.5598 |
| TRA2B | -0.0568 | 6.1673 | -3.6201 | 0.0003 | 0.0008 | -0.5612 |
| NLRP3 | -0.3730 | 2.8811 | -3.6192 | 0.0003 | 0.0008 | -0.5645 |
| HMGXB4 | -0.1664 | 5.3716 | -3.6187 | 0.0003 | 0.0008 | -0.5660 |
| UQCRFS1 | 0.0850 | 6.8741 | 3.6182 | 0.0003 | 0.0008 | -0.5677 |
| ATXN3 | 0.1486 | 3.8653 | 3.6167 | 0.0003 | 0.0008 | -0.5730 |
| FUT1 | -0.3441 | 4.0314 | -3.6164 | 0.0003 | 0.0008 | -0.5741 |
| TIA1 | -0.1159 | 6.0459 | -3.6164 | 0.0003 | 0.0008 | -0.5743 |
| CD59 | 0.0759 | 7.1841 | 3.6163 | 0.0003 | 0.0008 | -0.5746 |
| GTF2H1 | -0.0809 | 5.8498 | -3.6157 | 0.0003 | 0.0008 | -0.5765 |
| AMHR2 | -0.3224 | 0.5940 | -3.6156 | 0.0003 | 0.0008 | -0.5769 |
| MYOD1 | -0.2074 | 0.1408 | -3.6149 | 0.0003 | 0.0008 | -0.5794 |
| MARK1 | -0.4301 | 2.3574 | -3.6143 | 0.0003 | 0.0008 | -0.5815 |
| BRIP1 | -0.3568 | 3.8997 | -3.6137 | 0.0003 | 0.0009 | -0.5836 |
| NHLH2 | -0.2321 | 0.1639 | -3.6135 | 0.0003 | 0.0009 | -0.5842 |
| RAB40AL | -0.2030 | 0.5396 | -3.6132 | 0.0003 | 0.0009 | -0.5853 |
| NENF | 0.0947 | 7.2336 | 3.6123 | 0.0003 | 0.0009 | -0.5883 |
| FCAR | -0.3254 | 0.8818 | -3.6119 | 0.0003 | 0.0009 | -0.5897 |
| AMMECR1 | -0.1868 | 4.8312 | -3.6116 | 0.0003 | 0.0009 | -0.5907 |
| UBE2L6 | 0.1219 | 7.0021 | 3.6114 | 0.0003 | 0.0009 | -0.5915 |
| DONSON | -0.1441 | 5.8279 | -3.6103 | 0.0003 | 0.0009 | -0.5954 |
| RPS17 | 0.0729 | 7.5514 | 3.6102 | 0.0003 | 0.0009 | -0.5955 |
| GLOD4 | 0.0876 | 6.3073 | 3.6100 | 0.0003 | 0.0009 | -0.5962 |
| TMEM87A | -0.0941 | 5.9955 | -3.6086 | 0.0003 | 0.0009 | -0.6013 |
| IL15RA | -0.2083 | 5.8909 | -3.6075 | 0.0004 | 0.0009 | -0.6050 |
| WDR61 | 0.0772 | 6.0128 | 3.6075 | 0.0004 | 0.0009 | -0.6051 |
| NETO2 | -0.3801 | 3.2594 | -3.6073 | 0.0004 | 0.0009 | -0.6057 |
| SCFD1 | 0.0724 | 6.1378 | 3.6071 | 0.0004 | 0.0009 | -0.6063 |
| TSC22D1 | 0.1380 | 6.5915 | 3.6069 | 0.0004 | 0.0009 | -0.6071 |
| ANK1 | -0.3656 | 1.0159 | -3.6064 | 0.0004 | 0.0009 | -0.6088 |
| RPL27 | 0.0674 | 7.6747 | 3.6062 | 0.0004 | 0.0009 | -0.6096 |
| MNDA | -0.3708 | 4.5038 | -3.6057 | 0.0004 | 0.0009 | -0.6112 |
| SLC2A10 | 0.2921 | 6.0970 | 3.6047 | 0.0004 | 0.0009 | -0.6147 |
| SKIL | -0.1682 | 5.7334 | -3.6045 | 0.0004 | 0.0009 | -0.6153 |
| SSTR1 | 0.6220 | 4.5082 | 3.6041 | 0.0004 | 0.0009 | -0.6169 |
| IL3 | 0.2902 | 0.5146 | 3.6030 | 0.0004 | 0.0009 | -0.6207 |
| AMDHD2 | 0.1317 | 5.9816 | 3.6013 | 0.0004 | 0.0009 | -0.6264 |
| POSTN | -0.6385 | 4.5302 | -3.6009 | 0.0004 | 0.0009 | -0.6279 |
| PARP8 | -0.3109 | 4.6309 | -3.6007 | 0.0004 | 0.0009 | -0.6284 |
| ADAM29 | -0.0507 | 0.0462 | -3.5995 | 0.0004 | 0.0009 | -0.6325 |
| ZMIZ2 | -0.0799 | 6.3576 | -3.5991 | 0.0004 | 0.0009 | -0.6339 |

| RIOK2 | 0.0891 | 5.7119 | 3.5987 | 0.0004 | 0.0009 | -0.6356 |
| --- | --- | --- | --- | --- | --- | --- |
| NPC1 | -0.1339 | 5.7760 | -3.5983 | 0.0004 | 0.0009 | -0.6368 |
| HSPA4 | 0.0689 | 6.9314 | 3.5980 | 0.0004 | 0.0009 | -0.6377 |
| PLA2G12A | 0.1134 | 6.0971 | 3.5974 | 0.0004 | 0.0009 | -0.6400 |
| SAMD14 | -0.2882 | 2.5510 | -3.5969 | 0.0004 | 0.0009 | -0.6415 |
| RAB28 | -0.1039 | 5.7485 | -3.5960 | 0.0004 | 0.0009 | -0.6449 |
| SAR1A | 0.0585 | 6.6366 | 3.5949 | 0.0004 | 0.0009 | -0.6484 |
| DNAAF1 | 0.5658 | 1.6380 | 3.5947 | 0.0004 | 0.0009 | -0.6492 |
| GBP1 | 0.1971 | 6.2823 | 3.5942 | 0.0004 | 0.0009 | -0.6509 |
| KDELR3 | -0.2604 | 6.3059 | -3.5927 | 0.0004 | 0.0009 | -0.6561 |
| GCA | -0.2449 | 5.3907 | -3.5918 | 0.0004 | 0.0009 | -0.6591 |
| TWSG1 | -0.2545 | 5.2818 | -3.5913 | 0.0004 | 0.0009 | -0.6609 |
| GIF | -0.0915 | 0.0696 | -3.5898 | 0.0004 | 0.0009 | -0.6661 |
| HOXD10 | -0.5560 | 1.5409 | -3.5892 | 0.0004 | 0.0009 | -0.6683 |
| NDST4 | -0.2537 | 0.2240 | -3.5881 | 0.0004 | 0.0009 | -0.6721 |
| DDX4 | -0.2449 | 0.3853 | -3.5873 | 0.0004 | 0.0009 | -0.6748 |
| CD86 | -0.3225 | 4.6634 | -3.5871 | 0.0004 | 0.0009 | -0.6754 |
| GNAO1 | 0.6480 | 3.3433 | 3.5855 | 0.0004 | 0.0009 | -0.6809 |
| TOR1AIP1 | 0.1374 | 6.0079 | 3.5851 | 0.0004 | 0.0009 | -0.6825 |
| ELAVL3 | -0.1560 | 0.3966 | -3.5839 | 0.0004 | 0.0009 | -0.6866 |
| ATP10D | -0.2985 | 4.4237 | -3.5829 | 0.0004 | 0.0009 | -0.6900 |
| AKTIP | 0.1483 | 5.6900 | 3.5822 | 0.0004 | 0.0009 | -0.6923 |
| DEFA6 | -0.3723 | 0.4321 | -3.5821 | 0.0004 | 0.0009 | -0.6928 |
| ZNF506 | -0.3369 | 3.9423 | -3.5818 | 0.0004 | 0.0009 | -0.6936 |
| **CTAG2** | -1.0070 | 2.2151 | -3.5812 | 0.0004 | 0.0009 | -0.6958 |
| VPS8 | -0.1073 | 5.1214 | -3.5806 | 0.0004 | 0.0010 | -0.6977 |
| IGF1 | 0.5479 | 3.8090 | 3.5803 | 0.0004 | 0.0010 | -0.6989 |
| EFS | -0.4700 | 2.9419 | -3.5789 | 0.0004 | 0.0010 | -0.7035 |
| TGDS | 0.1667 | 5.9135 | 3.5785 | 0.0004 | 0.0010 | -0.7051 |
| CTNNA1 | 0.0537 | 7.0028 | 3.5780 | 0.0004 | 0.0010 | -0.7068 |
| TNNC2 | 0.3898 | 3.3643 | 3.5764 | 0.0004 | 0.0010 | -0.7123 |
| HIST1H2AD | -0.5551 | 3.8390 | -3.5756 | 0.0004 | 0.0010 | -0.7151 |
| CACNA1H | -0.6376 | 4.4020 | -3.5753 | 0.0004 | 0.0010 | -0.7161 |
| GNAI1 | 0.1814 | 6.1478 | 3.5748 | 0.0004 | 0.0010 | -0.7179 |
| EFNB2 | 0.2147 | 5.5827 | 3.5745 | 0.0004 | 0.0010 | -0.7190 |
| EVX1 | -0.5187 | 1.1081 | -3.5739 | 0.0004 | 0.0010 | -0.7210 |
| 44083.0000 | -0.0686 | 6.7770 | -3.5730 | 0.0004 | 0.0010 | -0.7240 |
| CHODL | -0.5212 | 1.1919 | -3.5724 | 0.0004 | 0.0010 | -0.7260 |
| RAP1B | -0.1245 | 5.0081 | -3.5723 | 0.0004 | 0.0010 | -0.7262 |
| SCRG1 | -0.2132 | 0.5490 | -3.5722 | 0.0004 | 0.0010 | -0.7265 |
| DLX5 | -0.5568 | 1.4277 | -3.5722 | 0.0004 | 0.0010 | -0.7266 |
| UBR2 | 0.0972 | 6.0409 | 3.5720 | 0.0004 | 0.0010 | -0.7273 |
| CHRNA4 | 0.8776 | 2.8018 | 3.5717 | 0.0004 | 0.0010 | -0.7286 |
| CTRB2 | -0.2840 | 0.3412 | -3.5714 | 0.0004 | 0.0010 | -0.7296 |
| FXYD5 | -0.1759 | 6.3011 | -3.5711 | 0.0004 | 0.0010 | -0.7305 |
| OR7A5 | -0.2445 | 0.3068 | -3.5711 | 0.0004 | 0.0010 | -0.7305 |
| NR1D2 | 0.1264 | 6.0911 | 3.5711 | 0.0004 | 0.0010 | -0.7306 |
| RASL10A | -0.2895 | 2.3396 | -3.5707 | 0.0004 | 0.0010 | -0.7317 |
| ZNF804A | -0.3934 | 1.0712 | -3.5695 | 0.0004 | 0.0010 | -0.7359 |
| GALNT10 | -0.1625 | 5.2384 | -3.5691 | 0.0004 | 0.0010 | -0.7373 |
| SETD5 | -0.1187 | 5.9494 | -3.5691 | 0.0004 | 0.0010 | -0.7375 |
| ZNF574 | -0.0931 | 5.6547 | -3.5685 | 0.0004 | 0.0010 | -0.7393 |
| HPRT1 | 0.1020 | 6.7387 | 3.5681 | 0.0004 | 0.0010 | -0.7406 |

| STUB1 | 0.0840 | 6.6985 | 3.5680 | 0.0004 | 0.0010 | -0.7409 |
| --- | --- | --- | --- | --- | --- | --- |
| LUC7L3 | -0.0730 | 6.2924 | -3.5665 | 0.0004 | 0.0010 | -0.7462 |
| ATP6AP2 | 0.0505 | 7.1203 | 3.5660 | 0.0004 | 0.0010 | -0.7480 |
| AKAP10 | -0.1439 | 5.3278 | -3.5647 | 0.0004 | 0.0010 | -0.7525 |
| NRL | 0.1861 | 4.3105 | 3.5642 | 0.0004 | 0.0010 | -0.7540 |
| DAAM2 | 0.3963 | 4.3000 | 3.5642 | 0.0004 | 0.0010 | -0.7542 |
| ROGDI | 0.1232 | 6.2082 | 3.5637 | 0.0004 | 0.0010 | -0.7557 |
| IRF2 | 0.1051 | 6.3822 | 3.5629 | 0.0004 | 0.0010 | -0.7586 |
| GLCE | -0.1414 | 5.9676 | -3.5628 | 0.0004 | 0.0010 | -0.7589 |
| MAPRE2 | 0.1541 | 6.1128 | 3.5628 | 0.0004 | 0.0010 | -0.7590 |
| SESN1 | 0.1531 | 5.8484 | 3.5626 | 0.0004 | 0.0010 | -0.7595 |
| HIST3H3 | -0.2367 | 0.2577 | -3.5609 | 0.0004 | 0.0010 | -0.7653 |
| DYNC1LI1 | -0.0823 | 5.8638 | -3.5604 | 0.0004 | 0.0010 | -0.7672 |
| LEMD3 | -0.1263 | 5.5094 | -3.5601 | 0.0004 | 0.0010 | -0.7683 |
| SIRPG | -0.5126 | 3.4310 | -3.5600 | 0.0004 | 0.0010 | -0.7685 |
| PPFIA2 | -0.2579 | 0.7971 | -3.5596 | 0.0004 | 0.0010 | -0.7697 |
| TOX4 | 0.0572 | 6.2946 | 3.5589 | 0.0004 | 0.0010 | -0.7722 |
| RPL41 | 0.0613 | 7.5310 | 3.5582 | 0.0004 | 0.0010 | -0.7745 |
| ADARB2 | -0.3619 | 0.6498 | -3.5572 | 0.0004 | 0.0010 | -0.7781 |
| LDOC1 | -0.3961 | 4.9779 | -3.5572 | 0.0004 | 0.0010 | -0.7781 |
| MARS | -0.0716 | 6.4994 | -3.5570 | 0.0004 | 0.0010 | -0.7787 |
| FBXO16 | -0.4270 | 2.9976 | -3.5561 | 0.0004 | 0.0010 | -0.7817 |
| RABGGTA | 0.0802 | 6.2662 | 3.5561 | 0.0004 | 0.0010 | -0.7817 |
| CHMP4A | -0.2890 | 4.2068 | -3.5558 | 0.0004 | 0.0010 | -0.7828 |
| EIF4ENIF1 | -0.1032 | 5.6152 | -3.5553 | 0.0004 | 0.0010 | -0.7844 |
| EXD2 | -0.1505 | 5.2489 | -3.5551 | 0.0004 | 0.0010 | -0.7852 |
| NSFL1C | 0.0693 | 6.6109 | 3.5549 | 0.0004 | 0.0010 | -0.7860 |
| TBX1 | -0.4896 | 1.5557 | -3.5546 | 0.0004 | 0.0010 | -0.7870 |
| ZNF134 | -0.2188 | 5.1145 | -3.5541 | 0.0004 | 0.0010 | -0.7885 |
| ADNP | -0.0739 | 6.3941 | -3.5541 | 0.0004 | 0.0010 | -0.7887 |
| DHRS11 | 0.1548 | 5.5101 | 3.5534 | 0.0004 | 0.0010 | -0.7910 |
| MCCC1 | 0.1176 | 6.4418 | 3.5534 | 0.0004 | 0.0010 | -0.7910 |
| CYB5R1 | 0.0934 | 6.7552 | 3.5530 | 0.0004 | 0.0010 | -0.7923 |
| HIST1H2BM | -0.3898 | 0.8928 | -3.5523 | 0.0004 | 0.0010 | -0.7947 |
| SAMSN1 | -0.3736 | 4.2135 | -3.5510 | 0.0004 | 0.0010 | -0.7991 |
| WDFY3 | -0.2446 | 4.5159 | -3.5507 | 0.0004 | 0.0010 | -0.8001 |
| S100A13 | -0.1973 | 6.4391 | -3.5507 | 0.0004 | 0.0010 | -0.8001 |
| RHOD | 0.1449 | 6.9719 | 3.5499 | 0.0004 | 0.0010 | -0.8030 |
| TRIM22 | 0.3175 | 5.4885 | 3.5486 | 0.0004 | 0.0011 | -0.8074 |
| C3orf14 | -0.5563 | 3.2393 | -3.5486 | 0.0004 | 0.0011 | -0.8074 |
| IDH2 | 0.0871 | 7.3894 | 3.5481 | 0.0004 | 0.0011 | -0.8092 |
| HK3 | -0.3741 | 4.1685 | -3.5478 | 0.0004 | 0.0011 | -0.8103 |
| GSK3A | -0.0583 | 6.5575 | -3.5472 | 0.0004 | 0.0011 | -0.8122 |
| TLR1 | -0.3621 | 4.1920 | -3.5467 | 0.0004 | 0.0011 | -0.8138 |
| KLK15 | -0.2604 | 0.2925 | -3.5457 | 0.0004 | 0.0011 | -0.8175 |
| C14orf159 | 0.1811 | 5.7746 | 3.5453 | 0.0004 | 0.0011 | -0.8186 |
| AGPS | -0.1023 | 6.0662 | -3.5441 | 0.0004 | 0.0011 | -0.8228 |
| ZNF468 | -0.4941 | 4.5360 | -3.5438 | 0.0004 | 0.0011 | -0.8238 |
| TTC22 | -0.3519 | 4.6031 | -3.5437 | 0.0004 | 0.0011 | -0.8241 |
| CASP6 | -0.0889 | 6.2497 | -3.5435 | 0.0004 | 0.0011 | -0.8246 |
| NXT1 | -0.0955 | 6.4593 | -3.5421 | 0.0004 | 0.0011 | -0.8294 |
| TCN2 | -0.1526 | 6.1420 | -3.5420 | 0.0004 | 0.0011 | -0.8301 |
| IMPG1 | -0.2343 | 0.8098 | -3.5406 | 0.0004 | 0.0011 | -0.8347 |

| DOCK10 | -0.3803 | 3.6549 | -3.5401 | 0.0005 | 0.0011 | -0.8364 |
| --- | --- | --- | --- | --- | --- | --- |
| SUPT7L | -0.0601 | 6.1380 | -3.5401 | 0.0005 | 0.0011 | -0.8364 |
| SCN2B | -0.1970 | 0.3369 | -3.5390 | 0.0005 | 0.0011 | -0.8400 |
| RUVBL1 | -0.0954 | 5.9666 | -3.5379 | 0.0005 | 0.0011 | -0.8439 |
| RNF220 | -0.0777 | 6.0202 | -3.5378 | 0.0005 | 0.0011 | -0.8440 |
| RFX7 | -0.1809 | 4.5461 | -3.5376 | 0.0005 | 0.0011 | -0.8449 |
| JRK | -0.2364 | 4.3464 | -3.5375 | 0.0005 | 0.0011 | -0.8451 |
| TMEM177 | 0.1147 | 6.2230 | 3.5375 | 0.0005 | 0.0011 | -0.8451 |
| DGCR8 | -0.0930 | 5.6583 | -3.5374 | 0.0005 | 0.0011 | -0.8456 |
| NQO2 | 0.1770 | 6.2086 | 3.5363 | 0.0005 | 0.0011 | -0.8493 |
| ZNF556 | -0.3452 | 0.6573 | -3.5362 | 0.0005 | 0.0011 | -0.8497 |
| FOXC2 | -0.4357 | 2.4914 | -3.5360 | 0.0005 | 0.0011 | -0.8502 |
| LRRFIP1 | 0.1016 | 6.1348 | 3.5358 | 0.0005 | 0.0011 | -0.8509 |
| GABBR1 | -0.4248 | 3.3796 | -3.5354 | 0.0005 | 0.0011 | -0.8524 |
| KCNK3 | -0.4536 | 1.4663 | -3.5327 | 0.0005 | 0.0011 | -0.8617 |
| KCNA2 | -0.2245 | 0.3505 | -3.5322 | 0.0005 | 0.0011 | -0.8631 |
| PSG1 | -0.0560 | 0.0518 | -3.5322 | 0.0005 | 0.0011 | -0.8632 |
| SCARF1 | 0.1641 | 5.2088 | 3.5322 | 0.0005 | 0.0011 | -0.8633 |
| AP1G1 | 0.1008 | 6.1219 | 3.5320 | 0.0005 | 0.0011 | -0.8640 |
| PDCD6IP | 0.0639 | 6.5073 | 3.5296 | 0.0005 | 0.0011 | -0.8721 |
| LBX1 | -0.2351 | 0.1920 | -3.5295 | 0.0005 | 0.0011 | -0.8723 |
| TMEM161A | 0.0880 | 6.7347 | 3.5295 | 0.0005 | 0.0011 | -0.8724 |
| CADM3 | -0.4852 | 1.2916 | -3.5279 | 0.0005 | 0.0011 | -0.8780 |
| ZNF358 | 0.1006 | 6.8853 | 3.5274 | 0.0005 | 0.0011 | -0.8795 |
| NUBP1 | 0.0826 | 6.2979 | 3.5264 | 0.0005 | 0.0011 | -0.8828 |
| CSPG5 | -0.4357 | 2.9992 | -3.5249 | 0.0005 | 0.0011 | -0.8880 |
| PTCD2 | 0.1518 | 4.2348 | 3.5246 | 0.0005 | 0.0011 | -0.8889 |
| NIN | -0.1901 | 5.0631 | -3.5229 | 0.0005 | 0.0011 | -0.8946 |
| COPS5 | 0.0878 | 6.3702 | 3.5229 | 0.0005 | 0.0011 | -0.8949 |
| PRL | -0.2317 | 0.2067 | -3.5220 | 0.0005 | 0.0012 | -0.8980 |
| DLEC1 | 0.2815 | 1.8549 | 3.5215 | 0.0005 | 0.0012 | -0.8995 |
| MRPS18A | 0.0769 | 6.7839 | 3.5214 | 0.0005 | 0.0012 | -0.8999 |
| MAST3 | 0.1385 | 5.8430 | 3.5209 | 0.0005 | 0.0012 | -0.9015 |
| RNASET2 | -0.1623 | 5.9125 | -3.5205 | 0.0005 | 0.0012 | -0.9028 |
| LOXL2 | -0.2022 | 5.4319 | -3.5199 | 0.0005 | 0.0012 | -0.9049 |
| RTP4 | 0.2365 | 5.9729 | 3.5194 | 0.0005 | 0.0012 | -0.9066 |
| MSLN | -0.4152 | 1.6919 | -3.5193 | 0.0005 | 0.0012 | -0.9071 |
| MYO5C | -0.2475 | 4.8928 | -3.5172 | 0.0005 | 0.0012 | -0.9141 |
| EAF2 | -0.2179 | 4.5637 | -3.5170 | 0.0005 | 0.0012 | -0.9148 |
| PDAP1 | 0.0544 | 7.0412 | 3.5169 | 0.0005 | 0.0012 | -0.9152 |
| ACTA2 | 0.1305 | 6.9354 | 3.5166 | 0.0005 | 0.0012 | -0.9161 |
| ANXA3 | -0.5091 | 2.5984 | -3.5156 | 0.0005 | 0.0012 | -0.9194 |
| ANKH | 0.1170 | 6.2884 | 3.5148 | 0.0005 | 0.0012 | -0.9222 |
| AKAP8 | -0.0673 | 6.0878 | -3.5145 | 0.0005 | 0.0012 | -0.9234 |
| ZFP64 | -0.1223 | 5.2410 | -3.5143 | 0.0005 | 0.0012 | -0.9238 |
| PTPN7 | -0.3886 | 3.9199 | -3.5133 | 0.0005 | 0.0012 | -0.9273 |
| MFF | -0.0647 | 6.3483 | -3.5110 | 0.0005 | 0.0012 | -0.9349 |
| HBQ1 | -0.4398 | 0.7367 | -3.5101 | 0.0005 | 0.0012 | -0.9379 |
| FKBP5 | 0.1851 | 6.4359 | 3.5100 | 0.0005 | 0.0012 | -0.9383 |
| PI4K2A | 0.0854 | 6.2262 | 3.5097 | 0.0005 | 0.0012 | -0.9394 |
| YWHAE | 0.0547 | 7.3122 | 3.5089 | 0.0005 | 0.0012 | -0.9422 |
| TCERG1 | -0.1345 | 5.5976 | -3.5084 | 0.0005 | 0.0012 | -0.9437 |
| PCDH9 | -0.3578 | 0.9513 | -3.5080 | 0.0005 | 0.0012 | -0.9452 |

| GBX1 | -0.1776 | 0.1637 | -3.5074 | 0.0005 | 0.0012 | -0.9473 |
| --- | --- | --- | --- | --- | --- | --- |
| PIK3C2G | 0.6386 | 4.2528 | 3.5067 | 0.0005 | 0.0012 | -0.9496 |
| ZFAND1 | 0.1070 | 6.1102 | 3.5053 | 0.0005 | 0.0012 | -0.9541 |
| KLHL23 | -0.2151 | 5.4624 | -3.5048 | 0.0005 | 0.0012 | -0.9558 |
| CAMSAP1 | -0.1346 | 5.2920 | -3.5048 | 0.0005 | 0.0012 | -0.9560 |
| PLP1 | -0.2936 | 0.3515 | -3.5046 | 0.0005 | 0.0012 | -0.9567 |
| MAML1 | -0.1468 | 5.3421 | -3.5045 | 0.0005 | 0.0012 | -0.9571 |
| NGDN | 0.0747 | 6.0762 | 3.5043 | 0.0005 | 0.0012 | -0.9577 |
| EDC3 | -0.0826 | 5.9456 | -3.5035 | 0.0005 | 0.0012 | -0.9604 |
| PIN4 | 0.1192 | 6.1079 | 3.5024 | 0.0005 | 0.0012 | -0.9640 |
| ASB4 | 0.5609 | 2.8253 | 3.5024 | 0.0005 | 0.0012 | -0.9640 |
| GABRB1 | -0.2912 | 0.4077 | -3.5024 | 0.0005 | 0.0012 | -0.9641 |
| SCGB1D2 | -0.7341 | 1.4309 | -3.5023 | 0.0005 | 0.0012 | -0.9644 |
| SLC5A3 | -0.2898 | 4.4868 | -3.5017 | 0.0005 | 0.0012 | -0.9664 |
| ARID3B | -0.2361 | 4.2496 | -3.5014 | 0.0005 | 0.0012 | -0.9673 |
| GOLGA7 | 0.0896 | 6.6522 | 3.5014 | 0.0005 | 0.0012 | -0.9675 |
| PTGER3 | 0.3810 | 1.7952 | 3.4988 | 0.0005 | 0.0012 | -0.9763 |
| CACNA1B | -0.2155 | 0.2512 | -3.4986 | 0.0005 | 0.0012 | -0.9770 |
| CD47 | -0.1418 | 6.1301 | -3.4979 | 0.0005 | 0.0012 | -0.9792 |
| NUP88 | 0.0957 | 6.1821 | 3.4975 | 0.0005 | 0.0012 | -0.9804 |
| TFEC | -0.3812 | 2.6465 | -3.4967 | 0.0005 | 0.0013 | -0.9834 |
| TIMM13 | 0.0843 | 7.0905 | 3.4944 | 0.0005 | 0.0013 | -0.9909 |
| RPL7A | 0.0635 | 7.7615 | 3.4944 | 0.0005 | 0.0013 | -0.9910 |
| ARPC1A | 0.0617 | 7.1607 | 3.4938 | 0.0005 | 0.0013 | -0.9931 |
| AHR | 0.1668 | 6.4155 | 3.4933 | 0.0005 | 0.0013 | -0.9945 |
| U2AF1 | -0.2071 | 1.4506 | -3.4930 | 0.0005 | 0.0013 | -0.9958 |
| ZNF562 | -0.1308 | 4.8135 | -3.4921 | 0.0005 | 0.0013 | -0.9988 |
| LIMCH1 | -0.3848 | 4.3116 | -3.4920 | 0.0005 | 0.0013 | -0.9989 |
| VIPR2 | -0.3432 | 0.7576 | -3.4913 | 0.0005 | 0.0013 | -1.0015 |
| DCTN3 | 0.0868 | 6.4695 | 3.4911 | 0.0005 | 0.0013 | -1.0019 |
| ADAM10 | -0.1064 | 5.9284 | -3.4910 | 0.0005 | 0.0013 | -1.0025 |
| MORC3 | 0.1718 | 5.5224 | 3.4892 | 0.0005 | 0.0013 | -1.0083 |
| FRMD1 | -0.4640 | 1.1505 | -3.4892 | 0.0005 | 0.0013 | -1.0086 |
| ZNF549 | -0.3945 | 2.7433 | -3.4888 | 0.0005 | 0.0013 | -1.0097 |
| EHD1 | -0.0854 | 6.1878 | -3.4882 | 0.0005 | 0.0013 | -1.0117 |
| TDRD12 | -0.2022 | 0.5681 | -3.4873 | 0.0005 | 0.0013 | -1.0148 |
| TRIT1 | -0.0902 | 5.8861 | -3.4871 | 0.0005 | 0.0013 | -1.0155 |
| SLC35A3 | 0.1679 | 5.9586 | 3.4868 | 0.0005 | 0.0013 | -1.0164 |
| MAB21L2 | -0.6341 | 3.0473 | -3.4854 | 0.0005 | 0.0013 | -1.0211 |
| EFNB1 | -0.1296 | 6.2345 | -3.4851 | 0.0006 | 0.0013 | -1.0223 |
| DAB1 | 0.5165 | 3.4356 | 3.4843 | 0.0006 | 0.0013 | -1.0250 |
| DNAH6 | 0.5118 | 2.2744 | 3.4841 | 0.0006 | 0.0013 | -1.0257 |
| SARS2 | -0.1558 | 5.1715 | -3.4829 | 0.0006 | 0.0013 | -1.0295 |
| ARHGDIG | -0.3381 | 0.6931 | -3.4821 | 0.0006 | 0.0013 | -1.0322 |
| MTA2 | -0.0472 | 6.6039 | -3.4809 | 0.0006 | 0.0013 | -1.0364 |
| ZBTB24 | -0.1361 | 4.8490 | -3.4803 | 0.0006 | 0.0013 | -1.0382 |
| LPIN1 | 0.2165 | 5.5726 | 3.4787 | 0.0006 | 0.0013 | -1.0438 |
| ASXL1 | -0.0857 | 6.1347 | -3.4785 | 0.0006 | 0.0013 | -1.0442 |
| PTPRZ1 | -0.2895 | 0.6214 | -3.4783 | 0.0006 | 0.0013 | -1.0448 |
| CLINT1 | 0.0938 | 6.5863 | 3.4777 | 0.0006 | 0.0013 | -1.0471 |
| TBR1 | -0.0653 | 0.0589 | -3.4775 | 0.0006 | 0.0013 | -1.0478 |
| HSD17B1 | -0.2247 | 3.3884 | -3.4773 | 0.0006 | 0.0013 | -1.0482 |
| WNT5B | 0.5187 | 4.2041 | 3.4766 | 0.0006 | 0.0013 | -1.0506 |

| EYA4 | -0.4602 | 0.7383 | -3.4759 | 0.0006 | 0.0013 | -1.0531 |
| --- | --- | --- | --- | --- | --- | --- |
| ABCD4 | 0.1053 | 5.8678 | 3.4758 | 0.0006 | 0.0013 | -1.0535 |
| PFDN2 | 0.0668 | 7.3852 | 3.4756 | 0.0006 | 0.0013 | -1.0540 |
| CELSR1 | -0.3856 | 4.3625 | -3.4755 | 0.0006 | 0.0013 | -1.0545 |
| SMYD3 | -0.2547 | 4.6821 | -3.4749 | 0.0006 | 0.0013 | -1.0564 |
| CUBN | -0.2458 | 1.3331 | -3.4730 | 0.0006 | 0.0014 | -1.0628 |
| HOXC8 | -0.4922 | 0.8096 | -3.4729 | 0.0006 | 0.0014 | -1.0631 |
| RGS13 | -0.3661 | 0.6924 | -3.4728 | 0.0006 | 0.0014 | -1.0633 |
| EPHA4 | 0.3738 | 2.8634 | 3.4725 | 0.0006 | 0.0014 | -1.0643 |
| PITPNM1 | -0.2064 | 5.5941 | -3.4721 | 0.0006 | 0.0014 | -1.0658 |
| ADM | -0.1876 | 6.0166 | -3.4717 | 0.0006 | 0.0014 | -1.0670 |
| SLC17A7 | -0.2932 | 1.2615 | -3.4717 | 0.0006 | 0.0014 | -1.0671 |
| RRP12 | -0.1401 | 5.7961 | -3.4706 | 0.0006 | 0.0014 | -1.0709 |
| EEF1B2 | 0.0685 | 7.5011 | 3.4690 | 0.0006 | 0.0014 | -1.0761 |
| APBA2 | -0.4944 | 2.4656 | -3.4689 | 0.0006 | 0.0014 | -1.0763 |
| SNCA | -0.3974 | 1.8620 | -3.4686 | 0.0006 | 0.0014 | -1.0776 |
| MYOZ3 | -0.2517 | 1.0701 | -3.4684 | 0.0006 | 0.0014 | -1.0781 |
| NDUFA3 | 0.1060 | 6.9288 | 3.4669 | 0.0006 | 0.0014 | -1.0829 |
| MDM1 | -0.2240 | 4.2143 | -3.4668 | 0.0006 | 0.0014 | -1.0833 |
| ADRB1 | 0.4792 | 1.9388 | 3.4649 | 0.0006 | 0.0014 | -1.0898 |
| MRPL18 | 0.0644 | 7.0478 | 3.4634 | 0.0006 | 0.0014 | -1.0947 |
| RPS23 | 0.0684 | 7.4352 | 3.4626 | 0.0006 | 0.0014 | -1.0975 |
| NCOR2 | -0.0980 | 6.1491 | -3.4625 | 0.0006 | 0.0014 | -1.0978 |
| FAM189A1 | -0.3403 | 0.9147 | -3.4620 | 0.0006 | 0.0014 | -1.0994 |
| ELMO1 | 0.1676 | 5.7664 | 3.4610 | 0.0006 | 0.0014 | -1.1029 |
| ATP6V1A | 0.0649 | 6.6214 | 3.4602 | 0.0006 | 0.0014 | -1.1053 |
| ARF6 | 0.0526 | 6.8941 | 3.4602 | 0.0006 | 0.0014 | -1.1053 |
| SORBS1 | 0.1735 | 5.8554 | 3.4598 | 0.0006 | 0.0014 | -1.1066 |
| HPCAL4 | -0.1882 | 0.3936 | -3.4597 | 0.0006 | 0.0014 | -1.1070 |
| MAGIX | 0.2936 | 5.3364 | 3.4597 | 0.0006 | 0.0014 | -1.1072 |
| BNC2 | -0.3322 | 1.2590 | -3.4546 | 0.0006 | 0.0014 | -1.1241 |
| IRGC | -0.1073 | 0.1116 | -3.4544 | 0.0006 | 0.0014 | -1.1247 |
| FOXO4 | 0.1268 | 5.9298 | 3.4533 | 0.0006 | 0.0014 | -1.1285 |
| KCNAB2 | -0.3481 | 5.0498 | -3.4522 | 0.0006 | 0.0014 | -1.1319 |
| MED17 | -0.1480 | 4.8707 | -3.4497 | 0.0006 | 0.0015 | -1.1402 |
| WRAP73 | -0.1160 | 5.1982 | -3.4494 | 0.0006 | 0.0015 | -1.1413 |
| UBE2D1 | -0.1196 | 5.7308 | -3.4492 | 0.0006 | 0.0015 | -1.1421 |
| DIP2A | -0.1674 | 4.7940 | -3.4485 | 0.0006 | 0.0015 | -1.1442 |
| SNAI2 | 0.3236 | 5.6227 | 3.4483 | 0.0006 | 0.0015 | -1.1451 |
| BATF | -0.3903 | 5.2812 | -3.4480 | 0.0006 | 0.0015 | -1.1459 |
| AKT2 | 0.0814 | 6.3437 | 3.4465 | 0.0006 | 0.0015 | -1.1510 |
| RDH8 | -0.5168 | 0.9832 | -3.4461 | 0.0006 | 0.0015 | -1.1521 |
| FASTK | 0.0727 | 6.9227 | 3.4454 | 0.0006 | 0.0015 | -1.1545 |
| PLOD2 | -0.1649 | 6.4139 | -3.4454 | 0.0006 | 0.0015 | -1.1547 |
| BTBD7 | -0.1569 | 4.8888 | -3.4442 | 0.0006 | 0.0015 | -1.1587 |
| FBXO42 | -0.1013 | 5.3072 | -3.4435 | 0.0006 | 0.0015 | -1.1610 |
| ORC4 | -0.1020 | 5.3485 | -3.4430 | 0.0006 | 0.0015 | -1.1627 |
| XPA | 0.0831 | 6.2200 | 3.4426 | 0.0006 | 0.0015 | -1.1640 |
| LAMB4 | -0.2553 | 0.9278 | -3.4424 | 0.0006 | 0.0015 | -1.1647 |
| AFTPH | -0.0774 | 6.0621 | -3.4420 | 0.0006 | 0.0015 | -1.1660 |
| RNF44 | -0.1197 | 6.0671 | -3.4412 | 0.0006 | 0.0015 | -1.1687 |
| MFSD5 | -0.0767 | 6.4934 | -3.4395 | 0.0006 | 0.0015 | -1.1740 |
| PHKA2 | -0.0960 | 6.2831 | -3.4393 | 0.0006 | 0.0015 | -1.1747 |

| GATA3 | -0.4281 | 3.1375 | -3.4386 | 0.0007 | 0.0015 | -1.1770 |
| --- | --- | --- | --- | --- | --- | --- |
| ATP5E | 0.0822 | 7.1864 | 3.4386 | 0.0007 | 0.0015 | -1.1770 |
| KHDRBS2 | -0.2834 | 0.5101 | -3.4382 | 0.0007 | 0.0015 | -1.1784 |
| SAP30 | -0.1494 | 5.7775 | -3.4378 | 0.0007 | 0.0015 | -1.1799 |
| EBF2 | 0.3708 | 2.3779 | 3.4362 | 0.0007 | 0.0015 | -1.1850 |
| APOBEC3A | -0.3830 | 1.7263 | -3.4347 | 0.0007 | 0.0015 | -1.1902 |
| SF3B5 | 0.0724 | 7.4568 | 3.4344 | 0.0007 | 0.0015 | -1.1909 |
| SLC9A8 | 0.1146 | 5.7670 | 3.4341 | 0.0007 | 0.0015 | -1.1922 |
| GREM2 | 0.7834 | 4.1760 | 3.4338 | 0.0007 | 0.0015 | -1.1930 |
| DENR | -0.0537 | 6.6029 | -3.4332 | 0.0007 | 0.0015 | -1.1952 |
| TMEM97 | 0.1385 | 6.9742 | 3.4329 | 0.0007 | 0.0015 | -1.1961 |
| CIZ1 | -0.0771 | 6.3945 | -3.4317 | 0.0007 | 0.0016 | -1.1999 |
| PCSK2 | -0.1571 | 0.1602 | -3.4313 | 0.0007 | 0.0016 | -1.2012 |
| HIST1H3C | -0.4998 | 2.7210 | -3.4292 | 0.0007 | 0.0016 | -1.2081 |
| TUBG1 | -0.0854 | 6.6730 | -3.4289 | 0.0007 | 0.0016 | -1.2093 |
| C19orf73 | 0.2491 | 5.0060 | 3.4285 | 0.0007 | 0.0016 | -1.2106 |
| MEX3C | -0.1761 | 5.3225 | -3.4278 | 0.0007 | 0.0016 | -1.2127 |
| FUBP3 | 0.0922 | 6.3458 | 3.4270 | 0.0007 | 0.0016 | -1.2157 |
| PIK3R4 | 0.1192 | 5.9768 | 3.4232 | 0.0007 | 0.0016 | -1.2279 |
| LAIR2 | -0.5048 | 2.3947 | -3.4232 | 0.0007 | 0.0016 | -1.2279 |
| TRIM9 | -0.5528 | 1.8316 | -3.4232 | 0.0007 | 0.0016 | -1.2280 |
| PDLIM5 | 0.1184 | 6.2401 | 3.4227 | 0.0007 | 0.0016 | -1.2297 |
| HTN1 | -0.1281 | 0.0963 | -3.4225 | 0.0007 | 0.0016 | -1.2302 |
| ASCC2 | 0.0651 | 6.6748 | 3.4222 | 0.0007 | 0.0016 | -1.2315 |
| LGALS2 | -0.4124 | 5.0561 | -3.4221 | 0.0007 | 0.0016 | -1.2318 |
| ESRP2 | 0.2456 | 6.2999 | 3.4220 | 0.0007 | 0.0016 | -1.2321 |
| MINPP1 | 0.1250 | 6.3913 | 3.4220 | 0.0007 | 0.0016 | -1.2322 |
| MRPL12 | 0.0911 | 7.1227 | 3.4219 | 0.0007 | 0.0016 | -1.2322 |
| SCAMP5 | -0.3087 | 5.4114 | -3.4219 | 0.0007 | 0.0016 | -1.2325 |
| FBXO2 | 0.3188 | 6.3113 | 3.4208 | 0.0007 | 0.0016 | -1.2360 |
| IMP4 | 0.0693 | 6.7149 | 3.4207 | 0.0007 | 0.0016 | -1.2361 |
| RAD17 | 0.0746 | 5.9759 | 3.4206 | 0.0007 | 0.0016 | -1.2365 |
| ACD | -0.1113 | 5.8216 | -3.4202 | 0.0007 | 0.0016 | -1.2381 |
| CYP3A7 | 0.4830 | 6.1135 | 3.4197 | 0.0007 | 0.0016 | -1.2397 |
| ADD2 | -0.2953 | 1.0358 | -3.4190 | 0.0007 | 0.0016 | -1.2419 |
| RAB1A | 0.0473 | 7.2017 | 3.4183 | 0.0007 | 0.0016 | -1.2443 |
| DOCK3 | -0.3266 | 1.3271 | -3.4172 | 0.0007 | 0.0016 | -1.2480 |
| SSRP1 | -0.0530 | 6.7039 | -3.4165 | 0.0007 | 0.0016 | -1.2501 |
| PFKFB2 | -0.2426 | 4.7720 | -3.4159 | 0.0007 | 0.0016 | -1.2521 |
| NDUFB3 | 0.0633 | 7.0661 | 3.4152 | 0.0007 | 0.0016 | -1.2543 |
| KIR2DL4 | -0.4223 | 1.5608 | -3.4151 | 0.0007 | 0.0016 | -1.2548 |
| PLCXD1 | -0.2381 | 5.7729 | -3.4149 | 0.0007 | 0.0016 | -1.2554 |
| CHRNG | -0.1617 | 0.4378 | -3.4147 | 0.0007 | 0.0016 | -1.2559 |
| PCDHB6 | -0.4176 | 1.8426 | -3.4147 | 0.0007 | 0.0016 | -1.2561 |
| COL4A3 | -0.4697 | 1.8325 | -3.4140 | 0.0007 | 0.0016 | -1.2583 |
| SLC7A2 | 0.2394 | 6.5723 | 3.4131 | 0.0007 | 0.0016 | -1.2614 |
| APBB2 | 0.1682 | 5.3843 | 3.4099 | 0.0007 | 0.0017 | -1.2718 |
| EIF3I | 0.0598 | 7.4109 | 3.4095 | 0.0007 | 0.0017 | -1.2731 |
| WDR60 | -0.1819 | 4.6871 | -3.4091 | 0.0007 | 0.0017 | -1.2744 |
| GPR107 | -0.0911 | 6.1915 | -3.4091 | 0.0007 | 0.0017 | -1.2746 |
| IGFBP6 | -0.2964 | 5.1648 | -3.4089 | 0.0007 | 0.0017 | -1.2750 |
| ATP9B | -0.1769 | 4.6252 | -3.4089 | 0.0007 | 0.0017 | -1.2750 |
| OR11A1 | -0.1572 | 0.1353 | -3.4087 | 0.0007 | 0.0017 | -1.2759 |

| SMARCC1 | -0.0832 | 6.3379 | -3.4086 | 0.0007 | 0.0017 | -1.2760 |
| --- | --- | --- | --- | --- | --- | --- |
| ARMCX3 | -0.3644 | 5.5646 | -3.4084 | 0.0007 | 0.0017 | -1.2767 |
| ZNF224 | -0.1772 | 4.4778 | -3.4083 | 0.0007 | 0.0017 | -1.2772 |
| DCAF10 | 0.0893 | 5.8828 | 3.4075 | 0.0007 | 0.0017 | -1.2798 |
| SLC3A1 | -0.7046 | 3.0162 | -3.4067 | 0.0007 | 0.0017 | -1.2824 |
| EPB41L1 | -0.3271 | 5.3941 | -3.4061 | 0.0007 | 0.0017 | -1.2842 |
| LANCL2 | 0.0902 | 5.9168 | 3.4055 | 0.0007 | 0.0017 | -1.2862 |
| PMFBP1 | -0.3905 | 2.3265 | -3.4055 | 0.0007 | 0.0017 | -1.2864 |
| EXTL3 | -0.1569 | 5.2923 | -3.4052 | 0.0007 | 0.0017 | -1.2874 |
| STEAP3 | 0.2276 | 6.6777 | 3.4051 | 0.0007 | 0.0017 | -1.2875 |
| ROR2 | -0.5225 | 2.6766 | -3.4047 | 0.0007 | 0.0017 | -1.2890 |
| CASP4 | -0.1431 | 5.9948 | -3.4037 | 0.0007 | 0.0017 | -1.2921 |
| ABHD5 | 0.1353 | 5.7551 | 3.4035 | 0.0007 | 0.0017 | -1.2930 |
| PRB3 | -0.2299 | 0.5036 | -3.4021 | 0.0007 | 0.0017 | -1.2973 |
| CIDEB | 0.4156 | 2.5726 | 3.4012 | 0.0007 | 0.0017 | -1.3005 |
| AHI1 | -0.2383 | 4.1350 | -3.4007 | 0.0007 | 0.0017 | -1.3020 |
| CCNC | 0.0960 | 6.3815 | 3.4003 | 0.0007 | 0.0017 | -1.3032 |
| APOBEC3G | -0.3252 | 4.3137 | -3.3992 | 0.0007 | 0.0017 | -1.3071 |
| NAALADL1 | -0.4194 | 3.9608 | -3.3987 | 0.0007 | 0.0017 | -1.3085 |
| WNT7A | -0.2970 | 0.4707 | -3.3985 | 0.0008 | 0.0017 | -1.3093 |
| MKNK1 | -0.0903 | 5.4766 | -3.3967 | 0.0008 | 0.0017 | -1.3150 |
| ACVR1B | -0.1099 | 6.3444 | -3.3963 | 0.0008 | 0.0017 | -1.3165 |
| CXorf21 | -0.3647 | 3.3040 | -3.3962 | 0.0008 | 0.0017 | -1.3169 |
| SIX1 | -0.5178 | 2.1698 | -3.3961 | 0.0008 | 0.0017 | -1.3171 |
| PLA2G5 | 0.3811 | 4.1447 | 3.3941 | 0.0008 | 0.0017 | -1.3235 |
| PAPPA | -0.3386 | 1.3068 | -3.3938 | 0.0008 | 0.0018 | -1.3245 |
| SLC1A7 | -0.6484 | 3.2676 | -3.3929 | 0.0008 | 0.0018 | -1.3274 |
| CDK11A | -0.1952 | 4.5057 | -3.3927 | 0.0008 | 0.0018 | -1.3284 |
| SERPINB7 | -0.2466 | 0.1938 | -3.3926 | 0.0008 | 0.0018 | -1.3286 |
| RPL7 | 0.0772 | 7.5853 | 3.3923 | 0.0008 | 0.0018 | -1.3297 |
| EIF3J | 0.0551 | 6.7414 | 3.3917 | 0.0008 | 0.0018 | -1.3315 |
| MFGE8 | -0.2159 | 5.6665 | -3.3877 | 0.0008 | 0.0018 | -1.3445 |
| NTNG1 | -0.1731 | 0.1539 | -3.3877 | 0.0008 | 0.0018 | -1.3446 |
| FZD6 | -0.3239 | 5.2627 | -3.3877 | 0.0008 | 0.0018 | -1.3447 |
| POLG2 | -0.1376 | 5.3195 | -3.3845 | 0.0008 | 0.0018 | -1.3549 |
| KCNN3 | 0.2960 | 2.9823 | 3.3827 | 0.0008 | 0.0018 | -1.3607 |
| AHSA1 | 0.0617 | 6.9446 | 3.3825 | 0.0008 | 0.0018 | -1.3615 |
| PTPN12 | -0.0984 | 6.2454 | -3.3821 | 0.0008 | 0.0018 | -1.3628 |
| TM4SF1 | -0.1524 | 6.8388 | -3.3807 | 0.0008 | 0.0018 | -1.3676 |
| TM7SF3 | 0.1064 | 6.7398 | 3.3803 | 0.0008 | 0.0018 | -1.3688 |
| HOXD12 | -0.1417 | 0.1150 | -3.3798 | 0.0008 | 0.0018 | -1.3702 |
| TMX4 | 0.0879 | 6.5178 | 3.3795 | 0.0008 | 0.0018 | -1.3712 |
| MTHFD2L | -0.2019 | 4.2562 | -3.3784 | 0.0008 | 0.0018 | -1.3749 |
| CD7 | -0.3522 | 5.0205 | -3.3780 | 0.0008 | 0.0018 | -1.3763 |
| TNFSF12-TNFSF13 | -0.1743 | 0.3655 | -3.3779 | 0.0008 | 0.0018 | -1.3765 |
| TTC9 | 0.3942 | 5.3415 | 3.3769 | 0.0008 | 0.0019 | -1.3798 |
| SNRPD3 | 0.0630 | 7.0858 | 3.3763 | 0.0008 | 0.0019 | -1.3817 |
| ANKRD28 | 0.1143 | 5.6582 | 3.3762 | 0.0008 | 0.0019 | -1.3820 |
| F2RL1 | -0.5289 | 5.2585 | -3.3742 | 0.0008 | 0.0019 | -1.3886 |
| SETMAR | -0.1228 | 5.4955 | -3.3740 | 0.0008 | 0.0019 | -1.3892 |
| ARHGEF4 | -0.4943 | 1.3506 | -3.3738 | 0.0008 | 0.0019 | -1.3900 |
| SLC5A7 | -0.1299 | 0.0911 | -3.3731 | 0.0008 | 0.0019 | -1.3921 |
| SDF2L1 | 0.0964 | 7.1702 | 3.3731 | 0.0008 | 0.0019 | -1.3921 |

| LGSN | 0.5810 | 2.3866 | 3.3708 | 0.0008 | 0.0019 | -1.3996 |
| --- | --- | --- | --- | --- | --- | --- |
| HSPA2 | -0.2479 | 4.9080 | -3.3707 | 0.0008 | 0.0019 | -1.4001 |
| SPCS2 | 0.0622 | 6.7019 | 3.3680 | 0.0008 | 0.0019 | -1.4086 |
| ARHGEF26 | 0.3275 | 5.6381 | 3.3666 | 0.0008 | 0.0019 | -1.4134 |
| TAF7 | 0.0726 | 6.9447 | 3.3665 | 0.0008 | 0.0019 | -1.4137 |
| DUSP26 | -0.4505 | 1.8607 | -3.3638 | 0.0008 | 0.0019 | -1.4224 |
| CYLD | 0.1267 | 5.4121 | 3.3626 | 0.0009 | 0.0019 | -1.4262 |
| MEX3D | -0.1441 | 5.9014 | -3.3624 | 0.0009 | 0.0019 | -1.4268 |
| TUFT1 | -0.1794 | 5.7213 | -3.3624 | 0.0009 | 0.0019 | -1.4270 |
| PAX3 | -0.2270 | 0.2010 | -3.3620 | 0.0009 | 0.0019 | -1.4281 |
| NUDT9 | 0.0973 | 6.5231 | 3.3617 | 0.0009 | 0.0019 | -1.4291 |
| PCBP4 | -0.1229 | 6.2180 | -3.3617 | 0.0009 | 0.0019 | -1.4291 |
| ENTPD1 | -0.1537 | 4.9996 | -3.3615 | 0.0009 | 0.0019 | -1.4298 |
| STXBP2 | -0.1561 | 5.9977 | -3.3610 | 0.0009 | 0.0019 | -1.4315 |
| PIR | 0.1722 | 6.4558 | 3.3606 | 0.0009 | 0.0020 | -1.4328 |
| ARR3 | -0.2914 | 1.7641 | -3.3601 | 0.0009 | 0.0020 | -1.4342 |
| SEC31A | 0.0655 | 6.7131 | 3.3580 | 0.0009 | 0.0020 | -1.4412 |
| AGER | -0.1914 | 4.7807 | -3.3565 | 0.0009 | 0.0020 | -1.4459 |
| TDRD1 | -0.3774 | 0.5387 | -3.3562 | 0.0009 | 0.0020 | -1.4471 |
| ZNF257 | -0.4744 | 1.9648 | -3.3556 | 0.0009 | 0.0020 | -1.4489 |
| ABCC4 | -0.4821 | 4.4709 | -3.3554 | 0.0009 | 0.0020 | -1.4494 |
| SLC35B1 | 0.0699 | 6.6243 | 3.3550 | 0.0009 | 0.0020 | -1.4510 |
| ZSCAN12 | -0.2404 | 4.0034 | -3.3546 | 0.0009 | 0.0020 | -1.4523 |
| VGLL4 | -0.2774 | 5.0268 | -3.3542 | 0.0009 | 0.0020 | -1.4535 |
| C18orf8 | 0.0756 | 6.2439 | 3.3540 | 0.0009 | 0.0020 | -1.4542 |
| CDK10 | 0.0902 | 6.3923 | 3.3533 | 0.0009 | 0.0020 | -1.4565 |
| KRT9 | -0.1566 | 0.1544 | -3.3529 | 0.0009 | 0.0020 | -1.4576 |
| TRIM16 | -0.3680 | 4.9499 | -3.3527 | 0.0009 | 0.0020 | -1.4583 |
| TTC37 | 0.1230 | 6.1690 | 3.3526 | 0.0009 | 0.0020 | -1.4587 |
| RBM14 | -0.0453 | 6.4017 | -3.3525 | 0.0009 | 0.0020 | -1.4591 |
| INO80D | -0.1799 | 4.4341 | -3.3524 | 0.0009 | 0.0020 | -1.4593 |
| C1orf27 | 0.0969 | 6.4278 | 3.3502 | 0.0009 | 0.0020 | -1.4663 |
| GREB1L | 0.3794 | 4.1271 | 3.3501 | 0.0009 | 0.0020 | -1.4666 |
| DHX57 | -0.1126 | 5.2793 | -3.3493 | 0.0009 | 0.0020 | -1.4692 |
| PGAP2 | 0.0837 | 6.3872 | 3.3489 | 0.0009 | 0.0020 | -1.4706 |
| CHMP1B | 0.0790 | 6.6254 | 3.3484 | 0.0009 | 0.0020 | -1.4724 |
| CHRDL1 | -0.5912 | 1.7652 | -3.3480 | 0.0009 | 0.0020 | -1.4736 |
| NSD1 | -0.1203 | 5.5446 | -3.3477 | 0.0009 | 0.0020 | -1.4745 |
| CEP192 | -0.2102 | 4.7189 | -3.3473 | 0.0009 | 0.0020 | -1.4759 |
| EPB41L4A | -0.4164 | 2.6610 | -3.3464 | 0.0009 | 0.0020 | -1.4787 |
| FEV | -0.1879 | 0.2697 | -3.3452 | 0.0009 | 0.0020 | -1.4826 |
| ADAM11 | -0.4060 | 2.1018 | -3.3446 | 0.0009 | 0.0021 | -1.4844 |
| ADCY1 | 0.5925 | 3.4361 | 3.3441 | 0.0009 | 0.0021 | -1.4862 |
| PELO | -0.1272 | 5.9830 | -3.3439 | 0.0009 | 0.0021 | -1.4866 |
| PPRC1 | -0.1118 | 5.9218 | -3.3438 | 0.0009 | 0.0021 | -1.4871 |
| HPGDS | -0.4119 | 2.9542 | -3.3437 | 0.0009 | 0.0021 | -1.4874 |
| SNRPN | -0.2449 | 6.4484 | -3.3391 | 0.0009 | 0.0021 | -1.5021 |
| SLC6A5 | -0.1075 | 0.0717 | -3.3381 | 0.0009 | 0.0021 | -1.5053 |
| C6orf10 | -0.1015 | 0.1117 | -3.3379 | 0.0009 | 0.0021 | -1.5059 |
| ETV7 | -0.4034 | 4.5767 | -3.3376 | 0.0009 | 0.0021 | -1.5070 |
| ICOS | -0.4805 | 2.4653 | -3.3371 | 0.0009 | 0.0021 | -1.5087 |
| SPDYE2 | -0.2535 | 0.8398 | -3.3345 | 0.0009 | 0.0021 | -1.5169 |
| HSPB3 | -0.3879 | 0.4914 | -3.3345 | 0.0009 | 0.0021 | -1.5170 |

| CDH13 | 0.2516 | 4.3647 | 3.3328 | 0.0009 | 0.0021 | -1.5225 |
| --- | --- | --- | --- | --- | --- | --- |
| PSPC1 | -0.0841 | 6.1324 | -3.3326 | 0.0009 | 0.0021 | -1.5230 |
| CRCT1 | -0.3926 | 0.5254 | -3.3325 | 0.0009 | 0.0021 | -1.5234 |
| MAGEB1 | -0.5604 | 0.7889 | -3.3322 | 0.0009 | 0.0021 | -1.5244 |
| IFFO1 | 0.1139 | 5.7896 | 3.3319 | 0.0009 | 0.0021 | -1.5253 |
| DNAJC3 | 0.0830 | 6.7805 | 3.3309 | 0.0010 | 0.0021 | -1.5286 |
| CHPF2 | -0.0741 | 6.5066 | -3.3304 | 0.0010 | 0.0021 | -1.5301 |
| TBL1XR1 | -0.0777 | 6.2228 | -3.3303 | 0.0010 | 0.0022 | -1.5304 |
| GPR137B | -0.2570 | 5.9456 | -3.3302 | 0.0010 | 0.0022 | -1.5309 |
| CNTFR | -0.7738 | 3.7703 | -3.3299 | 0.0010 | 0.0022 | -1.5319 |
| INSM1 | -0.3348 | 0.4274 | -3.3298 | 0.0010 | 0.0022 | -1.5322 |
| USP5 | 0.0587 | 6.8725 | 3.3280 | 0.0010 | 0.0022 | -1.5378 |
| RNF2 | -0.1821 | 5.4268 | -3.3280 | 0.0010 | 0.0022 | -1.5378 |
| MRPS2 | 0.0808 | 6.8404 | 3.3278 | 0.0010 | 0.0022 | -1.5384 |
| GCM1 | -0.3567 | 0.8453 | -3.3276 | 0.0010 | 0.0022 | -1.5393 |
| SYNGR1 | -0.5156 | 4.5141 | -3.3270 | 0.0010 | 0.0022 | -1.5410 |
| RNF208 | 0.1471 | 6.2730 | 3.3269 | 0.0010 | 0.0022 | -1.5414 |
| NAT10 | -0.0573 | 6.2817 | -3.3268 | 0.0010 | 0.0022 | -1.5418 |
| RNF170 | 0.1503 | 5.7210 | 3.3267 | 0.0010 | 0.0022 | -1.5422 |
| EEF1G | -0.3137 | 2.5766 | -3.3256 | 0.0010 | 0.0022 | -1.5457 |
| RPUSD2 | 0.0878 | 5.9732 | 3.3248 | 0.0010 | 0.0022 | -1.5482 |
| UBE2W | 0.1057 | 5.5818 | 3.3244 | 0.0010 | 0.0022 | -1.5495 |
| PTGS2 | -0.4276 | 2.0957 | -3.3228 | 0.0010 | 0.0022 | -1.5545 |
| ZFX | -0.1588 | 5.4353 | -3.3225 | 0.0010 | 0.0022 | -1.5557 |
| ATG2A | 0.0913 | 6.2024 | 3.3219 | 0.0010 | 0.0022 | -1.5576 |
| NPPC | -0.4067 | 1.0589 | -3.3217 | 0.0010 | 0.0022 | -1.5582 |
| ZNF749 | -0.2279 | 4.3047 | -3.3214 | 0.0010 | 0.0022 | -1.5590 |
| GBE1 | 0.1484 | 6.4881 | 3.3211 | 0.0010 | 0.0022 | -1.5599 |
| PCMTD2 | 0.1056 | 6.2525 | 3.3211 | 0.0010 | 0.0022 | -1.5600 |
| SYNJ2BP | 0.1074 | 6.0227 | 3.3209 | 0.0010 | 0.0022 | -1.5607 |
| RIMS2 | -0.3421 | 0.6112 | -3.3195 | 0.0010 | 0.0022 | -1.5650 |
| ZNF493 | -0.2902 | 3.2249 | -3.3192 | 0.0010 | 0.0022 | -1.5660 |
| EGLN1 | 0.0830 | 6.6189 | 3.3187 | 0.0010 | 0.0022 | -1.5677 |
| GON4L | -0.1391 | 5.2742 | -3.3161 | 0.0010 | 0.0022 | -1.5760 |
| RASGRP1 | -0.3854 | 2.7952 | -3.3159 | 0.0010 | 0.0022 | -1.5768 |
| CNKSR2 | 0.5572 | 3.1475 | 3.3157 | 0.0010 | 0.0023 | -1.5773 |
| TDP1 | -0.1290 | 5.0498 | -3.3156 | 0.0010 | 0.0023 | -1.5776 |
| ABCB1 | 0.4253 | 5.9849 | 3.3148 | 0.0010 | 0.0023 | -1.5802 |
| TYW1 | 0.0800 | 5.9430 | 3.3142 | 0.0010 | 0.0023 | -1.5821 |
| REG1B | -0.6174 | 0.8626 | -3.3142 | 0.0010 | 0.0023 | -1.5822 |
| RPL8 | 0.0704 | 7.9144 | 3.3136 | 0.0010 | 0.0023 | -1.5841 |
| UBXN4 | 0.0707 | 6.8587 | 3.3135 | 0.0010 | 0.0023 | -1.5843 |
| CXCL14 | -0.6770 | 3.0225 | -3.3129 | 0.0010 | 0.0023 | -1.5861 |
| HCRT | -0.3534 | 0.7359 | -3.3114 | 0.0010 | 0.0023 | -1.5912 |
| COL9A3 | -0.5191 | 3.7176 | -3.3077 | 0.0010 | 0.0023 | -1.6029 |
| PGPEP1 | 0.1133 | 6.3345 | 3.3075 | 0.0010 | 0.0023 | -1.6034 |
| BMP2K | -0.2239 | 3.5095 | -3.3072 | 0.0010 | 0.0023 | -1.6044 |
| CNTN2 | -0.2392 | 0.6229 | -3.3070 | 0.0010 | 0.0023 | -1.6052 |
| PHKB | 0.1192 | 5.8435 | 3.3060 | 0.0010 | 0.0023 | -1.6085 |
| TP53TG5 | -0.2592 | 1.6977 | -3.3049 | 0.0010 | 0.0023 | -1.6118 |
| MPHOSPH8 | 0.0727 | 6.1665 | 3.3041 | 0.0010 | 0.0023 | -1.6143 |
| BRD2 | 0.0616 | 6.8785 | 3.3041 | 0.0010 | 0.0023 | -1.6143 |
| DDX31 | -0.1071 | 5.2464 | -3.3031 | 0.0010 | 0.0023 | -1.6177 |

| LRRC47 | 0.0647 | 6.5311 | 3.3024 | 0.0011 | 0.0023 | -1.6197 |
| --- | --- | --- | --- | --- | --- | --- |
| CARS | -0.0968 | 5.8942 | -3.3019 | 0.0011 | 0.0024 | -1.6214 |
| DAG1 | 0.0686 | 6.8556 | 3.3006 | 0.0011 | 0.0024 | -1.6255 |
| MYO1A | -0.5408 | 3.3557 | -3.3005 | 0.0011 | 0.0024 | -1.6259 |
| UFSP2 | 0.0916 | 6.0113 | 3.2999 | 0.0011 | 0.0024 | -1.6278 |
| KLHL24 | 0.1257 | 5.9085 | 3.2998 | 0.0011 | 0.0024 | -1.6280 |
| TEX261 | 0.0531 | 6.7705 | 3.2996 | 0.0011 | 0.0024 | -1.6286 |
| CCDC90B | 0.0821 | 5.8680 | 3.2993 | 0.0011 | 0.0024 | -1.6296 |
| PHACTR1 | -0.2879 | 2.6253 | -3.2982 | 0.0011 | 0.0024 | -1.6331 |
| CRISP1 | -0.1570 | 0.2559 | -3.2979 | 0.0011 | 0.0024 | -1.6342 |
| TNS3 | 0.1188 | 6.4712 | 3.2977 | 0.0011 | 0.0024 | -1.6348 |
| ATP6V0A1 | 0.0977 | 6.3076 | 3.2958 | 0.0011 | 0.0024 | -1.6409 |
| PUS7 | -0.1486 | 5.5253 | -3.2957 | 0.0011 | 0.0024 | -1.6410 |
| TCEAL2 | -0.4015 | 0.7786 | -3.2950 | 0.0011 | 0.0024 | -1.6432 |
| SLC7A5 | -0.3091 | 5.5893 | -3.2946 | 0.0011 | 0.0024 | -1.6447 |
| LSM12 | -0.1145 | 5.3820 | -3.2926 | 0.0011 | 0.0024 | -1.6510 |
| CYP26A1 | 0.6191 | 2.6270 | 3.2925 | 0.0011 | 0.0024 | -1.6514 |
| MUTYH | -0.1212 | 5.5230 | -3.2906 | 0.0011 | 0.0024 | -1.6574 |
| SLC12A8 | -0.3573 | 5.2231 | -3.2905 | 0.0011 | 0.0024 | -1.6576 |
| POLR2J | 0.0770 | 7.0312 | 3.2901 | 0.0011 | 0.0024 | -1.6590 |
| ICAM1 | -0.1849 | 6.4649 | -3.2895 | 0.0011 | 0.0024 | -1.6608 |
| ERBB4 | -0.2121 | 0.2437 | -3.2890 | 0.0011 | 0.0024 | -1.6626 |
| ASXL3 | 0.3600 | 1.0788 | 3.2886 | 0.0011 | 0.0025 | -1.6636 |
| DACH1 | -0.3350 | 1.6625 | -3.2864 | 0.0011 | 0.0025 | -1.6706 |
| HAT1 | -0.1012 | 5.8093 | -3.2863 | 0.0011 | 0.0025 | -1.6709 |
| PLD1 | -0.2847 | 5.1564 | -3.2856 | 0.0011 | 0.0025 | -1.6733 |
| GABRA4 | -0.1205 | 0.0705 | -3.2849 | 0.0011 | 0.0025 | -1.6756 |
| PKP1 | -0.3822 | 1.1493 | -3.2847 | 0.0011 | 0.0025 | -1.6760 |
| DNAH2 | -0.2410 | 0.9071 | -3.2838 | 0.0011 | 0.0025 | -1.6790 |
| GRIN2B | 0.2641 | 0.6579 | 3.2837 | 0.0011 | 0.0025 | -1.6791 |
| STAM | -0.0982 | 5.8598 | -3.2834 | 0.0011 | 0.0025 | -1.6803 |
| PGC | -0.9246 | 3.0811 | -3.2821 | 0.0011 | 0.0025 | -1.6845 |
| FMOD | -0.4637 | 5.0520 | -3.2814 | 0.0011 | 0.0025 | -1.6866 |
| ISG20L2 | -0.0748 | 6.2414 | -3.2806 | 0.0011 | 0.0025 | -1.6892 |
| DES | -0.5742 | 2.5713 | -3.2805 | 0.0011 | 0.0025 | -1.6894 |
| ZNF12 | -0.1176 | 5.6226 | -3.2801 | 0.0011 | 0.0025 | -1.6907 |
| COMMD10 | 0.1019 | 6.1586 | 3.2790 | 0.0011 | 0.0025 | -1.6940 |
| PMP2 | -0.2249 | 0.2158 | -3.2775 | 0.0011 | 0.0025 | -1.6990 |
| STK38 | -0.0793 | 6.4716 | -3.2772 | 0.0011 | 0.0025 | -1.7000 |
| TMSB10 | -0.0761 | 7.9434 | -3.2761 | 0.0012 | 0.0026 | -1.7033 |
| CHST3 | -0.3352 | 4.0153 | -3.2758 | 0.0012 | 0.0026 | -1.7042 |
| HTR6 | -0.1012 | 0.1432 | -3.2757 | 0.0012 | 0.0026 | -1.7048 |
| FAM173A | 0.1194 | 6.2412 | 3.2741 | 0.0012 | 0.0026 | -1.7096 |
| RPS9 | 0.0669 | 7.5405 | 3.2737 | 0.0012 | 0.0026 | -1.7110 |
| VAV1 | -0.3048 | 4.4148 | -3.2722 | 0.0012 | 0.0026 | -1.7157 |
| RABEP1 | 0.0961 | 6.0837 | 3.2721 | 0.0012 | 0.0026 | -1.7160 |
| KIAA0895 | -0.3481 | 4.3331 | -3.2718 | 0.0012 | 0.0026 | -1.7170 |
| PSIP1 | -0.1286 | 5.9519 | -3.2713 | 0.0012 | 0.0026 | -1.7185 |
| ACSBG2 | -0.2170 | 0.6847 | -3.2693 | 0.0012 | 0.0026 | -1.7248 |
| KLF13 | 0.1516 | 6.1506 | 3.2691 | 0.0012 | 0.0026 | -1.7254 |
| ASPA | 0.4234 | 3.3524 | 3.2684 | 0.0012 | 0.0026 | -1.7276 |
| YIPF4 | 0.0783 | 5.9405 | 3.2682 | 0.0012 | 0.0026 | -1.7283 |
| HLCS | 0.1116 | 5.6236 | 3.2674 | 0.0012 | 0.0026 | -1.7308 |

| CST3 | 0.0798 | 7.3705 | 3.2663 | 0.0012 | 0.0026 | -1.7342 |
| --- | --- | --- | --- | --- | --- | --- |
| TAF4 | -0.1339 | 5.1943 | -3.2656 | 0.0012 | 0.0026 | -1.7365 |
| ZNF34 | 0.1266 | 5.5380 | 3.2656 | 0.0012 | 0.0026 | -1.7366 |
| TUBB6 | -0.1976 | 5.5839 | -3.2651 | 0.0012 | 0.0026 | -1.7380 |
| UNC50 | 0.0533 | 6.5237 | 3.2647 | 0.0012 | 0.0026 | -1.7392 |
| WDR7 | 0.1530 | 4.5063 | 3.2646 | 0.0012 | 0.0026 | -1.7397 |
| HABP4 | 0.1757 | 5.7044 | 3.2644 | 0.0012 | 0.0026 | -1.7402 |
| FXYD1 | 0.5694 | 5.3535 | 3.2639 | 0.0012 | 0.0027 | -1.7420 |
| FSCN1 | -0.1499 | 6.2234 | -3.2638 | 0.0012 | 0.0027 | -1.7422 |
| TBC1D15 | 0.0705 | 5.8726 | 3.2637 | 0.0012 | 0.0027 | -1.7424 |
| HAS2 | -0.5131 | 1.9397 | -3.2621 | 0.0012 | 0.0027 | -1.7475 |
| MRPS7 | 0.0662 | 6.8315 | 3.2616 | 0.0012 | 0.0027 | -1.7490 |
| ITGAL | 0.2088 | 5.8340 | 3.2609 | 0.0012 | 0.0027 | -1.7514 |
| GCKR | 0.2998 | 6.4373 | 3.2602 | 0.0012 | 0.0027 | -1.7536 |
| TJP1 | 0.1200 | 6.0543 | 3.2599 | 0.0012 | 0.0027 | -1.7545 |
| TJP3 | -0.4522 | 5.2883 | -3.2587 | 0.0012 | 0.0027 | -1.7581 |
| GPR3 | -0.2937 | 2.9678 | -3.2585 | 0.0012 | 0.0027 | -1.7588 |
| DYNC1H1 | -0.0593 | 6.5460 | -3.2585 | 0.0012 | 0.0027 | -1.7588 |
| 43895.0000 | 0.0695 | 6.2322 | 3.2581 | 0.0012 | 0.0027 | -1.7602 |
| ROBO1 | -0.3627 | 5.6968 | -3.2577 | 0.0012 | 0.0027 | -1.7616 |
| TBX5 | -0.1908 | 0.2164 | -3.2574 | 0.0012 | 0.0027 | -1.7622 |
| KRT36 | -0.2336 | 0.6063 | -3.2564 | 0.0012 | 0.0027 | -1.7656 |
| R3HCC1 | 0.0831 | 6.4456 | 3.2563 | 0.0012 | 0.0027 | -1.7658 |
| ARL17A | -0.1968 | 1.4985 | -3.2558 | 0.0012 | 0.0027 | -1.7673 |
| XPOT | -0.0942 | 6.4006 | -3.2550 | 0.0012 | 0.0027 | -1.7699 |
| CCND1 | 0.1762 | 6.7835 | 3.2539 | 0.0012 | 0.0027 | -1.7733 |
| CD8B | -0.4923 | 3.5129 | -3.2531 | 0.0012 | 0.0027 | -1.7759 |
| DAPP1 | -0.3732 | 2.7679 | -3.2531 | 0.0012 | 0.0027 | -1.7760 |
| ATP5L | 0.0631 | 7.1289 | 3.2528 | 0.0012 | 0.0027 | -1.7768 |
| DGKI | -0.2131 | 0.9907 | -3.2523 | 0.0012 | 0.0027 | -1.7783 |
| GTF3C3 | -0.0950 | 5.5337 | -3.2523 | 0.0012 | 0.0027 | -1.7784 |
| GIP | -0.4622 | 0.7677 | -3.2501 | 0.0013 | 0.0028 | -1.7852 |
| ING2 | -0.1043 | 5.8880 | -3.2498 | 0.0013 | 0.0028 | -1.7862 |
| BANF1 | 0.0552 | 7.2997 | 3.2497 | 0.0013 | 0.0028 | -1.7867 |
| ATM | -0.1805 | 4.6620 | -3.2474 | 0.0013 | 0.0028 | -1.7939 |
| SSFA2 | 0.1298 | 6.2767 | 3.2473 | 0.0013 | 0.0028 | -1.7940 |
| PF4 | -0.4890 | 1.6757 | -3.2472 | 0.0013 | 0.0028 | -1.7945 |
| ZBTB33 | 0.1183 | 6.2163 | 3.2462 | 0.0013 | 0.0028 | -1.7974 |
| AKAP8L | -0.0722 | 6.2708 | -3.2444 | 0.0013 | 0.0028 | -1.8031 |
| TAOK1 | -0.1345 | 5.3804 | -3.2442 | 0.0013 | 0.0028 | -1.8037 |
| GTPBP8 | -0.1118 | 4.9168 | -3.2434 | 0.0013 | 0.0028 | -1.8063 |
| MYL4 | -0.2686 | 2.4678 | -3.2425 | 0.0013 | 0.0028 | -1.8092 |
| PPCS | 0.0585 | 6.7606 | 3.2422 | 0.0013 | 0.0028 | -1.8100 |
| PPP1R7 | 0.0644 | 6.6806 | 3.2417 | 0.0013 | 0.0028 | -1.8116 |
| SCPEP1 | -0.1937 | 6.3521 | -3.2412 | 0.0013 | 0.0028 | -1.8133 |
| MRPS34 | 0.0681 | 7.2754 | 3.2411 | 0.0013 | 0.0028 | -1.8135 |
| SAV1 | 0.1488 | 5.5348 | 3.2410 | 0.0013 | 0.0028 | -1.8139 |
| UROS | 0.1029 | 6.1803 | 3.2409 | 0.0013 | 0.0028 | -1.8143 |
| DKC1 | -0.0767 | 6.4380 | -3.2404 | 0.0013 | 0.0029 | -1.8159 |
| HNRNPC | 0.0355 | 7.0943 | 3.2397 | 0.0013 | 0.0029 | -1.8178 |
| RPL24 | 0.0657 | 7.5695 | 3.2390 | 0.0013 | 0.0029 | -1.8201 |
| CHEK2 | -0.2066 | 5.3638 | -3.2384 | 0.0013 | 0.0029 | -1.8221 |
| CEBPE | -0.2473 | 0.7951 | -3.2383 | 0.0013 | 0.0029 | -1.8223 |

| PSMD14 | -0.0775 | 6.2572 | -3.2378 | 0.0013 | 0.0029 | -1.8239 |
| --- | --- | --- | --- | --- | --- | --- |
| DCUN1D4 | 0.0937 | 6.0031 | 3.2369 | 0.0013 | 0.0029 | -1.8266 |
| SF1 | 0.0340 | 6.9083 | 3.2366 | 0.0013 | 0.0029 | -1.8277 |
| NPHS1 | -0.3255 | 0.5893 | -3.2359 | 0.0013 | 0.0029 | -1.8298 |
| CFD | -0.2495 | 5.5962 | -3.2355 | 0.0013 | 0.0029 | -1.8310 |
| RTN2 | -0.3072 | 5.1785 | -3.2351 | 0.0013 | 0.0029 | -1.8324 |
| CLEC4A | -0.2311 | 4.4415 | -3.2327 | 0.0013 | 0.0029 | -1.8399 |
| KCNJ4 | 0.6395 | 3.9888 | 3.2323 | 0.0013 | 0.0029 | -1.8412 |
| HEMK1 | 0.1141 | 4.8775 | 3.2317 | 0.0013 | 0.0029 | -1.8430 |
| VENTX | -0.3246 | 2.3084 | -3.2305 | 0.0013 | 0.0029 | -1.8468 |
| PPP2CB | 0.0936 | 6.4892 | 3.2299 | 0.0013 | 0.0029 | -1.8486 |
| UGT2B28 | -0.4518 | 1.4279 | -3.2298 | 0.0013 | 0.0029 | -1.8488 |
| R3HDM2 | 0.0749 | 6.1846 | 3.2294 | 0.0014 | 0.0029 | -1.8501 |
| ANKS1A | -0.1251 | 5.5514 | -3.2289 | 0.0014 | 0.0030 | -1.8516 |
| MAP3K10 | -0.1544 | 5.2583 | -3.2287 | 0.0014 | 0.0030 | -1.8522 |
| CD37 | -0.2525 | 5.0417 | -3.2284 | 0.0014 | 0.0030 | -1.8531 |
| FRG1 | 0.0828 | 6.4709 | 3.2278 | 0.0014 | 0.0030 | -1.8551 |
| RPL13 | 0.0796 | 7.4638 | 3.2275 | 0.0014 | 0.0030 | -1.8559 |
| TM2D3 | 0.1097 | 5.6875 | 3.2274 | 0.0014 | 0.0030 | -1.8565 |
| TOM1 | 0.0762 | 6.7900 | 3.2262 | 0.0014 | 0.0030 | -1.8601 |
| CLK4 | 0.1303 | 5.0666 | 3.2254 | 0.0014 | 0.0030 | -1.8626 |
| FLT1 | 0.1704 | 5.5269 | 3.2250 | 0.0014 | 0.0030 | -1.8638 |
| NEFM | -0.2304 | 0.3188 | -3.2243 | 0.0014 | 0.0030 | -1.8661 |
| FFAR3 | -0.2363 | 0.5225 | -3.2237 | 0.0014 | 0.0030 | -1.8679 |
| CIDEC | -0.6160 | 3.7700 | -3.2235 | 0.0014 | 0.0030 | -1.8684 |
| RYR3 | -0.1829 | 1.1787 | -3.2232 | 0.0014 | 0.0030 | -1.8694 |
| S100A4 | -0.1854 | 6.5686 | -3.2231 | 0.0014 | 0.0030 | -1.8697 |
| PCDHA10 | -0.2577 | 0.5452 | -3.2216 | 0.0014 | 0.0030 | -1.8745 |
| TMEM109 | 0.0539 | 7.0371 | 3.2212 | 0.0014 | 0.0030 | -1.8758 |
| U2AF2 | -0.0368 | 6.9439 | -3.2203 | 0.0014 | 0.0030 | -1.8783 |
| PAFAH1B2 | -0.0601 | 6.3780 | -3.2199 | 0.0014 | 0.0030 | -1.8797 |
| DCAF15 | -0.0807 | 6.1897 | -3.2197 | 0.0014 | 0.0030 | -1.8803 |
| C9orf116 | -0.2950 | 4.1653 | -3.2192 | 0.0014 | 0.0030 | -1.8819 |
| PDGFRA | -0.6103 | 3.5555 | -3.2182 | 0.0014 | 0.0031 | -1.8849 |
| DNASE2 | 0.1068 | 6.9064 | 3.2181 | 0.0014 | 0.0031 | -1.8854 |
| DNAJC17 | 0.1091 | 5.6122 | 3.2167 | 0.0014 | 0.0031 | -1.8898 |
| LILRB1 | -0.3303 | 3.7431 | -3.2164 | 0.0014 | 0.0031 | -1.8907 |
| ALPK3 | -0.4238 | 4.4742 | -3.2160 | 0.0014 | 0.0031 | -1.8919 |
| RHAG | -0.2619 | 0.3174 | -3.2159 | 0.0014 | 0.0031 | -1.8922 |
| XAB2 | 0.0677 | 6.5847 | 3.2156 | 0.0014 | 0.0031 | -1.8932 |
| CELSR2 | -0.3042 | 4.2835 | -3.2149 | 0.0014 | 0.0031 | -1.8952 |
| PEX5L | -0.1417 | 0.4598 | -3.2148 | 0.0014 | 0.0031 | -1.8956 |
| ZNF415 | -0.4053 | 2.7096 | -3.2138 | 0.0014 | 0.0031 | -1.8987 |
| C3orf36 | 0.4072 | 1.7787 | 3.2138 | 0.0014 | 0.0031 | -1.8989 |
| HTATIP2 | 0.1048 | 7.0284 | 3.2133 | 0.0014 | 0.0031 | -1.9003 |
| NUP62CL | -0.4595 | 3.0735 | -3.2116 | 0.0014 | 0.0031 | -1.9057 |
| EDIL3 | -0.3674 | 4.1635 | -3.2115 | 0.0014 | 0.0031 | -1.9058 |
| TSC2 | 0.1162 | 6.1124 | 3.2115 | 0.0014 | 0.0031 | -1.9058 |
| THOC2 | -0.1102 | 5.7051 | -3.2107 | 0.0014 | 0.0031 | -1.9083 |
| UBXN1 | 0.0617 | 7.0283 | 3.2103 | 0.0014 | 0.0031 | -1.9095 |
| LSAMP | -0.4242 | 1.2387 | -3.2095 | 0.0014 | 0.0031 | -1.9119 |
| P2RY1 | -0.2705 | 3.1623 | -3.2085 | 0.0014 | 0.0031 | -1.9153 |
| PPFIBP2 | 0.1268 | 5.7643 | 3.2084 | 0.0014 | 0.0031 | -1.9154 |

| HIP1R | -0.1043 | 6.3121 | -3.2081 | 0.0015 | 0.0031 | -1.9163 |
| --- | --- | --- | --- | --- | --- | --- |
| PSMD12 | 0.0562 | 6.5360 | 3.2079 | 0.0015 | 0.0031 | -1.9169 |
| ETF1 | 0.0513 | 6.7646 | 3.2079 | 0.0015 | 0.0031 | -1.9170 |
| LYPD3 | -0.3622 | 3.4739 | -3.2071 | 0.0015 | 0.0032 | -1.9194 |
| PSMC4 | 0.0553 | 7.1699 | 3.2067 | 0.0015 | 0.0032 | -1.9208 |
| DUSP5 | -0.2230 | 5.9359 | -3.2047 | 0.0015 | 0.0032 | -1.9270 |
| REPIN1 | 0.0613 | 6.9946 | 3.2045 | 0.0015 | 0.0032 | -1.9276 |
| SCAMP2 | 0.0460 | 6.7477 | 3.2044 | 0.0015 | 0.0032 | -1.9278 |
| KCNK12 | -0.2196 | 0.4103 | -3.2041 | 0.0015 | 0.0032 | -1.9289 |
| KCNA4 | -0.0900 | 0.0590 | -3.2032 | 0.0015 | 0.0032 | -1.9315 |
| USP19 | 0.0595 | 6.4469 | 3.2029 | 0.0015 | 0.0032 | -1.9326 |
| GABARAP | 0.0637 | 6.8261 | 3.2028 | 0.0015 | 0.0032 | -1.9327 |
| CNNM3 | 0.0845 | 6.4706 | 3.2017 | 0.0015 | 0.0032 | -1.9364 |
| LEP | -0.2591 | 0.3980 | -3.2006 | 0.0015 | 0.0032 | -1.9396 |
| FAP | -0.4896 | 2.7353 | -3.2004 | 0.0015 | 0.0032 | -1.9402 |
| PIK3R3 | 0.1571 | 5.1259 | 3.2002 | 0.0015 | 0.0032 | -1.9410 |
| PNMA3 | -0.5996 | 3.1596 | -3.2001 | 0.0015 | 0.0032 | -1.9411 |
| P4HA1 | 0.1627 | 6.7246 | 3.1993 | 0.0015 | 0.0032 | -1.9436 |
| ARL4A | -0.1967 | 5.7304 | -3.1976 | 0.0015 | 0.0032 | -1.9489 |
| RPL12 | 0.0683 | 7.5793 | 3.1970 | 0.0015 | 0.0033 | -1.9509 |
| PDE8B | -0.2389 | 3.3463 | -3.1964 | 0.0015 | 0.0033 | -1.9526 |
| HSPA8 | 0.0654 | 7.5390 | 3.1963 | 0.0015 | 0.0033 | -1.9528 |
| BST2 | 0.1438 | 7.4331 | 3.1960 | 0.0015 | 0.0033 | -1.9539 |
| TNFSF13 | -0.2695 | 4.6479 | -3.1960 | 0.0015 | 0.0033 | -1.9539 |
| TIMM23 | 0.0499 | 7.1211 | 3.1959 | 0.0015 | 0.0033 | -1.9541 |
| VSX1 | -0.4122 | 1.4671 | -3.1955 | 0.0015 | 0.0033 | -1.9556 |
| TRIOBP | -0.1320 | 5.2799 | -3.1944 | 0.0015 | 0.0033 | -1.9589 |
| PDC | -0.1982 | 0.4967 | -3.1941 | 0.0015 | 0.0033 | -1.9599 |
| SIRPA | -0.1303 | 6.5309 | -3.1925 | 0.0015 | 0.0033 | -1.9646 |
| TRIM34 | -0.2540 | 1.6852 | -3.1919 | 0.0015 | 0.0033 | -1.9667 |
| SQSTM1 | 0.0892 | 7.3442 | 3.1914 | 0.0015 | 0.0033 | -1.9681 |
| ERAL1 | 0.0530 | 6.9150 | 3.1908 | 0.0015 | 0.0033 | -1.9699 |
| RUNX1T1 | 0.2947 | 1.8728 | 3.1907 | 0.0015 | 0.0033 | -1.9703 |
| RPL4 | 0.0584 | 7.5973 | 3.1905 | 0.0015 | 0.0033 | -1.9708 |
| ZNF20 | -0.1892 | 1.7968 | -3.1904 | 0.0015 | 0.0033 | -1.9713 |
| PRDM2 | -0.1411 | 4.8591 | -3.1903 | 0.0015 | 0.0033 | -1.9716 |
| SYNPO2L | -0.1776 | 0.4107 | -3.1900 | 0.0015 | 0.0033 | -1.9723 |
| PPP6C | 0.0556 | 6.4587 | 3.1898 | 0.0015 | 0.0033 | -1.9729 |
| XRCC4 | -0.1440 | 5.2509 | -3.1893 | 0.0015 | 0.0033 | -1.9745 |
| PSMB8 | 0.1076 | 7.1174 | 3.1890 | 0.0015 | 0.0033 | -1.9756 |
| TFDP1 | -0.1186 | 6.3562 | -3.1874 | 0.0016 | 0.0033 | -1.9804 |
| RYR2 | -0.4136 | 1.1215 | -3.1869 | 0.0016 | 0.0034 | -1.9819 |
| PRDX4 | 0.0748 | 7.2845 | 3.1863 | 0.0016 | 0.0034 | -1.9838 |
| FCN2 | 0.6541 | 3.3811 | 3.1861 | 0.0016 | 0.0034 | -1.9843 |
| CSAG2 | -0.6972 | 1.5522 | -3.1856 | 0.0016 | 0.0034 | -1.9858 |
| XCL1 | -0.4643 | 3.2984 | -3.1854 | 0.0016 | 0.0034 | -1.9866 |
| MEF2D | 0.0828 | 6.4064 | 3.1847 | 0.0016 | 0.0034 | -1.9886 |
| CTDP1 | -0.0862 | 5.6447 | -3.1840 | 0.0016 | 0.0034 | -1.9909 |
| ARRB1 | -0.2461 | 5.2305 | -3.1835 | 0.0016 | 0.0034 | -1.9924 |
| ZMYM2 | -0.1364 | 5.3363 | -3.1834 | 0.0016 | 0.0034 | -1.9928 |
| SMAD6 | -0.2814 | 4.1023 | -3.1827 | 0.0016 | 0.0034 | -1.9948 |
| PPCDC | -0.1329 | 5.4834 | -3.1799 | 0.0016 | 0.0034 | -2.0035 |
| RASSF7 | -0.1022 | 6.8177 | -3.1798 | 0.0016 | 0.0034 | -2.0040 |

| CCDC134 | -0.1472 | 5.2939 | -3.1791 | 0.0016 | 0.0034 | -2.0060 |
| --- | --- | --- | --- | --- | --- | --- |
| DSG3 | -0.3077 | 0.3843 | -3.1782 | 0.0016 | 0.0034 | -2.0087 |
| COTL1 | -0.1999 | 5.9339 | -3.1779 | 0.0016 | 0.0034 | -2.0096 |
| SGCE | -0.3425 | 5.7687 | -3.1764 | 0.0016 | 0.0035 | -2.0143 |
| TRIM62 | -0.1765 | 4.5346 | -3.1753 | 0.0016 | 0.0035 | -2.0176 |
| GPR173 | -0.3132 | 1.6773 | -3.1745 | 0.0016 | 0.0035 | -2.0201 |
| NACA2 | -0.3122 | 2.5325 | -3.1741 | 0.0016 | 0.0035 | -2.0213 |
| NUP50 | -0.0892 | 6.0311 | -3.1738 | 0.0016 | 0.0035 | -2.0224 |
| GTPBP3 | -0.0980 | 5.7631 | -3.1727 | 0.0016 | 0.0035 | -2.0256 |
| C14orf93 | -0.1047 | 5.3613 | -3.1718 | 0.0016 | 0.0035 | -2.0285 |
| BRD3 | -0.1673 | 5.4789 | -3.1710 | 0.0016 | 0.0035 | -2.0308 |
| P2RY4 | -0.3084 | 0.6294 | -3.1705 | 0.0016 | 0.0035 | -2.0323 |
| SH3BGR | 0.1633 | 5.4071 | 3.1705 | 0.0016 | 0.0035 | -2.0323 |
| EDEM2 | 0.0526 | 6.9157 | 3.1700 | 0.0017 | 0.0035 | -2.0341 |
| SNRPA | -0.0640 | 6.8000 | -3.1693 | 0.0017 | 0.0035 | -2.0361 |
| URB2 | -0.1580 | 5.0245 | -3.1686 | 0.0017 | 0.0035 | -2.0381 |
| RASL11B | -0.4984 | 2.0786 | -3.1680 | 0.0017 | 0.0036 | -2.0401 |
| ACSM1 | 0.5728 | 5.5522 | 3.1678 | 0.0017 | 0.0036 | -2.0408 |
| STRAP | 0.0477 | 7.0702 | 3.1677 | 0.0017 | 0.0036 | -2.0409 |
| TESK2 | 0.1929 | 5.0371 | 3.1674 | 0.0017 | 0.0036 | -2.0420 |
| PLSCR1 | -0.1665 | 6.0317 | -3.1673 | 0.0017 | 0.0036 | -2.0423 |
| USP20 | 0.0715 | 6.0246 | 3.1671 | 0.0017 | 0.0036 | -2.0428 |
| CDC42EP1 | -0.0851 | 7.1939 | -3.1668 | 0.0017 | 0.0036 | -2.0438 |
| AMPH | -0.3349 | 1.2784 | -3.1667 | 0.0017 | 0.0036 | -2.0441 |
| PANX1 | 0.1315 | 6.1358 | 3.1665 | 0.0017 | 0.0036 | -2.0446 |
| C16orf58 | 0.1915 | 6.4158 | 3.1664 | 0.0017 | 0.0036 | -2.0450 |
| TXNL1 | 0.0983 | 6.1858 | 3.1646 | 0.0017 | 0.0036 | -2.0505 |
| SH3YL1 | -0.3145 | 4.0794 | -3.1635 | 0.0017 | 0.0036 | -2.0540 |
| FMO1 | -0.5996 | 3.2428 | -3.1628 | 0.0017 | 0.0036 | -2.0558 |
| GRIP2 | -0.3468 | 1.1390 | -3.1612 | 0.0017 | 0.0036 | -2.0609 |
| HEXIM1 | -0.0881 | 6.4364 | -3.1602 | 0.0017 | 0.0036 | -2.0639 |
| ABHD14A | 0.1075 | 6.2810 | 3.1593 | 0.0017 | 0.0036 | -2.0666 |
| MCL1 | 0.0507 | 7.3274 | 3.1582 | 0.0017 | 0.0037 | -2.0700 |
| ZNF232 | -0.1298 | 5.3495 | -3.1576 | 0.0017 | 0.0037 | -2.0719 |
| HNRNPAB | 0.0484 | 7.2161 | 3.1572 | 0.0017 | 0.0037 | -2.0730 |
| KITLG | -0.3030 | 4.5504 | -3.1571 | 0.0017 | 0.0037 | -2.0735 |
| EIF4B | 0.0556 | 7.0868 | 3.1557 | 0.0017 | 0.0037 | -2.0777 |
| RPL10L | -0.5783 | 1.3815 | -3.1557 | 0.0017 | 0.0037 | -2.0779 |
| TAF7L | 0.3598 | 1.5169 | 3.1548 | 0.0017 | 0.0037 | -2.0803 |
| C1QTNF3 | 0.3816 | 4.6938 | 3.1548 | 0.0017 | 0.0037 | -2.0805 |
| NADK | 0.0967 | 6.5720 | 3.1544 | 0.0017 | 0.0037 | -2.0818 |
| RBMS2 | -0.2032 | 4.6116 | -3.1541 | 0.0017 | 0.0037 | -2.0826 |
| REC8 | -0.2540 | 4.3433 | -3.1528 | 0.0017 | 0.0037 | -2.0867 |
| LRP4 | -0.4635 | 2.7522 | -3.1525 | 0.0017 | 0.0037 | -2.0874 |
| ANKRD26 | -0.2131 | 3.9961 | -3.1507 | 0.0018 | 0.0037 | -2.0929 |
| HIST1H4I | 0.1596 | 6.4124 | 3.1499 | 0.0018 | 0.0038 | -2.0954 |
| KPNA5 | -0.2364 | 4.0647 | -3.1496 | 0.0018 | 0.0038 | -2.0964 |
| CBX4 | -0.0837 | 6.5760 | -3.1490 | 0.0018 | 0.0038 | -2.0981 |
| TEP1 | -0.1455 | 4.9705 | -3.1476 | 0.0018 | 0.0038 | -2.1024 |
| SLC5A12 | -0.5067 | 1.1772 | -3.1475 | 0.0018 | 0.0038 | -2.1029 |
| NUP85 | -0.0755 | 6.2263 | -3.1473 | 0.0018 | 0.0038 | -2.1033 |
| FAM169A | 0.4509 | 3.4852 | 3.1468 | 0.0018 | 0.0038 | -2.1050 |
| EIF3E | 0.0850 | 7.0874 | 3.1462 | 0.0018 | 0.0038 | -2.1068 |

| C11orf24 | 0.0793 | 6.7142 | 3.1418 | 0.0018 | 0.0039 | -2.1202 |
| --- | --- | --- | --- | --- | --- | --- |
| POU3F3 | -0.1868 | 0.1894 | -3.1416 | 0.0018 | 0.0039 | -2.1207 |
| NFKBIB | 0.0768 | 6.6822 | 3.1415 | 0.0018 | 0.0039 | -2.1211 |
| USP24 | -0.0766 | 5.8857 | -3.1389 | 0.0018 | 0.0039 | -2.1290 |
| GTF2A1 | 0.1054 | 5.8839 | 3.1384 | 0.0018 | 0.0039 | -2.1304 |
| GJC2 | -0.3780 | 2.8459 | -3.1380 | 0.0018 | 0.0039 | -2.1317 |
| TRIO | -0.1728 | 5.2498 | -3.1377 | 0.0018 | 0.0039 | -2.1325 |
| TRPM3 | 0.3449 | 0.9475 | 3.1377 | 0.0018 | 0.0039 | -2.1327 |
| SNN | -0.1009 | 6.0684 | -3.1376 | 0.0018 | 0.0039 | -2.1329 |
| NR4A1 | 0.3152 | 5.2267 | 3.1370 | 0.0018 | 0.0039 | -2.1346 |
| SHMT2 | 0.0697 | 7.1291 | 3.1368 | 0.0018 | 0.0039 | -2.1351 |
| MED12 | -0.1210 | 5.8098 | -3.1367 | 0.0018 | 0.0039 | -2.1355 |
| CEP112 | -0.2083 | 3.9602 | -3.1363 | 0.0018 | 0.0039 | -2.1369 |
| LRIG1 | 0.1336 | 6.0785 | 3.1362 | 0.0018 | 0.0039 | -2.1371 |
| NDUFS5 | 0.0611 | 7.6479 | 3.1343 | 0.0019 | 0.0039 | -2.1428 |
| ADAMTS12 | -0.3498 | 3.2510 | -3.1343 | 0.0019 | 0.0039 | -2.1429 |
| NFE2 | -0.4357 | 1.8798 | -3.1340 | 0.0019 | 0.0039 | -2.1437 |
| HOXD9 | -0.5763 | 3.6231 | -3.1336 | 0.0019 | 0.0039 | -2.1449 |
| SLC12A9 | -0.1144 | 5.8678 | -3.1336 | 0.0019 | 0.0039 | -2.1451 |
| SLAMF8 | -0.3042 | 4.9897 | -3.1336 | 0.0019 | 0.0039 | -2.1451 |
| CD82 | 0.1116 | 6.4455 | 3.1334 | 0.0019 | 0.0039 | -2.1456 |
| PRAMEF11 | 0.2552 | 0.3900 | 3.1326 | 0.0019 | 0.0040 | -2.1481 |
| CTIF | 0.1069 | 5.9691 | 3.1325 | 0.0019 | 0.0040 | -2.1483 |
| KDM4D | -0.2401 | 2.6887 | -3.1322 | 0.0019 | 0.0040 | -2.1492 |
| PSTPIP1 | -0.3536 | 3.8318 | -3.1314 | 0.0019 | 0.0040 | -2.1517 |
| PPA1 | 0.0787 | 6.8639 | 3.1313 | 0.0019 | 0.0040 | -2.1520 |
| VRTN | -0.0652 | 0.0739 | -3.1310 | 0.0019 | 0.0040 | -2.1528 |
| CCND2 | -0.2528 | 4.6099 | -3.1310 | 0.0019 | 0.0040 | -2.1530 |
| ERH | 0.0472 | 7.1795 | 3.1309 | 0.0019 | 0.0040 | -2.1530 |
| HBE1 | -0.5132 | 1.0458 | -3.1307 | 0.0019 | 0.0040 | -2.1536 |
| ATMIN | 0.0738 | 6.0500 | 3.1305 | 0.0019 | 0.0040 | -2.1543 |
| CDON | -0.2628 | 3.6311 | -3.1305 | 0.0019 | 0.0040 | -2.1544 |
| SLC41A3 | -0.0806 | 6.1047 | -3.1298 | 0.0019 | 0.0040 | -2.1566 |
| DNAJC15 | 0.2090 | 5.7029 | 3.1287 | 0.0019 | 0.0040 | -2.1597 |
| HIST1H4D | -0.4785 | 2.4965 | -3.1286 | 0.0019 | 0.0040 | -2.1601 |
| NDEL1 | 0.1234 | 5.5447 | 3.1279 | 0.0019 | 0.0040 | -2.1623 |
| HSPA13 | -0.1188 | 5.9738 | -3.1272 | 0.0019 | 0.0040 | -2.1643 |
| TOM1L1 | 0.1677 | 6.0626 | 3.1269 | 0.0019 | 0.0040 | -2.1652 |
| SEC16A | 0.0694 | 6.5813 | 3.1266 | 0.0019 | 0.0040 | -2.1662 |
| PRSS23 | -0.2543 | 5.1734 | -3.1256 | 0.0019 | 0.0040 | -2.1693 |
| PKP2 | 0.2779 | 5.5669 | 3.1243 | 0.0019 | 0.0041 | -2.1731 |
| CHRD | 0.2222 | 5.9321 | 3.1234 | 0.0019 | 0.0041 | -2.1760 |
| IL19 | -0.0635 | 0.0780 | -3.1230 | 0.0019 | 0.0041 | -2.1770 |
| ZNF80 | -0.2392 | 0.6152 | -3.1230 | 0.0019 | 0.0041 | -2.1772 |
| HSF2 | -0.1329 | 5.4841 | -3.1225 | 0.0019 | 0.0041 | -2.1787 |
| IBA57 | 0.1469 | 4.8763 | 3.1221 | 0.0019 | 0.0041 | -2.1798 |
| PTPN22 | -0.3552 | 2.9075 | -3.1212 | 0.0019 | 0.0041 | -2.1824 |
| NR1H2 | 0.0564 | 6.8315 | 3.1209 | 0.0019 | 0.0041 | -2.1836 |
| FASTKD2 | 0.0725 | 5.9195 | 3.1200 | 0.0019 | 0.0041 | -2.1862 |
| HCK | -0.2395 | 5.2993 | -3.1188 | 0.0020 | 0.0041 | -2.1897 |
| ARMC4 | -0.3747 | 0.7829 | -3.1174 | 0.0020 | 0.0041 | -2.1939 |
| SH3GLB2 | -0.0868 | 6.1409 | -3.1167 | 0.0020 | 0.0041 | -2.1960 |
| RARRES3 | 0.1419 | 6.9268 | 3.1163 | 0.0020 | 0.0042 | -2.1974 |

| OTUD7B | 0.1009 | 5.7179 | 3.1158 | 0.0020 | 0.0042 | -2.1988 |
| --- | --- | --- | --- | --- | --- | --- |
| FZD8 | -0.3564 | 4.2694 | -3.1149 | 0.0020 | 0.0042 | -2.2016 |
| EMID1 | -0.3740 | 4.8202 | -3.1134 | 0.0020 | 0.0042 | -2.2060 |
| KLK8 | -0.2168 | 0.2243 | -3.1131 | 0.0020 | 0.0042 | -2.2071 |
| GGA1 | -0.0762 | 6.0356 | -3.1122 | 0.0020 | 0.0042 | -2.2098 |
| SPARC | 0.0797 | 7.3372 | 3.1119 | 0.0020 | 0.0042 | -2.2107 |
| COMMD4 | 0.0855 | 6.5685 | 3.1114 | 0.0020 | 0.0042 | -2.2121 |
| ZNF646 | -0.1235 | 5.0689 | -3.1113 | 0.0020 | 0.0042 | -2.2125 |
| ALPI | -0.6477 | 1.5832 | -3.1112 | 0.0020 | 0.0042 | -2.2127 |
| ENDOD1 | -0.2614 | 5.1626 | -3.1106 | 0.0020 | 0.0042 | -2.2145 |
| CKB | -0.3167 | 6.1841 | -3.1104 | 0.0020 | 0.0042 | -2.2152 |
| CORO1A | -0.2250 | 5.7468 | -3.1093 | 0.0020 | 0.0042 | -2.2183 |
| PFDN5 | 0.0606 | 7.2377 | 3.1092 | 0.0020 | 0.0042 | -2.2188 |
| ATP2B2 | 0.3546 | 6.0012 | 3.1090 | 0.0020 | 0.0042 | -2.2194 |
| FBXL15 | 0.1111 | 6.1605 | 3.1082 | 0.0020 | 0.0043 | -2.2219 |
| SULT1E1 | 0.5988 | 4.4526 | 3.1074 | 0.0020 | 0.0043 | -2.2240 |
| KALRN | 0.2138 | 4.5375 | 3.1074 | 0.0020 | 0.0043 | -2.2241 |
| ADCY6 | -0.1225 | 5.7997 | -3.1060 | 0.0020 | 0.0043 | -2.2283 |
| YY1 | 0.0481 | 6.4729 | 3.1060 | 0.0020 | 0.0043 | -2.2285 |
| IMP3 | 0.0709 | 6.7668 | 3.1059 | 0.0020 | 0.0043 | -2.2286 |
| IL12RB1 | -0.3143 | 3.7211 | -3.1046 | 0.0021 | 0.0043 | -2.2325 |
| SLC8A1 | -0.2798 | 2.2264 | -3.1026 | 0.0021 | 0.0043 | -2.2385 |
| ATP5G1 | 0.0802 | 7.1302 | 3.1025 | 0.0021 | 0.0043 | -2.2388 |
| HNRNPD | -0.0456 | 6.7146 | -3.1025 | 0.0021 | 0.0043 | -2.2389 |
| TCL1B | -0.1297 | 0.0921 | -3.1019 | 0.0021 | 0.0043 | -2.2407 |
| FGF3 | -0.2992 | 0.4146 | -3.1019 | 0.0021 | 0.0043 | -2.2408 |
| ACTR3 | -0.0627 | 6.3258 | -3.1017 | 0.0021 | 0.0043 | -2.2412 |
| GLG1 | -0.0668 | 6.3205 | -3.1016 | 0.0021 | 0.0043 | -2.2416 |
| CIITA | -0.2899 | 4.0295 | -3.1015 | 0.0021 | 0.0043 | -2.2419 |
| SERPINB9 | -0.2141 | 5.3739 | -3.0997 | 0.0021 | 0.0044 | -2.2472 |
| CD19 | -0.4719 | 2.1480 | -3.0997 | 0.0021 | 0.0044 | -2.2473 |
| PDE5A | -0.2804 | 3.4641 | -3.0984 | 0.0021 | 0.0044 | -2.2512 |
| BANK1 | -0.4100 | 1.5229 | -3.0981 | 0.0021 | 0.0044 | -2.2521 |
| NPAS3 | -0.2483 | 0.7968 | -3.0980 | 0.0021 | 0.0044 | -2.2525 |
| MUSK | -0.3241 | 0.5204 | -3.0978 | 0.0021 | 0.0044 | -2.2529 |
| CORO2A | -0.3161 | 4.8910 | -3.0978 | 0.0021 | 0.0044 | -2.2529 |
| SOD3 | -0.3940 | 5.4930 | -3.0973 | 0.0021 | 0.0044 | -2.2544 |
| TAS2R4 | -0.2569 | 1.6580 | -3.0970 | 0.0021 | 0.0044 | -2.2553 |
| CCDC102B | -0.2445 | 3.8098 | -3.0970 | 0.0021 | 0.0044 | -2.2553 |
| BTNL2 | -0.1187 | 0.1610 | -3.0967 | 0.0021 | 0.0044 | -2.2562 |
| C11orf68 | 0.0583 | 6.7707 | 3.0965 | 0.0021 | 0.0044 | -2.2570 |
| SEMA4G | 0.1628 | 6.5879 | 3.0950 | 0.0021 | 0.0044 | -2.2614 |
| SIRT6 | -0.0901 | 6.1292 | -3.0950 | 0.0021 | 0.0044 | -2.2615 |
| TLR6 | -0.3085 | 2.9971 | -3.0945 | 0.0021 | 0.0044 | -2.2628 |
| PRR3 | -0.1097 | 5.5557 | -3.0945 | 0.0021 | 0.0044 | -2.2628 |
| PGGT1B | 0.0893 | 5.2850 | 3.0943 | 0.0021 | 0.0044 | -2.2635 |
| FBLN1 | -0.3588 | 5.5103 | -3.0936 | 0.0021 | 0.0044 | -2.2655 |
| TMEM9B | 0.0612 | 6.5681 | 3.0936 | 0.0021 | 0.0044 | -2.2656 |
| LMAN1 | 0.0817 | 6.9114 | 3.0924 | 0.0021 | 0.0045 | -2.2692 |
| HPS1 | 0.0759 | 6.5289 | 3.0916 | 0.0021 | 0.0045 | -2.2716 |
| ATG7 | -0.0787 | 5.3115 | -3.0911 | 0.0021 | 0.0045 | -2.2731 |
| CA7 | -0.2253 | 0.3682 | -3.0904 | 0.0021 | 0.0045 | -2.2753 |
| NMNAT2 | -0.3422 | 1.3324 | -3.0902 | 0.0022 | 0.0045 | -2.2758 |

| FAR2 | -0.2999 | 3.6441 | -3.0896 | 0.0022 | 0.0045 | -2.2776 |
| --- | --- | --- | --- | --- | --- | --- |
| NPM3 | -0.1552 | 6.5366 | -3.0894 | 0.0022 | 0.0045 | -2.2780 |
| BNIP3L | -0.0995 | 6.2737 | -3.0876 | 0.0022 | 0.0045 | -2.2836 |
| PFN1 | 0.0511 | 7.6797 | 3.0875 | 0.0022 | 0.0045 | -2.2838 |
| RHCG | 0.5711 | 2.9060 | 3.0874 | 0.0022 | 0.0045 | -2.2843 |
| THRAP3 | 0.0410 | 6.8137 | 3.0855 | 0.0022 | 0.0045 | -2.2897 |
| NOS2 | 0.3644 | 3.1036 | 3.0850 | 0.0022 | 0.0046 | -2.2912 |
| SMARCA2 | 0.1657 | 6.1932 | 3.0844 | 0.0022 | 0.0046 | -2.2931 |
| TH | -0.2909 | 0.5867 | -3.0843 | 0.0022 | 0.0046 | -2.2935 |
| CSDC2 | -0.4170 | 1.5049 | -3.0834 | 0.0022 | 0.0046 | -2.2960 |
| CHRNE | 0.3007 | 3.9958 | 3.0834 | 0.0022 | 0.0046 | -2.2961 |
| TUBB2B | -0.3488 | 4.3812 | -3.0830 | 0.0022 | 0.0046 | -2.2974 |
| CXXC1 | -0.0643 | 6.3407 | -3.0824 | 0.0022 | 0.0046 | -2.2990 |
| FOXB1 | -0.0998 | 0.1496 | -3.0823 | 0.0022 | 0.0046 | -2.2993 |
| CYBB | -0.2780 | 5.3889 | -3.0816 | 0.0022 | 0.0046 | -2.3014 |
| MYH6 | -0.1405 | 0.1390 | -3.0804 | 0.0022 | 0.0046 | -2.3051 |
| DCK | -0.1709 | 5.6862 | -3.0802 | 0.0022 | 0.0046 | -2.3056 |
| CDK9 | 0.0619 | 6.7660 | 3.0796 | 0.0022 | 0.0046 | -2.3076 |
| SDF4 | 0.0552 | 7.0633 | 3.0790 | 0.0022 | 0.0046 | -2.3094 |
| PHB | 0.0581 | 7.0264 | 3.0785 | 0.0022 | 0.0046 | -2.3109 |
| TTLL7 | -0.4101 | 3.0019 | -3.0781 | 0.0022 | 0.0046 | -2.3120 |
| C19orf54 | -0.0948 | 5.7220 | -3.0771 | 0.0022 | 0.0047 | -2.3150 |
| LAX1 | -0.3648 | 2.4279 | -3.0763 | 0.0023 | 0.0047 | -2.3174 |
| ST8SIA1 | -0.2489 | 1.0902 | -3.0760 | 0.0023 | 0.0047 | -2.3182 |
| SLC1A6 | -0.1125 | 0.1229 | -3.0748 | 0.0023 | 0.0047 | -2.3219 |
| HOMER1 | -0.4432 | 3.6546 | -3.0738 | 0.0023 | 0.0047 | -2.3246 |
| POU4F3 | -0.2045 | 0.5392 | -3.0731 | 0.0023 | 0.0047 | -2.3269 |
| LBP | 0.2298 | 7.5795 | 3.0716 | 0.0023 | 0.0047 | -2.3312 |
| PFKL | 0.0672 | 6.8188 | 3.0715 | 0.0023 | 0.0047 | -2.3317 |
| TRPV2 | -0.1886 | 5.3759 | -3.0714 | 0.0023 | 0.0047 | -2.3319 |
| RAI14 | 0.1156 | 6.0834 | 3.0712 | 0.0023 | 0.0047 | -2.3325 |
| ACVR2A | -0.1263 | 4.9488 | -3.0708 | 0.0023 | 0.0047 | -2.3337 |
| SENP2 | 0.0834 | 5.9060 | 3.0701 | 0.0023 | 0.0048 | -2.3357 |
| VAMP4 | 0.1110 | 5.6245 | 3.0685 | 0.0023 | 0.0048 | -2.3404 |
| CUX1 | 0.0980 | 5.8401 | 3.0673 | 0.0023 | 0.0048 | -2.3440 |
| TUBA1B | -0.1371 | 6.8445 | -3.0672 | 0.0023 | 0.0048 | -2.3445 |
| OR2B2 | -0.1284 | 0.1587 | -3.0669 | 0.0023 | 0.0048 | -2.3453 |
| PCSK1 | -0.3965 | 1.0864 | -3.0655 | 0.0023 | 0.0048 | -2.3495 |
| PCDHA9 | -0.0899 | 0.1218 | -3.0644 | 0.0023 | 0.0048 | -2.3528 |
| SCAND1 | 0.0773 | 7.0037 | 3.0637 | 0.0023 | 0.0049 | -2.3549 |
| PNP | -0.1234 | 6.2409 | -3.0635 | 0.0023 | 0.0049 | -2.3555 |
| SNW1 | 0.0489 | 6.7523 | 3.0632 | 0.0023 | 0.0049 | -2.3562 |
| GABRP | -0.4796 | 1.4778 | -3.0627 | 0.0024 | 0.0049 | -2.3578 |
| SHANK1 | -0.2105 | 0.6629 | -3.0624 | 0.0024 | 0.0049 | -2.3587 |
| TREML2 | -0.3022 | 0.9656 | -3.0620 | 0.0024 | 0.0049 | -2.3599 |
| CDKAL1 | -0.0973 | 5.6536 | -3.0611 | 0.0024 | 0.0049 | -2.3625 |
| SYNPO | 0.1088 | 6.4893 | 3.0606 | 0.0024 | 0.0049 | -2.3639 |
| TIMP3 | 0.4244 | 3.7657 | 3.0604 | 0.0024 | 0.0049 | -2.3647 |
| IMPG2 | -0.1683 | 0.8935 | -3.0601 | 0.0024 | 0.0049 | -2.3654 |
| ARHGEF12 | 0.1176 | 6.1108 | 3.0601 | 0.0024 | 0.0049 | -2.3655 |
| FGR | -0.2324 | 4.7341 | -3.0593 | 0.0024 | 0.0049 | -2.3678 |
| VEGFC | 0.1978 | 5.1650 | 3.0591 | 0.0024 | 0.0049 | -2.3683 |
| STAP1 | -0.3874 | 1.7519 | -3.0586 | 0.0024 | 0.0049 | -2.3699 |

| KCNE2 | 0.3118 | 3.2657 | 3.0582 | 0.0024 | 0.0049 | -2.3711 |
| --- | --- | --- | --- | --- | --- | --- |
| PRDM10 | -0.1382 | 4.6269 | -3.0578 | 0.0024 | 0.0049 | -2.3723 |
| PSG2 | -0.0799 | 0.0645 | -3.0574 | 0.0024 | 0.0049 | -2.3735 |
| KIFC3 | -0.1111 | 6.0870 | -3.0569 | 0.0024 | 0.0049 | -2.3751 |
| MAFG | -0.1527 | 5.7835 | -3.0566 | 0.0024 | 0.0049 | -2.3759 |
| RPL32 | 0.0644 | 7.5844 | 3.0555 | 0.0024 | 0.0050 | -2.3792 |
| ST8SIA4 | -0.2679 | 3.8614 | -3.0548 | 0.0024 | 0.0050 | -2.3813 |
| ERGIC3 | 0.0539 | 7.0722 | 3.0542 | 0.0024 | 0.0050 | -2.3831 |
| FOSB | 0.4439 | 4.6608 | 3.0540 | 0.0024 | 0.0050 | -2.3836 |
| SLC12A3 | -0.1608 | 0.4693 | -3.0538 | 0.0024 | 0.0050 | -2.3840 |
| SHC2 | 0.1727 | 6.5964 | 3.0536 | 0.0024 | 0.0050 | -2.3846 |
| PCDHGA8 | -0.2442 | 0.7841 | -3.0517 | 0.0024 | 0.0050 | -2.3905 |
| TMEM189 | -0.1056 | 5.9537 | -3.0506 | 0.0024 | 0.0050 | -2.3935 |
| ZFP30 | -0.3553 | 3.6112 | -3.0497 | 0.0025 | 0.0051 | -2.3963 |
| CHGB | -0.4543 | 0.9008 | -3.0495 | 0.0025 | 0.0051 | -2.3968 |
| JUN | 0.0936 | 7.0328 | 3.0490 | 0.0025 | 0.0051 | -2.3984 |
| ADRB3 | -0.1261 | 0.2025 | -3.0487 | 0.0025 | 0.0051 | -2.3992 |
| PDZK1 | 0.1984 | 6.8451 | 3.0486 | 0.0025 | 0.0051 | -2.3995 |
| TBCB | -0.0808 | 6.5442 | -3.0482 | 0.0025 | 0.0051 | -2.4007 |
| GAGE1 | -0.3876 | 0.4614 | -3.0481 | 0.0025 | 0.0051 | -2.4011 |
| FBXL7 | -0.2806 | 4.5336 | -3.0479 | 0.0025 | 0.0051 | -2.4015 |
| SPAG4 | -0.2690 | 5.2448 | -3.0475 | 0.0025 | 0.0051 | -2.4028 |
| RNF123 | 0.1691 | 5.8999 | 3.0474 | 0.0025 | 0.0051 | -2.4029 |
| UBE2E1 | -0.0523 | 6.5640 | -3.0473 | 0.0025 | 0.0051 | -2.4033 |
| SLC6A11 | -0.7997 | 2.6827 | -3.0467 | 0.0025 | 0.0051 | -2.4050 |
| ZNF747 | 0.1001 | 5.8622 | 3.0467 | 0.0025 | 0.0051 | -2.4053 |
| ZNF518A | -0.1849 | 4.8929 | -3.0461 | 0.0025 | 0.0051 | -2.4070 |
| KCNMB1 | -0.2749 | 2.5165 | -3.0458 | 0.0025 | 0.0051 | -2.4077 |
| CMA1 | 0.4789 | 1.7104 | 3.0452 | 0.0025 | 0.0051 | -2.4096 |
| BEST2 | -0.1319 | 0.1947 | -3.0443 | 0.0025 | 0.0051 | -2.4121 |
| PDE12 | 0.0798 | 5.7243 | 3.0442 | 0.0025 | 0.0051 | -2.4125 |
| HSD17B3 | 0.3812 | 4.3547 | 3.0420 | 0.0025 | 0.0052 | -2.4189 |
| ONECUT2 | 0.2621 | 5.7269 | 3.0420 | 0.0025 | 0.0052 | -2.4191 |
| DLG5 | -0.3021 | 4.7738 | -3.0413 | 0.0025 | 0.0052 | -2.4210 |
| SSH3 | -0.0970 | 6.1405 | -3.0412 | 0.0025 | 0.0052 | -2.4214 |
| PAPOLG | -0.1409 | 4.5333 | -3.0402 | 0.0025 | 0.0052 | -2.4243 |
| SIPA1L1 | 0.1001 | 5.6614 | 3.0399 | 0.0025 | 0.0052 | -2.4252 |
| RPL5 | 0.0528 | 7.6433 | 3.0397 | 0.0025 | 0.0052 | -2.4257 |
| MAN1A1 | 0.1063 | 6.9309 | 3.0396 | 0.0025 | 0.0052 | -2.4260 |
| EPHA2 | -0.1933 | 5.8922 | -3.0396 | 0.0025 | 0.0052 | -2.4262 |
| SNRPC | 0.0559 | 7.3095 | 3.0391 | 0.0025 | 0.0052 | -2.4276 |
| FOXL2 | -0.2890 | 0.4106 | -3.0388 | 0.0025 | 0.0052 | -2.4286 |
| MXRA8 | -0.2687 | 5.3924 | -3.0383 | 0.0025 | 0.0052 | -2.4299 |
| DCP1A | -0.1009 | 5.4537 | -3.0383 | 0.0025 | 0.0052 | -2.4300 |
| KRT7 | -0.6423 | 4.8307 | -3.0372 | 0.0026 | 0.0052 | -2.4333 |
| HIST1H4E | -0.4768 | 3.4226 | -3.0364 | 0.0026 | 0.0052 | -2.4354 |
| SMCHD1 | -0.1453 | 5.2466 | -3.0347 | 0.0026 | 0.0053 | -2.4405 |
| DCBLD2 | -0.1419 | 5.2729 | -3.0346 | 0.0026 | 0.0053 | -2.4408 |
| SS18L2 | 0.0781 | 6.5875 | 3.0346 | 0.0026 | 0.0053 | -2.4408 |
| C4A | 0.2077 | 6.7623 | 3.0342 | 0.0026 | 0.0053 | -2.4420 |
| FAM13C | 0.3548 | 2.9393 | 3.0331 | 0.0026 | 0.0053 | -2.4452 |
| RBL2 | 0.2003 | 5.9607 | 3.0331 | 0.0026 | 0.0053 | -2.4453 |
| BOLA1 | 0.0837 | 6.6479 | 3.0330 | 0.0026 | 0.0053 | -2.4455 |

| MAGEA2 | -0.1366 | 0.1838 | -3.0324 | 0.0026 | 0.0053 | -2.4472 |
| --- | --- | --- | --- | --- | --- | --- |
| POMZP3 | -0.1549 | 5.0411 | -3.0322 | 0.0026 | 0.0053 | -2.4479 |
| XAF1 | 0.3937 | 4.0914 | 3.0308 | 0.0026 | 0.0053 | -2.4521 |
| MAGEA6 | -0.8782 | 2.1552 | -3.0295 | 0.0026 | 0.0054 | -2.4559 |
| MCTP1 | -0.3626 | 3.5546 | -3.0294 | 0.0026 | 0.0054 | -2.4560 |
| EIF3K | 0.0640 | 7.2483 | 3.0290 | 0.0026 | 0.0054 | -2.4572 |
| AIP | 0.0590 | 7.0079 | 3.0289 | 0.0026 | 0.0054 | -2.4574 |
| GGTLC2 | -0.2274 | 0.6577 | -3.0287 | 0.0026 | 0.0054 | -2.4581 |
| CHERP | 0.0790 | 6.2612 | 3.0285 | 0.0026 | 0.0054 | -2.4588 |
| TAC1 | -0.2489 | 0.2776 | -3.0284 | 0.0026 | 0.0054 | -2.4591 |
| ATP5O | 0.0807 | 6.8965 | 3.0279 | 0.0026 | 0.0054 | -2.4607 |
| ZNF484 | -0.1884 | 3.6432 | -3.0266 | 0.0026 | 0.0054 | -2.4644 |
| PCBP1 | 0.0870 | 7.3645 | 3.0264 | 0.0026 | 0.0054 | -2.4648 |
| ARL6IP4 | -0.1872 | 4.5249 | -3.0264 | 0.0026 | 0.0054 | -2.4651 |
| HPS6 | 0.0730 | 6.1381 | 3.0240 | 0.0027 | 0.0054 | -2.4720 |
| AHCYL1 | 0.0552 | 6.8476 | 3.0238 | 0.0027 | 0.0054 | -2.4725 |
| CTSA | 0.0645 | 7.2272 | 3.0232 | 0.0027 | 0.0054 | -2.4742 |
| JAG1 | -0.2004 | 5.6305 | -3.0232 | 0.0027 | 0.0054 | -2.4743 |
| SF3A2 | -0.0718 | 6.7282 | -3.0229 | 0.0027 | 0.0055 | -2.4751 |
| NGB | -0.1925 | 0.2095 | -3.0223 | 0.0027 | 0.0055 | -2.4768 |
| PNLIPRP1 | -0.1517 | 0.1498 | -3.0216 | 0.0027 | 0.0055 | -2.4789 |
| GPRC5C | 0.0853 | 6.7808 | 3.0214 | 0.0027 | 0.0055 | -2.4797 |
| PROX1 | 0.1856 | 6.5857 | 3.0212 | 0.0027 | 0.0055 | -2.4802 |
| PLCL2 | 0.2365 | 5.2926 | 3.0210 | 0.0027 | 0.0055 | -2.4806 |
| PPP1R15A | -0.0971 | 6.5084 | -3.0210 | 0.0027 | 0.0055 | -2.4807 |
| GABRR2 | -0.2788 | 1.1591 | -3.0195 | 0.0027 | 0.0055 | -2.4853 |
| ZNF692 | -0.1169 | 6.0452 | -3.0190 | 0.0027 | 0.0055 | -2.4867 |
| CDH16 | -0.6110 | 2.4680 | -3.0185 | 0.0027 | 0.0055 | -2.4880 |
| SERPINA5 | 0.2378 | 7.3086 | 3.0178 | 0.0027 | 0.0055 | -2.4901 |
| PPP2R5E | -0.0778 | 5.6818 | -3.0170 | 0.0027 | 0.0055 | -2.4925 |
| RNF19B | -0.1211 | 6.0498 | -3.0166 | 0.0027 | 0.0056 | -2.4937 |
| FARP2 | -0.1553 | 4.6964 | -3.0162 | 0.0027 | 0.0056 | -2.4947 |
| JAK1 | 0.0697 | 6.7644 | 3.0154 | 0.0027 | 0.0056 | -2.4972 |
| TUBA4A | -0.1565 | 6.5057 | -3.0140 | 0.0028 | 0.0056 | -2.5013 |
| AARSD1 | -0.1300 | 4.6976 | -3.0139 | 0.0028 | 0.0056 | -2.5016 |
| TAF6L | 0.0847 | 6.0315 | 3.0136 | 0.0028 | 0.0056 | -2.5026 |
| APOL2 | 0.0832 | 6.8270 | 3.0135 | 0.0028 | 0.0056 | -2.5029 |
| SIVA1 | 0.1016 | 6.5226 | 3.0125 | 0.0028 | 0.0056 | -2.5056 |
| AP1M2 | -0.7397 | 4.2507 | -3.0118 | 0.0028 | 0.0056 | -2.5076 |
| CHRM3 | 0.4293 | 2.7230 | 3.0118 | 0.0028 | 0.0056 | -2.5078 |
| CACNA1A | -0.2072 | 1.0263 | -3.0116 | 0.0028 | 0.0056 | -2.5083 |
| NDST2 | -0.1252 | 3.3566 | -3.0115 | 0.0028 | 0.0056 | -2.5086 |
| NCLN | 0.0626 | 6.9150 | 3.0113 | 0.0028 | 0.0056 | -2.5092 |
| GSTM5 | 0.4495 | 1.8734 | 3.0112 | 0.0028 | 0.0056 | -2.5095 |
| PRAF2 | -0.1094 | 6.1998 | -3.0100 | 0.0028 | 0.0057 | -2.5128 |
| PDPN | -0.5571 | 2.3130 | -3.0086 | 0.0028 | 0.0057 | -2.5171 |
| HOXC5 | -0.1987 | 0.2537 | -3.0082 | 0.0028 | 0.0057 | -2.5183 |
| PPARD | -0.1072 | 6.2761 | -3.0080 | 0.0028 | 0.0057 | -2.5189 |
| BDH2 | 0.1485 | 6.1154 | 3.0072 | 0.0028 | 0.0057 | -2.5211 |
| TARBP2 | -0.0793 | 6.1109 | -3.0068 | 0.0028 | 0.0057 | -2.5222 |
| XPO7 | -0.0974 | 5.9621 | -3.0055 | 0.0028 | 0.0057 | -2.5260 |
| HS6ST1 | 0.1156 | 6.5518 | 3.0054 | 0.0028 | 0.0057 | -2.5264 |
| NR2E1 | -0.2332 | 0.5671 | -3.0041 | 0.0028 | 0.0058 | -2.5302 |

| CCR8 | -0.3855 | 1.3560 | -3.0039 | 0.0028 | 0.0058 | -2.5308 |
| --- | --- | --- | --- | --- | --- | --- |
| ZCCHC14 | 0.1102 | 6.0746 | 3.0037 | 0.0028 | 0.0058 | -2.5313 |
| ST8SIA5 | -0.1456 | 0.4289 | -3.0033 | 0.0029 | 0.0058 | -2.5326 |
| ACTR1A | 0.0533 | 6.8145 | 3.0013 | 0.0029 | 0.0058 | -2.5382 |
| MTMR6 | 0.1121 | 5.7192 | 3.0011 | 0.0029 | 0.0058 | -2.5390 |
| FUT9 | -0.1206 | 0.0801 | -3.0008 | 0.0029 | 0.0058 | -2.5399 |
| MAPK8IP3 | -0.1629 | 5.4123 | -2.9992 | 0.0029 | 0.0058 | -2.5444 |
| AFG3L2 | 0.0767 | 6.3360 | 2.9991 | 0.0029 | 0.0058 | -2.5448 |
| RPS6KA1 | -0.1674 | 5.7472 | -2.9979 | 0.0029 | 0.0059 | -2.5481 |
| ZSCAN16 | -0.1396 | 5.5704 | -2.9978 | 0.0029 | 0.0059 | -2.5486 |
| SNED1 | 0.2747 | 4.1921 | 2.9975 | 0.0029 | 0.0059 | -2.5495 |
| DNM3 | 0.2377 | 2.4084 | 2.9974 | 0.0029 | 0.0059 | -2.5495 |
| LSP1 | -0.2160 | 5.6714 | -2.9973 | 0.0029 | 0.0059 | -2.5501 |
| GOLGA4 | 0.1051 | 6.2478 | 2.9965 | 0.0029 | 0.0059 | -2.5523 |
| ESRRG | -0.4521 | 1.3036 | -2.9958 | 0.0029 | 0.0059 | -2.5542 |
| PDE6A | -0.1770 | 0.5601 | -2.9950 | 0.0029 | 0.0059 | -2.5566 |
| ZMIZ1 | -0.1403 | 5.8448 | -2.9945 | 0.0029 | 0.0059 | -2.5582 |
| SYNC | -0.3244 | 1.4475 | -2.9944 | 0.0029 | 0.0059 | -2.5584 |
| KDM6A | -0.1626 | 5.4152 | -2.9943 | 0.0029 | 0.0059 | -2.5587 |
| SLC31A2 | 0.3126 | 3.5001 | 2.9936 | 0.0029 | 0.0059 | -2.5607 |
| PDHA2 | -0.1350 | 0.1538 | -2.9923 | 0.0030 | 0.0060 | -2.5643 |
| CD27 | -0.3835 | 4.2712 | -2.9923 | 0.0030 | 0.0060 | -2.5645 |
| GPM6A | 0.4159 | 1.8608 | 2.9918 | 0.0030 | 0.0060 | -2.5659 |
| FIG4 | -0.1027 | 5.5680 | -2.9916 | 0.0030 | 0.0060 | -2.5664 |
| PWP2 | -0.4090 | 1.6692 | -2.9905 | 0.0030 | 0.0060 | -2.5696 |
| KANK1 | 0.1608 | 6.2154 | 2.9905 | 0.0030 | 0.0060 | -2.5697 |
| AKAP3 | 0.3247 | 2.2278 | 2.9882 | 0.0030 | 0.0060 | -2.5765 |
| TMLHE | 0.1179 | 5.3944 | 2.9874 | 0.0030 | 0.0060 | -2.5787 |
| RNASE3 | -0.2308 | 0.4427 | -2.9869 | 0.0030 | 0.0061 | -2.5803 |
| CTF1 | -0.3913 | 3.3353 | -2.9854 | 0.0030 | 0.0061 | -2.5845 |
| CLEC7A | -0.3376 | 3.7636 | -2.9844 | 0.0030 | 0.0061 | -2.5875 |
| RHOBTB1 | -0.3496 | 5.2239 | -2.9842 | 0.0030 | 0.0061 | -2.5880 |
| PORCN | -0.1726 | 4.9693 | -2.9841 | 0.0030 | 0.0061 | -2.5884 |
| ABLIM3 | 0.1599 | 6.2596 | 2.9840 | 0.0030 | 0.0061 | -2.5885 |
| CCNO | -0.6187 | 3.2743 | -2.9839 | 0.0030 | 0.0061 | -2.5887 |
| REG1A | -0.8344 | 2.8369 | -2.9820 | 0.0031 | 0.0061 | -2.5944 |
| MTMR14 | 0.0578 | 6.3200 | 2.9819 | 0.0031 | 0.0061 | -2.5947 |
| CLUL1 | -0.3148 | 1.2520 | -2.9810 | 0.0031 | 0.0062 | -2.5971 |
| PRPF6 | 0.0507 | 7.1556 | 2.9808 | 0.0031 | 0.0062 | -2.5978 |
| C3orf18 | 0.2018 | 5.2014 | 2.9804 | 0.0031 | 0.0062 | -2.5991 |
| CACNG4 | -0.6705 | 2.5486 | -2.9785 | 0.0031 | 0.0062 | -2.6046 |
| NEUROD1 | -0.1892 | 0.1403 | -2.9777 | 0.0031 | 0.0062 | -2.6068 |
| EXOC6B | -0.1412 | 4.8249 | -2.9769 | 0.0031 | 0.0062 | -2.6090 |
| VCPIP1 | 0.1069 | 5.5383 | 2.9768 | 0.0031 | 0.0062 | -2.6094 |
| RAPGEFL1 | -0.2521 | 4.7293 | -2.9754 | 0.0031 | 0.0063 | -2.6134 |
| CD180 | -0.3125 | 3.2253 | -2.9753 | 0.0031 | 0.0063 | -2.6137 |
| PITPNA | 0.0716 | 6.4288 | 2.9746 | 0.0031 | 0.0063 | -2.6156 |
| CASP8 | -0.1151 | 5.5833 | -2.9731 | 0.0031 | 0.0063 | -2.6202 |
| GTPBP1 | -0.0736 | 5.9463 | -2.9728 | 0.0031 | 0.0063 | -2.6210 |
| C1orf186 | -0.5896 | 3.1517 | -2.9723 | 0.0031 | 0.0063 | -2.6223 |
| SORCS3 | -0.2383 | 0.2848 | -2.9719 | 0.0032 | 0.0063 | -2.6234 |
| LYST | 0.1794 | 4.5095 | 2.9710 | 0.0032 | 0.0063 | -2.6262 |
| NLGN4Y | 0.5678 | 2.1937 | 2.9708 | 0.0032 | 0.0063 | -2.6266 |

| NPTXR | -0.4976 | 3.2924 | -2.9703 | 0.0032 | 0.0064 | -2.6280 |
| --- | --- | --- | --- | --- | --- | --- |
| WNT2 | -0.4750 | 1.3358 | -2.9703 | 0.0032 | 0.0064 | -2.6281 |
| TAF1A | -0.2177 | 4.4393 | -2.9698 | 0.0032 | 0.0064 | -2.6295 |
| NCOA6 | -0.1041 | 5.7288 | -2.9697 | 0.0032 | 0.0064 | -2.6299 |
| PIP | -0.4148 | 0.8299 | -2.9688 | 0.0032 | 0.0064 | -2.6324 |
| AIRE | -0.2458 | 0.5095 | -2.9688 | 0.0032 | 0.0064 | -2.6326 |
| GRM6 | -0.1974 | 0.5266 | -2.9684 | 0.0032 | 0.0064 | -2.6335 |
| DGKD | -0.1266 | 5.2304 | -2.9677 | 0.0032 | 0.0064 | -2.6355 |
| GORASP1 | 0.0549 | 6.1239 | 2.9674 | 0.0032 | 0.0064 | -2.6364 |
| OR3A2 | -0.1321 | 0.1239 | -2.9665 | 0.0032 | 0.0064 | -2.6392 |
| POU6F2 | -0.3562 | 0.7128 | -2.9658 | 0.0032 | 0.0064 | -2.6412 |
| ZBTB10 | 0.1365 | 5.5845 | 2.9647 | 0.0032 | 0.0065 | -2.6442 |
| ACP6 | 0.1505 | 5.1775 | 2.9637 | 0.0032 | 0.0065 | -2.6471 |
| PSMD6 | 0.0562 | 6.1068 | 2.9636 | 0.0032 | 0.0065 | -2.6474 |
| WDR12 | -0.0936 | 5.5353 | -2.9628 | 0.0032 | 0.0065 | -2.6496 |
| BPTF | -0.1319 | 5.3963 | -2.9624 | 0.0032 | 0.0065 | -2.6510 |
| DRD2 | -0.1497 | 0.2014 | -2.9620 | 0.0033 | 0.0065 | -2.6520 |
| ARPC5L | -0.0741 | 6.4492 | -2.9619 | 0.0033 | 0.0065 | -2.6522 |
| BEX4 | -0.2037 | 5.8014 | -2.9618 | 0.0033 | 0.0065 | -2.6525 |
| LRRN3 | -0.3068 | 1.2966 | -2.9613 | 0.0033 | 0.0065 | -2.6542 |
| PLEKHO2 | -0.1217 | 6.0408 | -2.9611 | 0.0033 | 0.0065 | -2.6545 |
| CLTB | 0.0683 | 7.0191 | 2.9604 | 0.0033 | 0.0065 | -2.6566 |
| SPAG1 | -0.2345 | 4.8157 | -2.9602 | 0.0033 | 0.0065 | -2.6573 |
| PRLR | 0.2680 | 5.2107 | 2.9597 | 0.0033 | 0.0065 | -2.6587 |
| NCF4 | -0.2119 | 5.4498 | -2.9583 | 0.0033 | 0.0066 | -2.6626 |
| SLC12A7 | 0.0662 | 6.8746 | 2.9578 | 0.0033 | 0.0066 | -2.6640 |
| PAFAH2 | 0.1055 | 5.9936 | 2.9575 | 0.0033 | 0.0066 | -2.6649 |
| PMF1 | 0.0860 | 6.6917 | 2.9573 | 0.0033 | 0.0066 | -2.6657 |
| BASP1 | -0.3179 | 5.1065 | -2.9572 | 0.0033 | 0.0066 | -2.6659 |
| SPTLC2 | -0.0843 | 5.6005 | -2.9571 | 0.0033 | 0.0066 | -2.6661 |
| DSC1 | 0.1650 | 0.5921 | 2.9563 | 0.0033 | 0.0066 | -2.6683 |
| FAM124B | 0.3743 | 3.5439 | 2.9561 | 0.0033 | 0.0066 | -2.6689 |
| ALG3 | 0.0609 | 6.9100 | 2.9558 | 0.0033 | 0.0066 | -2.6699 |
| BFSP2 | -0.4172 | 1.6833 | -2.9540 | 0.0033 | 0.0067 | -2.6751 |
| ST7L | -0.1100 | 4.5057 | -2.9539 | 0.0033 | 0.0067 | -2.6753 |
| DLG2 | 0.2653 | 1.6970 | 2.9528 | 0.0033 | 0.0067 | -2.6783 |
| RRBP1 | 0.0571 | 7.3723 | 2.9518 | 0.0034 | 0.0067 | -2.6813 |
| ENC1 | -0.2164 | 5.6755 | -2.9518 | 0.0034 | 0.0067 | -2.6814 |
| TAS2R14 | -0.2324 | 2.8075 | -2.9517 | 0.0034 | 0.0067 | -2.6815 |
| B3GAT1 | -0.5588 | 2.0247 | -2.9511 | 0.0034 | 0.0067 | -2.6833 |
| PSG9 | -0.1817 | 0.2245 | -2.9508 | 0.0034 | 0.0067 | -2.6842 |
| ENSA | 0.0561 | 7.0255 | 2.9500 | 0.0034 | 0.0067 | -2.6865 |
| WDR18 | 0.0689 | 6.7256 | 2.9486 | 0.0034 | 0.0068 | -2.6904 |
| LRP2 | -0.4669 | 1.2732 | -2.9483 | 0.0034 | 0.0068 | -2.6915 |
| KLK5 | -0.2101 | 0.2241 | -2.9479 | 0.0034 | 0.0068 | -2.6923 |
| ARMCX2 | -0.2976 | 4.0681 | -2.9464 | 0.0034 | 0.0068 | -2.6967 |
| TFB1M | 0.1185 | 5.6940 | 2.9457 | 0.0034 | 0.0068 | -2.6987 |
| AK4 | 0.2276 | 6.5128 | 2.9457 | 0.0034 | 0.0068 | -2.6988 |
| ARID5A | -0.1204 | 6.1964 | -2.9447 | 0.0034 | 0.0068 | -2.7016 |
| ARMC8 | -0.0793 | 5.4261 | -2.9446 | 0.0034 | 0.0068 | -2.7020 |
| HTRA1 | 0.1334 | 7.1870 | 2.9442 | 0.0034 | 0.0068 | -2.7032 |
| OAS1 | 0.1553 | 6.2183 | 2.9441 | 0.0034 | 0.0068 | -2.7033 |
| GSTM4 | 0.1484 | 6.0514 | 2.9441 | 0.0034 | 0.0068 | -2.7034 |

| LRIG2 | -0.1427 | 4.2970 | -2.9428 | 0.0035 | 0.0069 | -2.7072 |
| --- | --- | --- | --- | --- | --- | --- |
| NEDD8 | 0.0624 | 6.7238 | 2.9418 | 0.0035 | 0.0069 | -2.7100 |
| EBNA1BP2 | 0.0743 | 6.6914 | 2.9417 | 0.0035 | 0.0069 | -2.7102 |
| MED25 | -0.0719 | 6.0986 | -2.9411 | 0.0035 | 0.0069 | -2.7120 |
| PDZD8 | -0.1289 | 5.7612 | -2.9402 | 0.0035 | 0.0069 | -2.7145 |
| INSL6 | -0.4194 | 0.7091 | -2.9400 | 0.0035 | 0.0069 | -2.7151 |
| OR2B6 | -0.3994 | 2.6111 | -2.9396 | 0.0035 | 0.0069 | -2.7163 |
| LAG3 | -0.3176 | 4.3953 | -2.9385 | 0.0035 | 0.0070 | -2.7194 |
| ABCF1 | 0.0510 | 6.8590 | 2.9381 | 0.0035 | 0.0070 | -2.7205 |
| PRICKLE3 | -0.1142 | 5.0705 | -2.9347 | 0.0035 | 0.0070 | -2.7302 |
| RLF | -0.1466 | 5.1630 | -2.9344 | 0.0035 | 0.0070 | -2.7310 |
| BBC3 | -0.1336 | 5.7029 | -2.9339 | 0.0036 | 0.0071 | -2.7325 |
| EIF4E | -0.1095 | 4.7329 | -2.9338 | 0.0036 | 0.0071 | -2.7327 |
| HNRNPR | -0.0603 | 6.3760 | -2.9331 | 0.0036 | 0.0071 | -2.7347 |
| PGK2 | -0.2066 | 0.3162 | -2.9328 | 0.0036 | 0.0071 | -2.7356 |
| TSEN2 | -0.1261 | 4.7697 | -2.9326 | 0.0036 | 0.0071 | -2.7361 |
| RNF4 | -0.0770 | 6.0593 | -2.9319 | 0.0036 | 0.0071 | -2.7380 |
| KCTD9 | -0.1552 | 5.3240 | -2.9317 | 0.0036 | 0.0071 | -2.7387 |
| IL21 | -0.1816 | 0.2137 | -2.9307 | 0.0036 | 0.0071 | -2.7416 |
| BCL2L14 | -0.4324 | 2.3599 | -2.9304 | 0.0036 | 0.0071 | -2.7426 |
| CD164 | 0.0548 | 6.9799 | 2.9282 | 0.0036 | 0.0072 | -2.7487 |
| FYCO1 | 0.1160 | 5.8193 | 2.9278 | 0.0036 | 0.0072 | -2.7499 |
| TGM5 | -0.1656 | 0.2237 | -2.9274 | 0.0036 | 0.0072 | -2.7509 |
| TFEB | -0.1782 | 5.4069 | -2.9273 | 0.0036 | 0.0072 | -2.7514 |
| APAF1 | -0.2633 | 4.5718 | -2.9269 | 0.0036 | 0.0072 | -2.7523 |
| IFT122 | -0.1055 | 5.1660 | -2.9267 | 0.0036 | 0.0072 | -2.7530 |
| B4GALNT1 | -0.4933 | 3.5796 | -2.9263 | 0.0036 | 0.0072 | -2.7541 |
| MRPS15 | 0.0699 | 6.9812 | 2.9243 | 0.0037 | 0.0072 | -2.7597 |
| UBXN7 | -0.1273 | 5.2017 | -2.9231 | 0.0037 | 0.0073 | -2.7631 |
| RAD51B | -0.1844 | 3.0849 | -2.9231 | 0.0037 | 0.0073 | -2.7632 |
| MORC2 | -0.0645 | 6.1881 | -2.9227 | 0.0037 | 0.0073 | -2.7645 |
| TRIM37 | -0.1097 | 5.6513 | -2.9225 | 0.0037 | 0.0073 | -2.7648 |
| DSCR3 | 0.0802 | 5.9016 | 2.9222 | 0.0037 | 0.0073 | -2.7658 |
| LAMC1 | -0.1072 | 6.6744 | -2.9203 | 0.0037 | 0.0073 | -2.7711 |
| MME | 0.7494 | 3.5857 | 2.9200 | 0.0037 | 0.0073 | -2.7721 |
| IGFBP5 | 0.2275 | 6.1904 | 2.9188 | 0.0037 | 0.0074 | -2.7753 |
| ZNF614 | -0.2888 | 3.9613 | -2.9186 | 0.0037 | 0.0074 | -2.7759 |
| CBWD1 | -0.1581 | 3.8358 | -2.9179 | 0.0037 | 0.0074 | -2.7778 |
| TEAD3 | -0.0816 | 6.2726 | -2.9170 | 0.0037 | 0.0074 | -2.7804 |
| ARHGEF17 | -0.2376 | 4.9333 | -2.9166 | 0.0038 | 0.0074 | -2.7815 |
| JPH3 | -0.2549 | 0.6396 | -2.9165 | 0.0038 | 0.0074 | -2.7818 |
| ZNF385D | 0.4975 | 1.7964 | 2.9156 | 0.0038 | 0.0074 | -2.7845 |
| TOPORS | 0.1029 | 5.8511 | 2.9147 | 0.0038 | 0.0074 | -2.7869 |
| ZNF629 | -0.1301 | 5.5715 | -2.9144 | 0.0038 | 0.0075 | -2.7877 |
| NFATC1 | -0.2811 | 3.9235 | -2.9139 | 0.0038 | 0.0075 | -2.7892 |
| TLR8 | -0.3806 | 2.5996 | -2.9139 | 0.0038 | 0.0075 | -2.7893 |
| FLRT3 | -0.3507 | 4.9245 | -2.9136 | 0.0038 | 0.0075 | -2.7901 |
| CNR1 | -0.4633 | 1.5179 | -2.9134 | 0.0038 | 0.0075 | -2.7906 |
| ACOT8 | 0.0815 | 6.1768 | 2.9107 | 0.0038 | 0.0075 | -2.7983 |
| PICALM | 0.0457 | 6.8178 | 2.9107 | 0.0038 | 0.0075 | -2.7984 |
| SOX15 | -0.2346 | 2.7746 | -2.9104 | 0.0038 | 0.0075 | -2.7992 |
| YARS | -0.0813 | 6.4189 | -2.9091 | 0.0038 | 0.0076 | -2.8028 |
| HECW1 | -0.2879 | 0.9655 | -2.9085 | 0.0038 | 0.0076 | -2.8045 |

| NINJ1 | 0.0594 | 7.0228 | 2.9081 | 0.0039 | 0.0076 | -2.8055 |
| --- | --- | --- | --- | --- | --- | --- |
| PIGC | -0.0789 | 6.1947 | -2.9070 | 0.0039 | 0.0076 | -2.8088 |
| PYROXD1 | 0.1046 | 5.3760 | 2.9069 | 0.0039 | 0.0076 | -2.8091 |
| PSMA5 | 0.0642 | 6.8022 | 2.9064 | 0.0039 | 0.0076 | -2.8105 |
| MRPL17 | -0.0662 | 6.6951 | -2.9063 | 0.0039 | 0.0076 | -2.8109 |
| AICDA | -0.1734 | 0.2764 | -2.9063 | 0.0039 | 0.0076 | -2.8109 |
| WASF2 | -0.0724 | 6.4822 | -2.9057 | 0.0039 | 0.0076 | -2.8124 |
| SRPRB | 0.0620 | 6.9013 | 2.9053 | 0.0039 | 0.0076 | -2.8136 |
| LTBR | 0.0617 | 6.8620 | 2.9048 | 0.0039 | 0.0077 | -2.8150 |
| KHDRBS3 | 0.2178 | 5.0092 | 2.9044 | 0.0039 | 0.0077 | -2.8161 |
| EGFL7 | 0.1206 | 6.6708 | 2.9042 | 0.0039 | 0.0077 | -2.8166 |
| CLIP4 | -0.4215 | 4.2505 | -2.9042 | 0.0039 | 0.0077 | -2.8167 |
| RALYL | -0.3483 | 0.6877 | -2.9034 | 0.0039 | 0.0077 | -2.8189 |
| GGA3 | -0.0832 | 5.8791 | -2.9030 | 0.0039 | 0.0077 | -2.8201 |
| SAMD4B | -0.0534 | 6.3466 | -2.9027 | 0.0039 | 0.0077 | -2.8209 |
| PLCE1 | -0.2007 | 3.9796 | -2.9025 | 0.0039 | 0.0077 | -2.8214 |
| RPS6KC1 | -0.1079 | 5.6488 | -2.9011 | 0.0039 | 0.0077 | -2.8254 |
| DOK5 | 0.3492 | 3.3100 | 2.9007 | 0.0039 | 0.0077 | -2.8265 |
| EMX1 | -0.5251 | 2.2254 | -2.9006 | 0.0039 | 0.0077 | -2.8268 |
| EPN2 | -0.1266 | 5.0223 | -2.9005 | 0.0039 | 0.0077 | -2.8271 |
| SLAMF7 | -0.3537 | 4.2363 | -2.8987 | 0.0040 | 0.0078 | -2.8321 |
| WDR77 | -0.0707 | 6.2604 | -2.8984 | 0.0040 | 0.0078 | -2.8329 |
| TIGD6 | 0.0963 | 4.9479 | 2.8984 | 0.0040 | 0.0078 | -2.8331 |
| MLLT11 | -0.3152 | 4.3478 | -2.8968 | 0.0040 | 0.0078 | -2.8375 |
| NCALD | -0.3016 | 5.0239 | -2.8962 | 0.0040 | 0.0078 | -2.8391 |
| SH3BGRL3 | -0.0688 | 7.1744 | -2.8956 | 0.0040 | 0.0079 | -2.8409 |
| SUZ12 | -0.0960 | 5.9691 | -2.8946 | 0.0040 | 0.0079 | -2.8438 |
| CTRB1 | -0.2769 | 0.3896 | -2.8943 | 0.0040 | 0.0079 | -2.8446 |
| HRASLS | -0.3175 | 0.9719 | -2.8938 | 0.0040 | 0.0079 | -2.8461 |
| MVD | 0.1193 | 6.5620 | 2.8934 | 0.0040 | 0.0079 | -2.8471 |
| RPL36 | 0.0674 | 7.5271 | 2.8921 | 0.0041 | 0.0079 | -2.8507 |
| ZBP1 | -0.3624 | 2.2349 | -2.8910 | 0.0041 | 0.0080 | -2.8539 |
| MZT2A | -0.1097 | 6.2950 | -2.8908 | 0.0041 | 0.0080 | -2.8545 |
| NBPF1 | -0.2054 | 4.1344 | -2.8901 | 0.0041 | 0.0080 | -2.8563 |
| PSMD7 | 0.0585 | 6.7685 | 2.8891 | 0.0041 | 0.0080 | -2.8592 |
| LDLRAP1 | 0.0963 | 6.1673 | 2.8887 | 0.0041 | 0.0080 | -2.8602 |
| TP53BP2 | 0.1091 | 6.3943 | 2.8875 | 0.0041 | 0.0080 | -2.8636 |
| COG8 | 0.0852 | 5.5067 | 2.8874 | 0.0041 | 0.0080 | -2.8640 |
| CHD8 | -0.0747 | 5.8775 | -2.8859 | 0.0041 | 0.0081 | -2.8682 |
| CLK1 | 0.0838 | 6.3561 | 2.8857 | 0.0041 | 0.0081 | -2.8687 |
| ETS1 | 0.1465 | 6.0055 | 2.8851 | 0.0041 | 0.0081 | -2.8705 |
| CLN5 | 0.1126 | 5.5047 | 2.8848 | 0.0041 | 0.0081 | -2.8712 |
| WIPF2 | -0.0783 | 5.9118 | -2.8836 | 0.0042 | 0.0081 | -2.8747 |
| NOS1AP | 0.2990 | 3.1670 | 2.8833 | 0.0042 | 0.0081 | -2.8754 |
| RPS6KA2 | 0.2180 | 5.0314 | 2.8830 | 0.0042 | 0.0081 | -2.8763 |
| GLB1L | -0.1697 | 5.2614 | -2.8827 | 0.0042 | 0.0082 | -2.8772 |
| GAL | -0.4959 | 1.2753 | -2.8823 | 0.0042 | 0.0082 | -2.8783 |
| SPRR1B | -0.4209 | 0.6127 | -2.8819 | 0.0042 | 0.0082 | -2.8795 |
| PRKACA | 0.0693 | 6.7780 | 2.8818 | 0.0042 | 0.0082 | -2.8797 |
| ST14 | -0.2774 | 6.1145 | -2.8807 | 0.0042 | 0.0082 | -2.8829 |
| MT3 | -0.5439 | 1.5987 | -2.8783 | 0.0042 | 0.0083 | -2.8895 |
| CREM | 0.0851 | 5.8919 | 2.8780 | 0.0042 | 0.0083 | -2.8902 |
| KCNC1 | -0.2585 | 0.5411 | -2.8780 | 0.0042 | 0.0083 | -2.8904 |

| GOSR2 | -0.0703 | 5.7888 | -2.8773 | 0.0042 | 0.0083 | -2.8924 |
| --- | --- | --- | --- | --- | --- | --- |
| PRR14L | -0.1243 | 5.3073 | -2.8771 | 0.0042 | 0.0083 | -2.8927 |
| C19orf24 | 0.0830 | 6.9337 | 2.8770 | 0.0042 | 0.0083 | -2.8931 |
| FAM136A | -0.0632 | 6.5949 | -2.8766 | 0.0043 | 0.0083 | -2.8942 |
| HEATR1 | -0.1741 | 5.4945 | -2.8753 | 0.0043 | 0.0083 | -2.8978 |
| ICMT | -0.0598 | 6.4566 | -2.8753 | 0.0043 | 0.0083 | -2.8978 |
| IQCG | -0.1884 | 3.9762 | -2.8737 | 0.0043 | 0.0084 | -2.9022 |
| PPP1R14B | -0.0808 | 6.9769 | -2.8731 | 0.0043 | 0.0084 | -2.9040 |
| JAK2 | -0.1961 | 4.5298 | -2.8725 | 0.0043 | 0.0084 | -2.9056 |
| PFDN4 | -0.0978 | 6.1537 | -2.8723 | 0.0043 | 0.0084 | -2.9062 |
| GPSM3 | -0.1486 | 6.0077 | -2.8710 | 0.0043 | 0.0084 | -2.9098 |
| OR7C1 | -0.2839 | 0.3472 | -2.8707 | 0.0043 | 0.0084 | -2.9107 |
| TAF1D | -0.1014 | 5.9128 | -2.8703 | 0.0043 | 0.0084 | -2.9117 |
| DNAH3 | -0.1957 | 0.4837 | -2.8686 | 0.0044 | 0.0085 | -2.9165 |
| AGGF1 | 0.0649 | 6.1149 | 2.8682 | 0.0044 | 0.0085 | -2.9176 |
| ZNF667 | -0.3880 | 1.9344 | -2.8678 | 0.0044 | 0.0085 | -2.9187 |
| TMEM39A | -0.0804 | 5.8520 | -2.8677 | 0.0044 | 0.0085 | -2.9191 |
| PEX1 | 0.1410 | 5.4225 | 2.8660 | 0.0044 | 0.0085 | -2.9238 |
| GNPTAB | -0.1165 | 5.4002 | -2.8658 | 0.0044 | 0.0086 | -2.9244 |
| TGIF2 | -0.1239 | 6.0589 | -2.8656 | 0.0044 | 0.0086 | -2.9249 |
| SCN1A | -0.2696 | 0.4692 | -2.8655 | 0.0044 | 0.0086 | -2.9251 |
| MAGEA12 | -0.7448 | 1.7648 | -2.8641 | 0.0044 | 0.0086 | -2.9292 |
| DDX1 | 0.0408 | 6.8733 | 2.8636 | 0.0044 | 0.0086 | -2.9304 |
| UBE3A | 0.0731 | 5.9201 | 2.8613 | 0.0045 | 0.0087 | -2.9369 |
| MUL1 | 0.0590 | 6.6165 | 2.8609 | 0.0045 | 0.0087 | -2.9380 |
| LRRTM2 | 0.1786 | 0.8153 | 2.8601 | 0.0045 | 0.0087 | -2.9402 |
| TBL3 | 0.0740 | 6.2166 | 2.8599 | 0.0045 | 0.0087 | -2.9409 |
| ING1 | 0.1172 | 5.5706 | 2.8596 | 0.0045 | 0.0087 | -2.9415 |
| MBIP | 0.1190 | 5.7509 | 2.8588 | 0.0045 | 0.0087 | -2.9439 |
| KIF21B | -0.3394 | 4.0706 | -2.8570 | 0.0045 | 0.0088 | -2.9488 |
| ZNF266 | -0.2018 | 5.4466 | -2.8550 | 0.0045 | 0.0088 | -2.9544 |
| C2CD2 | -0.1659 | 5.1650 | -2.8548 | 0.0045 | 0.0088 | -2.9549 |
| RETN | -0.4556 | 1.5841 | -2.8547 | 0.0045 | 0.0088 | -2.9553 |
| HSPB1 | 0.0664 | 7.7338 | 2.8533 | 0.0046 | 0.0089 | -2.9591 |
| DYNC1I2 | -0.0923 | 6.2314 | -2.8530 | 0.0046 | 0.0089 | -2.9600 |
| MAGEA9 | -0.2387 | 0.2146 | -2.8527 | 0.0046 | 0.0089 | -2.9607 |
| SIRT2 | 0.0654 | 6.4017 | 2.8527 | 0.0046 | 0.0089 | -2.9608 |
| LGMN | 0.0676 | 6.8968 | 2.8524 | 0.0046 | 0.0089 | -2.9616 |
| DIAPH2 | 0.1154 | 5.5281 | 2.8496 | 0.0046 | 0.0090 | -2.9695 |
| C19orf53 | 0.0611 | 7.3182 | 2.8494 | 0.0046 | 0.0090 | -2.9699 |
| MAP4K5 | -0.1294 | 5.3010 | -2.8486 | 0.0046 | 0.0090 | -2.9723 |
| EGR2 | -0.3807 | 3.7455 | -2.8485 | 0.0046 | 0.0090 | -2.9725 |
| SLIRP | 0.0871 | 6.6317 | 2.8480 | 0.0046 | 0.0090 | -2.9737 |
| UXS1 | -0.0865 | 5.9799 | -2.8476 | 0.0046 | 0.0090 | -2.9750 |
| TPPP | 0.3688 | 4.6366 | 2.8440 | 0.0047 | 0.0091 | -2.9848 |
| UGGT2 | -0.1139 | 5.1730 | -2.8432 | 0.0047 | 0.0091 | -2.9870 |
| POLD2 | 0.0632 | 7.0040 | 2.8427 | 0.0047 | 0.0091 | -2.9885 |
| GFI1 | -0.3582 | 3.2670 | -2.8423 | 0.0047 | 0.0092 | -2.9897 |
| PTGFR | -0.6224 | 3.3436 | -2.8421 | 0.0047 | 0.0092 | -2.9900 |
| AZI2 | -0.1569 | 5.1424 | -2.8420 | 0.0047 | 0.0092 | -2.9905 |
| PILRA | -0.1917 | 5.0092 | -2.8416 | 0.0047 | 0.0092 | -2.9915 |
| SIN3B | -0.0888 | 5.6436 | -2.8412 | 0.0047 | 0.0092 | -2.9926 |
| KLF7 | -0.2994 | 4.0920 | -2.8412 | 0.0047 | 0.0092 | -2.9926 |

| GPR87 | -0.2137 | 0.3437 | -2.8411 | 0.0047 | 0.0092 | -2.9930 |
| --- | --- | --- | --- | --- | --- | --- |
| CTNNA2 | 0.6462 | 1.8058 | 2.8405 | 0.0048 | 0.0092 | -2.9946 |
| LEPROTL1 | -0.0909 | 6.0821 | -2.8403 | 0.0048 | 0.0092 | -2.9951 |
| SYF2 | 0.0537 | 6.5498 | 2.8400 | 0.0048 | 0.0092 | -2.9959 |
| ELF3 | -0.2154 | 6.3740 | -2.8392 | 0.0048 | 0.0092 | -2.9980 |
| CCR6 | -0.2569 | 0.7625 | -2.8391 | 0.0048 | 0.0092 | -2.9984 |
| PLD2 | -0.1048 | 5.6378 | -2.8391 | 0.0048 | 0.0092 | -2.9985 |
| MALT1 | -0.1388 | 5.2216 | -2.8390 | 0.0048 | 0.0092 | -2.9986 |
| CXCR2 | -0.3131 | 1.5314 | -2.8385 | 0.0048 | 0.0092 | -3.0000 |
| ERF | 0.0656 | 6.7637 | 2.8366 | 0.0048 | 0.0093 | -3.0055 |
| CDHR1 | -0.2984 | 1.2189 | -2.8353 | 0.0048 | 0.0093 | -3.0089 |
| SERPINB5 | -0.3268 | 0.6074 | -2.8345 | 0.0048 | 0.0093 | -3.0112 |
| SHARPIN | 0.0717 | 6.9829 | 2.8344 | 0.0048 | 0.0093 | -3.0115 |
| EDN2 | -0.4582 | 2.2884 | -2.8342 | 0.0048 | 0.0094 | -3.0119 |
| CKLF | -0.1136 | 6.0418 | -2.8333 | 0.0049 | 0.0094 | -3.0145 |
| METTL1 | -0.0922 | 6.1059 | -2.8326 | 0.0049 | 0.0094 | -3.0164 |
| TAF15 | 0.0410 | 6.8305 | 2.8318 | 0.0049 | 0.0094 | -3.0187 |
| HIST1H2AL | -0.4140 | 1.8474 | -2.8316 | 0.0049 | 0.0094 | -3.0192 |
| SLC24A2 | -0.1013 | 0.1364 | -2.8315 | 0.0049 | 0.0094 | -3.0193 |
| HSD3B2 | 0.3602 | 1.2506 | 2.8313 | 0.0049 | 0.0094 | -3.0199 |
| LUZP4 | -0.3590 | 0.4760 | -2.8306 | 0.0049 | 0.0094 | -3.0219 |
| GOLIM4 | 0.1055 | 6.5946 | 2.8291 | 0.0049 | 0.0095 | -3.0261 |
| STXBP1 | -0.2664 | 4.6779 | -2.8287 | 0.0049 | 0.0095 | -3.0271 |
| ZNF764 | -0.0889 | 5.5494 | -2.8281 | 0.0049 | 0.0095 | -3.0287 |
| CIC | -0.0744 | 6.1878 | -2.8273 | 0.0049 | 0.0095 | -3.0310 |
| DIS3 | 0.1006 | 5.4621 | 2.8268 | 0.0050 | 0.0095 | -3.0323 |
| KCNH4 | -0.3400 | 1.9077 | -2.8266 | 0.0050 | 0.0095 | -3.0329 |
| BTN3A3 | 0.1645 | 5.6138 | 2.8266 | 0.0050 | 0.0095 | -3.0329 |
| CNOT8 | 0.0534 | 6.3236 | 2.8263 | 0.0050 | 0.0096 | -3.0337 |
| COMT | 0.0977 | 6.8727 | 2.8263 | 0.0050 | 0.0096 | -3.0338 |
| TCF4 | 0.2179 | 4.1946 | 2.8260 | 0.0050 | 0.0096 | -3.0346 |
| ERCC3 | -0.0618 | 5.8794 | -2.8240 | 0.0050 | 0.0096 | -3.0400 |
| PAPD7 | 0.0859 | 5.9632 | 2.8234 | 0.0050 | 0.0096 | -3.0416 |
| KIAA0368 | 0.0532 | 6.4781 | 2.8227 | 0.0050 | 0.0097 | -3.0437 |
| GALC | -0.2682 | 4.8671 | -2.8223 | 0.0050 | 0.0097 | -3.0447 |
[truncated: 370,496 more chars]
