# Supplementary material for: Hypoxia Molecular Characterization in Hepatocellular Carcinoma Identifies One Risk Signature and Two Nomograms for Clinical Management
Source: J Oncol. 2021 Jan 20;2021:6664386. doi: 10.1155/2021/6664386 (PMC7846409; doi:10.1155/2021/6664386)
Supplement: Supplementary Materials — Figure S1: the identification of molecular subtypes in metacohort. (a) Using the unsupervised clustering algorithm to classify patients into different molecular subtypes in metacohort. The consensus score matrix of 831 HCC samples (K = 2∼9). A higher consensus score between two samples indicates they were more likely to be grouped into the same cluster in different iterations. The figure demonstrated k = 2 was the best choice. (b) The proportion of ambiguous clustering (PAC) score, a low value of PAC implied a flat middle segment in cumulative distribution functions (CDFs), allowing conjecture of the optimal k (k = 2) by the lowest PAC. (c) Recommended number of clusters using 26 criteria of Nbclust package in the metacohort. The highest of the column represented the optimal k (k = 2). Figure S2: the differences of HAG expression, known signatures, and fibroblast infiltration between C1 and C2 in metacohort. (a) The expression heatmap of 24 HAGs between C1 and C2. High expression, red; low expression, blue. (b) The expression box plot of 24 HAGs between C1 and C2. (c) Comparison of the signatures score between C1 and C2. (d) The abundance of fibroblasts was compared between C1 and C2. ns, P > 0.05; ∗P < 0.05; ∗∗P < 0.01; ∗∗∗P < 0.001. Figure S3: the difference of immune checkpoints (ICPs) and immune cells between C1 and C2. (a) The expression boxplot of ICPs between C1 and C2. ns, P > 0.05; ∗P < 0.05; ∗∗P < 0.01; ∗∗∗P < 0.001. (b) The heatmap of 23 immune cells between C1 and C2. High expression, red; low expression, blue. (c) Correlations between immune cells and HAGs using Spearman analysis. Negative correlation was marked with blue, and positive correlation was marked with red. No asterisks represented no statistical significance; ∗P < 0.05; ∗∗P < 0.01. Figure S4: the mutation signatures and significantly mutated genes (SMGs) in TCGA-LIHC cohort. (a) Comparison of tumor mutation burden (TMB) between C1 and C2. (b) The expression difference of 12 SMGs between muta [file 6664386.f1.zip › 6664386.f1/Table S8.docx]

| **Table S8: The univariable Cox regression and ROC analysis of each DEGs.**  ****The key genes are marked with bold.*** | | | | | | | |
| --- | --- | --- | --- | --- | --- | --- | --- |
| **ID** | **HR** | **95%CI** | **Z** | **P-value** | **AUC** | **Rank in AUC** | **Key Gene** |
| **GTSE1** | 2.0250 | (1.334~3.075) | 3.3110 | 0.0009 | 0.7200 | 1th | Yes |
| **CENPA** | 2.3740 | (1.573~3.584) | 4.1168 | 0.0000 | 0.7139 | 2th | Yes |
| **NEIL3** | 1.9690 | (1.405~2.759) | 3.9366 | 0.0001 | 0.7024 | 3th | Yes |
| **CLSPN** | 1.9070 | (1.35~2.694) | 3.6640 | 0.0002 | 0.6969 | 4th | Yes |
| **KIF18B** | 1.8850 | (1.284~2.768) | 3.2360 | 0.0012 | 0.6969 | 5th | Yes |
| **CEP55** | 2.5610 | (1.625~4.039) | 4.0491 | 0.0001 | 0.6941 | 6th | Yes |
| **KIF18A** | 1.9520 | (1.335~2.854) | 3.4522 | 0.0006 | 0.6899 | 7th | Yes |
| **KIF15** | 1.7110 | (1.214~2.412) | 3.0669 | 0.0022 | 0.6883 | 8th | Yes |
| **MCM10** | 2.0420 | (1.399~2.978) | 3.7045 | 0.0002 | 0.6851 | 9th | Yes |
| KIF23 | 1.9410 | (1.283~2.935) | 3.1409 | 0.0017 | 0.6812 | 10th | No |
| **RAD54L** | 1.8240 | (1.272~2.616) | 3.2656 | 0.0011 | 0.6746 | 11th | Yes |
| MMP10 | 1.5480 | (1.202~1.995) | 3.3851 | 0.0007 | 0.6627 | 12th | No |
| GCNT3 | 1.4160 | (1.102~1.82) | 2.7215 | 0.0065 | 0.6596 | 13th | No |
| IGF2BP3 | 1.2250 | (0.98~1.532) | 1.7860 | 0.0741 | 0.6582 | 14th | No |
| EGLN3 | 1.9540 | (1.411~2.706) | 4.0341 | 0.0001 | 0.6579 | 15th | No |
| MMP1 | 1.5930 | (1.239~2.049) | 3.6316 | 0.0003 | 0.6567 | 16th | No |
| TYRO3 | 1.5580 | (1.154~2.105) | 2.8917 | 0.0038 | 0.6491 | 17th | No |
| CA9 | 1.4870 | (1.228~1.8) | 4.0606 | 0.0000 | 0.6448 | 18th | No |
| ZNF239 | 1.6710 | (1.224~2.282) | 3.2329 | 0.0012 | 0.6383 | 19th | No |
| ETV4 | 1.4880 | (1.135~1.95) | 2.8810 | 0.0040 | 0.6263 | 20th | No |
| NR0B1 | 1.5580 | (1.271~1.91) | 4.2651 | 0.0000 | 0.6245 | 21th | No |
| USH1C | 1.2390 | (1.039~1.477) | 2.3891 | 0.0169 | 0.6187 | 22th | No |
| PPFIA4 | 1.5770 | (1.133~2.195) | 2.6987 | 0.0070 | 0.6185 | 23th | No |
| NRCAM | 1.2580 | (1.022~1.547) | 2.1701 | 0.0300 | 0.6183 | 24th | No |
| KCNF1 | 1.2850 | (1.029~1.605) | 2.2138 | 0.0268 | 0.6164 | 25th | No |
| CYP26B1 | 1.4320 | (1.111~1.846) | 2.7702 | 0.0056 | 0.6158 | 26th | No |
| FCGBP | 1.4680 | (1.048~2.059) | 2.2292 | 0.0258 | 0.6154 | 27th | No |
| EPS8L3 | 1.2310 | (0.967~1.568) | 1.6872 | 0.0916 | 0.6152 | 28th | No |
| EPO | 1.4060 | (1.165~1.696) | 3.5528 | 0.0004 | 0.6086 | 29th | No |
| C3orf52 | 1.9130 | (1.353~2.703) | 3.6742 | 0.0002 | 0.6079 | 30th | No |
| TTC39A | 1.4680 | (1.081~1.993) | 2.4583 | 0.0140 | 0.6072 | 31th | No |
| RIBC2 | 1.4040 | (1.022~1.93) | 2.0925 | 0.0364 | 0.6048 | 32th | No |
| GAL3ST | 1.1640 | (0.905~1.498) | 1.1854 | 0.2359 | 0.6025 | 33th | No |
| LIF | 1.1810 | (0.868~1.607) | 1.0607 | 0.2888 | 0.6022 | 34th | No |
| FHOD3 | 1.4440 | (1.08~1.933) | 2.4749 | 0.0133 | 0.6019 | 35th | No |
| DIRAS2 | 1.3570 | (1.069~1.722) | 2.5102 | 0.0121 | 0.6016 | 36th | No |
| CLEC5A | 1.8110 | (1.248~2.627) | 3.1274 | 0.0018 | 0.6014 | 37th | No |
| FABP6 | 1.4930 | (1.19~1.873) | 3.4640 | 0.0005 | 0.6008 | 38th | No |
| HK2 | 1.4310 | (1.069~1.915) | 2.4099 | 0.0160 | 0.6000 | 39th | No |
| PAEP | 1.3310 | (1.128~1.571) | 3.3846 | 0.0007 | 0.5994 | 40th | No |
| PTGES | 1.1940 | (0.947~1.506) | 1.4982 | 0.1341 | 0.5986 | 41th | No |
| MMP12 | 1.3180 | (1.067~1.629) | 2.5652 | 0.0103 | 0.5972 | 42th | No |
| PAQR5 | 1.2130 | (0.938~1.569) | 1.4704 | 0.1414 | 0.5956 | 43th | No |
| POF1B | 1.4230 | (1.152~1.758) | 3.2735 | 0.0011 | 0.5933 | 44th | No |
| MEP1A | 1.1720 | (0.984~1.397) | 1.7765 | 0.0756 | 0.5927 | 45th | No |
| SALL2 | 1.0490 | (0.795~1.385) | 0.3383 | 0.7351 | 0.5921 | 46th | No |
| CLDN4 | 1.1790 | (0.938~1.481) | 1.4110 | 0.1582 | 0.5889 | 47th | No |
| CTSE | 1.2740 | (1.015~1.6) | 2.0921 | 0.0364 | 0.5877 | 48th | No |

| MMP7 | 1.3450 | (1.098~1.647) | 2.8665 | 0.0042 | 0.5871 | 49th | No |
| --- | --- | --- | --- | --- | --- | --- | --- |
| NXPH4 | 1.4360 | (1.099~1.877) | 2.6529 | 0.0080 | 0.5871 | 50th | No |
| IGF2BP2 | 1.3090 | (0.999~1.715) | 1.9562 | 0.0504 | 0.5869 | 51th | No |
| SPHK1 | 1.2970 | (0.945~1.78) | 1.6071 | 0.1080 | 0.5863 | 52th | No |
| NPPB | 1.1930 | (0.932~1.528) | 1.4000 | 0.1615 | 0.5861 | 53th | No |
| PITX1 | 1.0810 | (0.9~1.297) | 0.8333 | 0.4047 | 0.5836 | 54th | No |
| FOLR1 | 1.2820 | (1.027~1.601) | 2.1957 | 0.0281 | 0.5831 | 55th | No |
| SLC6A8 | 1.2140 | (0.876~1.683) | 1.1657 | 0.2438 | 0.5807 | 56th | No |
| TNNT1 | 1.2610 | (1.008~1.577) | 2.0254 | 0.0428 | 0.5796 | 57th | No |
| TFF2 | 1.3220 | (1.104~1.583) | 3.0365 | 0.0024 | 0.5785 | 58th | No |
| INPP5J | 1.1660 | (0.82~1.658) | 0.8537 | 0.3933 | 0.5780 | 59th | No |
| CTAG2 | 1.1890 | (1.036~1.365) | 2.4708 | 0.0135 | 0.5772 | 60th | No |
| CXCL5 | 1.3540 | (1.114~1.645) | 3.0452 | 0.0023 | 0.5756 | 61th | No |
| LRP8 | 1.4950 | (0.992~2.253) | 1.9232 | 0.0545 | 0.5755 | 62th | No |
| AGR2 | 1.3310 | (1.125~1.574) | 3.3415 | 0.0008 | 0.5753 | 63th | No |
| SLC7A1 | 1.4880 | (1.043~2.124) | 2.1892 | 0.0286 | 0.5752 | 64th | No |
| ADORA1 | 1.0770 | (0.827~1.403) | 0.5481 | 0.5836 | 0.5745 | 65th | No |
| COL4A5 | 1.0880 | (0.87~1.361) | 0.7395 | 0.4596 | 0.5742 | 66th | No |
| CXCL1 | 1.3320 | (1.059~1.675) | 2.4511 | 0.0142 | 0.5738 | 67th | No |
| PPP1R14 | 1.1150 | (0.887~1.4) | 0.9335 | 0.3506 | 0.5737 | 68th | No |
| DNM1 | 1.3740 | (0.978~1.93) | 1.8335 | 0.0667 | 0.5732 | 69th | No |
| MPP2 | 1.4180 | (0.935~2.149) | 1.6439 | 0.1002 | 0.5713 | 70th | No |
| ENO2 | 1.4230 | (0.943~2.145) | 1.6820 | 0.0926 | 0.5710 | 71th | No |
| PKIA | 1.3270 | (0.968~1.818) | 1.7596 | 0.0785 | 0.5700 | 72th | No |
| NPTX2 | 1.1320 | (0.925~1.386) | 1.2058 | 0.2279 | 0.5699 | 73th | No |
| TNFAIP6 | 1.1990 | (0.886~1.623) | 1.1757 | 0.2397 | 0.5692 | 74th | No |
| PTHLH | 1.2360 | (0.941~1.623) | 1.5232 | 0.1277 | 0.5689 | 75th | No |
| HAVCR1 | 1.3430 | (1.061~1.7) | 2.4553 | 0.0141 | 0.5675 | 76th | No |
| FOXJ1 | 1.1540 | (0.926~1.437) | 1.2757 | 0.2021 | 0.5666 | 77th | No |
| UPK3A | 1.0660 | (0.896~1.267) | 0.7171 | 0.4733 | 0.5665 | 78th | No |
| IL4I1 | 1.3990 | (0.962~2.034) | 1.7547 | 0.0793 | 0.5662 | 79th | No |
| TMC5 | 1.1930 | (0.965~1.474) | 1.6319 | 0.1027 | 0.5609 | 80th | No |
| PEG10 | 1.0630 | (0.897~1.26) | 0.7066 | 0.4798 | 0.5608 | 81th | No |
| IBSP | 1.1120 | (0.89~1.39) | 0.9321 | 0.3513 | 0.5603 | 82th | No |
| ADAM12 | 1.4200 | (1.084~1.861) | 2.5428 | 0.0110 | 0.5592 | 83th | No |
| S100A2 | 1.4310 | (1.092~1.876) | 2.5987 | 0.0094 | 0.5584 | 84th | No |
| GUCA2A | 0.9910 | (0.806~1.219) | -0.0852 | 0.9321 | 0.5583 | 85th | No |
| GRAMD | 1.2860 | (0.968~1.708) | 1.7361 | 0.0825 | 0.5572 | 86th | No |
| COL9A2 | 1.1550 | (0.787~1.696) | 0.7361 | 0.4617 | 0.5572 | 87th | No |
| FAM155 | 1.1980 | (0.932~1.54) | 1.4103 | 0.1585 | 0.5544 | 88th | No |
| FXYD3 | 1.2040 | (0.964~1.503) | 1.6385 | 0.1013 | 0.5540 | 89th | No |
| MYEF2 | 1.1340 | (0.88~1.462) | 0.9698 | 0.3321 | 0.5523 | 90th | No |
| ZNF711 | 1.1790 | (0.874~1.59) | 1.0804 | 0.2800 | 0.5522 | 91th | No |
| ADORA2 | 1.1640 | (0.813~1.666) | 0.8272 | 0.4081 | 0.5513 | 92th | No |
| SLCO4C | 1.1990 | (0.979~1.469) | 1.7547 | 0.0793 | 0.5502 | 93th | No |
| NEB | 1.0990 | (0.849~1.421) | 0.7161 | 0.4739 | 0.5494 | 94th | No |
| PLEKHB | 1.1560 | (0.89~1.501) | 1.0886 | 0.2763 | 0.5488 | 95th | No |
| LYPD1 | 1.1600 | (0.907~1.485) | 1.1818 | 0.2373 | 0.5485 | 96th | No |
| PCSK1N | 1.0970 | (0.92~1.308) | 1.0312 | 0.3025 | 0.5485 | 97th | No |
| FRAS1 | 1.1970 | (0.939~1.526) | 1.4554 | 0.1456 | 0.5471 | 98th | No |
| DKK1 | 1.0640 | (0.893~1.268) | 0.6923 | 0.4887 | 0.5469 | 99th | No |
| ITGB6 | 1.3980 | (1.001~1.953) | 1.9629 | 0.0497 | 0.5468 | 100th | No |
| CA12 | 1.0950 | (0.888~1.351) | 0.8491 | 0.3958 | 0.5467 | 101th | No |

| NTS | 1.0920 | (0.903~1.32) | 0.9096 | 0.3630 | 0.5453 | 102th | No |
| --- | --- | --- | --- | --- | --- | --- | --- |
| ARNT2 | 1.0450 | (0.794~1.375) | 0.3150 | 0.7527 | 0.5448 | 103th | No |
| PRAME | 1.1200 | (0.948~1.324) | 1.3355 | 0.1817 | 0.5445 | 104th | No |
| ITPR3 | 1.1920 | (0.83~1.713) | 0.9522 | 0.3410 | 0.5427 | 105th | No |
| CXCL3 | 1.2850 | (0.933~1.769) | 1.5371 | 0.1243 | 0.5386 | 106th | No |
| PI3 | 1.1250 | (0.929~1.361) | 1.2060 | 0.2278 | 0.5384 | 107th | No |
| CXCL6 | 1.1760 | (0.979~1.412) | 1.7302 | 0.0836 | 0.5379 | 108th | No |
| FCHO1 | 1.1320 | (0.768~1.668) | 0.6246 | 0.5322 | 0.5377 | 109th | No |
| TFF1 | 1.1850 | (0.997~1.409) | 1.9222 | 0.0546 | 0.5354 | 110th | No |
| HUNK | 1.0730 | (0.815~1.412) | 0.5032 | 0.6148 | 0.5345 | 111th | No |
| MAPK13 | 1.3380 | (0.969~1.849) | 1.7692 | 0.0769 | 0.5339 | 112th | No |
| MFAP2 | 1.2820 | (0.944~1.741) | 1.5922 | 0.1113 | 0.5328 | 113th | No |
| GJB3 | 1.4530 | (1.113~1.898) | 2.7430 | 0.0061 | 0.5304 | 114th | No |
| BICC1 | 1.0890 | (0.843~1.407) | 0.6518 | 0.5145 | 0.5284 | 115th | No |
| OBSCN | 1.1600 | (0.862~1.561) | 0.9820 | 0.3261 | 0.5274 | 116th | No |
| SNAP25 | 1.0450 | (0.83~1.316) | 0.3731 | 0.7091 | 0.5274 | 117th | No |
| HTR3A | 1.3530 | (1.004~1.823) | 1.9832 | 0.0473 | 0.5261 | 118th | No |
| GAST | 1.2010 | (0.985~1.465) | 1.8074 | 0.0707 | 0.5252 | 119th | No |
| EPHB3 | 1.1160 | (0.789~1.577) | 0.6198 | 0.5354 | 0.5251 | 120th | No |
| AREG | 1.0150 | (0.766~1.345) | 0.1048 | 0.9165 | 0.5228 | 121th | No |
| MCOLN3 | 1.1840 | (0.926~1.513) | 1.3455 | 0.1785 | 0.5218 | 122th | No |
| LEFTY1 | 1.1210 | (0.856~1.469) | 0.8299 | 0.4066 | 0.5214 | 123th | No |
| PODXL2 | 1.0120 | (0.804~1.273) | 0.0997 | 0.9206 | 0.5211 | 124th | No |
| KCNH2 | 1.1920 | (0.93~1.528) | 1.3868 | 0.1655 | 0.5198 | 125th | No |
| LAMC2 | 1.0480 | (0.798~1.376) | 0.3362 | 0.7367 | 0.5194 | 126th | No |
| APLP1 | 1.2820 | (0.984~1.67) | 1.8384 | 0.0660 | 0.5190 | 127th | No |
| VTCN1 | 1.0590 | (0.835~1.344) | 0.4743 | 0.6353 | 0.5165 | 128th | No |
| KLHDC8 | 1.0190 | (0.745~1.393) | 0.1164 | 0.9074 | 0.5159 | 129th | No |
| DLG3 | 0.8590 | (0.584~1.265) | -0.7680 | 0.4425 | 0.5158 | 130th | No |
| SIX2 | 1.0500 | (0.833~1.323) | 0.4134 | 0.6793 | 0.5157 | 131th | No |
| GLRB | 1.0390 | (0.838~1.288) | 0.3484 | 0.7275 | 0.5149 | 132th | No |
| CDH17 | 1.0650 | (0.804~1.412) | 0.4417 | 0.6587 | 0.5147 | 133th | No |
| ARL14 | 1.2020 | (0.947~1.527) | 1.5123 | 0.1305 | 0.5113 | 134th | No |
| SLC34A2 | 1.1390 | (0.911~1.423) | 1.1406 | 0.2540 | 0.5103 | 135th | No |
| ZFY | 0.8740 | (0.743~1.028) | -1.6272 | 0.1037 | 0.5092 | 136th | No |
| SFRP5 | 0.9900 | (0.82~1.194) | -0.1094 | 0.9129 | 0.5092 | 137th | No |
| SPDEF | 1.0450 | (0.828~1.319) | 0.3709 | 0.7107 | 0.5088 | 138th | No |
| GULP1 | 1.3060 | (1.022~1.669) | 2.1310 | 0.0331 | 0.5080 | 139th | No |
| LARP6 | 1.0360 | (0.741~1.45) | 0.2077 | 0.8355 | 0.5079 | 140th | No |
| GP2 | 1.0180 | (0.84~1.234) | 0.1781 | 0.8586 | 0.5074 | 141th | No |
| SUSD4 | 1.1570 | (0.939~1.425) | 1.3715 | 0.1702 | 0.5074 | 142th | No |
| SYT13 | 1.0870 | (0.871~1.356) | 0.7380 | 0.4605 | 0.5072 | 143th | No |
| EPN3 | 0.9460 | (0.656~1.366) | -0.2937 | 0.7690 | 0.5067 | 144th | No |
| DDX3Y | 0.8940 | (0.782~1.023) | -1.6285 | 0.1034 | 0.5060 | 145th | No |
| KRT19 | 0.9330 | (0.771~1.13) | -0.7070 | 0.4796 | 0.5057 | 146th | No |
| IL20RA | 1.0990 | (0.854~1.415) | 0.7345 | 0.4626 | 0.5057 | 147th | No |
| ANXA13 | 1.1910 | (0.916~1.55) | 1.3070 | 0.1912 | 0.5033 | 148th | No |
| TMPRSS | 1.0020 | (0.801~1.253) | 0.0176 | 0.9860 | 0.5029 | 149th | No |
| C1orf116 | 0.8890 | (0.674~1.172) | -0.8340 | 0.4043 | 0.5025 | 150th | No |
| MST1R | 1.2300 | (0.911~1.659) | 1.3526 | 0.1762 | 0.5025 | 151th | No |
| PKP3 | 1.0840 | (0.843~1.395) | 0.6310 | 0.5281 | 0.5013 | 152th | No |
| EFNA5 | 1.3660 | (1.076~1.734) | 2.5590 | 0.0105 | 0.5009 | 153th | No |
| SYNGR3 | 0.8370 | (0.575~1.218) | -0.9311 | 0.3518 | 0.4981 | 154th | No |

| PDX1 | 1.1010 | (0.926~1.307) | 1.0898 | 0.2758 | 0.4981 | 155th | No |
| --- | --- | --- | --- | --- | --- | --- | --- |
| SCGB2A | 0.9830 | (0.785~1.231) | -0.1486 | 0.8819 | 0.4980 | 156th | No |
| FXYD2 | 1.0490 | (0.872~1.261) | 0.5046 | 0.6138 | 0.4949 | 157th | No |
| HOXB13 | 1.0830 | (0.854~1.374) | 0.6570 | 0.5112 | 0.4948 | 158th | No |
| DLX4 | 1.0860 | (0.781~1.512) | 0.4923 | 0.6225 | 0.4947 | 159th | No |
| SLC6A14 | 1.0220 | (0.745~1.402) | 0.1330 | 0.8942 | 0.4944 | 160th | No |
| FA2H | 1.1120 | (0.834~1.483) | 0.7243 | 0.4689 | 0.4942 | 161th | No |
| ASPHD1 | 1.1500 | (0.91~1.453) | 1.1694 | 0.2422 | 0.4933 | 162th | No |
| CTNND2 | 0.9460 | (0.785~1.141) | -0.5783 | 0.5631 | 0.4929 | 163th | No |
| HIST3H2 | 1.1120 | (0.869~1.421) | 0.8431 | 0.3992 | 0.4918 | 164th | No |
| SEMA3C | 1.0570 | (0.779~1.434) | 0.3555 | 0.7222 | 0.4906 | 165th | No |
| TPBG | 1.0900 | (0.769~1.545) | 0.4858 | 0.6271 | 0.4905 | 166th | No |
| CYP1A1 | 0.9740 | (0.831~1.143) | -0.3190 | 0.7498 | 0.4891 | 167th | No |
| HOXB9 | 1.0540 | (0.785~1.413) | 0.3477 | 0.7281 | 0.4880 | 168th | No |
| DUOX2 | 0.9360 | (0.755~1.162) | -0.5985 | 0.5495 | 0.4873 | 169th | No |
| CDCP1 | 0.9820 | (0.69~1.397) | -0.1019 | 0.9189 | 0.4865 | 170th | No |
| INHA | 0.9420 | (0.746~1.189) | -0.5039 | 0.6144 | 0.4859 | 171th | No |
| TCN1 | 0.8360 | (0.617~1.134) | -1.1518 | 0.2494 | 0.4843 | 172th | No |
| A4GNT | 1.0720 | (0.799~1.438) | 0.4654 | 0.6417 | 0.4840 | 173th | No |
| RASAL1 | 1.0040 | (0.75~1.345) | 0.0293 | 0.9767 | 0.4835 | 174th | No |
| BCL11A | 1.2460 | (0.873~1.779) | 1.2097 | 0.2264 | 0.4820 | 175th | No |
| MUC1 | 1.2110 | (0.901~1.629) | 1.2696 | 0.2042 | 0.4815 | 176th | No |
| ULBP1 | 1.0870 | (0.758~1.559) | 0.4533 | 0.6503 | 0.4793 | 177th | No |
| OLFM4 | 0.9230 | (0.685~1.243) | -0.5273 | 0.5980 | 0.4792 | 178th | No |
| B3GNT3 | 1.0390 | (0.822~1.312) | 0.3181 | 0.7504 | 0.4790 | 179th | No |
| PRSS22 | 0.9270 | (0.69~1.244) | -0.5070 | 0.6121 | 0.4781 | 180th | No |
| SSTR5 | 0.9840 | (0.81~1.195) | -0.1642 | 0.8696 | 0.4780 | 181th | No |
| KDM5D | 0.8410 | (0.728~0.971) | -2.3598 | 0.0183 | 0.4773 | 182th | No |
| SMPDL3 | 1.0430 | (0.788~1.38) | 0.2943 | 0.7685 | 0.4764 | 183th | No |
| EPHB6 | 1.0070 | (0.697~1.454) | 0.0358 | 0.9714 | 0.4750 | 184th | No |
| PRSS16 | 1.1070 | (0.861~1.422) | 0.7927 | 0.4280 | 0.4749 | 185th | No |
| CLDN10 | 0.9890 | (0.791~1.236) | -0.0975 | 0.9223 | 0.4729 | 186th | No |
| CAPN6 | 1.1970 | (0.97~1.476) | 1.6777 | 0.0934 | 0.4725 | 187th | No |
| GIPR | 0.9310 | (0.653~1.326) | -0.3975 | 0.6910 | 0.4723 | 188th | No |
| RPS4Y1 | 0.9000 | (0.792~1.022) | -1.6287 | 0.1034 | 0.4701 | 189th | No |
| GPR27 | 0.8730 | (0.674~1.131) | -1.0292 | 0.3034 | 0.4688 | 190th | No |
| EIF1AY | 0.8800 | (0.77~1.005) | -1.8916 | 0.0585 | 0.4667 | 191th | No |
| UTY | 0.8390 | (0.714~0.986) | -2.1246 | 0.0336 | 0.4661 | 192th | No |
| AQP8 | 0.9020 | (0.76~1.071) | -1.1759 | 0.2396 | 0.4640 | 193th | No |
| PAGE4 | 0.9350 | (0.815~1.072) | -0.9672 | 0.3335 | 0.4628 | 194th | No |
| CHST4 | 1.1260 | (0.909~1.395) | 1.0891 | 0.2761 | 0.4616 | 195th | No |
| TGFA | 0.9390 | (0.67~1.317) | -0.3629 | 0.7167 | 0.4595 | 196th | No |
| SCTR | 0.9150 | (0.688~1.217) | -0.6127 | 0.5401 | 0.4587 | 197th | No |
| SPIB | 0.9590 | (0.702~1.31) | -0.2627 | 0.7928 | 0.4557 | 198th | No |
| SPINT1 | 1.0270 | (0.774~1.362) | 0.1817 | 0.8558 | 0.4554 | 199th | No |
| EPCAM | 0.9190 | (0.76~1.11) | -0.8810 | 0.3783 | 0.4551 | 200th | No |
| GABRB3 | 1.0350 | (0.834~1.284) | 0.3097 | 0.7568 | 0.4534 | 201th | No |
| PAPPA2 | 0.9520 | (0.796~1.14) | -0.5306 | 0.5957 | 0.4519 | 202th | No |
| TMEM15 | 0.9710 | (0.718~1.313) | -0.1933 | 0.8467 | 0.4474 | 203th | No |
| EPS8L1 | 0.9610 | (0.701~1.318) | -0.2461 | 0.8056 | 0.4462 | 204th | No |
| OVOL2 | 0.9520 | (0.691~1.312) | -0.2978 | 0.7658 | 0.4461 | 205th | No |
| PRSS12 | 0.9280 | (0.722~1.192) | -0.5871 | 0.5571 | 0.4453 | 206th | No |
| CYP1A2 | 0.9130 | (0.79~1.056) | -1.2263 | 0.2201 | 0.4424 | 207th | No |

| SLC4A3 | 0.9090 | (0.692~1.194) | -0.6846 | 0.4936 | 0.4404 | 208th | No |
| --- | --- | --- | --- | --- | --- | --- | --- |
| NSUN7 | 0.9620 | (0.73~1.268) | -0.2734 | 0.7845 | 0.4399 | 209th | No |
| DSG1 | 0.8100 | (0.683~0.961) | -2.4115 | 0.0159 | 0.4396 | 210th | No |
| TRPV6 | 0.8450 | (0.585~1.22) | -0.8998 | 0.3682 | 0.4346 | 211th | No |
| RAB25 | 0.8060 | (0.625~1.039) | -1.6659 | 0.0957 | 0.4337 | 212th | No |
| WFDC2 | 1.0110 | (0.825~1.24) | 0.1084 | 0.9137 | 0.4319 | 213th | No |
| KCNJ16 | 1.1290 | (0.856~1.489) | 0.8569 | 0.3915 | 0.4312 | 214th | No |
| CA4 | 0.8610 | (0.716~1.035) | -1.5926 | 0.1112 | 0.4304 | 215th | No |
| WNT7B | 0.9960 | (0.743~1.336) | -0.0271 | 0.9784 | 0.4297 | 216th | No |
| DHRS2 | 0.8140 | (0.681~0.972) | -2.2711 | 0.0231 | 0.4276 | 217th | No |
| ABCB11 | 0.8750 | (0.727~1.053) | -1.4127 | 0.1577 | 0.4273 | 218th | No |
| DMBT1 | 0.8080 | (0.562~1.16) | -1.1558 | 0.2478 | 0.4271 | 219th | No |
| NEBL | 0.8990 | (0.69~1.171) | -0.7918 | 0.4285 | 0.4263 | 220th | No |
| KCNB1 | 0.8710 | (0.724~1.049) | -1.4548 | 0.1457 | 0.4242 | 221th | No |
| SEMA3E | 0.9950 | (0.769~1.287) | -0.0402 | 0.9679 | 0.4190 | 222th | No |
| ITGB8 | 0.8880 | (0.625~1.262) | -0.6612 | 0.5085 | 0.4185 | 223th | No |
| PROM1 | 0.9050 | (0.689~1.189) | -0.7164 | 0.4737 | 0.4176 | 224th | No |
| SLC1A2 | 0.7790 | (0.653~0.929) | -2.7794 | 0.0054 | 0.4175 | 225th | No |
| CEACAM | 0.8410 | (0.634~1.116) | -1.2012 | 0.2297 | 0.4174 | 226th | No |
| MYRIP | 0.7230 | (0.589~0.886) | -3.1254 | 0.0018 | 0.4163 | 227th | No |
| CFTR | 0.8470 | (0.661~1.086) | -1.3082 | 0.1908 | 0.4158 | 228th | No |
| CEACAM | 0.7400 | (0.547~1) | -1.9569 | 0.0504 | 0.4157 | 229th | No |
| NECAB2 | 0.8380 | (0.675~1.039) | -1.6116 | 0.1070 | 0.4136 | 230th | No |
| NR1I3 | 0.7700 | (0.591~1.003) | -1.9364 | 0.0528 | 0.4120 | 231th | No |
| MUC5B | 1.0050 | (0.84~1.203) | 0.0552 | 0.9560 | 0.4092 | 232th | No |
| ESRP1 | 0.8780 | (0.693~1.113) | -1.0741 | 0.2828 | 0.4070 | 233th | No |
| CNKSR1 | 0.8550 | (0.664~1.1) | -1.2203 | 0.2224 | 0.4064 | 234th | No |
| REN | 0.8350 | (0.696~1.002) | -1.9436 | 0.0519 | 0.4039 | 235th | No |
| CYP11A1 | 0.8000 | (0.673~0.951) | -2.5295 | 0.0114 | 0.4034 | 236th | No |
| SAA2-SA | 0.8850 | (0.741~1.057) | -1.3456 | 0.1784 | 0.4021 | 237th | No |
| CYP3A4 | 0.7520 | (0.623~0.907) | -2.9729 | 0.0029 | 0.4016 | 238th | No |
| GPRIN2 | 0.8550 | (0.609~1.201) | -0.9031 | 0.3665 | 0.3993 | 239th | No |
| TTYH1 | 0.6680 | (0.466~0.958) | -2.1912 | 0.0284 | 0.3987 | 240th | No |
| HAO2 | 0.7750 | (0.631~0.951) | -2.4414 | 0.0146 | 0.3961 | 241th | No |
| NAT2 | 0.9140 | (0.768~1.088) | -1.0090 | 0.3130 | 0.3946 | 242th | No |
| MUC6 | 0.9930 | (0.768~1.283) | -0.0568 | 0.9547 | 0.3942 | 243th | No |
| SRD5A2 | 0.7130 | (0.589~0.865) | -3.4432 | 0.0006 | 0.3921 | 244th | No |
| SLC6A13 | 0.7730 | (0.62~0.964) | -2.2861 | 0.0222 | 0.3920 | 245th | No |
| CYP7A1 | 0.7450 | (0.638~0.871) | -3.6945 | 0.0002 | 0.3877 | 246th | No |
| ABCA8 | 0.7600 | (0.618~0.936) | -2.5858 | 0.0097 | 0.3865 | 247th | No |
| ABCA6 | 0.6860 | (0.533~0.884) | -2.9161 | 0.0035 | 0.3829 | 248th | No |
| CD5L | 0.7150 | (0.602~0.851) | -3.7929 | 0.0001 | 0.3818 | 249th | No |
| TMEM10 | 0.6970 | (0.553~0.88) | -3.0327 | 0.0024 | 0.3797 | 250th | No |
| OTC | 0.7600 | (0.604~0.958) | -2.3247 | 0.0201 | 0.3763 | 251th | No |
| ACADL | 0.8230 | (0.691~0.981) | -2.1743 | 0.0297 | 0.3763 | 252th | No |
| CCL16 | 0.7790 | (0.628~0.968) | -2.2586 | 0.0239 | 0.3748 | 253th | No |
| AKR1D1 | 0.7180 | (0.584~0.883) | -3.1423 | 0.0017 | 0.3729 | 254th | No |
| CYP2A13 | 0.8560 | (0.722~1.015) | -1.7923 | 0.0731 | 0.3715 | 255th | No |
| GPLD1 | 0.6900 | (0.552~0.862) | -3.2649 | 0.0011 | 0.3712 | 256th | No |
| CYP2A7 | 0.8040 | (0.688~0.939) | -2.7502 | 0.0060 | 0.3711 | 257th | No |
| PCK1 | 0.6920 | (0.532~0.9) | -2.7427 | 0.0061 | 0.3702 | 258th | No |
| GCGR | 0.8210 | (0.697~0.967) | -2.3555 | 0.0185 | 0.3699 | 259th | No |
| MOGAT2 | 0.8320 | (0.7~0.989) | -2.0867 | 0.0369 | 0.3694 | 260th | No |

| ADRB2 | 0.6920 | (0.544~0.88) | -2.9994 | 0.0027 | 0.3690 | 261th | No |
| --- | --- | --- | --- | --- | --- | --- | --- |
| MNX1 | 0.7640 | (0.586~0.997) | -1.9800 | 0.0477 | 0.3684 | 262th | No |
| GLYAT | 0.8310 | (0.704~0.981) | -2.1819 | 0.0291 | 0.3677 | 263th | No |
| FETUB | 0.6930 | (0.55~0.873) | -3.1162 | 0.0018 | 0.3661 | 264th | No |
| CPS1 | 0.6850 | (0.552~0.85) | -3.4413 | 0.0006 | 0.3654 | 265th | No |
| CBLN1 | 0.7220 | (0.591~0.882) | -3.1894 | 0.0014 | 0.3642 | 266th | No |
| ADRA1A | 0.7280 | (0.603~0.88) | -3.2915 | 0.0010 | 0.3639 | 267th | No |
| CFHR5 | 0.7380 | (0.597~0.913) | -2.7947 | 0.0052 | 0.3630 | 268th | No |
| CYP3A43 | 0.6770 | (0.554~0.827) | -3.8127 | 0.0001 | 0.3597 | 269th | No |
| AR | 0.6940 | (0.555~0.868) | -3.1955 | 0.0014 | 0.3593 | 270th | No |
| APOF | 0.7080 | (0.569~0.881) | -3.0903 | 0.0020 | 0.3589 | 271th | No |
| LPA | 0.7110 | (0.577~0.876) | -3.2040 | 0.0014 | 0.3582 | 272th | No |
| FABP4 | 0.6880 | (0.544~0.87) | -3.1228 | 0.0018 | 0.3578 | 273th | No |
| PFKFB1 | 0.7220 | (0.563~0.926) | -2.5633 | 0.0104 | 0.3563 | 274th | No |
| ZG16 | 0.8040 | (0.663~0.975) | -2.2131 | 0.0269 | 0.3541 | 275th | No |
| CA5A | 0.7580 | (0.629~0.912) | -2.9298 | 0.0034 | 0.3534 | 276th | No |
| CYP2A6 | 0.7140 | (0.586~0.869) | -3.3554 | 0.0008 | 0.3530 | 277th | No |
| GFRA1 | 0.7800 | (0.653~0.932) | -2.7318 | 0.0063 | 0.3523 | 278th | No |
| NR1I2 | 0.7210 | (0.598~0.868) | -3.4524 | 0.0006 | 0.3522 | 279th | No |
| SLC5A1 | 0.6240 | (0.451~0.864) | -2.8449 | 0.0044 | 0.3520 | 280th | No |
| AQP9 | 0.6480 | (0.493~0.852) | -3.1098 | 0.0019 | 0.3518 | 281th | No |
| MASP2 | 0.6730 | (0.528~0.857) | -3.2039 | 0.0014 | 0.3514 | 282th | No |
| CYP4F2 | 0.7720 | (0.604~0.985) | -2.0778 | 0.0377 | 0.3505 | 283th | No |
| ACSM5 | 0.7860 | (0.605~1.021) | -1.8038 | 0.0713 | 0.3447 | 284th | No |
| DAO | 0.7720 | (0.602~0.991) | -2.0280 | 0.0426 | 0.3415 | 285th | No |
| LECT2 | 0.7220 | (0.61~0.853) | -3.8194 | 0.0001 | 0.3391 | 286th | No |
| TAT | 0.5830 | (0.446~0.761) | -3.9630 | 0.0001 | 0.3391 | 287th | No |
| ESR1 | 0.6470 | (0.515~0.812) | -3.7511 | 0.0002 | 0.3382 | 288th | No |
| F9 | 0.8340 | (0.672~1.037) | -1.6350 | 0.1020 | 0.3336 | 289th | No |
| STEAP4 | 0.6040 | (0.474~0.77) | -4.0742 | 0.0000 | 0.3334 | 290th | No |
| ACSM2A | 0.7480 | (0.592~0.945) | -2.4336 | 0.0149 | 0.3325 | 291th | No |
| SLC27A5 | 0.5250 | (0.375~0.736) | -3.7403 | 0.0002 | 0.3235 | 292th | No |
| CFHR2 | 0.7080 | (0.565~0.887) | -3.0047 | 0.0027 | 0.3225 | 293th | No |
| GYS2 | 0.7980 | (0.667~0.953) | -2.4836 | 0.0130 | 0.3204 | 294th | No |
| UPB1 | 0.7240 | (0.572~0.918) | -2.6714 | 0.0076 | 0.3203 | 295th | No |
| SLC22A1 | 0.6180 | (0.487~0.783) | -3.9866 | 0.0001 | 0.3186 | 296th | No |
| SLC10A1 | 0.6800 | (0.562~0.824) | -3.9456 | 0.0001 | 0.3165 | 297th | No |
| CFHR4 | 0.6670 | (0.551~0.807) | -4.1643 | 0.0000 | 0.2997 | 298th | No |
| SPP2 | 0.7170 | (0.597~0.863) | -3.5302 | 0.0004 | 0.2862 | 299th | No |
